# Factors associated with COVID-19 death in pregnant women hospitalized in Intensive Care Units

**DOI:** 10.1590/0034-7167-2023-0172

**Published:** 2024-08-26

**Authors:** Milena Ricioli Ribeiro, Marcela de Andrade Pereira Silva, Leticia Furlan de Lima Prates, Rosana Rosseto de Oliveira, Maria Dalva de Barros Carvalho, Sandra Marisa Pelloso

**Affiliations:** I Universidade Estadual de Maringá. Maringá, Paraná, Brazil; II Centro Universitário Ingá. Maringá, Paraná, Brazil

**Keywords:** COVID-19, Maternal Mortality, Intensive Care Unit, Severe Acute Respiratory Syndrome, Pregnant Women, COVID-19, Mortalidad Materna, Unidades de Cuidados Intensivos, Síndrome Respiratório Agudo Grave, Mujeres Embarazadas

## Abstract

**Objectives:**

to evaluate the factors associated with COVID-19 death in pregnant women hospitalized in Intensive Care Units in Brazil.

**Methods:**

this ecological study was conducted using secondary data from Brazilian pregnant women with COVID-19 hospitalized in Intensive Care Units between March 2020 and March 2022. Univariate analysis and logistic regression were employed.

**Results:**

out of 3,547 pregnant women with COVID-19 hospitalized in Intensive Care Units, 811 died (22.8%). It was found that lack of COVID-19 vaccination (OR: 2.73; 95% CI: 1.83; 4.04), dyspnea (OR: 1.73; 95% CI: 1.17; 2.56), obesity (OR: 1.51; 95% CI: 1.05; 2.17), chronic cardiovascular disease (OR: 1.65; 95% CI: 1.14; 2.38), and non-white race/color (OR: 1.29; 95% CI: 1.00; 1.66) were independently and significantly associated with death.

**Conclusions:**

it is concluded that vaccination status, presence of comorbidities, and clinical and ethnic-racial characteristics are associated with COVID-19 death in pregnant women hospitalized in Intensive Care Units in Brazil.

## RESUMEN

**Objetivos:**

evaluar los factores relacionados al óbito por COVID-19 en embarazadas hospitalizadas en Unidad de Cuidados Intensivos en Brasil.

**Métodos:**

se trata de un estudio ecológico realizado con datos secundarios de embarazadas brasileñas con COVID-19 hospitalizadas en Unidad de Cuidados Intensivos, entre marzo de 2020 y marzo de 2022. Empleado análisis univariado y regresión logística.

**Resultados:**

de 3.547 embarazadas con COVID-19 hospitalizadas en Unidad de Cuidados Intensivos, 811 evolucionaron a óbito (22,8%). Evidenciado que la no vacunación contra la COVID-19 (OR: 2,73; IC_95_: 1,83;4,04), disnea (OR: 1,73; IC_95_:1,17;2,56), obesidad (OR: 1,51; IC_95_: 1,05;2,17), enfermedad cardiovascular crónica (OR: 1,65; IC_95_: 1,14;2,38) y la raza/color no blanca (OR: 1,29; IC_95_: 1,00;1,66) se relacionaron de manera independiente y significante al óbito.

**Conclusiones:**

concluido que el estado de vacunación, presencia de comorbilidades y características clínicas y étnico-raciales están relacionadas al óbito por COVID-19 en embarazadas hospitalizadas en Unidad de Cuidados Intensivos en Brasil.

## INTRODUCTION

COVID-19, a disease caused by the novel coronavirus (SARS-CoV-2), was first identified in December 2019 in the city of Wuhan, China^(1)^. Due to its high transmissibility and a rapid increase in the number of cases, the World Health Organization (WHO) declared on January 30, 2020, that the outbreak of the novel coronavirus constituted a Public Health Emergency of International Concern and announced on March 11, 2020, that the situation had evolved into a pandemic^(2-3)^.

On May 5, 2023, the WHO declared the end of the Public Health Emergency of International Concern regarding COVID-19. Over approximately three years, the world recorded 765 million confirmed disease cases and almost 7 million deaths. Brazil ranks second in the world in absolute numbers of deaths, only behind the United States^(4)^.

Some risk factors for the severe progression of COVID-19 have been identified, such as advanced age, male sex, and comorbidities like hypertension, diabetes mellitus, and cardiovascular diseases^(5)^. Regarding the obstetric population, recent studies demonstrate an increased rate of admission to Intensive Care Units (ICU), obstetric complications, and mortality in pregnant women with COVID-19, highlighting that they are considered a risk group for severe acute respiratory syndrome (SARS) caused by SARS-CoV-2^(6)^.

Research published in July 2020 showed that 77% of maternal deaths due to COVID-19 worldwide occurred in Brazil. Between March 2020 and May 2021, the country recorded 3,291 maternal deaths, of which 1,352 deaths were beyond the expected, corresponding to a 70% excess in maternal deaths, most attributed to COVID-19^(7)^. Advanced maternal age, high body mass index, non-white ethnicity, pre-existing comorbidities, and specific gestational disorders such as gestational diabetes and preeclampsia have been identified as risk factors for severe disease progression in pregnant women^(8)^.

However, the factors associated with complications and severe progression of COVID-19 in pregnant women are not yet fully understood. Despite a significant increase in worldwide publications on the relationship between this disease and pregnancy, there is still a scarcity of studies involving the Brazilian obstetric population.

## OBJECTIVES

To evaluate the factors associated with COVID-19 death in pregnant women hospitalized in Intensive Care Units in Brazil.

## METHODS

### Ethical Aspects

As this study involves public domain data with unrestricted access and without identifying the individuals involved, in accordance with Resolution No. 510/16 of the National Health Council (CNS), the Permanent Ethics Committee in Research with Human Beings was not needed to approve it, and the Informed Consent Form was waived^(9)^.

### Study Design and Location

This ecological and retrospective study was conducted using secondary data sourced from the Influenza Epidemiological Surveillance Information System (SIVEP-Gripe). The Ministry of Health (OpenDATASUS) provided these data, which pertain to cases and deaths of Brazilian pregnant women with COVID-19 hospitalized in ICUs. The study was constructed based on the STROBE tool (Strengthening the reporting of observational studies in epidemiology).

### Period and Selection Criteria

The study included all cases reported between March 2020 and March 2022 that met the following criteria: Brazilian pregnant woman, aged between 10 and 49 years, diagnosed with SARS CoV 2 infection, hospitalized in an ICU, and either recovered or deceased. Cases of foreign pregnant women and those who died from other causes were excluded. In some cases, the patient was simultaneously indicated in the database as both pregnant and postpartum, indicating a data entry error. These cases were excluded due to the impossibility of discrimination. The final study sample consisted of 3,547 pregnant women with COVID-19 hospitalized in ICUs.

The database was downloaded on April 12, 2022, and the Ministry of Health last updated it on April 4, 2022. The research data were extracted from the database using R software.

### Study Protocol

The case progression (death or discharge) was considered as the dependent variable. The independent variables collected were: region of residence (North, South, Northeast, Southeast, Center-West), age (< 19 years; 20-34 years; > 34 years), gestational age (first trimester; second trimester; third trimester), race/color (white; non-white), residence zone (urban; rural or peri-urban), COVID-19 vaccination status, signs and symptoms at admission (fever, cough, sore throat/odynophagia, dyspnea, respiratory discomfort, O_2_ saturation < 95%, diarrhea, vomiting, abdominal pain, fatigue, olfactory disturbances, and gustatory disturbances, among others), and presence of comorbidities (chronic cardiovascular disease, diabetes mellitus, asthma/pneumopathy, chronic liver disease, chronic kidney disease, chronic neurological disease, immunodeficiency, and obesity). Individuals with missing data were excluded from analyzing specific variables with absent data.

### Data Analysis and Statistics

The data were analyzed using SPSS software version 20.1. A descriptive analysis was performed, presenting absolute and relative frequencies. Univariate analysis used the chi-square test (χ^2^) and Odds Ratio to identify death-related factors. A 95% confidence interval (CI) was applied, and statistical significance was set at p < 0.05. All variables with *p* < 0.20 in the univariate regression were included in the logistic regression analysis, with only those with *p* < 0.05 remaining in the final model.

## RESULTS

During the study period, 3,547 hospitalizations of pregnant women with COVID-19 in ICUs were reported, of which 811 resulted in death, accounting for 22.8% of the cases. Regarding the characteristics of the pregnant women, most were aged between 20 and 34 years (62.2%), were non-white (57.7%), resided in the Southeast region of the country (47.1%) and in urban areas (93.6%), were in the third trimester of pregnancy (59.0%), and had not received the COVID-19 vaccine (79.6%) (Table 1).

**Table 1:** Univariate analysis of the characteristics of pregnant women with COVID-19 hospitalized in Intensive Care Units, according to the outcome, Brazil, 2020-2022

Variables	n (%)	Desfecho		Crude OR	95% CI	*p*
		Death	Discharge			
Age						
< 20 years	170 (4.8)	22 (12.9)	148 (87.1)	0.54	0.34-0.85	0.008
20-34 years	2,209 (62.3)	478 (21.6)	1,731 (78.4)	-		
> 34 years	1,168 (32.9)	311 (26.6)	857 (73.4)	1.31	1.11-1.55	0.001
Race/Color^1^						
White	1,445 (47.3)	298 (20.6)	1,147 (79.4)	-		
Non-white	1,612 (57.7)	435 (27.0)	1,177(73.0)	1.42	1.20-1.68	< 0.001
Region of residence						
South	582 (16.4)	134 (23.0)	448 (77.0)	-		
Southeast	1,669 (47.1)	335 (20.1)	1,334 (79.9)	0.84	0.67-1.05	0.131
Center-West	440 (12.4)	102 (23.2)	338 (76.8)	1.01	0.75-1.35	0.953
North	269 (7.6)	92 (34.2)	177 (65.8)	1.74	1.26-2.39	0.001
Northeast	587 (16.5)	148 (25.2)	439 (74.8)	1.13	1.26-2.39	0.382
Residence zone^2^						
Urban	2,917 (93.6)	671 (23.0)	2,246 (77.0)	-		
Rural ou peri-urban	198 (6.4)	57 (28.8)	141 (71.2)	1.35	0.98-1.86	0.064
Gestacional Trimester^3^						
1st trimester	257 (7.5)	65 (25.3)	192 (74.7)	-		
2nd trimester	1154 (33.5)	262 (22.7)	892 (77.3)	0.87	0.63-1.19	0.374
3rd trimester	2,037 (59.0)	451 (22.1)	1,586 (77.9)	0.84	0.62-1.13	0.255
Received the COVID-19 vaccine^4^						
Yes	318 (20.4)	36 (11.3)	282 (88.7)	-		
No	1,243 (79.6)	360 (29.0)	883 (71.0)	3.19	2.21-4.61	< 0.001

1 n = 3.057; ^2^ n = 3.115; ^3^ n = 3.448; ^4^ n = 1.561; OR - Odds Ratio; CI - Confidence interval.

It was observed that the variables “age,” “race/color,” “region of residence,” and “COVID-19 vaccination status” were significantly associated with death. Pregnant women with advanced maternal age (OR = 1.31; p = 0.001), non-white race (OR = 1.42; p < 0.001), residing in the North region of the country, and not vaccinated against COVID-19 had a higher chance of death. Maternal age below 20 years was associated with the outcome but as a protective factor (OR = 0.54; p = 0.008) (Table 1).

Among the comorbidities analyzed, the most prevalent were chronic cardiovascular diseases (11.8%), obesity (11.53%), and diabetes mellitus (10.23%). Pregnant women with chronic cardiovascular disease (OR = 1.55; p < 0.001), diabetes mellitus (OR = 1.54; p < 0.001), immunodeficiency (OR = 2.17; p = 0.025), and obesity (OR = 1.94; p < 0.001) had higher chances of death (Table 2).

**Table 2:** Univariate analysis of comorbidities in pregnant women with COVID-19 hospitalized in Intensive Care Units, according to the outcome, Brazil, 2020-2022

Comorbidities	n (%)	Outcome		Crude OR	95% CI	*p*
		Death	Discharge			
Chronic cardiovascular disease						
Yes	419 (11.8)	127 (30.3)	292 (69.7)	1.55	1.24-1.95	< 0.001
No	3.128 (88.2)	684 (21.9)	2.444 (78.1)	-		
Chronic hematological disease						
Yes	17 (0.5)	6 (35.3)	11 (64.7)	1.85	0.68-5.01	0.228
No	3.530 (99.5)	805 (22.8)	2.725 (77.2)	-		
Chronic liver disease						
Yes	10 (0.3)	4 (40.0)	6 (60.0)	2.25	0.64-8.01	0.209
No	3,537 (99.7)	807 (22.8)	2,730 (77.2)	-		
Asthma and/or pneumopathy						
Yes	183 (5.1)	47 (25.7)	136 (74.3)	1.18	0.84-1.65	0.352
No	3,364 (94.9)	764 (22.7)	2,600 (77.3)	-		
Diabetes mellitus						
Yes	363 (10.2)	110 (30.3)	253 (69.7)	1.54	1.21-1.96	< 0.001
No	3,184 (89.8)	701 (22.0)	2,483 (78.0)	-		
Chronic neurological disease						
Yes	30 (0.8)	8 (26.7)	22 (73.3)	1.23	0.54-2.77	0.619
No	3,517 (99.2)	803 (22.8)	2,714 (77.2)	-		
Immunodeficiency						
Yes	36 (1.0)	14 (38.9)	22 (61.1)	2.17	1.10-4.25	0.025
No	3,511 (99.0)	797 (22.7)	2,714 (77.3)	-		
Chronic kidney disease						
Yes	30 (0.8)	7 (23.3)	23 (76.7)	1.02	0.44-2.40	0.951
No	3,517 (99.2)	804 (22.9)	2,713 (77.1)	-		
Obesity						
Yes	409 (11.5)	141 (34.5)	268 (65.5)	1.94	1.55-2.42	< 0.001
No	3,138 (88.5)	670 (21.4)	2,468 (78.6)	-		

*OR - Odds Ratio; CI - Confidence interval.*

Regarding clinical and respiratory symptoms, the most common among pregnant women were dyspnea (84.51%), cough (83.38%), O_2_ saturation < 95% (71.82%), respiratory discomfort (71.60%), and fever (69.02%). Pregnant women with cough (OR = 0.82; p = 0.073), vomiting (OR = 0.73; p = 0.040), and loss of smell (OR = 0.68; p = 0.004) had lower chances of death, while those with dyspnea (OR = 1.76; p < 0.001), O_2_ saturation < 95% (OR = 1.88; p < 0.001), and respiratory discomfort (OR = 1.37; p = 0.001) had higher chances of death (Table 3).

**Table 3:** Univariate analysis of clinical and respiratory symptoms of pregnant women with COVID-19 hospitalized in Intensive Care Units, according to the outcome, Brazil, 2020-2022

Clinical and Respiratory Symptoms	n (%)	Outcome		Crude OR (95% CI)	*p*
		Death	Discharge		
Fever^1^					
Yes	2,095 (69.0)	517 (24.7)	1,578 (75.3)	1.25 (1.04;1.51)	0.018
No	940 (31.0)	195 (20.7)	745 (19.3)	-	
Cough^2^					
Yes	2,674 (83.4)	611 (22.8)	2,063 (77.2)	0.82 (0.67;1.02)	0.073
No	533 (16.6)	141 (26.5)	392 (73.5)	-	
Sore throat^3^					
Yes	655 (25.0)	152 (23.2)	503 (76.8)	0.96 (0.78;1.19)	0.736
No	1,899 (75.0)	453 (23.9)	1.446 (76.1)	-	
Dyspnea^4^					
Yes	2,740 (84.5)	678 (24.7)	2,062 (75.3)	1.76 (1.36;2.27)	< 0.001
No	502 (15.5)	79 (15.7)	423 (84.3)	-	
Respiratory Discomfort^5^					
Yes	2,103 (71.6)	544 (25.9)	1,559 (74.1)	1.37 (1.13;1.67)	0.001
No	834 (28.4)	169 (20.3)	665 (79.7)	-	
O_2_ saturation < 95%^6^					
Yes	2,192 (71.8)	586 (26.7)	1,606 (73.3)	1.88 (1.53;2.30)	< 0.001
No	860 (28.2)	140 (16.3)	720 (83.7)	-	
Diarrhea^7^					
Yes	321 (12.7)	77 (24.0)	244 (76.0)	1.01 (0.77;1.33)	0.949
No	2,191 (87.3)	522 (23.8)	1,669 (76.2)	-	
Vomiting^8^					
Yes	325 (12.9)	61 (18.8)	264 (81.2)	0.73 (0.55;0.99)	0.040
No	2,180 (87.1)	522 (23.9)	1,658 (76.1)	-	
Abdominal Pain^9^					
Yes	215 (9.8)	52 (24.2)	163 (75.8)	1.01 (0.73;1.41)	0.941
No	1,966 (90.2)	471 (24.0)	1,495 (76.0)	-	
Fatigue^10^					
Yes	907 (39.3)	218 (24.0)	689 (76.0)	1.05 (0.86;1.27)	0.657
No	1,399 (60.7)	325 (23.2)	1,074 (76.8)	-	
Loss of smell^11^					
Yes	490 (21.5)	88 (18.0)	402 (82.0)	0.68 (0.53;0.88)	0.004
No	1,792 (78.5)	434 (24.2)	1,358 (75.8)	-	
Loss of taste^12^					
Yes	394 (17.6)	70 (17.8)	324 (82.2)	0.66 (0.50;0.87)	0.003
No	1,847 (82.4)	456 (24.7)	1,391 (75.3)	-	
Others^13^					
Yes	1,423 (52.9)	311 (21.9)	1,112 (78.1)	0.83 (0.70;0.99)	0.049
No	1,264 (47.1)	317 (25.1)	947 (74.9)		

1 n = 3.035; ^2^ n = 3.207; ^3^ n = 2.554; ^4^ n = 3.242; ^5^ n = 2.937; ^6^ n = 3.052; ^7^ n = 2.512; ^8^ n = 2.505; ^9^ n = 2.182; ^10^ n = 2.306; ^11^ n = 2.282; ^12^ n = 2.241; ^13^ n = 2.687; OR - Odds Ratio; CI - Confidence interval.

In the logistic regression, it was shown that pregnant women not vaccinated against COVID 19 had 2.72 times more chances of death, while those with dyspnea had 1.73 times higher chance. Regarding comorbidities, pregnant women with obesity or chronic cardiovascular disease had 1.51 and 1.65 times more chances of death, respectively. Being non-white also proved to be a risk factor for the outcome, with 1.29 times higher chance of death (Table 4).

**Table 4:** Final multiple logistic regression model of factors associated with the outcome in pregnant women with COVID-19 hospitalized in Intensive Care Units, Brazil, 2020-2022

Variables	Adjusted OR (95% CI)	*p*
No COVID-19 vaccination	2.72 (1.83;4.04)	< 0.001
Dyspnea	1.73 (1.17;2.56)	0.006
Obesity	1.51 (1.05;2.17)	0.024
Chronic cardiovascular disease	1.65 (1.14;2.38)	0.007
Non-white	1.29 (1.00;1.66)	0.047

## DISCUSSION

Due to the particularities of the gestational period, there is a higher risk of severe progression and death from COVID-19 among the obstetric population. However, it is still necessary to understand the factors related to the progression of this disease during pregnancy, especially in Brazil, a country with one of the highest numbers of maternal deaths associated with COVID 19^(10)^. The results of this study show that lack of COVID-19 vaccination, presence of dyspnea and comorbidities (such as obesity and chronic cardiovascular disease), and non-white race/color are significantly associated with the death of pregnant women with COVID-19 hospitalized in Intensive Care Units.

A retrospective cohort study conducted in Brazil between May and November 2021, involving 2,284 pregnant and postpartum women with COVID-19 and known vaccination status, identified that the chance of death in the vaccinated population was significantly lower compared to the unvaccinated group. Additionally, ICU hospitalization rates were considerably lower among vaccinated women^(11)^.

A prospective single-center cohort study conducted in a Brazilian maternity hospital between May 2020 and March 2022 found that, out of 1,609 pregnant women, 25.5% were infected with SARS-CoV-2, and COVID-19 vaccination reduced the risk of severe maternal morbidity and mortality^(12)^.

In the present study, unvaccinated pregnant women had 2.27 times higher chances of death compared to vaccinated pregnant women, corroborating current scientific evidence indicating the protective effects of the COVID-19 vaccine on ICU admission and death in pregnant women with the disease. These findings emphasize the importance of actions promoting vaccination among the obstetric population.

Regarding signs and symptoms, pregnant women who manifested dyspnea had 1.73 times higher chances of death, confirming the findings in the scientific literature that highlight respiratory symptoms as predictors of severe disease in the general and obstetric populations^(13-14)^.

A systematic review and meta-analysis involving 1,813 COVID-19 patients identified dyspnea as a predictive symptom of severe disease and ICU admission^(13)^. Similarly, a multicenter cohort study investigating the clinical progression of COVID-19 in pregnant women found that patients with severe disease had a higher frequency of dyspnea at hospital admission compared to those with non-severe disease^(14)^.

The association between severe progression of COVID-19 and the presence of comorbidities has been described since the beginning of the pandemic. The results of this research demonstrate a higher chance of death among pregnant women with obesity and chronic cardiovascular disease. A prospective cohort study conducted in Mexico with 13,062 pregnant women identified clinical risk factors associated with maternal mortality as pre-existing diabetes, chronic hypertension, and obesity^(15)^. Consistent with these results, other investigations reinforce that pregnant women with pre-existing comorbidities such as obesity, hypertension, and diabetes have a higher risk of severe disease progression and death^(15-17)^.

Ethnic-racial inequalities in Brazilian healthcare are highlighted by several indicators showing significant differences in mortality among racial categories^(18)^. During the COVID-19 pandemic, ethnic-racial inequality widened, contributing to negative and lethal maternal outcomes^(19)^. A Brazilian study conducted in 2020 with 12,556 pregnant and postpartum women with COVID-19 showed that the mortality rate was twice as high in women who self-identified as black compared to white women^(20)^.

Furthermore, Brazilian research has shown care failures and substantial barriers to access to intensive care for pregnant women, which significantly impacted the high number of maternal deaths in the country^(21)^. However, it is noteworthy that, in the present study, despite access to intensive care, non-white pregnant women had 1.29 times higher chances of death compared to white pregnant women. In this regard, a Brazilian study evaluating the disproportionate impact of structural racism on maternal deaths due to COVID-19 observed that, despite similar average age and morbidity profiles between black and white women, black women were hospitalized in more severe conditions with a higher risk of death, which can indicate that black pregnant women were disproportionately affected by the disease^(22)^.

### Study limitations

One limitation of the study was the use of secondary data, which are subject to underreporting or data entry issues, potentially leading to some underestimation. However, it is known that the health information systems of the Brazilian Ministry of Health are widely used in scientific research, as they allow for the epidemiological investigation of diseases on a national level, aiding in planning public health policies and programs. It is emphasized that SIVEP Gripe has been the primary source of data on COVID-19 hospitalizations in the country.

### Contributions to the Nursing field

The findings of this study can contribute to recognizing the factors associated with the death of Brazilian pregnant women with COVID-19 hospitalized in ICUs, favoring the planning of actions aimed at reducing maternal morbidity and mortality in the country. Additionally, these findings may support the development of health policies and programs that address reducing racial disparities and advancing COVID-19 vaccination among the obstetric population-this procedure has helped prevent case aggravation.

## CONCLUSIONS

It is concluded that vaccination status, the presence of comorbidities, and clinical and ethnic racial characteristics are associated with COVID-19 death in pregnant women hospitalized in ICUs in Brazil. Identifying risk factors can assist in planning actions to care for pregnant women affected by this disease.

The findings also reinforce the positive impact of COVID-19 vaccination in preventing severe cases with unfavorable outcomes, such as maternal death, and highlight the need for the country to advance in vaccination, given the significant proportion of unvaccinated pregnant women. It is also imperative to implement public policies addressing racial disparities to improve access and health care for black, brown, and indigenous pregnant women.

It is suggested that studies analyze the event considering socioeconomic, environmental, and care variables, as economic inequalities, insufficient social development, and limited access to health services are factors that can influence maternal mortality associated with COVID-19.

## Supplementary Material

0034-7167-reben-77-05-e20230172-suppl01

## Data Availability

https://doi.org/10.48331/scielodata.Y8JBGG
