# Supplementary material for: Factors associated with COVID-19 death in pregnant women hospitalized in Intensive Care Units
Source: Rev Bras Enferm. 2024 Aug 26;77(5):e20230172. doi: 10.1590/0034-7167-2023-0172 (PMC11346909; doi:10.1590/0034-7167-2023-0172)
Supplement: 0034-7167-reben-77-05-e20230172-suppl01 [file 0034-7167-reben-77-05-e20230172-suppl01.pdf]

DT_NOTIFI(	SEM_NOT	DT_SIN_PR	SEM_PRI	SG_UF_NO	D_REGION/	O_REGION	D_MUNICII	D_MUN_N(	D_UNIDAD
17/09/202(	38	18/06/202(	25	BA	NUCLEO RI	1380	SALVADOR	292740	HOSPITAL !
07/01/202	1	31/12/202(	53	AM	ENTORNO	5584	MANAUS	130260	MATERNID
22/12/202(	52	12/12/202(	50	RJ			RESENDE	330420	HOSPITAL !
23/04/202(	17	13/04/202(	16	RJ			RIO DE JAN	330455	SMS HOSPI
20/07/202(	30	07/07/202(	28	SP	GVE XIX M/	1344	MARILIA	352900	SANTA CAS
03/08/202(	32	28/07/202(	31	SP	GVE XVII C/	1342	SUMARE	355240	HOSPITAL I
03/08/202(	32	07/07/202(	28	SP	GVE I CAPI	1331	SAO PAUL(	355030	HC DA FMU
21/09/202(	39	10/09/202(	37	RJ			CAMPOS D	330100	HOSPITAL I
22/09/202(	39	15/09/202(	38	MS	NRS DE DO	1966	DOURADO:	500370	HOSPITAL I
06/05/202(	19	29/04/202(	18	RJ			NILOPOLIS	330320	DOMINGO:
28/08/202(	35	10/08/202(	33	SP	GVE I CAPI	1331	SAO PAUL(	355030	HOSP MUN
05/12/202(	49	26/11/202(	48	MS	EX ARS DE	1975	CAMPO GF	500270	HOSPITAL I
06/01/202	1	26/12/202(	52	AM	ENTORNO	5584	MANAUS	130260	MATERNID
21/01/202	3	24/11/202(	48	RJ			RIO DE JAN	330455	PERINATAL
30/11/202(	49	20/11/202(	47	MA	REGIONAL	1440	IMPERATRI	210530	HOSPITAL I
14/05/202(	20	09/05/202(	19	SP	GVE I CAPI	1331	SAO PAUL(	355030	HOSPITAL (
03/06/202(	23	27/05/202(	22	SP	GVE I CAPI	1331	SAO PAUL(	355030	HOSP MUN
08/07/202(	28	28/06/202(	27	CE	11 CRES SC	1521	SOBRAL	231290	HOSPITAL I
15/07/202(	29	05/07/202(	28	SP	GVE XI ARA	1336	ARACATUE	350280	HOSPITAL U
14/09/202(	38	08/09/202(	37	GO	CENTRAL	1779	GOIANIA	520870	HOSPITAL I
30/12/202(	53	17/11/202(	47	RJ			RIO DE JAN	330455	SMS COOR
21/12/202(	52	16/12/202(	51	RR			BOA VISTA	140010	HOSPITAL (
31/05/202(	23	25/05/202(	22	RJ			CAMPOS D	330100	HOSPITAL I
08/06/202(	24	29/05/202(	22	SP	GVE XXXI S	1353	SOROCABA	355220	HOSPITAL U
29/04/202(	18	21/04/202(	17	CE	1 CRES FOF	1519	FORTALEZ/	230440	HGCC HOS
06/06/202(	23	03/06/202(	23	SP	GVE XVII C/	1342	JUNDIAI	352590	HU HOSPIT
11/06/202(	24	31/05/202(	23	BA	NUCLEO RI	1380	SALVADOR	292740	HOSPITAL T
28/11/202(	48	17/11/202(	47	SP	GVE XVII C/	1342	CAMPINAS	350950	HOSPITAL I
19/10/202(	43	15/10/202(	42	PE	001	1497	RECIFE	261160	IMIP
07/09/202(	37	03/09/202(	36	RJ			NILOPOLIS	330320	DOMINGO:
19/05/202(	21	03/05/202(	19	SP	GVE I CAPI	1331	SAO PAUL(	355030	HC DA FMU
11/06/202(	24	01/06/202(	23	SP	GVE XXIX S	1354	SAO JOSE D	354980	HOSPITAL I
06/10/202(	41	30/09/202(	40	MG	BELO HORI	1449	CONTAGEN	311860	UNIDADE F
06/11/202(	45	15/10/202(	42	MG	UBERLAND	1462	UBERLAND	317020	HOSPITAL I
30/12/202(	53	27/12/202(	53	BA	NUCLEO RI	1380	SALVADOR	292740	MATERNID
23/06/202(	26	17/06/202(	25	SP	GVE XXIX S	1354	SAO JOSE D	354980	HOSPITAL I
19/08/202(	34	12/08/202(	33	GO	CENTRAL	1779	GOIANIA	520870	HOSPITAL I
24/06/202(	26	20/06/202(	25	SP	GVE XVII C/	1342	CAMPINAS	350950	HOSPITAL I
17/07/202(	29	10/07/202(	28	SP	GVE I CAPI	1331	SAO PAUL(	355030	HOSPITAL !
07/08/202(	32	07/08/202(	32	PE	001	1497	RECIFE	261160	IMIP
11/01/202	2	25/12/202(	52	SP	GVE XVII C/	1342	CAMPINAS	350950	MATERNID
26/04/202(	18	19/04/202(	17	AM	ENTORNO	5584	MANAUS	130260	MATERNID
01/05/202(	18	26/04/202(	18	SP	GVE I CAPI	1331	SAO PAUL(	355030	SAO LUIZ U
09/04/202(	15	04/04/202(	14	SP	GVE I CAPI	1331	SAO PAUL(	355030	HOSPITAL I
23/03/202(	13	22/03/202(	13	RJ			RIO DE JAN	330455	SMS HOSPI
23/01/202	3	08/08/202(	32	AL	1 MICRORI	1533	MACEIO	270430	HOSPITAL I

26/06/202	26	21/06/202	26	SP	GVE XVII C/	1342	CAMPINAS	350950	HOSPITAL I
27/04/202	18	14/04/202	16	SP	GVE I CAPIT	1331	SAO PAULO	355030	HOSPITAL I
18/06/202	25	05/06/202	23	MT	CUIABA	1578	CUIABA	510340	HOSPITAL U
13/07/202	29	27/05/202	22	AL	1 MICRORI	1533	MACEIO	270430	HOSPITAL U
02/07/202	27	26/06/202	26	BA	NUCLEO RI	1380	SALVADOR	292740	MATERNID
26/04/202	18	23/04/202	17	PA	01 REGION	1484	BELEM	150140	SANTA CAS
20/11/202	47	10/11/202	46	SC	BLUMENAU	1564	BLUMENAU	420240	HOSPITAL S
27/12/202	53	24/12/202	52	RJ			PETROPOLI	330390	HOSPITAL A
07/05/202	19	27/04/202	18	PE	001	1497	RECIFE	261160	IMIP
01/08/202	31	26/07/202	31	PR	03RS PONT	1357	PONTA GR	411990	HOSPITAL U
16/06/202	25	07/06/202	24	SP	GVE I CAPIT	1331	SAO PAULO	355030	HC DA FMU
03/08/202	32	30/07/202	31	BA	NUCLEO RI	1380	SALVADOR	292740	MATERNID
29/07/202	31	12/07/202	29	SP	GVE I CAPIT	1331	SAO PAULO	355030	MATERNID
13/08/202	33	08/08/202	32	BA	NUCLEO RI	1380	SALVADOR	292740	MATERNID
27/11/202	48	20/11/202	47	SP	GVE VII SAI	1332	SAO BERNA	354870	HOSPITAL I
16/12/202	51	06/11/202	45	SP	GVE I CAPIT	1331	SAO PAULO	355030	HOSP MUN
26/05/202	22	17/05/202	21	RS	002 CRS	1607	PORTO ALE	431490	HOSPITAL I
25/06/202	26	22/04/202	17	PA	01 REGION	1484	ANANINDE	150080	DIRETORIA
16/06/202	25	15/06/202	25	CE	1 CRES FOF	1519	FORTALEZA	230440	HGCC HOS
14/09/202	38	05/09/202	36	SP	GVE XX PIR	1345	PIRACICAB	353870	SANTA CAS
19/10/202	43	14/10/202	42	RS	013 CRS	1609	VENANCIO	432260	HOSPITAL S
10/05/202	20	23/04/202	17	RJ			RIO DE JAN	330455	HOSPITAL (
20/01/202	3	29/12/202	53	SP	GVE I CAPIT	1331	SAO PAULO	355030	MATERNID
30/05/202	22	25/05/202	22	RJ			NITEROI	330330	HOSPITAL U
23/06/202	26	15/06/202	25	SP	GVE XXVIII	1576	SAO SEBAS	355070	HOSPITAL I
29/06/202	27	21/06/202	26	BA	NUCLEO RI	1380	SALVADOR	292740	MATERNID
29/07/202	31	26/07/202	31	PR	02RS METF	1356	CURITIBA	410690	COMPLEXO
29/07/202	31	27/07/202	31	RJ			RIO DE JAN	330455	MS HSE HC
21/09/202	39	12/09/202	37	SP	GVE I CAPIT	1331	SAO PAULO	355030	MATERNID
02/08/202	32	25/07/202	30	RJ			RIO DE JAN	330455	SMS HOSPI
06/04/202	15	24/03/202	13	PE	001	1497	RECIFE	261160	HOSPITAL (
30/04/202	18	17/04/202	16	RJ			RIO DE JAN	330455	PERINATAL
14/04/202	16	09/04/202	15	CE	1 CRES FOF	1519	FORTALEZA	230440	MATERNID
20/07/202	30	15/07/202	29	CE	20 CRES CF	1530	CRATO	230420	HOSPITAL I
17/06/202	25	14/06/202	25	RJ			NITEROI	330330	SES RJ HOS
08/07/202	28	25/06/202	26	SP	GVE IX FRA	1334	CAIEIRAS	350900	HOSPITAL I
01/11/202	45	25/10/202	44	PB	III NRS CAN	1421	CAMPINA (	250400	HOSPITAL I
21/12/202	52	05/12/202	49	BA	NUCLEO RI	1380	SALVADOR	292740	HOSPITAL S
11/12/202	50	06/12/202	50	MT	CUIABA	1578	CUIABA	510340	HOSPITAL U
23/12/202	52	19/12/202	51	AL	1 MICRORI	1533	MACEIO	270430	SANTA CAS
30/04/202	18	28/04/202	18	SP	GVE I CAPIT	1331	SAO PAULO	355030	HC DA FMU
26/04/202	18	19/04/202	17	PB	I NRS JOAC	1377	JOAO PESS	250750	MATERNID
03/07/202	27	23/06/202	26	MG	BELO HORI	1449	BELO HORI	310620	HOSPITAL J
07/08/202	32	22/07/202	30	DF			BRASILIA	530010	HRAN
01/08/202	31	25/07/202	30	PR	02RS METF	1356	CURITIBA	410690	HOSPITAL S
18/11/202	47	15/11/202	47	RJ			RESENDE	330420	HOSPITAL I
10/11/202	46	02/11/202	45	PR	04RS IRATI	1358	IRATI	411070	SANTA CAS

02/10/202	40	21/09/202	39	PB	I NRS JOAC	1377	JOAO PESS	250750	MATERNID
28/12/202	53	19/12/202	51	SP	GVE I CAPI	1331	SAO PAUL	355030	HOSPITAL
21/04/202	17	16/04/202	16	RJ			RIO DE JAN	330455	CASA DE S
20/04/202	17	17/04/202	16	SP	GVE I CAPI	1331	SAO PAUL	355030	HC DA FMU
22/05/202	21	06/05/202	19	CE	1 CRES FOF	1519	FORTALEZ	230440	HOSPITAL
22/06/202	26	16/06/202	25	SP	GVE XVII C	1342	CAMPINAS	350950	MATERNID
26/06/202	26	22/06/202	26	SP	GVE XXIX S	1354	SAO JOSE D	354980	HOSPITAL
17/08/202	34	10/08/202	33	SP	GVE XXXI S	1353	SOROCABA	355220	HOSPITAL
05/07/202	28	03/07/202	27	PB	III NRS CAM	1421	CAMPINA	250400	INSTITUTO
08/04/202	15	29/03/202	14	SP	GVE I CAPI	1331	SAO PAUL	355030	MATERNID
18/05/202	21	15/05/202	20	BA	NUCLEO RI	1380	SALVADOR	292740	HOSPITAL
28/12/202	53	17/12/202	51	SP	GVE XVII C	1342	JUNDIAI	352590	INTERMED
01/05/202	18	25/04/202	17	SP	GVE I CAPI	1331	SAO PAUL	355030	HC DA FMU
02/07/202	27	27/06/202	26	SP	GVE I CAPI	1331	SAO PAUL	355030	HC DA FMU
22/04/202	17	22/04/202	17	SP	GVE I CAPI	1331	SAO PAUL	355030	HOSPITAL
12/06/202	24	04/06/202	23	PE	001	1497	RECIFE	261160	IMIP
03/07/202	27	11/06/202	24	AP			MACAPA	160030	HOSPITAL
08/09/202	37	20/08/202	34	TO			PALMAS	172100	HOSPITAL
11/07/202	28	04/07/202	27	RS	001 CRS	1592	NOVO HAM	431340	FUNDACAC
20/10/202	43	05/10/202	41	SP	GVE VII SA	1332	SANTO AN	354780	HOSPITAL
13/07/202	29	05/07/202	28	SP	GVE XXIX S	1354	SAO JOSE D	354980	HOSPITAL
02/07/202	27	19/06/202	25	RJ			TERESOPO	330580	HOSPITAL
11/09/202	37	09/09/202	37	SP	GVE VII SA	1332	SANTO AN	354780	HOSPITAL
10/09/202	37	25/08/202	35	SP	GVE I CAPI	1331	SAO PAUL	355030	HOSPITAL
26/04/202	18	21/04/202	17	RJ			RIO DE JAN	330455	SMS HOSPI
21/05/202	21	14/05/202	20	SP	GVE I CAPI	1331	SAO PAUL	355030	HOSPITAL
20/06/202	25	12/06/202	24	MT	CUIABA	1578	CUIABA	510340	HOSPITAL
12/07/202	29	11/07/202	28	RJ			RIO DE JAN	330455	CLINICA SA
21/10/202	43	12/10/202	42	SP	GVE XXIX S	1354	SAO JOSE D	354980	HOSPITAL
08/12/202	50	05/12/202	49	SP	GVE I CAPI	1331	SAO PAUL	355030	HOSPITAL
21/01/202	3	01/12/202	49	RJ			RIO DE JAN	330455	PERINATAL
01/05/202	18	26/04/202	18	AM	ENTORNO	5584	MANAUS	130260	MATERNID
13/06/202	24	09/06/202	24	DF			BRASILIA	530010	HOSPITAL
10/12/202	50	01/12/202	49	RS	002 CRS	1607	PORTO ALE	431490	IRMANDAD
17/07/202	29	06/07/202	28	AP			MACAPA	160030	HOSPITAL
21/08/202	34	16/08/202	34	RJ			CAMPOS D	330100	HOSPITAL
01/07/202	27	30/06/202	27	RJ			PETROPOLI	330390	HOSPITAL
23/10/202	43	19/10/202	43	PA	01 REGION	1484	BELEM	150140	SANTA CAS
10/05/202	20	10/05/202	20	CE	1 CRES FOF	1519	FORTALEZ	230440	HGCC HOS
03/09/202	36	25/08/202	35	GO	CENTRAL	1779	GOIANIA	520870	HOSPITAL
08/12/202	50	04/12/202	49	BA	NUCLEO RI	1380	SALVADOR	292740	MATERNID
16/12/202	51	07/12/202	50	PR	11RS CAMI	1365	CAMPO M	410430	HOSPITAL
30/03/202	14	23/03/202	13	SP	GVE I CAPI	1331	SAO PAUL	355030	HOSPITAL
25/06/202	26	16/06/202	25	RJ			RIO DE JAN	330455	MS HSE HC
23/07/202	30	08/07/202	28	SC	BLUMENAU	1564	BRUSQUE	420290	IMIGRANT
18/09/202	38	05/09/202	36	SP	GVE XVII C	1342	CAMPINAS	350950	CENTRO M
13/12/202	51	07/12/202	50	PA	11 REGION	1494	MARABA	150420	HOSPITAL

02/11/202	45	30/10/202	44	PA	01 REGION	1484	BELEM	150140	SANTA CAS
29/06/202	27	23/06/202	26	GO	CENTRAL	1779	GOIANIA	520870	HOSPITAL I
02/12/202	49	20/11/202	47	SC	JOINVILLE	1565	JOINVILLE	420910	HOSPITAL I
28/10/202	44	04/10/202	41	RJ			RIO DE JAN	330455	HOSPITAL (
26/11/202	48	21/11/202	47	SP	GVE I CAPI	1331	SAO PAUL(	355030	BP HOSPIT.
10/05/202	20	04/05/202	19	AM	ENTORNO	5584	MANAUS	130260	HOSPITAL I
06/05/202	19	07/04/202	15	SP	GVE I CAPI	1331	SAO PAUL(	355030	HC DA FMU
03/05/202	19	11/04/202	15	RJ			RIO DE JAN	330455	SMS HOSPI
01/07/202	27	27/06/202	26	SP	GVE I CAPI	1331	SAO PAUL(	355030	HC DA FMU
03/09/202	36	22/08/202	34	AM	ENTORNO	5584	MANAUS	130260	HOSPITAL I
04/05/202	19	28/04/202	18	RJ			DUQUE DE	330170	HOSPITAL (
11/05/202	20	27/04/202	18	SP	GVE I CAPI	1331	SAO PAUL(	355030	HOSP MUN
11/01/202	2	27/12/202	53	AM	ENTORNO	5584	MANAUS	130260	MATERNID
18/12/202	51	14/12/202	51	SP	GVE VII SAI	1332	SAO BERN/	354870	HOSPITAL S
02/06/202	23	23/05/202	21	PB	I NRS JOAC	1377	JOAO PESS	250750	MATERNID
02/06/202	23	29/05/202	22	SP	GVE I CAPI	1331	SAO PAUL(	355030	HC DA FMU
20/07/202	30	11/07/202	28	RJ			NILOPOLIS	330320	DOMINGO.
17/12/202	51	13/12/202	51	CE	11 CRES SC	1521	SOBRAL	231290	HOSPITAL I
30/06/202	27	21/06/202	26	SP	GVE XXVIII	1576	CARAGUAT	351050	CASA DE S/
18/05/202	21	12/05/202	20	SP	GVE XVII C/	1342	JUNDIAI	352590	HOSPITAL I
23/09/202	39	12/09/202	37	SC	JOINVILLE	1565	JOINVILLE	420910	HOSPITAL I
28/11/202	48	18/11/202	47	SP	GVE XXIX S	1354	SAO JOSE D	354980	HOSPITAL I
30/04/202	18	21/04/202	17	SP	GVE I CAPI	1331	SAO PAUL(	355030	HOSPITAL S
26/05/202	22	21/05/202	21	BA	NUCLEO RI	1380	SALVADOR	292740	MATERNID
05/05/202	19	28/04/202	18	PE	001	1497	RECIFE	261160	IMIP
24/07/202	30	19/07/202	30	SP	GVE XXIX S	1354	SAO JOSE D	354980	HOSPITAL I
09/07/202	28	28/06/202	27	MA	REGIONAL	1441	BALSAS	210140	HOSPITAL I
31/08/202	36	19/08/202	34	DF			BRASILIA	530010	HOSPITAL S
30/09/202	40	25/09/202	39	SP	GVE XXIX S	1354	SAO JOSE D	354980	HOSPITAL I
06/10/202	41	05/09/202	36	PR	16RS APUC	1370	APUCARAN	410140	HNSG HOS
08/01/202	1	25/12/202	52	SP	GVE I CAPI	1331	SAO PAUL(	355030	HOSPITAL S
01/12/202	49	18/11/202	47	RJ			RIO DE JAN	330455	MS HSE HC
28/12/202	53	23/12/202	52	SP	GVE I CAPI	1331	SAO PAUL(	355030	HC DA FMU
06/01/202	1	30/12/202	53	SP	GVE XXXI S	1353	SOROCABA	355220	HOSPITAL I
16/06/202	25	13/06/202	24	SC	CHAPECO	1553	CHAPECO	420420	HOSPITAL I
15/12/202	51	10/12/202	50	AM	ENTORNO	5584	MANAUS	130260	MATERNID
09/05/202	19	25/04/202	17	SP	GVE I CAPI	1331	SAO PAUL(	355030	HC DA FMU
14/05/202	20	10/05/202	20	SP	GVE I CAPI	1331	SAO PAUL(	355030	HOSPITAL I
12/06/202	24	10/06/202	24	PB	I NRS JOAC	1377	JOAO PESS	250750	MATERNID
22/06/202	26	20/06/202	25	SP	GVE XVII C/	1342	CAMPINAS	350950	HOSPITAL I
12/06/202	24	08/06/202	24	BA	NUCLEO RI	1385	ITABUNA	291480	HOSPITAL I
31/08/202	36	08/08/202	32	DF			BRASILIA	530010	HOSPITAL S
25/04/202	17	19/04/202	17	RS	016 CRS	1610	LAJEADO	431140	HOSPITAL I
20/05/202	21	13/05/202	20	SP	GVE XXVII S	1351	SAO JOSE D	354990	HOSPITAL V
24/04/202	17	15/04/202	16	RJ			RIO DE JAN	330455	SMS HOSPI
27/04/202	18	09/04/202	15	SP	GVE I CAPI	1331	SAO PAUL(	355030	SANTA CAS
11/06/202	24	05/06/202	23	SP	GVE I CAPI	1331	SAO PAUL(	355030	HOSP MUN

24/06/202	26	10/06/202	24	PI	4 GRS TERE	1883	TERESINA	221100	MATERNID
18/07/202	29	01/07/202	27	MT	CUIABA	1578	CUIABA	510340	HOSPITAL S
09/08/202	33	03/08/202	32	SP	GVE XII ARA	1337	TABATINGA	355270	CENTRO DI
16/09/202	38	09/09/202	37	RS	016 CRS	1610	LAJEADO	431140	HOSPITAL I
18/09/202	38	10/09/202	37	SP	GVE XXIX S	1354	CATANDUV	351110	UNIMED H
11/09/202	37	07/09/202	37	MG	CORONEL F	1468	IPATINGA	313130	HOSPITAL I
13/05/202	20	10/05/202	20	SP	GVE I CAPIT	1331	SAO PAULO	355030	HOSPITAL I
16/12/202	51	11/12/202	50	SP	GVE XIX MA	1344	MARILIA	352900	HCFAMEM
07/06/202	24	21/05/202	21	PB	I NRS JOAO	1377	JOAO PESS	250750	MATERNID
11/04/202	15	05/04/202	15	MA	REGI0NAL	1430	SAO LUIS	211130	CENTRO M
27/06/202	26	25/06/202	26	PI	4 GRS TERE	1883	TERESINA	221100	UNIDADE D
10/08/202	33	09/08/202	33	RS	014 CRS	1602	SANTA ROS	431720	HOSPITAL V
31/12/202	53	21/12/202	52	RN	VII URSAP	1416	NATAL	240810	HOSPITAL (
07/06/202	24	05/06/202	23	RN	II URSAP	1412	MOSSORO	240800	HOSPITAL I
30/04/202	18	25/04/202	17	SP	GVE I CAPIT	1331	SAO PAULO	355030	HOSPITAL I
14/07/202	29	04/07/202	27	RS	013 CRS	1609	SANTA CRU	431680	HOSPITAL S
31/07/202	31	22/07/202	30	PR	02RS METR	1356	CURITIBA	410690	HOSPITAL S
07/12/202	50	29/11/202	49	SP	GVE I CAPIT	1331	SAO PAULO	355030	HC DA FMU
17/06/202	25	10/06/202	24	MA	REGI0NAL	1430	SAO LUIS	211130	EBSERH HC
13/07/202	29	11/07/202	28	MG	JUIZ DE FO	1452	JUIZ DE FO	313670	SANTA CAS
14/08/202	33	14/08/202	33	GO	PIRINEUS	1792	ANAPOLIS	520110	SANTA CAS
21/10/202	43	15/10/202	42	AM	ENTORNO	5584	MANAUS	130260	MATERNID
19/11/202	47	15/11/202	47	PA	12 REGION	1495	REDENCAO	150613	HOSPITAL I
17/11/202	47	02/11/202	45	MG	BELO HORI	1449	BETIM	310670	UNIMED BI
11/06/202	24	01/06/202	23	SP	GVE XX PIR	1345	PIRACICAB	353870	SANTA CAS
17/07/202	29	15/07/202	29	MT	CUIABA	1578	CUIABA	510340	HOSPITAL S
24/08/202	35	20/08/202	34	CE	11 CRES SC	1521	SOBRAL	231290	HOSPITAL I
24/07/202	30	12/07/202	29	GO	CENTRAL	1779	GOIANIA	520870	HOSPITAL I
18/10/202	43	16/10/202	42	MG	UBERABA	1461	UBERABA	317010	HOSPITAL I
28/05/202	22	20/05/202	21	AM	ENTORNO	5584	MANAUS	130260	MATERNID
29/05/202	22	18/05/202	21	BA	NUCLEO RE	1385	JEQUIE	291800	HOSPITAL S
05/07/202	28	21/04/202	17	PB	I NRS JOAO	1377	JOAO PESS	250750	MATERNID
26/12/202	52	22/12/202	52	RJ			RIO DE JAN	330455	MS HSE HC
26/07/202	31	03/07/202	27	DF			BRASILIA	530010	HRAN
08/09/202	37	03/09/202	36	GO	CENTRAL	1779	GOIANIA	520870	HOSPITAL I
15/05/202	20	13/05/202	20	CE	1 CRES FOF	1519	FORTALEZA	230440	UNIDADE F
10/08/202	33	02/08/202	32	PR	17RS LOND	1371	LONDRINA	411370	HOSPITAL U
09/07/202	28	09/07/202	28	CE	1 CRES FOF	1519	FORTALEZA	230440	HGCC HOS
10/06/202	24	03/06/202	23	RN	VII URSAP	1416	NATAL	240810	HOSPITAL I
19/11/202	47	11/11/202	46	SP	GVE VII SAI	1332	SAO BERNA	354870	HOSPITAL I
19/04/202	17	17/04/202	16	RJ			RIO DE JAN	330455	SMS HOSPI
17/07/202	29	12/07/202	29	DF			BRASILIA	530010	HRSM
02/04/202	14	29/03/202	14	RJ			RIO DE JAN	330455	SMS RIO H
15/04/202	16	13/04/202	16	RJ			RIO DE JAN	330455	HOSPITAL (
16/07/202	29	05/07/202	28	SP	GVE I CAPIT	1331	SAO PAULO	355030	HC DA FMU
11/01/202	2	30/12/202	53	SP	GVE I CAPIT	1331	SAO PAULO	355030	SAO LUIZ U
10/07/202	28	07/07/202	28	SP	GVE XXVI S	1350	ESPIRITO S	351518	HOSPITAL I

24/06/202	26	15/06/202	25	MT	TANGARA	1585	TANGARA	510795	HOSPITAL I
29/12/202	53	20/12/202	52	DF			BRASILIA	530010	HRAN
13/01/202	2	01/01/202	53	AM	ENTORNO	5584	MANAUS	130260	MATERNID
26/12/202	52	19/12/202	51	SP	GVE I CAPI	1331	SAO PAUL(	355030	HOSP SAO
24/04/202	17	15/04/202	16	RJ			RIO DE JAN	330455	SMS HOSPI
04/06/202	23	23/05/202	21	DF			BRASILIA	530010	HOSPITAL !
16/06/202	25	06/06/202	23	SP	GVE I CAPI	1331	SAO PAUL(	355030	HOSPITAL I
21/07/202	30	10/07/202	28	SP	GVE XXIX S	1354	SAO JOSE D	354980	HOSPITAL I
28/08/202	35	23/08/202	35	AL	1 MICRORI	1533	MACEIO	270430	HOSPITAL U
21/12/202	52	15/12/202	51	SP	GVE I CAPI	1331	SAO PAUL(	355030	HOSPITAL I
13/12/202	51	13/12/202	51	RS	002 CRS	1607	CAMAQUA	430350	HOSPITAL I
01/10/202	40	24/09/202	39	RJ			NILOPOLIS	330320	DOMINGO:
23/12/202	52	14/12/202	51	MG	DIVINOPOI	1472	DIVINOPOI	312230	HOSPITAL !
03/05/202	19	26/04/202	18	SP	GVE I CAPI	1331	SAO PAUL(	355030	SAO LUIZ U
06/11/202	45	29/10/202	44	RR			BOA VISTA	140010	HOSPITAL (
03/07/202	27	02/07/202	27	GO	CENTRAL	1779	GOIANIA	520870	HOSPITAL I
07/07/202	28	16/06/202	25	PA	09 REGION	1492	SANTAREM	150680	HOSPITAL I
26/05/202	22	16/05/202	20	MG	TEOFILO O	1465	TEOFILO O	316860	HOSPITAL !
16/10/202	42	09/10/202	41	MS	EX ARS DE	1975	CAMPO GF	500270	HOSPITAL I
18/04/202	16	13/04/202	16	PA	09 REGION	1492	SANTAREM	150680	HOSPITAL I
26/07/202	31	19/07/202	30	PR	01RS PAR/	1355	PARANAGU	411820	HOSPITAL I
05/12/202	49	01/12/202	49	ES	METROPOI	1510	SERRA	320500	HOSPITAL I
12/01/202	2	02/01/202	53	RJ			RIO DE JAN	330455	MS HSE HC
29/09/202	40	29/09/202	40	SP	GVE I CAPI	1331	SAO PAUL(	355030	HOSP MUN
22/04/202	17	18/04/202	16	RJ			RIO DE JAN	330455	HOSPITAL (
29/11/202	49	23/11/202	48	RJ			RIO DAS O!	330452	PRONTO S(
13/07/202	29	04/07/202	27	BA	NUCLEO RI	1380	SALVADOR	292740	HOSPITAL J
23/07/202	30	19/07/202	30	SP	GVE XXXIII	1352	LORENA	352720	HOSPITAL U
18/12/202	51	10/12/202	50	SP	GVE VIII M	1333	GUARULH(	351880	HOSPITAL I
07/01/202	1	21/12/202	52	RJ			NILOPOLIS	330320	DOMINGO:
15/05/202	20	14/05/202	20	SP	GVE I CAPI	1331	SAO PAUL(	355030	HOSPITAL I
01/06/202	23	21/04/202	17	AL	1 MICRORI	1533	MACEIO	270430	HOSPITAL I
19/06/202	25	12/06/202	24	MT	CUIABA	1578	CUIABA	510340	HOSPITAL U
30/05/202	22	27/05/202	22	CE	1 CRES FOI	1519	FORTALEZ/	230440	HGCC HOS
21/01/202	3	01/01/202	53	BA	NUCLEO RI	1398	GUANAMB	291170	HOSPITAL I
16/06/202	25	06/06/202	23	GO	CENTRAL	1779	GOIANIA	520870	HOSPITAL I
18/09/202	38	10/09/202	37	MT	CUIABA	1578	CUIABA	510340	HOSPITAL U
20/11/202	47	12/11/202	46	GO	ESTRADA D	1802	CATALAO	520510	UPA DR JAI
15/05/202	20	04/05/202	19	SP	GVE I CAPI	1331	SAO PAUL(	355030	HC DA FMU
04/05/202	19	26/04/202	18	SP	GVE I CAPI	1331	SAO PAUL(	355030	HOSPITAL I
26/05/202	22	20/05/202	21	SE	REGIONAL	2056	ARACAJU	280030	HOSPITAL (
03/08/202	32	14/07/202	29	MT	CUIABA	1578	CUIABA	510340	HOSPITAL U
19/06/202	25	18/06/202	25	RJ			RIO DE JAN	330455	HOSPITAL I
23/06/202	26	15/06/202	25	SP	GVE XX PIR	1345	RIO CLARO	354390	SANTA CAS
17/12/202	51	12/12/202	50	SP	GVE XXV S/	1349	SANTOS	354850	HOSPITAL /
04/01/202	1	20/12/202	52	SP	GVE I CAPI	1331	SAO PAUL(	355030	PRO MATR
01/04/202	14	31/03/202	14	SP	GVE I CAPI	1331	SAO PAUL(	355030	HOSPITAL \

23/05/202	21	22/05/202	21	PE	001	1497	RECIFE	261160	IMIP
03/06/202	23	31/05/202	23	SP	GVE I CAPI	1331	SAO PAUL	355030	SAO LUIZ U
23/06/202	26	22/06/202	26	MG	JUIZ DE FO	1452	JUIZ DE FO	313670	SANTA CAS
06/07/202	28	20/06/202	25	MT	CUIABA	1578	CUIABA	510340	HOSPITAL
18/05/202	21	12/05/202	20	RJ			ITAPERUN	330220	HOSPITAL
11/08/202	33	16/07/202	29	PA	01 REGION	1484	BELEM	150140	HOSPITAL
25/08/202	35	22/08/202	34	RJ			RIO DE JAN	330455	SEMIU SER
23/03/202	13	14/03/202	11	SP	GVE I CAPI	1331	SAO PAUL	355030	HOSP SAO
09/01/202	1	02/01/202	53	MT	CUIABA	1578	CUIABA	510340	HOSPITAL
27/03/202	13	20/03/202	12	CE	1 CRES FOI	1519	FORTALEZ	230440	HOSPITAL
10/06/202	24	10/05/202	20	RJ			VOLTA RED	330630	SECRETARI
26/12/202	52	17/12/202	51	DF			BRASILIA	530010	HRAN
03/12/202	49	28/11/202	48	RJ			RIO DE JAN	330455	SMS HOSPI
06/07/202	28	27/06/202	26	GO	CENTRAL	1779	GOIANIA	520870	HOSPITAL
15/12/202	51	09/12/202	50	AM	ENTORNO	5584	MANAUS	130260	MATERNID
30/07/202	31	18/07/202	29	SP	GVE I CAPI	1331	SAO PAUL	355030	HC DA FMU
30/09/202	40	16/09/202	38	RS	001 CRS	1592	SAO LEOPO	431870	HOSPITAL
09/01/202	1	01/01/202	53	RJ			ITAPERUN	330220	HOSPITAL
01/07/202	27	25/06/202	26	BA	NUCLEO RI	1380	SALVADOR	292740	MATERNID
21/07/202	30	11/07/202	28	SP	GVE I CAPI	1331	SAO PAUL	355030	HC DA FMU
21/07/202	30	09/07/202	28	SP	GVE XXIX S	1354	SAO JOSE D	354980	HOSPITAL
13/11/202	46	28/10/202	44	PR	14RS PARA	1368	PARANAV	411840	SANTA CAS
19/07/202	30	16/07/202	29	SP	GVE XVII C	1342	HORTOLAN	351907	HOSPITAL
22/12/202	52	13/12/202	51	TO			PALMAS	172100	HOSPITAL
30/12/202	53	16/12/202	51	SP	GVE I CAPI	1331	SAO PAUL	355030	HOSPITAL
28/04/202	18	27/04/202	18	SP	GVE I CAPI	1331	SAO PAUL	355030	HOSPITAL
21/10/202	43	08/10/202	41	SP	GVE I CAPI	1331	SAO PAUL	355030	HOSPITAL
17/06/202	25	12/06/202	24	RJ			RIO DE JAN	330455	HOSPITAL
20/06/202	25	13/06/202	24	PI	4 GRS TERE	1883	TERESINA	221100	UNIDADE D
28/10/202	44	18/10/202	43	BA	NUCLEO RI	1380	SALVADOR	292740	MATERNID
19/11/202	47	14/11/202	46	RS	002 CRS	1607	PORTO ALE	431490	HOSPITAL
08/07/202	28	25/06/202	26	SP	GVE I CAPI	1331	SAO PAUL	355030	HOSPITAL
09/04/202	15	06/04/202	15	SP	GVE I CAPI	1331	SAO PAUL	355030	HOSPITAL
29/04/202	18	27/04/202	18	PE	001	1497	RECIFE	261160	HOSPITAL
13/01/202	2	28/12/202	53	AM	ENTORNO	5584	MANAUS	130260	MATERNID
14/10/202	42	07/10/202	41	AM	ENTORNO	5584	MANAUS	130260	MATERNID
29/04/202	18	23/04/202	17	RJ			SAO JOAO	330510	SES RJ HOS
25/03/202	13	19/03/202	12	RJ			RIO DE JAN	330455	SMS HOSPI
02/06/202	23	21/05/202	21	MG	CORONEL I	1468	IPATINGA	313130	HOSPITAL
19/06/202	25	12/06/202	24	CE	1 CRES FOI	1519	FORTALEZ	230440	HOSPITAL
25/06/202	26	10/06/202	24	RJ			RIO DE JAN	330455	MS HSE HC
09/06/202	24	04/06/202	23	GO	CENTRAL	1779	GOIANIA	520870	HOSPITAL
10/07/202	28	09/07/202	28	DF			BRASILIA	530010	DF STAR
19/07/202	30	10/07/202	28	BA	NUCLEO RI	1380	SALVADOR	292740	MATERNID
07/07/202	28	03/07/202	27	PI	4 GRS TERE	1883	TERESINA	221100	MATERNID
22/05/202	21	13/05/202	20	RJ			PETROPOLI	330390	HOSPITAL
21/08/202	34	15/08/202	33	SP	GVE XVII C	1342	HORTOLAN	351907	HOSPITAL

20/08/202	34	16/08/202	34	SP	GVE I CAPI	1331	SAO PAUL	355030	BP HOSPIT
11/11/202	46	04/11/202	45	SP	GVE XXV S	1349	CUBATAO	351350	HOSPITAL
25/10/202	44	14/10/202	42	SC	CRICIUMA	1558	CRICIUMA	420460	HOSPITAL
08/09/202	37	01/09/202	36	MT	CUIABA	1578	CUIABA	510340	HOSPITAL
16/04/202	16	06/04/202	15	RJ			RIO DE JAN	330455	MS HGB H
15/04/202	16	08/04/202	15	RJ			NILOPOLIS	330320	DOMINGO
06/06/202	23	02/06/202	23	DF			BRASILIA	530010	HOSPITAL
29/05/202	22	25/05/202	22	PA	09 REGION	1492	SANTAREM	150680	HOSPITAL
16/09/202	38	28/08/202	35	SP	GVE VII SA	1332	SANTO AN	354780	HOSPITAL
11/11/202	46	05/11/202	45	MG	BELO HORI	1449	BETIM	310670	HOSPITAL
18/11/202	47	20/06/202	25	AL	1 MICRORI	1533	MACEIO	270430	HOSPITAL
03/04/202	14	26/03/202	13	RS	016 CRS	1610	LAJEADO	431140	HOSPITAL
07/07/202	28	23/06/202	26	SP	GVE I CAPI	1331	SAO PAUL	355030	HC DA FMU
18/05/202	21	16/05/202	20	AM	ENTORNO	5584	MANAUS	130260	MATERNID
24/06/202	26	24/06/202	26	CE	1 CRES FOI	1519	FORTALEZ	230440	HGCC HOS
29/07/202	31	27/07/202	31	RS	002 CRS	1607	PORTO ALE	431490	HOSPITAL
11/08/202	33	30/07/202	31	PR	17RS LONE	1371	LONDRINA	411370	HOSPITAL
06/05/202	19	29/04/202	18	RJ			RIO DE JAN	330455	CLINICA SA
04/01/202	1	10/12/202	50	SP	GVE I CAPI	1331	SAO PAUL	355030	HOSPITAL
15/02/202	7	31/10/202	44	BA	NUCLEO RI	1380	SALVADOR	292740	HOSPITAL
26/05/202	22	12/05/202	20	SP	GVE I CAPI	1331	SAO PAUL	355030	HC DA FMU
08/05/202	19	03/05/202	19	SP	GVE I CAPI	1331	SAO PAUL	355030	MATERNID
05/06/202	23	01/06/202	23	PR	02RS METI	1356	CURITIBA	410690	COMPLEXC
02/06/202	23	21/05/202	21	SP	GVE I CAPI	1331	SAO PAUL	355030	HOSPITAL
21/08/202	34	16/08/202	34	AP			MACAPA	160030	HOSPITAL
30/05/202	22	12/05/202	20	SP	GVE I CAPI	1331	SAO PAUL	355030	HC DA FMU
31/12/202	53	25/12/202	52	DF			BRASILIA	530010	HOSPITAL
16/04/202	16	12/04/202	16	SP	GVE XVII C	1342	CAMPINAS	350950	MATERNID
24/06/202	26	16/06/202	25	SC	JARAGUA I	1567	JARAGUA I	420890	HOSPITAL
16/06/202	25	14/06/202	25	SP	GVE I CAPI	1331	SAO PAUL	355030	HOSPITAL
17/08/202	34	06/08/202	32	GO	CENTRAL	1779	GOIANIA	520870	HOSPITAL
18/09/202	38	13/09/202	38	SP	GVE XXIX S	1354	SAO JOSE I	354980	HOSPITAL
31/07/202	31	23/07/202	30	PR	07RS PATO	1361	PATO BRAI	411850	ISSAL
07/08/202	32	30/07/202	31	RJ			RESENDE	330420	HOSPITAL
14/08/202	33	10/08/202	33	MS	EX ARS DE	1975	CAMPO GI	500270	HOSPITAL
09/12/202	50	25/11/202	48	RS	002 CRS	1607	PORTO ALE	431490	IRMANDAI
27/09/202	40	19/09/202	38	MS	EX ARS DE	1975	CAMPO GI	500270	HOSPITAL
06/01/202	1	24/12/202	52	SP	GVE I CAPI	1331	SAO PAUL	355030	SAO LUIZ U
30/04/202	18	22/04/202	17	SP	GVE I CAPI	1331	SAO PAUL	355030	HOSPITAL
26/11/202	48	17/10/202	42	RJ			CAMPOS D	330100	HOSPITAL
06/05/202	19	05/05/202	19	CE	1 CRES FOI	1519	FORTALEZ	230440	HGCC HOS
13/07/202	29	05/07/202	28	SP	GVE XII AR	1337	SAO CARL	354890	SANTA CAS
05/08/202	32	27/07/202	31	SP	GVE XXIV F	1348	RIBEIRAO F	354340	HOSPITAL
19/05/202	21	11/05/202	20	AM	ENTORNO	5584	MANAUS	130260	MATERNID
08/06/202	24	04/06/202	23	SP	GVE VII SA	1332	SAO CAETA	354880	COMPLEXC
16/12/202	51	04/12/202	49	SP	GVE X OSA	1335	BARUERI	350570	PS E MATE
09/08/202	33	04/08/202	32	BA	NUCLEO RI	1380	SALVADOR	292740	MATERNID

19/07/202(	30	12/07/202(	29	SP	GVE I CAPI'	1331	SAO PAUL(	355030	SAO LUIZ U	
27/09/202(	40	23/09/202(	39	RS	012 CRS	1601	SANTO AN(	431750	HOSPITAL S	
13/07/202(	29	12/07/202(	29	SP	GVE VII SAI	1332	SAO BERN/	354870	HOSPITAL I	
01/01/202	53	23/12/202(	52	PR	10RS CASC	1364	CASCAVEL	410480	HOSPITAL U	
04/06/202(	23	27/05/202(	22	GO	CENTRAL	1779	GOIANIA	520870	HOSPITAL I	
12/05/202(	20	07/05/202(	19	AL	1 MICRORI	1533	MACEIO	270430	HOSPITAL U	
25/10/202(	44	20/10/202(	43	GO	CENTRAL	1779	GOIANIA	520870	HOSPITAL I	
28/08/202(	35	20/08/202(	34	MG	JUIZ DE FO	1452	JUIZ DE FO	313670	HOSPITAL I	
12/12/202(	50	09/12/202(	50	PR	02RS METF	1356	CAMPO LA	410420	HOSPITAL I	
09/12/202(	50	27/11/202(	48	SP	GVE I CAPI'	1331	SAO PAUL(	355030	HOSPITAL I	
09/09/202(	37	03/09/202(	36	SP	GVE VIII M	1333	GUARULH(	351880	HOSPITAL (	
01/09/202(	36	28/08/202(	35	PB	III NRS CAN	1421	CAMPINA (	250400	INSTITUTO	
27/08/202(	35	20/08/202(	34	DF			BRASILIA	530010	HRT HOSPI	
12/09/202(	37	12/09/202(	37	RJ			TERESOPOI	330580	HOSPITAL I	
03/11/202(	45	22/10/202(	43	SP	GVE I CAPI'	1331	SAO PAUL(	355030	HC DA FMU	
05/05/202(	19	26/04/202(	18	PR	02RS METF	1356	CURITIBA	410690	HNSG	
29/05/202(	22	26/05/202(	22	SP	GVE XXV S/	1349	SANTOS	354850	COMPLEX(	
24/08/202(	35	20/08/202(	34	RJ			RIO DE JAN	330455	MS HGB H(	
15/04/202(	16	08/04/202(	15	PE	001	1497	RECIFE	261160	IMIP	
25/07/202(	30	15/07/202(	29	BA	NUCLEO RI	1380	SALVADOR	292740	MATERNID	
05/12/202(	49	25/11/202(	48	MG	GOVERNAI	1471	GOVERNAI	312770	HOSPITAL I	
27/08/202(	35	26/08/202(	35	BA	NUCLEO RI	1380	SALVADOR	292740	MATERNID	
28/10/202(	44	24/10/202(	43	BA	NUCLEO RI	1380	SALVADOR	292740	MATERNID	
31/07/202(	31	30/07/202(	31	GO	CENTRAL	1779	GOIANIA	520870	HOSPITAL I	
27/07/202(	31	10/07/202(	28	MT	CUIABA	1578	CUIABA	510340	HOSPITAL S	
27/07/202(	31	23/07/202(	30	SP	GVE X OSA	1335	ITAPECERI(	352220	HOSPITAL (	
31/07/202(	31	21/07/202(	30	MG	BELO HORI	1449	CONTAGEN	311860	HOSPITAL I	
22/12/202(	52	08/12/202(	50	RJ			RIO DE JAN	330455	SMS HOSPI	
31/12/202(	53	22/12/202(	52	RJ			NITEROI	330330	SES RJ HOS	
09/06/202(	24	28/05/202(	22	SP	GVE I CAPI'	1331	SAO PAUL(	355030	HOSPITAL S	
12/05/202(	20	28/04/202(	18	RJ			NILOPOLIS	330320	DOMINGO.	
05/10/202(	41	22/09/202(	39	SP	GVE XI AR/	1336	BIRIGUI	350650	SANTA CAS	
17/12/202(	51	26/08/202(	35	AL	1 MICRORI	1533	MACEIO	270430	HOSPITAL U	
20/11/202(	47	15/11/202(	47	SP	GVE XXII PI	1573	PRESIDENT	354150	SANTA CAS	
12/03/202	10	07/07/202(	28	AL	1 MICRORI	1533	MACEIO	270430	HOSPITAL I	
25/05/202(	22	24/05/202(	22	RJ			RIO DE JAN	330455	HOSPITAL I	
22/06/202(	26	18/06/202(	25	BA	NUCLEO RI	1380	SALVADOR	292740	MATERNID	
26/06/202(	26	20/06/202(	25	SP	GVE XVIII F	1343	SAO JOAQU	354940	SANTA CAS	
16/07/202(	29	12/07/202(	29	PE	001	1497	RECIFE	261160	IMIP	
15/09/202(	38	05/09/202(	36	RS	002 CRS	1607	PORTO ALE	431490	HOSPITAL I	
28/08/202(	35	20/08/202(	34	SP	GVE I CAPI'	1331	SAO PAUL(	355030	HC DA FMU	
13/01/202	2	28/12/202(	53	AM	ENTORNO	5584	MANAUS	130260	MATERNID	
26/05/202(	22	22/05/202(	21	MA	REGI0NAL	1430	SAO LUIS	211130	MATERNID	
18/08/202(	34	17/08/202(	34	MG	BELO HORI	1449	BELO HORI	310620	HOSPITAL I	
18/06/202(	25	14/06/202(	25	RN	VII URSAP	1416	NATAL	240810	HOSPITAL I	
03/09/202(	36	28/08/202(	35	SP	GVE I CAPI'	1331	SAO PAUL(	355030	HOSPITAL S	
09/10/202(	41	19/09/202(	38	DF			BRASILIA	530010	HOSPITAL S	

08/05/202	19	29/04/202	18	CE	1 CRES FOR	1519	FORTALEZA	230440	HGCC HOS
29/07/202	31	15/07/202	29	SP	GVE I CAPI	1331	SAO PAULO	355030	HC DA FMU
31/07/202	31	25/07/202	30	GO	CENTRAL	1779	GOIANIA	520870	HOSPITAL
12/07/202	29	28/06/202	27	MG	GOVERNAD	1471	GOVERNAD	312770	HOSPITAL
27/04/202	18	13/04/202	16	RJ			RIO DE JAN	330455	SMS HOSP
25/06/202	26	14/06/202	25	PR	02RS METR	1356	CURITIBA	410690	COMPLEXO
10/07/202	28	10/07/202	28	MT	CUIABA	1578	CUIABA	510340	HOSPITAL
02/10/202	40	26/09/202	39	SP	GVE I CAPI	1331	SAO PAULO	355030	HOSPITAL
21/09/202	39	17/09/202	38	SP	GVE I CAPI	1331	SAO PAULO	355030	NEXT HOSP
11/11/202	46	05/11/202	45	PR	20RS TOLE	1374	TOLEDO	412770	CLINICA IN
19/10/202	43	16/10/202	42	BA	NUCLEO RE	1380	SALVADOR	292740	MATERNID
07/11/202	45	29/10/202	44	PA	01 REGION	1484	BELEM	150140	MATERNID
09/06/202	24	05/06/202	23	BA	NUCLEO RE	1380	SALVADOR	292740	MATERNID
19/04/202	17	14/04/202	16	ES	METROPOL	1510	SERRA	320500	HOSPITAL
10/06/202	24	02/06/202	23	RJ			CAMPOS D	330100	HOSPITAL
19/10/202	43	01/10/202	40	SP	GVE I CAPI	1331	SAO PAULO	355030	HOSPITAL
15/09/202	38	08/09/202	37	PA	01 REGION	1484	BELEM	150140	SANTA CAS
03/01/202	1	28/12/202	53	PR	10RS CASC	1364	CASCAVEL	410480	HOSPITAL
08/12/202	50	04/12/202	49	BA	NUCLEO RE	1380	SALVADOR	292740	MATERNID
02/07/202	27	26/06/202	26	PA	11 REGION	1494	PARAUAPE	150553	HOSPITAL
22/04/202	17	16/04/202	16	RJ			RIO DE JAN	330455	HOSPITAL
21/05/202	21	18/05/202	21	BA	NUCLEO RE	1380	SALVADOR	292740	HOSPITAL
12/06/202	24	12/06/202	24	CE	13 CRES TIA	1523	VICOSA DO	231410	HOSP MAT
02/07/202	27	20/06/202	25	DF			BRASILIA	530010	HOSPITAL
20/07/202	30	19/07/202	30	PR	17RS LOND	1371	LONDRINA	411370	ISCAL
15/10/202	42	04/10/202	41	MT	RONDONO	1579	CAMPO VE	510267	HOSPITAL
03/11/202	45	20/10/202	43	GO	CENTRAL	1779	GOIANIA	520870	CLIAME
13/06/202	24	06/06/202	23	SP	GVE I CAPI	1331	SAO PAULO	355030	HOSPITAL
16/12/202	51	11/12/202	50	BA	NUCLEO RE	1380	SALVADOR	292740	HOSPITAL
20/05/202	21	19/05/202	21	SP	GVE X OSA	1335	TABOAO D	355280	HOSPITAL
17/04/202	16	13/04/202	16	SP	GVE I CAPI	1331	SAO PAULO	355030	HOSPITAL
26/05/202	22	11/05/202	20	AM	ENTORNO	5584	MANAUS	130260	MATERNID
08/10/202	41	25/09/202	39	SP	GVE XXIV R	1348	RIBEIRAO P	354340	HOSPITAL
29/09/202	40	27/09/202	40	RJ			RIO DE JAN	330455	MS HSE HO
11/07/202	28	04/07/202	27	RJ			RIO DE JAN	330455	PERINATAL
24/05/202	22	24/05/202	22	SP	GVE I CAPI	1331	SAO PAULO	355030	HOSPITAL
22/07/202	30	20/07/202	30	GO	CENTRAL	1779	GOIANIA	520870	HOSPITAL
07/05/202	19	30/04/202	18	PI	4 GRS TERE	1883	TERESINA	221100	MATERNID
16/11/202	47	04/11/202	45	SC	JOINVILLE	1565	JOINVILLE	420910	HOSPITAL
09/06/202	24	26/05/202	22	PA	12 REGION	1495	REDENCAO	150613	HOSPITAL
21/12/202	52	08/12/202	50	SP	GVE XXIV R	1348	RIBEIRAO P	354340	HOSPITAL
03/05/202	19	27/04/202	18	MS	EX ARS DE	1975	CAMPO GR	500270	HOSPITAL
31/05/202	23	22/05/202	21	RJ			CAMPOS D	330100	HOSPITAL
14/07/202	29	06/07/202	28	SP	GVE I CAPI	1331	SAO PAULO	355030	HC DA FMU
14/07/202	29	11/07/202	28	MT	JUINA	1582	ARIPUANA	510140	HOSPITAL
10/07/202	28	05/07/202	28	PR	02RS METR	1356	CURITIBA	410690	COMPLEXO
21/07/202	30	05/05/202	19	MA	REGI0NAL	1430	SAO LUIS	211130	MATERNID

08/06/202	24	02/06/202	23	MT	CUIABA	1578	CUIABA	510340	HOSPITAL S
04/08/202	32	25/07/202	30	RS	002 CRS	1607	PORTO ALE	431490	HOSPITAL I
11/07/202	28	10/07/202	28	BA	NUCLEO RE	1382	ALAGOINH	290070	HOSPITAL I
31/08/202	36	29/08/202	35	BA	NUCLEO RE	1380	SALVADOR	292740	MATERNID
25/08/202	35	22/08/202	34	AM	ENTORNO	5584	MANAUS	130260	MATERNID
14/07/202	29	13/07/202	29	PE	001	1497	RECIFE	261160	IMIP
02/04/202	14	24/03/202	13	SP	GVE XXV SA	1349	SANTOS	354850	SANTA CAS
23/04/202	17	13/04/202	16	RJ			RIO DE JAN	330455	SMS HOSPI
25/07/202	30	19/07/202	30	SP	GVE XXIX S	1354	SAO JOSE D	354980	HOSPITAL I
20/05/202	21	17/05/202	21	SP	GVE I CAPI	1331	SAO PAULO	355030	SAO LUIZ U
22/05/202	21	17/05/202	21	SP	GVE I CAPI	1331	SAO PAULO	355030	HOSPITAL I
17/07/202	29	09/07/202	28	SP	GVE I CAPI	1331	SAO PAULO	355030	HOSP MUN
23/11/202	48	10/11/202	46	AM	BAIXO AMA	5589	BOA VISTA	130068	UNIDADE H
03/08/202	32	28/07/202	31	RJ			NILOPOLIS	330320	DOMINGOS
24/04/202	17	13/04/202	16	MA	REGI0NAL	1430	SAO LUIS	211130	EBSERH HO
26/04/202	18	21/04/202	17	RJ			RIO DE JAN	330455	HOSPITAL (
04/12/202	49	27/11/202	48	SC	JOINVILLE	1565	JOINVILLE	420910	HOSPITAL I
21/06/202	26	18/06/202	25	GO	CENTRAL	1779	GOIANIA	520870	HOSPITAL I
03/11/202	45	27/10/202	44	RS	017 CRS	1604	IJUI	431020	HOSPITAL I
16/05/202	20	11/05/202	20	RJ			RIO DE JAN	330455	SES RJ UPA
26/05/202	22	18/05/202	21	PI	4 GRS TERE	1883	TERESINA	221100	MATERNID
25/06/202	26	18/06/202	25	PI	4 GRS TERE	1883	TERESINA	221100	MATERNID
21/08/202	34	10/08/202	33	SP	GVE VII SAI	1332	DIADEMA	351380	HOSPITAL I
17/12/202	51	10/12/202	50	MG	BELO HORI	1449	BELO HORI	310620	HOSPITAL J
29/09/202	40	26/09/202	39	PI	4 GRS TERE	1883	TERESINA	221100	MATERNID
07/05/202	19	05/05/202	19	BA	NUCLEO RE	1380	SALVADOR	292740	MATERNID
03/06/202	23	27/05/202	22	SP	GVE I CAPI	1331	SAO PAULO	355030	HC DA FMU
15/06/202	25	07/06/202	24	PR	10RS CASC	1364	CASCAVEL	410480	HOSPITAL U
28/03/202	13	23/03/202	13	AM	ENTORNO	5584	MANAUS	130260	HOSPITAL S
14/09/202	38	04/09/202	36	SP	GVE XVII CA	1342	AMERICAN	350160	HOSPITAL S
17/11/202	47	13/11/202	46	BA	NUCLEO RE	1381	FEIRA DE SA	291080	HOSPITAL I
22/11/202	48	15/11/202	47	RJ			RIO DE JAN	330455	SMS HOSPI
06/05/202	19	27/04/202	18	AM	ENTORNO	5584	MANAUS	130260	SAMEL
18/06/202	25	14/06/202	25	BA	NUCLEO RE	1380	SALVADOR	292740	MATERNID
28/10/202	44	02/07/202	27	RJ			RIO DE JAN	330455	SMS COOR
27/06/202	26	23/06/202	26	BA	NUCLEO RE	1380	SALVADOR	292740	MATERNID
05/06/202	23	27/05/202	22	SP	GVE I CAPI	1331	SAO PAULO	355030	HC DA FMU
20/07/202	30	16/07/202	29	SP	GVE VIII M	1333	GUARULHO	351880	HOSPITAL (
18/09/202	38	30/05/202	22	BA	NUCLEO RE	1380	SALVADOR	292740	HOSPITAL S
06/05/202	19	22/04/202	17	RJ			RIO DE JAN	330455	MS HSE HO
09/11/202	46	05/11/202	45	RJ			RIO DE JAN	330455	HOSPITAL (
25/11/202	48	19/11/202	47	RJ			NILOPOLIS	330320	DOMINGOS
11/12/202	50	09/12/202	50	PR	19RS JACAI	1373	JOAQUIM T	411280	HOSPITAL (
29/04/202	18	18/04/202	16	SP	GVE I CAPI	1331	SAO PAULO	355030	HC DA FMU
29/04/202	18	22/04/202	17	CE	1 CRES FOR	1519	FORTALEZA	230440	HGF HOSPI
23/06/202	26	17/06/202	25	MG	GOVERNAD	1471	GOVERNAD	312770	HOSPITAL I
08/06/202	24	29/05/202	22	RN	VII URSAP	1416	NATAL	240810	HOSPITAL I

01/07/202	27	22/06/202	26	SE	REGIONAL	2057	ESTANCIA	280210	HOSPITAL I
30/11/202	49	22/11/202	48	SP	GVE I CAPI	1331	SAO PAULO	355030	SAO LUIZ U
13/04/202	16	02/04/202	14	SP	GVE I CAPI	1331	SAO PAULO	355030	HOSPITAL I
27/09/202	40	17/09/202	38	RJ			NITEROI	330330	CHN COMP
01/04/202	14	29/03/202	14	RJ			RIO DE JAN	330455	SMS RIO H
20/10/202	43	01/10/202	40	SP	GVE I CAPI	1331	SAO PAULO	355030	INSTITUTO
08/10/202	41	02/10/202	40	GO	CENTRAL	1779	GOIANIA	520870	HOSPITAL I
07/10/202	41	06/10/202	41	AM	ENTORNO	5584	MANAUS	130260	HOSPITAL I
06/07/202	28	05/07/202	28	CE	1 CRES FOR	1519	FORTALEZA	230440	HGCC HOS
27/04/202	18	27/04/202	18	RJ			RIO DE JAN	330455	MS HGB H
11/07/202	28	11/07/202	28	SP	GVE VII SA	1332	SANTO AN	354780	CENTRO H
06/05/202	19	26/04/202	18	CE	1 CRES FOR	1519	FORTALEZA	230440	HGCC HOS
19/06/202	25	10/06/202	24	PR	20RS TOLE	1374	GUAIRA	410880	UNIDADE C
16/04/202	16	13/04/202	16	RJ			RIO DE JAN	330455	MS HGB H
28/06/202	27	19/06/202	25	BA	NUCLEO RE	1380	SALVADOR	292740	MATERNID
20/06/202	25	23/05/202	21	SP	GVE I CAPI	1331	SAO PAULO	355030	HOSP MUN
17/04/202	16	11/04/202	15	MA	REGI0NAL	1430	SAO LUIS	211130	EBSERH HC
23/12/202	52	13/12/202	51	GO	CENTRAL	1779	GOIANIA	520870	HOSPITAL I
24/11/202	48	23/11/202	48	RJ			RIO DE JAN	330455	SMS HOSPI
10/04/202	15	04/04/202	14	AM	ENTORNO	5584	MANAUS	130260	HOSPITAL A
23/07/202	30	13/07/202	29	RS	003 CRS	1593	PELOTAS	431440	HOSPITAL I
31/08/202	36	25/08/202	35	SP	GVE XXIX S	1354	CATANDUV	351110	UNIMED H
18/07/202	29	17/07/202	29	SP	GVE XXV SA	1349	BERTIOGA	350635	UNIDADE D
15/08/202	33	12/08/202	33	MG	POUSO ALE	1459	ITAJUBA	313240	AISI HOSPI
20/01/202	3	02/01/202	53	SP	GVE X OSA	1335	OSASCO	353440	HOSPITAL S
27/12/202	53	25/12/202	52	SP	GVE XXVII S	1351	SAO JOSE D	354990	HOSPITAL V
30/12/202	53	16/12/202	51	SP	GVE XX PIR	1345	RIO CLARO	354390	SANTA CAS
08/06/202	24	30/05/202	22	SP	GVE XVII CA	1342	VALINHOS	355620	HOSPITAL I
06/01/202	1	04/12/202	49	RJ			RIO DE JAN	330455	HOSPITAL I
29/08/202	35	15/08/202	33	DF			BRASILIA	530010	HRG
03/01/202	1	27/12/202	53	RS	002 CRS	1607	PORTO ALE	431490	HOSPITAL I
10/05/202	20	10/05/202	20	SP	GVE I CAPI	1331	SAO PAULO	355030	HOSPITAL S
23/04/202	17	19/04/202	17	PE	001	1497	RECIFE	261160	IMIP
11/05/202	20	05/05/202	19	AM	ENTORNO	5584	PRESIDENT	130353	HOSPITAL (
08/05/202	19	30/04/202	18	SP	GVE I CAPI	1331	SAO PAULO	355030	HOSPITAL S
17/05/202	21	10/05/202	20	CE	1 CRES FOR	1519	FORTALEZA	230440	MATERNID
09/06/202	24	30/05/202	22	RS	005 CRS	1608	CAXIAS DO	430510	HOSPITAL U
16/06/202	25	04/06/202	23	SP	GVE XXV SA	1349	GUARUJA	351870	HOSPITAL S
12/01/202	2	30/12/202	53	SP	GVE I CAPI	1331	SAO PAULO	355030	MATERNID
28/06/202	27	20/06/202	25	ES	METROPOL	1510	SERRA	320500	HOSPITAL I
11/08/202	33	08/08/202	32	RJ			ANGRA DO	330010	HOSPITAL I
30/07/202	31	22/07/202	30	MT	CUIABA	1578	CUIABA	510340	HOSPITAL S
10/09/202	37	04/09/202	36	SP	GVE XXIX S	1354	SAO JOSE D	354980	HOSPITAL I
15/12/202	51	20/11/202	47	AM	ENTORNO	5584	MANAUS	130260	MATERNID
02/11/202	45	27/10/202	44	PE	001	1497	RECIFE	261160	IMIP
17/09/202	38	02/06/202	23	BA	NUCLEO RE	1380	SALVADOR	292740	HOSPITAL S
05/09/202	36	03/09/202	36	PB	I NRS JOAO	1377	JOAO PESS	250750	MATERNID

04/05/202	19	27/04/202	18	SP	GVE I CAPI	1331	SAO PAUL	355030	HOSPITAL
24/06/202	26	16/06/202	25	PI	4 GRS TERE	1883	TERESINA	221100	MATERNID
06/07/202	28	30/05/202	22	AP			MACAPA	160030	HOSPITAL
20/07/202	30	18/07/202	29	SP	GVE XVII C	1342	AMPARO	350190	SANTA CAS
26/10/202	44	20/10/202	43	SP	GVE I CAPI	1331	SAO PAUL	355030	BP HOSPIT
29/08/202	35	23/08/202	35	MT	CUIABA	1578	CUIABA	510340	HOSPITAL
30/07/202	31	13/07/202	29	SP	GVE I CAPI	1331	SAO PAUL	355030	HOSPITAL
06/05/202	19	02/05/202	18	PA	01 REGION	1484	BELEM	150140	SANTA CAS
24/08/202	35	17/08/202	34	RJ			RESENDE	330420	HOSPITAL
09/12/202	50	06/12/202	50	MG	UBERLAND	1462	UBERLAND	317020	HOSPITAL
10/10/202	41	05/10/202	41	SP	GVE I CAPI	1331	SAO PAUL	355030	HOSP MUN
13/06/202	24	07/06/202	24	SP	GVE XIX M	1344	MARILIA	352900	HOSPITAL
16/10/202	42	15/10/202	42	PB	I NRS JOAC	1377	JOAO PESS	250750	HOSPITAL
11/01/202	2	02/01/202	53	RJ			NITEROI	330330	CHN COMP
06/06/202	23	03/06/202	23	SP	GVE XVII C	1342	CAMPINAS	350950	HOSPITAL
04/09/202	36	03/09/202	36	RS	002 CRS	1607	PORTO ALE	431490	HOSPITAL
24/08/202	35	16/08/202	34	SP	GVE I CAPI	1331	SAO PAUL	355030	SAO LUIZ U
14/12/202	51	07/12/202	50	RJ			RIO DE JAN	330455	MS HSE HC
06/01/202	1	29/12/202	53	SP	GVE I CAPI	1331	SAO PAUL	355030	HOSPITAL
25/05/202	22	21/05/202	21	RN	II URSAP	1412	MOSSORO	240800	HOSPITAL
19/07/202	30	16/07/202	29	BA	NUCLEO RI	1398	VITORIA D	293330	SAMUR
27/07/202	31	20/07/202	30	SP	GVE X OSA	1335	BARUERI	350570	HOSPITALI
24/07/202	30	09/07/202	28	PR	02RS METF	1356	CURITIBA	410690	HOSPITAL
23/08/202	35	21/08/202	34	PR	17RS LOND	1371	LONDRINA	411370	HOSPITAL
13/05/202	20	12/05/202	20	PE	001	1497	RECIFE	261160	IMIP
27/04/202	18	22/04/202	17	RJ			RIO DE JAN	330455	CASA DE S
05/06/202	23	27/05/202	22	CE	20 CRES CF	1530	CRATO	230420	HOSPITAL
01/06/202	23	28/05/202	22	PA	09 REGION	1492	SANTAREM	150680	HOSPITAL
12/07/202	29	03/07/202	27	PR	02RS METF	1356	CAMPINA	410400	HOSPITAL
21/08/202	34	12/08/202	33	MG	DIVINOPOI	1472	DIVINOPOI	312230	HOSPITAL
16/12/202	51	07/12/202	50	SP	GVE XVII C	1342	CAMPINAS	350950	HOSPITAL
15/01/202	2	31/12/202	53	GO	CENTRAL	1779	GOIANIA	520870	HOSPITAL
02/06/202	23	29/05/202	22	SP	GVE I CAPI	1331	SAO PAUL	355030	SAO LUIZ U
15/07/202	29	07/07/202	28	SP	GVE XXIX S	1354	SAO JOSE D	354980	HOSPITAL
01/12/202	49	14/11/202	46	RJ			RIO DE JAN	330455	HOSPITAL
19/10/202	43	09/10/202	41	SP	GVE VII SA	1332	SAO CAETA	354880	HOSPITAL
22/04/202	17	18/04/202	16	RJ			RIO DE JAN	330455	HOSPITAL
27/06/202	26	18/06/202	25	AL	1 MICRORF	1533	MACEIO	270430	HOSPITAL
29/04/202	18	26/04/202	18	PB	I NRS JOAC	1377	JOAO PESS	250750	MATERNID
15/06/202	25	09/06/202	24	SP	GVE I CAPI	1331	SAO PAUL	355030	HC DA FMU
22/08/202	34	06/07/202	28	PB	I NRS JOAC	1377	JOAO PESS	250750	MATERNID
21/10/202	43	17/10/202	42	SC	FLORIANOI	1476	FLORIANOI	420540	HOSPITAL
06/10/202	41	29/09/202	40	RS	010 CRS	1599	ALEGRETE	430040	SANTA CAS
14/12/202	51	06/12/202	50	MG	POUSO ALI	1459	ITAJUBA	313240	AISI HOSPI
02/10/202	40	26/09/202	39	SP	GVE VII SA	1332	SAO CAETA	354880	HOSPITAL
07/01/202	1	26/12/202	52	PR	15RS MARI	1369	MARINGA	411520	SANTA CAS
08/04/202	14	11/11/202	46	RJ			RIO DE JAN	330455	HOSPITAL

19/05/202	21	11/05/202	20	SP	GVE I CAPI	1331	SAO PAUL	355030	HOSPITAL
10/07/202	28	03/07/202	27	MG	POUSO ALI	1459	ITAJUBA	313240	AISI HOSPI
29/12/202	53	14/12/202	51	CE	1 CRES FOI	1519	FORTALEZ	230440	HOSPITAL
28/05/202	22	07/05/202	19	BA	NUCLEO RI	1380	SALVADOR	292740	HOSPITAL
19/07/202	30	04/07/202	27	SP	GVE XXV S	1349	SANTOS	354850	HOSPITAL
31/07/202	31	22/06/202	26	RJ			RIO DE JAN	330455	UERJ HOSP
01/10/202	40	23/09/202	39	PR	02RS METI	1356	CURITIBA	410690	COMPLEXO
13/11/202	46	01/11/202	45	PE	001	1497	RECIFE	261160	REAL HOSP
25/08/202	35	25/08/202	35	GO	SAO PATRI	6252	JARAGUA	521180	HOSPITAL
23/05/202	21	20/05/202	21	RJ			RIO DE JAN	330455	SMS HOSPI
20/10/202	43	19/10/202	43	RJ			CAMPOS D	330100	SOCIEDADI
01/05/202	18	21/04/202	17	SP	GVE I CAPI	1331	SAO PAUL	355030	HC DA FMU
12/06/202	24	05/06/202	23	SP	GVE XXIX S	1354	SAO JOSE I	354980	HOSPITAL
03/07/202	27	19/06/202	25	DF			BRASILIA	530010	HOSPITAL
04/01/202	1	22/12/202	52	SP	GVE I CAPI	1331	SAO PAUL	355030	HC DA FMU
08/10/202	41	31/08/202	36	MA	REGI0NAL	1430	SAO LUIS	211130	HOSPITAL
04/09/202	36	04/09/202	36	PE	001	1497	RECIFE	261160	IMIP
22/06/202	26	15/06/202	25	MT	CUIABA	1578	CUIABA	510340	HOSPITAL
10/06/202	24	21/05/202	21	SP	GVE I CAPI	1331	SAO PAUL	355030	HC DA FMU
04/01/202	1	01/01/202	53	SP	GVE I CAPI	1331	SAO PAUL	355030	PRO MATR
20/08/202	34	10/08/202	33	SP	GVE XVII C	1342	NOVA ODE	353340	HOSPITAL
29/06/202	27	29/06/202	27	PA	01 REGION	1484	BELEM	150140	SANTA CAS
24/05/202	22	20/05/202	21	SP	GVE I CAPI	1331	SAO PAUL	355030	SAO LUIZ U
22/04/202	17	12/04/202	16	SP	GVE I CAPI	1331	SAO PAUL	355030	HC DA FMU
14/08/202	33	03/08/202	32	PA	11 REGION	1494	PARAUAPE	150553	HOSPITAL
17/07/202	29	08/07/202	28	SP	GVE XII AR	1337	IBATE	351930	HOSPITAL
26/04/202	18	24/04/202	17	PB	I NRS JOAC	1377	JOAO PESS	250750	MATERNID
27/07/202	31	18/07/202	29	MG	UBERLAND	1462	UBERLAND	317020	HOSPITAL
29/06/202	27	25/06/202	26	GO	CENTRAL	1779	GOIANIA	520870	HOSPITAL
11/05/202	20	07/05/202	19	SP	GVE I CAPI	1331	SAO PAUL	355030	HOSPITAL
23/07/202	30	20/07/202	30	SP	GVE XII AR	1337	PORTO FEI	354070	HOSPITAL
08/06/202	24	04/06/202	23	MA	REGI0NAL	1430	SAO LUIS	211130	HOSPITAL
29/10/202	44	22/10/202	43	PA	11 REGION	1494	MARABA	150420	CLIMEC
23/05/202	21	18/05/202	21	RN	II URSAP	1412	MOSSORO	240800	HOSPITAL
23/11/202	48	16/11/202	47	PR	02RS METI	1356	CURITIBA	410690	COMPLEXO
04/02/202	5	31/12/202	53	CE	1 CRES FOI	1519	FORTALEZ	230440	HOSPITAL
04/07/202	27	03/07/202	27	PE	001	1497	RECIFE	261160	IMIP
22/07/202	30	18/07/202	29	MG	BELO HORI	1449	BELO HORI	310620	HOSPITAL
16/05/202	20	16/05/202	20	CE	1 CRES FOI	1519	FORTALEZ	230440	HGCC HOS
11/01/202	2	18/12/202	51	AL	1 MICRORI	1533	MACEIO	270430	HOSPITAL
15/04/202	16	12/04/202	16	SP	GVE I CAPI	1331	SAO PAUL	355030	HOSPITAL
10/04/202	15	06/04/202	15	SP	GVE I CAPI	1331	SAO PAUL	355030	HC DA FMU
23/12/202	52	14/12/202	51	PR	02RS METI	1356	CAMPO LA	410420	HOSPITAL
12/10/202	42	04/10/202	41	SP	GVE XXIX S	1354	SAO JOSE I	354980	HOSPITAL
28/04/202	18	18/04/202	16	RJ			RIO DE JAN	330455	CLINICA SA
07/11/202	45	04/11/202	45	MG	PONTE NO	1458	PONTE NO	315210	HOSPITAL
28/12/202	53	24/12/202	52	PR	02RS METI	1356	CAMPINA	410400	HOSPITAL

15/05/202	20	14/05/202	20	PB	III NRS CAN	1421	CAMPINA	250400	INSTITUTO
15/08/202	33	04/08/202	32	SP	GVE XXIX S	1354	CATANDU	351110	UNIMED H
26/05/202	22	14/05/202	20	SP	GVE I CAPI	1331	SAO PAUL	355030	BP HOSPIT
20/07/202	30	12/07/202	29	SP	GVE IX FRA	1334	CAIEIRAS	350900	HOSPITAL
03/08/202	32	27/07/202	31	PE	001	1497	RECIFE	261160	IMIP
24/11/202	48	21/11/202	47	RJ			RIO DE JAN	330455	SMS HOSP
29/09/202	40	19/08/202	34	PA	01 REGION	1484	BELEM	150140	SANTA CAS
21/01/202	3	06/12/202	50	RJ			RIO DE JAN	330455	PERINATAL
17/11/202	47	08/11/202	46	SP	GVE XVII C	1342	JUNDIAI	352590	HOSPITAL
16/04/202	16	16/04/202	16	SP	GVE XXV S	1349	SANTOS	354850	HOSPITAL
19/05/202	21	09/05/202	19	SP	GVE I CAPI	1331	SAO PAUL	355030	SANTA CAS
30/03/202	14	26/03/202	13	SP	GVE I CAPI	1331	SAO PAUL	355030	MATERNID
11/07/202	28	11/07/202	28	CE	1 CRES FOI	1519	FORTALEZ	230440	HGCC HOS
18/05/202	21	13/05/202	20	SP	GVE XXVI S	1350	MOCOCA	353050	SANTA CAS
01/06/202	23	26/05/202	22	RR			BOA VISTA	140010	HOSPITAL
26/09/202	39	17/09/202	38	SP	GVE I CAPI	1331	SAO PAUL	355030	HOSPITAL
23/05/202	21	13/05/202	20	SP	GVE I CAPI	1331	SAO PAUL	355030	HC DA FM
23/03/202	13	15/03/202	12	SP	GVE I CAPI	1331	SAO PAUL	355030	SAO LUIZ U
20/07/202	30	17/07/202	29	MG	UBERLAND	1462	UBERLAND	317020	HOSPITAL
08/06/202	24	28/05/202	22	SP	GVE I CAPI	1331	SAO PAUL	355030	HC DA FM
29/12/202	53	12/12/202	50	CE	1 CRES FOI	1519	FORTALEZ	230440	HOSPITAL
06/07/202	28	03/07/202	27	AM	ENTORNO	5584	MANAUS	130260	MATERNID
26/05/202	21	15/11/202	47	SP	GVE VIII M	1333	MOGI DAS	353060	SECRETARI
22/07/202	30	10/07/202	28	MT	CUIABA	1578	CUIABA	510340	HOSPITAL
27/11/202	48	25/11/202	48	RJ			RIO DE JAN	330455	SMS HOSP
24/12/202	52	16/12/202	51	ES	METROPOI	1510	SERRA	320500	HOSPITAL
11/08/202	33	25/07/202	30	RJ			CAMPOS D	330100	HOSPITAL
16/12/202	51	05/12/202	49	SP	GVE I CAPI	1331	SAO PAUL	355030	HC DA FM
03/05/202	19	23/04/202	17	SP	GVE XVII C	1342	JUNDIAI	352590	HU HOSPIT
30/06/202	27	18/06/202	25	AL	1 MICRORI	1533	MACEIO	270430	HOSPITAL
01/09/202	36	29/08/202	35	GO	CENTRAL	1779	GOIANIA	520870	HOSPITAL
11/11/202	46	06/11/202	45	RJ			RIO DE JAN	330455	SMS HOSP
10/09/202	37	06/09/202	37	RJ			NILOPOLIS	330320	DOMINGO
23/07/202	30	19/07/202	30	MG	GOVERNAI	1471	GOVERNAI	312770	HOSPITAL
23/05/202	21	08/05/202	19	CE	1 CRES FOI	1519	FORTALEZ	230440	HGCC HOS
28/12/202	53	19/12/202	51	SP	GVE I CAPI	1331	SAO PAUL	355030	HC DA FM
23/06/202	26	20/05/202	21	RJ			CAMPOS D	330100	HOSPITAL
06/04/202	15	21/03/202	12	AM	ENTORNO	5584	MANAUS	130260	HOSPITAL
25/11/202	48	17/11/202	47	AM	ENTORNO	5584	MANAUS	130260	MATERNID
29/05/202	22	24/05/202	22	MG	BELO HORI	1449	BELO HORI	310620	HOSPITAL
27/04/202	18	21/04/202	17	PE	001	1497	RECIFE	261160	IMIP
28/08/202	35	21/08/202	34	SP	GVE I CAPI	1331	SAO PAUL	355030	HC DA FM
09/12/202	50	20/11/202	47	PR	02RS METF	1356	CAMPINA	410400	HOSPITAL
30/12/202	53	26/12/202	52	GO	ENTORNO	1784	FORMOSA	520800	HOSPITAL
13/07/202	29	02/07/202	27	PB	I NRS JOAC	1377	JOAO PESS	250750	MATERNID
23/06/202	26	19/06/202	25	PI	4 GRS TERE	1883	TERESINA	221100	MATERNID
02/05/202	18	25/04/202	17	RJ			RIO DE JAN	330455	HOSPITAL

08/10/202	41	05/10/202	41	MS	NRS DE DO	1966	DOURADO	500370	EBSERH HC
21/11/202	47	21/11/202	47	RJ			RIO DE JAN	330455	SMS HOSP
27/05/202	22	24/05/202	22	PI	4 GRS TERE	1883	TERESINA	221100	MATERNID
28/12/202	53	18/12/202	51	MG	POUSO ALI	1459	POCOS DE	315180	SANTA CAS
02/05/202	18	25/04/202	17	RJ			RIO DE JAN	330455	PERINATAL
06/11/202	45	05/11/202	45	ES	METROPOI	1510	SERRA	320500	HOSPITAL
28/09/202	39	10/08/202	33	PA	12 REGION	1495	REDENCAC	150613	HOSPITAL
16/07/202	29	15/07/202	29	RS	002 CRS	1607	PORTO ALE	431490	IRMANDAD
11/11/202	46	03/11/202	45	SP	GVE I CAPI	1331	SAO PAUL	355030	HOSPITAL
08/12/202	50	06/12/202	50	ES	METROPOI	1510	SERRA	320500	HOSPITAL
29/06/202	27	18/06/202	25	RO	VI GRS POF	1483	PORTO VEL	110020	HOSPITAL
27/11/202	48	23/11/202	48	RS	001 CRS	1592	CANOAS	430460	HOSPITAL
13/06/202	24	17/05/202	21	MA	REGI0NAL	1430	SAO LUIS	211130	MATERNID
13/05/202	20	10/05/202	20	PI	4 GRS TERE	1883	TERESINA	221100	HOSPITAL
21/08/202	34	11/08/202	33	RJ			ITAPERUN	330220	HOSPITAL
17/11/202	47	08/11/202	46	CE	1 CRES FOF	1519	FORTALEZ	230440	HOSPITAL
13/07/202	29	12/05/202	20	AL	1 MICRORI	1533	MACEIO	270430	HOSPITAL
24/04/202	17	22/04/202	17	SP	GVE IX FRA	1334	CAIEIRAS	350900	HOSPITAL
02/05/202	18	22/04/202	17	SP	GVE I CAPI	1331	SAO PAUL	355030	SAO LUIZ U
06/12/202	50	29/11/202	49	PR	02RS METF	1356	CURITIBA	410690	HOSPITAL
06/05/202	19	28/04/202	18	RJ			NILOPOLIS	330320	DOMINGO
23/06/202	26	16/06/202	25	RS	002 CRS	1607	PORTO ALE	431490	HOSPITAL
02/05/202	18	27/04/202	18	RJ			NOVA FRIB	330340	HOSPITAL
16/07/202	29	12/07/202	29	MG	BELO HORI	1449	BELO HORI	310620	MATERNID
11/01/202	2	02/01/202	53	SP	GVE I CAPI	1331	SAO PAUL	355030	HOSPITAL
17/09/202	38	08/09/202	37	MG	BELO HORI	1449	BETIM	310670	HOSPITAL
05/10/202	41	29/09/202	40	MG	BELO HORI	1449	CONTAGEN	311860	HOSPITAL
04/12/202	49	01/12/202	49	BA	NUCLEO RI	1381	FEIRA DE S	291080	HOSPITAL
03/06/202	23	27/05/202	22	GO	CENTRAL	1779	GOIANIA	520870	HOSPITAL
13/08/202	33	08/08/202	32	SP	GVE VIII M	1333	GUARULH	351880	HOSPITAL
09/10/202	41	30/09/202	40	SP	GVE XXIX S	1354	SAO JOSE D	354980	SANTA CAS
16/11/202	47	14/11/202	46	RN	II URSAP	1412	MOSSORO	240800	HOSPITAL
09/07/202	28	05/07/202	28	SP	GVE XXVII	1351	SAO JOSE D	354990	HOSPITAL
28/04/202	18	24/04/202	17	PE	001	1497	CABO DE S	260290	HOSPITAL
14/06/202	25	14/06/202	25	DF			BRASILIA	530010	HOSPITAL
20/08/202	34	10/08/202	33	SP	GVE XIX M	1344	MARILIA	352900	HOSPITAL
05/01/202	1	28/12/202	53	CE	1 CRES FOF	1519	FORTALEZ	230440	HOSPITAL
16/09/202	38	05/09/202	36	PR	02RS METF	1356	CURITIBA	410690	COMPLEXO
11/11/202	46	31/10/202	44	SP	GVE I CAPI	1331	SAO PAUL	355030	HC DA FMU
04/01/202	1	30/12/202	53	RS	007 CRS	1596	BAGE	430160	SANTA CAS
14/06/202	25	04/06/202	23	MA	REGIONAL	1447	CAXIAS	210300	UNIDADE D
07/05/202	19	27/04/202	18	RJ			PETROPOL	330390	HOSPITAL
07/06/202	24	27/05/202	22	BA	NUCLEO RI	1380	SALVADOR	292740	MATERNID
21/11/202	47	13/11/202	46	SC	CONCORDI	1560	CONCORDI	420430	HOSPITAL
22/01/202	3	10/12/202	50	RJ			RIO DE JAN	330455	PERINATAL
03/08/202	32	03/08/202	32	PA	12 REGION	1495	REDENCAC	150613	HOSPITAL
11/05/202	20	01/05/202	18	BA	NUCLEO RI	1380	SALVADOR	292740	MATERNID

04/07/202	27	02/07/202	27	SP	GVE I CAPI	1331	SAO PAUL	355030	HOSPITAL
29/06/202	27	18/06/202	25	PR	02RS METF	1356	CURITIBA	410690	COMPLEXC
25/05/202	22	19/05/202	21	CE	1 CRES FOF	1519	FORTALEZ	230440	HGCC HOS
18/05/202	21	16/05/202	20	RJ			RIO DE JAN	330455	HOSPITAL
15/07/202	29	15/07/202	29	PA	01 REGION	1484	BELEM	150140	SANTA CAS
27/08/202	35	21/08/202	34	GO	CENTRAL	1779	GOIANIA	520870	HOSPITAL
04/12/202	49	23/11/202	48	BA	NUCLEO RI	1380	SALVADOR	292740	MATERNID
10/09/202	37	01/09/202	36	AM	ENTORNO	5584	MANAUS	130260	MATERNID
25/11/202	48	11/11/202	46	PR	15RS MARI	1369	MARINGA	411520	SANTA CAS
04/01/202	1	29/12/202	53	PA	01 REGION	1484	BELEM	150140	SANTA CAS
13/01/202	2	01/01/202	53	SP	GVE I CAPI	1331	SAO PAUL	355030	MATERNID
08/05/202	19	08/05/202	19	PE	001	1497	RECIFE	261160	IMIP
17/06/202	25	06/05/202	19	RJ			RIO DE JAN	330455	UERJ HOSP
24/07/202	30	19/07/202	30	PB	I NRS JOAC	1377	JOAO PESS	250750	MATERNID
03/06/202	23	02/06/202	23	SP	GVE XX PIR	1345	RIO CLARO	354390	UPA 24 HC
03/08/202	32	01/08/202	31	PR	02RS METF	1356	CURITIBA	410690	COMPLEXC
21/08/202	34	18/08/202	34	SP	GVE XVII C	1342	CAMPINAS	350950	HOSPITAL
14/09/202	38	11/09/202	37	GO	SUDOESTE	1799	SANTA HEL	521930	HOSPITAL
24/12/202	52	19/12/202	51	TO			ARAGUAIN	170210	HOSPITAL
20/08/202	34	19/08/202	34	SP	GVE I CAPI	1331	SAO PAUL	355030	HOSPITAL
22/04/202	17	15/04/202	16	AM	ENTORNO	5584	MANAUS	130260	SAMEL
22/12/202	52	15/12/202	51	RJ			SAO JOAO	330510	SES RJ HOS
06/07/202	28	01/07/202	27	SP	GVE I CAPI	1331	SAO PAUL	355030	BP HOSPIT
26/11/202	48	21/11/202	47	RJ			DUQUE DE	330170	HOSPITAL
19/07/202	30	13/07/202	29	SP	GVE I CAPI	1331	SAO PAUL	355030	HOSPITAL
29/07/202	31	18/07/202	29	RS	002 CRS	1607	PORTO ALE	431490	HOSPITAL
10/08/202	33	01/08/202	31	MT	CUIABA	1578	CUIABA	510340	HOSPITAL
23/05/202	21	14/05/202	20	SP	GVE XXV S	1349	CUBATAO	351350	HOSPITAL
01/07/202	27	01/07/202	27	MG	BELO HORI	1449	BELO HORI	310620	HOSPITAL
17/09/202	38	09/09/202	37	MG	POUSO ALI	1459	ITAJUBA	313240	AISI HOSPI
02/12/202	49	25/11/202	48	PR	02RS METF	1356	CURITIBA	410690	HOSPITAL
17/04/202	16	11/04/202	15	SP	GVE I CAPI	1331	SAO PAUL	355030	HOSPITAL
02/04/202	14	27/03/202	13	PR	02RS METF	1356	CURITIBA	410690	HOSPITAL
12/06/202	24	02/06/202	23	SP	GVE I CAPI	1331	SAO PAUL	355030	MATERNID
01/04/202	14	31/03/202	14	RJ			RIO DE JAN	330455	UERJ HOSP
16/11/202	47	10/11/202	46	SP	GVE XXIX S	1354	SAO JOSE I	354980	HOSPITAL
20/08/202	34	01/08/202	31	DF			BRASILIA	530010	HOSPITAL
15/12/202	51	10/12/202	50	RJ			RIO DE JAN	330455	SMS HOSPI
30/04/202	18	19/04/202	17	SP	GVE I CAPI	1331	SAO PAUL	355030	HC DA FMU
11/07/202	28	02/07/202	27	PE	001	1497	RECIFE	261160	IMIP
23/07/202	30	15/07/202	29	CE	11 CRES SC	1521	SOBRAL	231290	HOSPITAL
20/08/202	34	13/08/202	33	AP			MACAPA	160030	HOSPITAL
24/06/202	26	20/06/202	25	CE	21 CRES JU	1531	JUAZEIRO I	230730	HOSPITAL
06/05/202	19	01/05/202	18	RJ			DUQUE DE	330170	HOSPITAL
02/04/202	14	29/03/202	14	MA	REGI0NAL	1430	SAO LUIS	211130	EBSERH HC
12/12/202	50	05/12/202	49	MS	EX NRS DE	6256	CORUMBA	500320	SANTA CAS
05/01/202	1	24/12/202	52	GO	CENTRAL	1779	GOIANIA	520870	HOSPITAL

18/06/202	25	07/06/202	24	PB	I NRS JOAC	1377	JOAO PESS	250750	MATERNID
23/05/202	21	13/05/202	20	PI	4 GRS TERE	1883	TERESINA	221100	MATERNID
21/12/202	52	20/12/202	52	SP	GVE I CAPI	1331	SAO PAULO	355030	HOSPITAL I
30/12/202	53	24/12/202	52	SP	GVE XXI PR	1346	PRESIDENT	354140	HOSPITAL (
06/07/202	28	24/06/202	26	MT	CUIABA	1578	CUIABA	510340	HOSPITAL I
20/07/202	30	08/07/202	28	SP	GVE I CAPI	1331	SAO PAULO	355030	HOSPITAL S
24/08/202	35	10/08/202	33	MG	TEOFILO OT	1465	TEOFILO OT	316860	HOSPITAL S
07/04/202	15	04/04/202	14	PE	001	1497	RECIFE	261160	IMIP
24/07/202	30	18/07/202	29	PE	008	1503	PETROLINA	261110	HOSPITAL I
03/12/202	49	24/11/202	48	SP	GVE I CAPI	1331	SAO PAULO	355030	SAO LUIZ U
07/12/202	50	02/12/202	49	RJ			BOM JESUS	330060	HOSPITAL S
16/08/202	34	06/08/202	32	PB	III NRS CAM	1421	CAMPINA (	250400	INSTITUTO
11/04/202	15	06/04/202	15	SP	GVE I CAPI	1331	SAO PAULO	355030	HOSP DO S
05/05/202	19	25/04/202	17	SP	GVE I CAPI	1331	SAO PAULO	355030	HOSPITAL I
13/07/202	29	15/05/202	20	AL	1 MICRORI	1533	MACEIO	270430	HOSPITAL U
24/06/202	26	17/06/202	25	RS	002 CRS	1607	PORTO ALE	431490	HOSPITAL I
30/12/202	53	22/11/202	48	RJ			CAMPOS D	330100	HOSPITAL I
30/06/202	27	14/06/202	25	BA	NUCLEO RI	1380	SALVADOR	292740	MATERNID
10/08/202	33	27/07/202	31	SP	GVE XXIV R	1348	RIBEIRAO P	354340	HOSPITAL I
22/10/202	43	14/10/202	42	SP	GVE XXIV R	1348	RIBEIRAO P	354340	HOSPITAL I
04/01/202	1	25/12/202	52	SP	GVE I CAPI	1331	SAO PAULO	355030	PRO MATR
06/08/202	32	25/07/202	30	SP	GVE I CAPI	1331	SAO PAULO	355030	HOSPITAL I
03/05/202	19	25/04/202	17	RJ			ITAGUAI	330200	GRUPO CEI
19/05/202	21	25/04/202	17	PB	III NRS CAM	1421	CAMPINA (	250400	INSTITUTO
28/11/202	48	26/11/202	48	SP	GVE XVII CA	1342	CAMPINAS	350950	HOSPITAL I
23/07/202	30	08/07/202	28	DF			BRASILIA	530010	HRSM
14/05/202	20	02/05/202	18	SP	GVE XXV SA	1349	SANTOS	354850	CASA DE SA
14/05/202	20	10/04/202	15	SP	GVE I CAPI	1331	SAO PAULO	355030	HOSP MUN
28/12/202	53	18/12/202	51	SP	GVE XVII CA	1342	AMERICAN	350160	HOSPITAL S
08/12/202	50	26/11/202	48	RJ			RIO DE JAN	330455	SMS HOSPI
08/05/202	19	27/04/202	18	SP	GVE I CAPI	1331	SAO PAULO	355030	HOSP MUN
15/01/202	2	02/01/202	53	SP	GVE I CAPI	1331	SAO PAULO	355030	HC DA FMU
22/05/202	21	11/05/202	20	SP	GVE I CAPI	1331	SAO PAULO	355030	HOSPITAL S
04/08/202	32	01/08/202	31	SP	GVE XVII CA	1342	JUNDIAI	352590	HU HOSPIT
19/06/202	25	16/06/202	25	SP	GVE I CAPI	1331	SAO PAULO	355030	SAO LUIZ U
10/12/202	50	29/11/202	49	TO			PALMAS	172100	HOSPITAL (
12/05/202	20	11/05/202	20	BA	NUCLEO RI	1380	SALVADOR	292740	MATERNID
13/07/202	29	06/07/202	28	SP	GVE XI ARA	1336	PENAPOLIS	353730	SANTA CAS
03/08/202	32	26/07/202	31	PE	001	1497	RECIFE	261160	IMIP
07/12/202	50	28/11/202	48	MG	UBA	1464	UBA	316990	HOSPITAL S
18/06/202	25	12/06/202	24	GO	CENTRAL	1779	GOIANIA	520870	HOSPITAL I
16/12/202	51	02/12/202	49	RJ			RIO DE JAN	330455	HOSPITAL I
18/08/202	34	05/08/202	32	PB	I NRS JOAC	1377	JOAO PESS	250750	MATERNID
01/10/202	40	16/09/202	38	BA	NUCLEO RI	1385	ILHEUS	291360	HOSPITAL S
13/05/202	20	09/05/202	19	BA	NUCLEO RI	1380	SALVADOR	292740	MATERNID
22/06/202	26	14/06/202	25	SP	GVE I CAPI	1331	SAO PAULO	355030	HC DA FMU
07/01/202	1	27/11/202	48	SP	GVE I CAPI	1331	SAO PAULO	355030	PRO MATR

21/06/202	26	21/06/202	26	CE	1 CRES FOF	1519	FORTALEZ/	230440	HGCC HOS
11/05/202	20	11/05/202	20	MG	PATOS DE I	1456	PATOS DE I	314800	HOSPITAL I
29/05/202	22	22/05/202	21	CE	1 CRES FOF	1519	FORTALEZ/	230440	HOSPITAL (
12/05/202	20	24/04/202	17	SP	GVE I CAPI	1331	SAO PAULC	355030	HC DA FMU
29/09/202	40	22/09/202	39	PR	03RS PONT	1357	PONTA GR	411990	HOSPITAL (
08/07/202	28	01/06/202	23	GO	CENTRAL	1779	GOIANIA	520870	HOSPITAL I
04/08/202	32	01/08/202	31	GO	CENTRAL	1779	GOIANIA	520870	HOSPITAL I
28/05/202	22	21/05/202	21	RO	VI GRS POF	1483	PORTO VEL	110020	HOSPITAL I
09/07/202	28	03/07/202	27	SP	GVE XX PIR	1345	PIRACICAB	353870	HOSPITAL I
14/10/202	42	09/10/202	41	RJ			NOVA IGU/	330350	HGNI
04/02/202	5	26/01/202	4	MG	UBERLAND	1462	ROMARIA	315640	CENTRO DI
16/03/202	11	06/03/202	9	SP	GVE I CAPI	1331	SAO PAULC	355030	HOSPITAL S
21/03/202	12	15/03/202	11	RJ			RIO DE JAN	330455	MS HSE HC
15/03/202	11	08/03/202	10	SP	GVE I CAPI	1331	SAO PAULC	355030	HOSPITAL I
16/04/202	15	12/04/202	15	GO	CENTRAL	1779	GOIANIA	520870	HOSPITAL I
20/04/202	16	10/04/202	14	SP	GVE I CAPI	1331	SAO PAULC	355030	PRO MATR
29/05/202	21	26/05/202	21	RS	005 CRS	1608	VACARIA	432250	HOSPITAL I
24/05/202	21	12/05/202	19	RR			BOA VISTA	140010	HOSPITAL (
22/06/202	25	07/06/202	23	DF			BRASILIA	530010	HRSAM HC
23/06/202	25	17/06/202	24	BA	NUCLEO RI	1381	FEIRA DE S.	291080	HOSPITAL I
29/08/202	35	23/08/202	34	SP	GVE VIII M	1333	GUARULHC	351880	HOSPITAL I
02/04/202	13	26/03/202	12	RN	VII URSAP	1416	NATAL	240810	HOSPITAL /
06/02/202	5	29/01/202	4	PA	01 REGION	1484	BELEM	150140	SANTA CAS
19/02/202	7	09/02/202	6	MG	BELO HORI	1449	CONTAGEN	311860	CMI JUVEN
24/02/202	8	14/02/202	7	SP	GVE XVII C.	1342	CAMPINAS	350950	MATERNID
08/03/202	10	28/02/202	9	MG	BELO HORI	1449	CONTAGEN	311860	CMI JUVEN
16/03/202	11	10/03/202	10	SP	GVE I CAPI	1331	SAO PAULC	355030	HOSPITAL I
17/04/202	15	13/04/202	15	SP	GVE IX FRA	1334	FRANCISCC	351630	HOSPITAL I
25/05/202	21	15/03/202	11	ES	METROPOI	1510	SERRA	320500	HOSPITAL I
21/06/202	25	08/06/202	23	SP	GVE XXVII S	1351	SAO JOSE D	354990	HOSPITAL S
11/06/202	23	07/06/202	23	RJ			RIO DE JAN	330455	HOSPITAL (
21/06/202	25	10/06/202	23	RJ			RIO DE JAN	330455	MS HSE HC
22/09/202	38	17/09/202	37	DF			BRASILIA	530010	HRAN
01/06/202	22	17/05/202	20	RN	VII URSAP	1416	NATAL	240810	HOSPITAL /
07/01/202	1	26/12/202	52	GO	CENTRAL	1779	GOIANIA	520870	HOSPITAL I
07/03/202	10	28/02/202	9	RJ			NITEROI	330330	HOSPITAL I
25/03/202	12	10/03/202	10	GO	CENTRAL	1779	GOIANIA	520870	HOSPITAL I
20/03/202	11	14/03/202	11	RJ			RIO DE JAN	330455	HOSPITAL (
28/03/202	13	24/03/202	12	RJ			RIO DE JAN	330455	CASA DE S/
05/04/202	14	24/03/202	12	PB	I NRS JOAC	1377	JOAO PESS	250750	HOSPITAL I
18/03/202	11	18/03/202	11	CE	1 CRES FOF	1519	FORTALEZ/	230440	HGCC HOS
05/06/202	22	24/05/202	21	GO	CENTRAL	1779	GOIANIA	520870	HOSPITAL I
20/03/202	11	20/03/202	11	CE	1 CRES FOF	1519	FORTALEZ/	230440	HGCC HOS
01/07/202	26	01/06/202	22	RS	002 CRS	1607	PORTO ALE	431490	HOSPITAL I
04/07/202	27	27/06/202	26	MT	RONDONO	1579	PRIMAVER	510704	SECRETARI
16/07/202	28	10/07/202	27	ES	METROPOI	1510	SERRA	320500	HOSPITAL I
11/08/202	32	27/07/202	30	MG	BELO HORI	1449	BETIM	310670	HOSPITAL I

19/05/202	20	10/05/202	19	SP	GVE I CAPI	1331	SAO PAULO	355030	HOSPITAL S
06/09/202	36	20/08/202	33	MG	DIVINOPOL	1472	BOM DESP.	310740	HOSP SANT
01/04/202	13	26/03/202	12	RN	VII URSAP	1416	NATAL	240810	HOSPITAL (
28/04/202	17	15/03/202	11	ES	METROPOL	1510	VITORIA	320530	HOSPITAL I
24/05/202	21	15/05/202	19	SP	GVE XXIV F	1348	RIBEIRAO F	354340	HOSPITAL I
23/05/202	21	19/04/202	16	ES	METROPOL	1510	SERRA	320500	HOSPITAL I
13/06/202	24	02/06/202	22	SP	GVE XXIX S	1354	SAO JOSE D	354980	HOSPITAL I
26/02/202	8	26/02/202	8	CE	1 CRES FOF	1519	FORTALEZA	230440	HGCC HOS
03/06/202	22	31/05/202	22	PR	15RS MARI	1369	SARANDI	412625	METROPOL
24/07/202	29	11/07/202	28	RS	002 CRS	1607	PORTO ALE	431490	HOSPITAL I
16/07/202	28	13/07/202	28	MS	EX ARS DE	1975	CAMPO GF	500270	EBSERH HC
23/07/202	29	12/05/202	19	PR	16RS APUC	1370	APUCARAN	410140	HNSG HOS
25/08/202	34	14/08/202	32	MT	CUIABA	1578	CUIABA	510340	HOSPITAL U
03/09/202	35	25/08/202	34	SC	JOINVILLE	1565	JOINVILLE	420910	HOSPITAL I
21/10/202	42	10/09/202	36	GO	CENTRAL	1779	GOIANIA	520870	HOSPITAL I
04/02/202	5	26/01/202	4	RS	006 CRS	1611	PASSO FUN	431410	HOSPITAL S
01/03/202	9	24/02/202	8	SP	GVE I CAPI	1331	SAO PAULO	355030	HOSPITAL I
24/03/202	12	13/03/202	10	SP	GVE XII ARA	1337	ARARAQUA	350320	HOSPITAL S
30/03/202	13	18/03/202	11	RJ			RIO DE JAN	330455	CASA DE SA
08/04/202	14	27/03/202	12	MA	REGI0NAL	1430	SAO LUIS	211130	MATERNID
19/04/202	16	10/04/202	14	SP	GVE XVII CA	1342	VALINHOS	355620	HOSPITAL I
09/04/202	14	30/03/202	13	SP	GVE X OSA	1335	OSASCO	353440	HOSPITAL S
18/05/202	20	15/05/202	19	PB	III NRS CAN	1421	CAMPINA (	250400	INSTITUTO
03/05/202	18	19/04/202	16	RJ			RIO DE JAN	330455	HOSPITAL I
14/05/202	19	03/05/202	18	SP	GVE I CAPI	1331	SAO PAULO	355030	HOSPITAL I
10/02/202	6	10/02/202	6	CE	1 CRES FOF	1519	FORTALEZA	230440	HGCC HOS
22/02/202	8	22/02/202	8	CE	1 CRES FOF	1519	FORTALEZA	230440	HGCC HOS
03/06/202	22	25/05/202	21	PA	10 REGION	1493	ALTAMIRA	150060	HOSPITAL I
06/02/202	5	04/02/202	5	SP	GVE I CAPI	1331	SAO PAULO	355030	MATERNID
29/09/202	39	21/09/202	38	MT	CUIABA	1578	CUIABA	510340	HOSPITAL U
11/06/202	23	17/05/202	20	MT	CUIABA	1578	CUIABA	510340	CENTRO DI
24/03/202	12	13/03/202	10	GO	CENTRO SU	1781	APARECIDA	520140	HOSPITAL (
06/09/202	36	09/08/202	32	RJ			RIO DE JAN	330455	MS HSE HC
24/08/202	34	10/03/202	10	RJ			RIO DE JAN	330455	HOSPITAL I
14/09/202	37	05/09/202	36	RJ			RIO DE JAN	330455	MS HSE HC
22/09/202	38	15/09/202	37	RJ			RIO DE JAN	330455	MS HSE HC
02/01/202	1	30/12/202	52	GO	ESTRADA D	1802	CATALAO	520510	SANTA CAS
03/02/202	5	21/01/202	3	AM	ENTORNO	5584	MANAUS	130260	MATERNID
25/02/202	8	12/02/202	6	PA	09 REGION	1492	MONTE AL	150480	HOSPITAL I
17/03/202	11	08/03/202	10	GO	CENTRAL	1779	GOIANIA	520870	HOSPITAL I
18/03/202	11	09/03/202	10	SP	GVE I CAPI	1331	SAO PAULO	355030	PRO MATR
26/04/202	17	21/04/202	16	SP	GVE VIII M	1333	ITAQUAQU	352310	HOSPITAL (
30/04/202	17	26/04/202	17	RS	018 CRS	1605	TRAMAND.	432160	HOSPITAL T
31/05/202	22	21/05/202	20	SC	JARAGUA D	1567	JARAGUA D	420890	HOSPITAL J
24/04/202	16	24/04/202	16	CE	1 CRES FOF	1519	FORTALEZA	230440	HGCC HOS
24/06/202	25	22/06/202	25	CE	11 CRES SC	1521	SOBRAL	231290	HOSPITAL I
14/06/202	24	01/06/202	22	MG	BELO HORI	1449	BELO HORI	310620	HOSPITAL J

03/09/202	35	21/08/202	33	RS	006 CRS	1611	CARAZINH	430470	HOSPITAL I
01/11/202	44	26/10/202	43	DF			BRASILIA	530010	HOSPITAL I
15/02/202	7	03/02/202	5	SP	GVE XVII C	1342	CAMPINAS	350950	MATERNID
25/03/202	12	23/03/202	12	RS	002 CRS	1607	PORTO ALE	431490	HOSPITAL I
29/03/202	13	23/03/202	12	SP	GVE XVII C	1342	CAMPINAS	350950	HOSPITAL
26/03/202	12	03/03/202	9	BA	NUCLEO RI	1380	SALVADOR	292740	HOSPITAL
30/03/202	13	16/03/202	11	SP	GVE XVII C	1342	CAMPINAS	350950	MATERNID
25/03/202	12	16/03/202	11	CE	1 CRES FOI	1519	FORTALEZ	230440	HGCC HOS
06/04/202	14	27/03/202	12	SP	GVE XXXI S	1353	ITAPETININ	352230	HOSPITAL I
06/04/202	14	24/03/202	12	SP	GVE XVII C	1342	JUNDIAI	352590	HU HOSPIT
09/04/202	14	03/04/202	13	MA	REGI0NAL	1430	SAO LUIS	211130	MATERNID
01/06/202	22	18/05/202	20	SP	GVE I CAPI	1331	SAO PAULC	355030	HC DA FMU
30/05/202	22	19/05/202	20	RJ			DUQUE DE	330170	HOSPITAL
15/06/202	24	11/06/202	23	SP	GVE XVII C	1342	CAMPINAS	350950	HOSPITAL I
23/06/202	25	16/06/202	24	SP	GVE I CAPI	1331	SAO PAULC	355030	HOSPITAL
10/08/202	32	10/08/202	32	PR	10RS CASC	1364	CASCAVEL	410480	HOSPITAL
29/11/202	48	22/11/202	47	RS	001 CRS	1592	NOVO HAN	431340	HOSPITAL I
20/02/202	7	14/02/202	7	MG	BELO HORI	1449	MATEUS LE	314070	MATEUS LE
01/03/202	9	11/02/202	6	SP	GVE I CAPI	1331	SAO PAULC	355030	HOSP MUN
14/04/202	15	24/03/202	12	SP	GVE I CAPI	1331	SAO PAULC	355030	HC DA FMU
17/05/202	20	06/05/202	18	PR	11RS CAMI	1365	CAMPO M	410430	HOSPITAL
20/05/202	20	11/05/202	19	PR	04RS IRATI	1358	IRATI	411070	SANTA CAS
04/06/202	22	19/05/202	20	SP	GVE I CAPI	1331	SAO PAULC	355030	HC DA FMU
24/05/202	21	10/05/202	19	MG	MONTES C	1473	TAIOBEIRA	316800	HOSPITAL
05/07/202	27	20/06/202	25	MA	REGI0NAL	1430	SAO LUIS	211130	MATERNID
19/06/202	24	01/06/202	22	PA	01 REGION	1484	BELEM	150140	SANTA CAS
30/06/202	26	22/06/202	25	SP	GVE I CAPI	1331	SAO PAULC	355030	MATERNID
20/07/202	29	10/07/202	27	PI	4 GRS TERE	1883	TERESINA	221100	HOSPITAL
21/07/202	29	10/07/202	27	RS	005 CRS	1608	CAXIAS DO	430510	HOSPITAL
23/07/202	29	01/07/202	26	SP	GVE I CAPI	1331	SAO PAULC	355030	MATERNID
10/04/202	14	28/03/202	13	CE	1 CRES FOI	1519	FORTALEZ	230440	HGCC HOS
30/09/202	39	25/09/202	38	RJ			NILOPOLIS	330320	DOMINGO
07/01/202	1	31/12/202	52	CE	1 CRES FOI	1519	FORTALEZ	230440	MATERNID
25/03/202	12	17/03/202	11	SP	GVE I CAPI	1331	SAO PAULC	355030	HC DA FMU
12/04/202	15	05/04/202	14	MT	CUIABA	1578	CUIABA	510340	HOSPITAL
09/04/202	14	26/03/202	12	GO	CENTRAL	1779	GOIANIA	520870	HOSPITAL I
07/05/202	18	27/04/202	17	PR	08RS FRAN	1362	AMPERE	410100	INSTITUTO
13/04/202	15	06/04/202	14	SP	GVE XI ARA	1336	ARACATUE	350280	SANTA CAS
10/05/202	19	30/04/202	17	MG	BELO HORI	1449	NOVA LIM	314480	NOVA LIM
10/05/202	19	05/05/202	18	PR	10RS CASC	1364	CASCAVEL	410480	HOSPITAL
19/05/202	20	11/05/202	19	RJ			RIO DE JAN	330455	MS HSE HC
18/05/202	20	10/05/202	19	MA	REGI0NAL	1430	SAO LUIS	211130	MATERNID
14/06/202	24	04/06/202	22	SP	GVE XXIX S	1354	SAO JOSE D	354980	HOSPITAL I
03/06/202	22	03/06/202	22	CE	1 CRES FOI	1519	FORTALEZ	230440	HGCC HOS
15/07/202	28	02/07/202	26	SP	GVE I CAPI	1331	SAO PAULC	355030	PRO MATR
15/10/202	41	27/09/202	39	DF			BRASILIA	530010	HRSM
16/08/202	33	09/08/202	32	SP	GVE XXXI S	1353	SAO ROQU	355060	HOSPITAL

16/08/202	33	15/07/202	28	RJ			RIO DE JAN	330455	MS HSE HC
29/07/202	30	26/07/202	30	RS	018 CRS	1605	TRAMAND	432160	HOSPITAL T
12/01/202	2	05/01/202	1	RJ			NILOPOLIS	330320	DOMINGO
08/09/202	36	30/08/202	35	RJ			RIO DE JAN	330455	MS HSE HC
30/11/202	48	28/11/202	48	PE	008	1503	PETROLINA	261110	HOSPITAL I
02/03/202	9	25/02/202	8	DF			BRASILIA	530010	HOSPITAL S
28/02/202	9	22/02/202	8	RS	002 CRS	1607	PORTO ALE	431490	HOSPITAL I
11/04/202	15	05/04/202	14	PB	I NRS JOAC	1377	JOAO PESS	250750	MATERNID
23/05/202	21	08/05/202	18	PB	III NRS CAN	1421	CAMPINA (	250400	HOSPITAL I
26/04/202	17	21/04/202	16	SP	GVE I CAPI	1331	SAO PAULC	355030	SAO LUIZ U
21/05/202	20	15/04/202	15	DF			BRASILIA	530010	HRAN
16/05/202	20	10/05/202	19	PA	11 REGION	1494	MARABA	150420	HOSPITAL I
04/06/202	22	19/05/202	20	MT	CUIABA	1578	CUIABA	510340	HOSPITAL U
16/05/202	20	16/05/202	20	CE	1 CRES FOF	1519	FORTALEZA	230440	HGCC HOS
23/06/202	25	15/06/202	24	SP	GVE XVI BC	1341	BOTUCATU	350750	HOSPITAL I
31/05/202	22	19/05/202	20	GO	CENTRAL	1779	GOIANIA	520870	HOSPITAL I
19/06/202	24	04/06/202	22	BA	NUCLEO RE	1380	SALVADOR	292740	MATERNID
30/06/202	26	20/06/202	25	AM	ENTORNO	5584	MANAUS	130260	MATERNID
21/10/202	42	19/10/202	42	SP	GVE I CAPI	1331	SAO PAULC	355030	SANTA CAS
02/08/202	31	29/07/202	30	BA	NUCLEO RE	1380	SALVADOR	292740	MATERNID
28/12/202	52	24/12/202	51	SP	GVE I CAPI	1331	SAO PAULC	355030	HOSPITAL I
21/09/202	38	14/09/202	37	MT	CUIABA	1578	CUIABA	510340	HOSPITAL U
16/01/202	2	10/01/202	2	SP	GVE XXXI S	1353	SOROCABA	355220	HOSPITAL S
13/01/202	2	09/01/202	1	AM	ENTORNO	5584	MANAUS	130260	MATERNID
04/03/202	9	27/02/202	8	BA	NUCLEO RE	1380	SALVADOR	292740	MATERNID
14/03/202	11	05/03/202	9	SP	GVE XI ARA	1336	ARACATUE	350280	HOSPITAL U
22/03/202	12	06/03/202	9	SP	GVE I CAPI	1331	SAO PAULC	355030	HOSP MUN
08/04/202	14	04/04/202	14	RJ			RIO DE JAN	330455	SMS HOSPI
07/04/202	14	23/03/202	12	MG	BELO HORI	1449	CONTAGEN	311860	CMI JUVEN
14/04/202	15	30/03/202	13	SP	GVE I CAPI	1331	SAO PAULC	355030	HC DA FMU
18/06/202	24	04/06/202	22	PR	10RS CASC	1364	CASCAVEL	410480	HOSPITAL U
05/05/202	18	28/04/202	17	RJ			SAO GONC	330490	HOSPITAL S
28/10/202	43	26/10/202	43	BA	NUCLEO RE	1380	SALVADOR	292740	MATERNID
08/04/202	14	21/03/202	12	SP	GVE I CAPI	1331	SAO PAULC	355030	SAO LUIZ U
14/05/202	19	07/05/202	18	SP	GVE XXIX S	1354	VOTUPORA	355710	SANTA CAS
29/04/202	17	26/04/202	17	SP	GVE I CAPI	1331	SAO PAULC	355030	HOSPITAL (
03/04/202	13	25/03/202	12	MT	CUIABA	1578	CUIABA	510340	FEMINA H(
18/05/202	20	04/05/202	18	DF			BRASILIA	530010	HRAN
23/05/202	21	19/05/202	20	MA	REGI0NAL	1430	SAO LUIS	211130	MATERNID
28/01/202	4	17/01/202	3	SP	GVE I CAPI	1331	SAO PAULC	355030	MATERNID
14/05/202	19	07/05/202	18	MG	ITABIRA	1451	ITABIRA	313170	FSFX O S S
11/07/202	28	06/07/202	27	SP	GVE VII SAI	1332	MAUA	352940	HOSPITAL A
21/06/202	25	07/06/202	23	SP	GVE I CAPI	1331	SAO PAULC	355030	SAO LUIZ U
10/06/202	23	01/06/202	22	SP	GVE I CAPI	1331	SAO PAULC	355030	MATERNID
26/05/202	21	25/05/202	21	MT	CUIABA	1578	CUIABA	510340	HOSPITAL S
10/06/202	23	10/06/202	23	CE	1 CRES FOF	1519	FORTALEZA	230440	HGCC HOS
12/01/202	2	09/01/202	1	SP	GVE I CAPI	1331	SAO PAULC	355030	UNIDADE D

25/02/202	8	13/02/202	6	MG	UBERLAND	1462	UBERLAND	317020	HOSPITAL I
25/03/202	12	03/03/202	9	DF			BRASILIA	530010	HOSPITAL /
08/03/202	10	01/03/202	9	RO	VI GRS POF	1483	PORTO VEL	110020	HOSPITAL S
18/03/202	11	05/03/202	9	PR	02RS METF	1356	CURITIBA	410690	COMPLEXC
26/03/202	12	14/03/202	11	PB	I NRS JOAC	1377	JOAO PESS	250750	MATERNID
14/04/202	15	07/04/202	14	SP	GVE I CAPI	1331	SAO PAULC	355030	HOSP MUN
07/04/202	14	28/03/202	13	MT	CUIABA	1578	CUIABA	510340	FEMINA HC
17/06/202	24	04/06/202	22	RS	011 CRS	1600	ERECHIM	430700	HOSPITAL I
23/04/202	16	12/04/202	15	PA	01 REGION	1484	BELEM	150140	UPA DAICC
27/07/202	30	18/07/202	29	SC	ITAJAI	1550	BALNEARIC	420200	HOSPITAL I
06/08/202	31	30/07/202	30	PA	12 REGION	1495	REDENCAC	150613	HOSPITAL I
14/04/202	15	04/04/202	14	CE	1 CRES FOF	1519	FORTALEZ/	230440	HGCC HOS
28/11/202	48	22/11/202	47	PA	01 REGION	1484	BELEM	150140	SANTA CAS
05/02/202	5	29/01/202	4	SP	GVE I CAPI	1331	SAO PAULC	355030	HOSPITAL I
08/10/202	40	28/09/202	39	PR	10RS CASC	1364	CASCAVEL	410480	HOSPITAL U
08/12/202	49	30/11/202	48	SC	JOINVILLE	1565	JOINVILLE	420910	CENTRO HC
11/01/202	2	26/12/202	52	PR	10RS CASC	1364	CASCAVEL	410480	HOSPITAL I
31/03/202	13	21/03/202	12	SP	GVE VII SAI	1332	SANTO AN	354780	HOSPITAL I
06/03/202	9	20/02/202	7	CE	1 CRES FOF	1519	FORTALEZ/	230440	HOSPITAL /
03/04/202	13	25/03/202	12	GO	CENTRAL	1779	GOIANIA	520870	HOSPITAL I
05/04/202	14	23/03/202	12	SP	GVE I CAPI	1331	SAO PAULC	355030	HC DA FMU
12/04/202	15	01/03/202	9	SP	GVE I CAPI	1331	SAO PAULC	355030	HOSP MUN
08/03/202	10	08/03/202	10	RR			BOA VISTA	140010	HOSPITAL (
24/05/202	21	15/05/202	19	SP	GVE XXXI S	1353	ALUMINIO	350115	SMS DEPAI
13/04/202	15	31/03/202	13	SP	GVE XVII C/	1342	CAMPINAS	350950	MATERNID
27/05/202	21	17/05/202	20	RN	II URSAP	1412	MOSSORO	240800	HOSPITAL S
31/03/202	13	21/03/202	12	RJ			RIO DAS OS	330452	CLINICA M
17/04/202	15	10/04/202	14	PB	I NRS JOAC	1377	JOAO PESS	250750	MATERNID
07/05/202	18	03/05/202	18	RJ			RIO DE JAN	330455	MS HSE HC
13/05/202	19	08/05/202	18	SP	GVE I CAPI	1331	SAO PAULC	355030	UNIDADE D
04/05/202	18	27/04/202	17	PR	02RS METF	1356	CURITIBA	410690	COMPLEXC
25/05/202	21	20/05/202	20	PR	10RS CASC	1364	CASCAVEL	410480	HOSPITAL U
07/06/202	23	27/05/202	21	SC	JOINVILLE	1565	JOINVILLE	420910	HOSPITAL I
12/06/202	23	01/06/202	22	SP	GVE I CAPI	1331	SAO PAULC	355030	INSTITUTO
30/06/202	26	15/06/202	24	RS	003 CRS	1593	RIO GRANI	431560	HOSPITAL U
27/07/202	30	17/07/202	28	DF			BRASILIA	530010	HRAN
18/05/202	20	10/05/202	19	MT	CUIABA	1578	CUIABA	510340	CENTRO DI
19/08/202	33	10/08/202	32	GO	CENTRAL	1779	GOIANIA	520870	HOSPITAL I
13/02/202	6	05/02/202	5	AM	ENTORNO	5584	MANAUS	130260	MATERNID
06/03/202	9	01/03/202	9	MG	UBERLAND	1462	UBERLAND	317020	HOSPITAL I
27/01/202	4	21/01/202	3	CE	1 CRES FOF	1519	FORTALEZ/	230440	HOSPITAL I
22/03/202	12	04/03/202	9	SP	GVE I CAPI	1331	SAO PAULC	355030	HOSP SAO
21/03/202	12	09/03/202	10	PA	01 REGION	1484	BELEM	150140	SANTA CAS
10/03/202	10	10/03/202	10	DF			BRASILIA	530010	HOSPITAL S
02/05/202	18	21/04/202	16	BA	NUCLEO RI	1380	SALVADOR	292740	MATERNID
17/05/202	20	02/05/202	18	SP	GVE VIII M	1333	GUARULHC	351880	HOSPITAL I
21/05/202	20	18/05/202	20	BA	NUCLEO RI	1380	SALVADOR	292740	MATERNID

26/05/202	21	18/05/202	20	RN	VI URSAP	1417	PAU DOS F	240940	HOSPITAL
28/05/202	21	14/05/202	19	SP	GVE I CAPI	1331	SAO PAULO	355030	MATERNID
06/06/202	23	06/06/202	23	PA	01 REGION	1484	BELEM	150140	SANTA CAS
24/01/202	4	03/01/202	1	SP	GVE I CAPI	1331	SAO PAULO	355030	MATERNID
26/05/202	21	22/05/202	20	SP	GVE XX PIR	1345	LEME	352670	HOSPITAL
03/09/202	35	23/08/202	34	PR	09RS FOZ D	1363	FOZ DO IGU	410830	HOSPITAL
18/02/202	7	08/02/202	6	SC	ITAJAI	1550	BALNEARIO	420200	HOSPITAL
05/03/202	9	23/02/202	8	MG	BELO HORI	1449	CONTAGEM	311860	HOSPITAL
12/03/202	10	05/03/202	9	CE	1 CRES FOR	1519	FORTALEZA	230440	HGCC HOS
21/05/202	20	16/05/202	20	MG	ALFENAS	1379	ALFENAS	310160	SANTA CAS
01/05/202	17	22/04/202	16	RN	VII URSAP	1416	NATAL	240810	HOSPITAL
01/05/202	17	26/04/202	17	CE	1 CRES FOR	1519	FORTALEZA	230440	HGCC HOS
13/06/202	24	05/06/202	22	SP	GVE I CAPI	1331	SAO PAULO	355030	HOSPITAL
23/03/202	12	23/03/202	12	CE	1 CRES FOR	1519	FORTALEZA	230440	HGCC HOS
06/07/202	27	24/06/202	25	GO	CENTRAL	1779	GOIANIA	520870	HOSPITAL
29/06/202	26	16/06/202	24	DF			BRASILIA	530010	HRSM
08/07/202	27	03/07/202	26	BA	NUCLEO RE	1380	SALVADOR	292740	MATERNID
20/07/202	29	10/07/202	27	AM	ENTORNO	5584	MANAUS	130260	HOSPITAL A
27/08/202	34	25/08/202	34	PR	09RS FOZ D	1363	FOZ DO IGU	410830	HOSPITAL
16/01/202	2	06/01/202	1	SP	GVE XXVII S	1351	SAO JOSE D	354990	HOSPITAL V
13/01/202	2	07/01/202	1	TO			ARAGUAIN	170210	HOSPITAL
05/03/202	9	18/02/202	7	RS	013 CRS	1609	SANTA CRU	431680	HOSPITAL S
10/03/202	10	01/03/202	9	SP	GVE I CAPI	1331	SAO PAULO	355030	HOSPITAL S
12/04/202	15	26/03/202	12	AM	ENTORNO	5584	MANAUS	130260	MATERNID
17/03/202	11	08/03/202	10	SP	GVE I CAPI	1331	SAO PAULO	355030	HC DA FMU
04/03/202	9	26/02/202	8	RS	002 CRS	1607	PORTO ALE	431490	HOSPITAL
17/05/202	20	08/04/202	14	MA	REGIONAL	1446	BACABAL	210120	HOSPITAL
08/04/202	14	31/03/202	13	RJ			RIO DE JAN	330455	MS HSE HC
06/04/202	14	30/03/202	13	RR			BOA VISTA	140010	HOSPITAL
24/05/202	21	10/05/202	19	MG	DIVINOPOL	1472	DIVINOPOL	312230	HOSPITAL S
27/05/202	21	19/05/202	20	SP	GVE XI ARA	1336	AURIFLAM	350420	SANTA CAS
19/05/202	20	10/05/202	19	BA	NUCLEO RE	1380	SALVADOR	292740	MATERNID
31/08/202	35	30/07/202	30	SP	GVE I CAPI	1331	SAO PAULO	355030	HOSPITAL
16/03/202	11	01/03/202	9	PR	01RS PARA	1355	PARANAGU	411820	HOSPITAL
24/03/202	12	15/03/202	11	MT	CUIABA	1578	CUIABA	510340	HOSPITAL U
19/03/202	11	11/03/202	10	RJ			RIO DE JAN	330455	SMS HOSPI
17/03/202	11	06/03/202	9	PR	17RS LOND	1371	LONDRINA	411370	HOSPITAL
13/02/202	6	12/02/202	6	PR	15RS MARI	1369	MARINGA	411520	SANTA CAS
14/01/202	2	10/01/202	2	AM	ENTORNO	5584	MANAUS	130260	MATERNID
20/03/202	11	15/03/202	11	MG	PONTE NO	1458	PONTE NO	315210	HOSPITAL
17/03/202	11	15/03/202	11	SP	GVE I CAPI	1331	SAO PAULO	355030	BP HOSPIT
20/03/202	11	12/03/202	10	GO	CENTRAL	1779	GOIANIA	520870	HOSPITAL
08/05/202	18	27/04/202	17	MG	PONTE NO	1458	PONTE NO	315210	HOSPITAL
07/04/202	14	30/03/202	13	PR	02RS METR	1356	CURITIBA	410690	ONIX MAT
25/05/202	21	18/04/202	16	PA	01 REGION	1484	BELEM	150140	SANTA CAS
20/04/202	16	04/04/202	14	SP	GVE I CAPI	1331	SAO PAULO	355030	HC DA FMU
02/06/202	22	24/05/202	21	SP	GVE VII SA	1332	MAUA	352940	HOSPITAL

19/05/202	20	07/05/202	18	DF			BRASILIA	530010	HRAN
27/05/202	21	16/05/202	20	PB	III NRS CAN	1421	CAMPINA (	250400	INSTITUTO
18/08/202	33	10/08/202	32	RJ			MACAE	330240	CLINICA SA
25/05/202	21	22/05/202	20	MG	JUIZ DE FO	1452	JUIZ DE FO	313670	HOSPITAL I
28/03/202	13	16/03/202	11	CE	1 CRES FOF	1519	FORTALEZA	230440	HGCC HOS
19/07/202	29	15/07/202	28	PA	01 REGION	1484	BELEM	150140	SANTA CAS
26/08/202	34	19/08/202	33	TO			PALMAS	172100	HOSPITAL (
19/10/202	42	15/10/202	41	PR	03RS PONT	1357	PONTA GR(	411990	HOSPITAL (
18/02/202	7	17/02/202	7	PA	12 REGION	1495	REDENCAC	150613	HOSPITAL I
05/04/202	14	25/03/202	12	TO			PALMAS	172100	HOSPITAL (
24/03/202	12	18/03/202	11	CE	1 CRES FOF	1519	FORTALEZA	230440	HOSPITAL I
06/03/202	9	01/03/202	9	RJ			RIO DE JAN	330455	MS HSE HC
26/04/202	17	14/04/202	15	SC	FLORIANOI	1476	FLORIANOI	420540	HOSPITAL U
27/05/202	21	26/05/202	21	PR	10RS CASC	1364	CASCAVEL	410480	HOSPITAL U
23/05/202	21	12/05/202	19	SP	GVE XXXI S	1353	SOROCABA	355220	UPA UNIDA
11/04/202	15	08/04/202	14	AP			OIAPOQUE	160050	SES AP HOS
22/04/202	16	11/04/202	15	CE	1 CRES FOF	1519	FORTALEZA	230440	HOSPITAL I
15/04/202	15	04/04/202	14	SP	GVE I CAPI	1331	SAO PAULC	355030	HOSP MUN
23/04/202	16	01/04/202	13	MG	BELO HORI	1449	BETIM	310670	HOSPITAL I
28/05/202	21	19/05/202	20	SP	GVE I CAPI	1331	SAO PAULC	355030	SAO LUIZ U
06/04/202	14	30/03/202	13	SP	GVE X OSA	1335	JANDIRA	352500	COVID 19 (
20/05/202	20	12/05/202	19	PR	02RS METF	1356	CURITIBA	410690	HNSG
25/06/202	25	12/06/202	23	MT	CUIABA	1578	CUIABA	510340	HOSPITAL U
13/08/202	32	06/08/202	31	TO			PALMAS	172100	HOSPITAL (
22/01/202	3	10/01/202	2	AM	ENTORNO	5584	MANAUS	130260	INSTITUTO
29/07/202	30	13/05/202	19	PR	09RS FOZ D	1363	FOZ DO IGU	410830	HOSPITAL I
17/03/202	11	06/03/202	9	SP	GVE I CAPI	1331	SAO PAULC	355030	HC DA FMU
12/03/202	10	07/03/202	10	SP	GVE VIII M	1333	GUARULHC	351880	HOSPITAL U
11/03/202	10	02/03/202	9	MG	DIVINOPOI	1472	DIVINOPOI	312230	HOSPITAL S
01/04/202	13	24/03/202	12	SP	GVE XXIX S	1354	SAO JOSE D	354980	HOSPITAL I
13/04/202	15	04/04/202	14	RJ			RIO DE JAN	330455	MS HSE HC
17/06/202	24	11/06/202	23	RJ			RIO DE JAN	330455	MS HSE HC
30/04/202	17	16/02/202	7	PR	09RS FOZ D	1363	FOZ DO IGU	410830	HOSPITAL I
31/03/202	13	23/03/202	12	SP	GVE VIII M	1333	GUARULHC	351880	HOSPITAL I
07/01/202	1	01/01/202	52	SP	GVE VII SAI	1332	SAO BERNA	354870	HOSPITAL A
24/11/202	47	19/11/202	46	PA	01 REGION	1484	BELEM	150140	SANTA CAS
08/01/202	1	04/01/202	1	SP	GVE XXIX S	1354	SAO JOSE D	354980	SANTA CAS
15/01/202	2	08/01/202	1	PR	02RS METF	1356	CURITIBA	410690	COMPLEXC
01/02/202	5	22/01/202	3	PA	09 REGION	1492	MONTE AL	150480	HOSPITAL I
04/04/202	14	27/03/202	12	BA	NUCLEO RI	1380	SALVADOR	292740	MATERNID
06/03/202	9	27/02/202	8	RS	004 CRS	1595	SANTA MA	431690	HOSPITAL I
14/05/202	19	06/05/202	18	SP	GVE XXIX S	1354	SAO JOSE D	354980	HOSPITAL I
12/04/202	15	11/04/202	15	PE	001	1497	RECIFE	261160	IMIP
15/05/202	19	15/05/202	19	CE	1 CRES FOF	1519	FORTALEZA	230440	HGCC HOS
30/05/202	22	12/05/202	19	MG	TEOFILO O	1465	TEOFILO O	316860	HOSPITAL S
27/05/202	21	21/03/202	12	SP	GVE I CAPI	1331	SAO PAULC	355030	MATERNID
10/06/202	23	31/05/202	22	SP	GVE XXIV F	1348	RIBEIRAO F	354340	HOSPITAL I

03/06/202	22	20/05/202	20	SP	GVE XIII AS	1338	ASSIS	350400	UPA RUY S
20/05/202	20	14/05/202	19	BA	NUCLEO RE	1380	SALVADOR	292740	MATERNID
14/06/202	24	05/06/202	22	SP	GVE XXVI S	1350	ITAPIRA	352260	HOSPITAL I
22/06/202	25	18/06/202	24	GO	CENTRAL	1779	GOIANIA	520870	HOSPITAL I
02/06/202	22	21/05/202	20	RN	VII URSAP	1416	NATAL	240810	HOSPITAL I
15/03/202	11	28/02/202	9	GO	CENTRAL	1779	GOIANIA	520870	HOSPITAL I
30/03/202	13	05/03/202	9	RS	004 CRS	1595	SANTA MA	431690	HOSPITAL I
28/03/202	13	24/03/202	12	PA	01 REGION	1484	BELEM	150140	SANTA CAS
26/03/202	12	17/03/202	11	SP	GVE XXIX S	1354	SAO JOSE D	354980	HOSPITAL I
16/04/202	15	07/04/202	14	SP	GVE I CAPI	1331	SAO PAULO	355030	HC DA FMU
28/06/202	26	23/06/202	25	SP	GVE I CAPI	1331	SAO PAULO	355030	HOSP MUN
23/04/202	16	15/04/202	15	SP	GVE XXIX S	1354	SAO JOSE D	354980	HOSPITAL I
14/05/202	19	01/03/202	9	MA	REGI0NAL	1430	SAO LUIS	211130	MATERNID
03/06/202	22	04/05/202	18	ES	METROPOL	1510	SERRA	320500	HOSPITAL I
06/05/202	18	26/04/202	17	CE	11 CRES SO	1521	SOBRAL	231290	HOSPITAL I
24/05/202	21	17/05/202	20	PR	14RS PARA	1368	PARANAVA	411840	SANTA CAS
10/08/202	32	09/08/202	32	PE	001	1497	RECIFE	261160	IMIP
28/05/202	21	25/05/202	21	RJ			RIO DE JAN	330455	MS HSE HO
22/02/202	8	15/02/202	7	SC	ITAJAI	1550	BALNEARIO	420200	HOSPITAL I
24/02/202	8	19/02/202	7	MG	UBERLAND	1462	UBERLAND	317020	HOSPITAL I
26/03/202	12	14/03/202	11	SP	GVE I CAPI	1331	SAO PAULO	355030	HC DA FMU
16/04/202	15	20/03/202	11	SP	GVE I CAPI	1331	SAO PAULO	355030	MATERNID
03/05/202	18	20/04/202	16	RO	VI GRS POR	1483	PORTO VEL	110020	HOSPITAL I
16/04/202	15	07/04/202	14	GO	CENTRAL	1779	GOIANIA	520870	HOSPITAL I
09/05/202	19	29/04/202	17	SP	GVE VIII M	1333	MOGI DAS	353060	MOGI MAT
06/06/202	23	30/05/202	22	SP	GVE XI ARA	1336	ARACATUB	350280	SANTA CAS
24/05/202	21	18/05/202	20	SP	GVE XXIV R	1348	RIBEIRAO P	354340	HOSPITAL S
25/02/202	8	29/01/202	4	SP	GVE I CAPI	1331	SAO PAULO	355030	MATERNID
06/06/202	23	30/05/202	22	RS	002 CRS	1607	PORTO ALE	431490	HOSPITAL I
19/06/202	24	10/06/202	23	MA	REGI0NAL	1430	SAO LUIS	211130	MATERNID
05/09/202	36	29/08/202	35	SP	GVE XXVII S	1351	SAO JOSE D	354990	HOSPITAL V
08/09/202	36	01/09/202	35	RJ			CAMPOS D	330100	HOSPITAL I
04/03/202	9	24/02/202	8	RN	II URSAP	1412	MOSSORO	240800	HOSPITAL I
13/03/202	10	01/03/202	9	RS	012 CRS	1601	SANTO AN	431750	HOSPITAL S
29/03/202	13	16/03/202	11	SP	GVE I CAPI	1331	SAO PAULO	355030	MATERNID
25/03/202	12	14/03/202	11	PB	I NRS JOAO	1377	JOAO PESS	250750	MATERNID
10/06/202	23	23/05/202	21	MG	BELO HORI	1449	BETIM	310670	HOSPITAL I
20/04/202	16	12/04/202	15	GO	CENTRAL	1779	GOIANIA	520870	HOSPITAL I
24/05/202	21	14/05/202	19	TO			PALMAS	172100	HOSPITAL G
16/06/202	24	12/06/202	23	RJ			RIO DE JAN	330455	MS HSE HO
25/05/202	21	19/05/202	20	MT	CUIABA	1578	CUIABA	510340	HOSPITAL U
25/05/202	21	15/05/202	19	SC	JOINVILLE	1565	JOINVILLE	420910	HOSPITAL I
19/06/202	24	08/06/202	23	SP	GVE VIII M	1333	GUARULHO	351880	COMPLEXO
14/06/202	24	31/05/202	22	BA	NUCLEO RE	1380	LAURO DE	291920	HOSPITAL I
25/05/202	21	21/05/202	20	SP	GVE VII SA	1332	SANTO AN	354780	HOSPITAL I
13/07/202	28	02/07/202	26	MG	POUSO ALE	1459	ITAJUBA	313240	SANTA CAS
23/07/202	29	16/06/202	24	GO	CENTRAL	1779	GOIANIA	520870	HOSPITAL I

30/07/202	30	02/07/202	26	SP	GVE I CAPI	1331	SAO PAUL(	355030	HC DA FMU
08/10/202	40	02/10/202	39	RJ			RIO DE JAN	330455	MS HSE HC
15/04/202	15	08/04/202	14	PB	I NRS JOAC	1377	JOAO PESS	250750	MATERNID
28/01/202	4	13/01/202	2	AM	ENTORNO	5584	MANAUS	130260	MATERNID
16/03/202	11	08/03/202	10	PA	01 REGION	1484	BELEM	150140	SANTA CAS
20/04/202	16	11/04/202	15	SP	GVE XVI BC	1341	BOTUCATU	350750	HOSPITAL I
18/04/202	16	13/04/202	15	RJ			RIO DE JAN	330455	HOSPITAL (
04/04/202	14	29/03/202	13	SP	GVE I CAPI	1331	SAO PAUL(	355030	HOSPITAL I
01/07/202	26	29/06/202	26	SP	GVE I CAPI	1331	SAO PAUL(	355030	UNIDADE D
10/05/202	19	26/04/202	17	PR	02RS METF	1356	CAMPO LA	410420	HOSPITAL I
10/06/202	23	25/05/202	21	SE	REGIONAL	2056	ARACAJU	280030	HOSPITAL (
24/05/202	21	13/05/202	19	PR	10RS CASC	1364	CASCAVEL	410480	HOSPITAL U
24/06/202	25	18/06/202	24	SP	GVE XVII C.	1342	VALINHOS	355620	HOSPITAL I
24/06/202	25	09/06/202	23	CE	11 CRES SC	1521	SOBRAL	231290	HOSPITAL I
05/07/202	27	21/06/202	25	MA	REGIONAL	1441	BALSAS	210140	HOSPITAL I
15/07/202	28	08/07/202	27	SP	GVE XX PIR	1345	LIMEIRA	352690	HOSPITAL I
09/03/202	10	08/03/202	10	PR	15RS MARI	1369	MARINGA	411520	HOSPITAL U
01/05/202	17	22/04/202	16	SP	GVE XXIX S	1354	SAO JOSE D	354980	HOSPITAL I
16/04/202	15	09/04/202	14	RJ			RIO DE JAN	330455	MS HSE HC
17/05/202	20	11/05/202	19	SP	GVE XVIII F	1343	ORLANDIA	353430	HOSPITAL I
24/03/202	12	14/03/202	11	MG	CORONEL I	1468	CARATINGA	311340	CASU IRMA
25/05/202	21	14/05/202	19	MA	REGIONAL	1440	IMPERATRI	210530	HRMI HOSI
11/06/202	23	01/06/202	22	MG	DIVINOPOI	1472	DIVINOPOI	312230	HOSPITAL S
25/05/202	21	23/05/202	21	MG	LEOPOLDIN	1453	LEOPOLDIN	313840	CASA DE C.
25/07/202	30	20/07/202	29	BA	NUCLEO RI	1380	SALVADOR	292740	MATERNID
26/07/202	30	17/07/202	28	SP	GVE XII AR.	1337	SAO CARLC	354890	SANTA CAS
12/07/202	28	06/07/202	27	RJ			NILOPOLIS	330320	DOMINGO.
03/08/202	31	27/07/202	30	MT	CUIABA	1578	CUIABA	510340	HOSPITAL I
22/12/202	51	21/12/202	51	PE	001	1497	RECIFE	261160	IMIP
04/04/202	14	23/03/202	12	SP	GVE XI ARA	1336	BIRIGUI	350650	SANTA CAS
17/04/202	15	02/04/202	13	RJ			RIO DE JAN	330455	MS HSE HC
26/04/202	17	14/04/202	15	DF			BRASILIA	530010	HRAN
08/04/202	14	01/04/202	13	PR	15RS MARI	1369	MARINGA	411520	SANTA CAS
13/04/202	15	03/04/202	13	GO	SUDOESTE	1799	RIO VERDE	521880	HOSPITAL U
13/05/202	19	04/05/202	18	SP	GVE XXIV F	1348	RIBEIRAO F	354340	HOSPITAL I
16/06/202	24	09/06/202	23	MG	UBERLAND	1462	UBERLAND	317020	HOSPITAL S
01/06/202	22	26/04/202	17	AP			MACAPA	160030	HOSPITAL I
08/06/202	23	03/06/202	22	BA	NUCLEO RI	1380	SALVADOR	292740	HOSPITAL S
18/06/202	24	04/06/202	22	RS	004 CRS	1595	SANTA MA	431690	HUSM HOS
20/07/202	29	20/07/202	29	MA	REGI0NAL	1430	SAO LUIS	211130	MATERNID
05/07/202	27	21/06/202	25	GO	CENTRAL	1779	GOIANIA	520870	HOSPITAL I
17/06/202	24	05/06/202	22	BA	NUCLEO RI	1381	FEIRA DE S.	291080	HOSPITAL I
14/03/202	11	21/02/202	8	SP	GVE I CAPI	1331	SAO PAUL(	355030	MATERNID
29/06/202	26	18/06/202	24	CE	11 CRES SC	1521	SOBRAL	231290	HOSPITAL I
14/01/202	2	03/01/202	1	RS	002 CRS	1607	PORTO ALE	431490	IRMANDAD
30/06/202	26	23/06/202	25	MT	CUIABA	1578	CUIABA	510340	HOSPITAL U
31/12/202	52	21/12/202	51	PR	09RS FOZ D	1363	FOZ DO IGU	410830	HOSPITAL I

22/02/202	8	17/02/202	7	RS	013 CRS	1609	SANTA CRU	431680	HOSPITAL S
09/03/202	10	28/02/202	9	PR	02RS METR	1356	CURITIBA	410690	ONIX MATE
31/03/202	13	19/03/202	11	BA	NUCLEO RE	1380	SALVADOR	292740	MATERNID
15/02/202	7	05/02/202	5	PR	04RS IRATI	1358	IRATI	411070	SANTA CAS
13/04/202	15	06/04/202	14	RJ			RIO DE JAN	330455	HOSPITAL (
28/02/202	9	24/02/202	8	SP	GVE VIII M	1333	GUARULHO	351880	HOSPITAL I
27/04/202	17	26/04/202	17	RJ			RIO DE JAN	330455	PERINATAL
27/05/202	21	11/05/202	19	SP	GVE XIX MA	1344	TUPA	355500	SANTA CAS
14/06/202	24	02/06/202	22	MT	CUIABA	1578	CUIABA	510340	HOSPITAL U
04/10/202	40	13/05/202	19	BA	NUCLEO RE	1380	SALVADOR	292740	HOSPITAL T
20/07/202	29	10/07/202	27	SP	GVE I CAPIT	1331	SAO PAULO	355030	HC DA FMU
19/11/202	46	07/11/202	45	ES	METROPOL	1510	SERRA	320500	HOSPITAL I
03/04/202	13	25/03/202	12	MS	EX ARS DE	1975	CAMPO GR	500270	HOSPITAL I
16/03/202	11	13/03/202	10	RJ			NITEROI	330330	HOSPITAL I
01/04/202	13	24/03/202	12	MG	POUSO ALE	1459	POUSO ALE	315250	HOSPITAL I
05/04/202	14	01/04/202	13	SP	GVE XVI BO	1341	BOTUCATU	350750	HOSPITAL I
18/03/202	11	14/03/202	11	BA	NUCLEO RE	1381	FEIRA DE SA	291080	HOSPITAL I
26/04/202	17	23/04/202	16	RS	005 CRS	1608	CAXIAS DO	430510	HOSPITAL (
10/03/202	10	19/02/202	7	MT	CUIABA	1578	CUIABA	510340	HOSPITAL S
07/04/202	14	29/03/202	13	SP	GVE XVII CA	1342	CAMPINAS	350950	HOSPITAL I
26/05/202	21	10/05/202	19	SP	GVE I CAPIT	1331	SAO PAULO	355030	MATERNID
28/06/202	26	19/06/202	24	PR	17RS LOND	1371	LONDRINA	411370	HOSPITAL U
24/05/202	21	17/05/202	20	SP	GVE X OSA	1335	ITAPEVI	352250	HOSPITAL (
09/08/202	32	03/08/202	31	DF			BRASILIA	530010	HRAN
21/06/202	25	19/06/202	24	MG	CORONEL F	1468	IPATINGA	313130	HOSPITAL I
25/06/202	25	22/06/202	25	SP	GVE XVII CA	1342	CAMPINAS	350950	MATERNID
22/07/202	29	03/07/202	26	SP	GVE I CAPIT	1331	SAO PAULO	355030	SAO LUIZ U
29/07/202	30	20/07/202	29	MT	CUIABA	1578	CUIABA	510340	HOSPITAL U
23/07/202	29	15/07/202	28	MG	UBA	1464	MURIAE	314390	CASA DE CA
06/08/202	31	29/07/202	30	PR	10RS CASC	1364	CASCAVEL	410480	HOSPITAL U
31/07/202	30	27/07/202	30	PR	17RS LOND	1371	LONDRINA	411370	HOSPITAL I
08/11/202	45	02/11/202	44	SP	GVE VII SAI	1332	SAO CAETA	354880	HOSPITAL S
21/01/202	3	17/01/202	3	SP	GVE XXVII S	1351	SAO JOSE D	354990	HOSPITAL V
02/04/202	13	15/03/202	11	SP	GVE XXI PR	1346	PRESIDENT	354140	SANTA CAS
13/03/202	10	04/03/202	9	SC	VIDEIRA	1546	CURITIBAN	420480	HOSPITAL I
07/03/202	10	27/02/202	8	PA	01 REGION	1484	BELEM	150140	SANTA CAS
20/04/202	16	09/04/202	14	GO	CENTRAL	1779	GOIANIA	520870	HOSPITAL I
26/05/202	21	29/04/202	17	SP	GVE I CAPIT	1331	SAO PAULO	355030	MATERNID
05/05/202	18	26/04/202	17	BA	NUCLEO RE	1380	SALVADOR	292740	MATERNID
14/05/202	19	02/02/202	5	MA	REGI0NAL	1430	SAO LUIS	211130	MATERNID
28/05/202	21	16/05/202	20	SP	GVE X OSA	1335	OSASCO	353440	HOSPITAL (
24/05/202	21	09/05/202	19	BA	NUCLEO RE	1403	BARREIRAS	290320	HOSPITAL I
25/05/202	21	13/05/202	19	MS	EX ARS DE	1975	CAMPO GR	500270	HOSPITAL I
10/05/202	19	23/04/202	16	RJ			RIO DE JAN	330455	PERINATAL
20/04/202	16	16/04/202	15	SP	GVE VII SAI	1332	MAUA	352940	HOSPITAL I
27/05/202	21	20/04/202	16	SP	GVE I CAPIT	1331	SAO PAULO	355030	MATERNID
18/05/202	20	10/05/202	19	MG	JUIZ DE FO	1452	JUIZ DE FO	313670	SANTA CAS

22/06/202	25	10/06/202	23	RJ			RIO DE JAN	330455	SMS RIO H
14/06/202	24	01/06/202	22	RN	II URSAP	1412	MOSSORO	240800	HOSPITAL
08/07/202	27	29/06/202	26	BA	NUCLEO RE	1380	SALVADOR	292740	MATERNID
24/08/202	34	20/08/202	33	SP	GVE XXI PR	1346	PRESIDENT	354140	HOSPITAL
29/05/202	21	22/05/202	20	SP	GVE XXIX S	1354	VOTUPORA	355710	SANTA CAS
26/07/202	30	13/07/202	28	GO	CENTRAL	1779	GOIANIA	520870	HOSPITAL
04/06/202	22	31/05/202	22	TO			PALMAS	172100	HOSPITAL
09/06/202	23	05/06/202	22	SP	GVE VII SA	1332	SAO BERNA	354870	HOSPITAL
08/07/202	27	05/07/202	27	GO	CENTRAL	1779	GOIANIA	520870	HOSPITAL
06/03/202	9	03/03/202	9	MG	UBERLAND	1462	UBERLAND	317020	HOSPITAL
03/03/202	9	15/02/202	7	RS	008 CRS	1597	CACHOEIRA	430300	HOSPITAL
05/03/202	9	14/02/202	7	SP	GVE I CAPI	1331	SAO PAUL	355030	HOSP MUN
18/03/202	11	04/03/202	9	RS	006 CRS	1611	PASSO FUN	431410	HOSPITAL
13/03/202	10	04/03/202	9	SP	GVE I CAPI	1331	SAO PAUL	355030	HOSPITAL
02/04/202	13	30/03/202	13	SP	GVE IX FRA	1334	FRANCISCO	351630	HOSPITAL
10/03/202	10	28/02/202	9	PR	11RS CAMI	1365	CAMPO M	410430	HOSPITAL
11/05/202	19	07/05/202	18	RS	006 CRS	1611	ESPUMOSO	430750	HOSPITAL
25/05/202	21	18/05/202	20	GO	CENTRAL	1779	GOIANIA	520870	HOSPITAL
07/06/202	23	01/06/202	22	SP	GVE I CAPI	1331	SAO PAUL	355030	HOSP MUN
30/07/202	30	20/07/202	29	SP	GVE XXVII	1351	SAO JOSE D	354990	HOSPITAL
01/07/202	26	18/06/202	24	MG	PATOS DE	1456	PATOS DE	314800	HOSPITAL
18/07/202	29	14/07/202	28	SP	GVE XXVI S	1350	ITAPIRA	352260	HOSPITAL
13/07/202	28	03/07/202	26	MT	CUIABA	1578	CUIABA	510340	FEMINA H
08/03/202	10	25/02/202	8	GO	CENTRAL	1779	GOIANIA	520870	HOSPITAL
16/04/202	15	20/03/202	11	RS	002 CRS	1607	PORTO ALE	431490	HOSPITAL
26/03/202	12	23/03/202	12	RJ			RIO DE JAN	330455	SMS HOSPI
05/04/202	14	21/03/202	12	SP	GVE XII AR	1337	SAO CARL	354890	SANTA CAS
04/03/202	9	03/02/202	5	GO	CENTRAL	1779	GOIANIA	520870	HOSPITAL
30/04/202	17	19/04/202	16	DF			BRASILIA	530010	HRSM
24/04/202	16	10/04/202	14	RO	VI GRS PO	1483	PORTO VEL	110020	HOSPITAL
10/05/202	19	28/04/202	17	MT	CUIABA	1578	CUIABA	510340	HOSPITAL
08/04/202	14	27/03/202	12	AL	1 MICROR	1533	MACEIO	270430	HOSPITAL
22/02/202	8	22/02/202	8	CE	1 CRES FO	1519	FORTALEZ	230440	HGCC HOS
06/06/202	23	01/06/202	22	PA	01 REGION	1484	BELEM	150140	SANTA CAS
26/06/202	25	12/06/202	23	SP	GVE XVII C	1342	JUNDIAI	352590	HU HOSPIT
02/07/202	26	21/06/202	25	DF			BRASILIA	530010	HRAN
15/04/202	15	04/04/202	14	RJ			RIO DE JAN	330455	PERINATAL
14/06/202	24	05/06/202	22	GO	CENTRAL	1779	GOIANIA	520870	HOSPITAL
17/06/202	24	16/05/202	20	GO	CENTRAL	1779	GOIANIA	520870	HOSPITAL
23/03/202	12	09/03/202	10	RR			BOA VISTA	140010	HOSPITAL
30/08/202	35	11/08/202	32	RJ			CAMPOS D	330100	HOSPITAL
12/05/202	19	12/05/202	19	CE	1 CRES FO	1519	FORTALEZ	230440	HGCC HOS
12/01/202	2	10/01/202	2	GO	CENTRAL	1779	GOIANIA	520870	HOSPITAL
21/07/202	29	15/07/202	28	MG	VARGINHA	1466	VARGINHA	317070	HOSPITAL
04/09/202	35	28/08/202	34	RS	002 CRS	1607	PORTO ALE	431490	HOSPITAL
01/01/202	52	29/12/202	52	AC	REGIONAL	1941	CRUZEIRO	120020	HOSPITAL
05/04/202	14	26/03/202	12	SP	GVE I CAPI	1331	SAO PAUL	355030	HOSPITAL

07/04/202	14	01/03/202	9	CE	11 CRES SC	1521	SOBRAL	231290	HOSPITAL I
15/04/202	15	09/04/202	14	AC	REGIONAL	1938	RIO BRANC	120040	HOSPITAL (
10/05/202	19	30/04/202	17	RS	018 CRS	1605	TRAMAND.	432160	HOSPITAL T
03/05/202	18	24/04/202	16	SP	GVE XVII C.	1342	CAMPINAS	350950	HOSPITAL S
28/05/202	21	14/05/202	19	SP	GVE I CAPI	1331	SAO PAULC	355030	PRO MATR
09/06/202	23	28/05/202	21	RJ			GUAPIMIR	330185	HOSPITAL I
08/06/202	23	31/05/202	22	RJ			CAMPOS D	330100	HOSPITAL I
29/04/202	17	19/04/202	16	RJ			RIO DE JAN	330455	MS HSE HC
21/06/202	25	02/06/202	22	GO	CENTRAL	1779	GOIANIA	520870	HOSPITAL I
23/06/202	25	31/05/202	22	SP	GVE XXXI S	1353	SOROCABA	355220	SANTA CAS
30/05/202	22	20/05/202	20	SP	GVE X OSA	1335	OSASCO	353440	HOSPITAL I
27/09/202	39	16/09/202	37	ES	METROPOI	1510	SERRA	320500	HOSPITAL I
23/08/202	34	10/08/202	32	RJ			RIO DE JAN	330455	SMS HOSPI
21/06/202	25	21/06/202	25	MT	CUIABA	1578	CUIABA	510340	FEMINA HC
06/07/202	27	12/06/202	23	PE	001	1497	RECIFE	261160	PRONTO A
26/02/202	8	21/02/202	8	SP	GVE IX FRA	1334	FRANCISCC	351630	HOSPITAL I
05/02/202	5	03/02/202	5	SC	JOINVILLE	1565	JOINVILLE	420910	HOSPITAL I
06/03/202	9	25/02/202	8	SP	GVE VII SAI	1332	SANTO AN	354780	HOSPITAL I
22/03/202	12	13/03/202	10	RS	001 CRS	1592	NOVO HAN	431340	HOSPITAL I
14/04/202	15	03/04/202	13	PR	17RS LONE	1371	LONDRINA	411370	HOSPITAL U
18/05/202	20	01/05/202	17	MS	EX ARS DE	1975	CAMPO GF	500270	HOSPITAL I
26/05/202	21	13/05/202	19	MT	SINOP	1584	SORRISO	510792	HOSPITAL I
23/05/202	21	20/05/202	20	MS	NRS DE DO	1966	DOURADO.	500370	EBSERH HC
26/05/202	21	10/05/202	19	SP	GVE XVII C.	1342	CAMPINAS	350950	MATERNID
27/04/202	17	27/04/202	17	CE	1 CRES FOF	1519	FORTALEZ/	230440	HGCC HOS
20/07/202	29	10/07/202	27	PA	11 REGION	1494	MARABA	150420	HOSPITAL I
10/06/202	23	30/05/202	22	PR	15RS MARI	1369	SARANDI	412625	METROPOI
22/07/202	29	13/07/202	28	SP	GVE I CAPI	1331	SAO PAULC	355030	SAO LUIZ U
29/07/202	30	19/07/202	29	MT	CUIABA	1578	CUIABA	510340	HOSPITAL I
18/10/202	42	29/09/202	39	SP	GVE I CAPI	1331	SAO PAULC	355030	MATERNID
10/08/202	32	01/08/202	31	PR	10RS CASC	1364	CASCAVEL	410480	HOSPITAL U
27/02/202	8	25/01/202	4	RJ			RIO DE JAN	330455	UERJ HOSP
22/04/202	16	09/04/202	14	PR	02RS METF	1356	CURITIBA	410690	COMPLEXC
14/04/202	15	03/04/202	13	SP	GVE I CAPI	1331	SAO PAULC	355030	HC DA FMU
26/04/202	17	14/04/202	15	MT	CUIABA	1578	CUIABA	510340	HOSPITAL U
04/06/202	22	27/05/202	21	PR	10RS CASC	1364	CASCAVEL	410480	HOSPITAL U
26/05/202	21	20/03/202	11	BA	NUCLEO RI	1380	CAMACARI	290570	HOSPITAL (
04/08/202	31	27/07/202	30	PR	02RS METF	1356	CAMPINA (	410400	HOSPITAL A
04/08/202	31	25/07/202	30	MT	CUIABA	1578	CUIABA	510340	HOSPITAL U
19/04/202	16	13/04/202	15	CE	1 CRES FOF	1519	FORTALEZ/	230440	HGCC HOS
24/06/202	25	13/06/202	24	MG	BELO HORI	1449	BELO HORI	310620	HOSPITAL J
19/07/202	29	12/07/202	28	RJ			RIO DE JAN	330455	MS HSE HC
21/07/202	29	12/07/202	28	MG	POUSO ALI	1459	ITAJUBA	313240	AISI HOSPI
07/02/202	6	31/01/202	5	SP	GVE I CAPI	1331	SAO PAULC	355030	HOSPITAL I
03/03/202	9	15/02/202	7	GO	CENTRAL	1779	GOIANIA	520870	HOSPITAL I
24/02/202	8	13/02/202	6	SC	JOINVILLE	1565	JOINVILLE	420910	HOSPITAL I
29/03/202	13	15/03/202	11	SP	GVE I CAPI	1331	SAO PAULC	355030	HOSPITAL S

29/03/202	13	19/03/202	11	MG	GOVERNAI	1471	MANTENA	313960	HOSPITAL
22/03/202	12	15/03/202	11	RJ			MIGUEL PE	330290	HOSPITAL
08/06/202	23	28/05/202	21	SP	GVE XXIX S	1354	SAO JOSE D	354980	HOSPITAL
24/06/202	25	21/06/202	25	PR	10RS CASC	1364	CASCAVEL	410480	HOSPITAL
26/05/202	21	13/05/202	19	PR	02RS METF	1356	CAMPINA	410400	HOSPITAL
22/06/202	25	14/06/202	24	PA	10 REGION	1493	PORTO DE	150590	HOSPITAL
31/03/202	13	30/03/202	13	SP	GVE XXV S	1349	SANTOS	354850	HOSPITAL
26/06/202	25	13/06/202	24	RN	VII URSAP	1416	NATAL	240810	HOSPITAL
12/07/202	28	01/07/202	26	SP	GVE XXIV F	1348	RIBEIRAO F	354340	HOSPITAL
16/07/202	28	02/07/202	26	MA	REGIONAL	1440	IMPERATRI	210530	HRMI HOSI
06/03/202	9	23/02/202	8	SP	GVE XXIX S	1354	SAO JOSE D	354980	HOSPITAL
21/07/202	29	14/07/202	28	RR			BOA VISTA	140010	HOSPITAL
11/04/202	15	06/04/202	14	RJ			RIO DE JAN	330455	SMS COOR
29/04/202	17	19/04/202	16	RS	005 CRS	1608	CAXIAS DO	430510	HOSPITAL
24/03/202	12	05/03/202	9	DF			BRASILIA	530010	HRSM
14/05/202	19	10/05/202	19	PR	15RS MARI	1369	MARINGA	411520	SANTA CAS
31/05/202	22	27/05/202	21	RS	007 CRS	1596	BAGE	430160	SANTA CAS
26/05/202	21	18/05/202	20	SP	GVE I CAPI	1331	SAO PAULO	355030	HC DA FMU
16/06/202	24	24/05/202	21	CE	13 CRES TI	1523	TIANGUA	231340	HOSPITAL
08/09/202	36	21/08/202	33	MT	CUIABA	1578	CUIABA	510340	HOSPITAL
12/07/202	28	02/07/202	26	RN	II URSAP	1412	MOSSORO	240800	HOSPITAL
25/06/202	25	11/06/202	23	RS	004 CRS	1595	SANTA MA	431690	HUSM HOS
08/07/202	27	05/07/202	27	SP	GVE XVII C	1342	CAMPINAS	350950	MATERNID
21/05/202	20	21/05/202	20	CE	1 CRES FOF	1519	FORTALEZA	230440	HGCC HOS
17/03/202	11	11/03/202	10	SP	GVE XVII C	1342	JUNDIAI	352590	HU HOSPIT
23/03/202	12	13/03/202	10	RS	002 CRS	1607	PORTO ALE	431490	HOSPITAL
10/03/202	10	03/03/202	9	SP	GVE XVII C	1342	CAMPINAS	350950	HOSPITAL
01/03/202	9	26/02/202	8	PA	12 REGION	1495	REDENCAC	150613	HOSPITAL
28/03/202	13	17/03/202	11	SP	GVE XVI BC	1341	SAO MANU	355010	HOSPITAL
21/04/202	16	04/04/202	14	MG	BELO HORI	1449	CONTAGEN	311860	HOSPITAL
07/06/202	23	25/05/202	21	SP	GVE I CAPI	1331	SAO PAULO	355030	HC DA FMU
08/04/202	14	24/03/202	12	SP	GVE XXIX S	1354	SAO JOSE D	354980	HOSPITAL
10/05/202	19	27/04/202	17	BA	NUCLEO RI	1381	FEIRA DE S	291080	HOSPITAL
20/05/202	20	17/05/202	20	MG	UBA	1464	VISCONDE	317200	HOSPITAL
12/04/202	15	04/04/202	14	SP	GVE XXI PR	1346	PRESIDENT	354140	HOSPITAL
24/04/202	16	07/04/202	14	TO			PALMAS	172100	HOSPITAL
04/06/202	22	11/04/202	15	ES	METROPOI	1510	SERRA	320500	HOSPITAL
07/06/202	23	03/06/202	22	RJ			RIO DE JAN	330455	MS HSE HC
10/05/202	19	02/05/202	18	ES	METROPOI	1510	SERRA	320500	HOSPITAL
22/07/202	29	10/07/202	27	RJ			RIO DE JAN	330455	HOSPITAL
15/05/202	19	12/05/202	19	RN	VII URSAP	1416	NATAL	240810	HOSPITAL
01/06/202	22	27/05/202	21	MG	PASSOS	1455	PIUMHI	315150	SANTA CAS
12/01/202	2	08/01/202	1	PR	17RS LOND	1371	LONDRINA	411370	HOSPITAL
16/08/202	33	15/08/202	33	RR			BOA VISTA	140010	HOSPITAL
20/03/202	11	08/03/202	10	SP	GVE XXIX S	1354	SAO JOSE D	354980	HOSPITAL
25/03/202	12	14/03/202	11	MG	BELO HORI	1449	CONTAGEN	311860	HOSPITAL
19/03/202	11	13/03/202	10	SP	GVE XXXI S	1353	SOROCABA	355220	CONJUNTC

05/05/202	18	28/04/202	17	GO	CENTRAL	1779	GOIANIA	520870	HOSPITAL
24/05/202	21	19/05/202	20	RJ			RIO DE JAN	330455	MS HSE HC
15/05/202	19	14/05/202	19	PE	001	1497	RECIFE	261160	IMIP
16/06/202	24	12/06/202	23	PR	10RS CASC	1364	CASCAVEL	410480	HOSPITAL
20/05/202	20	17/05/202	20	PA	11 REGION	1494	MARABA	150420	HOSPITAL
15/05/202	19	08/05/202	18	CE	21 CRES JU	1531	JUAZEIRO	230730	HOSPITAL
17/05/202	20	10/05/202	19	SP	GVE VIII M	1333	MOGI DAS	353060	HOSPITAL
10/07/202	27	29/06/202	26	SP	GVE VIII M	1333	GUARULHO	351880	HOSPITAL
09/09/202	36	27/08/202	34	SC	CHAPECO	1553	CHAPECO	420420	HOSPITAL
30/08/202	35	01/08/202	31	SP	GVE I CAPI	1331	SAO PAULO	355030	HC DA FMU
25/06/202	25	11/06/202	23	SP	GVE I CAPI	1331	SAO PAULO	355030	HC DA FMU
05/10/202	40	29/09/202	39	RJ			RIO DE JAN	330455	MS HSE HC
14/06/202	24	07/06/202	23	BA	NUCLEO RE	1380	SALVADOR	292740	MATERNID
09/04/202	14	09/04/202	14	CE	1 CRES FOR	1519	FORTALEZA	230440	HGCC HOS
26/09/202	39	20/09/202	38	SP	GVE XXI PR	1346	PRESIDENT	354140	HOSPITAL
17/07/202	28	30/06/202	26	RS	002 CRS	1607	PORTO ALE	431490	HOSPITAL
11/03/202	10	08/03/202	10	RJ			RIO DAS OS	330452	PRONTO SO
14/04/202	15	11/04/202	15	SP	GVE IX FRA	1334	FRANCISCO	351630	HOSPITAL
26/02/202	8	06/02/202	5	RO	VI GRS POR	1483	PORTO VEL	110020	HOSPITAL
18/03/202	11	03/03/202	9	RN	VII URSAP	1416	NATAL	240810	HOSPITAL
19/04/202	16	07/04/202	14	GO	CENTRAL	1779	GOIANIA	520870	HOSPITAL
31/05/202	22	20/05/202	20	PA	01 REGION	1484	BELEM	150140	SANTA CAS
19/04/202	16	16/04/202	15	MT	CUIABA	1578	CUIABA	510340	HOSPITAL
03/06/202	22	28/05/202	21	PR	17RS LOND	1371	LONDRINA	411370	HOSPITAL
20/06/202	25	02/06/202	22	PB	I NRS JOAO	1377	JOAO PESS	250750	MATERNID
11/11/202	45	17/10/202	42	SP	GVE I CAPI	1331	SAO PAULO	355030	HC DA FMU
22/02/202	8	07/02/202	6	SP	GVE I CAPI	1331	SAO PAULO	355030	HOSPITAL
22/03/202	12	11/03/202	10	SP	GVE I CAPI	1331	SAO PAULO	355030	HOSPITAL
25/02/202	8	24/02/202	8	RR			BOA VISTA	140010	HOSPITAL
19/01/202	3	15/01/202	2	AC	REGIONAL	1938	RIO BRANC	120040	HOSPITAL
04/03/202	9	24/02/202	8	MT	CUIABA	1578	CUIABA	510340	HOSPITAL
16/02/202	7	03/02/202	5	SC	JOINVILLE	1565	JOINVILLE	420910	CENTRO HO
22/04/202	16	15/04/202	15	GO	CENTRO SU	1781	APARECIDA	520140	HOSPITAL
01/04/202	13	25/03/202	12	SP	GVE I CAPI	1331	SAO PAULO	355030	MATERNID
21/03/202	12	13/03/202	10	SP	GVE XVII CA	1342	JUNDIAI	352590	HU HOSPIT
12/04/202	15	12/04/202	15	SP	GVE XXVIII	1576	ILHABELA	352040	HOSPITAL
10/05/202	19	06/05/202	18	MG	SETE LAGO	1467	SETE LAGO	316720	HOSPITAL
23/07/202	29	14/06/202	24	GO	CENTRAL	1779	GOIANIA	520870	HOSPITAL
24/06/202	25	16/06/202	24	PB	III NRS CAM	1421	CAMPINA	250400	INSTITUTO
04/08/202	31	31/07/202	30	RJ			RIO DE JAN	330455	SMS RIO H
29/07/202	30	26/07/202	30	SP	GVE VIII M	1333	GUARULHO	351880	HOSPITAL
13/04/202	15	04/04/202	14	PR	20RS TOLE	1374	MARECHAL	411460	HOSPITAL
19/03/202	11	09/03/202	10	SP	GVE XVII CA	1342	CAMPINAS	350950	MATERNID
07/04/202	14	01/04/202	13	TO			PALMAS	172100	HOSPITAL
18/05/202	20	30/04/202	17	RO	IV GRS ARI	1481	CUJUBIM	110094	HOSPITAL
02/04/202	13	27/03/202	12	PA	01 REGION	1484	BELEM	150140	SANTA CAS
20/03/202	11	15/03/202	11	PR	15RS MARI	1369	SARANDI	412625	METROPOL

22/03/202	12	01/03/202	9	SP	GVE I CAPI	1331	SAO PAULO	355030	HC DA FMU
07/05/202	18	27/04/202	17	SP	GVE VIII M	1333	GUARULHO	351880	HOSPITAL
23/03/202	12	14/03/202	11	RO	VI GRS POF	1483	PORTO VEL	110020	HOSPITAL
24/06/202	25	12/06/202	23	SP	GVE I CAPI	1331	SAO PAULO	355030	MATERNID
12/08/202	32	23/07/202	29	PI	4 GRS TERE	1883	TERESINA	221100	MATERNID
02/07/202	26	25/06/202	25	RJ			RIO DE JAN	330455	SMS RIO H
22/07/202	29	14/07/202	28	PR	17RS LOND	1371	LONDRINA	411370	HOSPITAL
29/07/202	30	10/07/202	27	RS	002 CRS	1607	PORTO ALE	431490	HOSPITAL
12/11/202	45	02/11/202	44	SC	FLORIANOI	1476	FLORIANOI	420540	HOSPITAL
18/03/202	11	02/03/202	9	SP	GVE I CAPI	1331	SAO PAULO	355030	PRO MATR
10/02/202	6	07/02/202	6	AM	ENTORNO	5584	MANAUS	130260	MATERNID
17/03/202	11	01/03/202	9	SP	GVE VII SA	1332	SAO CAETA	354880	HOSPITAL
17/03/202	11	08/03/202	10	GO	CENTRAL	1779	GOIANIA	520870	HOSPITAL
03/04/202	13	17/03/202	11	GO	CENTRAL	1779	GOIANIA	520870	HOSPITAL
22/03/202	12	21/03/202	12	RJ			RIO DE JAN	330455	SMS HOSP
25/03/202	12	06/03/202	9	SP	GVE I CAPI	1331	SAO PAULO	355030	HOSPITAL
04/05/202	18	13/04/202	15	SP	GVE XXIV F	1348	RIBEIRAO F	354340	HOSPITAL
16/03/202	11	01/03/202	9	GO	SUDOESTE	1799	RIO VERDE	521880	OX RV
02/04/202	13	01/04/202	13	CE	1 CRES FOF	1519	FORTALEZA	230440	HGCC HOS
22/05/202	20	18/05/202	20	PR	10RS CASC	1364	CASCAVEL	410480	HOSPITAL
13/04/202	15	13/04/202	15	CE	11 CRES SC	1521	SOBRAL	231290	SANTA CAS
25/06/202	25	18/06/202	24	MA	REGIONAL	1440	IMPERATRI	210530	HRMI HOSI
17/06/202	24	11/06/202	23	MG	JUIZ DE FO	1452	JUIZ DE FO	313670	HOSPITAL
20/08/202	33	15/08/202	33	RJ			RIO DE JAN	330455	MS HSE HC
16/03/202	11	07/03/202	10	PR	15RS MARI	1369	MARINGA	411520	SANTA CAS
23/02/202	8	10/02/202	6	SC	CHAPECO	1553	CHAPECO	420420	HOSPITAL
12/04/202	15	01/04/202	13	ES	METROPOI	1510	SERRA	320500	HOSPITAL
20/04/202	16	17/04/202	15	SP	GVE XXXI S	1353	ITU	352390	VIGILANCI
22/04/202	16	17/04/202	15	PR	01RS PARA	1355	PARANAGU	411820	HOSPITAL
26/04/202	17	13/04/202	15	TO			PALMAS	172100	HOSPITAL
08/06/202	23	21/05/202	20	MG	PATOS DE I	1456	PATOS DE I	314800	HOSPITAL
22/07/202	29	12/06/202	23	RS	002 CRS	1607	PORTO ALE	431490	HOSPITAL
05/07/202	27	16/06/202	24	SP	GVE XVII C	1342	VALINHOS	355620	HOSPITAL
10/12/202	49	29/11/202	48	SC	RIO DO SUI	1478	RIO DO SUI	421480	HOSPITAL
10/03/202	10	22/02/202	8	ES	METROPOI	1510	SERRA	320500	HOSPITAL
03/03/202	9	22/02/202	8	SP	GVE XVII C	1342	CAMPINAS	350950	HOSPITAL
26/03/202	12	14/03/202	11	SP	GVE XVII C	1342	CAMPINAS	350950	MATERNID
13/04/202	15	22/03/202	12	SP	GVE I CAPI	1331	SAO PAULO	355030	HC DA FMU
08/03/202	10	04/03/202	9	GO	PIRINEUS	1792	ANAPOLIS	520110	SANTA CAS
14/04/202	15	06/04/202	14	MS	EX ARS DE	1975	CAMPO GF	500270	HOSPITAL
03/05/202	18	15/04/202	15	RS	018 CRS	1605	TRAMAND	432160	HOSPITAL
21/04/202	16	16/04/202	15	MT	SINOP	1584	SORRISO	510792	HOSPITAL
28/04/202	17	22/04/202	16	CE	21 CRES JU	1531	JUAZEIRO I	230730	HOSPITAL
17/05/202	20	13/05/202	19	RJ			VALENCA	330610	HOSPITAL
28/04/202	17	10/04/202	14	PR	02RS METF	1356	CAMPINA	410400	HOSPITAL
14/06/202	24	02/06/202	22	SP	GVE I CAPI	1331	SAO PAULO	355030	HOSPITAL
16/08/202	33	13/08/202	32	SP	GVE XXVI S	1350	MOGI GUA	353070	SANTA CAS

10/06/202	23	06/06/202	23	MA	REGI0NAL	1430	SAO LUIS	211130	MATERNID
20/05/202	20	15/04/202	15	RJ			RIO DE JAN	330455	HOSPITAL
24/05/202	21	18/05/202	20	SP	GVE XXVI S	1350	MOGI GUA	353070	SANTA CAS
08/03/202	10	24/02/202	8	PR	10RS CASC	1364	CASCAVEL	410480	HOSPITAL
22/02/202	8	17/02/202	7	SC	FLORIANOI	1476	SAO JOSE	421660	HOSPITAL
04/03/202	9	20/02/202	7	SP	GVE I CAPI	1331	SAO PAUL	355030	HOSPITAL
05/04/202	14	30/03/202	13	RJ			NITEROI	330330	HOSPITAL
09/03/202	10	27/02/202	8	PR	03RS PONT	1357	PONTA GR	411990	HOSPITAL
22/06/202	25	12/06/202	23	MT	CUIABA	1578	CUIABA	510340	HOSPITAL
16/06/202	24	14/01/202	2	RS	001 CRS	1592	CANOAS	430460	HOSPITAL
06/05/202	18	06/05/202	18	SC	MAFRA	1566	MAFRA	421010	HOSPITAL
28/05/202	21	28/05/202	21	PE	001	1497	RECIFE	261160	IMIP
25/03/202	12	16/03/202	11	RN	VII URSAP	1416	NATAL	240810	HOSPITAL
09/12/202	49	13/11/202	45	TO			PALMAS	172100	HOSPITAL
16/07/202	28	01/07/202	26	AP			MACAPA	160030	HOSPITAL
22/10/202	42	30/09/202	39	GO	CENTRAL	1779	GOIANIA	520870	HOSPITAL
02/08/202	31	02/08/202	31	SP	GVE X OSA	1335	OSASCO	353440	HOSPITAL
31/08/202	35	23/08/202	34	SP	GVE I CAPI	1331	SAO PAUL	355030	PRO MATR
12/02/202	6	30/01/202	4	GO	CENTRAL	1779	GOIANIA	520870	HOSPITAL
12/02/202	6	05/02/202	5	SP	GVE XXXI S	1353	SOROCABA	355220	HOSPITAL
26/04/202	17	11/04/202	15	SP	GVE XXVII	1351	JACAREI	352440	HOSPITAL
05/04/202	14	24/03/202	12	BA	NUCLEO RI	1394	IRECE	291460	HOSPITAL
15/04/202	15	07/04/202	14	DF			BRASILIA	530010	HRAN
03/05/202	18	19/04/202	16	SP	GVE XXV S	1349	SANTOS	354850	HOSPITAL
09/06/202	23	23/04/202	16	GO	CENTRAL	1779	GOIANIA	520870	HOSPITAL
06/06/202	23	26/05/202	21	BA	NUCLEO RI	1398	VITORIA D	293330	HOSPITAL
21/06/202	25	07/06/202	23	RJ			RIO DE JAN	330455	MS HSE HC
02/06/202	22	21/05/202	20	SP	GVE I CAPI	1331	SAO PAUL	355030	HOSPITAL
12/07/202	28	03/07/202	26	PR	10RS CASC	1364	CASCAVEL	410480	HOSPITAL
04/01/202	1	01/01/202	52	DF			BRASILIA	530010	HOSPITAL
18/01/202	3	12/01/202	2	SP	GVE VIII M	1333	GUARULH	351880	HOSPITAL
23/12/202	51	22/12/202	51	SP	GVE XXVII	1351	SAO JOSE	354990	HOSPITAL
20/04/202	16	09/04/202	14	SC	LAGES	1571	LAGES	420930	HOSPITAL
17/05/202	20	07/05/202	18	RS	018 CRS	1605	TRAMAND	432160	HOSPITAL
17/02/202	7	09/02/202	6	PR	17RS LOND	1371	LONDRINA	411370	HOSPITAL
04/06/202	22	22/05/202	20	SP	GVE I CAPI	1331	SAO PAUL	355030	MATERNID
01/06/202	22	20/05/202	20	RS	018 CRS	1605	TRAMAND	432160	HOSPITAL
05/06/202	22	28/05/202	21	PR	05RS GUAI	1359	GUARAPU	410940	HOSPITAL
01/10/202	39	30/09/202	39	PR	10RS CASC	1364	CASCAVEL	410480	HOSPITAL
29/03/202	13	01/03/202	9	ES	METROPOI	1510	SERRA	320500	HOSPITAL
21/03/202	12	15/03/202	11	SP	GVE X OSA	1335	OSASCO	353440	HOSPITAL
03/04/202	13	23/03/202	12	PA	01 REGION	1484	BELEM	150140	SANTA CAS
10/06/202	23	03/06/202	22	SC	JOINVILLE	1565	JOINVILLE	420910	HOSPITAL
16/06/202	24	21/05/202	20	PR	03RS PONT	1357	PONTA GR	411990	SANTA CAS
06/06/202	23	03/06/202	22	SP	GVE I CAPI	1331	SAO PAUL	355030	HOSPITAL
09/07/202	27	01/07/202	26	AM	ENTORNO	5584	MANAUS	130260	MATERNID
15/06/202	24	01/06/202	22	PR	15RS MARI	1369	SARANDI	412625	METROPOI

10/07/202	27	10/07/202	27	RJ			BOM JESUS	330060	HOSPITAL S
23/08/202	34	09/08/202	32	GO	CENTRAL	1779	GOIANIA	520870	HOSPITAL I
06/12/202	49	01/12/202	48	BA	NUCLEO RE	1380	SALVADOR	292740	MATERNID
08/11/202	45	28/10/202	43	SC	FLORIANOI	1476	FLORIANOI	420540	HOSPITAL U
24/08/202	34	12/08/202	32	SP	GVE XXII PI	1573	DRACENA	351440	SANTA CAS
22/02/202	8	10/02/202	6	CE	1 CRES FOF	1519	FORTALEZA	230440	HOSPITAL (
18/02/202	7	05/02/202	5	SC	JOINVILLE	1565	JOINVILLE	420910	HOSPITAL I
26/04/202	17	14/04/202	15	PR	17RS LOND	1371	LONDRINA	411370	HOSPITAL I
05/05/202	18	15/04/202	15	SP	GVE I CAPI	1331	SAO PAULO	355030	HC DA FMU
07/06/202	23	29/05/202	21	SP	GVE I CAPI	1331	SAO PAULO	355030	MATERNID
24/06/202	25	30/03/202	13	RS	002 CRS	1607	PORTO ALE	431490	IRMANDAD
29/07/202	30	24/07/202	29	SP	GVE XXIX S	1354	SAO JOSE D	354980	SANTA CAS
17/05/202	20	05/05/202	18	RJ			CAMPOS D	330100	HOSPITAL I
14/09/202	37	03/09/202	35	DF			BRASILIA	530010	HRSM
25/06/202	25	13/03/202	10	PA	01 REGION	1484	BELEM	150140	SANTA CAS
19/06/202	24	31/05/202	22	AL	1 MICRORI	1533	MACEIO	270430	SANTA CAS
10/02/202	6	31/01/202	5	RS	005 CRS	1608	CAXIAS DO	430510	HOSPITAL I
11/02/202	6	07/02/202	6	AM	ENTORNO	5584	MANAUS	130260	MATERNID
26/03/202	12	10/03/202	10	SP	GVE XIII AS	1338	ASSIS	350400	SANTA CAS
25/03/202	12	15/03/202	11	BA	NUCLEO RE	1380	SALVADOR	292740	MATERNID
20/04/202	16	09/04/202	14	GO	CENTRAL	1779	GOIANIA	520870	HOSPITAL I
08/04/202	14	27/03/202	12	MT	CUIABA	1578	CUIABA	510340	HOSPITAL U
20/04/202	16	27/03/202	12	PR	03RS PONT	1357	JAGUARIAI	411200	HOSPITAL I
26/04/202	17	16/04/202	15	SP	GVE XVII CA	1342	JUNDIAI	352590	HU HOSPIT
18/05/202	20	08/05/202	18	BA	NUCLEO RE	1380	SALVADOR	292740	MATERNID
14/03/202	11	03/03/202	9	RJ			RIO DE JAN	330455	CLINICA SA
15/03/202	11	09/03/202	10	SP	GVE XVII CA	1342	CAMPINAS	350950	HOSPITAL I
12/07/202	28	22/06/202	25	SP	GVE I CAPI	1331	SAO PAULO	355030	SAO LUIZ U
20/06/202	25	10/06/202	23	SP	GVE XVII CA	1342	CAMPINAS	350950	HOSPITAL I
14/06/202	24	03/06/202	22	MG	PATOS DE I	1456	PATOS DE I	314800	HOSPITAL I
06/05/202	18	28/04/202	17	BA	NUCLEO RE	1380	SALVADOR	292740	MATERNID
05/05/202	18	25/04/202	17	DF			BRASILIA	530010	HOSPITAL S
01/04/202	13	19/03/202	11	MG	SETE LAGO	1467	SETE LAGO	316720	HOSPITAL I
13/09/202	37	28/08/202	34	RJ			RIO DE JAN	330455	MS HSE HC
28/03/202	13	19/03/202	11	RJ			RIO DE JAN	330455	MS HSE HC
20/04/202	16	06/04/202	14	SP	GVE VIII M	1333	MOGI DAS	353060	MOGI MAT
16/02/202	7	06/02/202	5	SP	GVE I CAPI	1331	SAO PAULO	355030	HC DA FMU
05/04/202	14	28/03/202	13	SP	GVE XXIX S	1354	SAO JOSE D	354980	HOSPITAL I
29/04/202	17	28/04/202	17	SP	GVE XXVIII	1576	SAO SEBAS	355070	HOSPITAL I
21/04/202	16	14/04/202	15	MA	REGIONAL	1440	IMPERATRI	210530	HRMI HOSI
10/09/202	36	24/08/202	34	RJ			RIO DE JAN	330455	HOSPITAL I
12/07/202	28	01/07/202	26	RJ			CAMPOS D	330100	SOCIEDADE
16/08/202	33	05/08/202	31	SP	GVE I CAPI	1331	SAO PAULO	355030	HOSPITAL I
12/05/202	19	29/04/202	17	RN	II URSAP	1412	APODI	240100	HOSPITAL I
18/11/202	46	12/11/202	45	SP	GVE XVII CA	1342	CAMPINAS	350950	HOSPITAL V
26/11/202	47	21/11/202	47	PA	09 REGION	1492	SANTAREM	150680	UNIDADE D
30/03/202	13	18/03/202	11	RJ			RIO DE JAN	330455	HOSPITAL I

31/08/202	35	20/03/202	11	RJ			RIO DE JAN	330455	HOSPITAL (
25/02/202	8	24/02/202	8	SP	GVE I CAPI	1331	SAO PAULC	355030	HOSP MUN
17/05/202	20	04/05/202	18	SP	GVE I CAPI	1331	SAO PAULC	355030	HOSPITAL I
07/04/202	14	06/04/202	14	SP	GVE XXV SA	1349	ITANHAEM	352210	UNIDADE D
01/05/202	17	24/04/202	16	ES	METROPOI	1510	SERRA	320500	HOSPITAL I
09/08/202	32	29/07/202	30	SP	GVE I CAPI	1331	SAO PAULC	355030	HOSP MUN
22/09/202	38	25/04/202	17	PE	001	1497	RECIFE	261160	HOSPITAL I
26/05/202	21	17/05/202	20	RJ			RIO DE JAN	330455	MS HSE HC
08/11/202	45	29/09/202	39	SP	GVE I CAPI	1331	SAO PAULC	355030	HC DA FMU
04/08/202	31	23/07/202	29	GO	CENTRAL	1779	GOIANIA	520870	HOSPITAL I
27/10/202	43	18/10/202	42	DF			BRASILIA	530010	HOSPITAL I
15/06/202	24	02/06/202	22	SP	GVE XXXI S	1353	ITU	352390	VIGILANCIA
25/08/202	34	09/08/202	32	SP	GVE I CAPI	1331	SAO PAULC	355030	MATERNID
17/03/202	11	16/03/202	11	SP	GVE XVII CA	1342	CAMPINAS	350950	HOSPITAL I
12/05/202	19	25/04/202	17	MG	UBERLAND	1462	UBERLAND	317020	HOSPITAL S
19/04/202	16	07/04/202	14	GO	CENTRAL	1779	GOIANIA	520870	HOSPITAL I
16/03/202	11	11/03/202	10	SP	GVE XXIII R	1347	REGISTRO	354260	HOSPITAL S
28/05/202	21	21/05/202	20	MG	DIVINOPOI	1472	PITANGUI	315140	SANTA CAS
05/07/202	27	03/06/202	22	SP	GVE I CAPI	1331	SAO PAULC	355030	HC DA FMU
11/01/202	2	03/01/202	1	GO	CENTRAL	1779	GOIANIA	520870	HOSPITAL I
12/02/202	6	02/02/202	5	SC	JARAGUA D	1567	JARAGUA D	420890	HOSPITAL J
27/03/202	12	14/03/202	11	SC	BLUMENAU	1564	BRUSQUE	420290	HOSPITAL A
22/03/202	12	08/03/202	10	AC	REGIONAL	1938	RIO BRANC	120040	HOSPITAL (
24/03/202	12	18/03/202	11	SP	GVE XVII CA	1342	CAMPINAS	350950	MATERNID
29/03/202	13	15/03/202	11	RS	002 CRS	1607	PORTO ALE	431490	HOSPITAL I
28/03/202	13	18/03/202	11	RJ			CONCEICA(	330140	HOSPITAL I
22/03/202	12	19/03/202	11	SP	GVE I CAPI	1331	SAO PAULC	355030	BP HOSPITA
06/05/202	18	19/04/202	16	SP	GVE I CAPI	1331	SAO PAULC	355030	HC DA FMU
13/04/202	15	09/04/202	14	SP	GVE XIX MA	1344	MARILIA	352900	HOSPITAL U
11/06/202	23	01/06/202	22	GO	SUDOESTE	1800	JATAI	521190	HOSPITAL I
24/05/202	21	18/05/202	20	BA	NUCLEO RE	1380	SALVADOR	292740	MATERNID
18/06/202	24	04/06/202	22	SP	GVE XXIX S	1354	VOTUPORA	355710	SANTA CAS
19/06/202	24	12/06/202	23	GO	PIRINEUS	1792	ANAPOLIS	520110	SANTA CAS
11/06/202	23	07/06/202	23	PA	01 REGION	1484	BELEM	150140	SANTA CAS
03/07/202	26	23/06/202	25	MG	DIVINOPOI	1472	BOM DESP	310740	HOSP SANT
28/02/202	9	14/02/202	7	SC	CHAPECO	1553	CHAPECO	420420	HOSPITAL I
20/04/202	16	08/04/202	14	SP	GVE XXIV R	1348	RIBEIRAO P	354340	HOSPITAL I
16/04/202	15	02/04/202	13	SP	GVE I CAPI	1331	SAO PAULC	355030	MATERNID
13/05/202	19	03/05/202	18	SP	GVE X OSA	1335	OSASCO	353440	HOSPITAL I
15/06/202	24	01/06/202	22	SP	GVE X OSA	1335	OSASCO	353440	HOSPITAL S
17/06/202	24	06/06/202	23	DF			BRASILIA	530010	HRAN
25/06/202	25	16/06/202	24	SP	GVE XXI PR	1346	PRESIDENT	354140	HOSPITAL I
07/04/202	14	20/03/202	11	SP	GVE XXV SA	1349	SANTOS	354850	HOSPITAL (
18/04/202	16	08/04/202	14	MG	UBERABA	1461	UBERABA	317010	HOSPITAL S
24/08/202	34	11/07/202	28	GO	CENTRAL	1779	GOIANIA	520870	HOSPITAL I
07/06/202	23	07/06/202	23	SP	GVE VII SAI	1332	SAO BERNA	354870	HOSPITAL I
29/03/202	13	24/03/202	12	PI	4 GRS TERE	1883	TERESINA	221100	MATERNID

07/12/202	49	22/11/202	47	GO	PIRINEUS	1792	ANAPOLIS	520110	SANTA CAS
03/03/202	9	21/02/202	8	PB	I NRS JOAC	1377	JOAO PESS	250750	MATERNID
09/03/202	10	25/02/202	8	RS	002 CRS	1607	PORTO ALE	431490	HOSPITAL I
01/04/202	13	30/01/202	4	PR	17RS LOND	1371	LONDRINA	411370	HOSPITAL U
04/04/202	14	26/03/202	12	SC	CRICIUMA	1558	ICARA	420700	FUNDACAO
18/04/202	16	07/04/202	14	PR	16RS APUC	1370	APUCARAN	410140	HNSG HOS
18/05/202	20	05/05/202	18	PR	05RS GUAF	1359	GUARAPUA	410940	HOSPITAL I
23/05/202	21	14/05/202	19	GO	CENTRAL	1779	GOIANIA	520870	HOSPITAL I
12/05/202	19	04/05/202	18	MT	CUIABA	1578	CUIABA	510340	HOSPITAL U
11/06/202	23	31/05/202	22	SP	GVE I CAPI	1331	SAO PAULO	355030	MATERNID
02/07/202	26	28/06/202	26	TO			PALMAS	172100	HOSPITAL G
09/08/202	32	04/08/202	31	PR	02RS METF	1356	CURITIBA	410690	COMPLEXO
25/08/202	34	15/08/202	33	RS	005 CRS	1608	CAXIAS DO	430510	HOSPITAL U
18/08/202	33	12/08/202	32	SP	GVE XXXI S	1353	PORTO FEL	354060	SANTA CAS
28/01/202	4	22/01/202	3	SP	GVE XXVII S	1351	SAO JOSE D	354990	HOSPITAL V
05/02/202	5	25/01/202	4	AM	ENTORNO	5584	MANAUS	130260	MATERNID
25/01/202	4	15/01/202	2	SP	GVE VII SAI	1332	SAO CAETA	354880	HOSPITAL S
20/07/202	29	08/07/202	27	DF			BRASILIA	530010	HRAN
07/02/202	6	03/02/202	5	PA	01 REGION	1484	BELEM	150140	SANTA CAS
25/03/202	12	22/03/202	12	SP	GVE XVII CA	1342	JUNDIAI	352590	HOSPITAL I
13/04/202	15	28/03/202	13	RS	001 CRS	1592	CANOAS	430460	DIRETORIA
31/03/202	13	21/03/202	12	SC	FLORIANOI	1476	SAO JOSE	421660	HOSPITAL I
24/06/202	25	12/06/202	23	SP	GVE I CAPI	1331	SAO PAULO	355030	HC DA FMU
02/04/202	13	28/03/202	13	SP	GVE XIII AS	1338	ASSIS	350400	UPA RUY S
07/06/202	23	28/05/202	21	MG	BELO HORI	1449	BELO HORI	310620	MATERNID
31/03/202	13	23/03/202	12	MG	LEOPOLDIN	1453	LEOPOLDIN	313840	CASA DE CA
05/08/202	31	27/07/202	30	PR	02RS METF	1356	CAMPINA G	410400	HOSPITAL A
31/05/202	22	15/05/202	19	GO	CENTRAL	1779	GOIANIA	520870	HOSPITAL I
04/01/202	1	01/12/202	48	RS	001 CRS	1592	IGREJINHA	431010	HOSPITAL I
30/11/202	48	01/11/202	44	RJ			RIO DE JAN	330455	MS HSE HC
12/03/202	10	06/03/202	9	MG	BELO HORI	1449	BELO HORI	310620	BH HOSPIT
01/04/202	13	25/03/202	12	SP	GVE XVII CA	1342	CAMPINAS	350950	HOSPITAL S
17/04/202	15	04/04/202	14	MS	EX ARS DE	1975	CAMPO GR	500270	HOSPITAL I
18/04/202	16	07/04/202	14	RS	005 CRS	1608	CAXIAS DO	430510	HOSPITAL U
17/05/202	20	02/05/202	18	RJ			RIO DE JAN	330455	SMS HOSPI
06/06/202	23	27/05/202	21	MG	PIRAPORA	1469	PIRAPORA	315120	HOSPITAL I
22/03/202	12	08/03/202	10	SP	GVE XII ARA	1337	MATAO	352930	HOSPITAL C
01/06/202	22	18/05/202	20	RS	002 CRS	1607	PORTO ALE	431490	HOSPITAL I
23/06/202	25	27/05/202	21	SP	GVE I CAPI	1331	SAO PAULO	355030	HOSP MUN
16/07/202	28	07/07/202	27	GO	CENTRAL	1779	GOIANIA	520870	HOSPITAL I
04/08/202	31	25/07/202	30	DF			BRASILIA	530010	HRAN
30/08/202	35	30/08/202	35	PR	17RS LOND	1371	LONDRINA	411370	HOSPITAL I
21/05/202	20	14/05/202	19	MG	PASSOS	1455	PIUMHI	315150	SANTA CAS
10/02/202	6	05/02/202	5	SP	GVE XVII CA	1342	INDAIATUE	352050	HOSPITAL S
10/02/202	6	29/01/202	4	GO	CENTRAL	1779	GOIANIA	520870	HOSPITAL I
19/03/202	11	12/03/202	10	PA	01 REGION	1484	BELEM	150140	SANTA CAS
23/02/202	8	14/02/202	7	MT	CUIABA	1578	CUIABA	510340	HOSPITAL U

06/04/202	14	25/03/202	12	RS	004 CRS	1595	SANTA MA	431690	HUSM HOS
17/04/202	15	15/04/202	15	SP	GVE XVII C/	1342	JUNDIAI	352590	HU HOSPIT
31/03/202	13	16/03/202	11	TO			PALMAS	172100	HOSPITAL (
25/04/202	17	12/04/202	15	MG	SAO JOAO	1460	SAO JOAO	316250	SANTA CAS
03/05/202	18	20/04/202	16	MG	JUIZ DE FO	1452	JUIZ DE FO	313670	HOSPITAL U
09/05/202	19	26/04/202	17	PR	17RS LOND	1371	LONDRINA	411370	HOSPITAL U
10/05/202	19	28/04/202	17	MG	DIVINOPOL	1472	DIVINOPOL	312230	HOSPITAL S
21/05/202	20	11/05/202	19	SP	GVE XXIV R	1348	RIBEIRAO P	354340	HOSPITAL S
29/03/202	13	17/03/202	11	SP	GVE XVII C/	1342	CAMPINAS	350950	HOSPITAL I
17/06/202	24	07/06/202	23	RJ			RIO DE JAN	330455	HOSPITAL (
13/07/202	28	02/07/202	26	DF			BRASILIA	530010	HRAN
11/05/202	19	01/05/202	17	SP	GVE I CAPIT	1331	SAO PAULO	355030	HC DA FMU
20/03/202	11	11/03/202	10	ES	COLATINA	1509	LINHARES	320320	HOSPITAL I
08/06/202	23	02/06/202	22	RN	VII URSAP	1416	NATAL	240810	HOSPITAL I
03/03/202	9	27/02/202	8	BA	NUCLEO RI	1381	FEIRA DE S/	291080	HOSPITAL I
28/03/202	13	11/02/202	6	MA	REGI0NAL	1430	SAO LUIS	211130	MATERNID
10/03/202	10	20/02/202	7	SP	GVE XVII C/	1342	CAMPINAS	350950	HOSPITAL I
21/05/202	20	17/05/202	20	RJ			MARICA	330270	HOSPITAL I
02/04/202	13	17/03/202	11	SP	GVE VII SAI	1332	SANTO AN	354780	HOSPITAL I
11/03/202	10	02/03/202	9	MG	MONTES C	1473	MONTES C	314330	HOSPITAL U
06/05/202	18	29/04/202	17	PE	001	1497	RECIFE	261160	IMIP
15/05/202	19	07/05/202	18	PR	05RS GUAF	1359	GUARAPUA	410940	HOSPITAL I
18/05/202	20	14/05/202	19	PR	02RS METF	1356	CAMPINA (	410400	HOSPITAL A
13/05/202	19	10/03/202	10	SP	GVE X OSA	1335	BARUERI	350570	PS E MATE
30/04/202	17	20/04/202	16	PA	01 REGION	1484	BELEM	150140	SANTA CAS
25/02/202	8	16/02/202	7	SP	GVE XXXI S	1353	CERQUILH(	351150	SANTA CAS
29/06/202	26	21/06/202	25	SP	GVE XIX M/	1344	MARILIA	352900	HOSPITAL I
19/07/202	29	15/07/202	28	SP	GVE XXIV R	1348	RIBEIRAO P	354340	HOSPITAL I
16/08/202	33	04/08/202	31	PR	02RS METF	1356	CURITIBA	410690	COMPLEXO
22/03/202	12	05/03/202	9	PR	02RS METF	1356	CURITIBA	410690	COMPLEXO
01/04/202	13	29/03/202	13	SP	GVE XII AR/	1337	SAO CARLO	354890	SANTA CAS
09/03/202	10	04/03/202	9	RJ			RIO DE JAN	330455	MS HSE HO
17/03/202	11	05/03/202	9	MT	CUIABA	1578	CUIABA	510340	HOSPITAL I
02/05/202	18	27/04/202	17	SP	GVE XVII C/	1342	CAMPINAS	350950	COMPLEXO
12/08/202	32	16/07/202	28	RJ			NITEROI	330330	HOSPITAL I
07/05/202	18	26/04/202	17	RO	VI GRS POF	1483	PORTO VEL	110020	HOSPITAL I
18/05/202	20	18/05/202	20	CE	1 CRES FOF	1519	FORTALEZ/	230440	HGCC HOS
30/12/202	52	15/12/202	50	MS	NRS DE DO	1966	DOURADO:	500370	EBSERH HO
18/01/202	3	05/01/202	1	SC	RIO DO SUI	1478	RIO DO SUI	421480	VIGILANCI/
13/10/202	41	04/07/202	27	SP	GVE X OSA	1335	OSASCO	353440	HOSPITAL I
11/03/202	10	26/02/202	8	SP	GVE XVII C/	1342	CAMPINAS	350950	MATERNID
30/07/202	30	14/07/202	28	MT	CUIABA	1578	CUIABA	510340	HOSPITAL U
09/02/202	6	03/02/202	5	PR	02RS METF	1356	CAMPO LA	410420	HOSPITAL I
29/03/202	13	22/03/202	12	RS	012 CRS	1601	SAO BORJA	431800	HOSPITAL I
15/03/202	11	03/03/202	9	MS	NRS DE TRI	6255	TRES LAGO	500830	HOSPITAL I
09/04/202	14	05/04/202	14	MS	EX NRS DE	6256	CORUMBA	500320	SANTA CAS
24/03/202	12	13/03/202	10	PE	001	1497	RECIFE	261160	IMIP

13/05/202	19	02/05/202	18	SP	GVE XVI BC	1341	BOTUCATU	350750	HOSPITAL I
28/04/202	17	16/04/202	15	SP	GVE XX PIR	1345	PIRASSUNU	353930	SANTA CAS
22/06/202	25	15/06/202	24	GO	SERRA DA I	1790	URUACU	522160	UPA UNIDA
01/06/202	22	24/05/202	21	RJ			RIO DE JAN	330455	MS HSE HC
03/07/202	26	26/06/202	25	RJ			CAMBUCI	330090	HOSPITAL I
08/07/202	27	05/07/202	27	MA	REGI0NAL	1430	SAO LUIS	211130	MATERNID
20/09/202	38	10/09/202	36	PR	09RS FOZ D	1363	FOZ DO IGU	410830	HOSPITAL I
05/10/202	40	29/09/202	39	PA	09 REGION	1492	SANTAREM	150680	HOSPITAL I
22/08/202	34	14/08/202	32	RN	VII URSAP	1416	NATAL	240810	HOSPITAL I
22/10/202	42	03/10/202	40	GO	CENTRAL	1779	GOIANIA	520870	HOSPITAL I
20/01/202	3	11/01/202	2	SP	GVE XXVIII	1576	CARAGUAT	351050	CASA DE SA
09/03/202	10	24/02/202	8	AL	1 MICRORI	1533	MACEIO	270430	HOSPITAL I
10/03/202	10	06/03/202	9	MA	REGIONAL	1435	CURURUPU	210370	SANTA CAS
08/02/202	6	06/02/202	5	SP	GVE XXV SA	1349	GUARUJA	351870	HOSPITAL S
17/05/202	20	03/04/202	13	MG	JUIZ DE FO	1452	JUIZ DE FO	313670	HOSPITAL I
17/04/202	15	10/04/202	14	BA	NUCLEO RI	1380	SALVADOR	292740	HOSPITAL T
30/04/202	17	17/04/202	15	SP	GVE I CAPI	1331	SAO PAULO	355030	HC DA FMU
17/04/202	15	05/03/202	9	PA	01 REGION	1484	BELEM	150140	HOSPITAL I
21/05/202	20	06/05/202	18	SP	GVE I CAPI	1331	SAO PAULO	355030	HC DA FMU
22/04/202	16	10/04/202	14	SP	GVE XXIV R	1348	RIBEIRAO P	354340	HOSPITAL S
15/05/202	19	05/05/202	18	MG	PONTE NO	1458	PONTE NO	315210	HOSPITAL I
26/03/202	12	16/03/202	11	MG	DIVINOPOI	1472	LAGOA DA	313720	HOSPITAL S
29/05/202	21	25/05/202	21	MS	EX ARS DE	1975	CAMPO GR	500270	EBSERH HC
21/04/202	16	21/04/202	16	CE	1 CRES FOI	1519	FORTALEZA	230440	HGCC HOS
15/07/202	28	29/06/202	26	SP	GVE XXIX S	1354	SAO JOSE D	354980	HOSPITAL I
21/06/202	25	05/06/202	22	GO	CENTRAL	1779	GOIANIA	520870	HOSPITAL I
25/02/202	8	25/02/202	8	CE	1 CRES FOI	1519	FORTALEZA	230440	HGCC HOS
26/05/202	21	23/05/202	21	SE	REGIONAL	2056	ARACAJU	280030	HOSPITAL (
04/06/202	22	30/05/202	22	SP	GVE XV BA	1340	BAURU	350600	HOSPITAL I
01/02/202	5	24/01/202	4	PR	02RS METF	1356	CURITIBA	410690	COMPLEXC
25/02/202	8	07/02/202	6	DF			BRASILIA	530010	HRAN
16/08/202	33	04/08/202	31	SP	GVE I CAPI	1331	SAO PAULO	355030	PRO MATR
12/08/202	32	07/08/202	31	RR			BOA VISTA	140010	HOSPITAL (
26/04/202	17	20/04/202	16	SP	GVE XVI BC	1341	BOTUCATU	350750	HOSPITAL I
29/04/202	17	12/04/202	15	RO	III GRS VILH	1479	VILHENA	110030	HOSPITAL I
12/04/202	15	04/04/202	14	GO	CENTRAL	1779	GOIANIA	520870	HOSPITAL I
22/04/202	16	17/04/202	15	MA	REGIONAL	1440	IMPERATRI	210530	HRMI HOSI
14/06/202	24	04/06/202	22	SP	GVE I CAPI	1331	SAO PAULO	355030	HC DA FMU
18/03/202	11	08/03/202	10	RS	002 CRS	1607	PORTO ALE	431490	HOSPITAL I
15/05/202	19	15/05/202	19	SP	GVE XXVI S	1350	MOGI GUA	353070	SANTA CAS
17/05/202	20	01/05/202	17	RR			BOA VISTA	140010	HOSPITAL (
28/05/202	21	14/05/202	19	SP	GVE VIII M	1333	GUARULHO	351880	HOSPITAL I
13/06/202	24	13/06/202	24	PR	17RS LOND	1371	LONDRINA	411370	HOSPITAL I
01/09/202	35	26/08/202	34	RJ			CAMPOS D	330100	HOSPITAL I
04/11/202	44	23/10/202	42	PR	15RS MARI	1369	MARINGA	411520	SANTA CAS
26/05/202	21	22/05/202	20	MT	SINOP	1584	NOVA MUT	510622	HOSPITAL I
15/06/202	24	18/05/202	20	SP	GVE VIII M	1333	SUZANO	355250	HOSPITAL S

16/07/202	28	12/07/202	28	MT	RONDONO	1579	PRIMAVER	510704	SECRETARI
04/03/202	9	27/02/202	8	SP	GVE XVII C/	1342	CAMPINAS	350950	HOSPITAL \
02/03/202	9	21/02/202	8	SP	GVE I CAPI	1331	SAO PAUL(	355030	HC DA FMU
23/04/202	16	11/04/202	15	SP	GVE I CAPI	1331	SAO PAUL(	355030	HC DA FMU
30/03/202	13	11/03/202	10	MG	PONTE NO	1458	VICOSA	317130	HOSPITAL S
01/06/202	22	24/05/202	21	SC	FLORIANOI	1476	FLORIANOI	420540	HOSPITAL U
18/06/202	24	06/06/202	23	SP	GVE XXVI S	1350	MOGI GUA	353070	SANTA CAS
24/06/202	25	13/06/202	24	SP	GVE X OSA	1335	OSASCO	353440	HOSPITAL I
31/05/202	22	27/05/202	21	RS	010 CRS	1599	URUGUAIA	432240	SANTA CAS
27/05/202	21	10/05/202	19	SP	GVE I CAPI	1331	SAO PAUL(	355030	MATERNID
11/08/202	32	01/08/202	31	PR	02RS METF	1356	CURITIBA	410690	HOSPITAL U
12/07/202	28	03/07/202	26	RJ			RIO DE JAN	330455	MS HSE HC
11/08/202	32	22/07/202	29	MG	VARGINHA	1466	VARGINHA	317070	HOSPITAL I
18/08/202	33	12/08/202	32	MG	PATOS DE I	1456	PATOS DE I	314800	HOSPITAL I
11/03/202	10	26/02/202	8	MT	CUIABA	1578	CUIABA	510340	HOSPITAL U
29/04/202	17	22/04/202	16	DF			BRASILIA	530010	HRSM
21/03/202	12	15/03/202	11	RJ			RIO DE JAN	330455	SMS COOR
17/06/202	24	21/05/202	20	GO	CENTRAL	1779	GOIANIA	520870	HOSPITAL I
26/06/202	25	18/06/202	24	SP	GVE X OSA	1335	BARUERI	350570	PS E MATE
17/06/202	24	08/06/202	23	PR	02RS METF	1356	CAMPO LA	410420	HOSPITAL I
24/06/202	25	08/06/202	23	SP	GVE I CAPI	1331	SAO PAUL(	355030	HC DA FMU
21/06/202	25	13/06/202	24	SP	GVE XVII C/	1342	CAMPINAS	350950	MATERNID
27/06/202	26	18/06/202	24	SP	GVE XXXI S	1353	SOROCABA	355220	CONJUNTC
20/07/202	29	01/07/202	26	SP	GVE I CAPI	1331	SAO PAUL(	355030	SAO LUIZ U
01/12/202	48	26/11/202	47	PR	09RS FOZ D	1363	FOZ DO IGU	410830	HOSPITAL I
04/10/202	40	18/03/202	11	BA	NUCLEO RI	1380	SALVADOR	292740	HOSPITAL T
06/12/202	49	21/11/202	47	BA	NUCLEO RI	1380	SALVADOR	292740	MATERNID
03/08/202	31	20/07/202	29	BA	NUCLEO RI	1403	BARREIRAS	290320	HOSPITAL I
24/02/202	8	18/02/202	7	RJ			RIO DE JAN	330455	MS HSE HC
25/03/202	12	22/03/202	12	RJ			PETROPOLI	330390	HOSPITAL /
22/04/202	16	26/03/202	12	SP	GVE I CAPI	1331	SAO PAUL(	355030	HC DA FMU
29/03/202	13	15/03/202	11	SP	GVE I CAPI	1331	SAO PAUL(	355030	MATERNID
20/05/202	20	05/05/202	18	MA	REGI0NAL	1430	SAO LUIS	211130	MATERNID
22/07/202	29	13/07/202	28	MS	NRS DE TRI	6255	TRES LAGO	500830	HOSPITAL I
23/07/202	29	13/06/202	24	PR	16RS APUC	1370	APUCARAN	410140	HNSG HOS
31/05/202	22	23/05/202	21	SP	GVE XXIX S	1354	SAO JOSE D	354980	HOSPITAL /
21/04/202	16	13/04/202	15	RN	VII URSAP	1416	NATAL	240810	HOSPITAL I
14/06/202	24	01/06/202	22	GO	CENTRAL	1779	GOIANIA	520870	HOSPITAL I
30/03/202	13	20/03/202	11	PR	10RS CASC	1364	CASCAVEL	410480	HOSPITAL I
17/03/202	11	12/03/202	10	CE	1 CRES FOF	1519	FORTALEZ/	230440	HGCC HOS
04/02/202	5	28/01/202	4	RO	VI GRS POF	1483	PORTO VEL	110020	HOSPITAL I
27/04/202	17	15/01/202	2	MA	REGI0NAL	1430	SAO LUIS	211130	MATERNID
05/06/202	22	28/05/202	21	SP	GVE XXXI S	1353	SOROCABA	355220	HOSPITAL U
03/05/202	18	30/04/202	17	PR	01RS PAR/	1355	PARANAGU	411820	HOSPITAL I
26/05/202	21	21/05/202	20	SP	GVE I CAPI	1331	SAO PAUL(	355030	SANTA CAS
14/05/202	19	09/05/202	19	SC	JOINVILLE	1565	JOINVILLE	420910	HOSPITAL I
08/02/202	6	02/02/202	5	SP	GVE VIII M	1333	MOGI DAS	353060	MOGI MAT

17/04/202	15	24/03/202	12	ES	METROPOI	1510	SERRA	320500	HOSPITAL I
24/05/202	21	19/05/202	20	MG	UBA	1464	VISCONDE	317200	HOSPITAL S
12/05/202	19	28/04/202	17	SP	GVE I CAPI	1331	SAO PAULC	355030	HC DA FMU
26/03/202	12	18/03/202	11	SP	GVE VIII M	1333	GUARULHC	351880	HOSPITAL I
07/04/202	14	04/04/202	14	MG	UBERLAND	1462	COROMAN	311930	PRONTO S(
20/07/202	29	08/07/202	27	BA	NUCLEO RI	1388	PORTO SEG	292530	HOSPITAL I
23/02/202	8	06/02/202	5	SP	GVE I CAPI	1331	SAO PAULC	355030	MATERNID
25/04/202	17	23/04/202	16	SP	GVE I CAPI	1331	SAO PAULC	355030	HOSP MUN
10/05/202	19	29/04/202	17	RS	014 CRS	1602	SANTA ROS	431720	HOSPITAL V
23/01/202	3	15/01/202	2	GO	CENTRAL	1779	GOIANIA	520870	HOSPITAL I
25/02/202	8	18/02/202	7	GO	CENTRAL	1779	GOIANIA	520870	HOSPITAL I
17/03/202	11	07/03/202	10	SP	GVE I CAPI	1331	SAO PAULC	355030	HC DA FMU
04/03/202	9	19/02/202	7	MT	CUIABA	1578	CUIABA	510340	HOSPITAL I
09/04/202	14	31/03/202	13	SP	GVE X OSA	1335	CARAPICUI	351060	HOSPITAL (
08/07/202	27	01/07/202	26	RJ			NILOPOLIS	330320	DOMINGO.
05/05/202	18	03/05/202	18	PA	01 REGION	1484	BELEM	150140	SANTA CAS
12/04/202	15	27/03/202	12	SP	GVE I CAPI	1331	SAO PAULC	355030	HC DA FMU
16/05/202	20	06/05/202	18	SP	GVE XXVII S	1351	SAO JOSE D	354990	HOSPITAL I
05/01/202	1	31/12/202	52	SP	GVE XXXI S	1353	ITU	352390	SANTA CAS
19/04/202	16	10/04/202	14	SC	CONCORDI	1560	CONCORDI	420430	HOSPITAL S
04/06/202	22	02/02/202	5	RO	I GRS JI PAI	1474	OURO PRET	110015	HOSPITAL I
22/03/202	12	11/03/202	10	MG	BELO HORI	1449	BETIM	310670	HOSPITAL I
30/05/202	22	20/05/202	20	GO	CENTRAL	1779	GOIANIA	520870	HOSPITAL (
29/06/202	26	17/06/202	24	MG	DIVINOPOI	1472	DIVINOPOI	312230	HOSPITAL S
09/03/202	10	28/02/202	9	GO	CENTRAL	1779	GOIANIA	520870	HOSPITAL I
18/08/202	33	12/08/202	32	SP	GVE XXVII S	1351	SAO JOSE D	354990	HOSPITAL V
17/07/202	28	08/07/202	27	MG	GOVERNAI	1471	GOVERNAI	312770	HOSPITAL I
02/07/202	26	18/06/202	24	RS	002 CRS	1607	PORTO ALE	431490	HOSPITAL I
04/08/202	31	25/07/202	30	PR	10RS CASC	1364	CASCAVEL	410480	UPA VENEZ
06/08/202	31	01/08/202	31	SP	GVE VII SAI	1332	SAO BERNA	354870	HOSPITAL I
10/01/202	2	26/12/202	52	RJ			RIO DE JAN	330455	MS HSE HC
29/03/202	13	20/03/202	11	SP	GVE XVII C.	1342	CAMPINAS	350950	HOSPITAL I
23/09/202	38	19/09/202	38	RS	005 CRS	1608	CAXIAS DO	430510	HOSPITAL (
19/02/202	7	11/02/202	6	GO	CENTRAL	1779	GOIANIA	520870	HOSPITAL I
06/04/202	14	07/03/202	10	MA	REGI0NAL	1430	SAO LUIS	211130	MATERNID
29/05/202	21	20/05/202	20	MG	JUIZ DE FO	1452	JUIZ DE FO	313670	SANTA CAS
20/05/202	20	10/05/202	19	SC	RIO DO SUI	1478	IBIRAMA	420690	HOSPITAL I
31/05/202	22	25/05/202	21	PR	17RS LONE	1371	LONDRINA	411370	HOSPITAL U
07/05/202	18	04/05/202	18	PR	13RS CIAN(	1367	CIANORTE	410550	HOSPITAL S
18/05/202	20	10/05/202	19	GO	CENTRAL	1779	GOIANIA	520870	HOSPITAL I
06/08/202	31	11/07/202	28	RJ			DUQUE DE	330170	HOSPITAL I
02/07/202	26	18/06/202	24	SP	GVE IX FRA	1334	FRANCISCC	351630	HOSPITAL I
01/06/202	22	20/05/202	20	SP	GVE XXVI S	1350	ITAPIRA	352260	HOSPITAL I
17/12/202	50	09/12/202	49	PR	02RS METF	1356	CURITIBA	410690	COMPLEXC
05/07/202	27	01/07/202	26	SP	GVE XXVIII	1576	CARAGUAT	351050	CASA DE S.
10/03/202	10	26/02/202	8	RS	017 CRS	1604	IJUI	431020	HOSPITAL U
13/08/202	32	06/08/202	31	MT	CUIABA	1578	CUIABA	510340	HOSPITAL U

08/03/202	10	03/03/202	9	RJ			RIO DE JAN	330455	HOSPITAL
10/06/202	23	04/06/202	22	MA	REGI0NAL	1430	SAO LUIS	211130	EBSERH HC
24/06/202	25	17/06/202	24	PR	02RS METR	1356	CURITIBA	410690	COMPLEXO
04/07/202	27	30/06/202	26	PR	15RS MARI	1369	MARINGA	411520	SANTA CAS
10/04/202	14	06/04/202	14	RS	002 CRS	1607	PORTO ALE	431490	HOSPITAL
05/07/202	27	18/06/202	24	BA	NUCLEO RE	1381	FEIRA DE S	291080	HOSPITAL
02/09/202	35	23/08/202	34	SC	JOINVILLE	1565	JOINVILLE	420910	HOSPITAL
19/05/202	20	03/05/202	18	BA	NUCLEO RE	1381	FEIRA DE S	291080	HOSPITAL
05/11/202	44	01/11/202	44	RR			BOA VISTA	140010	HOSPITAL
26/04/202	17	18/04/202	16	SP	GVE I CAPI	1331	SAO PAUL	355030	HOSP MUN
09/12/202	49	02/12/202	48	PA	01 REGION	1484	BELEM	150140	SANTA CAS
12/04/202	15	01/04/202	13	SP	GVE XVII C	1342	CAMPINAS	350950	MATERNID
10/03/202	10	10/03/202	10	CE	1 CRES FOR	1519	FORTALEZA	230440	HGCC HOS
05/05/202	18	20/04/202	16	RJ			RIO DE JAN	330455	MS HSE HC
26/05/202	21	17/05/202	20	SP	GVE XXIV R	1348	RIBEIRAO P	354340	HOSPITAL
04/08/202	31	31/05/202	22	PR	09RS FOZ D	1363	FOZ DO IGU	410830	HOSPITAL
07/01/202	1	27/12/202	52	MA	REGIONAL	1440	IMPERATRI	210530	HRMI HOSI
09/08/202	32	01/08/202	31	PR	17RS LOND	1371	LONDRINA	411370	HOSPITAL
03/01/202	1	28/12/202	52	BA	NUCLEO RE	1398	VITORIA DA	293330	SAMUR
09/04/202	14	02/04/202	13	RJ			VOLTA RED	330630	SECRETARI
17/04/202	15	08/04/202	14	SP	GVE I CAPI	1331	SAO PAUL	355030	HOSPITAL
23/04/202	16	16/03/202	11	SP	GVE VIII M	1333	MOGI DAS	353060	MOGI MAT
15/07/202	28	25/06/202	25	SP	GVE I CAPI	1331	SAO PAUL	355030	HC DA FMU
25/01/202	4	16/01/202	2	BA	NUCLEO RE	1380	SALVADOR	292740	MATERNID
27/04/202	17	08/04/202	14	SP	GVE XXXI S	1353	SOROCABA	355220	CONJUNTO
03/03/202	9	01/03/202	9	MG	PONTE NO	1458	PONTE NO	315210	HOSPITAL
10/03/202	10	21/02/202	8	SP	GVE I CAPI	1331	SAO PAUL	355030	HOSPITAL
24/02/202	8	22/02/202	8	PR	02RS METR	1356	CURITIBA	410690	HOSPITAL
13/03/202	10	07/03/202	10	SP	GVE XXVI S	1350	VARGEM G	355640	CENTRO DI
08/03/202	10	03/03/202	9	SP	GVE XXXI S	1353	SOROCABA	355220	HOSPITAL
04/06/202	22	02/06/202	22	MT	SINOP	1584	NOVA MU	510622	HOSPITAL
09/08/202	32	31/07/202	30	SP	GVE XXVII	1351	JACAREI	352440	HOSPITAL
24/05/202	21	08/05/202	18	AL	9 MICRORE	1541	PALMEIRA	270630	HOSPITAL
20/10/202	42	14/10/202	41	PR	20RS TOLE	1374	TOLEDO	412770	HOESP
11/06/202	23	01/06/202	22	MT	CUIABA	1578	CUIABA	510340	HOSPITAL
27/12/202	52	21/12/202	51	PA	11 REGION	1494	MARABA	150420	CLIMEC
16/08/202	33	13/08/202	32	AM	ENTORNO	5584	MANAUS	130260	MATERNID
30/08/202	35	18/08/202	33	RJ			CAMPOS D	330100	HOSPITAL
17/02/202	7	22/01/202	3	RJ			CAMPOS D	330100	HOSPITAL
08/09/202	36	28/08/202	34	SP	GVE XXXI S	1353	SOROCABA	355220	CONJUNTO
03/02/202	5	23/01/202	3	DF			BRASILIA	530010	HRAN
06/03/202	9	05/03/202	9	AM	ENTORNO	5584	MANAUS	130260	MATERNID
12/04/202	15	15/03/202	11	SP	GVE I CAPI	1331	SAO PAUL	355030	SAO LUIZ U
23/02/202	8	16/02/202	7	DF			BRASILIA	530010	HRSAM HC
23/05/202	21	18/05/202	20	RS	001 CRS	1592	NOVO HAN	431340	FUNDACAO
28/04/202	17	17/04/202	15	RJ			RIO DE JAN	330455	MS HOSPIT
21/05/202	20	18/05/202	20	SP	GVE I CAPI	1331	SAO PAUL	355030	SAO LUIZ U

18/04/202	16	04/04/202	14	SP	GVE XXIX S	1354	SAO JOSE D	354980	HOSPITAL I
09/04/202	14	03/04/202	13	RJ			RIO DE JAN	330455	SMS HOSPI
12/04/202	15	08/04/202	14	RJ			RIO DE JAN	330455	SMS COOR
20/04/202	16	11/04/202	15	BA	NUCLEO RE	1380	SALVADOR	292740	HOSPITAL S
22/02/202	8	22/02/202	8	CE	1 CRES FOR	1519	FORTALEZA	230440	HGCC HOS
19/07/202	29	12/07/202	28	SP	GVE XVII CA	1342	CAMPINAS	350950	HOSPITAL V
23/07/202	29	30/06/202	26	PR	16RS APUC	1370	APUCARAN	410140	HNSG HOS
30/08/202	35	20/08/202	33	SP	GVE XV BA	1340	BAURU	350600	MATERNID
01/04/202	13	10/03/202	10	RJ			RIO DE JAN	330455	SMS HOSPI
10/01/202	2	09/01/202	1	RS	003 CRS	1593	PELOTAS	431440	HOSPITAL I
26/08/202	34	11/08/202	32	GO	CENTRAL	1779	GOIANIA	520870	HOSPITAL I
23/02/202	8	12/02/202	6	RS	002 CRS	1607	PORTO ALE	431490	HOSPITAL I
20/05/202	20	05/05/202	18	SP	GVE VIII M	1333	MOGI DAS	353060	MOGI MAT
22/03/202	12	13/03/202	10	SP	GVE VIII M	1333	GUARULHO	351880	HOSPITAL I
22/04/202	16	15/04/202	15	PA	11 REGION	1494	MARABA	150420	HOSPITAL I
04/01/202	1	04/01/202	1	MG	GOVERNAD	1471	GOVERNAD	312770	HOSPITAL U
16/07/202	28	12/06/202	23	BA	NUCLEO RE	1380	SALVADOR	292740	HOSPITAL J
12/04/202	15	20/03/202	11	PR	15RS MARI	1369	MARINGA	411520	SANTA CAS
23/03/202	12	15/03/202	11	BA	NUCLEO RE	1403	BARREIRAS	290320	HOSPITAL I
13/05/202	19	02/05/202	18	PR	02RS METR	1356	CURITIBA	410690	COMPLEXO
18/08/202	33	11/08/202	32	RS	005 CRS	1608	GRAMADO	430910	HOSPITAL A
08/07/202	27	01/07/202	26	SP	GVE XX PIR	1345	PIRACICAB	353870	SANTA CAS
17/05/202	20	10/05/202	19	SP	GVE XXVI S	1350	MOGI GUA	353070	SANTA CAS
16/08/202	33	03/08/202	31	DF			BRASILIA	530010	HRAN
09/08/202	32	29/07/202	30	PR	02RS METR	1356	CURITIBA	410690	COMPLEXO
18/08/202	33	07/08/202	31	SP	GVE I CAPI	1331	SAO PAULO	355030	PRO MATR
21/05/202	20	17/05/202	20	RJ			MARICA	330270	HOSPITAL I
19/02/202	7	18/02/202	7	RJ			MIGUEL PE	330290	HOSPITAL I
12/03/202	10	26/02/202	8	RS	002 CRS	1607	PORTO ALE	431490	HOSPITAL I
16/03/202	11	26/02/202	8	RS	002 CRS	1607	PORTO ALE	431490	HOSPITAL I
15/06/202	24	05/06/202	22	PR	10RS CASC	1364	CASCAVEL	410480	HOSPITAL U
28/03/202	13	12/03/202	10	CE	1 CRES FOR	1519	FORTALEZA	230440	HGCC HOS
05/07/202	27	29/06/202	26	SC	FLORIANOI	1476	FLORIANOI	420540	HOSPITAL U
28/05/202	21	15/05/202	19	SP	GVE I CAPI	1331	SAO PAULO	355030	MATERNID
25/03/202	12	20/03/202	11	CE	1 CRES FOR	1519	FORTALEZA	230440	HOSPITAL I
13/06/202	24	17/05/202	20	SE	REGIONAL	2056	ARACAJU	280030	HOSPITAL I
26/04/202	17	14/04/202	15	MT	CUIABA	1578	CUIABA	510340	HOSPITAL U
20/09/202	38	14/09/202	37	PR	20RS TOLEI	1374	TOLEDO	412770	HOESP
23/02/202	8	21/02/202	8	RR			BOA VISTA	140010	HOSPITAL (
10/06/202	23	01/06/202	22	SE	REGIONAL	2056	ARACAJU	280030	HOSPITAL (
13/04/202	15	03/04/202	13	BA	NUCLEO RE	1403	BARREIRAS	290320	HOSPITAL I
26/09/202	39	15/09/202	37	PR	15RS MARI	1369	MARINGA	411520	SANTA CAS
08/07/202	27	27/06/202	26	RS	002 CRS	1607	PORTO ALE	431490	HOSPITAL I
24/05/202	21	13/05/202	19	CE	11 CRES SO	1521	SOBRAL	231290	HOSPITAL I
03/11/202	44	25/10/202	43	SP	GVE VII SAI	1332	SAO CAETA	354880	HOSPITAL S
08/08/202	32	30/07/202	30	SP	GVE XXVI S	1350	ITAPIRA	352260	HOSPITAL I
27/09/202	39	16/09/202	37	SP	GVE XVII CA	1342	CAMPINAS	350950	HOSPITAL I

24/02/202	8	22/02/202	8	CE	1 CRES FOF	1519	FORTALEZ/	230440	HOSPITAL (
23/02/202	8	09/02/202	6	SP	GVE VIII M	1333	GUARULHC	351880	HOSPITAL I
29/05/202	21	19/05/202	20	PR	17RS LONE	1371	LONDRINA	411370	HOSPITAL U
23/03/202	12	19/03/202	11	SP	GVE VIII M	1333	GUARULHC	351880	HOSPITAL I
22/03/202	12	12/03/202	10	SP	GVE I CAPI	1331	SAO PAULC	355030	HOSPITAL S
08/04/202	14	04/02/202	5	MA	REGI0NAL	1430	SAO LUIS	211130	MATERNID
03/04/202	13	23/03/202	12	PA	01 REGION	1484	BELEM	150140	SANTA CAS
17/06/202	24	07/06/202	23	RS	017 CRS	1604	IJUI	431020	HOSPITAL U
07/06/202	23	31/05/202	22	AC	REGIONAL	1938	RIO BRANC	120040	HOSPITAL I
24/05/202	21	17/05/202	20	TO			PALMAS	172100	HOSPITAL (
07/06/202	23	30/05/202	22	RJ			RIO DE JAN	330455	MS HSE HC
04/08/202	31	27/07/202	30	AL	3 MICRORI	1535	SAO MIGU	270860	SANTA CAS
03/05/202	18	03/05/202	18	CE	1 CRES FOF	1519	FORTALEZ/	230440	HGCC HOS
02/04/202	13	26/03/202	12	CE	1 CRES FOF	1519	FORTALEZ/	230440	HGCC HOS
06/06/202	23	28/05/202	21	SP	GVE XVII C/	1342	ITATIBA	352340	SANTA CAS
23/08/202	34	08/08/202	32	SP	GVE XVII C/	1342	CAMPINAS	350950	MATERNID
24/08/202	34	20/03/202	11	RJ			RIO DE JAN	330455	HOSPITAL (
22/03/202	12	16/03/202	11	MG	ALFENAS	1379	ALFENAS	310160	SANTA CAS
16/06/202	24	09/06/202	23	SP	GVE XXV S/	1349	CUBATAO	351350	HOSPITAL I
22/03/202	12	06/03/202	9	RJ			RIO DE JAN	330455	CASA DE S/
17/07/202	28	14/07/202	28	GO	CENTRAL	1779	GOIANIA	520870	HOSPITAL I
05/03/202	9	26/02/202	8	CE	15 CRES CF	1525	CRATEUS	230410	HOSPITAL S
18/03/202	11	08/03/202	10	SP	GVE I CAPI	1331	SAO PAULC	355030	HOSP MUN
06/03/202	9	06/03/202	9	CE	1 CRES FOF	1519	FORTALEZ/	230440	HGCC HOS
12/04/202	15	04/04/202	14	CE	1 CRES FOF	1519	FORTALEZ/	230440	HGCC HOS
22/03/202	12	22/03/202	12	CE	1 CRES FOF	1519	FORTALEZ/	230440	HGCC HOS
24/06/202	25	17/06/202	24	TO			PALMAS	172100	HOSPITAL (
13/08/202	32	10/08/202	32	SP	GVE XVI BC	1341	AVARE	350450	SANTA CAS
12/05/202	19	14/04/202	15	RJ			NILOPOLIS	330320	DOMINGO
13/03/202	10	07/03/202	10	MG	BELO HORI	1449	BETIM	310670	HOSPITAL I
20/05/202	20	14/05/202	19	TO			PALMAS	172100	HOSPITAL (
17/03/202	11	25/02/202	8	SP	GVE I CAPI	1331	SAO PAULC	355030	SAO LUIZ U
26/03/202	12	18/03/202	11	PR	15RS MARI	1369	MARINGA	411520	HOSPITAL U
12/04/202	15	05/04/202	14	MG	DIVINOPOI	1472	FORMIGA	312610	CENTRO DI
06/04/202	14	23/03/202	12	MG	BELO HORI	1449	BETIM	310670	UPA JOSE S
29/03/202	13	16/03/202	11	MG	BELO HORI	1449	NOVA LIM/	314480	NOVA LIM/
01/04/202	13	23/03/202	12	RO	VI GRS POF	1483	PORTO VEL	110020	HOSPITAL J
08/07/202	27	01/07/202	26	RJ			DUQUE DE	330170	HOSPITAL (
09/06/202	23	01/06/202	22	SP	GVE XXVII S	1351	SAO JOSE D	354990	HOSPITAL I
01/04/202	13	15/03/202	11	MG	MONTES C	1473	MONTES C	314330	PRONTOCC
26/03/202	12	17/03/202	11	PB	III NRS CAN	1421	CAMPINA (	250400	HOSPITAL I
10/05/202	19	04/05/202	18	PI	4 GRS TERE	1883	TERESINA	221100	MATERNID
10/04/202	14	09/04/202	14	RJ			RIO BONIT	330430	HOSPITAL I
01/05/202	17	18/04/202	16	MS	EX ARS DE	1975	CAMPO GF	500270	HOSPITAL I
02/08/202	31	10/07/202	27	SP	GVE I CAPI	1331	SAO PAULC	355030	MATERNID
02/06/202	22	26/05/202	21	SP	GVE VII SAI	1332	SANTO AN	354780	HOSPITAL I
09/09/202	36	28/08/202	34	MS	EX ARS DE	1975	CAMPO GF	500270	HOSPITAL I

20/06/202	25	17/06/202	24	RJ			PARAIBA D	330370	EPIDEMIOL
01/01/202	52	30/12/202	52	SP	GVE XI ARA	1336	PENAPOLIS	353730	SANTA CAS
30/10/202	43	26/10/202	43	CE	11 CRES SC	1521	SANTA QU	231220	SECRETARI
19/05/202	20	10/05/202	19	RJ			RIO DE JAN	330455	MS HSE HC
03/01/202	1	18/12/202	50	GO	CENTRAL	1779	GOIANIA	520870	HOSPITAL I
24/05/202	21	09/05/202	19	PR	10RS CASC	1364	CASCAVEL	410480	HOSPITAL I
22/04/202	16	13/04/202	15	MT	CUIABA	1578	CUIABA	510340	HOSPITAL I
22/04/202	16	11/04/202	15	DF			BRASILIA	530010	HOSPITAL S
26/05/202	21	10/05/202	19	SP	GVE XVII C.	1342	CAMPINAS	350950	MATERNID
20/05/202	20	10/05/202	19	SP	GVE XXI PR	1346	PRESIDENT	354140	HOSPITAL (
13/06/202	24	05/06/202	22	MT	CUIABA	1578	CUIABA	510340	HOSPITAL S
10/06/202	23	01/06/202	22	PA	01 REGION	1484	BELEM	150140	SANTA CAS
28/06/202	26	12/06/202	23	RS	013 CRS	1609	VENANCIO	432260	HOSPITAL S
19/01/202	3	05/01/202	1	MG	BELO HORI	1449	CONTAGEN	311860	CMI JUVEN
05/07/202	27	27/06/202	26	TO			PALMAS	172100	HOSPITAL (
14/10/202	41	29/09/202	39	RJ			RIO DE JAN	330455	MS HSE HC
07/06/202	23	30/05/202	22	SP	GVE XIX M.	1344	OSVALDO (	353460	SANTA CAS
15/07/202	28	10/07/202	27	SP	GVE XVII C.	1342	CAMPINAS	350950	MATERNID
16/07/202	28	28/06/202	26	SP	GVE I CAPI	1331	SAO PAULC	355030	MATERNID
29/03/202	13	23/03/202	12	PR	02RS METF	1356	CURITIBA	410690	COMPLEXC
12/02/202	6	01/02/202	5	CE	8 CRES QU	1517	QUIXERAN	231140	HOSPITAL I
17/02/202	7	13/02/202	6	PR	15RS MARI	1369	MARINGA	411520	SANTA CAS
14/03/202	11	25/02/202	8	MT	CUIABA	1578	CUIABA	510340	HOSPITAL S
25/02/202	8	29/12/202	52	RS	013 CRS	1609	RIO PARDC	431570	HOSPITAL I
02/04/202	13	18/03/202	11	ES	METROPOI	1510	SERRA	320500	HOSPITAL I
20/05/202	20	12/05/202	19	SC	JARAGUA I	1567	JARAGUA I	420890	HOSPITAL J
27/05/202	21	13/05/202	19	MT	CUIABA	1578	CUIABA	510340	HOSPITAL I
09/03/202	10	27/02/202	8	GO	PIRINEUS	1792	ANAPOLIS	520110	HOSPITAL I
09/05/202	19	27/04/202	17	PR	17RS LONI	1371	LONDRINA	411370	HOSPITAL I
15/03/202	11	02/03/202	9	MG	DIVINOPOI	1472	DIVINOPOI	312230	HOSPITAL S
18/06/202	24	08/06/202	23	MA	REGI0NAL	1430	SAO LUIS	211130	HOSPITAL S
13/09/202	37	12/08/202	32	SP	GVE VII SAI	1332	DIADEMA	351380	HOSPITAL I
19/07/202	29	10/07/202	27	MG	UBERLAND	1462	PATROCINI	314810	HOSPITAL S
22/06/202	25	05/06/202	22	MG	BELO HORI	1449	BELO HORI	310620	HOSPITAL J
10/09/202	36	02/09/202	35	SC	JOINVILLE	1565	JOINVILLE	420910	HOSPITAL (
17/05/202	20	09/05/202	19	PR	15RS MARI	1369	MARINGA	411520	HOSPITAL I
07/08/202	31	31/07/202	30	MT	SINOP	1584	SORRISO	510792	HOSPITAL I
10/11/202	45	31/10/202	44	PR	14RS PARA	1368	PARANAVA	411840	SANTA CAS
26/03/202	12	18/03/202	11	AM	ENTORNO	5584	MANAUS	130260	INSTITUTO
13/01/202	2	08/01/202	1	AM	ENTORNO	5584	MANAUS	130260	MATERNID
22/02/202	8	12/02/202	6	MG	JUIZ DE FO	1452	JUIZ DE FO	313670	HOSPITAL I
12/04/202	15	01/04/202	13	DF			BRASILIA	530010	HOSPITAL S
03/04/202	13	20/03/202	11	SP	GVE XXIV F	1348	RIBEIRAO F	354340	HOSPITAL I
27/01/202	4	25/01/202	4	BA	NUCLEO RI	1380	SALVADOR	292740	MATERNID
28/06/202	26	16/06/202	24	SP	GVE XVII C.	1342	CAMPINAS	350950	HOSPITAL V
02/06/202	22	20/05/202	20	BA	NUCLEO RI	1380	SALVADOR	292740	MATERNID
28/05/202	21	15/05/202	19	RJ			NITEROI	330330	HOSPITAL I

04/08/202	31	21/07/202	29	GO	CENTRAL	1779	GOIANIA	520870	HOSPITAL I
03/08/202	31	19/07/202	29	SP	GVE I CAPI	1331	SAO PAULO	355030	SAO LUIZ U
20/08/202	33	14/08/202	32	PR	09RS FOZ D	1363	FOZ DO IGU	410830	HOSPITAL I
23/01/202	3	17/01/202	3	MG	PEDRA AZU	1457	ALMENARA	310170	HOSPITAL I
15/04/202	15	24/03/202	12	RS	002 CRS	1607	PORTO ALE	431490	HOSPITAL I
14/03/202	11	03/03/202	9	GO	PIRINEUS	1792	ANAPOLIS	520110	SANTA CAS
15/02/202	7	09/02/202	6	SP	GVE I CAPI	1331	SAO PAULO	355030	HOSPITAL I
31/01/202	5	29/01/202	4	AP			MACAPA	160030	HOSPITAL I
25/08/202	34	13/08/202	32	DF			BRASILIA	530010	HRSM
17/05/202	20	10/05/202	19	MG	SETE LAGO	1467	CURVELO	312090	PRONTO A
14/04/202	15	09/04/202	14	RJ			RIO DE JAN	330455	SMS HOSPI
25/07/202	30	20/07/202	29	MG	UBERLAND	1462	UBERLAND	317020	HOSPITAL S
10/08/202	32	21/07/202	29	SP	GVE I CAPI	1331	SAO PAULO	355030	HOSPITAL S
23/08/202	34	19/08/202	33	RJ			RIO DE JAN	330455	MS HSE HO
14/08/202	32	06/08/202	31	MA	REGIONAL	1440	IMPERATRI	210530	HRMI HOSI
15/03/202	11	15/03/202	11	RS	002 CRS	1607	PORTO ALE	431490	HOSPITAL I
05/05/202	18	27/04/202	17	PR	08RS FRAN	1362	FRANCISCO	410840	HOSPITAL I
25/05/202	21	21/05/202	20	TO			PALMAS	172100	HOSPITAL (
07/04/202	14	25/03/202	12	PR	17RS LOND	1371	LONDRINA	411370	HOSPITAL U
10/08/202	32	01/08/202	31	SP	GVE XXI PR	1346	PRESIDENT	354140	HOSPITAL I
15/05/202	19	02/05/202	18	RO	VI GRS POF	1483	PORTO VEL	110020	HOSPITAL I
06/07/202	27	04/07/202	27	SP	GVE I CAPI	1331	SAO PAULO	355030	HOSPITAL S
05/04/202	14	15/03/202	11	SP	GVE I CAPI	1331	SAO PAULO	355030	SAO LUIZ U
27/05/202	21	12/05/202	19	MT	CUIABA	1578	CUIABA	510340	HOSPITAL U
17/03/202	11	08/03/202	10	SP	GVE XVII C	1342	CAMPINAS	350950	MATERNID
07/04/202	14	03/04/202	13	SP	GVE XII AR	1337	SAO CARLO	354890	SANTA CAS
25/06/202	25	28/05/202	21	SP	GVE XVII C	1342	JAGUARIUI	352470	HOSPITAL I
02/06/202	22	25/05/202	21	MT	CUIABA	1578	CUIABA	510340	HOSPITAL U
15/06/202	24	11/05/202	19	RS	002 CRS	1607	PORTO ALE	431490	IRMANDAD
01/04/202	13	22/03/202	12	RS	002 CRS	1607	PORTO ALE	431490	HOSPITAL I
22/06/202	25	14/06/202	24	BA	NUCLEO RI	1380	SALVADOR	292740	MATERNID
21/06/202	25	13/06/202	24	MG	DIVINOPOI	1472	DIVINOPOI	312230	HOSPITAL I
13/09/202	37	04/09/202	35	RJ			RIO DE JAN	330455	MS HSE HO
16/04/202	15	15/02/202	7	BA	NUCLEO RI	1380	SALVADOR	292740	HOSPITAL S
23/02/202	8	15/02/202	7	SP	GVE XXIX S	1354	SAO JOSE D	354980	HOSPITAL I
04/10/202	40	26/09/202	39	MT	CUIABA	1578	CUIABA	510340	HOSPITAL U
07/03/202	10	01/03/202	9	MG	DIVINOPOI	1472	PITANGUI	315140	SANTA CAS
25/03/202	12	17/03/202	11	SP	GVE XXVI S	1350	MOJI MIRII	353080	HOSPITAL 2
30/03/202	13	24/03/202	12	PE	001	1497	RECIFE	261160	IMIP
14/04/202	15	07/04/202	14	CE	1 CRES FOF	1519	FORTALEZA	230440	HGCC HOS
08/09/202	36	02/09/202	35	SP	GVE XXXI S	1353	SALTO DE F	354530	SANTA CAS
23/04/202	16	15/04/202	15	MT	SINOP	1584	SINOP	510790	HOSPITAL S
11/06/202	23	31/05/202	22	PR	10RS CASC	1364	CASCAVEL	410480	HOSPITAL U
15/04/202	15	11/04/202	15	MG	LEOPOLDIN	1453	LEOPOLDIN	313840	CASA DE C
17/03/202	11	11/03/202	10	CE	1 CRES FOF	1519	FORTALEZA	230440	HOSPITAL I
30/07/202	30	24/07/202	29	RR			BOA VISTA	140010	HOSPITAL (
28/08/202	34	18/08/202	33	GO	ESTRADA D	1802	CATALAO	520510	SANTA CAS

06/08/202	31	29/07/202	30	RJ			RIO DE JAN	330455	HOSPITAL (
20/07/202	29	08/07/202	27	TO			PALMAS	172100	HOSPITAL (
14/07/202	28	06/07/202	27	MG	POUSO ALE	1459	POUSO ALE	315250	HOSPITAL I
26/04/202	17	15/04/202	15	CE	1 CRES FOF	1519	FORTALEZA	230440	IJF INSTITU
08/02/202	6	29/01/202	4	SC	BLUMENAU	1564	BLUMENAU	420240	SECRETARI
02/06/202	22	24/05/202	21	BA	NUCLEO RI	1380	SALVADOR	292740	MATERNID
31/03/202	13	24/03/202	12	MT	CUIABA	1578	CUIABA	510340	HOSPITAL U
12/03/202	10	09/03/202	10	SP	GVE XXVI S	1350	VARGEM G	355640	CENTRO DI
14/04/202	15	05/04/202	14	PE	001	1497	RECIFE	261160	REAL HOSP
04/03/202	9	19/02/202	7	DF			BRASILIA	530010	HRAN
10/03/202	10	23/02/202	8	GO	CENTRAL	1779	GOIANIA	520870	HOSPITAL I
08/03/202	10	22/02/202	8	RS	006 CRS	1611	PASSO FUN	431410	HOSPITAL S
06/09/202	36	29/08/202	35	MT	CUIABA	1578	CUIABA	510340	HOSPITAL U
26/03/202	12	25/03/202	12	SP	GVE XVII C.	1342	JUNDIAI	352590	HU HOSPIT
22/10/202	42	21/10/202	42	PB	I NRS JOAC	1377	JOAO PESS	250750	MATERNID
05/04/202	14	24/03/202	12	MG	BELO HORI	1449	BETIM	310670	HOSPITAL I
14/02/202	7	06/02/202	5	RO	VI GRS POF	1483	PORTO VEL	110020	HOSPITAL I
21/04/202	16	13/04/202	15	AL	1 MICRORI	1533	MACEIO	270430	HOSPITAL U
20/04/202	16	15/04/202	15	PR	15RS MARI	1369	MARINGA	411520	SANTA CAS
03/06/202	22	25/05/202	21	SP	GVE XVII C.	1342	CAMPINAS	350950	HOSPITAL V
19/05/202	20	18/03/202	11	PA	01 REGION	1484	BELEM	150140	SANTA CAS
20/09/202	38	13/09/202	37	RS	018 CRS	1605	CAPAO DA	430463	HOSPITAL I
16/05/202	20	16/05/202	20	MG	JUIZ DE FO	1452	JUIZ DE FO	313670	SANTA CAS
03/07/202	26	30/06/202	26	MG	BELO HORI	1449	BELO HORI	310620	HOSPITAL I
11/05/202	19	03/05/202	18	SP	GVE XVII C.	1342	SUMARE	355240	HOSPITAL I
03/09/202	35	22/08/202	34	MT	CUIABA	1578	CUIABA	510340	HOSPITAL U
29/11/202	48	13/05/202	19	GO	OESTE I	1795	BOM JARD	520340	HOSPITAL I
19/07/202	29	06/07/202	27	SP	GVE XVII C.	1342	CAMPINAS	350950	HOSPITAL I
04/07/202	27	25/06/202	25	SP	GVE XX PIR	1345	RIO CLARO	354390	SANTA CAS
21/06/202	25	06/06/202	23	RN	VII URSAP	1416	NATAL	240810	HOSPITAL I
06/12/202	49	30/11/202	48	RJ			DUQUE DE	330170	HOSPITAL (
04/04/202	14	01/04/202	13	PI	4 GRS TERE	1883	TERESINA	221100	MATERNID
04/03/202	9	22/02/202	8	SC	CRICIUMA	1558	CRICIUMA	420460	HOSPITAL S
02/04/202	13	23/03/202	12	CE	1 CRES FOF	1519	FORTALEZA	230440	HOSPITAL I
03/05/202	18	29/04/202	17	SC	XANXERE	1556	XANXERE	421950	HOSPITAL I
02/06/202	22	19/05/202	20	RS	002 CRS	1607	PORTO ALE	431490	HOSPITAL I
11/05/202	19	24/04/202	16	PB	I NRS JOAC	1377	JOAO PESS	250750	MATERNID
21/05/202	20	25/04/202	17	SP	GVE I CAPI	1331	SAO PAULO	355030	HC DA FMU
23/06/202	25	13/06/202	24	TO			ARAGUAIN	170210	HOSPITAL I
28/06/202	26	17/06/202	24	RJ			RIO DE JAN	330455	HOSPITAL (
03/04/202	13	02/04/202	13	SP	GVE XXI PR	1346	PRESIDENT	354140	HOSPITAL I
18/07/202	29	09/07/202	27	GO	PIRINEUS	1792	ANAPOLIS	520110	SANTA CAS
05/05/202	18	23/04/202	16	SP	GVE I CAPI	1331	SAO PAULO	355030	HC DA FMU
15/06/202	24	10/06/202	23	MS	EX ARS DE	1975	CAMPO GR	500270	HOSPITAL I
19/04/202	16	28/03/202	13	MS	EX ARS DE	1975	CAMPO GR	500270	HOSPITAL I
26/01/202	4	12/01/202	2	MG	BELO HORI	1449	BETIM	310670	HOSPITAL I
10/07/202	27	28/06/202	26	RS	002 CRS	1607	PORTO ALE	431490	HOSPITAL I

30/04/202	17	20/04/202	16	SP	GVE XIX M/	1344	MARILIA	352900	HOSPITAL l
15/03/202	11	03/03/202	9	DF			BRASILIA	530010	HOSPITAL S
05/10/202	40	03/10/202	40	MT	CUIABA	1578	CUIABA	510340	HOSPITAL S
08/03/202	10	28/02/202	9	RJ			NITEROI	330330	SES RJ HOS
19/06/202	24	16/06/202	24	PR	15RS MARI	1369	MARINGA	411520	SANTA CAS
13/04/202	15	03/04/202	13	SC	JOINVILLE	1565	JOINVILLE	420910	HOSPITAL I
16/07/202	28	04/07/202	27	PR	17RS LOND	1371	LONDRINA	411370	HOSPITAL l
09/06/202	23	27/05/202	21	MT	CUIABA	1578	CUIABA	510340	HOSPITAL l
27/02/202	8	22/02/202	8	PR	15RS MARI	1369	SARANDI	412625	METROPOI
21/05/202	20	09/05/202	19	SP	GVE I CAPI	1331	SAO PAULC	355030	HC DA FMU
28/06/202	26	18/06/202	24	BA	NUCLEO RI	1380	SALVADOR	292740	MATERNID
04/06/202	22	28/05/202	21	RS	002 CRS	1607	PORTO ALE	431490	HOSPITAL I
11/07/202	28	06/07/202	27	GO	CENTRAL	1779	GOIANIA	520870	HOSPITAL I
19/07/202	29	09/07/202	27	MG	PASSOS	1455	SAO SEBAS	316470	SANTA CAS
31/05/202	22	26/05/202	21	RJ			RIO DE JAN	330455	MS HSE HC
01/08/202	31	26/07/202	30	PA	12 REGION	1495	REDENCAC	150613	HOSPITAL I
07/06/202	23	22/05/202	20	RN	VII URSAP	1416	NATAL	240810	HOSPITAL I
19/07/202	29	09/07/202	27	RR			BOA VISTA	140010	HOSPITAL (
28/07/202	30	02/07/202	26	RS	016 CRS	1610	LAJEADO	431140	HOSPITAL I
23/08/202	34	15/08/202	33	SC	JOINVILLE	1565	JOINVILLE	420910	HOSPITAL I
09/09/202	36	02/09/202	35	SC	FLORIANOI	1476	FLORIANOI	420540	HOSPITAL I
11/05/202	19	09/05/202	19	AM	ENTORNO	5584	MANAUS	130260	MATERNID
26/01/202	4	12/01/202	2	SP	GVE VII SAI	1332	SAO BERN/	354870	HOSPITAL S
10/11/202	45	24/10/202	43	SC	JARAGUA I	1567	JARAGUA I	420890	HOSPITAL J
12/04/202	15	28/03/202	13	GO	CENTRAL	1779	GOIANIA	520870	HOSPITAL I
18/01/202	3	10/01/202	2	PR	02RS METF	1356	CAMPO LA	410420	HOSPITAL I
08/04/202	14	31/03/202	13	RS	001 CRS	1592	MONTENE(	431240	HOSPITAL l
04/03/202	9	22/02/202	8	SP	GVE I CAPI	1331	SAO PAULC	355030	MATERNID
24/05/202	21	20/05/202	20	BA	NUCLEO RI	1380	SALVADOR	292740	MATERNID
21/06/202	25	02/06/202	22	MG	PATOS DE I	1456	PATOS DE I	314800	HOSPITAL V
29/03/202	13	28/03/202	13	PR	15RS MARI	1369	SARANDI	412625	METROPOI
02/04/202	13	24/03/202	12	SP	GVE XII AR/	1337	MATAO	352930	HOSPITAL (
15/06/202	24	02/06/202	22	SP	GVE XXIV R	1348	RIBEIRAO F	354340	HOSPITAL I
26/05/202	21	17/05/202	20	SP	GVE XXVI S	1350	MOGI GUA	353070	SANTA CAS
14/06/202	24	12/06/202	23	RS	002 CRS	1607	PORTO ALE	431490	HOSPITAL I
02/05/202	18	02/05/202	18	CE	1 CRES FOF	1519	FORTALEZ/	230440	HGCC HOS
30/06/202	26	20/06/202	25	PR	07RS PATO	1361	PATO BRAI	411850	ISSAL
01/10/202	39	30/09/202	39	PB	I NRS JOAC	1377	JOAO PESS	250750	MATERNID
19/04/202	16	08/04/202	14	PR	17RS LOND	1371	LONDRINA	411370	HOSPITAL I
30/03/202	13	22/03/202	12	CE	11 CRES SC	1521	SOBRAL	231290	HOSPITAL I
27/04/202	17	23/04/202	16	SP	GVE XVII C/	1342	CAMPINAS	350950	HOSPITAL I
10/03/202	10	01/03/202	9	MT	CUIABA	1578	CUIABA	510340	HOSPITAL S
03/02/202	5	27/01/202	4	PR	10RS CASC	1364	CASCAVEL	410480	HOSPITAL I
20/06/202	25	11/06/202	23	GO	PIRINEUS	1792	ANAPOLIS	520110	SANTA CAS
01/07/202	26	22/06/202	25	SC	BLUMENAU	1564	BLUMENAU	420240	HOSPITAL S
31/05/202	22	26/05/202	21	RJ			CAMPOS D	330100	HOSPITAL I
28/07/202	30	18/07/202	29	PR	09RS FOZ D	1363	FOZ DO IGU	410830	HOSPITAL I

26/03/202	12	18/03/202	11	PR	08RS FRAN	1362	SANTO ANT	412440	HOSPITAL I
23/02/202	8	22/02/202	8	SP	GVE XXXI S	1353	SOROCABA	355220	HOSPITAL U
24/09/202	38	19/09/202	38	SP	GVE VII SAI	1332	SAO BERNA	354870	HOSPITAL I
26/05/202	21	16/05/202	20	SP	GVE XXXI S	1353	SOROCABA	355220	HOSPITAL I
10/06/202	23	03/06/202	22	ES	METROPOI	1510	SERRA	320500	HOSPITAL I
21/02/202	8	12/02/202	6	PA	09 REGION	1492	SANTAREM	150680	UNIDADE D
10/03/202	10	01/03/202	9	SP	GVE XXIX S	1354	SAO JOSE D	354980	HOSPITAL A
27/04/202	17	12/04/202	15	SP	GVE I CAPI	1331	SAO PAULO	355030	HC DA FMU
26/04/202	17	16/04/202	15	SP	GVE XXI PR	1346	PRESIDENT	354140	HOSPITAL (
15/07/202	28	01/07/202	26	MT	BARRA DO	1580	BARRA DO	510180	UPA 24 HO
22/04/202	16	11/04/202	15	ES	METROPOI	1510	SERRA	320500	HOSPITAL I
22/05/202	20	15/05/202	19	SP	GVE I CAPI	1331	SAO PAULO	355030	HOSP MAT
18/05/202	20	13/05/202	19	SP	GVE I CAPI	1331	SAO PAULO	355030	HC DA FMU
24/05/202	21	11/05/202	19	SP	GVE XXXI S	1353	SOROCABA	355220	CONJUNTO
27/05/202	21	14/05/202	19	DF			BRASILIA	530010	HRAN
21/06/202	25	16/06/202	24	SP	GVE XV BA	1340	BAURU	350600	MATERNID
03/08/202	31	01/08/202	31	SP	GVE I CAPI	1331	SAO PAULO	355030	SAO LUIZ U
16/07/202	28	01/07/202	26	SP	GVE I CAPI	1331	SAO PAULO	355030	SAO LUIZ U
24/09/202	38	16/09/202	37	GO	CENTRO SU	1781	APARECIDA	520140	HOSPITAL (
20/09/202	38	12/09/202	37	PR	17RS LOND	1371	LONDRINA	411370	HOSPITAL U
24/04/202	16	20/04/202	16	RJ			VASSOURA	330620	HUV HOSP
27/04/202	17	26/04/202	17	PR	15RS MARI	1369	MARINGA	411520	SANTA CAS
24/05/202	21	18/05/202	20	GO	CENTRAL	1779	GOIANIA	520870	HOSPITAL I
22/04/202	16	10/03/202	10	RN	VII URSAP	1416	NATAL	240810	PROMATEF
08/03/202	10	25/02/202	8	RS	002 CRS	1607	PORTO ALE	431490	HOSPITAL I
13/03/202	10	01/03/202	9	RS	007 CRS	1596	BAGE	430160	SANTA CAS
07/06/202	23	26/05/202	21	SP	GVE I CAPI	1331	SAO PAULO	355030	MATERNID
21/04/202	16	12/04/202	15	MS	NRS DE DO	1966	DOURADO	500370	EBSERH HO
14/04/202	15	05/04/202	14	SE	REGIONAL	2056	ARACAJU	280030	HOSPITAL S
03/05/202	18	21/04/202	16	GO	CENTRAL	1779	GOIANIA	520870	HOSPITAL I
06/09/202	36	28/08/202	34	SC	BLUMENAU	1564	BLUMENAU	420240	HOSPITAL S
14/06/202	24	03/06/202	22	SP	GVE XXVI S	1350	MOGI GUA	353070	SANTA CAS
17/08/202	33	15/08/202	33	SP	GVE XVIII F	1343	GUARA	351770	SANTA CAS
12/03/202	10	02/03/202	9	SP	GVE I CAPI	1331	SAO PAULO	355030	HC DA FMU
30/03/202	13	20/03/202	11	RJ			RIO DE JAN	330455	SMS HOSPI
05/04/202	14	24/03/202	12	RS	002 CRS	1607	PORTO ALE	431490	HOSPITAL I
31/03/202	13	17/03/202	11	SP	GVE I CAPI	1331	SAO PAULO	355030	HC DA FMU
26/02/202	8	20/02/202	7	RJ			VOLTA RED	330630	SECRETARI
17/04/202	15	06/04/202	14	MG	CORONEL F	1468	CARATINGA	311340	CASU IRMA
11/03/202	10	28/02/202	9	RO	I GRS JI PA	1474	JI-PARANA	110012	HOSPITAL I
12/04/202	15	12/04/202	15	CE	1 CRES FOF	1519	FORTALEZA	230440	HGCC HOS
01/06/202	22	30/05/202	22	SP	GVE VIII M	1333	GUARULHO	351880	HOSPITAL I
10/05/202	19	06/05/202	18	MA	REGI0NAL	1430	SAO LUIS	211130	MATERNID
13/05/202	19	07/05/202	18	SP	GVE I CAPI	1331	SAO PAULO	355030	HC DA FMU
09/05/202	19	01/05/202	17	MG	PASSOS	1455	CAPITOLIO	311280	SANTA CAS
23/08/202	34	10/08/202	32	GO	CENTRAL	1779	GOIANIA	520870	HOSPITAL I
19/07/202	29	06/07/202	27	MT	CUIABA	1578	CUIABA	510340	HOSPITAL U

02/08/202	31	25/07/202	30	SC	JOINVILLE	1565	JOINVILLE	420910	CENTRO H(
30/07/202	30	18/04/202	16	PR	16RS APUC	1370	APUCARAN	410140	HNSG HOS
05/07/202	27	22/06/202	25	RJ			CAMPOS D	330100	HOSPITAL I
29/03/202	13	17/03/202	11	RS	006 CRS	1611	PASSO FUN	431410	HOSPITAL I
12/08/202	32	27/07/202	30	RS	002 CRS	1607	PORTO ALE	431490	HOSPITAL I
02/05/202	18	24/04/202	16	SC	ARARANGU	1562	ARARANGU	420140	IMAS HOSP
22/09/202	38	28/08/202	34	PR	10RS CASC	1364	CASCAVEL	410480	HOSPITAL U
24/03/202	12	08/03/202	10	SP	GVE I CAPI	1331	SAO PAULO	355030	HOSP MUN
08/09/202	36	29/08/202	35	TO			PALMAS	172100	HOSPITAL (
24/04/202	16	18/04/202	16	SE	REGIONAL	2056	ARACAJU	280030	PRONTO CI
13/10/202	41	03/10/202	40	PR	02RS METF	1356	CAMPINA (	410400	HOSPITAL A
29/03/202	13	18/03/202	11	GO	CENTRAL	1779	GOIANIA	520870	HOSPITAL I
22/04/202	16	13/04/202	15	MT	CUIABA	1578	CUIABA	510340	HOSPITAL U
08/04/202	14	02/04/202	13	RO	VI GRS POF	1483	PORTO VEL	110020	HOSPITAL I
05/03/202	9	27/02/202	8	MG	UBERLAND	1462	UBERLAND	317020	UAI MARTI
26/04/202	17	07/04/202	14	SP	GVE I CAPI	1331	SAO PAULO	355030	SAO LUIZ U
27/03/202	12	26/03/202	12	SP	GVE XXV SA	1349	GUARUJA	351870	HOSPITAL S
01/04/202	13	16/03/202	11	SP	GVE I CAPI	1331	SAO PAULO	355030	HOSP MUN
15/06/202	24	25/05/202	21	BA	NUCLEO RE	1380	SALVADOR	292740	HOSPITAL I
01/03/202	9	27/02/202	8	MG	BELO HORI	1449	BELO HORI	310620	HOSPITAL S
29/09/202	39	17/09/202	37	SP	GVE I CAPI	1331	SAO PAULO	355030	HOSPITAL I
09/04/202	14	02/04/202	13	RJ			RIO DE JAN	330455	SMS MATE
08/11/202	45	06/11/202	44	PI	4 GRS TERE	1883	TERESINA	221100	MATERNID
13/01/202	2	03/01/202	1	SP	GVE I CAPI	1331	SAO PAULO	355030	SAO LUIZ U
30/01/202	4	22/01/202	3	AM	ENTORNO	5584	MANAUS	130260	INSTITUTO
14/03/202	11	11/03/202	10	SP	GVE I CAPI	1331	SAO PAULO	355030	HOSP MUN
10/02/202	6	06/02/202	5	MT	CUIABA	1578	CUIABA	510340	HOSPITAL U
12/04/202	15	10/04/202	14	SP	GVE XIX MA	1344	OSVALDO (	353460	SANTA CAS
28/07/202	30	20/07/202	29	PR	02RS METF	1356	CURITIBA	410690	COMPLEXO
21/05/202	20	13/05/202	19	SP	GVE I CAPI	1331	SAO PAULO	355030	MATERNID
25/03/202	12	20/03/202	11	SP	GVE I CAPI	1331	SAO PAULO	355030	HOSPITAL I
08/05/202	18	23/04/202	16	SP	GVE X OSA	1335	OSASCO	353440	HOSPITAL I
25/06/202	25	14/06/202	24	SP	GVE I CAPI	1331	SAO PAULO	355030	SAO LUIZ U
18/05/202	20	07/05/202	18	SP	GVE I CAPI	1331	SAO PAULO	355030	HC DA FMU
10/04/202	14	01/04/202	13	MG	BELO HORI	1449	BELO HORI	310620	HOSPITAL I
09/03/202	10	23/02/202	8	PR	03RS PONT	1357	PONTA GR(	411990	UNIDADE D
06/05/202	18	03/05/202	18	SP	GVE I CAPI	1331	SAO PAULO	355030	HOSP MUN
26/05/202	21	11/05/202	19	SP	GVE I CAPI	1331	SAO PAULO	355030	HC DA FMU
10/08/202	32	30/07/202	30	PR	02RS METF	1356	CURITIBA	410690	HOSPITAL S
22/01/202	3	09/01/202	1	SP	GVE XVIII F	1343	FRANCA	351620	SANTA CAS
31/03/202	13	23/03/202	12	SP	GVE I CAPI	1331	SAO PAULO	355030	HOSP MUN
28/06/202	26	01/06/202	22	MS	EX ARS DE	1975	CAMPO GF	500270	HOSPITAL I
01/02/202	5	25/01/202	4	BA	NUCLEO RE	1380	SALVADOR	292740	HOSPITAL I
28/02/202	9	18/02/202	7	RO	VI GRS POF	1483	PORTO VEL	110020	HOSPITAL I
14/03/202	11	14/03/202	11	RS	002 CRS	1607	PORTO ALE	431490	HOSPITAL I
10/03/202	10	28/02/202	9	PA	01 REGION	1484	BELEM	150140	MATERNID
07/06/202	23	25/05/202	21	SP	GVE I CAPI	1331	SAO PAULO	355030	MATERNID

12/04/202	15	03/04/202	13	SP	GVE XVII C/	1342	JUNDIAI	352590	HU HOSPIT
30/03/202	13	24/03/202	12	SP	GVE X OSA	1335	OSASCO	353440	HOSPITAL I
20/07/202	29	02/07/202	26	RJ			DUQUE DE	330170	HOSPITAL I
11/08/202	32	05/08/202	31	MT	CUIABA	1578	CUIABA	510340	HOSPITAL U
31/03/202	13	17/03/202	11	SP	GVE XXXI S	1353	SOROCABA	355220	HOSPITAL S
06/03/202	9	25/02/202	8	RJ			RIO DE JAN	330455	HOSPITAL (
29/06/202	26	16/06/202	24	SP	GVE XXI PR	1346	PRESIDENT	354140	HOSPITAL I
08/04/202	14	31/03/202	13	SP	GVE I CAPI	1331	SAO PAULO	355030	HOSPITAL S
19/03/202	11	06/03/202	9	RS	002 CRS	1607	PORTO ALE	431490	HOSPITAL I
17/03/202	11	06/03/202	9	BA	NUCLEO RI	1380	SALVADOR	292740	INSTITUTO
09/04/202	14	16/03/202	11	ES	METROPOI	1510	SERRA	320500	HOSPITAL I
08/07/202	27	29/06/202	26	PR	02RS METF	1356	CURITIBA	410690	COMPLEXO
26/04/202	17	08/04/202	14	BA	NUCLEO RI	1380	SALVADOR	292740	HOSPITAL S
24/06/202	25	08/06/202	23	SP	GVE XXIV R	1348	RIBEIRAO P	354340	HOSPITAL S
04/08/202	31	29/07/202	30	SP	GVE XVII C/	1342	CAMPINAS	350950	HOSPITAL I
18/05/202	20	28/04/202	17	DF			BRASILIA	530010	HOSPITAL S
30/07/202	30	13/04/202	15	PR	16RS APUC	1370	APUCARAN	410140	HNSG HOS
24/06/202	25	12/06/202	23	PR	14RS PARA	1368	PARANAVA	411840	SANTA CAS
25/05/202	21	13/05/202	19	MS	EX ARS DE	1975	CAMPO GR	500270	HOSPITAL I
13/04/202	15	09/04/202	14	MT	CUIABA	1578	CUIABA	510340	HOSPITAL S
25/05/202	21	16/05/202	20	MT	SINOP	1584	NOVA MUT	510622	HOSPITAL I
13/07/202	28	01/07/202	26	SP	GVE I CAPI	1331	SAO PAULO	355030	HC DA FMU
06/08/202	31	31/07/202	30	SP	GVE XV BA	1340	JAU	352530	SANTA CAS
31/05/202	22	27/05/202	21	SP	GVE XVII C/	1342	CAMPINAS	350950	HOSPITAL I
10/05/202	19	27/04/202	17	GO	CENTRAL	1779	GOIANIA	520870	HOSPITAL I
07/06/202	23	26/05/202	21	RN	II URSAP	1412	MOSSORO	240800	HOSPITAL I
11/03/202	10	26/02/202	8	SP	GVE XIX M/	1344	MARILIA	352900	HOSPITAL I
09/06/202	23	07/04/202	14	DF			BRASILIA	530010	HOSPITAL S
12/03/202	10	12/03/202	10	RJ			VASSOURA	330620	HUV HOSP
05/05/202	18	23/04/202	16	RO	VI GRS POF	1483	PORTO VEL	110020	HOSPITAL I
05/05/202	18	10/04/202	14	MT	CUIABA	1578	CUIABA	510340	HOSPITAL U
02/05/202	18	01/05/202	17	PR	07RS PATO	1361	PATO BRAN	411850	ISSAL
26/05/202	21	15/05/202	19	SP	GVE I CAPI	1331	SAO PAULO	355030	HOSPITAL S
23/06/202	25	13/06/202	24	RR			BOA VISTA	140010	HOSPITAL (
09/06/202	23	28/05/202	21	PR	17RS LOND	1371	LONDRINA	411370	HOSPITAL U
18/04/202	16	09/04/202	14	PR	17RS LOND	1371	LONDRINA	411370	HOSPITAL U
23/04/202	16	10/04/202	14	SP	GVE XXXI S	1353	VOTORANT	355700	DEPARTAM
19/04/202	16	13/04/202	15	RS	004 CRS	1595	SANTA MA	431690	HUSM HOS
29/07/202	30	18/07/202	29	MS	EX ARS DE	1975	CAMPO GR	500270	HOSPITAL I
22/02/202	8	12/02/202	6	SC	BLUMENAU	1564	BLUMENAU	420240	HOSPITAL S
25/02/202	8	10/02/202	6	SP	GVE I CAPI	1331	SAO PAULO	355030	HOSPITAL I
14/02/202	7	30/01/202	4	SP	GVE I CAPI	1331	SAO PAULO	355030	MATERNID
17/05/202	20	17/05/202	20	CE	1 CRES FOF	1519	FORTALEZA	230440	HGCC HOS
18/02/202	7	16/02/202	7	RJ			ANGRA DO	330010	HOSPITAL I
29/01/202	4	26/01/202	4	SP	GVE VII SAI	1332	RIBEIRAO P	354330	HOSPITAL I
26/03/202	12	16/03/202	11	PR	02RS METF	1356	CURITIBA	410690	COMPLEXO
08/04/202	14	29/03/202	13	GO	CENTRAL	1779	GOIANIA	520870	HOSPITAL I

19/01/202	3	09/01/202	1	SP	GVE XX PIR	1345	LIMEIRA	352690	HOSPITAL I
06/06/202	23	21/05/202	20	SC	CHAPECO	1553	CHAPECO	420420	HOSPITAL I
22/06/202	25	13/06/202	24	SP	GVE XVI BC	1341	PARANAPA	353580	HOSPITAL I
27/02/202	8	19/02/202	7	MA	REGIONAL	1440	IMPERATRI	210530	HRMI HOSI
23/04/202	16	10/04/202	14	MS	NRS DE TRI	6255	TRES LAGO	500830	HOSPITAL (
29/04/202	17	26/04/202	17	AM	ENTORNO	5584	MANAUS	130260	MATERNID
13/09/202	37	31/08/202	35	RJ			RIO DE JAN	330455	MS HSE HC
07/06/202	23	27/05/202	21	SP	GVE I CAPI	1331	SAO PAULC	355030	HC DA FMU
24/03/202	12	09/03/202	10	MT	CUIABA	1578	CUIABA	510340	FEMINA HC
11/05/202	19	04/05/202	18	RN	II URSAP	1412	MOSSORO	240800	HOSPITAL I
11/07/202	28	02/07/202	26	MA	REGI0NAL	1430	SAO LUIS	211130	MATERNID
20/05/202	20	18/05/202	20	MG	BELO HORI	1449	BELO HORI	310620	BIOCOR IN
01/02/202	5	10/01/202	2	GO	CENTRAL	1779	GOIANIA	520870	HOSPITAL I
10/09/202	36	07/09/202	36	RJ			RIO DE JAN	330455	MS HSE HC
18/05/202	20	08/05/202	18	BA	NUCLEO RI	1381	FEIRA DE S.	291080	HOSPITAL I
12/06/202	23	04/06/202	22	TO			PALMAS	172100	HOSPITAL (
10/06/202	23	02/06/202	22	SP	GVE XV BA	1340	BAURU	350600	MATERNID
23/06/202	25	10/06/202	23	GO	CENTRAL	1779	GOIANIA	520870	HOSPITAL I
24/02/202	8	18/02/202	7	SP	GVE I CAPI	1331	SAO PAULC	355030	HOSPITAL S
25/08/202	34	15/08/202	33	GO	CENTRAL	1779	GOIANIA	520870	HOSPITAL I
17/05/202	20	06/02/202	5	CE	1 CRES FOF	1519	FORTALEZ/	230440	HOSPITAL I
04/06/202	22	26/05/202	21	SP	GVE I CAPI	1331	SAO PAULC	355030	HC DA FMU
09/09/202	36	29/08/202	35	PR	10RS CASC	1364	CASCAVEL	410480	HOSPITAL U
27/04/202	17	25/04/202	17	BA	NUCLEO RI	1380	SALVADOR	292740	HOSPITAL I
15/04/202	15	13/04/202	15	SP	GVE VIII M	1333	GUARULHC	351880	HOSPITAL I
30/08/202	35	20/08/202	33	SP	GVE XVII C.	1342	CAMPINAS	350950	HOSPITAL I
18/05/202	20	08/05/202	18	PR	20RS TOLEI	1374	MARECHAI	411460	HOSPITAL I
27/07/202	30	20/07/202	29	PR	09RS FOZ D	1363	FOZ DO IGU	410830	HOSPITAL I
31/08/202	35	23/08/202	34	MS	NRS DE DO	1966	DOURADO.	500370	EBSERH HC
24/05/202	21	15/05/202	19	RS	002 CRS	1607	PORTO ALE	431490	HOSPITAL I
01/06/202	22	28/05/202	21	SP	GVE I CAPI	1331	SAO PAULC	355030	HOSPITAL S
03/12/202	48	28/10/202	43	ES	METROPOI	1510	SERRA	320500	HOSPITAL I
18/03/202	11	18/03/202	11	SP	GVE XVII C.	1342	CAMPINAS	350950	HOSPITAL I
07/04/202	14	31/03/202	13	SP	GVE I CAPI	1331	SAO PAULC	355030	PRO MATR
14/05/202	19	29/04/202	17	SP	GVE I CAPI	1331	SAO PAULC	355030	HC DA FMU
06/04/202	14	18/03/202	11	CE	1 CRES FOF	1519	FORTALEZ/	230440	HOSPITAL /
09/05/202	19	30/04/202	17	MA	REGIONAL	1440	IMPERATRI	210530	HRMI HOSI
17/06/202	24	08/05/202	18	GO	CENTRAL	1779	GOIANIA	520870	HOSPITAL I
04/06/202	22	24/05/202	21	PR	02RS METF	1356	CAMPO LA	410420	HOSPITAL I
12/07/202	28	01/07/202	26	SP	GVE XVII C.	1342	CAMPINAS	350950	MATERNID
03/05/202	18	25/04/202	17	GO	ESTRADA D	1802	CATALAO	520510	HOSPITAL I
19/04/202	16	16/04/202	15	SP	GVE XVII C.	1342	CAMPINAS	350950	MATERNID
04/08/202	31	25/05/202	21	PR	09RS FOZ D	1363	FOZ DO IGU	410830	HOSPITAL I
30/04/202	17	18/04/202	16	SP	GVE I CAPI	1331	SAO PAULC	355030	HC DA FMU
02/07/202	26	25/06/202	25	SP	GVE XXI PR	1346	PRESIDENT	354140	HOSPITAL I
01/07/202	26	23/06/202	25	PR	02RS METF	1356	CAMPINA (	410400	HOSPITAL /
08/09/202	36	01/08/202	31	RJ			RIO DE JAN	330455	HOSPITAL I

29/03/202	13	22/03/202	12	PE	001	1497	RECIFE	261160	IMIP
08/04/202	14	30/03/202	13	RS	002 CRS	1607	PORTO ALE	431490	HOSPITAL
03/06/202	22	31/05/202	22	RJ			MARICA	330270	HOSPITAL
09/02/202	6	30/01/202	4	RO	VI GRS POR	1483	PORTO VEL	110020	HOSPITAL
11/05/202	19	23/03/202	12	BA	NUCLEO RE	1380	SALVADOR	292740	HOSPITAL
28/04/202	17	20/04/202	16	SP	GVE XXIX S	1354	SAO JOSE D	354980	HOSPITAL
12/04/202	15	31/03/202	13	SP	GVE I CAPIT	1331	SAO PAULO	355030	HC DA FMU
03/05/202	18	23/04/202	16	PR	02RS METR	1356	CURITIBA	410690	COMPLEXO
14/04/202	15	14/04/202	15	SP	GVE XXIX S	1354	SAO JOSE D	354980	HOSPITAL
09/06/202	23	01/06/202	22	SP	GVE XXIX S	1354	SAO JOSE D	354980	SANTA CAS
04/06/202	22	27/05/202	21	SP	GVE I CAPIT	1331	SAO PAULO	355030	PRO MATR
02/06/202	22	20/05/202	20	CE	11 CRES SO	1521	SOBRAL	231290	HOSPITAL
08/04/202	14	02/04/202	13	MG	MONTES C	1473	JANAUBA	313510	FUNDAJAN
25/06/202	25	23/06/202	25	RJ			DUQUE DE	330170	HOSPITAL
31/05/202	22	20/05/202	20	SP	GVE XXIV R	1348	RIBEIRAO P	354340	HOSPITAL
08/05/202	18	28/04/202	17	MG	MONTES C	1473	JANAUBA	313510	FUNDAJAN
13/05/202	19	04/05/202	18	SP	GVE I CAPIT	1331	SAO PAULO	355030	HOSP SAO
20/01/202	3	15/01/202	2	GO	CENTRAL	1779	GOIANIA	520870	HOSPITAL
10/07/202	27	01/07/202	26	SP	GVE XXX JA	1574	JALES	352480	SANTA CAS
02/06/202	22	17/05/202	20	RN	VII URSAP	1416	NATAL	240810	HOSPITAL
29/03/202	13	25/03/202	12	PA	01 REGION	1484	BELEM	150140	SANTA CAS
05/04/202	14	20/03/202	11	DF			BRASILIA	530010	HOSPITAL
10/05/202	19	10/05/202	19	CE	1 CRES FOR	1519	FORTALEZA	230440	HGCC HOS
25/05/202	21	13/05/202	19	GO	CENTRAL	1779	GOIANIA	520870	HOSPITAL
14/06/202	24	03/06/202	22	PR	03RS PONT	1357	PONTA GR	411990	HOSPITAL
30/03/202	13	21/03/202	12	SP	GVE XXIX S	1354	CATANDUV	351110	HOSPITAL
01/03/202	9	20/02/202	7	BA	NUCLEO RE	1380	SALVADOR	292740	MATERNID
12/05/202	19	28/04/202	17	MS	NRS DE DO	1966	DOURADOS	500370	EBSERH HC
08/06/202	23	26/05/202	21	SP	GVE I CAPIT	1331	SAO PAULO	355030	MATERNID
16/04/202	15	04/04/202	14	SP	GVE I CAPIT	1331	SAO PAULO	355030	HC DA FMU
18/05/202	20	05/05/202	18	SP	GVE I CAPIT	1331	SAO PAULO	355030	SAO LUIZ U
29/03/202	13	20/03/202	11	CE	1 CRES FOR	1519	FORTALEZA	230440	HGCC HOS
21/09/202	38	07/09/202	36	PR	10RS CASC	1364	CASCAVEL	410480	UPA TANC
20/03/202	11	20/03/202	11	CE	1 CRES FOR	1519	FORTALEZA	230440	HGCC HOS
21/05/202	20	13/05/202	19	AM	ENTORNO	5584	MANAUS	130260	MATERNID
06/06/202	23	30/05/202	22	SP	GVE XXXI S	1353	SOROCABA	355220	UPA UNIDA
27/09/202	39	24/09/202	38	SP	GVE XVII CA	1342	CAMPINAS	350950	MATERNID
19/03/202	11	18/03/202	11	SP	GVE X OSA	1335	OSASCO	353440	HOSPITAL
29/09/202	39	08/08/202	32	GO	CENTRAL	1779	GOIANIA	520870	HOSPITAL
12/03/202	10	21/02/202	8	DF			BRASILIA	530010	HRAN
11/04/202	15	29/03/202	13	DF			BRASILIA	530010	HOSPITAL
15/01/202	2	05/01/202	1	SP	GVE XXVII	1351	SAO JOSE D	354990	HOSPITAL
04/05/202	18	23/04/202	16	MT	CUIABA	1578	CUIABA	510340	HOSPITAL
14/06/202	24	05/06/202	22	AL	9 MICRORE	1541	PALMEIRA	270630	HOSPITAL
18/05/202	20	11/05/202	19	SP	GVE XXXI S	1353	SOROCABA	355220	HOSPITAL
23/11/202	47	18/11/202	46	GO	CENTRAL	1779	GOIANIA	520870	HOSPITAL
05/07/202	27	23/06/202	25	BA	NUCLEO RE	1380	SALVADOR	292740	MATERNID

05/05/202	18	27/04/202	17	MS	EX ARS DE	1975	CAMPO GR	500270	HOSPITAL
10/08/202	32	28/07/202	30	MT	CUIABA	1578	CUIABA	510340	HOSPITAL
01/06/202	22	25/05/202	21	PR	01RS PARA	1355	PARANAGU	411820	HOSPITAL
29/11/202	48	10/11/202	45	MA	REGIONAL	1440	IMPERATRI	210530	HRMI HOS
24/08/202	34	13/08/202	32	MT	CUIABA	1578	CUIABA	510340	HOSPITAL
17/02/202	7	07/02/202	6	RJ			VOLTA RED	330630	SECRETARI
24/02/202	8	05/02/202	5	PR	03RS PONT	1357	PONTA GR	411990	HOSPITAL
27/07/202	30	23/06/202	25	GO	CENTRAL	1779	GOIANIA	520870	HOSPITAL
08/03/202	10	03/03/202	9	SP	GVE I CAPI	1331	SAO PAULO	355030	PRO MATR
22/05/202	20	12/05/202	19	MG	MONTES C	1473	MONTES C	314330	HOSPITAL
03/10/202	40	29/09/202	39	PR	02RS METR	1356	CAMPO LA	410420	HOSPITAL
15/04/202	15	28/03/202	13	MA	REGI0NAL	1430	SAO LUIS	211130	MATERNID
04/06/202	22	13/05/202	19	ES	METROPOL	1510	SERRA	320500	HOSPITAL
28/01/202	4	14/01/202	2	SP	GVE I CAPI	1331	SAO PAULO	355030	MATERNID
07/05/202	18	27/04/202	17	SP	GVE XVI BO	1341	BOTUCATU	350750	HOSPITAL
26/05/202	21	15/05/202	19	PA	09 REGION	1492	SANTAREM	150680	HOSPITAL
08/04/202	14	28/03/202	13	SP	GVE XXIV R	1348	RIBEIRAO P	354340	HOSPITAL
18/06/202	24	08/06/202	23	MT	CUIABA	1578	CUIABA	510340	HOSPITAL
31/05/202	22	17/05/202	20	RS	002 CRS	1607	PORTO ALE	431490	HOSPITAL
01/04/202	13	01/04/202	13	CE	1 CRES FOR	1519	FORTALEZA	230440	HGCC HOS
14/05/202	19	13/05/202	19	PE	001	1497	RECIFE	261160	IMIP
17/04/202	15	04/04/202	14	MS	EX ARS DE	1975	CAMPO GR	500270	HOSPITAL
19/05/202	20	19/05/202	20	CE	1 CRES FOR	1519	FORTALEZA	230440	HGCC HOS
01/06/202	22	21/05/202	20	MG	UBA	1464	MURIAE	314390	CASE DE C
19/08/202	33	05/08/202	31	PI	4 GRS TERE	1883	TERESINA	221100	MATERNID
21/09/202	38	06/09/202	36	PR	02RS METR	1356	CAMPINA	410400	HOSPITAL
14/05/202	19	09/05/202	19	RJ			CAMPOS D	330100	HOSPITAL
17/05/202	20	09/05/202	19	RS	004 CRS	1595	SANTA MA	431690	HUSM HOS
28/06/202	26	18/06/202	24	TO			PALMAS	172100	HOSPITAL
13/06/202	24	10/06/202	23	SP	GVE VIII M	1333	MOGI DAS	353060	HOSPITAL
19/01/202	3	11/01/202	2	SC	CHAPECO	1553	CHAPECO	420420	HOSPITAL
01/03/202	9	25/02/202	8	MT	CUIABA	1578	CUIABA	510340	HOSPITAL
07/03/202	10	01/03/202	9	AM	ENTORNO	5584	MANAUS	130260	HOSPITAL
28/03/202	13	23/03/202	12	RJ			RIO DE JAN	330455	MS HSE HO
10/05/202	19	01/05/202	17	SP	GVE XVIII F	1343	FRANCA	351620	SANTA CAS
20/05/202	20	12/05/202	19	SP	GVE XXXI S	1353	SOROCABA	355220	HOSPITAL
17/06/202	24	17/06/202	24	CE	1 CRES FOR	1519	FORTALEZA	230440	HGCC HOS
28/05/202	21	23/05/202	21	SP	GVE X OSA	1335	CARAPICUI	351060	HOSPITAL
25/04/202	17	22/04/202	16	SP	GVE VIII M	1333	GUARULHO	351880	HOSPITAL
25/08/202	34	17/08/202	33	SP	GVE I CAPI	1331	SAO PAULO	355030	SAO LUIZ U
16/08/202	33	10/08/202	32	PR	09RS FOZ D	1363	FOZ DO IGU	410830	HOSPITAL
16/07/202	28	07/07/202	27	RJ			CAMPOS D	330100	HOSPITAL
09/03/202	10	08/03/202	10	GO	CENTRAL	1779	GOIANIA	520870	HOSPITAL
20/05/202	20	11/05/202	19	RJ			RIO DE JAN	330455	HOSPITAL
17/03/202	11	14/03/202	11	PE	001	1497	RECIFE	261160	IMIP
20/03/202	11	11/03/202	10	CE	15 CRES CR	1525	CRATEUS	230410	HOSPITAL
29/04/202	17	13/04/202	15	MA	REGI0NAL	1430	SAO LUIS	211130	MATERNID

03/05/202	18	24/04/202	16	GO	CENTRAL	1779	GOIANIA	520870	HOSPITAL I
22/06/202	25	05/06/202	22	PE	001	1497	RECIFE	261160	REAL HOSP
04/03/202	9	27/02/202	8	CE	1 CRES FOF	1519	FORTALEZ/	230440	HOSPITAL (
24/05/202	21	20/05/202	20	GO	CENTRAL	1779	GOIANIA	520870	HOSPITAL I
08/09/202	36	03/09/202	35	RJ			CAMPOS D	330100	HOSPITAL I
26/07/202	30	16/07/202	28	BA	NUCLEO RI	1380	SALVADOR	292740	MATERNID
18/11/202	46	04/11/202	44	CE	1 CRES FOF	1519	FORTALEZ/	230440	MATERNID
16/05/202	20	16/05/202	20	PR	02RS METF	1356	CURITIBA	410690	CENTRO H(
01/12/202	48	18/11/202	46	SP	GVE XXVIII	1576	CARAGUAT	351050	CASA DE S/
02/10/202	39	26/09/202	39	SC	JOINVILLE	1565	JOINVILLE	420910	HOSPITAL I
13/04/202	15	10/04/202	14	RJ			VOLTA REC	330630	SECRETARI
16/04/202	15	07/04/202	14	SP	GVE I CAPI	1331	SAO PAULC	355030	MATERNID
21/04/202	16	20/04/202	16	CE	1 CRES FOF	1519	FORTALEZ/	230440	HGCC HOS
29/03/202	13	14/03/202	11	SP	GVE XXIX S	1354	SAO JOSE D	354980	HOSPITAL I
04/06/202	22	25/05/202	21	RS	019 CRS	1606	TENENTE P	432140	HOSPITAL S
10/03/202	10	04/03/202	9	ES	METROPOI	1510	SERRA	320500	HOSPITAL I
28/06/202	26	16/06/202	24	SP	GVE XXIX S	1354	CATANDUV	351110	UNIMED H
16/05/202	20	17/04/202	15	ES	METROPOI	1510	SERRA	320500	HOSPITAL I
07/06/202	23	02/06/202	22	PR	17RS LONE	1371	LONDRINA	411370	HOSPITAL I
04/06/202	22	23/05/202	21	DF			BRASILIA	530010	HRAN
25/05/202	21	13/05/202	19	SP	GVE XXVI S	1350	MOGI GUA	353070	SANTA CAS
08/01/202	1	05/01/202	1	MS	NRS DE TRI	6255	TRES LAGO	500830	HOSPITAL I
02/03/202	9	22/02/202	8	MG	CORONEL I	1468	TIMOTEO	316870	HOSPITAL I
25/02/202	8	19/02/202	7	SP	GVE I CAPI	1331	SAO PAULC	355030	HOSPITAL I
02/03/202	9	22/02/202	8	MG	ALFENAS	1379	ALFENAS	310160	SANTA CAS
16/03/202	11	04/03/202	9	MT	CUIABA	1578	CUIABA	510340	HOSPITAL U
23/04/202	16	19/03/202	11	SP	GVE VIII M	1333	MOGI DAS	353060	MOGI MAT
28/04/202	17	17/04/202	15	SP	GVE I CAPI	1331	SAO PAULC	355030	HC DA FMU
06/05/202	18	24/04/202	16	RN	VII URSAP	1416	NATAL	240810	HOSPITAL /
29/04/202	17	15/04/202	15	MT	CUIABA	1578	CUIABA	510340	HOSPITAL U
04/05/202	18	17/04/202	15	MG	BELO HORI	1449	BELO HORI	310620	SANTA CAS
26/10/202	43	19/10/202	42	SP	GVE XI ARA	1336	PENAPOLIS	353730	SANTA CAS
07/07/202	27	28/06/202	26	PA	01 REGION	1484	BELEM	150140	SANTA CAS
25/03/202	12	25/03/202	12	CE	1 CRES FOF	1519	FORTALEZ/	230440	HGCC HOS
31/03/202	13	12/03/202	10	MT	CUIABA	1578	CUIABA	510340	HOSPITAL U
23/03/202	12	11/03/202	10	SP	GVE I CAPI	1331	SAO PAULC	355030	HOSP MUN
19/04/202	16	18/04/202	16	RJ			DUQUE DE	330170	HOSPITAL (
22/02/202	8	17/02/202	7	AM	ENTORNO	5584	MANAUS	130260	HOSPITAL /
17/06/202	24	26/05/202	21	PA	01 REGION	1484	BELEM	150140	SANTA CAS
12/07/202	28	01/07/202	26	GO	CENTRAL	1779	GOIANIA	520870	HOSPITAL I
28/05/202	21	20/05/202	20	SP	GVE I CAPI	1331	SAO PAULC	355030	HOSPITAL S
25/10/202	43	12/09/202	37	SP	GVE I CAPI	1331	SAO PAULC	355030	HOSPITAL S
06/01/202	1	30/12/202	52	SP	GVE VIII M	1333	GUARULHC	351880	HOSPITAL I
09/03/202	10	27/02/202	8	SP	GVE XXIX S	1354	SAO JOSE D	354980	HOSPITAL I
22/03/202	12	18/03/202	11	SP	GVE XXIX S	1354	VOTUPOR/	355710	SANTA CAS
31/03/202	13	19/03/202	11	RJ			RIO DE JAN	330455	HOSPITAL I
25/08/202	34	11/08/202	32	SP	GVE XXI PR	1346	PRESIDENT	354140	HOSPITAL I

11/04/202	15	04/04/202	14	MG	SETE LAGO	1467	SETE LAGO	316720	HOSPITAL I
11/05/202	19	06/05/202	18	PB	I NRS JOAC	1377	JOAO PESS	250750	MATERNID
17/06/202	24	07/06/202	23	PR	17RS LOND	1371	LONDRINA	411370	HOSPITAL U
29/03/202	13	25/03/202	12	PB	III NRS CAM	1421	CAMPINA (	250400	CLINICA SA
18/05/202	20	04/05/202	18	RJ			RIO DE JAN	330455	HOSPITAL I
19/05/202	20	30/04/202	17	SP	GVE XXV S/	1349	GUARUJA	351870	HOSPITAL S
15/04/202	15	07/04/202	14	BA	NUCLEO RI	1380	SANTO AN'	292870	HOSPITAL I
22/03/202	12	06/03/202	9	RS	001 CRS	1592	SAO LEOPC	431870	HOSPITAL (
15/01/202	2	04/01/202	1	PI	4 GRS TERE	1883	TERESINA	221100	HOSPITAL U
13/04/202	15	07/04/202	14	AC	REGIONAL	1941	CRUZEIRO	120020	HOSPITAL I
27/01/202	4	28/12/202	52	SP	GVE I CAPI'	1331	SAO PAULC	355030	SAO LUIZ U
02/06/202	22	25/05/202	21	RS	002 CRS	1607	PORTO ALE	431490	HOSPITAL I
03/06/202	22	01/06/202	22	SP	GVE I CAPI'	1331	SAO PAULC	355030	HOSP MUN
16/04/202	15	08/04/202	14	SP	GVE I CAPI'	1331	SAO PAULC	355030	HOSP MUN
19/03/202	11	07/03/202	10	SP	GVE I CAPI'	1331	SAO PAULC	355030	HC DA FMU
16/07/202	28	07/07/202	27	SP	GVE XXIX S	1354	CATANDUV	351110	HOSPITAL I
01/06/202	22	23/05/202	21	MA	REGI0NAL	1430	SAO LUIS	211130	MATERNID
14/04/202	15	02/04/202	13	SP	GVE I CAPI'	1331	SAO PAULC	355030	HOSPITAL V
24/05/202	21	10/05/202	19	PB	I NRS JOAC	1377	JOAO PESS	250750	HOSPITAL (
25/05/202	21	18/05/202	20	BA	NUCLEO RI	1380	SALVADOR	292740	MATERNID
26/07/202	30	23/07/202	29	RO	VI GRS POF	1483	PORTO VEL	110020	HOSPITAL I
09/02/202	6	27/01/202	4	SP	GVE I CAPI'	1331	SAO PAULC	355030	HC DA FMU
30/12/202	52	27/12/202	52	PR	02RS METF	1356	CURITIBA	410690	HOSPITAL U
05/03/202	9	01/03/202	9	SP	GVE I CAPI'	1331	SAO PAULC	355030	HOSPITAL I
09/03/202	10	06/03/202	9	MT	CUIABA	1578	CUIABA	510340	HOSPITAL S
20/03/202	11	12/03/202	10	MG	MONTES C	1473	MONTES C	314330	HOSPITAL U
28/05/202	21	15/05/202	19	RN	VII URSAP	1416	NATAL	240810	HOSPITAL I
04/03/202	9	23/02/202	8	CE	1 CRES FOF	1519	FORTALEZ/	230440	HOSPITAL I
09/04/202	14	13/03/202	10	MG	PONTE NO'	1458	PONTE NO'	315210	HOSPITAL I
20/04/202	16	10/04/202	14	MT	SINOP	1584	SINOP	510790	HOSPITAL S
23/03/202	12	13/03/202	10	GO	CENTRAL	1779	GOIANIA	520870	HOSPITAL I
28/08/202	34	17/08/202	33	BA	NUCLEO RI	1398	GUANAMB	291170	HOSPITAL I
15/03/202	11	05/03/202	9	MG	POUSO ALI	1459	POUSO ALI	315250	HOSPITAL I
02/06/202	22	28/05/202	21	SP	GVE I CAPI'	1331	SAO PAULC	355030	HOSPITAL S
08/07/202	27	30/06/202	26	MS	EX ARS DE	1975	CAMPO GF	500270	HOSPITAL I
20/01/202	3	18/01/202	3	RJ			RIO DE JAN	330455	MS HSE HC
19/06/202	24	13/06/202	24	PR	10RS CASC	1364	CASCAVEL	410480	HOSPITAL U
26/07/202	30	18/07/202	29	PR	10RS CASC	1364	CASCAVEL	410480	HOSPITAL U
05/04/202	14	19/03/202	11	SP	GVE I CAPI'	1331	SAO PAULC	355030	HC DA FMU
06/04/202	14	20/03/202	11	SP	GVE XXVII S	1351	JACAREI	352440	HOSPITAL S
28/05/202	21	23/05/202	21	PB	I NRS JOAC	1377	JOAO PESS	250750	MATERNID
09/03/202	10	02/03/202	9	SP	GVE I CAPI'	1331	SAO PAULC	355030	HC DA FMU
21/06/202	25	20/06/202	25	SP	GVE X OSA	1335	OSASCO	353440	HOSPITAL (
13/06/202	24	03/06/202	22	SP	GVE XVII C/	1342	VALINHOS	355620	HOSPITAL I
22/04/202	16	20/03/202	11	SP	GVE I CAPI'	1331	SAO PAULC	355030	SAO LUIZ U
03/05/202	18	23/04/202	16	SP	GVE I CAPI'	1331	SAO PAULC	355030	HOSPITAL I
07/05/202	18	26/04/202	17	SP	GVE VIII M	1333	ITAQUAQU	352310	HOSPITAL (

06/09/202	36	05/09/202	36	PR	10RS CASC	1364	CASCAVEL	410480	HOSPITAL U
06/08/202	31	25/07/202	30	GO	CENTRAL	1779	GOIANIA	520870	HOSPITAL I
12/01/202	2	04/01/202	1	AM	ENTORNO	5584	MANAUS	130260	MATERNID
15/02/202	7	06/02/202	5	PR	15RS MARI	1369	MARINGA	411520	SANTA CAS
16/02/202	7	09/02/202	6	PB	I NRS JOAC	1377	JOAO PESS	250750	MATERNID
03/03/202	9	01/03/202	9	MG	POUSO ALI	1459	EXTREMA	312510	HOSPITAL I
08/03/202	10	17/02/202	7	RJ			RIO DE JAN	330455	HOSPITAL I
30/03/202	13	19/02/202	7	CE	1 CRES FOF	1519	FORTALEZA	230440	HOSPITAL (
05/04/202	14	13/03/202	10	MG	JUIZ DE FO	1452	JUIZ DE FO	313670	SANTA CAS
21/04/202	16	06/04/202	14	SP	GVE XIV BA	1339	BARRETOS	350550	SANTA CAS
08/07/202	27	30/06/202	26	SP	GVE VIII M	1333	ITAQUAQU	352310	HOSPITAL (
26/05/202	21	20/05/202	20	SC	BLUMENAU	1564	GASPAR	420590	HOSPITAL I
29/06/202	26	13/06/202	24	SP	GVE XII ARA	1337	ARARAQUA	350320	HOSPITAL S
06/06/202	23	05/06/202	22	SP	GVE VII SAI	1332	RIBEIRAO F	354330	HOSPITAL I
11/08/202	32	05/08/202	31	RJ			ANGRA DO	330010	HOSPITAL I
04/03/202	9	22/02/202	8	SE	REGIONAL	2056	ARACAJU	280030	HOSPITAL I
05/10/202	40	23/09/202	38	RJ			RIO DE JAN	330455	MS HSE HC
26/04/202	17	20/04/202	16	BA	NUCLEO RI	1381	FEIRA DE S.	291080	HOSPITAL I
11/03/202	10	05/03/202	9	SP	GVE XVII C.	1342	CAMPINAS	350950	MATERNID
10/05/202	19	30/04/202	17	RJ			VOLTA RED	330630	SECRETARI
11/06/202	23	26/05/202	21	PR	14RS PARA	1368	PARANAVA	411840	SANTA CAS
05/06/202	22	28/05/202	21	MA	REGI0NAL	1430	SAO LUIS	211130	MATERNID
21/05/202	20	19/05/202	20	PR	17RS LOND	1371	LONDRINA	411370	HOSPITAL I
16/04/202	15	17/03/202	11	SP	GVE I CAPI	1331	SAO PAULO	355030	SAO LUIZ U
10/06/202	23	04/06/202	22	PR	02RS METF	1356	CAMPINA (	410400	HOSPITAL A
12/05/202	19	07/05/202	18	RJ			NILOPOLIS	330320	DOMINGO.
16/03/202	11	08/03/202	10	MT	CUIABA	1578	CUIABA	510340	HOSPITAL S
11/02/202	6	02/02/202	5	SP	GVE XXIX S	1354	SAO JOSE D	354980	HOSPITAL I
27/10/202	43	21/10/202	42	SP	GVE XI ARA	1336	PENAPOLIS	353730	SANTA CAS
02/01/202	1	30/12/202	52	PB	I NRS JOAC	1377	JOAO PESS	250750	MATERNID
27/04/202	17	16/04/202	15	MG	BELO HORI	1449	CONTAGEN	311860	HOSPITAL I
24/03/202	12	15/03/202	11	SC	JOINVILLE	1565	JOINVILLE	420910	HOSPITAL I
22/03/202	12	17/03/202	11	SP	GVE XXV SA	1349	SANTOS	354850	COMPLEXC
26/03/202	12	16/03/202	11	SP	GVE I CAPI	1331	SAO PAULO	355030	HC DA FMU
19/08/202	33	14/08/202	32	SC	JARAGUA D	1567	JARAGUA D	420890	HOSPITAL J
14/04/202	15	02/04/202	13	SP	GVE I CAPI	1331	SAO PAULO	355030	SAO LUIZ U
12/04/202	15	31/03/202	13	PA	01 REGION	1484	BELEM	150140	MATERNID
11/08/202	32	05/08/202	31	RJ			RIO DE JAN	330455	INI FIOCRU
18/03/202	11	13/03/202	10	RO	VI GRS POF	1483	PORTO VEL	110020	HOSPITAL I
27/01/202	4	24/01/202	4	MG	BELO HORI	1449	BETIM	310670	HOSPITAL I
23/02/202	8	15/02/202	7	PR	02RS METF	1356	CURITIBA	410690	COMPLEXC
03/01/202	1	01/01/202	52	SP	GVE I CAPI	1331	SAO PAULO	355030	HOSPITAL (
24/09/202	38	21/09/202	38	GO	CENTRAL	1779	GOIANIA	520870	HOSPITAL I
17/03/202	11	06/03/202	9	SP	GVE XIX M.	1344	MARILIA	352900	HOSPITAL U
20/05/202	20	10/05/202	19	SP	GVE XXXI S	1353	SOROCABA	355220	UPH LESTE
04/04/202	14	28/03/202	13	SP	GVE I CAPI	1331	SAO PAULO	355030	HOSPITAL S
08/02/202	6	01/02/202	5	GO	CENTRAL	1779	GOIANIA	520870	HOSPITAL I

05/05/202	18	30/04/202	17	PI	10 GRS FLC	1889	FLORIANO	220390	HOSPITAL I
30/04/202	17	19/04/202	16	GO	CENTRAL	1779	GOIANIA	520870	SAU I SERV
11/08/202	32	08/08/202	32	RJ			ITAPERUN/	330220	HOSPITAL S
27/08/202	34	17/08/202	33	SP	GVE I CAPI	1331	SAO PAUL(	355030	MATERNID
21/03/202	12	14/03/202	11	SP	GVE XIX M/	1344	MARILIA	352900	HOSPITAL I
01/07/202	26	16/06/202	24	SP	GVE I CAPI	1331	SAO PAUL(	355030	HOSPITAL S
13/10/202	41	01/10/202	39	SP	GVE VIII M	1333	GUARULH(	351880	HOSPITAL (
06/04/202	14	31/03/202	13	PB	I NRS JOAC	1377	JOAO PESS	250750	MATERNID
18/02/202	7	27/01/202	4	CE	1 CRES FOF	1519	FORTALEZ/	230440	HOSPITAL /
23/03/202	12	18/03/202	11	CE	1 CRES FOF	1519	FORTALEZ/	230440	HGCC HOS
23/04/202	16	09/04/202	14	SP	GVE I CAPI	1331	SAO PAUL(	355030	HC DA FMU
15/03/202	11	07/03/202	10	RJ			RIO DE JAN	330455	MS HSE HC
16/12/202	50	12/12/202	50	SP	GVE XX PIR	1345	ARARAS	350330	HOSPITAL S
03/05/202	18	20/04/202	16	RS	002 CRS	1607	PORTO ALE	431490	HOSPITAL I
21/05/202	20	19/05/202	20	RS	001 CRS	1592	NOVO HAN	431340	HOSPITAL I
25/05/202	21	16/05/202	20	MA	REGIONAL	1440	IMPERATRI	210530	HRMI HOSI
30/03/202	13	20/03/202	11	SP	GVE I CAPI	1331	SAO PAUL(	355030	HC DA FMU
01/02/202	5	23/01/202	3	BA	NUCLEO RI	1380	SALVADOR	292740	HOSPITAL I
11/04/202	15	01/04/202	13	DF			BRASILIA	530010	HOSPITAL S
14/06/202	24	01/06/202	22	SP	GVE I CAPI	1331	SAO PAUL(	355030	HC DA FMU
24/05/202	21	14/05/202	19	SP	GVE I CAPI	1331	SAO PAUL(	355030	MATERNID
18/07/202	29	14/07/202	28	RR			BOA VISTA	140010	HOSPITAL (
19/03/202	11	10/03/202	10	BA	NUCLEO RI	1380	SALVADOR	292740	HOSPITAL /
16/04/202	15	13/03/202	10	SP	GVE I CAPI	1331	SAO PAUL(	355030	MATERNID
04/06/202	22	27/05/202	21	RS	001 CRS	1592	PAROBE	431405	HOSPITAL S
12/06/202	23	05/06/202	22	MG	DIAMANTI	1450	DIAMANTI	312160	SANTA CAS
12/05/202	19	04/05/202	18	PI	4 GRS TERE	1883	TERESINA	221100	MATERNID
11/08/202	32	29/07/202	30	SP	GVE I CAPI	1331	SAO PAUL(	355030	MATERNID
30/01/202	4	27/01/202	4	PE	001	1497	RECIFE	261160	IMIP
18/05/202	20	13/05/202	19	SP	GVE XXI PR	1346	PRESIDENT	354140	HOSPITAL I
10/06/202	23	01/06/202	22	MG	DIVINOPOI	1472	DIVINOPOI	312230	HOSPITAL S
11/06/202	23	30/05/202	22	MA	REGI0NAL	1430	SAO LUIS	211130	MATERNID
29/04/202	17	16/04/202	15	SP	GVE XVI BC	1341	LARANJAL	352640	SANTA CAS
10/06/202	23	07/06/202	23	SP	GVE XX PIR	1345	PIRACICAB	353870	SANTA CAS
18/02/202	7	04/02/202	5	AM	ENTORNO	5584	MANAUS	130260	INSTITUTO
06/03/202	9	24/02/202	8	GO	CENTRAL	1779	GOIANIA	520870	HOSPITAL /
24/09/202	38	22/09/202	38	SP	GVE I CAPI	1331	SAO PAUL(	355030	SAO LUIZ U
29/03/202	13	23/03/202	12	SP	GVE XXI PR	1346	PRESIDENT	354140	HOSPITAL I
07/06/202	23	02/05/202	18	RN	VII URSAP	1416	NATAL	240810	HOSPITAL I
08/09/202	36	29/08/202	35	RJ			CAMPOS D	330100	HOSPITAL I
27/09/202	39	21/09/202	38	MG	BELO HORI	1449	BELO HORI	310620	HOSPITAL I
28/06/202	26	19/06/202	24	PR	10RS CASC	1364	CASCAVEL	410480	HOSPITAL U
09/06/202	23	28/05/202	21	MG	BELO HORI	1449	NOVA LIM/	314480	NOVA LIM/
06/08/202	31	30/07/202	30	MG	BELO HORI	1449	CONTAGEN	311860	HOSPITAL I
12/03/202	10	03/03/202	9	PR	20RS TOLEI	1374	TOLEDO	412770	HOESP
04/02/202	5	29/01/202	4	SP	GVE XVII C/	1342	ATIBAIA	350410	HOSPITAL I
02/09/202	35	23/08/202	34	GO	ESTRADA D	1802	MARZAGA(	521290	HOSPITAL I

12/03/202	10	01/03/202	9	RJ			RIO DE JAN	330455	SMS HOSPI
27/04/202	17	22/04/202	16	MG	UBERLAND	1462	UBERLAND	317020	UBERLAND
28/09/202	39	25/09/202	38	SP	GVE XXXI S	1353	SOROCABA	355220	CONJUNTO
23/04/202	16	23/04/202	16	CE	1 CRES FOF	1519	FORTALEZA	230440	HGCC HOS
15/05/202	19	14/05/202	19	SP	GVE I CAPI	1331	SAO PAULO	355030	HOSP SAO
28/05/202	21	11/05/202	19	SP	GVE I CAPI	1331	SAO PAULO	355030	MATERNID
19/07/202	29	07/07/202	27	GO	CENTRAL	1779	GOIANIA	520870	HOSPITAL I
14/06/202	24	07/06/202	23	SP	GVE I CAPI	1331	SAO PAULO	355030	HC DA FMU
08/04/202	14	23/03/202	12	SP	GVE I CAPI	1331	SAO PAULO	355030	HC DA FMU
12/07/202	28	28/06/202	26	PR	17RS LOND	1371	LONDRINA	411370	HOSPITAL U
02/05/202	18	27/04/202	17	PR	10RS CASC	1364	CASCAVEL	410480	HOSPITAL U
30/11/202	48	23/11/202	47	PR	02RS METF	1356	CURITIBA	410690	COMPLEXO
23/07/202	29	16/07/202	28	SP	GVE XI ARA	1336	PENAPOLIS	353730	SANTA CAS
26/01/202	4	18/01/202	3	MT	CUIABA	1578	CUIABA	510340	HOSPITAL U
22/10/202	42	23/09/202	38	GO	CENTRAL	1779	GOIANIA	520870	HOSPITAL I
09/04/202	14	07/03/202	10	SP	GVE I CAPI	1331	SAO PAULO	355030	HOSP MUN
05/05/202	18	01/05/202	17	PE	001	1497	RECIFE	261160	IMIP
10/03/202	10	02/03/202	9	MS	NRS DE DO	1966	DOURADOS	500370	HOSPITAL S
16/05/202	20	10/05/202	19	RJ			VALENCA	330610	HOSPITAL I
19/03/202	11	07/03/202	10	SP	GVE I CAPI	1331	SAO PAULO	355030	HC DA FMU
05/06/202	22	29/05/202	21	MA	REGIONAL	1440	IMPERATRI	210530	HRMI HOSI
09/04/202	14	02/04/202	13	CE	1 CRES FOF	1519	FORTALEZA	230440	HOSPITAL I
07/04/202	14	31/03/202	13	SP	GVE XXIX S	1354	SAO JOSE D	354980	HOSPITAL I
03/05/202	18	18/04/202	16	GO	CENTRAL	1779	GOIANIA	520870	HOSPITAL I
29/03/202	13	21/03/202	12	SP	GVE VIII M	1333	GUARULHO	351880	HOSPITAL I
24/03/202	12	16/03/202	11	SP	GVE X OSA	1335	OSASCO	353440	HOSPITAL I
26/03/202	12	16/03/202	11	SP	GVE I CAPI	1331	SAO PAULO	355030	MATERNID
23/02/202	8	10/02/202	6	MG	PATOS DE I	1456	PATOS DE I	314800	HOSPITAL I
21/03/202	12	11/03/202	10	PB	I NRS JOAO	1377	JOAO PESS	250750	MATERNID
25/05/202	21	23/05/202	21	RS	016 CRS	1610	ESTRELA	430780	HOSPITAL I
29/07/202	30	14/07/202	28	PR	09RS FOZ D	1363	FOZ DO IGU	410830	HOSPITAL I
31/03/202	13	26/03/202	12	AM	ENTORNO	5584	MANAUS	130260	INSTITUTO
31/03/202	13	18/03/202	11	SP	GVE I CAPI	1331	SAO PAULO	355030	HOSPITAL S
19/04/202	16	05/04/202	14	SP	GVE XII AR	1337	SAO CARLO	354890	SANTA CAS
01/04/202	13	19/03/202	11	SP	GVE VII SAI	1332	SAO CAETA	354880	HOSPITAL S
03/04/202	13	28/03/202	13	GO	SUDOESTE	1799	RIO VERDE	521880	HOSPITAL I
06/02/202	5	22/01/202	3	AM	ENTORNO	5584	MANAUS	130260	MATERNID
04/03/202	9	22/02/202	8	PR	02RS METF	1356	CURITIBA	410690	HNSG
24/09/202	38	20/09/202	38	PR	10RS CASC	1364	CASCAVEL	410480	HOSPITAL U
02/07/202	26	28/06/202	26	SP	GVE XXII PI	1573	DRACENA	351440	SANTA CAS
26/04/202	17	11/04/202	15	SP	GVE I CAPI	1331	SAO PAULO	355030	HOSPITAL S
15/06/202	24	09/06/202	23	BA	NUCLEO RI	1388	PORTO SEG	292530	HOSPITAL I
19/07/202	29	08/07/202	27	MT	JUINA	1582	ARIPUANA	510140	HOSPITAL I
26/01/202	4	16/01/202	2	AM	ENTORNO	5584	MANAUS	130260	MATERNID
03/01/202	1	20/12/202	51	RS	002 CRS	1607	PORTO ALE	431490	HOSPITAL I
05/04/202	14	26/03/202	12	SP	GVE I CAPI	1331	SAO PAULO	355030	HOSPITAL I
14/04/202	15	05/04/202	14	SP	GVE I CAPI	1331	SAO PAULO	355030	HC DA FMU

14/06/202	24	30/05/202	22	PB	III NRS CAM	1421	CAMPINA (	250400	INSTITUTO
11/03/202	10	27/02/202	8	DF			BRASILIA	530010	HRL
26/05/202	21	19/05/202	20	SP	GVE XXXI S	1353	SALTO DE F	354530	SANTA CAS
25/01/202	4	22/01/202	3	AM	ENTORNO	5584	MANAUS	130260	SAMEL
11/06/202	23	02/06/202	22	SP	GVE I CAPIT	1331	SAO PAULO	355030	UNIDADE D
25/11/202	47	22/11/202	47	PR	19RS JACAI	1373	SANTO AN	412410	HOSPITAL I
19/05/202	20	10/05/202	19	SP	GVE XXXI S	1353	SALTO	354520	HOSPITAL I
13/03/202	10	28/02/202	9	PA	01 REGION	1484	BELEM	150140	SANTA CAS
12/04/202	15	29/03/202	13	SP	GVE XXIX S	1354	SAO JOSE D	354980	HOSPITAL I
25/06/202	25	19/06/202	24	SP	GVE XV BA	1340	BAURU	350600	PRONTO SO
18/03/202	11	09/03/202	10	MA	REGI0NAL	1430	SAO LUIS	211130	MATERNID
14/06/202	24	04/06/202	22	SP	GVE XIX MA	1344	MARILIA	352900	HOSPITAL U
09/03/202	10	21/02/202	8	GO	PIRINEUS	1792	ANAPOLIS	520110	HOSPITAL I
19/03/202	11	14/03/202	11	PA	01 REGION	1484	BELEM	150140	SANTA CAS
15/05/202	19	08/05/202	18	SP	GVE XXVI S	1350	MOGI GUA	353070	SANTA CAS
26/06/202	25	04/06/202	22	MT	CUIABA	1578	CUIABA	510340	HOSPITAL U
24/05/202	21	12/05/202	19	RN	VII URSAP	1416	NATAL	240810	MATERNID
16/07/202	28	05/07/202	27	SP	GVE XI ARA	1336	BIRIGUI	350650	PRONTO SO
23/04/202	16	13/03/202	10	SP	GVE VIII M	1333	MOGI DAS	353060	MOGI MAT
01/06/202	22	24/05/202	21	GO	CENTRAL	1779	GOIANIA	520870	HOSPITAL I
20/05/202	20	10/05/202	19	MA	REGI0NAL	1430	SAO LUIS	211130	MATERNID
09/06/202	23	31/05/202	22	SP	GVE XXIX S	1354	SAO JOSE D	354980	HOSPITAL I
28/01/202	4	09/01/202	1	SP	GVE I CAPIT	1331	SAO PAULO	355030	MATERNID
24/03/202	12	13/03/202	10	SP	GVE I CAPIT	1331	SAO PAULO	355030	HOSPITAL I
31/03/202	13	20/03/202	11	GO	CENTRAL	1779	GOIANIA	520870	HOSPITAL I
11/05/202	19	02/05/202	18	SP	GVE I CAPIT	1331	SAO PAULO	355030	HC DA FMU
21/06/202	25	11/06/202	23	SP	GVE XVII CA	1342	CAMPINAS	350950	HOSPITAL I
05/07/202	27	23/06/202	25	BA	NUCLEO RE	1380	SALVADOR	292740	MATERNID
13/01/202	2	05/01/202	1	TO			PALMAS	172100	HOSPITAL (
20/02/202	7	13/02/202	6	SP	GVE XVII CA	1342	AMERICAN	350160	HOSPITAL U
10/05/202	19	25/04/202	17	SP	GVE XXXI S	1353	SOROCABA	355220	HOSPITAL U
16/05/202	20	10/05/202	19	SP	GVE XI ARA	1336	VALPARAIS	355630	SANTA CAS
27/04/202	17	19/04/202	16	PR	02RS METR	1356	CURITIBA	410690	HNSG
21/06/202	25	16/06/202	24	SP	GVE I CAPIT	1331	SAO PAULO	355030	HOSPITAL I
12/07/202	28	02/07/202	26	CE	11 CRES SO	1521	SOBRAL	231290	HOSPITAL I
15/07/202	28	08/07/202	27	SC	JOINVILLE	1565	JOINVILLE	420910	HOSPITAL I
26/07/202	30	11/07/202	28	PR	09RS FOZ D	1363	FOZ DO IGU	410830	HOSPITAL I
11/09/202	36	02/09/202	35	PR	20RS TOLEI	1374	MARECHAI	411460	UNIDADE D
22/06/202	25	08/06/202	23	SP	GVE XXV SA	1349	GUARUJA	351870	HOSPITAL S
26/03/202	12	15/03/202	11	RJ			RIO DE JAN	330455	MS HSE HC
21/05/202	20	07/05/202	18	SP	GVE I CAPIT	1331	SAO PAULO	355030	MATERNID
07/06/202	23	23/05/202	21	SP	GVE I CAPIT	1331	SAO PAULO	355030	HC DA FMU
30/06/202	26	19/06/202	24	RS	003 CRS	1593	RIO GRAND	431560	HOSPITAL U
08/06/202	23	02/06/202	22	SP	GVE I CAPIT	1331	SAO PAULO	355030	MATERNID
04/06/202	22	24/05/202	21	BA	NUCLEO RE	1380	SALVADOR	292740	MATERNID
15/04/202	15	05/04/202	14	PE	001	1497	RECIFE	261160	REAL HOSP
31/07/202	30	28/07/202	30	SP	GVE VII SAI	1332	MAUA	352940	UPA BARAO

03/04/202	13	23/03/202	12	CE	1 CRES FOF	1519	FORTALEZ/	230440	HGCC HOS
12/03/202	10	08/03/202	10	MG	BELO HORI	1449	BELO HORI	310620	HOSPITAL I
02/03/202	9	23/02/202	8	RJ			RIO DE JAN	330455	SMS HOSPI
01/03/202	9	21/02/202	8	SC	RIO DO SUI	1478	RIO DO SUI	421480	HOSPITAL I
24/04/202	16	23/03/202	12	RO	VI GRS POF	1483	PORTO VEL	110020	HOSPITAL I
03/05/202	18	26/04/202	17	MG	BELO HORI	1449	BETIM	310670	HOSPITAL I
13/10/202	41	04/10/202	40	PR	10RS CASC	1364	CASCAVEL	410480	HOSPITAL U
28/12/202	52	14/12/202	50	RN	II URSAP	1412	MOSSORO	240800	HOSPITAL I
05/03/202	9	25/02/202	8	PR	17RS LOND	1371	LONDRINA	411370	HOSPITAL U
21/03/202	12	14/03/202	11	SC	CHAPECO	1553	CHAPECO	420420	HOSPITAL I
29/03/202	13	25/03/202	12	SC	CRICIUMA	1558	CRICIUMA	420460	HOSPITAL U
10/05/202	19	01/05/202	17	RS	004 CRS	1595	SANTA MA	431690	HUSM HOS
24/06/202	25	19/06/202	24	SP	GVE XIII AS	1338	SANTA CRU	354640	SANTA CAS
27/07/202	30	18/06/202	24	GO	CENTRAL	1779	GOIANIA	520870	HOSPITAL I
08/06/202	23	28/05/202	21	PR	10RS CASC	1364	CASCAVEL	410480	HOSPITAL U
29/07/202	30	16/07/202	28	MT	CUIABA	1578	CUIABA	510340	HOSPITAL U
14/05/202	19	14/05/202	19	CE	1 CRES FOF	1519	FORTALEZ/	230440	HGCC HOS
12/09/202	37	19/08/202	33	MG	UNAI	1463	PARACATU	314700	HOSPITAL I
02/12/202	48	28/11/202	48	RJ			RIO DE JAN	330455	MS HSE HC
16/01/202	2	13/01/202	2	MG	MONTES C	1473	MONTES C	314330	HOSPITAL U
07/04/202	14	31/03/202	13	SP	GVE I CAPI	1331	SAO PAULC	355030	HOSP MUN
18/04/202	16	06/04/202	14	MS	EX ARS DE	1975	CAMPO GF	500270	HOSPITAL (
14/06/202	24	01/06/202	22	SP	GVE VII SAI	1332	SANTO AN	354780	HOSPITAL I
02/08/202	31	26/07/202	30	SC	XANXERE	1556	XANXERE	421950	HOSPITAL I
30/03/202	13	25/03/202	12	SP	GVE I CAPI	1331	SAO PAULC	355030	HOSPITAL /
26/03/202	12	18/03/202	11	SP	GVE I CAPI	1331	SAO PAULC	355030	HOSPITAL S
03/05/202	18	26/04/202	17	PB	III NRS CAN	1421	CAMPINA (	250400	HOSPITAL I
06/05/202	18	02/05/202	18	PR	02RS METF	1356	CURITIBA	410690	HOSPITAL U
04/05/202	18	27/03/202	12	GO	CENTRAL	1779	GOIANIA	520870	HOSPITAL I
17/05/202	20	01/05/202	17	SP	GVE I CAPI	1331	SAO PAULC	355030	HOSPITAL I
23/03/202	12	16/03/202	11	MG	SETE LAGO	1467	SETE LAGO	316720	HOSPITAL I
21/03/202	12	15/03/202	11	SP	GVE I CAPI	1331	SAO PAULC	355030	HOSP MUN
06/04/202	14	25/03/202	12	SP	GVE XIX M/	1344	MARILIA	352900	HOSPITAL U
10/05/202	19	24/04/202	16	GO	CENTRAL	1779	GOIANIA	520870	CRER
12/05/202	19	26/04/202	17	SP	GVE I CAPI	1331	SAO PAULC	355030	HOSP MUN
18/06/202	24	08/06/202	23	SP	GVE XXIX S	1354	SAO JOSE D	354980	HOSPITAL I
29/03/202	13	20/03/202	11	CE	1 CRES FOF	1519	FORTALEZ/	230440	HOSPITAL I
12/05/202	19	03/05/202	18	MG	UBERLAND	1462	PATROCINI	314810	HOSPITAL S
28/07/202	30	20/07/202	29	SP	GVE XXXI S	1353	SALTO	354520	HOSPITAL I
13/08/202	32	29/07/202	30	PA	12 REGION	1495	REDENCAC	150613	HOSPITAL I
24/03/202	12	22/03/202	12	SP	GVE X OSA	1335	OSASCO	353440	HOSPITAL I
17/03/202	11	07/03/202	10	AL	1 MICRORI	1533	MACEIO	270430	HOSPITAL V
14/06/202	24	03/06/202	22	SP	GVE XI ARA	1336	PENAPOLIS	353730	SANTA CAS
30/08/202	35	19/08/202	33	RJ			RIO DE JAN	330455	MS HSE HC
03/08/202	31	26/07/202	30	PR	02RS METF	1356	CURITIBA	410690	COMPLEXC
24/03/202	12	17/03/202	11	PE	001	1497	RECIFE	261160	IMIP
23/03/202	12	01/03/202	9	DF			BRASILIA	530010	HRAN

28/04/202	17	02/03/202	9	MG	PATOS DE I	1456	PATOS DE I	314800	HOSPITAL I
12/04/202	15	28/03/202	13	SP	GVE XVII C	1342	CAMPINAS	350950	MATERNID
06/03/202	9	28/02/202	9	CE	1 CRES FOF	1519	FORTALEZA	230440	HOSPITAL I
10/06/202	23	03/06/202	22	PA	09 REGION	1492	ORIXIMINA	150530	HOSPITAL I
11/06/202	23	08/06/202	23	AM	ENTORNO	5584	MANAUS	130260	MATERNID
27/06/202	26	09/06/202	23	MG	UBERLAND	1462	UBERLAND	317020	HOSPITAL S
06/04/202	14	03/03/202	9	RJ			NITEROI	330330	MATERNID
03/10/202	40	25/09/202	38	SC	JOINVILLE	1565	JOINVILLE	420910	HOSPITAL (
15/03/202	11	06/03/202	9	PA	01 REGION	1484	BELEM	150140	SANTA CAS
18/05/202	20	04/05/202	18	RJ			RIO DE JAN	330455	HOSPITAL (
18/06/202	24	08/06/202	23	SP	GVE XVII C	1342	CAMPINAS	350950	MATERNID
23/07/202	29	09/07/202	27	RJ			NILOPOLIS	330320	DOMINGO
25/10/202	43	18/10/202	42	PR	14RS PARA	1368	PARANAVA	411840	SANTA CAS
20/07/202	29	20/07/202	29	PR	17RS LOND	1371	LONDRINA	411370	HOSPITAL I
29/04/202	17	18/04/202	16	PA	09 REGION	1492	SANTAREM	150680	HOSPITAL I
30/03/202	13	18/03/202	11	RS	002 CRS	1607	PORTO ALE	431490	HOSPITAL I
02/08/202	31	21/07/202	29	SC	CHAPECO	1553	CHAPECO	420420	HOSPITAL I
29/03/202	13	16/03/202	11	SP	GVE I CAPI	1331	SAO PAUL(	355030	MATERNID
26/05/202	21	23/05/202	21	MA	REGI0NAL	1430	SAO LUIS	211130	MATERNID
27/04/202	17	10/04/202	14	MT	CUIABA	1578	CUIABA	510340	HOSPITAL I
05/06/202	22	30/05/202	22	MG	UBERLAND	1462	UBERLAND	317020	HOSPITAL I
15/05/202	19	10/05/202	19	MA	REGI0NAL	1430	SAO LUIS	211130	MATERNID
04/03/202	9	27/02/202	8	SP	GVE XVII C	1342	CAMPINAS	350950	MATERNID
18/03/202	11	06/03/202	9	SP	GVE I CAPI	1331	SAO PAUL(	355030	PRO MATR
26/04/202	17	21/04/202	16	GO	CENTRAL	1779	GOIANIA	520870	HOSPITAL I
04/06/202	22	20/05/202	20	SP	GVE XVII C	1342	JUNDIAI	352590	HU HOSPIT
20/05/202	20	10/05/202	19	MG	BELO HORI	1449	BELO HORI	310620	HOSPITAL J
20/05/202	20	15/05/202	19	BA	NUCLEO RI	1381	FEIRA DE S	291080	HOSPITAL I
20/04/202	16	20/03/202	11	MG	GOVERNAI	1471	GOVERNAI	312770	HOSPITAL I
12/05/202	19	01/05/202	17	MT	CUIABA	1578	CUIABA	510340	HOSPITAL U
14/07/202	28	10/07/202	27	GO	CENTRAL	1779	GOIANIA	520870	HOSPITAL I
15/03/202	11	08/03/202	10	RN	VII URSAP	1416	NATAL	240810	HOSPITAL (
13/04/202	15	13/04/202	15	RJ			RIO DE JAN	330455	SMS HOSPI
24/05/202	21	18/05/202	20	PA	01 REGION	1484	BELEM	150140	SANTA CAS
13/06/202	24	07/06/202	23	BA	NUCLEO RI	1380	SALVADOR	292740	MATERNID
03/07/202	26	25/06/202	25	SP	GVE XI ARA	1336	PENAPOLIS	353730	SANTA CAS
14/07/202	28	06/07/202	27	MG	SETE LAGO	1467	SETE LAGO	316720	HOSPITAL I
23/02/202	8	12/02/202	6	GO	CENTRAL	1779	GOIANIA	520870	HOSPITAL I
01/07/202	26	17/06/202	24	RR			BOA VISTA	140010	HOSPITAL (
22/05/202	20	11/05/202	19	SP	GVE XXIX S	1354	SAO JOSE D	354980	HOSPITAL I
31/03/202	13	22/03/202	12	MS	NRS DE DO	1966	DOURADO	500370	EBSERH HC
29/04/202	17	20/04/202	16	RJ			RIO DE JAN	330455	HOSPITAL I
28/09/202	39	20/09/202	38	PR	15RS MARI	1369	SARANDI	412625	METROPOI
23/06/202	25	14/06/202	24	SP	GVE XII AR	1337	SAO CARL(	354890	SANTA CAS
12/05/202	19	03/05/202	18	CE	11 CRES SC	1521	SOBRAL	231290	HOSPITAL I
25/06/202	25	17/06/202	24	RR			BOA VISTA	140010	HOSPITAL (
27/02/202	8	25/02/202	8	PR	10RS CASC	1364	CASCAVEL	410480	HOSPITAL U

26/03/202	12	21/03/202	12	CE	11 CRES SC	1521	SOBRAL	231290	HOSPITAL I
26/03/202	12	18/02/202	7	MT	CUIABA	1578	CUIABA	510340	HOSPITAL U
16/06/202	24	06/06/202	23	DF			BRASILIA	530010	HOSPITAL S
28/04/202	17	16/04/202	15	SP	GVE I CAPI	1331	SAO PAULO	355030	SAO LUIZ U
01/06/202	22	01/06/202	22	PR	17RS LOND	1371	LONDRINA	411370	HOSPITAL I
08/05/202	18	02/05/202	18	RO	VI GRS POF	1483	PORTO VEL	110020	HOSPITAL I
23/07/202	29	18/07/202	29	MG	BELO HORI	1449	CONTAGEM	311860	HOSPITAL I
30/03/202	13	25/03/202	12	GO	SUDOESTE	1799	RIO VERDE	521880	HOSPITAL I
31/03/202	13	10/03/202	10	CE	1 CRES FOF	1519	FORTALEZA	230440	HOSPITAL (
03/04/202	13	22/03/202	12	BA	NUCLEO RI	1380	SALVADOR	292740	MATERNID
04/05/202	18	13/04/202	15	MG	BELO HORI	1449	BETIM	310670	HOSPITAL I
01/05/202	17	26/04/202	17	MG	CORONEL I	1468	CORONEL I	311940	PRO SAUDI
26/04/202	17	10/04/202	14	MG	BELO HORI	1449	BELO HORI	310620	HOSPITAL I
21/07/202	29	07/07/202	27	SP	GVE I CAPI	1331	SAO PAULO	355030	HC DA FMU
21/05/202	20	17/05/202	20	MG	POUSO ALI	1459	EXTREMA	312510	HOSPITAL I
27/07/202	30	23/07/202	29	MA	REGIONAL	1440	IMPERATRI	210530	HRMI HOSI
02/06/202	22	23/05/202	21	SP	GVE XXIV R	1348	RIBEIRAO P	354340	HOSPITAL S
05/05/202	18	26/04/202	17	BA	NUCLEO RI	1380	SALVADOR	292740	MATERNID
17/06/202	24	02/06/202	22	SP	GVE I CAPI	1331	SAO PAULO	355030	MATERNID
11/06/202	23	06/06/202	23	PR	05RS GUAF	1359	GUARAPUA	410940	HOSPITAL I
27/05/202	21	17/05/202	20	SP	GVE XX PIR	1345	PIRACICAB	353870	HOSPITAL I
15/03/202	11	13/03/202	10	RS	002 CRS	1607	PORTO ALE	431490	HOSPITAL I
03/04/202	13	21/03/202	12	GO	SERRA DA I	1790	URUACU	522160	UPA UNIDA
15/04/202	15	11/03/202	10	RS	005 CRS	1608	CAXIAS DO	430510	HOSPITAL I
31/05/202	22	13/05/202	19	SP	GVE XXIV R	1348	RIBEIRAO P	354340	HOSPITAL I
01/06/202	22	20/05/202	20	MG	VARGINHA	1466	TRES PONT	316940	SANTA CAS
04/06/202	22	26/05/202	21	SP	GVE VIII M	1333	ITAQUAQU	352310	HOSPITAL (
01/06/202	22	24/05/202	21	SP	GVE XV BA	1340	BAURU	350600	UPA GEISE
14/06/202	24	30/05/202	22	MA	REGI0NAL	1430	SAO LUIS	211130	HOSPITAL I
09/10/202	40	05/10/202	40	SC	TUBARAO	1555	TUBARAO	421870	SOCIMED
11/06/202	23	03/06/202	22	GO	PIRINEUS	1792	ANAPOLIS	520110	SANTA CAS
30/04/202	17	29/04/202	17	MG	UBERABA	1461	UBERABA	317010	HOSPITAL I
02/06/202	22	19/05/202	20	MG	BELO HORI	1449	BETIM	310670	HOSPITAL I
27/05/202	21	18/05/202	20	BA	NUCLEO RI	1380	SALVADOR	292740	HOSPITAL (
26/07/202	30	19/07/202	29	SP	GVE I CAPI	1331	SAO PAULO	355030	HOSP MUN
20/07/202	29	15/07/202	28	SP	GVE I CAPI	1331	SAO PAULO	355030	HOSPITAL I
05/06/202	22	21/05/202	20	RS	002 CRS	1607	PORTO ALE	431490	HOSPITAL I
06/04/202	14	06/04/202	14	CE	1 CRES FOF	1519	FORTALEZA	230440	HGCC HOS
29/05/202	21	23/05/202	21	MG	ALFENAS	1379	GUAXUPE	312870	SANTA CAS
01/04/202	13	01/04/202	13	SP	GVE XXI PR	1346	PRESIDENT	354140	HOSPITAL I
25/05/202	21	21/05/202	20	RJ			RIO DE JAN	330455	MS HSE HC
12/06/202	23	31/05/202	22	MG	UBERLAND	1462	UBERLAND	317020	HOSPITAL S
08/07/202	27	01/07/202	26	SP	GVE XXXI S	1353	SOROCABA	355220	HOSPITAL I
02/03/202	9	24/02/202	8	RR			BOA VISTA	140010	HOSPITAL (
19/04/202	16	09/04/202	14	RJ			RIO DE JAN	330455	MS HSE HC
13/05/202	19	19/04/202	16	RJ			RIO DE JAN	330455	SMS HOSPI
13/04/202	15	08/04/202	14	SP	GVE VIII M	1333	GUARULHO	351880	HOSPITAL I

06/05/202	18	27/04/202	17	MG	BELO HORI	1449	BELO HORI	310620	HOSPITAL J
06/07/202	27	20/06/202	25	SC	ITAJAI	1550	BALNEARIO	420200	HOSPITAL I
22/07/202	29	07/07/202	27	BA	NUCLEO RE	1380	SALVADOR	292740	MATERNID
10/03/202	10	07/03/202	10	MG	PATOS DE I	1456	PATOS DE I	314800	HOSPITAL I
03/03/202	9	24/02/202	8	GO	CENTRAL	1779	GOIANIA	520870	HOSPITAL I
19/02/202	7	10/02/202	6	PR	08RS FRAN	1362	FRANCISCO	410840	POLICLINIC
13/04/202	15	11/04/202	15	BA	NUCLEO RE	1380	SALVADOR	292740	MATERNID
22/04/202	16	08/04/202	14	PR	10RS CASC	1364	CASCAVEL	410480	HOSPITAL U
25/05/202	21	19/05/202	20	MG	DIAMANTI	1450	CARBONITA	311350	HOSPITAL S
25/04/202	17	09/04/202	14	SP	GVE I CAPIT	1331	SAO PAULO	355030	HOSP MUN
11/05/202	19	01/05/202	17	CE	1 CRES FOR	1519	FORTALEZA	230440	HGCC HOS
23/08/202	34	20/08/202	33	SC	VIDEIRA	1546	CURITIBAN	420480	HOSPITAL I
16/03/202	11	04/03/202	9	SP	GVE I CAPIT	1331	SAO PAULO	355030	HC DA FMU
05/04/202	14	30/03/202	13	SP	GVE XXIV R	1348	RIBEIRAO P	354340	HOSPITAL I
07/06/202	23	25/05/202	21	BA	NUCLEO RE	1382	RIO REAL	292700	UPA 24HS A
10/04/202	14	05/04/202	14	PR	17RS LOND	1371	LONDRINA	411370	HOSPITAL U
20/05/202	20	07/05/202	18	MG	SETE LAGO	1467	SETE LAGO	316720	HOSPITAL I
31/05/202	22	15/05/202	19	PR	02RS METR	1356	CURITIBA	410690	COMPLEXO
06/06/202	23	22/05/202	20	GO	ENTORNO	1782	LUZIANIA	521250	UTI SANTA
22/05/202	20	08/05/202	18	SC	ARARANGU	1562	ARARANGU	420140	IMAS HOSP
27/05/202	21	20/05/202	20	SP	GVE I CAPIT	1331	SAO PAULO	355030	HC DA FMU
12/05/202	19	10/05/202	19	MG	JUIZ DE FO	1452	JUIZ DE FO	313670	SANTA CAS
09/02/202	6	03/02/202	5	BA	NUCLEO RE	1380	SALVADOR	292740	INSTITUTO
06/02/202	5	30/01/202	4	BA	NUCLEO RE	1380	SALVADOR	292740	MATERNID
05/04/202	14	14/03/202	11	DF			BRASILIA	530010	HRAN
02/06/202	22	23/05/202	21	PR	02RS METR	1356	CURITIBA	410690	COMPLEXO
07/03/202	10	03/03/202	9	PR	15RS MARI	1369	MARINGA	411520	SANTA CAS
05/08/202	31	26/07/202	30	SC	JOINVILLE	1565	JOINVILLE	420910	HOSPITAL I
19/08/202	33	21/07/202	29	SP	GVE I CAPIT	1331	SAO PAULO	355030	HOSPITAL I
23/09/202	38	08/09/202	36	SP	GVE I CAPIT	1331	SAO PAULO	355030	MATERNID
11/03/202	10	08/03/202	10	SP	GVE XXIII R	1347	PARIQUERA	353620	HOSPITAL I
06/05/202	18	25/04/202	17	SP	GVE I CAPIT	1331	SAO PAULO	355030	HOSPITAL I
17/06/202	24	14/05/202	19	PR	15RS MARI	1369	MARINGA	411520	HOSPITAL I
15/06/202	24	04/06/202	22	RJ			RIO DE JAN	330455	HOSPITAL I
24/08/202	34	06/07/202	27	GO	CENTRAL	1779	GOIANIA	520870	HOSPITAL I
19/02/202	7	11/02/202	6	SP	GVE X OSA	1335	BARUERI	350570	HOSPITALIS
01/04/202	13	25/03/202	12	MG	UBERABA	1461	ARAXA	310400	SANTA SAN
07/05/202	18	25/04/202	17	MG	MANHUAC	1454	MANHUAC	313940	HOSPITAL C
08/07/202	27	21/06/202	25	SP	GVE I CAPIT	1331	SAO PAULO	355030	HOSPITAL S
22/07/202	29	17/07/202	28	MS	EX ARS DE	1975	CAMPO GR	500270	HOSPITAL I
31/05/202	22	18/05/202	20	DF			BRASILIA	530010	HRAN
09/06/202	23	25/05/202	21	RN	II URSAP	1412	MOSSORO	240800	HOSPITAL S
11/07/202	28	26/05/202	21	MG	DIVINOPOL	1472	OLIVEIRA	314560	HOSPITAL S
12/05/202	19	01/05/202	17	RJ			NILOPOLIS	330320	DOMINGOS
04/03/202	9	24/02/202	8	AM	ENTORNO	5584	MANAUS	130260	MATERNID
24/03/202	12	09/03/202	10	SP	GVE XII AR	1337	ARARAQUA	350320	SANTA CAS
13/04/202	15	05/04/202	14	AL	1 MICRORE	1533	MACEIO	270430	HOSPITAL I

22/06/202	25	17/06/202	24	MT	CUIABA	1578	CUIABA	510340	HOSPITAL I
07/12/202	49	27/11/202	47	AP			SANTANA	160060	HOSPITAL I
30/04/202	17	15/04/202	15	SP	GVE I CAPI	1331	SAO PAULC	355030	SAO LUIZ U
31/03/202	13	18/03/202	11	PR	02RS METF	1356	CAMPINA (	410400	HOSPITAL /
23/02/202	8	20/02/202	7	RJ			NITEROI	330330	SES RJ HOS
25/05/202	21	17/05/202	20	SP	GVE XXIV F	1348	RIBEIRAO F	354340	HOSPITAL I
27/04/202	17	17/04/202	15	SP	GVE I CAPI	1331	SAO PAULC	355030	HOSPITAL (
29/04/202	17	22/04/202	16	SP	GVE XXVI S	1350	MOGI GUA	353070	SANTA CAS
17/06/202	24	15/05/202	19	SP	GVE XIX M.	1344	MARILIA	352900	HCFAMEM
08/07/202	27	01/07/202	26	SC	JARAGUA I	1567	JARAGUA I	420890	HOSPITAL J
22/04/202	16	15/04/202	15	BA	NUCLEO RI	1381	FEIRA DE S.	291080	HOSPITAL I
09/03/202	10	27/02/202	8	SP	GVE XXXI S	1353	SOROCABA	355220	HOSPITAL I
28/07/202	30	22/07/202	29	RS	010 CRS	1599	SAO GABRI	431830	SANTA CAS
05/07/202	27	27/06/202	26	MA	REGIONAL	1441	BALSAS	210140	HOSPITAL I
10/02/202	6	07/02/202	6	RJ			VALENCA	330610	HOSPITAL I
15/10/202	41	11/10/202	41	MG	ALFENAS	1379	ALFENAS	310160	SANTA CAS
16/04/202	15	07/04/202	14	SP	GVE XXI PR	1346	PRESIDENT	354140	HOSPITAL I
05/05/202	18	21/04/202	16	MT	CUIABA	1578	CUIABA	510340	FEMINA HC
25/05/202	21	22/05/202	20	GO	CENTRAL	1779	GOIANIA	520870	HOSPITAL I
17/05/202	20	01/05/202	17	SP	GVE XVI BC	1341	BOTUCATU	350750	HOSPITAL I
20/05/202	20	12/05/202	19	RS	012 CRS	1601	SANTO AN	431750	HOSPITAL S
11/06/202	23	01/06/202	22	RJ			RIO DE JAN	330455	SMS RIO H
28/09/202	39	17/09/202	37	DF			BRASILIA	530010	HRAN
13/04/202	15	05/04/202	14	RJ			RIO DE JAN	330455	MS HSE HC
04/03/202	9	20/02/202	7	SP	GVE XXIX S	1354	SAO JOSE I	354980	HOSPITAL I
30/04/202	17	27/04/202	17	SP	GVE I CAPI	1331	SAO PAULC	355030	SAO LUIZ U
27/05/202	21	16/05/202	20	SP	GVE XVIII F	1343	FRANCA	351620	HOSPITAL I
15/06/202	24	06/06/202	23	BA	NUCLEO RI	1398	JACARACI	291740	HOSPITAL I
31/05/202	22	17/05/202	20	MS	EX ARS DE	1975	CAMPO GF	500270	HOSPITAL I
02/08/202	31	20/03/202	11	PR	16RS APUC	1370	APUCARAN	410140	HNSG HOS
03/12/202	48	13/11/202	45	SP	GVE I CAPI	1331	SAO PAULC	355030	HC DA FMU
10/02/202	6	04/02/202	5	PR	17RS LOND	1371	LONDRINA	411370	HOSPITAL I
25/02/202	8	22/02/202	8	SP	GVE I CAPI	1331	SAO PAULC	355030	PS MUN V
18/03/202	11	08/03/202	10	MT	CUIABA	1578	CUIABA	510340	HOSPITAL I
12/03/202	10	22/02/202	8	DF			BRASILIA	530010	HRAN
13/04/202	15	11/04/202	15	GO	PIRINEUS	1792	ANAPOLIS	520110	SANTA CAS
29/04/202	17	14/04/202	15	RS	001 CRS	1592	CANOAS	430460	DIRETORIA
19/11/202	46	08/11/202	45	RS	003 CRS	1593	RIO GRANI	431560	SANTA CAS
19/03/202	11	15/03/202	11	RN	II URSAP	1412	MOSSORO	240800	HOSPITAL I
31/05/202	22	23/05/202	21	MG	GOVERNAI	1471	GOVERNAI	312770	HOSPITAL I
28/07/202	30	23/07/202	29	SC	TUBARAO	1555	TUBARAO	421870	HOSPITAL I
03/01/202	1	01/01/202	52	SP	GVE XVIII F	1343	FRANCA	351620	HOSPITAL I
24/06/202	25	14/06/202	24	SP	GVE XVI BC	1341	BOTUCATU	350750	HOSPITAL I
14/01/202	2	08/01/202	1	SP	GVE X OSA	1335	OSASCO	353440	HOSPITAL I
18/03/202	11	11/03/202	10	RS	004 CRS	1595	SANTA MA	431690	HOSPITAL I
14/03/202	11	14/03/202	11	PA	11 REGION	1494	MARABA	150420	HOSPITAL I
07/05/202	18	06/05/202	18	RJ			RIO DE JAN	330455	SMS HOSPI

29/05/202	21	22/05/202	20	SP	GVE I CAPI	1331	SAO PAUL	355030	HOSP MAT
29/04/202	17	21/04/202	16	MG	UBERABA	1461	UBERABA	317010	HOSPITAL
13/03/202	10	01/03/202	9	GO	CENTRAL	1779	GOIANIA	520870	HOSPITAL
16/04/202	15	09/04/202	14	PR	10RS CASC	1364	CASCAVEL	410480	HOSPITAL
07/05/202	18	06/05/202	18	PA	11 REGION	1494	MARABA	150420	HOSPITAL
09/08/202	32	27/07/202	30	GO	CENTRAL	1779	GOIANIA	520870	HOSPITAL
18/06/202	24	13/05/202	19	MT	CUIABA	1578	CUIABA	510340	HOSPITAL
06/01/202	1	22/12/202	51	MA	REGIONAL	1440	IMPERATR	210530	HRMI HOS
15/03/202	11	01/03/202	9	PR	17RS LON	1371	LONDRINA	411370	HOSPITAL
25/01/202	4	18/01/202	3	PB	I NRS JOA	1377	JOAO PESS	250750	MATERNID
03/05/202	18	25/04/202	17	SP	GVE XXIV	1348	RIBEIRAO	354340	HOSPITAL
18/03/202	11	15/03/202	11	AM	ENTORNO	5584	MANAUS	130260	MATERNID
27/03/202	12	20/03/202	11	PE	001	1497	RECIFE	261160	IMIP
18/06/202	24	04/04/202	14	PA	01 REGION	1484	BELEM	150140	SANTA CAS
26/04/202	17	16/04/202	15	BA	NUCLEO R	1380	SALVADOR	292740	MATERNID
16/08/202	33	01/08/202	31	MT	CUIABA	1578	CUIABA	510340	HOSPITAL
11/01/202	2	10/01/202	2	PR	02RS METR	1356	CURITIBA	410690	HOSPITAL
17/03/202	11	14/03/202	11	MA	REGIONAL	1447	CAXIAS	210300	COMPLEX
28/06/202	26	16/06/202	24	RS	018 CRS	1605	TRAMAND	432160	HOSPITAL
08/03/202	10	27/02/202	8	BA	NUCLEO R	1380	SALVADOR	292740	HOSPITAL
15/03/202	11	03/03/202	9	RJ			RIO DE JAN	330455	HOSPITAL
21/03/202	12	12/03/202	10	CE	1 CRES FO	1519	FORTALEZ	230440	HGCC HOS
14/07/202	28	28/06/202	26	SP	GVE I CAPI	1331	SAO PAUL	355030	MATERNID
12/06/202	23	07/06/202	23	SP	GVE IX FRA	1334	CAIEIRAS	350900	VIGILANCI
14/06/202	24	03/06/202	22	RS	004 CRS	1595	SANTA MA	431690	HUSM HOS
16/08/202	33	04/08/202	31	PR	02RS METR	1356	CURITIBA	410690	ONIX MAT
20/05/202	20	10/05/202	19	PR	17RS LON	1371	LONDRINA	411370	HOSPITAL
26/01/202	4	10/01/202	2	RS	002 CRS	1607	PORTO ALE	431490	HOSPITAL
08/06/202	23	25/05/202	21	SC	CANOINH	1570	CANOINH	420380	HOSPITAL
23/07/202	29	12/07/202	28	SP	GVE XXIX S	1354	SAO JOSE	354980	HOSPITAL
18/06/202	24	13/06/202	24	RJ			RIO DE JAN	330455	HOSPITAL
13/05/202	19	13/05/202	19	CE	1 CRES FO	1519	FORTALEZ	230440	HGCC HOS
18/11/202	46	07/11/202	45	SC	FLORIANO	1476	FLORIANO	420540	HOSPITAL
17/04/202	15	07/04/202	14	BA	NUCLEO R	1398	VITORIA D	293330	SAMUR
14/04/202	15	06/04/202	14	MS	EX ARS DE	1975	CAMPO G	500270	HOSPITAL
17/06/202	24	06/06/202	23	SP	GVE I CAPI	1331	SAO PAUL	355030	MATERNID
15/02/202	7	03/02/202	5	RS	016 CRS	1610	LAJEADO	431140	HOSPITAL
03/03/202	9	24/02/202	8	GO	CENTRAL	1779	GOIANIA	520870	HOSPITAL
17/03/202	11	16/02/202	7	SP	GVE I CAPI	1331	SAO PAUL	355030	HOSP IGES
05/04/202	14	19/03/202	11	PB	I NRS JOA	1377	JOAO PESS	250750	MATERNID
13/07/202	28	06/07/202	27	PR	10RS CASC	1364	CASCAVEL	410480	HOSPITAL
05/08/202	31	23/07/202	29	RJ			RESENDE	330420	HOSPITAL
14/01/202	2	11/01/202	2	SP	GVE XX PIR	1345	PIRACICAB	353870	SANTA CAS
27/01/202	4	19/01/202	3	MG	VARGINHA	1466	AIURUOCA	310120	HOSPITAL
08/02/202	6	04/01/202	1	SP	GVE I CAPI	1331	SAO PAUL	355030	HOSP MUN
08/02/202	6	30/01/202	4	SP	GVE I CAPI	1331	SAO PAUL	355030	HOSP AVIC
05/04/202	14	01/04/202	13	PA	01 REGION	1484	BELEM	150140	MATERNID

18/04/202	16	01/04/202	13	SP	GVE I CAPI	1331	SAO PAUL(	355030	UNIDADE D
16/06/202	24	13/06/202	24	SP	GVE I CAPI	1331	SAO PAUL(	355030	HC DA FMU
14/06/202	24	10/06/202	23	RN	VII URSAP	1416	NATAL	240810	HOSPITAL I
27/03/202	12	17/03/202	11	SP	GVE XVII C.	1342	JAGUARIUI	352470	HOSPITAL I
26/05/202	21	11/05/202	19	SP	GVE I CAPI	1331	SAO PAUL(	355030	HC DA FMU
01/06/202	22	14/05/202	19	SP	GVE XV BA	1340	BAURU	350600	HOSPITAL U
25/08/202	34	20/08/202	33	SC	JOINVILLE	1565	JOINVILLE	420910	HOSPITAL I
27/01/202	4	22/01/202	3	RJ			NITEROI	330330	HOSPITAL I
04/04/202	14	25/03/202	12	RS	007 CRS	1596	BAGE	430160	SANTA CAS
29/05/202	21	20/05/202	20	RJ			RIO DE JAN	330455	SMS RIO H
31/01/202	5	19/01/202	3	AM	ENTORNO	5584	MANAUS	130260	INSTITUTO
03/03/202	9	23/02/202	8	PR	02RS METF	1356	CAMPINA (	410400	HOSPITAL A
19/02/202	7	19/02/202	7	MG	BELO HORI	1449	BETIM	310670	UPA JOSE S
22/04/202	16	21/03/202	12	SP	GVE I CAPI	1331	SAO PAUL(	355030	SAO LUIZ U
10/06/202	23	30/05/202	22	PR	02RS METF	1356	CURITIBA	410690	COMPLEXO
11/06/202	23	31/05/202	22	SP	GVE I CAPI	1331	SAO PAUL(	355030	MATERNID
19/07/202	29	15/07/202	28	PR	15RS MARI	1369	MARINGA	411520	SANTA CAS
23/08/202	34	14/08/202	32	PR	09RS FOZ D	1363	FOZ DO IGU	410830	HOSPITAL I
13/01/202	2	05/01/202	1	DF			BRASILIA	530010	HOSPITAL S
12/03/202	10	02/03/202	9	PE	001	1497	RECIFE	261160	HOSPITAL (
17/03/202	11	01/03/202	9	GO	CENTRAL	1779	GOIANIA	520870	HOSPITAL I
28/03/202	13	13/03/202	10	PR	17RS LOND	1371	LONDRINA	411370	HOSPITAL U
27/04/202	17	24/04/202	16	RS	002 CRS	1607	CACHOEIRI	430310	HOSPITAL I
02/06/202	22	15/05/202	19	RN	VII URSAP	1416	NATAL	240810	HOSPITAL I
05/09/202	36	02/09/202	35	SC	BLUMENAU	1564	BLUMENAU	420240	HOSPITAL S
14/01/202	2	09/01/202	1	GO	CENTRAL	1779	GOIANIA	520870	HOSPITAL I
15/03/202	11	09/03/202	10	PB	I NRS JOAC	1377	JOAO PESS	250750	MATERNID
06/04/202	14	12/03/202	10	SP	GVE I CAPI	1331	SAO PAUL(	355030	HC DA FMU
30/04/202	17	23/04/202	16	RS	001 CRS	1592	SAPIRANG/	431990	HOSPITAL S
20/10/202	42	15/10/202	41	SP	GVE XVII C.	1342	CAMPINAS	350950	HOSPITAL I
05/03/202	9	25/02/202	8	PR	15RS MARI	1369	MARINGA	411520	HOSPITAL U
06/02/202	5	25/01/202	4	SC	CONCORDI	1560	CONCORDI	420430	HOSPITAL S
04/06/202	22	04/06/202	22	GO	CENTRAL	1779	GOIANIA	520870	HOSPITAL I
26/08/202	34	19/08/202	33	DF			BRASILIA	530010	HOSPITAL S
14/03/202	11	10/03/202	10	GO	ESTRADA D	1802	CATALAO	520510	SANTA CAS
04/05/202	18	25/04/202	17	RS	005 CRS	1608	GRAMADO	430910	HOSPITAL A
22/06/202	25	11/06/202	23	MA	REGI0NAL	1430	SAO LUIS	211130	MATERNID
23/03/202	12	16/03/202	11	ES	METROPOI	1510	VITORIA	320530	DIVISAO VI
17/05/202	20	17/05/202	20	CE	1 CRES FOF	1519	FORTALEZ/	230440	HGCC HOS
12/08/202	32	03/08/202	31	RJ			RIO DE JAN	330455	MS HSE HC
04/06/202	22	28/05/202	21	MT	CUIABA	1578	CUIABA	510340	HOSPITAL U
22/06/202	25	05/06/202	22	RS	005 CRS	1608	CAXIAS DO	430510	HOSPITAL U
01/10/202	39	25/09/202	38	MG	JANUARIA	1470	JANUARIA	313520	HOSPITAL I
06/03/202	9	01/03/202	9	PR	10RS CASC	1364	CASCAVEL	410480	HOSPITAL U
16/01/202	2	06/01/202	1	MG	POUSO ALI	1459	ITAJUBA	313240	AISI HOSPI
01/06/202	22	25/05/202	21	AL	5 MICRORF	1537	ARAPIRAC/	270030	HOSPITAL I
01/04/202	13	17/03/202	11	GO	CENTRAL	1779	GOIANIA	520870	HOSPITAL I

10/05/202	19	01/05/202	17	BA	NUCLEO RI	1380	SALVADOR	292740	HOSPITAL /
06/04/202	14	30/03/202	13	SP	GVE XX PIR	1345	ARARAS	350330	HOSPITAL S
11/06/202	23	04/06/202	22	SP	GVE XXIX S	1354	SAO JOSE D	354980	HOSPITAL I
27/04/202	17	25/04/202	17	SC	FLORIANOI	1476	FLORIANOI	420540	HOSPITAL U
06/07/202	27	30/06/202	26	SP	GVE XXXI S	1353	SOROCABA	355220	SANTA CAS
22/05/202	20	14/05/202	19	SP	GVE I CAPI	1331	SAO PAULO	355030	HOSPITAL S
05/02/202	5	04/02/202	5	MG	VARGINHA	1466	BOA ESPER	310710	SANTA CAS
06/09/202	36	24/08/202	34	SC	CHAPECO	1553	CHAPECO	420420	HOSPITAL I
01/05/202	17	28/04/202	17	RN	VII URSAP	1416	NATAL	240810	PROMATEI
29/06/202	26	19/06/202	24	RR			BOA VISTA	140010	HOSPITAL (
18/06/202	24	05/06/202	22	PB	I NRS JOAC	1377	JOAO PESS	250750	MATERNID
23/07/202	29	31/05/202	22	PR	16RS APUC	1370	APUCARAN	410140	HNSG HOS
26/03/202	12	14/03/202	11	MG	BELO HORI	1449	BELO HORI	310620	MATERNID
20/04/202	16	16/04/202	15	DF			BRASILIA	530010	HRT HOSPI
21/03/202	12	17/03/202	11	PA	01 REGION	1484	BELEM	150140	SANTA CAS
17/05/202	20	10/05/202	19	RJ			CAMPOS D	330100	HOSPITAL I
18/01/202	3	14/01/202	2	GO	SUDOESTE	1800	MINEIROS	521310	HOSPITAL I
09/06/202	23	02/06/202	22	MG	DIVINOPOI	1472	NOVA SERI	314520	UPA ANTO
09/08/202	32	06/08/202	31	RJ			RIO DE JAN	330455	SMS RIO H
04/05/202	18	31/03/202	13	GO	CENTRAL	1779	GOIANIA	520870	HOSPITAL I
05/03/202	9	16/02/202	7	AP			MACAPA	160030	HOSPITAL I
23/03/202	12	14/03/202	11	AL	1 MICRORI	1533	MACEIO	270430	HOSPITAL I
20/04/202	16	10/04/202	14	GO	CENTRAL	1779	GOIANIA	520870	HOSPITAL I
15/07/202	28	12/02/202	6	RJ			RIO DE JAN	330455	UERJ HOSP
17/02/202	7	13/02/202	6	SC	CHAPECO	1553	CHAPECO	420420	HOSPITAL I
05/04/202	14	18/03/202	11	RS	005 CRS	1608	CAXIAS DO	430510	HOSPITAL I
26/04/202	17	11/04/202	15	BA	NUCLEO RI	1380	SALVADOR	292740	HOSPITAL /
07/07/202	27	05/07/202	27	RJ			RIO DE JAN	330455	SMS RIO H
11/07/202	28	28/06/202	26	MS	NRS DE TRI	6255	TRES LAGO	500830	HOSPITAL (
02/06/202	22	21/05/202	20	PR	11RS CAMI	1365	CAMPO M	410430	HOSPITAL S
13/10/202	41	02/10/202	39	SC	FLORIANOI	1476	FLORIANOI	420540	HOSPITAL U
08/01/202	1	07/01/202	1	SP	GVE I CAPI	1331	SAO PAULO	355030	SAO LUIZ U
31/05/202	22	24/05/202	21	PI	4 GRS TERE	1883	TERESINA	221100	MATERNID
26/10/202	43	14/10/202	41	SC	BLUMENAU	1564	BLUMENAU	420240	HOSPITAL S
15/03/202	11	26/02/202	8	RS	001 CRS	1592	CANOAS	430460	HOSPITAL U
14/03/202	11	04/03/202	9	RS	002 CRS	1607	PORTO ALE	431490	HOSPITAL I
11/05/202	19	04/05/202	18	MS	EX ARS DE	1975	CAMPO GF	500270	HOSPITAL I
14/06/202	24	07/06/202	23	SC	LAGES	1571	LAGES	420930	HOSPITAL I
06/04/202	14	06/04/202	14	CE	1 CRES FOF	1519	FORTALEZA	230440	HGCC HOS
19/03/202	11	01/03/202	9	SP	GVE XVII C.	1342	CAMPINAS	350950	MATERNID
01/04/202	13	29/03/202	13	RS	006 CRS	1611	PASSO FUN	431410	HOSPITAL I
27/03/202	12	23/03/202	12	MG	SAO JOAO	1460	SAO JOAO	316250	SANTA CAS
19/07/202	29	14/07/202	28	MT	CUIABA	1578	CUIABA	510340	HOSPITAL U
28/05/202	21	24/05/202	21	RS	006 CRS	1611	PASSO FUN	431410	HOSPITAL S
13/04/202	15	04/04/202	14	DF			BRASILIA	530010	HOSPITAL U
07/05/202	18	28/04/202	17	RR			BOA VISTA	140010	HOSPITAL (
30/05/202	22	22/05/202	20	SP	GVE XVII C.	1342	CAMPINAS	350950	HOSPITAL I

23/11/202	47	11/11/202	45	MT	SINOP	1584	SINOP	510790	HOSPITAL S
14/06/202	24	05/06/202	22	RJ			RIO DE JAN	330455	MS HSE HC
28/04/202	17	18/04/202	16	SP	GVE I CAPI	1331	SAO PAULO	355030	HC DA FMU
30/04/202	17	20/04/202	16	GO	CENTRAL	1779	GOIANIA	520870	HOSPITAL I
12/05/202	19	06/05/202	18	RJ			RIO DE JAN	330455	INI FIOCRU
25/03/202	12	11/03/202	10	SP	GVE VIII M	1333	GUARULHO	351880	HOSPITAL U
27/07/202	30	10/07/202	27	PR	09RS FOZ D	1363	FOZ DO IGU	410830	HOSPITAL I
18/01/202	3	07/01/202	1	SP	GVE XIV BA	1339	BARRETOS	350550	FUNDACAO
12/04/202	15	02/04/202	13	PR	17RS LOND	1371	LONDRINA	411370	HOSPITAL U
23/02/202	8	13/02/202	6	SP	GVE XX PIR	1345	PIRACICAB	353870	HOSPITAL I
22/04/202	16	07/04/202	14	PR	02RS METF	1356	CAMPINA (	410400	HOSPITAL A
04/06/202	22	24/05/202	21	RS	005 CRS	1608	CAXIAS DO	430510	HOSPITAL (
03/08/202	31	27/07/202	30	SP	GVE XXIX S	1354	SAO JOSE D	354980	HOSPITAL I
18/02/202	7	12/02/202	6	RS	002 CRS	1607	PORTO ALE	431490	HOSPITAL I
31/03/202	13	19/03/202	11	MA	REGI0NAL	1430	SAO LUIS	211130	EBSERH HC
25/05/202	21	14/05/202	19	RJ			RIO DE JAN	330455	HOSPITAL I
19/07/202	29	10/07/202	27	RJ			RIO DE JAN	330455	MS HSE HC
14/05/202	19	25/04/202	17	SP	GVE XXXIII	1352	TAUBATE	355410	PRONTO S(
15/04/202	15	06/04/202	14	SP	GVE I CAPI	1331	SAO PAULO	355030	HOSPITAL S
05/06/202	22	02/06/202	22	RJ			VASSOURA	330620	HUV HOSP
27/07/202	30	26/07/202	30	TO			PALMAS	172100	HOSPITAL (
23/07/202	29	23/07/202	29	CE	1 CRES FOF	1519	FORTALEZA	230440	HGCC HOS
12/05/202	19	05/05/202	18	RJ			NILOPOLIS	330320	DOMINGO
19/10/202	42	13/10/202	41	SC	XANXERE	1556	XANXERE	421950	HOSPITAL I
11/03/202	10	13/02/202	6	RS	001 CRS	1592	CANOAS	430460	HOSPITAL U
02/04/202	13	25/03/202	12	RO	VI GRS POF	1483	PORTO VEL	110020	HOSPITAL I
20/02/202	7	12/02/202	6	SP	GVE I CAPI	1331	SAO PAULO	355030	MATERNID
22/08/202	34	14/08/202	32	PB	III NRS CAN	1421	CAMPINA (	250400	HOSPITAL I
24/05/202	21	14/05/202	19	MG	LEOPOLDIN	1453	ALEM PARA	310150	HOSPITAL S
27/08/202	34	17/08/202	33	PR	10RS CASC	1364	CASCAVEL	410480	UPA BRASI
12/11/202	45	30/10/202	43	SC	ARARANGU	1562	ARARANGU	420140	IMAS HOSF
10/07/202	27	28/06/202	26	RS	002 CRS	1607	PORTO ALE	431490	HOSPITAL I
08/04/202	14	27/03/202	12	SP	GVE I CAPI	1331	SAO PAULO	355030	HC DA FMU
27/03/202	12	25/03/202	12	MT	SINOP	1584	NOVA MUT	510622	HOSPITAL I
22/06/202	25	07/06/202	23	DF			BRASILIA	530010	HRSM
14/07/202	28	14/07/202	28	MG	BELO HORI	1449	BELO HORI	310620	HOSPITAL I
18/11/202	46	06/11/202	44	SP	GVE XII ARA	1337	SAO CARLO	354890	SANTA CAS
14/05/202	19	01/05/202	17	RS	005 CRS	1608	CAXIAS DO	430510	HOSPITAL (
09/10/202	40	03/10/202	40	TO			ARAGUAIN	170210	HOSPITAL I
22/07/202	29	08/07/202	27	MG	BELO HORI	1449	CONTAGEM	311860	HOSPITAL I
17/08/202	33	03/08/202	31	TO			PALMAS	172100	HOSPITAL (
10/03/202	10	28/02/202	9	PR	02RS METF	1356	CURITIBA	410690	COMPLEXO
31/12/202	52	25/12/202	51	SC	JOINVILLE	1565	JOINVILLE	420910	HOSPITAL I
01/04/202	13	17/03/202	11	SP	GVE XVII C/	1342	CAMPINAS	350950	HOSPITAL I
08/04/202	14	29/03/202	13	BA	NUCLEO RE	1380	SALVADOR	292740	MATERNID
11/02/202	6	03/02/202	5	GO	CENTRAL	1779	GOIANIA	520870	HOSPITAL I
18/01/202	3	14/01/202	2	AC	REGIONAL	1938	RIO BRANC	120040	MATERNID

05/05/202	18	09/04/202	14	MT	CUIABA	1578	CUIABA	510340	HOSPITAL I
07/06/202	23	25/05/202	21	RJ			RIO DE JAN	330455	MS HSE HC
09/04/202	14	26/03/202	12	SP	GVE XXIV F	1348	RIBEIRAO F	354340	HOSPITAL I
02/05/202	18	26/04/202	17	AL	1 MICRORI	1533	MACEIO	270430	HOSPITAL I
15/04/202	15	05/04/202	14	RJ			RIO DE JAN	330455	CLINICA SA
24/05/202	21	21/05/202	20	BA	NUCLEO RI	1380	SALVADOR	292740	MATERNID
12/05/202	19	09/05/202	19	MG	BELO HORI	1449	BELO HORI	310620	HOSPITAL J
15/06/202	24	06/06/202	23	BA	NUCLEO RI	1380	SALVADOR	292740	MATERNID
26/07/202	30	12/07/202	28	SP	GVE XXVII S	1351	JACAREI	352440	HOSPITAL S
11/05/202	19	01/05/202	17	SP	GVE XVI BC	1341	BOTUCATU	350750	HOSPITAL I
02/03/202	9	17/02/202	7	GO	CENTRAL	1779	GOIANIA	520870	SAU I SERV
13/03/202	10	01/03/202	9	CE	1 CRES FOI	1519	FORTALEZA	230440	HGCC HOS
14/03/202	11	07/03/202	10	CE	1 CRES FOI	1519	FORTALEZA	230440	HGCC HOS
11/04/202	15	01/04/202	13	SP	GVE XXIX S	1354	SAO JOSE D	354980	HOSPITAL I
01/06/202	22	18/05/202	20	BA	NUCLEO RI	1403	BARREIRAS	290320	HOSPITAL I
24/05/202	21	15/05/202	19	SP	GVE XXIV F	1348	RIBEIRAO F	354340	HOSPITAL I
14/07/202	28	08/07/202	27	SP	GVE VII SAI	1332	SAO CAETA	354880	HOSPITAL S
09/03/202	10	20/02/202	7	SP	GVE I CAPI	1331	SAO PAULO	355030	MATERNID
16/04/202	15	12/04/202	15	BA	NUCLEO RI	1380	SALVADOR	292740	MATERNID
24/05/202	21	21/05/202	20	GO	CENTRAL	1779	GOIANIA	520870	HOSPITAL I
27/05/202	21	18/05/202	20	GO	CENTRAL	1779	GOIANIA	520870	HOSPITAL I
06/04/202	14	28/03/202	13	CE	1 CRES FOI	1519	FORTALEZA	230440	HGCC HOS
01/10/202	39	30/09/202	39	SP	GVE VII SAI	1332	SANTO AN	354780	HOSPITAL I
13/10/202	41	01/10/202	39	SC	FLORIANOI	1476	FLORIANOI	420540	HOSPITAL I
17/04/202	15	15/04/202	15	SP	GVE I CAPI	1331	SAO PAULO	355030	HOSP MUN
26/04/202	17	25/04/202	17	SC	FLORIANOI	1476	SAO JOSE	421660	HOSPITAL I
03/05/202	18	19/04/202	16	RJ			VOLTA RED	330630	SECRETARI
25/05/202	21	22/05/202	20	MS	EX NRS DE	6256	CORUMBA	500320	PRONTO S(
07/06/202	23	06/06/202	23	SP	GVE I CAPI	1331	SAO PAULO	355030	UNIDADE D
15/07/202	28	05/07/202	27	PR	19RS JACAI	1373	SANTO ANT	412410	HOSPITAL I
21/08/202	33	20/08/202	33	SP	GVE I CAPI	1331	SAO PAULO	355030	HOSPITAL A
23/09/202	38	17/09/202	37	PR	19RS JACAI	1373	SANTO ANT	412410	HOSPITAL I
27/05/202	21	26/05/202	21	SP	GVE XXV SA	1349	GUARUJA	351870	HOSPITAL S
04/06/202	22	20/05/202	20	BA	NUCLEO RI	1398	VITORIA DA	293330	SAMUR
26/06/202	25	15/06/202	24	SP	GVE XXIX S	1354	CATANDUV	351110	UNIMED H
24/03/202	12	06/03/202	9	DF			BRASILIA	530010	HRSM
03/05/202	18	21/04/202	16	SP	GVE XVII CA	1342	VALINHOS	355620	HOSPITAL I
20/04/202	16	20/04/202	16	MA	REGIONAL	1440	IMPERATRI	210530	HRMI HOSI
08/06/202	23	28/05/202	21	PR	10RS CASC	1364	CASCAVEL	410480	HOSPITAL I
01/07/202	26	06/06/202	23	PR	03RS PONT	1357	JAGUARIAI	411200	HOSPITAL I
01/11/202	44	28/10/202	43	CE	11 CRES SC	1521	SOBRAL	231290	HOSPITAL I
20/04/202	16	10/04/202	14	PR	02RS METF	1356	CURITIBA	410690	HNSG
06/05/202	18	29/04/202	17	MG	DIVINOPOI	1472	FORMIGA	312610	HOSPITAL S
28/05/202	21	19/05/202	20	GO	CENTRAL	1779	GOIANIA	520870	HOSPITAL I
08/11/202	45	03/11/202	44	MG	JUIZ DE FO	1452	JUIZ DE FO	313670	HOSPITAL I
19/05/202	20	10/05/202	19	MA	REGIONAL	1440	IMPERATRI	210530	HRMI HOSI
07/06/202	23	29/05/202	21	SP	GVE X OSA	1335	OSASCO	353440	HOSPITAL I

17/04/202	15	17/04/202	15	CE	1 CRES FOF	1519	FORTALEZ/	230440	HGCC HOS
17/09/202	37	14/09/202	37	MT	AGUA BOA	1590	CANARAN/	510270	HOSPITAL I
03/09/202	35	25/08/202	34	SP	GVE XII AR/	1337	SAO CARLC	354890	SANTA CAS
10/07/202	27	02/07/202	26	MG	TEOFILO O	1465	TEOFILO O	316860	HOSPITAL !
05/03/202	9	03/03/202	9	PB	I NRS JOAC	1377	JOAO PESS	250750	MATERNID
22/04/202	16	14/04/202	15	MA	REGIONAL	1440	IMPERATRI	210530	HRMI HOSI
08/06/202	23	30/05/202	22	PR	10RS CASC	1364	CASCAVEL	410480	HOSPITAL U
04/06/202	22	29/05/202	21	SP	GVE XXIV F	1348	RIBEIRAO F	354340	HOSPITAL I
23/07/202	29	24/06/202	25	PR	16RS APUC	1370	APUCARAN	410140	HNSG HOS
28/07/202	30	21/07/202	29	PB	I NRS JOAC	1377	JOAO PESS	250750	MATERNID
22/08/202	34	10/08/202	32	SC	LAGES	1571	LAGES	420930	HOSPITAL I
28/12/202	52	03/11/202	44	SP	GVE VII SAI	1332	RIBEIRAO F	354330	HOSPITAL I
17/02/202	7	02/02/202	5	AP			MACAPA	160030	HOSPITAL I
07/05/202	18	24/04/202	16	SP	GVE I CAPI	1331	SAO PAULC	355030	HC DA FMU
23/08/202	34	05/01/202	1	RJ			RIO DE JAN	330455	HOSPITAL I
17/02/202	7	16/02/202	7	RR			BOA VISTA	140010	HOSPITAL (
12/05/202	19	01/05/202	17	PR	17RS LONC	1371	LONDRINA	411370	HOSPITAL I
31/05/202	22	19/05/202	20	BA	NUCLEO RI	1385	ILHEUS	291360	HOSPITAL !
03/03/202	9	26/02/202	8	CE	11 CRES SC	1521	SOBRAL	231290	HOSPITAL I
29/03/202	13	25/03/202	12	BA	NUCLEO RI	1380	SALVADOR	292740	MATERNID
18/03/202	11	28/02/202	9	SP	GVE I CAPI	1331	SAO PAULC	355030	PRO MATR
20/03/202	11	16/03/202	11	SP	GVE VIII M	1333	MOGI DAS	353060	MOGI MAT
13/08/202	32	06/08/202	31	MG	MONTES C	1473	MONTES C	314330	HOSPITAL U
05/03/202	9	03/02/202	5	PR	17RS LONC	1371	LONDRINA	411370	HOSPITAL I
01/06/202	22	01/06/202	22	MA	REGI0NAL	1430	SAO LUIS	211130	HOSPITAL I
17/08/202	33	04/08/202	31	MT	CUIABA	1578	CUIABA	510340	HOSPITAL U
02/06/202	22	24/05/202	21	PR	02RS METF	1356	CAMPO LA	410420	HOSPITAL I
16/07/202	28	08/07/202	27	ES	METROPOI	1510	SERRA	320500	HOSPITAL I
16/07/202	28	01/07/202	26	SP	GVE I CAPI	1331	SAO PAULC	355030	SAO LUIZ U
25/03/202	12	16/03/202	11	SP	GVE I CAPI	1331	SAO PAULC	355030	HC DA FMU
15/04/202	15	05/04/202	14	CE	1 CRES FOF	1519	FORTALEZ/	230440	HGCC HOS
18/03/202	11	06/03/202	9	AM	ENTORNO	5584	MANAUS	130260	MATERNID
31/03/202	13	14/03/202	11	PR	17RS LONC	1371	LONDRINA	411370	HOSPITAL I
17/05/202	20	15/05/202	19	SP	GVE XXI PR	1346	PRESIDENT	354140	SANTA CAS
16/06/202	24	24/05/202	21	SP	GVE I CAPI	1331	SAO PAULC	355030	HOSPITAL !
21/07/202	29	28/06/202	26	SP	GVE I CAPI	1331	SAO PAULC	355030	HOSP AVIC
14/09/202	37	09/09/202	36	RJ			RESENDE	330420	HOSPITAL !
26/03/202	12	16/03/202	11	SP	GVE I CAPI	1331	SAO PAULC	355030	SAO LUIZ U
10/04/202	14	02/04/202	13	SP	GVE I CAPI	1331	SAO PAULC	355030	HOSPITAL I
23/04/202	16	13/03/202	10	SP	GVE VIII M	1333	MOGI DAS	353060	MOGI MAT
09/08/202	32	27/07/202	30	PR	09RS FOZ D	1363	FOZ DO IGU	410830	HOSPITAL I
15/04/202	15	13/03/202	10	SP	GVE I CAPI	1331	SAO PAULC	355030	MATERNID
15/05/202	19	08/05/202	18	SP	GVE XXIX S	1354	SAO JOSE D	354980	HOSPITAL I
17/07/202	28	16/07/202	28	MG	POUSO ALI	1459	POCOS DE	315180	SANTA CAS
09/01/202	1	03/01/202	1	SP	GVE XVII C/	1342	CAMPINAS	350950	HOSPITAL I
13/05/202	19	12/05/202	19	SP	GVE XXXI S	1353	SOROCABA	355220	HOSPITAL U
02/08/202	31	10/06/202	23	BA	NUCLEO RI	1380	SALVADOR	292740	HOSPITAL .

13/01/202	2	09/01/202	1	SP	GVE I CAPI	1331	SAO PAUL	355030	HOSPITAL
06/02/202	5	24/01/202	4	GO	CENTRAL	1779	GOIANIA	520870	HOSPITAL
14/04/202	15	03/04/202	13	SP	GVE I CAPI	1331	SAO PAUL	355030	HC DA FM
15/05/202	19	15/05/202	19	SP	GVE VIII M	1333	MOGI DAS	353060	MOGI MAT
25/06/202	25	03/06/202	22	PA	01 REGION	1484	BELEM	150140	SANTA CAS
18/03/202	11	02/03/202	9	MT	CUIABA	1578	CUIABA	510340	HOSPITAL
18/03/202	11	01/03/202	9	SP	GVE VIII M	1333	GUARULH	351880	HOSPITAL
14/03/202	11	14/03/202	11	RS	002 CRS	1607	PORTO ALE	431490	HOSPITAL
01/03/202	9	01/03/202	9	AL	1 MICROR	1533	MACEIO	270430	HOSPITAL
16/04/202	15	30/03/202	13	SP	GVE I CAPI	1331	SAO PAUL	355030	MATERNID
30/06/202	26	22/06/202	25	MT	CUIABA	1578	CUIABA	510340	CENTRO DI
12/02/202	6	28/01/202	4	RJ			RIO DE JAN	330455	HOSPITAL
22/02/202	8	20/02/202	7	AL	1 MICROR	1533	MACEIO	270430	HOSPITAL
19/03/202	11	09/03/202	10	RJ			RIO DE JAN	330455	HOSPITAL
23/03/202	12	18/03/202	11	SP	GVE XVII C	1342	HORTOLAN	351907	HOSPITAL
05/01/202	1	24/11/202	47	DF			BRASILIA	530010	HOSPITAL
02/12/202	48	27/10/202	43	CE	11 CRES SC	1521	SOBRAL	231290	HOSPITAL
15/03/202	11	06/03/202	9	DF			BRASILIA	530010	HOSPITAL
19/04/202	16	08/04/202	14	SP	GVE XIX M	1344	MARILIA	352900	HCFAMEM
17/06/202	24	26/05/202	21	GO	CENTRAL	1779	GOIANIA	520870	HOSPITAL
22/02/202	8	12/02/202	6	RN	VII URSAP	1416	NATAL	240810	HOSPITAL
17/03/202	11	08/03/202	10	SP	GVE I CAPI	1331	SAO PAUL	355030	HOSPITAL
12/02/202	6	05/02/202	5	SP	GVE XXI PR	1346	PRESIDENT	354140	SANTA CAS
31/05/202	22	29/05/202	21	SP	GVE XVII C	1342	CAMPINAS	350950	HOSPITAL
15/07/202	28	07/07/202	27	SP	GVE XX PIR	1345	PIRACICAB	353870	HOSPITAL
09/08/202	32	26/07/202	30	GO	CENTRAL	1779	GOIANIA	520870	HOSPITAL
19/02/202	7	12/02/202	6	PR	02RS MET	1356	CURITIBA	410690	COMPLEX
19/03/202	11	11/03/202	10	MG	SETE LAGO	1467	SETE LAGO	316720	HOSPITAL
23/04/202	16	09/04/202	14	SP	GVE X OSA	1335	OSASCO	353440	HOSPITAL
27/05/202	21	18/05/202	20	SP	GVE I CAPI	1331	SAO PAUL	355030	HC DA FM
20/07/202	29	12/07/202	28	SP	GVE VIII M	1333	GUARULH	351880	HOSPITAL
19/05/202	20	08/04/202	14	MT	CUIABA	1578	CUIABA	510340	HOSPITAL
03/11/202	44	24/10/202	43	RS	012 CRS	1601	SANTO AN	431750	UNIMED M
25/04/202	17	16/04/202	15	CE	3 CRES MA	1513	MARACAN	230765	HOSPITAL
14/05/202	19	10/05/202	19	PR	17RS LOND	1371	LONDRINA	411370	HOSPITAL
25/06/202	25	17/06/202	24	PR	15RS MARI	1369	MARINGA	411520	SANTA CAS
29/12/202	52	22/12/202	51	PI	4 GRS TERE	1883	TERESINA	221100	MATERNID
12/03/202	10	28/02/202	9	SP	GVE XXVI S	1350	MOJI MIRI	353080	IRMANDAD
01/06/202	22	28/05/202	21	PR	02RS MET	1356	CURITIBA	410690	HOSPITAL
18/09/202	37	11/09/202	36	SP	GVE X OSA	1335	OSASCO	353440	HOSPITAL
19/04/202	16	10/04/202	14	CE	1 CRES FOR	1519	FORTALEZ	230440	HGCC HOS
01/07/202	26	22/06/202	25	SP	GVE I CAPI	1331	SAO PAUL	355030	SAO LUIZ
14/09/202	37	06/09/202	36	DF			BRASILIA	530010	HRL
04/08/202	31	25/07/202	30	PR	02RS MET	1356	SAO JOSE	412550	NOVACLIN
29/05/202	21	20/05/202	20	MA	REGI0NAL	1430	SAO LUIS	211130	MATERNID
01/11/202	44	13/10/202	41	AL	1 MICROR	1533	MACEIO	270430	SANTA CAS
02/03/202	9	26/02/202	8	MG	CORONEL	1468	CARATING	311340	CASU IRM

05/04/202	14	19/03/202	11	SP	GVE XVII C/	1342	CAMPINAS	350950	HOSPITAL I
07/06/202	23	29/03/202	13	TO			ARAGUAIN	170210	HOSPITAL I
14/05/202	19	05/05/202	18	GO	CENTRAL	1779	GOIANIA	520870	HOSPITAL I
27/07/202	30	19/07/202	29	GO	CENTRAL	1779	GOIANIA	520870	HOSPITAL I
27/10/202	43	18/10/202	42	PR	15RS MARI	1369	MARINGA	411520	SANTA CAS
31/03/202	13	19/03/202	11	SP	GVE XVII C/	1342	JUNDIAI	352590	HU HOSPIT
07/04/202	14	01/04/202	13	MG	JUIZ DE FO	1452	JUIZ DE FO	313670	HOSPITAL I
11/06/202	23	03/06/202	22	SP	GVE XXVI S	1350	MOGI GUA	353070	HOSPITAL S
24/03/202	12	03/03/202	9	SP	GVE I CAPI	1331	SAO PAULO	355030	HC DA FMU
11/04/202	15	02/04/202	13	AL	9 MICRORE	1541	PALMEIRA	270630	HOSPITAL I
02/08/202	31	16/04/202	15	PR	16RS APUC	1370	APUCARAN	410140	HNSG HOS
05/08/202	31	18/07/202	29	GO	CENTRAL	1779	GOIANIA	520870	HOSPITAL I
11/04/202	15	09/04/202	14	MA	REGIONAL	1441	BALSAS	210140	HOSPITAL I
24/06/202	25	14/06/202	24	SP	GVE XVII C/	1342	JUNDIAI	352590	INTERMED
19/03/202	11	11/03/202	10	PA	01 REGION	1484	BELEM	150140	SANTA CAS
12/03/202	10	05/03/202	9	PR	02RS METF	1356	CURITIBA	410690	HNSG
23/04/202	16	09/04/202	14	SP	GVE I CAPI	1331	SAO PAULO	355030	HC DA FMU
10/05/202	19	25/04/202	17	SP	GVE XXXI S	1353	SOROCABA	355220	SANTA CAS
06/04/202	14	31/03/202	13	SP	GVE XXIX S	1354	SAO JOSE D	354980	HOSPITAL I
03/09/202	35	27/08/202	34	PR	02RS METF	1356	CURITIBA	410690	COMPLEXO
05/07/202	27	03/07/202	26	RS	001 CRS	1592	NOVO HAN	431340	HOSPITAL I
29/03/202	13	10/03/202	10	CE	1 CRES FOF	1519	FORTALEZA	230440	HOSPITAL (
11/03/202	10	04/03/202	9	BA	NUCLEO RE	1380	SALVADOR	292740	HOSPITAL S
19/05/202	20	08/05/202	18	PR	02RS METF	1356	CAMPO LA	410420	HOSPITAL I
01/07/202	26	17/06/202	24	GO	CENTRAL	1779	GOIANIA	520870	HOSPITAL I
27/05/202	21	17/05/202	20	PR	09RS FOZ D	1363	MATELAND	411560	HOSPITAL I
11/01/202	2	03/01/202	1	PR	02RS METF	1356	CAMPO LA	410420	HOSPITAL I
21/07/202	29	29/06/202	26	PR	15RS MARI	1369	MARINGA	411520	SANTA CAS
24/02/202	8	15/02/202	7	SP	GVE XI ARA	1336	BIRIGUI	350650	SANTA CAS
04/05/202	18	24/04/202	16	SP	GVE I CAPI	1331	SAO PAULO	355030	HOSP DA S
27/07/202	30	12/07/202	28	GO	CENTRAL	1779	GOIANIA	520870	HOSPITAL I
16/03/202	11	08/03/202	10	RJ			DUQUE DE	330170	HOSPITAL (
10/03/202	10	25/02/202	8	SP	GVE X OSA	1335	OSASCO	353440	HOSPITAL I
04/05/202	18	23/04/202	16	SC	CRICIUMA	1558	CRICIUMA	420460	HOSPITAL U
22/05/202	20	17/05/202	20	BA	NUCLEO RE	1380	SALVADOR	292740	MATERNID
09/06/202	23	31/05/202	22	RS	003 CRS	1593	PELOTAS	431440	HOSPITAL I
24/02/202	8	12/02/202	6	SC	JOINVILLE	1565	JOINVILLE	420910	CENTRO H(
14/04/202	15	01/04/202	13	SP	GVE XXIV F	1348	RIBEIRAO F	354340	HOSPITAL I
30/04/202	17	24/04/202	16	SP	GVE XVII C/	1342	CAMPINAS	350950	HOSPITAL I
10/06/202	23	09/06/202	23	PA	01 REGION	1484	BELEM	150140	SANTA CAS
24/06/202	25	13/06/202	24	MG	POUSO ALE	1459	ITAJUBA	313240	AISI HOSPI
06/10/202	40	21/09/202	38	AL	1 MICRORE	1533	MACEIO	270430	SANTA CAS
25/06/202	25	12/06/202	23	RJ			RIO DE JAN	330455	HOSPITAL I
04/01/202	1	02/01/202	1	PR	09RS FOZ D	1363	FOZ DO IGU	410830	HOSPITAL I
14/01/202	2	03/01/202	1	SP	GVE X OSA	1335	BARUERI	350570	HOSPITALIS
03/02/202	5	27/01/202	4	TO			ARAGUAIN	170210	HOSPITAL I
17/01/202	3	11/01/202	2	SP	GVE VII SAI	1332	SAO CAETA	354880	HOSPITAL S

25/02/202	8	18/02/202	7	RS	003 CRS	1593	PELOTAS	431440	UCPEL HOS
10/02/202	6	06/02/202	6	PR	09RS FOZ D	1363	FOZ DO IGU	410830	HOSPITAL I
24/01/202	4	18/01/202	3	TO			ARAGUAIN	170210	HOSPITAL I
07/01/202	1	03/01/202	1	SP	GVE XII AR.	1337	SAO CARLO	354890	SANTA CAS
22/01/202	3	20/01/202	3	PR	10RS CASC	1364	CASCAVEL	410480	HOSPITAL U
14/01/202	2	09/01/202	2	MG	PATOS DE I	1456	PATOS DE I	314800	HOSPITAL I
23/01/202	4	07/01/202	1	TO			ARAGUAIN	170210	HOSPITAL I
20/01/202	3	18/01/202	3	PR	10RS CASC	1364	CASCAVEL	410480	HOSPITAL U
04/02/202	5	27/01/202	4	MT	BARRA DO	1580	BARRA DO	510180	HOSPITAL I
19/02/202	7	19/01/202	3	AM	ENTORNO	5584	MANAUS	130260	MATERNID
10/01/202	2	05/01/202	1	SP	GVE I CAPI	1331	SAO PAULO	355030	SAO LUIZ U
20/01/202	3	10/01/202	2	BA	NUCLEO RE	1380	SALVADOR	292740	HOSPITAL S
15/01/202	2	07/01/202	1	SP	GVE I CAPI	1331	SAO PAULO	355030	HOSPITAL I
21/01/202	3	20/01/202	3	PR	10RS CASC	1364	CASCAVEL	410480	HOSPITAL U
26/01/202	4	19/01/202	3	MG	PATOS DE I	1456	PATOS DE I	314800	HOSPITAL V
01/02/202	5	29/01/202	4	SC	JARAGUA D	1567	JARAGUA D	420890	HOSPITAL J
08/02/202	6	03/02/202	5	MG	PATOS DE I	1456	PATOS DE I	314800	HOSPITAL I
02/02/202	5	30/01/202	5	PA	11 REGION	1494	PARAUAPE	150553	HOSPITAL (
09/02/202	6	05/02/202	5	PR	20RS TOLEI	1374	TOLEDO	412770	HOESP
21/01/202	3	19/01/202	3	RJ			ITAPERUNA	330220	HOSPITAL S
27/01/202	4	23/01/202	4	BA	NUCLEO RE	1380	SALVADOR	292740	MATERNID
06/01/202	1	05/01/202	1	SP	GVE XXXI S	1353	SOROCABA	355220	SANTA CAS
15/01/202	2	15/01/202	2	PR	10RS CASC	1364	CASCAVEL	410480	HOSPITAL U
13/01/202	2	11/01/202	2	PA	01 REGION	1484	BELEM	150140	SANTA CAS
22/01/202	3	18/01/202	3	ES	METROPOL	1510	SERRA	320500	VITORIA AP
19/01/202	3	14/01/202	2	SC	ARARANGU	1562	ARARANGU	420140	IMAS HOSP
09/03/202	10	30/01/202	5	PR	02RS METR	1356	CAMPO LA	410420	HOSPITAL I
17/01/202	3	11/01/202	2	PR	10RS CASC	1364	CASCAVEL	410480	HOSPITAL U
22/01/202	3	21/01/202	3	PR	10RS CASC	1364	CASCAVEL	410480	HOSPITAL U
04/02/202	5	01/02/202	5	PA	01 REGION	1484	BELEM	150140	SANTA CAS
27/01/202	4	24/01/202	4	PR	09RS FOZ D	1363	FOZ DO IGU	410830	HOSPITAL I
15/01/202	2	11/01/202	2	BA	NUCLEO RE	1380	SALVADOR	292740	MATERNID
11/03/202	10	16/02/202	7	PR	02RS METR	1356	CAMPO LA	410420	HOSPITAL I
26/01/202	4	12/01/202	2	SP	GVE I CAPI	1331	SAO PAULO	355030	MATERNID
24/01/202	4	13/01/202	2	SP	GVE I CAPI	1331	SAO PAULO	355030	HOSPITAL S
06/01/202	1	04/01/202	1	SP	GVE XIII AS	1338	ASSIS	350400	HOSPITAL I
17/01/202	3	17/01/202	3	SC	CRICIUMA	1558	CRICIUMA	420460	HOSPITAL I
07/01/202	1	05/01/202	1	RJ			RIO DE JAN	330455	MS HSE HC
15/01/202	2	15/01/202	2	PR	10RS CASC	1364	CASCAVEL	410480	HOSPITAL U
25/02/202	8	21/02/202	8	SP	GVE XXVII S	1351	SAO JOSE D	354990	HOSPITAL V
16/03/202	11	09/03/202	10	SP	GVE XXII PI	1573	DRACENA	351440	SANTA CAS
10/01/202	2	05/01/202	1	PI	4 GRS TERE	1883	TERESINA	221100	MATERNID
20/01/202	3	04/01/202	1	PB	I NRS JOAO	1377	JOAO PESS	250750	MATERNID
17/01/202	3	17/01/202	3	RS	001 CRS	1592	NOVO HAM	431340	FUNDACAO
08/02/202	6	06/02/202	6	MG	PATOS DE I	1456	PATOS DE I	314800	HOSPITAL I
17/01/202	3	10/01/202	2	RJ			RIO DE JAN	330455	HOSPITAL (
24/01/202	4	11/01/202	2	SP	GVE VII SAI	1332	SAO CAETA	354880	HOSPITAL S

19/01/202	3	17/01/202	3	PR	10RS CASC	1364	CASCAVEL	410480	HOSPITAL U
19/01/202	3	17/01/202	3	PR	09RS FOZ D	1363	FOZ DO IGU	410830	HOSPITAL I
28/02/202	9	20/01/202	3	MG	MANHUAC	1454	MANHUAC	313940	HOSPITAL (
14/01/202	2	12/01/202	2	SP	GVE XXVII S	1351	SAO JOSE D	354990	HOSPITAL I
12/01/202	2	11/01/202	2	RJ			RIO DE JAN	330455	HOSPITAL (
19/01/202	3	16/01/202	3	RJ			RIO DE JAN	330455	HOSPITAL I
26/01/202	4	26/01/202	4	MG	UBERLAND	1462	UBERLAND	317020	UAI MARTI
11/01/202	2	08/01/202	1	RJ			NILOPOLIS	330320	DOMINGO
20/01/202	3	17/01/202	3	SP	GVE XVII C	1342	JUNDIAI	352590	HOSPITAL I
25/01/202	4	23/01/202	4	GO	CENTRAL	1779	GOIANIA	520870	HOSPITAL I
17/01/202	3	12/01/202	2	BA	NUCLEO RI	1380	SALVADOR	292740	MATERNID
17/01/202	3	10/01/202	2	PI	4 GRS TERE	1883	TERESINA	221100	ASSOCIACA
28/01/202	4	21/01/202	3	AP			SANTANA	160060	HOSPITAL I
11/01/202	2	05/01/202	1	RJ			RIO DE JAN	330455	SMS HOSPI
16/02/202	7	10/02/202	6	SP	GVE VII SAI	1332	RIBEIRAO P	354330	HOSPITAL I
18/02/202	7	02/02/202	5	SP	GVE I CAPIT	1331	SAO PAULO	355030	SAO LUIZ U
17/02/202	7	02/02/202	5	SP	GVE I CAPIT	1331	SAO PAULO	355030	SAO LUIZ U
26/01/202	4	10/01/202	2	SP	GVE VII SAI	1332	SAO CAETA	354880	HOSPITAL S
09/02/202	6	01/02/202	5	SP	GVE XXI PR	1346	PRESIDENT	354140	HOSPITAL I
20/01/202	3	10/01/202	2	MA	REGIONAL	1440	IMPERATRI	210530	HRMI HOSI
10/01/202	2	04/01/202	1	SP	GVE I CAPIT	1331	SAO PAULO	355030	HOSPITAL S
17/01/202	3	17/01/202	3	SP	GVE XX PIR	1345	ARARAS	350330	HOSPITAL S
20/02/202	8	13/02/202	7	PB	III NRS CAN	1421	CAMPINA (	250400	HOSPITAL I
25/01/202	4	19/01/202	3	SP	GVE XVI BC	1341	SAO MANU	355010	HOSPITAL I
23/01/202	4	20/01/202	3	PR	10RS CASC	1364	CASCAVEL	410480	HOSPITAL U
24/01/202	4	14/01/202	2	SP	GVE VII SAI	1332	SAO CAETA	354880	HOSPITAL S
19/01/202	3	17/01/202	3	PR	10RS CASC	1364	CASCAVEL	410480	HOSPITAL U
13/01/202	2	06/01/202	1	GO	CENTRAL	1779	GOIANIA	520870	HOSPITAL J
07/02/202	6	09/01/202	2	SP	GVE VII SAI	1332	SAO BERNA	354870	HOSPITAL I
10/01/202	2	05/01/202	1	RJ			ITAPERUNA	330220	HOSPITAL S
27/01/202	4	24/01/202	4	PR	09RS FOZ D	1363	FOZ DO IGU	410830	HOSPITAL I
16/01/202	3	12/01/202	2	SP	GVE VIII M	1333	GUARULHO	351880	HOSPITAL I
04/02/202	5	28/01/202	4	MA	REGIONAL	1440	IMPERATRI	210530	HRMI HOSI
14/02/202	7	25/01/202	4	RJ			RIO DE JAN	330455	MS HSE HC
31/01/202	5	31/01/202	5	MG	BELO HORI	1449	BELO HORI	310620	MATERNID
17/01/202	3	12/01/202	2	SP	GVE I CAPIT	1331	SAO PAULO	355030	HOSP MUN
22/01/202	3	14/01/202	2	MG	VARGINHA	1466	VARGINHA	317070	HOSPITAL I
30/01/202	5	29/01/202	4	RJ			ITAPERUNA	330220	HOSPITAL S
20/01/202	3	16/01/202	3	PR	09RS FOZ D	1363	FOZ DO IGU	410830	HOSPITAL I
27/01/202	4	25/01/202	4	PA	11 REGION	1494	MARABA	150420	HOSPITAL I
23/01/202	4	15/01/202	2	AP			SANTANA	160060	HOSPITAL I
22/02/202	8	13/02/202	7	GO	CENTRAL	1779	GOIANIA	520870	HOSPITAL I
02/02/202	5	28/01/202	4	MG	BELO HORI	1449	BELO HORI	310620	MATERNID
12/01/202	2	11/01/202	2	MG	VARGINHA	1466	AIURUOCA	310120	HOSPITAL S
21/02/202	8	13/02/202	7	CE	8 CRES QU	1517	QUIXERAM	231140	HOSPITAL I
21/02/202	8	16/02/202	7	SP	GVE XIX MA	1344	MARILIA	352900	HCFAMEM
20/01/202	3	17/01/202	3	RJ			ITAPERUNA	330220	HOSPITAL S

17/01/202	3	16/01/202	3	SP	GVE I CAPI	1331	SAO PAULO	355030	HOSPITAL (
19/01/202	3	17/01/202	3	PR	09RS FOZ D	1363	FOZ DO IGU	410830	HOSPITAL I
23/01/202	4	18/01/202	3	PR	10RS CASC	1364	CASCAVEL	410480	HOSPITAL U
17/01/202	3	10/01/202	2	SP	GVE VII SAI	1332	RIBEIRAO P	354330	HOSPITAL I
12/02/202	6	08/02/202	6	PI	10 GRS FLO	1889	FLORIANO	220390	HOSPITAL I
07/02/202	6	01/02/202	5	SP	GVE I CAPI	1331	SAO PAULO	355030	PRO MATR
12/02/202	6	17/01/202	3	PR	17RS LOND	1371	LONDRINA	411370	HOSPITAL U
13/01/202	2	02/01/202	1	MG	PATOS DE I	1456	PATOS DE I	314800	HOSPITAL I
27/01/202	4	27/01/202	4	SP	GVE XXVII S	1351	JACAREI	352440	HOSPITAL S
04/02/202	5	01/02/202	5	SC	CRICIUMA	1558	ICARA	420700	FUNDACAO
04/02/202	5	30/01/202	5	SP	GVE XVII CA	1342	CAMPINAS	350950	HOSPITAL I
10/02/202	6	05/02/202	5	BA	NUCLEO RE	1380	SALVADOR	292740	MATERNID
21/02/202	8	12/02/202	6	SC	LAGES	1571	LAGES	420930	HOSPITAL I
20/01/202	3	20/01/202	3	RS	007 CRS	1596	BAGE	430160	SANTA CAS
09/02/202	6	31/01/202	5	SP	GVE XXXII I	1575	ITAPEVA	352240	SANTA CAS
23/02/202	8	19/01/202	3	AP			SANTANA	160060	HOSPITAL I
17/01/202	3	13/01/202	2	SP	GVE XIX MA	1344	MARILIA	352900	HOSPITAL U
23/01/202	4	18/01/202	3	PR	10RS CASC	1364	CASCAVEL	410480	HOSPITAL U
28/03/202	13	21/03/202	12	RS	001 CRS	1592	SAO LEOPO	431870	HOSPITAL (
23/01/202	4	19/01/202	3	MS	EX NRS DE	6256	CORUMBA	500320	PRONTO SO
03/01/202	1	02/01/202	1	MG	POUSO ALE	1459	POCOS DE	315180	SANTA CAS
20/01/202	3	08/01/202	1	SP	GVE I CAPI	1331	SAO PAULO	355030	HC DA FMU
30/01/202	5	25/01/202	4	SP	GVE VII SAI	1332	SANTO AN	354780	HOSPITAL I

O_UNI_NO	CS_SEXO	DT_NASC	IU_IDADE_	TP_IDADE	COD_IDADE	CS_GESTAN	CS_RACA	CS_ESCOL_	ID_PAIS
5407591	F	22/01/199	28	3	3028	3	4	3	BRASIL
3151794	F	10/03/197	42	3	3042	2	4	3	BRASIL
2299216	F	19/09/199	29	3	3029	3	1		BRASIL
2298120	F	09/10/199	26	3	3026	3	1	9	BRASIL
2083116	F	02/04/198	37	3	3037	2	1	3	BRASIL
2083981	F	21/01/199	29	3	3029	2	4	2	BRASIL
2078015	F	06/10/198	32	3	3032	2	1	9	BRASIL
2298317	F	07/04/200	18	3	3018	1	4	9	BRASIL
2371375	F	08/01/199	24	3	3024	1	1		BRASIL
5073898	F	10/10/197	44	3	3044	3	4	9	BRASIL
2079186	F	21/05/199	30	3	3030	3	9	9	BRASIL
9725	F	22/12/200	19	3	3019	3	4	2	BRASIL
3151794	F	15/01/197	42	3	3042	2	1	4	BRASIL
6159397	F	02/03/198	39	3	3039	3	9		BRASIL
9065768	F	02/07/200	20	3	3020	2	4	9	BRASIL
2077493	F	29/05/199	29	3	3029	2	1		BRASIL
2079186	F	29/12/198	37	3	3037	2	1	2	BRASIL
6848710	F	13/03/199	29	3	3029	3			BRASIL
3366219	F	11/04/199	30	3	3030	1	9	9	BRASIL
2338424	F	19/10/199	22	3	3022	3	4		BRASIL
5462835	F	05/02/198	36	3	3036	3	2	3	BRASIL
2319659	F	20/10/197	41	3	3041	4	4	4	BRASIL
2298317	F	12/09/199	28	3	3028	3	9	9	BRASIL
2708566	F	13/12/198	33	3	3033	2	1	3	BRASIL
2499363	F	24/12/199	23	3	3023	3			BRASIL
3012212	F	13/10/199	20	3	3020	4	1		BRASIL
6558143	F	05/07/198	30	3	3030	3			BRASIL
2079798	F	25/03/198	39	3	3039	3	1	3	BRASIL
434	F	06/04/199	30	3	3030	2	4	9	BRASIL
5073898	F	06/02/199	27	3	3027	3	4		BRASIL
2078015	F	29/06/199	23	3	3023	2	4	9	BRASIL
2077396	F	30/05/198	36	3	3036	3	2	3	BRASIL
7818858	F	12/08/198	37	3	3037	1	1	9	BRASIL
6601804	F	23/05/200	19	3	3019	4	4	9	BRASIL
3956369	F	16/01/198	36	3	3036	3	4		BRASIL
2097613	F	14/01/199	26	3	3026	3	1	4	BRASIL
2339196	F	18/12/198	31	3	3031	2	1	3	BRASIL
2078678	F	21/03/199	25	3	3025	3	2	9	BRASIL
2077485	F	26/07/197	40	3	3040	3	1	9	BRASIL
434	F	05/12/198	33	3	3033	2	4		BRASIL
2022621	F	07/04/198	32	3	3032	2	4	3	BRASIL
2012480	F	19/04/199	28	3	3028	3	4	3	BRASIL
2688611	F	01/08/198	31	3	3031	3		4	BRASIL
3367371	F	29/01/199	30	3	3030	3	4		BRASIL
6995462	F	21/12/198	34	3	3034	3	1	9	BRASIL
9923837	F	07/10/198	31	3	3031	1	4	9	BRASIL

2079798	F	31/12/200	19	3	3019	2	1	3	BRASIL
2078082	F	16/09/198	33	3	3033	3	1	9	BRASIL
2655411	F	10/12/198	34	3	3034	3	4		BRASIL
2006197	F	17/11/199	29	3	3029	3	4	2	BRASIL
3956369	F	14/03/199	30	3	3030	3	4	9	BRASIL
2752700	F	15/06/198	30	3	3030	3	4		BRASIL
2522101	F	24/02/198	33	3	3033	3	1	3	BRASIL
2275562	F	18/01/199	22	3	3022	1	4		BRASIL
434	F	26/04/199	23	3	3023	2	1		BRASIL
6542638	F	02/08/199	23	3	3023	1	1	1	BRASIL
2078015	F	17/09/198	38	3	3038	3			BRASIL
3956369	F	18/12/199	24	3	3024	2	4	9	BRASIL
2079542	F	10/08/198	36	3	3036	3	4	4	BRASIL
3956369	F	10/05/197	41	3	3041	3	4		BRASIL
2027356	F	18/03/198	34	3	3034	2	2	3	BRASIL
2079186	F	11/07/199	30	3	3030	3	1	3	BRASIL
2237601	F	09/09/199	29	3	3029	3	1	3	BRASIL
2615673	F	25/07/198	35	3	3035	3			BRASIL
2499363	F	13/07/199	20	3	3020	3	4	9	BRASIL
2772310	F	25/01/199	30	3	3030	1	1	9	BRASIL
2236370	F	23/11/199	27	3	3027	3	1	2	BRASIL
3521133	F	22/02/198	35	3	3035	3	1	3	BRASIL
2079542	F	05/11/198	31	3	3031	2	1	2	BRASIL
12505	F	10/11/199	29	3	3029	3	2	9	BRASIL
2765934	F	05/11/198	35	3	3035	2	1	1	BRASIL
3956369	F	20/10/199	26	3	3026	3	4	9	BRASIL
15369	F	12/01/199	22	3	3022	2	1		BRASIL
2269988	F	17/12/198	34	3	3034	2	9	9	BRASIL
2079542	F	22/07/198	39	3	3039	2	9		BRASIL
2298120	F	15/04/198	31	3	3031	3	4	9	BRASIL
9624864	F	23/10/198	33	3	3033	3	1	4	BRASIL
6159397	F	23/04/198	35	3	3035	3	9	4	BRASIL
2481286	F	07/10/199	24	3	3024	3	4	2	BRASIL
2415488	F	08/03/199	30	3	3030	3	1	4	BRASIL
12521	F	16/12/198	35	3	3035	2	4	9	BRASIL
6817203	F	24/12/199	29	3	3029	3	4	3	BRASIL
2363070	F	27/09/198	34	3	3034	2	1	9	BRASIL
3808	F	27/01/198	39	3	3039	1	9	9	BRASIL
2655411	F	27/06/198	39	3	3039	2	4	9	BRASIL
2007037	F	09/08/197	44	3	3044	2	9	9	BRASIL
2078015	F	10/12/199	21	3	3021	2	9	9	BRASIL
2399644	F	11/12/198	38	3	3038	3	4	3	BRASIL
27022	F	26/03/199	30	3	3030	3	4		BRASIL
10464	F	12/01/198	40	3	3040	3	2	9	BRASIL
3000869	F	06/02/199	30	3	3030	2	1	3	BRASIL
2288893	F	06/10/198	37	3	3037	2	2		BRASIL
2783789	F	30/05/198	33	3	3033	1	1		BRASIL

2707527	F	20/07/198	36	3	3036	3	4	4	BRASIL
2089602	F	13/03/198	31	3	3031	2	1	4	BRASIL
3046281	F	11/03/198	31	3	3031	2	4	9	BRASIL
2078015	F	21/06/199	25	3	3025	3	1	9	BRASIL
2611686	F	06/04/198	31	3	3031	3	4	4	BRASIL
2022621	F	04/06/198	36	3	3036	3	1	2	BRASIL
2097613	F	25/06/199	29	3	3029	3	1	4	BRASIL
2078732	F	20/03/199	24	3	3024	2	1	9	BRASIL
2362287	F	10/02/199	28	3	3028	2	4	1	BRASIL
2079542	F	10/02/197	41	3	3041	2	9	4	BRASIL
3808	F	14/06/198	33	3	3033	2	9	9	BRASIL
2078066	F	24/12/198	30	3	3030	3	9	9	BRASIL
2078015	F	08/10/198	35	3	3035	2	4	9	BRASIL
2078015	F	19/08/199	28	3	3028	2	1	9	BRASIL
2089602	F	25/03/199	22	3	3022	3	4		BRASIL
434	F	16/06/199	23	3	3023	3	4	3	BRASIL
176664	F	08/02/198	34	3	3034	3	4	3	BRASIL
2786117	F	02/09/198	34	3	3034	3	4	4	BRASIL
2232146	F	19/01/197	42	3	3042	2	1	9	BRASIL
2080273	F	22/11/198	34	3	3034	3	1	3	BRASIL
2077396	F	13/08/198	32	3	3032	1	1	3	BRASIL
2297795	F	22/04/199	23	3	3023	1	1	1	BRASIL
2789590	F	20/05/199	24	3	3024	3	1	9	BRASIL
9554157	F	03/04/197	41	3	3041	3	4	9	BRASIL
5717256	F	21/03/198	34	3	3034	3	1	3	BRASIL
2089777	F	01/10/198	33	3	3033	3	1	9	BRASIL
2655411	F	27/10/198	37	3	3037	2	4	3	BRASIL
3058336	F	04/08/199	22	3	3022	2	4		BRASIL
2077396	F	06/05/198	36	3	3036	2	1	4	BRASIL
2089602	F	09/09/199	27	3	3027	1	1	9	BRASIL
6159397	F	18/06/198	37	3	3037	2	9		BRASIL
2012480	F	08/02/198	36	3	3036	3	4	2	BRASIL
3005402	F	14/11/199	23	3	3023	3	9	9	BRASIL
2237253	F	22/01/198	36	3	3036	3	1		BRASIL
176664	F	26/04/199	29	3	3029	3	2	1	BRASIL
2298317	F	14/02/199	25	3	3025	3	4	9	BRASIL
2275635	F	02/12/198	37	3	3037	3	1	9	BRASIL
2752700	F	24/02/198	33	3	3033	3	2	9	BRASIL
2499363	F	28/03/198	34	3	3034	3			BRASIL
2338424	F	10/01/198	31	3	3031	3	4		BRASIL
3956369	F	20/12/200	14	3	3014	2	9	9	BRASIL
14109	F	18/01/198	34	3	3034	3	1	4	BRASIL
2823209	F	01/01/198	39	3	3039	2	1	2	BRASIL
2269988	F	15/11/198	31	3	3031	3	4	9	BRASIL
9543856	F	25/04/198	33	3	3033	3	1	9	BRASIL
2079666	F	24/09/197	43	3	3043	3	1	3	BRASIL
2615797	F	27/03/199	21	3	3021	2	1	3	BRASIL

2752700	F	25/05/1999	21	3	3021	3	4	2	BRASIL
2338424	F	19/06/1989	31	3	3031	2	9		BRASIL
2436450	F	28/04/1987	33	3	3033	3	1		BRASIL
7659415	F	14/06/1988	32	3	3032	3	4	9	BRASIL
6984649	F	22/05/1991	29	3	3029	3	4	9	BRASIL
9115153	F	10/03/1987	33	3	3033	3	4	3	BRASIL
2078015	F	13/03/1992	25	3	3025	3	4	9	BRASIL
2298120	F	08/04/1992	25	3	3025	3	1		BRASIL
2078015	F	27/04/1972	45	3	3045	2	1	9	BRASIL
2019574	F	28/11/2002	16	3	3016	1	5	9	BRASIL
7244339	F	04/08/1989	30	3	3030	4	9	9	BRASIL
2079186	F	13/04/1998	22	3	3022	2	1	3	BRASIL
3151794	F	06/10/1982	38	3	3038	2	4		BRASIL
2081539	F	18/01/1992	27	3	3027	2	1	3	BRASIL
2707527	F	04/02/1986	34	3	3034	3	1	2	BRASIL
2078015	F	08/05/1999	21	3	3021	2	1	9	BRASIL
5073898	F	27/01/1992	25	3	3025	3	1	3	BRASIL
6848710	F	19/03/1980	40	3	3040	3	9		BRASIL
2082926	F	21/03/1990	30	3	3030	3	1	4	BRASIL
6194168	F	16/09/1980	39	3	3039	3	1		BRASIL
2521385	F	22/10/198	38	3	3038	2	1	3	BRASIL
2097613	F	24/06/198	39	3	3039	3	1	4	BRASIL
2089602	F	29/07/1990	29	3	3029	1			BRASIL
3956369	F	30/03/1988	32	3	3032	3	4	9	BRASIL
434	F	28/09/199	28	3	3028	3	4		BRASIL
2077396	F	12/09/1994	25	3	3025	3	1	9	BRASIL
9336508	F	09/12/1984	35	3	3035	3	3	9	BRASIL
3018520	F	02/09/1980	39	3	3039	2	9	9	BRASIL
2077396	F	08/03/1982	35	3	3035	2	1	3	BRASIL
2439360	F	27/12/1990	23	3	3023	2	1	9	BRASIL
2077485	F	14/08/1982	35	3	3035	3	2	1	BRASIL
2269988	F	23/04/1990	30	3	3030	3	9	9	BRASIL
2078015	F	10/09/1984	36	3	3036	2	1	9	BRASIL
2708566	F	07/07/1982	37	3	3037	2	9	9	BRASIL
2537788	F	12/07/1984	35	3	3035	3	1	3	BRASIL
3151794	F	25/05/1982	35	3	3035	3	4	3	BRASIL
2078015	F	03/01/1982	35	3	3035	3	9	9	BRASIL
3045625	F	21/02/1972	48	3	3048	2	1	9	BRASIL
2707527	F	08/05/1988	32	3	3032	3	4	2	BRASIL
2082128	F	04/12/1984	35	3	3035	2	1	9	BRASIL
2525569	F	15/12/2000	19	3	3019	3	4		BRASIL
3018520	F	05/02/1988	32	3	3032	2	9	9	BRASIL
2252287	F	04/02/1989	31	3	3031	3	1	3	BRASIL
6988938	F	19/02/1988	32	3	3032	2			BRASIL
6995462	F	20/01/1999	21	3	3021	3	4	9	BRASIL
2688689	F	10/11/1992	27	3	3027	2	9	9	BRASIL
3212130	F	16/04/200	19	3	3019	3	1	9	BRASIL

2323397	F	06/01/199	28	3	3028	3	9		BRASIL
2604388	F	01/04/198	35	3	3035	3	1	4	BRASIL
2038730	F	07/06/197	41	3	3041	2	2	9	BRASIL
2252287	F	21/07/198	38	3	3038	3	1	3	BRASIL
2070413	F	16/09/199	23	3	3023	2	1	3	BRASIL
2193310	F	26/04/198	39	3	3039	4	4	3	BRASIL
2065665	F	23/08/200	17	3	3017	2	1		BRASIL
2025523	F	01/12/198	31	3	3031	3	1	4	BRASIL
2707527	F	20/11/198	30	3	3030	3	4	3	BRASIL
2308983	F	14/02/198	32	3	3032	2	4	4	BRASIL
2679663	F	27/01/199	28	3	3028	1	9	2	BRASIL
2254611	F	05/02/200	19	3	3019	2	1	2	BRASIL
4013484	F	28/10/198	34	3	3034	1	4	9	BRASIL
2410281	F	08/07/198	30	3	3030	1	1	3	BRASIL
9554157	F	07/01/199	26	3	3026	1		9	BRASIL
2254964	F	13/01/198	38	3	3038	1	1	3	BRASIL
3000869	F	07/09/198	32	3	3032	1	1	3	BRASIL
2078015	F	25/10/198	39	3	3039	3	1	9	BRASIL
2726653	F	21/08/198	32	3	3032	2	4	3	BRASIL
2153882	F	26/04/198	35	3	3035	4	3		BRASIL
2361787	F	17/03/198	31	3	3031	3	4		BRASIL
3151794	F	15/04/200	17	3	3017	3	4		BRASIL
5498465	F	01/04/199	30	3	3030	3	4	3	BRASIL
2126451	F	29/03/199	24	3	3024	3	9	9	BRASIL
2772310	F	20/12/198	30	3	3030	2	1	2	BRASIL
2604388	F	14/04/198	33	3	3033	3	4	9	BRASIL
6848710	F	23/08/198	34	3	3034	1	4	9	BRASIL
2338424	F	19/02/198	37	3	3037	3	9		BRASIL
2206595	F	02/04/197	45	3	3045	1	1	3	BRASIL
3151794	F	07/05/198	31	3	3031	3	4	1	BRASIL
2550555	F	25/12/198	36	3	3036	3	4	4	BRASIL
2707527	F	30/09/198	31	3	3031	3	4	3	BRASIL
2269988	F	21/08/198	35	3	3035	3	9	9	BRASIL
10464	F	28/07/197	42	3	3042	3	9	9	BRASIL
2339196	F	20/03/199	22	3	3022	3	4	3	BRASIL
6985483	F	08/04/197	44	3	3044	4			BRASIL
2781859	F	18/10/199	26	3	3026	2	1	9	BRASIL
2499363	F	15/04/199	22	3	3022	3	4	9	BRASIL
2408570	F	04/11/198	33	3	3033	3	4	9	BRASIL
2027356	F	30/01/199	27	3	3027	3	1	3	BRASIL
2298120	F	18/05/198	34	3	3034	2	4	9	BRASIL
5717515	F	02/10/198	38	3	3038	2	4	9	BRASIL
2270609	F	02/03/198	39	3	3039	3	4	3	BRASIL
7659415	F	17/06/199	26	3	3026	2	4	9	BRASIL
2078015	F	13/08/199	29	3	3029	3	1	9	BRASIL
2688611	F	18/09/199	30	3	3030	2	9	4	BRASIL
2751623	F	23/06/198	36	3	3036	2	1	3	BRASIL

2767384	F	18/07/198	38	3	3038	3	4	3	BRASIL
10464	F	20/04/198	33	3	3033	3	9		BRASIL
3151794	F	19/07/199	22	3	3022	3	4		BRASIL
5907594	F	20/04/198	39	3	3039	3	9		BRASIL
2270714	F	09/11/198	38	3	3038	2	1	9	BRASIL
2815966	F	24/03/197	41	3	3041	3	9	9	BRASIL
9554157	F	09/06/198	32	3	3032	3	1	9	BRASIL
2077396	F	08/12/198	34	3	3034	3	1	4	BRASIL
2006197	F	30/06/200	18	3	3018	3	4	2	BRASIL
9554157	F	25/09/199	27	3	3027	3	9		BRASIL
2257548	F	06/09/198	31	3	3031	2	1		BRASIL
5073898	F	31/10/198	36	3	3036	3	4	4	BRASIL
2159252	F	18/10/199	24	3	3024	2	9		BRASIL
2688611	F	27/11/198	33	3	3033	3	1	4	BRASIL
2319659	F	01/03/199	25	3	3025	2	5	9	BRASIL
2339196	F	02/03/199	22	3	3022	2	9	9	BRASIL
5585422	F	29/09/199	24	3	3024	3	4	2	BRASIL
2208172	F	14/10/199	29	3	3029	3	4	9	BRASIL
9725	F	09/08/198	36	3	3036	2	4	1	BRASIL
5585422	F	08/02/199	29	3	3029	2	4	3	BRASIL
2687127	F	21/10/199	28	3	3028	3	1	3	BRASIL
7257406	F	17/08/197	41	3	3041	3	4		BRASIL
2269988	F	11/01/198	39	3	3039	2	1	9	BRASIL
2079186	F	07/11/199	28	3	3028	3	1	0	BRASIL
7659415	F	29/09/199	27	3	3027	1	4	9	BRASIL
2275287	F	23/04/199	30	3	3030	3	9	9	BRASIL
2802082	F	14/07/198	37	3	3037	3	4		BRASIL
3557081	F	11/01/199	26	3	3026	4	1	9	BRASIL
2040069	F	09/10/199	30	3	3030	2	1	4	BRASIL
5073898	F	25/03/199	30	3	3030	2	4	3	BRASIL
2091585	F	28/05/200	19	3	3019	4	1	2	BRASIL
9923837	F	10/12/199	25	3	3025	3	4	9	BRASIL
2655411	F	27/07/198	36	3	3036	2	4		BRASIL
2499363	F	12/09/198	37	3	3037	3	4		BRASIL
2804034	F	28/11/198	38	3	3038	2	9		BRASIL
2339196	F	11/01/199	21	3	3021	3	4	3	BRASIL
2655411	F	29/08/198	36	3	3036	1	3	9	BRASIL
7977123	F	03/02/199	26	3	3026	2	1	3	BRASIL
2078015	F	31/03/198	37	3	3037	3	9	3	BRASIL
2823209	F	26/11/198	32	3	3032	2	1		BRASIL
2816210	F	02/11/198	31	3	3031	3	9	9	BRASIL
2655411	F	29/06/199	30	3	3030	3	4	3	BRASIL
5158044	F	30/11/199	25	3	3025	3	4	3	BRASIL
2082888	F	10/10/199	21	3	3021	3	4	3	BRASIL
2025760	F	07/06/198	33	3	3033	3	2	4	BRASIL
3037274	F	22/04/198	35	3	3035	2	9	9	BRASIL
6668143	F	27/08/198	37	3	3037	3	9	9	BRASIL

434	F	11/08/200	19	3	3019	3	9	9	BRASIL
2688611	F	05/05/198	36	3	3036	2		4	BRASIL
2153882	F	25/11/200	18	3	3018	3	1	2	BRASIL
2604388	F	05/09/199	24	3	3024	3	2	4	BRASIL
2278855	F	26/05/198	31	3	3031	2	4	9	BRASIL
103284	F	03/03/197	41	3	3041	3	4	9	BRASIL
3531228	F	23/10/197	48	3	3048	2	1		BRASIL
5907594	F	09/11/198	35	3	3035	2	9	9	BRASIL
2655411	F	03/08/199	28	3	3028	3	4		BRASIL
2611686	F	29/07/199	25	3	3025	2	4	9	BRASIL
6086381	F	10/06/198	33	3	3033	3	1	9	BRASIL
10464	F	05/05/199	27	3	3027	2	9	9	BRASIL
6995462	F	31/07/200	20	3	3020	2	4	9	BRASIL
2338424	F	26/02/198	32	3	3032	1	4		BRASIL
3151794	F	07/08/198	32	3	3032	2	4		BRASIL
2078015	F	03/07/200	20	3	3020	3	1	9	BRASIL
2232022	F	13/12/197	40	3	3040	1	2	3	BRASIL
2278855	F	15/12/199	26	3	3026	2	9	9	BRASIL
3956369	F	20/09/199	29	3	3029	3	9		BRASIL
2078015	F	28/04/198	33	3	3033	1	1	9	BRASIL
2077396	F	09/09/198	36	3	3036	2	1	3	BRASIL
2754738	F	30/12/198	39	3	3039	2	1		BRASIL
2087715	F	14/12/199	22	3	3022	2	4	3	BRASIL
2786117	F	30/05/200	14	3	3014	2	5	1	BRASIL
9554157	F	10/08/198	33	3	3033	3	9	9	BRASIL
2077493	F	14/02/199	28	3	3028	3	4	9	BRASIL
2076896	F	02/11/200	19	3	3019	2	9		BRASIL
7421710	F	17/02/199	22	3	3022	2	9	9	BRASIL
2679663	F	28/08/199	28	3	3028	2	1	3	BRASIL
3956369	F	04/09/198	32	3	3032	1	4		BRASIL
2237598	F	05/11/198	32	3	3032	2	1	3	BRASIL
2066092	F	26/09/199	26	3	3026	1	4	2	BRASIL
9554157	F	07/05/199	27	3	3027	3	9	9	BRASIL
2427427	F	14/03/199	23	3	3023	3	4	9	BRASIL
3151794	F	11/05/198	31	3	3031	2	4		BRASIL
3151794	F	10/06/199	26	3	3026	1	4		BRASIL
6518893	F	19/06/199	24	3	3024	2	1	9	BRASIL
2270714	F	21/08/199	28	3	3028	3	4	9	BRASIL
2205440	F	22/12/197	42	3	3042	3	4	3	BRASIL
3394514	F	09/12/198	34	3	3034	3			BRASIL
2269988	F	17/04/198	39	3	3039	3	1	9	BRASIL
2338424	F	11/07/199	27	3	3027	2			BRASIL
9727353	F	27/06/198	32	3	3032	4	4	9	BRASIL
3956369	F	01/05/198	39	3	3039	1	4		BRASIL
2323397	F	07/02/197	41	3	3041	3	9		BRASIL
3972976	F	17/11/199	25	3	3025	2	1	9	BRASIL
2087715	F	07/02/198	37	3	3037	3	1	9	BRASIL

6984649	F	25/05/199	22	3	3022	3	4	9	BRASIL
2078473	F	05/02/198	37	3	3037	3	4	9	BRASIL
5712068	F	11/06/198	38	3	3038	3	1	4	BRASIL
2655411	F	04/02/200	16	3	3016	3	4	3	BRASIL
2269880	F	01/11/199	24	3	3024	3	1		BRASIL
5073898	F	16/01/199	26	3	3026	3	1	9	BRASIL
3019608	F	19/10/199	29	3	3029	2	9	9	BRASIL
5585422	F	28/10/198	39	3	3039	2	4	2	BRASIL
2084090	F	02/08/197	41	3	3041	2	1	9	BRASIL
2126494	F	13/05/199	23	3	3023	3	9	9	BRASIL
9923837	F	28/09/198	35	3	3035	3	4	9	BRASIL
2252287	F	14/02/198	38	3	3038	3	2	2	BRASIL
2078015	F	11/10/198	36	3	3036	3	1	3	BRASIL
3151794	F	22/11/198	33	3	3033	3	4	3	BRASIL
2499363	F	29/10/199	23	3	3023	3			BRASIL
2237571	F	07/04/198	33	3	3033	3	1	4	BRASIL
2781859	F	10/10/198	33	3	3033	3	1	9	BRASIL
3058336	F	25/12/198	37	3	3037	3	4	9	BRASIL
6668143	F	10/12/198	35	3	3035	3	9		BRASIL
5407591	F	24/03/198	39	3	3039	3	9		BRASIL
2078015	F	31/10/199	26	3	3026	3	1	9	BRASIL
2079542	F	12/01/198	32	3	3032	2	9	9	BRASIL
2384299	F	30/10/197	42	3	3042	3	1	9	BRASIL
2089602	F	01/11/199	21	3	3021	3	1	9	BRASIL
176664	F	30/09/198	34	3	3034	1	4	9	BRASIL
2078015	F	03/01/199	25	3	3025	3	4	9	BRASIL
3013162	F	06/09/198	33	3	3033	2	9	9	BRASIL
2022621	F	20/03/200	17	3	3017	2	1	2	BRASIL
2306344	F	06/05/199	23	3	3023	3	1	4	BRASIL
2077604	F	20/12/198	39	3	3039	3	1	3	BRASIL
2338424	F	25/06/198	34	3	3034	3	4		BRASIL
2077396	F	08/09/199	23	3	3023	3	9	9	BRASIL
17884	F	28/05/199	24	3	3024	3	1	3	BRASIL
2288893	F	31/01/199	28	3	3028	1	2		BRASIL
9725	F	24/06/197	41	3	3041	4	4	3	BRASIL
2237253	F	16/10/199	29	3	3029	3	4		BRASIL
9725	F	12/01/199	22	3	3022	3	4	3	BRASIL
2688611	F	19/01/198	38	3	3038	3	1	4	BRASIL
2089602	F	14/04/200	20	3	3020	3	1		BRASIL
2298317	F	13/05/199	25	3	3025	3	1	9	BRASIL
2499363	F	12/04/199	24	3	3024	3			BRASIL
2080931	F	17/12/197	43	3	3043	3	9	9	BRASIL
2082187	F	13/11/198	39	3	3039	3	1		BRASIL
2019558	F	14/01/199	28	3	3028	2	4		BRASIL
2082594	F	08/10/198	39	3	3039	2	9	9	BRASIL
2082209	F	05/07/198	32	3	3032	3	2	2	BRASIL
3956369	F	02/06/199	27	3	3027	1	9	9	BRASIL

2688611	F	19/01/198	37	3	3037	1	9	4	BRASIL
2259907	F	02/03/200	19	3	3019	2	1		BRASIL
2027356	F	09/10/199	23	3	3023	3	1	3	BRASIL
2738368	F	05/05/199	21	3	3021	3	1		BRASIL
2338424	F	14/09/198	38	3	3038	3			BRASIL
5253381	F	02/04/198	37	3	3037	4	9	9	BRASIL
2337754	F	25/09/198	33	3	3033	2	4	3	BRASIL
2111624	F	04/04/199	25	3	3025	2	1		BRASIL
13846	F	12/10/199	29	3	3029	3	1	1	BRASIL
2078589	F	23/10/198	32	3	3032	2	1	3	BRASIL
2081628	F	02/01/199	27	3	3027	3	9	3	BRASIL
2362287	F	26/09/198	32	3	3032	2	4	9	BRASIL
10499	F	03/08/200	18	3	3018	3	4	9	BRASIL
2297795	F	25/04/198	32	3	3032	4	2	9	BRASIL
2078015	F	10/08/198	37	3	3037	3	2	9	BRASIL
15318	F	26/05/198	30	3	3030	3			BRASIL
6998704	F	19/09/200	19	3	3019	3	9	9	BRASIL
2269880	F	23/09/200	19	3	3019	2	9	9	BRASIL
434	F	15/08/198	30	3	3030	3	4	4	BRASIL
3956369	F	25/04/198	36	3	3036	3	4		BRASIL
2222043	F	03/03/197	42	3	3042	2	4	9	BRASIL
3956369	F	07/08/197	44	3	3044	2	4		BRASIL
3956369	F	27/09/198	39	3	3039	3	2		BRASIL
2339196	F	20/07/198	35	3	3035	3	1	3	BRASIL
2604388	F	19/12/199	23	3	3023	3	4	4	BRASIL
2792176	F	04/05/198	34	3	3034	3	4	3	BRASIL
2200473	F	21/09/199	28	3	3028	1	2	3	BRASIL
6995462	F	14/07/198	36	3	3036	3	4	9	BRASIL
12521	F	28/03/197	41	3	3041	2	1	9	BRASIL
2089602	F	06/11/198	31	3	3031	2	1	9	BRASIL
5073898	F	26/04/198	37	3	3037	3			BRASIL
2078252	F	17/07/198	40	3	3040	3	1	3	BRASIL
2006197	F	05/03/200	20	3	3020	2	4	2	BRASIL
2078139	F	04/12/198	37	3	3037	2	1	3	BRASIL
9923837	F	27/06/200	20	3	3020	2	2	9	BRASIL
7421710	F	22/02/199	29	3	3029	1	9	9	BRASIL
3956369	F	21/03/198	37	3	3037	3	9		BRASIL
2080044	F	28/08/198	33	3	3033	1	9	9	BRASIL
434	F	01/03/199	29	3	3029	2	4		BRASIL
2237571	F	10/10/199	25	3	3025	3	1	2	BRASIL
2078015	F	01/04/198	40	3	3040	1	1	9	BRASIL
3151794	F	21/08/199	29	3	3029	3	4		BRASIL
2309254	F	01/05/198	32	3	3032	3	4	9	BRASIL
27863	F	08/06/200	17	3	3017	3	4	1	BRASIL
2408570	F	26/08/198	31	3	3031	3	2	3	BRASIL
2077477	F	04/05/198	33	3	3033	2	1	9	BRASIL
3018520	F	22/12/199	29	3	3029	3	9	9	BRASIL

2499363	F	11/04/198[illegible]	35	3	3035	3			BRASIL
2078015	F	28/01/199[illegible]	25	3	3025	2	1	9	BRASIL
2338424	F	24/08/198[illegible]	33	3	3033	2	9		BRASIL
2222043	F	20/04/198[illegible]	37	3	3037	3	4	3	BRASIL
2295407	F	04/04/198[illegible]	32	3	3032	3	4	9	BRASIL
2384299	F	06/08/198[illegible]	38	3	3038	3	1	9	BRASIL
2604388	F	05/01/198[illegible]	36	3	3036	3	1	4	BRASIL
2089602	F	28/01/199[illegible]	23	3	3023	2	2	9	BRASIL
2680785	F	26/10/199[illegible]	22	3	3022	2	1	3	BRASIL
7781237	F	18/05/198[illegible]	31	3	3031	2	1		BRASIL
3956369	F	16/05/199[illegible]	29	3	3029	3	4		BRASIL
5332419	F	02/07/198[illegible]	35	3	3035	3	2	9	BRASIL
3956369	F	25/10/198[illegible]	32	3	3032	3	4	9	BRASIL
7257406	F	19/09/199[illegible]	25	3	3025	2	1	1	BRASIL
2298317	F	15/09/199[illegible]	24	3	3024	3	4	9	BRASIL
161438	F	15/07/197[illegible]	42	3	3042	2	1	3	BRASIL
2752700	F	02/12/199[illegible]	21	3	3021	3	3		BRASIL
2738368	F	13/02/198[illegible]	33	3	3033	1	1	3	BRASIL
3956369	F	25/06/199[illegible]	30	3	3030	3	9	9	BRASIL
2615746	F	18/06/200[illegible]	18	3	3018	2	2	3	BRASIL
7421710	F	17/03/199[illegible]	30	3	3030	2	1		BRASIL
3859	F	05/08/200[illegible]	15	3	3015	2	4		BRASIL
2561425	F	02/07/199[illegible]	21	3	3021	4	4		BRASIL
2815966	F	02/07/198[illegible]	38	3	3038	2	9	9	BRASIL
2580055	F	14/08/199[illegible]	26	3	3026	1	1	9	BRASIL
2396106	F	09/05/199[illegible]	26	3	3026	2	1	3	BRASIL
9146261	F	19/03/199[illegible]	26	3	3026	3	3	9	BRASIL
2091801	F	17/12/199[illegible]	27	3	3027	3	1	3	BRASIL
4251	F	20/07/198[illegible]	39	3	3039	2	4	4	BRASIL
2079828	F	03/01/199[illegible]	25	3	3025	3	9	9	BRASIL
2089599	F	24/03/199[illegible]	25	3	3025	1	9	9	BRASIL
3151794	F	22/10/198[illegible]	30	3	3030	3	4	3	BRASIL
2082187	F	05/04/199[illegible]	28	3	3028	3	1	1	BRASIL
2269988	F	03/08/198[illegible]	32	3	3032	3	4	9	BRASIL
6159397	F	03/11/199[illegible]	26	3	3026	2	1	9	BRASIL
7676484	F	22/05/198[illegible]	32	3	3032	3	4	3	BRASIL
2339196	F	08/06/197[illegible]	43	3	3043	2	2	1	BRASIL
2323397	F	07/02/199[illegible]	23	3	3023	3	4	1	BRASIL
2436450	F	31/10/199[illegible]	28	3	3028	3	1		BRASIL
5498465	F	21/09/199[illegible]	22	3	3022	2	4	3	BRASIL
2079038	F	28/02/198[illegible]	33	3	3033	3	1		BRASIL
9725	F	08/09/198[illegible]	34	3	3034	2	4	4	BRASIL
5108969	F	26/10/198[illegible]	39	3	3039	3	1		BRASIL
2078015	F	18/01/197[illegible]	43	3	3043	3	1	9	BRASIL
4069099	F	03/06/199[illegible]	21	3	3021	3	1	3	BRASIL
2384299	F	30/11/199[illegible]	29	3	3029	3	1	9	BRASIL
2309254	F	08/03/199[illegible]	28	3	3028	3	3	4	BRASIL

2604388	F	27/10/198	31	3	3031	3	9	9	BRASIL
2237571	F	29/08/199	28	3	3028	4	1	3	BRASIL
2487438	F	29/07/197	46	3	3046	1	1	3	BRASIL
3956369	F	06/12/199	28	3	3028	2	4	9	BRASIL
3151794	F	01/12/198	33	3	3033	3	4	2	BRASIL
434	F	22/08/199	24	3	3024	4	4	9	BRASIL
2025752	F	21/07/198	38	3	3038	2	1	3	BRASIL
2270714	F	19/07/198	38	3	3038	2	4	1	BRASIL
2077396	F	04/04/200	16	3	3016	3	1	2	BRASIL
2688611	F	03/06/199	27	3	3027	1	9	4	BRASIL
2078082	F	08/06/198	32	3	3032	3	1	9	BRASIL
3212130	F	14/12/198	33	3	3033	3	4	9	BRASIL
2016354	F	27/10/198	34	3	3034	2	4	4	BRASIL
5073898	F	06/05/198	31	3	3031	2	4	3	BRASIL
2726653	F	07/07/198	38	3	3038	2	4	3	BRASIL
7659415	F	10/01/199	28	3	3028	3	4	9	BRASIL
2436450	F	25/07/199	30	3	3030	1	1		BRASIL
2338424	F	07/04/199	22	3	3022	3	4		BRASIL
2261057	F	17/08/199	26	3	3026	3	1	2	BRASIL
6038883	F	19/04/198	36	3	3036	4	9		BRASIL
2323397	F	26/12/199	20	3	3020	1	4		BRASIL
2323397	F	22/04/199	21	3	3021	3	9		BRASIL
2084163	F	26/09/198	32	3	3032	2	1	2	BRASIL
27022	F	15/11/199	25	3	3025	2	1		BRASIL
2323397	F	09/09/200	19	3	3019	3	4	9	BRASIL
3956369	F	06/01/199	21	3	3021	2	4	9	BRASIL
2078015	F	13/11/197	45	3	3045	3	1	9	BRASIL
2738368	F	15/06/199	29	3	3029	3	1		BRASIL
2018055	F	17/09/199	28	3	3028	3	4		BRASIL
2082179	F	02/08/198	31	3	3031	2	1	4	BRASIL
6602533	F	16/10/199	26	3	3026	1	4		BRASIL
2270714	F	20/02/200	19	3	3019	3	4	3	BRASIL
2017199	F	07/08/199	28	3	3028	3	9		BRASIL
3956369	F	03/05/199	26	3	3026	2	2	3	BRASIL
5466032	F	27/07/199	27	3	3027	3	4	3	BRASIL
3956369	F	22/11/199	26	3	3026	3	2	9	BRASIL
2078015	F	11/10/199	24	3	3024	2	4	9	BRASIL
2080338	F	02/10/198	32	3	3032	2	1	9	BRASIL
5407591	F	15/05/198	37	3	3037	3	4	3	BRASIL
2269988	F	03/11/198	34	3	3034	2	1	9	BRASIL
7659415	F	02/06/198	38	3	3038	2	9	9	BRASIL
5073898	F	24/09/198	38	3	3038	2	1	3	BRASIL
2781719	F	10/01/198	31	3	3031	3	1	4	BRASIL
2078015	F	23/06/198	32	3	3032	3	1	9	BRASIL
2497654	F	06/03/199	22	3	3022	1	4	3	BRASIL
2222043	F	13/02/199	27	3	3027	2	9	9	BRASIL
3708926	F	13/08/197	42	3	3042	2	3	3	BRASIL

6901743	F	30/06/197	41	3	3041	3	1	9	BRASIL
2688611	F	09/10/198	31	3	3031	3	1		BRASIL
2077604	F	17/01/199	28	3	3028	3	4	9	BRASIL
3065634	F	17/01/198	37	3	3037	3	9	9	BRASIL
2270609	F	11/10/198	33	3	3033	3	1	9	BRASIL
2028840	F	22/07/197	45	3	3045	3	1	9	BRASIL
2338424	F	13/07/199	26	3	3026	3	4		BRASIL
2019574	F	04/08/199	22	3	3022	2	1	2	BRASIL
2499363	F	24/01/198	33	3	3033	3	4		BRASIL
2269880	F	22/10/198	34	3	3034	3	2	2	BRASIL
8923	F	11/08/197	43	3	3043	2	2	1	BRASIL
2499363	F	21/02/198	37	3	3037	2	4		BRASIL
4052994	F	10/12/199	22	3	3022	2	4	2	BRASIL
2269880	F	28/04/198	34	3	3034	3	9	9	BRASIL
3956369	F	14/08/199	29	3	3029	3	4	9	BRASIL
7711980	F	05/10/198	36	3	3036	3	4	9	BRASIL
2726653	F	08/05/199	28	3	3028	2	2	3	BRASIL
24074	F	17/05/200	17	3	3017	2	4	2	BRASIL
2298120	F	11/01/198	36	3	3036	3	4	4	BRASIL
2017245	F	19/10/199	28	3	3028	3	4	3	BRASIL
2252694	F	27/09/198	36	3	3036	2	1	3	BRASIL
2070413	F	06/12/199	20	3	3020	2	1	3	BRASIL
7786913	F	14/01/198	40	3	3040	2	1	3	BRASIL
2208857	F	23/11/198	33	3	3033	2	1	4	BRASIL
5101654	F	16/04/199	28	3	3028	2	1	3	BRASIL
6988938	F	13/05/199	28	3	3028	2	1	3	BRASIL
2082888	F	10/03/199	23	3	3023	2	1	3	BRASIL
5515483	F	24/01/199	30	3	3030	3	1	4	BRASIL
7421710	F	16/01/198	40	3	3040	3	1	4	BRASIL
10472	F	07/05/199	27	3	3027	3	4	9	BRASIL
3006522	F	31/01/198	32	3	3032	3	1	9	BRASIL
2089599	F	13/10/199	28	3	3028	1	9	9	BRASIL
434	F	30/03/198	32	3	3032	2	2	2	BRASIL
2013320	F	30/04/199	24	3	3024	4	4	9	BRASIL
2077477	F	21/12/199	26	3	3026	2	1	3	BRASIL
2481286	F	21/09/199	20	3	3020	3	4	9	BRASIL
3356868	F	07/11/198	30	3	3030	3	9	9	BRASIL
2754843	F	18/03/199	26	3	3026	3	4	9	BRASIL
2079542	F	04/03/199	29	3	3029	2	1	4	BRASIL
7257406	F	18/09/200	17	3	3017	2	4		BRASIL
7354746	F	30/04/200	20	3	3020	2	4		BRASIL
2604388	F	04/10/198	34	3	3034	3	1	9	BRASIL
2097613	F	17/02/198	38	3	3038	3	1	3	BRASIL
3151794	F	02/05/199	28	3	3028	3	4		BRASIL
434	F	18/08/199	29	3	3029	2	4		BRASIL
5407591	F	23/10/199	29	3	3029	3	9	9	BRASIL
2707527	F	01/04/198	36	3	3036	3	4	0	BRASIL

2077485	F	26/12/198	35	3	3035	2	4	9	BRASIL
2323397	F	13/04/199	28	3	3028	2	9		BRASIL
176664	F	17/10/199	26	3	3026	3	2		BRASIL
2078848	F	13/01/199	21	3	3021	3	4	3	BRASIL
6984649	F	20/11/199	21	3	3021	3	2	9	BRASIL
2311682	F	11/11/199	23	3	3023	2	4	9	BRASIL
2089602	F	09/02/199	30	3	3030	3	1	9	BRASIL
2752700	F	09/11/198	35	3	3035	3	4	2	BRASIL
2299216	F	05/03/199	28	3	3028	1	2		BRASIL
2151871	F	09/06/198	37	3	3037	3	4	3	BRASIL
2079186	F	25/12/199	27	3	3027	2	4	2	BRASIL
2081148	F	16/10/198	32	3	3032	2	1	4	BRASIL
3056724	F	12/07/199	25	3	3025	3	9	9	BRASIL
3065634	F	19/12/198	36	3	3036	3	1	9	BRASIL
2081938	F	10/01/199	30	3	3030	3	4		BRASIL
3006522	F	30/05/198	35	3	3035	2	1	9	BRASIL
2688611	F	31/03/198	36	3	3036	3	1	4	BRASIL
2269988	F	23/05/198	38	3	3038	3	1	9	BRASIL
2058391	F	24/02/198	34	3	3034	4	9		BRASIL
2410281	F	20/01/199	24	3	3024	2	4	3	BRASIL
2407205	F	03/10/198	32	3	3032	3	4	4	BRASIL
3272591	F	21/05/197	42	3	3042	3	1	9	BRASIL
2361167	F	02/07/198	40	3	3040	2	1	3	BRASIL
2781859	F	16/08/198	31	3	3031	3	5		BRASIL
434	F	31/07/198	35	3	3035	2	4	9	BRASIL
3046281	F	18/10/200	19	3	3019	2	4	9	BRASIL
2415488	F	21/12/199	26	3	3026	2	4	3	BRASIL
5585422	F	16/08/198	30	3	3030	2	4	9	BRASIL
13633	F	27/05/198	37	3	3037	2	1	3	BRASIL
2159252	F	30/03/199	26	3	3026	2	1		BRASIL
2023148	F	10/08/199	26	3	3026	3	2	9	BRASIL
2338424	F	23/03/199	24	3	3024	3	9		BRASIL
2688611	F	11/11/198	37	3	3037	2		4	BRASIL
2077396	F	12/11/199	26	3	3026	3	1	2	BRASIL
5158044	F	25/06/198	35	3	3035	3	9	3	BRASIL
2082330	F	30/11/198	31	3	3031	2	1	9	BRASIL
5510341	F	29/03/198	37	3	3037	3	4	9	BRASIL
9923837	F	28/09/198	35	3	3035	3	4	2	BRASIL
2707527	F	20/05/199	20	3	3020	3	4	9	BRASIL
2078015	F	25/09/198	36	3	3036	3	4	9	BRASIL
2707527	F	15/05/200	18	3	3018	3	1	2	BRASIL
3157245	F	25/01/199	28	3	3028	3	1	9	BRASIL
2248328	F	03/10/198	34	3	3034	3	1	2	BRASIL
2208857	F	26/11/198	35	3	3035	2	1	3	BRASIL
9194258	F	16/02/198	38	3	3038	3	4		BRASIL
2594714	F	15/06/198	33	3	3033	3	4		BRASIL
2295415	F	04/01/198	34	3	3034	3	9	9	BRASIL

9554157	F	20/04/198	36	3	3036	3	9	9	BRASIL
2208857	F	21/04/199	23	3	3023	2	1	4	BRASIL
2415631	F	14/12/198	31	3	3031	4	4	9	BRASIL
2389746	F	07/12/198	37	3	3037	2	4	4	BRASIL
2025760	F	27/04/199	27	3	3027	2	9	9	BRASIL
2269783	F	22/03/200	17	3	3017	2	4	2	BRASIL
15369	F	22/06/198	31	3	3031	1	9		BRASIL
1120	F	25/10/198	37	3	3037	2	9	9	BRASIL
2361949	F	09/08/199	26	3	3026	3	4	3	BRASIL
7041624	F	09/02/198	31	3	3031	3	4		BRASIL
2287250	F	01/06/198	31	3	3031	4	4	9	BRASIL
2078015	F	10/03/199	21	3	3021	1	1	9	BRASIL
2077396	F	03/03/198	32	3	3032	3	4	4	BRASIL
3030121	F	07/06/199	23	3	3023	3	9	9	BRASIL
2078015	F	04/08/199	23	3	3023	2	1	9	BRASIL
2308665	F	10/02/199	25	3	3025	2	4	3	BRASIL
434	F	08/12/199	24	3	3024	3	4		BRASIL
2604388	F	11/08/199	22	3	3022	3	1	4	BRASIL
2078015	F	13/07/199	27	3	3027	3	4	9	BRASIL
3037274	F	13/01/198	31	3	3031	3	9	4	BRASIL
2058308	F	15/10/197	40	3	3040	2	1		BRASIL
2752700	F	30/03/200	16	3	3016	3	4	3	BRASIL
2688611	F	02/12/198	37	3	3037	3	9	3	BRASIL
2078015	F	12/06/198	30	3	3030	2	2	9	BRASIL
2615746	F	05/09/198	30	3	3030	2	4	9	BRASIL
2092395	F	29/07/198	35	3	3035	3	1	3	BRASIL
2399644	F	22/05/199	20	3	3020	2	4	3	BRASIL
2151871	F	23/09/198	31	3	3031	3	4	3	BRASIL
2339218	F	16/02/199	22	3	3022	1	1	9	BRASIL
2078082	F	29/12/199	27	3	3027	1	9	9	BRASIL
2082322	F	20/09/200	19	3	3019	3	4	2	BRASIL
2308665	F	28/07/197	47	3	3047	4	4	4	BRASIL
2615770	F	01/10/199	29	3	3029	3	5	4	BRASIL
9119701	F	28/10/199	28	3	3028	2	4	2	BRASIL
15369	F	13/05/198	32	3	3032	3	1		BRASIL
3242587	F	20/11/198	36	3	3036	2	9		BRASIL
434	F	09/04/199	28	3	3028	3	4	9	BRASIL
27022	F	10/10/199	25	3	3025	3	4	9	BRASIL
2499363	F	01/03/200	18	3	3018	3	4		BRASIL
2006197	F	01/06/198	38	3	3038	2	4	3	BRASIL
2078082	F	07/10/199	24	3	3024	3			BRASIL
2078015	F	15/06/198	31	3	3031	3	2	3	BRASIL
13846	F	21/08/198	34	3	3034	2	1		BRASIL
2077396	F	03/10/197	41	3	3041	3	1	9	BRASIL
3058336	F	20/03/198	33	3	3033	3	1	3	BRASIL
2206382	F	25/10/199	22	3	3022	3	4	3	BRASIL
13633	F	08/02/199	27	3	3027	3	1	2	BRASIL

2362287	F	21/06/198	32	3	3032	3	1	2	BRASIL
2070413	F	11/11/198	32	3	3032	2	1	3	BRASIL
6984649	F	29/12/198	37	3	3037	3	4		BRASIL
6817203	F	23/08/199	26	3	3026	3	4	3	BRASIL
434	F	20/04/199	21	3	3021	3	4	9	BRASIL
6995462	F	11/03/197	45	3	3045	2	9	9	BRASIL
2752700	F	27/02/198	34	3	3034	3	4	9	BRASIL
6159397	F	19/08/198	40	3	3040	3	9		BRASIL
2078767	F	05/06/198	39	3	3039	1	1		BRASIL
2079720	F	02/06/198	38	3	3038	1	4	2	BRASIL
2688689	F	25/11/198	35	3	3035	2	4	3	BRASIL
2079542	F	01/06/197	44	3	3044	3	9	9	BRASIL
2499363	F	09/03/198	35	3	3035	3	4		BRASIL
2705222	F	07/10/198	33	3	3033	2	1	2	BRASIL
2319659	F	07/05/199	28	3	3028	4	4		BRASIL
2078082	F	18/11/200	18	3	3018	3	1	3	BRASIL
2078015	F	13/03/200	20	3	3020	2	9	9	BRASIL
2688611	F	08/12/198	32	3	3032	1	9	9	BRASIL
2146355	F	12/06/199	28	3	3028	1	4	9	BRASIL
2078015	F	14/04/198	37	3	3037	3	1	9	BRASIL
2415631	F	08/03/198	33	3	3033	2	4	9	BRASIL
3151794	F	24/07/200	19	3	3019	3	4	1	BRASIL
5824990	F	03/05/199	30	3	3030	2	1	3	BRASIL
2604388	F	12/02/199	30	3	3030	3	4		BRASIL
6995462	F	24/11/200	16	3	3016	3	4	9	BRASIL
7257406	F	12/12/199	25	3	3025	3	9		BRASIL
2298317	F	26/05/200	18	3	3018	2	1	9	BRASIL
2078015	F	01/10/198	33	3	3033	2	1	9	BRASIL
3012212	F	27/10/199	25	3	3025	3	4		BRASIL
9923837	F	04/06/199	30	3	3030	2	9	9	BRASIL
2338424	F	05/12/199	25	3	3025	2	4		BRASIL
6995462	F	24/10/198	35	3	3035	3	2	4	BRASIL
5073898	F	27/08/199	21	3	3021	3	4	3	BRASIL
2222043	F	31/08/199	29	3	3029	3	4	4	BRASIL
2499363	F	09/05/200	17	3	3017	3	4	9	BRASIL
2078015	F	07/02/198	38	3	3038	2	3	9	BRASIL
2298317	F	16/09/198	37	3	3037	3	4	9	BRASIL
2018055	F	03/06/198	39	3	3039	2	4	3	BRASIL
3151794	F	08/12/199	22	3	3022	3	4		BRASIL
27863	F	04/03/197	43	3	3043	2	2	1	BRASIL
434	F	26/02/199	29	3	3029	3	4	9	BRASIL
2078015	F	30/10/199	27	3	3027	3	1	9	BRASIL
13633	F	21/12/198	36	3	3036	3	1	3	BRASIL
2534967	F	23/02/200	17	3	3017	1	4		BRASIL
2707527	F	17/06/198	37	3	3037	2	1	9	BRASIL
2323397	F	14/06/199	30	3	3030	1	4		BRASIL
7659415	F	13/10/198	38	3	3038	2	1		BRASIL

2710935	F	02/02/198	40	3	3040	3	4		BRASIL
2298120	F	22/09/199	29	3	3029	1	1	2	BRASIL
2323397	F	30/05/199	28	3	3028	1	4	9	BRASIL
2129469	F	13/02/199	27	3	3027	1	1	3	BRASIL
6159397	F	05/01/198	39	3	3039	3	4		BRASIL
7257406	F	07/05/198	32	3	3032	3	4	1	BRASIL
5498465	F	10/09/198	30	3	3030	3	4	3	BRASIL
2237253	F	08/09/198	39	3	3039	2	1	4	BRASIL
2089602	F	22/01/198	32	3	3032	3	1	4	BRASIL
7257406	F	19/11/199	26	3	3026	3	4		BRASIL
4001303	F	06/03/198	37	3	3037	2	4	3	BRASIL
3508528	F	24/12/199	28	3	3028	1	1	9	BRASIL
2309254	F	10/07/198	32	3	3032	2	4		BRASIL
9624325	F	07/07/198	36	3	3036	2	9	9	BRASIL
2278855	F	26/07/198	36	3	3036	4	4	9	BRASIL
2611686	F	19/07/198	31	3	3031	3	4		BRASIL
2006197	F	13/09/199	21	3	3021	3	4	3	BRASIL
6817203	F	04/05/198	37	3	3037	3	4	3	BRASIL
2688611	F	02/06/198	31	3	3031	3	9	4	BRASIL
6878318	F	04/04/198	36	3	3036	3	1		BRASIL
5073898	F	04/12/198	37	3	3037	2	1	4	BRASIL
2237571	F	23/10/199	29	3	3029	3	2	2	BRASIL
2298104	F	03/02/198	36	3	3036	2	1	9	BRASIL
3702693	F	28/10/198	36	3	3036	3	4	4	BRASIL
2089602	F	06/11/199	25	3	3025	2	1		BRASIL
2126494	F	01/08/198	34	3	3034	1	9	9	BRASIL
2200473	F	12/05/200	17	3	3017	3	1	9	BRASIL
6602533	F	06/02/198	33	3	3033	3	4		BRASIL
2338424	F	27/01/199	25	3	3025	2			BRASIL
2040069	F	25/09/199	20	3	3020	2	4	3	BRASIL
2798298	F	31/05/199	25	3	3025	3	2	9	BRASIL
2410281	F	10/08/198	39	3	3039	2	1	3	BRASIL
9539	F	13/04/198	34	3	3034	1	1	3	BRASIL
6559379	F	19/09/198	33	3	3033	2	9		BRASIL
3048551	F	03/11/197	41	3	3041	3	9	4	BRASIL
5860490	F	05/10/199	29	3	3029	3	1	3	BRASIL
2611686	F	27/12/198	34	3	3034	2	4	9	BRASIL
2384299	F	20/01/199	25	3	3025	3	4	9	BRASIL
2078015	F	12/11/198	35	3	3035	3	1	9	BRASIL
2261987	F	05/06/198	34	3	3034	2	1	3	BRASIL
9037780	F	21/08/198	32	3	3032	2	4		BRASIL
2275562	F	21/12/198	32	3	3032	2	1		BRASIL
3956369	F	04/09/199	21	3	3021	3	4	9	BRASIL
2303892	F	05/07/198	40	3	3040	1	1	2	BRASIL
6159397	F	09/08/198	34	3	3034	3	9		BRASIL
5498465	F	16/01/198	36	3	3036	3	1	2	BRASIL
3956369	F	04/07/199	27	3	3027	3	4	9	BRASIL

2077485	F	12/06/199	29	3	3029	3	1	3	BRASIL
2384299	F	23/07/199	26	3	3026	3	4	9	BRASIL
2499363	F	27/12/198	37	3	3037	3	4	9	BRASIL
9151583	F	02/09/198	34	3	3034	1			BRASIL
2752700	F	21/10/198	31	3	3031	2	4	9	BRASIL
2339196	F	10/01/199	28	3	3028	3	4	4	BRASIL
3956369	F	27/05/199	29	3	3029	2	4		BRASIL
3151794	F	10/01/197	43	3	3043	2	4	1	BRASIL
2594714	F	28/09/199	27	3	3027	3	1	3	BRASIL
2752700	F	07/07/199	22	3	3022	3	4	3	BRASIL
2079542	F	20/11/198	39	3	3039	2	9	4	BRASIL
434	F	15/12/200	15	3	3015	3	4	9	BRASIL
2269783	F	10/07/198	37	3	3037	2	1	1	BRASIL
2707527	F	20/01/198	35	3	3035	3	4	4	BRASIL
7038895	F	13/09/198	31	3	3031	2	1	3	BRASIL
15369	F	03/11/200	19	3	3019	2	1		BRASIL
2079798	F	06/03/198	34	3	3034	3	1	3	BRASIL
6665322	F	22/02/199	25	3	3025	3	4		BRASIL
2755165	F	15/12/199	27	3	3027	3	1	4	BRASIL
2077477	F	22/10/200	16	3	3016	1	1	2	BRASIL
2017199	F	07/11/198	33	3	3033	3	2		BRASIL
6518893	F	01/05/198	37	3	3037	2	4		BRASIL
6984649	F	21/01/199	30	3	3030	2	2	9	BRASIL
6007317	F	28/07/198	37	3	3037	2	9	9	BRASIL
3367371	F	25/07/198	30	3	3030	2	4		BRASIL
2237601	F	18/04/199	24	3	3024	3	2		BRASIL
2655411	F	25/10/199	26	3	3026	1	4		BRASIL
2078473	F	24/05/199	23	3	3023	4	1	3	BRASIL
27847	F	19/02/199	29	3	3029	3	4	9	BRASIL
2208857	F	08/03/199	25	3	3025	2	1	2	BRASIL
3000850	F	16/09/198	33	3	3033	3	9	9	BRASIL
7676484	F	03/11/198	37	3	3037	3	1	3	BRASIL
6878318	F	10/01/198	40	3	3040	3	1		BRASIL
2079542	F	16/05/199	28	3	3028	2	9	4	BRASIL
2269783	F		36	3	3036	3	2	2	BRASIL
2097117	F	30/10/198	34	3	3034	3	1	3	BRASIL
3030121	F	20/09/199	27	3	3027	3	4	9	BRASIL
2298120	F	14/08/198	39	3	3039	3	2	9	BRASIL
2078015	F	10/08/198	35	3	3035	3	9	9	BRASIL
434	F	25/05/199	25	3	3025	3	4		BRASIL
6848710	F	30/09/197	43	3	3043	3	9		BRASIL
176664	F	25/05/199	27	3	3027	1	4	2	BRASIL
6779522	F	28/10/198	38	3	3038	4			BRASIL
7244339	F	12/10/199	25	3	3025	4	9	9	BRASIL
2726653	F	28/04/198	37	3	3037	2	4	3	BRASIL
2376334	F	17/05/198	37	3	3037	2	4	4	BRASIL
24074	F	13/09/199	28	3	3028	2	4	3	BRASIL

2707527	F	10/02/199	28	3	3028	3	4	3	BRASIL
2323397	F	06/10/199	28	3	3028	3	9		BRASIL
2089777	F	08/08/198	33	3	3033	2	9		BRASIL
2080516	F	19/07/198	31	3	3031	3	4	4	BRASIL
9841903	F	18/12/198	30	3	3030	3	4	4	BRASIL
2077477	F	29/06/198	38	3	3038	3	4	9	BRASIL
2208172	F	25/11/197	41	3	3041	3	4	9	BRASIL
434	F	01/02/199	29	3	3029	2	4	3	BRASIL
2430711	F	28/09/199	26	3	3026	2	4		BRASIL
2688611	F	07/02/199	29	3	3029	3	1		BRASIL
2696940	F	29/09/198	40	3	3040	1	4	1	BRASIL
2362287	F	27/11/198	30	3	3030	2	4		BRASIL
2752077	F	08/02/199	26	3	3026	3	2	9	BRASIL
2078589	F	01/11/197	40	3	3040	4	9	9	BRASIL
2006197	F	10/06/200	17	3	3017	3	4	9	BRASIL
2237601	F	17/05/198	35	3	3035	2	1		BRASIL
2298317	F	01/11/198	36	3	3036	4	4	9	BRASIL
3956369	F	18/03/198	33	3	3033	3	4	9	BRASIL
2082187	F	07/08/199	24	3	3024	3	4		BRASIL
2082187	F	03/08/197	43	3	3043	2	4		BRASIL
3037274	F	29/10/199	30	3	3030	2	9	9	BRASIL
2077701	F	02/09/199	29	3	3029	3	4	9	BRASIL
7436734	F	14/07/199	26	3	3026	2	1	4	BRASIL
2362287	F	05/02/199	27	3	3027	2	1	2	BRASIL
2079798	F	08/12/199	28	3	3028	3	4	3	BRASIL
5717515	F	30/09/199	24	3	3024	3	4	9	BRASIL
3008398	F	14/06/198	31	3	3031	3	1	3	BRASIL
7711980	F	18/02/199	25	3	3025	2	4	9	BRASIL
2688700	F	19/02/198	31	3	3031	4	1	3	BRASIL
2295407	F	06/02/199	30	3	3030	1	9	9	BRASIL
2080583	F	05/02/198	36	3	3036	3	4	3	BRASIL
2078015	F	10/08/198	38	3	3038	3	4	9	BRASIL
2089602	F	20/05/198	33	3	3033	3	1	9	BRASIL
3012212	F	08/12/198	36	3	3036	3	2		BRASIL
2688611	F	23/02/198	33	3	3033	2	9	4	BRASIL
2786117	F	02/06/200	18	3	3018	2	4	3	BRASIL
3956369	F	26/11/199	25	3	3025	2	9	9	BRASIL
2078503	F	12/08/200	19	3	3019	3	1	3	BRASIL
434	F	12/06/200	17	3	3017	2	4	9	BRASIL
2195437	F	03/04/198	34	3	3034	2	1		BRASIL
3546551	F	10/08/198	32	3	3032	3	9		BRASIL
7421710	F	03/12/198	34	3	3034	3	9	9	BRASIL
2707527	F	08/11/198	36	3	3036	3	4	3	BRASIL
2802112	F	30/09/200	19	3	3019	1	4	3	BRASIL
3956369	F	31/07/199	29	3	3029	3	4	9	BRASIL
2078015	F	29/05/198	40	3	3040	3	9	9	BRASIL
3037274	F	21/05/198	34	3	3034	2	9	9	BRASIL

2499363	F	08/07/198	34	3	3034	3	4	9	BRASIL
2726726	F	06/12/197	40	3	3040	1	4		BRASIL
3394514	F	24/07/198	37	3	3037	2			BRASIL
2078015	F	21/01/199	24	3	3024	3	4	9	BRASIL
5833418	F	21/03/198	35	3	3035	3	1	3	BRASIL
2338424	F	13/02/198	32	3	3032	3			BRASIL
2339196	F	04/01/199	28	3	3028	3	4	9	BRASIL
4001303	F	20/12/198	32	3	3032	2	4	3	BRASIL
2080699	F	22/12/198	34	3	3034	2	2	2	BRASIL
2798662	F	11/10/199	21	3	3021	2	9	9	BRASIL
2145316	F	16/03/199	29	3	3029	2	2	9	BRASIL
2077477	F	16/10/200	20	3	3020	3	4	3	BRASIL
2269988	F	15/07/198	33	3	3033	2	4	9	BRASIL
2082225	F	26/09/198	35	3	3035	3	1	9	BRASIL
2338424	F	22/09/198	36	3	3036	1	9		BRASIL
3037274	F	30/09/198	34	3	3034	2	9	9	BRASIL
2241048	F	28/12/199	27	3	3027	2	1		BRASIL
2319659	F	22/07/198	33	3	3033	2	4	3	BRASIL
2672197	F	15/05/198	33	3	3033	3	4		BRASIL
6602533	F	09/03/199	28	3	3028	3	4		BRASIL
2040069	F	30/07/199	29	3	3029	1	4	3	BRASIL
2654024	F	05/01/199	30	3	3030	3	4	3	BRASIL
2752700	F	26/10/198	34	3	3034	3	3	3	BRASIL
2191164	F	06/03/198	31	3	3031	2	9	9	BRASIL
2022621	F	18/08/199	25	3	3025	3	4	3	BRASIL
2191164	F	23/10/199	26	3	3026	3	1	3	BRASIL
3367371	F	21/10/199	26	3	3026	2	1		BRASIL
3028399	F	15/08/198	35	3	3035	3	1		BRASIL
7257406	F	06/06/199	21	3	3021	3	1	9	BRASIL
3517918	F	26/06/198	32	3	3032	2	4	4	BRASIL
7659415	F	01/04/199	29	3	3029	3	2	3	BRASIL
2269988	F	10/09/198	39	3	3039	2	4	9	BRASIL
10464	F	07/01/198	37	3	3037	3	4		BRASIL
2654024	F	08/10/198	40	3	3040	2	9	9	BRASIL
24074	F	24/10/199	28	3	3028	2	4	3	BRASIL
6734014	F	06/08/199	25	3	3025	2	1	9	BRASIL
2338424	F	14/02/198	32	3	3032	3	1		BRASIL
7659415	F	15/08/198	34	3	3034	3	1		BRASIL
3046281	F	08/02/198	37	3	3037	3	4		BRASIL
3056724	F	27/02/198	33	3	3033	3	1		BRASIL
2499363	F	08/03/199	28	3	3028	3	4	9	BRASIL
2339234	F	25/03/197	44	3	3044	4	4	9	BRASIL
2499363	F	13/04/198	33	3	3033	3	4	9	BRASIL
2237601	F	08/03/198	37	3	3037	2	1		BRASIL
2674971	F	01/04/198	32	3	3032	2	9		BRASIL
7257406	F	20/08/198	31	3	3031	2	4	3	BRASIL
2126494	F	20/09/197	43	3	3043	3	9		BRASIL

2076896	F	11/01/199	30	3	3030	4	9	9	BRASIL
2168707	F	04/10/197	44	3	3044	2	4	1	BRASIL
4013484	F	21/11/198	31	3	3031	2	4	9	BRASIL
7621442	F	04/03/198	39	3	3039	3	4		BRASIL
2082187	F	05/09/198	40	3	3040	3	1		BRASIL
7257406	F	13/08/199	22	3	3022	2	4	3	BRASIL
2077396	F	07/10/198	32	3	3032	2	1	9	BRASIL
2499363	F	22/02/200	19	3	3019	3	4	9	BRASIL
2825589	F	04/08/199	25	3	3025	2	4	4	BRASIL
2237601	F	21/07/198	33	3	3033	3	1	3	BRASIL
9709	F	18/07/197	42	3	3042	2	1	9	BRASIL
2439263	F	15/11/198	38	3	3038	2	4	9	BRASIL
2655411	F	09/09/199	26	3	3026	3	4		BRASIL
2521385	F	17/04/199	31	3	3031	3	1	4	BRASIL
24074	F	30/05/200	20	3	3020	2	4	2	BRASIL
2246988	F	22/11/199	26	3	3026	3	1	3	BRASIL
2091585	F	24/08/199	30	3	3030	2	4		BRASIL
3625087	F	19/07/198	38	3	3038	3	1	4	BRASIL
3046281	F	18/06/199	23	3	3023	3	9		BRASIL
2309254	F	19/01/198	34	3	3034	3	4		BRASIL
5515483	F	06/07/199	23	3	3023	3	1	3	BRASIL
5101654	F	05/01/198	36	3	3036	2	1	9	BRASIL
2362287	F	02/03/200	18	3	3018	1	4		BRASIL
6681573	F	16/05/197	47	3	3047	4	4	9	BRASIL
9554157	F	12/07/198	36	3	3036	2	9		BRASIL
2499363	F	27/05/199	21	3	3021	3	4	9	BRASIL
2499363	F	20/09/199	24	3	3024	3	4	9	BRASIL
5597501	F	25/09/199	28	3	3028	3	4		BRASIL
2079542	F	19/07/198	35	3	3035	3	1	4	BRASIL
2655411	F	29/03/199	24	3	3024	3	1		BRASIL
6003575	F	10/05/197	49	3	3049	2	9		BRASIL
2589737	F	08/09/199	21	3	3021	2	2		BRASIL
2269988	F	14/01/199	27	3	3027	3	4	9	BRASIL
5510341	F	24/05/198	33	3	3033	3	9	9	BRASIL
2269988	F	01/03/199	25	3	3025	2	1	9	BRASIL
2269988	F	20/08/198	35	3	3035	1	9	9	BRASIL
2442612	F	17/05/199	25	3	3025	3	2	2	BRASIL
3151794	F	27/03/198	36	3	3036	1	4	4	BRASIL
2652706	F	20/12/198	39	3	3039	2	4	3	BRASIL
24074	F	08/10/199	29	3	3029	2	4	3	BRASIL
3037274	F	11/12/198	35	3	3035	3	9	9	BRASIL
2078562	F	12/12/198	31	3	3031	2	4		BRASIL
2793008	F	24/09/198	34	3	3034	3	9	9	BRASIL
2306344	F	08/02/198	33	3	3033	3	1	3	BRASIL
2499363	F	15/08/198	35	3	3035	3	4	9	BRASIL
6848710	F	24/02/198	40	3	3040	1	4		BRASIL
27022	F	05/08/199	26	3	3026	2	4	9	BRASIL

2262274	F	18/12/199	29	3	3029	2	1		BRASIL
3013162	F	17/09/198	36	3	3036	3	4	9	BRASIL
2022621	F	29/06/198	33	3	3033	2	4	2	BRASIL
2237571	F	21/01/198	35	3	3035	2	1	3	BRASIL
2078376	F	05/02/197	42	3	3042	3	1	9	BRASIL
2802082	F	27/06/198	40	3	3040	2	9	9	BRASIL
2022621	F	03/02/199	29	3	3029	1	1		BRASIL
2499363	F	22/01/198	34	3	3034	3	4	9	BRASIL
3139050	F	13/09/199	24	3	3024	3	1		BRASIL
3012212	F	06/07/199	25	3	3025	1	4	9	BRASIL
2309254	F	23/11/200	18	3	3018	2	4	0	BRASIL
2078015	F	07/03/199	30	3	3030	3	4	9	BRASIL
7244339	F	22/05/198	40	3	3040	2	9	9	BRASIL
2079798	F	22/11/199	30	3	3030	2	1	9	BRASIL
2089602	F	05/02/198	35	3	3035	4	1		BRASIL
2738368	F	05/08/199	27	3	3027	3	1	1	BRASIL
2232057	F	01/11/198	38	3	3038	3	9		BRASIL
7061838	F	02/07/198	38	3	3038	2	4	1	BRASIL
7711980	F	18/03/198	35	3	3035	3	4	9	BRASIL
2078015	F	29/12/199	27	3	3027	3	1	9	BRASIL
14109	F	24/04/199	30	3	3030	3	1	3	BRASIL
2783789	F	15/08/198	35	3	3035	3	1		BRASIL
2078015	F	13/09/199	24	3	3024	3	4	9	BRASIL
2098369	F	17/01/199	28	3	3028	2	1	3	BRASIL
2309254	F	20/06/198	33	3	3033	2	4		BRASIL
2752700	F	23/08/200	14	3	3014	2	4		BRASIL
2079542	F	21/11/197	43	3	3043	2	2	3	BRASIL
9101993	F	28/06/197	46	3	3046	2	4		BRASIL
2223538	F	01/12/198	36	3	3036	1	1		BRASIL
2079542	F	21/06/198	38	3	3038	3	4	2	BRASIL
2499363	F	29/09/200	16	3	3016	3	4		BRASIL
5073898	F	18/10/198	40	3	3040	3	4	3	BRASIL
2481286	F	22/11/198	36	3	3036	3	4	2	BRASIL
2078015	F	13/07/199	26	3	3026	3	1	9	BRASIL
2655411	F	01/01/198	33	3	3033	3	4		BRASIL
24074	F	25/11/199	29	3	3029	2	4	1	BRASIL
5621674	F	26/09/198	34	3	3034	2	1		BRASIL
2078775	F	19/06/199	27	3	3027	2	1	3	BRASIL
2115891	F	13/05/198	37	3	3037	3	2	4	BRASIL
2738368	F	08/11/199	29	3	3029	3	1		BRASIL
2269988	F	19/06/199	24	3	3024	2	1	9	BRASIL
2309254	F	04/04/199	22	3	3022	3	1	3	BRASIL
2077396	F	04/01/199	31	3	3031	2	1	3	BRASIL
2499363	F	31/07/199	28	3	3028	3	4	9	BRASIL
3037274	F	24/09/198	37	3	3037	2	9	9	BRASIL
5717515	F	01/12/199	30	3	3030	3	1	9	BRASIL
5237750	F	06/05/198	35	3	3035	2	2	9	BRASIL

2269988	F	06/11/200	15	3	3015	3	9	1	BRASIL
2793008	F	26/08/199	27	3	3027	3	1	9	BRASIL
5073898	F	16/09/199	27	3	3027	2	4	3	BRASIL
2269988	F	26/05/200	18	3	3018	3	9	9	BRASIL
9698469	F	07/12/200	19	3	3019	2	4	3	BRASIL
2649497	F	16/02/198	36	3	3036	1	4	3	BRASIL
2237571	F	15/05/198	38	3	3038	3	2	2	BRASIL
2707527	F	02/12/200	19	3	3019	3	4	3	BRASIL
2363070	F	14/01/198	35	3	3035	3	4	9	BRASIL
2688611	F	11/03/199	28	3	3028	3	1		BRASIL
10464	F	12/10/198	35	3	3035	3	4		BRASIL
2615797	F	09/10/199	21	3	3021	4	4		BRASIL
2655411	F	11/09/199	29	3	3029	2	4		BRASIL
2499363	F	30/07/200	15	3	3015	3	4	9	BRASIL
2748223	F	10/04/198	39	3	3039	2	4		BRASIL
24074	F	22/11/198	32	3	3032	2	4	3	BRASIL
3956369	F	05/04/199	28	3	3028	2	4	9	BRASIL
3151794	F	18/01/199	30	3	3030	2	4		BRASIL
2688689	F	02/02/199	30	3	3030	2	1	9	BRASIL
3956369	F	21/04/199	22	3	3022	3	4	9	BRASIL
9554157	F	19/09/199	27	3	3027	1	9		BRASIL
2655411	F	26/10/199	29	3	3029	3	9		BRASIL
2078732	F	19/09/198	32	3	3032	3	1	4	BRASIL
3151794	F	26/10/199	28	3	3028	3	1	3	BRASIL
3956369	F	01/01/199	23	3	3023	2	9		BRASIL
3366219	F	15/02/199	31	3	3031	3	1	4	BRASIL
2079186	F	07/09/198	31	3	3031	3	1	9	BRASIL
6995462	F	30/04/198	35	3	3035	3	4		BRASIL
2191164	F	16/05/199	26	3	3026	3	4	9	BRASIL
2078015	F	01/04/198	35	3	3035	2	1	9	BRASIL
2738368	F	11/09/197	42	3	3042	3	2		BRASIL
2297434	F	13/11/199	26	3	3026	1	4	2	BRASIL
3956369	F	13/08/200	19	3	3019	3	4	2	BRASIL
2688611	F	22/03/198	38	3	3038	2	1	4	BRASIL
2081377	F	19/10/200	17	3	3017	3	4	1	BRASIL
2077620	F	27/07/198	38	3	3038	3	4	3	BRASIL
2494523	F	13/10/198	32	3	3032	3	4	9	BRASIL
10464	F	22/04/198	37	3	3037	2	9		BRASIL
2309254	F	06/12/198	34	3	3034	3	4		BRASIL
2079542	F	02/01/199	31	3	3031	3	1	4	BRASIL
2218690	F	11/03/199	30	3	3030	3	4	9	BRASIL
2690764	F	05/04/199	31	3	3031	2	1	3	BRASIL
2688611	F	09/04/198	36	3	3036	3	1		BRASIL
2079542	F	26/10/198	34	3	3034	2	1	3	BRASIL
2604388	F	02/02/198	36	3	3036	3	1	4	BRASIL
2499363	F	04/10/198	32	3	3032	3	4	9	BRASIL
2077523	F	30/12/199	28	3	3028	3	1		BRASIL

2146355	F	20/08/198	37	3	3037	3	4	9	BRASIL
3030121	F	23/06/198	35	3	3035	2	4		BRASIL
2515369	F	01/08/200	15	3	3015	3	4	9	BRASIL
2384299	F	14/02/198	34	3	3034	3	1	9	BRASIL
2707527	F	22/09/199	26	3	3026	3	4	3	BRASIL
2079186	F	09/11/198	32	3	3032	3	9	9	BRASIL
2494523	F	31/08/198	35	3	3035	2	1	9	BRASIL
2249472	F	19/06/199	29	3	3029	3	1	4	BRASIL
7260784	F	05/05/199	22	3	3022	4	4		BRASIL
6854729	F	24/05/199	29	3	3029	2	1	3	BRASIL
5498465	F	25/03/200	17	3	3017	3	4	2	BRASIL
2499363	F	16/06/198	31	3	3031	2	4		BRASIL
2752700	F	14/03/199	29	3	3029	3	4	3	BRASIL
9554157	F	11/05/198	31	3	3031	3	9		BRASIL
2738368	F	18/07/198	35	3	3035	3	1	4	BRASIL
2521431	F	05/08/198	35	3	3035	3	1	3	BRASIL
2738309	F	19/06/199	24	3	3024	3	1	3	BRASIL
2789590	F	13/06/198	37	3	3037	3	1	4	BRASIL
7371985	F	22/11/199	24	3	3024	3	1	3	BRASIL
2338424	F	18/08/197	41	3	3041	3	4		BRASIL
2078015	F	17/11/199	23	3	3023	3	1	9	BRASIL
5718368	F	14/03/198	40	3	3040	4	1	9	BRASIL
2319659	F	04/04/199	30	3	3030	3	4	4	BRASIL
6429459	F	05/03/199	28	3	3028	3	1	3	BRASIL
2022621	F	30/04/198	34	3	3034	2	1		BRASIL
9119701	F	25/08/198	34	3	3034	2	4	3	BRASIL
3193993	F	31/03/199	29	3	3029	2	9	9	BRASIL
2707527	F	11/06/198	32	3	3032	3	4	2	BRASIL
2269988	F	05/04/198	36	3	3036	3	1	2	BRASIL
7479387	F	26/01/198	35	3	3035	2	9	9	BRASIL
2384299	F	20/02/199	26	3	3026	2	1	9	BRASIL
2738368	F	29/01/198	40	3	3040	3	1	2	BRASIL
2521385	F	28/09/198	34	3	3034	2	1	3	BRASIL
2028840	F	23/12/198	33	3	3033	2	1	9	BRASIL
2707675	F	18/09/198	35	3	3035	3	1	9	BRASIL
10464	F	04/02/200	21	3	3021	3	9		BRASIL
6003575	F	08/06/199	21	3	3021	2	1		BRASIL
24074	F	05/10/199	26	3	3026	3	4	3	BRASIL
3151794	F	27/05/199	26	3	3026	1	4		BRASIL
2146355	F	17/03/198	31	3	3031	3	1	4	BRASIL
9297502	F	22/07/198	36	3	3036	2	4	9	BRASIL
5907594	F	20/03/197	41	3	3041	2	1	9	BRASIL
2752700	F	23/03/200	19	3	3019	3	4	2	BRASIL
2649497	F	13/02/198	32	3	3032	3	9	9	BRASIL
3956369	F	26/03/197	42	3	3042	1	4		BRASIL
9255826	F	08/07/198	34	3	3034	3	1	3	BRASIL
3956369	F	15/01/199	23	3	3023	2	9		BRASIL

2409275	F	04/05/198	37	3	3037	2	1		BRASIL
2079542	F	15/05/198	31	3	3031	3	1	4	BRASIL
2752700	F	09/05/199	29	3	3029	3	1	3	BRASIL
2079542	F	16/04/197	41	3	3041	2	1	4	BRASIL
2082462	F	27/05/198	32	3	3032	3	1	3	BRASIL
2591049	F	11/10/200	18	3	3018	3	4	9	BRASIL
6854729	F	24/03/198	36	3	3036	3	1	3	BRASIL
2200473	F	13/10/199	26	3	3026	2	9		BRASIL
2499363	F	11/07/199	26	3	3026	3	4		BRASIL
2171945	F	28/08/199	30	3	3030	3	1	3	BRASIL
4013484	F	28/06/199	26	3	3026	2	4	3	BRASIL
2499363	F	31/01/200	19	3	3019	2	4		BRASIL
3367371	F	24/06/198	39	3	3039	2	1		BRASIL
2499363	F	20/05/198	31	3	3031	3	4	9	BRASIL
2337754	F	21/11/199	23	3	3023	3	1	3	BRASIL
5717515	F	02/03/199	27	3	3027	3	9	9	BRASIL
3956369	F	16/11/199	24	3	3024	3	4		BRASIL
2017245	F	20/03/198	34	3	3034	2	4	3	BRASIL
2591049	F	23/01/199	28	3	3028	1	4	9	BRASIL
6988938	F	27/09/198	40	3	3040	2	1	9	BRASIL
2755165	F	29/10/198	36	3	3036	3	1	3	BRASIL
2254964	F	28/01/199	31	3	3031	3	1		BRASIL
2089602	F	21/04/198	34	3	3034	2	1		BRASIL
3151794	F	22/10/199	21	3	3021	3	4		BRASIL
2078015	F	26/03/199	29	3	3029	3	4	9	BRASIL
2237571	F	29/08/198	39	3	3039	3	1	3	BRASIL
2460262	F	15/10/197	47	3	3047	4	4	3	BRASIL
2269988	F	02/02/199	23	3	3023	2	2	9	BRASIL
2319659	F	02/07/198	31	3	3031	3	1	1	BRASIL
2159252	F	27/04/198	37	3	3037	2	1	9	BRASIL
2081768	F	18/03/199	29	3	3029	3	1		BRASIL
3956369	F	13/05/198	36	3	3036	3	4		BRASIL
2079240	F	25/12/198	37	3	3037	1	2	3	BRASIL
2687127	F	06/11/197	42	3	3042	2	1	2	BRASIL
2655411	F	11/12/198	34	3	3034	3	4		BRASIL
2295407	F	28/05/198	40	3	3040	3	4	9	BRASIL
2550792	F	08/10/198	40	3	3040	3	1	4	BRASIL
2594714	F	27/01/198	36	3	3036	3	1	3	BRASIL
3151794	F	17/01/198	32	3	3032	3	4		BRASIL
2111640	F	28/12/199	26	3	3026	3	4	9	BRASIL
6984649	F	06/04/199	26	3	3026	2	1	9	BRASIL
9744665	F	13/07/198	37	3	3037	2	9	9	BRASIL
2111640	F	25/06/199	30	3	3030	3	4	2	BRASIL
9582800	F	29/04/198	32	3	3032	4	9	9	BRASIL
2752700	F	19/05/199	30	3	3030	3	4	9	BRASIL
2078015	F	25/04/199	25	3	3025	3	1	9	BRASIL
2082349	F	04/11/198	35	3	3035	1	1	9	BRASIL

10464	F	04/01/198	38	3	3038	2	4		BRASIL
2362287	F	24/02/199	31	3	3031	2	4		BRASIL
3978044	F	31/01/199	22	3	3022	3	9		BRASIL
2153084	F	17/11/199	26	3	3026	3	1	3	BRASIL
2499363	F	24/09/199	27	3	3027	3	4		BRASIL
2752700	F	03/08/199	30	3	3030	3	4	3	BRASIL
2786117	F	09/07/198	32	3	3032	3	1		BRASIL
5833418	F	03/10/199	22	3	3022	2	4		BRASIL
5498465	F	19/10/198	40	3	3040	3	4	2	BRASIL
2786117	F	02/05/199	23	3	3023	2	1	9	BRASIL
9297502	F	01/09/199	28	3	3028	3	4		BRASIL
2269988	F	30/09/198	33	3	3033	3	9	9	BRASIL
3157245	F	25/06/198	32	3	3032	3	1	3	BRASIL
2738368	F	27/02/198	38	3	3038	3	1		BRASIL
5883229	F	04/06/199	27	3	3027	3	1	4	BRASIL
2021463	F	10/05/200	16	3	3016	2	4	3	BRASIL
7047428	F	18/04/198	38	3	3038	1	9		BRASIL
5718368	F	15/03/198	40	3	3040	2	1	9	BRASIL
2126494	F	26/03/198	32	3	3032	3	4	3	BRASIL
2688611	F	12/03/198	38	3	3038	3	1		BRASIL
105058	F	21/06/198	38	3	3038	2	4		BRASIL
15318	F	27/08/198	38	3	3038	3	1	4	BRASIL
2655411	F	05/03/198	38	3	3038	2	4		BRASIL
2786117	F	21/10/199	27	3	3027	3	4	4	BRASIL
6627595	F	29/03/199	28	3	3028	3	4		BRASIL
2591049	F	06/01/199	27	3	3027	3	1		BRASIL
2078015	F	19/03/200	16	3	3016	3	4	9	BRASIL
7013981	F	30/06/198	38	3	3038	4	9	9	BRASIL
2159252	F	10/03/198	36	3	3036	2	4	9	BRASIL
2077396	F	29/10/198	31	3	3031	2	1	9	BRASIL
2269988	F	27/05/198	38	3	3038	3	9	9	BRASIL
2269988	F	20/09/198	37	3	3037	3	1	9	BRASIL
2591049	F	03/11/199	30	3	3030	3	1	1	BRASIL
2040069	F	20/12/199	29	3	3029	3	1	3	BRASIL
2025361	F	01/03/199	22	3	3022	1	1	4	BRASIL
2752700	F	15/10/198	41	3	3041	3	4	9	BRASIL
2798298	F	10/03/199	27	3	3027	2	1	3	BRASIL
2384299	F	06/02/199	23	3	3023	3	1	9	BRASIL
2652706	F	07/04/198	40	3	3040	2	4	3	BRASIL
3956369	F	08/01/200	18	3	3018	3	9		BRASIL
2244276	F	27/05/198	36	3	3036	3	1	3	BRASIL
2077396	F	13/07/199	27	3	3027	3	1	3	BRASIL
434	F	30/01/200	21	3	3021	3	4	3	BRASIL
2499363	F	28/05/198	37	3	3037	3	4	9	BRASIL
2208172	F	17/08/199	21	3	3021	2	4	9	BRASIL
2079542	F	11/11/198	40	3	3040	3	4	4	BRASIL
2082187	F	27/11/198	38	3	3038	2	1		BRASIL

7640307	F	24/06/199[illegible]	27	3	3027	3	1	3	BRASIL
3956369	F	23/02/200[illegible]	18	3	3018	3	4		BRASIL
2081091	F	21/07/199[illegible]	26	3	3026	3	2	3	BRASIL
24074	F	22/09/199	29	3	3029	2	1	3	BRASIL
2408570	F	21/11/198	39	3	3039	3	1	2	BRASIL
24074	F	06/03/199[illegible]	24	3	3024	3	4	3	BRASIL
2244276	F	04/10/198[illegible]	31	3	3031	2	1	9	BRASIL
2752700	F	22/10/199	29	3	3029	2	4	1	BRASIL
2077396	F	01/05/199[illegible]	25	3	3025	3	1	3	BRASIL
2078015	F	06/06/198[illegible]	38	3	3038	2	1	9	BRASIL
2786680	F	21/11/199[illegible]	27	3	3027	2	1	4	BRASIL
2097613	F	07/03/199[illegible]	26	3	3026	3	1	3	BRASIL
2309254	F	19/01/200[illegible]	14	3	3014	3	4	2	BRASIL
7257406	F	26/02/198[illegible]	35	3	3035	3	4	3	BRASIL
6848710	F	16/06/198[illegible]	33	3	3033	2	4		BRASIL
2754738	F	25/08/198[illegible]	38	3	3038	3	4		BRASIL
434	F	25/06/200[illegible]	16	3	3016	3	9	9	BRASIL
2269988	F	14/07/198[illegible]	40	3	3040	3	2	9	BRASIL
6854729	F	17/06/199	29	3	3029	1	1	3	BRASIL
6601804	F	20/10/198[illegible]	40	3	3040	2	1	3	BRASIL
2078015	F	20/04/199	29	3	3029	2	1	9	BRASIL
2079542	F	03/02/198[illegible]	33	3	3033	3	9	9	BRASIL
4001303	F	14/03/199[illegible]	29	3	3029	2	4	0	BRASIL
24074	F	02/04/200	20	3	3020	3	1	3	BRASIL
2784874	F	12/01/198	40	3	3040	2	1	3	BRASIL
2078775	F	03/07/200[illegible]	15	3	3015	3	1		BRASIL
5171946	F	11/01/198[illegible]	36	3	3036	3	4	3	BRASIL
2079542	F	03/07/198	39	3	3039	2	1	4	BRASIL
2237571	F	30/07/198[illegible]	35	3	3035	2	1	3	BRASIL
2309254	F	30/05/199[illegible]	26	3	3026	2	4		BRASIL
6988938	F	01/07/198[illegible]	33	3	3033	3	4	4	BRASIL
2298317	F	20/12/200[illegible]	17	3	3017	1	4	9	BRASIL
2410281	F	08/10/198[illegible]	35	3	3035	2	4	2	BRASIL
2259907	F	02/01/198[illegible]	34	3	3034	3	1		BRASIL
2079542	F	28/12/198[illegible]	33	3	3033	2	9	9	BRASIL
2707527	F	04/12/199[illegible]	25	3	3025	3	4	3	BRASIL
2126494	F	30/07/199[illegible]	25	3	3025	3	9		BRASIL
2337754	F	18/04/198[illegible]	32	3	3032	2	1		BRASIL
2786117	F	14/02/198[illegible]	33	3	3033	2	4		BRASIL
2269988	F	25/09/197[illegible]	45	3	3045	3	1	9	BRASIL
2655411	F	09/11/198[illegible]	34	3	3034	2	4	9	BRASIL
2521296	F	08/04/199[illegible]	25	3	3025	2	1	2	BRASIL
2079410	F	12/02/197[illegible]	43	3	3043	1	9		BRASIL
607126	F	07/02/198	40	3	3040	2	4	9	BRASIL
6020917	F	07/05/199[illegible]	26	3	3026	3	3	3	BRASIL
2127687	F	26/01/197[illegible]	43	3	3043	3	1	3	BRASIL
24074	F	03/06/200[illegible]	17	3	3017	3	4	2	BRASIL

2078015	F	01/06/198[illegible]	35	3	3035	3	1	9	BRASIL
2269988	F	09/04/199[illegible]	25	3	3025	2	9	9	BRASIL
2707527	F	27/09/198[illegible]	38	3	3038	3	4	3	BRASIL
3151794	F	12/05/198[illegible]	35	3	3035	2	4	2	BRASIL
2752700	F	16/06/198[illegible]	40	3	3040	3	1	1	BRASIL
2748223	F	11/08/197[illegible]	41	3	3041	3	1	2	BRASIL
7659415	F	18/04/198[illegible]	40	3	3040	3	1	4	BRASIL
2077426	F	23/10/199[illegible]	23	3	3023	3	1		BRASIL
2077523	F	18/12/199[illegible]	26	3	3026	3	4		BRASIL
13846	F	25/07/200[illegible]	20	3	3020	3	1		BRASIL
2816210	F	27/11/198[illegible]	34	3	3034	3	4	3	BRASIL
2738368	F	22/01/198[illegible]	37	3	3037	3	1	4	BRASIL
5515483	F	14/11/200[illegible]	20	3	3020	3	1	9	BRASIL
6848710	F	18/04/200[illegible]	21	3	3021	3	9		BRASIL
9336508	F	16/11/198[illegible]	36	3	3036	3	4	9	BRASIL
2825309	F	07/10/198[illegible]	31	3	3031	3	9	4	BRASIL
2587335	F	29/03/199[illegible]	30	3	3030	1	4	9	BRASIL
2077396	F	16/07/199[illegible]	27	3	3027	3	1	3	BRASIL
2269988	F	10/11/199[illegible]	25	3	3025	2	9	9	BRASIL
2745798	F	17/01/199[illegible]	27	3	3027	3	1		BRASIL
6697054	F	26/01/199[illegible]	27	3	3027	2	1	9	BRASIL
2452383	F	28/08/199[illegible]	28	3	3028	2	4	2	BRASIL
2159252	F	14/08/198[illegible]	37	3	3037	1	9		BRASIL
2122650	F	28/08/198[illegible]	33	3	3033	2	4	3	BRASIL
3956369	F	10/08/198[illegible]	40	3	3040	3	4	1	BRASIL
2080931	F	19/03/199	30	3	3030	2	1	3	BRASIL
5073898	F	29/05/199[illegible]	31	3	3031	2	4	3	BRASIL
2495015	F	11/10/197[illegible]	45	3	3045	2	2		BRASIL
434	F	08/01/200[illegible]	21	3	3021	2	2	9	BRASIL
2078252	F	13/08/198	39	3	3039	3	1	9	BRASIL
2269988	F	23/04/198[illegible]	33	3	3033	3	9	9	BRASIL
10464	F	31/10/198[illegible]	40	3	3040	3	1	3	BRASIL
2594714	F	08/08/199[illegible]	23	3	3023	3	1		BRASIL
7695004	F	17/09/199	29	3	3029	3	1	2	BRASIL
2078791	F	18/03/198[illegible]	38	3	3038	3	1		BRASIL
2151871	F	22/10/199	29	3	3029	2	1	3	BRASIL
176664	F	02/03/198[illegible]	32	3	3032	3	4	9	BRASIL
3808	F	24/10/198[illegible]	34	3	3034	2	9	9	BRASIL
2244306	F	26/06/198[illegible]	38	3	3038	2	1	2	BRASIL
2309254	F	09/02/199[illegible]	27	3	3027	1	2	0	BRASIL
24074	F	05/05/199[illegible]	24	3	3024	3	4	3	BRASIL
6602533	F	07/10/198[illegible]	32	3	3032	1	4		BRASIL
2079542	F	25/06/198[illegible]	34	3	3034	2	1	4	BRASIL
6848710	F	13/10/198[illegible]	36	3	3036	3	4		BRASIL
2237253	F	01/08/199[illegible]	30	3	3030	3	1		BRASIL
2655411	F	14/11/199	29	3	3029	3	4		BRASIL
2591049	F	01/03/198[illegible]	33	3	3033	2	1	3	BRASIL

2254964	F	25/11/198	31	3	3031	3	2	3	BRASIL
9582800	F	08/02/198	35	3	3035	2	1	9	BRASIL
3956369	F	31/08/198	39	3	3039	1	4		BRASIL
2783789	F	30/11/199	28	3	3028	2	1		BRASIL
7659415	F	23/02/199	22	3	3022	3	2	3	BRASIL
5200105	F	07/02/199	23	3	3023	3	9		BRASIL
6159397	F	22/03/198	38	3	3038	3	2	4	BRASIL
2080664	F	23/09/197	41	3	3041	2	1	2	BRASIL
2655411	F	20/02/199	30	3	3030	3	4		BRASIL
6558143	F	07/09/198	39	3	3039	3	4	9	BRASIL
2078015	F	21/09/198	32	3	3032	3	1	9	BRASIL
7257406	F	03/11/199	28	3	3028	3	4	3	BRASIL
9725	F	07/08/199	25	3	3025	3	1	9	BRASIL
6734014	F	13/06/199	23	3	3023	3	1	9	BRASIL
2127989	F	02/11/197	41	3	3041	3	1	3	BRASIL
2748223	F	08/01/199	27	3	3027	3	1	9	BRASIL
6602533	F	06/09/199	28	3	3028	1	4		BRASIL
2223538	F	19/03/200	20	3	3020	2	1	1	BRASIL
2311682	F	24/04/198	36	3	3036	2	4		BRASIL
2079798	F	22/10/199	24	3	3024	3	1	3	BRASIL
2079542	F	09/07/199	28	3	3028	3	9		BRASIL
2781859	F	26/07/199	28	3	3028	3	1	9	BRASIL
2078104	F	26/08/198	38	3	3038	2	4	3	BRASIL
10464	F	16/10/199	29	3	3029	3	4	9	BRASIL
2205440	F	11/03/200	21	3	3021	3	4		BRASIL
2022621	F	13/12/199	23	3	3023	3	4		BRASIL
2688611	F	25/09/198	39	3	3039	2	1		BRASIL
2655411	F	28/12/200	20	3	3020	3	4		BRASIL
4042085	F	02/08/198	34	3	3034	3	1	3	BRASIL
2738368	F	17/03/199	26	3	3026	3	1	1	BRASIL
2550792	F	31/08/197	42	3	3042	3	1		BRASIL
9194258	F	20/05/198	37	3	3037	3	4		BRASIL
6988938	F	30/03/198	31	3	3031	2	1	9	BRASIL
2080532	F	05/01/198	33	3	3033	4	1	4	BRASIL
2302101	F	19/07/200	20	3	3020	3	1	9	BRASIL
2752700	F	08/11/199	22	3	3022	3	4	4	BRASIL
24074	F	26/08/198	33	3	3033	3	4	3	BRASIL
2079542	F	07/05/199	30	3	3030	2	9		BRASIL
3956369	F	16/01/199	24	3	3024	2	9		BRASIL
2309254	F	22/12/199	26	3	3026	3	4	2	BRASIL
2079887	F	03/01/199	25	3	3025	3	4		BRASIL
3972925	F	10/06/199	25	3	3025	3	4	9	BRASIL
9725	F	05/10/198	37	3	3037	2	1	9	BRASIL
6159397	F	13/04/198	32	3	3032	3	9	9	BRASIL
2082349	F	06/03/199	27	3	3027	3	1	9	BRASIL
2079542	F	23/12/198	37	3	3037	3	1	4	BRASIL
2153882	F	03/02/198	32	3	3032	2	1	9	BRASIL

2270609	F	02/07/199	30	3	3030	3	9		BRASIL
2410281	F	12/09/199	24	3	3024	2	4	3	BRASIL
3956369	F	04/07/198	34	3	3034	3	4	9	BRASIL
2755130	F	22/05/201	10	3	3010	4	9	9	BRASIL
2081377	F	05/07/199	28	3	3028	3	4		BRASIL
2337754	F	07/06/198	35	3	3035	3	4	4	BRASIL
2786117	F	11/07/198	32	3	3032	3	2	2	BRASIL
2027356	F	01/07/198	40	3	3040	2	1	2	BRASIL
2339196	F	15/08/198	37	3	3037	2	4	3	BRASIL
2151871	F	27/03/197	45	3	3045	3	4	9	BRASIL
2266474	F	11/07/198	39	3	3039	3	1	0	BRASIL
2079186	F	03/04/197	41	3	3041	2	4	9	BRASIL
2246988	F	02/05/198	33	3	3033	3	1	2	BRASIL
5590450	F	23/01/199	28	3	3028	3	1	9	BRASIL
3028399	F	28/10/199	22	3	3022	2	1		BRASIL
14109	F	27/02/199	30	3	3030	3	4	3	BRASIL
2246813	F	06/05/198	37	3	3037	1	1	9	BRASIL
2337754	F	01/12/200	17	3	3017	3	4		BRASIL
2079186	F	26/12/199	26	3	3026	1	4	9	BRASIL
6988938	F	11/09/198	37	3	3037	3	1	4	BRASIL
2726734	F	27/07/198	38	3	3038	2	4	9	BRASIL
2081091	F	13/11/198	40	3	3040	2	1	3	BRASIL
2494523	F	16/11/199	28	3	3028	3	1		BRASIL
24074	F	25/06/199	24	3	3024	3	4	3	BRASIL
3006522	F	18/08/198	37	3	3037	3	1		BRASIL
6995462	F	11/05/199	30	3	3030	3	9	9	BRASIL
2080931	F	01/08/199	26	3	3026	1	1	3	BRASIL
24074	F	13/01/199	28	3	3028	2	3	4	BRASIL
5717515	F	31/01/199	29	3	3029	3	9	9	BRASIL
4001303	F	03/09/198	33	3	3033	3	1	4	BRASIL
2655411	F	29/03/198	35	3	3035	3	4		BRASIL
2006448	F	13/08/199	25	3	3025	4	9		BRASIL
2499363	F	13/02/198	38	3	3038	3	4	9	BRASIL
2752700	F	25/06/199	25	3	3025	3	4	9	BRASIL
3012212	F	28/02/198	32	3	3032	1	9		BRASIL
10464	F	01/08/199	29	3	3029	3	9		BRASIL
6159397	F	17/10/198	34	3	3034	2	1	9	BRASIL
24074	F	22/07/199	24	3	3024	3	4	1	BRASIL
24074	F	26/07/198	38	3	3038	2	1	4	BRASIL
2319659	F	25/12/199	29	3	3029	3	4	9	BRASIL
2298317	F	15/03/200	20	3	3020	3	1	9	BRASIL
2499363	F	20/08/198	31	3	3031	3	4	9	BRASIL
24074	F	14/03/197	41	3	3041	3	4		BRASIL
2761041	F	21/07/198	35	3	3035	3	2	9	BRASIL
2237601	F	08/11/199	22	3	3022	3	1	3	BRASIL
5336171	F	28/03/199	31	3	3031	3	4		BRASIL
2078589	F	04/06/198	39	3	3039	3	9	9	BRASIL

6848710	F	26/07/198	31	3	3031	2	4		BRASIL
2001578	F	28/01/198	40	3	3040	3	4	3	BRASIL
2793008	F	04/04/198	32	3	3032	3	9	9	BRASIL
2081938	F	22/02/198	38	3	3038	3	3	4	BRASIL
3037274	F	12/11/198	36	3	3036	3	9	9	BRASIL
6146376	F	18/02/199	26	3	3026	2	1	9	BRASIL
2298317	F	22/11/198	31	3	3031	3	2	9	BRASIL
2269988	F	12/11/199	28	3	3028	2	4	3	BRASIL
24074	F	04/02/198	39	3	3039	2	4	3	BRASIL
2708779	F	20/08/198	32	3	3032	3	1	9	BRASIL
8028	F	22/04/198	34	3	3034	1	1	4	BRASIL
7257406	F	25/07/199	26	3	3026	3	4	4	BRASIL
6995462	F	01/09/197	45	3	3045	3	9	9	BRASIL
2494523	F	18/06/200	20	3	3020	2	1		BRASIL
9643486	F	12/12/197	46	3	3046	4	4		BRASIL
3028399	F	19/05/198	33	3	3033	3	1		BRASIL
2436450	F	19/05/199	23	3	3023	3	1		BRASIL
2789590	F	02/02/197	47	3	3047	2	1	9	BRASIL
2232057	F	05/09/197	41	3	3041	2	1		BRASIL
2781859	F	03/03/199	30	3	3030	2	1	9	BRASIL
9725	F	31/08/198	36	3	3036	2	4	3	BRASIL
2795655	F	15/11/197	44	3	3044	4	4	3	BRASIL
2710935	F	25/01/197	48	3	3048	2	1		BRASIL
2022621	F	14/11/199	29	3	3029	3	1	4	BRASIL
2499363	F	27/12/199	23	3	3023	3	4	9	BRASIL
2615797	F	29/05/199	23	3	3023	4	4	3	BRASIL
2825589	F	10/02/198	34	3	3034	2	1	9	BRASIL
2688611	F	22/04/199	27	3	3027	2	1		BRASIL
2495015	F	20/11/197	49	3	3049	3	4		BRASIL
2079542	F	17/10/198	37	3	3037	2	1		BRASIL
2738368	F	12/10/198	34	3	3034	2	1	3	BRASIL
2269783	F	29/11/199	30	3	3030	3	1	3	BRASIL
2384299	F	17/09/198	32	3	3032	3	1	9	BRASIL
2078015	F	21/11/198	35	3	3035	2	1	9	BRASIL
2655411	F	02/03/198	38	3	3038	3	4		BRASIL
2738368	F	07/10/199	29	3	3029	3	1	1	BRASIL
2388057	F	27/01/199	26	3	3026	3	2		BRASIL
13633	F	16/07/200	18	3	3018	3	1	9	BRASIL
2655411	F	29/09/198	31	3	3031	3	4		BRASIL
2499363	F	02/02/199	24	3	3024	3	4		BRASIL
27022	F	15/09/198	35	3	3035	3	4	9	BRASIL
2269988	F	12/06/199	31	3	3031	3	4	9	BRASIL
2208857	F	16/11/198	39	3	3039	1	1	1	BRASIL
2089777	F	27/10/199	29	3	3029	3	9		BRASIL
24074	F	18/01/198	34	3	3034	3	4	3	BRASIL
2436450	F	29/06/199	30	3	3030	2	1		BRASIL
2077477	F	14/01/197	48	3	3048	1	4	1	BRASIL

2099209	F	04/03/198	39	3	3039	3	4		BRASIL
2283239	F	30/10/199	22	3	3022	3	4	9	BRASIL
2077396	F	01/11/199	27	3	3027	3	1	4	BRASIL
2738368	F	03/07/199	25	3	3025	2	1		BRASIL
13633	F	27/03/198	35	3	3035	3	4	3	BRASIL
2711184	F	14/09/199	30	3	3030	3	4	2	BRASIL
2079720	F	28/11/200	17	3	3017	4	9	9	BRASIL
4013484	F	06/04/199	30	3	3030	1	4	9	BRASIL
2079038	F	10/03/198	40	3	3040	2	1		BRASIL
2452383	F	07/07/198	36	3	3036	3	4	3	BRASIL
2077396	F	01/07/199	29	3	3029	3	9	4	BRASIL
2319659	F	20/12/199	23	3	3023	2	1	1	BRASIL
6716938	F	23/09/200	18	3	3018	3	1	3	BRASIL
2223546	F	16/07/199	23	3	3023	2	1	1	BRASIL
5717515	F	14/11/198	34	3	3034	3	9	9	BRASIL
2594714	F	11/02/199	31	3	3031	3	2	9	BRASIL
2261987	F	14/07/198	31	3	3031	3	1	2	BRASIL
2078015	F	24/04/199	30	3	3030	2	4	9	BRASIL
2560852	F	26/02/199	24	3	3024	3	4		BRASIL
2655411	F	23/07/198	38	3	3038	2	4		BRASIL
2503654	F	28/10/199	25	3	3025	2	9	2	BRASIL
2244306	F	02/03/199	22	3	3022	3	1	3	BRASIL
2022621	F	20/10/199	23	3	3023	3	1		BRASIL
2499363	F	29/08/198	32	3	3032	3	1	9	BRASIL
3012212	F	15/02/198	32	3	3032	3	1		BRASIL
2237571	F	30/09/199	30	3	3030	3	1	2	BRASIL
7044666	F	04/07/198	34	3	3034	2	1	9	BRASIL
5498465	F	25/10/198	36	3	3036	3	4	3	BRASIL
2080443	F	20/05/199	21	3	3021	3	1	3	BRASIL
2200473	F	23/05/198	34	3	3034	2	4	3	BRASIL
2078015	F	02/02/198	36	3	3036	1	4	9	BRASIL
2077396	F	03/02/198	33	3	3033	3	1	9	BRASIL
6602533	F	04/04/198	37	3	3037	1	4		BRASIL
2760843	F	17/04/199	29	3	3029	3	4	9	BRASIL
2755130	F	24/02/199	31	3	3031	2	4	3	BRASIL
2786117	F	28/11/199	28	3	3028	1	4	1	BRASIL
7257406	F	25/06/199	24	3	3024	3	4	3	BRASIL
2269988	F	13/09/199	26	3	3026	3	4	9	BRASIL
7257406	F	28/04/198	40	3	3040	3	2	4	BRASIL
7659415	F	07/05/198	35	3	3035	3	9	9	BRASIL
2408570	F	03/12/198	39	3	3039	3	4		BRASIL
2776006	F	04/05/198	35	3	3035	2	1	3	BRASIL
2781859	F	21/02/197	42	3	3042	3	1	9	BRASIL
2319659	F	28/06/198	32	3	3032	2	5	9	BRASIL
2097117	F	05/05/198	34	3	3034	3	9		BRASIL
2200473	F	07/05/199	24	3	3024	3	4	9	BRASIL
2081695	F	04/08/198	39	3	3039	3	1	2	BRASIL

2338424	F	05/06/198	40	3	3040	3	4	9	BRASIL
2269988	F	18/08/199	28	3	3028	3	9		BRASIL
434	F	24/04/198	39	3	3039	3	9	9	BRASIL
2738368	F	11/10/198	35	3	3035	3	1		BRASIL
2615797	F	06/10/200	20	3	3020	2	4		BRASIL
6779522	F	30/07/200	15	3	3015	2	9		BRASIL
2080052	F	05/08/198	36	3	3036	2	1	3	BRASIL
2040069	F	26/08/199	28	3	3028	2	1	3	BRASIL
2537788	F	30/12/198	35	3	3035	3	1	3	BRASIL
2078015	F	26/07/199	23	3	3023	3	4	9	BRASIL
2078015	F	10/05/199	28	3	3028	3	2	9	BRASIL
2269988	F	11/12/199	22	3	3022	2	1	9	BRASIL
3956369	F	22/04/198	38	3	3038	3	4	9	BRASIL
2499363	F	15/08/198	31	3	3031	3	4	9	BRASIL
2755130	F	06/05/197	43	3	3043	2	1	9	BRASIL
2237601	F	11/11/199	24	3	3024	3	1	2	BRASIL
2275287	F	28/10/199	28	3	3028	3	1	9	BRASIL
3028399	F	21/05/200	17	3	3017	3	1		BRASIL
4001303	F	05/05/198	35	3	3035	3	4	2	BRASIL
3649563	F	14/03/199	26	3	3026	3	9	9	BRASIL
24074	F	12/09/199	26	3	3026	3	4	1	BRASIL
2752700	F	22/02/198	36	3	3036	2	1	2	BRASIL
2604388	F	28/08/198	40	3	3040	3	4	3	BRASIL
2781859	F	21/06/200	18	3	3018	3	1	9	BRASIL
2707527	F	10/04/198	35	3	3035	3	4	3	BRASIL
2078015	F	09/09/199	30	3	3030	2	9	9	BRASIL
2077477	F	04/02/199	29	3	3029	2	1	9	BRASIL
9554157	F	04/03/198	39	3	3039	2	9		BRASIL
2319659	F	23/11/199	30	3	3030	2	4	3	BRASIL
2001578	F	23/03/199	28	3	3028	3	1	4	BRASIL
2655411	F	23/09/197	42	3	3042	3	4		BRASIL
2521431	F	05/10/198	32	3	3032	3	1	3	BRASIL
2589737	F	27/02/198	33	3	3033	2	4	4	BRASIL
2079542	F	25/12/198	31	3	3031	3	9		BRASIL
3012212	F	05/08/198	35	3	3035	2	1	4	BRASIL
2747871	F	07/09/198	31	3	3031	3	4	3	BRASIL
2206528	F	14/12/199	24	3	3024	3	1	9	BRASIL
24074	F	10/04/198	34	3	3034	2	4	2	BRASIL
2362287	F	28/12/199	22	3	3022	3	4		BRASIL
2270609	F	05/12/200	17	3	3017	3	1	2	BRASIL
5200105	F	05/09/198	32	3	3032	2	9		BRASIL
4053907	F	27/07/198	31	3	3031	1	1	3	BRASIL
2022621	F	24/03/198	37	3	3037	3	1		BRASIL
2786117	F	20/10/200	20	3	3020	4	4	9	BRASIL
2808579	F	20/01/198	35	3	3035	1	3		BRASIL
2752700	F	26/04/199	21	3	3021	2	4	9	BRASIL
2825589	F	29/01/199	23	3	3023	3	4	2	BRASIL

2078015	F	21/02/1984	37	3	3037	2	1	9	BRASIL
2081628	F	23/03/1984	37	3	3037	3	9		BRASIL
4001303	F	28/12/1988	32	3	3032	3	4	3	BRASIL
2079542	F	22/07/1986	34	3	3034	3	1	4	BRASIL
2323397	F	10/05/199	30	3	3030	3	4	3	BRASIL
2270609	F	12/12/1998	22	3	3022	3	1	9	BRASIL
2781859	F	14/05/1982	39	3	3039	2	4	9	BRASIL
3007847	F	04/08/1983	37	3	3037	3	1		BRASIL
3157245	F	15/12/2002	18	3	3018	3	1	3	BRASIL
3037274	F	16/02/1979	42	3	3042	2	9	9	BRASIL
3151794	F	14/11/1995	25	3	3025	3	4		BRASIL
9194258	F	21/12/1986	34	3	3034	3	4	9	BRASIL
24074	F	21/09/1998	22	3	3022	3	2	3	BRASIL
3400573	F	17/03/1992	29	3	3029	3	9		BRASIL
6995462	F	22/06/200	19	3	3019	3	4	3	BRASIL
2078082	F	28/02/199	30	3	3030	3	4		BRASIL
2082187	F	13/04/199	30	3	3030	2	1		BRASIL
9060456	F	19/10/1993	27	3	3027	3	4	4	BRASIL
2499363	F	12/05/1989	31	3	3031	2	4		BRASIL
2738368	F	23/06/1988	32	3	3032	3	1		BRASIL
3021114	F	06/04/1992	29	3	3029	3	4		BRASIL
2452383	F	27/11/1997	23	3	3023	3	4	4	BRASIL
2153084	F	10/03/1992	29	3	3029	3	2	4	BRASIL
2269988	F	28/04/1999	22	3	3022	3	1		BRASIL
2594714	F	02/01/199	30	3	3030	3	1	4	BRASIL
2537788	F	17/10/1989	31	3	3031	3	1	3	BRASIL
7257406	F	30/10/1986	34	3	3034	1	1	3	BRASIL
2802511	F	10/06/1982	38	3	3038	3	4	9	BRASIL
2687127	F	09/03/1984	37	3	3037	3	1	2	BRASIL
2786117	F	12/04/2000	21	3	3021	2	4	3	BRASIL
2726726	F	05/05/1995	26	3	3026	2	1	2	BRASIL
3007847	F	09/03/1986	35	3	3035	3	1		BRASIL
5515483	F	09/09/1985	35	3	3035	3	1	3	BRASIL
2568713	F	28/03/1994	27	3	3027	2	1		BRASIL
7257406	F	14/08/1998	22	3	3022	3	4	3	BRASIL
2081938	F	06/01/1989	32	3	3032	2	1	9	BRASIL
2022621	F	03/04/1978	42	3	3042	3	1		BRASIL
2078015	F	16/09/1987	33	3	3033	3	9	9	BRASIL
2361787	F	18/07/1987	33	3	3033	3	4	4	BRASIL
3018903	F	23/01/198	40	3	3040	3	4	9	BRASIL
2793008	F	21/02/200	20	3	3020	3	4	9	BRASIL
2795655	F	08/09/1997	23	3	3023	2	4	3	BRASIL
2426099	F	20/09/1987	33	3	3033	3	4	4	BRASIL
2292912	F	11/07/198	39	3	3039	3	1	2	BRASIL
13633	F	04/09/1996	24	3	3024	3	1	2	BRASIL
2079127	F	07/06/1987	34	3	3034	3	1	9	BRASIL
2096463	F	09/10/1978	42	3	3042	3	1	1	BRASIL

2309254	F	27/10/197	42	3	3042	2	4		BRASIL
3521133	F	15/02/199	30	3	3030	3	4	3	BRASIL
2096463	F	17/12/198	33	3	3033	2	4	3	BRASIL
2738309	F	13/03/198	34	3	3034	3	1		BRASIL
7620098	F	26/10/198	40	3	3040	2	9	3	BRASIL
2089602	F	17/01/198	32	3	3032	1	4		BRASIL
6734014	F	08/03/197	42	3	3042	3	9	9	BRASIL
6542638	F	10/01/199	27	3	3027	2	9		BRASIL
2655411	F	10/08/198	31	3	3031	2	4		BRASIL
3508528	F	04/05/198	31	3	3031	3	4		BRASIL
2379333	F	30/10/199	26	3	3026	3	1	3	BRASIL
434	F	21/05/198	37	3	3037	4	9	9	BRASIL
2654024	F	04/08/199	28	3	3028	3	9	9	BRASIL
2786117	F	05/02/198	34	3	3034	3	4	3	BRASIL
176664	F	03/01/199	22	3	3022	3	2	3	BRASIL
24074	F	20/08/198	41	3	3041	2	4	9	BRASIL
5101654	F	07/03/198	32	3	3032	2	1		BRASIL
3037274	F	12/09/199	30	3	3030	3	9	9	BRASIL
2518058	F	20/09/198	33	3	3033	4	4	4	BRASIL
2078732	F	14/02/199	22	3	3022	1	2	3	BRASIL
2085194	F	13/06/198	39	3	3039	3	1		BRASIL
4026896	F	29/11/197	48	3	3048	3	4	3	BRASIL
10464	F	13/09/198	38	3	3038	3	1		BRASIL
2078740	F	21/04/198	32	3	3032	2	1	4	BRASIL
2338424	F	28/04/199	21	3	3021	2	4		BRASIL
2487756	F	18/12/199	30	3	3030	2	4		BRASIL
2269988	F	21/01/199	23	3	3023	3	9	9	BRASIL
2058502	F	10/04/200	20	3	3020	3	1		BRASIL
2738368	F	08/07/199	24	3	3024	3	4	9	BRASIL
3005402	F	31/05/198	38	3	3038	3	1		BRASIL
6625649	F	02/10/198	36	3	3036	2	1		BRASIL
9628	F	02/08/199	27	3	3027	1	9		BRASIL
2504332	F	04/02/198	36	3	3036	3	1	4	BRASIL
2793008	F	08/05/200	20	3	3020	2	9	9	BRASIL
7758391	F	25/12/198	40	3	3040	1	1	9	BRASIL
2079542	F	13/01/198	39	3	3039	2	1	4	BRASIL
2793008	F	28/04/198	38	3	3038	2	1	9	BRASIL
2741989	F	24/09/198	35	3	3035	2	1	3	BRASIL
2738368	F	20/04/198	32	3	3032	3	1	1	BRASIL
7257406	F	17/10/198	35	3	3035	2	4	3	BRASIL
5101654	F	09/05/199	28	3	3028	3	4	4	BRASIL
2752700	F	24/03/198	31	3	3031	2	4		BRASIL
2436450	F	18/07/199	24	3	3024	3	1		BRASIL
2686953	F	31/12/198	40	3	3040	3	1		BRASIL
2065665	F	21/05/197	42	3	3042	2	4	9	BRASIL
3151794	F	13/10/198	35	3	3035	1	4		BRASIL
2825589	F	17/07/199	25	3	3025	2	1	2	BRASIL

2696940	F	21/03/198	33	3	3033	4	2	9	BRASIL
24074	F	19/07/198	32	3	3032	3	1	3	BRASIL
3956369	F	24/10/198	36	3	3036	2	4	9	BRASIL
3157245	F	30/06/198	36	3	3036	2	1	9	BRASIL
2750988	F	23/11/198	33	3	3033	2	4		BRASIL
2611686	F	17/10/198	37	3	3037	3	4	3	BRASIL
2521385	F	29/11/199	29	3	3029	3	1	4	BRASIL
2550792	F	05/08/198	32	3	3032	3	1	4	BRASIL
2078015	F	04/07/198	31	3	3031	2	1	9	BRASIL
2079542	F	20/04/199	28	3	3028	2	1	4	BRASIL
2237253	F	04/06/198	36	3	3036	3	1	9	BRASIL
2798298	F	20/05/198	34	3	3034	3	1	3	BRASIL
2298317	F	02/03/198	40	3	3040	3	1		BRASIL
5717515	F	16/10/199	24	3	3024	3	9	9	BRASIL
2752700	F	25/06/199	28	3	3028	3	4	3	BRASIL
2007037	F	05/07/198	38	3	3038	3	1		BRASIL
2223546	F	16/05/198	31	3	3031	1	4	3	BRASIL
3151794	F	17/11/198	39	3	3039	3	4		BRASIL
2081083	F	08/09/199	28	3	3028	3	1	4	BRASIL
3956369	F	19/01/198	35	3	3035	3	9		BRASIL
2337754	F	25/08/199	30	3	3030	2	1	3	BRASIL
2655411	F	22/08/199	27	3	3027	3	4	9	BRASIL
2687038	F	01/09/198	33	3	3033	1	1		BRASIL
3012212	F	10/02/199	27	3	3027	3	1	9	BRASIL
3956369	F	31/05/199	26	3	3026	3	4		BRASIL
3058336	F	24/05/199	29	3	3029	3	1	9	BRASIL
7044666	F	17/11/200	19	3	3019	3	4		BRASIL
2688611	F	13/06/199	30	3	3030	3	1		BRASIL
2079798	F	15/09/198	38	3	3038	3	4	3	BRASIL
2726726	F	03/11/198	37	3	3037	3	1	1	BRASIL
3956369	F	02/08/199	30	3	3030	3	4		BRASIL
2649497	F	20/04/197	42	3	3042	1	9	9	BRASIL
2206528	F	18/10/200	20	3	3020	3	4	4	BRASIL
2269988	F	14/07/199	30	3	3030	2	9	9	BRASIL
2269988	F	29/11/199	27	3	3027	3	1	9	BRASIL
2784874	F	18/06/198	36	3	3036	1	9	3	BRASIL
2078015	F	09/05/199	27	3	3027	3	9	9	BRASIL
2077396	F	10/04/199	22	3	3022	3	1	1	BRASIL
2765934	F	04/07/198	31	3	3031	2	4	1	BRASIL
2452383	F	15/06/198	33	3	3033	2	4	2	BRASIL
7421710	F	13/12/199	24	3	3024	3	9		BRASIL
2287250	F	21/06/198	39	3	3039	2	4	9	BRASIL
2058391	F	23/02/198	34	3	3034	3	9	9	BRASIL
2410443	F	20/06/197	41	3	3041	2	1	9	BRASIL
2078376	F	10/07/197	43	3	3043	3	1	4	BRASIL
7530005	F	02/09/199	30	3	3030	3	4	9	BRASIL
7421710	F	18/05/198	36	3	3036	3	9		BRASIL

2273438	F	08/05/198	32	3	3032	2	9	9	BRASIL
2079186	F	23/08/198	39	3	3039	1	4	2	BRASIL
2078082	F	05/07/198	39	3	3039	3	9	9	BRASIL
7711077	F	27/06/198	39	3	3039	4	9	9	BRASIL
7257406	F	05/10/198	31	3	3031	2	2	3	BRASIL
7711980	F	27/02/198	35	3	3035	1	1	9	BRASIL
2752808	F	22/09/197	42	3	3042	1	9		BRASIL
2269988	F	15/09/199	25	3	3025	3	1	9	BRASIL
2078015	F	29/12/198	37	3	3037	3	4	9	BRASIL
24074	F	15/06/199	22	3	3022	1	4	3	BRASIL
3013162	F	28/05/199	30	3	3030	2	4	9	BRASIL
2802511	F	03/11/198	36	3	3036	3	1		BRASIL
2079542	F	16/03/198	39	3	3039	3	2	4	BRASIL
2079798	F	05/08/199	21	3	3021	3	9	9	BRASIL
2151871	F	16/09/199	25	3	3025	2	1	9	BRASIL
24074	F	12/09/199	28	3	3028	3	4	1	BRASIL
5426936	F	20/01/199	27	3	3027	3	1		BRASIL
2142406	F	03/12/198	39	3	3039	3	3		BRASIL
2078015	F	27/09/198	32	3	3032	2	9	9	BRASIL
2338424	F	04/02/199	26	3	3026	1	9		BRASIL
2306344	F	02/05/197	41	3	3041	3	1	3	BRASIL
2522411	F	08/09/199	26	3	3026	3	1		BRASIL
2001578	F		36	3	3036	3	4		BRASIL
2022621	F	23/03/198	36	3	3036	1	4		BRASIL
2237571	F	02/04/198	32	3	3032	3	1	2	BRASIL
2290073	F	09/04/198	33	3	3033	2	2		BRASIL
6984649	F	21/07/198	33	3	3033	2	4		BRASIL
2078015	F	27/05/198	31	3	3031	2	1	9	BRASIL
5860490	F	08/06/199	30	3	3030	2	1	1	BRASIL
2535556	F	11/12/198	35	3	3035	3	1	4	BRASIL
3956369	F	14/09/199	25	3	3025	3	9		BRASIL
2081377	F	23/06/198	32	3	3032	3	1	9	BRASIL
2361787	F	02/07/199	23	3	3023	2	4		BRASIL
2752700	F	02/03/198	32	3	3032	2	4	9	BRASIL
2168707	F	13/04/197	49	3	3049	2	4	2	BRASIL
2537788	F	11/06/199	28	3	3028	2	1	3	BRASIL
2082187	F	04/11/199	29	3	3029	2	1	2	BRASIL
2079542	F	11/02/198	35	3	3035	3	9	9	BRASIL
8036	F	12/02/199	22	3	3022	2	4	2	BRASIL
5101654	F	17/12/198	35	3	3035	2	1	9	BRASIL
10464	F	02/02/198	34	3	3034	3	4		BRASIL
2755130	F	04/01/198	32	3	3032	2	1	9	BRASIL
2079720	F	17/06/199	24	3	3024	3	1	9	BRASIL
9745041	F	10/08/198	39	3	3039	1	1	4	BRASIL
24074	F	16/09/199	26	3	3026	1	4	3	BRASIL
2027356	F	13/02/200	19	3	3019	3	1		BRASIL
2323397	F	05/09/198	36	3	3036	2	9		BRASIL

2361787	F	26/06/199	31	3	3031	3	1	4	BRASIL
2707527	F	17/09/198	34	3	3034	2	4	3	BRASIL
2237571	F	15/02/198	38	3	3038	3	1	2	BRASIL
2781859	F	21/01/198	33	3	3033	3	1	9	BRASIL
2420015	F	19/01/198	37	3	3037	2	1	3	BRASIL
2439360	F	13/08/197	42	3	3042	2	3	9	BRASIL
2741989	F	17/06/199	21	3	3021	2	1	3	BRASIL
2337754	F	09/04/199	29	3	3029	3	1		BRASIL
2655411	F	12/08/199	23	3	3023	3	2		BRASIL
2079542	F	09/07/199	27	3	3027	3	3		BRASIL
2786117	F	08/02/200	17	3	3017	3	4	9	BRASIL
15369	F	09/07/199	29	3	3029	2	1		BRASIL
3356868	F	28/03/198	32	3	3032	3	1		BRASIL
2079925	F	19/12/199	24	3	3024	3	1	3	BRASIL
6988938	F	27/01/198	37	3	3037	3	1	4	BRASIL
3151794	F	16/02/199	29	3	3029	2	4		BRASIL
9194258	F	20/05/197	41	3	3041	2	1	9	BRASIL
10464	F	28/03/199	25	3	3025	3	9		BRASIL
2752700	F	30/12/198	31	3	3031	3	4	3	BRASIL
2078767	F	22/12/199	30	3	3030	3	1	9	BRASIL
3696162	F	12/08/198	31	3	3031	3	9	9	BRASIL
2555646	F	23/08/198	34	3	3034	2	1	3	BRASIL
2078015	F	09/03/199	31	3	3031	3	9	9	BRASIL
7640307	F	05/02/198	41	3	3041	2	1	3	BRASIL
3702693	F	20/12/198	37	3	3037	2	4	9	BRASIL
2122650	F	08/07/198	38	3	3038	3	1	4	BRASIL
13633	F	16/04/199	29	3	3029	3	1	3	BRASIL
2339196	F	04/08/199	21	3	3021	3	4	3	BRASIL
2227665	F	25/03/199	26	3	3026	3	1		BRASIL
2269988	F	05/11/200	20	3	3020	1	4	9	BRASIL
6575560	F	20/08/199	29	3	3029	3	4	3	BRASIL
2081938	F	26/05/198	36	3	3036	3	2	3	BRASIL
9725	F	21/09/199	25	3	3025	3	9	9	BRASIL
3356868	F	19/06/198	36	3	3036	3	1		BRASIL
6995462	F	28/09/198	34	3	3034	3	4	2	BRASIL
2119528	F	05/10/198	32	3	3032	3	1	3	BRASIL
2090961	F	29/11/199	28	3	3028	3	1	3	BRASIL
2237601	F	11/12/198	37	3	3037	3	1	2	BRASIL
7711980	F	04/08/200	20	3	3020	3	4	9	BRASIL
24074	F	21/01/200	20	3	3020	2	1	3	BRASIL
10464	F	14/01/199	25	3	3025	2	1		BRASIL
2550792	F	21/06/198	37	3	3037	3	1		BRASIL
2776006	F	26/05/198	33	3	3033	2	4	3	BRASIL
2078872	F		26	3	3026	3	1		BRASIL
24074	F	13/02/198	39	3	3039	2	2	4	BRASIL
2752700	F	04/11/199	26	3	3026	3	3	2	BRASIL
2655411	F	29/07/200	20	3	3020	3	4	2	BRASIL

2244306	F	03/06/1992	28	3	3028	2	1	2	BRASIL
3012212	F	28/01/1992	26	3	3026	2	1	3	BRASIL
2786117	F	03/04/1991	29	3	3029	3	4	9	BRASIL
2161354	F	08/02/1982	39	3	3039	3	4		BRASIL
2218798	F	05/04/1992	28	3	3028	3	1		BRASIL
2781859	F	25/04/1992	28	3	3028	3	1	9	BRASIL
2159252	F	01/02/1988	33	3	3033	3	1		BRASIL
2079038	F	06/06/1988	32	3	3032	2	1		BRASIL
2079798	F	18/07/1985	35	3	3035	3	1	3	BRASIL
6043941	F	24/04/1987	34	3	3034	3	2	4	BRASIL
10464	F	06/05/1992	29	3	3029	3	4		BRASIL
2078015	F	22/08/1991	29	3	3029	2	1	9	BRASIL
2465833	F	30/11/1984	36	3	3036	2	1	2	BRASIL
2408570	F	17/08/1983	37	3	3037	3	2	3	BRASIL
6602533	F	23/02/1995	26	3	3026	3	4		BRASIL
2309254	F	17/09/1986	34	3	3034	3	4	3	BRASIL
2079798	F	07/12/1982	38	3	3038	2	1	3	BRASIL
2266733	F	19/09/2002	18	3	3018	2	4		BRASIL
2789590	F	26/11/1981	39	3	3039	1	1	9	BRASIL
2219654	F	24/12/1985	35	3	3035	2	9	9	BRASIL
434	F	08/04/1988	33	3	3033	3	9	9	BRASIL
2741989	F	12/11/1998	22	3	3022	2	1	3	BRASIL
13633	F	02/07/2000	20	3	3020	3	1	2	BRASIL
2082209	F	21/08/1992	28	3	3028	1	1	4	BRASIL
2752700	F	22/09/1979	41	3	3041	3	4	4	BRASIL
2751569	F	27/08/1990	30	3	3030	3	1		BRASIL
2025507	F	04/07/2002	18	3	3018	1	1	2	BRASIL
2082187	F	13/11/1980	40	3	3040	3	1		BRASIL
2384299	F	14/04/1990	31	3	3031	2	1	9	BRASIL
2384299	F	11/07/1988	32	3	3032	3	1	9	BRASIL
2080931	F	10/11/1981	39	3	3039	3	1	9	BRASIL
2269988	F	12/12/1978	42	3	3042	2	1	9	BRASIL
2495015	F	04/07/1990	30	3	3030	2	4	3	BRASIL
6053858	F	28/11/1984	36	3	3036	2	1		BRASIL
105317	F	23/06/1997	24	3	3024	2	2		BRASIL
4001303	F	08/04/1988	33	3	3033	2	4	2	BRASIL
2499363	F	18/03/1987	34	3	3034	3	4	9	BRASIL
2710935	F	15/07/1994	27	3	3027	3	9	3	BRASIL
2641461	F	13/10/1983	37	3	3037	2	1	4	BRASIL
8052	F	14/12/1990	30	3	3030	3	1	9	BRASIL
2022621	F	16/11/1982	38	3	3038	1	4		BRASIL
2655411	F	15/01/2001	20	3	3020	3	1		BRASIL
13846	F	01/04/1986	34	3	3034	3	1	4	BRASIL
2248298	F	05/08/1988	32	3	3032	2	1		BRASIL
2756951	F	09/04/1984	36	3	3036	1	4	9	BRASIL
2376334	F	27/05/1999	21	3	3021	2	4	3	BRASIL
434	F	03/09/1994	26	3	3026	3	9	9	BRASIL

2748223	F	07/05/198	32	3	3032	3	4	3	BRASIL
2785382	F	15/06/199	27	3	3027	2	1	1	BRASIL
9138153	F	31/07/198	34	3	3034	3	4	3	BRASIL
2269988	F	28/08/199	24	3	3024	3	4	9	BRASIL
2283794	F	21/02/198	32	3	3032	2	1		BRASIL
2309254	F	08/06/199	22	3	3022	2	4	4	BRASIL
2591049	F	24/10/199	30	3	3030	2	2	2	BRASIL
5585422	F	16/06/198	35	3	3035	3	4	3	BRASIL
2408570	F	24/01/198	33	3	3033	3	4	9	BRASIL
24074	F	04/01/200	21	3	3021	3	4	3	BRASIL
2082926	F	17/03/198	40	3	3040	3	2		BRASIL
9923837	F	05/07/198	35	3	3035	3	4	9	BRASIL
2454696	F	19/08/199	29	3	3029	3	1	4	BRASIL
2754843	F	29/10/198	32	3	3032	3	4	3	BRASIL
2153084	F	17/03/199	26	3	3026	3	1		BRASIL
6558143	F	17/03/198	41	3	3041	3	4	9	BRASIL
2078015	F	20/08/198	32	3	3032	3	4	9	BRASIL
9917322	F	10/11/198	37	3	3037	3	1	9	BRASIL
2078015	F	20/11/198	39	3	3039	3	1	9	BRASIL
2079275	F	14/12/198	37	3	3037	3	9	9	BRASIL
2111640	F	05/01/198	33	3	3033	3	1	3	BRASIL
2132877	F	21/05/197	42	3	3042	3	1	3	BRASIL
9709	F	20/08/198	36	3	3036	3	5	3	BRASIL
2499363	F	30/01/199	31	3	3031	3	4	9	BRASIL
2077396	F	05/04/198	38	3	3038	3	1	3	BRASIL
24074	F	25/06/199	30	3	3030	2	4	1	BRASIL
2499363	F	22/09/198	35	3	3035	3	4	9	BRASIL
2816210	F	13/05/199	27	3	3027	2	3	4	BRASIL
7482450	F	18/06/198	32	3	3032	3	1	9	BRASIL
2384299	F	27/05/199	29	3	3029	3	1	9	BRASIL
10464	F	08/10/198	35	3	3035	3	9		BRASIL
3037274	F	09/10/198	35	3	3035	2	9	9	BRASIL
2319659	F	06/10/199	27	3	3027	2	4	1	BRASIL
2748223	F	13/02/198	35	3	3035	2	4	2	BRASIL
2798484	F	21/08/198	35	3	3035	2	1		BRASIL
24074	F	20/02/198	32	3	3032	2	9	9	BRASIL
2452383	F	15/06/198	33	3	3033	2	4	2	BRASIL
2078015	F	19/05/198	38	3	3038	2	1		BRASIL
2237571	F	21/04/198	33	3	3033	2	2	4	BRASIL
2096463	F	04/08/197	42	3	3042	2	1	9	BRASIL
2319659	F	11/11/199	25	3	3025	2	5	2	BRASIL
2040069	F	13/12/199	28	3	3028	3	1		BRASIL
2550792	F	15/03/200	21	3	3021	3	1		BRASIL
2298317	F	24/06/199	31	3	3031	2	1	9	BRASIL
2594714	F	06/04/199	31	3	3031	3	1		BRASIL
2398680	F	18/04/199	23	3	3023	1	4	3	BRASIL
7849184	F	25/04/198	35	3	3035	3	4		BRASIL

2674971	F	20/09/199	29	3	3029	4	4		BRASIL
2078376	F	19/01/198	33	3	3033	3	1	3	BRASIL
2078015	F	13/03/199	27	3	3027	3	4	9	BRASIL
2078015	F	26/05/199	25	3	3025	1	1		BRASIL
2099454	F	26/12/199	30	3	3030	4	2	4	BRASIL
3157245	F	10/03/198	40	3	3040	3	1	9	BRASIL
2096463	F	16/01/198	32	3	3032	2	1	3	BRASIL
8036	F	19/07/198	40	3	3040	3	1		BRASIL
2248190	F	23/10/199	23	3	3023	3	1	9	BRASIL
2079542	F	29/01/199	25	3	3025	3	2	4	BRASIL
15245	F	02/01/198	35	3	3035	2	9	9	BRASIL
2269988	F	02/07/199	29	3	3029	3	1	3	BRASIL
2761041	F	21/07/199	28	3	3028	3	9	9	BRASIL
2726726	F	18/06/200	20	3	3020	3	4		BRASIL
2655411	F	07/07/198	34	3	3034	2	4		BRASIL
5717515	F	18/10/198	38	3	3038	2	9	9	BRASIL
6716849	F	09/09/199	23	3	3023	3	4		BRASIL
24074	F	15/09/198	34	3	3034	1	4	2	BRASIL
2082209	F	04/09/198	40	3	3040	3	4		BRASIL
13846	F	21/09/197	42	3	3042	2	1		BRASIL
2078015	F	08/06/198	38	3	3038	3	1	9	BRASIL
2022621	F	18/01/199	26	3	3026	2	4		BRASIL
2081695	F	06/12/198	32	3	3032	3	1	2	BRASIL
2688611	F	07/08/198	36	3	3036	2	1		BRASIL
2591049	F	23/11/197	43	3	3043	3	4	9	BRASIL
6558143	F	16/02/199	28	3	3028	2	4	9	BRASIL
3956369	F	24/10/199	24	3	3024	2	9	9	BRASIL
3972925	F	13/01/198	32	3	3032	3	4	2	BRASIL
2269988	F	23/05/198	36	3	3036	3	1	9	BRASIL
2275562	F	17/03/199	25	3	3025	2	4		BRASIL
2078015	F	15/04/198	31	3	3031	3	1	9	BRASIL
2079542	F	26/12/198	37	3	3037	2	9	9	BRASIL
2309254	F	20/12/198	31	3	3031	1	4	0	BRASIL
2756951	F	20/09/198	32	3	3032	3	4	9	BRASIL
2439263	F	04/09/200	19	3	3019	3	1	2	BRASIL
2097117	F	29/06/199	29	3	3029	3	2		BRASIL
2408570	F	11/10/198	34	3	3034	2	4	3	BRASIL
24074	F	04/02/199	22	3	3022	2	4	1	BRASIL
2738899	F	25/01/198	33	3	3033	2	1	3	BRASIL
2499363	F	06/07/199	26	3	3026	1	4		BRASIL
4001303	F	02/05/198	31	3	3031	3	4	2	BRASIL
2309254	F	15/10/199	29	3	3029	3	4	0	BRASIL
2708566	F	19/12/199	23	3	3023	3	1	4	BRASIL
2687127	F	04/04/198	35	3	3035	2	4	3	BRASIL
2688689	F	17/04/198	37	3	3037	2	1	9	BRASIL
2436450	F	04/05/198	37	3	3037	2	2		BRASIL
2784874	F	11/12/199	30	3	3030	2	1	4	BRASIL

7257406	F	12/06/199	29	3	3029	3	2	3	BRASIL
2760843	F	09/05/198	39	3	3039	3	1	9	BRASIL
2078015	F	12/03/198	38	3	3038	3	4	9	BRASIL
2040069	F	13/03/198	34	3	3034	3	4	3	BRASIL
2151758	F	26/07/197	42	3	3042	3	1	4	BRASIL
2802090	F	20/01/198	37	3	3037	1	1		BRASIL
2079542	F	09/09/199	23	3	3023	2	1	3	BRASIL
2080583	F	16/01/198	35	3	3035	3	9		BRASIL
2254611	F	18/04/198	32	3	3032	2	1	2	BRASIL
2338424	F	09/05/198	32	3	3032	3	9	9	BRASIL
24074	F	04/07/199	25	3	3025	2	2	4	BRASIL
2078015	F	26/12/198	39	3	3039	1	1	9	BRASIL
7349270	F	17/07/198	39	3	3039	2	9	9	BRASIL
2792168	F	04/02/198	36	3	3036	2	4	9	BRASIL
5073898	F	30/06/198	38	3	3038	2	4	4	BRASIL
2752700	F	05/08/199	30	3	3030	2	4	2	BRASIL
2078015	F	31/05/199	27	3	3027	3	4	9	BRASIL
9628	F	08/07/199	23	3	3023	1	9		BRASIL
2092298	F	24/07/198	33	3	3033	3	1	9	BRASIL
2303892	F	21/12/200	20	3	3020	2	1	3	BRASIL
2496879	F	13/04/198	33	3	3033	2	4	3	BRASIL
9696733	F	19/08/199	27	3	3027	3	4	4	BRASIL
9744665	F	22/09/199	24	3	3024	3	9	9	BRASIL
2159252	F	07/06/199	23	3	3023	3	1		BRASIL
24074	F	18/08/198	40	3	3040	3	1	4	BRASIL
6988938	F	22/01/198	34	3	3034	2	1	9	BRASIL
2222043	F	26/06/199	24	3	3024	3	4	3	BRASIL
3006522	F	22/10/198	31	3	3031	3	9		BRASIL
2738864	F	28/10/198	37	3	3037	3	1		BRASIL
2027356	F	08/12/199	27	3	3027	3	2	2	BRASIL
2269988	F	16/11/199	27	3	3027	3	9		BRASIL
2079798	F	29/01/199	22	3	3022	2	1	9	BRASIL
2223538	F	12/10/198	39	3	3039	3	1		BRASIL
2337754	F	05/05/198	33	3	3033	2	9	4	BRASIL
2309254	F	19/08/199	29	3	3029	3	4	4	BRASIL
2153882	F	03/01/198	32	3	3032	1	1		BRASIL
2691884	F	27/03/199	31	3	3031	2	1		BRASIL
2781859	F	01/02/199	28	3	3028	3	1	9	BRASIL
2733676	F	18/09/198	40	3	3040	3	1		BRASIL
24074	F	11/10/199	25	3	3025	2	4	3	BRASIL
3014673	F	08/08/198	34	3	3034	3	9	9	BRASIL
3028399	F	01/11/198	34	3	3034	2	1		BRASIL
2081091	F	10/08/199	23	3	3023	3	1	3	BRASIL
2384299	F	10/05/199	23	3	3023	3	1	9	BRASIL
2082926	F	25/04/198	35	3	3035	3	4	9	BRASIL
3506959	F	10/04/198	37	3	3037	2	1	3	BRASIL
2655411	F	08/05/199	28	3	3028	3	1		BRASIL

2295415	F	20/07/199	21	3	3021	2	2	9	BRASIL
2726653	F	09/09/198	37	3	3037	3	1	3	BRASIL
2384299	F	05/08/198	31	3	3031	3	1	9	BRASIL
2594714	F	19/05/199	25	3	3025	3	1		BRASIL
2237571	F	30/09/199	23	3	3023	2	2	2	BRASIL
6602533	F	13/07/198	35	3	3035	3	4		BRASIL
2436450	F	20/11/200	17	3	3017	3	1	3	BRASIL
6602533	F	03/04/199	31	3	3031	3	4	9	BRASIL
2319659	F	02/04/200	19	3	3019	2	5	3	BRASIL
3212130	F	09/02/198	33	3	3033	3	1	9	BRASIL
2752700	F	19/12/198	34	3	3034	3	4	1	BRASIL
2022621	F	23/07/199	28	3	3028	2	1	4	BRASIL
2499363	F	12/01/198	33	3	3033	3	4	9	BRASIL
2269988	F	04/06/199	22	3	3022	3	9	9	BRASIL
2082187	F	21/11/199	27	3	3027	3	1		BRASIL
2591049	F	07/01/199	29	3	3029	3	4		BRASIL
2452383	F	15/01/199	22	3	3022	2	4		BRASIL
2781859	F	15/10/199	28	3	3028	2	4	9	BRASIL
2407205	F	13/07/200	21	3	3021	2	9	9	BRASIL
6086381	F	07/07/200	20	3	3020	3	1	9	BRASIL
2688573	F	29/04/199	22	3	3022	2	1		BRASIL
2784874	F	17/05/199	26	3	3026	3	1	3	BRASIL
2078015	F	08/02/198	39	3	3039	3	1	9	BRASIL
3956369	F	09/10/198	34	3	3034	3	1		BRASIL
2081695	F	04/09/197	41	3	3041	3	1	9	BRASIL
2111640	F	07/11/198	40	3	3040	3	4	4	BRASIL
2089602	F	07/02/198	35	3	3035	2	4		BRASIL
15245	F	12/01/198	33	3	3033	3	9	9	BRASIL
2030314	F	30/07/198	32	3	3032	2	4		BRASIL
2708566	F	13/05/198	33	3	3033	2	1	3	BRASIL
2398680	F	27/09/200	18	3	3018	3	4		BRASIL
2085194	F	01/07/198	38	3	3038	3	1		BRASIL
2010631	F	27/02/199	30	3	3030	3	4	3	BRASIL
4056752	F	07/02/199	25	3	3025	3	4		BRASIL
2655411	F	27/09/200	18	3	3018	3	9		BRASIL
2615770	F	23/08/198	39	3	3039	3	1	3	BRASIL
3151794	F	29/07/198	32	3	3032	3	4	1	BRASIL
2298317	F	04/12/199	22	3	3022	3	4	9	BRASIL
2298317	F	09/09/199	23	3	3023	3	4	2	BRASIL
2081695	F	20/02/199	28	3	3028	1	1		BRASIL
10464	F	05/07/198	31	3	3031	2	9		BRASIL
3151794	F	12/05/198	40	3	3040	1	4		BRASIL
2688611	F	21/01/198	37	3	3037	3	1	4	BRASIL
2672197	F	25/11/199	30	3	3030	3	2		BRASIL
2232146	F	30/12/197	49	3	3049	2	1		BRASIL
2273659	F	03/06/197	46	3	3046	2	1	9	BRASIL
2688611	F	26/08/197	41	3	3041	3	1		BRASIL

2077396	F	27/10/198	32	3	3032	3	1	2	BRASIL
2295407	F	22/02/199	25	3	3025	3	2		BRASIL
6716849	F	12/06/199	30	3	3030	3	4	9	BRASIL
3808	F	06/06/199	26	3	3026	2	4	9	BRASIL
2499363	F	12/12/199	24	3	3024	3	4	9	BRASIL
2078376	F	16/08/199	26	3	3026	2	1	9	BRASIL
2439263	F	19/12/198	40	3	3040	2	1	3	BRASIL
2790580	F	02/10/199	26	3	3026	3	1	3	BRASIL
2280183	F	08/02/198	34	3	3034	3	9	9	BRASIL
2252694	F	22/04/199	23	3	3023	2	1	1	BRASIL
24074	F	13/11/200	17	3	3017	1	1	3	BRASIL
2237571	F	09/03/198	37	3	3037	3	4	3	BRASIL
2784874	F	13/08/199	28	3	3028	3	1	9	BRASIL
2040069	F	04/09/198	36	3	3036	3	4	3	BRASIL
2615797	F	17/01/198	38	3	3038	1	4	3	BRASIL
6236367	F	08/07/198	32	3	3032	2	4	2	BRASIL
2802082	F	01/05/197	42	3	3042	4	9	9	BRASIL
2594714	F	19/03/198	36	3	3036	3	1	3	BRASIL
2659069	F	22/03/199	29	3	3029	1	4		BRASIL
2384299	F	07/04/199	25	3	3025	3	1	9	BRASIL
2241153	F	20/06/199	29	3	3029	1	1	2	BRASIL
2772310	F	26/06/198	35	3	3035	3	1	9	BRASIL
2096463	F	16/01/199	27	3	3027	3	1	2	BRASIL
10464	F	22/07/199	27	3	3027	2	9	9	BRASIL
2384299	F	31/08/198	32	3	3032	3	1	9	BRASIL
3037274	F	16/04/197	47	3	3047	2	9	4	BRASIL
2266733	F	30/06/198	39	3	3039	3	4		BRASIL
2283239	F	13/01/198	41	3	3041	2	4	9	BRASIL
2237601	F	11/12/198	32	3	3032	2	2		BRASIL
3007847	F	27/03/198	35	3	3035	3	1		BRASIL
2738368	F	23/02/198	37	3	3037	2	1		BRASIL
2499363	F	04/04/198	35	3	3035	2	4		BRASIL
3157245	F	17/06/199	22	3	3022	3	1	9	BRASIL
2079542	F	15/04/199	27	3	3027	3	4	4	BRASIL
9297502	F	23/08/198	35	3	3035	3	4		BRASIL
2283	F	25/11/198	37	3	3037	1	1		BRASIL
2655411	F	08/04/199	24	3	3024	3	4		BRASIL
4056752	F	05/02/200	21	3	3021	1	1		BRASIL
2319659	F	27/06/199	30	3	3030	2	4		BRASIL
2816210	F	30/04/199	31	3	3031	2	4		BRASIL
3972925	F	01/06/198	40	3	3040	3	4	4	BRASIL
2594714	F	12/08/199	30	3	3030	3	1	3	BRASIL
2237571	F	03/03/197	42	3	3042	2	1	2	BRASIL
6848710	F	07/10/199	22	3	3022	3	9		BRASIL
9194258	F	02/06/199	31	3	3031	3	1		BRASIL
2081091	F	19/10/198	38	3	3038	4	1	3	BRASIL
2082128	F	01/09/199	28	3	3028	1	1		BRASIL

3394514	F	06/01/199	26	3	3026	3	9		BRASIL
9149511	F	31/07/198	40	3	3040	3	4		BRASIL
2781859	F	22/01/199	25	3	3025	3	1	9	BRASIL
2040069	F	16/09/198	37	3	3037	2	4	3	BRASIL
2077485	F	06/02/200	21	3	3021	3	1	9	BRASIL
2309254	F	08/12/198	34	3	3034	2	4	0	BRASIL
2752700	F	17/09/198	36	3	3036	3	3	2	BRASIL
3506959	F	20/04/198	35	3	3035	2	1	9	BRASIL
2000385	F	23/02/199	29	3	3029	2	4	9	BRASIL
2786117	F	16/10/198	32	3	3032	3	4	3	BRASIL
2269988	F	10/05/199	31	3	3031	3	1	9	BRASIL
2010151	F	28/03/199	22	3	3022	3	4		BRASIL
2499363	F	15/04/198	34	3	3034	3	4	9	BRASIL
2499363	F	19/06/198	31	3	3031	3	4		BRASIL
2023709	F	30/10/197	43	3	3043	2	1		BRASIL
2022621	F	09/07/198	39	3	3039	2	1		BRASIL
2273438	F	30/09/199	26	3	3026	2	9	9	BRASIL
2171945	F	27/02/198	38	3	3038	3	1	4	BRASIL
2078473	F	04/03/199	25	3	3025	3	2	9	BRASIL
3046281	F	19/10/198	31	3	3031	3	4		BRASIL
2337754	F	15/08/198	34	3	3034	2	1		BRASIL
2481073	F	13/04/198	38	3	3038	3	4		BRASIL
5718368	F	29/01/198	38	3	3038	3	4	9	BRASIL
2499363	F	27/09/198	31	3	3031	3	4	9	BRASIL
2499363	F	29/06/197	41	3	3041	3	4		BRASIL
2499363	F	31/12/199	30	3	3030	2	4	9	BRASIL
2786117	F	28/04/198	33	3	3033	3	4	3	BRASIL
2083604	F	10/01/199	31	3	3031	2	1	3	BRASIL
5073898	F	09/09/197	41	3	3041	3	4	3	BRASIL
2126494	F	21/12/199	25	3	3025	3	1		BRASIL
2786117	F	30/04/199	31	3	3031	3	4	3	BRASIL
2688611	F	06/06/198	34	3	3034	3	1	4	BRASIL
2587335	F	12/02/198	35	3	3035	2	4	3	BRASIL
228192	F	29/08/198	37	3	3037	2	1	3	BRASIL
2126133	F	14/03/199	22	3	3022	3	4	2	BRASIL
2115891	F	04/03/197	47	3	3047	4	9	9	BRASIL
2493888	F	18/01/198	33	3	3033	4	4	9	BRASIL
7244339	F	17/03/199	29	3	3029	3	9	9	BRASIL
9628	F	30/03/198	40	3	3040	2	9		BRASIL
2219662	F	05/05/198	32	3	3032	3	4	4	BRASIL
2363070	F	13/04/198	34	3	3034	3	4	9	BRASIL
2323397	F	28/04/198	37	3	3037	2	4		BRASIL
2296241	F	16/01/199	24	3	3024	4	1	1	BRASIL
9725	F	14/05/197	42	3	3042	2	9	9	BRASIL
2079542	F	17/10/198	31	3	3031	3	1	4	BRASIL
2789590	F	01/01/198	39	3	3039	1	4	3	BRASIL
9725	F	05/09/198	33	3	3033	2	4	9	BRASIL

3008207	F	30/09/199	29	3	3029	3	2		BRASIL
2078503	F	23/06/200	21	3	3021	3	1	3	BRASIL
6533000	F	15/02/200	18	3	3018	4	4	9	BRASIL
2269988	F	10/10/198	40	3	3040	3	4	9	BRASIL
24074	F	02/09/198	40	3	3040	1	3	3	BRASIL
2738899	F	20/03/199	31	3	3031	3	1	4	BRASIL
2655411	F	30/10/199	24	3	3024	2	4		BRASIL
3019608	F	04/06/198	35	3	3035	3	1	9	BRASIL
2022621	F	07/06/199	26	3	3026	3	1	3	BRASIL
2080516	F	12/02/198	38	3	3038	2	4	4	BRASIL
2604388	F	31/08/198	39	3	3039	2	1		BRASIL
2752700	F	08/06/197	43	3	3043	2	4	1	BRASIL
2236370	F	02/02/199	23	3	3023	3	1	4	BRASIL
2191164	F	01/11/199	29	3	3029	2	4		BRASIL
2786117	F	12/04/199	27	3	3027	2	4	3	BRASIL
2269988	F	18/10/198	32	3	3032	3	4	9	BRASIL
2082586	F	06/01/198	40	3	3040	3	4	4	BRASIL
2022621	F	24/10/198	35	3	3035	3	4		BRASIL
2079542	F	29/03/198	39	3	3039	3	1	4	BRASIL
2384299	F	25/06/198	37	3	3037	2	1	9	BRASIL
7061021	F	10/03/199	27	3	3027	3	9	9	BRASIL
2594714	F	26/03/198	35	3	3035	3	1	3	BRASIL
2311682	F	28/09/198	33	3	3033	3	4		BRASIL
2792974	F	25/09/198	36	3	3036	3	1	2	BRASIL
7257406	F	26/08/198	36	3	3036	3	1	4	BRASIL
2306344	F	27/07/199	25	3	3025	3	1	3	BRASIL
2655411	F	09/12/198	33	3	3033	3	9		BRASIL
2442108	F	04/03/199	22	3	3022	3	4	3	BRASIL
2781859	F	21/07/198	35	3	3035	3	1	9	BRASIL
2159252	F	16/05/199	27	3	3027	2	9	9	BRASIL
2308665	F	20/11/199	29	3	3029	3	4		BRASIL
2084163	F	11/02/198	36	3	3036	3	1	4	BRASIL
2209195	F	14/07/198	39	3	3039	3	4	9	BRASIL
27022	F	13/01/199	28	3	3028	3	1	9	BRASIL
9757090	F	26/04/198	34	3	3034	3	1	3	BRASIL
2587165	F	05/09/198	38	3	3038	3	1	3	BRASIL
2795655	F	21/04/199	26	3	3026	3	4	2	BRASIL
2754738	F	27/12/200	17	3	3017	2	1		BRASIL
6627595	F	05/01/198	34	3	3034	3	4	3	BRASIL
2019558	F	13/03/198	38	3	3038	3	4		BRASIL
2153084	F	21/10/199	28	3	3028	3	2		BRASIL
3019608	F	18/02/198	38	3	3038	3	4	9	BRASIL
2082187	F	14/11/198	37	3	3037	2	1		BRASIL
3956369	F	26/06/198	35	3	3035	3	4		BRASIL
2078376	F	21/11/198	32	3	3032	3	1	4	BRASIL
3956369	F	19/03/198	37	3	3037	3	9	9	BRASIL
3594432	F	08/08/199	30	3	3030	2	1	9	BRASIL

24074	F	17/12/199	23	3	3023	2	4	3	BRASIL
2688611	F	05/01/199	27	3	3027	3	1		BRASIL
2591049	F	04/05/198	37	3	3037	1	1	1	BRASIL
2108992	F	17/11/199	22	3	3022	3	4	2	BRASIL
2237571	F	14/06/200	16	3	3016	2	1	2	BRASIL
2361787	F	24/04/199	24	3	3024	3	4	9	BRASIL
9554157	F	10/12/198	32	3	3032	1	9	9	BRASIL
176664	F	14/08/199	29	3	3029	3	1	9	BRASIL
5717515	F	28/04/198	32	3	3032	2	4	9	BRASIL
2204762	F	19/03/198	38	3	3038	3	4	3	BRASIL
2280183	F	03/02/199	22	3	3022	2	2	9	BRASIL
2151871	F	12/04/199	31	3	3031	3	2	3	BRASIL
2079127	F	06/05/199	30	3	3030	3	1		BRASIL
2269988	F	10/03/200	19	3	3019	3	4	9	BRASIL
2452383	F	15/07/197	44	3	3044	3	4		BRASIL
2237571	F	05/11/198	39	3	3039	3	1	2	BRASIL
6424341	F	07/12/198	37	3	3037	3	1	4	BRASIL
2786117	F	21/05/198	32	3	3032	3	4	3	BRASIL
2781859	F	30/01/198	38	3	3038	3	1	9	BRASIL
2755130	F	03/12/199	24	3	3024	3	4	3	BRASIL
4001303	F	28/03/198	36	3	3036	3	4	3	BRASIL
2077485	F	23/02/198	34	3	3034	3	4	9	BRASIL
2688611	F	28/11/198	37	3	3037	3	1	4	BRASIL
2655411	F	04/05/198	33	3	3033	2	4		BRASIL
2022621	F	12/03/198	35	3	3035	3	1		BRASIL
2080931	F	22/04/199	21	3	3021	3	1	9	BRASIL
2023474	F	12/03/199	31	3	3031	1	4	2	BRASIL
2655411	F	02/09/199	25	3	3025	2	4		BRASIL
2237253	F	01/01/197	42	3	3042	3	1		BRASIL
2237601	F	25/08/198	39	3	3039	3	1		BRASIL
3956369	F	26/06/199	29	3	3029	3	4	9	BRASIL
2159376	F	14/07/198	31	3	3031	1	9	9	BRASIL
2269988	F	18/10/200	16	3	3016	3	4	9	BRASIL
5407591	F	25/02/198	39	3	3039	1	9		BRASIL
2077396	F	04/03/198	34	3	3034	2	1	4	BRASIL
2655411	F	17/12/200	18	3	3018	2	4		BRASIL
2142406	F	03/01/199	23	3	3023	3	9	9	BRASIL
6210937	F	05/04/199	30	3	3030	3	1		BRASIL
434	F	13/07/198	37	3	3037	4	2		BRASIL
2499363	F	08/09/199	28	3	3028	3	4		BRASIL
2079429	F	11/01/199	23	3	3023	2	1	3	BRASIL
2795671	F	30/10/198	35	3	3035	3	4	9	BRASIL
2738368	F	02/08/199	23	3	3023	3	1		BRASIL
2122650	F	02/03/199	27	3	3027	3	2	9	BRASIL
9297502	F	02/05/198	40	3	3040	3	4		BRASIL
2319659	F	01/09/198	40	3	3040	3	2	2	BRASIL
2442612	F	17/08/200	16	3	3016	3	4	2	BRASIL

7659415	F	29/07/198	37	3	3037	3	1	9	BRASIL
2786117	F	27/01/199	31	3	3031	4	4	4	BRASIL
2127989	F	16/07/198	35	3	3035	3	1	4	BRASIL
2529149	F	17/05/197	42	3	3042	4	4		BRASIL
5444462	F	07/07/198	40	3	3040	2	1	3	BRASIL
3956369	F	01/05/198	34	3	3034	3	9		BRASIL
2655411	F	18/06/197	41	3	3041	3	4		BRASIL
2030314	F	04/04/199	28	3	3028	3	1		BRASIL
1120	F	22/04/199	24	3	3024	1	9	9	BRASIL
10464	F	12/11/199	21	3	3021	3	9		BRASIL
24074	F	10/12/199	22	3	3022	3	4	3	BRASIL
2246988	F	30/12/199	30	3	3030	3	4	2	BRASIL
2655411	F	25/11/198	31	3	3031	2	4	3	BRASIL
3012212	F	14/10/199	22	3	3022	2	1		BRASIL
2707527	F	25/09/200	21	3	3021	3	4	2	BRASIL
2126494	F	19/07/199	23	3	3023	3	9		BRASIL
4001303	F	23/04/198	32	3	3032	3	4	3	BRASIL
5253381	F	25/03/198	37	3	3037	2	9	9	BRASIL
2594714	F	06/06/198	34	3	3034	3	2	3	BRASIL
2078376	F	03/05/198	33	3	3033	2	1	3	BRASIL
2752700	F	25/06/199	28	3	3028	3	4	3	BRASIL
2707969	F	05/03/199	25	3	3025	3	1	3	BRASIL
2153882	F	24/10/199	29	3	3029	3	1		BRASIL
27979	F	17/04/198	33	3	3033	3	1	9	BRASIL
2083981	F	21/06/199	30	3	3030	3	1	3	BRASIL
2655411	F	07/09/198	34	3	3034	3	4		BRASIL
2535238	F	20/01/199	24	3	3024	3	4	9	BRASIL
2082128	F	08/09/199	21	3	3021	3	4	9	BRASIL
2082888	F	24/01/199	29	3	3029	2	9	3	BRASIL
2408570	F	24/08/200	17	3	3017	3	9	2	BRASIL
7244339	F	28/06/198	36	3	3036	2	1	9	BRASIL
2323397	F	12/09/199	24	3	3024	2	4	9	BRASIL
2758164	F	17/03/199	25	3	3025	3	1	2	BRASIL
7047428	F	11/02/198	36	3	3036	4	9	9	BRASIL
2411393	F	08/02/198	37	3	3037	3	1	1	BRASIL
2237598	F	24/03/198	39	3	3039	1	9	3	BRASIL
2707527	F	24/11/198	31	3	3031	3	4	3	BRASIL
2078015	F	19/03/198	36	3	3036	3	4	9	BRASIL
2755165	F	15/07/198	34	3	3034	3	4		BRASIL
9065946	F	12/08/198	34	3	3034	2	9		BRASIL
2755130	F	08/07/199	26	3	3026	3	4	3	BRASIL
2361787	F	07/12/199	28	3	3028	2	1		BRASIL
2078015	F	26/01/199	31	3	3031	3	4	9	BRASIL
9725	F	22/03/199	28	3	3028	2	4	9	BRASIL
9725	F	31/10/199	25	3	3025	3	4	9	BRASIL
2126494	F		36	3	3036	3	9		BRASIL
2237601	F	07/02/199	30	3	3030	2	1	3	BRASIL

5860490	F	23/08/198	37	3	3037	2	1	3	BRASIL
7865791	F	11/05/198	34	3	3034	2	1	9	BRASIL
2311682	F	10/11/198	32	3	3032	4	4		BRASIL
12521	F	15/09/198	37	3	3037	3	9	9	BRASIL
2594714	F	20/05/200	19	3	3019	3	1	3	BRASIL
2436450	F	20/01/198	35	3	3035	3	1	9	BRASIL
2781859	F	30/08/198	36	3	3036	2	1	9	BRASIL
2655411	F	09/08/198	38	3	3038	3	4		BRASIL
2825589	F	17/06/199	28	3	3028	2	1	3	BRASIL
2078015	F	30/05/199	27	3	3027	3	1	9	BRASIL
3956369	F	10/12/198	35	3	3035	2	9	9	BRASIL
2237571	F	03/03/200	21	3	3021	3	1	2	BRASIL
2339196	F	12/03/198	37	3	3037	2	1	9	BRASIL
2146525	F	13/02/199	25	3	3025	3	4	3	BRASIL
2269988	F	29/05/199	30	3	3030	3	4	3	BRASIL
5498465	F	11/09/198	37	3	3037	2	4	2	BRASIL
2408570	F	01/09/198	35	3	3035	3	4	2	BRASIL
2319659	F	11/11/199	21	3	3021	3	4		BRASIL
2252287	F	26/03/198	35	3	3035	3	1	1	BRASIL
2436450	F	13/03/199	22	3	3022	3	1	9	BRASIL
6680305	F	10/06/198	35	3	3035	1	1	4	BRASIL
3151794	F	02/05/199	30	3	3030	2	4	1	BRASIL
2081539	F	27/12/198	34	3	3034	3	4		BRASIL
2306344	F	20/07/198	41	3	3041	2	1	3	BRASIL
24074	F	06/08/198	35	3	3035	4	1	3	BRASIL
13846	F	17/05/198	33	3	3033	2	1	3	BRASIL
2227959	F	14/11/199	27	3	3027	2	1	3	BRASIL
2079542	F	08/02/198	34	3	3034	2	9	9	BRASIL
3956369	F	22/01/199	22	3	3022	3	4		BRASIL
2726734	F	15/09/198	38	3	3038	3	4	9	BRASIL
2825589	F	22/03/200	21	3	3021	3	4	2	BRASIL
2090961	F	19/11/199	21	3	3021	3	1	3	BRASIL
2082187	F	14/12/199	21	3	3021	3	1		BRASIL
2096463	F	19/02/200	18	3	3018	3	4	3	BRASIL
2237571	F	10/06/198	34	3	3034	3	1	2	BRASIL
2499363	F	25/09/198	39	3	3039	2	4	9	BRASIL
17884	F	12/10/199	30	3	3030	3	1	4	BRASIL
2707527	F	01/01/199	25	3	3025	3	4	2	BRASIL
2550792	F	04/02/198	38	3	3038	3	1	4	BRASIL
6848710	F	15/09/198	38	3	3038	3	9	9	BRASIL
2082128	F	08/08/198	39	3	3039	3	4		BRASIL
2604388	F	15/08/198	34	3	3034	3	1		BRASIL
2738899	F	19/06/198	36	3	3036	3	1	4	BRASIL
2361787	F	31/05/199	30	3	3030	1	4		BRASIL
2558254	F	17/06/198	40	3	3040	2	1	3	BRASIL
2298317	F	10/01/199	22	3	3022	3	1	9	BRASIL
2591049	F	06/09/198	36	3	3036	3	1	9	BRASIL

2585057	F	08/01/198	36	3	3036	2	1	4	BRASIL
2708566	F	08/02/199	25	3	3025	3	9	9	BRASIL
2027356	F	20/09/199	27	3	3027	3	4	3	BRASIL
2082101	F	04/06/199	24	3	3024	1	1	3	BRASIL
7257406	F	27/03/199	28	3	3028	3	4	2	BRASIL
7530005	F	28/01/199	31	3	3031	3	4		BRASIL
2097117	F	26/05/199	23	3	3023	3	1		BRASIL
2078015	F	02/05/198	31	3	3031	3	4	9	BRASIL
2080516	F	21/12/198	31	3	3031	3	1	3	BRASIL
7761929	F	03/10/199	25	3	3025	2	1	3	BRASIL
7257406	F	17/10/199	25	3	3025	2	4	3	BRASIL
2080796	F	06/04/199	28	3	3028	3	2		BRASIL
2078015	F	02/07/200	20	3	3020	3	4	9	BRASIL
2081695	F	07/02/199	23	3	3023	2	1		BRASIL
10464	F	02/11/198	35	3	3035	3	1	9	BRASIL
2790580	F	11/06/199	28	3	3028	3	1	4	BRASIL
2688611	F	07/04/198	40	3	3040	3	1		BRASIL
2688611	F	11/11/198	36	3	3036	3	1		BRASIL
2589737	F	27/03/199	25	3	3025	1	4	1	BRASIL
2781859	F	22/09/199	26	3	3026	3	1	9	BRASIL
2273748	F	23/08/199	22	3	3022	2	9		BRASIL
2594714	F	14/01/199	27	3	3027	3	1	4	BRASIL
24074	F	12/10/199	29	3	3029	2	1	3	BRASIL
2654032	F	06/03/198	33	3	3033	3	1	3	BRASIL
2237571	F	01/04/199	27	3	3027	3	1	2	BRASIL
2261987	F	08/11/198	33	3	3033	3	1	4	BRASIL
2079542	F	24/06/198	40	3	3040	3	1	4	BRASIL
2710935	F	25/05/200	19	3	3019	2	1		BRASIL
2232	F	16/07/198	35	3	3035	3	4		BRASIL
24074	F	17/07/199	30	3	3030	3	4	3	BRASIL
2558254	F	19/01/199	30	3	3030	1	1	2	BRASIL
2096463	F	03/03/198	41	3	3041	3	1	4	BRASIL
2083973	F	15/08/199	30	3	3030	3	4		BRASIL
2078015	F	12/02/198	39	3	3039	2	1	3	BRASIL
6995462	F	22/03/199	29	3	3029	3	9		BRASIL
2237571	F	23/04/199	29	3	3029	3	1	2	BRASIL
2078015	F	20/06/198	31	3	3031	2	1	9	BRASIL
6086381	F	28/02/199	26	3	3026	3	5		BRASIL
6697054	F	22/03/198	39	3	3039	3	1	3	BRASIL
2495279	F	12/11/199	25	3	3025	2	4	9	BRASIL
2499363	F	25/08/199	30	3	3030	3	1	9	BRASIL
2040069	F	29/11/197	41	3	3041	2	4	3	BRASIL
2309254	F	23/03/199	23	3	3023	2	4	0	BRASIL
2078015	F	06/05/198	35	3	3035	2	1	9	BRASIL
2146398	F	21/03/199	24	3	3024	3	1	3	BRASIL
24074	F	27/01/199	29	3	3029	2	1	3	BRASIL
2655411	F	02/07/199	30	3	3030	3	4		BRASIL

2521431	F	15/03/199	27	3	3027	3	1	3	BRASIL
2439263	F	31/05/198	38	3	3038	3	1	4	BRASIL
2298317	F	28/10/198	31	3	3031	3	4	9	BRASIL
2246929	F	16/10/198	34	3	3034	3	4	3	BRASIL
3007847	F	22/09/199	24	3	3024	1	1		BRASIL
2691515	F	17/02/198	41	3	3041	2	1	2	BRASIL
2738368	F	25/10/199	26	3	3026	1	1	3	BRASIL
7711980	F	07/11/197	44	3	3044	3	4	9	BRASIL
2786117	F	20/03/199	26	3	3026	2	4	3	BRASIL
2658496	F	08/07/198	38	3	3038	2	1	3	BRASIL
13633	F	18/02/198	33	3	3033	2	1	3	BRASIL
24074	F	04/10/198	35	3	3035	2	1	3	BRASIL
2655411	F	24/11/199	22	3	3022	2	1		BRASIL
4001303	F	29/06/198	36	3	3036	3	4	3	BRASIL
3170527	F	27/12/199	29	3	3029	3	1	3	BRASIL
2688611	F	28/10/198	31	3	3031	2	1		BRASIL
2754843	F	08/08/198	35	3	3035	3	4	9	BRASIL
2079186	F	12/07/198	31	3	3031	3	1	9	BRASIL
4251	F	03/04/199	29	3	3029	2	4	2	BRASIL
26794	F	30/08/198	32	3	3032	2	2	4	BRASIL
9554157	F	31/08/199	31	3	3031	1	9		BRASIL
2296624	F	29/04/199	30	3	3030	2	2		BRASIL
2323397	F	10/10/198	32	3	3032	1	4		BRASIL
2688611	F	10/05/198	33	3	3033	3	1	4	BRASIL
6627595	F	13/11/199	26	3	3026	3	4		BRASIL
2079186	F	12/10/199	30	3	3030	3	4	3	BRASIL
2655411	F	25/03/198	38	3	3038	2	4	9	BRASIL
2082586	F	23/10/199	29	3	3029	3	4	3	BRASIL
2384299	F	17/06/199	22	3	3022	2	1	9	BRASIL
2079542	F	19/06/197	46	3	3046	3	9		BRASIL
2082225	F	06/02/198	34	3	3034	3	4	9	BRASIL
8036	F	15/11/198	34	3	3034	3	1	3	BRASIL
2688611	F	05/04/198	39	3	3039	3	1		BRASIL
2078015	F	29/11/200	16	3	3016	3	1	9	BRASIL
27863	F	17/06/198	34	3	3034	3	4	3	BRASIL
7549156	F	10/01/199	27	3	3027	2	4	9	BRASIL
2080583	F	18/09/199	23	3	3023	3	4	2	BRASIL
2078015	F	28/04/200	16	3	3016	2	2	9	BRASIL
3000869	F	03/02/199	30	3	3030	3	1		BRASIL
2705982	F	27/08/198	36	3	3036	2	1	4	BRASIL
2079186	F	05/03/198	41	3	3041	3	1		BRASIL
9725	F	26/06/198	34	3	3034	2	4	9	BRASIL
4251	F	22/07/198	37	3	3037	3	2	3	BRASIL
4001303	F	18/06/199	25	3	3025	2	4	9	BRASIL
2237571	F	20/08/198	33	3	3033	3	1	2	BRASIL
5332427	F	05/10/199	29	3	3029	3	9	4	BRASIL
2079542	F	07/11/198	39	3	3039	3	3	4	BRASIL

3012212	F	29/12/198	38	3	3038	3	4	3	BRASIL
8028	F	10/02/197	48	3	3048	1	4	4	BRASIL
3014673	F	09/02/197	47	3	3047	3	9	9	BRASIL
2655411	F	31/07/198	35	3	3035	3	4		BRASIL
2765942	F	22/11/199	30	3	3030	2	1	3	BRASIL
7659415	F	14/01/199	30	3	3030	3	9		BRASIL
2755130	F	25/11/199	26	3	3026	2	1	3	BRASIL
2080818	F	28/12/197	43	3	3043	2	1	4	BRASIL
2237601	F	09/05/199	24	3	3024	3	1		BRASIL
5428	F	08/07/197	44	3	3044	4	4	3	BRASIL
7257406	F	19/10/199	27	3	3027	3	1	3	BRASIL
2384299	F	23/06/199	31	3	3031	3	1	9	BRASIL
5407591	F	06/06/199	26	3	3026	3	9		BRASIL
2079038	F	13/11/198	31	3	3031	3	1		BRASIL
2079798	F	19/08/198	39	3	3039	2	1	9	BRASIL
3018520	F	06/09/198	34	3	3034	3	9		BRASIL
2439263	F	23/07/199	25	3	3025	3	1	3	BRASIL
2754738	F	08/06/198	34	3	3034	3	9	9	BRASIL
9725	F	16/04/199	25	3	3025	3	1	9	BRASIL
2311682	F	31/07/198	37	3	3037	3	4		BRASIL
2398680	F	20/06/199	26	3	3026	2	4	3	BRASIL
2078015	F	24/11/198	38	3	3038	3	1	9	BRASIL
2791722	F	14/08/198	33	3	3033	2	2	2	BRASIL
2079798	F	25/02/198	38	3	3038	3	4	9	BRASIL
24074	F	14/10/198	33	3	3033	2	4	1	BRASIL
2410281	F	05/10/198	33	3	3033	3	4	3	BRASIL
2025507	F	28/01/198	33	3	3033	3	1	4	BRASIL
9699848	F	05/10/200	19	3	3019	2	9	9	BRASIL
2273748	F	14/09/199	27	3	3027	3	1		BRASIL
4001303	F	17/08/198	33	3	3033	2	4	3	BRASIL
2655411	F	15/06/199	30	3	3030	3	4		BRASIL
17884	F	15/07/199	25	3	3025	3	1		BRASIL
2089602	F	08/11/198	35	3	3035	3	1		BRASIL
2319659	F	24/07/199	30	3	3030	3	4	4	BRASIL
2781859	F	20/04/198	41	3	3041	1	1	9	BRASIL
2781859	F	21/02/198	33	3	3033	3	1	9	BRASIL
2087502	F	27/02/198	38	3	3038	3	1	9	BRASIL
2244306	F	25/10/199	24	3	3024	3	1	2	BRASIL
9725	F	31/12/199	26	3	3026	3	4	9	BRASIL
2558254	F	18/03/198	37	3	3037	3	1	4	BRASIL
2058391	F	03/04/199	30	3	3030	2	1	9	BRASIL
2079542	F	03/04/198	36	3	3036	2	1	4	BRASIL
2499363	F	08/04/198	35	3	3035	3	4	9	BRASIL
7354746	F	29/01/199	28	3	3028	2	1		BRASIL
3580822	F	04/02/198	34	3	3034	3	9	9	BRASIL
2384299	F	19/02/198	39	3	3039	3	1	9	BRASIL
2338424	F	28/04/198	34	3	3034	2	4		BRASIL

2825309	F	02/04/1988	32	3	3032	3	1	3	BRASIL
2537788	F	16/01/1999	22	3	3022	3	1	9	BRASIL
6603378	F	08/10/1989	31	3	3031	3	4	9	BRASIL
2452383	F	01/10/1996	24	3	3024	3	1	4	BRASIL
2756978	F	02/11/1994	26	3	3026	2	9	9	BRASIL
3151794	F	12/09/1984	36	3	3036	3	4	3	BRASIL
2269988	F	09/05/1984	37	3	3037	2	4	9	BRASIL
2078015	F	21/08/1989	31	3	3031	3	1	2	BRASIL
2494523	F	19/04/1985	35	3	3035	2	4	3	BRASIL
2410281	F	18/12/1994	26	3	3026	1	4	3	BRASIL
2309254	F	13/01/1990	31	3	3031	2	4		BRASIL
2695634	F	21/05/197	49	3	3049	4	9	9	BRASIL
2338424	F	27/03/1984	36	3	3036	2	9	9	BRASIL
2269988	F	20/07/1989	32	3	3032	2	3	9	BRASIL
6602533	F	29/04/1998	23	3	3023	2	4		BRASIL
2786117	F	18/11/1987	33	3	3033	2	4	3	BRASIL
2790580	F	20/01/1983	38	3	3038	3	1	3	BRASIL
24074	F	27/09/1998	22	3	3022	3	4	3	BRASIL
2089602	F	26/10/1979	41	3	3041	3	1		BRASIL
24074	F	30/12/1992	27	3	3027	3	4	3	BRASIL
3242587	F	22/05/1984	36	3	3036	3	9	9	BRASIL
2078015	F	31/03/1988	33	3	3033	3	4	9	BRASIL
2738368	F	05/12/2000	20	3	3020	1	4	3	BRASIL
4251	F	08/06/1986	34	3	3034	3	2	4	BRASIL
2040069	F	09/01/1992	29	3	3029	2	4	3	BRASIL
2082128	F	15/02/1996	25	3	3025	4	1		BRASIL
4053907	F	22/09/198	39	3	3039	3	1	4	BRASIL
2591049	F	30/05/2002	16	3	3016	3	4	9	BRASIL
2710935	F	05/04/198	40	3	3040	4	4	3	BRASIL
2237571	F	27/09/1987	33	3	3033	2	1	4	BRASIL
2077485	F	02/01/199	30	3	3030	4	1	3	BRASIL
7257406	F	19/05/1997	24	3	3024	3	4	2	BRASIL
2079798	F	15/03/1982	38	3	3038	3	4	3	BRASIL
3037274	F	14/10/1984	36	3	3036	3	9	9	BRASIL
2078015	F	04/10/1984	36	3	3036	2	1	9	BRASIL
2415631	F	23/08/1985	35	3	3035	3	4		BRASIL
2452383	F	28/01/1984	37	3	3037	3	4	1	BRASIL
24074	F	01/04/1999	22	3	3022	3	4	3	BRASIL
13846	F	28/10/1986	34	3	3034	4	1	9	BRASIL
2022621	F	15/12/1997	23	3	3023	3	4		BRASIL
2442620	F	22/04/198	40	3	3040	3	4	3	BRASIL
2022621	F	25/08/1990	30	3	3030	3	2	3	BRASIL
2591049	F	29/08/1987	33	3	3033	2	4		BRASIL
2078015	F	16/01/1996	25	3	3025	3	1	9	BRASIL
2755130	F	23/11/1995	25	3	3025	3	1	9	BRASIL
13633	F	06/07/1988	32	3	3032	3	1	9	BRASIL
7421710	F	16/08/1998	22	3	3022	3	2	4	BRASIL

434	F	04/12/199	29	3	3029	3	1	9	BRASIL
2237571	F	14/10/199	29	3	3029	2	1	2	BRASIL
2266733	F	13/09/199	26	3	3026	3	4		BRASIL
4001303	F	13/04/198	33	3	3033	2	4	3	BRASIL
5407591	F	03/01/198	32	3	3032	3	9		BRASIL
2077396	F	19/11/198	34	3	3034	2	1	4	BRASIL
2078015	F	03/04/198	32	3	3032	3	1	9	BRASIL
2384299	F	11/03/199	30	3	3030	3	1	9	BRASIL
2077396	F	25/11/198	35	3	3035	3	1	3	BRASIL
2798298	F	10/05/198	32	3	3032	3	1	3	BRASIL
3037274	F	26/02/198	35	3	3035	3	9	9	BRASIL
6848710	F	30/05/198	38	3	3038	3	4		BRASIL
2205939	F	30/08/199	25	3	3025	2	4	3	BRASIL
7244339	F	30/05/199	28	3	3028	3	9	9	BRASIL
2082187	F	22/10/198	35	3	3035	3	1		BRASIL
2205939	F	10/02/198	36	3	3036	3	4	9	BRASIL
5907594	F	27/07/198	33	3	3033	3	1	9	BRASIL
24074	F	12/06/200	15	3	3015	2	2	2	BRASIL
2079895	F	11/01/200	20	3	3020	3	1	4	BRASIL
2408570	F	09/09/198	38	3	3038	2	1	3	BRASIL
2752700	F	01/07/198	38	3	3038	2	4	3	BRASIL
3019608	F	21/02/198	37	3	3037	3	1	9	BRASIL
2499363	F	23/06/198	32	3	3032	3	4	9	BRASIL
2337800	F	23/02/198	32	3	3032	3	9		BRASIL
5833418	F	03/12/199	27	3	3027	2	1	3	BRASIL
2089327	F	16/11/199	29	3	3029	3	1	9	BRASIL
3956369	F	11/09/199	26	3	3026	3	4		BRASIL
2710935	F	04/11/198	40	3	3040	3	4	4	BRASIL
2079542	F	16/02/198	32	3	3032	3	1	4	BRASIL
2078015	F	11/08/199	21	3	3021	2	1	9	BRASIL
2688611	F	02/09/198	33	3	3033	3	1		BRASIL
2499363	F	25/06/199	30	3	3030	2	4		BRASIL
7119097	F	18/08/198	41	3	3041	3	4	9	BRASIL
2499363	F	28/07/200	20	3	3020	3	4	9	BRASIL
3151794	F	13/03/199	26	3	3026	2	4		BRASIL
5883229	F	13/04/198	35	3	3035	2	1	4	BRASIL
2022621	F	06/12/198	32	3	3032	3	1	3	BRASIL
8036	F	24/04/198	36	3	3036	2	1	4	BRASIL
24074	F	30/03/198	34	3	3034	2	1	4	BRASIL
10464	F	04/12/199	28	3	3028	2	9		BRASIL
3005402	F	15/04/198	32	3	3032	3	4		BRASIL
6988938	F	15/12/198	34	3	3034	2	1	4	BRASIL
2655411	F	10/09/198	39	3	3039	2	4		BRASIL
2010631	F	25/10/198	32	3	3032	3	1	3	BRASIL
2708566	F	24/02/198	37	3	3037	2	1	9	BRASIL
24074	F	16/09/200	21	3	3021	2	4	3	BRASIL
3956369	F	14/04/198	36	3	3036	1	3	9	BRASIL

9725	F	30/06/199	21	3	3021	3	1	9	BRASIL
2655411	F	17/06/199	29	3	3029	3	1		BRASIL
2687127	F	17/04/199	28	3	3028	3	4	3	BRASIL
2452383	F	08/11/198	36	3	3036	3	4	3	BRASIL
2655411	F	07/02/199	23	3	3023	3	9		BRASIL
6086381	F	15/02/198	31	3	3031	2	1	9	BRASIL
6542638	F	22/06/199	29	3	3029	2	9	2	BRASIL
24074	F	05/07/199	28	3	3028	3	4	3	BRASIL
3037274	F	10/08/197	41	3	3041	3	4	1	BRASIL
2149990	F	30/05/198	38	3	3038	3	9		BRASIL
13846	F	09/03/199	31	3	3031	2	1	1	BRASIL
2309254	F	06/08/198	39	3	3039	3	4	3	BRASIL
7257406	F	30/12/198	31	3	3031	3	1	2	BRASIL
2079542	F	01/05/198	34	3	3034	3	9	4	BRASIL
2748223	F	28/06/199	27	3	3027	2	1	1	BRASIL
5585422	F	19/11/199	30	3	3030	2	4	3	BRASIL
2082187	F	13/04/199	22	3	3022	3	1		BRASIL
2655411	F	01/09/199	25	3	3025	3	4		BRASIL
2237571	F	20/11/199	26	3	3026	3	1	3	BRASIL
2499363	F	06/07/198	32	3	3032	3	4	9	BRASIL
434	F	28/07/198	35	3	3035	2	9		BRASIL
9725	F	10/12/198	33	3	3033	3	4	9	BRASIL
2499363	F	26/06/197	45	3	3045	2	4	9	BRASIL
4042085	F	21/03/200	21	3	3021	2	2		BRASIL
2323397	F	22/05/198	36	3	3036	3	4	3	BRASIL
13633	F	26/03/200	19	3	3019	3	1	3	BRASIL
2298317	F	13/02/198	36	3	3036	3	1	9	BRASIL
2244306	F	05/11/198	40	3	3040	2	1	4	BRASIL
2786117	F	11/04/199	23	3	3023	2	4	3	BRASIL
2080052	F	15/06/199	22	3	3022	3	1	1	BRASIL
2537788	F	28/06/198	33	3	3033	3	1	1	BRASIL
2655411	F	11/02/199	28	3	3028	3	4		BRASIL
7564546	F	12/01/198	32	3	3032	1	4	3	BRASIL
2269988	F	06/09/199	24	3	3024	3	1	9	BRASIL
2705982	F	15/10/199	26	3	3026	2	9	9	BRASIL
2708566	F	29/11/198	35	3	3035	3	1	9	BRASIL
2499363	F	20/01/198	34	3	3034	3	4	9	BRASIL
2792168	F	22/07/198	36	3	3036	3	9		BRASIL
2040069	F	16/02/198	36	3	3036	3	1	2	BRASIL
2688611	F	01/10/198	36	3	3036	3	4		BRASIL
2591049	F	25/03/199	28	3	3028	2	1	9	BRASIL
2298317	F	07/01/199	25	3	3025	2	4		BRASIL
2339196	F	07/04/198	36	3	3036	3	1	1	BRASIL
7421710	F	29/08/199	25	3	3025	2	9		BRASIL
434	F	01/10/199	29	3	3029	2	9	9	BRASIL
2481073	F	22/09/199	27	3	3027	3	4	2	BRASIL
2309254	F	23/04/199	23	3	3023	2	2		BRASIL

2338424	F	19/07/199[illegible]	30	3	3030	2	4		BRASIL
1120	F	25/06/198[illegible]	33	3	3033	3	9	9	BRASIL
3394514	F	09/05/198[illegible]	36	3	3036	2	4		BRASIL
24074	F	22/12/199[illegible]	25	3	3025	2	4	3	BRASIL
2298317	F	22/09/199[illegible]	23	3	3023	3	1	9	BRASIL
3956369	F	21/03/200[illegible]	21	3	3021	3	4		BRASIL
2481286	F	28/07/199[illegible]	31	3	3031	2	4	3	BRASIL
3971465	F	14/01/198[illegible]	32	3	3032	2	9	9	BRASIL
2082926	F	07/08/199[illegible]	25	3	3025	2	1	9	BRASIL
2436450	F	11/08/198	40	3	3040	2	1	2	BRASIL
6086381	F	09/06/199	29	3	3029	4	1	9	BRASIL
2079542	F	06/12/199	29	3	3029	2	9	9	BRASIL
2499363	F	06/01/198[illegible]	34	3	3034	3	4		BRASIL
2077396	F	09/10/198[illegible]	34	3	3034	2	1	3	BRASIL
5384117	F	27/02/198[illegible]	39	3	3039	2	1	4	BRASIL
7257406	F	01/08/198[illegible]	35	3	3035	3	4	3	BRASIL
2070413	F	29/04/199[illegible]	29	3	3029	2	1	3	BRASIL
7257406	F	14/12/199[illegible]	27	3	3027	3	4	9	BRASIL
2550792	F	30/04/198[illegible]	36	3	3036	2	1		BRASIL
10464	F	08/01/198[illegible]	32	3	3032	3	4		BRASIL
2096463	F	16/06/198[illegible]	37	3	3037	2	1	9	BRASIL
2756951	F	20/07/199[illegible]	25	3	3025	2	1	9	BRASIL
2140217	F	25/04/200[illegible]	20	3	3020	3	1	3	BRASIL
2823209	F	29/04/198	39	3	3039	2	1	3	BRASIL
2171945	F	13/03/198[illegible]	33	3	3033	3	1	4	BRASIL
2655411	F	15/12/199[illegible]	26	3	3026	3	4		BRASIL
2784874	F	27/09/198[illegible]	31	3	3031	2	1	9	BRASIL
2078015	F	26/10/199[illegible]	26	3	3026	2	1	9	BRASIL
2654024	F	04/03/198[illegible]	32	3	3032	3	9	9	BRASIL
2655411	F	03/07/198[illegible]	37	3	3037	2	9		BRASIL
27014	F	10/04/199[illegible]	28	3	3028	2	1	3	BRASIL
2078503	F	11/07/198[illegible]	32	3	3032	3	4	2	BRASIL
2752700	F	29/10/198[illegible]	34	3	3034	3	4	4	BRASIL
2499363	F	05/11/199[illegible]	30	3	3030	3	4	9	BRASIL
2655411	F	22/01/198[illegible]	32	3	3032	3	4		BRASIL
2079186	F	16/09/198[illegible]	35	3	3035	3	1	3	BRASIL
7244339	F	08/01/198[illegible]	36	3	3036	3	9	9	BRASIL
2017245	F	22/06/199[illegible]	22	3	3022	3	3		BRASIL
2752700	F	02/02/198[illegible]	33	3	3033	3	4	1	BRASIL
24074	F	25/06/198[illegible]	39	3	3039	2	4	3	BRASIL
2089602	F	12/05/198[illegible]	41	3	3041	2	1		BRASIL
2089602	F	26/07/198[illegible]	32	3	3032	2	1		BRASIL
2040069	F	24/10/198[illegible]	35	3	3035	2	1	2	BRASIL
2077396	F	30/03/199	29	3	3029	3	1	3	BRASIL
2081377	F	06/07/198[illegible]	34	3	3034	3	1	9	BRASIL
7421710	F	29/08/197[illegible]	41	3	3041	3	9		BRASIL
2755130	F	29/11/199[illegible]	26	3	3026	2	1	9	BRASIL

2206528	F	17/11/197	42	3	3042	2	4	3	BRASIL
2707527	F	06/06/199	24	3	3024	3	4	1	BRASIL
2781859	F	03/11/200	20	3	3020	2	1	9	BRASIL
3006204	F	26/12/198	34	3	3034	2	1	9	BRASIL
7421710	F	11/09/198	34	3	3034	2	9		BRASIL
2754843	F	18/04/198	32	3	3032	3	4	9	BRASIL
6414702	F	17/09/197	46	3	3046	2	4		BRASIL
2232022	F	01/11/199	30	3	3030	3	1	9	BRASIL
3285391	F	10/10/198	38	3	3038	2	4	9	BRASIL
5336171	F	23/08/198	38	3	3038	3	4		BRASIL
2688611	F	17/09/197	42	3	3042	3	1	4	BRASIL
2237601	F	22/10/199	21	3	3021	3	1		BRASIL
2079186	F	17/01/198	38	3	3038	2	4	9	BRASIL
5718368	F	08/02/198	36	3	3036	2	2	9	BRASIL
2078015	F	02/07/199	25	3	3025	3	4	9	BRASIL
2089327	F	31/05/198	38	3	3038	2	1	4	BRASIL
2309254	F	26/06/200	18	3	3018	3	2		BRASIL
6668143	F	19/03/198	36	3	3036	2	9		BRASIL
2399989	F	29/06/198	34	3	3034	2	9		BRASIL
3956369	F	26/12/198	40	3	3040	3	9		BRASIL
4001303	F	13/05/199	28	3	3028	3	4	2	BRASIL
2078015	F	08/04/198	31	3	3031	1	2	3	BRASIL
15245	F	27/04/199	24	3	3024	3	1	9	BRASIL
2065665	F	20/08/197	41	3	3041	2	4	9	BRASIL
2311682	F	22/07/200	17	3	3017	3	4	1	BRASIL
2219654	F	10/01/198	37	3	3037	2	4	9	BRASIL
2408570	F	10/07/198	39	3	3039	2	4	2	BRASIL
9297502	F	04/02/199	28	3	3028	2	4		BRASIL
2111640	F	16/04/197	41	3	3041	2	4	4	BRASIL
2795671	F	27/09/199	29	3	3029	2	4	9	BRASIL
24074	F	21/06/199	21	3	3021	2	4	3	BRASIL
2804034	F	26/08/199	29	3	3029	2	4		BRASIL
2761068	F	13/09/197	49	3	3049	2	1	9	BRASIL
2089602	F	02/06/198	33	3	3033	3	1		BRASIL
9725	F	18/02/200	19	3	3019	3	5	2	BRASIL
2269988	F	29/12/199	23	3	3023	3	9	9	BRASIL
2738368	F	05/02/199	24	3	3024	3	1		BRASIL
2738368	F	11/02/199	26	3	3026	3	4		BRASIL
2078015	F	06/03/199	24	3	3024	3	1	9	BRASIL
2085194	F	17/11/199	24	3	3024	2	1		BRASIL
2707527	F	09/04/198	37	3	3037	1	4	4	BRASIL
2078015	F	10/01/198	36	3	3036	3	1	9	BRASIL
2079887	F	04/04/198	36	3	3036	2	4		BRASIL
5515483	F	10/05/198	36	3	3036	3	1	9	BRASIL
2688611	F	21/10/200	17	3	3017	1	2		BRASIL
2058391	F	01/10/198	39	3	3039	3	9	9	BRASIL
2078562	F	01/12/199	23	3	3023	3	4		BRASIL

2738368	F	15/10/199[illegible]	27	3	3027	1	1	3	BRASIL
24074	F	21/11/198[illegible]	34	3	3034	2	3	9	BRASIL
2019558	F	21/04/198[illegible]	36	3	3036	2	4		BRASIL
2594714	F	15/09/199[illegible]	30	3	3030	3	1	4	BRASIL
2707527	F	27/10/198[illegible]	40	3	3040	2	4	3	BRASIL
2127881	F	16/06/199[illegible]	26	3	3026	2	1	2	BRASIL
5158044	F	17/09/199[illegible]	24	3	3024	2	9	3	BRASIL
3394514	F	16/11/198[illegible]	38	3	3038	3	9		BRASIL
2153882	F	02/06/198[illegible]	31	3	3031	3	1	9	BRASIL
2092611	F	11/03/200[illegible]	21	3	3021	3	2	9	BRASIL
2078562	F	09/08/198[illegible]	38	3	3038	3	1		BRASIL
2691485	F	13/03/199[illegible]	30	3	3030	1	4	3	BRASIL
7564902	F	27/11/198[illegible]	33	3	3033	3	2	3	BRASIL
3580822	F	20/09/198[illegible]	35	3	3035	3	1	4	BRASIL
7354746	F	01/05/199[illegible]	30	3	3030	3	4	9	BRASIL
2283	F	29/07/198[illegible]	40	3	3040	4	4		BRASIL
2269988	F	05/08/199[illegible]	26	3	3026	2	4	9	BRASIL
6602533	F	09/11/199[illegible]	27	3	3027	2	4		BRASIL
2022621	F	08/08/200[illegible]	18	3	3018	3	1		BRASIL
6086381	F	20/12/198[illegible]	40	3	3040	3	4	4	BRASIL
2754738	F	20/04/199[illegible]	28	3	3028	3	9	9	BRASIL
2309254	F	15/11/200[illegible]	19	3	3019	2	4	0	BRASIL
2550792	F	09/07/199[illegible]	28	3	3028	3	1		BRASIL
2688611	F	19/03/198[illegible]	38	3	3038	2	1		BRASIL
13633	F	08/12/198[illegible]	38	3	3038	2	1	3	BRASIL
5073898	F	04/07/198[illegible]	34	3	3034	3	4	3	BRASIL
2311682	F	11/11/199[illegible]	25	3	3025	2	4	9	BRASIL
2097613	F	27/08/198[illegible]	36	3	3036	2	1	4	BRASIL
2078503	F	08/03/199[illegible]	23	3	3023	2	4	3	BRASIL
2707527	F	23/04/197[illegible]	44	3	3044	3	4	2	BRASIL
2200473	F	03/06/200[illegible]	17	3	3017	2	4	9	BRASIL
2436450	F	10/05/198[illegible]	35	3	3035	3	1		BRASIL
6998704	F	02/09/198[illegible]	33	3	3033	2	1		BRASIL
2078015	F	01/11/199[illegible]	26	3	3026	3	1	9	BRASIL
2306344	F	21/05/199[illegible]	30	3	3030	2	1	2	BRASIL
2688611	F	31/05/199[illegible]	22	3	3022	3	1	4	BRASIL
5332419	F	30/05/198[illegible]	32	3	3032	3	4	4	BRASIL
2288338	F	16/09/199[illegible]	30	3	3030	4	4	9	BRASIL
4001303	F	13/09/199[illegible]	28	3	3028	2	4	3	BRASIL
2126494	F	13/01/199[illegible]	24	3	3024	3	4	9	BRASIL
2384299	F	11/01/198[illegible]	39	3	3039	2	1	9	BRASIL
2079240	F	11/07/199[illegible]	30	3	3030	3	1	3	BRASIL
24074	F	03/12/199[illegible]	26	3	3026	2	4	1	BRASIL
5860490	F	25/07/197[illegible]	47	3	3047	3	1	3	BRASIL
7299532	F	31/05/199[illegible]	24	3	3024	2	1	2	BRASIL
2077485	F	21/03/199[illegible]	30	3	3030	3	4	1	BRASIL
2338424	F	19/09/199[illegible]	28	3	3028	3	9	9	BRASIL

2365146	F	01/06/200	19	3	3019	3	4	9	BRASIL
2518384	F	14/04/197	46	3	3046	2	4	9	BRASIL
2278855	F	29/06/200	20	3	3020	3	4	9	BRASIL
2079542	F	13/06/198	35	3	3035	3	1	4	BRASIL
2025507	F	15/05/198	34	3	3034	1	4	2	BRASIL
2089602	F	10/02/199	25	3	3025	3	1		BRASIL
2081628	F	24/10/198	38	3	3038	2	9		BRASIL
2707527	F	16/10/198	31	3	3031	3	4	3	BRASIL
2415631	F	22/07/198	36	3	3036	2	9		BRASIL
2499363	F	24/09/199	22	3	3022	3	4		BRASIL
2078015	F	20/10/198	34	3	3034	2	4	9	BRASIL
2269988	F	03/07/199	29	3	3029	3	4	9	BRASIL
2081253	F	04/04/197	42	3	3042	3	1	4	BRASIL
2237601	F	23/01/199	23	3	3023	3	1		BRASIL
2232057	F	05/06/198	32	3	3032	3	1		BRASIL
2452383	F	23/04/199	30	3	3030	3	4	3	BRASIL
2078015	F	19/02/198	37	3	3037	3	4	9	BRASIL
6595197	F	13/12/198	37	3	3037	2	9	9	BRASIL
3005402	F	15/07/199	28	3	3028	3	4		BRASIL
2078015	F	22/03/198	33	3	3033	3	1	9	BRASIL
2079542	F	10/05/198	38	3	3038	3	9		BRASIL
2319659	F	27/04/199	23	3	3023	3	4	2	BRASIL
2389746	F	13/05/198	40	3	3040	2	1	9	BRASIL
2079542	F	14/06/199	28	3	3028	3	9	9	BRASIL
2227762	F	06/09/197	47	3	3047	4	1	2	BRASIL
2135132	F	05/01/200	21	3	3021	4	4	9	BRASIL
2323397	F	19/01/198	38	3	3038	2	4		BRASIL
2079542	F	17/09/198	38	3	3038	1	1	4	BRASIL
434	F	06/09/199	25	3	3025	4	1	9	BRASIL
2755130	F	03/07/198	37	3	3037	2	1	9	BRASIL
2159252	F	10/11/199	23	3	3023	3	4	9	BRASIL
2309254	F	09/09/198	32	3	3032	2	4	3	BRASIL
2079976	F	13/09/198	40	3	3040	3	1	9	BRASIL
2772310	F	05/03/199	25	3	3025	3	4	9	BRASIL
6627595	F	30/08/198	32	3	3032	2	4	4	BRASIL
7975023	F	24/07/199	29	3	3029	2	4		BRASIL
2688611	F	21/03/198	41	3	3041	3	4	4	BRASIL
2755130	F	05/09/199	25	3	3025	3	4	9	BRASIL
2408570	F	11/05/199	28	3	3028	2	4	2	BRASIL
2298317	F	15/07/198	39	3	3039	2	1	9	BRASIL
27863	F	20/08/198	38	3	3038	2	4	9	BRASIL
2738368	F	19/05/199	25	3	3025	3	1	9	BRASIL
2115891	F	23/12/198	34	3	3034	3	9	9	BRASIL
2200473	F	30/07/199	29	3	3029	4	9		BRASIL
4056752	F	02/02/198	34	3	3034	3	1	9	BRASIL
2078228	F	17/07/199	22	3	3022	3	4	9	BRASIL
2371367	F	24/07/198	33	3	3033	3	4	3	BRASIL

2270714	F	04/11/199	29	3	3029	3	4	9	BRASIL
7433867	F	15/09/198	31	3	3031	3	2	3	BRASIL
2081695	F	24/03/198	33	3	3033	3	1	9	BRASIL
2499363	F	18/03/198	37	3	3037	2	4	9	BRASIL
5907594	F	08/09/198	37	3	3037	3	1	9	BRASIL
2079542	F	01/03/198	32	3	3032	3	4	4	BRASIL
24074	F	29/01/198	34	3	3034	2	1	4	BRASIL
2078015	F	22/10/199	26	3	3026	3	4	9	BRASIL
2078015	F	25/06/199	29	3	3029	3	1	9	BRASIL
2781859	F	25/05/198	37	3	3037	3	9	3	BRASIL
2738368	F	22/11/199	29	3	3029	3	1	4	BRASIL
2384299	F	20/05/199	22	3	3022	3	1	9	BRASIL
2078503	F	01/10/198	33	3	3033	3	1	3	BRASIL
2655411	F	08/03/198	37	3	3037	3	4	2	BRASIL
24074	F	24/06/198	36	3	3036	2	4	2	BRASIL
5718368	F	27/02/198	40	3	3040	2	9	9	BRASIL
434	F	03/09/198	33	3	3033	3	9		BRASIL
3074889	F	31/12/199	30	3	3030	2	1	3	BRASIL
2292912	F	15/03/198	32	3	3032	3	4		BRASIL
2078015	F	05/06/198	36	3	3036	3	4		BRASIL
2452383	F	06/12/199	29	3	3029	2	4	4	BRASIL
9297502	F	25/11/198	35	3	3035	3	4	2	BRASIL
2077396	F	10/08/200	18	3	3018	2	1	9	BRASIL
24074	F	24/03/199	24	3	3024	3	1	3	BRASIL
2040069	F	27/04/199	28	3	3028	3	2	9	BRASIL
8036	F	03/06/199	26	3	3026	1	2	3	BRASIL
2079542	F	13/03/199	25	3	3025	3	9	9	BRASIL
2726726	F	30/05/198	33	3	3033	3	4	1	BRASIL
2707527	F	09/07/199	26	3	3026	3	4	3	BRASIL
2252260	F	23/10/199	29	3	3029	3	1	4	BRASIL
2591049	F	02/05/200	20	3	3020	3	4	9	BRASIL
6627595	F	21/01/198	39	3	3039	3	4		BRASIL
2077477	F	23/04/199	27	3	3027	3	1	9	BRASIL
2080931	F	23/09/198	35	3	3035	3	1	9	BRASIL
9194258	F	24/10/198	36	3	3036	2	1	3	BRASIL
2340690	F	10/10/198	36	3	3036	2	4	4	BRASIL
2019558	F	04/05/198	32	3	3032	1	4		BRASIL
15318	F	30/10/198	37	3	3037	2	1	3	BRASIL
2738368	F	16/05/199	24	3	3024	3	1	1	BRASIL
2750988	F	01/06/199	24	3	3024	3	1		BRASIL
2089602	F	28/06/199	24	3	3024	2	1		BRASIL
2802090	F	02/07/199	29	3	3029	2	4		BRASIL
4069099	F	11/12/197	41	3	3041	4	1	2	BRASIL
2019558	F	25/05/198	36	3	3036	3	4		BRASIL
2237601	F	01/02/199	28	3	3028	3	1	3	BRASIL
9554157	F	11/07/198	36	3	3036	2	9		BRASIL
2078015	F	06/10/199	24	3	3024	2	1	9	BRASIL

2362287	F	09/11/198	37	3	3037	2	4		BRASIL
2645157	F	03/09/199	28	3	3028	3	4	3	BRASIL
2079429	F	22/12/199	26	3	3026	3	1	2	BRASIL
2017199	F	31/05/199	28	3	3028	3	4		BRASIL
7479387	F	27/03/198	32	3	3032	2	9	9	BRASIL
3316300	F	25/03/199	22	3	3022	3	1		BRASIL
3774554	F	30/05/199	27	3	3027	3	4	9	BRASIL
2752700	F	15/07/198	31	3	3031	3	4	2	BRASIL
2077396	F	29/11/199	26	3	3026	3	1	3	BRASIL
4047303	F	19/06/199	28	3	3028	3	1	3	BRASIL
2309254	F	15/07/199	30	3	3030	3	4	2	BRASIL
5860490	F	20/09/199	27	3	3027	2	1	4	BRASIL
2442108	F	05/08/199	27	3	3027	2	4	9	BRASIL
2752700	F	05/12/198	39	3	3039	2	4	3	BRASIL
2096463	F	18/07/200	16	3	3016	1	1	9	BRASIL
2655411	F	04/11/199	22	3	3022	3	4	9	BRASIL
2409208	F	29/11/198	31	3	3031	3	4	3	BRASIL
9200223	F	13/12/200	15	3	3015	3	1	2	BRASIL
2784874	F	15/05/199	26	3	3026	4	9	9	BRASIL
24074	F	05/09/198	32	3	3032	2	4	3	BRASIL
2309254	F	15/04/199	23	3	3023	3	4		BRASIL
2077396	F	02/05/199	25	3	3025	3	1	3	BRASIL
2079542	F	24/09/199	29	3	3029	1	1	4	BRASIL
2078589	F	31/10/198	35	3	3035	3	9	9	BRASIL
24074	F	30/08/198	35	3	3035	3	4	9	BRASIL
2078015	F	09/10/198	31	3	3031	3	1	9	BRASIL
2079798	F	19/09/199	30	3	3030	3	4		BRASIL
3956369	F	27/11/199	30	3	3030	3	4		BRASIL
2786117	F	25/02/198	39	3	3039	1	4	9	BRASIL
3038130	F	27/06/199	27	3	3027	2	1		BRASIL
2708566	F	27/12/199	26	3	3026	3	9	9	BRASIL
2081105	F	01/05/199	25	3	3025	3	4		BRASIL
15318	F	30/05/198	35	3	3035	2	1	4	BRASIL
2078589	F	06/06/198	32	3	3032	3	9	9	BRASIL
6848710	F	11/01/199	26	3	3026	3	3		BRASIL
2436450	F	11/07/199	24	3	3024	3	2	1	BRASIL
2591049	F	06/01/198	38	3	3038	3	4	9	BRASIL
9614990	F	22/10/199	28	3	3028	3	1		BRASIL
2754843	F	04/12/198	34	3	3034	3	4	9	BRASIL
2269988	F	10/04/198	34	3	3034	3	4	9	BRASIL
2079542	F	29/09/198	38	3	3038	1	9		BRASIL
2078015	F	04/11/198	35	3	3035	2	4	9	BRASIL
2707675	F	13/04/199	22	3	3022	3	9	9	BRASIL
2079542	F	29/05/199	31	3	3031	3	1	4	BRASIL
3956369	F	22/07/198	31	3	3031	3	4		BRASIL
1120	F	24/04/198	39	3	3039	2	9	9	BRASIL
2061562	F	10/01/200	19	3	3019	2	1	9	BRASIL

2499363	F	25/05/198	35	3	3035	3	4		BRASIL
27863	F	31/08/199	26	3	3026	3	4	2	BRASIL
2270714	F	09/12/197	46	3	3046	2	1	9	BRASIL
2568713	F	31/08/199	27	3	3027	3	1		BRASIL
4001303	F	15/11/199	27	3	3027	2	4	4	BRASIL
2126494	F	12/09/199	30	3	3030	2	9		BRASIL
2738368	F	20/06/198	38	3	3038	3	1	9	BRASIL
2410281	F	02/03/198	36	3	3036	3	4	2	BRASIL
2781859	F	03/11/198	33	3	3033	3	1	9	BRASIL
2537788	F	26/01/198	40	3	3040	3	1	2	BRASIL
5712068	F	11/09/198	31	3	3031	3	1	4	BRASIL
2244306	F	25/11/198	37	3	3037	2	1		BRASIL
2084058	F	11/02/198	40	3	3040	3	1	9	BRASIL
24074	F	26/04/199	28	3	3028	3	1	3	BRASIL
2738368	F	11/02/200	21	3	3021	3	1		BRASIL
2655411	F	05/12/198	33	3	3033	2	1		BRASIL
2499363	F	04/12/198	38	3	3038	3	4	9	BRASIL
2100754	F	11/08/198	38	3	3038	4	4	3	BRASIL
2269988	F	11/05/199	25	3	3025	2	4	9	BRASIL
2219654	F	26/10/198	38	3	3038	3	4	9	BRASIL
2082829	F	01/08/198	36	3	3036	3	4	3	BRASIL
9081496	F	12/09/198	33	3	3033	3	4	9	BRASIL
2789590	F	31/01/197	43	3	3043	1	1	4	BRASIL
2411393	F	14/07/199	28	3	3028	3	1	1	BRASIL
2077388	F	29/04/198	34	3	3034	2	1		BRASIL
2089580	F	03/12/198	34	3	3034	2	4	3	BRASIL
2363070	F	15/05/198	31	3	3031	2	4	9	BRASIL
15245	F	17/07/199	27	3	3027	4	1	9	BRASIL
24074	F	05/12/198	36	3	3036	2	4	3	BRASIL
2078082	F	09/06/198	31	3	3031	3	9	9	BRASIL
2206528	F	16/01/198	35	3	3035	2	4	4	BRASIL
2082829	F	28/05/198	35	3	3035	2	1	4	BRASIL
5860490	F	10/08/198	36	3	3036	2	1	4	BRASIL
2673932	F	15/09/198	40	3	3040	1	4	9	BRASIL
5420938	F	17/12/198	35	3	3035	3	1	9	BRASIL
2077396	F	29/01/199	26	3	3026	3	1	3	BRASIL
9297502	F	06/01/199	28	3	3028	3	4		BRASIL
2209195	F	23/03/199	25	3	3025	3	1	9	BRASIL
3774554	F	02/09/198	37	3	3037	3	1	4	BRASIL
5498465	F	15/11/199	28	3	3028	1	4	3	BRASIL
8036	F	17/05/199	30	3	3030	3	1	3	BRASIL
2006448	F	09/05/198	31	3	3031	3	4		BRASIL
2078503	F	23/11/200	20	3	3020	3	1	3	BRASIL
2269988	F	18/06/199	30	3	3030	3	4		BRASIL
2384299	F	25/01/200	14	3	3014	3	1	9	BRASIL
434	F	03/02/199	30	3	3030	3	1		BRASIL
10464	F	02/04/199	27	3	3027	3	9		BRASIL

2197022	F	25/05/198	31	3	3031	3	9		BRASIL
2022621	F	20/11/199	25	3	3025	3	2		BRASIL
9297502	F	07/07/198	32	3	3032	3	4		BRASIL
2331969	F	18/10/198	35	3	3035	3	4	4	BRASIL
3151794	F	19/11/199	29	3	3029	2	4		BRASIL
2151812	F	25/04/198	34	3	3034	2	3	3	BRASIL
6861873	F	14/09/197	41	3	3041	3	1	4	BRASIL
9757090	F	31/07/199	28	3	3028	2	1	2	BRASIL
2752700	F	20/11/197	42	3	3042	3	4	3	BRASIL
9065946	F	02/09/198	33	3	3033	3	9		BRASIL
2022621	F	18/05/198	32	3	3032	3	2		BRASIL
5073898	F	13/12/198	36	3	3036	3	4	3	BRASIL
2754738	F	04/05/199	23	3	3023	2	1		BRASIL
2550792	F	25/10/198	38	3	3038	2	1		BRASIL
5585422	F	06/03/200	17	3	3017	3	4		BRASIL
2237571	F	24/12/198	37	3	3037	2	2	2	BRASIL
2537788	F	29/06/198	35	3	3035	2	1	3	BRASIL
2079542	F	14/01/199	29	3	3029	3	9	9	BRASIL
2309254	F	29/10/198	36	3	3036	2	1		BRASIL
2495015	F	06/03/199	28	3	3028	3	1	9	BRASIL
2146355	F	28/01/199	26	3	3026	2	1	3	BRASIL
2309254	F	28/01/199	31	3	3031	3	4	3	BRASIL
2022621	F	05/05/198	40	3	3040	3	1	3	BRASIL
3037274	F	16/06/198	35	3	3035	2	9	9	BRASIL
24074	F	04/07/198	36	3	3036	3	2	3	BRASIL
3012212	F	16/11/198	33	3	3033	2	4	3	BRASIL
27022	F	19/03/198	35	3	3035	3	4	9	BRASIL
6602533	F	06/10/198	33	3	3033	3	4		BRASIL
2222043	F	03/05/198	35	3	3035	3	1	4	BRASIL
2655411	F	22/04/199	28	3	3028	3	4		BRASIL
2337754	F	04/11/198	38	3	3038	4	1		BRASIL
4013484	F	12/06/199	23	3	3023	2	9	3	BRASIL
7041624	F	26/12/197	43	3	3043	3	4	9	BRASIL
2752700	F	03/12/199	25	3	3025	3	4	9	BRASIL
3956369	F	25/06/200	17	3	3017	2	4	9	BRASIL
2078503	F	07/07/199	29	3	3029	2	1	2	BRASIL
2206528	F	12/09/197	42	3	3042	2	1	3	BRASIL
24074	F	01/06/199	28	3	3028	3	9	3	BRASIL
2319659	F	10/07/198	32	3	3032	3	4	9	BRASIL
2077396	F	01/05/198	34	3	3034	2	1	4	BRASIL
2710935	F	18/08/199	27	3	3027	4	4		BRASIL
7421710	F	06/12/199	29	3	3029	2	1		BRASIL
2825589	F	03/07/198	39	3	3039	1	2	4	BRASIL
2080931	F	27/07/199	23	3	3023	2	2	3	BRASIL
6848710	F	06/07/198	37	3	3037	2	9		BRASIL
2319659	F	06/08/198	35	3	3035	2	4	9	BRASIL
2738368	F	12/03/199	24	3	3024	3	1	3	BRASIL

6848710	F	28/12/200	19	3	3019	3	9	9	BRASIL
2655411	F	20/10/199	26	3	3026	3	4		BRASIL
3005402	F	30/03/198	36	3	3036	2	4		BRASIL
2688611	F	18/05/199	30	3	3030	2	1		BRASIL
2550792	F	25/01/199	31	3	3031	2	1		BRASIL
4001303	F	30/11/198	36	3	3036	3	4	9	BRASIL
2200473	F	24/07/198	37	3	3037	3	4		BRASIL
2340690	F	05/01/198	33	3	3033	2	1	9	BRASIL
3394514	F	06/04/198	37	3	3037	3	9		BRASIL
3956369	F	14/11/199	25	3	3025	2	4		BRASIL
2126494	F	21/09/198	35	3	3035	3	1	3	BRASIL
7869223	F	06/01/199	29	3	3029	3	4	9	BRASIL
2192896	F	25/03/198	34	3	3034	3	4	4	BRASIL
2078015	F	22/06/199	30	3	3030	3	4	9	BRASIL
2127881	F	14/06/198	33	3	3033	3	1		BRASIL
2452383	F	19/08/199	21	3	3021	3	4	3	BRASIL
2079038	F	05/01/199	31	3	3031	2	1		BRASIL
3956369	F	29/08/199	30	3	3030	3	4		BRASIL
2079542	F	07/11/199	30	3	3030	2	4	3	BRASIL
213845	F	14/06/198	37	3	3037	3	4	2	BRASIL
2087057	F	05/08/198	35	3	3035	2	1		BRASIL
2237571	F	11/04/199	26	3	3026	3	1	2	BRASIL
9138153	F	18/10/197	43	3	3043	2	1	2	BRASIL
2223546	F	27/05/198	34	3	3034	3	1	2	BRASIL
2082187	F	20/02/198	35	3	3035	2	4		BRASIL
2139200	F	13/11/198	35	3	3035	3	1	3	BRASIL
2078562	F	10/07/200	20	3	3020	3	1		BRASIL
7206771	F	11/09/199	30	3	3030	3	9	3	BRASIL
9161449	F	10/06/197	48	3	3048	4	1		BRASIL
3061388	F	20/10/199	29	3	3029	2	1		BRASIL
2361787	F	22/09/200	19	3	3019	3	1	3	BRASIL
2206595	F	06/06/199	29	3	3029	3	1		BRASIL
2126494	F	12/12/198	37	3	3037	3	9		BRASIL
3859	F	23/05/198	35	3	3035	3	2		BRASIL
2079186	F	11/08/198	33	3	3033	2	1	2	BRASIL
2823209	F	29/03/198	35	3	3035	3	1	3	BRASIL
2237601	F	08/02/198	32	3	3032	2	1	3	BRASIL
2499363	F	11/12/198	36	3	3036	3	4	9	BRASIL
2796449	F	16/11/199	26	3	3026	3	1	9	BRASIL
2755130	F	23/12/199	22	3	3022	2	1	3	BRASIL
2269988	F	16/09/198	33	3	3033	3	4	9	BRASIL
2151871	F	03/10/199	28	3	3028	3	2	3	BRASIL
2082101	F	11/09/199	29	3	3029	3	1	9	BRASIL
2319659	F	18/07/200	19	3	3019	3	4	9	BRASIL
2269988	F	25/10/198	35	3	3035	3	2	9	BRASIL
2280183	F	05/06/198	38	3	3038	2	1	3	BRASIL
9255826	F	16/11/198	32	3	3032	3	4		BRASIL

27022	F	01/09/1990	30	3	3030	2	4	9	BRASIL
6854729	F	21/11/1998	22	3	3022	2	3	3	BRASIL
3956369	F	13/07/1980	40	3	3040	3	4	9	BRASIL
2726726	F	07/11/200	19	3	3019	3	4	2	BRASIL
24074	F	07/02/1987	34	3	3034	3	4	3	BRASIL
2666723	F	02/05/1984	36	3	3036	3	1	4	BRASIL
3956369	F	20/06/1983	37	3	3037	2	1		BRASIL
2738368	F	22/12/1998	22	3	3022	3	1	3	BRASIL
2135116	F	02/03/1994	27	3	3027	1	1	3	BRASIL
2079186	F	24/10/198	39	3	3039	2	4	3	BRASIL
2499363	F	20/05/1982	38	3	3038	3	4	9	BRASIL
2302101	F	29/08/1980	40	3	3040	3	1	4	BRASIL
2078015	F	13/10/1984	36	3	3036	2	4	9	BRASIL
2082187	F	16/08/1994	26	3	3026	3	1		BRASIL
9488510	F	28/07/1993	27	3	3027	3	1		BRASIL
2781859	F	20/04/1995	25	3	3025	3	1	9	BRASIL
2206528	F	14/12/1996	24	3	3024	3	4	9	BRASIL
2384299	F	28/02/1984	37	3	3037	3	1	9	BRASIL
6263151	F	24/08/1980	40	3	3040	1	1	9	BRASIL
2691515	F	24/11/1985	35	3	3035	2	1	2	BRASIL
2078015	F	22/12/1994	26	3	3026	3	1	9	BRASIL
2153882	F	31/07/1979	41	3	3041	3	1	9	BRASIL
5428	F	18/08/1990	30	3	3030	3	4	9	BRASIL
3956369	F	12/04/1998	22	3	3022	3	4		BRASIL
10464	F	15/08/1995	25	3	3025	1	9		BRASIL
2384299	F	20/07/1984	36	3	3036	3	1	9	BRASIL
2594714	F	18/04/1995	25	3	3025	3	4	4	BRASIL
2436450	F	06/05/1984	37	3	3037	3	1	1	BRASIL
9554157	F	05/11/198	39	3	3039	3	1		BRASIL
2079542	F	06/03/1979	42	3	3042	2	1	4	BRASIL
2077434	F	07/10/1984	36	3	3036	2	4		BRASIL
2089777	F	26/08/1982	38	3	3038	3	9		BRASIL
9365230	F	25/03/1993	28	3	3028	3	1	3	BRASIL
5158044	F	09/11/1990	30	3	3030	3	9	3	BRASIL
24074	F	11/01/1982	39	3	3039	2	4	3	BRASIL
3272591	F	10/05/1984	36	3	3036	2	4	3	BRASIL
2164620	F	03/09/1990	30	3	3030	2	4		BRASIL
2173166	F	03/07/1997	23	3	3023	3	4		BRASIL
2089602	F	11/02/1990	31	3	3031	3	1		BRASIL
9725	F	24/02/1984	37	3	3037	2	4	9	BRASIL
10464	F	28/04/1999	22	3	3022	3	9		BRASIL
9119701	F	12/03/1990	31	3	3031	2	4	3	BRASIL
2144298	F	03/09/197	49	3	3049	4	4		BRASIL
5073898	F	17/04/1978	43	3	3043	2	4	3	BRASIL
3151794	F	03/05/1984	36	3	3036	3	4		BRASIL
2082527	F	17/05/199	29	3	3029	2	4	9	BRASIL
9923837	F	24/02/199	30	3	3030	1	1	9	BRASIL

2655411	F	11/08/199	26	3	3026	3	4		BRASIL
144975	F	29/04/198	32	3	3032	2	1	3	BRASIL
2688611	F	14/06/198	37	3	3037	1	4		BRASIL
13633	F	30/10/199	29	3	3029	2	1	2	BRASIL
12521	F	17/09/198	33	3	3033	3	1	9	BRASIL
2082187	F	02/10/200	18	3	3018	3	4		BRASIL
2077671	F	29/11/200	17	3	3017	3	1	9	BRASIL
2096463	F	02/02/198	38	3	3038	2	2	3	BRASIL
2025523	F	08/12/198	36	3	3036	1	4	1	BRASIL
2306344	F	06/10/199	21	3	3021	2	1	3	BRASIL
6602533	F	13/02/199	30	3	3030	3	4		BRASIL
2082101	F	15/08/199	28	3	3028	3	1	3	BRASIL
2248204	F	10/09/199	27	3	3027	1	1	3	BRASIL
9336508	F	05/07/199	25	3	3025	3	4	3	BRASIL
2292912	F	09/05/198	35	3	3035	2	2	3	BRASIL
2171945	F	25/09/199	23	3	3023	3	1	3	BRASIL
2755130	F	21/06/198	32	3	3032	3	1	3	BRASIL
2494523	F	26/05/198	35	3	3035	2	4	9	BRASIL
2338424	F	13/01/198	32	3	3032	3	1	9	BRASIL
2748223	F	21/08/198	35	3	3035	3	1	3	BRASIL
2259907	F	06/11/199	30	3	3030	3	1	3	BRASIL
2270609	F	11/08/200	18	3	3018	3	2	3	BRASIL
10464	F	12/08/199	25	3	3025	3	4	9	BRASIL
2269988	F	20/09/198	31	3	3031	2	3	9	BRASIL
2077396	F	24/04/199	23	3	3023	2	1	3	BRASIL
2688611	F	01/04/199	31	3	3031	3	1		BRASIL
2081601	F	26/04/198	33	3	3033	3	9	9	BRASIL
2466694	F	07/10/198	32	3	3032	3	4		BRASIL
9725	F	02/01/199	29	3	3029	3	4	9	BRASIL
2439263	F	29/11/198	34	3	3034	3	1		BRASIL
2078015	F	20/08/198	39	3	3039	2	1	9	BRASIL
2550792	F	06/04/199	28	3	3028	3	4	9	BRASIL
2075407	F	01/04/198	37	3	3037	2	4	9	BRASIL
2655411	F	13/04/198	33	3	3033	3	4		BRASIL
10464	F	15/05/198	37	3	3037	2	4		BRASIL
2361787	F	02/03/199	25	3	3025	3	4		BRASIL
3696162	F	21/08/198	39	3	3039	2	1	9	BRASIL
2232995	F	19/04/199	30	3	3030	3	1	9	BRASIL
2410281	F	11/03/199	29	3	3029	2	4	3	BRASIL
2222043	F	21/09/197	43	3	3043	3	4	9	BRASIL
2491710	F	15/01/199	29	3	3029	3	1	3	BRASIL
2080753	F	02/12/199	29	3	3029	3	1	9	BRASIL
2748223	F	08/10/198	31	3	3031	3	1	3	BRASIL
8036	F	30/05/199	30	3	3030	3	4	3	BRASIL
2244276	F	31/12/198	36	3	3036	2	4	3	BRASIL
5599504	F	20/11/198	37	3	3037	3	4	3	BRASIL
2295407	F	22/03/199	30	3	3030	3	4	9	BRASIL

2080796	F	09/03/199	25	3	3025	2	9	9	BRASIL
2206595	F	13/01/198	40	3	3040	1	4		BRASIL
2338424	F	02/09/197	41	3	3041	2	9		BRASIL
2738368	F	21/01/199	26	3	3026	3	1	1	BRASIL
5599504	F	17/08/199	22	3	3022	2	4	9	BRASIL
24074	F	19/01/200	17	3	3017	2	3	3	BRASIL
2655411	F	09/08/198	33	3	3033	3	4		BRASIL
2452383	F	15/01/199	22	3	3022	2	4	1	BRASIL
2781859	F	29/06/198	34	3	3034	3	1	9	BRASIL
2707527	F	27/11/199	27	3	3027	3	4	4	BRASIL
2082187	F	23/08/198	34	3	3034	2	1		BRASIL
3151794	F	17/09/198	33	3	3033	3	4		BRASIL
434	F	18/09/198	32	3	3032	4	4		BRASIL
2752700	F	24/12/199	30	3	3030	2	4	1	BRASIL
3956369	F	08/02/199	30	3	3030	3	4		BRASIL
2655411	F	13/06/198	36	3	3036	3	4		BRASIL
15245	F	29/11/198	39	3	3039	2	1	9	BRASIL
3388301	F	28/02/198	37	3	3037	1	4		BRASIL
2793008	F	02/05/198	36	3	3036	4	1	9	BRASIL
3808	F	05/03/198	38	3	3038	4	9	9	BRASIL
7421710	F	18/02/198	32	3	3032	3	9		BRASIL
2499363	F	07/07/199	25	3	3025	3	4		BRASIL
2079542	F	06/12/199	27	3	3027	3	1	4	BRASIL
2698684	F	05/08/199	30	3	3030	2	4	4	BRASIL
2244306	F	27/09/200	20	3	3020	3	1		BRASIL
9582800	F	24/10/198	39	3	3039	1	9	9	BRASIL
2781859	F	04/05/198	38	3	3038	2	1	9	BRASIL
2237601	F	17/01/198	35	3	3035	3	1	2	BRASIL
2491249	F	09/06/198	35	3	3035	2	1	4	BRASIL
2077396	F	05/07/198	32	3	3032	2	1	3	BRASIL
7659415	F	07/03/200	19	3	3019	3	1		BRASIL
2499363	F	04/06/198	32	3	3032	3	4	9	BRASIL
3157245	F	16/11/199	27	3	3027	3	1	3	BRASIL
2407205	F	18/01/198	33	3	3033	2	9	9	BRASIL
9725	F	27/07/199	27	3	3027	1	9	9	BRASIL
2079542	F	21/07/198	39	3	3039	3	1	4	BRASIL
2252287	F	10/06/197	41	3	3041	2	1	3	BRASIL
24074	F	06/06/199	26	3	3026	3	4	4	BRASIL
2084333	F	19/05/198	37	3	3037	1	9	9	BRASIL
2707527	F	11/01/198	39	3	3039	2	4	3	BRASIL
2738368	F	06/02/199	29	3	3029	3	1	9	BRASIL
2288893	F	27/11/197	41	3	3041	1	9	9	BRASIL
2772310	F	27/08/199	24	3	3024	2	1	9	BRASIL
2760681	F	15/03/199	30	3	3030	2	1	3	BRASIL
2084473	F	23/09/199	27	3	3027	3	1		BRASIL
2078481	F	13/06/199	29	3	3029	2	4	4	BRASIL
5332419	F	16/03/198	37	3	3037	3	2	9	BRASIL

7479387	F	14/08/198	31	3	3031	2	9	9	BRASIL
2078015	F	13/06/199	30	3	3030	2	1	9	BRASIL
2408570	F	17/07/198	32	3	3032	3	4	2	BRASIL
2023474	F	15/03/198	41	3	3041	2	4	3	BRASIL
2078015	F	18/07/199	23	3	3023	2	1	9	BRASIL
2790661	F	04/11/199	24	3	3024	3	9	9	BRASIL
2521385	F	30/10/197	43	3	3043	2	1	4	BRASIL
6734014	F	14/12/198	36	3	3036	3	1	9	BRASIL
2261987	F	21/07/198	37	3	3037	2	1	3	BRASIL
2270609	F	03/10/199	23	3	3023	3	1	4	BRASIL
6627595	F	19/01/198	39	3	3039	3	4		BRASIL
13633	F	01/06/199	28	3	3028	2	1	3	BRASIL
2126133	F	15/02/198	33	3	3033	2	1	9	BRASIL
2688611	F	13/06/198	33	3	3033	2	1		BRASIL
2384299	F	02/03/198	36	3	3036	3	1	9	BRASIL
2079542	F	09/08/198	35	3	3035	2	1	4	BRASIL
2594714	F	08/02/199	26	3	3026	3	1		BRASIL
2591049	F	15/02/199	24	3	3024	3	1	3	BRASIL
3005402	F	19/01/198	38	3	3038	3	4	9	BRASIL
477	F	03/05/199	25	3	3025	2	4		BRASIL
24074	F	05/12/199	29	3	3029	3	4	4	BRASIL
2781859	F	17/03/199	29	3	3029	2	1	9	BRASIL
2232103	F	16/04/197	46	3	3046	1	1	3	BRASIL
2408570	F	05/10/198	31	3	3031	3	4	9	BRASIL
2558254	F	16/11/200	18	3	3018	2	4	3	BRASIL
2338424	F	03/11/198	33	3	3033	2	9		BRASIL
2707527	F	11/06/198	39	3	3039	3	4	4	BRASIL
2078015	F	29/02/199	29	3	3029	3	1	9	BRASIL
2232154	F	28/01/198	36	3	3036	2	1		BRASIL
2079798	F	05/03/199	27	3	3027	1	2	9	BRASIL
2587335	F	11/08/197	41	3	3041	3	1	3	BRASIL
2303892	F	12/05/199	22	3	3022	3	1	3	BRASIL
2338424	F	09/10/199	30	3	3030	2	9		BRASIL
3005402	F	04/03/199	30	3	3030	2	1		BRASIL
2442612	F	12/04/199	22	3	3022	3	1	9	BRASIL
2241153	F	29/09/198	39	3	3039	2	1	9	BRASIL
2309254	F	15/09/198	33	3	3033	3	4		BRASIL
2485818	F	26/08/198	36	3	3036	3	1	4	BRASIL
2499363	F	08/02/200	19	3	3019	3	4	9	BRASIL
2269988	F	03/01/199	29	3	3029	2	9		BRASIL
2655411	F	29/05/199	22	3	3022	2	4		BRASIL
3356868	F	06/03/199	29	3	3029	4	1		BRASIL
2204622	F	23/05/198	36	3	3036	3	4	9	BRASIL
2738368	F	30/09/198	36	3	3036	3	1	2	BRASIL
2208857	F	07/10/199	30	3	3030	2	1	3	BRASIL
2005050	F	05/12/199	25	3	3025	2	4		BRASIL
24074	F	26/09/199	24	3	3024	2	4	1	BRASIL

2389746	F	19/03/197	43	3	3043	3	9	9	BRASIL
2081253	F	04/05/198	33	3	3033	4	1	9	BRASIL
2077396	F	23/08/199	26	3	3026	3	1	9	BRASIL
3157245	F	07/09/199	30	3	3030	3	1	4	BRASIL
2708779	F	21/01/199	24	3	3024	3	1	3	BRASIL
2688603	F	17/07/199	30	3	3030	2	9		BRASIL
2775972	F	29/09/198	38	3	3038	1	4		BRASIL
2537788	F	31/01/197	48	3	3048	1	1	2	BRASIL
2654032	F	06/10/198	36	3	3036	3	1		BRASIL
2319659	F	09/05/198	37	3	3037	3	4	2	BRASIL
2707527	F	30/04/199	22	3	3022	3	4	3	BRASIL
2439263	F	26/01/199	22	3	3022	2	4	4	BRASIL
3702693	F	22/07/199	29	3	3029	3	4		BRASIL
10499	F	08/01/198	38	3	3038	3	9	9	BRASIL
2752700	F	29/10/198	33	3	3033	1	4	1	BRASIL
2298317	F	08/07/199	23	3	3023	2	4	1	BRASIL
8013543	F	07/05/199	27	3	3027	1	1		BRASIL
2160137	F	14/08/198	37	3	3037	1	4	9	BRASIL
2270609	F	15/05/198	35	3	3035	2	4		BRASIL
24074	F	16/08/198	35	3	3035	2	4	3	BRASIL
176664	F	07/12/198	31	3	3031	3	4	3	BRASIL
9923837	F	15/12/198	33	3	3033	1	4	9	BRASIL
24074	F	11/07/199	29	3	3029	2	4	3	BRASIL
2269783	F	13/05/199	21	3	3021	3	4	2	BRASIL
2537788	F	04/08/198	34	3	3034	3	2	3	BRASIL
2223546	F	15/12/198	38	3	3038	3	1	2	BRASIL
2389746	F	02/06/198	37	3	3037	2	9	9	BRASIL
2270609	F	25/08/197	41	3	3041	3	4	1	BRASIL
2756978	F	22/12/198	33	3	3033	2	9	9	BRASIL
14109	F	29/11/198	31	3	3031	3	4	3	BRASIL
3157245	F	07/02/198	34	3	3034	2	1	9	BRASIL
2688611	F	11/09/199	23	3	3023	3	1	4	BRASIL
2323397	F	19/12/199	29	3	3029	2	4		BRASIL
2522101	F	15/05/198	33	3	3033	1	1	4	BRASIL
3508528	F	01/10/197	41	3	3041	3	4		BRASIL
2237571	F	27/02/198	32	3	3032	2	1	3	BRASIL
9725	F	26/03/199	29	3	3029	3	4	9	BRASIL
2504332	F	26/10/198	36	3	3036	2	1	3	BRASIL
2499363	F	28/03/197	42	3	3042	3	4	9	BRASIL
2022621	F	02/02/198	36	3	3036	3	4		BRASIL
2246929	F	29/10/198	35	3	3035	1	1	4	BRASIL
2161354	F	03/01/198	32	3	3032	3	4		BRASIL
2655411	F	06/10/198	35	3	3035	2	4		BRASIL
2246988	F	16/03/199	25	3	3025	3	1	4	BRASIL
10510	F	10/08/199	26	3	3026	3	4		BRASIL
2319659	F	16/10/199	29	3	3029	3	4	3	BRASIL
2079798	F	01/09/198	36	3	3036	3	1	9	BRASIL

2795671	F	15/06/199	27	3	3027	3	4	2	BRASIL
2269988	F	06/07/198	31	3	3031	3	4	9	BRASIL
2078015	F	03/06/199	28	3	3028	3	1		BRASIL
2337754	F	09/02/199	24	3	3024	3	1		BRASIL
2288338	F	25/11/197	43	3	3043	2	4		BRASIL
6625649	F	11/02/198	38	3	3038	1	9	9	BRASIL
2591049	F	11/03/199	31	3	3031	3	4	9	BRASIL
2090236	F	16/02/198	31	3	3031	3	4		BRASIL
2781859	F	25/08/198	39	3	3039	2	4	9	BRASIL
2087057	F	12/12/199	26	3	3026	1	9		BRASIL
13633	F	15/04/198	35	3	3035	2	4	1	BRASIL
2223538	F	24/07/198	36	3	3036	2	1		BRASIL
2077396	F	01/03/199	30	3	3030	3	1	4	BRASIL
2237571	F	28/06/199	29	3	3029	2	2		BRASIL
2726653	F	12/08/199	24	3	3024	3	1	3	BRASIL
7421710	F	04/01/199	27	3	3027	3	1		BRASIL
2269988	F	13/09/200	17	3	3017	3	1	9	BRASIL
4050665	F	03/09/199	22	3	3022	1	1	2	BRASIL
2077477	F	24/02/199	30	3	3030	3	1	3	BRASIL
2273748	F	24/07/198	40	3	3040	3	1		BRASIL
2786117	F	09/05/199	29	3	3029	2	4	3	BRASIL
2499363	F	18/05/198	38	3	3038	3	4	9	BRASIL
5073898	F	10/12/199	29	3	3029	3	2	3	BRASIL
2411393	F	10/01/198	34	3	3034	2	1	3	BRASIL
3508528	F	24/08/198	36	3	3036	3	4		BRASIL
4001303	F	22/07/199	30	3	3030	3	4	2	BRASIL
2079542	F	08/09/198	31	3	3031	3	1	4	BRASIL
2363070	F	11/08/198	36	3	3036	2	4	9	BRASIL
2122677	F	12/03/198	36	3	3036	3	1	9	BRASIL
3293262	F	11/11/198	36	3	3036	3	1		BRASIL
2691515	F	02/06/199	28	3	3028	3	1	1	BRASIL
2237601	F	20/06/199	28	3	3028	3	1	9	BRASIL
2078015	F	26/04/198	31	3	3031	3	1	9	BRASIL
2398680	F	09/03/199	27	3	3027	4	2	2	BRASIL
5717515	F	03/08/200	17	3	3017	3	9	9	BRASIL
2192896	F	08/12/199	22	3	3022	3	1	9	BRASIL
2080931	F	14/10/199	25	3	3025	3	1	3	BRASIL
2223538	F	27/05/198	32	3	3032	2	1		BRASIL
2755165	F	09/04/200	13	3	3013	3	4	2	BRASIL
2200473	F	24/03/199	23	3	3023	3	9		BRASIL
2786117	F	14/02/198	37	3	3037	2	4	2	BRASIL
2384299	F	04/07/199	23	3	3023	3	1	9	BRASIL
2436450	F	02/05/198	36	3	3036	1	1	9	BRASIL
2079798	F	29/07/199	24	3	3024	3	1	9	BRASIL
3956369	F	21/04/200	17	3	3017	3	9		BRASIL
24074	F	19/04/198	37	3	3037	3	4	2	BRASIL
2000733	F	06/08/198	40	3	3040	2	4	4	BRASIL

2655411	F	27/07/198	36	3	3036	3	4		BRASIL
2269988	F	31/10/197	42	3	3042	3	1	9	BRASIL
2082187	F	20/03/200	17	3	3017	3	4		BRASIL
9923837	F	21/08/200	19	3	3019	2	3	9	BRASIL
3058336	F	22/03/199	29	3	3029	2	1		BRASIL
3956369	F	24/03/200	20	3	3020	2	4		BRASIL
27022	F	28/01/199	27	3	3027	3	4	9	BRASIL
3956369	F	12/09/198	32	3	3032	3	4		BRASIL
2085194	F	10/07/199	25	3	3025	2	1	3	BRASIL
2748223	F	14/08/198	31	3	3031	3	1	2	BRASIL
2518384	F	18/02/200	19	3	3019	2	1	3	BRASIL
2499363	F	10/12/199	27	3	3027	3	4	9	BRASIL
2499363	F	23/10/199	27	3	3027	3	4	9	BRASIL
2077396	F	27/10/198	33	3	3033	2	1	3	BRASIL
3972925	F	24/10/198	35	3	3035	2	4	2	BRASIL
2082187	F	19/11/198	33	3	3033	3	4		BRASIL
9194258	F	24/05/199	29	3	3029	2	1		BRASIL
2079542	F	28/09/198	35	3	3035	3	1	4	BRASIL
3956369	F	20/10/198	36	3	3036	3	4		BRASIL
24074	F	02/12/198	38	3	3038	3	1	3	BRASIL
2338424	F	09/12/198	33	3	3033	3	4		BRASIL
2499363	F	09/08/198	37	3	3037	3	4		BRASIL
2789590	F	10/02/198	40	3	3040	2	1	4	BRASIL
3157245	F	22/06/199	23	3	3023	3	1	2	BRASIL
2084473	F	05/03/199	31	3	3031	3	4	9	BRASIL
2555646	F	02/06/199	30	3	3030	3	1		BRASIL
6086381	F	03/12/198	32	3	3032	3	1	9	BRASIL
2558815	F	24/03/200	21	3	3021	1	4		BRASIL
7479387	F	24/06/199	22	3	3022	2	9	9	BRASIL
3316300	F	16/10/199	28	3	3028	1	9		BRASIL
2077388	F	02/09/200	16	3	3016	2	1		BRASIL
3316300	F	28/02/199	30	3	3030	3	1		BRASIL
2754843	F	08/12/197	41	3	3041	3	1	9	BRASIL
2407205	F	01/02/199	30	3	3030	2	9	9	BRASIL
2070413	F	28/10/198	31	3	3031	2	2	4	BRASIL
5717515	F	17/11/198	38	3	3038	3	4	9	BRASIL
5515483	F	12/10/198	37	3	3037	2	1	3	BRASIL
2452383	F	26/10/199	25	3	3025	3	4	1	BRASIL
2738368	F	18/12/199	25	3	3025	3	1		BRASIL
2687038	F	26/06/198	38	3	3038	3	1		BRASIL
6848710	F	15/03/200	18	3	3018	2	9		BRASIL
15318	F	06/04/198	34	3	3034	4	1		BRASIL
2142376	F	17/01/198	32	3	3032	3	1	3	BRASIL
24074	F	17/09/199	23	3	3023	3	4	3	BRASIL
2153084	F	28/08/199	22	3	3022	3	1	3	BRASIL
2452383	F	12/06/199	25	3	3025	3	4	2	BRASIL
8036	F	04/01/198	37	3	3037	1	2	2	BRASIL

2499363	F	20/04/199	27	3	3027	3	4	9	BRASIL
7768400	F	26/04/199	26	3	3026	3	4	3	BRASIL
2080931	F	27/06/199	30	3	3030	2	1	3	BRASIL
2208172	F	05/05/198	33	3	3033	2	4	9	BRASIL
2707527	F	07/09/198	37	3	3037	3	4	3	BRASIL
2452383	F	05/10/199	27	3	3027	3	4	1	BRASIL
2738368	F	09/10/199	28	3	3028	3	1		BRASIL
2082187	F	21/10/197	45	3	3045	3	4	1	BRASIL
2439263	F	13/02/198	33	3	3033	3	4		BRASIL
2707527	F	08/03/198	33	3	3033	3	4	3	BRASIL
2504332	F	15/03/198	37	3	3037	3	1		BRASIL
3580822	F	07/02/198	34	3	3034	3	4	3	BRASIL
176664	F	05/03/198	31	3	3031	2	4	4	BRASIL
2078015	F	07/02/198	35	3	3035	3	1	9	BRASIL
5510341	F	12/05/199	23	3	3023	4	9	9	BRASIL
2319659	F	13/08/198	32	3	3032	2	4	4	BRASIL
2550792	F	30/07/198	32	3	3032	3	4	9	BRASIL
2802112	F	27/08/199	23	3	3023	3	4	9	BRASIL
6848710	F	19/03/199	27	3	3027	3	4	9	BRASIL
3956369	F	17/08/199	21	3	3021	3	9		BRASIL
3037274	F	06/10/198	33	3	3033	3	9	9	BRASIL
2784874	F	23/10/199	29	3	3029	2	1	9	BRASIL
2219654	F	17/12/198	33	3	3033	2	1	9	BRASIL
2550792	F	25/09/199	29	3	3029	3	4	4	BRASIL
2308762	F	26/10/197	47	3	3047	4	4	1	BRASIL
2655411	F	05/09/199	25	3	3025	2	4		BRASIL
13846	F	23/01/199	30	3	3030	2	1	2	BRASIL
7257406	F	30/10/200	15	3	3015	3	4	2	BRASIL
2688611	F	18/02/199	31	3	3031	3	1		BRASIL
2078015	F	16/06/198	34	3	3034	3	1	9	BRASIL
2499363	F	25/04/199	27	3	3027	1	4		BRASIL
3151794	F	23/05/198	37	3	3037	3	4		BRASIL
2550792	F	09/11/198	38	3	3038	3	1	4	BRASIL
2080532	F	05/05/200	17	3	3017	1	1	3	BRASIL
2089602	F	16/09/198	38	3	3038	3	1		BRASIL
2078481	F	08/02/198	41	3	3041	1	4	4	BRASIL
2299216	F	01/03/198	32	3	3032	3	4	9	BRASIL
2688611	F	22/11/198	39	3	3039	2	1	4	BRASIL
9554157	F	04/05/198	37	3	3037	3	9		BRASIL
2784874	F	02/07/198	35	3	3035	3	1	3	BRASIL
2591049	F	09/06/198	35	3	3035	2	1	9	BRASIL
2079542	F	31/08/198	32	3	3032	2	9	9	BRASIL
2077396	F	16/02/199	23	3	3023	3	1	3	BRASIL
2129469	F	07/09/198	35	3	3035	2	1	9	BRASIL
2078678	F	24/06/200	20	3	3020	1	1	9	BRASIL
2708566	F	08/09/199	30	3	3030	3	9	9	BRASIL
2802082	F	27/04/198	37	3	3037	3	9	9	BRASIL

2089777	F	27/05/198	37	3	3037	2	9	9	BRASIL
24074	F	15/10/198	39	3	3039	2	1	3	BRASIL
2078015	F	15/05/198	35	3	3035	3	9	9	BRASIL
2784874	F	21/06/198	35	3	3035	3	1	9	BRASIL
2752700	F	15/09/198	32	3	3032	3	4	3	BRASIL
2655411	F	18/10/199	28	3	3028	3	4		BRASIL
9255826	F	22/12/198	38	3	3038	3	1	4	BRASIL
2237571	F	25/12/197	42	3	3042	2	1	3	BRASIL
2006197	F	04/05/197	42	3	3042	3	4	2	BRASIL
2079542	F	13/05/198	38	3	3038	3	9	9	BRASIL
6003575	F	10/07/198	37	3	3037	2	9		BRASIL
2295415	F	12/12/198	39	3	3039	2	1	9	BRASIL
9923837	F	27/04/199	26	3	3026	2	4	9	BRASIL
7421710	F	23/02/199	30	3	3030	3	9		BRASIL
2087715	F	10/02/199	28	3	3028	3	1	3	BRASIL
3048551	F	01/10/199	30	3	3030	2	4		BRASIL
6848710	F	13/05/199	30	3	3030	3	4	9	BRASIL
3005402	F	26/10/199	30	3	3030	3	4		BRASIL
2025523	F	31/07/198	39	3	3039	3	2	3	BRASIL
7306504	F	28/02/198	40	3	3040	3	1		BRASIL
4013484	F	18/01/198	34	3	3034	1	4	2	BRASIL
9554157	F	17/03/198	32	3	3032	2	9		BRASIL
2080532	F	08/02/199	29	3	3029	1	2	4	BRASIL
2079798	F	26/04/199	27	3	3027	1	1	9	BRASIL
2087057	F	14/10/198	33	3	3033	3	1		BRASIL
24074	F	25/08/199	24	3	3024	3	4	1	BRASIL
15369	F	04/12/198	34	3	3034	2	2		BRASIL
2206528	F	25/02/198	38	3	3038	2	4	3	BRASIL
2079887	F	27/06/198	39	3	3039	3	4	4	BRASIL
2078015	F	07/05/199	26	3	3026	3	1	9	BRASIL
2040069	F	19/08/199	21	3	3021	1	4	3	BRASIL
2604388	F	15/03/199	28	3	3028	2	1	9	BRASIL
2257890	F	08/10/198	36	3	3036	3	1		BRASIL
2806215	F	17/02/198	35	3	3035	4	1		BRASIL
2781859	F	28/02/198	41	3	3041	3	1	9	BRASIL
2594714	F	24/03/198	40	3	3040	3	1	3	BRASIL
2323397	F	10/11/198	39	3	3039	3	4		BRASIL
2088193	F	04/06/198	32	3	3032	1	1		BRASIL
15245	F	17/07/200	20	3	3020	3	9	9	BRASIL
5101654	F	10/03/198	35	3	3035	1	1	9	BRASIL
2499363	F	15/04/199	21	3	3021	3	4		BRASIL
2688611	F	03/10/199	28	3	3028	3	1		BRASIL
2645157	F	09/11/199	26	3	3026	3	9	9	BRASIL
3073300	F	07/03/197	42	3	3042	2	1	3	BRASIL
2309254	F	08/07/198	33	3	3033	2	4		BRASIL
2007037	F	06/07/198	40	3	3040	3	4		BRASIL
6697054	F	05/12/199	30	3	3030	2	1	3	BRASIL

2082128	F	15/01/199	31	3	3031	3	1	9	BRASIL
2755165	F	04/02/199	30	3	3030	2	4		BRASIL
24074	F	16/06/199	27	3	3027	3	1	3	BRASIL
24074	F	11/04/198	40	3	3040	3	4	3	BRASIL
2594714	F	23/06/199	30	3	3030	1	1	3	BRASIL
3012212	F	11/02/198	37	3	3037	3	1		BRASIL
2111624	F	24/01/198	33	3	3033	3	9	9	BRASIL
2081016	F	27/05/200	21	3	3021	3	1	3	BRASIL
2078015	F	11/06/200	18	3	3018	3	4	9	BRASIL
2010631	F	17/01/199	26	3	3026	2	1	2	BRASIL
2439263	F	10/01/198	39	3	3039	2	1	9	BRASIL
24074	F	01/07/198	35	3	3035	1	4	3	BRASIL
9336508	F	02/07/199	21	3	3021	3	4		BRASIL
2078066	F	15/03/199	31	3	3031	3	2	3	BRASIL
2752700	F	06/04/199	27	3	3027	1	4	9	BRASIL
15318	F	05/09/199	23	3	3023	3	1	9	BRASIL
2078015	F	02/07/198	34	3	3034	3	4		BRASIL
2708779	F	22/09/198	36	3	3036	3	1	2	BRASIL
2077396	F	18/04/199	30	3	3030	3	1	2	BRASIL
15369	F	21/10/200	20	3	3020	3	1		BRASIL
2232057	F	12/03/198	41	3	3041	3	1		BRASIL
3394514	F	06/09/197	41	3	3041	3	1		BRASIL
5407591	F	11/08/198	31	3	3031	3	9		BRASIL
13846	F	01/03/199	30	3	3030	2	1	3	BRASIL
24074	F	29/07/199	28	3	3028	3	4	3	BRASIL
2588188	F	23/10/197	41	3	3041	3	4		BRASIL
13846	F	08/09/200	17	3	3017	3	1	3	BRASIL
2594714	F	15/04/199	27	3	3027	3	2		BRASIL
2078252	F	15/03/199	26	3	3026	2	1	3	BRASIL
2075962	F	11/05/198	38	3	3038	2	2	3	BRASIL
24074	F	26/07/199	31	3	3031	3	4	4	BRASIL
7244339	F	23/09/198	39	3	3039	3	9	9	BRASIL
8036	F	12/02/198	35	3	3035	2	4	2	BRASIL
5712068	F	04/02/198	37	3	3037	2	1	3	BRASIL
3956369	F	17/01/198	36	3	3036	1	4		BRASIL
2252694	F	21/08/198	35	3	3035	3	1	4	BRASIL
2521431	F	28/01/198	39	3	3039	3	1	3	BRASIL
2082187	F	26/05/199	24	3	3024	3	1		BRASIL
2082128	F	06/05/198	39	3	3039	3	4	9	BRASIL
2752700	F	08/02/200	15	3	3015	3	4	1	BRASIL
2208857	F	30/06/199	21	3	3021	3	1	2	BRASIL
2007037	F	17/06/199	28	3	3028	3	4		BRASIL
7421710	F	19/01/199	23	3	3023	3	1		BRASIL
2591049	F	01/01/199	26	3	3026	1	1		BRASIL
3272591	F	01/12/199	27	3	3027	2	1		BRASIL
2755165	F	14/03/198	38	3	3038	2	4	3	BRASIL
9194258	F	25/03/198	36	3	3036	4	1	9	BRASIL

2253046	F	24/04/200	21	3	3021	3	1	1	BRASIL
2591049	F	16/08/199	25	3	3025	3	4	9	BRASIL
2755165	F	18/11/199	26	3	3026	3	4		BRASIL
2080931	F	26/11/197	44	3	3044	3	1	3	BRASIL
2738368	F	05/05/199	24	3	3024	3	1	1	BRASIL
2726726	F	17/01/200	17	3	3017	3	1	1	BRASIL
2755165	F	15/08/199	28	3	3028	3	2	2	BRASIL
2738368	F	16/12/199	30	3	3030	3	1		BRASIL
2395886	F	23/11/199	22	3	3022	2	4	2	BRASIL
3151794	F	26/01/199	22	3	3022	2	4	3	BRASIL
2688611	F	24/04/198	35	3	3035	2	4	4	BRASIL
5407591	F	14/08/199	29	3	3029	2	9	9	BRASIL
2089777	F	05/04/199	27	3	3027	2	9		BRASIL
2738368	F	08/04/199	25	3	3025	3	1		BRASIL
2726734	F	06/03/199	30	3	3030	3	4	9	BRASIL
2306344	F	18/09/199	26	3	3026	2	1	3	BRASIL
2726726	F	15/01/198	35	3	3035	3	1		BRASIL
2615746	F	30/11/199	26	3	3026	2	2	1	BRASIL
4056752	F	02/05/200	16	3	3016	1	4	9	BRASIL
2278855	F	11/06/199	31	3	3031	3	1	9	BRASIL
3956369	F	21/07/199	30	3	3030	2	4	9	BRASIL
2708779	F	18/10/199	22	3	3022	1	1	3	BRASIL
2738368	F	05/12/199	28	3	3028	3	1	3	BRASIL
2752700	F	23/11/199	23	3	3023	2	4	9	BRASIL
3450198	F	14/05/199	22	3	3022	3	9	9	BRASIL
2691515	F	24/04/198	38	3	3038	2	1	1	BRASIL
13846	F	17/06/198	41	3	3041	3	1		BRASIL
2738368	F	27/05/199	28	3	3028	3	1		BRASIL
2738368	F	18/12/199	25	3	3025	3	1	2	BRASIL
2752700	F	12/03/198	32	3	3032	2	4	3	BRASIL
2591049	F	25/10/200	16	3	3016	3	2		BRASIL
3956369	F	22/08/200	18	3	3018	3	2	9	BRASIL
13846	F	18/09/199	23	3	3023	3	1		BRASIL
2079542	F	28/03/199	28	3	3028	2	1	4	BRASIL
2077477	F	26/09/199	22	3	3022	3	1	2	BRASIL
2083094	F	04/07/198	32	3	3032	3	1		BRASIL
2594277	F	24/04/198	38	3	3038	2	1		BRASIL
2269988	F	27/03/198	33	3	3033	2	9	9	BRASIL
2738368	F	21/04/200	20	3	3020	3	1		BRASIL
6988938	F	16/03/199	29	3	3029	2	1		BRASIL
2750988	F	15/03/198	38	3	3038	3	4	4	BRASIL
2323397	F	24/07/200	20	3	3020	2	4	2	BRASIL
2707527	F	02/08/198	37	3	3037	2	4	4	BRASIL
2232146	F	26/03/199	26	3	3026	3	1		BRASIL
2726726	F	21/10/199	28	3	3028	3	1		BRASIL
7659415	F	31/05/199	28	3	3028	3	1	3	BRASIL
9194258	F	05/02/198	34	3	3034	2	1		BRASIL

2738368	F	22/07/199	23	3	3023	3	4		BRASIL
2591049	F	26/06/199	22	3	3022	3	1		BRASIL
2173166	F	12/07/199	24	3	3024	2	4		BRASIL
9628	F	08/02/199	23	3	3023	3	9		BRASIL
7659415	F	04/03/199	25	3	3025	3	2	3	BRASIL
5510341	F	06/08/199	23	3	3023	3	9	9	BRASIL
3170527	F	08/05/199	27	3	3027	2	4	9	BRASIL
5073898	F	11/10/198	32	3	3032	3	4	4	BRASIL
2078767	F	02/12/199	31	3	3031	3	1		BRASIL
2339196	F	04/04/200	21	3	3021	1	4	3	BRASIL
3956369	F	14/12/199	27	3	3027	3	2	3	BRASIL
2726998	F	07/11/200	21	3	3021	2	9	2	BRASIL
144975	F	26/06/200	15	3	3015	2	4	2	BRASIL
6995462	F	21/03/200	19	3	3019	3	9	9	BRASIL
3580822	F	03/01/199	31	3	3031	2	1	4	BRASIL
2688611	F	19/11/199	24	3	3024	1	1	4	BRASIL
2688611	F	02/04/199	24	3	3024	3	1	4	BRASIL
9194258	F	27/12/198	36	3	3036	3	1		BRASIL
2755130	F	22/08/198	38	3	3038	3	4	3	BRASIL
2452383	F	08/12/199	26	3	3026	1	4		BRASIL
2077477	F	03/01/197	44	3	3044	4	1		BRASIL
2081253	F	03/06/199	27	3	3027	2	4		BRASIL
2363070	F	13/03/198	32	3	3032	1	4	9	BRASIL
2080443	F	18/05/199	23	3	3023	1	2	3	BRASIL
2738368	F	22/01/199	31	3	3031	3	1		BRASIL
9194258	F	16/12/199	23	3	3023	3	4		BRASIL
2738368	F	06/06/198	32	3	3032	3	1		BRASIL
2337800	F	07/07/199	29	3	3029	3	4		BRASIL
2027356	F	13/09/198	37	3	3037	2	2	3	BRASIL
2278855	F	23/06/198	33	3	3033	3	1	9	BRASIL
2591049	F	30/06/200	18	3	3018	3	1		BRASIL
2040069	F	27/03/199	31	3	3031	1	4	3	BRASIL
2452383	F	07/07/198	36	3	3036	2	4		BRASIL
2269988	F	03/12/198	34	3	3034	2	9		BRASIL
3702693	F	31/01/198	37	3	3037	3	4	9	BRASIL
2079186	F	24/11/198	39	3	3039	3	1	2	BRASIL
2761041	F	04/09/198	38	3	3038	3	4	3	BRASIL
2278855	F	03/08/198	38	3	3038	1	1	9	BRASIL
2591049	F	09/02/199	23	3	3023	2	1		BRASIL
2615797	F	01/06/200	16	3	3016	2	4	3	BRASIL
144975	F	09/01/198	38	3	3038	2	2	3	BRASIL
24074	F	21/03/199	27	3	3027	3	4	4	BRASIL
3702693	F	03/01/199	28	3	3028	2	2	3	BRASIL
2760681	F	26/05/198	33	3	3033	3	1	3	BRASIL
7061021	F	01/05/200	19	3	3019	3	4		BRASIL
2025523	F	13/05/199	25	3	3025	3	4	3	BRASIL
2278855	F	06/06/199	25	3	3025	3	4	9	BRASIL

2091801	F	09/01/199	31	3	3031	1	4	4	BRASIL
2591049	F	14/07/199[illegible]	25	3	3025	2	4		BRASIL
2738368	F	20/01/200[illegible]	17	3	3017	3	9		BRASIL
3580822	F	18/04/198[illegible]	36	3	3036	3	1	1	BRASIL
2365146	F	05/04/200[illegible]	15	3	3015	3	4	2	BRASIL
3037274	F	31/07/199[illegible]	31	3	3031	3	9	9	BRASIL
2781859	F	25/02/199[illegible]	22	3	3022	3	1	9	BRASIL
2726726	F	25/01/200[illegible]	21	3	3021	3	1	1	BRASIL
2085194	F	22/11/198[illegible]	35	3	3035	2	4	1	BRASIL
2420015	F	28/08/200[illegible]	17	3	3017	2	1		BRASIL
2079798	F	29/03/199[illegible]	23	3	3023	3	1	9	BRASIL
3956369	F	12/10/198[illegible]	34	3	3034	3	4	3	BRASIL
2504332	F	16/09/198[illegible]	37	3	3037	1	1	2	BRASIL
2261987	F	11/01/200[illegible]	18	3	3018	3	1	3	BRASIL
2027186	F	13/10/198[illegible]	34	3	3034	3	1	9	BRASIL
144975	F	08/10/199[illegible]	25	3	3025	1	2	1	BRASIL
5860490	F	09/09/198[illegible]	41	3	3041	3	1	4	BRASIL
2738368	F	06/04/200	20	3	3020	3	4		BRASIL
2232022	F	10/01/198[illegible]	35	3	3035	3	4		BRASIL
2558815	F	05/06/198[illegible]	34	3	3034	3	4	9	BRASIL
2129469	F	05/04/199[illegible]	22	3	3022	2	4	3	BRASIL
2078015	F	05/10/199[illegible]	22	3	3022	3	4	9	BRASIL
6020917	F	29/08/199[illegible]	27	3	3027	3	2	3	BRASIL

CO_PAIS	SG_UF	ID_RG_RES	CO_RG_RES	D_MN_RES	O_MUN_RI	CS_ZONA	SURTO_SG	OSOCOMI	AVE_SUINO
1	BA	NUCLEO RI	1380	CAMACARI	290570	1	1	2	9
1	AM	ENTORNO	5584	MANAUS	130260	1		2	2
1	RJ			ITATIAIA	330225				
1	RJ			RIO DE JAN	330455	1	9	2	2
1	SP	GVE XIX M/	1344	QUINTANA	354200	1	2	2	2
1	SP	GVE XVII C/	1342	SUMARE	355240	1	2	2	2
1	SP	GVE IX FRA	1334	MAIRIPORA	352850	1	2	2	2
1	RJ			CAMPOS D	330100	1	2	2	2
1	MS	NRS DE DO	1966	DOURADO	500370	1			
1	RJ			MESQUITA	330285	1	1	2	2
1	SP	GVE I CAPI	1331	SAO PAULO	355030	1			
1	MS	EX ARS DE	1975	CAMPO GF	500270	1	2	2	2
1	AM	ENTORNO	5584	MANAUS	130260	1		2	2
1	RJ			RIO DE JAN	330455	1		2	
1	MA	REGIONAL	1440	IMPERATRI	210530	1		2	2
1	SP	GVE I CAPI	1331	SAO PAULO	355030	1	2	2	2
1	SP	GVE I CAPI	1331	SAO PAULO	355030	1		2	2
1	CE	11 CRES SC	1521	URUOCA	231390	1	1	2	2
1	SP	GVE XI ARA	1336	ARACATUB	350280	1	2	2	2
1	GO	CENTRAL	1779	GOIANIA	520870	1		2	2
1	RJ			RIO DE JAN	330455				
1	RR			BOA VISTA	140010	1	1	2	2
1	RJ			CAMPOS D	330100	1	2	2	2
1	SP	GVE XXXI S	1353	SOROCABA	355220	1	1	2	2
1	CE	1 CRES FOF	1519	FORTALEZA	230440	1	9	2	2
1	SP	GVE XVII C/	1342	JUNDIAI	352590	1			
1	BA	NUCLEO RI	1380	SALVADOR	292740	1	1		9
1	SP	GVE XVII C/	1342	CAMPINAS	350950	1	2	2	2
1	PE	001	1497	RECIFE	261160	1		2	9
1	RJ			NOVA IGU/	330350	1			
1	SP	GVE I CAPI	1331	SAO PAULO	355030	3	2	2	9
1	SP	GVE XXIX S	1354	SAO JOSE D	354980	1		2	2
1	MG	BELO HORI	1449	CONTAGEN	311860	1	2	2	2
1	MG	UBERLAND	1462	UBERLAND	317020	1	2	2	2
1	BA	NUCLEO RI	1394	SAUDE	292980		9	9	9
1	SP	GVE XXX JA	1574	TRES FRON	355490	1		2	2
1	GO	CENTRAL	1779	GOIANIA	520870	1	1	2	2
1	SP	GVE XVII C/	1342	ARTUR NO	350380	1	2	2	2
1	SP	GVE I CAPI	1331	SAO PAULO	355030	1	9	9	9
1	PE	006	1501	ARCOVERD	260120	1			
1	SP	GVE XVII C/	1342	CAMPINAS	350950	3	2		2
1	AM	ENTORNO	5584	MANAUS	130260	1		2	2
1	SP	GVE I CAPI	1331	SAO PAULO	355030	1	1	2	2
1	SP	GVE X OSA	1335	CARAPICUI	351060	1	2	2	2
1	RJ			RIO DE JAN	330455	1		2	1
1	AL	1 MICRORI	1533	MACEIO	270430	1	1	2	2

1	SP	GVE XVII CA	1342	CAMPINAS	350950	1	2	2	2
1	SP	GVE IX FRA	1334	CAJAMAR	350920	1	9	2	9
1	MT	CUIABA	1578	CHAPADA	510300	1		9	9
1	AL	1 MICRORF	1533	MACEIO	270430	1		9	9
1	BA	NUCLEO RE	1380	SALVADOR	292740		9	9	9
1	PA	01 REGION	1484	BELEM	150140	1	1	2	9
1	SC	BLUMENAU	1564	BLUMENAU	420240		2	2	2
1	RJ			PETROPOLI	330390	1			
1	PE	001	1497	CAMARAG	260345	1	1	2	2
1	PR	03RS PONT	1357	PONTA GR	411990	1	2	2	9
1	SP	GVE VIII M	1333	FERRAZ DE	351570	1	9	9	9
1	BA	NUCLEO RE	1380	SALVADOR	292740	1	2	2	2
1	SP	GVE X OSA	1335	ITAPEVI	352250	1	2	2	2
1	BA	NUCLEO RE	1394	TAPIRAMU	293130		9	9	9
1	SP	GVE VII SAI	1332	SAO BERNA	354870	1		2	2
1	SP	GVE I CAPI	1331	SAO PAULO	355030	1	1	2	2
1	RS	002 CRS	1607	CACHOEIRI	430310		2	2	2
1	PA	01 REGION	1484	ANANINDE	150080	1			
1	CE	2 CRES CAU	1512	CAUCAIA	230370	1	9	2	9
1	SP	GVE XX PIR	1345	ELIAS FAUS	351490	1	1	2	2
1	RS	013 CRS	1609	VENANCIO	432260	1	2	2	2
1	RJ			RIO DE JAN	330455	1		2	2
1	SP	GVE X OSA	1335	TABOAO D	355280	1		2	2
1	RJ			SAO GONC	330490	1	2	2	2
1	SP	GVE XXVIII	1576	SAO SEBAS	355070	1	2	2	2
1	BA	NUCLEO RE	1380	SALVADOR	292740		9	9	9
1	PR	02RS METF	1356	CURITIBA	410690	1	2	2	2
1	RJ			RIO DE JAN	330455	1		2	2
1	SP	GVE I CAPI	1331	SAO PAULO	355030	1	2	2	2
1	RJ			RIO DE JAN	330455	9	9	9	9
1	PE	001	1497	RECIFE	261160	1	2	2	2
1	RJ			RIO DE JAN	330455	1	2	2	2
1	CE	1 CRES FOF	1519	FORTALEZA	230440	3	1	2	2
1	CE	20 CRES CF	1530	CRATO	230420	1	1	2	2
1	RJ			NITEROI	330330	1			
1	SP	GVE IX FRA	1334	FRANCISCO	351630	1	2	2	2
1	PB	III NRS CAN	1421	CAMPINA (	250400	1	2	2	2
1	BA	NUCLEO RE	1380	SALVADOR	292740	1	9	2	
1	MT	CUIABA	1578	CUIABA	510340	1		2	2
1	SP	GVE VII SAI	1332	SANTO AN	354780	1	1	2	2
1	SP	GVE VIII M	1333	MOGI DAS	353060	1	9	9	9
1	PB	I NRS JOAC	1377	JOAO PESS	250750	1			
1	MG	BELO HORI	1449	RIBEIRAO D	315460	1	9	2	2
1	DF	SUPERINTE	6262	SOBRADIN	530170	1	1	2	2
1	PR	02RS METF	1356	ARAUCARI	410180	1	2	2	2
1	RJ			RESENDE	330420	1		2	2
1	PR	04RS IRATI	1358	IRATI	411070	2		2	2

1	PB	VI NRS PAT	1422	PATOS	251080	1	2	2	2
1	SP	GVE VII SAI	1332	MAUA	352940		1	2	2
1	RJ			RIO DE JAN	330455	1	1	2	2
1	SP	GVE I CAPI	1331	SAO PAULO	355030	1	2	9	9
1	CE	1 CRES FOF	1519	FORTALEZA	230440	1			
1	SP	GVE XVII CA	1342	SUMARE	355240	1	2	2	2
1	SP	GVE XXIX S	1354	SAO JOSE D	354980	1	2	2	2
1	SP	GVE XXXI S	1353	SOROCABA	355220	1	9	9	9
1	PB	VIII NRS CA	1424	RIACHO DO	251280	1	2	2	2
1	SP	GVE I CAPI	1331	SAO PAULO	355030	1	9	9	2
1	BA	NUCLEO RE	1380	SALVADOR	292740	1	2	2	2
1	SP	GVE XVII CA	1342	JUNDIAI	352590	9		2	
1	SP	GVE X OSA	1335	EMBU DAS	351500	1	1	2	2
1	SP	GVE X OSA	1335	BARUERI	350570	1			
1	SP	GVE I CAPI	1331	SAO PAULO	355030		1	2	2
1	PE	002	1507	LAGOA DO	260850	1		2	2
1	AP			MACAPA	160030	1		2	2
1	TO			PALMAS	172100	1		2	2
1	RS	001 CRS	1592	NOVO HAM	431340	1	2	2	2
1	SP	GVE VII SAI	1332	MAUA	352940	1	2	2	2
1	SP	GVE XXIX S	1354	SAO JOSE D	354980	1		2	2
1	RJ			TERESOPO	330580	2		2	2
1	SP	GVE X OSA	1335	COTIA	351300	1	9	9	9
1	SP	GVE I CAPI	1331	SAO PAULO	355030	1			
1	RJ			RIO DE JAN	330455	1		2	2
1	SP	GVE I CAPI	1331	SAO PAULO	355030	1	2	2	9
1	MT	CUIABA	1578	VARZEA GI	510840	1	1	9	2
1	RJ			RIO DE JAN	330455		2	2	2
1	SP	GVE XXIX S	1354	ORINDIUVA	353420	1		2	2
1	SP	GVE I CAPI	1331	SAO PAULO	355030			2	2
1	RJ			RIO DE JAN	330455	1		2	
1	AM	ENTORNO	5584	MANAUS	130260	1	2	2	2
1	PA	12 REGION	1495	RIO MARIA	150616	1	2	2	2
1	RS	002 CRS	1607	CACHOEIRI	430310	1	2	2	9
1	AP			MACAPA	160030	1	1	2	2
1	RJ			CAMPOS D	330100	1		2	2
1	RJ			TRES RIOS	330600		2	2	2
1	PA	01 REGION	1484	ANANINDE	150080	1			2
1	CE	1 CRES FOF	1519	FORTALEZA	230440	1	9	2	
1	GO	SUDOESTE	1799	ITARUMA	521130	1	2	2	2
1	BA	NUCLEO RE	1403	JABORAND	291735	1			
1	PR	12RS UMU	1366	UMUARAN	412810	1	2	2	2
1	SP	GVE X OSA	1335	OSASCO	353440	1			
1	RJ			SAO JOAO	330510	1		9	9
1	SC	ITAJAI	1550	ITAJAI	420820	1	2	2	2
1	SP	GVE XVII CA	1342	PAULINIA	353650	1	2	2	2
1	PA	11 REGION	1494	MARABA	150420	1			2

1	PA	05 REGION	1488	IPIXUNA D	150345	1	2	2	2
1	GO	SUDOESTE	1799	RIO VERDE	521880				
1	SC	JOINVILLE	1565	SAO JOAO	421635	1	1	2	2
1	RJ			RIO DE JAN	330455	1		2	2
1	SP	GVE I CAPI	1331	SAO PAUL	355030	1	2	2	2
1	AM	ENTORNO	5584	MANAUS	130260	1		2	2
1	SP	GVE I CAPI	1331	SAO PAUL	355030		1	2	9
1	RJ			RIO DE JAN	330455	1		2	2
1	SP	GVE VIII M	1333	FERRAZ DE	351570	1	2	2	9
1	AM	ENTORNO	5584	MANAUS	130260	1		9	1
1	RJ			RIO DE JAN	330455	1		2	2
1	SP	GVE I CAPI	1331	SAO PAUL	355030	1	2	2	2
1	AM	TRIANGUL	5587	JURUA	130220	1			
1	SP	GVE VII SAI	1332	DIADEMA	351380	1	1	2	2
1	PB	I NRS JOAC	1377	JOAO PESS	250750	1	2	2	2
1	SP	GVE X OSA	1335	BARUERI	350570	1	2	2	9
1	RJ			RIO DE JAN	330455	1		2	2
1	CE	11 CRES SC	1521	MORAUJO	230880			2	
1	SP	GVE XXVIII	1576	CARAGUAT	351050	1	1	9	9
1	SP	GVE XVII C	1342	JUNDIAI	352590				
1	SC	VIDEIRA	1546	VIDEIRA	421930	1	2	2	2
1	SP	GVE XXIX S	1354	SAO JOSE D	354980	1	1	2	2
1	SP	GVE I CAPI	1331	SAO PAUL	355030		1	2	2
1	BA	NUCLEO RI	1380	SALVADOR	292740	1	2	2	2
1	PE	001	1497	RECIFE	261160	2	2	2	2
1	SP	GVE XXIX S	1354	SAO JOSE D	354980	1		2	2
1	MA	REGIONAL	1441	BALSAS	210140	1	1	2	2
1	DF			TAGUATIN	530180	1		2	9
1	SP	GVE XXIX S	1354	SAO JOSE D	354980	1		2	2
1	PR	16RS APUC	1370	ARAPONG	410150	1	1	2	2
1	SP	GVE I CAPI	1331	SAO PAUL	355030	1	2	2	2
1	RJ			RIO DAS O	330452	1		2	9
1	SP	GVE I CAPI	1331	SAO PAUL	355030	1	2	9	9
1	SP	GVE XXXI S	1353	ALUMINIO	350115	1	9	9	9
1	SC	CHAPECO	1553	CHAPECO	420420	1	2	2	2
1	AM	ENTORNO	5584	MANAUS	130260	1			
1	SP	GVE I CAPI	1331	SAO PAUL	355030	1	2	2	9
1	SP	GVE I CAPI	1331	SAO PAUL	355030	1	2	2	2
1	PB	I NRS JOAC	1377	JOAO PESS	250750	1	2	2	2
1	SP	GVE XVII C	1342	CAMPINAS	350950	1	2	2	2
1	BA	NUCLEO RI	1385	GANDU	291120	1	2	2	2
1	DF			CEILANDIA	530040	1	1	2	9
1	RS	016 CRS	1610	LAJEADO	431140	1	2	2	2
1	SP	GVE XXVII	1351	SAO JOSE D	354990	1	2	2	
1	RJ			RIO DE JAN	330455	1			
1	SP	GVE I CAPI	1331	SAO PAUL	355030	1	2	2	2
1	SP	GVE I CAPI	1331	SAO PAUL	355030	1	2	2	2

1	PI	4 GRS TERE	1883	TERESINA	221100	1			
1	MT	CUIABA	1578	CUIABA	510340	1		2	2
1	SP	GVE XII AR/	1337	TABATING/	355270	1	2	2	2
1	RS	016 CRS	1610	BOQUEIRA	430245			2	2
1	SP	GVE XXIX S	1354	CATANDU\	351110	1	1	2	2
1	MG	CORONEL F	1468	SANTANA I	315895	1	2	2	2
1	SP	GVE I CAPI	1331	SAO PAULC	355030	1	2	2	2
1	SP	GVE XIX M/	1344	MARILIA	352900	1	2	2	2
1	PB	VII NRS PIA	1423	OLHO D'AG	251040	1	2	2	2
1	MA	REGI0NAL	1430	SAO JOSE D	211120	1	2	2	2
1	PI	4 GRS TERE	1883	TERESINA	221100	1			
1	RS	014 CRS	1602	SANTA ROS	431720	3	1	2	2
1	RN	VII URSAP	1416	NATAL	240810	1		2	2
1	RN	II URSAP	1412	MOSSORO	240800	1		2	2
1	SP	GVE I CAPI	1331	SAO PAULC	355030	1			
1	RS	018 CRS	1605	TERRA DE /	432143	1	1	2	2
1	PR	02RS METF	1356	ALMIRANT	410040	1	2	2	2
1	SP	GVE I CAPI	1331	SAO PAULC	355030	1	1	2	9
1	MA	REGIONAL	1447	CAXIAS	210300	2	1	2	2
1	MG	JUIZ DE FO	1452	JUIZ DE FO	313670		9	9	9
1	GO	PIRINEUS	1792	ANAPOLIS	520110	1	1		
1	AM	ENTORNO	5584	MANAUS	130260	1			
1	PA	12 REGION	1495	REDENCAC	150613	1	2	2	2
1	MG	BELO HORI	1449	BELO HORI	310620	1			
1	SP	GVE XX PIR	1345	PIRACICAB	353870	1	1	2	2
1	MT	CUIABA	1578	CUIABA	510340	1		2	2
1	CE	11 CRES SC	1521	SOBRAL	231290	1	1	2	2
1	GO	CENTRAL	1779	NEROPOLIS	521450	1			
1	SP	GVE XVIII F	1343	ITUVERAV/	352410		2	1	2
1	AM	ENTORNO	5584	MANAQUI	130255	2			
1	BA	NUCLEO RI	1385	JEQUIE	291800	1			
1	PB	XII NRS ITA	1428	CALDAS BR	250380	1	1	2	2
1	RJ			MACUCO	330245	1	9	9	9
1	DF			CEILANDIA	530040	1	2	2	2
1	GO	CENTRO SU	1781	APARECID/	520140	1	2	2	2
1	CE	1 CRES FOF	1519	FORTALEZ/	230440	1			
1	PR	17RS LOND	1371	LONDRINA	411370	1		2	2
1	CE	6 CRES ITA	1515	ITAPIPOCA	230640	1	9	9	9
1	RN	VII URSAP	1416	NATAL	240810	1	2	2	2
1	SP	GVE VII SAI	1332	SAO BERN/	354870	1		2	2
1	RJ			RIO DE JAN	330455	1			
1	GO	ENTORNO	1782	VALPARAIS	522185	1		2	2
1	RJ			RIO DE JAN	330455	1			
1	RJ			RIO DE JAN	330455	1	9	2	9
1	SP	GVE XX PIR	1345	CONCHAL	351220	1	1	2	9
1	SP	GVE I CAPI	1331	SAO PAULC	355030	1		2	2
1	SP	GVE XXVI S	1350	ESPIRITO S	351518	1	2	2	2

1	MT	TANGARA	1585	TANGARA	510795	1		2	2
1	DF			GAMA	530060	1		2	9
1	AM	ENTORNO	5584	MANAUS	130260	1	2	2	2
1	SP	GVE I CAPI	1331	SAO PAUL	355030	1		2	9
1	RJ			RIO DE JAN	330455	1		2	2
1	DF			CEILANDIA	530040	1	2	2	2
1	SP	GVE VIII M	1333	GUARULH	351880	1			
1	SP	GVE XXIX S	1354	TANABI	355340	1		2	2
1	AL	1 MICROR	1533	SATUBA	270890	1	1	2	2
1	SP	GVE IX FRA	1334	CAIEIRAS	350900	1	2	2	9
1	RS	002 CRS	1607	CAMAQUA	430350	3			2
1	RJ			SAO JOAO	330510	1			
1	MG	DIVINOPOI	1472	ITAUNA	313380	1	2	2	2
1	SP	GVE I CAPI	1331	SAO PAUL	355030	1	2	2	2
1	RR			CANTA	140017	2		2	9
1	GO	CENTRAL	1779	GOIANIA	520870	1	2	2	2
1	PA	09 REGION	1492	JURUTI	150390	1		2	2
1	MG	TEOFILO O	1465	TEOFILO O	316860	1	1	2	2
1	MS	EX ARS DE	1975	CAMPO GF	500270	1		2	2
1	PA	09 REGION	1492	SANTAREN	150680	1		2	2
1	PR	01RS PARA	1355	PARANAG	411820	1			
1	ES	SAO MATE	1508	JAGUARE	320305	1	1	2	2
1	RJ			RIO DAS O	330452	1	2	9	9
1	SP	GVE I CAPI	1331	SAO PAUL	355030	1			
1	RJ			RIO DE JAN	330455	1	9	9	9
1	RJ			RIO DAS O	330452	1			
1	BA	NUCLEO RI	1380	SALVADOR	292740	1	2	2	2
1	SP	GVE XXXIII	1352	CRUZEIRO	351340	1	2	2	2
1	SP	GVE VIII M	1333	GUARULH	351880	1	2	2	2
1	RJ			NOVA IGU	330350	1			
1	SP	GVE I CAPI	1331	SAO PAUL	355030	1	2	2	2
1	AL	1 MICROR	1533	MACEIO	270430	1	1	2	9
1	MT	DIAMANTI	1586	NOBRES	510590			9	9
1	CE	3 CRES MA	1513	MARANGU	230770	1	9	2	
1	BA	NUCLEO RI	1398	TANQUE N	293105	2		2	2
1	GO	SUDOESTE	1799	RIO VERDE	521880	1	2	2	2
1	MT	TANGARA	1585	TANGARA	510795			9	9
1	GO	ESTRADA D	1802	CATALAO	520510	1	1	2	2
1	SP	GVE I CAPI	1331	SAO PAUL	355030			2	
1	SP	GVE I CAPI	1331	SAO PAUL	355030	1	1	2	2
1	SE	REGIONAL	2056	ARACAJU	280030	1	2	2	2
1	MT	CUIABA	1578	CUIABA	510340	1	1	9	9
1	RJ			SAO PEDR	330520	1		2	2
1	SP	GVE XX PIR	1345	RIO CLARO	354390	1	2	2	2
1	SP	GVE XXV S	1349	SANTOS	354850	1			9
1	SP	GVE I CAPI	1331	SAO PAUL	355030	1	2		2
1	SP	GVE I CAPI	1331	SAO PAUL	355030		2	2	2

1	PE	001	1497	RECIFE	261160	1	2	2	2
1	SP	GVE X OSA	1335	OSASCO	353440	1	2	2	2
1	MG	JUIZ DE FO	1452	JUIZ DE FO	313670	1	2	2	2
1	MT	CUIABA	1578	VARZEA GF	510840	1		2	2
1	RJ			ITAPERUNA	330220	1	1	2	2
1	PA	06 REGION	1489	BARCAREN	150130	1		2	2
1	RJ			RIO DE JAN	330455	1			
1	SP	GVE I CAPI	1331	SAO PAULC	355030	1	9	9	9
1	MT	CUIABA	1578	CUIABA	510340	1		2	2
1	CE	3 CRES MA	1513	MARACAN	230765	1	2	2	9
1	RJ			RIO CLARO	330440	1	9		9
1	DF			VICENTE PI	539934	1		9	9
1	RJ			RIO DE JAN	330455	1		2	2
1	GO	CENTRAL	1779	GOIANIA	520870	1			
1	AM	ENTORNO	5584	MANAUS	130260	1	1	2	2
1	SP	GVE I CAPI	1331	SAO PAULC	355030	1	2	2	2
1	RS	001 CRS	1592	SAO LEOPC	431870				
1	RJ			PORCIUNC	330410	1	1	2	2
1	BA	NUCLEO RE	1382	RIBEIRA DC	292660			2	2
1	SP	GVE X OSA	1335	CARAPICUI	351060	1	2	2	9
1	SP	GVE XXIX S	1354	MONTE AP	353140	1		2	2
1	PR	12RS UMU	1366	UMUARAN	412810	1	2	2	2
1	SP	GVE XVII CA	1342	HORTOLAN	351907	1	2		2
1	TO			LAGOA DA	171190	2			
1	SP	GVE VIII M	1333	MOGI DAS	353060	1	2	2	2
1	SP	GVE I CAPI	1331	SAO PAULC	355030	1	2	2	2
1	SP	GVE I CAPI	1331	SAO PAULC	355030	1	2	2	2
1	RJ			RIO DE JAN	330455	1		9	9
1	PI	4 GRS TERE	1883	TERESINA	221100	1			
1	BA	NUCLEO RE	1385	ITAJU DO C	291540		9	9	9
1	RS	002 CRS	1607	PORTO ALE	431490	1	1	2	2
1	SP	GVE I CAPI	1331	SAO PAULC	355030	1	2	2	2
1	SP	GVE X OSA	1335	BARUERI	350570	1	2		2
1	PE	010	1505	AFOGADOS	260010	1	9	9	9
1	AM	ENTORNO	5584	IRANDUBA	130185	2			
1	AM	ENTORNO	5584	MANAUS	130260	1		2	2
1	RJ			BELFORD R	330045	1	9	9	2
1	RJ			RIO DE JAN	330455	1		2	2
1	MG	CORONEL F	1468	MESQUITA	314170	2	9	9	9
1	CE	22 CRES CA	5596	PACAJUS	230960				
1	RJ			TRES RIOS	330600	1	2	9	9
1	GO	ENTORNO	1782	AGUAS LIN	520025				
1	DF			CEILANDIA	530040	1		2	2
1	BA	NUCLEO RE	1381	TERRA NOV	293170		9	9	9
1	PI	11 GRS SAC	1890	SAO JOAO	221000	2			
1	RJ			DUQUE DE	330170		2	2	2
1	SP	GVE XVII CA	1342	HORTOLAN	351907	1	2	2	2

1	SP	GVE I CAPI	1331	SAO PAULO	355030	1	2		2
1	SP	GVE XXV SA	1349	CUBATAO	351350	1	2	2	2
1	SC	TUBARAO	1555	CAPIVARI D	420395		2	2	2
1	MT	CUIABA	1578	CUIABA	510340			9	9
1	RJ			RIO DE JAN	330455	1		2	2
1	RJ			NOVA IGUA	330350	1			
1	DF			LAGO NOR	530080	1	2	2	2
1	PA	09 REGION	1492	TERRA SAN	150797	1	1	2	2
1	SP	GVE VII SA	1332	SANTO AN	354780	1	9	2	2
1	MG	BELO HORI	1449	BETIM	310670	1	9	9	9
1	AL	12 MICROR	1544	COLONIA L	270210	2	1	2	2
1	RS	016 CRS	1610	LAJEADO	431140	1	1	2	1
1	SP	GVE X OSA	1335	OSASCO	353440	1	1	2	2
1	AM	ENTORNO	5584	MANAUS	130260	1		2	2
1	CE	1 CRES FOR	1519	FORTALEZA	230440	1	9	2	9
1	RS	001 CRS	1592	CAMPO BC	430390			2	2
1	PR	02RS METR	1356	CURITIBA	410690	1	2	2	2
1	RJ			RIO DE JAN	330455	1		2	2
1	SP	GVE I CAPI	1331	SAO PAULO	355030				
1	BA	NUCLEO RE	1380	SALVADOR	292740	1		2	
1	SP	GVE X OSA	1335	COTIA	351300		9	9	9
1	SP	GVE I CAPI	1331	SAO PAULO	355030	1	2	2	2
1	PR	02RS METR	1356	PIRAQUAR	411950	1	2	2	2
1	SP	GVE I CAPI	1331	SAO PAULO	355030		2	2	2
1	AP			SERRA DO	160005	1	1	2	2
1	SP	GVE X OSA	1335	EMBU DAS	351500	1	2	2	9
1	DF			AGUAS CLA	539901	1		2	9
1	SP	GVE XVII CA	1342	CAMPINAS	350950	1		2	2
1	SC	JARAGUA D	1567	JARAGUA D	420890	1		2	2
1	SP	GVE I CAPI	1331	SAO PAULO	355030	1	2	2	2
1	GO	CENTRAL	1779	GOIANIRA	520880	1	2	2	2
1	SP	GVE XXIX S	1354	MIRASSOL	353030	1		2	2
1	SC	CHAPECO	1553	SAO BERNA	421575	1	2	1	1
1	RJ			RESENDE	330420	1		2	2
1	MS	EX ARS DE	1975	CAMPO GR	500270	1	2	2	2
1	RS	002 CRS	1607	PORTO ALE	431490		2	2	9
1	MS	EX ARS DE	1975	CAMPO GR	500270	1	2	2	2
1	SP	GVE I CAPI	1331	SAO PAULO	355030	1		2	2
1	SP	GVE I CAPI	1331	SAO PAULO	355030		1	2	2
1	RJ			CAMPOS D	330100	3			
1	CE	3 CRES MA	1513	MARACAN	230765	1	9	2	9
1	SP	GVE XII AR	1337	DESCALVA	351370	1	2	2	2
1	SP	GVE XXIV R	1348	SAO SIMAO	355090	1	2	2	2
1	AM	ALTO SOLI	5582	TONANTIN	130423	1	1	2	2
1	SP	GVE VII SA	1332	SAO CAETA	354880	1	2	2	9
1	SP	GVE X OSA	1335	BARUERI	350570	1	2	2	1
1	BA	NUCLEO RE	1382	CATU	290750		9	9	9

1	SP	GVE I CAPI	1331	SAO PAUL(	355030	1	2	2	2
1	RS	012 CRS	1601	SANTO AN	431750	1	2	2	2
1	SP	GVE VII SA	1332	SAO BERN/	354870	1		2	2
1	PR	10RS CASC	1364	CASCAVEL	410480	1		9	9
1	GO	PIRINEUS	1792	GOIANAPC	520840	1			
1	AL	1 MICRORI	1533	MACEIO	270430	1	1	2	2
1	GO	CENTRAL	1779	GOIANIA	520870	1		2	2
1	MG	MANHUAC	1454	DIVINO	312200		2	2	2
1	PR	03RS PONT	1357	CASTRO	410490		2	2	2
1	SP	GVE VIII M	1333	POA	353980	1	2	2	2
1	SP	GVE VIII M	1333	GUARULH(	351880	1	2	2	2
1	PB	III NRS CAI	1421	LAGOA SE(	250830	1			
1	DF			SAMAMBA	530140	1			
1	RJ			TERESOPO	330580	1	2	2	2
1	SP	GVE I CAPI	1331	SAO PAUL(	355030	1	2	2	2
1	PR	02RS METF	1356	CURITIBA	410690	1	2	2	2
1	SP	GVE XXV S/	1349	SANTOS	354850	1	9	9	9
1	RJ			RIO DE JAN	330455	1	1	2	9
1	PE	001	1497	VITORIA DI	261640	1	2	2	2
1	BA	NUCLEO RI	1382	ALAGOINH	290070		2	2	2
1	MG	GOVERNAI	1471	SARDOA	316550	1		2	9
1	BA	NUCLEO RI	1380	SALVADOR	292740		9	9	9
1	BA	NUCLEO RI	1381	FEIRA DE S	291080		9	9	9
1	GO	CENTRAL	1779	GOIANIA	520870	1	2	2	2
1	MT	CUIABA	1578	CUIABA	510340	1		2	2
1	SP	GVE X OSA	1335	ITAPECERI(	352220	1	9	2	2
1	MG	BELO HORI	1449	CONTAGEI	311860	1		2	2
1	RJ			RIO DE JAN	330455	1		9	9
1	RJ			NITEROI	330330	1	1	2	2
1	SP	GVE X OSA	1335	CARAPICUI	351060			2	2
1	RJ			NOVA IGU/	330350	1	2	2	2
1	SP	GVE XI AR/	1336	BIRIGUI	350650	1	2	2	2
1	AL	11 MICROI	1543	SAO JOSE I	270830	1	9	2	2
1	SP	GVE XXII PI	1573	PRESIDENT	354150	1	1	2	2
1	AL	1 MICRORI	1533	MACEIO	270430	1	1	2	2
1	RJ			RIO DE JAN	330455	1		2	2
1	BA	NUCLEO RI	1381	ARACI	290210		9	9	9
1	SP	GVE XVIII F	1343	SAO JOAQU	354940	1		2	2
1	PE	006	1501	CUSTODIA	260510	1	2	2	2
1	RS	016 CRS	1610	TAQUARI	432130	1	2	2	2
1	SP	GVE X OSA	1335	CARAPICUI	351060	1	9	9	9
1	AM	TRIANGUL(	5587	TEFE	130420	1			
1	MA	REGIONAL	1446	BACABAL	210120	1		2	2
1	MG	BELO HORI	1449	LAGOA SAI	313760	1	9	2	9
1	RN	I URSAP	1411	SERRINHA	241350	2	2	2	9
1	SP	GVE I CAPI	1331	SAO PAUL(	355030	1	2	9	2
1	DF			CEILANDIA	530040	1	1	2	9

1	CE	1 CRES FOF	1519	FORTALEZ/	230440	1	9	2	
1	SP	GVE X OSA	1335	OSASCO	353440	1	2	2	9
1	GO	SUL	1801	GOIATUBA	520910	1			
1	MG	GOVERNAI	1471	GOVERNAI	312770	1		2	2
1	RJ			RIO DE JAN	330455	1	1	2	2
1	PR	02RS METF	1356	SAO JOSE I	412550	1	2	2	9
1	MT	CUIABA	1578	CUIABA	510340	1		2	2
1	SP	GVE I CAPI	1331	SAO PAUL(	355030		2	2	2
1	SP	GVE VII SAI	1332	SANTO AN	354780	1	2	2	2
1	PR	20RS TOLEI	1374	TOLEDO	412770	1		2	2
1	BA	NUCLEO RI	1380	SALVADOR	292740	1	9	9	9
1	PA	01 REGION	1484	BELEM	150140	1		2	2
1	BA	NUCLEO RI	1380	SALVADOR	292740		9	9	9
1	ES	METROPOI	1510	FUNDAO	320220	2	1	2	2
1	RJ			CAMPOS D	330100	1	2	2	2
1	SP	GVE I CAPI	1331	SAO PAUL(	355030	1		2	2
1	PA	11 REGION	1494	NOVO REP/	150506	1	2	2	2
1	PR	10RS CASC	1364	CASCAVEL	410480	1	2	2	9
1	BA	NUCLEO RI	1394	IRECE	291460	9		2	9
1	PA	11 REGION	1494	PARAUAPE	150553	1	2	2	2
1	RJ			RIO DE JAN	330455	1		2	2
1	BA	NUCLEO RI	1380	SALVADOR	292740	1	2	2	
1	CE	13 CRES TI/	1523	VICOSA DC	231410	2	2	2	2
1	DF	SUPERINTE	6262	SOBRADIN	530170	1			
1	PR	17RS LOND	1371	LONDRINA	411370	1	2	2	2
1	MT	RONDONO	1579	CAMPO VE	510267	1	2	2	2
1	GO	SAO PATRI	1791	RIALMA	521860	1	9	9	9
1	SP	GVE I CAPI	1331	SAO PAUL(	355030	1	2	2	2
1	BA	NUCLEO RI	1380	SALVADOR	292740	1	9	9	9
1	SP	GVE X OSA	1335	EMBU DAS	351500	1	2	2	2
1	SP	GVE I CAPI	1331	SAO PAUL(	355030	1	9	9	9
1	AM	RIO NEGRC	5588	MANACAP	130250	2		2	2
1	SP	GVE XXIV F	1348	BARRINHA	350560	1	2	2	2
1	RJ			RIO DE JAN	330455	1	2	9	9
1	RJ			RIO DE JAN	330455	1	2	2	2
1	SP	GVE VIII M	1333	FERRAZ DE	351570	1	2	2	2
1	GO	CENTRAL	1779	GOIANIA	520870	1	2	2	2
1	PI	4 GRS TERE	1883	TERESINA	221100	1	2	2	2
1	SC	JOINVILLE	1565	JOINVILLE	420910	1	1	2	2
1	PA	12 REGION	1495	REDENCAC	150613	1	2	2	2
1	SP	GVE XXIV F	1348	RIBEIRAO F	354340	1	1	2	2
1	MS	EX ARS DE	1975	CAMPO GF	500270		2	2	2
1	RJ			CAMPOS D	330100	1	2	2	2
1	SP	GVE I CAPI	1331	SAO PAUL(	355030	1	9	9	9
1	MT	JUINA	1582	ARIPUANA	510140	2	1	2	2
1	PR	02RS METF	1356	CURITIBA	410690	1	2	2	9
1	MA	REGIONAL	1438	CANDIDO I	210260	1		2	2

1	MT	CUIABA	1578	VARZEA GI	510840	1	2	2	2
1	RS	002 CRS	1607	ALVORADA	430060	1	2	2	2
1	BA	NUCLEO RI	1382	ALAGOINH	290070	1			2
1	BA	NUCLEO RI	1380	SALVADOR	292740		9	9	9
1	AM	ENTORNO	5584	MANAUS	130260	1	2	2	2
1	PE	003	1498	AMARAJI	260090	2		9	9
1	SP	GVE XXV S/	1349	SAO VICEN	355100	1	2	2	2
1	RJ			RIO DE JAN	330455			2	2
1	SP	GVE XXIX S	1354	SAO JOSE I	354980	1		2	2
1	SP	GVE I CAPI	1331	SAO PAULC	355030	1	2	2	2
1	SP	GVE I CAPI	1331	SAO PAULC	355030	1	9	2	9
1	SP	GVE I CAPI	1331	SAO PAULC	355030	1	2	2	2
1	AM	BAIXO AM/	5589	BOA VISTA	130068	1	2		2
1	RJ			NOVA IGU/	330350	1			
1	MA	REGI0NAL	1430	SAO LUIS	211130	1	1	2	2
1	RJ			RIO DE JAN	330455	1	2	2	9
1	SC	JOINVILLE	1565	BARRA VEL	420210	1	1	2	
1	GO	CENTRAL	1779	GOIANIA	520870	1			
1	RS	017 CRS	1604	IJUI	431020	1	2	2	2
1	RJ			BELFORD R	330045	1	9	9	9
1	PI	4 GRS TERE	1883	TERESINA	221100		2	2	9
1	PI			MIGUEL AL	220620	1			
1	SP	GVE VIII M	1333	FERRAZ DE	351570	1		9	2
1	MG	BARBACEN	1448	CONSELHE	311830	1		2	2
1	PI			SANTA LUZ	220930	2		2	9
1	BA	NUCLEO RI	1380	SALVADOR	292740	1	9	2	2
1	SP	GVE I CAPI	1331	SAO PAULC	355030	1		2	2
1	PR	10RS CASC	1364	CASCAVEL	410480	1	2	2	2
1	AM	ENTORNO	5584	MANAUS	130260	1		2	
1	SP	GVE XVII C/	1342	SANTA BAI	354580	1	2	2	2
1	BA	NUCLEO RI	1381	FEIRA DE S.	291080	1			
1	RJ			RIO DE JAN	330455	1	2	2	2
1	AM	ENTORNO	5584	MANAUS	130260	1			
1	BA	NUCLEO RI	1398	VITORIA D/	293330		2		2
1	RJ			RIO DE JAN	330455	1		2	2
1	BA	NUCLEO RI	1380	SALVADOR	292740		9	9	9
1	SP	GVE I CAPI	1331	SAO PAULC	355030	1	2	2	9
1	SP	GVE I CAPI	1331	SAO PAULC	355030	1			
1	BA	NUCLEO RI	1380	SALVADOR	292740	1		2	
1	RJ			SAO GONC	330490	1	2	9	9
1	RJ			RIO DE JAN	330455	1		9	9
1	RJ			NOVA IGU/	330350	1			
1	PR	19RS JACAI	1373	JOAQUIM	411280	1	2	2	
1	SP	GVE IX FRA	1334	FRANCO D/	351640	1	2	2	9
1	CE	5 CRES CAI	1514	BOA VIAGE	230240	1	2		2
1	MG	GOVERNAI	1471	GOVERNAI	312770	1	9	9	9
1	RN	VII URSAP	1416	NATAL	240810	1	2	2	2

1	SE	REGIONAL	2057	ESTANCIA	280210	1	2	2	2
1	SP	GVE IX FRA	1334	CAJAMAR	350920	1	2	2	2
1	SP	GVE I CAPI	1331	SAO PAULO	355030	1	1	2	9
1	RJ			NITEROI	330330	1		9	9
1	RJ			RIO DE JAN	330455	1		2	2
1	SP	GVE VIII M	1333	MOGI DAS	353060	9	1	2	9
1	GO	CENTRO SU	1781	PIRACANJU	521710	2	2	2	2
1	AM	ENTORNO	5584	MANAUS	130260	1	1	2	2
1	CE	1 CRES FOR	1519	FORTALEZA	230440	1	9	2	9
1	RJ			RIO DE JAN	330455	1			
1	SP	GVE VII SA	1332	SANTO AN	354780	1			
1	CE	1 CRES FOR	1519	FORTALEZA	230440	1		2	9
1	PR	20RS TOLE	1374	GUAIRA	410880	1			
1	RJ			RIO DE JAN	330455	1		9	9
1	BA	NUCLEO RE	1380	SALVADOR	292740		9	9	9
1	SP	GVE I CAPI	1331	SAO PAULO	355030			2	9
1	MA	REGI0NAL	1430	SAO LUIS	211130	1	1	2	2
1	GO	CENTRAL	1779	GOIANIA	520870	1	2	2	2
1	RJ			RIO DE JAN	330455	1		2	9
1	AM	ENTORNO	5584	MANAUS	130260	1	1	2	2
1	RS	003 CRS	1593	PELOTAS	431440	1	2	2	2
1	SP	GVE XXIX S	1354	NOVO HOR	353350	1	2	2	2
1	SP	GVE XXVIII	1576	SAO SEBAS	355070	1	1	2	2
1	MG	POUSO ALE	1459	ITAJUBA	313240	2	2	2	2
1	SP	GVE X OSA	1335	OSASCO	353440	1		2	2
1	SP	GVE XXVII S	1351	SAO JOSE D	354990	1			
1	SP	GVE XX PIR	1345	RIO CLARO	354390	1	2	2	2
1	SP	GVE XVII CA	1342	VALINHOS	355620	1			
1	RJ			DUQUE DE	330170	1		2	2
1	DF			TAGUATIN	530180	1		2	2
1	RS	002 CRS	1607	CACHOEIRI	430310	1			
1	SP	GVE I CAPI	1331	SAO PAULO	355030	1	9	9	9
1	PE	001	1497	SAO LOURI	261370	2	1	2	2
1	AM	ENTORNO	5584	PRESIDENT	130353	1	2	2	2
1	SP	GVE I CAPI	1331	SAO PAULO	355030	1	2	2	2
1	CE	1 CRES FOR	1519	FORTALEZA	230440	1	9	2	2
1	RS	005 CRS	1608	BENTO GO	430210	1	9	9	9
1	SP	GVE XXV SA	1349	GUARUJA	351870	1		2	2
1	SP	GVE I CAPI	1331	SAO PAULO	355030	1		2	2
1	ES	METROPOL	1510	CARIACICA	320130	1	1	2	2
1	RJ			ANGRA DO	330010	3			9
1	MT	CUIABA	1578	CUIABA	510340	1	2	2	2
1	SP	GVE XXIX S	1354	SAO JOSE D	354980	1	1	2	2
1	AM	ENTORNO	5584	MANAUS	130260	1			
1	PE	003	1498	PRIMAVER	261140	2	1	2	2
1	BA	NUCLEO RE	1380	CAMACARI	290570		1	2	
1	PB	IX NRS CAJ	1425	CAJAZEIRA	250370	1	2	2	2

1	SP	GVE I CAPI	1331	SAO PAUL(	355030	1	9	9	9
1	PI	4 GRS TERE	1883	TERESINA	221100	1			
1	AP			MACAPA	160030	1	1	2	2
1	SP	GVE XVII C	1342	AMPARO	350190	1	2	2	2
1	SP	GVE I CAPI	1331	SAO PAUL(	355030	1	2		2
1	MT	CUIABA	1578	CUIABA	510340	1	2	2	2
1	SP	GVE I CAPI	1331	SAO PAUL(	355030		1	2	2
1	PA	01 REGION	1484	BENEVIDES	150150	1	2	2	2
1	RJ			RESENDE	330420	1			
1	MG	ITUIUTABA	1378	CAMPINA \	311110	1	2	2	2
1	SP	GVE I CAPI	1331	SAO PAUL(	355030	1	2	2	2
1	SP	GVE XIX M	1344	MARILIA	352900	1	2	2	2
1	PB	I NRS JOAC	1377	JOAO PESS	250750	9	9	9	9
1	RJ			NITEROI	330330	1		9	9
1	SP	GVE XVII C	1342	CAMPINAS	350950	1		2	2
1	RS	002 CRS	1607	PORTO ALE	431490	1	9	9	9
1	SP	GVE I CAPI	1331	SAO PAUL(	355030	1	2	2	2
1	RJ			MARICA	330270	1		9	2
1	SP	GVE I CAPI	1331	SAO PAUL(	355030	1	1		
1	RN	II URSAP	1412	MOSSORO	240800	1	2	2	2
1	BA	NUCLEO RI	1398	VITORIA D	293330	1			
1	SP	GVE X OSA	1335	ITAPEVI	352250	1	2		2
1	PR	02RS METF	1356	CURITIBA	410690	1	2	2	2
1	PR	17RS LONE	1371	TAMARAN	412667	3	2	2	2
1	PE	001	1497	OLINDA	260960	1	2	2	1
1	RJ			RIO DE JAN	330455	1	1	9	9
1	CE	20 CRES CF	1530	NOVA OLIN	230920	2	2	2	2
1	PA	09 REGION	1492	SANTAREM	150680	1	1	2	2
1	PR	02RS METF	1356	PIRAQUAR	411950	1		2	2
1	MG	DIVINOPOI	1472	PITANGUI	315140	1	2	2	2
1	SP	GVE XVII C	1342	SANTA BAI	354580	1	2	2	2
1	GO	CENTRAL	1779	GOIANIA	520870				
1	SP	GVE I CAPI	1331	SAO PAUL(	355030	1	2	2	2
1	SP	GVE XXIX S	1354	SAO JOSE I	354980	1		2	2
1	RJ			MESQUITA	330285	1	1	2	9
1	SP	GVE VII SAI	1332	SAO CAETA	354880	1	2	2	2
1	RJ			RIO DE JAN	330455	1		9	9
1	AL	12 MICROF	1544	COLONIA L	270210	2		2	2
1	PB	I NRS JOAC	1377	JOAO PESS	250750	1	2	2	2
1	SP	GVE IX FRA	1334	FRANCO D	351640	1	2	2	9
1	PB	XI NRS PRII	1427	PRINCESA I	251230	1	2	2	2
1	SC	FLORIANOI	1476	CANELINH	420370	3	9	9	2
1	RS	010 CRS	1599	ITAQUI	431060	1		2	2
1	MG	POUSO ALI	1459	ITAJUBA	313240	1	2	2	2
1	SP	GVE XXXI S	1353	CAPELA DC	351030	1	1	2	2
1	PR	15RS MARI	1369	MARINGA	411520	1		2	2
1	RJ			RIO DE JAN	330455	1		2	2

1	SP	GVE XXV SA	1349	GUARUJA	351870	1	2	2	2
1	MG	POUSO ALE	1459	ITAJUBA	313240	1	2	2	2
1	CE	1 CRES FOR	1519	FORTALEZA	230440	1		2	9
1	BA	NUCLEO RE	1380	SALVADOR	292740	1	1	2	2
1	SP	GVE XXV SA	1349	SAO VICEN	355100	1	9	9	9
1	RJ			RIO DE JAN	330455	1			
1	PR	02RS METR	1356	TIJUCAS DO	412760	1	2	2	2
1	PE	001	1497	CAMARAG	260345	1		2	2
1	GO	SAO PATRI	6252	JARAGUA	521180	1			
1	RJ			RIO DE JAN	330455			2	
1	RJ			CAMPOS D	330100	1	2	2	2
1	SP	GVE I CAPIT	1331	SAO PAULO	355030	1	2	2	9
1	SP	GVE XXIX S	1354	SAO JOSE D	354980	1		2	2
1	DF			TAGUATIN	530180	1	2	2	2
1	SP	GVE I CAPIT	1331	SAO PAULO	355030	1	2	2	2
1	MA	REGIONAL	1440	IMPERATRI	210530		1		2
1	PE	001	1497	RECIFE	261160	1			
1	MT	CUIABA	1578	CUIABA	510340	1	2	2	2
1	SP	GVE X OSA	1335	TABOAO D	355280	1	1	2	9
1	SP	GVE I CAPIT	1331	SAO PAULO	355030	1	2		2
1	SP	GVE XVII CA	1342	NOVA ODE	353340	1	2	2	2
1	PA	10 REGION	1493	MEDICILAN	150445	2	2	2	2
1	SP	GVE I CAPIT	1331	SAO PAULO	355030	1	2	2	2
1	SP	GVE I CAPIT	1331	SAO PAULO	355030	1	9	9	9
1	PA	11 REGION	1494	PARAUAPE	150553	1	2	2	2
1	SP	GVE XII ARA	1337	IBATE	351930	1	2	2	2
1	PB	I NRS JOAO	1377	JOAO PESS	250750	1		9	9
1	MG	UBERLAND	1462	UBERLAND	317020	1	2	2	2
1	GO	CENTRAL	1779	GOIANIA	520870	1			
1	SP	GVE VIII M	1333	SUZANO	355250	1	9	2	2
1	SP	GVE XII ARA	1337	PORTO FER	354070	1	2	2	2
1	MA	REGIONAL	1443	PRESIDENT	210910		1		2
1	PA	11 REGION	1494	BOM JESUS	150157	2	2	2	2
1	RN	II URSAP	1412	MOSSORO	240800	1	2	2	2
1	PR	02RS METR	1356	CURITIBA	410690	1	2	2	2
1	CE	3 CRES MA	1513	MARACAN	230765	1	1	2	
1	PE	001	1497	RECIFE	261160	1	2	2	2
1	MG	BELO HORI	1449	NOVA LIMA	314480	1	1	2	2
1	CE	1 CRES FOR	1519	FORTALEZA	230440	1	9	2	9
1	AL	1 MICRORR	1533	MACEIO	270430	1			9
1	SP	GVE I CAPIT	1331	SAO PAULO	355030		2	2	2
1	SP	GVE I CAPIT	1331	SAO PAULO	355030	1	1	2	9
1	PR	05RS GUAR	1359	GUARAPUA	410940				
1	SP	GVE XXIX S	1354	MACAUBA	352810	1	2	1	2
1	RJ			RIO DE JAN	330455	1	1	2	2
1	MG	PONTE NOV	1458	PONTE NOV	315210	1		2	2
1	PR	02RS METR	1356	CAMPINA (	410400	1	2	2	2

1	PB	III NRS CAN	1421	CAMPINA (	250400	1	2	2	2
1	SP	GVE XXIX S	1354	ITAJOBI	352190	1	2	2	2
1	SP	GVE I CAPI'	1331	SAO PAULC	355030	1	2	2	2
1	SP	GVE IX FRA	1334	FRANCO D/	351640	1	2	2	2
1	PE	001	1497	PAULISTA	261070	1		2	2
1	RJ			RIO DE JAN	330455	1	9	9	9
1	PA	04 REGION	1487	BRAGANC/	150170	1	1	2	2
1	RJ			RIO DE JAN	330455	1			
1	SP	GVE XVII C/	1342	ITUPEVA	352400	1	1	2	2
1	SP	GVE XXV S/	1349	SANTOS	354850	1	9		9
1	SP	GVE I CAPI'	1331	SAO PAULC	355030	1	2	2	2
1	SP	GVE VIII M(	1333	GUARULH(	351880	1	2	2	2
1	CE	6 CRES ITA	1515	ITAPIPOCA	230640	2	9	2	9
1	SP	GVE XXVI S	1350	MOCOCA	353050	1	2	2	2
1	RR			CARACARA	140020	1		2	2
1	SP	GVE I CAPI'	1331	SAO PAULC	355030	1	9	2	9
1	SP	GVE I CAPI'	1331	SAO PAULC	355030	1	1	9	9
1	SP	GVE I CAPI'	1331	SAO PAULC	355030	1	2	2	2
1	MG	UBERLAND	1462	MONTE AL	314280	1	2	2	2
1	SP	GVE I CAPI'	1331	SAO PAULC	355030	1	2	2	9
1	RN	II URSAP	1412	MOSSORO	240800	1		2	9
1	AM	ENTORNO	5584	MANAUS	130260	1	2	2	2
1	SP	GVE VIII M(	1333	MOGI DAS	353060	1		2	2
1	MT	CUIABA	1578	CUIABA	510340	1		2	2
1	RJ			RIO DE JAN	330455	9			
1	ES	CACHOEIR(	1511	ITAPEMIRII	320280	1	2	2	2
1	RJ			CAMPOS D	330100				
1	SP	GVE I CAPI'	1331	SAO PAULC	355030	1	2	2	2
1	SP	GVE XVII C/	1342	JUNDIAI	352590				
1	AL	8 MICRORI	1540	OLHO D'AG	270570	2	1	2	2
1	GO	NORTE	1788	SAO MIGU	522020	1	2	2	2
1	RJ			RIO DE JAN	330455	1		9	9
1	RJ			RIO DE JAN	330455	1			
1	MG	GOVERNAI	1471	GOVERNAI	312770	1		2	2
1	CE	6 CRES ITA	1515	UMIRIM	231375	1	2	2	9
1	SP	GVE I CAPI'	1331	SAO PAULC	355030	1	2	2	2
1	RJ			CAMPOS D	330100	1	1	2	2
1	AM	ENTORNO	5584	MANAUS	130260	1	1	2	2
1	AM	ENTORNO	5584	MANAUS	130260	1			
1	MG	BELO HORI	1449	BELO HORI	310620	1		2	2
1	PE	002	1507	CARPINA	260400	1	2	2	9
1	SP	GVE IX FRA	1334	CAIEIRAS	350900	1	2	2	9
1	PR	02RS METF	1356	COLOMBO	410580	1	2	2	2
1	GO	ENTORNO	1784	FORMOSA	520800	1			
1	PB	II NRS GUA	1420	CUITEGI	250520	1	1	2	2
1	PI	11 GRS SA(	1890	SAO JOAO	221000	1			
1	RJ			RIO DE JAN	330455	1	2	2	2

1	MS	NRS DE PO	1972	PARANHOS	500635	1	1	2	2
1	RJ			RIO DE JAN	330455	1		2	9
1	PI	4 GRS TERE	1883	TERESINA	221100	1		2	2
1	MG	POUSO ALE	1459	POCOS DE	315180	1		2	2
1	RJ			RIO DE JAN	330455	1	1	2	2
1	ES	METROPOL	1510	SERRA	320500	1	2	2	2
1	PA	12 REGION	1495	SANTA MA	150658	2		2	2
1	RS	002 CRS	1607	ALVORADA	430060	1	2	2	9
1	SP	GVE VII SA	1332	SAO BERNA	354870	1	1	2	2
1	ES	METROPOL	1510	VILA VELHA	320520	1	2	2	2
1	RO	VI GRS POR	1483	PORTO VEL	110020	1		2	2
1	RS	002 CRS	1607	CACHOEIRI	430310	1		2	2
1	MA	REGIONAL	1437	SATUBINHA	211172	2	2	2	2
1	PI	4 GRS TERE	1883	TERESINA	221100	1		2	2
1	RJ			ITAPERUNA	330220	1	1	2	2
1	CE	1 CRES FOR	1519	FORTALEZA	230440		9		9
1	AL	1 MICRORR	1533	MACEIO	270430	1	1	9	2
1	SP	GVE IX FRA	1334	FRANCISCO	351630	1	2	2	2
1	SP	GVE I CAPIT	1331	SAO PAULO	355030	1	2	2	2
1	PR	02RS METR	1356	CURITIBA	410690	1		2	2
1	RJ			BELFORD R	330045	1		2	2
1	RS	002 CRS	1607	PORTO ALE	431490	1	2	2	2
1	RJ			NOVA FRIB	330340		9	9	9
1	MG	BELO HORI	1449	BELO HORI	310620	1		2	2
1	SP	GVE I CAPIT	1331	SAO PAULO	355030	1	1	2	2
1	MG	BELO HORI	1449	BETIM	310670	1	9	9	9
1	MG	BELO HORI	1449	CONTAGEM	311860	1	9	9	9
1	BA	NUCLEO RE	1381	FEIRA DE S	291080	1			
1	GO	SUDOESTE	1799	RIO VERDE	521880	1			1
1	SP	GVE VIII M	1333	GUARULHO	351880	1	2	2	2
1	SP	GVE XXIX S	1354	SAO JOSE D	354980	1			2
1	RN	II URSAP	1412	MOSSORO	240800	1	2	2	2
1	SP	GVE XXVII S	1351	SAO JOSE D	354990	1		2	2
1	PE	001	1497	CABO DE S	260290				
1	DF			BRASILIA -	530005	1	2	2	2
1	SP	GVE XIX MA	1344	MARILIA	352900	1	2	2	2
1	AM	ENTORNO	5584	MANAUS	130260	1	9	9	9
1	PR	02RS METR	1356	CURITIBA	410690	1	2	2	9
1	SP	GVE I CAPIT	1331	SAO PAULO	355030	1	9	2	2
1	RS	004 CRS	1595	VILA NOVA	432345	1	2	2	2
1	MA	REGIONAL	1447	CAXIAS	210300		2	2	2
1	RJ			PETROPOLI	330390		2		2
1	BA	NUCLEO RE	1380	SALVADOR	292740	1	9	9	9
1	SC	CONCORDI	1560	CONCORDI	420430	1	2	2	2
1	RJ			RIO DE JAN	330455	1		2	
1	PA	12 REGION	1495	REDENCAO	150613	1	2	2	2
1	BA	NUCLEO RE	1380	SALVADOR	292740		2	2	2

1	SP	GVE I CAPI	1331	SAO PAUL	355030	1	9	9	9
1	PR	02RS METF	1356	CURITIBA	410690	1	2	2	9
1	CE	4 CRES BAT	1520	BATURITE	230210	2	9	2	9
1	RJ			SAO GONC	330490		1	2	
1	PA	01 REGION	1484	BELEM	150140	1	2	2	9
1	GO	CENTRAL	1779	GOIANIA	520870	1	2	2	2
1	BA	NUCLEO RI	1398	BRUMADO	290460				
1	AM	ENTORNO	5584	CAREIRO	130110	2	2	2	2
1	PR	15RS MARI	1369	SARANDI	412625	1	2	2	2
1	PA	06 REGION	1489	BARCAREN	150130		2	2	2
1	SP	GVE I CAPI	1331	SAO PAUL	355030	1	2	2	2
1	PE	002	1507	PAUDALHO	261060	1	9	9	9
1	RJ			RIO DE JAN	330455	1		9	9
1	PB	I NRS JOAC	1377	JOAO PESS	250750	1	2	2	2
1	SP	GVE XX PIR	1345	RIO CLARO	354390	1	1	2	2
1	PR	02RS METF	1356	CURITIBA	410690	1	2	2	2
1	SP	GVE XVII C	1342	MORUNGA	353200	1		2	9
1	GO	SUDOESTE	1800	MINEIROS	521310	1	2		2
1	TO			ARAGUAIN	170210	1	2	2	2
1	SP	GVE I CAPI	1331	SAO PAUL	355030	1		2	9
1	AM	ENTORNO	5584	MANAUS	130260	1	1	2	2
1	RJ			SAO JOAO	330510	1	1	2	2
1	SP	GVE I CAPI	1331	SAO PAUL	355030	1	2		2
1	RJ			ARRAIAL D	330025	1		2	2
1	SP	GVE VII SAI	1332	SAO BERN	354870	1	1	2	2
1	RS	002 CRS	1607	TAPES	432110		1		
1	MT	TANGARA	1585	NOVA OLIN	510623	1		9	9
1	SP	GVE XXV S	1349	CUBATAO	351350	1	1	2	2
1	MG	BELO HORI	1449	BELO HORI	310620	1	2	2	2
1	MG	POUSO ALI	1459	ITAJUBA	313240	1		2	2
1	PR	02RS METF	1356	COLOMBO	410580	1	1	2	2
1	SP	GVE I CAPI	1331	SAO PAUL	355030	1	2	2	2
1	PR	02RS METF	1356	CURITIBA	410690	1	2	2	2
1	SP	GVE VII SAI	1332	DIADEMA	351380	1	2	2	2
1	RJ			RIO DE JAN	330455	1	2	2	2
1	SP	GVE XXIX S	1354	SAO JOSE I	354980	1	2	2	2
1	DF			CEILANDIA	530040	1	9	2	9
1	RJ			RIO DE JAN	330455	1		9	9
1	SP	GVE I CAPI	1331	SAO PAUL	355030	1	1	2	9
1	PE	005	1500	LAGOA DO	260860	1	9	9	9
1	CE	11 CRES SC	1521	SOBRAL	231290				
1	AP			MACAPA	160030	1	1	2	2
1	CE	18 CRES IG	1528	MOMBACA	230850	1	2		
1	RJ			NOVA IGU	330350	1			
1	MA	REGI0NAL	1430	SAO LUIS	211130	1	2	2	2
1	MS	EX NRS DE	6256	CORUMBA	500320	1	2	2	2
1	GO	CENTRO SU	1781	APARECID	520140	1	2	2	2

1	PB	II NRS GUA	1420	BELEM	250190	1	2	2	2
1	PI	4 GRS TERE	1883	TERESINA	221100	1			
1	SP	GVE I CAPIT	1331	SAO PAULO	355030				
1	SP	GVE XXI PR	1346	SANDOVAL	354550	1	1	2	2
1	MT	CUIABA	1578	CUIABA	510340	1		2	2
1	SP	GVE I CAPIT	1331	SAO PAULO	355030	1	2	2	9
1	MG	TEOFILO OT	1465	NANUQUE	314430	1	1	9	9
1	PE	001	1497	JABOATAO	260790	1	2	2	2
1	BA	NUCLEO RE	1393	JAGUARAR	291770	1			
1	SP	GVE I CAPIT	1331	SAO PAULO	355030	1	2	2	2
1	RJ			BOM JESUS	330060	1			2
1	PB	III NRS CAM	1421	PUXINANA	251240	1	1	2	9
1	SP	GVE I CAPIT	1331	SAO PAULO	355030	1	2	2	2
1	SP	GVE I CAPIT	1331	SAO PAULO	355030	1	1	2	2
1	AL	1 MICRORI	1533	MACEIO	270430	1	1	9	9
1	RS	001 CRS	1592	PORTAO	431480		2	2	2
1	RJ			CAMPOS D	330100	1		2	2
1	BA	NUCLEO RE	1385	COARACI	290800		9	9	9
1	SP	GVE XXIV R	1348	RIBEIRAO P	354340	1	2	2	2
1	SP	GVE XXIV R	1348	RIBEIRAO P	354340	1	2	2	2
1	SP	GVE XXIV R	1348	RIBEIRAO P	354340	1	2		2
1	SP	GVE I CAPIT	1331	SAO PAULO	355030	1	2	2	9
1	RJ			RIO DE JAN	330455	1	1	2	2
1	PB	III NRS CAM	1421	CAMPINA G	250400		2	2	2
1	SP	GVE XVII CA	1342	CAMPINAS	350950	1	2	2	2
1	GO	ENTORNO	1782	VALPARAIS	522185	1		2	2
1	SP	GVE XXV SA	1349	GUARUJA	351870	1	1	2	2
1	SP	GVE I CAPIT	1331	SAO PAULO	355030		1	2	9
1	SP	GVE XVII CA	1342	CAMPINAS	350950	1	2	2	2
1	RJ			NOVA IGUA	330350	1			
1	SP	GVE I CAPIT	1331	SAO PAULO	355030				
1	SP	GVE VIII M	1333	MOGI DAS	353060	1	1	2	9
1	SP	GVE I CAPIT	1331	SAO PAULO	355030	1	9	2	2
1	SP	GVE XVII CA	1342	VARZEA PA	355650				
1	SP	GVE X OSA	1335	TABOAO D	355280	1	2	2	2
1	TO			JUARINA	171180	1	1	2	2
1	BA	NUCLEO RE	1380	SALVADOR	292740		9		9
1	SP	GVE XI ARA	1336	PENAPOLIS	353730	1	2	2	2
1	PE	001	1497	SAO LOURE	261370	1	2	2	2
1	MG	UBA	1464	UBA	316990	1	2	2	2
1	GO	CENTRAL	1779	GOIANIA	520870	1	9	2	2
1	RJ			RIO DE JAN	330455	1	1	2	2
1	PB	I NRS JOAO	1377	JOAO PESS	250750	1	2	2	2
1	BA	NUCLEO RE	1385	CANAVIEIR	290630	1	2	2	
1	BA	NUCLEO RE	1380	SALVADOR	292740		9	9	9
1	SP	GVE I CAPIT	1331	SAO PAULO	355030	1	2	2	2
1	SP	GVE VIII M	1333	SALESOPOI	354500	1	2	2	2

1	CE	1 CRES FOF	1519	FORTALEZ/	230440	1	9	2	9
1	MG	UNAI	1463	UNAI	317040	1	2	2	2
1	CE	1 CRES FOF	1519	FORTALEZ/	230440				
1	SP	GVE I CAPI	1331	SAO PAULC	355030		2	2	9
1	PR	03RS PONT	1357	CASTRO	410490	1	1	2	2
1	GO	SUDOESTE	1800	JATAI	521190				
1	GO	CENTRAL	1779	GOIANIA	520870	1	2	2	2
1	RO	VI GRS POF	1483	PORTO VEL	110020	1		2	2
1	SP	GVE XX PIR	1345	PIRACICAB	353870	1	2	2	2
1	RJ			NOVA IGU/	330350			9	9
1	MG	UBERLAND	1462	ROMARIA	315640	1		2	2
1	SP	GVE I CAPI	1331	SAO PAULC	355030	1		2	9
1	RJ			TERESOPO	330580	1		9	9
1	SP	GVE I CAPI	1331	SAO PAULC	355030	1		2	2
1	GO	CENTRO SL	1781	APARECID/	520140				
1	SP	GVE I CAPI	1331	SAO PAULC	355030	1		2	2
1	RS	005 CRS	1608	VACARIA	432250	1		2	2
1	RR			BOA VISTA	140010	1		2	2
1	GO	ENTORNO	1784	VILA BOA	522220	1		2	9
1	BA	NUCLEO RI	1380	CAMACARI	290570	1		2	2
1	SP	GVE VIII M	1333	GUARULHC	351880	1		2	2
1	RN	VII URSAP	1416	PARNAMIF	240325	1		2	9
1	PA	09 REGION	1492	RUROPOLIS	150619	2	1	2	2
1	MG	BELO HORI	1449	CONTAGEN	311860	1		9	9
1	SP	GVE XVII C/	1342	CAMPINAS	350950	1			2
1	MG	BELO HORI	1449	CONTAGEN	311860	1		9	9
1	SP	GVE I CAPI	1331	SAO PAULC	355030	1		2	2
1	SP	GVE IX FRA	1334	FRANCO D/	351640	1		2	2
1	ES	METROPOI	1510	CARIACICA	320130			2	9
1	SP	GVE XXVII S	1351	SAO JOSE D	354990	1		2	2
1	RJ			RIO DE JAN	330455	1		9	9
1	RJ			VOLTA REC	330630	1		9	9
1	DF			CEILANDIA	530040	1		2	9
1	RN	VII URSAP	1416	NATAL	240810	1		2	9
1	GO	CENTRAL	1779	GOIANIA	520870	1		2	2
1	RJ			SAO GONC	330490	1		2	2
1	GO	CENTRAL	1779	GOIANIA	520870	1			
1	RJ			RIO DE JAN	330455			2	
1	RJ			RIO DE JAN	330455	1			
1	PB	I NRS JOAC	1377	SOBRADO	251597	2		2	2
1	CE	3 CRES MA	1513	PACATUBA	230970	1		9	9
1	GO	CENTRAL	1779	GOIANIA	520870	1		2	2
1	CE	3 CRES MA	1513	MARACAN	230765	1		9	9
1	RS	002 CRS	1607	ALVORADA	430060				
1	MT	RONDONO	1579	PRIMAVER	510704	1			
1	ES	SAO MATE	1508	CONCEICA(	320160	2		2	2
1	MG	BELO HORI	1449	ESMERALD	312410	1		9	9

1	SP	GVE I CAPI	1331	SAO PAULC	355030	1		2	2
1	MG	DIVINOPOI	1472	MARTINHC	314050	1		2	2
1	RN	VII URSAP	1416	NATAL	240810	1		2	2
1	MG	GOVERNAD	1471	MANTENA	313960	1			
1	SP	GVE XXIV F	1348	RIBEIRAO F	354340	1		2	2
1	ES	CACHOEIR	1511	MARATAIZ	320332			2	9
1	MS	PARANAIB	1973	APARECID	500100	1		2	2
1	CE	2 CRES CAU	1512	ITAPAGE	230630	1		9	9
1	PR	13RS CIAN	1367	CIANORTE	410550	1		2	2
1	RS	002 CRS	1607	PORTO ALE	431490				
1	MS	EX ARS DE	1975	CAMPO GF	500270	1		1	2
1	PR	16RS APUC	1370	FAXINAL	410760	1		2	2
1	MT	JUARA	1588	PORTO DO	510680	1		2	2
1	SC	JOINVILLE	1565	BALNEARIC	420205	1		2	2
1	GO	CENTRO SU	1781	SENADOR (	522045	1		2	2
1	RS	006 CRS	1611	TAPEJARA	432090	2		2	2
1	SP	GVE I CAPI	1331	SAO PAULC	355030	1		2	2
1	SP	GVE XII AR	1337	AMERICO E	350170	1		2	2
1	RJ			RIO DE JAN	330455	1			
1	MA	REGIONAL	1443	PRESIDENT	210910	1		2	2
1	SP	GVE XVII C	1342	VALINHOS	355620	1		2	9
1	SP	GVE X OSA	1335	OSASCO	353440	1		2	2
1	PB	III NRS CAN	1421	SAO SEBAS	251510				
1	RJ			RIO DE JAN	330455	1		2	2
1	SP	GVE I CAPI	1331	SAO PAULC	355030	1		2	2
1	CE	1 CRES FOF	1519	FORTALEZ	230440	1		9	9
1	CE	1 CRES FOF	1519	FORTALEZ	230440	1		9	9
1	PA	10 REGION	1493	VITORIA D(	150835	1		2	2
1	SP	GVE XXV S	1349	SANTOS	354850	1		2	2
1	MT	CUIABA	1578	VARZEA GF	510840			2	2
1	MT	CUIABA	1578	CUIABA	510340	1		2	2
1	GO	CENTRO SU	1781	APARECID	520140	1		2	9
1	RJ			DUQUE DE	330170	1			
1	RJ			DUQUE DE	330170	1		2	9
1	RJ			SAO JOAO	330510	1		2	9
1	RJ			SAO JOAO	330510	1		2	9
1	GO	ESTRADA D	1802	CATALAO	520510	1		2	2
1	AM	ENTORNO	5584	MANAUS	130260	1	1	2	2
1	PA	09 REGION	1492	MONTE AL	150480	1		2	2
1	GO	RIO VERME	1794	ARAGUAP	520215	1	2	2	2
1	SP	GVE X OSA	1335	BARUERI	350570	1		2	2
1	SP	GVE VIII M	1333	ITAQUAQU	352310			2	2
1	RS	018 CRS	1605	TRAMAND	432160	1		2	2
1	SC	JARAGUA D	1567	JARAGUA D	420890	1		2	2
1	CE	2 CRES CAU	1512	SAO GONC	231240	2		9	9
1	CE	13 CRES TI	1523	IBIAPINA	230530			2	2
1	MG	BELO HORI	1449	NOVA LIM	314480	9		2	2

1	RS	006 CRS	1611	CARAZINH	430470	1		2	2
1	DF			SANTA MA	530150	1		2	2
1	SP	GVE XVII C.	1342	JAGUARIU	352470	1	2		2
1	RS	002 CRS	1607	PORTO ALE	431490	1		2	2
1	SP	GVE XVII C.	1342	CAMPINAS	350950	1		2	2
1	BA	NUCLEO RI	1380	SALVADOR	292740			2	2
1	SP	GVE XVII C.	1342	CAMPINAS	350950			2	2
1	CE	10 CRES LII	1532	TABULEIRC	231310	1		2	9
1	SP	GVE XXXI S	1353	GUAREI	351850	1			
1	SP	GVE XVII C.	1342	JUNDIAI	352590	1		2	2
1	MA	REGIONAL	1444	BERNARDC	210193	2		2	2
1	SP	GVE I CAPI	1331	SAO PAULC	355030	1		2	9
1	RJ			DUQUE DE	330170	1		2	9
1	SP	GVE XVII C.	1342	PEDREIRA	353710	1		9	9
1	SP	GVE I CAPI	1331	SAO PAULC	355030			2	2
1	PR	10RS CASC	1364	CASCAVEL	410480	1		2	2
1	RS	001 CRS	1592	CANOAS	430460	1		2	2
1	MG	BELO HORI	1449	MATEUS LI	314070	1			2
1	SP	GVE I CAPI	1331	SAO PAULC	355030	1	2	2	9
1	SP	GVE XXXI S	1353	RIBEIRAO (	354325	1		2	2
1	MT	JUINA	1582	JUINA	510515	2		2	2
1	PR	04RS IRATI	1358	FERNANDE	410773	1		2	2
1	SP	GVE VIII M	1333	GUARULHC	351880	1		2	2
1	MG	MONTES C	1473	TAIOBEIRA	316800	1		2	2
1	MA	REGI0NAL	1430	SAO LUIS	211130	1		2	2
1	PA	06 REGION	1489	MOJU	150470	1		2	
1	SP	GVE I CAPI	1331	SAO PAULC	355030	1		2	2
1	PI	4 GRS TERE	1883	TERESINA	221100	1		2	2
1	RS	005 CRS	1608	CAXIAS DO	430510			2	2
1	SP	GVE X OSA	1335	OSASCO	353440	1		2	2
1	CE	1 CRES FOF	1519	FORTALEZ/	230440	1		2	9
1	RJ			BELFORD R	330045	1			
1	CE	1 CRES FOF	1519	FORTALEZ/	230440	1		2	2
1	SP	GVE VII SAI	1332	RIBEIRAO F	354330	1		2	
1	MT	TANGARA	1585	TANGARA	510795			2	2
1	GO	CENTRAL	1779	GOIANIA	520870	1			
1	PR	08RS FRAN	1362	AMPERE	410100	1		2	2
1	SP	GVE XI ARA	1336	GUARARAF	351820	1		2	
1	MG	BELO HORI	1449	BELO HORI	310620	1		2	2
1	PR	09RS FOZ D	1363	SAO MIGU	412570	2		2	
1	RJ			DUQUE DE	330170	1		9	9
1	MA	REGI0NAL	1430	SAO LUIS	211130	1		2	2
1	SP	GVE XXX JA	1574	JALES	352480	1		2	2
1	CE	10 CRES LII	1532	JAGUARIB/	230680	1		9	9
1	SP	GVE I CAPI	1331	SAO PAULC	355030	9		2	9
1	DF			CEILANDIA	530040	1		2	2
1	SP	GVE XXXI S	1353	ARACARIG	350275	1		2	2

1	RJ			MAGE	330250	1		2	2
1	RS	018 CRS	1605	CIDREIRA	430545	1		2	9
1	RJ			JAPERI	330227	1			
1	RJ			ITAGUAI	330200	1		2	9
1	BA	NUCLEO RI	1393	JUAZEIRO	291840	1		2	2
1	DF			TAGUATIN	530180	1			
1	RS	001 CRS	1592	SAPIRANG/	431990	1		9	2
1	PB	I NRS JOAC	1377	SANTA RIT/	251370	1		2	2
1	PB	III NRS CAN	1421	CAMPINA (	250400	1		2	2
1	SP	GVE VIII M	1333	GUARULH(	351880	1		2	2
1	DF	SUPERINTE	6262	PLANALTIN	530120	1		2	9
1	PA	11 REGION	1494	MARABA	150420	1		2	2
1	MT	ALTA FLOR	1587	APIACAS	510080			2	2
1	CE	17 CRES IC	1527	OROS	230950	1		9	9
1	SP	GVE XVI BC	1341	BOTUCATU	350750	1		2	2
1	GO	CENTRAL	1779	GOIANIA	520870	1		2	2
1	BA	NUCLEO RI	1398	PALMAS DI	292340	9		2	2
1	AM	ENTORNO	5584	MANAUS	130260	1		2	2
1	SP	GVE I CAPI	1331	SAO PAUL(	355030	1		2	2
1	BA	NUCLEO RI	1380	SALVADOR	292740	1		2	9
1	SP	GVE I CAPI	1331	SAO PAUL(	355030	1		2	2
1	MT	CUIABA	1578	CUIABA	510340			2	2
1	SP	GVE XXXI S	1353	SOROCABA	355220	1	2	2	2
1	AM	ENTORNO	5584	MANAUS	130260	1	2	2	1
1	BA	NUCLEO RI	1380	SALVADOR	292740			9	9
1	SP	GVE XI ARA	1336	ARACATUE	350280	1		2	2
1	SP	GVE I CAPI	1331	SAO PAUL(	355030	1		2	2
1	RJ			RIO DE JAN	330455	1		9	9
1	MG	BELO HORI	1449	BELO HORI	310620	1		2	9
1	SP	GVE XVII C/	1342	NOVA ODE	353340	1		2	2
1	PR	10RS CASC	1364	QUEDAS D	412090	1		2	2
1	RJ			RIO DE JAN	330455	1		2	2
1	BA	NUCLEO RI	1380	LAURO DE	291920	1		2	2
1	SP	GVE VIII M	1333	GUARULH(	351880	1		2	2
1	MG	UBERABA	1461	ITURAMA	313440	1		2	2
1	SP	GVE I CAPI	1331	SAO PAUL(	355030	1		2	2
1	MT	CUIABA	1578	VARZEA GI	510840	1		2	2
1	GO	ENTORNO	1782	CIDADE OC	520549	1		2	9
1	MA	REGIONAL	1436	SAO BENT(	211050	2			
1	SP	GVE I CAPI	1331	SAO PAUL(	355030	1		2	2
1	MG	ITABIRA	1451	FERROS	312590	1			
1	SP	GVE VII SAI	1332	MAUA	352940	1		2	2
1	SP	GVE X OSA	1335	EMBU DAS	351500	1		2	2
1	SP	GVE I CAPI	1331	SAO PAUL(	355030	1		2	2
1	MT	CUIABA	1578	CUIABA	510340	1		2	2
1	CE	9 CRES RUS	1518	JAGUARUA	230700	1		9	9
1	SP	GVE I CAPI	1331	SAO PAUL(	355030			2	2

1	MG	ITUIUTABA	1378	ITUIUTABA	313420	1	2	2	2
1	DF			TAGUATIN	530180	1		2	2
1	RO	VI GRS POF	1483	PORTO VEL	110020	1		2	9
1	PR	02RS METF	1356	CERRO AZU	410520	1		2	2
1	PB	I NRS JOAC	1377	JOAO PESS	250750	1		2	2
1	SP	GVE I CAPI	1331	SAO PAULC	355030	1		2	2
1	MT	CUIABA	1578	VARZEA GF	510840	1		2	2
1	RS	011 CRS	1600	ERECHIM	430700	1		2	2
1	PA	01 REGION	1484	BELEM	150140				
1	SC	ITAJAI	1550	BALNEARIC	420200	1		2	2
1	PA	12 REGION	1495	SANTANA I	150670	1		2	2
1	CE	4 CRES BAT	1520	CAPISTRAN	230290	2		2	1
1	PA	05 REGION	1488	SAO MIGU	150760	1		2	2
1	SP	GVE X OSA	1335	TABOAO D	355280				
1	PR	10RS CASC	1364	GUARANIA	410930	1		2	2
1	SC	JOINVILLE	1565	JOINVILLE	420910			2	2
1	PR	10RS CASC	1364	CASCAVEL	410480	1		2	
1	SP	GVE VII SAI	1332	SAO BERNA	354870	1		2	2
1	CE	1 CRES FOF	1519	FORTALEZA	230440	1		2	2
1	GO	CENTRAL	1779	GOIANIA	520870	1			
1	SP	GVE I CAPI	1331	SAO PAULC	355030	1		9	9
1	SP	GVE I CAPI	1331	SAO PAULC	355030	1		1	2
1	RR			CARACARA	140020	1		2	2
1	SP	GVE XXXI S	1353	ALUMINIO	350115	1		2	2
1	SP	GVE XVII C	1342	ARTUR NO	350380	1		2	2
1	RN	II URSAP	1412	MOSSORO	240800	1		2	2
1	RJ			RIO DAS OS	330452	1			
1	PB	IX NRS CAJ	1425	CAJAZEIRA	250370	1		2	2
1	RJ			RIO DE JAN	330455	1		2	2
1	SP	GVE I CAPI	1331	SAO PAULC	355030	1		2	2
1	PR	02RS METF	1356	CURITIBA	410690	1		2	2
1	PR	10RS CASC	1364	CASCAVEL	410480	1		2	2
1	SC	JOINVILLE	1565	JOINVILLE	420910	1		2	2
1	SP	GVE XXXII I	1575	ITARARE	352320				
1	RS	003 CRS	1593	CHUI	430543	1		2	9
1	DF			SAO SEBAS	530160	1		2	9
1	MT	SINOP	1584	SORRISO	510792	1		2	2
1	GO	CENTRAL	1779	ABADIA DE	520005	1		2	2
1	AM	RIO NEGRC	5588	MANACAP	130250	1			
1	MG	UBERLAND	1462	UBERLAND	317020	1		2	2
1	CE	1 CRES FOF	1519	FORTALEZA	230440	1	2		2
1	SP	GVE I CAPI	1331	SAO PAULC	355030	1		2	2
1	PA	04 REGION	1487	SANTA LUZ	150655	2		2	2
1	DF			SAMAMBA	530140	1		2	
1	BA	NUCLEO RI	1380	SALVADOR	292740			9	9
1	SP	GVE I CAPI	1331	SAO PAULC	355030	1			
1	BA	NUCLEO RI	1380	SALVADOR	292740			2	9

1	RN	VI URSAP	1417	PAU DOS F	240940	1		2	2
1	SP	GVE X OSA	1335	OSASCO	353440	1		2	2
1	PA	11 REGION	1494	CANAA DO	150215	2		2	1
1	SP	GVE I CAPI	1331	SAO PAULO	355030	1		2	2
1	SP	GVE XX PIR	1345	LEME	352670	1		2	2
1	PR	09RS FOZ D	1363	FOZ DO IGU	410830	1		2	2
1	RS	010 CRS	1599	ALEGRETE	430040	1	1	2	2
1	MG	BELO HORI	1449	CONTAGEM	311860	1		9	9
1	CE	1 CRES FOF	1519	AQUIRAZ	230100	1		2	9
1	MG	ALFENAS	1379	BOTELHOS	310840	1		2	2
1	RN	V URSAP	1415	SITIO NOVO	241370	2		2	2
1	CE	22 CRES CA	5596	OCARA	230945	1		2	9
1	SP	GVE I CAPI	1331	SAO PAULO	355030	1		2	2
1	CE	6 CRES ITA	1515	TRAIRI	231350	1		9	9
1	GO	CENTRAL	1779	GOIANIA	520870	1		2	2
1	GO	ENTORNO	1782	VALPARAIS	522185	1		2	9
1	BA	NUCLEO RE	1393	CAMPO FO	290600			9	9
1	AM	ENTORNO	5584	MANAUS	130260	1		2	2
1	PR	09RS FOZ D	1363	FOZ DO IGU	410830	1		2	2
1	SP	GVE XXVII S	1351	SAO JOSE D	354990	1			
1	TO			AGUIARNO	170030	1	2	2	9
1	RS	013 CRS	1609	VENANCIO	432260	1		2	2
1	SP	GVE I CAPI	1331	SAO PAULO	355030			2	2
1	AM	MEDIO AM	5590	SAO SEBAS	130395	1		2	2
1	SP	GVE X OSA	1335	BARUERI	350570	1		2	9
1	RS	002 CRS	1607	ELDORADO	430676	1		2	9
1	MA	REGIONAL	1446	BACABAL	210120	1		2	9
1	RJ			MARICA	330270	1		2	9
1	RR			CANTA	140017	2		2	2
1	MG	DIVINOPOI	1472	LAGOA DA	313720	1		2	2
1	SP	GVE XI ARA	1336	GUZOLAND	351890	1		2	2
1	BA	NUCLEO RE	1380	VERA CRUZ	293320			9	9
1	SP	GVE I CAPI	1331	SAO PAULO	355030	1		2	2
1	PR	01RS PARA	1355	PARANAGU	411820	1			
1	MT	CUIABA	1578	VARZEA GR	510840	1		2	2
1	RJ			RIO DE JAN	330455	1		2	2
1	PR	17RS LOND	1371	LONDRINA	411370	1		2	2
1	PR	15RS MARI	1369	MARINGA	411520	1		2	2
1	AM	ENTORNO	5584	MANAUS	130260	2	2	2	2
1	MG	PONTE NO	1458	ORATORIO	314585	2		2	2
1	SP	GVE I CAPI	1331	SAO PAULO	355030	1		2	2
1	GO	CENTRAL	1779	GOIANIA	520870	1		2	9
1	MG	PONTE NO	1458	PONTE NO	315210	1		2	2
1	PR	02RS METR	1356	CURITIBA	410690	1		2	2
1	PA	03 REGION	1486	CASTANHA	150240	1		2	2
1	SP	GVE XXXI S	1353	ITAPETININ	352230	1		2	2
1	SP	GVE VII SA	1332	MAUA	352940	1		2	2

1	DF			SAMAMBA	530140	1		2	9
1	PB	IV NRS CUI	1418	BARAUNA	250153				
1	RJ			MACAE	330240	1			
1	MG	JUIZ DE FO	1452	ANDRELAN	310280			2	2
1	CE	2 CRES CAU	1512	CAUCAIA	230370	1		2	9
1	PA	06 REGION	1489	MOJU	150470	1		2	2
1	TO			ALVORADA	170070	1		2	9
1	PR	03RS PONT	1357	PONTA GR	411990	1		2	2
1	PA	12 REGION	1495	REDENCAC	150613	1		2	2
1	TO			PORTO NA	171820	1		2	2
1	CE	3 CRES MA	1513	MARACAN	230765	1		2	2
1	RJ			ARARUAM	330020			9	9
1	SC	FLORIANOI	1476	PALHOCA	421190	1		2	
1	PR	10RS CASC	1364	CASCAVEL	410480	1		2	2
1	SP	GVE XXXI S	1353	SOROCABA	355220	1		2	2
1	AP			OIAPOQUE	160050	1			2
1	CE	1 CRES FOF	1519	FORTALEZA	230440	1		2	9
1	SP	GVE I CAPI	1331	SAO PAULC	355030	1		2	2
1	MG	BELO HORI	1449	BETIM	310670	1		9	9
1	SP	GVE X OSA	1335	CARAPICUI	351060	1		2	2
1	SP	GVE X OSA	1335	JANDIRA	352500			2	2
1	PR	02RS METF	1356	CAMPO LA	410420			2	2
1	MT	TANGARA	1585	CAMPO NC	510263	2		2	2
1	TO			CRISTALAN	170610	1		9	9
1	AM	ENTORNO	5584	IRANDUBA	130185	1		2	2
1	PR	09RS FOZ D	1363	MEDIANEII	411580	1		2	2
1	SP	GVE VIII M	1333	SUZANO	355250	1		2	2
1	SP	GVE VIII M	1333	GUARULHC	351880	1		2	2
1	MG	DIVINOPOI	1472	ITATIAIUCU	313370	1	2	2	2
1	SP	GVE XXIX S	1354	NHANDEAI	353260	1		2	2
1	RJ			QUEIMADC	330414	1		2	9
1	RJ			RIO DE JAN	330455	1		9	9
1	PR	09RS FOZ D	1363	FOZ DO IGU	410830	1		2	2
1	SP	GVE VIII M	1333	GUARULHC	351880	1		2	2
1	SP	GVE VII SAI	1332	SAO BERN	354870	1		2	2
1	PA	02 REGION	1485	CONCORDI	150275	1		2	9
1	SP	GVE XIV BA	1339	OLIMPIA	353390	1		2	2
1	PR	02RS METF	1356	CURITIBA	410690	1	2	2	9
1	PA	09 REGION	1492	MONTE AL	150480	1		2	2
1	BA	NUCLEO RI	1380	SALVADOR	292740	1		2	9
1	RS	004 CRS	1595	SANTA MA	431690	1		2	2
1	SP	GVE XXIX S	1354	NOVA GRA	353300	1		2	2
1	PE	001	1497	SAO LOURI	261370	1		2	2
1	CE	22 CRES CA	5596	PACAJUS	230960	1		9	9
1	MG	PEDRA AZU	1457	ITAOBIM	313330	1		2	2
1	SP	GVE I CAPI	1331	SAO PAULC	355030	1		2	2
1	SP	GVE XXIV R	1348	RIBEIRAO F	354340	1		2	2

1	SP	GVE XIII AS	1338	ASSIS	350400	1		2	2
1	BA	NUCLEO RE	1382	ANTAS	290160			9	9
1	SP	GVE XXVI S	1350	ITAPIRA	352260	1		2	2
1	GO	CENTRO SU	1781	APARECIDA	520140	1		2	2
1	RN	VII URSAP	1416	MACAIBA	240710	2		2	9
1	GO	CENTRAL	1779	GOIANIA	520870	1		2	2
1	RS	004 CRS	1595	SANTA MA	431690	1		2	2
1	PA	01 REGION	1484	ANANINDE	150080	1		1	2
1	SP	GVE XXIX S	1354	JOSE BONI	352570	1		2	2
1	SP	GVE X OSA	1335	EMBU DAS	351500	1		2	9
1	SP	GVE I CAPI	1331	SAO PAULO	355030	1		2	2
1	MG	UBERABA	1461	ITURAMA	313440	1		2	2
1	MA	REGIONAL	1431	ROSARIO	210960	2		2	9
1	ES	METROPOL	1510	SERRA	320500	1		2	2
1	CE	16 CRES CA	1526	GRANJA	230470	1		2	2
1	PR	14RS PARA	1368	PARANAVA	411840	1		2	2
1	PE	006	1501	INAJA	260700	2		2	9
1	RJ			RIO DE JAN	330455	1			
1	SC	ITAJAI	1550	BOMBINHA	420245	1	9	9	2
1	MG	UBERLAND	1462	UBERLAND	317020	1		2	2
1	SP	GVE I CAPI	1331	SAO PAULO	355030	1		2	9
1	SP	GVE I CAPI	1331	SAO PAULO	355030	1		2	9
1	RO	VI GRS POR	1483	PORTO VEL	110020	1		2	2
1	GO	SUDOESTE	1800	MINEIROS	521310	1		2	2
1	SP	GVE VIII M	1333	SANTA ISA	354680	1		2	2
1	SP	GVE XI ARA	1336	GUARARAP	351820			2	2
1	SP	GVE XXIV R	1348	RIBEIRAO P	354340	1		2	2
1	SP	GVE I CAPI	1331	SAO PAULO	355030	1		2	2
1	RS	002 CRS	1607	ALVORADA	430060	1		2	2
1	MA	REGIONAL	1431	BARREIRIN	210170	1		2	9
1	SP	GVE XXVII S	1351	SAO JOSE D	354990				
1	RJ			CAMPOS D	330100	2		2	2
1	RN	II URSAP	1412	ACU	240020	1	1	2	2
1	RS	006 CRS	1611	TAPERA	432100	1		2	2
1	SP	GVE VIII M	1333	ITAQUAQU	352310	1		2	2
1	PB	VI NRS PAT	1422	PATOS	251080	1		2	2
1	MG	BELO HORI	1449	CONTAGEM	311860	1		9	9
1	GO	CENTRO SU	1781	CEZARINA	520545	1			
1	TO			PONTE ALT	171790	1		2	2
1	RJ			ARARUAM	330020	1		9	9
1	MT	CUIABA	1578	CUIABA	510340	1		2	2
1	SC	MAFRA	1566	PAPANDUV	421220	1		2	2
1	SP	GVE I CAPI	1331	SAO PAULO	355030	1		2	9
1	BA	NUCLEO RE	1382	NOVO TRIU	292305			2	9
1	SP	GVE I CAPI	1331	SAO PAULO	355030	1		9	2
1	MG	POUSO ALE	1459	MARIA DA	313990	1		2	2
1	GO	CENTRAL	1779	TRINDADE	522140	1		2	2

1	SP	GVE I CAPIT	1331	SAO PAULO	355030	1		2	2
1	RJ			DUQUE DE	330170	1		9	9
1	PB	I NRS JOAC	1377	JOAO PESS	250750	1		2	2
1	AM	ENTORNO	5584	MANAUS	130260	1		2	2
1	PA	02 REGION	1485	CONCORDI	150275	1		2	2
1	SP	GVE XVI BO	1341	PIRAJU	353880	1		2	2
1	RJ			RIO DE JAN	330455	1		2	2
1	SP	GVE I CAPIT	1331	SAO PAULO	355030				
1	SP	GVE XIV BA	1339	BARRETOS	350550	1		2	2
1	PR	02RS METR	1356	ARAUCARI	410180	2		2	2
1	SE	REGIONAL	2062	PROPRIA	280570	1		2	2
1	PR	10RS CASC	1364	CEU AZUL	410530	2		2	2
1	SP	GVE XVII CA	1342	VALINHOS	355620	1		9	9
1	CE	11 CRES SO	1521	RERIUTABA	231170			2	2
1	MA	REGIONAL	1441	FEIRA NOV	210407	2		2	2
1	SP	GVE XX PIR	1345	LIMEIRA	352690	1		2	2
1	PR	15RS MARI	1369	MARINGA	411520	1		2	2
1	SP	GVE XXIX S	1354	BADY BASS	350460	1		2	2
1	RJ			DUQUE DE	330170	1			
1	SP	GVE XVIII F	1343	ORLANDIA	353430	1			2
1	MG	CORONEL F	1468	CARATINGA	311340	1		2	1
1	MA	REGIONAL	1440	JOAO LISBO	210550	1		2	2
1	MG	DIVINOPOL	1472	NOVA SERI	314520	1		2	2
1	MG	LEOPOLDIN	1453	LEOPOLDIN	313840	1		2	2
1	BA	NUCLEO RE	1381	SEABRA	292990	1		2	2
1	SP	GVE XII ARA	1337	SAO CARLO	354890	1		2	2
1	RJ			BELFORD R	330045	1			
1	MT	CUIABA	1578	CUIABA	510340	1		2	2
1	PE	010	1505	TUPARETA	261590	2		2	2
1	SP	GVE XI ARA	1336	BIRIGUI	350650	1		2	2
1	RJ			JAPERI	330227	1			
1	DF			JARDIM BO	539930			2	2
1	PR	15RS MARI	1369	MARINGA	411520	1		2	2
1	GO	SUDOESTE	1799	QUIRINOPO	521850	1		2	2
1	SP	GVE XXIV R	1348	GUARIBA	351860	1		2	2
1	MG	UBERLAND	1462	UBERLAND	317020	1		2	2
1	AP			MACAPA	160030	1		2	2
1	BA	NUCLEO RE	1380	SALVADOR	292740	1		2	9
1	RS	004 CRS	1595	RESTINGA	431550			2	1
1	MA	REGIONAL	1438	GODOFRED	210430	2		2	2
1	GO	CENTRO SU	1781	APARECIDA	520140	1		2	2
1	BA	NUCLEO RE	1398	JACARACI	291740	1			
1	SP	GVE XVII CA	1342	JUNDIAI	352590	1		2	2
1	CE	16 CRES CA	1526	CAMOCIM	230260			2	2
1	RS	002 CRS	1607	PORTO ALE	431490		2	2	9
1	MT	TANGARA	1585	TANGARA	510795			2	2
1	PR	09RS FOZ D	1363	FOZ DO IGU	410830	1		2	2

1	RS	013 CRS	1609	SANTA CRU	431680	1	2	2	2
1	PR	02RS METR	1356	CURITIBA	410690	1		2	2
1	BA	NUCLEO RE	1381	AMELIA RO	290110			2	2
1	PR	04RS IRATI	1358	IMBITUVA	411010	1		2	2
1	RJ			RIO DE JAN	330455	1		2	2
1	SP	GVE VIII M	1333	GUARULHO	351880				
1	RJ			RIO DE JAN	330455	1		2	2
1	SP	GVE XIX MA	1344	TUPA	355500	1		2	2
1	MT	TANGARA	1585	TANGARA	510795			2	1
1	BA	NUCLEO RE	1380	SALVADOR	292740	1		2	9
1	SP	GVE XXXI S	1353	CERQUILHO	351150	9		9	9
1	ES	METROPOL	1510	VITORIA	320530	1		2	2
1	MS	NRS DE CO	1965	RIO VERDE	500740	1		2	2
1	RJ			SAO GONC	330490	1		1	2
1	MG	POUSO ALE	1459	POUSO ALE	315250	1		2	2
1	SP	GVE XVI BO	1341	BOTUCATU	350750	1		2	2
1	BA	NUCLEO RE	1382	NOVA SOU	292290	1			
1	RS	005 CRS	1608	CAXIAS DO	430510	1		2	2
1	MT	CUIABA	1578	CUIABA	510340	1		2	2
1	SP	GVE XVII CA	1342	CAMPINAS	350950	1		2	9
1	SP	GVE I CAPIT	1331	SAO PAULO	355030			2	9
1	PR	18RS CORN	1372	ABATIA	410010	1		2	2
1	SP	GVE X OSA	1335	ITAPEVI	352250	1		2	2
1	DF			PARANOA	530110	1		2	2
1	MG	CORONEL F	1468	IPATINGA	313130	1		9	2
1	SP	GVE XVII CA	1342	CAMPINAS	350950	1		2	2
1	MG	JUIZ DE FO	1452	JUIZ DE FO	313670	1		2	2
1	MT	AGUA BOA	1590	AGUA BOA	510020	1		2	2
1	MG	UBA	1464	MURIAE	314390	1		2	2
1	PR	10RS CASC	1364	CASCAVEL	410480	1		2	9
1	PR	17RS LOND	1371	LONDRINA	411370	1		2	2
1	SP	GVE X OSA	1335	OSASCO	353440	1		2	2
1	SP	GVE XXVII S	1351	SAO JOSE D	354990	1			
1	SP	GVE XXI PR	1346	TACIBA	355290	1		2	2
1	SC	VIDEIRA	1546	VIDEIRA	421930	1		2	2
1	PA	01 REGION	1484	BELEM	150140	1		2	2
1	GO	CENTRAL	1779	GOIANIA	520870	1		2	2
1	SP	GVE X OSA	1335	EMBU DAS	351500			2	
1	BA	NUCLEO RE	1380	SAO SEBAS	292950			2	9
1	MA	REGI0NAL	1430	SAO LUIS	211130	1		2	9
1	SP	GVE I CAPIT	1331	SAO PAULO	355030	1		2	2
1	BA	NUCLEO RE	1403	ANGICAL	290140	1		2	9
1	MS	EX ARS DE	1975	CAMPO GR	500270	1			
1	RJ			RIO DE JAN	330455	1		2	2
1	SP	GVE VII SA	1332	MAUA	352940	1		2	2
1	SP	GVE I CAPIT	1331	SAO PAULO	355030	1		2	2
1	SP	GVE I CAPIT	1331	SAO PAULO	355030	1		2	2

1	RJ			RIO DE JAN	330455			2	2
1	RN	II URSAP	1412	MOSSORO	240800	1		2	2
1	BA	NUCLEO RI	1380	SALVADOR	292740	9		2	9
1	SP	GVE XXI PR	1346	PRESIDENT	354140	1		2	2
1	SP	GVE XXIX S	1354	VOTUPOR/	355710	1		2	2
1	GO	CENTRAL	1779	GOIANIA	520870	1			
1	TO			PORTO NA	171820	1		2	2
1	SP	GVE VII SAI	1332	SAO BERN/	354870	1		2	2
1	GO	CENTRAL	1779	GOIANIA	520870	1		2	2
1	MG	UBERLAND	1462	UBERLAND	317020	1		2	2
1	RS	008 CRS	1597	CACHOEIR/	430300	1		2	2
1	SP	GVE I CAPI	1331	SAO PAULC	355030	1	2	2	2
1	RS	006 CRS	1611	COQUEIRO	430585	1		2	2
1	SP	GVE VIII M	1333	GUARULHC	351880	1		2	2
1	SP	GVE IX FRA	1334	FRANCISCC	351630	1		2	2
1	PR	11RS CAMI	1365	CAMPO MC	410430	1		2	2
1	RS	006 CRS	1611	LAGOA DO	431127	2		2	1
1	GO	CENTRO SL	1781	APARECID/	520140	1		2	2
1	SP	GVE X OSA	1335	TABOAO D	355280	1		2	
1	SP	GVE XXVII S	1351	SAO JOSE D	354990	1			
1	MG	UBERABA	1461	ARAXA	310400	1			
1	SP	GVE XXVI S	1350	ITAPIRA	352260	1		2	2
1	MT	CUIABA	1578	CUIABA	510340	1		2	2
1	GO	CENTRO SL	1781	APARECID/	520140	1		2	2
1	RS	002 CRS	1607	GRAVATAI	430920				
1	RJ			RIO DE JAN	330455	9			
1	SP	GVE XII AR/	1337	SAO CARLC	354890	1		2	9
1	GO	CENTRAL	1779	GOIANIA	520870	1		2	2
1	GO	ENTORNO	1782	CIDADE OC	520549	1		2	9
1	RO	I GRS JI PAI	1474	JI-PARANA	110012	1		1	2
1	MT	CACERES	1581	LAMBARI D	510523			2	2
1	AL	1 MICRORI	1533	MACEIO	270430				
1	CE	1 CRES FOF	1519	FORTALEZ/	230440	1		9	9
1	PA	04 REGION	1487	CAPANEM/	150220	1		2	2
1	SP	GVE XXXI S	1353	ITU	352390	1		2	9
1	DF			TAGUATIN	530180	1		2	9
1	RJ			RIO DE JAN	330455	1		2	2
1	GO	CENTRO SL	1781	ORIZONA	521530	1		2	2
1	GO	RIO VERMI	1794	GOIAS	520890	1		2	2
1	RR			BOA VISTA	140010	1		9	9
1	RJ			CAMPOS D	330100	2		2	2
1	CE	13 CRES TI/	1523	CARNAUB/	230340	1		9	9
1	GO	ESTRADA D	1802	PIRES DO R	521740	1	2	2	9
1	MG	VARGINHA	1466	VARGINHA	317070	1		2	2
1	RS	002 CRS	1607	CACHOEIRI	430310			2	2
1	AC	REGIONAL	1941	RODRIGUE	120042	2			
1	SP	GVE I CAPI	1331	SAO PAULC	355030	1		9	9

1	CE	12 CRES AC	1522	BELA CRUZ	230230		2	2
1	AC	REGIONAL	1938	RIO BRANC	120040	1	2	2
1	RS	018 CRS	1605	TRAMAND.	432160	1	2	2
1	SP	GVE XVII C.	1342	CAMPINAS	350950	1	2	2
1	SP	GVE I CAPI	1331	SAO PAUL(	355030	9	2	9
1	RJ			GUAPIMIR	330185	1	2	2
1	RJ			CAMPOS D	330100	1	2	2
1	RJ			RIO DE JAN	330455	1	9	9
1	GO	CENTRAL	1779	GOIANIA	520870	1	2	2
1	SP	GVE XXXI S	1353	SOROCABA	355220	1	2	2
1	SP	GVE X OSA	1335	OSASCO	353440	1	2	9
1	ES	METROPOI	1510	IBATIBA	320245	2	2	2
1	RJ			RIO DE JAN	330455	1		
1	MT	RONDONO	1579	RONDONO	510760	1	2	2
1	PE	001	1497	RECIFE	261160	1		
1	SP	GVE IX FRA	1334	CAJAMAR	350920	1	2	2
1	SC	JOINVILLE	1565	JOINVILLE	420910	1	2	2
1	SP	GVE VII SAI	1332	SAO CAETA	354880	1		
1	RS	001 CRS	1592	CAMPO BC	430390	1	2	2
1	PR	18RS CORN	1372	BANDEIRAI	410240	1	2	2
1	MS	NRS JARDII	5546	BONITO	500220	1	2	2
1	MT	SINOP	1584	LUCAS DO	510525	1	1	2
1	MS	NRS DE DO	1966	DOURADO.	500370	1	9	9
1	SP	GVE XVII C.	1342	HORTOLAN	351907	1	2	2
1	CE	1 CRES FOI	1519	FORTALEZ/	230440	1	9	9
1	PA	11 REGION	1494	PALESTINA	150549	1	2	2
1	PR	15RS MARI	1369	SARANDI	412625	1	2	2
1	SP	GVE I CAPI	1331	SAO PAUL(	355030	1	2	2
1	MT	BARRA DO	1580	BARRA DO	510180	1	2	2
1	SP	GVE VII SAI	1332	SAO CAETA	354880			
1	PR	10RS CASC	1364	CASCAVEL	410480	1	2	9
1	RJ			NITEROI	330330	1		
1	PR	02RS METF	1356	CURITIBA	410690	1	2	2
1	SP	GVE X OSA	1335	BARUERI	350570	9	2	9
1	MT	CACERES	1581	MIRASSOL	510562		2	2
1	PR	10RS CASC	1364	CASCAVEL	410480	1		
1	BA	NUCLEO RI	1380	CAMACARI	290570	1	9	9
1	PR	02RS METF	1356	COLOMBO	410580	1	2	2
1	MT	CUIABA	1578	CUIABA	510340	1	2	2
1	CE	1 CRES FOI	1519	FORTALEZ/	230440	1	2	9
1	MG	BELO HORI	1449	BELO HORI	310620	1	2	2
1	RJ			SAO JOAO	330510	1	9	9
1	MG	POUSO ALI	1459	MARIA DA	313990	2	2	2
1	SP	GVE VIII M	1333	GUARULH(	351880	1	2	2
1	GO	CENTRAL	1779	TRINDADE	522140	1	2	2
1	SC	JOINVILLE	1565	BARRA VEI	420210	1	2	2
1	SP	GVE I CAPI	1331	SAO PAUL(	355030	1	2	9

1	MG	GOVERNAD	1471	MANTENA	313960				2
1	RJ			MIGUEL PE	330290	1		2	9
1	SP	GVE XXIX S	1354	SAO JOSE D	354980	1		2	2
1	PR	10RS CASC	1364	CASCAVEL	410480	1		2	2
1	PR	02RS METR	1356	CAMPINA (	410400	2		2	2
1	PA	10 REGION	1493	PORTO DE	150590	1		2	2
1	SP	GVE XXV SA	1349	ITANHAEM	352210	1		9	9
1	RN	V URSAP	1415	SENADOR I	241310	2		2	2
1	SP	GVE XXIV R	1348	JARDINOPO	352510	1		2	2
1	MA	REGIONAL	1441	BALSAS	210140	1		2	2
1	SP	GVE XXX JA	1574	OUROESTE	353475	1		2	2
1	RR			BOA VISTA	140010	1		2	2
1	RJ			NOVA IGUA	330350	1			2
1	RS	005 CRS	1608	CAXIAS DO	430510	1		2	2
1	GO	ENTORNO	1782	LUZIANIA	521250	1		2	2
1	PR	15RS MARI	1369	MARINGA	411520	1		2	
1	RS	007 CRS	1596	BAGE	430160	1		2	2
1	SP	GVE XXXI S	1353	VOTORANT	355700	9		2	9
1	CE	13 CRES TIA	1523	GUARACIA	230500	2		2	2
1	MT	CUIABA	1578	CUIABA	510340			2	2
1	RN	II URSAP	1412	MOSSORO	240800	1		2	2
1	RS	017 CRS	1604	PANAMBI	431390	1		2	9
1	SP	GVE XXVI S	1350	CASA BRAN	351080	1		2	2
1	CE	7 CRES ARA	1516	ARACATI	230110	1		9	9
1	SP	GVE XVII CA	1342	JUNDIAI	352590				
1	RS	002 CRS	1607	PORTO ALE	431490	1		2	2
1	SP	GVE XVII CA	1342	CAMPINAS	350950	1		2	2
1	PA	12 REGION	1495	REDENCAO	150613	1		2	2
1	SP	GVE XVI BO	1341	SAO MANU	355010	1		1	2
1	MG	BELO HORI	1449	CONTAGEM	311860	1		2	2
1	SP	GVE XXXI S	1353	IBIUNA	351970	1		2	2
1	MG	UBERABA	1461	ITURAMA	313440	1		2	2
1	BA	NUCLEO RE	1393	GLORIA	291140	1			
1	MG	UBA	1464	VISCONDE	317200	2		2	2
1	SP	GVE XXII PI	1573	MIRANTE D	353020	1		2	2
1	TO			PARAISO D	171610	1		2	9
1	ES	METROPOL	1510	SERRA	320500			2	2
1	RJ			MARICA	330270	1		9	9
1	ES	METROPOL	1510	VITORIA	320530	1		2	2
1	RJ			RIO DE JAN	330455	1		9	9
1	RN	VII URSAP	1416	NATAL	240810	1		2	2
1	MG	PASSOS	1455	PIUMHI	315150	1		2	2
1	PR	17RS LOND	1371	LONDRINA	411370	1	2	2	2
1	RR			CANTA	140017	2		2	9
1	SP	GVE XXIX S	1354	SAO JOSE D	354980	1		2	2
1	MG	BELO HORI	1449	CONTAGEM	311860	1		9	9
1	SP	GVE XXXI S	1353	TATUI	355400	1		2	2

1	GO	CENTRO SU	1781	APARECIDA	520140	1		2	2
1	RJ			NOVA IGUA	330350	1		2	2
1	PE	006	1501	ARCOVERD	260120	1		9	9
1	PR	10RS CASC	1364	BOA VISTA	410305	1		2	2
1	PA	11 REGION	1494	MARABA	150420	1		2	2
1	CE	17 CRES IC	1527	OROS	230950				
1	SP	GVE VIII M	1333	MOGI DAS	353060	1		2	2
1	SP	GVE VIII M	1333	GUARULHO	351880	1		2	2
1	SC	CHAPECO	1553	CHAPECO	420420	1		9	9
1	SP	GVE I CAPI	1331	SAO PAULO	355030	1		2	2
1	SP	GVE X OSA	1335	JANDIRA	352500				
1	RJ			NOVA IGUA	330350	1		2	9
1	BA	NUCLEO RE	1380	SAPEACU	292960	9		2	9
1	CE	22 CRES CA	5596	CASCAVEL	230350	1		9	9
1	SP	GVE XXI PR	1346	PRESIDENT	354140	1		2	2
1	RS	002 CRS	1607	CACHOEIRI	430310			2	2
1	RJ			RIO DAS OS	330452	1			
1	SP	GVE IX FRA	1334	FRANCO DA	351640	1		2	2
1	RO	VI GRS POF	1483	PORTO VEL	110020	1	2	2	2
1	RN	VII URSAP	1416	NATAL	240810	1	2	2	2
1	GO	CENTRAL	1779	ABADIA DE	520005	1		2	2
1	PA	11 REGION	1494	JACUNDA	150380	1		2	2
1	MT	CUIABA	1578	CUIABA	510340	1		2	2
1	PR	19RS JACA	1373	CAMBARA	410360	1		2	2
1	PB	I NRS JOAO	1377	JOAO PESS	250750	1		2	2
1	SP	GVE VII SA	1332	SANTO AN	354780	1		2	2
1	SP	GVE I CAPI	1331	SAO PAULO	355030	1	2	2	9
1	SP	GVE I CAPI	1331	SAO PAULO	355030				
1	RR			BOA VISTA	140010	1	1	2	2
1	AC	REGIONAL	1938	RIO BRANC	120040	1			
1	MT	CACERES	1581	CACERES	510250	1		1	2
1	SC	JOINVILLE	1565	ARAQUARI	420130	1		2	2
1	GO	CENTRO SU	1781	APARECIDA	520140	1		2	9
1	SP	GVE IX FRA	1334	CAJAMAR	350920	9		2	9
1	SP	GVE XVII C	1342	JUNDIAI	352590				
1	SP	GVE XXVIII	1576	ILHABELA	352040	1		2	2
1	MG	SETE LAGO	1467	SETE LAGO	316720	1		2	2
1	GO	CENTRO SU	1781	BONFINOP	520355	1		2	2
1	PB	III NRS CAM	1421	QUEIMADA	251250	2			
1	RJ			RIO DE JAN	330455			2	2
1	SP	GVE VIII M	1333	GUARULHO	351880				
1	PR	20RS TOLE	1374	MARECHAL	411460	1		2	2
1	SP	GVE XVII C	1342	CAMPINAS	350950			2	2
1	TO			PORTO ALE	171800	1		2	9
1	RO	IV GRS ARI	1481	CUJUBIM	110094	1		2	2
1	PA	02 REGION	1485	SANTA IZA	150650	1		2	2
1	PR	15RS MARI	1369	SARANDI	412625	1		2	2

1	SP	GVE I CAPIT	1331	SAO PAULO	355030	1		2	2
1	SP	GVE VIII MO	1333	GUARULHO	351880	1		2	9
1	RO	VI GRS POR	1483	PORTO VEL	110020	1		2	2
1	SP	GVE I CAPIT	1331	SAO PAULO	355030	1		2	2
1	PI			LUIS CORR	220570	1		2	2
1	RJ			RIO DE JAN	330455			2	9
1	PR	17RS LOND	1371	CAMBE	410370	1		2	2
1	RS	001 CRS	1592	CANOAS	430460				
1	SC	ITAJAI	1550	BOMBINHA	420245	1		2	9
1	SP	GVE I CAPIT	1331	SAO PAULO	355030	1		2	2
1	AM	ENTORNO	5584	NOVA OLIN	130310	2	2		2
1	SP	GVE I CAPIT	1331	SAO PAULO	355030	1		2	2
1	GO	CENTRAL	1779	TRINDADE	522140	1		2	2
1	GO	CENTRAL	1779	GOIANIA	520870	1		2	2
1	RJ			RIO DE JAN	330455	9			
1	SP	GVE I CAPIT	1331	SAO PAULO	355030	1		2	
1	SP	GVE XXIV R	1348	RIBEIRAO P	354340	1		2	2
1	GO	SUDOESTE	1799	RIO VERDE	521880	1		2	2
1	CE	1 CRES FOR	1519	FORTALEZA	230440	1		2	
1	PR	10RS CASC	1364	SANTA TER	412402	1		2	2
1	CE	11 CRES SO	1521	SOBRAL	231290	1		2	2
1	MA	REGIONAL	1439	ACAILANDI	210005	1		2	2
1	MG	JUIZ DE FO	1452	DESCOBERT	312130	1		2	2
1	RJ			MAGE	330250	9		2	2
1	PR	12RS UMU	1366	IPORA	411060	1		2	2
1	SC	CHAPECO	1553	CHAPECO	420420	1	2	2	2
1	ES	METROPOL	1510	CARIACICA	320130			2	2
1	SP	GVE XXXI S	1353	ITU	352390	1		2	2
1	PR	01RS PARA	1355	PARANAGU	411820	1			
1	TO			PALMAS	172100	1		2	9
1	MG	PATOS DE I	1456	GUARDA-M	312860	1		2	2
1	RS	005 CRS	1608	VERANOPO	432280	1			
1	SP	GVE XVII CA	1342	AMPARO	350190	1		2	9
1	SC	RIO DO SUL	1478	RIO DO SUL	421480	1		2	2
1	ES	COLATINA	1509	LINHARES	320320	1	1	2	9
1	SP	GVE XVII CA	1342	SUMARE	355240	1		2	2
1	SP	GVE XVII CA	1342	CAMPINAS	350950			2	2
1	SP	GVE XXXI S	1353	SOROCABA	355220	1		1	9
1	GO	PIRINEUS	1792	ANAPOLIS	520110	1		2	2
1	MS	EX ARS DE	1975	CAMPO GR	500270	1		2	2
1	RS	018 CRS	1605	TRAMANDA	432160	1		2	9
1	MT	SINOP	1584	VERA	510850			2	2
1	CE	21 CRES JU	1531	CARIRIACU	230320	1		2	2
1	RJ			VALENCA	330610	1		2	2
1	PR	02RS METR	1356	COLOMBO	410580	1		2	2
1	MT	RONDONO	1579	CAMPO VE	510267	1		2	2
1	SP	GVE XXVI S	1350	MOGI GUA	353070	1		2	2

1	MA	REGIONAL	1442	SAO JOAO	211110	1		2	9
1	RJ			RIO DE JAN	330455	1		2	2
1	SP	GVE XXVI S	1350	MOGI GUA	353070	1		2	2
1	PR	10RS CASC	1364	CASCAVEL	410480	1		2	2
1	SC	FLORIANOI	1476	FLORIANOI	420540	1		2	2
1	SP	GVE I CAPI	1331	SAO PAULO	355030		1		2
1	RJ			SAO GONC	330490	1		2	2
1	PR	03RS PONT	1357	PONTA GR	411990				
1	MT	AGUA BOA	1590	AGUA BOA	510020			2	2
1	RS	001 CRS	1592	CANOAS	430460	1		2	9
1	SC	MAFRA	1566	PAPANDUV	421220	1		2	2
1	PE	002	1507	LIMOEIRO	260890	1		2	2
1	RN	VII URSAP	1416	PARNAMIR	240325	1		2	9
1	TO			MIRACEMA	171320	1		9	9
1	AP			ITAUBAL	160025	2		2	2
1	GO	CENTRAL	1779	NEROPOLIS	521450	1		2	2
1	SP	GVE X OSA	1335	OSASCO	353440			2	2
1	SP	GVE I CAPI	1331	SAO PAULO	355030	1		2	9
1	GO	CENTRAL	1779	GOIANIA	520870	1			
1	SP	GVE XXXI S	1353	SOROCABA	355220	1		9	9
1	SP	GVE XXVII S	1351	JACAREI	352440	1		2	2
1	BA	NUCLEO RE	1403	IPUPIARA	291410	1		2	2
1	GO	ENTORNO	1784	FORMOSA	520800	1		2	9
1	SP	GVE XXV SA	1349	SAO VICEN	355100	1		2	2
1	CE	21 CRES JU	1531	JARDIM	230710	2		2	9
1	BA	NUCLEO RE	1398	CAETITE	290520	1		2	2
1	RJ			NOVA IGUA	330350	1		2	2
1	SP	GVE IX FRA	1334	FRANCO DA	351640	1		9	9
1	PR	10RS CASC	1364	CASCAVEL	410480	1		2	9
1	DF			GUARA	530070	1		2	2
1	SP	GVE VIII M	1333	GUARULHO	351880	1		9	9
1	SP	GVE XXVII S	1351	SAO JOSE D	354990	1		2	2
1	SC	LAGES	1571	LAGES	420930	1		2	2
1	RS	018 CRS	1605	CAPAO DA	430463	1		2	9
1	PR	17RS LOND	1371	CENTENAR	410510	9	2	2	2
1	SP	GVE I CAPI	1331	SAO PAULO	355030	1		2	
1	RS	008 CRS	1597	ENCRUZILH	430690	1		2	9
1	PR	05RS GUAR	1359	LARANJEIR	411330			2	2
1	PR	10RS CASC	1364	CASCAVEL	410480	1		2	2
1	ES	METROPOI	1510	SERRA	320500			2	
1	SP	GVE X OSA	1335	BARUERI	350570	1		2	2
1	PA	01 REGION	1484	BENEVIDES	150150	2		2	2
1	SC	JOINVILLE	1565	JOINVILLE	420910	1		2	2
1	PR	03RS PONT	1357	PONTA GR	411990	1		2	2
1	SP	GVE I CAPI	1331	SAO PAULO	355030	1		2	2
1	AM	ENTORNO	5584	MANAUS	130260	2		2	2
1	PR	13RS CIAN	1367	CIDADE GA	410560	1		2	2

1	RJ			BOM JESUS	330060	1			
1	GO	SUDOESTE	1800	JATAI	521190	1		2	2
1	BA	NUCLEO RE	1394	MIGUEL CA	292120	1		2	2
1	SC	FLORIANOI	1476	BIGUACU	420230	1		2	
1	SP	GVE XXII PI	1573	DRACENA	351440	1		2	2
1	CE	2 CRES CAU	1512	CAUCAIA	230370	1			
1	SC	JOINVILLE	1565	JOINVILLE	420910	1		2	2
1	PR	17RS LOND	1371	IBIPORA	410980	1		2	2
1	SP	GVE XXVIII	1576	SAO SEBAS	355070	1		2	9
1	SP	GVE X OSA	1335	OSASCO	353440	1		2	2
1	RS	001 CRS	1592	ESTEIO	430770	1		2	9
1	SP	GVE XXIX S	1354	SAO JOSE D	354980	1		2	2
1	RJ			ITAOCARA	330210	2		2	2
1	GO	ENTORNO	1784	PLANALTIN	521760	1		2	9
1	PA	03 REGION	1486	CASTANHA	150240	2		2	2
1	AL	1 MICRORE	1533	MACEIO	270430	1		1	2
1	RS	005 CRS	1608	CAXIAS DO	430510	1		2	2
1	AM	ENTORNO	5584	MANAUS	130260	1	2	2	2
1	SP	GVE XIII AS	1338	ASSIS	350400	1		2	2
1	BA	NUCLEO RE	1380	SALVADOR	292740			2	2
1	GO	CENTRAL	1779	GOIANIA	520870			2	2
1	MT	TANGARA	1585	CAMPO NO	510263	1		2	9
1	PR	03RS PONT	1357	JAGUARIAI	411200	1			
1	SP	GVE XVII CA	1342	ITUPEVA	352400	1		2	9
1	BA	NUCLEO RE	1393	PINDOBAC	292460			9	9
1	RJ			RIO DE JAN	330455	1		2	2
1	SP	GVE XVII CA	1342	CAMPINAS	350950	1			
1	SP	GVE X OSA	1335	OSASCO	353440	1		2	2
1	SP	GVE XVII CA	1342	CAMPINAS	350950	1		2	9
1	MG	UNAI	1463	PARACATU	314700	1		2	
1	BA	NUCLEO RE	1382	RIBEIRA DO	292650			9	9
1	DF			BRAZLAND	530020	1		9	9
1	MG	SETE LAGO	1467	CAETANOP	310990	1		2	2
1	RJ			NITEROI	330330	1		2	9
1	RJ			RIO DE JAN	330455	1			
1	SP	GVE VIII M	1333	POA	353980	1		2	1
1	SP	GVE I CAPI	1331	SAO PAULO	355030	1	2	2	2
1	SP	GVE XXIX S	1354	IPIGUA	352115	1		2	2
1	SP	GVE XXVIII	1576	SAO SEBAS	355070	1		9	9
1	MA	REGIONAL	1445	GRAJAU	210480	2		2	1
1	RJ			SAO JOAO	330510	1		2	2
1	RJ			CAMPOS D	330100	1		2	2
1	SP	GVE I CAPI	1331	SAO PAULO	355030	1		2	9
1	RN	VI URSAP	1417	TENENTE A	241410	1		2	2
1	SP	GVE XVII CA	1342	HORTOLAN	351907	1		2	2
1	PA	09 REGION	1492	SANTAREM	150680	2		2	2
1	RJ			RIO DE JAN	330455			2	9

1	RJ			RIO DE JAN	330455	1		9	9
1	SP	GVE I CAPI	1331	SAO PAULO	355030	1		2	2
1	SP	GVE I CAPI	1331	SAO PAULO	355030	1		2	2
1	SP	GVE XXV SA	1349	ITANHAEM	352210	1		2	2
1	ES	SAO MATE	1508	SAO MATE	320490	1		2	9
1	SP	GVE I CAPI	1331	SAO PAULO	355030	1		2	9
1	PE	001	1497	RECIFE	261160	1			
1	RJ			RIO DE JAN	330455	1		2	9
1	SP	GVE X OSA	1335	CARAPICUI	351060	1		2	
1	GO	CENTRAL	1779	TRINDADE	522140	1		2	2
1	DF			SANTA MA	530150	1		2	2
1	SP	GVE XXXI S	1353	SOROCABA	355220			2	2
1	SP	GVE I CAPI	1331	SAO PAULO	355030	1			
1	SP	GVE XXVI S	1350	SAO JOAO	354910	1		2	9
1	MG	ITUIUTABA	1378	ITUIUTABA	313420	1		2	2
1	GO	CENTRAL	1779	GOIANIA	520870	1		2	2
1	SP	GVE XXIII R	1347	CAJATI	350925			2	2
1	MG	DIVINOPOI	1472	PITANGUI	315140	1		2	2
1	SP	GVE X OSA	1335	EMBU-GUA	351510	9		9	9
1	GO	SAO PATRI	1791	URUANA	522170				
1	SC	JARAGUA D	1567	JARAGUA D	420890	1		2	2
1	SC	BLUMENAU	1564	BRUSQUE	420290	1		2	2
1	AC	REGIONAL	1938	RIO BRANC	120040	1		2	2
1	SP	GVE XVII CA	1342	CAMPINAS	350950			2	2
1	RS	002 CRS	1607	PORTO ALE	431490	1		2	2
1	RJ			CONCEICA	330140	1		2	
1	SP	GVE I CAPI	1331	SAO PAULO	355030	1		2	2
1	SP	GVE I CAPI	1331	SAO PAULO	355030	1		2	2
1	SP	GVE XIX MA	1344	MARILIA	352900	1		2	2
1	GO	SUDOESTE	1800	JATAI	521190	1		2	2
1	BA	NUCLEO RE	1382	CIPO	290790			9	9
1	SP	GVE XXIX S	1354	NHANDEAI	353260	1		2	2
1	GO	PIRINEUS	1792	CAMPO LIN	520485	2		2	2
1	PA	11 REGION	1494	CANAA DO	150215	1		2	2
1	MG	DIVINOPOI	1472	BOM DESP	310740	1		2	2
1	SC	CHAPECO	1553	QUILOMBO	421420	1	2	2	2
1	SP	GVE XXIV R	1348	RIBEIRAO P	354340	1		2	2
1	SP	GVE I CAPI	1331	SAO PAULO	355030	1		2	9
1	SP	GVE X OSA	1335	OSASCO	353440	1		2	2
1	SP	GVE X OSA	1335	OSASCO	353440	1		2	2
1	DF	SUPERINTE	6262	PLANALTIN	530120	1		2	9
1	SP	GVE XXI PR	1346	SANTO AN	354770	1		2	2
1	SP	GVE XXV SA	1349	SAO VICEN	355100	1		9	9
1	MG	UBERABA	1461	UBERABA	317010			2	2
1	GO	CENTRAL	1779	GOIANIRA	520880	1		2	2
1	SP	GVE VII SAI	1332	SAO BERNA	354870	1		2	2
1	PI			LAGOA DE	220557	2		2	2

1	GO	PIRINEUS	1792	ABADIANI/	520010	1		2	2
1	PB	I NRS JOAC	1377	JOAO PESS	250750	1	2	2	2
1	RS	002 CRS	1607	VIAMAO	432300	1		2	2
1	PR	19RS JACAI	1373	SIQUEIRA (	412660	1		2	2
1	SC	CRICIUMA	1558	CRICIUMA	420460	1		2	2
1	PR	16RS APUC	1370	APUCARAN	410140	1		2	2
1	PR	05RS GUAF	1359	GUARAPU/	410940			2	2
1	GO	SAO PATRI	1791	RIALMA	521860	2		2	
1	MT	PONTES E L	1591	COMODOF	510330			2	2
1	SP	GVE X OSA	1335	SANTANA I	354730	1		2	
1	TO			PEDRO AF(	171650	1		9	9
1	PR	02RS METF	1356	CURITIBA	410690	1		2	2
1	RS	005 CRS	1608	GARIBALDI	430860	1		2	2
1	SP	GVE XXXI S	1353	PORTO FEL	354060	1		2	2
1	SP	GVE XXVII S	1351	JACAREI	352440	1			
1	AM	ENTORNO	5584	MANAUS	130260	1		2	2
1	SP	GVE I CAPI	1331	SAO PAUL(	355030	1	2	2	2
1	DF			SAO SEBAS	530160	1		2	9
1	PA	03 REGION	1486	TERRA ALT	150796	1		2	2
1	SP	GVE XVII C/	1342	JUNDIAI	352590				
1	RS	002 CRS	1607	VIAMAO	432300	1		2	2
1	SC	FLORIANOI	1476	SAO JOSE	421660	1			
1	SP	GVE I CAPI	1331	SAO PAUL(	355030	1		9	9
1	SP	GVE XIII AS	1338	ASSIS	350400	1		2	2
1	MG	BELO HORI	1449	PEDRO LEC	314930	1		2	2
1	MG	LEOPOLDIN	1453	PALMA	314670	1		9	2
1	PR	02RS METF	1356	COLOMBO	410580	1		2	2
1	GO	CENTRAL	1779	GOIANIA	520870	1		2	2
1	RS	001 CRS	1592	TAQUARA	432120				
1	RJ			RIO DE JAN	330455	1		9	9
1	MG	BELO HORI	1449	IBIRITE	312980			2	2
1	SP	GVE XVII C/	1342	CAMPINAS	350950	1		2	2
1	DF			BRASILIA	530010	1		2	2
1	RS	005 CRS	1608	CAXIAS DO	430510	1			
1	RJ			RIO DE JAN	330455	9			
1	MG	PIRAPORA	1469	BURITIZEIF	310940	1		2	9
1	SP	GVE XII AR/	1337	ITAPOLIS	352270	1		2	2
1	RS	002 CRS	1607	PORTO ALE	431490				
1	SP	GVE I CAPI	1331	SAO PAUL(	355030	1		2	9
1	GO	CENTRAL	1779	TRINDADE	522140	1		2	2
1	DF			RECANTO I	530130	1		2	9
1	PR	17RS LOND	1371	LONDRINA	411370	1		2	2
1	MG	PASSOS	1455	PIUMHI	315150	1		2	2
1	SP	GVE XXXI S	1353	SALTO	354520			2	2
1	GO	CENTRAL	1779	GOIANIA	520870	1		2	2
1	PA	01 REGION	1484	BELEM	150140	1		2	2
1	MT	CACERES	1581	CURVELAN	510343	3		2	2

1	RS	004 CRS	1595	SANTA MA	431690	1		2	2
1	SP	GVE XVII C	1342	JUNDIAI	352590	1		2	2
1	TO			PORTO NA	171820	1		2	2
1	MG	SAO JOAO	1460	PRADOS	315270	1			
1	MG	JUIZ DE FO	1452	JUIZ DE FO	313670	1		2	
1	PR	17RS LOND	1371	LONDRINA	411370	1		2	2
1	MG	DIVINOPOL	1472	LUZ	313880	1		2	2
1	SP	GVE XXIV R	1348	RIBEIRAO P	354340	1		2	2
1	SP	GVE XXVI S	1350	CASA BRAN	351080	1		2	9
1	RJ			RIO DE JAN	330455	1		2	2
1	DF			CEILANDIA	530040	1		2	9
1	SP	GVE X OSA	1335	SANTANA	354730	1		2	2
1	ES	COLATINA	1509	LINHARES	320320	1		2	2
1	RN	VII URSAP	1416	SAO GONC	241200	1		2	2
1	BA	NUCLEO RE	1381	FEIRA DE S	291080				
1	MA	REGIONAL	1439	BURITICUP	210232	2		2	9
1	SP	GVE XVII C	1342	SUMARE	355240	1		2	2
1	RJ			MARICA	330270	1			
1	SP	GVE VII SA	1332	SANTO AN	354780	1		2	2
1	MG	MONTES C	1473	MONTES C	314330	1	2	2	2
1	PE	001	1497	CAMARAG	260345	1		9	9
1	PR	05RS GUAR	1359	GUARAPU	410940	1		2	2
1	PR	02RS METR	1356	ALMIRANT	410040	1		2	2
1	SP	GVE X OSA	1335	BARUERI	350570				
1	PA	01 REGION	1484	ANANINDE	150080	1		2	2
1	SP	GVE XXXI S	1353	CERQUILH	351150	1	2	2	2
1	SP	GVE XIX M	1344	MARILIA	352900	1		2	2
1	SP	GVE XXVI S	1350	ITAPIRA	352260	1		2	2
1	PR	02RS METR	1356	CURITIBA	410690	1		2	2
1	PR	02RS METR	1356	CURITIBA	410690	1		2	2
1	SP	GVE XII AR	1337	SAO CARLO	354890	1		2	2
1	RJ			RIO DE JAN	330455	1			
1	MT	PEIXOTO D	1589	NOVO MU	510626	1		2	2
1	SP	GVE XVII C	1342	CAMPINAS	350950	1		2	2
1	RJ			NITEROI	330330	1			
1	RO	VI GRS POR	1483	PORTO VEL	110020	2		2	1
1	CE	20 CRES CR	1530	NOVA OLIN	230920	1		9	9
1	MS	NRS DE PO	1972	PONTA PO	500660	1		2	9
1	SC	RIO DO SU	1478	RIO DO SU	421480		2	2	2
1	SP	GVE X OSA	1335	OSASCO	353440	1		2	9
1	SP	GVE XVII C	1342	SUMARE	355240			2	2
1	MT	CACERES	1581	CACERES	510250	1		2	2
1	PR	04RS IRATI	1358	GUAMIRAN	410895	1		2	2
1	RS	012 CRS	1601	SAO BORJA	431800	1		2	2
1	MS	NRS DE TRI	6255	TRES LAGO	500830	1		2	2
1	MS	EX NRS DE	6256	CORUMBA	500320	1		2	2
1	PE	008	1503	PETROLINA	261110	1		2	2

1	SP	GVE XVI BC	1341	AVARE	350450	1		2	2
1	SP	GVE XX PIR	1345	PIRASSUNU	353930	1		2	2
1	GO	SERRA DA I	1790	URUACU	522160	1		2	2
1	RJ			BELFORD R	330045	1		9	9
1	RJ			CAMBUCI	330090	1		2	2
1	MA	REGIONAL	1443	CAPINZAL I	210275	1		2	2
1	PR	09RS FOZ D	1363	FOZ DO IGU	410830	1		2	2
1	PA	09 REGION	1492	SANTAREM	150680	2		2	2
1	RN	IV URSAP	1414	PARELHAS	240890	2		2	9
1	GO	SUDOESTE	1799	RIO VERDE	521880	1		2	2
1	SP	GVE XXVIII	1576	CARAGUAT	351050	1	9	9	9
1	AL	8 MICRORI	1540	SANTANA I	270800	2		2	2
1	MA	REGIONAL	1435	CURURUPU	210370	1		2	2
1	SP	GVE XXV SA	1349	GUARUJA	351870	1		9	9
1	MG	JUIZ DE FO	1452	JUIZ DE FO	313670	1		2	2
1	BA	NUCLEO RE	1380	SALVADOR	292740	1		2	2
1	SP	GVE XVII CA	1342	VINHEDO	355670	1		2	9
1	PA	11 REGION	1494	TUCURUI	150810	1		9	9
1	SP	GVE I CAPI	1331	SAO PAULO	355030	1		2	9
1	SP	GVE XXIV R	1348	RIBEIRAO P	354340			2	9
1	MG	PONTE NO	1458	SERICITA	316630	1		9	2
1	MG	DIVINOPOI	1472	LAGOA DA	313720	1		2	2
1	MS	NRS DE AQ	1964	MIRANDA	500560	1		2	2
1	CE	10 CRES LIN	1532	JAGUARIBE	230690	1		9	9
1	SP	GVE XXIX S	1354	MIRASSOL	353030	1		2	2
1	GO	CENTRAL	1779	GOIANIA	520870	1		2	2
1	CE	22 CRES CA	5596	PACAJUS	230960	1		9	9
1	SE	REGIONAL	2057	SANTA LUZ	280630				
1	SP	GVE XV BA	1340	BAURU	350600	1		2	2
1	PR	02RS METR	1356	CURITIBA	410690	1	2	2	9
1	DF	SUPERINTE	6262	PLANALTIN	530120	2		2	9
1	SP	GVE I CAPI	1331	SAO PAULO	355030	1		2	9
1	RR			BOA VISTA	140010	1		2	2
1	SP	GVE XVI BC	1341	SAO MANU	355010	1		2	2
1	RO	III GRS VILH	1479	VILHENA	110030			2	2
1	GO	CENTRO SU	1781	SENADOR (	522045	1		9	9
1	MA	REGIONAL	1445	GRAJAU	210480	2		2	2
1	SP	GVE XIX MA	1344	MARILIA	352900	1		2	2
1	RS	002 CRS	1607	PORTO ALE	431490	1		2	2
1	SP	GVE XXVI S	1350	MOGI GUA	353070	1		2	2
1	RR			BOA VISTA	140010	2		2	2
1	SP	GVE VIII M	1333	GUARULHO	351880	1		2	2
1	PR	17RS LOND	1371	CAMBE	410370	1		2	2
1	RJ			CAMPOS D	330100	1		2	2
1	PR	15RS MARI	1369	MARINGA	411520	1		2	2
1	MT	SINOP	1584	NOVA MUT	510622			2	2
1	SP	GVE VIII M	1333	ITAQUAQU	352310	1		2	9

1	MT	RONDONO	1579	PRIMAVER	510704	1			
1	SP	GVE XVII C/	1342	CAMPINAS	350950	1		2	2
1	SP	GVE I CAPI	1331	SAO PAULO	355030	1	1	2	9
1	SP	GVE IX FRA	1334	FRANCISCO	351630	1		2	
1	MG	PONTE NO	1458	VICOSA	317130	1		2	2
1	SC	FLORIANOI	1476	PALHOCA	421190	1		2	2
1	SP	GVE XXVI S	1350	MOGI GUA	353070	1		2	2
1	SP	GVE X OSA	1335	OSASCO	353440	1		2	2
1	RS	010 CRS	1599	URUGUAIA	432240	1		2	2
1	SP	GVE VIII M	1333	FERRAZ DE	351570	1		2	2
1	PR	02RS METF	1356	FAZENDA F	410765	1		2	2
1	RJ			RIO DE JAN	330455	1		9	9
1	MG	VARGINHA	1466	VARGINHA	317070	1		2	2
1	MG	PATOS DE I	1456	GUARDA-M	312860	2		2	
1	MT	CACERES	1581	CACERES	510250	1			9
1	DF			SANTA MA	530150	1		2	2
1	RJ			RIO DE JAN	330455	1			
1	GO	SAO PATRI	6252	GOIANESIA	520860	1		2	2
1	SP	GVE X OSA	1335	BARUERI	350570	1		2	2
1	PR	02RS METF	1356	QUITANDII	412120				
1	SP	GVE XXXII I	1575	ITARARE	352320	1		2	2
1	SP	GVE XVII C/	1342	MONTE MO	353180	1		2	2
1	SP	GVE XXXI S	1353	JUMIRIM	352585	1		2	2
1	SP	GVE XVII C/	1342	JUNDIAI	352590	1		2	2
1	PR	09RS FOZ D	1363	FOZ DO IGU	410830			2	2
1	BA	NUCLEO RI	1380	CAMACARI	290570	1		2	2
1	BA	NUCLEO RI	1380	SALVADOR	292740	1		2	9
1	BA	NUCLEO RI	1403	SAO DESID	292890	1		2	9
1	SC	FLORIANOI	1476	FLORIANOI	420540	9			
1	RJ			PETROPOLI	330390	1			
1	SP	GVE XVII C/	1342	SUMARE	355240	1		2	2
1	SP	GVE X OSA	1335	COTIA	351300	1		2	2
1	MA	REGI0NAL	1430	SAO JOSE D	211120	1		2	2
1	MS	NRS DE TRI	6255	TRES LAGO	500830	1		2	2
1	PR	16RS APUC	1370	FAXINAL	410760	1		2	2
1	SP	GVE XXIX S	1354	JOSE BONII	352570	1		2	2
1	RN	VII URSAP	1416	SAO GONC	241200	1		2	2
1	GO	CENTRAL	1779	TRINDADE	522140	1		2	2
1	PR	11RS CAMI	1365	UBIRATA	412800	1		2	2
1	CE	3 CRES MA	1513	MARACAN	230765	1		2	9
1	RO	VI GRS POF	1483	NOVA MAI	110033	2		2	9
1	MA	REGI0NAL	1430	SAO LUIS	211130	1		9	9
1	SP	GVE XXXI S	1353	ALUMINIO	350115			2	9
1	PR	01RS PARA	1355	PARANAGU	411820	1			
1	SP	GVE I CAPI	1331	SAO PAULO	355030	1		2	2
1	SC	JOINVILLE	1565	JOINVILLE	420910	1		2	2
1	SP	GVE VIII M	1333	MOGI DAS	353060	1	2	2	2

1	ES	METROPOI	1510	AFONSO CI	320010	1		2	2
1	MG	UBA	1464	VISCONDE	317200	1		2	2
1	SP	GVE X OSA	1335	EMBU DAS	351500	1		2	2
1	SP	GVE VIII M	1333	GUARULHO	351880	1		2	2
1	MG	UBERLAND	1462	COROMAN	311930	1		2	2
1	BA	NUCLEO RI	1388	PORTO SEG	292530	1		2	2
1	SP	GVE I CAPI	1331	SAO PAULO	355030	1		2	2
1	SP	GVE I CAPI	1331	SAO PAULO	355030	1			
1	RS	014 CRS	1602	SANTA ROS	431720	2		2	2
1	GO	SUDOESTE	1799	RIO VERDE	521880	1	9		9
1	GO	NORTE	1788	PORANGAT	521800	1		2	2
1	SP	GVE XXXII I	1575	BURI	350800	1		2	2
1	MT	CUIABA	1578	VARZEA GI	510840	1		2	2
1	SP	GVE X OSA	1335	CARAPICUI	351060	1			
1	RJ			NOVA IGU/	330350	1			
1	PA	12 REGION	1495	SANTANA I	150670	2		9	2
1	SP	GVE I CAPI	1331	SAO PAULO	355030	1		2	9
1	SP	GVE XXVII S	1351	SAO JOSE D	354990	1		2	2
1	SP	GVE XXXI S	1353	ITU	352390	1		2	2
1	SC	CONCORDI	1560	CONCORDI	420430	1		2	2
1	RO	I GRS JI PAI	1474	OURO PRET	110015	1		1	2
1	MG	BELO HORI	1449	BETIM	310670	1		2	2
1	GO	CENTRAL	1779	GOIANIA	520870	1		2	9
1	MG	DIVINOPOI	1472	PARA DE M	314710	1		2	2
1	GO	OESTE I	1795	IPORA	521020	1		2	2
1	SP	GVE XXXIII	1352	SAO BENTO	354860	1			
1	MG	GOVERNAI	1471	GOVERNAI	312770	1		2	2
1	RS	018 CRS	1605	CAPAO DA	430463				
1	PR	10RS CASC	1364	CASCAVEL	410480			2	2
1	SP	GVE VII SAI	1332	SAO BERN/	354870	1		2	2
1	RJ			NOVA IGU/	330350	1		2	2
1	SP	GVE XVII C/	1342	PAULINIA	353650	1		2	9
1	RS	005 CRS	1608	CAXIAS DO	430510	1		2	2
1	GO	CENTRAL	1779	GOIANIA	520870	1		2	9
1	MA	REGIONAL	1435	CURURUPU	210370	2		2	1
1	MG	JUIZ DE FO	1452	JUIZ DE FO	313670			2	
1	SC	RIO DO SUI	1478	PRESIDENT	421400	1		2	2
1	PR	17RS LOND	1371	LONDRINA	411370	1		2	2
1	PR	13RS CIAN	1367	CIANORTE	410550	1		2	2
1	GO	CENTRO SU	1781	APARECIDA	520140	1		2	2
1	RJ			BELFORD R	330045	1		2	9
1	SP	GVE IX FRA	1334	FRANCO D/	351640	1		2	2
1	SP	GVE XXVI S	1350	ITAPIRA	352260	1		2	2
1	PR	02RS METF	1356	CURITIBA	410690	1		2	2
1	SP	GVE XXVIII	1576	UBATUBA	355540	1		2	2
1	RS	017 CRS	1604	IJUI	431020	1		2	2
1	MT	JUINA	1582	COTRIGUA	510337	1		2	2

1	RJ			RIO DE JAN	330455	1			
1	MA	REGI0NAL	1430	SAO LUIS	211130	1		2	9
1	PR	02RS METF	1356	CURITIBA	410690	1		2	2
1	PR	15RS MARI	1369	NOVA ESPE	411690	1		2	2
1	RS	002 CRS	1607	ALVORADA	430060	1		2	2
1	BA	NUCLEO RE	1393	JEREMOAB	291810	1			
1	SC	JOINVILLE	1565	JOINVILLE	420910	1		2	2
1	BA	NUCLEO RE	1381	FEIRA DE S.	291080	1		2	
1	RR			CANTA	140017	2		2	2
1	SP	GVE I CAPI	1331	SAO PAULO	355030	1		2	2
1	PA	08 REGION	1491	BAGRE	150110	1		2	9
1	SP	GVE XVII C.	1342	CAMPINAS	350950	1		2	2
1	CE	4 CRES BAT	1520	BATURITE	230210	1		9	9
1	RJ			MANGARA	330260	9		9	9
1	SP	GVE XXIV R	1348	CAJURU	350940	1			
1	PR	09RS FOZ D	1363	FOZ DO IGU	410830	1		2	2
1	MA	REGIONAL	1445	BARRA DO	210160			2	2
1	PR	17RS LOND	1371	IBIPORA	410980	1		2	2
1	BA	NUCLEO RE	1398	VITORIA DA	293330	1		9	9
1	RJ			VOLTA RED	330630	1		2	9
1	SP	GVE I CAPI	1331	SAO PAULO	355030	1		2	2
1	SP	GVE VIII M	1333	POA	353980	1		2	9
1	SP	GVE VII SAI	1332	DIADEMA	351380	1		9	9
1	BA	NUCLEO RE	1398	GUANAMB	291170		2	2	2
1	SP	GVE XXXI S	1353	SOROCABA	355220	1		2	2
1	MG	PONTE NO	1458	PONTE NO	315210	1		2	2
1	SP	GVE I CAPI	1331	SAO PAULO	355030		1	2	2
1	PR	02RS METF	1356	CURITIBA	410690	1		2	2
1	SP	GVE XXVI S	1350	VARGEM G	355640	1		2	2
1	SP	GVE XXXI S	1353	SOROCABA	355220	1		2	2
1	MT	SINOP	1584	NOVA MU	510622	1		2	2
1	SP	GVE XXVII S	1351	JACAREI	352440	1		2	2
1	AL	9 MICRORE	1541	PALMEIRA	270630	2		2	2
1	PR	10RS CASC	1364	IRACEMA D	411065	1		2	2
1	MT	SINOP	1584	NOVA MU	510622			2	2
1	PA	11 REGION	1494	BOM JESUS	150157	2		2	2
1	AM	ENTORNO	5584	MANAUS	130260	1		2	2
1	RJ			CAMPOS D	330100	1		2	2
1	RJ			CAMPOS D	330100	1	2	2	2
1	SP	GVE XXXI S	1353	BOITUVA	350700	1		2	2
1	DF			CEILANDIA	530040	1		2	9
1	AM	ENTORNO	5584	MANAUS	130260	1	2	2	2
1	SP	GVE I CAPI	1331	SAO PAULO	355030	1		2	2
1	MG	UNAI	1463	BURITIS	310930	1			
1	RS	001 CRS	1592	NOVO HAM	431340	1		2	2
1	RJ			RIO DE JAN	330455	1		2	2
1	SP	GVE X OSA	1335	OSASCO	353440	1		2	2

1	SP	GVE XXIX S	1354	NOVO HOF	353350	1		2	2
1	RJ			RIO DE JAN	330455	1			
1	RJ			RIO DE JAN	330455	1		9	9
1	BA	NUCLEO RI	1380	SALVADOR	292740	1		2	9
1	CE	1 CRES FOF	1519	FORTALEZA	230440	1		9	9
1	SP	GVE XVII C	1342	CAMPINAS	350950	1		2	2
1	PR	16RS APUC	1370	ARAPONGA	410150	1		2	2
1	SP	GVE XV BA	1340	BAURU	350600	1		2	2
1	RJ			BELFORD R	330045	1		9	9
1	RS	003 CRS	1593	PELOTAS	431440	1	2	2	2
1	GO	SAO PATRI	1791	CAMPOS V	520495	1		2	2
1	RS	001 CRS	1592	ESTEIO	430770			2	2
1	SP	GVE VIII M	1333	MOGI DAS	353060	1		2	2
1	SP	GVE VIII M	1333	GUARULHO	351880	1		2	2
1	PA	11 REGION	1494	SAO DOMI	150715	2		2	2
1	MG	GOVERNAI	1471	GOVERNAI	312770	1	1	2	9
1	BA	NUCLEO RI	1380	SALVADOR	292740	2		2	2
1	PR	15RS MARI	1369	SARANDI	412625	1		2	2
1	BA	NUCLEO RI	1403	BARREIRAS	290320	1		2	
1	PR	02RS METF	1356	CURITIBA	410690	1		2	9
1	RS	005 CRS	1608	CANELA	430440	1		2	2
1	SP	GVE XX PIR	1345	SAO PEDRO	355040	1		2	2
1	SP	GVE XXVI S	1350	MOGI GUA	353070	1		2	2
1	GO	ENTORNO	1784	FORMOSA	520800	1		2	9
1	PR	02RS METF	1356	CURITIBA	410690	1		2	2
1	SP	GVE I CAPI	1331	SAO PAULO	355030	1		2	2
1	RJ			ITABORAI	330190	1			
1	RJ			MIGUEL PE	330290	1		9	
1	RS	002 CRS	1607	PORTO ALE	431490				
1	RS	002 CRS	1607	PORTO ALE	431490	1			
1	PR	10RS CASC	1364	CASCAVEL	410480	1		2	2
1	CE	6 CRES ITA	1515	URUBURET	231380	1		2	9
1	SC	FLORIANOI	1476	FLORIANOI	420540	1		2	
1	SP	GVE I CAPI	1331	SAO PAULO	355030	1		2	2
1	CE	3 CRES MA	1513	PACATUBA	230970	1		2	2
1	SE	REGIONAL	2056	ARACAJU	280030			2	
1	MT	CUIABA	1578	CUIABA	510340			2	2
1	PR	20RS TOLEI	1374	MARECHAI	411460	1		1	9
1	RR			BOA VISTA	140010	1		2	
1	SE	REGIONAL	2056	ARACAJU	280030	1		2	2
1	BA	NUCLEO RI	1403	SAO DESID	292890	1		2	9
1	PR	13RS CIAN	1367	CIANORTE	410550	1		2	2
1	RS	001 CRS	1592	SAO LEOPC	431870	1		1	2
1	CE	16 CRES CA	1526	GRANJA	230470	2		2	
1	SP	GVE VII SAI	1332	SAO BERNA	354870	1		2	2
1	SP	GVE XXVI S	1350	ITAPIRA	352260	1		2	2
1	SP	GVE XVII C	1342	CAMPINAS	350950	1			

1	CE	1 CRES FOF	1519	FORTALEZA	230440	1			
1	SP	GVE VII SAI	1332	RIBEIRAO F	354330	1	2	2	2
1	PR	21RS TELEN	1375	CURIUVA	410700	1		2	2
1	SP	GVE VIII M	1333	GUARULHO	351880	1		2	2
1	SP	GVE I CAPI	1331	SAO PAULO	355030			2	2
1	MA	REGIONAL	1446	BACABAL	210120	1		2	2
1	PA	01 REGION	1484	ANANINDE	150080	1		2	2
1	RS	019 CRS	1606	TRES PASS	432190	1		2	2
1	AC	REGIONAL	1938	RIO BRANC	120040	1		2	2
1	TO			PALMAS	172100	1		2	2
1	RJ			RIO DE JAN	330455	1		9	9
1	AL	3 MICRORF	1535	ROTEIRO	270780	1		2	9
1	CE	1 CRES FOF	1519	FORTALEZA	230440	1		9	9
1	CE	1 CRES FOF	1519	FORTALEZA	230440	1		2	9
1	SP	GVE XVII C	1342	ITATIBA	352340	1		2	2
1	SP	GVE XVII C	1342	HORTOLAN	351907	1		2	2
1	RJ			MESQUITA	330285	1		9	9
1	MG	ALFENAS	1379	ALFENAS	310160	1		2	2
1	SP	GVE XXV S	1349	CUBATAO	351350	1		2	2
1	RJ			RIO DE JAN	330455	1			
1	GO	CENTRAL	1779	GOIANIA	520870				
1	CE	15 CRES CF	1525	NOVO ORII	230940	2		2	2
1	SP	GVE I CAPI	1331	SAO PAULO	355030	1		2	2
1	CE	6 CRES ITA	1515	TRAIRI	231350	1		9	9
1	CE	1 CRES FOF	1519	FORTALEZA	230440	1		2	9
1	CE	2 CRES CAU	1512	CAUCAIA	230370	1		9	9
1	TO			PALMAS	172100	1		2	2
1	SP	GVE XVI BC	1341	AVARE	350450	1		2	2
1	RJ			BELFORD R	330045	1			
1	MG	BELO HORI	1449	SAO JOAQU	316292	1			
1	TO			PARAISO D	171610			2	9
1	SP	GVE I CAPI	1331	SAO PAULO	355030	1		2	2
1	PR	15RS MARI	1369	MARINGA	411520	1		2	2
1	MG	DIVINOPOI	1472	FORMIGA	312610	1		2	2
1	MG	BELO HORI	1449	BETIM	310670	1		9	9
1	MG	BELO HORI	1449	BELO HORI	310620	1			
1	RO	VI GRS POF	1483	PORTO VEL	110020			2	2
1	RJ			SAO JOAO	330510	1		2	9
1	SP	GVE XXVII S	1351	SAO JOSE D	354990			2	2
1	MG	MONTES C	1473	MONTES C	314330	1		2	2
1	PB	III NRS CAN	1421	MASSARAN	250920	1		9	9
1	PI	8 GRS OEIR	1887	OEIRAS	220700	1		2	2
1	RJ			RIO BONIT	330430	2		2	2
1	MS	EX ARS DE	1975	FIGUEIRAC	500390	1		2	2
1	SP	GVE I CAPI	1331	SAO PAULO	355030	1		2	2
1	SP	GVE VII SAI	1332	SAO BERNA	354870	1		2	2
1	MS	EX NRS DE	6256	CORUMBA	500320	1		2	2

1	RJ			PARAIBA D	330370				
1	SP	GVE XI ARA	1336	PENAPOLIS	353730	1		2	2
1	CE	11 CRES SC	1521	SANTA QU	231220	1		2	9
1	RJ			SILVA JARD	330560	1		2	2
1	GO	CENTRAL	1779	TRINDADE	522140	1		2	2
1	PR	10RS CASC	1364	CASCAVEL	410480	1		2	2
1	MT	TANGARA	1585	TANGARA	510795			2	2
1	DF			TAGUATIN	530180	9		9	9
1	SP	GVE XVII C/	1342	CAMPINAS	350950			2	2
1	SP	GVE XXI PR	1346	PRESIDENT	354140	1		2	2
1	MT	DIAMANTI	1586	NOVA MAI	510890	1		2	2
1	PA	03 REGION	1486	CURUCA	150290	2		2	2
1	RS	013 CRS	1609	VENANCIO	432260	1			
1	MG	BELO HORI	1449	CONTAGEN	311860	1		9	9
1	TO			PALMAS	172100	1		2	9
1	RJ			SAO GONC	330490	1		2	2
1	SP	GVE XIX M/	1344	OSVALDO (	353460	1		2	2
1	SP	GVE XVII C/	1342	CAMPINAS	350950	1		2	2
1	SP	GVE I CAPI	1331	SAO PAULO	355030	1		2	2
1	PR	02RS METF	1356	CURITIBA	410690	1		2	9
1	CE	14 CRES TA	1524	PARAMBU	231030	1	1	2	9
1	PR	15RS MARI	1369	MARINGA	411520	1		2	2
1	MT	CUIABA	1578	VARZEA GF	510840	1		2	2
1	RS	013 CRS	1609	RIO PARDC	431570	1			
1	ES	METROPOI	1510	VITORIA	320530			2	2
1	SC	JOINVILLE	1565	ARAQUARI	420130	1		2	2
1	MT	CUIABA	1578	VARZEA GF	510840			2	2
1	GO	PIRINEUS	1792	ANAPOLIS	520110	1		2	2
1	PR	19RS JACAI	1373	JABOTI	411170	1		2	2
1	MG	DIVINOPOI	1472	PITANGUI	315140	1		2	2
1	MA	REGI0NAL	1430	SAO JOSE D	211120			2	2
1	SP	GVE VII SAI	1332	DIADEMA	351380	1		2	2
1	MG	UNAI	1463	PARACATU	314700	1		2	9
1	MG	BELO HORI	1449	CONTAGEN	311860	1		2	2
1	SC	JOINVILLE	1565	JOINVILLE	420910	1		2	2
1	PR	15RS MARI	1369	SAO JORGE	412530	1		2	2
1	MT	SINOP	1584	SORRISO	510792	1		2	2
1	PR	14RS PARA	1368	PARANAV/	411840	1		2	2
1	AM	ENTORNO	5584	CAREIRO	130110	2		2	1
1	AM	ENTORNO	5584	MANAUS	130260	1			2
1	MG	JUIZ DE FO	1452	JUIZ DE FO	313670	1	2	2	2
1	DF			BRASILIA -	530005	9		2	2
1	SP	GVE XXIV R	1348	SAO SIMAC	355090	1		2	2
1	BA	NUCLEO RI	1380	SALVADOR	292740		2	2	2
1	SP	GVE XVII C/	1342	HORTOLAN	351907	1		2	2
1	BA	NUCLEO RI	1385	CAMAMU	290580	9		2	9
1	RJ			NITEROI	330330	1		2	2

1	GO	CENTRAL	1779	GOIANIA	520870	1		2	2
1	SP	GVE I CAPIT	1331	SAO PAULO	355030	1		2	2
1	PR	09RS FOZ D	1363	FOZ DO IGU	410830	1		2	2
1	MG	PEDRA AZU	1457	ALMENARA	310170	1		2	2
1	RS	001 CRS	1592	TRIUNFO	432200	1		2	2
1	GO	PIRINEUS	1792	ANAPOLIS	520110	1		2	2
1	SP	GVE VII SA	1332	MAUA	352940	1	1	2	9
1	AP			MACAPA	160030	1		2	2
1	GO	ENTORNO	1782	VALPARAIS	522185	1		2	9
1	MG	SETE LAGO	1467	CURVELO	312090	1		2	2
1	RJ			RIO DE JAN	330455	1		2	9
1	MG	UBERLAND	1462	UBERLAND	317020	1		2	2
1	PB	I NRS JOAO	1377	JOAO PESS	250750			2	9
1	RJ			SAO GONC	330490	1		2	9
1	MA	REGIONAL	1445	JENIPAPO	210547	2		2	2
1	RS	001 CRS	1592	ESTEIO	430770	1		2	2
1	PR	08RS FRAN	1362	DOIS VIZIN	410720	1		2	2
1	TO			PALMAS	172100	1		2	9
1	PR	17RS LOND	1371	CAMBE	410370	1		2	2
1	SP	GVE XXII PI	1573	MIRANTE D	353020	1		2	2
1	RO	VI GRS POR	1483	PORTO VEL	110020	2			2
1	SP	GVE I CAPIT	1331	SAO PAULO	355030	1		2	2
1	SP	GVE X OSA	1335	SANTANA	354730	1		2	2
1	MT	TANGARA	1585	CAMPO NO	510263			2	2
1	SP	GVE XVII CA	1342	CAMPINAS	350950	1		2	2
1	SP	GVE XII ARA	1337	SAO CARLO	354890	1		2	2
1	SP	GVE XVII CA	1342	JAGUARIU	352470	1		2	2
1	MT	TANGARA	1585	TANGARA	510795			2	2
1	RS	002 CRS	1607	PORTO ALE	431490			2	9
1	RS	002 CRS	1607	PORTO ALE	431490				
1	BA	NUCLEO RE	1388	PORTO SEG	292530	9		2	9
1	MG	DIVINOPOL	1472	SANTO ANT	316040	1		2	2
1	RJ			NOVA FRIB	330340	1		2	9
1	BA	NUCLEO RE	1380	SALVADOR	292740	1		2	
1	SP	GVE XXIX S	1354	SAO JOSE D	354980	1		2	2
1	MT	SINOP	1584	IPIRANGA	510452			2	2
1	MG	DIVINOPOL	1472	PITANGUI	315140	1		2	2
1	SP	GVE XXVI S	1350	MOJI MIRI	353080	1		2	2
1	PE	001	1497	RECIFE	261160	1		2	9
1	CE	1 CRES FOR	1519	FORTALEZA	230440	1		2	9
1	SP	GVE XXXI S	1353	SALTO DE P	354530	2		2	2
1	MT	SINOP	1584	SINOP	510790	1		2	
1	PR	10RS CASC	1364	CASCAVEL	410480	1		2	2
1	MG	LEOPOLDIN	1453	RECREIO	315410	3		2	2
1	CE	1 CRES FOR	1519	FORTALEZA	230440	1		2	2
1	RR			BOA VISTA	140010	1		2	2
1	GO	ESTRADA D	1802	CATALAO	520510	1		2	2

1	RJ			RIO DE JAN	330455	1		2	2
1	TO			PORTO NA	171820	1		2	9
1	MG	POUSO ALE	1459	POUSO ALE	315250	1		2	2
1	CE	3 CRES MA	1513	MARACAN	230765	1		2	9
1	SC	BLUMENAU	1564	BLUMENAU	420240	1		2	2
1	BA	NUCLEO RE	1403	LUIS EDUA	291955			9	9
1	MT	CUIABA	1578	CUIABA	510340			2	2
1	SP	GVE XXVI S	1350	VARGEM G	355640	1		2	2
1	PE	001	1497	CABO DE S	260290	1		9	9
1	DF			CEILANDIA	530040	1		2	9
1	GO	SERRA DA I	1790	NIQUELAN	521460	1		9	9
1	RS	006 CRS	1611	MARAU	431180	1		2	2
1	MT	CUIABA	1578	VARZEA GR	510840	1		2	2
1	SP	GVE XVII CA	1342	JUNDIAI	352590			2	2
1	PB	XII NRS ITA	1428	PEDRAS DE	251120	1		2	2
1	MG	BELO HORI	1449	BETIM	310670	1		9	9
1	RO	I GRS JI PA	1474	JI-PARANA	110012	1		2	2
1	AL	5 MICRORE	1537	ARAPIRACA	270030			2	2
1	PR	15RS MARI	1369	SARANDI	412625	1		2	2
1	SP	GVE XVII CA	1342	COSMOPO	351280	1		2	2
1	PA	03 REGION	1486	CASTANHA	150240	2		2	
1	RS	018 CRS	1605	CAPAO DA	430463	1		1	3
1	MG	JUIZ DE FO	1452	JUIZ DE FO	313670			2	9
1	MG	BELO HORI	1449	BELO HORI	310620	1		2	2
1	SP	GVE XVII CA	1342	SANTA BAR	354580	1		2	2
1	MT	CACERES	1581	MIRASSOL	510562			2	2
1	GO	OESTE I	1795	BOM JARD	520340	1		2	2
1	SP	GVE XVII CA	1342	MONTE MO	353180	1			2
1	SP	GVE XX PIR	1345	RIO CLARO	354390	1		2	2
1	RN	V URSAP	1415	SANTA CRU	241120	1		2	2
1	RJ			RIO DE JAN	330455	1		2	9
1	PI			SEBASTIAO	221063	2		2	2
1	SC	CRICIUMA	1558	ICARA	420700			2	2
1	CE	1 CRES FOR	1519	FORTALEZA	230440	1		2	9
1	SC	XANXERE	1556	XANXERE	421950	1		2	9
1	RS	014 CRS	1602	BOA VISTA	430220	1		2	9
1	PB	VI NRS PAT	1422	PATOS	251080	1		2	2
1	SP	GVE XXXI S	1353	ARACOIAB	350290	1		2	9
1	TO			PORTO NA	171820	1		2	2
1	RJ			RIO DE JAN	330455	1		2	2
1	SP	GVE XXI PR	1346	PRESIDENT	354140	1		2	2
1	MT	ALTA FLOR	1587	NOVA BAN	510615	2		2	2
1	SP	GVE I CAPIT	1331	SAO PAULO	355030	1		2	9
1	MS	EX ARS DE	1975	MARACAJU	500540	1		2	2
1	MS	NRS JARDII	5546	BONITO	500220	1			
1	MG	BELO HORI	1449	MATEUS LE	314070	1	9	9	9
1	RS	002 CRS	1607	CHARQUEA	430535				

1	SP	GVE XIX MA	1344	MARILIA	352900	1		2	2
1	GO	ENTORNO	1782	VALPARAIS	522185	1		2	2
1	MT	CUIABA	1578	CUIABA	510340	1		2	2
1	RJ			SAO GONC	330490	1		9	9
1	PR	15RS MARI	1369	MARINGA	411520	1		2	2
1	SC	JOINVILLE	1565	JOINVILLE	420910	1		2	2
1	PR	17RS LOND	1371	CAMBE	410370	1		2	2
1	MT	PORTO ALE	1583	PORTO ALE	510677	2		2	2
1	PR	14RS PARA	1368	PARANAVA	411840	1		2	2
1	SP	GVE XXXI S	1353	SALTO	354520	9		2	9
1	BA	NUCLEO RE	1393	MACURUR	291990	9		9	9
1	RS	002 CRS	1607	GUAIBA	430930	1		2	2
1	GO	OESTE II	1797	SAO LUIS D	522010	1		1	9
1	MG	ALFENAS	1379	GUAXUPE	312870	1		2	2
1	RJ			RIO DE JAN	330455	1		9	9
1	PA	12 REGION	1495	SANTANA I	150670	1			
1	RN	I URSAP	1411	PEDRO VEL	240980	1		2	2
1	RR			BONFIM	140015	1		2	2
1	RS	002 CRS	1607	GRAVATAI	430920	1		2	2
1	SC	JOINVILLE	1565	JOINVILLE	420910	1		2	2
1	SC	FLORIANOI	1476	FLORIANOI	420540	1			
1	AM	BAIXO AMA	5589	MAUES	130290	2		2	2
1	SP	GVE VII SAI	1332	SAO BERNA	354870	1	2	2	2
1	SC	JARAGUA D	1567	JARAGUA D	420890	1		2	2
1	GO	CENTRAL	1779	GOIANIA	520870			2	2
1	PR	02RS METR	1356	CURITIBA	410690	1	2	2	2
1	RS	001 CRS	1592	SAO PEDRO	431935	1		2	2
1	SP	GVE I CAPIT	1331	SAO PAULO	355030	9		2	9
1	BA	NUCLEO RE	1398	BARRA DA	290280			9	9
1	MG	PATOS DE I	1456	PATOS DE I	314800	1		9	
1	PR	15RS MARI	1369	SARANDI	412625	1		2	2
1	SP	GVE XII ARA	1337	MATAO	352930	1		2	2
1	SP	GVE XXIV R	1348	JARDINOPO	352510	1		2	2
1	SP	GVE XXVI S	1350	MOGI GUA	353070	1		2	2
1	RS	002 CRS	1607	VIAMAO	432300	1		2	2
1	CE	1 CRES FOR	1519	FORTALEZA	230440	1		9	9
1	SC	CHAPECO	1553	SAO LOURI	421690	1		2	2
1	PB	I NRS JOAO	1377	CABEDELO	250320	1		2	2
1	PR	17RS LOND	1371	LONDRINA	411370	1		2	2
1	CE	11 CRES SO	1521	MASSAPE	230800	1		2	2
1	SP	GVE XVII CA	1342	CAMPINAS	350950	1		2	2
1	MT	CUIABA	1578	CUIABA	510340	1		2	2
1	PR	20RS TOLEI	1374	TOLEDO	412770	1	2	2	2
1	GO	PIRINEUS	1792	ANAPOLIS	520110	1		2	2
1	SC	BLUMENAU	1564	INDAIAL	420750	1		2	2
1	RJ			CAMPOS D	330100	1		2	2
1	PR	09RS FOZ D	1363	FOZ DO IGU	410830	1		2	2

1	PR	08RS FRAN	1362	SANTO AN	412440	1		2	2
1	SP	GVE XXXI S	1353	SOROCABA	355220	1	9	9	9
1	SP	GVE VII SA	1332	SAO BERN	354870	1		2	2
1	SP	GVE XXXI S	1353	SOROCABA	355220	1		2	2
1	ES	METROPOI	1510	SERRA	320500	1		2	2
1	PA	09 REGION	1492	SANTAREM	150680	1	1	2	2
1	SP	GVE XXIX S	1354	SAO JOSE D	354980	1		2	2
1	SP	GVE I CAPI	1331	SAO PAUL(	355030	1		2	2
1	SP	GVE XXI PR	1346	PRESIDENT	354140	1		2	2
1	MT	BARRA DO	1580	BARRA DO	510180	1		2	2
1	ES	CACHOEIR(	1511	MARATAIZ	320332	1		2	2
1	SP	GVE I CAPI	1331	SAO PAUL(	355030	1		2	9
1	SP	GVE I CAPI	1331	SAO PAUL(	355030	1		2	2
1	SP	GVE XXXI S	1353	VOTORANT	355700	1		2	2
1	DF			TAGUATIN	530180	1		2	9
1	SP	GVE XV BA	1340	BAURU	350600	1		2	2
1	SP	GVE I CAPI	1331	SAO PAUL(	355030	1		2	2
1	SP	GVE I CAPI	1331	SAO PAUL(	355030	1		2	2
1	GO	CENTRO SU	1781	APARECID/	520140			2	9
1	PR	17RS LOND	1371	IBIPORA	410980	1		2	2
1	RJ			PATY DO A	330385			2	2
1	PR	15RS MARI	1369	NOVA ESPI	411690	1		2	2
1	GO	CENTRO SU	1781	APARECID/	520140	1		2	2
1	RN	V URSAP	1415	BOM JESUS	240170	1		2	9
1	RS	002 CRS	1607	PORTO ALE	431490	1		2	2
1	RS	008 CRS	1597	CACAPAVA	430280	1		2	2
1	SP	GVE I CAPI	1331	SAO PAUL(	355030	1		2	2
1	MS	NRS DE DO	1966	DOURADO	500370	1			
1	SE	REGIONAL	2056	SAO CRIST(	280670	1		2	2
1	GO	CENTRAL	1779	GOIANIA	520870	1		2	2
1	SC	BLUMENAU	1564	BLUMENAU	420240	1		2	2
1	SP	GVE XXVI S	1350	MOGI GUA	353070	1		2	2
1	SP	GVE XVIII F	1343	GUARA	351770	1			
1	SP	GVE X OSA	1335	EMBU DAS	351500	1		2	
1	RJ			RIO DE JAN	330455				
1	RS	008 CRS	1597	ARROIO DO	430120	1		2	2
1	SP	GVE I CAPI	1331	SAO PAUL(	355030	1		2	9
1	RJ			VOLTA RED	330630	1			2
1	MG	CORONEL I	1468	CARATING/	311340	1		2	2
1	RO	I GRS JI PA	1474	JI-PARANA	110012	1		2	1
1	CE	3 CRES MA	1513	MARACAN	230765	1		9	9
1	SP	GVE VIII M	1333	GUARULH(	351880	1		2	2
1	MA	REGI0NAL	1430	SAO JOSE D	211120	1		2	2
1	SP	GVE X OSA	1335	EMBU-GU/	351510	9		2	9
1	MG	PASSOS	1455	CAPITOLIO	311280	1		2	2
1	GO	CENTRAL	1779	ANICUNS	520130	1		2	2
1	MT	CUIABA	1578	VARZEA GI	510840	1		2	2

1	SC	JOINVILLE	1565	JOINVILLE	420910	1		2	2
1	PR	16RS APUC	1370	APUCARAN	410140	1		2	2
1	RJ			SANTO ANT	330470	1		2	2
1	RS	006 CRS	1611	PASSO FUN	431410	1		2	2
1	RS	002 CRS	1607	PORTO ALE	431490			2	
1	SC	CRICIUMA	1558	CRICIUMA	420460	1		2	2
1	PR	10RS CASC	1364	CASCAVEL	410480	1		2	2
1	SP	GVE I CAPI	1331	SAO PAUL	355030	1		2	9
1	TO			PALMAS	172100	1		2	2
1	SE	REGIONAL	2056	ARACAJU	280030	1		2	2
1	PR	02RS METR	1356	CAMPINA	410400	2		2	2
1	GO	CENTRAL	1779	GOIANIA	520870	1		2	2
1	MT	TANGARA	1585	TANGARA	510795			2	2
1	RO	VI GRS POR	1483	PORTO VEL	110020	1		2	2
1	MG	UBERLAND	1462	UBERLAND	317020	1		2	2
1	SP	GVE I CAPI	1331	SAO PAUL	355030	1		2	2
1	SP	GVE XXV S	1349	GUARUJA	351870	1		2	2
1	SP	GVE I CAPI	1331	SAO PAUL	355030	1		2	2
1	BA	NUCLEO RE	1380	SALVADOR	292740	1		2	2
1	MG	BELO HORI	1449	BELO HORI	310620	1		2	2
1	SP	GVE VIII M	1333	MOGI DAS	353060	1		2	2
1	RJ			RIO DE JAN	330455				
1	PI			AGRICOLAI	220010	2		2	2
1	SP	GVE I CAPI	1331	SAO PAUL	355030	1		2	2
1	AM	ENTORNO	5584	RIO PRETO	130356	2	1	2	
1	SP	GVE I CAPI	1331	SAO PAUL	355030	1		2	2
1	MT	CUIABA	1578	VARZEA GF	510840	1		2	2
1	SP	GVE XIX M	1344	OSVALDO	353460	1		2	2
1	PR	02RS METR	1356	CURITIBA	410690	1		2	2
1	SP	GVE XXV S	1349	SANTOS	354850	1		2	9
1	SP	GVE I CAPI	1331	SAO PAUL	355030	1			
1	SP	GVE X OSA	1335	OSASCO	353440	1		2	2
1	SP	GVE I CAPI	1331	SAO PAUL	355030	1		2	2
1	SP	GVE I CAPI	1331	SAO PAUL	355030	1		2	2
1	MG	BELO HORI	1449	SANTA LUZ	315780	1		2	2
1	PR	03RS PONT	1357	PONTA GR	411990	1		2	2
1	SP	GVE VIII M	1333	GUARULH	351880	1			
1	SP	GVE XII AR	1337	PORTO FER	354070	9		2	9
1	PR	02RS METR	1356	CURITIBA	410690	1		2	2
1	SP	GVE XVIII F	1343	FRANCA	351620	1		2	2
1	SP	GVE I CAPI	1331	SAO PAUL	355030	1		2	2
1	MS	EX ARS DE	1975	CAMPO GF	500270	1		2	2
1	BA	NUCLEO RE	1380	SALVADOR	292740	1		9	9
1	RO	VI GRS POR	1483	PORTO VEL	110020	2		2	2
1	RS	001 CRS	1592	CANOAS	430460	1		2	2
1	PA	01 REGION	1484	ANANINDE	150080	1		2	2
1	SP	GVE VII SA	1332	SAO BERN	354870	1		2	2

1	SP	GVE XVII C/	1342	JUNDIAI	352590	1			2
1	SP	GVE X OSA	1335	OSASCO	353440	1		2	2
1	RJ			BELFORD R	330045	1			
1	MT	DIAMANTI	1586	SAO JOSE D	510730	2		2	2
1	SP	GVE XXXI S	1353	SOROCABA	355220	1		2	2
1	RJ			RIO DE JAN	330455	1	2		
1	SP	GVE XXI PR	1346	PRESIDENT	354140	1		2	2
1	SP	GVE I CAPI	1331	SAO PAUL(	355030	1			
1	RS	001 CRS	1592	SAO LEOP(	431870				
1	BA	NUCLEO RI	1380	SALVADOR	292740	1		2	2
1	ES	METROPOI	1510	SERRA	320500			2	9
1	PR	02RS METF	1356	ITAPERUCU	411125	1		2	2
1	BA	NUCLEO RI	1380	SALVADOR	292740	1		2	
1	SP	GVE XXIV F	1348	RIBEIRAO F	354340	1		2	2
1	SP	GVE XVII C/	1342	CAMPINAS	350950	1		2	9
1	DF			CEILANDIA	530040	9		2	9
1	PR	16RS APUC	1370	APUCARAN	410140	1		2	2
1	PR	19RS JACAI	1373	WENCESLA	412850	1		2	2
1	MS	EX ARS DE	1975	MARACAJU	500540	1		2	2
1	MT	TANGARA	1585	NOVA OLIN	510623	1		2	2
1	MT	SINOP	1584	NOVA MUT	510622	2		2	2
1	SP	GVE XXXI S	1353	IPERO	352100	9			
1	SP	GVE XV BA	1340	JAU	352530	1		2	2
1	SP	GVE XXVI S	1350	AGUAI	350030	1		2	9
1	GO	CENTRAL	1779	GOIANIA	520870	1		2	2
1	RN	II URSAP	1412	MOSSORO	240800	1		2	2
1	SP	GVE XIII AS	1338	OURINHOS	353470	1	2	2	2
1	DF			VICENTE PI	539934			2	9
1	RJ			VASSOURA	330620	1		2	2
1	RO	VI GRS POF	1483	PORTO VEL	110020	1		2	2
1	MT	CUIABA	1578	CUIABA	510340			2	2
1	PR	07RS PATO	1361	PATO BRAI	411850	1		2	2
1	SP	GVE I CAPI	1331	SAO PAUL(	355030			2	2
1	RR			BOA VISTA	140010	1		2	2
1	PR	19RS JACAI	1373	WENCESLA	412850	1		2	2
1	PR	17RS LOND	1371	ASSAI	410190	1		2	2
1	SP	GVE XXXI S	1353	VOTORANT	355700	1		2	2
1	RS	004 CRS	1595	SANTA MA	431690	1		2	
1	MS	EX NRS DE	6256	CORUMBA	500320	1		2	2
1	SC	BLUMENAI	1564	POMEROD	421320	1		2	2
1	SP	GVE I CAPI	1331	SAO PAUL(	355030	1		2	2
1	SP	GVE I CAPI	1331	SAO PAUL(	355030	1		2	2
1	CE	9 CRES RUS	1518	MORADA N	230870	1		9	9
1	RJ			RIO DAS OS	330452	1			
1	SP	GVE VII SAI	1332	SANTO AN	354780	1		2	2
1	PR	02RS METF	1356	CURITIBA	410690	1		2	9
1	GO	CENTRAL	1779	GOIANIA	520870	1			

1	SP	GVE XX PIR	1345	LIMEIRA	352690	1	2	2	2
1	SC	SAO MIGU	1548	SAO MIGU	421720	1		2	2
1	SP	GVE XVI BC	1341	PARANAPA	353580	2		2	2
1	MA	REGIONAL	1440	IMPERATRI	210530	1	1	2	2
1	MS	NRS DE TRI	6255	BRASILANI	500230	1		2	2
1	AM	ENTORNO	5584	BARCELOS	130040	2		2	2
1	RJ			BELFORD R	330045	1		2	9
1	SP	GVE VIII M	1333	MOGI DAS	353060	1		2	2
1	MT	CUIABA	1578	CUIABA	510340	1		2	2
1	RN	II URSAP	1412	MOSSORO	240800	1		2	2
1	MA	REGIONAL	1444	PEDREIRAS	210820	1		2	2
1	MG	DIVINOPOI	1472	CANDEIAS	311200	9			
1	GO	CENTRAL	1779	SAO FRANC	521990	1			9
1	RJ			DUQUE DE	330170	1		2	2
1	BA	NUCLEO RI	1393	FILADELFIA	291085	1			
1	TO			PALMAS	172100	1		2	2
1	SP	GVE XV BA	1340	BAURU	350600	1		2	2
1	GO	CENTRO SU	1781	APARECIDA	520140	1		2	2
1	SP	GVE I CAPIT	1331	SAO PAULO	355030	1	1	2	2
1	GO	CENTRO SU	1781	APARECIDA	520140	1		2	2
1	CE	1 CRES FOF	1519	EUSEBIO	230428	1			
1	SP	GVE XVII CA	1342	SANTA BAF	354580	1		2	2
1	PR	10RS CASC	1364	SANTA TER	412402	1		2	2
1	BA	NUCLEO RI	1380	SALVADOR	292740	1		2	9
1	SP	GVE VIII M	1333	GUARULHO	351880	1		2	2
1	SP	GVE XVII CA	1342	CAMPINAS	350950				
1	PR	20RS TOLEI	1374	ENTRE RIO	410753	2		2	2
1	PR	09RS FOZ D	1363	MEDIANEII	411580	1		2	2
1	MS	NRS DE DO	1966	DOURADIN	500350	1		9	9
1	RS	018 CRS	1605	XANGRI-LA	432380	1		2	2
1	SP	GVE I CAPIT	1331	SAO PAULO	355030	1		2	2
1	ES	METROPOI	1510	VENDA NO	320506	1		2	9
1	SP	GVE XVII CA	1342	ITATIBA	352340	1		2	9
1	SP	GVE I CAPIT	1331	SAO PAULO	355030	9		2	9
1	SP	GVE XVII CA	1342	SANTA BAF	354580	9		9	9
1	CE	3 CRES MA	1513	MARACAN	230765			2	2
1	MA	REGIONAL	1440	IMPERATRI	210530	1		2	9
1	GO	SUDOESTE	1800	JATAI	521190	1		2	2
1	PR	02RS METF	1356	FAZENDA F	410765	1		2	2
1	SP	GVE XVII CA	1342	CAMPINAS	350950	1		2	2
1	GO	ESTRADA D	1802	CATALAO	520510	1		2	2
1	SP	GVE XVII CA	1342	CAMPINAS	350950	1		2	2
1	PR	09RS FOZ D	1363	MATELANI	411560	1		2	2
1	SP	GVE X OSA	1335	TABOAO D	355280	9		2	
1	SP	GVE XXII PI	1573	MIRANTE D	353020	1		2	2
1	PR	20RS TOLEI	1374	TUPASSI	412795	2		2	2
1	RJ			RIO DE JAN	330455			2	2

1	PE	004	1499	CARUARU	260410	1		9	9
1	RS	008 CRS	1597	ENCRUZILH	430690	1		2	2
1	RJ			MARICA	330270	1			
1	RO	I GRS JI PAI	1474	OURO PRET	110015	1		2	2
1	BA	NUCLEO RI	1380	CAMACARI	290570	1		2	
1	SP	GVE XXIX S	1354	SAO JOSE D	354980	1		2	2
1	SP	GVE I CAPI	1331	SAO PAULC	355030	1		9	9
1	PR	02RS METF	1356	CURITIBA	410690	1		2	9
1	SP	GVE XXIX S	1354	SAO JOSE D	354980	1		2	2
1	SP	GVE XXIX S	1354	SAO JOSE D	354980	1		2	2
1	SP	GVE X OSA	1335	CARAPICUI	351060	1		2	2
1	CE	11 CRES SC	1521	IRAUCUBA	230610	2		2	2
1	MG	MONTES C	1473	JANAUBA	313510	1		2	2
1	RJ			NOVA IGUA	330350	1		2	9
1	SP	GVE XXIV R	1348	BATATAIS	350590	1		1	2
1	MG	MONTES C	1473	JANAUBA	313510	1		2	2
1	SP	GVE I CAPI	1331	SAO PAULC	355030	1		2	2
1	GO	CENTRAL	1779	GOIANIA	520870	1	2	2	2
1	SP	GVE XXX JA	1574	APARECIDA	350260	1		2	2
1	RN	VII URSAP	1416	PARNAMIF	240325	1		2	2
1	PA	01 REGION	1484	ANANINDE	150080	1		2	2
1	DF			SUDOESTE,	539931	9		2	2
1	CE	1 CRES FOF	1519	FORTALEZA	230440	1		9	9
1	GO	CENTRAL	1779	GOIANIA	520870	1			
1	PR	03RS PONT	1357	PONTA GR	411990			2	2
1	SP	GVE XXIX S	1354	CATANDUV	351110	1		2	2
1	BA	NUCLEO RI	1380	DIAS D'AVI	291005		9	9	9
1	MS	NRS DE DO	1966	DOURADO	500370	1		2	9
1	SP	GVE X OSA	1335	TABOAO D	355280	1		2	2
1	SP	GVE X OSA	1335	ITAPECERI	352220	9		2	9
1	RJ			RIO DE JAN	330455	1		2	2
1	CE	3 CRES MA	1513	MARANGU	230770	1		2	9
1	PR	10RS CASC	1364	CASCAVEL	410480	1			
1	CE	1 CRES FOF	1519	FORTALEZA	230440	1		9	9
1	AM	ENTORNO	5584	MANAUS	130260	1		2	2
1	SP	GVE XXXI S	1353	SOROCABA	355220	1		2	2
1	SP	GVE XVII C	1342	CAMPINAS	350950	1		2	2
1	SP	GVE X OSA	1335	OSASCO	353440	1		2	2
1	GO	SUDOESTE	1800	CAIAPONIA	520440	1		2	2
1	DF			GUARA	530070	1		2	9
1	DF	SUPERINTE	6262	SOBRADIN	530170	1		2	2
1	SP	GVE XXVII S	1351	SAO JOSE D	354990	1			
1	MT	CACERES	1581	CACERES	510250			2	2
1	AL	9 MICRORE	1541	PALMEIRA	270630	1		2	2
1	SP	GVE XXXI S	1353	SOROCABA	355220	1		2	2
1	GO	CENTRAL	1779	GOIANIA	520870	1		2	2
1	BA	NUCLEO RI	1380	MILAGRES	292130	9		2	9

1	MS	EX ARS DE	1975	CAMPO GF	500270	1		2	2
1	MT	CACERES	1581	SAO JOSE [	510710	1		2	2
1	PR	01RS PARA	1355	MORRETES	411620	1			
1	MA	REGIONAL	1440	IMPERATRI	210530	1		2	2
1	MT	CUIABA	1578	PLANALTO	510645	1		2	2
1	MG	CORONEL F	1468	IPATINGA	313130	1		9	9
1	PR	03RS PONT	1357	PONTA GR	411990	1		2	2
1	GO	CENTRAL	1779	GOIANIA	520870	1		2	2
1	SP	GVE I CAPI	1331	SAO PAULO	355030	1		2	2
1	MG	JANUARIA	1470	JANUARIA	313520			2	2
1	PR	02RS METF	1356	ALMIRANT	410040			2	2
1	MA	REGIONAL	1446	OLHO D'AG	210740	2		2	2
1	ES	METROPOL	1510	SERRA	320500			2	2
1	SP	GVE I CAPI	1331	SAO PAULO	355030	1	2	2	9
1	SP	GVE XVI BC	1341	LARANJAL	352640	1		2	2
1	PA	09 REGION	1492	BELTERRA	150145	3		2	2
1	SP	GVE XXVI S	1350	SANTA CRU	354630	1		2	2
1	MT	TANGARA	1585	TANGARA	510795	1		2	2
1	RS	001 CRS	1592	CANOAS	430460	1		2	2
1	CE	3 CRES MA	1513	BARREIRA	230195	1		9	9
1	PE	002	1507	CUMARU	260490	1		2	2
1	MS	EX ARS DE	1975	CAMPO GF	500270	1		2	2
1	CE	22 CRES CA	5596	OCARA	230945	1		9	9
1	MG	LEOPOLDIN	1453	LARANJAL	313800	1		2	2
1	PI			BARREIRAS	220130	1		2	9
1	PR	02RS METF	1356	COLOMBO	410580	1		2	2
1	RJ			SAO JOAO	330500	3			
1	RS	004 CRS	1595	SANTA MA	431690	1		2	2
1	TO			AXIXA DO T	170290	2		9	9
1	SP	GVE VIII M	1333	BIRITIBA-M	350660	1		2	2
1	SC	CHAPECO	1553	QUILOMBO	421420	1	2	2	2
1	MT	CUIABA	1578	CUIABA	510340			2	2
1	AM	ENTORNO	5584	MANAUS	130260	1		2	2
1	RJ			QUEIMADO	330414	1		2	
1	SP	GVE XVIII F	1343	FRANCA	351620	1		2	2
1	SP	GVE XXXI S	1353	SOROCABA	355220	1		9	9
1	CE	17 CRES IC	1527	LAVRAS DA	230750	1		9	9
1	SP	GVE X OSA	1335	CARAPICUI	351060				
1	SP	GVE VIII M	1333	GUARULHO	351880	1		2	2
1	SP	GVE I CAPI	1331	SAO PAULO	355030	1		2	2
1	PR	09RS FOZ D	1363	FOZ DO IGU	410830	1		2	2
1	RJ			CAMPOS D	330100	1		2	2
1	GO	ENTORNO	1784	FORMOSA	520800	1		2	2
1	RJ			RIO DE JAN	330455				
1	PE	006	1501	CUSTODIA	260510	1		9	9
1	CE	15 CRES CF	1525	QUITERIAN	231126	1		2	9
1	MA	REGIONAL	1433	CHAPADIN	210320	1			

1	GO	CENTRAL	1779	GOIANIA	520870	1			
1	PE	001	1497	RECIFE	261160	1		2	2
1	CE	2 CRES CAU	1512	SAO GONC	231240				
1	GO	ENTORNO	1784	FORMOSA	520800	1		2	2
1	RJ			CAMPOS D	330100			2	2
1	BA	NUCLEO RE	1403	BARRA	290270			9	9
1	CE	1 CRES FOR	1519	FORTALEZA	230440	1		2	2
1	PR	02RS METR	1356	CURITIBA	410690	1		2	2
1	SP	GVE XXVIII	1576	CARAGUAT	351050	1		2	2
1	SC	JOINVILLE	1565	JOINVILLE	420910	1		2	2
1	RJ			VOLTA RED	330630				
1	SP	GVE I CAPI	1331	SAO PAULO	355030	1		2	2
1	CE	1 CRES FOR	1519	FORTALEZA	230440	1		2	9
1	SP	GVE XXIX S	1354	MIRASSOL	353030	1		2	2
1	RS	019 CRS	1606	TENENTE P	432140	1		2	2
1	ES	METROPOL	1510	SERRA	320500	1			9
1	SP	GVE XXIX S	1354	PONTES GE	354030	1		2	2
1	MG	GOVERNAD	1471	NOVA BELE	314467			2	9
1	PR	17RS LOND	1371	LONDRINA	411370	1		2	2
1	DF			VICENTE PI	539934	1		2	9
1	SP	GVE XXVI S	1350	MOGI GUA	353070	1		2	2
1	MS	NRS DE TRI	6255	AGUA CLAI	500020	2	9	9	9
1	MG	CORONEL F	1468	DIONISIO	312180	1	2	2	2
1	SP	GVE X OSA	1335	OSASCO	353440	1	2	2	2
1	MG	VARGINHA	1466	BOA ESPER	310710	1	2	2	2
1	MT	CUIABA	1578	CUIABA	510340	1			2
1	SP	GVE VIII M	1333	MOGI DAS	353060	1		2	2
1	SP	GVE X OSA	1335	JUQUITIBA	352620	1		2	2
1	RN	VII URSAP	1416	NATAL	240810	1		2	9
1	MT	CACERES	1581	CACERES	510250			2	2
1	MG	BELO HORI	1449	BELO HORI	310620	1		2	2
1	SP	GVE XI ARA	1336	PENAPOLIS	353730	1		2	2
1	PA	03 REGION	1486	CASTANHA	150240	1		9	9
1	CE	6 CRES ITA	1515	TRAIRI	231350	1		9	9
1	MT	PONTES E L	1591	PONTES E L	510675			2	2
1	SP	GVE I CAPI	1331	SAO PAULO	355030	1			
1	RJ			RIO DE JAN	330455	1		2	9
1	AM	ENTORNO	5584	MANAUS	130260	1			2
1	PA	08 REGION	1491	BREVES	150180	2		2	2
1	GO	CENTRAL	1779	GOIANIA	520870	1		2	2
1	SP	GVE I CAPI	1331	SAO PAULO	355030			2	2
1	SP	GVE I CAPI	1331	SAO PAULO	355030			2	2
1	SP	GVE VIII M	1333	GUARULHO	351880	1		2	2
1	SP	GVE XXIX S	1354	CEDRAL	351130	1		2	2
1	SP	GVE XXIX S	1354	NHANDEAI	353260	1		2	2
1	RJ			NOVA IGUA	330350			2	9
1	SP	GVE XXII PI	1573	DRACENA	351440	1		2	2

1	MG	SETE LAGO	1467	CAETANOF	310990	1		9	2
1	PB	XI NRS PRII	1427	SAO JOSE [	251455	2		2	2
1	PR	18RS CORN	1372	NOVA FATI	411700	1		2	2
1	PB	III NRS CAN	1421	CAMPINA (	250400	1		2	2
1	RJ			SAO JOAO	330510				
1	SP	GVE XXV S/	1349	GUARUJA	351870	1		2	2
1	BA	NUCLEO RI	1380	SANTO AN	292870	1		9	
1	RS	001 CRS	1592	PORTAO	431480	2		2	
1	AM	ENTORNO	5584	MANAUS	130260	1	1	2	2
1	AC	REGIONAL	1940	TARAUACA	120060			2	2
1	SP	GVE I CAPI	1331	SAO PAULC	355030	1		2	2
1	RS	008 CRS	1597	CACHOEIR/	430300				
1	SP	GVE I CAPI	1331	SAO PAULC	355030	1			
1	SP	GVE I CAPI	1331	SAO PAULC	355030	1		2	2
1	SP	GVE I CAPI	1331	SAO PAULC	355030	1		9	9
1	SP	GVE XXIX S	1354	PARAISO	353570	1		2	2
1	MA	REGIONAL	1444	PEDREIRAS	210820	1		2	9
1	SP	GVE VII SAI	1332	SAO BERN/	354870				
1	PB	I NRS JOAC	1377	SANTA RIT/	251370	1		2	2
1	BA	NUCLEO RI	1381	CONCEICA(	290840			9	9
1	RO	VI GRS POF	1483	PORTO VEI	110020	2		2	2
1	SP	GVE I CAPI	1331	SAO PAULC	355030	1	1	2	2
1	PR	02RS METF	1356	CURITIBA	410690	1		2	2
1	SP	GVE I CAPI	1331	SAO PAULC	355030	1		2	2
1	MT	CUIABA	1578	CUIABA	510340	1		2	2
1	MG	MONTES C	1473	MONTES C	314330	1		2	2
1	RN	VII URSAP	1416	NATAL	240810	1		2	2
1	CE	1 CRES FOF	1519	FORTALEZ/	230440	1		2	2
1	MG	ITABIRA	1451	BARAO DE	310540	1		2	2
1	MT	SINOP	1584	SINOP	510790	1		2	2
1	GO	CENTRAL	1779	GOIANIA	520870	1		2	2
1	BA	NUCLEO RI	1398	GUANAMB	291170	1		2	2
1	MG	POUSO ALI	1459	OURO FIN(	314600	1		2	2
1	SP	GVE I CAPI	1331	SAO PAULC	355030			2	2
1	MS	NRS DE AQ	1964	AQUIDAU/	500110	2		2	2
1	RJ			RIO DE JAN	330455	1	9	9	9
1	PR	10RS CASC	1364	CORBELIA	410630	1		2	2
1	PR	10RS CASC	1364	NOVA AUR	411670	1			
1	SP	GVE I CAPI	1331	SAO PAULC	355030	1		2	9
1	SP	GVE XXVII S	1351	JACAREI	352440	1		2	2
1	PB	I NRS JOAC	1377	JOAO PESS	250750	1		2	2
1	SP	GVE XXXIII	1352	TAUBATE	355410	9			
1	SP	GVE X OSA	1335	OSASCO	353440	1		2	2
1	SP	GVE XVII C/	1342	VALINHOS	355620	1		9	9
1	SP	GVE I CAPI	1331	SAO PAULC	355030	1		2	2
1	SP	GVE I CAPI	1331	SAO PAULC	355030	1		2	2
1	SP	GVE VIII M	1333	ITAQUAQU	352310			2	2

1	PR	10RS CASC	1364	CASCAVEL	410480	2		2	2
1	GO	CENTRAL	1779	GOIANIA	520870	1		2	2
1	AM	ENTORNO	5584	MANAUS	130260	1	1		
1	PR	15RS MARI	1369	MARINGA	411520	1	2	2	2
1	PB	I NRS JOAC	1377	SANTA RIT/	251370	1		2	2
1	MG	POUSO ALE	1459	EXTREMA	312510	1		2	2
1	RJ			MESQUITA	330285	1		2	2
1	CE	1 CRES FOF	1519	FORTALEZ/	230440	1			
1	MG	JUIZ DE FO	1452	MARIPA DI	314020	1		2	9
1	SP	GVE XIV BA	1339	COLOMBIA	351210	1		2	2
1	SP	GVE VIII M	1333	ITAQUAQU	352310			2	2
1	SC	BLUMENAU	1564	GASPAR	420590	1		2	2
1	SP	GVE XII AR/	1337	ARARAQU/	350320	1		2	2
1	SP	GVE VII SAI	1332	MAUA	352940	1		2	2
1	RJ			ANGRA DC	330010	1			
1	SE	REGIONAL	2058	AREIA BRA	280050	1			
1	RJ			RIO DE JAN	330455	1		2	9
1	BA	NUCLEO RI	1403	PARATING/	292370	2			
1	SP	GVE XVII C/	1342	CAMPINAS	350950			2	2
1	RJ			VOLTA REC	330630			9	9
1	PR	14RS PARA	1368	SANTA ISA	412370	1		2	2
1	MA	REGIONAL	1437	SANTA INE	210990	1		2	2
1	PR	16RS APUC	1370	ARAPONG/	410150	1		1	
1	SP	GVE I CAPI	1331	SAO PAULC	355030	1		2	2
1	PR	02RS METF	1356	COLOMBO	410580	1		2	2
1	RJ			NOVA IGU/	330350	1			
1	MT	CUIABA	1578	VARZEA GI	510840	1		2	2
1	SP	GVE XXIX S	1354	JOSE BONII	352570	1		2	2
1	SP	GVE XI AR/	1336	PENAPOLIS	353730	1		2	2
1	PB	I NRS JOAC	1377	JOAO PESS	250750	1		2	2
1	MG	BELO HORI	1449	CONTAGEN	311860	1		2	2
1	SC	JOINVILLE	1565	JOINVILLE	420910	1		2	2
1	SP	GVE XXV S/	1349	SANTOS	354850	1			
1	SP	GVE XVII C/	1342	SANTA BAF	354580	1		2	9
1	SC	JARAGUA I	1567	GUARAMIF	420650	1		2	2
1	SP	GVE I CAPI	1331	SAO PAULC	355030	1		2	2
1	PA	01 REGION	1484	BELEM	150140	1		2	2
1	RJ			RIO DE JAN	330455	1		9	9
1	RO	I GRS JI PAI	1474	PRESIDENT	110025	1		2	2
1	MG	BELO HORI	1449	BETIM	310670	1		2	2
1	PR	02RS METF	1356	ALMIRANT	410040	1	2	2	9
1	SP	GVE I CAPI	1331	SAO PAULC	355030	1		2	2
1	GO	CENTRAL	1779	GOIANIA	520870	1		2	2
1	SP	GVE XIX M/	1344	MARILIA	352900	1		2	2
1	SP	GVE XXXI S	1353	SOROCABA	355220	1		9	9
1	SP	GVE I CAPI	1331	SAO PAULC	355030	9		2	2
1	GO	RIO VERMI	1794	ITAPURAN	521120	1			

1	PI	10 GRS FLC	1889	FLORIANO	220390	1		2	2
1	GO	CENTRAL	1779	GOIANIA	520870	1		2	2
1	RJ			ITAPERUN/	330220	1		1	1
1	SP	GVE I CAPI	1331	SAO PAULC	355030	1		2	
1	SP	GVE XIII AS	1338	PARAGUAC	353550	1		2	2
1	SP	GVE VII SAI	1332	DIADEMA	351380			2	2
1	SP	GVE I CAPI	1331	SAO PAULC	355030	1		9	9
1	PB	I NRS JOAC	1377	JOAO PESS	250750	1		2	2
1	CE	1 CRES FOF	1519	FORTALEZ/	230440		2		2
1	CE	9 CRES RUS	1518	MORADA N	230870				
1	SP	GVE X OSA	1335	EMBU-GU/	351510	1		2	2
1	RJ			MIRACEM/	330300	9		9	9
1	SP	GVE XX PIR	1345	ARARAS	350330	1		2	2
1	RS	008 CRS	1597	NOVO CAB	431339				
1	RS	001 CRS	1592	ESTANCIA '	430760	1		2	2
1	TO			BURITI DO	170380	1		2	2
1	SP	GVE X OSA	1335	VARGEM G	355645	1		2	2
1	BA	NUCLEO RI	1380	SALVADOR	292740	1	2	2	9
1	PA	12 REGION	1495	OURILAND	150543	1		2	2
1	SP	GVE I CAPI	1331	SAO PAULC	355030	1		2	2
1	SP	GVE I CAPI	1331	SAO PAULC	355030			2	
1	RR			CANTA	140017	1		2	9
1	BA	NUCLEO RI	1380	LAURO DE	291920	1		2	9
1	SP	GVE X OSA	1335	OSASCO	353440	1		2	2
1	RS	001 CRS	1592	SANTA MA	431695	1		2	2
1	MG	DIAMANTI	1450	COUTO DE	312010	1		9	9
1	PI			CAPITAO G	220245	2		2	2
1	SP	GVE I CAPI	1331	SAO PAULC	355030	1		2	2
1	PE	001	1497	JABOATAO	260790	1			
1	MS	NRS DE DO	1966	RIO BRILH/	500720	1		2	2
1	MG	DIVINOPOI	1472	NOVA SERI	314520	1		2	2
1	MA	REGIONAL	1434	CODO	210330	1		2	2
1	SP	GVE XVI BC	1341	LARANJAL	352640	1		2	2
1	SP	GVE XX PIR	1345	RIO DAS PE	354400	1		2	2
1	AM	ENTORNO	5584	MANAUS	130260	1	2	2	2
1	GO	CENTRAL	1779	GOIANIA	520870	1		2	2
1	SP	GVE X OSA	1335	COTIA	351300	1		2	2
1	SP	GVE XXI PR	1346	PRESIDENT	354140	1		2	2
1	RN	VII URSAP	1416	NATAL	240810	1		2	2
1	RJ			CAMPOS D	330100	1		2	2
1	MG	BELO HORI	1449	VESPASIAN	317120			2	2
1	PR	10RS CASC	1364	IGUATU	411005	1		2	2
1	MG	BELO HORI	1449	BELO HORI	310620				
1	MG	BELO HORI	1449	CONTAGEN	311860	1		9	9
1	PR	20RS TOLEI	1374	PALOTINA	411790			2	2
1	SP	GVE XVII C/	1342	ITATIBA	352340	1		2	2
1	GO	ESTRADA D	1802	MARZAGA(	521290	1		2	2

1	RJ			RIO DE JAN	330455	1		2	2
1	MG	UBERLAND	1462	UBERLAND	317020	1		2	2
1	SP	GVE XXXI S	1353	SOROCABA	355220	1		1	2
1	CE	17 CRES IC	1527	CEDRO	230380	1		9	9
1	SP	GVE I CAPI	1331	SAO PAULO	355030	1		2	2
1	SP	GVE I CAPI	1331	SAO PAULO	355030	1		2	2
1	GO	SUDOESTE	1799	ITAJA	521080	1		2	2
1	SP	GVE XXXI S	1353	GUAREI	351850	1		2	2
1	SP	GVE I CAPI	1331	SAO PAULO	355030	1		2	9
1	PR	17RS LOND	1371	LONDRINA	411370	1		2	2
1	PR	10RS CASC	1364	CASCAVEL	410480				
1	PR	02RS METR	1356	CURITIBA	410690	1		2	2
1	SP	GVE XI ARA	1336	PENAPOLIS	353730	1		2	2
1	MT	TANGARA	1585	TANGARA	510795	1		2	2
1	GO	SAO PATRI	6252	BARRO ALT	520320	1		2	2
1	SP	GVE I CAPI	1331	SAO PAULO	355030	1		2	2
1	PE	004	1499	BREJO DA I	260260	1		2	2
1	MS	NRS DE DO	1966	DOURADO	500370	1		2	2
1	RJ			VALENCA	330610	1		2	2
1	SP	GVE I CAPI	1331	SAO PAULO	355030	1		2	2
1	MA	REGIONAL	1440	JOAO LISBO	210550	2		2	9
1	CE	2 CRES CAU	1512	CAUCAIA	230370	1		2	2
1	SP	GVE XXIX S	1354	MONTE AP	353140	1		2	2
1	GO	SUL	1801	ITUMBIARA	521150	1		2	2
1	SP	GVE VIII M	1333	GUARULHO	351880	1		2	2
1	SP	GVE X OSA	1335	OSASCO	353440	1		2	2
1	SP	GVE X OSA	1335	TABOAO D	355280	9		2	9
1	MG	PATOS DE I	1456	PATOS DE I	314800	1		2	2
1	PB	I NRS JOAO	1377	SANTA RITA	251370	1		2	2
1	RS	016 CRS	1610	LAJEADO	431140	1		2	2
1	PR	09RS FOZ D	1363	FOZ DO IGU	410830	1		2	2
1	AM	ENTORNO	5584	MANAUS	130260	1		2	2
1	SP	GVE I CAPI	1331	SAO PAULO	355030	1		2	2
1	SP	GVE XII ARA	1337	SAO CARLO	354890	1		2	9
1	SP	GVE VIII M	1333	GUARULHO	351880	1		2	2
1	GO	SUDOESTE	1799	RIO VERDE	521880	1		2	2
1	AM	ENTORNO	5584	MANAUS	130260	1	1	1	
1	PR	02RS METR	1356	CURITIBA	410690	1		2	2
1	PR	10RS CASC	1364	CORBELIA	410630	1		2	2
1	SP	GVE XXII PI	1573	DRACENA	351440	1		2	2
1	SP	GVE I CAPI	1331	SAO PAULO	355030	1		2	2
1	BA	NUCLEO RE	1388	ITABELA	291465	1		2	2
1	MT	JUINA	1582	ARIPUANA	510140	2		2	2
1	AM	ENTORNO	5584	MANAUS	130260	1	1	2	
1	RS	001 CRS	1592	IGREJINHA	431010	1			
1	SP	GVE VII SAI	1332	DIADEMA	351380	1		2	2
1	SP	GVE XXVII S	1351	IGARATA	352020			2	9

1	PB	VIII NRS CA	1424	SAO BENTO	251390	1	2	1
1	DF			ITAPOA	539928	1	2	2
1	SP	GVE XXXI S	1353	SALTO DE F	354530	1	2	2
1	AM	ENTORNO	5584	MANAUS	130260	1		
1	SP	GVE I CAPI	1331	SAO PAULO	355030	1	2	2
1	PR	19RS JACA	1373	JACAREZIN	411180	1	2	2
1	SP	GVE XXXI S	1353	SALTO	354520	1	9	9
1	PA	01 REGION	1484	BELEM	150140	1	2	2
1	SP	GVE XXX JA	1574	SANTA FE D	354660	1	2	2
1	SP	GVE XV BA	1340	BAURU	350600	1	2	2
1	MA	REGIONAL	1432	VARGEM G	211270	1	2	9
1	SP	GVE XIX M	1344	MARILIA	352900	1	2	2
1	GO	PIRINEUS	1792	ANAPOLIS	520110	1	2	2
1	PA	01 REGION	1484	BELEM	150140	1	2	2
1	SP	GVE XXVI S	1350	MOGI GUA	353070	1	2	2
1	MT	PORTO ALE	1583	CANABRAV	510269		2	2
1	RN	IV URSAP	1414	SAO VICEN	241300	1	2	2
1	SP	GVE XI ARA	1336	BIRIGUI	350650	1	2	2
1	SP	GVE VIII M	1333	MOGI DAS	353060	1	2	9
1	GO	ESTRADA D	1802	CATALAO	520510	1	2	2
1	MA	REGI0NAL	1430	SAO LUIS	211130	1		
1	SP	GVE XXIX S	1354	SAO JOSE D	354980	1	2	2
1	AM	ENTORNO	5584	MANAUS	130260	1	2	2
1	SP	GVE I CAPI	1331	SAO PAULO	355030	1	2	2
1	GO	SUDOESTE	1799	RIO VERDE	521880	1	2	2
1	SP	GVE VII SA	1332	MAUA	352940	9	2	9
1	SP	GVE XVII C	1342	JAGUARIU	352470	1	2	9
1	BA	NUCLEO RE	1381	SERRINHA	293050		9	9
1	TO			PORTO NA	171820	1	2	2
1	SP	GVE XVII C	1342	AMERICAN	350160	1		2
1	SP	GVE XXXI S	1353	VOTORANT	355700	1	9	9
1	SP	GVE XI ARA	1336	VALPARAIS	355630	1	2	2
1	PR	02RS METR	1356	CURITIBA	410690	1	2	2
1	SP	GVE VII SA	1332	SANTO AN	354780	1	9	9
1	CE	13 CRES TI	1523	TIANGUA	231340	2	2	2
1	SC	JOINVILLE	1565	JOINVILLE	420910	1	2	2
1	PR	09RS FOZ D	1363	FOZ DO IG	410830	1	2	2
1	PR	20RS TOLE	1374	MARECHA	411460		2	2
1	SP	GVE XXV S	1349	GUARUJA	351870	1		
1	RJ			TRES RIOS	330600	1		
1	SP	GVE VII SA	1332	SAO BERN	354870		2	
1	SP	GVE VII SA	1332	MAUA	352940	1	2	2
1	RS	003 CRS	1593	SAO JOSE D	431850	9	2	2
1	SP	GVE X OSA	1335	BARUERI	350570	1	2	2
1	BA	NUCLEO RE	1381	SEABRA	292990		2	9
1	PI	1 GRS PAR	1868	PARNAIBA	220770	9	2	2
1	SP	GVE VII SA	1332	MAUA	352940	1	2	2

1	CE	1 CRES FOF	1519	FORTALEZ/	230440	1		2	9
1	MG	BELO HORI	1449	BELO HORI	310620	1		2	2
1	RJ			RIO DE JAN	330455	1		2	2
1	SC	RIO DO SUI	1478	POUSO REI	421370	1		2	2
1	RO	II GRS CAC(	1480	ESPIGAO D	110009	1		1	2
1	MG	BELO HORI	1449	ESMERALD	312410	1		9	9
1	PR	10RS CASC	1364	QUEDAS D(	412090	2		2	2
1	RN	II URSAP	1412	MOSSORO	240800	1		2	
1	PR	17RS LONE	1371	LONDRINA	411370	1		2	2
1	SC	CHAPECO	1553	MARAVILH	421050	1		2	2
1	SC	CRICIUMA	1558	CRICIUMA	420460			2	2
1	RS	004 CRS	1595	SANTIAGO	431740	1		2	9
1	SP	GVE XIII AS	1338	ESPIRITO S	351519	1		2	2
1	GO	CENTRAL	1779	GOIANIA	520870	1		2	2
1	PR	10RS CASC	1364	SANTA TER	412402	1		2	2
1	MT	SINOP	1584	SINOP	510790	1		2	2
1	CE	9 CRES RUS	1518	PALHANO	231000	1		9	9
1	MG	UNAI	1463	PARACATU	314700	1		2	2
1	RJ			RIO DE JAN	330455	1		9	9
1	MG	MONTES C	1473	MONTES C	314330	1	1	2	2
1	SP	GVE I CAPI	1331	SAO PAUL(	355030	1		2	2
1	MS	EX ARS DE	1975	JARAGUAR	500490	2		2	2
1	SP	GVE VII SAI	1332	SANTO AN	354780	1		2	2
1	SC	XANXERE	1556	GALVAO	420560	1		2	9
1	SP	GVE I CAPI	1331	SAO PAUL(	355030	1		2	2
1	SP	GVE I CAPI	1331	SAO PAUL(	355030	1		2	2
1	PB	XII NRS ITA	1428	INGA	250680	1		2	2
1	PR	02RS METF	1356	CAMPO M/	410425	1		2	2
1	GO	CENTRAL	1779	GOIANIA	520870	1		2	2
1	SP	GVE X OSA	1335	OSASCO	353440	1		2	2
1	MG	SETE LAGO	1467	SETE LAGO	316720	1		2	2
1	SP	GVE I CAPI	1331	SAO PAUL(	355030	1		2	2
1	SP	GVE XIX M/	1344	MARILIA	352900	1		2	2
1	GO	CENTRAL	1779	NOVA VEN	521500	1		2	9
1	SP	GVE I CAPI	1331	SAO PAUL(	355030	1		2	2
1	SP	GVE XXIX S	1354	SAO JOSE I	354980	1		2	2
1	CE	1 CRES FOF	1519	FORTALEZ/	230440			2	2
1	MG	UBERLAND	1462	PATROCINI	314810	1		2	2
1	SP	GVE XXXI S	1353	SALTO	354520	1		2	2
1	PA	12 REGION	1495	XINGUARA	150840	1		2	2
1	SP	GVE X OSA	1335	OSASCO	353440	1		2	2
1	AL	1 MICRORI	1533	COQUEIRO	270220			2	2
1	SP	GVE XI AR/	1336	PENAPOLIS	353730	1		2	2
1	RJ			BELFORD R	330045	1		2	
1	PR	02RS METF	1356	CURITIBA	410690	1		2	2
1	PE	009	1504	OURICURI	260990	1		2	2
1	GO	ENTORNO	1782	LUZIANIA	521250	1		2	

1	MG	PATOS DE I	1456	VAZANTE	317100				
1	SP	GVE XVII CA	1342	CAMPINAS	350950	1		2	2
1	CE	1 CRES FOF	1519	FORTALEZA	230440	1		2	
1	PA	09 REGION	1492	ORIXIMINA	150530	1		2	2
1	AM	ENTORNO	5584	MANAUS	130260	1		2	2
1	MG	UBERLAND	1462	UBERLAND	317020	1		2	2
1	RJ			CABO FRIO	330070	1			
1	SC	JOINVILLE	1565	JOINVILLE	420910	1		2	2
1	PA	01 REGION	1484	MARITUBA	150442	1		2	2
1	RJ			RIO DE JAN	330455	1		2	2
1	SP	GVE XVII CA	1342	MONTE MO	353180	1		2	2
1	RJ			BELFORD R	330045	1			
1	PR	14RS PARA	1368	SAO JOAO	412490	2		2	2
1	PR	17RS LOND	1371	LONDRINA	411370	1		1	2
1	PA	09 REGION	1492	ALENQUER	150040	2		2	2
1	RS	002 CRS	1607	PORTO ALE	431490	1		2	2
1	SC	CHAPECO	1553	CHAPECO	420420	1		9	9
1	SP	GVE I CAPI	1331	SAO PAULO	355030	1		2	2
1	MA	REGI0NAL	1430	SAO LUIS	211130	1		2	
1	MT	CUIABA	1578	CUIABA	510340	1		2	2
1	MG	UBERLAND	1462	MONTE CA	314310	1		2	2
1	MA	REGIONAL	1445	BARRA DO	210160	1		2	2
1	SP	GVE XVII CA	1342	CAMPINAS	350950	1			
1	SP	GVE I CAPI	1331	SAO PAULO	355030	1		2	2
1	GO	CENTRAL	1779	GOIANIA	520870	1		2	2
1	SP	GVE XVII CA	1342	JUNDIAI	352590	1		2	2
1	MG	SETE LAGO	1467	CURVELO	312090	9		2	2
1	BA	NUCLEO RE	1381	FEIRA DE S	291080	1			
1	MG	GOVERNAD	1471	GOVERNAD	312770	1		2	2
1	MT	JUINA	1582	BRASNORT	510190			2	2
1	GO	CENTRAL	1779	GOIANIA	520870	1			
1	RN	VII URSAP	1416	NATAL	240810	1		2	2
1	RJ			RIO DE JAN	330455	1		2	2
1	PA	13 REGION	1496	CAMETA	150210	2		2	2
1	BA	NUCLEO RE	1398	IUIU	291733	9		9	9
1	SP	GVE XI ARA	1336	PENAPOLIS	353730	1		2	2
1	MG	SETE LAGO	1467	SETE LAGO	316720	1		2	2
1	GO	CENTRAL	1779	PETROLINA	521680	1		2	9
1	RR			BONFIM	140015	1		2	9
1	SP	GVE XXIX S	1354	SAO JOSE D	354980	1		2	2
1	MS	NRS DE NA	1969	NAVIRAI	500570	1		2	2
1	RJ			RIO DE JAN	330455				
1	PR	15RS MARI	1369	MANDAGU	411410	1		2	2
1	SP	GVE XII ARA	1337	SAO CARLO	354890	1		2	2
1	CE	16 CRES CA	1526	GRANJA	230470			2	2
1	RR			MUCAJAI	140030	1		2	9
1	PR	10RS CASC	1364	BRAGANEY	410335	1	2	2	2

1	CE	11 CRES SC	1521	SOBRAL	231290	1	2	2
1	MT	CUIABA	1578	CUIABA	510340		2	2
1	GO	ENTORNO	1782	VALPARAIS	522185	1	2	2
1	SP	GVE X OSA	1335	ITAPECERI(	352220	1	2	2
1	PR	17RS LONE	1371	IBIPORA	410980	1	2	2
1	RO	VI GRS POF	1483	PORTO VEL	110020	1	2	2
1	MG	BELO HORI	1449	CONTAGEN	311860	1	2	2
1	GO	SUDOESTE	1799	RIO VERDE	521880	1	2	2
1	CE	1 CRES FOF	1519	FORTALEZ/	230440	1		
1	BA	NUCLEO RI	1380	DIAS D'AVI	291005		9	9
1	MG	BELO HORI	1449	BETIM	310670	1	9	9
1	MG	CORONEL I	1468	TIMOTEO	316870	1	9	2
1	MG	BELO HORI	1449	RIBEIRAO I	315460	1		2
1	SP	GVE X OSA	1335	JANDIRA	352500			
1	MG	POUSO ALI	1459	MUNHOZ	314380	2	2	2
1	MA	REGIONAL	1440	MONTES A	210700	1	2	2
1	SP	GVE XXIV R	1348	BATATAIS	350590	1	2	2
1	PE	008	1503	SANTA MA	261260	1	2	9
1	SP	GVE I CAPI	1331	SAO PAUL(	355030		2	
1	PR	05RS GUAF	1359	PINHAO	411930		2	2
1	SP	GVE XX PIR	1345	SAO PEDR(	355040		2	2
1	RS	002 CRS	1607	PORTO ALI	431490	1	9	9
1	GO	SERRA DA I	1790	URUACU	522160	1	2	2
1	RS	005 CRS	1608	CAXIAS DO	430510	1	2	2
1	SP	GVE XXIV R	1348	CRAVINHO	351310	1	2	2
1	MG	VARGINHA	1466	TRES PONT	316940	1	2	2
1	SP	GVE VIII M	1333	ITAQUAQU	352310		2	2
1	SP	GVE XV BA	1340	BAURU	350600	1	2	2
1	MA	REGI0NAL	1430	SAO LUIS	211130	1	2	2
1	SC	BRACO DO	5608	SAO LUDGI	421700	1		
1	GO	PIRINEUS	1792	ANAPOLIS	520110	1	2	2
1	MG	UBERABA	1461	UBERABA	317010	1	2	2
1	MG	BELO HORI	1449	ESMERALD	312410	1	9	9
1	BA	NUCLEO RI	1380	SALVADOR	292740	1	2	2
1	SP	GVE I CAPI	1331	SAO PAUL(	355030	1	2	9
1	SP	GVE I CAPI	1331	SAO PAUL(	355030	1	2	2
1	RS	002 CRS	1607	GRAVATAI	430920			
1	CE	1 CRES FOF	1519	FORTALEZ/	230440	1	9	9
1	MG	ALFENAS	1379	GUAXUPE	312870	1	2	2
1	SP	GVE XXI PR	1346	TACIBA	355290	1	2	2
1	RJ			NOVA IGU/	330350	1	9	2
1	MG	UBERABA	1461	ITURAMA	313440	1	2	2
1	SP	GVE XXXI S	1353	SOROCABA	355220	1	2	
1	RR			BOA VISTA	140010	1	2	9
1	RJ			RIO DE JAN	330455	1	2	2
1	RJ			RIO DE JAN	330455	1	9	9
1	SP	GVE XXV S/	1349	MONGAGU	353110	1		

1	MG	BELO HORI	1449	NOVA UNI/	313660			2	2
1	SC	ITAJAI	1550	PENHA	421250	1		2	2
1	BA	NUCLEO RI	1382	CICERO DA	290780	1		2	9
1	MG	PATOS DE I	1456	CARMO DC	311430	1		1	
1	GO	CENTRAL	1779	GOIANIA	520870	1		2	2
1	PR	08RS FRAN	1362	FRANCISCC	410840	1	2	2	2
1	BA	NUCLEO RI	1380	SALVADOR	292740			2	2
1	PR	10RS CASC	1364	CASCAVEL	410480	1		2	2
1	MG	DIAMANTI	1450	CARBONIT/	311350	1		2	2
1	SP	GVE I CAPI	1331	SAO PAULC	355030	1		9	2
1	CE	1 CRES FOF	1519	ITAITINGA	230625	1		2	9
1	SC	VIDEIRA	1546	CURITIBAN	420480	1		2	2
1	SP	GVE X OSA	1335	EMBU-GU/	351510	1			
1	SP	GVE XXIV F	1348	RIBEIRAO F	354340	1		2	2
1	BA	NUCLEO RI	1382	JANDAIRA	291790	2		2	9
1	PR	17RS LONE	1371	CAMBE	410370	1		2	2
1	MG	SETE LAGO	1467	SETE LAGO	316720	1		2	2
1	PR	02RS METF	1356	CURITIBA	410690	1		2	2
1	GO	ENTORNO	1782	VALPARAIS	522185	1		2	2
1	SC	CRICIUMA	1558	ORLEANS	421170	1		2	2
1	SP	GVE XXXI S	1353	SALTO DE F	354530	1		2	9
1	PE	001	1497	JABOATAO	260790	1		2	9
1	BA	NUCLEO RI	1380	DIAS D'AVI	291005	1	2		2
1	BA	NUCLEO RI	1398	SEBASTIAC	293000		9	9	9
1	GO	ENTORNO	1784	PLANALTIN	521760	1		2	1
1	PR	02RS METF	1356	CURITIBA	410690	1		2	9
1	PR	15RS MARI	1369	MANDAGU	411410	1		2	2
1	SC	JOINVILLE	1565	JOINVILLE	420910	1		2	2
1	SP	GVE XVII C/	1342	VARZEA P/	355650	1		2	2
1	SP	GVE I CAPI	1331	SAO PAULC	355030	1		2	2
1	SP	GVE XXIII R	1347	REGISTRO	354260	3			
1	SP	GVE I CAPI	1331	SAO PAULC	355030				
1	PR	15RS MARI	1369	SARANDI	412625	1		2	2
1	RJ			SAO PEDRC	330520	1		2	9
1	GO	CENTRAL	1779	GOIANIA	520870	1		2	2
1	SP	GVE I CAPI	1331	SAO PAULC	355030	1		9	9
1	MG	UBERABA	1461	ARAXA	310400	1		2	2
1	MG	MANHUAC	1454	ABRE CAM	310030	1		2	2
1	SP	GVE XVII C/	1342	INDAIATUE	352050			2	2
1	MS	EX ARS DE	1975	CAMPO GF	500270	1		2	2
1	GO	ENTORNO	1782	AGUAS LIN	520025	1		2	9
1	RN	II URSAP	1412	IPANGUAC	240470	2		2	2
1	MG	DIVINOPOI	1472	OLIVEIRA	314560	2		2	2
1	RJ			NILOPOLIS	330320	1			
1	AM	MEDIO AM	5590	ITACOATIA	130190		2	2	2
1	SP	GVE XII AR/	1337	ARARAQU/	350320	1		2	2
1	AL	9 MICRORE	1541	PALMEIRA	270630	1		1	2

1	MT	TANGARA	1585	TANGARA	510795		2	2
1	AP			MACAPA	160030	1	2	2
1	SP	GVE I CAPI	1331	SAO PAULO	355030	1	2	2
1	PR	02RS METF	1356	COLOMBO	410580	1	2	2
1	RJ			SAO GONC	330490	1	2	2
1	SP	GVE XXIV F	1348	CRAVINHO	351310	1	2	2
1	SP	GVE I CAPI	1331	SAO PAULO	355030	1	2	2
1	SP	GVE XXVI S	1350	MOGI GUA	353070	1	2	2
1	SP	GVE XIX MA	1344	QUINTANA	354200	2	2	2
1	SC	JARAGUA D	1567	JARAGUA D	420890	1	2	2
1	BA	NUCLEO RE	1381	FEIRA DE S.	291080	1		
1	SP	GVE XXXI S	1353	VOTORANT	355700	1	2	2
1	RS	010 CRS	1599	SAO GABRI	431830	1	2	2
1	MA	REGIONAL	1441	TASSO FRA	211200	1	2	2
1	RJ			VALENCA	330610	1		
1	MG	ALFENAS	1379	ALFENAS	310160	2	2	2
1	SP	GVE XXI PR	1346	PRESIDENT	354140	1	2	2
1	MT	CUIABA	1578	VARZEA GF	510840	1	2	2
1	GO	CENTRO SU	1781	APARECIDA	520140	1		
1	SP	GVE XVI BC	1341	AVARE	350450	1	2	2
1	RS	012 CRS	1601	SAO MIGU	431915	2	2	2
1	RJ			RIO DE JAN	330455	1	2	2
1	GO	ENTORNO	1782	AGUAS LIN	520025	1	2	9
1	RJ			QUEIMADO	330414	3	2	9
1	SP	GVE XXIX S	1354	GUAPIACU	351750	1	2	2
1	SP	GVE I CAPI	1331	SAO PAULO	355030	1	2	2
1	SP	GVE XIV BA	1339	VIRADOUR	355680	1	2	2
1	BA	NUCLEO RE	1398	JACARACI	291740	2	2	2
1	MS	EX ARS DE	1975	BANDEIRA	500150	1	2	2
1	PR	16RS APUC	1370	APUCARAN	410140	1	2	9
1	SP	GVE I CAPI	1331	SAO PAULO	355030	9	2	9
1	PR	17RS LOND	1371	BELA VISTA	410280	1		2
1	SP	GVE I CAPI	1331	SAO PAULO	355030	1		
1	MT	CUIABA	1578	VARZEA GF	510840	1	2	2
1	DF			SAMAMBA	530140	1	2	9
1	GO	PIRINEUS	1792	ANAPOLIS	520110	1	2	2
1	RS	001 CRS	1592	CANOAS	430460	1	2	9
1	RS	003 CRS	1593	RIO GRAND	431560	3	2	2
1	RN	II URSAP	1412	MOSSORO	240800	1	2	2
1	MG	GOVERNAD	1471	GOVERNAD	312770	1	2	2
1	SC	BRACO DO	5608	GRAO PAR.	420610	1	2	2
1	SP	GVE XVIII F	1343	FRANCA	351620	1	2	2
1	SP	GVE XVI BC	1341	PARANAPA	353580	1	2	2
1	SP	GVE X OSA	1335	OSASCO	353440	1	2	2
1	RS	004 CRS	1595	SANTA MA	431690	1	2	2
1	PA	11 REGION	1494	SAO DOMI	150715	1	2	2
1	RJ			RIO DE JAN	330455	1	2	2

1	SP	GVE I CAPI'	1331	SAO PAULC	355030	1		2	2
1	MG	UBERABA	1461	UBERABA	317010	1		2	9
1	GO	NORTE	1788	PORANGAT	521800	1			
1	PR	10RS CASC	1364	QUEDAS D	412090	1		2	2
1	PA	12 REGION	1495	XINGUARA	150840	1		2	2
1	GO	CENTRO SU	1781	JANDAIA	521170	1		2	2
1	MT	TANGARA	1585	TANGARA	510795			2	2
1	MA	REGIONAL	1445	BARRA DO	210160	1		2	2
1	PR	17RS LOND	1371	ASSAI	410190	1		2	2
1	PB	VI NRS PAT	1422	PATOS	251080	1	2	2	2
1	SP	GVE XXIV F	1348	RIBEIRAO F	354340	1			
1	AM	ENTORNO	5584	MANAUS	130260	1		2	2
1	PE	002	1507	PAUDALHC	261060	1		2	9
1	PA	07 REGION	1490	MUANA	150490	1		2	2
1	BA	NUCLEO RE	1393	JUAZEIRO	291840			2	9
1	MT	CACERES	1581	SAO JOSE D	510710	1		2	2
1	PR	02RS METR	1356	CURITIBA	410690	1	2	2	2
1	MA	REGIONAL	1447	CAXIAS	210300	1		2	2
1	RS	001 CRS	1592	IGREJINHA	431010	1		2	9
1	BA	NUCLEO RE	1380	SALVADOR	292740	1		2	9
1	RJ			RIO DE JAN	330455		1	2	9
1	CE	1 CRES FOF	1519	AQUIRAZ	230100	2		2	9
1	SP	GVE VIII M	1333	MOGI DAS	353060	1		2	2
1	SP	GVE IX FRA	1334	FRANCISCC	351630	1			
1	RS	004 CRS	1595	SANTA MA	431690			2	2
1	PR	02RS METR	1356	CURITIBA	410690	1		2	2
1	SP	GVE XIII AS	1338	PEDRINHA:	353715	1		2	2
1	RS	002 CRS	1607	PORTO ALE	431490				
1	SC	CANOINHA	1570	CANOINHA	420380	1		2	2
1	SP	GVE XXX JA	1574	POPULINA	354040	1		2	2
1	RJ			RIO DE JAN	330455	1			
1	CE	10 CRES LII	1532	TABULEIRC	231310	1		9	9
1	SC	FLORIANOI	1476	TIJUCAS	421800	1		2	
1	BA	NUCLEO RE	1385	JEQUIE	291800	1		2	9
1	MS	NRS DE TRI	6255	TRES LAGO	500830	1		2	2
1	SP	GVE I CAPI'	1331	SAO PAULC	355030	1		2	2
1	RS	002 CRS	1607	GUAIBA	430930	1		2	2
1	GO	SAO PATRI	1791	SANTA TER	521970	1		2	2
1	SP	GVE I CAPI'	1331	SAO PAULC	355030	1	2	2	2
1	PB	I NRS JOAC	1377	JOAO PESS	250750	1		2	2
1	PR	10RS CASC	1364	SANTA TER	412402	1		2	9
1	RJ			RESENDE	330420	1			
1	SP	GVE XX PIR	1345	PIRACICAB	353870	1	1	2	2
1	MG	VARGINHA	1466	CAXAMBU	311550	1		2	2
1	SP	GVE I CAPI'	1331	SAO PAULC	355030		2	2	2
1	SP	GVE VII SAI	1332	MAUA	352940	1		2	2
1	PA	01 REGION	1484	BELEM	150140	1		2	2

1	SP	GVE I CAPI	1331	SAO PAUL(	355030	1		2	2
1	SP	GVE VIII M	1333	SANTA ISA	354680	1		2	2
1	RN	VII URSAP	1416	NATAL	240810	1		2	9
1	SP	GVE XVII C.	1342	JAGUARIUI	352470	1		2	2
1	SP	GVE XXXI S	1353	BOITUVA	350700	9		2	9
1	SP	GVE XV BA	1340	BAURU	350600	1		2	2
1	SC	JOINVILLE	1565	JOINVILLE	420910	1		2	2
1	RJ			NITEROI	330330	1	2	2	2
1	RS	007 CRS	1596	BAGE	430160	1		2	2
1	RJ			RIO DE JAN	330455			2	2
1	AM	ENTORNO	5584	MANAUS	130260	1		2	
1	PR	02RS METF	1356	PIRAQUAR	411950	1		2	2
1	MG	BELO HORI	1449	BETIM	310670	1			
1	SP	GVE X OSA	1335	ITAPECERI(	352220	1		2	2
1	PR	02RS METF	1356	CERRO AZU	410520	2		2	9
1	SP	GVE X OSA	1335	COTIA	351300			2	2
1	PR	15RS MARI	1369	MANDAGU	411420	1		2	2
1	PR	09RS FOZ D	1363	FOZ DO IGU	410830	1		2	2
1	DF	SUPERINTE	6262	SOBRADIN	530170	1		2	2
1	PE	002	1507	CARPINA	260400	1			
1	GO	ESTRADA D	1802	RIO QUENT	521878	1		2	2
1	PR	17RS LOND	1371	CAMBE	410370	1		2	2
1	RS	002 CRS	1607	CACHOEIRI	430310	1		2	2
1	RN	V URSAP	1415	SAO JOSE D	241230	1		2	2
1	SC	BLUMENAU	1564	GASPAR	420590	1		2	2
1	GO	CENTRAL	1779	GOIANIA	520870				
1	PB	I NRS JOAC	1377	CABEDELO	250320	1		2	2
1	SP	GVE XXV S	1349	SAO VICEN	355100	1		2	2
1	RS	001 CRS	1592	NOVA HAR	431306	1		2	2
1	SP	GVE XVII C.	1342	HORTOLAN	351907	1		1	9
1	PR	15RS MARI	1369	MARINGA	411520	1	2	2	2
1	SC	CONCORDI	1560	SEARA	421750	1	2	2	2
1	GO	PIRINEUS	1792	TEREZOPO	522119				
1	GO	ENTORNO	1784	PLANALTIN	521760	1		2	2
1	GO	ESTRADA D	1802	CATALAO	520510	1		2	2
1	RS	005 CRS	1608	GRAMADO	430910	1		2	2
1	MA	REGIONAL	1434	SAO MATE	211150	1		2	2
1	ES	METROPOI	1510	VITORIA	320530	1		2	2
1	CE	5 CRES CAN	1514	CARIDADE	230300	1		9	9
1	RJ			RIO DE JAN	330455	1		2	2
1	MT	ALTA FLOR	1587	NOVA MOI	510895				
1	RS	005 CRS	1608	VACARIA	432250			2	2
1	MG	JANUARIA	1470	JANUARIA	313520	1		2	2
1	PR	10RS CASC	1364	CASCAVEL	410480	1		2	2
1	MG	POUSO ALI	1459	ITAJUBA	313240	1	2	2	2
1	AL	9 MICRORE	1541	PALMEIRA	270630	1			
1	GO	CENTRO SU	1781	CEZARINA	520545	1		2	2

1	BA	NUCLEO RI	1380	SALVADOR	292740	1		2	9
1	SP	GVE XX PIR	1345	ARARAS	350330	1		2	2
1	SP	GVE XXIX S	1354	TABAPUA	355260	1		2	2
1	SC	FLORIANOI	1476	FLORIANOI	420540	1		2	
1	SP	GVE XXXI S	1353	SOROCABA	355220	1		2	2
1	SP	GVE I CAPI	1331	SAO PAULO	355030	1		2	
1	MG	VARGINHA	1466	BOA ESPER	310710	1	1	2	2
1	SC	CHAPECO	1553	CHAPECO	420420	1		9	9
1	RN	VII URSAP	1416	NATAL	240810	1		2	2
1	RR			CAROEBE	140023	1		2	2
1	PB	II NRS GUA	1420	MULUNGU	250980	2		2	2
1	PR	16RS APUC	1370	ARAPONGA	410150	1		2	2
1	MG	BELO HORI	1449	CONTAGEN	311860	1		2	2
1	DF			CEILANDIA	530040	1		2	2
1	PA	01 REGION	1484	BENEVIDES	150150	1		2	2
1	RJ			CAMPOS D	330100	1		2	2
1	GO	SUDOESTE	1800	MINEIROS	521310	1	2	2	2
1	MG	DIVINOPOI	1472	NOVA SERI	314520	1		2	2
1	RJ			RIO DE JAN	330455			2	2
1	GO	CENTRO SU	1781	PONTALINA	521770	1		2	2
1	AP			MACAPA	160030	1		2	2
1	AL	3 MICRORI	1535	CAMPO AL	270140	2		2	2
1	GO	CENTRAL	1779	GOIANIA	520870	1		2	2
1	RJ			MAGE	330250	1			
1	SC	CHAPECO	1553	CHAPECO	420420	1		2	2
1	RS	005 CRS	1608	CAXIAS DO	430510	1			
1	BA	NUCLEO RI	1380	LAURO DE	291920	1		2	9
1	RJ			RIO DE JAN	330455			2	
1	SP	GVE XI ARA	1336	GUARACAI	351780	1		2	2
1	PR	11RS CAMI	1365	ARARUNA	410170	1		2	2
1	SC	FLORIANOI	1476	PALHOCA	421190	1		2	
1	SP	GVE I CAPI	1331	SAO PAULO	355030	1		2	2
1	PI	15 GRS URI	1894	URUCUI	221120	2		2	2
1	SC	BLUMENAU	1564	GASPAR	420590	1		2	2
1	RS	001 CRS	1592	CANOAS	430460	1		9	2
1	RS	002 CRS	1607	VIAMAO	432300	1		2	2
1	MS	NRS DE TRI	6255	TRES LAGO	500830	1		2	2
1	SC	LAGES	1571	LAGES	420930	1		2	2
1	CE	1 CRES FOI	1519	FORTALEZA	230440	1		9	9
1	SP	GVE XVII CA	1342	CAMPINAS	350950			2	2
1	RS	006 CRS	1611	PASSO FUN	431410	1		2	2
1	MG	SAO JOAO	1460	SANTA CRU	315733	1		2	2
1	MT	CUIABA	1578	CUIABA	510340			2	2
1	RS	006 CRS	1611	PASSO FUN	431410	1		2	2
1	DF			TAGUATIN	530180	1		2	9
1	RR			BOA VISTA	140010	1		2	2
1	SP	GVE XVII CA	1342	JAGUARIUI	352470	1		2	9

1	MT	SINOP	1584	SINOP	510790	1		2	2
1	RJ			RIO DE JAN	330455	1		9	9
1	SP	GVE XVII C.	1342	HORTOLAN	351907	1		2	9
1	GO	CENTRAL	1779	GOIANIA	520870	1		2	2
1	RJ			RIO DE JAN	330455	1		2	2
1	SP	GVE VIII M	1333	GUARULH(	351880	1		2	2
1	PR	09RS FOZ D	1363	SANTA TER	412405	1		2	2
1	SP	GVE XIV BA	1339	TAQUARAL	355365	1	2		2
1	PR	17RS LOND	1371	ROLANDIA	412240	1		2	2
1	SP	GVE XX PIR	1345	PIRACICAB	353870			2	2
1	PR	02RS METF	1356	COLOMBO	410580	1		2	2
1	RS	005 CRS	1608	CAXIAS DO	430510			2	2
1	SP	GVE XXIX S	1354	SAO JOSE D	354980	1		2	2
1	RS	002 CRS	1607	PORTO ALE	431490	1		2	2
1	MA	REGIONAL	1438	ZE DOCA	211400	2		2	2
1	RJ			RIO DE JAN	330455				
1	RJ			MARICA	330270			9	9
1	SP	GVE XXXIII	1352	TAUBATE	355410	1		2	2
1	SP	GVE I CAPI	1331	SAO PAULO	355030	1		2	2
1	RJ			VASSOURA	330620			2	2
1	TO			PALMAS	172100	1		9	9
1	CE	1 CRES FOF	1519	FORTALEZA	230440	1		9	9
1	RJ			MESQUITA	330285	1			
1	SC	XANXERE	1556	ENTRE RIO	420517	1		2	9
1	RS	001 CRS	1592	CANOAS	430460	1		2	2
1	RO	VI GRS POF	1483	PORTO VEL	110020	1		2	2
1	SP	GVE VII SAI	1332	DIADEMA	351380	1		2	2
1	PB	III NRS CAN	1421	MONTADA	250950	1		2	2
1	MG	LEOPOLDIN	1453	PIRAPETIN	315110	1		2	2
1	PR	10RS CASC	1364	CASCAVEL	410480	1		2	2
1	SC	BLUMENAU	1564	BRUSQUE	420290	1		2	2
1	RS	005 CRS	1608	CANELA	430440				
1	SP	GVE X OSA	1335	ITAPECERI(	352220	1		2	2
1	MT	SINOP	1584	NOVA MUT	510622	1		2	2
1	DF			CEILANDIA	530040	1		2	9
1	MG	BELO HORI	1449	RIBEIRAO D	315460	1		2	2
1	SP	GVE XII AR.	1337	SAO CARLC	354890	1		2	2
1	RS	005 CRS	1608	NOVA PETI	431320			2	2
1	TO			ARAPOEM.	170230	1			
1	MG	BELO HORI	1449	CONTAGEN	311860	1		9	
1	TO			PORTO NA	171820	1		2	9
1	PR	02RS METF	1356	ITAPERUCU	411125	1		2	2
1	SC	JOINVILLE	1565	JOINVILLE	420910	1		2	2
1	SP	GVE XVII C.	1342	ARTUR NO	350380	1		2	9
1	BA	NUCLEO RI	1381	CANSANCA	290680	9		9	9
1	GO	CENTRAL	1779	GOIANIA	520870	1		2	2
1	AC	REGIONAL	1938	PLACIDO D	120038	2			

1	MT	CUIABA	1578	CHAPADA	510300		2	2
1	RJ			RIO DE JAN	330455	1	9	9
1	SP	GVE XXIV R	1348	RIBEIRAO F	354340	1	9	9
1	AL	3 MICRORR	1535	CAMPO AL	270140	2	2	2
1	RJ			RIO DE JAN	330455	1		
1	BA	NUCLEO RE	1380	MATA DE S	292100		9	9
1	MG	BELO HORI	1449	BELO HORI	310620	1	2	2
1	BA	NUCLEO RE	1385	ITUBERA	291730		9	9
1	SP	GVE XXVII S	1351	JACAREI	352440	1	2	2
1	SP	GVE XVI BC	1341	MANDURI	352860	1	2	2
1	GO	CENTRO SU	1781	APARECIDA	520140		2	
1	CE	22 CRES CA	5596	HORIZONT	230523	2	2	9
1	CE	4 CRES BAT	1520	BATURITE	230210	2	2	9
1	SP	GVE XXIX S	1354	SAO JOSE D	354980	1	2	2
1	BA	NUCLEO RE	1403	LUIS EDUA	291955		2	9
1	SP	GVE XXIV R	1348	JABOTICAB	352430	1		
1	SP	GVE VIII M	1333	GUARULHO	351880	1	2	2
1	SP	GVE I CAPIT	1331	SAO PAULO	355030	1	2	2
1	BA	NUCLEO RE	1385	JEQUIE	291800		2	9
1	MG	PATOS DE I	1456	PATOS DE I	314800	1		
1	GO	SUDOESTE	1799	CACU	520430	1	9	9
1	CE	1 CRES FOR	1519	FORTALEZA	230440	1	2	9
1	SP	GVE VII SAI	1332	SANTO AN	354780	1	2	2
1	SC	FLORIANOI	1476	FLORIANOI	420540	1	2	
1	SP	GVE I CAPIT	1331	SAO PAULO	355030	1	2	2
1	SC	FLORIANOI	1476	PALHOCA	421190	1	2	2
1	RJ			VOLTA RED	330630	1	9	9
1	MS	EX NRS DE	6256	CORUMBA	500320	1	2	2
1	SP	GVE X OSA	1335	TABOAO D	355280	1	2	2
1	PR	19RS JACAI	1373	SANTO AN	412410	1	2	2
1	SP	GVE I CAPIT	1331	SAO PAULO	355030	1	2	2
1	PR	19RS JACAI	1373	JAPIRA	411230	1	2	2
1	SP	GVE XXV SA	1349	GUARUJA	351870	1	1	2
1	BA	NUCLEO RE	1398	VITORIA DA	293330	1		
1	SP	GVE XI ARA	1336	ANDRADIN	350210	1	2	2
1	GO	ENTORNO	1784	PLANALTIN	521760	1	2	2
1	SP	GVE XVII CA	1342	CAMPINAS	350950	1	2	9
1	MA	REGIONAL	1441	RIACHAO	210950	1	2	2
1	PR	10RS CASC	1364	SANTA LUC	412382	1	2	2
1	PR	03RS PONT	1357	JAGUARIAI	411200	1		
1	CE	11 CRES SC	1521	SANTA QU	231220	1	2	2
1	PR	02RS METR	1356	CURITIBA	410690	1	2	2
1	MG	DIVINOPOI	1472	FORMIGA	312610	1	2	2
1	GO	SUDOESTE	1799	CACU	520430	1	2	2
1	MG	JUIZ DE FO	1452	JUIZ DE FO	313670	1	2	2
1	MA	REGIONAL	1441	BALSAS	210140	1	2	2
1	SP	GVE X OSA	1335	OSASCO	353440	1	2	2

1	CE	22 CRES CA	5596	PINDORETA	231085	1		9	9
1	MT	AGUA BOA	1590	CANARANA	510270	1		2	2
1	SP	GVE XII ARA	1337	SAO CARLO	354890	1		2	2
1	MG	TEOFILO OT	1465	MALACACH	313920	1		2	9
1	PB	II NRS GUA	1420	BELEM	250190	1	9	9	2
1	MA	REGIONAL	1440	SENADOR I	211176	1		2	2
1	PR	10RS CASC	1364	CASCAVEL	410480	1		2	2
1	SP	GVE XXIV R	1348	RIBEIRAO P	354340	1		2	2
1	PR	16RS APUC	1370	APUCARAN	410140	1		2	2
1	PB	I NRS JOAO	1377	JOAO PESS	250750	1		2	2
1	SC	LAGES	1571	BOM RETIR	420260			2	9
1	SP	GVE VII SAI	1332	RIBEIRAO P	354330	1		2	2
1	AP			MACAPA	160030	1	1	2	2
1	SP	GVE XVII CA	1342	PEDREIRA	353710				
1	RJ			RIO DE JAN	330455	1		2	2
1	RR			BOA VISTA	140010	1	1	2	2
1	PR	17RS LOND	1371	CAMBE	410370	1		2	2
1	BA	NUCLEO RE	1385	ILHEUS	291360	1		2	2
1	CE	11 CRES SO	1521	SOBRAL	231290	1		2	2
1	BA	NUCLEO RE	1380	SIMOES FIL	293070			9	9
1	SP	GVE I CAPIT	1331	SAO PAULO	355030	1		2	2
1	SP	GVE VIII MO	1333	SUZANO	355250	1		2	9
1	MG	MONTES C	1473	MONTES C	314330	1		2	2
1	PR	17RS LOND	1371	PRIMEIRO	412050	1		2	2
1	MA	REGIONAL	1435	SANTA HEL	210980	2		9	2
1	MT	CUIABA	1578	CUIABA	510340	1		2	2
1	PR	03RS PONT	1357	IPIRANGA	411050	1		9	2
1	ES	COLATINA	1509	BAIXO GUA	320080	2		2	2
1	SP	GVE I CAPIT	1331	SAO PAULO	355030	1		2	2
1	SP	GVE I CAPIT	1331	SAO PAULO	355030	9		2	2
1	CE	22 CRES CA	5596	CASCAVEL	230350	1		2	9
1	AM	ENTORNO	5584	PRESIDENT	130353	2		2	2
1	PR	17RS LOND	1371	LONDRINA	411370	1		2	2
1	SP	GVE XXI PR	1346	PIRAPOZIN	353920	1		2	2
1	SP	GVE I CAPIT	1331	SAO PAULO	355030			2	2
1	SP	GVE I CAPIT	1331	SAO PAULO	355030	1		2	2
1	RJ			RESENDE	330420	1			
1	SP	GVE I CAPIT	1331	SAO PAULO	355030	2		1	2
1	SP	GVE I CAPIT	1331	SAO PAULO	355030	1		2	2
1	SP	GVE VIII MO	1333	SUZANO	355250	1		2	2
1	PR	09RS FOZ D	1363	FOZ DO IGU	410830	1		2	2
1	SP	GVE I CAPIT	1331	SAO PAULO	355030	1		2	2
1	SP	GVE XXIX S	1354	SAO JOSE D	354980	1		2	2
1	MG	POUSO ALE	1459	POCOS DE	315180	1		9	2
1	SP	GVE XVII CA	1342	SUMARE	355240	1	2	2	2
1	SP	GVE XXXI S	1353	SOROCABA	355220	1			
1	BA	NUCLEO RE	1380	SALVADOR	292740	9		9	9

1	SP	GVE IX FRA	1334	MAIRIPOR	352850	1		2	2
1	GO	ESTRADA D	1802	CALDAS NO	520450	1			2
1	SP	GVE XXXI S	1353	SAO ROQU	355060	9		2	9
1	SP	GVE VIII M	1333	SUZANO	355250	1		2	2
1	PA	03 REGION	1486	CASTANHA	150240	1		2	2
1	MT	JUINA	1582	JUINA	510515	1			
1	SP	GVE I CAPI	1331	SAO PAUL	355030	1	2	2	2
1	RS	002 CRS	1607	ELDORADO	430676	1		2	2
1	AL	1 MICROR	1533	MACEIO	270430	1			
1	SP	GVE I CAPI	1331	SAO PAUL	355030	1		2	2
1	MT	CUIABA	1578	CUIABA	510340	1		2	2
1	RJ			TRES RIOS	330600			9	9
1	AL	13 MICROR	1545	TEOTONIO	270915	2		2	9
1	RJ			DUQUE DE	330170			2	9
1	SP	GVE XVII C	1342	HORTOLAN	351907	1		2	2
1	GO	ENTORNO	1782	SANTO AN	521975	1		2	2
1	CE	13 CRES TI	1523	GUARACIA	230500	2		2	2
1	DF			SUDOESTE,	539931	1		9	
1	SP	GVE XIX M	1344	MARILIA	352900	1		2	2
1	MT	AGUA BOA	1590	CANARAN	510270	1		2	2
1	RN	VII URSAP	1416	NATAL	240810	1		2	2
1	SP	GVE VII SA	1332	RIBEIRAO	354330			2	2
1	SP	GVE XXI PR	1346	SANTO AN	354770	1		2	2
1	SP	GVE XVII C	1342	CAMPINAS	350950	1		2	9
1	SP	GVE XX PIR	1345	PIRACICAB	353870			1	1
1	GO	CENTRAL	1779	GOIANIA	520870	1		2	2
1	PR	21RS TELEI	1375	RESERVA	412170	1		2	2
1	MG	SETE LAGO	1467	SETE LAGO	316720	1		2	2
1	SP	GVE X OSA	1335	OSASCO	353440	1		2	2
1	SP	GVE I CAPI	1331	SAO PAUL	355030	1		2	9
1	SP	GVE VIII M	1333	GUARULH	351880	1		2	2
1	MT	CACERES	1581	ARAPUTAN	510125	1		2	2
1	RS	012 CRS	1601	SAO LUIZ G	431890	1		2	2
1	CE	3 CRES MA	1513	MARACAN	230765	1		9	9
1	PR	17RS LOND	1371	LONDRINA	411370	1		2	2
1	PR	12RS UMU	1366	PEROBAL	411885	2		2	2
1	PI			ALTOS	220040	1		2	2
1	SP	GVE XXVI S	1350	SAO JOSE D	354970	2		2	2
1	PR	01RS PAR	1355	PARANAG	411820	1		2	2
1	SP	GVE X OSA	1335	OSASCO	353440	1		2	2
1	CE	4 CRES BA	1520	ARACOIAB	230120	2		2	9
1	SP	GVE XVII C	1342	ITUPEVA	352400	1		2	2
1	DF			SAO SEBAS	530160	1		2	2
1	PR	02RS METF	1356	SAO JOSE D	412550	1		2	2
1	MA	REGI0NAL	1430	SAO LUIS	211130	1		2	9
1	AL	1 MICROR	1533	MACEIO	270430	1			
1	MG	MANHUAC	1454	SANTA MA	315790	1	1	2	2

1	SP	GVE XVII C/	1342	CAMPINAS	350950	1	2	2
1	TO			COLINAS D	170550	1	2	2
1	GO	SUDOESTE	1799	MONTIVID	521375	1	2	2
1	GO	CENTRAL	1779	GOIANIA	520870	1	2	2
1	PR	13RS CIAN(	1367	RONDON	412260	1	2	2
1	SP	GVE XVII C/	1342	JUNDIAI	352590		2	2
1	MG	JUIZ DE FO	1452	JUIZ DE FO	313670	1	2	2
1	SP	GVE XXVI S	1350	MOGI GUA	353070	1	2	2
1	SP	GVE VII SAI	1332	DIADEMA	351380	1	2	2
1	AL	9 MICRORE	1541	PALMEIRA	270630	2	2	2
1	PR	16RS APUC	1370	MAUA DA .	411575	1	2	2
1	GO	CENTRAL	1779	NEROPOLIS	521450	1	2	2
1	MA	REGIONAL	1441	TASSO FRA	211200	1	2	
1	SP	GVE XVII C/	1342	HORTOLAN	351907	1	2	2
1	PA	04 REGION	1487	CAPANEM/	150220	1	2	2
1	PR	02RS METF	1356	SAO JOSE D	412550		2	2
1	SP	GVE X OSA	1335	BARUERI	350570	1	2	9
1	SP	GVE XXXI S	1353	SOROCABA	355220	1	2	2
1	SP	GVE XXIX S	1354	SAO JOSE D	354980	1	2	2
1	PR	02RS METF	1356	CURITIBA	410690	1	2	2
1	RS	001 CRS	1592	NOVO HAN	431340	1	2	2
1	CE	16 CRES CA	1526	CHAVAL	230390			
1	BA	NUCLEO RE	1380	SALVADOR	292740	1	2	
1	PR	02RS METF	1356	ITAPERUCU	411125		2	2
1	GO	CENTRAL	1779	GOIANIA	520870	1	2	2
1	PR	09RS FOZ D	1363	MATELAND	411560	1	2	2
1	PR	04RS IRATI	1358	IMBITUVA	411010	2	2	2
1	PR	13RS CIAN(	1367	CIANORTE	410550	1	2	2
1	SP	GVE XI ARA	1336	BIRIGUI	350650	1	2	2
1	SP	GVE I CAPI	1331	SAO PAULC	355030	1	2	2
1	GO	SUDOESTE	1800	MINEIROS	521310	1	2	2
1	RJ			BELFORD R	330045	1	2	9
1	SP	GVE X OSA	1335	OSASCO	353440	1	2	2
1	SC	CRICIUMA	1558	CRICIUMA	420460		2	2
1	BA	NUCLEO RE	1380	SALVADOR	292740		9	9
1	RS	003 CRS	1593	PELOTAS	431440	1	2	2
1	SC	JOINVILLE	1565	JOINVILLE	420910	1	2	2
1	SP	GVE XVIII F	1343	RIFAINA	354360	1	2	2
1	SP	GVE XVII C/	1342	CAMPINAS	350950	1	2	2
1	PA	13 REGION	1496	BAIAO	150120	1	2	2
1	MG	POUSO ALE	1459	ANDRADAS	310260	1	2	2
1	AL	1 MICRORE	1533	MACEIO	270430	1	2	2
1	RJ			RIO DE JAN	330455			
1	PR	09RS FOZ D	1363	FOZ DO IGU	410830	1	2	1
1	SP	GVE X OSA	1335	CARAPICUI	351060	1	2	2
1	TO			TOCANTIN	172120	1	2	2
1	SP	GVE VII SAI	1332	SAO CAETA	354880	1	2	2

1	RS	003 CRS	1593	PELOTAS	431440	1	2	2
1	PR	09RS FOZ D	1363	FOZ DO IGU	410830	1	2	2
1	TO			TOCANTIN	172110	1		
1	SP	GVE XII AR/	1337	SAO CARLO	354890	1	2	9
1	PR	10RS CASC	1364	CASCAVEL	410480	1		
1	MG	PATOS DE I	1456	PATOS DE I	314800	1	2	2
1	TO			MIRACEMA	171320	1	2	2
1	PR	10RS CASC	1364	VERA CRUZ	412855	1	2	
1	MT	BARRA DO	1580	BARRA DO	510180	1	2	2
1	AM	ENTORNO	5584	MANAUS	130260	1	2	2
1	SP	GVE I CAPIT	1331	SAO PAULO	355030	1	2	2
1	BA	NUCLEO RE	1380	SALVADOR	292740	1	2	9
1	SP	GVE I CAPIT	1331	SAO PAULO	355030	1	2	2
1	PR	10RS CASC	1364	CASCAVEL	410480	1	2	
1	MG	PATOS DE I	1456	PATOS DE I	314800	1		
1	SC	JARAGUA D	1567	SCHROEDE	421740	1	2	2
1	MG	PATOS DE I	1456	PATOS DE I	314800	1	2	2
1	PA	11 REGION	1494	PARAUAPE	150553	1	2	2
1	PR	20RS TOLEI	1374	TOLEDO	412770	1		
1	RJ			ITAPERUNA	330220	1	2	2
1	BA	NUCLEO RE	1385	JEQUIE	291800	9	9	9
1	SP	GVE XXXI S	1353	SOROCABA	355220	1	2	2
1	PR	10RS CASC	1364	CASCAVEL	410480	1	2	
1	PA	01 REGION	1484	ANANINDE	150080	1	2	2
1	ES	METROPOL	1510	SERRA	320500	1	2	2
1	SC	CRICIUMA	1558	COCAL DO	420425	1	2	2
1	PR	21RS TELEI	1375	ORTIGUEIR	411730		2	2
1	PR	10RS CASC	1364	SANTA TER	412402		2	
1	PR	10RS CASC	1364	CASCAVEL	410480	2		
1	PA	09 REGION	1492	ALMEIRIM	150050	1	1	2
1	PR	09RS FOZ D	1363	FOZ DO IGU	410830	1	2	2
1	BA	NUCLEO RE	1380	LAURO DE	291920	9	9	9
1	PR	02RS METR	1356	CAMPO LA	410420		2	2
1	SP	GVE X OSA	1335	OSASCO	353440	1	2	2
1	SP	GVE I CAPIT	1331	SAO PAULO	355030	1	2	2
1	SP	GVE XIII AS	1338	SANTA CRU	354640	1	2	2
1	SC	CRICIUMA	1558	COCAL DO	420425	1	2	2
1	RJ			RIO DE JAN	330455	1		
1	PR	10RS CASC	1364	CASCAVEL	410480	1	2	
1	SP	GVE XXVII S	1351	SAO JOSE D	354990	1		
1	SP	GVE XXII PI	1573	IRAPURU	352160	1	2	2
1	PI	4 GRS TERE	1883	TERESINA	221100	1	2	2
1	PB	I NRS JOAO	1377	JOAO PESS	250750	1	2	2
1	RS	001 CRS	1592	IVOTI	431080	1	1	
1	MG	PATOS DE I	1456	SERRA DO	316680	1	2	2
1	RJ			RIO DE JAN	330455	1	9	9
1	SP	GVE VII SAI	1332	SANTO AN	354780	1	2	2

1	PR	10RS CASC	1364	CAPITAO LI	410460	1	2	
1	PR	09RS FOZ D	1363	FOZ DO IGU	410830	1	2	2
1	MG	MANHUAC	1454	SANTA MA	315790	1	2	2
1	SP	GVE XXVII S	1351	SAO JOSE D	354990	1	2	2
1	RJ			SAO JOAO	330510	1	2	2
1	RJ			RIO DE JAN	330455	1	9	9
1	MG	UBERLAND	1462	UBERLAND	317020	1	2	2
1	RJ			NOVA IGUA	330350	1	2	2
1	SP	GVE XVII CA	1342	VARZEA PA	355650	1	2	
1	GO	CENTRAL	1779	GOIANIA	520870	1	2	2
1	BA	NUCLEO RE	1381	NORDESTIN	292265	9	2	2
1	PI	1 GRS PAR	1868	PARNAIBA	220770	1		
1	AP			SANTANA	160060	1	9	2
1	RJ			RIO DE JAN	330455	9		
1	SP	GVE VII SAI	1332	MAUA	352940	1	2	2
1	SP	GVE X OSA	1335	SANTANA I	354730	1	2	9
1	SP	GVE X OSA	1335	BARUERI	350570	1	2	9
1	SP	GVE VII SAI	1332	DIADEMA	351380	1	2	2
1	SP	GVE XXI PR	1346	PRESIDENT	354140	1	2	2
1	MA	REGIONAL	1439	BURITICUP	210232	1	2	2
1	SP	GVE I CAPI	1331	SAO PAULO	355030	1	2	2
1	SP	GVE XX PIR	1345	LEME	352670	1	2	2
1	PB	III NRS CAM	1421	SERRA RED	251580	1	2	2
1	SP	GVE XVI BO	1341	SAO MANU	355010	1	2	2
1	PR	10RS CASC	1364	CASCAVEL	410480	1	2	
1	SP	GVE I CAPI	1331	SAO PAULO	355030	1	2	2
1	PR	10RS CASC	1364	CASCAVEL	410480	2	2	
1	GO	CENTRAL	1779	GOIANIA	520870	1		
1	SP	GVE VII SAI	1332	SAO BERNA	354870	1	2	2
1	RJ			ITAPERUNA	330220	1	2	2
1	PR	09RS FOZ D	1363	FOZ DO IGU	410830	1	2	2
1	SP	GVE VIII M	1333	GUARULHO	351880	1	2	2
1	MA	REGIONAL	1440	IMPERATRI	210530			
1	RJ			NOVA FRIB	330340	1	2	2
1	MG	BELO HORI	1449	BELO HORI	310620	1	2	2
1	SP	GVE I CAPI	1331	SAO PAULO	355030	1	2	2
1	MG	VARGINHA	1466	ELOI MEND	312360	1	2	2
1	RJ			ITAPERUNA	330220	1	2	2
1	PR	09RS FOZ D	1363	FOZ DO IGU	410830	1	2	2
1	PA	11 REGION	1494	MARABA	150420	2	2	2
1	AP			MACAPA	160030	1	2	2
1	GO	SUDOESTE	1800	JATAI	521190	1	2	2
1	MG	BELO HORI	1449	BELO HORI	310620	1	2	2
1	MG	VARGINHA	1466	AIURUOCA	310120	2	2	2
1	CE	8 CRES QU	1517	QUIXERAM	231140	1	2	9
1	SP	GVE XIX MA	1344	GARCA	351670	1	2	2
1	RJ			ITAPERUNA	330220	1	2	2

1	SP	GVE I CAPIT	1331	SAO PAULC	355030	1			2	2
1	PR	09RS FOZ D	1363	MATELAND	411560	2			2	2
1	PR	10RS CASC	1364	DIAMANTE	410712	1				
1	SP	GVE I CAPIT	1331	SAO PAULC	355030	1			2	2
1	PI			ITAUEIRA	220510	1			2	2
1	SP	GVE X OSA	1335	ITAPEVI	352250	9			2	2
1	PR	17RS LOND	1371	CAMBE	410370	1			2	2
1	MG	PATOS DE I	1456	PATOS DE I	314800	3			2	2
1	SP	GVE XXVII S	1351	JACAREI	352440	1			2	9
1	SC	ARARANGU	1562	ARARANGU	420140				2	2
1	SP	GVE XVII CA	1342	HOLAMBRA	351905	1			2	9
1	BA	NUCLEO RE	1381	IRARA	291450	2			2	1
1	SC	LAGES	1571	CAPAO ALT	420325	3			2	2
1	RS	007 CRS	1596	BAGE	430160	1			2	2
1	SP	GVE XXXII I	1575	ITAPEVA	352240	1			2	2
1	AP			MAZAGAO	160040	2			2	2
1	SP	GVE XIX MA	1344	MARILIA	352900	1			2	2
1	PR	10RS CASC	1364	VERA CRUZ	412855	1				
1	RS	001 CRS	1592	SAO LEOPC	431870	1			2	2
1	MS	EX NRS DE	6256	CORUMBA	500320	1			2	2
1	MG	POUSO ALE	1459	POCOS DE	315180	2			2	2
1	SP	GVE XVII CA	1342	MONTE MO	353180	1			2	9
1	SP	GVE VII SAI	1332	SANTO AN	354780	1			2	2

FEBRE	TOSSE	GARGANTA	DISPNEIA	DESC_RESP	SATURACA	DIARREIA	VOMITO	OUTRO_SIN	OUTRO_DES
1	1	2	1	1	1	2	2	1	MIALGIA
2	1	1	1	1	1	2	2	2	
		1				1		1	CEFALEIA
1	1	1	1	1	1	2	2	1	MIALGIA/ F
1	1	2	1	2	2	2	2	1	CORIZA, DC
1	1	1	1	1	1	1	2	2	
			1	1	1				
2	1	2	1	2	2	2	2	2	
1	2	2	2	1	2	2	2	2	
	1				1				
	1		1	1	1				
1	1	2	1	1	1	2	2	2	
1	1	2	1	1	1	2	2	2	
1					1			1	ODINOFAG
2	2	2	1	2	2	2	2	2	
1	2	2	2	1	1	1	2	2	
1	1		1	1	1			1	CORIZA,MI
2	1	1	1	1	1	2	2	2	
1	1	2	1	1	2	2	1	1	ASTENIA, N
2	1	2	1	1	1	2	2	2	
1	1		1	1	1				
1	1	2	1	1	1	1	2	1	CORIZA MI
1	1	2	1	2	2	2	2	1	CEFALEIA E
1	1	2	2	1	2	2	2	1	CEFALEIA
1	1	2	1	2	1	2	2	2	
1	1				1			1	MIALGIA
1	1	2	1	1	1	2	2	2	
1	2	2	1	1	2	2	2	1	CEFALEIA N
1	1	2	1	1	1				
1			1	1	1				
1	1	2	1	1	1	2	2	1	CORIZA, NA
1	1	2	1	2	1	2	2	2	
2	1	2	1	1	1	2	2	1	CEFALEIA
2	1	2	1	2	2	2	2	2	
2	2	2	2	1	1	2	2	1	CORIZA
1	1	1	1	2	2	1	2	1	MIALGIA, C
1	1	1	1	1	1	2	2	2	
1	2	2	2	1	1	2	1	1	NAUSEA
1	1	2	1	1	1	2	2	1	ANOSMIA,
1			1	1					
2	1	2	1	1	1	2	2		
1	1	2	1	1	1	2	2	2	
2	1	2	1	2	2	2	2	1	CONGESTA
	1		1					1	POLIMIALG
1	1		1	1	1				
1	2	2	1	1	1	2	2	2	

1	1		1		1			1	ANOSMIA,
2	1	1	1	1	1	2	2	1	MIALGIA, C
	1		1	1	1	2	2	1	MIALGIA, C
1	1		1	1		1	1	1	ASTENIA, M
1	1	2	2	2	2	2	2	1	MIALGIA, A
1	1	2	1	1	2	2	2	2	
2	1	2	1	1	1	2	2	1	DOR DE GA
	1						1	1	CEFALEIA
1	1	2	2	2	2	2	2	9	
1	1	1	1	1	1	2	1	1	CEFALEIA E
	1		1		1			1	CONGESTA
1	1	9	1	2	2	2	2	2	
1	2	2	1	1	1	2	2	1	CEFALEIA E
1	1	2	2	2	2	1	1	2	
2	1	2	1	1	1	2	2	2	
1	1	1	1	1	1	2	2	1	MIALGIA D
1	1		1		1			1	ANOSMIA
				1					
2	2	2	2	2	2	2	2	1	ASSINTOM
1	9	9	1	9	9	9	9	9	
1	1	2	1	2	2	2	2	1	CEFALEIA E
1	1	1	1	1	2	2	2	2	
1	2	2	1	1	1	2	2		
1	1	1	1	1	1	2	2	1	ANOSMIA
1	1	2	1	1	1	2	2	2	
1	2	2	1	1	1	2	2	2	
2	1	2	2	1	2	2	1	1	HIPOTENSA
2	2	2	1	1	1	2	2	1	DOR TORA
1	1	2	1	1	1	2	2	1	MIALGIA
2	1	2	1	1	2	2	2	2	
1	1	2	2	2	2	2	2	1	FALTA DE A
2	1	2	2	2	2	2	2	2	
1	1	2	1	1	1	2	2	2	
1	1	1	1	1	1	2	2	2	
2	1	2	1	1	1				
1	1		1	1	1			1	MIALGIA
1	1	2	2	2	2	2	2	2	
1	1	2	2	2	1	2	2	1	CEFALEIA C
2	1	2	1	2		2	2	1	MIALGIA E
2	1	1	1	1	1	2	2	1	MIALGIA
2	2								
2	1	2	1	1	1	2	2	1	CEFALEIA
1	1	1	1	1	1	2	1	9	
1	2	2	1	1	1	2	2	1	MIALGIA, C
1	1	1		1	1				
1	1	1	1	1	1	2	2		
2	1	2	1	2	2	1	2	2	

1	2	2	1	2	2	2	2	2	
2	1	2	1	1	1	2	2	1	TAQUICAR
1	1	1	1	1	1	2	1	2	
2	1	2	2	2	2	2	2	1	CEFALEIA
1	1		1	1	1			1	MIALGIA
1	1	2	1	1	1	1	2	1	TAQUICAR
2	1	1	2	2	1	2	2	1	CORIZA, G
1	1	1	1	1	1	1	2	9	
2	2	2	1	1	1	2	1	1	DOR TORA
2	1	1	1	1	1	2	2	1	ESPIRROS
2	2	2	2	2	2	2	2	1	CEFALEIA
1	1				1			1	MIALGIA
1	1		1	1	1		1		
2	1	2	1	2	1	2	2	2	
1	2	2	2	2	2	2	2	1	PERDA DE
1	1	1	2	2	2	2	2	2	
1	2	2	1	1	1	2	2	2	
1	9	9	1	1	9	9	9		
1	1			1	1			1	CEFALEIA
1	1	2	1	2	2	2	2	2	
1	1	2	1	2	1	2	2	1	CEFALEIA,
1	1	2	1	2	2	2	2	1	MIALGIA
1	1	2	1	1	1	2	2	2	
1	1	2	1	1	1	2	2	1	CEFALEIA,
1	1	2	1	1	1	2	2	2	
	1		1	1				1	MIALGIA
1	1	1	2	1	2	2	2	1	ANOSMIA,
	1								
1	1	2	1	2	1	2	1	1	MIALGIA,
1	2	2	2	2	2	2	1	1	CEFALEIA,
			1	1					
2	1	2	1	1	1	2	2	1	DOR TORA
1	2	2	1	2	1	2	2	1	DOR TORA
			1	1	1				
2	1	2	1	1	1	2	2	1	CEFALEIA
1	1	2	1	2	2	2	2	1	ADNAMIA
			1						
1	1	1	1	1	2	2	2	2	
1	1	1	2	2	2	2	2	1	ANOSMIA+
2	2	2	1	1	1	2	2	2	
1	2	2	2	1		2	2		
1	1	1	2	1	2	2	2	2	
1	1	1	1	1	2	1	1	2	
1	1		1	1	1			1	CORIZA, M
1	1	2	2	2	2	2	2	1	ASTENIA
1	1	2	1	2	1	2	2	2	
2	1	1	2	2	2	2	2	2	

1	1		1	1	1			1	ANASARCA
2	1	2	1	2	2	2	2	2	
			1	1	1				
2	1	2	1	1	2	2	2	2	
1	1	2	1	1	1	2	2	2	
1	1	1	1	1	1				
9	1	9	1	2	2	9	9	1	ANOSMIA,
1	2	2	2	2	2	2	2	2	
1	1	2	1	1	1	2	2	1	CEFALEIA
1	2	2	1		1	1	1		
1					1				
1	1	1	2	1	1	2	1	1	ANOSMIA
2	1	1	1	1	1	2	2	2	
1	1	1	1	1	1	2	2	2	
1	1	2	2	1	2	2	2	1	CEFALEIA,
1	1	2	1	1	1	2	2	1	ASTENIA, P
2	2	2	2	2	2	2	1	1	DOR ABDO
1			1					1	TAQUICAR
1	1	2	1	1	1	2	2	2	
1	1	2	2	1	1	2	2		
2	2	2	1	1	1	2	2	2	
1	1	1	1	1	1	1	1	1	CEFALEIA/
1	1	2	1	2	2	2	1	1	VPMITO, N
1	1	2	1	1	1	2	2	2	
1	1	2	1	1	1	2	2	2	
1	1	2	1	2	1	2	2	1	CORIZA
1	1	2	1	1	1	2	1	2	
9	1	2	1	9	1	9	9	9	
1	1	2	1	2	1	2	2	2	
1	1	1	1	1	1	1	1	2	
2	1	2	2	2	2	2	2	2	
1	1	2	1	2	2	2	2	9	
	1				1			1	CORIZA,OB
2	2	2	1	1	2	2	2	9	
1	1	2	1	1	1	2	2	2	
2	1	1	1	1	2	2	2		
1	1	1	1	1	1	9	9	1	MIALGIA, A
1	1	2	2	1	2	2	2	1	DOR NO C
2	1	2	2	2	2	2	2	1	CEFALEIA,
2	1	2	1	2	1	1	2	2	
1	1	2	2	2	2	2	2	1	CORIZA
1	2	2	2	2	2	1	2	2	
1	1	2	1	1	1	1	1	2	
			1		1				
1	1	2	1	1	1	2	2	2	
2	2	2	1	2	2	2	2	2	
1	2	2	2	2	2			1	DOR LOMB

	1		1					1	MIALGIA
1	1	2	2	2	2	2	2	2	
1	1	1	1	1	1	2	2		
1	1	2	1	1	1	2	2		
1	1	1	1	1	2	2	2		
2	1	2	1	1	1	2	2	2	
1	1	2	2	2	2	1	2	2	
1	1	2	1	1	2	2	2	2	
2	1	2	2	2	2	2	2	1	CEFALEIA,
1	1	2	1	1	2	2	2	2	
1	2	2	2	2	2	2	1	2	
2	1	2	1	1	1	2	2		
1	1	2	1	1	1	2	2	1	CEFALEIA,
1	1	2	1	1	1	2	2	2	
2	1	1	2	1	2	2	2	1	PERDA DE
1	1	1	1	1	1	2	1	2	
1	1		1	1	1				
9	1	9	9	9	1	9	9	1	CORIZA E C
1	1	2	1	2	1	2	2	2	
1	1	1	1	2	1	2	2	1	CEFALEIA
1	1		1	1	1				
2	2	1	1	1	2	2	2	1	DOR TORA
2	2	2	2	2	2	2	2	2	
			1				1	1	MIALGIA N
1	1	2	2	2	2	2	2	9	
2	2	2	2	2	2	2	2	2	
1	1	2	1	1	2	2	2	2	
1	1		1					1	MIALGIA
1	1	1	2	2	1	1	1	9	
1	1	2	1	1	1	2	2	2	
1	1	2	1	1	1	2	2	1	PERDA OLF
2	1	2	2	2	2	2	2	1	CEFALEIA,
1	2	2	1	2	1	2	2	2	
1	1	1	1	1	1	2	2	1	CEFALEIA/
2	1	2	1	2	1	2	2	2	
1	1		1		1			1	CEFALEIA,
1	1	2	1	2	2	2	2	1	MEG, CEFA
2	2	2	2	2	2	2	2	1	ASSINTOM
1	1	1	2	1	2	2	2	1	TAQUIDISP
1	1	2	1	1	1	2	2	2	
2	1	2	1	1	1	2	2		
1	1	2	1	2	1	2	2	2	
1	1	2	1	1	1	2	2	1	CEFALEIA
1	1	2	2	1	2	2	2	2	
1	1	2	1	2	1	1	2	1	CEFALEIA,
	1							1	CANSACO
1	1	2	1	1	1	2	1	1	DOR NO C

1	1	1	1	1	2	2	2	2	
1	1	2	1	2	2	2	2	1	MIALGIA, F
2	1	1	1	1	1	2	2	2	
2	1	2	1	1	2				
1	1	2	1	1	1	2	1	9	
2	1	2	2	9	2	2	2	1	FRAQUEZA
2	2	1	1	1	1	2	2		
1	1	1	1	1	1	1	2	1	MAL ESTAF
1	1	2	1	1	2	2	2	2	
1	1								
1			1						
1	1			1		1		1	ANOSMIA
1	2	2	1	2	1	2	2	1	CEFALEIA, (
1							1	1	MIALGIA
1	1	2	2	1	2	2	2	1	TAQUIDISP
1	1	2	1	1	1	2	2	1	
1	1	2	2	2	2	2	2	2	
2	1	2	1	2	1	2	2	2	
1	1	2	1	2	2	2	2	1	DORES NO
1	1	2	1	2	2	2	2	1	MIALGIA/C
1	1	1	1	1	1				
2	1	2	1	1	2	1	2	1	MIALGIA
1	1	1	1	1	1				
1	1	2	2	1	1	2	2	2	
2	2	2	2	1	1	2	2	2	
	1		1					1	CORIZA E N
1	1	1	1	1	2	1	2	1	OBSTRUCA
1	1	1	1	2	1	2	2		
1	1		1	1	1				
1									
1	2	2	1	1	1	2	2	1	CEFALEIA, (
1	9	9	1	9	1	9	9	9	
1	1	2	1	2	1	2	2	2	
1			1					1	DOR TORA(
1	1	1	1	1	1	2	2	1	CONGESTA
1	2	2	1	2	2	2	2	9	
1	1	2	1	1	2	2	2	1	DORMENCI
			1	1					
1			1		1				
2	2	2	1	1	1	2	2	1	ASTENIA E
1	1	2	2	1	1	2	2	1	MIALGIA, C
	1		1	1	1				
2	2	2	1	1	1	2	2	1	DOR EM VI
2	1	2	2	1	2	2	1	2	
	1		1	1	1				
1	1	2	1	1	1	2	2	2	

2	1	1	2	2	2	2	2	1	ANOSMIA
	1		1					1	CEFALEIA,
1	1	2	1	1	2	2	2	2	
1	2	2	1	2	2	2	2	1	SINAIS GRI
1	1	2	1	2	2	2	2	2	
1	1	1	2	2	2	2	2	1	CANSACO
1	1		1	1	2		1		
1	1	2	9	9	2	1	2	1	CORIZA E
	1			1				1	CEFALEIA,
1	1		1						
1	1	2	1	1	1	2	2	2	
1	2	2	1	2	2	2	2	1	CEFALEIA,T
1	1	1	1	1	1	2	2	2	
1	1	2	2	2	2	2	2	2	
2	1	2	1	1	2	2	2	1	CEFALEIA
	1							1	CORIZA
2	2	2	1	1	1	2	2	2	
1	1	2	2	2	2	2	2	1	MAILGIA/D
2	1	2	2	1	1	2	2		
1	1							1	CEFALEIA,C
1	1	2	2	2	2	2	2	1	MIALGIA/C
1	1	2	1	2	1	2	2	1	CEFALEIA
1	1			1				1	CEFALEIA,
1			1	1			1	1	ICTERICIA,
	1		1		1				
1	1	2	2	1	2	2	2	2	
1	2	2	2	1	1	2	2	2	
1	1		1					1	TAQUICAR
2	1	1	2	2	2	1	2	2	
			1	1	1				
2	1	2	1	1	1	2	2	2	
1	1	2	1	1	1	2	2	1	GEMENTE,
	1	2	1	1	1	2	2	2	
9	9	9	1	1	1	2	2	1	ORTOPNEI
	1		1	1				1	CEFALEIA,
	1		1						
1	1			1	1			1	CEFALEIA
1	1	2	1	2	2	2	2	9	
1	1	2	1	1	1	2	2	2	
1	1		1					1	MIALGIA
1	1	1	1	1					
1	1	2	1	2	1	2	2	1	MIALGIA
1	2	2	2	2	2	2	2	1	MIALGIA ;
2	1	2	2	2	2	2	1	1	NAUSEAS
1			1		1		1		
1	1	1	1	2	2	2	2	2	
1	1	2	1	2	2	2	2		

2	1	2	2	2	2	2	2		
	1		1	1	1				
2	2	2	1	1	2	2	2	2	
2	2	2	2	2	2	2	1	1	CEFALEIA
1	1							1	MIALGIA
1	1		1	1	1			1	
2	1	1	2	2	2	2	2	2	
2	1	2	2	2	1	2	1	2	
1	1	1	1	1	2	2	2	1	CEFALEIA
9	1		1						
1	1	2	1	1	2	2	2	1	ANOSMIA
1	1	2	1	1	1	2	2	1	DOR NO C[illegible]
1	1		1		1			1	CEFALEIA,[illegible]
2	1	2	1	1	1	2	2	2	
1	2	2	2	2	2	2	1	2	
2	1	2	2	1	1	1	2	1	MIALGIA,C
2	1	2	1	1	1	2	2	2	
1	1	2	1	1	1	2	2	1	ANOSMIA
	1		1	1	1				
	1		1		1			1	MIALGIA
1	9	1	1	1	1	9	9	1	CEFALEIA, [illegible]
1	1	2	2	1	1	2	2	1	CEFALEIA E
1	1		1	1			1	1	ANOSMIA/
2	2	2	1	1	2	2	2	1	ANOSMIA/
2	1	2	1	1	1	2	2	2	
1	1	1	1		1			1	FADIGA
1		2	1	2	1	1	2	1	CEFALEIA
1	1	1	1	2	2	2	2	1	HB 5,1, PC[illegible]
1	1	1	1	1	2	2	2	1	CEFALEIA +
2	1	2	2	1	2	2	2	2	
1	1	1	2	2	1	2	2	2	
1	1	2	1	2	1	1	2	1	MIALGIA
1	1	2	1	1	1	2	2	2	
1	1		1	1	1				
2	1	2	1	1	2	2	2	2	
1	1	2	1	1	1	2	1	1	CEFALEIA, [illegible]
1	1	2	1	1	2	2	1	2	
				1	1			1	CANSACO,
1	1	2	1	2	2		2	2	
			1	1	1				
1	1	2	2	1	2	1	2	2	
1	1	2	1	1	1	2	2	1	CEFALEIA
1	1	2	1	2	1	2	2	1	TAQUIPNE
2	2		1	1	1			1	TAQUICAR[illegible]
2	2	2	2	1	2	2	2	2	
1	1	1		1		1	1		
1	1	2	2	2	2	2	2	2	

1			1		1			1	CANSACO
1	1	2	1	1	1	2	1	2	
1	1	1	2	2	2	2	1		
1	2	1	2	2	9	1	2	2	
1	1		1	2		1	2	1	MIALGIA/C
1	1	1	1	2	2	2	2	2	
1	1		1						
2	1	2	1	2	1	2	2	1	MIALGIA
2	1	2	1	1	1	2	2	2	
1	2	2	1	1	2	2	2	2	
								1	CEFALEIA
	1		1	1	1				
	1		1		1			1	TAQUICARI
2	1	1	2	2	2	2	2	2	
	1		1		1			1	CEFALEIA
1	1	2	2	1	2	2	2	2	
9	1	9	1	9	9	9	9	9	
1	2	2	2	1	1	2	2		
1	1	2	1	2	2	2	2		
1	1	2	2		2	2	2	1	OBSTRUCA
1	1	9	1	1	1	9	9	2	
2	1		2	2	2	2	2	2	
	1		1	1	2	2	2	2	
2	1	1	1	1	2	2	2	2	
1	1	2	2	1	2	2	2	2	
2	1	2	1	1	1	2	2		
1	1	2	1	1	1	2	2	1	MIALGIA/C
9	2	2	2	1	1	2	2	2	
1	2	1	1	1	2	2	2	1	DORSALGI
1	1	1	1	2	2	2	2	1	DIMINUICA
2	2	2	2	1	1	2	1	1	TAQUICARI
1	1	2	1	2	2	2	2	2	
1	2	2	1	1	1	2	2	1	CALAFRIOS
1	1	2	2	1	1	1	2	1	FRAQUEZA
1	1	2	1	2	2	2	2	1	CEFALEIA, I
2	2	2	2	2	2	2	2	1	SEM INTER
2	1	2	1	2	2	2	2		
1	1	2	1	1	2	2	2	2	
1	1	2	1	2	2	2	2	2	
1	1	1	1	1	1	1	1	2	
1	1		1	1	1			1	CEFALEIA
2	1	1	1	1	2	2	2	2	
1	1	2	1	9	9	2	2	1	MIALGIA
2	1	2	2	2	1	1	2	9	
1	2	2	2	2	2	2	2	1	CONGESTA
1	1	2	1	1	2	2	2	2	
	1								

1	1	2	1	2	2	2	2	1	MIALGIA, A
1	1		1					1	CORIZA,CE
1	1		1					1	DOR TORA
1	1	2	1	1	1	2	1	2	
2	1	2	1	2	2	2	2	1	PARESTESI
	1		1	1	1				
2	2	2	2	2	2	2	2	2	
1	1	2	1	2	1	2	2		
2	1	2	1	1	1	2	2	2	
1	1	1	1	1	2	2	2	2	
1	1	2	2	1	1	2	2		
1	1	2	1	1	1	2	2	1	TAQUICAR
			1	1				1	ANOSMIA
1	1	2	1	1	1	1	2	1	ADINAMIA,
1	1	2	1	1	2	2	2	2	
1	1	2	2	1	1	2	2	1	NAUSEAS
1								1	DOR LOMB
2	1	2	1	1	2	2	2	1	DOR TORA
1	1	2	2	1	2	2	2	2	
			1	1	1				
1	1		1	1	1				
1	1	1	1	1	2	2	2	2	
2	1	2	1	2	2	2	2	1	MAL ESTAR
1	2	2	1	1	1	2	2	2	
1	1	1	1	1	2	1	1		
	1		1			1			
1	1	1	1	1	1	1	2	2	
2	2	2	2	2	2	2	2	2	
1	2	2	2	1	1	2	2	2	
1	1		1					1	CEFALEIA E
1	1	2	1	1	1	2	2	2	
1	1	2	1	1	1	1		1	MAL ESTAR
1	1		1	1	1			1	DOR LOMB
1	2	1	1	2	2	2	1	2	
2	1	2	1	1	1	2	2	2	
1	1	1	1	1	1	2	2	2	
1	1	2	2	1	2	2	2	1	CEFALEIA
2	2	2	1	1	1	2	2	2	
1	1	1	2	1	1	2	2	2	
1	1	2	1	1	1	2	2	2	
1	1	2	1	1	2	2	2	1	DOR TORA
2	1	2	1	1	2	2	2	1	CEFALEIA
	1		1						
1	1	1	1	1	1	2	2		
1	1		1			1	1	1	MIALGIA
1	1	1	1	1	1	1	1	2	

1	2	2	1	1	2	2	2	1	ADINURIA,
1	1	1	1	1	1	2	2	2	
2	2	2	1	2	2	2	2	2	
1	1		2	2	2	2	2	1	CONGESTA
2	1	2	1	1	2	2	2	1	NAUSEA, C
1	1	2	1	1	9	1	2	2	
1	1	1	1	1	1	2	2	2	
1	1	2	1	2	1	2	2	1	CEFALEIA
	1		1			1		1	MIALGIA, A
1	1	2	1	1	2	2	2	1	CEFALEIA
1	1	2	1	1	1	2	2	1	CONFUSAC
1	1	1	1	1	1				
1	1		1	1		1		1	ANOSMIA
1	1	2	1	1	2	2	2	1	MIALGIA,
1	1	1	1	1	2	2	1	2	
2	2	2	1	1	1	2	2	1	NAUSEAS
1	1	1	1	1	2	1		1	CEFALEIA,
1	1	2	1	2	1	2	2	1	CORIZA
1	1	2	1	2	1	2	2	2	
1	2	2	1	1	2	2	2	2	
1	1		1		1				
1	1	2	1	1	1	2	2		
2	1	2	1	1	1	2	2	2	
1	1	2	2	2	2	2	2	1	CEFALEIA
2	1	1	2	1	2	2	2		
1	1	2	1	2	1	2	2	1	CEFALEIA
2	1	1	2	2	2	2	2	1	CEFALEIA,
			1	1					
1	1	1	1	1	1		1		
2	1	2	1	1	2	2	2	1	MIALGIA+
			1				1		
1	1	2	1	1	1	2	2	2	
2	1	2	1	2	1	2	2	2	
2	2	2	2	2	2	2	2	1	CRISE CON
1	1		1	2	2	2	2	2	
1	1	2	1	2	1	2	2	1	CEFALEIA,
	1	1	1	1	1				
1	1	1	1	1	1			1	MIALGIA,C
1	1	2	1	1	1	2	2	1	CORIZA
1	1	2	2	1	1	2	2	2	
1	1		1	1	1			1	MIALGIA
1	1		1	1	1	2	2	2	
1	1	2	1	1	1	2	2		
1	1	2	1	1	1	2	2	2	
1	2	2	1	2	2	2	2	1	CANSACO,
1	1	2	1	2	2	2	2	1	CEFALEIA,

			1	1	1				
	1	1						1	MIALGIA
1	1	1	1		1				
1	1	2	1	2	1	2	2	2	
2	2	2	1	1	2	2	2	2	
2	1	2	1	2	1	2	2	2	
2	1	2	1	2	2	2	2	1	ASTENIA; M
1	1	2	1	1	2	2	2	2	
1	1	2	1	2	2	2	2	2	
			1					1	ARTROFIA
1	1	2	2	1	1	2	2	9	
2	1	2	1	2	2	2	2	2	
1	1	1	1	2	1				
1	1		1						
1	1	2	2	1	1	2	2	1	ANOSMIA,
1	1	9	1	1	1	2	1	2	
1	1	2	1	1	2	1	1	1	INAPETENC
1	1	2	1	1	1	2	2	2	
1	2	2	1	1	1	2	2	2	
1	1	2	1	1	2	2	1	1	CORIZA
1	1	2	1	1	1	2	2	2	
2	1	1	1	1	2	1	1		
1	1	2	1	1		2	2		
1	1	2	2	2	2	2	2	1	CEFALEIA ,
1	1	2	1	1	1	2	2	2	
			1	1				1	CEFALEIA
2	2	2	1	1	1	2	2	2	
1	1	2	2	1	2	2	2	1	MIALGIA,C
1	1	1	2	2	1	2	9	9	
1	1	2	1	1	1	2	2	2	
1	1	2	1	2	2	2	2	1	INAPETENC
			1					1	CORIZA, M
1	1	2	1	1	1	2	2	9	
					1				
1	1	2	1	1	1	2	2	2	
1	1	1	1	1	2	1	2	1	HIPOREXIA
1	1	2	1	1	1	2	1	1	CEFALEIA E
2	2	2	2	2	2	2	2	1	DOR TORAC
1	1	1	1	2	1	2	1		
	1	1	1	1					
	1		1						
2	1	2	1	2	1	2	2	2	
2	1	2	2	1	1	2	1	1	HAS
1	1	2	1	1	1	2	2	1	NAUSEAS
1	1		1	1	1				
1	1	2	1	1	1	2	2	9	
2	1	2	1	1	1	2	2	2	

1	1	2	1	1	1	2	2	1	MIALGIA
	1			1		1			
2	1	1	1	1	1	2	2	2	
1	1	2	1	1	1	2	2	2	
1	1	2	1	2	2	2	2	2	
2	1	2	1	1	2	2	2	2	
2	1	1	1	1	1	2	2	1	MIALGIA/C
1	1	2	1	1	1	2	2	9	
								1	CEFALEIA
1	1	2	2	2	2	2	2	1	DOR RETR
	1			1					
2	2	2	2	2	2	2	2	1	CORIZA+ O
1	2	2	2	2	2	1	2	1	DOR TORA
2	1	2	1	1	1	2	2	1	DOR NO C
1	1	2	2	2	2	1	2	1	MIALGIA
2	2	2	1	1	1	2	2	2	
1	1		1		1				
1	2	2	1	1	1	2	2	2	
1	1	1		1	1				
1	2	2	1	2	1	2	2	2	
1		1	1	1	1			1	TAQUICAR
2	2	2	1	1	2	2	2		
2	1	2	1	1	1	2	2	2	
1	1	2	2	2	2	2	2	1	TAQUICAR
1	1	2	1	1	1	2	1	2	
1	1	2	1	1	1	2	2	2	
1	1	2	1	1	2	2	2	2	
1	1	2	1	1	1	1	2	2	
1	1	1	1	1	1	2	2	1	CEFALEIA,
1	1	2	1	1	2	2	2	2	
1	1	1	1		1	2	2	1	MIALGIA,
1	1	1			1			1	CEFALEIA
								1	CANSACO
1	1	2	1	2	1	1	1	1	MIALGIA,
2	1	2	1	2	1	2	2	2	
1			1						
1	1	2	1	2	2	2	2	2	
1	1	2	1	1	1	2	2	2	
1	2	2	1	2	2	2	2	1	MIALGIA,
2	1	9	1	1	1	9	9	1	DISGEUSIA
2	1	2	2	2	2	2	2	1	CEFALEIA
1	1	2	1	1	1	2	2	2	
2	1		2	1	2	2	2	2	
2	2	2	1	2	2	2	1	1	CORIZA OB
1	1	2	1	1	1	2	2	2	
2	2	2	1	1	1	2	2	1	TAQUIPNE
1	1	1	1	1	1	2	2	1	ROUQUIDA

1	1		1		1			1	TAQUIPNE
1	1	1	1	2	2	1	2	1	CORIZA TA
1	1	2	2	2	2	2	2	1	TAQUIPNE
1	1	1	1	1	2	2	2	1	CEFALEIA,
1	9	9	1	1	1	9	9	9	
1		1	1	1	1				
1	1			1		1		1	MIALGIA
2	1	2	1	2	1	2	2	1	CEFALEIA
1	1		1	1	1	1			
2	1	2	1	2	2	2	1	2	
1	1	2	2	1	1	2	2	1	CEFALEIA,
1	1	1	1	1	1	2	2	2	
1	1	2	1	1	1	2	2	2	
1	1	9	1	9	1	9	9	1	CORIZA
1	1	2	1	2	1	1	2	2	
1	1	1							
1	1	2	2	2	2	2	2	2	
1	1	1	2	2	2	2	2	1	CORIZA,MI
			1	1		1	1		
			1	1	1				
2	1	2	1	1		2	2		
1	1		1					1	MIALGIA,
1	1	2	2	1	2	2	2	2	
1	1	1	1	1	1	2	2	2	
2	1	2	2	1	1	2	2	2	
1	1	2	2	1	1	2	2	2	
2	1	1	1	1	1	2	2	2	
			1	1	1				
1	1	2		1	1	2	2	1	MIALGIA
1	1	2	1	2	1	2	1		
1	1	2	1	1	1	1	2	2	
1	1	1	1	1	1	2	2	2	
1	1	2	1	1	1	2	2	2	
	1		1	1	1				
			1		1	1		1	MIALGIA,
1	1	2	2	2	2	1	2	2	
1	1	1	1	2	2	2	2	2	
2	2	2	2	2	2	2	2	1	ASSINTOM
	1				1			1	ASTENIA
1			1	1					
1	1		1	1	2			1	ANOSMIA,
1	1	2	1	1	1	2	2	2	
2	2	2	1	2	1	2	2	1	CEFALEIA;
1	1		1	1	1				
1	1	2	1	1	2	2	2	2	
1	1	2	1	2	2	2	2	2	

2	1	2	1	2	2	2	2	2	
1	1	2	1	2	2	2	2	2	
1	1	2	1	1	1	2	2	2	
1	1	2	1	2	2	2	2		
2	2	2	1	2	2	2	2	2	
2	1	2	1	1	2	2	2	1	ESTENIA IN
2	1	2	1	2	2	2	2		
								1	MIALGIA E
1	1	1	1	1	1	1	2	2	
2	2	2	2	2	2	2	2	1	TAQUICAR
1	1	2	1	1	1	2	2	2	
1	1	2	1	2	2	2	2	2	
2	2	2	2	2	2	2	2	1	ASSINTOM
1	2	2	1	2	1	2	2	1	DOR ABDO
1	1	2	1	1	1	2	2	2	
1	2	2	1	1	1	2	2	1	DORSALGIA
1	1	2	1	2	1	2	2	1	FADIGA, CE
1	1	2	2	2	2	2	2	2	
1	1	2	1	1	1	1	2	2	
1	1	2	1	1	1	2	2	1	ASTENIA,M
2	1	2	1	1	1	2	2	2	
1	1		1	1	1	1		1	TAQUIPNE
1	1	2	1	1	1	2	2	2	
2	1	2	2	2	2	2	2	2	
	1							1	CEFALEIA
1	1	1	1	2	2	2	2	1	CORIZA
1	1	2	1	2	2	2	1	1	INFECCAO
	1		1	1	1			1	CEFALEIA,M
1	1	2	1	1	1	1	2	1	ANOSMIA
1	1	2	1	2	1	2	2	2	
2	2	2	2	2	2	2	2	1	CEFALEIA;
2	1	2	1	1	2	2	2	2	
1			1	1					
2	1	2	1	1	1	2	2	1	DOR TORA
1	1	2	2	2	2	2	2	2	
1	1							1	MIALGIA
1	2	2	1	1	2	2	2	1	DIF RESPIR
1	1	1	1	1	2	1	1	1	DOR TORA
2	2	2	1	1	2	2	2	1	CEFALEIA E
1	1	2	1	1	2	2	2	2	
2	1	2	1	2	1	2	2	2	
1	1	1	1						
2	1	2	2	1	1	2	9	1	MIALGIA
	1	1		1					
2	2	2	1	1	1	2	2	2	
1	1		1						
1	1	1	1	1	1	1	2		

2	1	1	1	2	1	2	2	1	MIALGIA
	1		1	1	1				
1	1	2	2	1	1	2	2	2	
1	2	2	1	1	2	1	2	2	
1			1					1	MIALGIA
			1	1					
				1	1				
1	1	2	1	1	1	2	2	2	
1	1	1	1	1	2	2	2	1	CEFALEIA,M
	1		1		1				
1	1	2	2	2	2	2	2	2	
2	2	2	1	2	1		2	1	
1	1		1	1	1	2	2	2	
1	2	2	1	1	2	2	2	1	MIALGIA E
1	1	2	2	2	2	2	2	2	
1	1		1		1				
	1	1	1	1	1		1	1	CEFALEIA
2	2	1	2	2	2	2	2	1	NAUSEAS,
1	1	1	1	2	2	2	2	1	ANOSMIA,
1	1	1	2	2	2	2	2	1	OBITO FET
1	1		1	1	1			1	MIALGIA
2	2	2	1	1	1	2	2	2	
1	1	9	1	9	9	9	9	1	CORIZA
2	1	2	1	1	2	2	2	1	MIALGIA D
2	2	2	2	2	2	1	2	1	DOR NO CO
1	1		1			1		1	DOR TORAC
1	1	2	1	1	1	2	1	9	
1	1	2	1	1	2	2	1	1	MIALGIA
1	1	2	1	2	1	2	2		
1	2	2	2	2	2	2	2	1	DOR NO CO
1	2	1	1	1	1	1	1	1	CEFALEIA,
2	2	2	1	1	1	2	2	2	
2	2	2	1	2	2	1	1	1	DOR TORAC
2	1	2	2	1	2	2	2	2	
1	1	2	1	1	1	1	2	1	TONTURA
2	1	2	1	1	2	2	2	2	
1	1		1		1				
			1		1				
	1				1			1	MAL ESTAR
1	1	1	2	2	2	2	2	2	
1	1	2	1	1	2	2	2		
1	1	2	1	2	2	2	2	2	
1	1	2	1	2	2	2	2		
1	1	2	1	1	2	1	1	1	CORIZA, CE
1	1		1					1	DOR NO CO
2	2	2	2	2	2	2	2	1	DOR EMBA
1	1	2	1	1	2	2	2	2	

1	1	2	1	2	2	2	2	2	
1	1					1		1	CEFALEIA, I
1	1	2	1	2	2	2	2	2	
1	2	1					1		
1	1	9	1	1				1	ANOSMIA I
1	1	2	1	1	2	1	1	2	
2	1	2	1	1	1	2	2	1	CONGESTA
1	2	2	1	1	1	2	2	2	
1	2	2	2	2	2	2	2	2	
1	1	2	1	1	1	1	2		
2	1	1	1	2	1	2	2	1	CANSACO
1	2	2	2	2	2				
1	1		1		1				
1	2	2	2	2	2	2	2	1	MIALGIA
1	1	1	1	1	1	2	2	2	
1	1	1	1	1	1	2	2		
1	1	2	1	1	1	2	1	2	
1	1	1	1	1	1	1	2		
2	2	2	2	2	2	2	2	1	CEFALEIA
1	2	2	1	2	2	2	1	1	MIALGIA, N
1	2	1	1	1	2	2	2	1	MIALGIA, N
1	1	1	1	1	1	1	1		
1	1	2	1	1	1	2	2	2	
			1	1	1				
1	1							1	OTALGIA
1	1	2	1	1		2	2		
1	1	2	1	2	2	2	2	2	
1	2	1	2	2	2	2	2	1	CEFALEIA, I
2	1	2	1	2	2	2	2	1	CEFALEIA, I
1	1	2	1	1	1	2	2	1	SEM SINTO
1	1	2	1	2	2	2	2	1	CEFALEIA
1	2	2	1	1	1	2	2	2	
1	1	2	1	1	1	2	2	1	CORIZA E N
2	1	2	1	1	1	2	2	1	DOR TORAX
1	1	1	1	1	1				
1	1	2	1	1	1	2	2	1	CORIZA/MI
1	1	2	1	2	1	2	2	1	MIALGIA
2	1	2	1	1	2	2	2	2	
1	1	2	1		1	2	1	1	CEFALEIA,C
1	1		1					1	VOMITOS
								1	DOR EM BA
1	2	1	1	1	1	2	2	2	
2	2	2	1	1	1	2	2	2	
1	1								
1	1	1	1	1	1	2	2	2	
1	2		2	1	1	1	2	2	
1	1	2	1	1	1	2	2	1	MIALGIA, A

1	1	2	1	2	2	2	2	1	MIALGIA
1	1			1			1	1	CEFALEIA
	1							1	CORIZA
1	1	1	2	2	2	2	2		
2	2	2	2	2	2	2	2	1	TONTURA,
1	1	2	1	2	2	2	2	2	
9	9	9	1	1	1	9	9	1	SINTOMAS
1	1	2	1	1	1	2	2	2	
		1		1				1	TAQUICAR
1								1	MIALGIA, P
1	9	9	1	1	1	1	9	9	
1	1	9	1	1	1	2	2		
1	1	2	1	1	1	2	2	2	
1	1	9	1	1	9	9	9	9	
			1	1	1	1	1		
1	2	2	1	1	1				
2	1	1	2	2	2	2	2	2	
1	2	2	2	1	1	2	2	1	CORIZA, M
1	1	1	1	1	1	2	2	1	MIALGIA, T
1	1	2	1				2	1	MIALGIA, D
1	1		1	1	1				
	1		1	1	1			1	CORIZA
1	1	2	1	1	1	2	2	9	
2	1	2	1	2	1	1	2	2	
1	2	1	1	1	2	2	2	1	MIALGIA
2	1	2	1	2	2	2	1	2	
1	1	1	2	1	2	2	1	2	
1	1	1	1	2	1	2	2	2	
2	1	2	2	1	1	2	1	2	
			1	1	1				
1	1	1	1	1	1	1	1		
1	1								
1	1	2	2	2	2	2	2	1	CEFALEIA/
2	1	1	1	1	2	2	1		
								1	DOR EM HI
1	1	2	1	1	1	2	2	1	DOR TORA
2	1	2	1	2	2	2	2		
2	1	2	1	1	1	2	2	2	
1	2	2	2	2	2	2	2	2	
2	2	2	2	2	2	2	2	2	
2	2	2	2	2	2	2	2	1	CEFALEIA
1	1	2	1	1	2	2	1	1	TAQUICAR
2	2	2	2	1	2	2	2	1	CEFALEIA,
1	1		1	1				1	CEFALEIA
2	1	2	1	2	2	2	9	1	MIALGIA
1	2	2	1	2	1	2	2	1	ANOSMIA
			1	1	1				

2	1	2	1	2	2	2	1	2	
1	1	2	1	1	2	2	2	2	
1			1						
1	1	9	1	1	2	9	9	1	FADIGA, M
1	2	1	2	1	1	2	2	1	HIPOREXIA
2	2	2	1	1	1	2	2	1	OBESIDADI
1	1	2	2	1	2	2	2	1	CORIZA
1	1	2	1	2	2	1	1	2	
1	1	2	1	1	1	2	2	2	
								1	INS RES AG
1	1	2	1	1	1	2	2		
1	1	2	1	1	1	2	2	1	MIALGIA
1		2	1	1	1	2	2	2	
1	1	1	1	1	1	1	2	1	CEFALEIA
1	1							1	CALAFRIOS
1		1	1	1					
			1	1	1			1	DOR TORA
1	1	2	1	1	1	2	2	1	DOR TORA
1	2	2	1	2	1	2	2		
1	1	2	1	1	2	2	2	1	TAQUIPNEI
1	1	2	1	1	1	2	2	2	
1	1	2	1	1	1	2	2	1	DOR TORA
1	1	1	2	1	1	2	1	9	
2	1	2	1	2	2	2	9	2	
2	1	2	1	1	1	2	2	1	LOMBALGI
1	1	9	9	9	9	9	9	9	
			1		1				
1	1	1	1		1				
1	1	1	1	1	1			1	CORIZA,CEI
2	1	2	1	1	1	2	2	2	
1	1	1	1	1	1	2	2	2	
1	2	2	1	1	1	2	2	1	HIPOREXIA
1	1	2	1	2	1	2	2	1	CORIZA, OI
1	1	1	1	1	2	1	2	1	CORIZA, C
1	1	2	1	1	1	2	2	1	ASTENIA, C
1	1	2	1	1	1	2	1	2	
1	1								
1	1	2	1	2	1	2	2		
1	1	2	2		1	1	2		
1	2	2	2	1	2	2	2	1	DOR EM B
2	1	2	1	2	2	2	2	1	CORIZA
2	2	2	1	1	1	2	2	1	CEFALEIA
1	1	2	1	2	2	2	2	1	SINT. GRIP
1	1		1		1		1		
1	1		1	1				1	NAUSEA
1	1		1	1	1			1	MIALGIA
	1								

1	1	1	1	1	1				
2	1	2	1	2	1	2	2	2	
1	1	1	1	1	1	1	2	1	MIALGIA,D
1	1			2		1		1	CORIZA, C
1	1	1	1		1				
	1		1	1	1			1	OBS NASAL
1	1	2	1	1	1	2	2	1	CORIZA
2	2	2	2	1	2	2	2	1	SINT GRIPA
1	1	1	2	2	1	2	2	2	
	1		1		1				
1	1	2	2	1	1	2	2	1	ASTENIA, C
2	1	2	1	1	1	2	2	2	
1	1	1	1	9	1	2	2		
1	1	1	1	1	1	2	2	1	CEFALEIA E
			1		1			1	ASTENIA, D
1	1	2	1	1	1	1	2	1	HIPOXEMIA
1	1		1	1	1				
2	1	2	1	1	1	2	2	2	
1			1						
1	1	2	1	1	1	2	2	2	
2	2	2	1	2	2	2	2	2	
2	1	2	1	1	1	2	2		
1	1								
2	1	2	2	2	2	2	2	2	
2	1	2	1	1	1	2	2	2	
2	2	2	2	2	2	2	2	1	SINT GRIPA
2	2	2	2	2	2	2	2	1	SINT GRIPA
1	1	1		1				1	CEFALEIA, I
1	1	2	1	1	1	2	2		
1	1	2	1	2	2	2	2	2	
1	1		1		1				
2	2	2	1	2	2	2	2	1	ASTENIA, C
1	1		1	1	1	2	2		
2	2	2	1	1	1	2	2	2	
1	1	9	1		1	2	2	1	TAQUICARI
1			1	1	1			1	PETEQUIAS
1	1	1	2	1	2	2	1	2	
	1	2	1	1	1	2	2	2	
1	1	1	1	1	1	2	2		
1	1	2	1	1	1	2	2	1	ASTENIA, H
			1	1	1				
1	1		1	1	1				
2	2	2	1	1	1	2	1	2	
2	2	2	1	2	1	2	2	2	
2	2	2	2	2	2	2	2	1	SINT GRIPA
1	1		1	1	1				
1	1	2	1	1	1	2	2	2	

1	1		1	1	1	1			
1	2	2	1	1	2	2	2	2	
1	1		1	1	1		1	1	TAQUICARI
1	1	1	1	1	1	2	1	1	SINTOMAS
2	2	2	1	2	1	2	2	1	CANSACO,
1	1	2	1	1	1	2	2	2	
			1	1	1				
2	1	2	1	1	1	2	2	1	CORIZA
1	1	1	2	2	1	2	2	2	
1	1	1	1	1	1	2	2	2	
1	1		1	1	1	2	2	2	
	1		1					1	CORIZA,MI
2	1	2	1	1	1	2	2		
1	1		1	1	1			1	INS. RESP.,
			1		1			1	PALPITACA
2	2	2	2	2	2	2	2	2	
1			1	1	1				
1	1	2	1	1	1	2	1	1	MIALGIA, C
1	1	1	1	1	1				
1	1		1		1			1	CEFALEIA, I
1	1	2	1	1	1	2	2	2	
1	1	1	1	1	2	2	1	1	MIALGIA
	1		1		1			1	CORIZA
2	1	2	1	1	1	1	2	2	
9	1	2	1	1	2	2	2	2	
1	1	1	1	1	2	2	2		
1	1		1	1	1				
1	1	2	2	2	2	2	2		
	1		1	1	1				
1	1	1	1	1		2	2		
2	1	2	1	2	1	2	2	2	
1			1	1			1	1	CONGESTA
1	1	1	1	1	1	2	2	2	
	1			1	1	1			
2	1	2	1	1	2	2	2		
1	1	2	1	1	1	2	2	1	ANOSMIA
1	1	2	1	1	1	2	2		
1	1		1						
1	1	2	1	1	1	2	2	1	CALAFRIOS
2	2	2	2	2	2	2	2	1	ASSINTOM
	1		1		1			1	CIANOSE P
1	1	1	2	1	1	2	2	2	
1	2	2	1	1	1	2	2	1	TAQUIPNE
2	2	2	2	2	2	2	2	1	SINT GRIPA
1	2	2	1	2	1	2	2	2	
2	2	2	2	2	1	2	2	1	DISURIA
2	1	2	2	2	1	2	2	2	

1		1	1	2	1	1	2	1	CEFALEIA
9	9	9	1	9	9	9	9	1	TAQUIPNE
1	1			1				1	ANOSMIA,
1	1		1	1	1	2	2	1	MIALGIA
1	1		1	1	1			1	DOR LOMB
1	1			1					
2	1	1	1	2	1	2	1	1	CORIZA, C
2	1	1	2	2	2	2	2	1	MIALGIA
2	1	1	1	1	1	2	2	2	
								2	
2	1	2	1	1	1	2	2	1	CEFALEIA,
1	1	1	1	1	1				
1	1	1	1		2	2	2	1	CEFALEIA
2	2	2	2	2	2	2	2	1	ASSINTOM
1	1	2	2	2	1	2	2	1	DOR LOMB
1	1	2	1	1	1	2	2		
1	1		1						
2	1	2	1	1	1	2	2	2	
	1		1						
9	1	9	1	1	9	9	9		
2	2	2	1	2	1	2	1	1	DOR TORA
2	1	2	1	2	2	2	2	1	CORIZA
1	2	2	1	1	1	1	2	2	
1	1	2	1	1	1	2	2	1	CEFALEIA
1	1	2	1	1	1	2	2		
1	1	2	1	1	1	2	2	1	DOR NOS
1	1		1	1	1				
1	1	2	1	2	2	2	2	1	TAQUICAR
2	1	1	1	1	1			1	APATIA
	1		1		1				
2	1	2	2	2	2	2	2	1	CORIZA
	1			1	1				
2	2	2	1	1	1	2	2		
	1		1		1			1	MIALGIA,
1	1	2	2	2	2	2	2	2	
1	1	2	1	1	1	2	2	2	
1	1	1	1	1	1	1	1	1	DOR ABDO
2	2	2	1	1	1	2	2	2	
2	1	2	1	1	1	2	2	2	
1	1	1	2	2	1	2	2		
	1		1						
1	1	2	2	1	2	2	1	2	
	1		1					1	CEFALEIA
1	1	2	1	1	1	2	2		
								1	ASSINTOM
2	1	2	1	2	2	2	2	1	SINT GRIPA
1	2	2	1	1	1	2	2	2	

2	1	2	2	1	1	2	2	2	
1	1	2	1	1	1	2	2	1	CANSACO,
1	1	1	1	1		9	9		
1	1	1			1			1	MIALGIA
1	1	2	1	1	1	2	2	2	
1	1	2	2	1	1	1	2	2	
2	1	2	2	1	1	2	2	1	CORIZA
	1	1	1	1	1	2	2	1	MIALGIA,C
			1	1	1				
2	2	2	1	1	1	2	2	2	
1	1		1	1				1	CEFALEIA, I
1	1	1	1	2	1	2	2	2	
1	1	2	1	1	1	1	1	2	
1			1					1	CEFALEIA
2	1	1	1	1	1	2	2	1	CEFALEIA,
1	1	2	1	2	1	2	2	2	
2	2	2	2	2	2	2	2	2	
1	1	2	1	1	1	2	2	2	
	1		1		1			1	NAUSEAS
			1	1	1				
1	1	9	1	9	9	9	9		
2	1	2	1	2	1	2	2	2	
1	1	1	1	1	1	2	2	2	
	1	1	1					1	DOR NO CC
2	2	2	1	1	1	2	2		
1	1	1	1	1	1				
1	1	1	1		1	1			
1	1	2	2	2	2	2	2	1	CORIZA, CE
1	1	2	1	1	1	2	2	1	MIALGIA
1	1	2	1	2	1	2	2	1	MIALGIA,C
			1		1				
2	1	2	1	1	1	2	2	2	
1	1	2	1	1	1	2	2	1	CEFALEIA
					1				
1	1	2	1	1	1	2	2	9	
1	2	2	2	1	1	2	1	1	ASTENIA, N
1	1		1		1				
1	1	2	1	1	1	2	1	1	MIALGIA
2	1	2	1	1	1	2	2	2	
2	2	2	2	2	2	2	2	1	CEFALEIA
2	1		1	1	1				
1	1		1					1	CONGESTA
1	1	1	1	1	1	1	2	2	
1	1	2	2	2	1	2	2	1	TAQUIDISP
	1	2	1	1	1	2	2	1	CEFALEIA, I
1	1			1					
1	1	2		1	1	2	2	2	

1	1	1	1	1	1				
1	1		1	1	1	2	2		
1	1	1	1	1	1	2	2	2	
2	1	2	1	1		2	2		
2	1	1	2	2	2	2	2	1	MIALGIA, C
1	1	2	1	1	2	2	2	2	
1	1	1	1	1	1	2	2	2	
1	1	9	1	1	1	9	9	9	
2	1	2	1	1	1	2	2	1	MIALGIA
2	2	2	1	2	1	2	2	1	MIALGIA.
1	1	2	1	1	1	2	2	1	MIALGIA E
2	1	2	1	2	2	2	2	2	
1	1		1						
1	2	2	2	1	1	2	2	2	
			1					1	ASTENIA
1	1	2	1	1	1	2	2	1	MIALGIA, D
1	1		1	2	1	2	2	2	
1	1	1	1	1	1	2	2	2	
2	2	2	2	2	2	2	1	2	
	1							1	CANSACO D
1	1	2	1	1	1	2	2	1	CANSACO,
1	1	2	1	1	1	2	2	1	MIALGIA
	1		1		1			1	MIALGIA
1	1	2	1	1	1	2	2	2	
	1		1		1		1	1	ASTENIA,M
1	1	1	1	1	1	2	2	1	CEFALEIA
1	1	1	1	1	1	2	2		
			1	1	1				
1	1	1	1	1	1	2	2	2	
2	1	2	1	2	2	2	2	1	CORIZA
	1		1		1				
1	1	2	9	1	2	2	2	1	DOR TORAC
1	1	2	2	1	2	2	2	2	
	1				1	1	1	1	CIANOSE/A
2	1	1	1	1	1	2	1	1	MIALGIA
2	1	2	1	1	1	2	2	1	DOR TORAX
1	1	2	1	2	1	2	2	2	
1	1	2	2	2	2	2	2	1	MIALGIA, C
1	1	1	1	1	1	1	1		
2	2	2	1	1	2	2	2	2	
1	2	2	2	2	2	2	2	2	
1	1		1	1	1		1	1	NAUSEAS E
1	1	2	1	1	1	2	2	2	
1	1	2	1	2	1	2	2	2	
2	1	1	1	1	1	2	2	2	
	1		1		1				
		1	1					1	DOR NO CO

2	1	2	1	1	1	1	2	1	ODINOFAG
	1		1						
1	1		1			1		1	MIALGIA
			1		1				
1	1	1	2	1	1	2	2	2	
1	1	1	1	1	1	2	2	2	
1	1	1	1	1	1	2	2	2	
1	2	2	2	2	2	2	2	1	DOR LOMB
2	1	2	1	1	2	1	1	2	
1	1	1	1	1	1	2	2	2	
1	1		1	1	1				
1	2	1	1	1	1	2	2		
			1		1				
2	2	2	2	2	2	2	2	2	
1	1	1	1	1	1	1	1		
1	1	1	1	1	2	2	2	2	
9	9	9	1	1	1	2	2	1	DOR TORA
1	1	2	1	1	2	2	2	1	CEFALEIA+
	1	1	1	1	1				
1	1							1	MAL ESTA
1	1								
2	1	2	2	2	2	2	2		
1	1	1		1	2	2	2	1	MIALGIA,
1	1	2	1	1	1	2	2	2	
1	1	1	1	1	1	1	1	2	
1	1	2	1	1	1	2	2	1	ASTENIA
	1				1			1	CEFALEIA,
1	1	1	2	2	2	2	2	1	CEFALEIA
2	1	2	1	1	2	2	2	1	CORIZA
1	1	2	1	1	1	2	2	1	ASTENIA E
1	1	1	1	1	1	9	9	1	MIALGIA,
1	1	2	1	2	1	2	2	1	MIALGIA,
1	2	2	1	1	1	2	2	1	MIALGIA/
2	1	1	1	1	1	2	2	2	
1	2	2	2	2	2	2	2	1	
1	1	1	1		1	1	9	1	
1	2	2	1	1	1	2	2	2	
1	1		1		1			1	CEFALEI/A
1	1	2	1	1	2	2	2	1	MIALGIA
1	1	2	1	2	2	2	2	2	
1	1	2	1	1	1	2	2		
1	1	2	1	1	1	2	2	2	
2	2	2	2	1	1	1	2	2	
1	1	2	2	1	2	2	2	1	CORIZA
1	1	1	2	1	1	2	2	1	MIALGIA,C
1	1	1	1	1	1	2			
1	1	2	1	2		2	2	1	CEFALEIA,

1	1	2	1	1	1	2	2	2	
1	1		1	1	2	2	2	1	CEFALEIA
1	1	1	1	1	1	2	1	1	CEFALEIA, I
1	1	2	1	1	1	2	2	1	ASTENIA, II
2	1	1	1	1	1	2	2	1	MIALGIA
1	1	2	1	1	1	2	2	1	CEFALEIA
2	2	2	1	1	1	2	2	1	MIALGIA
1	1	1	2	1	2	2	2	2	
1	1	1	1	1	1	2	2	1	MIALGIA
			1	1	1				
1	2	2	1	1	1	2	1		
1	1	1	1	1	1	2	1		
2	2	2	2	1	1	2	2		
	1	1		1	1			1	CEFALEIA, (
1	1	2	1	2	2	2	2	2	
2	1	2	1	1	1	2	2	2	
1	1	2	1	1	1	2	2		
2	2	2	2	2	2	2	2	1	PRE-ECLAM
1	1	2	1	1	2	1	1	2	
1	1	1	1	1	1	2	2	2	
			1		1			1	CEFALEIA, (
1	1		1	1					
2	1	2	1	1	2	2	2	2	
2	1	2	1	1	1	2	2	2	
1	1	2	2	2	1	2	2	2	
1	1		1	1					
1	1	2	1	2	2	2	2	1	DORSALGI/
1	1	1	1	1	1		1		
2	1	2	1	1	1	2	2	1	CEFALEIA, I
1	1		1						
1	1		1					1	TREMORES
1			1						
2	2	2	2	1	1	1	2		
1	1	1	1	1	1	1	2	2	
1		1	1	1	1				
2	2	2	2	1	2	2	2	2	
1			1	1	1				
	1			1	1			1	DOR TORA(
1	1	1	1	1	1	2	2	1	TAQUIPNEI
	1			1	1	2	2	1	SINTOMAS
1	1	2	1	1				1	CEFALEIA
2	2	2	1	2	1	2	2	2	
2	1	2	1	1	1	2	2	1	ASTENIA,IN
1	1	2	1	1	1	2	2	1	CEFALEIA
1	2	1	1	1	2	2	2	2	
1	1	1	1	2	1	1	2	2	
1	1		1	2	1				

	1		1		1			1	MIALGIA, E
1	1	2	1	1	1	2	2	2	
1	1	2	1	1	1	2	2	2	
1	1	1	1	1	1	2	2	1	CEFALEIA E
1	1	2	1	1	1	2	1	2	
1	1	1	1	1	1	2	2	2	
2	1	2	1	1	1	2	1	2	
	1		1	1	1				
1	1	2	1	1	1	2	2	2	
2	2	2	1	1	2	2	2	2	
2	2	2	1	1	2	2	2	1	MIALGIA, T
1	1	2	2	2	2	2	2	1	CEFALEIA;
1	1	2	1	1	1	2	2	2	
1	1			1					
				1	1			1	CORIZA E C
1	1	2	2	1	2	2	2	2	
2	1	2	1	2	2	2	2	1	DOR TORA
2	1	2	1	1	1	2	2	1	NAUSEA, C
1	1	2	1	1	1	2	2	2	
2	2	2	1	1	2	2	1	2	
1	1	2	1	1	1	1	1	2	
	1							1	DOR DE CA
1	2	2	1	2	1	2	2	2	
2	1	2	1	1	1	2	2	2	
1	1	1	1	1	2	2	2	9	
	1	1	1	1		1			
			1	1	1				
1	1		1	1	1			1	CEFALEIA, I
1	1	2	2	1	1	1	2	2	
1	1	2	1	1	1	2	2	2	
2	1	2	2	2	2		1	1	HEPATITE,
1	1	2	1	1	1	2	2	1	TONTURA/
1	1	2	1	1	2	2	2	1	CEFALEIA/I
1	2	2	1	1	1	2	2	2	
2	2	2	1	1	1	2	2	2	
2	2	2	2	2	2	2	2	1	CEFALEIA
2	2	2	1	1	1	2	2	1	GRAVIDA 3
2	1	2	1	2	2	2	2	1	CORIZA CO
2	2	1	1	1	1	2	2	1	CEFALEIA
2	1	2	1	1	2	2	2	2	
1	1	2	1	1	1	2	2	1	ASTENIA, M
1	1		1	1					
1	1	1	1	1	1	2	2		
1	1		1	1					
1	1	2	1	2	1	2	2	2	
1	1	2	2	2	2	2	1	2	
1	1	2	1	2	2	1	1	2	

1	1	2	1	1	1	2	2	2	
2	1	2	1	2	1	2	2	2	
1	1	2	9	1	1	2	2		
1	1	1	1	1	1	2	2	2	
1	1	2	1	1	1	1	1	2	
9	1	9	9	9	9	9	1	1	FALTA DE A
2	1	2	1	1	1	2	2	2	
1	1	2	1	1	1	2	1	2	
1	1	2	1	2	2	2	2	2	
1	2	2	2	1		2	2		
1	1		1					1	CEFALEIA
	1	1	1	1	1			1	FRAQUEZA
2	2	1	1	1	1	2	2	1	PRE CARDI
2	1	2	1	2	2	2	2	2	
	1		1		1			1	DOR TORA
1	1	2	1	1	1	2	2	2	
2	1	2	1	1	1	2	2	2	
	1		1		1				
2	1	2	1	1	1	2	2	2	
1	1	1	1	1	1	1	2	1	INSUF. RES
1	1	1	1	1	1				
2	1	2	1	1	2	2	2	1	PRECORDI
1	2		2						
1	2	2	1	2	1	2	2	1	DOR TORA
1	1		1						
2	1	2	1	1	1	2	2	2	
1	1	2	1	1	2	2	2	1	CEFALEIA
2	1	2	1	1	2	2	2	2	
2	1	2	2	2	2	2	2	1	CORIZA
1	1	2	1	1	1		2	1	MIALGIA
1	1		1	1	1			1	CORIZA
1			1					1	SENSACAO
1	1	2	1	1	1	1	2	2	
2	2	2	2	2	2	2	2	1	CRISE CON
2	1	2	1	1	1	2	2	2	
1	2	2	1	1	1	2	2	1	CORIZA
			1	1	1				
1	1		1	1	1	2	2	2	
1	1	2	1	1	1	2	2		
2	1	2	1	1	1	1	2	2	
1	1	1	1	1	1	2	2	1	CORIZA E N
1	1	2	1	1	2	2	2	1	CEFALEIA
2	2	2	1	1	1	2	2	2	
1			1				1		
1	1	2	1	1	1	2	2		
1	1		1	1	1			1	PROSTRACA

2	2	2	1	1	1	2	2	2	
2	1	2	1	1	1	2	2	2	
1	1	2	2	1	2	2	2	2	
1	1	2	1	1	1	2	2	2	
2	1	1	2	2	2	2	2		
			1	1		1			
1	1	2	1	1	1	2	2	1	DOR TORA
2	1	2	2	2	2	2	2	1	CORIZA OB
1	1	2	1	2	2	2	2	2	
2	2	2	1	1	1	2	2	2	
2	1	1	1	1	2	1	2	1	CORIZA, M
1	1				1				
2	2	2	1	1	1	2	2	2	
1	1	1	1	1	1	1			
	1	1		1	1				
2	1	2	1	1	2	2	2	2	
2	1	2	1	1	1	2	2		
2	1	2	1	1	1	2	2		
1	1	1	1	1	1				
					1				
								1	HIPOXEMIA
1	1	1	1	1	1	2	2	1	MIALGIA
	1	1	1	1	1	1		1	CEFALEIA
1	1	2	1	1	1	2	2	1	ASTENIA, A
1	1	2	1	1	1	2	2	2	
1	1		1	1					
2	2	2	1	2	1	2	2	9	
1	1	2	1	1	1	2	2	1	PCT EM (T
1	1	2	1	2		2	2	1	TAQUIDISP
2	1	2	1	1	2	2	2	2	
2	1	1	1	2	1	2	2	1	CEFALEIA
1	1		1	1	1				
2	2	2	2	2	2	2	2	1	SINT GRIPA
1	1	1	1	1	1	2	2	2	
2	1	2	1	1	2	2	2	2	
1	1	2	1	1	1	1	2	1	MIALGIA,
2	1	2	1	2	1	2	2	2	
2	1	2	1	1	1	2	1	1	CORIZA
1	2	2	1	1	1	2	2		
			1	1	1				
			1					1	CEFALEIA,
2	1	2	1	2	2	2	2	1	SINT GRIPA
2	1	2	1	1	1	2	2	1	DOR TORA
2	1	2	1	2	1	2	2	2	
1	2	2	1	1	1	2	2	1	MIALGIA
1	1		1	1					
1	1	9	1	9	1	9	9	9	

1			1	1					
1	1	2	1	1	1	2	2	9	
2	2	2	1	1	2	2	2	2	
1	1	1	1	1	1	2	1	2	
	1				1				
1	1	2	1	1	1	2	1	2	
1	1	2	1	2	2	2	2	1	DIARREIA;
1	2		1		1	2	2	1	OBITO FET
2	1	2	1	1	1	2	1	2	
2	2	2	1	2	1	2	2	2	
1	1	2	2	2	1		2	2	
	1		1	1	1				
1				1	1			1	HAS
1	1		1	1				1	MIALGIA,
1	1		1						
1	1		1						
1	1	2	1	1	1	1	2	2	
			1	1	1			1	CEFALEIA
1	1	1	1	1	1	2	1		
1	1	1	1	1	1	2	2	1	NAUSEAS
2	1	2	2	2	2	2	2	2	
1	2	2	1	1	1	1	2	1	SONOLENC
2	2	2	1	2	1	2	2	2	
1	1	1	1	1	1	2	2		
2	2	2	1	2	2	2	2	1	SINT. GRIP
			1	1	1				
1	1	2	1	1	1	2	2	1	CEFALEIA,
1	1		1	1	1			1	CORIZA, M
1	1	2	2	1	1	2	2		
2	1	2	1	2	2	2	1	2	
1	1		1						
	1				1				
9	1	1	1	1	1	9	9		
1	1		1	2	2	1	2	2	
	1	1	2	1	1		1		
1	1	2	1	1	2	2	2	9	
1	1	2	1	2	1	2	2	2	
2	1	2	1	2	2	2	2	1	ASTENIA ,
1	1	2	1	2	2	2	2	1	MIALGIA+
1	1	2	1	1	1	2	1	2	
	1	2	1	1	1	2	2	1	CEFALEIA,
2	2	1	1	1	1	2	1	1	MIALGIA
	1		1				1	1	MIALGIA
1	1	2	1	1	1	2	2	1	ASTENIA,
1	2	2	1	1	1	2	2		
1	1		1		1			1	CEFALEIA

	1		1		1				
1	1	1	1	1	1	1	2	2	
2	1	2	1	1	1	2	2	1	INAPETENC
2	1	2	1	2	2	2	2	1	TAQUICARI
2	2	2	1	2	1	2	2	1	CORIZA
1	1	2	2	1	1	2	2	1	ASTENIA ,D
9	9	9	9	9	9	9	9	9	
1	1	1	1	1	1	2	2	1	CORIZA, DO
2	2	1	1	1	1	1	2	1	MIALGIA,C
1	1		1					2	
1	1	2	1	2	1	2	2	1	MIALGIA, C
1	1	2	1	2	2	2	2	2	
1	1	1	1	1	1		1	1	CANSACO
1	1	2	1	1	1	2	2	1	DOR NO CO
2	1	2	1	2	1	2	2	1	CEFALEIA,M
2	2	2	1	1	1	2	2	2	
2	1	2	1	1	1	2	2	2	
1	1		1		1				
1	2	2	1	1	1	2	2	2	
2	2	2	1	1	1	2	2	1	CORIZA, M
1	1	2	1	1	1	2	2	1	SINTOMAS
2	1	2	1	2	1	2	2	1	ASTENIA
1	1	2	1	1	1	2	2	2	
2	1	2	1	2	2	2	2	1	SINT GRIPA
1			1	1	1				
2	1	2	1	2	2	2	1	1	MIALGIA
1	2	2	1	1	1	2	1	2	
	1		1				1		
1	1	1	1	1	1	2	2	1	INAPETENC
2	2	2	1	1	1	2	2	1	MIALGIA, N
1	1				1			1	CALAFRIOS
1	1	2	1	1	1	2	2	1	MIALGIA, C
2	1	2	1	2	2	2	2	1	MIALGIA. A
2	1	2	1	2	2	2	2	2	
1	1	2	1	1	1	2	2	2	
2	1	2	1	1	1	2	2	1	MIALGIA
	1		1	1	1				
1	1	2	2	1	2	2	2	1	ACIDOSE M
2	2	2	1	2	1	2	2	1	MIALGIA
1	2	2	2	2	2	2	2	2	
2	1	2	1	1	1	2	2	2	
2	1	2	1	2	1	2	2	2	
1	1	2	1	1	1	2	1	2	
2	1	2	1	2	1	2	2	2	
9	2	2	1	2	1	2	1	1	CEFALEIA
1	1	9	9	9	9	9	9	1	ODILOFAGI
1	2	2	1	1	1	2	2	1	DOR NO PE

1	1								
1	1	2	1	1	1	2	2	1	CEFALEIA,
2	1	1	1	2	1	2	2	2	
2	1	2	1	2	2	2	2	1	ANOSMIA
	1		1	1	1				
1	1				1	1		1	CEFALEIA
1	1	2	1	1	1	2	2	1	INAPETEN
1	1	1	1	1	2	2	2	1	DOR NO C
2	1	2	1	2	1	2	2		
1	1		1		1			1	MIALGIA,
1	1		1						
1	1	2	1	1	2	2	2	1	CORIZA
1	1	1	1						
1	1	2	1	2	2	2		1	MIALGIA
1	1	2	1	2	1	2	2	1	CORIZA
1	1	1	1	1	1	1	2	2	
1	1		1		1				
1	1		1	1	1				
1	1	2	1	1	1	2	1	2	
1	2	2	1	2	1	2	2	1	CEFALEIA,
1	1	2	1	1	1	2	2	1	CORIZA, A
1	1	1	1	1	1	2	2	2	
1	1		1	1				1	CEFALEIA,
1	1	2	1	1	1	2	2	1	CORIZA, C
2	1	2	2	2	2	2	2	1	DOR TORA
					1				
2	1	2	1	2	2	1	2	1	CORIZA
			1		1				
1	1	2	1	1	1	2	2	2	
	1		1					1	ASTENIA
	1		1	1	1			1	ADNAMIA
1	1	2	1	2	1	1	2	1	MIALGIA
2	2	2	1	1	1	2	1	1	MIALGIA,
			1	1	1				
	1	1	1	1					
1	2	2	2	1	2	2	2	2	
1	1	2	1	1	1	2	2	1	CEFALEIA
	1	1	1		1				
1	1	2	1	1	2	2	2		
2	1	2	1	1	1	2	2	2	
9	1		1	1	1	9	9	9	
2	1	2	2	2	2	2	2	2	
			1	1	1				
1	1	2	1	1	1	2	2	2	
1	1	2	1	1	1	2	2		
1	1	1	1	1	1	2	1	2	
2	2	2	1	1	1	2	1	2	

2	1	2	1	2	1	2	2	1	ASTENIA, C
1	1		1						
2	1	2	1	1	2	2	2	2	
			1	1	1				
2	1	2	2	1	1	2	2	2	
2	1	2	1	1	1	2	2	2	
2	1	2	1	1	1	2	2	1	CEFALEIA, (
2	2	2	1	1	1	2	2	2	
			1	1	1				
	1		1					1	CEFALEIA
			1		1			1	MIALGIA/C
2	1	2	1	1	1	2	2	1	INAPETENC
1	1		1	1	1			1	MIALGIA A
1	1				1				
	1	1	1	1	1				
1	1	2	1		1	2	2	1	TAQUIDISP
1	1	1	1	1	1				
2	1	2	2	2	2	2	2	2	
2	2	1	2	2	2	2	2	1	CORIZA
2	2	2	2	2	2	2	2	1	CORIZA
1	1		1						
	1		1	1					
1	1	2	1	1	1	2	2	1	TAQUICAR
1	2	2	1	1	2	2	2	1	INAPEDENC
1	1	2	1	1	1	2	2	2	
1	1		1					1	CORIZA, CC
1	1	2	1	1	1	2	2	1	CEFALEIA A
			1		1				
2	1	2	1	1	1	2	2	1	MIALGIAAS
2	1	2	1	1	2	2	2	1	GESTACAO
			1	1	1				
1	1	2	2	2	2	2	2	2	
1	1		1	1					
1	1		1		1			1	CEFALEIA, A
1	1	2	2	1	1	1	1	1	CEFALEIA
			1	1	1				
9	1	9	1	1	1	9	9	1	MIALGIA, S
1	1	2	1	2	2	2	2	2	
1	1	2	2	2	1	2	2	1	MIALGIA,C
9	1	2	1	9		2	9		
	1		1	1	1			1	TAQUICARI
1	1	1	2	1	1	2	2	2	
1	1		1	1	1				
1	2	2	1	2	1	2	1	1	MIALGIA, N
1	1			1	1				
1	2	2	2	2	2	2	2	2	

1	1	2	1	2	1	2	2	2	
1	1		1					1	CEFALEIA
1	1	2	1	2	2	2	1	2	
1	1	2	1	1	1	1	2	2	
2	1	2	2	1	2	2	2	1	CEFALEIA, I
1	1	1	1	1	1			1	MIALGIA
1	2	2	1	1	1	2	2	2	
2	2	2	1	2	1	2	2	1	SINTOMAS
1	1	2	1	1		2	2	1	CEFALEIA, (
1	1	2	1	2	1	2	2	1	CEFALEIA/I
2	2	2	1	1	1	2	2	1	MIALGIA
1	1		1	1	1				
1	1	2	1	1	2	1	2	1	DOR TORA(
2	1	1	1	1	1	2	2	1	INSUF.RESI
1	1	1	1	1	1	2	2	2	
2	1	2	1	1	1	2	2	1	MAL ESTAF
2	1	2	1	1	2	2	2	2	
1	1	2	1	1	1	2	2	1	DOR NO CC
2	1	1	1	1	1	2	2		
1	1	2	1	1	1	1	1	9	
1	1	1	1	1	1	1	2	2	
	1			1				1	ODINOFAG
1	1	2	1	1	1	2	2	1	CANSACO
1	2	1	1	2	2	1	2	1	MIALGIA
1			1						
1	1		1	1	1	1	1	2	
1			1		1			1	DOR TORA(
1	1		1	1				1	DOR DE CA
2	1	2	1	2	2	1	2	2	
1						1		1	CEFALEIA, /
1	1	2	1	1	1	2	2	2	
			1	1					
9	9	9	9	9	9	9	9	9	
2	2	2	1	2	1	2	2	1	DISURIA.
2	1	2	2	2	2	2	2	1	MIALGIA.
2	1		1	1	1	2	1		
1	1	1	1	1	1	9	1	1	CORIZA;
2	1	1	2	2	2	2	2	1	MIALGIA CI
2	2	2	2	2	2	2	2	2	
1	1			1	1			1	PROSTRAC
1	1	2	2	1	1	2	2		
1	1	1	1	1	2	1	1	2	
2	2	2	1	1	1	2	2	2	
1	1	2	1	2	2	2	2	1	CALAFRIO
1	1	2	2	1	1	1	2	2	
1	1	2	1	1	1	2	2	2	
1	1	1	2	2	2	2	2	1	CEFALEIA

1	9	9	9	9	9	9	9	1	CEPLEIA, M
2	1	2	1	1	1	2	2	1	MIALGIA
1	1	2	2	2	1	2	2		
2	1	1	2	1	2	2	2	2	
	1				1			1	CEFALEIA, I
1	2	1	1	1	2	2	2	1	MIALGIA E
2	1	2	2	1	1	2	2	2	
1	1		1	1	1				
1	2	2	1	1	1				
1	2	2	1	1	1			1	NAUSEAS+
2	1	1	1	1	1	2	2	1	CORIZA
1	1		1						
2	1	1	1	2	1	2	2	2	
1	1	1	1	1	1	2	2	9	
1	1	2	1	1	1	2	2	1	CEFALEIA
2	2	2	1	2	2	2	1	2	
2	2	2	1	1	2	2	2	2	
1	2	1	1	2	1	1	2	1	MIALGIA
2	1		1	2	1	2	2		
2	2	2	1	1	1	2	2		
1	1	2	1		9	2	2	1	ODINOFAG
	1		1	1	1				
	1		1		1	1			
2	1	2	1	2	2	2	2	2	
1	1		1		1				
1	1	2	1	2	1	2	2		
			1					1	DOR TORA(
1	1	1	1	1	1				
1	1	2	1	1	1	2	1	2	
2	1	2	2	1	1	2	1	1	CONGESTA
2	1	2	2	2	2	2	2	2	
2	1	2	1	1	1	2	2	2	
1	1	9	1	1	1	2	2	1	CORIZA, TA
1	1	2	1	1	1	2	2	9	
1	1	2	1	1	1	2	2		
1	1		1					1	MIALGIA
1	2	2	1	1	1	2	2	1	MIALGIA
2	1	1	1	1	2	2	2	2	
1	1	1	1	1					
1	1		1		1				
2	2	2	1	1	1	2	2	2	
1	1	2	1	1	1	2	2		
2	1	2	1	2	1	2	2		
1	2	1	1	2	1	2	2	2	
2	1	2	1	2	1	2	2	2	
1	1							1	CONGESTA

1	1	2	1	2	2	2	2	2	
1	1			1	1				
2	1	2	1	1	1	2	2	2	
1	1			1	1				
1	1		1	1	1				
1	1		1	1	1			1	CEFALEIA, I
	1		1	1		2	2	1	ANOREXIA
1	1		1	1	1				
1	1	2	1	1	1	2	2	1	MIALGIA, C
1	1	2	1	1	2	2	2	2	
1	1	1	1	1	1	1	2	2	
1	1	1	1	1	1	2	2		
1	1	2	1	1	1	2	2	9	
1	1	2	1	2	2	2	2	2	
1	2	2	1	1	1	2	2	1	ASTENIA, H
1	1						1		
1	1		1	1	1				
	1		1						
1	1		1						
2	1	2	1	1	2	2	2	1	CALAFRIOS
1	1	1	2	1	1	2	2	1	CEFALEIA,C
1	1	1	1	1	1	2	2	2	
1	1		1	1	1				
1	1	2	1	1	1	2	1	1	MIALGIA
1	2	2	1	2	2	1	2	2	
2	2	2	1	1	1	2	2	1	CEFALEIA
			1		1			1	DOR TORAC
1	2	2	1	1	1	2	2	2	
2	1	2	1	1	1	2	2	1	ASTENIA
1	1		1	9	1	2	2	1	HIPOXEMIA
2	2	2	1	1	1	2	2	2	
1	1	1	1	1	1	2	2	2	
1	1	1	1	1	1	1	1	2	
2	1	1	1	1	1	2	2	2	
2	2	2	1	1	1	2	2	2	
1	1		1	1	1			1	CEFALEIA N
1	1		1	1	1	1	1		
1	1	2	1	1	2	2	2	2	
1	1	2	1	1	1	2	2	1	CELAFEIA
1	1	2	1	2	1	1	2	1	CANSACO
2	1	2	1	1	1	2	2	1	CEFALEIA
2	1	2	2	1	1	2	2	9	
1	1		1	1	1			1	ADINANIA
	1		1	1					
1	2	2	2	1	1	2	2	2	
2	1	2	1	1	1	2	2	2	

1	1	2	1	1	1	2	2	2	
1	1	2	1	2	2	2	2	2	
1	1	2	1	2	1	2	2	1	
2	2	2	1	1	1	2	2	2	
1	1	1	1	1	1	2	2	1	MIALGIA E
1	1	2	1	1	1	2	2	9	
	2	1	2	2	2	2	2	2	
	1		1	1					
1	1	1	2	2	2	2	2	2	
1	1	1	1		1	1	2		
2	1	2	1	1	1	2	2	1	MIALGIA
1	1	1	1		1				
1	1		1					1	CEFALEIA
2	1	2	2	2	2	2	2	2	
1		1	1					1	CEFALEIA,
2	1	2	1	1	1	2	2	2	
		1		1	1			1	MIALGIA, C
1	1	2	1	2	1	2	2	1	MIALGIA,S
1	1	2	1	1	2	2	2	2	
	1	1						2	
2	2	1	1	2	1	2	2	2	
1	1		1		2			1	CEFALEIA
	1				1			1	CALAFRIOS
2	1	2	1	1	1	2	2	2	
2	1	2	1	1	1	2	2	2	
1	1	2	1	1	1	2	2	2	
1	1	2	2	1	1	1	1	1	INAPETEN
2	1	2	2	2	1	1	1	1	CORIZA, O
	1		1	1		1	1	1	DOR NO C
1	1	1	1	1	1	2	2	1	CEFALEIA,
1	1	2	1	1	1	2	2	2	
1	1	2	1	1	1	1	1	2	
1	1	2	1	2	1	2	2	1	MIALGIA,F
1	1			1	1				
				1	1				
1	1	1	1	2	1	2	2	2	
1	1	1	1	1	1	1	2	2	
	1	1	1		1			1	CEFALEIA E
1	1		1	1	1			1	CORIZA, C
2	1	2	1	1	1	2	2	1	MIALGIA,
1	1	2	1	1	1	2	2	1	CANSACO,
1	1	2	1	2	1	2	2	1	MIALGIA
2	1	2	1	1	1	2	2	2	
1	1	1	1						
1	1	2	1	1	1	2	2		
1	1	1	1	1	1	2	9	2	
1	2	2	1	1	1	2		1	CEFALEIA

1	1	1	1	1	1	2	2	1	CORIZA CE
1	1	2	1			2	2		
2	1	2	1	1	2	2	2	1	CEFALEIA,
1	1	2	1	1	1	2	1	1	CEFALEIA ,
			1	1	1			1	MIALGIA
1	1	2	1	1	1	2	2	1	CEFALEIA E
2	2	2	1	2	2	2	2	1	MIALGIA
1	2	2	1	1	1	2	2	2	
1	1	2	1	1	1	2	2	1	MIALGIA,
2	1	1	1	1	2	1	1	2	
1	1	2	1	1	1	2	2	1	MIALGIA,
	1		1		1			1	CEFALEIA,
1	1	2	1	2	2	2	2	1	ALGIA
2	2	2	2	1	2	1	2	1	OBSTRUCA
2	1	2	1	1	2	2	2	2	
1	1	2	2	1	1	2	2	1	GESTANTE
1	1	2	1	2	2	2	2	1	MIALGIA
1	1	1	1	1	1				
1	2	2	2	1	1	2	2	1	CONFUSA
2	1	2	1	1	1	2	2	1	CORIZA, C
1	1		1	1	1				
1	1	2	1	1	1	2	2	2	
1	1	1	1	1	1	2	2	1	CORIZA, M
1	1				1				
2	1	1	1	1	1	2	2	1	CEFALEIA,
1	1	1	1	1	1	2	1	2	
2	1	2	1	1	1	2	2	2	
			1	1	1				
1	1		1		1				
1	1		1		1			1	MIALGIA
2	1	2	1	2	2	2	2	9	
	1		1	1	1			1	PROSTRAC
2	1	2	1	1	1	2	2	1	CEFALEIA,
1	1	2	1	2	2	2	2	1	CEFALEIA
1			1		1				
1	1	2	1	1	1	2	2	1	MIALGIA
1	1	2	2	2	2	2	2	2	
2	1	2	1	1	1	1	2		
	1		1		1				
2	1	2	1	2	1	2	2	2	
2	1	2	1	1	1	9			
1	1	2	1	2	2	2	2	1	CANSACO
2	9	2	1	1	9	2	2	2	
2	1	2	1	2	1	2	1	2	
1	2	2	1	2	1	2	2	2	
1	1	2	1	1	1	2	2	2	
1	1		1	1	1				

1	1	2	1	2	1	2	2	1	NAUSEA E
1	1	1	1	2	1	1	2	2	
2	1	2	1	1	1	2	2	2	
1	1	2	1	1	1	2	2	1	CEFALEIA
1			1	1	1				
2	1	2	1	1	2	2	2	1	DOR LOMB
2	2	2	1	1	2	2	2	2	
2	1	2	1	1		2	2	2	
1	1	1	1	1	1	2	2	1	CEFALEIA
2	1	2	1	1	1	2	2	1	DOR TORA(
1	1	1	1	1	1				
1	1	2	1	2	1	1	2	1	CEFALEIA E
2	1	2	1	1	1	2	2	2	
1	1	9	1	1	1	9	9	9	
1	1		1		1				
1	1	2	2	1	1	1	2	1	MIALGIA
1	1	9	1	1	9	9	9	1	CEFALEIA, I
1	1	1						2	
			1		1			1	MIALGIA
1	1							1	CORIZA E A
2	1		1	1	1	2	2	2	
1	1	2				1			
1	1	2	1	1	2	2	2	1	MIALGIA
1	1	2	2	2	2	2	2	1	MIALGIA/C
1	1	2	1	1	1	2	1	1	CORIZA, CE
2	1	2	1	1	1	2	2		
1	1	2	2	2	2	2	2	1	SINT GRIPA
			1					1	INAPETENC
2	1	2	1	1	1	2	2	1	LOMBALGI
1	1				1			1	ASTENIA
1	1	2	1	1	1	2	2	1	CEFALEIA, (
2	1	2	1	1	1	2	2	2	
1	1	1	1	2	1	2	2	1	CEFALEIA
2	1	1	1	2	1	2	2	1	CEFALEIA
			1						
2	1	2	1	1	1	2	2		
	1		1	1					
	1		1		1			1	CALAFRIOS
1	1	2	1	1	1	2	2	2	
1	1	2	1	1	2	2	1	1	CEFALEIA
1	1	2	1	1	1	2	2	2	
2	1	1	1	1	1	1	2	2	
2	1	2	1	1	2	2	2	2	
	1		1						
1	1	2	1	1	2	2	2	2	
1	1	1	1	1	1	2	2	2	
	1		1						

1	1							1	CORIZA
2	1	2	2	2	2	1	2	1	CEFALEIA /
1	1		1		1			1	MIALGIA
1	1		1						
1	1	1	2	2	2	2	2	2	
1	1		1					1	CEFALEIA,
1	1	1	1	1	2	2	1	1	CALAFRIOS
2	2	2	2	2	2	2	2		
1	1	2	1	1	1	1	2	2	
1	1	2	1	1	1	2	2		
1	1	1	1	1	1	2	2	1	CEFALEIA,
1	1	2	1	1	1	2	1	1	MIALGIA
1	1	2	2	1	2	2	2	2	
2	1	2	1	2	2	2	2	2	
	1							1	ASTENIA
1	1	1	1	2	1	2	2	1	MIALGIA
1	1	2	1	1	1	2	2	2	
	1		1	1					
1	1			1	1				
	1		1					1	FRAQUEZA
1	1		1		1			1	CORIZA,NA
2	1	2	1	1	1	2	9	1	DOR EM HI
			1	1	1				
					1				
1	1	1	1	2	2	2	2	2	
1	1	2	2	1	2	2	2	2	
2	1	2	1	2	2	2	2		
2	1	2	1	1	1	2	2	1	CORIZA, CE
1	1		1		1			1	CEFALEIA, I
	1		1						
	1		1		1				
1	1	1	1	1	1	1			
1	1	2	1	1	1	2	2	2	
2	1	2	1	2	1	2	2	2	
1	1	2	1	1	1	9	2		
1	2	2	1	1	1	2	2	2	
1	1	2	1	1	1	2	2	2	
2	1	2	1	1	1	2	2	1	ASTENIA
1	2	2	1	1	1	2	2	2	
1	2	2	1	1	2	2	2	1	ADINAMIA
1	1	2	1	1	1	2	2	1	MIALGIA,
1	1		1					1	MIALGIA C
1	1	1	1	1	1				
1								1	CEFALEIA/
1					1				
2	2	2	1	1	1	2	2	2	
1	1	2	1	1	1	2	2	2	

1	1		1	1	1				
1	1	2	1	2	2	2	2	2	
1	1		1					1	CALAFRIO,I
2	1	2	1	2	1	2	2	1	CORIZA
2	1	1	1	1	2	2	2	2	
2	1	2	1	1	1	2	2	2	
1	2	2	2	2	1	2	2		
1		1	1	1	1				
2	2	2	1	1	1	2	2	1	MIALGIA
			1					1	CEFALEIA, I
2	1	2	1	1	1	2	2	2	
	1		1		1				
2	1	2	1	1	2	2	2	1	CEFALEIA
	1	1	1						
1	1		1	1	1			1	ASTENIA, T
1	1	2	2	1	2	1	2	1	CONGESTA
9	9	9	1	9	1	9	9	9	
								1	FALTA DE A
1	1	2	1	1	1	2	2	2	
1	1	2	1	1	1	2	2	2	
1	1	1	1	1		2	2		
2	1	2	1	1	1	2	2	2	
1	1		1	1	1			1	MIASTENIA
2	2	2	1	2	1	2	2	2	
1	1	2	1	1	1	2	2	1	ASTENIA
	1		1						
2	2	2	2	2	2	2	2	1	DOR EPIGA
1			1		1				
			1	1	1			2	
1	1		1					1	CORIZA
1	1	9	9	1	9	9	9	1	MIALGIA
1	1	1	1	1	1	2	2	1	MIALGIA
1				1	1	1		1	VOMITO
2	2	1	1	1	1	2	2		
1	1	2	1	2	1	2	2	1	DOR LOMB
1	1		1	1		1		1	CEFALEIA, I
2	2	2	1	1	1	2	2	2	
1	1	1	1	1	1	2	2	1	DOR EM BV
1	1	1	1	1	1	2	2	2	
2	1	2	1	1	1	2	2	1	MIALGIA
1	1	2		2	2	2	2	1	MIALGIA
1	1	1	1	1			1	1	INAPETENC
2	1	2	1	2	1	1	1	1	MIALGIA , /
1	1		1		1				
1	1	2	9	1	1	2	2		
1			1			1		1	MIALGIA
1	1	2	1	1	2	2	2	1	ADINAMIA,

2	2	2	1	1	1	2	2	2	
1	1	2	1	1	1	2	2	1	DOR TORA
	1	1	1		1			1	CEFALEIA
1	1	2	1	2	2	2	2	2	
2	1	2	1	1	1	2	2	1	MIALGIA
1	1	2	2	1	2	1	2	2	
2	1	2	1	1	1	2	2	2	
1	1			1	1	2	2	1	CEFALEIA
			1	1	1				
2	1	2	1	2	1	2	2	1	TAQUIPNE
2	1	2	1	1	1	2	2	1	MAL ESTAR
2	1	2	1	1	1	2	2		
1	1	2	1	2	2	2	2	1	CORIZA
1	1	1	1	1	1	2	2	1	CEFALEIA,
1	1		1	1				1	MIALGIA, T
2	1	2	1	1	2	2	2	2	
				1				1	GRIPE
2	1	2	2	2	2	2	2	1	CEFALEIA,
1	1	2	1	2	1	2	2	2	
1	1	2	1	1	1	2	2	2	
1	1	2	2	1	1	1	2		
1			1	1	1				
1	1							1	MIALGIA
1	1		1	2	2	2	2	1	CORIZA, O
2	1	2	1	1	1	2	2	1	CEFALEIA/
2	1	2	1	1	2	2	2	1	
1	1	1	1			1		1	MIALGIA, C
1	1	2	1	1	1	2	2	1	CEFALEIA
1	1	1	1	1	1	2	2	2	
1	1	2	2	2	2	2	2	1	
2	1	1	2	2	2	2	2	1	CORIZA, M
1	1	2	1	1	1	2	2	2	
1	1	2	1	1	1	1	2	1	CEFALEIA,
1	1	2	1	1	1	2	1	1	CEFALEIA
2	1	1	2	2	2	2	2	1	CORIZA
2	1	2	1	1	1	2	1	2	
1	1	1	1	1	1	2	2	2	
1			1				1	1	NAUSEA
2	2	2	1	2	1	2	2	1	CRISE CON
1	1	1	2	2	2	2	2	1	CALAFRIO
1	1	2	1	1	1	2	2	2	
1	1		1	1	1				
1	1		1						
1	1			1	1				
1	1	1	1	1					
1	1	2	1	2	1	2	2	2	
	1		1					1	MIALGIA,

2	1	1	1	1	1	2	2	2	
1	1		1	1	1			1	CORIZA
2	1	2	1	1	1	2	2	2	
2	1	2	1	2	1	2	1	1	ADINAMIA
1	1	2	1	2	2	2	1	1	SINT GRIPA
2	1	2	2	2	2	2	2	1	CORIZA,DC
1	1	9	1	1	1	9	9		
2	1	2	1	1	1	2	2	2	
1	1	2	1	1	1	2	2	1	MIALGIA
1	1	2	1	1	1	2	2	2	
2	1	2	1	1	1	2	1	1	CORIZA, M
2	1	2	1	1	1	1	1	2	
2	2	2	1	1	1	2	2	2	
1	2	2	2	1	2	2	2	2	
1	1	1	1	1	1	2	2	2	
1	2	2	1	1	1	2	2	2	
1	1	1	1	1	1			1	MIALGIA
2	2	2	1	1	1	2	2	1	TAQUICAR
1	1		1	1	1				
		1	1		1			1	MIALGIA
1	1	1	2	1	2	1	1	2	
2	2	2	1	1	1	2	2	2	
2	1	2	1	1	1	1	1	2	
2	2	2	1	1	1	2	2	1	SINTOMAS
1	1	1	1	2	1	2	2	1	RINORREIA
2	1	2	1	1	1	2	2	1	MIALGIA E
1	1		1	1				1	CEFALEIA
1	1	1	2	1	2	1	2	2	
			1		1				
			1	1	1				
2	1	2	2	2	1	2	2	2	
1	2	2	2	1	2	2	2	1	CEFALEIA
	1		1	1	1				
2	1		1	1	1	2	2		
1		1	1	1					
1	1	2	1	1	1	2	2		
1	1	2	2	2	2	2	2	1	MIALGIA
1		9	1	1	1	1			
	1		1					1	CORISA, OS
1	1	2	1	2	2	2	2	2	
2	1	2	1	1	1	2	2	1	CEFALEIA
1	1	2	1	1	1	2	2	2	
1	2	2	2	2	1	1	2	2	
1	1		1	1	1				
			1		1				
1	1	2	1	2	1	2	2	2	
	1		1	1	1			1	CANSACO,

	1								
1	1	1	1	1	1	2	2	1	FRAQUEZA
1	1	2	1	1	1	2	2	1	MIALGIA, T
1	1	2	1	1	1	1	1	2	
1	2	2	1	1	1	2	2	1	DOR TORA
1	1	2	1	2	2	2	2	2	
1	1	1	1	1	2	2	1	1	GESTANTE
1	1		1		1				
1	1	2	1	1	1	1	2	1	DOR NAS C
1	1	2	2	2	2	2	2	1	CEFALEIA, I
1	1	2	1	1	1	2	1	1	NAUSEAS
2	2	2	1		1	2	2	1	CEFALEIA E
2	1	2	1		2	2	2	2	
1	1	2	1	2	1	2	2	2	
1	2	2	1	1	1	2	2	2	
2	1	2	1	1	1	2	2	2	
1	1	1	1	2	1	2	2	2	
2	1	2	1	2	1	2	2	2	
1	1	1	1		1	1	2		
				1					
2	1	2	1	1	1	2	2		
1	1	2	1	1	1	2	2	2	
2	1	2	1	1	1	2	2	2	
2	2	2	1	2	2	2	2	2	
1	1	1	1	2	2	2	2	2	
1	2	2	2	1	2	2	2	1	SINT GRIPA
1	1	2	1	1	1	2	2	1	DOR LOMB
				1					
	1		1	1	1			1	ANOSMIA I
1	1	1	1	1					
9	9	9	9	9	9	9	9		
	1			1					
1	1	2	1	1	1	2	2	1	INDISPOSI
		1	1	1	1				
			1	1	1				
2	2	2	2	2	2	2	2	1	MIALGIA, F
1	2	2	1	1	1	1	1	1	CEFALEIA
2	2	2	2	2	2	2	2	1	CORIZA MI
1	1	1	1	1	2	1	1	2	
1	1	2	1	1	1	1	2	2	
1	1	2	1	1	1	2	2	1	DOR TORA
1	2	2	1	1	1	2	2	1	MIALGIA, C
1	1	1	1	1	1	2	2		
2	1	2	1	2	1	2	2	2	
2	1	2	1	1	2	2	2	2	

1	1							1	MIALGIA E
2	2	2	2	2	1	2	2	1	CEFALEIA, I
1	1	2	2	2	2	2	2	2	
2	1	2	1	1	2	2	2	1	MIALGIA N
2	1	2	1	1	2	1	2		
2	1	2	1	1	1	2	2	2	
2	2	2	1	2	2	2	2	2	
1	2	2	2	1	2	2	2	2	
2	1	2	1	1	1	2	2		
2	1	2	2	2	1	2	2	1	MIALGIA, C
1	1			1				1	CANSACO
1	1	2	2	2	1	2	2	2	
2	1	2	1	2	1	2	2	1	ALGIA TOR
1	9	9	9	9	9	9	1	1	CEFALEIA N
2	1	2	1	1	1	2	2	2	
			1	1	1				
1	1	1	1	1	1	1	2	2	
2	1	2	1	1	1	2	2	2	
2	1	1	1	1	1	2	2		
1	1		1		1		1	1	DOR TORA
			1		1				
2	2	2	2	2	2	2	2	1	FRAQUEZA
1	2	2	1	1	2	2	2	1	MIALGIA
1	1		1	1	1			1	CEFALEIA D
1	1		1	1	1			1	MIALGIA
2	2	1	1	1	1	2	2	2	
1	1	2	2	2	2	2	2	1	MIALGIA
1	1	1	2	2	2	2	2	2	
2	1	2	1	1	1	2	2	1	
1	1	2	1	2	2	2	1	2	
1	1	2	1	1	1	2	2		
1	1		1	1	1	1			
2	1	2	1	2	2	2	2	2	
1	1	2	1	1	1	2	1	2	
1	1	2	1	2	1	1	2	2	
9	1	9	9	1	9	9	9	1	CORIZA
1	1	2	1	1	1	2	2	1	CEFALEIA
1	2	2	1	2	1	2	2	2	
1	1	1	1	1	1	2	2		
1	1	1		1					
1	1	2	1	2	1	2	2	1	GESTANTE,
1	1	2	2	2	2	1	1	2	
1	2	2	1	1	1	2	2	1	CEFALEIA, I
1	2	2	1	1	2	2	2		
2	1	2	1	2	2	2	2	2	
1	1			1		1			
1	1	2	1	1	1	2	2	2	

2	2	2	1	1	1	2	2		
1	1	2	1	1	2	2	2	2	
1	1	1	2	1	2	2	2	2	
2	1	1	1	1	1	1	1	1	IOT 01/04
1	1	2	1	1	1	2	2	2	
2	2	1	2	1	2	2	2	2	
2	2	2	1	1	1	2	2	1	DOR TORA
1	1	2	2	2	2	2	2	2	
1	1	1	1	1	1	2	2	9	
1	1	2	2	2	2	2	2	1	CEFALEIA,
1	1	2	1	1	1	2	2	1	CEFALEIA
1	1		1		1				
2	2	2	1	2	1	2	2	2	
1	1	1	1	2	1	2	2	2	
1	1	2	1	1	1	2	2	1	CORIZA
2	1	2	1	1	1	2	2	1	MIALGIA H
1	1	2	1	2	1	2	2	2	
1	1	2	2	2		2	2	1	CEFALEIA E
2	1	2	1	2	1	2	2	2	
1	1							1	MIALGIA
1	1	2	1	2	2	2	2		
1	1		1	1	1				
1	1	1	1	9	2	2	2	9	
2	1	2	1	1	1	2	2	2	
2	1	2	2	2	2	2	2	1	CORIZA, CA
1	1	1	1	1	1			1	MIALGIA, N
1	1		1		1	1			
1	1	1	1						
1	1	2	1	2	2	2	2	2	
1	1	9	2	2	2	2	2	1	CORIZA
2	1	2	1	1	1	2	2	1	NAUSEAS,
1	1	1		1		1	1	1	CEFALEIA,
			1		1				
	1	1	1					1	CORIZA
2	1	2	1	2	2	2	2	2	
1	1	2	1	1	1	2	2		
2	1	2	1	1	2	2	2	2	
2	2	2	2	2	2	2	2	2	
2	1	2	1	1	1	2	2	2	
1	1		1	1	1				
1	1	2	1	2	1	2	2	2	
1	1	1	1	1	1	2	1	2	

1	1	2	1	1	1	2	2	2	
2	1	2	1	1		2	2	1	DOR TORA
1	1	2	1	2	2	2	2	1	MILAGIA E
	1		1	1	1			1	CEFALEIA,
1	1	1	1	1	1	1	2	2	
1	1		1	1	1	2	2	2	
2	1	2	1	1	1	2	2	1	MIALGIA
1	1	1	1	1	1	2	2	2	
1	1	2	1	1	2	2	2	2	
1	1	2	1	1	1	2	2	2	
2	1	2	1	1	1	2	2		
1	1	2	1	1	1	2	2	1	CEFALEIA
1	2	2	1	1	2	2	2	1	INAPETEN
1	2	2	1	1	2	1	2		
2	2	2	2	2	2	2	2	2	
1	1	1	1	1					
1	2	2	1	1	1	2	2	1	MIALGIA;
2	1	2	1	1	1	2	2		
2	1	2	1	2	1	2	2	2	
1	1	2	2	1	1	1	1	2	
1	1	2	1	2	1	2	2	2	
1	1	2	1	1	1	2	2	2	
			1		1			1	DOR TORA
2	1	2	1	1	1	2	2	2	
1	1	1	1	1	1	2	9	2	
2	1		1	1	1	2	2	1	MAL-ESTA
2	1	1	1	1	9	2	2	1	ESPIRRO E
1	1	2	1		1	2	2	1	CORIZA MI
1	1	2	1	2	2	2	1		
1	1	2	1	2	1	2	1	1	CORIZA, D
1	1	2	2	2	2	2	2	1	ESPIRROS
2	2	2	1	1	1	2	2	2	
			1	1	1				
1	1	1	1	1	1	2	2	1	MIALGIA,
2	2	2	1	1	1	2	2	1	TAQUIPNE
1	1	1	1	1	1		1		
2	1	2	1	1	1	2	2	1	DOR TORA
1	1		1					1	CORIZA
2	2	2	1	1	2	2	2	2	
								1	PROSTRAC
1	1	2	1	1	1	2	2	2	
2	1	2	1	1	1	2	2	2	
1	1		1	1	1				
1	1	2	1	1	1	2	2	2	
2	1	2	1	1	1	2	2	1	VERTIGEM,
1	1		1		1				
	1		1		1			1	CEFALEIA,

1	1	2	2	1	1	2	2	2	
2	1	2	1	1	1	2	2		
			1	1	1			1	CEFALEIA
1	1	2	1	2	2	2	2	1	MIALGIA C
2	2	2	1	2	1	2	2	2	
2	2	2	1	1	1	2	2	2	
2	1	2	1	1	2	2	2	1	CEFALEIA, I
1	2	1	2	2	2	2	2	1	FRAQUEZA
1	1	2	1	1	1	2	2	2	
1	1		1	1	1			1	CEFALEIA
	1		1		1				
1	1	2	1	1	1	2	2	1	MIALGIA
2	2	1	1	1	2	2	2	1	NAUSEAS,
2	2	2	1	1	1	2	2	2	
1	2	2	1	1	1	2	2	1	CONGESTA
	1		1	1	1				
1	1		1	1	1				
1	1		1		1			1	CEFALEIA
1	1	2	1	1	1	2	2	1	MIALGIA
2	2	2	1	1	1	2	2		
				1					
1	1		1	1	1				
1	1	2	1	1	1	2	2	2	
1	1	2	1	1	1	2	2	1	MIALGIAS (
2	2	2	1	1	1	2	2		
1	1	1	1	2	2	2	2	2	
2	1	2	1	2	2	2	2	2	
2	1	2	1	1	1	1	2	1	CEFALEIA
2	1		1	1		2	2		
2	1	2	1	1	1	2	2	1	ASTENIA +
2	1	2	1	1	1	2	1	2	
1	1	2	1	2	1	2	2	2	
1	1		1				1		
1	1	1	1	1	1	2	1	2	
1	2	2	1	1	1	2	2	1	CEFALEIA
2	1	2	1	2	2	2	2	2	
2	1	2	2	1	1	2	2		
2	2	2	2	2	2	2	2	2	
2	1	2	1	1	1	2	2	2	
1	1		1	1				1	CEFALEIA, (
1	1							1	CEFALEIA E
	1		1	1	1			1	CANSACO
1	1	2	1	1	1	2	2	1	
1	1	2	1	1	2	2	2	2	
1	2	2	1	1	1	2	2	2	
2	1	2	1	1	1	2	2	2	
1	1	2	1	1	2	2	2	2	

1	1	2	1	2	1	2	2	1	GESTANTE
2	2	2	2	1	2	2	2	2	
1	1	2	1	2	2	2	2	1	CEFALEIA
2	1	2	1	1	1	1	2	2	
	1		1	1	1				
2	1	1	1	1	1	2	2	2	
1	1	2	1	1	1	1	1	2	
1	1							1	CALAFRIOS
1	1	1	2	1	2	2	2	1	CEFALEIA,
1	1		1	1	1				
	1	1	1	1	1			1	CEFALEIA
	1							1	CEFALEIA
1			1	1				1	MIALGIA, C
1	1	2	1	1	1	1	2	2	
1	1	2	1	1	1	2	2	1	FALTA DE A
1	1	2	2	2	2	2	2	2	
	1							1	CORIZA
1	2	2	1	1	2	2	2	2	
1	1	2	1	1	1	2	2	1	
1	1	1			1				
2	2	2	2	2	2	2	2	2	
2	1	2	1	1	1	2	2	2	
1	1	2	1	1	1	2	2	2	
1	1	2	1	2	1	2	1	1	GESTANTE
1	1	2	1	1	1	2	2	2	
1	1	2	1	1	2	2	1		
1	1	2	1	2	1	1	2	2	
1	1	1	1	1	1	2	2	2	
2	1	2	1	1	1	2	2	2	
2	1	1	1	1	1	2	2	1	TAQUIPNE
1	1	1	2	2	1	2	1	1	MIALGIA
			1	1	1			1	CORIZA
1	1						1	1	ASTENIA,C
	1		1					1	PROSTRAC
2	2	2	1	1	1	2	2	2	
1	1		1		1			1	MIALGIA,C
1	1		1					1	MIALGIA/C
1	1	2	2	1	1	2	2	2	
1			1	1	1			1	MIALGIA ,
2	2	2	2	2	2	2	2	1	SINT. GRIP.
1	1	1	1	1	1	2	2	2	
1	1	1	1	1	2	2	2	2	
			1	1	1			1	CEFALEIA
1	1	2	1	1	1	2	2	2	
1	1	2	1	1	1	2	2	1	ASTENIA
1	1	1	1	1	1	2	2	1	TAQUIPNE

2	1	2	1	2	1	2	2	1	CORIZA, M
1	1				1	2	2		
2	1	2	1	2	2	2	2	2	
1	1	2	1	1	1	2	2	2	
1			1		1				
1	1	2	1	2	2	2	2	1	ASTENIA
2	1	1	2	2	2	2	2	2	
1	1		1	1	1				
1	1	2	1	1	1	2	2	1	DOR EM TC
	1		1					1	MIOARTRA
1	2	2	2	2	2	2	2	1	CEFALEIA, I
1	1	2	1	1	1	2	2	1	MIALGIA, II
2	2	2	2	2	2	2	2	1	DOR TORA(
1	2	2	1	2	2	2	2	1	CORIZA+AS
1	1	1	1	2	2	2	2	2	
1	1							1	MAL ESTAR
1	1	1	1	1	1	2	2	2	
2	1	2	1	2	2	2	1	2	
1	1	2	1	1	1	2	2		
1	1	2	2	1	1	2	2	2	
2	1	1	2	2	2	2	2	1	CEFALEIA,A
	1			1	1				
2	2	2	1	1	1	2	2	2	
			1						
1			1	1	1				
1			1	1	1			1	CEFALEIA, I
1	2	2	2	2	2	2	2	1	CORIZA, M
2	2	2	2	2	2	2	2	2	
1	1	1	1		1	1	1	1	CORIZA/MI
1	1			1	1				
9	1	9	1	1	9	9	9		
1	1	1	2	1	1	2	2	2	
								1	ASSINTOM.
1	1		1		1			1	ASTENIA
1	1	2	1	1	1	2	2	1	CORIZA MI.
2	1	2	1	1	1	2	2	2	
1		1	1	1	1				
1	1		1	1	1				
			1	1	1				
1	1	2	1	1	1	2	2	2	
	1		1	1				1	MIALGIA
2	1	1	1	1	1	2	2	2	
1	1	1	1	2	2	2	2	2	
1			1					1	MIALGIA
1	1	1	1	2	1	2	2	1	DOR TORA(
1	1	1	1	1	1	1	1	2	
1	1		1	1	2	2	1		

1	1	2	2		2	1	2		
1	1	1	1	1	2	1	2	2	
	1		1						
2	1	2	1	9	9	2	2	1	ASTENIA, N
1			1	1	1				
2	2	2	1	2	1	2			
1	1	2	1	1	1	2	2	1	CORIZA TA
1	1		1		1		1	1	CEFALEIA
1	1		1	1	1				
1	1		1	1	1				
1	1		1	1	1	1		1	CORIZA, DC
	1		1						
	1		1		1		1	1	TAQUICARI
1	1	2	1	1	1	2	2		
	1		1	1	1			1	DOR TORAC
1	1	2	1	1	1	9	2		
1	1	9	1	1	1	2	2		
2	1	2	1	1	1	2	2	2	
1	1	2	2	1	2	2	2	2	
1	1		1	1				2	
2	1	2	1	1	2	2	2	2	
	1		1	1	1			1	CEFALEIA
1	1		1		1				
1	1	1	1	1	1				
2	1	2	1	1	1	2	2		
2	1	2	1	1	1			1	INAPETENC
1	1	2	1	2	1	2	2	1	MIALGIA
1	1								
	1		1			1			
1	2	2	1	2	2	2	2	1	CORIZA, M
2	1	2	2	1	1	2	2	1	MIALGIA
1	1	2	1	2	2	2	1		
	1				1			1	DOR TORAC
1	1	1	1	2	1	2	2	2	
2	2	2	1	1	1	2	2	1	GEMENCIA
2	1	2	1	1	1	2	2	2	
1	2	2	1	1	1	1	1	1	1
2	1	2	1	1	1	2	2	1	CEFALEIA, I
1	1	1	1	1	1	2	2	1	CORIZA
2	1	1	1	1	2	2	2	2	
1	2	2	1	2	1	2	2	1	INDISPOSIC
1	1		1		1	2	2		
1	1			1				1	MIALGIA
			1	1	1				
1	2	2	2	2	2	2	2	1	CEFALEIA E
1			1		1				
	1		1						

2	1	2	1	1	1	2	2	2	
1	1	2	1	1	1	2	1	1	MIALGIA A
1	1	1	2	1	1	1	2	2	
2	1	2	1	1	1	2	2	2	
1	1	2	1	1	1	2	2	2	
1	1		1	1	1			1	BRADICARD
	1	2	1	1	1	2	2		
	1		1		1	1		1	CEFALEIA
1	1	1	1	1	1	1	2	1	CEFALEIA
	1		1	1	1			1	HEMOPTIS
1	1	2	1	1	1	2	2	1	CEFALEIA,
2	2	2	1	2	1	2	2	2	
2	2	2	1	1	1	2	2	2	
1	2	2	2	2	1	2	2	1	MIALGIA+
2	2	2	1	1	1	2	2		
1	1	2	1	1	1	2	2	1	CORIZA
	1		1		1			1	DOR NO CO
2	1	2	2	1	1	2	2	2	
1	1	1	1	1	1	2	2		
1			1		1			1	MIALGIA, A
			1		1				
2	2	1	2	2	2	2	2	1	CALAFRIO
1	1	2	1	2	2	2	2	1	DOR LOMB
1	1	1	2	2	2	2	2	2	
1	1		1		1			1	DOR NO PE
1	1	1	1	1	1	2	2		
1	1	1	2	2	2	2	2	2	
1	1	2	1	1	1	2	2	2	
1	1	1	1	2	1	2	2	1	CEFALEIA;
1	1		1		1			1	CEFALEIA,
1	1		1		1	1		1	MIALGIA, C
2	1	2	1	1	1	1	1	1	CORIZA
			1		1				
	1		1		1	1		1	CEFALEIA,M
1		1	1	1				1	CEFALEI, M
1	1		1	1					
1	2	2	1	1	1			1	CEFALEIA,
1	1	2	1	1	1	2	2	1	MIALGIA
2	1	2	1	1	1	2	2	2	
1	1	1	1	1	1	1	1	2	
1	1	2	1	1	1	2	2		
1	1	1	1	1	1	2	2	2	
1			1	2	2			1	MIALGIA
1	1	1	1	1	1	2	2	2	
2	1	2	2	1	1	2	2	1	CORIZA
1	1			1	1				

1	1	1	1	2	2	2	2	2	
1	2	2	1	2	1	2	2	2	
1	1							1	CEFALEIA/
1	1	2	1	1	1	2	2	1	
	1		1						
1	2	2	1	1	1	2	2	2	
1	1				1			1	CEFALEIA E
1	1		1		1			1	MIALGIA, C
2	2	2	1	1	1	1	2	1	CEFALEIA
1	1	1	1	1	1	1	2	1	CEFALEIA,M
1			1	1	1				
	1		1	1					
1	1	2	2	2	1	2	2	1	CEFALEIA E
2	2	2	2	2	2	2	2	1	
	1	1	1	1	1	2	1	1	CEFALEIA,C
1	1	2	1	2	2	2	2	2	
1	2	2	1	1	2	2	2	1	CORIZA,ESI
2	1	2	1	1	1	2	2	2	
2	1	1	1	2	1	2	2	2	
1	1	2	1	1	1	2	2	1	CORIZA
2	2	1	1	1	1	2	9	2	
1	2	2	1	2	1	2	2	1	NAUSEA
1	1	2	2	1	2	2	2	1	SINT GRIPA
1	1	1	1	1	1				
1	1	2	1	1	1	2	2	1	HIPOREXIA
1	1	1	1	1	1	2	2	2	
1	1		1	2	2	2	2	2	
1	1	2	1	2	1	2	2	2	
2	1	2	2	1	1	2	2		
1			1	1	1				
	1		1					2	
1	1	2	1	2	2	2	1	2	
1	1	2	1	1	1	2	2	1	MIALGIA
1	1	2	2	1	2	2	2	2	
2	2	2	1	1	1	2	2	2	
1	1	1	1	1	1	2	2	1	LOMBALGI
2	1	2	1	1	1	2	2	2	
1	1	2	1	1	2	2	2	2	
	1			1	1				
1	1	2	1	1	1	2	2	1	PRECORDI
1		1	1		1			2	
1	1		1		1				
2	1	2	2	2	2	2		1	CORIZA, M
1	1	2	1	1	1	2	2	2	
	1		1	1	1				
2	1	2	1	1	1	2	2	1	CORIZA
9	1	9	1	9	9	9	9	1	DOR DE CA

1	1	2	1	1	1	2	2	2	
2	2	1	1	1	1	2	2	1	DOR OCUL
			1		1				
	1	1	1						
1	1	2	1	1	1	2	2	1	MIALGIA, T
2	1	2	1	2	1	2	2	1	CEFALEIA /
1	1	2	1	1	1	2	2	1	IRRITABILI
2	1	2	1	1	1	2	2	1	CORIZA, CA
					1				
1	1	2	1	1	1	2	2		
2	2	2	1	2	2	2	2	1	MIALGIA/C
1	1	2	1	1	1	2	2	2	
1	1		1	1	1			1	MIALGIA
1	1	2	1	1	2	2	2		
2	2	2	1	1	1	2	2	1	MIALGIA
1	1		1	1	1	2	2	2	
	1		1					1	TAQUIPNE
2	2	1	2	2	2	2	1	1	CEFALEIA
1	2	1	1	2	2	2	2	2	
1	1		1	1	2	2	2	1	SINT. GRIP
1	1	2	1	1	1	2	2	2	
1	1	2	1	1	1	2	2	1	MIALGIA,R
1	1	2	1	2	2	2	2	1	SINT GRIP
1	1	2	1	2	2	2	2	2	
1	1	1	1	1	1	2	2	2	
1	1	2	1	1	1	2	2	1	DOR TORA
	1		1						
2	1	2	1	2	1	2	2	2	
1	1	2	1	1	1	2	2	1	MIALGIA, T
1	1	1	1	2	1	2	2	2	
1	1	2	1	1	1	2	2	2	
1	1	2	1	1	1	2	2	1	CEFALEIA
1	1	1	1	1	1	2	2	1	DOR DE CA
1	1		1		1			1	HIPOTENS
2	1	2	1	1	1	2	2	1	NAUSEAS
1	1				1	1	1		
2	2	2	2	2	2	2	2	1	SINT GRIP
1	1		1						
1	1	1	1	1	1	2	2	2	
	1			1				1	CORIZA E C
1	1	2	1	1	2	2	2	2	
2	1	2	2	1	2	2	2	1	CEFALEIA,
2	1	2	2	2	2	2	2	2	
	1		1		1				
1	1	2	2	2	1	2	2	2	
2	1	2	1	1	1	2	2	2	
1	1	2	1	2	2	2	2	1	CEFALEIA

	1		1						
1	2	2	1	2	1	2	2	1	ASTENIA
	1			1				1	MIALGIA
2	2	2	1	1	1	2	2		
	1		1					1	CONGESTA
2	1	2	1		2	2	2	1	MIALGIA
1	1	2	1	1	2	2	2	2	
1	1	1	1	1	1	1	1	2	
9	1	1	1	1	9	9	9		
1	1	2	1	1	1	2	2	1	MIALGIA
1	1	1	1		1			1	DOR DE CA
2	1	2	1	1		2	2		
2	1	2	2	1	1	2	2	2	
1	1	1	1	1	1	2	2	1	MIALGIA
2	1	2	1	2	1	2	2	2	
			1	1	1				
1	1		1	1	1	2	2	2	
1	1			1	1				
2	1	2	1	1	2	2	2	2	
1	1	2	1	1	1	2	2	1	CEFALEIA,C
2	2	1	1	1	1	1	2	2	
1	1	2	1	2	1	2	2	1	DOR TORA
2	1	2	1	1	1	2	2	2	
1	2	2	1	1	1	2	2	2	
2	2	2	1	2	2	2	2	2	
1	1	9	9	9	2	1	1		
1			1	1	1			1	DOR NAS C
1	1		1	1	1				
1	1		1		1			1	MIALGIA, C
1	1	1	1	1	1		2	1	MIALGIA, A
1	2	2	2	2	2	2	2	2	
2	2	1	1	1	2	2	2	2	
1	1	1	1	1	1	2	2		
2	1	2	1	1	1	2	2	1	CEFALEIA
2	1	2	1	1	1	2	2	1	MIALGIA
	1		1					1	MIALGIA, C
2	2	2	2	2	2	2	2	1	DOR
1	1	1	2	1	2	1	1	2	
1	1	1	1	2	1	2	2	2	
1	1	2	1	1	1	2	2	1	CEFALEIA, A
1	1		1		1				
				1					
2	2	1	2	2	2	2	2	2	
2	1	2	1	1	1	2	2	1	CORIZA, M
2	2	2	1	1	1	2	2	2	
1	1		1		1			1	DORSALGIA
1	1	1	1	1	1	2	2	1	ASTENIA

1	1	2	1	1	1	2	2	1	MIALGIA,C
1	1	1	2	2	2	1	2	1	CALAFRIOS
2	1	2	1	1	1	2	2	2	
1	1	2	1	1	1	2	2	2	
	1		1	1	1			1	RESSECAM
9	1	9	1	9	1	9	9		
1	1		1	1	1				
	1		1	1	1				
1	1	2	1	1	1	1	2	2	
1	1	2	1	2	9	2	2	2	
	1							1	TONTURA,
1	1		1		1				
	1			1					
1	2	2	1	2	1	2	2	1	ASTENIA ,I
1		1	1		1			1	INAPETENC
1	1	1	1	1	1	1	2	2	
1	1	2	1	1	1	2	2	2	
	1		1	1	1				
2	1	2	1	1	1	2	2	2	
1	1		1	2	2	2	2	2	
1	1	2	1	1	1	2	2	2	
1	1			1	1				
2	1	1	2	1	2	2	2	1	CORIZA
1	1	1	1	1	2	2	2	2	
1	1	2	2	1	2	2	2	1	CORIZA
1	1	2	1	1	1	1	2	2	
1	1	1	1	1	2	1	1	1	HEMATEM
	1		1	1					
2	2	2	1	1	1	2	2	2	
2	2	2	1	1	2	2	2	2	
2	1	2	1	1	1	2	2	2	
	1		1		1			1	TAQUIPNE
2	2	2	1	1	1	2	2	2	
		1			1			1	MIALGIA, C
1	1	2	1	2	1	2	2	1	CEFALEIA
2	2	2	1	1	2	2	2	1	DOR PLEUR
1	2	2	1	2	2	2	1	2	
1	1	9	1	9	9	9	9	1	MIALGIA
1	1	2	1	1	1	1	1	2	
1	1	2	1	1	1	2	2	1	CEFALEIA
	1		1	1				1	MIALGIA
2	2	2	1	1	1	2	2	2	
2	2	2	1	1	1	2	2	1	MAL ESTAR
	1		1					1	CORIZA
2	1	2	2	2	1	1	2	2	
	1		1	1	1				

1	2	2	2	2	2	2	2	1	CEFALEIA
1	1	2	1	1	1	2	2		
1	1		1					1	FRAQUEZA
2	1	2	1	2	2	2	2	1	DOR NO PE
2	1	2	2	1	2	1	2	1	MIALGIA, C
1	1		1	1	1				
1	1		1	1	1			1	CEFALEIA D
			1	1	1				
1	1		1	1	1			1	MIALGIA,H
1	2	2	2	2	2	2	2	1	SANGRAMI
1	1		1	1	1				
1	1	2	1	1	1	1	2	2	
1	1		1	1	1				
1	2	1	2	2	2	2	2	1	CEFALEIA, C
1	1	1	1	1					
1	1	2	1	2	2	1	2	1	CEFALEIA, I
1	1		1	1	1	2	2	1	QUEDA DO
	1	1	1		1				
2	1	2	1	1	1	2	2		
2	2	2	1	2	1	2	2	2	
2	2	2	1	1	1	2	2	1	MIALGIA, C
1	1	2	1	1	1	2	2	2	
2	1	2	2	2	2	2	2	1	CORIZA
1	1		1						
2	1	2	1	2	1	2	2	1	DOR TORAC
1	1							1	CONGESTA
1	2	2	1	1	1	2	2	2	
1	1	1	1	1	1	2	2	1	CEFALEIA/C
1	1	2	1	1	2	1	2	2	
1	1	2	1	2	2	2	2	1	CEFALEIA
1	1	2	1	1	2	2	2	1	PROSTRAC
2	2	2	2	1	1	2	2	2	
1	1		1					1	MIALGIA
1	1		1		1			1	CEFALEIA, I
1	1	2	1	1	1	2	2	2	
			1					1	CANSACO
1	1	2	1	2	1	2	2	2	
	1							1	CEFALEIA, C
1	1	2	1	2	1	2	2	2	
2	1	2	1	2	2	2	2	1	CORIZA
			1		1			1	SANGRAMI
1	1						1	1	MAL ESTAR
2	1	2	1	1	1	2	2		
1	1	2	1	1	1	2	2	2	
2	2	2	1	2	2	2	2	2	
2	1	2	1	2	2	2	2	1	CEFALEIA
1	1							1	MIALGIA, C

1								1	CEFALEIA ,
2	2	2	1	1	1	2	2	2	
2	1	2	1	1	1				
1	1	2	1	1	1	2	2	2	
			1		1			1	ASTENIA
			1	1					
1	1	1	1	2	2	2	2	1	MIALGIA
	1		1	1	1				
1	1	2	1	1	2	2	1	2	
1	1		1		1				
1	2	2	1	1	1	2	2	1	CORIZA, M
2	1	2	2	2	2	2	2	2	
1	1	2	1	1	1	1	2	1	CEFALEIA, I
1			1	1	1			1	CEFALEIA
1	1		1					1	MIALGIA, D
1	1			1	1			1	MIALGIA
1	1		1	1	1	2	2	2	
1	1	1	1	1	1	1	2	2	
			1		1				
	1		1	1	1				
1	1	1	1	1	1	2	2	2	
2	1	2	1	2	2	2	2		
			1	1	1				
2	2	2	1	1	1	2	2	2	
2	2	2	2	2	2	2	2	2	
1	1	2	1	1	1	2	2	2	
2	1	2	1	1	1	2	2		
	1		1	1					
2	2	2	1	1	1	2	2	1	CIANOSE P
1	1		1		1			1	CEFALEIA
1	1	9	1	9	1	2	2	1	CEFALEIA
2	1	2	1	1	1	2	2	2	
1	1	2	1	2	2	2	2	2	
2	1	2	1	1	1	2	2	2	
1	1	1	2	2	1	2	2	2	
1	1		1					1	MIALGIA
1	1	2	1	2	2	2	2	2	
	1		1	1	1	1			
	1		1					1	CEFALEIA
1	1	1	1	1	2	2	2	2	
2	1	1	1	1	2	2	2	2	
2	2	2	1	2	1	2	2	1	SINDROME
9	1	9	1	9	9	9	9	1	DOR NO PE
2	2	2	1	1	2	2	2	2	
2	1	2	1	1	1	2	2	2	
1								1	CORIZA, M

1	1	2	1	1	1	2	2	1	MIALGIA
2	2	2	1	1	1	2	2	2	
1	1	2	1	1	1	2	2	1	CORIZA, D
2	1	2	1	2	2	2	2	2	
1	1	2	1	1	1	2	2	1	CORIZA
1	1		2	1	1	2	2		
1	1	2	1	1	1	1	2	1	CEFALEIA,
1	1		1					1	MIALGIA,C
1	1		1		1			1	MIALGIA
2	1	2	1	1	1	2	2	2	
2	1	2	1	1	1	2	2		
1	1		1		1		1	1	MIALGIA,
2	1	2	2	1	2	2	2	2	
2	2	2	2	1	1	2	2	1	CIANOSE C
1	1	2	1	1	1	2	2	1	MIALGIA, A
2	2	2	1	1	1	2	2	2	
1	1	2	1	1	1	2	2		
1	1	2	2	2	2	1	1	1	CEFALEIA E
1			1		1				
	1		1						
1	1		1	1				1	FRAQUEZA
1	1		1	1					
1	1	2	1	1	1	2	2	1	DOR NO C
1	2	2	1	1	1	2	2		
2	1	1	1	1	2	2	2	2	
1	1	2	1	1	1	2	2	2	
1				1	1				
2	2	2	1	2	1	2	2	2	
1	1	2	1	2	2	2	2	2	
2	1	2	1	1	1	2	2	2	
1	1	1	1	1	2	2	1	2	
2	2	2	1	1	1	2	2	2	
1	1		1			1		1	MIALGIA
2	1	2	1	1	1	2	2	1	TAQUICAR
2	1	2	2	2	1	2	2	2	
2	1	2	1	1	1	2	2	1	MIALGIA
1	1		1	1	1				
2	1	1	1	1	9	2	2	2	
2	1	1	2	2	2	2	2	1	CEFALEIA
2	2	2	1	2	1	2	2	2	
	1		1		1		1	1	CALAFRIOS
1	1	2	1	1	1	2	2	1	CORIZA, P
1	2		1	1	1	2	2		
1	1	1	1	1	1				
1			1		1				
1	1	2	1	2	1	2	2	1	CEFALEIA,
9	1	1	1	1	1	9	9	1	MIALGIA

2	1	2	1	1	2	2	2		
1	1	1	1	1	1	2	2	1	CEFALEIA/
1	1	2	1	1	1	2	2	2	
1	1		1	1	1				
1	1	2	1	2	1	2	2	1	CEFALEIA
2	2	2	2	2	2	2	2	1	CORIZA
2	1	1	1	2	1	2	2	2	
1	1	1	2	1	2	1	2	2	
2	1	2	1	1	1	2	2	2	
1	1	2	1	1	1	2	2	2	
1	1	2	1	1	1	2	2	1	CEFALEIA,
1	1	1	1	1	1	2	2	2	
1	1	2	1	1	2	2	1	1	ASTENIA, T
1	1	1	1	1	1	1	1	2	
1	1	2	1	2	2	2	2	2	
2	1	2	2	1	2	2	2	1	DOR DE CA
	1		1	1	1			1	CORIZA, CE
1	1	2	1	1	1	2	2	1	MIALGIA
			1	1	1				
2	1	2	1	1	1	2	2		
1	1		1					1	CEFALEIA
2	2	2	1	1	1	2	2	2	
2	1	2	1	1	1	2	2		
9	9	9	9	9	9	1	9	1	CEFALEIA,
1	2	2	1	1	1	2	2		
1	1		1	1	1	1			
		1	1		1				
2	1	2	1	2	2	2	2	2	
2	2	2	1	1	1	2	2	2	
1	1		1	1	1				
1	1		1		1				
2	1	2	1	1	1	2	2	2	
1	1				1				
2	1	2	2	2	2	2	2	1	CEFALEIA,
2	1	2	2	2	1	2	2	1	CEFALEIA
1	1	2	1	1	1	2	2	1	CALAFRIO,
1	1	2	1	1	2	2	2	2	
2	1	2	1	1	1	2	1	2	
1	1	2	1	1	1	2	2	2	
9	9	9	1	9	1	9	9	1	SINDROME
	1		1		1	1	1		
1	1		1	1				1	ASTENIA/C
1	1	2	1	1	1	2	2	2	
2	1	2	2	1	1		1		
2	1	2	1	1	1	2	2		
1	1	2	1	1	1	2	2	2	
2	2	2	1	1	1	1	1	1	PNEUMON

1	1	2	1	1	2	2	1	2	
1	1	1	1	1	2	9	1	1	HIPOREXIA
2	2	2	2	2	2	2	2	2	
2	2	2	1	2	2	2	2	1	ASTENIA
			1	1	1				
1	2	2	2	2	2	2	2	1	CEFALEIA, [illegible]
1	1	1	1	1	1			1	ASTENIA, H
1	1	2	1	1	1	2	1	2	
2	1	1	1	1	1	2	2	2	
1	1	2	2	1	1	2	2	2	
	1	1	1	1	1		1	1	DOR BAIXC
1	1	1	2	2	1	2	1	2	
	1		1		1			1	CEFALEIA
2	2	2	1	1	1	2	2	2	
1	1	1	1	1	1	1	1	1	MIALGIA, [illegible]
2	1	2	1	2	1	2	2	2	
1	1								
2	1	2	1	1	1	2	2		
1	1	1	1	1	1	2	2	2	
2	1	2	1	1	1	2	2	1	CANSACO
1	1	2	1	1	2	2	2	2	
1	1	1	2	1	1	2	2	1	DOR NO CC
1	1	2	1	1	1	2	2	2	
1	1	2	1	1	1	2	1	1	ASTENIA, C
1	2	2	1	1	1	1	1	1	CORIZA,MI
2	1	2	1	1	1	2	2	2	
	1		1		1			1	CORIZA, CE
			1	1	1				
1	1	2	1	1	1	2	2	2	
1	1	1	1	1	1	2	1	1	CORIZA E N
2	1	2	1	1	1	2	2	1	GEST. 2Âº [illegible]
1	1	2	1	1	1	2	2	1	ASTENIA
1	1		1	1	1			2	
2	1	1	1	1	1	2	2	2	
1	1			1					
2	1	2	1	1	1	2	1	1	MIALGIA/N
2	2	2	1	1	1	2	2	2	
1	1		1	1	1			1	INAPETENC
2	1	2	1	1	1	2	2		
1	1	2	1	1	1	2	2	2	
1	1	2	1	1	1	2	2	2	
1	1	2	1	1	2	2	1	1	MIALGIA, A
1			1		1			1	CEFALEIA/I
1	1	2	1	1	1	2	2		
1	1		1	1	1	1		1	DOR NO PE

1	1		1	1	1				
2	1	2	1	1	1	2	2		
	1			1	1			2	
1	1	1	1	1	1	2	2	2	
2	1	1	1	1	1	2	2	2	
2	1	2	1	2	1	2	2	2	
1	1		1	1	1			1	MIALGIA
2	1	2	1	2	1	2	2	2	
1	1	1	1	1	2	2	2	1	MIALGIA, A
	1	1			1			1	CORIZA
2	1	2	1	1	1	2	2		
	1				1				
2	1	2	1	2	1	2	2	1	CORIZA, HI
2	1	2	1	1	2	2	2	2	
2	2	2	2	2	2	2	2	1	ASSINTOM
2	1	2	1	1	1	2	2	1	CEFALEIA,
2	2	2	1	1	1	2	2	1	ESFORCO R
1	1		1	1	1		1		
1	1	2	1	2	1	2	2	2	
1	1	2	1	1	1	2	2	1	CANSACO,I
1	1	2	1	1	1	2	2	2	
2	1	2	1	1	2	2	2	2	
2	1	2	1	1	1	2	2	1	MIALGIA E
	1		1	1	1				
2	1	2	1	1	1	2	2	9	
1	1	2	1		1	2	2		
1	1	1	1	1	1	2	2	2	
	1							1	CEFALIA, M
1	1	2	1	1	1	1	2	1	CEFALEIA ,
1	1	2	2	1	1	2	2	1	CORIZA, M
			1	1	1	1			
1	2	1	1	1	1	2	2	2	
2	2	2	2	1	1	2	2	2	
1	2	1	1	1	1	2	2	2	
	1		1	1					
	1	2	2	2	1	1	2		
1	1	2	1	1	1	2	2	1	CORIZA E C
2	1	2	1	1	1	2	2		
1	1	1	1		1			1	CEFALEIA,D
1	1	2	1	1	1	2	2	1	CORIZA
1	1	1	1	2	2	2	2	2	
1	1		1	1	1			1	CORIZA E C
2	2	2	2	2	2	2	2	2	
2	1	2	1	1	1	2	2	1	CORIZA
				1	1				
			1	1	1				
2	1	2	2	2	2	1	1	2	

	1		1		1			1	CEFALEIA, S
2	1		1	1	2	2	2	1	ASTENIA
	1		1		1				
1	1				1			1	QUADRO G
1	1	2	1	1	2	2	2	2	
1	1	2	1	2	2	2	2	2	
2	1	2	1	1	1	2	2	1	CHOQUE H
	1		1	1	1			1	MIALGIA.
1	1		1					1	MIALGIA
1	1	2	1	2	2	2	2		
	1				1				
1	1	1	1	1	1	2	2	2	
1	1	2	1	2	2	2	2	1	RINORREIA
1	1		1	1				1	FRAQUEZA
2	1	2	1	1	1	2	2	2	
1	1		1						
1	1	2	1	1	1	2	2	2	
1	1	2	1	1	1	2	2		
2	1	2	1	1	1	1	2		
2	1	2	1	1	1	2	2	1	MIALGIA
1			1		1				
1	1	1	1	2	1	2	2	2	
1	1	2	1	1	1	1	2	2	
2	1	2	1	1	1	2	2	1	DOR TORAC
1	1	2	1	1	1			1	TAQUIPNE
2	1	2	2	2	1	2	2	2	
1	1	1	1	1	1				
1	1	2	1	2	1	2	2	2	
2	1	2	1	1	1	2	1	2	
2	2	2	2	1	1	2	2		
1	1	1	1	1	2	2	2	2	
1	1								
1			1	1	1				
1	2	2	1	2	1	2	2	1	DOR TORAC
	1		1	1	1				
2	1	2	1	1	1	2	2	2	
1	1		1	1	1			1	CEFALEIA, I
1	1	2	1	2	2	2	2	1	SINT GRIPA
2	2	2	1	1	2	2	2	2	
2	1	2	1	2	2	2	2	2	
2	1		1	1	1	2	2	1	DESORIENT
1	1	2	1	1	1	2	2	2	
2	2	2	2	2	1	2	2	2	
	1							1	DOR TORAX
1	2	9	1	1	1	2	2	1	MIALGIA, D
1	1	2	1	1	2	2	1	2	
1	1	1	1	1	1	1			

1	1	2	1	1	1	1	2	2	
1	1	1	1	1	1	1	2	2	
1	1	9	1	9	9	9	9	1	ANOSMIA
1	1	2	2	2	1	2	2	1	CEFALEIA,
1	2	2	1	1	1	2	2	1	ARTRALGIA
1	1	1	2	2	2	2	2	2	
2	1	2	1	1	2	2	2	2	
2	1	1	1	1	1	1	2	2	
1	1	1	1	1	1	2	2	2	
1	1	1	1		1	1			
2	2	2	1	2	2	2	2	2	
1	2	2	2	2	2	2	2	1	CEFALEIA
1			1		1			1	CEFALEIA
1	1	1	1	2		2	2	1	CORIZA, O
2	1	2	1		1	2	2	2	
1	1	2	1	1	1	1	2	2	
1	1	2	1	1	1	2	2	1	MIALGIA E
1	1				1				
1	1	2	1	1	1	2	2		
2	2	2	1	1	1	2	2	2	
1	1		1	1	1				
	1		1	1				1	SINTOMAS
			1						
	1		1	2	2	2	2	1	ODINOFAG
2	1	2	1	2	1	2	2	1	CEFALEIA
1	1		1		1			1	ASTENIA
2	1	2	2	1	2	2	2	1	CANSACO
2	1	2	1	1	1	2	2	2	
2	2	2	1	2	1	2	2	2	
1	1	2	1	1	1	2	2		
2	2	2	1	1	1	2	2	2	
1	1		1	1	1				
2	2	2	1	1	1	2	2	1	PROSTRAC
1	1	1	1	1	1			1	ODINOFAG
1	1		1	1	1			1	OBSTRUCA
9	1	9	1	1	9	9	9	9	
2	1	2	1	2	2	2	2	1	MAL ESTA
2	1	2	1	2	2	2	2	1	DOR TORA
	1			1	1				
2	2	2	2	2	2	2	2	1	CEFALEIA,
1	2	2	1	1	1	2	2	1	MIALGIA,
1	1	1	1	1	1			1	TIRAGEM I
2	2	2	1	1	1	2	2	2	
1			1		1			1	CEFALEIA,
1	2	2	1	1	1	2	2		
1	1	2	1	2	2	2	2	1	ASTENIA,
1	1	2	1	2	1	2	2	2	

2	1	2	1	2	1	2	2	1	TAQUIDISP
1	1	1	1	2	1	2	2	2	
								1	NAUSEAS
1	1	2	1	2	1	2	2	1	CEFALEIA
1	1	2	1	1	1	2	2	2	
	1	2	1	1	1	2		1	PROSTRAC
2	1	2	2	1	1	2	2	1	TAQUICAR
2	1	1	2	2	1	2	2	2	
1	1	2	1	1	1	1	2	1	CEFALEIA E
2	1	1	1	1	1	2	1	1	MIALGIAS,
1	1	2	1	1	2	2	2	2	
9	1	2	1	1	1	2	2	2	
2	1	2	1	1	1	2	2	1	CEFALEIA,
1	1	1	1	1	1			1	ASTENIA E
1			1	1	1				
1	1	2	1	2	2	2	2	2	
1	2	1	1	2	1	2	1	1	MIALGIA
1	1	2	2	1	2	2	2	2	
						1		1	ASTENIA
1	1	2	1	1	1	2	2	2	
1	1	1	1	1	2	2	2	2	
1	1	2	1	1	1	2	2	2	
2	1	2	1	1	1	2	2	1	MIALGIA,
1	1	2	1	1	1	2	2	1	CEFALEIA,
2	1	2	1	1	1	2	2	1	MIALGIA
								2	
1	2	2	1	1	1	2	2	2	
1	1	2	1	2	1	2	2		
1	1	1	1	1	1	2	2	2	
1	1	9	1	1	1	2	2		
	1				1			1	MIALGIA/
1	1	2	1	2	2	2	2	2	
1	1			1					
1	1	2	1	1	1	2	2		
1	1	2	1	1	1	2	1	1	CFALEIA/L
2	2	2	1	2	1	2	2	2	
2	1	2	1	1	1	2	2		
2	2	2	1	1	1	2	2	2	
1	1	2	1	1	1	1	2	2	
1	1	2	1	1	1	2	2	1	CORIZA E
1	2	2	1	1	1	2	2	2	
1	1	2	1	1	2	2	2	2	
1	1	2	1	1	1	2	2	2	
1	1	2	1	1	2	1	2	1	MIALGIA
1	1	2	1	1	1	2	2	2	
2	2	2	2	2	2	2	2	1	ASSINTOM
1	1	2	2	2	2	2	2	2	

1	1		1	1	1				
1			1		1			2	
	1		1						
1	1	2	2	2	2	1	2	1	ESCARRO
1	1	1	2	2	2	2	2	2	
1	2	2	1	1	1	2	2	1	CEFALEIA
1	1		1	1	2	2	2	1	ASTENIA E
2	1	1	1	2	2	2	2	2	
1	2	2	1	1	1	2	2	1	NAUSEAS
1	1	2	1	2	2	2	2	1	CEFALEIA, (
1	1	2	1	1	1	1	2	1	VOMITOS
1	1		1	1	1			1	EPISTAXE
1	1		1					1	CORIZA
2	1	2	2	2	2	2	2	2	
1	1	2	1		2	1	2	1	CEFALEIA, I
1	1	2	1	1	1	2	2	1	ALGIA MUS
2	1	2	1	1	1	2	2	1	MIALGIA, (
2	1	2	1	1	1	2	2		
9	1	1	1	1	1	9	9	1	CORIZA.
2	1	2	1	1	1	2	1	1	MIALGIA,C
	1		1	1	1			1	CORIZA + II
1	1	2	1	2	2	2	2	2	
1	1	1	1	1	1	2	2		
1	1		1	1	1				
1	1	2	1	1	1	2	2	1	CORIZA,INA
2	2	2	2	1	1	2	2	1	TAQUIPNEI
2	2	2	1	1	1	2	2	2	
1		1	1					1	MIALGIA
2	1	1	1	1	1	2	2	2	
2	1	2	1	1	1	2	2	1	CEFALEIA, (
1	1	1	1	1	2	2	2		
1	1	2	1	2	2	2	2	2	
								1	SINTOMAS
2	1	2	1	1	1	2	1		
2	1	2	1	1	1	2	2	1	MIALGIA
1	1	2	1	1	1	2	1		
2	2	2	1	1	1	2	2	2	
2	1	2	1	1	1	2	2	2	
1	1	2	1	1	1	2	2	1	CEFALEIA/
1	1	2	2	1	1	1	2	2	
2	2	2	2	2	2	2	2	2	
			1		1				
9	1	9	9	1	9	9	9	9	
1	1	1	1	1	1	1	1	2	
								1	CEFALEIA (
1	1	2	1	1	1	2	2	2	
1	1	2	1	2	2	2	2	1	CORIZA

1	1	2	1	1	1	2	2	2	
					1				
1	1	1	1	1	1	1	1	2	
1	1	2	1	1	1	2	2	1	MIALGIA
1			1		1				
2	2	2	1	2	1	2	2	2	
1	1	2	2	1	2	2	2	1	CORIZA, IN
1	1	2	1	2	1	1	1	2	
2	1	2	1	1	1	2	2	2	
1	1	2	1	1	1	2	2	2	
1	1		1	1	1			1	ARTRALGIA
1	1	2	1	2	2	2	2	1	MIALGIA
2	2	2	2	2	2	2	2	1	AGEUSIA, A
1	1		1					1	DOR TORAC
1	1		1	1				1	LOMBALGIA
1	1	2	1	1	1	2	2		
1	1	2	1	2	1	2	2	1	MIALGIA
2	1	2	2	2	2	1	1	2	
2	1	2	2	2	1	2	2	2	
1	1	2	1	1	1	2	2		
2	1	2	1	1	1	2	2		
2	1	1	1	1	2	1	2	2	
2	1	2	1	2	1	2	2	2	
2	1	2	1	1	1	2	2	2	
2	1	2	1	1	1	2	1	1	HIPEREMES
			1				1	1	NAUSEAS
1	2	2	1	2	2	1	2	1	MIALGIA,D
1			1		1			1	MIALGIA
1	1	2	1	1	1	2	1	2	
1					1			1	CONVULSA
1	1	2	1	1	1	2	1	1	TAQUIDISP
2	2	2	1	2	1	2	2	2	
	1								
1	1							1	MIALGIA, C
2	2	2	1	2	2	2	2	1	MIALGIA E
1	1	2	1	2	1	2	2	1	DOR BAIXO
1	1	1	1	1	1	2	2	2	
1	1					1		1	MIALGIA
2	1	2	1	2	2	2	2	2	
1	1	2	1	1	1	2	2	1	TAQUICARD
1	1	2	2	2	2	2	2	1	CEFALEIA
1	1		1	1	1		1	1	LOMBALGIA
1	1	1	1	1	2	2	2		
2	1	2	1	2	2	2	2	1	CEFALEIA, I
2	1	1	1	2	2	2	2	2	
2	1	2	1	1	2	2	2	1	MIALGIA , T
1	1	2	1	1	1	2	2	1	CEFALEIA, I

1	1	2	2	2	1	1	2	2	
2	2	2	2	2	2	2	2	2	
2	1	2	1	2	1	2	2	1	LOMBALGI
	1	1	1						
1								1	DOR TORA
2	2	2	1	2	1	2	2	1	CRISE CON
2	2	2	1	1	1	2	2		
1	1	1	1	1	1	2	2	1	DORSALGI
1	1	2	1	2	1	2	2	2	
1	1	2	1	2	2	2	2	1	MIALGIA
1	1	9	1	1	1	2	2		
1	1	1	1	1	1	2	2		
1		1	1				1	1	NAUSEAS
1	1	1	1	1	1	1	2	2	
1	2	2	1	1	2	2	2	1	INS RESPIR
1	1	1	2	2	2	2	2	1	DOR DE CA
1	1	2	1	1	1	2	2	1	CALAFRIO,
2	2	2	1	1	1	2	2	2	
	1		1	1	1				
1	2	2	1	1	1	1	1	2	
1	1	2	1	2	1	2	2	1	ASTENIA IN
2	1	2	1	1	1	2	2		
					1				
2	2	2	1	1	1	2	2	1	DOR TORA
								1	SINTOMAS
1	1	1	1	2	2	1	2	1	MIALGIA,C
2	1	2	1	1	2	2	2	2	
1	1	2	1	1	1	2	2	2	
2	2	2	1	1	1	2	2	1	CEFALEIA
1						1		1	CORIZA, M
			1					1	MIALGIA
1	1	1	1	1	1	2	2		
2	1	2	1	1	1	2	2	1	HIPOCORA
1	1	2	1	2	1	2	2	1	CEFALEIA, I
2	1	1	1	2	1	2	2	2	
1	1	2	1	1	1	2	2	1	CONTRACA
2	1	2	1	1	1	2	2	2	
2	1	2	1	1	2		2	2	
	1		1	1	1				
1	2	2	1	1	1	2	2	2	
1	1	2	1	2	1	2	1	2	
2	1	2		1	1	2	2	1	ADINAMIA
1	1	2	1	1	1	2	2	1	MIALGIA
1	1	2	1	1	1	2	1		
1	1	2	1	1	1	2	2	2	
1	1			1	1			1	TAQUIDISP

2	1	2	1	1	1	2	2	2	
1	1	2	1	1	2	2	2	1	LOMBLGIA
	1		1		1				
			1	2	1			1	ASTENIA
1	1		1					1	CEFALEIA/
1	1				1			1	DOR NO C
1	1	2	1	1	1	2	2	2	
2	1	2	1	2	2	2	2	2	
1	1	2	1	1	1	2	2	2	
1			1				1	1	HIPOREXIA
2	2	2	1	2	1	1	2	2	
	1			1	1			1	
1	1	1	1	1	1	2	2	2	
1	1	1	1	1	1	1	2	2	
1	1	1	1	1	1	1	2	1	DOR TORA
1	1		1		1			1	TAQUICAR
2	1		1	1	1			1	ESPIRROS,
1	2	2	2	2	2	2	2	2	
1	1	2	2	2	1	2	1	1	INAPETEN
1	1		1		1				
		1	1	1	1	2	2	1	
1	1	2	1	2	2	2	2	2	
	1		1					1	CONGESTA
1	1	2	1	1	1	2	2	1	INAPETEN
1	1	2	1	2	1	2	1	1	NAUSEAS
1	1	2	1	1	1	2	2	1	MIALGIA
2	1	1	1		1	2	2		
1	1	2	1	1	1	2	2	2	
1	1	1	1	1	1	2	2	1	CEFALEIA
2	2	2	1	2	1	2	2	2	
2	2	2	1	1	1	2	2	2	
	1		1	1	1	1			
			1	1				1	MIALGIA
1	1	2	1	1	2	2	2	2	
2	2	2	1	1	1	9	2	1	MIALGIA
1	2	2	1	1	1	2	2	1	MIALGIA D
2	1	1	2	2	2	2	2	9	
1	1		1	1	1		1		
1	1	2	1	1	1	2	2	1	ASTENIA
1	1	2	1	1	1	2	1	2	
2	1	2	1	1	1	2	2	2	
1			1		1				
1	1	2	1	1	1	2	2	2	
1	1	1	1	1	1	2	2	9	
1	1	1	1	2	2	2	2	2	
1	1	2	1	1	1	2	2	1	ASTENIA,
	1		1	1				9	

1	1	2	2	2	2	2	2	2	
2	1	2	1	1	1	2	2	1	ADINAMIA,
1	1	1	1	1	1		1	1	CEFALEIA,
1	1	9	1	2	1	2	2	2	
1	1		1		1				
1	1		1	9	1	2	2		
1	1	2	1	1	1	2	2	2	
1	1	2	1	2	2	2	2	2	
1	1	2	1	1	1	1	1	2	
2	1	2	1	1	1	2	2	1	HEMOPTIS
1	1		1	1	1		1		
2	1	2	2	1	1	2	2	2	
1	1	2	1	2	1	2	2	2	
2	1	2	1	1	1	2	2	1	MIALGIA, N
1	1	2	1	1	1	2	2	2	
1	1		1		1			1	DOR NAS C
1		1	1		1	1	1		
1	1	2	1	1	1	2	2		
2	1		1	2	2	2	2	1	DOR DE CA
2	2	2	1	1	1	2	2		
	1		1					1	ASTENIA, C
1	1	2	1	2	2	1	2	2	
2	2	2	1	2	2	1	2	2	
	1		1		1			1	CEFALEIA
1				1	1				
1									
2	2	2	1	2	2	2	2	2	
1	2	2	1	2	2	2	2	2	
1	1	2	1	2	1	2	2	1	MIALGIA
								1	CEFALEIA, I
2	2	2	1	1	1	1	1	1	CEFALEIA
1	2	1	2	1	2	2	2	1	CEFALEIA, I
1	9	9	1	1	1	9	9		
2	2	2	2	2	1	2	2	2	
2	1	2	1	1	1	2	2		
1	1	2	2	2	2	2	2	1	FADIGA / N
2	1	2	1	2	2	2	2	2	
1	1	2	1	2	2	2	1	2	
			1	1	1			2	
	1								
	1		1	1	1				
1	1	1	1					1	DOR DE CA
2	1	2	1	1	1	2	2	1	MIALGIA, C
		1	1	1	1				
1	1		1		1	1			
2	1	2	1	2	1	2	2	1	ASTOMIA

2	1	2	1	2	2	2		1	SINT GRIP
2	2	1	2	2	2	1	2	1	CEFALEIA
1	1	2	2	2	2	2	2	1	MAL ESTA
1	1	2	1	1	1	1	2	2	
2	2	2	1	1	1	2	2	2	
1	1	1	2						
1	1	1	1	1		2	2	2	
1	1	1		1	1			1	HIPOREXIA
2	1	9	1	1	1		2		
1	1	2	1	2	2	2	2	2	
2	2	2	1	2	1	2	2	1	NAUSEAS
2	2	2	2	1	1	2	2	2	
1	1	1	1	1	1	1	2	1	CEFALEIA
2	1	2	1	1	1	2	2	2	
1	1	1	1	2	1	2	2	2	
1	1	2	1	2	2	2	2	2	
	1			1					
1	1	2	2	1	2	2	2	2	
1	1	2	1	2	2	2	2		
			1	1	1				
					1				
1	1	1	1	1	1	2	2	2	
2	2	2	1	1	1	2	2	2	
2	2	2	2	2	2	2	2	2	
1	1	1	1	1	1	2	2	1	MIALGIA, C
1	1	2	1	1	9	2	2	1	INAPETEN
1			1	1	1			2	
1	1		1		1	1		1	MIALGIA,F
1	1	2	1	2	2	1	1	2	
	1		1					1	DOR NO CO
2	1	2	1	1	1	2	2	2	
2	1	2	1	1	1	2	2	1	CEFALEIA
			1		1				
1	1	2	2	2	2	2	2	1	MIALGIA
								1	
1								1	CORIZA, CE
1	1	2	1	2	2	2	2	1	MIALGIA E
			1	1	1				
1	1	2	1	1	1	2	2	2	
			1	1	1				
1	1	1	2	2	1	2	1	1	CEFALEIA,
2	2	2	1	2	1	2	2	2	
2	1	2	1	2	2	2	2	1	ASTENIA,
1	1	1	1		1				
1	1			1	1			1	DOR DE GA

1			1		1			1	ANASARCA
2	1	1	1	1	1	2	2		
1	1	9	1	1	1	9	1	1	CEFALEIA
1	1	2	1	1	1	2	2	2	
1	1	9	1	9	1	2	9	9	
1			1	1	2				
1	1	2	1	2	1	2	2	2	
1	1	2	2	2	2	2	2	1	DOR DE CA
	1	1	1	1	1				
1	1				1			1	MIALGIA
	1		1						
1	1	2	1	1	1	2	2	1	ASTENIA
1	1		1		1			1	
1	1		1	1	1			1	DISFAGIA
1	1	2	2	2	2	2	2	1	MIALGIA, A
	1	1	1	1					
1			1	1	1				
1	1	2	1	1	1	2	2	1	CEFALEIA
1	2	2	1	1	1	2	2	2	
1	1	2	1	1	1	2	2	1	MIALGIA
1	1		1		1			1	MIALGIA E
1	1	1	1	2	2	1	1	1	DOR NO CO
1	1		1	1	1				
1	1		1	1	1		1	1	DOR ABDO
1	2	1	1	1	1	2	2	1	ASTENIA
1	1	2	1	1	1	2	2	2	
2	1	2	1	1	1	2	1	1	MIALGIA,C
1	1	1	1	1	1	2	2	2	
			1		1				
1	1	2	1	1	1	2	1	2	
1	2	2	1	1	2	2	2	1	CANSACO
1	1	2	2	2	2	1	1	2	
			1						
1	1	2	2	1	1	2	2	1	CEFALEIA,
1	1	2	2	2	2	2	1	1	CEFALEIA,C
2	1	2	1	1	1	2	2	1	FALA INTEI
2	1	2	1	2	1	2	2	1	CORIZA
2	2	2	1	1	2	2	2	2	
2	2	2	1	1	2	2	2	2	
1	1	2	2	2	2	2	2	1	ADINAMIA
	1		1					1	CEFALEIA
1	1	1	1	1	1	2	2	1	CORIZA, CE
2	1	2	1	1	1	2	2	2	
2	1	2		1	1	2	2	2	
	1		1	1	1				
1	1	1	1	1	1	1	1	2	

1			1	1	1				
2	1	2	1	1	2	2	1	1	ASTENIA
1	1	2	1	1	1	2	2	1	ASTENIA
2	1	1	1	1	1	2	2		
1	1	2	1	2	2	2	2	1	MIALGIA
	1		1	1	1				
1	1		1		1		1		
2	1	1	2	2	2	2	2	1	CORIZA
	1		1		1				
1	1		1	1	1	2	2	2	
1	1	1	1	1	1	2	2		
1	1	1	1	1	1	2	2	1	MIALGIA
1	1		1	1	1			1	CEFALEIA
1	1							1	CANSACO
1	1	1	1	1	1	2	2	2	
				1	1			2	
1	1		1		1			1	MIALGIA
2	1	2	1	2	2	2	2	2	
1	1	2	1	1	1	2	2	1	MIALGIA E
1	1		1	1	1				
1	1		1		1				
	1		1	1				1	MIALGIA, C
1				1	1			1	MIALGIA
1	1	2	2	2	1	2	2	1	MIALGIA
2	1	2	1	1	1	2	2	1	MIALGIA, C
1	1	2	1	1	1	2	2	1	INAPETENC
2	2	2	1	2	2	1	2	2	
2	1	2	1	2	2	2	2	1	ASTENIA, C
2	1	2	1	1	1	2	2	2	
2	1	2	1	1	1	2	2	1	MIALGIA
1	1	2	1	1	1	2	2	1	ASTENIA
1	2	2	1	2	1	1	1	2	
2	2	2	1	1	1	2	2	2	
2	1		1	1	2	2	2	2	
9	1	2	1	1	1	2	2	2	
1	1	2	1	1	1	2	2	1	CORIZA E C
1	1	2	1	1	1	2	1	1	MIALGIA
1	1	2	1	1	1	2	2	1	ASTENIA, R
	1		1						
2	1	2	2	2	2	2	2	2	
1	1	2	1	2	1	2	2	1	HIPOREXIA
	1		1	1	1				
1	1		1	1	1	1		1	CORIZA + N
1	1	2	2	2	2	2	1	1	CEFALEIA
1	1	1	1	1		9	9		
1	2	2	2	2	2	2	1	1	ASTENIA, I
					1				

1	1	2	1	1	1	2	2	2	
2	1	2	2	2	2	2	2	1	
1	1	2	1	1	1	2	1	2	
2	2	1	2	2	2	2	2		
2	2	2	2	2	2	2	2	2	
1	1	1	2	2	2	2	1	1	CEFALEIA E
2	2	2	2	2	2	2	2	2	
1	1	1							
1	1	2	1	1	1	2	2	2	
1	1								
1	1		1		1		1	1	ASTENIA
	1					1		1	MIALGIA
2	2	2	2	2	2	2	2	2	
				1			1	1	CEFALEIA
2	2	2	1	2	1	2	2	2	
2	2	2	2	2	2	2	2	2	
1	1	1	2	1	2	2	2	2	
1	1	1	2	2	2	2	1	2	
9	9	9	9	9	9	9	9	9	
1	1	2	9	1	1	2	1	1	CEFALEIA E
1								1	GESTANTE
1	2	2	2	2	2	2	2	2	
2	1	1	2	1	2	2	1	1	
1	1	1						1	CEFALEIA, (
1	1	2	1	1	1	2	2		
2	2	1	2	1	1	2	2	2	
2	1	2	2	2	2	2	2	1	DESCONFO
1	1	1	1	1	1	2	2	2	
2	2	2	2	2	2	2	2	2	
1	1	2	2	1	1	2	2	1	OBSTRUCA
2	2	2	2	2	2	2	2	2	
1	2			1	1			1	CAFALEIA,
1	1	2	1	2	1	2	2	1	CEFALEIA, [illegible]
1	1	1	1	1	2	2	2	2	
9	1	9	9	1	1	9	9	1	SINTOMAS
2	2	2	2	2	2	2	2	2	
1								1	CEFALEIA
1	2	1	2	2	2	2	2	2	
1	1	1	1	1	1	2	2	1	CORIZA
1	2	2	1	1	1	2	2	1	CORIZA
1			1		1				
1	1	1	2	2	2	2	2	2	
	1							1	MIALGIA

2	2	2	2	2	2			1	PARTO CES
2	2	2	2	2	2	2	2	2	
1	1	2	2	2	2	2	2	1	DESIDRATA
1	1		1		1			1	HIPOTENSA
1	1	1	2	2	2	2	2	2	
2	2	2	2	2	2	2	2	2	
1	2	2	2	2	2	2	2	1	OBSERVAC
1	1							1	, CEFALEIA,
	1							1	CORIZA
1	1	2	1	2	1	2	2	2	
1	1	2	1	1	1	2	2		
			1	1	1				
2	2	2	1	1	1	2	2	2	
1	1			1				1	CONVULSA
								1	DOR DE CA
	1								
								1	PERDA DE I
1	1								
2	1	2	1	1	1	2	1	1	MIALGIA
1	1		1						
2	2	2	2	2	2	2	2	1	
2	2	2	2	2	2	1	2	2	
2	2	2	1	1	1	2	2	1	ASTENIA,C
2	1	2	2	1	2	2	2	1	CEFALEIA /
	1				1			1	CEFALEIA
1	1	1	1	1	1				
1	2	2	2	2	2	2	2	2	
1	1							1	CEFALEIA, I
2	2	2	2	2	2	2	2	2	
2	1	2	2	2	2	1	2	1	CEFALEIA
	1		1						
1			1		1			1	SINTOMAS
1	1	2	2	2	2	2	2	2	
	1		1	1			1		
2	2	2	1	2	2	2	2	2	
								1	NAUSEAS
1	1	1	1	1	1	2	2	2	
1	1	2	1	1	1	2	2		
1	1	1	2	2	2	1	2	2	
1	1	2	1	1	1	2	2	1	MIALGIA, A
1	1	1	1	1	1	2	2	2	
2	2	2	2	2	2	2	2	1	CORIZA
1	1		1						
2	1	2	1	1	1	2	2	1	CORIZA
2	1	2	2	2	2	2	2	1	CORIZA, CE

1	2	2	1	1	2	2	2	2	
1	1	1	2	2	2	2	2	1	CORIZA
	1	1							
9	9	9	9	9	9	9	9	1	CALAFRIOS
								1	CEFALEIA /
2	2	2	2	2	1	1	2	1	MAL ESTAR
1	1	1	1	1	2	2	2	1	CEFALEIA,
1	2	2	2	2	2	2	2	2	
2	1	2	2	1	1	2	2	1	CONVULCA
			1				1		
	1			1	1			1	INSUF. RES
1	1	1	1	1	1	1	2	2	
2	2	2	2	2	2	2	1	1	PANCITOPE
2	2	2	2	2	2	2	2	2	
2	1	1	1	1	1	2	2	2	
1	1	2	1	2	2	2	2	2	
1	1	1	1	2	2	2	2	2	
1						1		1	MIALGIA, C
			1	1					
2	1	1	1	1	1	2	2	2	
1	1	2	1	2	1	2	2	2	
1	1		1	1				1	MIALGIA
2	2	2	2	2	2	2	2	1	CORIZA

	PUERPERA	ATOR_RIS	CARDIOPAT	EMATOLO	IND_DOWI	HEPATICA	ASMA	DIABETES	IEUROLOGI	NEUMOPA
		N								
	2	S	2	2	2	2	1	2	2	2
		N								
PERDA DE P		N								
ORES NO CC		N								
	2	S	2	2	2	2	2	2	2	2
		N								
	2	S	2	2	2	2	2	2	2	2
		N								
		N								
		N								
	2	S	1	2	2	2	2	2	2	2
		S					1			
IA		N								
		N								
		N								
ALGIA		N								
		N								
	2	S	2	2	2	2	2	2	2	2
	2	S	2	2	2	2	2	2	2	2
		S						1		
ALGIA		N								
MIALGIA		N								
		N								
		N								
		N								
		N								
MIALGIA		N								
		S								
		N								
AUSEAS, CE		S								
	2	S	2	2	2	2	2	2	2	2
	2	S	1	2	2	2	2	2	2	2
	2	S	2	2	2	2	2	2	2	2
	2	S	1	2	2	2	2		2	2
CEFALEIA, D		N								
	2	S	2	2	2	2	2	1	2	2
	2	S	2	2	2	2	2	2	2	2
	2	S	2	2	2	2	2	2	2	2
		S						1		
		N								
	2	S	2	2	2	2	2	2	2	2
O NASAL, C		N								
IA		N								
		S								
	2	S	2	2	2	1	2	2	2	2

PERDA DE I		S								
CALAFRIOS		N								
CEFALEIA E (		N								
MIALGIA		N								
AGEUSIA		N								
		N								
	2	S	2	2	2	2	2	2	2	2
		S								
		S								
MIALGIA		N								
O NASAL		N								
	2	S	2	1	2	2	2	2	2	2
CALAFRIOS		N								
	2	S	2	2	2	2	2	2	2	2
	2	S	1	2	2	2	2	1	2	2
	2	S						1		
		N								
		N								
ATICO		N								
	9	S	9	9	9	9	1	1	9	9
ALGIA COF		N								
		N								
		N								
		N								
	2	S	1	2	2	2	1	2	2	2
		N								
AO CEFALEI		N								
	2	S	1	2	2	2	2	2	2	2
		S								
		N								
AR E NAUSE		N								
		N								
	2	S	2	2	2	2	2	2	2	2
		N								
		S	1					1		
		N								
	2	S	2	2	1	2	2	2	2	2
	2	S	2		1	1	2	2		1
	2	S	2	2	2	2	2	2	2	2
		N								
		S								
	2	S	1	2	2	2	2	2	2	2
		S	1							
CORIZA		N								
		S								
		S								
		N								

		N								
DIA,CEFALE		N								
		N								
		S								
		N								
DIA, MIALC		N								
	2	S	2	2	2	2	2	2	2	1
		N								
	2	S	2	2	2	2	2	2	2	2
E CALAFRIO		N								
	2	S	2	2	2	2	2	2	2	2
		S								
		S		1						
		S								
OLFATO		N								
		S							1	
	2	S	2	2	2	2	2	2	2	2
	2	S	1	2	2	2	1	2	2	2
		S								
	2	S	2	2	2	2	2	2	2	2
	2	S	2	2	2	2	2	2	2	2
		S					1			
		N								
	2	S	1							
		S								
		N								
CORIZA, M		N								
		N								
NAUSEAS		N								
NAUSEA,DO		N								
		S								
CICA		N								
	2	S	2	2	2	2	2	2	2	2
		N								
	2	S	2	2	2	2	1	2	2	2
CEFALEIA		N								
		S								
		N								
	2	S	2	2	2	2	2	1	2	2
	2	S	2	2	2	2	2	2	2	2
		N								
		N								
		N								
AL ESTAR		N								
	2	S	2	2	2	2	2	2	2	2
		N								
		N								

, CEFALEIA	S								
	N								
2	S	2	2	2	2	2	2	2	2
2	S	2	2	2	2	2	2	2	2
	N								
2	S		1						
DISGEUSIA	S								
2	S						1		
	S								
	S		1						
	S								
	S								
	S						1		
	N								
2	S	2	2	2	2	2	1	2	2
IELONEFRIT	N								
MINAL	N								
DIA	N								
2	S	2	2	2	2	2	2	2	2
	N								
2	S	2	2	2	2	2	2	2	2
CORIZA/MI	N								
AUSEAS, D	N								
	N								
2	S	2	2	2	2	2	2	2	2
2	S	2	2	2	2	2	2	2	2
	N								
2	S	1	2	2	2	2	2	2	2
2	S	2	2	2	2	2	2	2	2
2	S	1	2	2	2	2	2	2	2
2	S	2	2	2	2	2	2	2	2
	N								
STRUCAO N	S						1		
2	S	2	2	2	2	2	2	2	2
2	S	1	2	2		2	2	2	2
2	S	1	2	2	2	2	2	2	2
ANOSMIA, A	S								
2	S	1	2	2	2	2	2	2	2
ANOSMIA	N								
	S					1	1		
2	S	2	2	2	2	2	2	2	2
2	S	2	2	2	2	2	2	2	2
2	S	2	2	2	2	2	2	2	2
	N								
2	S	2	2	2	2	2	2	2	2
2	S	1	2	2	2	2	2	2	1
AR, DISURI	S								

		N								
	2	S	2	2	2	2	2	2	2	2
		S	1							
		N								
	2	S	2	2	2	2	2	2	2	2
		N								
	2	S	2	2	2	2	1	2	2	2
		N								
VOMITO, M		N								
		N								
		N								
		S					1			
	2	S	1	2	2	2	1	2	2	2
		S								
LFATO E P		N								
	2	S	1	2	2	2	2	2	2	2
		N								
ALAFRIOS		S								
	2	S	2	2	2	2	2	1	2	2
	2	S	2	2	2	2	2	1	2	2
		N								
	2	S	2	2	2		2	2	2	2
		S	1					1		
AUSEAS		N								
		N								
	2	S	2	2	2	2	2	2	2	2
		S								
		S								
	2	S	2	2	2	2	2	2	2	2
		S						1		
ATO E PAL		N								
	2	S	2	2	2	2	2	1	2	2
	2	S	1	2	2	2	2	1	2	2
ORIZA/MI		N								
		N								
ESACORD		S								
	2	S	2	2	2	2	2	2	2	2
ATICO		N								
	2	S	2	2	2	2	2	2	2	2
		N								
		N								
	2	S	1	2	2	2	2	2	2	2
	2	S	2	2	2	2	2		2	2
	2	S	2	2	2	2	2	2	2	2
NAUSEA		S								
		S								
ORPO		N								

		N								
	2	S	2	2	2	2	2	2	2	2
		S								
		S	2				2	1		
	2	S	2	2	2	2	2	1	2	2
		N								
	2	S	1					1		
	2	S	2	2	2	2	2	2	2	2
	2	S	2	2	2	2	1	2	2	2
		N								
		N								
		N								
CANSACO		N								
		S								
		N								
NEIA		N								
	2	S	1	2	2	2	2	2	2	2
		N								
		S								
	2	S	2	2	2	2	1	2	2	2
EFALEIA/AI		S								
		N								
		N								
		S						1		
	2	S	2	2	2	2	2	2	2	2
		N								
MIALGIA DI		N								
O NASAL		N								
		N								
		S								
		S								
CONGESTA		N								
		N								
	2	S	2	2	2	2	2	1	2	2
CICA,		S								
	2	S	2	2	2	2	2	2	2	2
	2	S	2	2	2	2	2	2	2	2
IA NOS OLH		N								
		N								
		S								
DOR TORA		N								
CEFALEIA		N								
	2	S						1		
RILHA		N								
	2	S	2	2	2	2	2	1	2	2
		S								
		N								

AGEUSIA		N								
MIALGIA		N								
	2	S	2	2	2	2	2	2	2	2
PAIS		N								
	2	S	2	2	2	2	2	2	2	2
	2	S	2	2	2	2	2	1	2	2
		S								
MIALGIA		N								
ANOSMIA, /		S								
		N								
	2	S	2	2	2	2	2	2	2	2
ONTURA		N								
		N								
	2	S	2	2	2	2	2	2	2	2
	2	S	2	2	2	2	1	2	2	2
		S							1	
		N								
DOR RETOOI		N								
		N								
CORIZA		S								
	2	S	1	2	2	2	2	2	2	2
		N								
CORIZA, MI		N								
	2	S	2	2	2	2	2	2	2	2
		N								
		N								
		S								
DIA/ANOSN		S						1		
		S								
	2	S		2	2	2	2	1	2	2
		N								
	2	S	2	2	2	2	2	2	2	2
		N								
A		N								
CORIZA E D		N								
		S								
		N								
		S								
		N								
		N								
		S	1							
		N								
CORIZA		N								
		N								
		S								
	2	S	2	2	2	2	2	2	2	2
		N								

		N								
		S								
		S	2	2	2	2	2	2	2	2
		N								
	2	S								
		S								
		N								
	2	S	2	2	2	2	2	2	2	2
		N								
		N								
	2	S	2	2	2	2	2	2	2	2
ORPO		N								
MIALGIA		N								
		N								
		N								
	2	S	2	2	2	2	2	2	2	2
	2	S	2	2	2	2	2	2	2	2
		S								
		N								
		S								
FADIGA		S								
MIALGIA		S	1	2	2	2	2	2	2	2
AGENESIA/		N								
MIALGIA		N								
		N								
		N								
		N								
R 127		N								
	2	S	2	2	2	2	1	2	2	2
		N								
	2	S	2	2	2	2	2	2	2	2
	2	S	2	2	2	2	2	2	2	2
	2	S	2	2	2	2	2	2	2	2
		N								
	2	S	2	2	2	2	2	2	2	2
DOR TORAC		N								
		N								
TAQUIPNEI		N								
	2	S	2	2	2	2	2	1	2	2
		S								
		N								
	2	S	2	2	2	2	2	1	2	2
	2	S	2	2	2	2	2	2	2	2
DIA		S								
		N								
		N								
	2	S	2	2	2	2	2	2	2	2

	N								
2	S	2	2	2	2	1	2	2	2
	N								
	N								
EFALIA, AR	S								
2	S	2	2	2	2	2	2	2	2
	N								
	N								
2	S	2	2	2	2	2	2	2	2
	S	1							
	S					1			
	S						1		
DIA,CON CC	S								
	S								
	S	1					1		
	N								
	N								
2	S	2	1	2	2	2	2	2	2
	S								
O NASAL	N								
2	S	9	9	2	9	1	9	2	9
2	S	2	2	2	2	2	2	2	2
2	S	2	2	2	2	2	1	2	2
2	S	1	2	2	2	2	2	2	2
	N								
	S					1			
2	S	1	2	2	2	1	1	2	2
2	S	1	2	2	2	2	1	2	2
2	S	2	2	2	2	2	2	2	2
AO DE OLFA	N								
DIA/DOR AI	N								
2	S	2	2	2	2	2	2	2	2
2	S	2	2	2	2	2	2	2	1
2	S	2	2	2	2	2	1	2	2
DOR TORAC	N								
AGIR COM	N								
	N								
	N								
	N								
2	S	2	2	2	2	1	2	2	2
	S								
	S								
2	S		2	2	2	2	2	2	2
	N								
2	S	2	2	2	2	2	1	2	2
	N								
	S								

ASTENIA		N								
FALEIA		N								
CICA , MAL		S								
	2	S	2	2	2	2	2	2	2	2
A		N								
		S								
	2	S	2	2	2	2	2	2	2	2
		N								
		S						1		
	2	S	2	2	2	2	2	2	2	2
		N								
	2	S	2	2	2	2	2	2	2	2
		N								
,CIANOSE		S								
	2	S	2	2	2	2	2	2	2	2
	2	S	1	2	2	2	2	2	2	2
		N								
AR, MIALGI		N								
	2	S	2	2	2	2	2	2	2	2
		N								
		N								
		S								
		N								
R		S								
		N								
		N								
		S								
		N								
		N								
	2	S	2	2	2	2	2	2	2	2
MIALGIA		N								
		N								
R, MIALGIA,		S								
AR, NAUSE		S								
		N								
	2	S	2	2	2	2	2	1	2	2
	2	S	2	2	2	2	1	2	2	2
		N								
		N								
	2	S	2	2	2	2	2	2	2	2
		N								
CICA,HIPOR		S								
	2	S	1	2	2	2	2	1	2	2
		S	1							
		N								
		S								
		N								

CORIZA		N								
	2	S	2	2	2	2	2	2	2	2
	2	S	2	2	2	2	2	2	2	2
	2	S		2	2	2	2	2	2	2
ORIZA		N								
		N								
	2	S	2	2	2	2	2	2	1	2
		S						1		
	2	S	2	2	2	2	2	1	2	2
ANOSMIA		N								
		N								
	2	S	1	2	2	2	2	2	2	2
		S								
E AGEUSIA		N								
	2	S	2	2	2	2	2	2	2	2
	2	S	2	2	2	2	1	2	2	2
		N								
CORIZA, PR		S								
	2	S	1	2	2	2	2	1	2	2
		N								
	2	S	2	2	2	2	2	2	2	2
		S								
		S						1		
		N								
	2	S	2	2	2	2	2	2	2	2
		N								
	2	S								
CORIZA E FI		N								
		N								
		S								
CEFALEIA		N								
		S								
		S								
	2	S	1	2	2	2	2	2	2	2
VULSIVA		N								
		N								
CORIZA		S								
		S								
EFALEIA		S						1		
	2	S	2	2	2	2	2	2	1	2
	2	S	2	2	2	2	2	2	2	2
		N								
		N								
		S						1		
	2	S	2	2	2	2	2	2	2	2
	2	S	2	2	2		2	2	2	2
	2	S	2	2	2	2	2	1	2	2

		N								
		N								
	2	S								
	2	S	2	2	2	2	2	2	2	2
	2	S	2	2	2	2	2	2	2	2
	2	S	2	2	2	2	2	2	2	2
	2	S	1	2	2	2	2	2	2	2
	2	S	2	2	2	2	2	2	1	2
	2	S	2	2	2	2	2	2	2	2
		S								
	2	S	1	2	2	2	2	2	2	2
		N								
	2	S	2	2	2	2	2	2	2	2
	2	S	2	2	2	2	2	2	2	2
	2	S	1	2	2	2	2	1	2	2
	2	S	2	2	2	2	2	2	2	2
	2	S	2	2	2	2	2	2	2	2
		N								
	2	S	2	2	2	2	2	1	2	2
	2	S	2	2	2	2	2	2	2	2
	2	S	2	2	2	2	2	1	2	2
		N								
	2	S	2	2	2	2	2	2	2	1
DOR TORA		N								
		N								
		S								
	2	S	1	2	2	2	1	2	2	2
	2	S	2	2	2	2	2	2	2	2
	2	S	1	2	2	2	2	2	2	2
	2	S	2	2	2	2	2	2	2	2
CIA		N								
IALGIA, CEF		N								
	2	S	2	2	2	2	2	2	2	2
		N								
	2	S	2	2	2	2	2	2	2	2
	2	S	2	2	2	2	2	2	2	2
MIALGIA		S	2	2	2	2	2	2	2	2
CICA		N								
		N								
		N								
		S					1			
	2	S	2	2	2	2	1	1	2	2
	2	S	2	2	2	2	2	2	2	2
		N								
		S						1		
	2	S	2	2	2	2	2	2	2	2
		N								

	2	S	1	2	2	2	2	2	2	2
		S								
	2	S	2	2	2	2	2	2	2	2
	2	S	2	2	2	2	2	2	2	2
		N								
	2	S	2	2		2	2	2	2	2
CORIZA		S								
		N								
		N								
	2	S	1	2	2	2	2	2	2	2
		N								
	2	S	2	2	2	2	2	2	2	2
CICO. MIAL		N								
	2	S	2	2	2	2	2	2	2	2
	2	S								
	2	S	2	2	2	2	1	2	1	2
		N								
	2	S	1	2	2	2	2	1	2	2
		S								
		S								
DIA		N								
		N								
		S								
	2	S	2	2	2	2	2	2	2	2
		S					1			
		N								
	2	S	2	2	2	2	2	2	2	2
	2	S	2	2	2	2	2	2	2	2
MIALGIA,		N								
		N								
	2	S	1	2	2	2	2	1	2	2
		N								
		S	1							
	2	S	1	2	2	2	2	2	2	2
	2	S	2	2	2	2	2	1	2	2
		S								
	2	S								
	2	S	2	2	2	2	2	2	2	2
NAUSEAS		N								
,ANOSMIA,		S								
		N								
		S								
	2	S	2	2	2	2	2	1	2	2
STRUCAO		N								
		N								
	2	S	2	2	2	2	2	2	2	2
	2	S	2	2	2	2	2	1	2	2

IA		N								
	2	S	2	2	2	2	2	2	2	2
	2	S	2	2	2	2	2	2	2	2
MIALGIA		N								
	9	S	9	9	9	9	9	9	9	9
		S				1				
		S								
	2	S	2	2	2	2	2	2	2	2
		N								
		S								
	2	S	1	2	2	2	2	2	2	2
CORIZA, MI		N								
	2	S	2	2	2	1	2	2	2	2
	2	S	2	2	2	2	2	2	2	2
		N								
	2	S	2	2	2	2	1	2	2	2
		N								
	2	S	2	2	2	2	2	2	2	2
ALGIA		S								
		S					1			
		S						1		
		S					1			
CEFALEIA		N								
	2	S	2	2	2	2	2	2	2	2
		N								
	2	S	2	2	2	2	2	2	2	2
	2	S	2	2	2	2	1	2	2	2
		N								
		S		1						
	2	S	2	2	2	2	9	2	2	2
	2	S	1	2	2	2	2	1	2	2
	2	S	2	2	2	2	2	2	2	2
		N								
		S								
		S						1		
CORIZA, CAI		N								
	2	S	1	2	2	2	2	2	2	2
		N								
ATICO		N								
		N								
		N								
MILGIA E A		S						1		
	2	S	2	2	2	2	2	2	2	2
	2	S	2	2	2	2	1	2	2	2
		S	1							
	2	S	2	2	2	2	2	2	2	2
	2	S	2	2	2	2	2	1	2	2

	N								
	N								
	N								
2	S	2	2	2	2	2	2	2	2
2	S	2	2	2	2	2	2	2	2
NAPETENCI	S								
	N								
CONGESTA	N								
	N								
2	S	1	2	2	2	2	1	2	2
2	S	2	2	2	2	2	2	2	2
	N								
ATICO	N								
MINAL, CEI	N								
2	S	2	2	2	2	1	2	2	2
A	N								
EFALEIA, DI	S						1		
2	S	2	2	2	2	2	2	2	2
2	S	2	2	2	2	2	2	2	2
IIALGIA	N								
	N								
2	S	2	2	2	2	1	2	2	2
2	S	2	2	2	2	2	2	2	2
	N								
	S								1
	N								
2	S	2	2	2	2	2	2	2	2
MIALGIA,CC	S								
	N								
2	S	1	2	2	2	2	2	2	2
2	S	2	2	2	2	2	2	2	2
	S								
	S								
2	S	1	2	2	2	2	2	2	2
2	S	2	2	2	2	2	2	2	2
	S								
2	S	1	2	2	2	2	1	2	2
2	S	1	2	2	2	2	1	2	2
ALGIA	S								
2	S	2	2	2	2	1	1	2	2
	S								
	S								
2	S	2	2	2	2	2	2	2	2
	S								
	N								
	S								
	S								

2	S	2	2	2	2	2	2	2	2
	S					1			
2	S	2	2	2	2	2	2	2	2
2	S	2	2	2	2	2	2	2	2
	S	1							
	S	1							
	S								
2	S	2	2	2	2	2	2	2	2
MIALGIA,CC	N								
	N								
	N								
2	S	2	2	2	2	2	1	2	2
	N								
ASTENIA	N								
	N								
	N								
	N								
FRAQUEZA,	N								
PERDA DO	N								
2	S	2	2	2	2	2	2	2	2
	N								
2	S	2	2	2	2	2	1	2	2
9	S	9	9	9	9	9	9	9	9
2	S	2	2	2	2	1	2	2	2
ORPO, PERD	N								
CICA	N								
	N								
2	S	2	2	2	2	2	2	2	2
	N								
2	S	2	2	2	2	2	2	2	2
MIALGIA E (	N								
	S	1							
CICA	N								
	N								
2	S	2	2	2	2	2	2	2	2
	N								
	N								
	S								
R	S								
2	S	2	2	2	2	1	2	2	2
	S						1		
2	S	2	2	2	2	2	1	2	2
2	S	1	2	2	2	2	2	2	2
2	S	1	2	2	2	2	2	2	2
ORPO	N								
IXO VENTR	N								
	S	2	2	2	2	2	2	2	2

	2	S	2	2	2	2	2	2	2	2
MIALGIA, A		S	1							
	2	S	2	2	2	2	2	1	2	2
		N								
E AGEUSIA		N								
	2	S	2	2	2	2	1	2	2	2
O NASAL		N								
	2	S	2	2	2	2	2	2	2	2
	2	S	2	2	2	2	2	2	2	2
		S								
	2	S	2	2	2	2	2	2	2	2
		N								
		S	1					1		
	2	S	2	2	2	2	2	2	2	2
	2	S	1	2	2	2	2	2	2	2
		N								
	2	S	2		2	2	1	2		2
		N								
		N								
NAUSEA		S								
	2	S	2	2	2	2	2	2	2	2
		S								
		N								
		N								
		S						1		
		N								
	2	S	2	2	2	2	2	1	2	2
MAL ESTAR		N								
DOR TORAC		N								
MAS CESA		N								
	2	S	2	2	2	2	2	2	2	2
	2	S	2	2	2	2	2	2	2	2
	2	S	1	2	2	2	2	2	2	2
X		N								
		S						1		
IALGIA		N								
		N								
	2	S								
CALAFRIOS,I		S	2	2	2	2	1	1	2	2
		S	1					1		
AIXO VENTR		N								
		N								
		N								
		N								
	2	S	2	2	2	2	2	2	2	2
		N								
STENIA		N								

		N								
		S								
		N								
	2	S	2	2	2	2	2	2	2	2
	2	S	2	2	2	2	2	2	2	2
		S								
GRIPAIS		N								
	2	S	2	2	2	2	1	2	2	2
DIA, CONGI		S								
'ROSTRACA		N								
		N								
	2	S	2	2	2	2	2	2	2	2
		S								
	9	S	9	9	9	9	9	9	9	9
		N								
		S								
	2	S	2	2	2	2	2	2	2	2
IALGIA		N								
	2	S		2	2	2	2	1	2	2
	2	S	2	2	2			2		
		S								
		S								
	9	S	9	9	9	9	9	9	9	9
		N								
		N								
		N								
	2	S	2	2	2	2	2	1	2	2
	2	S	1	2	2	2	2	1	2	2
	2	S	1	2	2	2	2	1	2	1
		N								
		N								
		S								
MIALGIA/C		N								
		S	1				1			
POGASTRIC		S								
	2	S	2	2	2	2	2	2	2	2
	2	S	1	2	2	2	2	2	2	2
		N								
	2	S	2	2	2	2	2	2	2	2
	2	S	2	2	2	2	2	2	2	2
	2	S	2	2	2	2	2	2	2	2
D, TAQUIPN		N								
	2	S	2	2	2	2	2	2	2	2
		S	1							
	2	S	2	2	2	2	2	2	2	2
		S								
		N								

2	S	2	2	2	2	2	2	2	2
2	S	2	2	2	2	2	2	2	2
	N								
IALGIA, DIG	S								
2	S	2	2	2	2	2	2	2	2
2	S	1	2	2	2	2	2	2	2
	N								
2	S	2	2	2	2	2	2	2	2
	S	1					1		
, CHOQUE	N								
2	1	2	2	2	2	2	2	2	2
2	1	2	2	2	2	2	2	2	2
	2								
2	1	2	2	2	2	2	2	2	2
, ODINOFA	1						1		
	2								
X	1								
CICA	2								
	2								
IA	2								
	2								
CICA	1								
	1								
2	1	1	2	2	2	2	1	2	2
A,MIALGIA	1						1		
2	1	9	9	9	9	9	1	9	9
	2								
	1								
FALEIA, DOI	2								
	2								
2	1	2	2	2	2	2	2	2	2
, MIALGIA	2								
DINOFAGIA	2								
LAFRIO, DC	2								
EFALEIA	2								
2	1	2	2	2	2	2	2	2	2
	1	1							
	2								
	1						1		
AIXO DO VE	2								
	2								
MIALGIA	2								
2	1	2	2	2	2	2	2	2	2
	1	1					1		
	1						1		
	1	1							
	1	1							

	2	1								
	2	1	2	2	2	2	2	1	2	2
OR TORACI		2								
)NGESTAO		1				1				
		1								
-		2								
	2	1	1	2	2	2	2	2	2	2
AIS		2								
		2								
		2								
	2	1	1	2	2	2	2	1		2
	2	1	2	2	2	2	2	2	2	2
		1			2					
	2	1	2	2	2	2	2	2	2	2
DOR TORACI		2								
	2	1	2	2	2	2	1	2	2	2
		2								
		1								
		2								
	2	1	2	2	2	2	2	2	2	2
		2								
	2	1	2	2	2	2	2	2	2	1
		1								
	2	1	2	2	2	2	2	2	2	2
		2								
AIS		2								
AIS		2								
DOR TORAC		2								
		2								
	2	1	2	2	2	2	2	2	2	2
		2								
EFALEIA, C(		2								
		1								
		2								
DIA		1								
S		1								
	2	1	2	2	2	2	2	2	2	2
		1						1		
	2	1	2	2	2	2	2	2	2	2
IIPOREXIA		1						1		
		1								
		2								
	2	1	1	2	2	2	2	2	9	2
	2	1	2	2	2	2	2	2	2	2
AIS		2								
		2								
	2	1	2	2	2	2	2	2	2	2

		1								
		2								
DIA E TAQU		2								
	2	1	1	2	2	2	1	2	2	2
DOR NO CC		2								
	2	1	2	2	2	2	2	2	2	2
		2								
	2	1	2	2	2	2	2	2	2	2
		2								
		2								
	2	1	2	2	2	2	2	2	2	2
ALGIA		1								
		2								
CEFALEIA		2								
.O		1								
	2	1	2	2	2	2	2	2	2	2
		2								
CALAFRIO		1								
		1								
MIALGIA		2								
		2								
	2	1								
		2								
	2	1	2	2	2	2	2	2	2	2
	2	1	2	2	2	2	2	2	2	2
		2								
		1								
	2	1	2	2	2	2	2	2	2	2
		1								
		2								
		2								
.O NASAL		2								
	2	1	2	2	2	2	2	2	2	2
		2								
	2	1	2	2	2	2	2	1	2	2
		1								
		2								
		2								
		2								
ATICA		2								
ERIFERICA		1								
		2								
	2	1	2	2	2	2	2	2	2	2
AIS		2								
		2								
	2	1	2	2	2	2	2	2	2	2
		2								

		1								
	2	1	2	2	2	2	2	2	2	2
AGEUSIA, N		2								
		1								1
ALGIA, MIA		2								
		2								
	2	1	1	2	2	2	2	1	1	2
		2								
	2	1	1	2	2	2	2	2	2	2
		2								
	2	1	2	2	2	2	2	2	2	2
		2								
		2								
ATICO		2								
AR		2								
		2								
		1								
		1								
		1					1			1
	9	1	9	9	9	9		9	9	9
CICA,NAUSI		2								
		2								
		2								
		1								
	2	1	2	2	2	2	2	2	2	2
	2	1	2	2	2	2	2	2	2	2
		2								
DIA		2								
		2								
		2								
		2								
		1								
		2								
DOR NO COI		2								
		2								
		2								
MINAL, FAI		1								
	2	1	2	2	2	2	1	2	2	2
	2	1	2	2	2	2	2	2	2	2
		2								
		1	1							
		2								
		2								
		2								
ATICA		1								
AIS		2								
		2								

2	1	2	2	2	2	2	2	2	2
F. DE APETI	2								
	2								
	2								
	2								
	2								
2	1	2	2	2	2	2	2	2	2
ORIZA	2								
	1								
2	1	2	2	2	2	2	2	2	2
DOR LOMBA	1								
	2								
	1						1		
	2								
2	1	1	2	2	2	2	2	2	2
2	1	2	2	2	2	2	2	2	2
	2								
2	1	2	2	2	2	2	2	2	2
	1								
	1								
	1						1		
2	1	2	2	2	2	2	2	2	2
	2								
ORPO, CEFA	2								
	1	2	2	2	2	1	2	2	2
	2								
	2								
2	1	2	2	2	2	2	2	2	2
	1	2	2	2	2	2	2	2	2
2	1	2	2	2	2	2	2	2	2
	1								
2	1	2	2	2	2	2	2	2	2
	2								
	1								
2	1	2	2	2	2	2	2	2	2
2	1	2	2	2	2	2	1	2	2
	1								
	2								
	1					1			
2	1	2	2	2	2	2	2	2	2
	1								
O NASAL	2								
	2								
2	1	2	2	2	2	2	2	2	2
2	1	2	2	2	2	2	1	2	2
	1								
	2								

		1								
		2								
		1								
		2								
	2	1	2	2	2	2	2	2	2	2
		2								
		2								
	9	1	9	9	9	9	1	9	9	9
		2								
	2	1	2	2	2	2	2	2	2	2
IRRITABILII		2								
	2	1	2	2	2	2	2	2	2	2
		2								
		2								
		2								
	2	1	1	2	2	2	2	1	2	2
	2	1	2	2	2	2	2	2	2	2
		2								
		2								
DOER PLEUI		1								
	2	1	2	2	2	2	2	1	2	2
	2	1	2	2	2	2	2	2	2	2
		1						1		
	2	1								
IIALIA		2								
	2	1	2	2	2	2	2	1	2	2
	2	1	1	2	2	2	2	2	2	2
		2								
		2								
	2	1	2	2	2	2	2	2	2	2
		2								
CICA		2								
		2								
AGITADA/LC		1								
		2								
XICA		2								
	2	1	2	2	2	2	2	1	2	2
CEFALEIA, C		2								
		2								
		2								
		2								
E HIPOREXI		1								
	2	1	2	2	2	2	2	2	2	2
		2								
		1								
		2								
ORPO		2								

IA,ASTENIA		2								
		1					1	1		
		2								
		1	1							
		2								
		2								
		2								
	2	1	2	2	2	2	2	2	2	2
	2	1	2	2	2	2	2	2	2	2
	2	1	2	2	2	2	2	2	2	2
		2								
	2	1					1			
		2								
		2								
		1								
		2								
CICA		2								
	2	1	2	2	2	2	2	2	2	2
		2								
R		2								
		2								
		2								
CORIZA, FRA		2								
	2	1	2	2	2	2	2	2	2	2
	2	1	2	2	2	2	2	2	2	2
	2	1	2	2	2	2	2	2	2	2
MIALGIA		1								
		2								
	2	1	2	2	2	2	2	2	2	2
	2	1	1	2	2	2	2	1	2	2
CEFALEIA, C		1								
CEFALEIA, D		2								
CEFALEIA		2								
		2								
		2								
		1								
	2	1	2	2	2	2	2	2	2	2
STENIA		1								
	2	1	1	2	2	2	2	2	2	2
	2	1	2	2	2	2	2	2	2	2
		2								
	2	1	2	2	2	2	2	2	2	2
		2								
		2								
	2	1	2	2	2	2	2	2	2	2
		2								
	2	1	2	2	2	2	2	2	2	2

2	1	1	2	2	2	2	2	2	2
	2								
2	1	1	2	2	2	2	2	2	2
NAPETENCI.	2								
2	1	2	2	2	2	2	2	2	2
	1								1
	2								
	1								
2	1	2	2	2	2	2	1	2	2
	2								
	2								
2	1	2	2	2	2	2	2	2	2
2	1	2	2	2	2	2	2	2	2
CANSACO	2								
	2								
2	1	2	2	2	2	2	1	2	2
	1								
MPSIA, ELEV	1						1		
2	1	2	2	2	2	2	2	2	2
2	1	2	2	2	2	1	2	2	2
CORIZA, MI.	2								
	1						1		
	2								
	2								
2	1	2	2	2	2	2	2	2	2
	2								
2	1	2	2	2	2	1	2	2	2
	2								
2	1	2	2	2	2	1	2	2	2
	2								
, CALAFRIO	2								
	1								
	1								
	2								
	2								
	2								
	2								
CICA	2								
ICA	2								
REPIRATOF	2								
	2								
	2								
NAPETENCIA	1	1							
	2								
2	1	2	2	2	2	1	2	2	2
2	1	2	2	2	2	2	1	2	2
	2								

XPECTORA	1					1			
	1								
2	1	2	2	2	2	2	2	2	2
2	1	1	2	2	2	1	1	2	2
	1								
	2								
	2								
	1								
2	1	2	2	2	2	2	2	2	2
	2								
TAQUIPNEI	2								
2	1	2	2	2	2	2	2	2	2
	2								
	1				1				
ONGESTAC	1					1			
2	1	2	2	2	2	2	2	2	2
2	1	2	2	2	2	1	2	2	2
2	1	1	2	2	2	2	1	2	2
	1	1							
	2								
	2								
BECA	1								
2	1	2	2	2	2	2	2	2	2
	2								
9	1	9	9	9	9	9	9	9	9
	1								
	2								
DOR TORAX	2								
2	1	2	1	2	2	2	2	2	2
2	1	2	2	2	2	2	1	2	2
2	1						1		
2	1	2	2	2	2	2	2	2	2
2	1	2	2	2	2	2	2	2	2
	1								
	2								
2	1	2	2	2	2	2	2	2	2
2	1	2	2	2	2	2	2	2	2
ONGESTAO	2								
2	1	2	2	2	2	2	1	2	2
2	1	1	2	2	2	2	2	2	2
MIALGIA, C	2								
	1								
	2								
	2								
2	1	2	2	2	2	2	2	2	2
	2								
	2								

	2	1	2	2	2	2	2	1	2	2
		1								
		2								
		2								
		2								
APETITE		2								
	2	1	2	2	2	2	2	1	2	2
	2	1	2	2	2	2		2	2	2
		2								
		2								
		2								
		2								
ALGIA		1								
	2	1	2	2	2	2	2	2	2	1
	2	1	1	2	2	2	2	2	2	2
	2	1	2	2	2	2	2	2	2	2
		2								
		1								
	2	1	2	2	1	2	2	2	2	2
PIRAT.		1				1				
		2								
ALGIA		2								
		2								
	2	1	2	2	2	2	2	2	2	2
		2								
		2								
		2								
		2								
		2								
	2	1	2	2	2	2	2	2	2	2
	2	1	2	2	2	2	2	2	2	2
		2								
DE PESO N		1								
	2	1	2	2	2	2	1	2	2	2
	2	1	2	2	2	2	2	2	2	2
		1					1			
		1								
		1								
		2								
	2	1	2	2	2	2	2	2	2	2
		2								
MIALGIA		2								
	2	1	2	2	2	2	2	1	2	2
	2	1	2	2	2	2	2	2	2	2
		1								
		1								
AO,MIALGI		1								

		2								
		2								
	9	1	9	9	9	9	9	9	9	9
	2	1	2	2	2	2	2	2	2	2
	2	1	2	2	2	2	2	2	2	2
		2								
CICA		2								
	2	1	1	2	2	2	2	2	2	2
	2	1	2	2	2	2	1	2	2	2
		2								
IALGIA E CE		2								
		1						1		
		2								
		2								
		2								
	2	1	2	2	2	2	1	2	2	2
		1								
	2	1	2	2	2	2	2	2	2	2
		2								
		1								
A		1								
	2	1	1	2	2	2	2	1	2	2
		2								
ADINAMIA,		2								
		2								
		1	1					1		
		2								
OT) HIST. IN		2								
	2	1	2	2	2	2	2	2	2	2
	2	1	2	2	2	2	2	2	2	2
	2	1	2	2	2	2	2	2	2	2
		2								
AIS		2								
		2								
	2	1	2	2	2	2	2	2	2	2
	2	1	2	2	2	2	2	2	2	2
		2								
		2								
		1	1							
		2								
CORIZA,COI		1								
AIS		2								
	2	1	2	2	2	2	2	2	2	2
	2	1	2	2	2	2	2	2	2	2
	2	1	2	2	2	2	2	2	2	2
		2								
		2								

		2								
		2								
		2								
	2	1	2	2	2	2	2	2	2	2
		2								
	2	1	2	2	2	2	2	2	2	2
DOR AURIC		2								
AL EM 10/5,		1	1					1		
		2								
		2								
		2								
		2								
		1	1					1		
RTRALGIA		1								
		2								
		1	1					1		
		2								
		2								
		1								
	2	1	2	2	2	2	2	1	2	2
		2								
	2	1	2	2	2	2	2	2	1	2
		2								
	2	1	2	2	2	2	2	1	2	2
	2	1	2	2	2	2	1	2	2	2
		2								
MIALGIA		2								
		2								
IALGIA		1								
		2								
	2	1	2	2	2	2	2	2	2	2
		1								
		1								
		2								
		2								
		2								
	2	1	1	2	2	2	2	2	1	2
		2								
CEFALEIA E		2								
DNAMIA		2								
		2								
MIALGIA, A		1	1							
	2	1	2	2	2	2	2	2	2	2
		2								
MIALGIA, CE		2								
		2								
MIALGIA		1	1					1		

		2								
		2								
CIA		2								
DIA		2								
	2	1	2	2	2	2	2	2	2	2
OR TORACI		1	1							
		2								
	2	1	2	2	2	2	2	2	2	2
EFALEIA		2								
		2								
	2	1	1	2	2	2	2	2	2	2
		2								
		2								
	2	1	2	2	2	2	2	2	2	2
MIALGIA		2								
	2	1	2	2	2	2	2	2	2	2
	2	1	2	2	2	2	2	2	2	2
		2								
	2	1	2	2	2	2	2	2	2	2
IALGIA, CEF		2								
GRIPAIS, C		2								
		2								
		2								
AIS		2								
		1								
	2	1	2	2	2	2	2	1	2	2
		2								
	2	1								
	2	1	1	2	2	2	2	2	2	2
	2	1	2	2	2	2	1	2	2	2
		1								
CEFALEIA		2								
ANOSMIA		2								
		2								
	2	1	2	2	2	2	2	1	2	2
	2	1	1	2	2	2	2	2	2	2
		2								
IETABOLIC		1								
		2								
	2	1	1	2	2	2	2	2	2	2
	2	1	2	2	2	2	2	2	2	2
	2	1	2	2	2	2	2	2	2	2
	2	1	1	2	2	2	2	1	2	2
		2								
		2								
A INAPETEI		2								
	2	1	2	2	2	2	2	2	2	2

		1								
	2	1								
	2	1	2	2	2	2	2	2	2	2
E CORIZA		2								
		2								
		2								
	2	1	2	2	2	2	2	2	2	2
ORPO		2								
		1					1			
ORIZA,ASTE		2								
		1								
		1								
		1								
		2								
		2								
		1					1			
		2								
		2								
		2								
MIALGIA E (		1								
DINAMIA		1						1		
		1								
CANSACO, A		1								
	2	1	2	2	2	2	2	2	2	2
CICA, EPIGA		2								
		1								
		2								
		2								
		2								
		1								
		1								
		2								
DORSALGIA		2								
		2								
		1	1							
	2	1	1	2	2	2	2	2	2	2
	2	1	2	2	2	2	2	2	2	2
		2								
		2								
		2								
		2								
		2								
		2								
	2	1	2	2	2	2	2	2	2	2
	2	1	2	2	2	2	2	2	2	2
		2								
	2	1	2	2	2	2	2	2	2	2

	2	1	2	2	2	2	2	2	2	2
		1	1							
	2	1	2	2	2	2	2	2	1	2
		2								
	2	1	2	2	2	2	2	2	2	2
	2	1	2	2	2	2	2	2	2	2
CALAFRIOS,		2								
		1								
		2								
		2								
		2								
ONG. NAS		1								
CIA		2								
STENIA		2								
	2	1								
		2								
NEIA, MIAL		1								
		1								
		2								
	2	1	2	2	2	2	2	2	2	2
		2								
		1								
		1								
	2	1	2	2	2	2	1	2	2	2
CIA/CEFALE		2								
	2	1	1	2	2	2	2	1	2	2
OGESTAO N.		1								
	2	1	1	2	2	2	2	2	2	2
	2	1								
	2	1	2	2	2	2	2	1	2	2
		2								
		2								
		2								
		1								
ADINAMIA,		2								
		2								
		2								
INCOPE		2								
		2								
	2	1	2	2	2	2	2	2	2	2
		2								
DIA, QUED		1								
	2	1	2	2	2	2	2	2	2	2
		1								
NAUSEAS, T.		2								
		2								
	2	1	2	2	2	2	2	2	2	2

		2								
		2								
	2	1	2	2	2	2	2	1	2	2
	2	1	2	2	2	2	2	2	2	2
MIALGIA, C.		2								
		2								
	2	1	2	2	2	2	2	2	2	2
	2	1	2	2	2	2	2	2	2	2
CORIZA		2								
	2	1	2	2	2	2	2	2	2	2
		2								
		2								
CICA E CEF		2								
PIRATORIA		2								
	2	1	2	2	2	2	2	2	2	2
R		2								
		2								
	2	1	2	2	2	2	2	2	2	2
		2								
	2	1	2	2	2	2	1	1	2	2
		2								
IA, LOMAB.		2								
	2	1	2	2	2	2	2	2	2	2
	9	1	9	9	9	9	9	9	9	9
		1								
	2	1	2	2	2	2	2	2	2	2
CICA, INFEC		1	1							
BECA		2								
	2	1	2	2	2	2	2	2	2	2
ARTRALGIA		2								
	2	1	2	2	2	2	2	2	2	2
		2								
		2								
		2								
	2	1	2	2	2	2	2	2	2	2
		2								
	9	1	9	9	2	9	9	9	9	9
EFALEIA CO		2								
		2								
	2	1					1	1		
		2								
		1								
	2	1	2	2	2	2	2	2	2	2
	2	1	2	2	2	2	2	2	2	2
	2	1	2	2	2	2	2	1	2	2
		1								
	2	1	2	2	2	2	2	2	2	2

IIALGIA		2								
		2								
	2	1	2	2	2	2	2	2	2	2
		2								
	2	1	2	2	2	2	2	2	2	2
MIALGIA		2								
	2	1	2	2	2	2	2	2	2	2
	2	1	2	2	2	2	2	2	2	2
		2								
		2								
	2	1	2	2	2	2	1	2	2	2
		2								
		1								
	2	1	2	2	2	2	2	2	2	2
		1								
		2								
	2	1	2	2	2	2	1	2	2	2
		1								
	2	1	2	2	2	2	2	2		2
		2								
		2								
IA		2								
		2								
		1	1							
		2								
		1								
		2								
CICA		2								
		1	1					1		1
	2	1	2	2	2	2	2	2	2	2
	2	1	2	2	2	2	2	1	2	2
		2								
		2								
AQUICARDI		2								
		1	1							
	2	1	2	2	2	2	2	2	2	2
		2								
		2								
		2								
		2								
		2								
		2								
		2								
	2	1	1	2	2	2	2	2	2	2
	2	1	2	2	2	2	2	2	2	2
	2	1	1	2	2	2	2	2	2	2
O NASAL +		1								

		2								
		1								
		2								
		2								
		1	1							
		2								
DOR TORAC		1	1							
		1	1							
		1						1		
CEFALEIA		2								
	2	1	2	2	2	2	2	1	2	2
		2								
		2								
		2								
	2	1	2	2	2	2	2	2	2	2
IIPOREXIA E		2								
		2								
		2								
		1						1		
		2								
	2	1	2	2	2	2	2	2	2	2
	2	1	2	2	2	2	2	2	2	1
		2								
		2								
	2	1	2	2	2	2	2	2	2	2
		2								
		2								
CICA, ADIN		2								
	2	1	2	2	2	2	2	2	2	2
		2								
A		2								
	2	1	2	2	2	2	2	2	2	2
	2	1	2	2	2	2	2	2	2	2
		1								
	2	1	2	2	2	2	2	1	2	2
		2								
	2	1	2	2	2	2	1	2	2	2
		2								
		2								
		2								
	2	1	2	2	2	2	2	2	2	2
	2	1	1	2	2	2	2	2	2	2
		2								
		2								
		1					1			
		2								
	2	1	2	2	2	2	2	2	2	2

		2								
	2	1	2	2	2	2	1	2	2	2
		2								
	2	1	2	2	2	2	2	1	2	2
	2	1	1	2	2	2	2	2	2	2
	2	1	9	9	9	9	9	1	1	9
		2								
		2								
		2								
		2								
		2								
		2								
		1								
	2	1	2	2	2	2	2	1	2	2
		2								
		1								
CALAFRIOS		2								
	2	1	2	2	2	2	2	2	2	2
		2								
		1								
		2								
		1					1			
,CORIZA,CE		2								
		2								
		2								
		2								
	2	1	2	2	2	2	2	1	2	2
BSTRUCAO		2								
ORPO		1								
MIALGIA		2								
	2	1	1	2	2	2	2	1	2	2
	2	1	2	2	2	2	2	2	2	2
RAQUEZA		1								
		1								
	2	1						1		
	2	1	2	2	2		2	2	2	2
		2								
MIALGIA		2								
EFALEIA		2								
CEFALEIA		2								
MIALGIA,		2								
	2	1	1	2	2	2	2	1	2	2
	2	1	2	2	2	2	2	2	2	2
		2								
		2								
		2								
		1								

FALEIA		2								
		1								
	2	1	2	2	2	2	2	1	2	2
	2	1	2	2	2	2	2	2	2	2
		2								
	2	1	2	1	2	2	2	2	2	2
	2	1	2	2	2	2	2	2	2	2
		2								
	2	1	1	2	2	2	2	2	2	2
		2								
	2	1	2	2	2	2	1	2	2	2
ASTENIA,CO		2								
		2								
	2	1	2	2	2	2	2	2	2	2
	2	1	2	2	2	2	2	2	2	2
COM SHEG		2								
	2	1	1	2	2	2	2	1	2	2
		2								
	2	1	2	2	2	2	2	2	2	2
ONGESTAO		2								
		1								
	2	1	2	2	2	2	2	2	2	2
	2	1	2	2	2	2	2	2	2	2
	2	1						1		
DOR TORAC		2								
		2								
	2	1	1	2	2	2	2	2	2	2
		1						1		
		1								
		1								
	2	1	2	2	2	2	2	1	2	2
AO		2								
DOR TORAC		2								
		2								
		1								
	2	1	2	2	2	2	2	2	2	2
		2								
	2	1	2	2	2	2	2	2	2	2
		1								
		2								
		2								
		2								
		2								
		2								
		2								
		2								
		2								

	2	1	2	2	2	2	2	2	2	2
	2	1	1	2	2	2		2	2	2
		2								
		1								
		2								
	2	1	2	2	2	2	2	2	2	2
		2								
	2	1	2	2	2	2	2	2	1	2
	2	1	2	2	2	2	2	2	2	2
CICA		2								
		2								
	2	1	1	2	2	2	2	2	2	2
		1								
	2	1	1	2	2	2	2	9	2	2
		1	1				1			
	2	1	2	2	2	2	2	1	2	2
MIALGIA		1	1							
		2								
		2								
DINAMIA		2								
		2								
		2								
	2	1	2	2	2	2	2	2	2	2
	2	1	2	2	2	2	2	1	2	2
	2	1	2	2	2	2	2	2	2	2
		2								
IS/ODINOF		2								
CIA		1								
	2	1	2	2	2	2	1	2	2	2
		1					1			
	2	1	1	2	2	2	2	2	2	2
		2								
		2								
	2	1	1	2	2	2	2	2	2	2
		2								
		1								
		2								
, ODINAFA		1					1			
	2	1	1	2	2	2	2	2	2	2
	2	1	2	2	2	2	2	2	2	2
		2								
		2								
	2	1	2	2	2	2	2	2	2	2
		1								
		2								
	2	1	2	2	2	2	2	2	2	2
		2								

		1								
	2	1	2	2	2	2	2	2	2	2
		2								
		1								
		2								
ASTENIA, C(		2								
[illegible]		2								
		1								
	2	1	2	2	2	2	2	2	2	2
		2								
OTALGIA, M		2								
		1						1		
	2	1	2	2	2	2	2	2	2	2
	2	1	2	2	2	2	2	2	2	2
		1								
	2	1	1	2	2	2	2	1	2	2
		2								
		1								
		2								
/INAPETEN		2								
AUSEAS		1								
	2	1	2	2	2	2	2	2	2	2
		2								
		1								
		2								
		1								
	2	1	1	2	2	2	2	2	2	2
EFALEIA		1								
MIALGIA		1	1					1		
		1								
		1						1		
		1	1							
	2	1	2	2	2	2	2	2	2	2
		2								
	2	1	2	2	2	2	2	2	1	2
	2	1	2	2	2	2	2	2	2	2
	2	1	2	2	2	2	2	2	2	2
		2								
		2								
	2	1	2	2	2	2	1	2	2	2
	2	1	2	2	2	2	2	2	2	2
ANSACO		2								
		2								
MIALGIA		1								
		1								
	2	1	2	2	2	2	2	1	2	2
	2	1	2	2	2	2	2	2	2	2

		2								
		2								
MIALGIA,OI		2								
		2								
	2	1	2	2	2	2	2	1	2	2
		2								
		1							1	
		2								
		1					1	1		
MIALGIA		2								
		2								
		1						1		
	2	1	2	2	2	2	2	2	2	2
		1								
AQUIPNEIA		2								
O NASAL		1								
		2								
AR		2								
		2								
	2	1	1	2	2	2	2	2	2	2
		2								
	2	1	2	2	2	2	2	2	2	2
, PROSTRA		2								
	2	1	2	2	2	2	2	2	2	2
		1						1		
		1					1			
	2	1	2	2	2	2	2	2	2	2
		2								
		2								
		2								
		1					1			
		2								
		1								
		2								
	2	1	2	2	2	2	2	2	2	2
DOR LOMB		1								
		2								
	2	1	2	2	2	2	2	2	2	2
		2								
		2								
		1								
CIA		1								
	2	1	2	2	2	2	2	2	2	2
		1								
		2								
		2								
, ASTENIA		2								

		1								
	2	1	2	2	2	2	2	2	2	2
		1								
	2	1	1	2	2	2	2	2	2	2
	2	1	2	2	2	2	2	2	2	2
		2								
		2								
		1						1		
		1								
	2	1	2	2	2	2	2	2	2	2
R GERAL		2								
		2								
		2								
COMPROM		2								
TAQUICARD		1								
		2								
		1								
	2	1	2	2	2	2	1	2	2	2
		2								
		2								
		2								
		1						1		
		1								
DINOFAGIA		2								
	2	1	2	2	2	2	2	2	2	2
		2								
CEFALEIA		2								
	2	1	2	2	2	2	1	2	2	2
		2								
		2								
IALGIA CEF		2								
		2								
	2	1	1	2	2	2	2	2	2	2
		2								
		2								
	2	1	2	2	2	2	2	2	2	2
		1								
		1								
	2	1	1	2	2	2	2	2	2	2
DOR NO CO		2								
	2	1	2	2	2	2	2	2	2	2
		2								
		2								
	2	1								
		1								
		2								
		1					1			

	2	1	2	2	2	2	2	1	2	2
		1								
		2								
CEFALEIA		2								
AIS		2								
	2	1	1	2	2	2	2	2	2	2
	2	1	1	2	2	2	2	2	2	2
		2								
		2								
	2	1	2	2	2	2	2	2	2	2
IALGIA, CEF		2								
	2	1	2	2	2	2	1	1	2	2
		1								
		2								
		2								
		2								
		1					1			
	2	1	2	2	2	2	2	1	2	2
		2								
		1								
		2								
	2	1	2	2	2	2	2	2	2	2
	2	1	2	2	2	2	2	1	2	2
GRIPAIS		2								
	2	1	2	2	2	2	2	2	2	2
CORIZA		1						1		
		2								
		2								
		1						1		
		2								
		2								
		2								
		1	1							
		2								
		2								
	2	1	2	2	2	2	2	2	2	2
		2								
		2								
STENIA		2								
		2								
		2								
		2								
	2	1	1	2	2	2	2	2	2	2
		2								
		2								
		2								
NAUSEA		1								

		1								
, CALAFRIO		2								
TAQUICARD		2								
		1								
CICA		2								
		2								
		1								
		2								
OSTAS		2								
MIALGIA, IN		2								
		1	1					1		
	2	1	1	2	2	2	2	2	2	2
	2	1	2	2	2	2	1	2	2	2
	2	1	1	2	2	2	2	2	2	2
	2	1	2	2	2	2	2	2	2	2
		2								
		2								
		2								
		2								
		1								
		2								
	2	1	2	2	2	2	2	2	2	2
	2	1	2	2	2	2	2	2	2	2
		2								
		2								
AIS		2								
	2	1	2				1	2	2	2
		1								
E MIALGIA		2								
		1								
	2	1	2	2	2	2	2	2	2	2
		2								
	2	1	2	2	2	2	2	2	2	2
		2								
		2								
	2	1	2	2	2	2	2	2	2	2
		2								
ALGIA		2								
		2								
	2	1	2	2	2	2	2	2	2	2
		2								
	2	1	2	2	2	2	2	2	2	2
		1								
	2	1	2	2	2	2	2	2	2	2
		2								
		2								
		1								

VOMITOS		2								
DOR NO CO		2								
	2	1	2	2	2	2	2	2	2	2
	2	1	2	2	2	1	2	2	2	2
		2								
	2	1	2	2	2	2	2	2	2	2
		2								
	2	1	2	2	2	2	2	2	2	2
	2	1	2	2	2	2	2	2	2	2
CEFALEIA, C		2								
		2								
		1								
	2	1	1	2	2	2	2	2	2	2
MIALGIA		2								
		2								
		2								
	2	1	2	2	2	2	2	1	2	2
		2								
		2								
CICA, INAPE		1								
		2								
	2	1	2	2	2	2	2	1	2	2
	2	1	2	2	2	2	2	2	2	2
DORSALGIA		2								
		1					1			
	2	1	2	2	2	2	2	2	2	2
		2								
		2								
	2	1	1	2	2	2	2	2	2	2
		2								
		2								
		1					1			
	2	1	2	2	2	2	2	2	2	2
		2								
		2								
		2								
		2								
	2	1	2	2	2	2	2	1	2	2
		2								
		1								
	2	1	2	2	2	2	2	2	2	2
	2	1	2	2	2	2	2	2	2	2
MIALGIA, C		1								
	2	1	2	1	2	2	2	2	2	2
	2	1	2	2	2	2	2	2	2	2
		1								
		2								

		1								
		2								
		2								
		2								
	2	1	2	2	2	2	1	2	2	1
	2	1	2	2	2	2	2	2	2	2
	2	1	2	2	2	2	2	2	2	2
	2	1	1	2	2	2	2	2	2	2
CICA		2								
	2	1	2	2	2	2	2	2	2	2
	2	1	2	2	2	2	2	2	2	2
	2	1	2	2	2	2	2	1	2	2
		2								
		2								
		2								
	2	1	2	2	2	2	2	2	2	2
	2	1	2	2	2	2	2	1	2	2
		1								
	2	1	2	2	2	2	2	1	2	2
		2								
	2	1	2	2	2	2	2	2	2	2
	2	1	1	2	2	2	2	1	2	2
		2								
		2								
		2								
	2	1	2	2	2	2	2	1	2	1
	2	1	2	2	2	2	2	2	2	2
ANSACO		2								
NAUSEA, CE		1								
		2								
		1								
	2	1	2	2	2	2	2	2	2	2
		2								
		1						1		
	2	1	1	2	2	2	2	2	2	2
	2	1	2	2	2	2	2	2	1	2
		1								
	2	1	2	2	2	2	2	2	2	2
		1								
	2	1	2	2	2	2	2	2	2	9
		1								
		1								
	2	1	2	2	2	2	2	2	2	2
		2								
		2								
		2								
	2	1	2	2	2	2	2	2	2	2

	2	1	2	2	2	2	2	1	2	2
CICA		2								
	2	1	1	2	2	2	2	2	2	2
ASTENIA		2								
	2	1	2	2	2	2	2	1	2	2
	2	1	1	2	2	2	2	2	2	2
	2	1	2	2	2	2	2	2	2	2
	2	1	2	2	2	2	2	2	2	2
		2								
		2								
		2								
	2	1	2	2	2	2	1	2	2	2
CIA, ASTENI		2								
		1								
	2	1	2	2	2	2	2	2	2	2
		2								
	2	1	2	2	2	2	2	2	2	2
		2								
	2	1	2	1	2	2	2	2	2	2
	2	1	2	2	2	2	2	2	2	2
		2								
	2	1	1	1	2	2	2	1	2	2
CICA,MAL E		2								
		2								
	9	1	2	2	2	2	2	2	2	2
R GERAL, FR		2								
	2	1	2	2	2	2	2	2	2	2
ALGIA		1								
		2								
	2	1	2	2	2	2	2	2	2	2
		2								
	2	1	2	2	2	2	2	2	2	2
	2	1						1		
CEFALEIA, A		2								
	2	1	2	2	2	2	2	2	2	2
		2								
CICA		2								
		1								
	2	1	1	2	2	2	2	2	2	2
AO, CONFU		1						1		
	2	1	2	2	2	2	2	2	2	2
	2	1	2	2	2	2	2	2	2	2
		1						1		
	2	1	2	2	2	2	2	2	2	2
CANSACO		1	2	2	2	2	2	2	2	2
		1					1	1		
MIALGIA		2								

2	1	1	2	2	2	2	2	2	2
	2								
	1								
2	1	2	2	2	2	2	2	2	2
2	1	2	2	2	2	2	2	2	2
2	1	2	2	2	2	2	1	2	2
MIALGIA, PI	2								
, E DOR NO	2								
2	1	2	2	2	2	2	2	2	2
	2								
	1								
2	1	2	2	2	2	1	1	2	2
2	1	2	2	2	2	2	2	2	2
2	1	2	2	2	2	2	2	2	2
O NASAL	1						1		
	1								
	1						1		
	1								
2	1	2	2	2	2	1	2	2	2
2	1	2	2	2	2	2	2	2	2
	2								
	2								
2	1	2	2	2	2	2	2	2	2
2	1	2	2	2	2	2	2	2	2
	2								
	1								
	2								
2	1	2	2	2	2	2	1	2	2
	2								
FRAQUEZA	2								
	2								
2	1	2	2	2	2	2	2	2	2
2	1	2	2	2	2	2		2	2
	2								
2	1	1	2	2	2	2	2	2	2
	2								
	2								
2	1	2	2	2	2	2	2	2	2
2	1	2	2	2	2	2	2	2	2
ODINOFAGI	2								
2	1	2	2	2	2	2	2	2	2
	2								
	2								
	2								
2	1	2	2	2	2	2	1	2	2
2	1	2	2	2	2	2	2	2	2
	2								

		2								
	2	1	2	2	2	2	2	2	2	2
	2	1	2	2	2	2	2	2	2	2
		2								
		1						1		
	2	1	2	2	2	2	2	2	2	2
		2								
, MIALGIA		1								
	2	1	2	2	2	2	2	2	2	2
		2								
		2								
		1	1					1		
CEFALEIA		2								
	2	1	2	2	2	2	2	1	2	2
	2	1	2	2	2	2	2	2	2	2
		2								
		2								
		2								
		2								
	2	1	2	2	2	2	2	2	2	2
		2								
		2								
		2								
		1								
	2	1	2	2	2	2	2	2	2	2
		2								
		2								
		2								
		2								
		2								
	2	1	2	2	2	2	2	2	2	2
	2	1	2	2	2	2	2	2	2	2
		1					1	1		
ALAFRIOS,M		1						1		
	2	1						1		
	2	1	1	2	2	2	2	2	2	2
EFALEIA		1					1			
CONGESTAC		1					1			
		2								
MAL ESTAR		1								
	2	1	2	2	2	2	2	2	2	2
		2								
	2	1	2	2	2	2		2	2	2
		2								
		1								
		2								
IA		2								

2	1	2	2	2	2	2	2	2	2
2	1	1	2	2	2	2	1	2	2
2	1	2	2	2	2	2	2	2	2
	1								
	2								
2	1	2	2	2	2	2	2	2	2
	2								
	1	1							
ORAX	2								
LGIA	2								
MIALGIA, C	2								
NAPETENCI	1								
CICA E TQU	2								
2	1	2	2	2	2	2	2	2	2
2	1	2	2	2	2	2	2	2	2
R, OBSTRUC	2								
	2								
	1	1					1		
	2								
2	1	2	2	2	2	1	2	2	2
ASTENIA	2								
	1								
2	1	2	2	2	2	2	2	2	2
	2								
	1								
MIALGIA	2								
2	1	2	2	2	2	2	2	2	2
	2								
IALGIA	1								
	2								
	2								
	2								
ATICO	2								
	2								
ALGIA CEFA	2								
	2								
	2								
	2								
	1								
	2								
	1	1							
2	1	2	2	2	2	2	2	2	2
	2								
	2								
2	1	2	1	2	2	1	1	2	2
2	1	2	2	2	2	2	2	2	2
	2								

	1	1	2	2	2	9	1	2	9
	2								
	1	1							
2	1	1	2	2	2	2	1	2	2
	2								
	1								
2	1	2	2	2	2	1	2	2	2
	2								
	1	1							
	1								
OR CORPO	2								
	1								
DIA	2								
	2								
CICA E BAIX	1	1							
	2								
2	1	1	2	2	2	1	1	2	2
	2								
2	1	2	2	2	2	2	1	2	2
	1								
2	1	2	2	2	2	2	2	2	2
	1								
	2								
	1	1					1		
	2								
CIA, ASTENI	1	1					1		
	2								
	2								
	2								
2	1	2	2	2	2	2	1	2	2
	2								
2	1	2	2	2	2	2	2	2	2
CICA, FALTA	2								
2	1	2	2	2	2	1	1	2	2
2	1	2	2	2	2	2	2	2	2
2	1	2	2	2	2	2	2	2	2
2	1	2	2	2	2	2	1	2	2
2	1	2	2	2	2	2	2	2	2
2	1	2	2	2	2	2	1	2	2
2	1	2	2	2	2	2	2	2	2
CAO, MIALG	2								
	2								
2	1	2	2	2	2	2	2	2	2
	2								
MIALGIA	2								
	1								
	2								

2	1	2	2	2	2	2	2	2	2
STENIA NAI	2								
	2								
	2								
	2								
DIA E HIPOT	1								
	1							1	
	2								
	1								
E	2								
2	1	2	2	2	2	2	1	2	2
2	1	2	2	2	2	2	1	2	2
	1								
	2								
DOR TORAX	2								
	2								
	2								
ORPO, CEFA	2								
2	1	2	2	2	2	2	1	2	2
	1						1		
ADINAMIA.	1								
	1								
2	1	2	2	2	2	1	2	2	2
2	1	2	2	2	2	2	2	2	2
	2								
EITO, CORIZ	2								
	2								
	2								
2	1	1	2	2	2	2	2	2	2
2	1	2	2	2	2	2	1	2	2
HIPOREXIA	1								
CORIZA	2								
	2								
	2								
MIALGIA,AR	1								
IALGIA , CC	1						1		
	2								
MIALGIA	2								
	2								
	2								
	2								
	1						1		
2	1	2	2	2	2	2	2	2	2
	1								
	2								
	2								
	2								

		2								
		2								
DOR LOMB		2								
	2	1	2	2	2	2	2	2	2	2
		2								
	2	1	1	2	2	2	2	1	2	2
CORIZA		2								
CORIZA, CEF		1								
	2	1	1	2	2	2	2	1	2	2
	2	1	2	2	2	2	2	2	2	2
		2								
		2								
	2	1	2	2	2	2	2	2	2	
		2								
	2	1	1	2	2	2	2	1	2	2
		2								
	2	1	1	2	2	2	2	2	2	2
		2								
		2								
		1								
		2								
		2								
IS/MIALGI		2								
		2								
,FRAQUEZA		2								
	2	1	2	2	2	2	2	2	2	2
		2								
		1								
	2	1	2	2	2	2	2	1	2	2
		1								
		2								
	2	1	2	2	2	2	2	2	2	2
		2								
		2								
		1								
A		1								
		2								
	2	1	2	2	2	2	2	2	2	2
		2								
	2	1	2	2	2	2	1	2	2	2
		2								
		1								
	2	1	2	2	2	2	2	2	2	2
	2	1	1	2	2	2	2	2	2	2
		2								
		2								
	9	1	9	9	9	9	9	9	9	9

		2								
AR, GARGA		2								
	2	1								
		2								
TAQUICARD		2								
	2	1	2	2	2	2	2	2	2	2
	2	1	2	2	2	2	2	2	2	2
ANSACO		1								
		2								
		2								
	9	1	2	2	2	2	2	2	2	2
	2	1	2	2	2	2	2	2	2	2
		1								
		1								
	2	1	1	2	2	2	2	2	2	2
	2	1	2	2	2	2	2	2	2	2
	2	1						1		
		2								
	2	1	2	2	2	2	1	2	2	2
AIS		2								
		1								
	2	1	2	2	2	2	2	2	2	2
AIS/ODINOF		2								
	2	1	2	2	2	2	2	2	2	2
	2	1	2	2	2	2	2	2	2	2
	2	1	2	2	2	2	2	2	2	2
		1								
	2	1	2	2	2	2	2	1	2	2
TAQUICARD		2								
		2								
	2	1	2	2	2	2	2	2	2	2
	2	1	1	2	2	2	2	2	2	2
	2	1	2	1	2	2	2	2	2	2
	2	1								
	2	1	2	2	2	2	2	2	2	2
		2								
AIS		2								
		2								
	2	1	2	2	2	2	2	2	2	2
CONGESTAC		2								
	2	1	2	2	2	2	2	2	2	2
	2	1	2	2	2	2	2	2	2	2
		1	2	2	2	2	2	1	2	2
		1								1
		2								
		2								
	2	1	2	2	2	2	2	2	2	2

		1								
		1								
		1								
		2								
O NASAL		1								
		2								
		2								
	2	1	2	2	2	2	2	2	2	2
		2								
	2	1	2	2	2	2	2	2	2	2
BECA, COR		2								
		2								
	2	1	2	2	2	2	2	2	2	2
	2	1	2	2	2	2	2	2	2	2
	2	1	2	2	2	2	2	2	2	2
		2								
	2	1	2	2	2	2	2	2	2	2
		2								
	2	1	2	2	2	2	2	2	2	2
DINOFAGI		2								
		2								
	2	1	2	2	2	2	2	2	2	2
		2								
	2	1	2	2	2	2	2	2	2	2
	2	1	2	2	2	2	2	2	2	2
	2	1	2	1	2	2	2	2	2	2
OSTAS E SC		2								
		1								
EFALEIA		1	1							
STENIA, TA		1								
		2								
	2	1	2	2	2	2	2	1	2	2
	2	1	2	2	2	2	1	2	2	2
		2								
		2								
EFALEIA		1	1					1		
		2								
		2								
		1								
STENIA		2								
		2								
		1								
		2								
	2	1	2	2	2	2	2	2	2	2
	2	1	2	2	2	2	2	2	2	2
A		1	1							
	2	1	2	2	2	2	2	2	2	2

2	1	2	2	2	2	2	2	2	2
2	1	2	2	2	2	2	1	2	2
	2								
	2								
ENTO VAS -	2								
	2								
	2								
	2								
	2								
	2								
NAUSEAS	2								
	1						1		
	1								
NAPETENCI	2								
CIA	1						1		
	2								
2	1	2	2	2	2	2	2	2	2
	1								
2	1	2	2	2	2	2	2	2	2
	2								
	2								
	1								
2	1	2	2	2	2	1	2	2	2
2	1	1	2	2	2	2	2	2	2
2	1	2	2	2	2	2	2	2	2
2	1	2	2	2	2	2	2	2	2
2	1	2	2	2	2	2	2	2	2
	2								
2	1	2	2	2	2	2	2	2	2
	1								
	2								
IA	1								
2	1	2	2	2	2	2	2	2	2
CANSACO	2								
	2								
2	1	2	2	2	2	2	1	2	2
	2								
2	1	2	2	2	9	2	2	2	2
	1								
	2								
2	1	2	2	2	2	2	1	2	2
	1								
	1						1		
R	2								
	2								
	2								
	1	1							

		2								
		1								
		1								
EITO		2								
	2	1	2	2	2	2	2	1	2	2
		2								
DOR LOMBA		2								
		1								
IPOREXEMI		1								
ENTO NASA		2								
		2								
		1								
		1						1		
CORIZA		2								
		2								
MIALGIA		2								
ESTADO GI		1								
		2								
		2								
	2	1	2	2	2	2	2	2	2	2
CORIZA, OTI		2								
	2	1	2	2	2	2	2	2	2	2
	2	1	2	2	2	2	2	2	2	2
		2								
CICA		2								
O NASAL		1								
	2	1	2	2	2	2	2	2	2	2
CORIZA		2								
	2	1	1	2	2	2	2	1	2	2
	2	1	2	2	2	2	1	2	2	2
	2	1					1			1
		2								
		2								
MIALGIA		2								
	2	1	2	2	2	2	2	2	2	2
		2								
	2	1	2	2	2	2	2	2	2	2
CANSACO, I		2								
	2	1	2	2	2	2	2	2	2	2
	2	1	2	2	2	2	2	2	2	2
ENTO VAGI		1								
R		1								
		2								
		2								
		2								
	2	1	1	2	2	2	2	2	2	2
CORIZA		1								

CRISE CONV	2								
	2								
	1								
	2								
	2								
	1								
	2								
2	1	2	2	2	2	2	1	2	2
	1								
	2								
	2								
IALGIA, AN	2								
2	1	2	2	2	2	2	2	2	2
LOMBALGIA	2								
	2								
DOR TORAC	2								
	2								
2	1	2	2	2	2	2	2	2	
	2								
	1								
	2								
	2								
	2								
	2								
2	1	1	2	2	2	2	2	2	2
	2								
2	1	2	2	2	2	2	2	2	2
	2								
	1								
2	1	1	2	2	2	2	2	2	2
	1								
2	1	2	2	2	2	2	2	2	2
2	1	2	2	2	2	2	2	2	2
2	1	2	2	2	2	2	2	2	2
2	1	2	2	2	2	2	2	2	2
2	1	2	1	2	2	2	2	2	2
	2								
	2								
	1								
	1								
	2								
2	1	2	2	2	2	2	2	2	2
2	1	2	2	2	2	2	2	2	2
9	1	9	9	9	9	9	9		9
	2								
	2								
IALGIA, CEF	1								

	1					1	1		
2	1	2	2	2	2	2	1	2	2
2	1	2	2	2	2	2	2	2	2
	2								
2	1	1	2	2	2	2	1	2	2
	2								
CORIZA, AS	2								
EFALEIA	2								
	1								
	2								
	1								
MAL ESTAR	1								
	2								
ENTRAL	2								
ASTENIA	1						1		
2	1	2	2	2	2	2	2	2	2
	1								
MIALGIA	2								
	2								
	2								
, MIALGIA	1								
	1								
RPO.	2								
	2								
	2								
2	1	2	2	2	2	1	2	2	2
	2								
2	1	2	2	2	2	2	2	2	2
	2								
	1								
	2								
	2								
	2								
DIA E TAQU	2								
	1							1	
	2								
	1								
	2								
2	1	2	2	2	2	2	2	2	2
2	1	2	2	2	2	2	2	2	2
, SINTOMA	2								
LIMIALGIA	2								
	2								
	1								
	2								
CORIZA E M	2								
	1								

	1						1		
2	1	1	2	2	2	2	2	2	2
	1					1			
	2								
	2								
	2								
	2								
	2								
	2								
2	1	2	2	2	2	2	2	2	2
2	1	2	2	2	2	2	2	2	2
	2								
AQUICARD	2								
	1								
	2								
BECA	2								
EFALEIA	2								
2	1	2	2	2	2	2	1	2	2
	1								
	1								
	2								
2	1	2	2	2	2	2	2	2	2
	2								
9	1	1	9	9	9	9	9	9	9
	2								
	1	1							
	2								
	2								
2	1	1	2	2	2	2	2	2	2
	1						1		
	2								
	2								
	1								
2	1	2	2	2	2	2	2	2	2
	2								
DOR LOMB	2								
2	1	2	2	2	2	2	2	2	2
	2								
2	1	2	2	2	2	2	2	2	2
GRIPAL PL	1						1		
	2								
EFALEIA	2								
2	1	1	2	2	2	2	2	2	2
	2								
	1								
	2								
2	1	2	2	2	2	2	2	2	2

	2	1	2	2	2	2	2	2	2	2
ANOSMIA		2								
	2	1	1	2	2	2	2	1	2	2
		1								
	2	1	2	2	2	2	2	2	2	2
		2								
ARDENCIA I		2								
IIPEREXIA		1	1							
	2	1	2	2	2	2	2	2	2	2
		2								
		2								
	2	1	2	2	2	2	2	2	2	2
		2								
		2								
	2	1	2	2	2	2	2	2	2	2
	2	1	2	2	2	2	1	1	2	2
		2								
		2								
		1					1			
	2	1	2	2	2	2	2	1	2	2
	2	1	1	2	2	2	2	2	2	2
	2	1	2	2	2	2	2	1	2	2
ORPO		2								
	2	1	2	2	2	2	2	2	2	2
ORIZA, MIA		2								
	2	1	2	2	2	2	2	2	2	2
		2								
EFALEIA		1								
		2								
		2								
	2	1	2	2	2	2	1	2	2	2
	2	1	2	2	2	2	2	2	2	2
		2								
		2								
		1								
	2	1	2	2	2	2	2	2	2	2
		2								
IAUSEAS		2								
	2	1	2	2	2	2	2	2	2	2
CIA		1								
	2	1	2	2	2	2	2	2	2	2
	2	1	2	2	2	2	2	2	2	2
	2	1	2	2	2	2	2	2	2	2
ANOREXIA, I		1								
NAPETENC		1								
		1								
EITO,CEFALE		2								

		2								
		2								
		2								
	2	1	2	2	2	2	2	2	2	2
		2								
		2								
		2								
		2								
ARTRALGIA		2								
		2								
		2								
		1								
POTENSAO		2								
	2	1	2	2	2	2	2	2	2	2
	2	1	2	2	2	2	2	2	2	2
	2	1	1	2	2	2	2	2	2	2
ESPIRATOR		2								
		2								
		2								
MIALGIA		1							1	
	2	1	2	2	2	2	2	2	2	2
	2	1	2	2	2	2	2	2	2	2
CORIZA		1	1					1		
		1	1							
		2								
	2	1	2	2	2	2	2	2	2	2
		2								
IALGIA, CO		2								
	2	1	2	2	2	2	2	2	2	2
IALGIA		2								
		2								
		2								
		2								
		2								
		1								
		2								
	2	1	2	2	2	2	1	2	2	2
		2								
DOR TORAX		2								
	2	1	2	2	2	2	2	1	2	2
	2	1	2	2	2	2	2	2	2	2
OBSTRUCAC		2								
		2								
	2	1	2	2	2	2	2	2	2	2
		2								
		2								
		2								

SINDROME		2								
	2	1	2	2	2	2	1	2	2	
		2								
RIPAL		2								
		2								
	2	1	2	2	2	2	2	1	2	2
IPOVOLEM		2								
		2								
		1								
		2								
		2								
		2								
	2	1	2	2	2	2	2	2	2	2
/CEFALEIA/		1	1							
		1								
		1								
		2								
		1								
		2								
		2								
		2								
	2	1	1	2	2	2	1	2	2	2
		2								
CICA/LOMB		2								
	2	1	2	2	2	2	2	2	2	2
		2								
		2								
		2								
	2	1	2	2	2	2	2	1	2	2
		2								
		2								
		1								
		2								
CICA		2								
		2								
		2								
MIALGIA		1	1				1			
IS/MIALGI		2								
		2								
	2	1	2	2	2	2	2	2	2	2
TACAO, SIN		2								
	2	1	1	2	2	2	2	2	2	2
		2								
XICA		1								
DISURIA		1								
	2	1	2	2	2	2	2	2	2	2
		1						1		

	2								
2	1	2	2	2	2	2	2	2	2
9	1	9	9	9	9	9	9	9	9
2	1	2	2	2	2	2	2	2	2
A, MIALGIA	2								
	2								
2	1	2	2	2	2	1	2	2	2
	2								
2	1	2	2	2	2	2	2	2	1
	2								
	2								
	2								
	1								
BSTRUCAO	1								
	2								
2	1	2	2	2	2	2	2	2	2
2	1	2	2	2	2	2	2	2	2
2	1								
	2								
2	1	2	2	2	2	2	2	2	2
	1					1			
GRIPAIS, C	2								
	1								
2	1	2	2	2	2	2	1	2	2
2	1	1	2	2	2	2	2	2	2
	1					1	1		
E MIALGIA	2								
	2								
	2								
	2								
2	1	1	2	2	2	1	1	2	2
	2								
AO, ODINO	2								
IA	2								
O NASAL	2								
	2								
2	1	2	2	2	2	2	2	2	2
2	1	2	2	2	2	2	2	2	2
	1					1			
2	1	2	2	2	2	2	2	2	2
2	1	2	2	2	2	1	2	2	2
NTERCOSTA	1	1							
2	1	2	2	2	2	2	1	2	2
MIALGIA, A	1							1	
	1								
2	1	2	2	2	2	2	2	2	2
2	1	2	2	2		2	1	2	2

NEIA		2								
		2								
		2								
	2	1	1	2	2	2	2	1	2	2
	2	1	2	2	2	2	2	2	2	2
AO, MIALGI		1								
	2	1	2	2	2	2	2	2	2	2
	2	1	2	2	2	2	2	2	2	2
	2	1	2	2	2	2	2	2	2	2
	2	1	9	2	2	2	2	2	2	2
		2								
	2	1	2	2	2	2	2	2	2	2
	2	1	2	2	2	2	2	2	2	2
FADIGA		2								
		2								
	2	1	2	2	2	2	2	2	2	2
	2	1	2	2	2	2	2	2	2	2
		2								
		1								
		2								
	2	1	2	2	2	2	2	2	2	2
		2								
	2	1	2	2	2	2	2	2	2	2
DOR NO PEI		2								
	2	1	2	2	2	2	1	2	2	2
		2								
	2	1	2	2	2	2	2	2	2	2
		2								
	2	1	2	2	2	2	2	2	2	2
	2	1	2	2	2	2	2	2	1	2
MAL ESTAR		2								
		1					1			1
	2	1	2	1	2	2	2	2	2	2
	9	1								
OMBALGIA		2								
	2	1	2	2	2	2	2	2	2	2
	2	1	2	2	2	2	2	2	2	2
	2	1	2	2	2	2	1	2	2	2
		1								
	2	1	1	2	2	2	2	2	2	2
	2	1	2	2	2	2	1	1	2	2
	2	1	2	2	2	2	2	2	2	2
		2								
	2	1	2	2	2	2	2	2	2	2
	2	1	2	2	2	2	1	2	2	2
ATICO		2								
		2								

		1								
		1	1					1		
		1								
		2								
		2								
		2								
MIALGIA		2								
		2								
		2								
CORIZA		2								
		1						1		
		1								
		1								
		2								
MIALGIA		2								
	2	1	1	2	2	2	2	2	2	2
	2	1	1	2	2	2	2	2	2	2
		2								
		2								
	2	1	2	2	2	2	2	2	2	2
NAPETENCI		2								
		2								
		2								
		1								
	2	1	2	2	2	2	2	2	2	2
ICA		2								
		2								
		2								
		2								
	2	1	1	2	2	2	2	2	2	2
		2								
		2								
N GRIPAIS		2								
		2								
	2	1	2	2	2	2	2	2	2	2
		1					1			
		2								
		2								
	2	1	2	2	2	2	2	2	2	2
		2								
	2	1	2	2	2	2	2	2	1	2
		2								
		2								
	2	1	2	2	2	2	2	2	2	2
CORIZA HEL		1								1
	2	1	1	2	2	2	2	2	2	2
	2	1	2	2	2	2	2	2	2	2

		2								
		1						1		
	2	1	2	2	2	2	1	2	2	2
	2	1	1	2	2	2	2	1	2	2
		1								
		2								
	2	1	1	2	2	2	2	2	2	2
	2	1	2	2	2	2	2	1	2	2
		2								
	2	1	2	2	2		2	2	2	2
E MIALGIA		1								
	2	1	2	2	2	2	2	2	2	1
	2	1	1	2	2	2	2	2	2	2
CICA, SINTC		2								
A		1	1							
		2								
	2	1	1	2	2	2	2	1	2	2
		2								
		2								
		2								
		2								
	2	1	2		2	2	2	2	2	2
	2	1	2	2	2	2	2	2	2	2
	2	1	2	2	2	2	2	2	2	2
	2	1	2	2	2	2	2	2	2	2
		2								
ORES NAS		2								
		1								
	2	1	2	2	2	2	2	2	1	2
O, INSUF. F		1							1	
	2	1	2	2	2	2	2	2	2	2
		1								
		1	1							
CORIZA		1	1							
CEFALEIA		2								
	2	1	2	2	2	2	2	2	2	2
	2	1	1	2	2	2	2	1	2	2
		2								
		2								
DIA, CORIZ		2								
		2								
A		1								
		2								
MIALGIA		2								
		2								
TAQUICARD		2								
MIALGIA		2								

		2								
	2	1	1	2	2	2	2	2	2	2
	2	1	1	2	2	2	2	2	2	2
		1								
		2								
CICA,CEFAL		1								
	2	1	1	2	2	2	2	2	2	2
		2								
A		2								
		2								
		2								
	2	1	2				2	2	2	2
		2								
		1						1		
		2								
	2	1	2	2	2	2	2	2	2	2
BECA		2								
	2	1	2	2	2	2	2	1	2	2
		2								
		2								
	2	1	2	2	2	2	2	2	2	2
	2	1	1	2	2	2	2	2	2	2
		2								
		2								
	2	1	2	2	2	2	2	2	2	2
GRIPAIS		1								
EFALEIA,CC		2								
	2	1	1	2	2	2	1	2	2	2
		2								
	2	1	2	2	2	2	2	2	2	2
IALGIA		2								
		1								
	2	1	2	2	2	2	2	2	2	2
	2	1	2	2	2	2	2	2	2	2
MIALGIA		1	1	2	2	2	2	2	2	2
	2	1	2	2	2	2	2	1	2	2
AO EM REGI		2								
		2								
		2								
		2								
		1								
		2								
	2	1	2	2	2	2	2	1	2	2
		2								
	2	1	2	2	2	2	2	2	2	2
		2								
NEIA		2								

2	1	2	2	2	2	1	2	2	1
, CONGEST/	2								
	2								
	2								
DOR/AGEUS	1								
ORPO	1								
2	1	2	2	2	2	2	2	2	2
	2								
2	1	1	2	2	2	2	2	2	2
	2								
2	1	1	2	2	2	2	1	2	2
	1								
2	1	2	2	2	2	2	1	2	2
2	1	2	2	2	2	2	2	2	2
2	1	2	2	2	2	2	1	2	2
DIA+HIPOTI	1								
HIPOREXIA	2								
2	1	2	2	2	2	2	2	1	2
CIA , MIALG	1					1			
	2								
	2								
2	1	2	2	2	2	2	2	2	2
O NASAL, C	2								
2	1	2	2	2	2	2	2	2	2
2	1	2	2	2	2	2	2	2	2
	2								
	2								
	2								
2	1	1	2	2	2	2	2	1	2
	2								
2	1	1	2	2	2	2	2	2	2
	1								
	1								
	2								
2	1	2	2	2	2	2	2	2	2
2	1	2	2	2	2	2	1	2	2
2	1	2	2	2	2	2	2	2	1
	1								
2	1	2	2	2	2	2	2	2	2
9	1	9	9	9	9	1	9	9	9
2	1	2	2	2	2	2	1	2	2
	1								
2	1	2	2	2	2	2	1	2	2
	2								
	2								
MIALGIA	2								
	1								

		2								
, HIPOREXI		1								
	2	1	2	2	2	2	2	2	2	2
	2	1	2	2	2	2	1	2	2	2
		1								
		2								
		2								
	2	1	1	2	2	2	2	2	2	2
	2	1	2	2	2	2	2	2	2	2
	2	1	2	2	2	2	2	1	2	2
		2								
	2	1	1	2	2	2	2	2	2	2
	2	1	2	2	2	2	2	2	2	2
	2	1	2	2	2	2	2	2	2	2
	2	1	2	2	2	2	2	1	2	2
	2	1	2	2	2	2	2	2	2	2
		2								
		2								
	2	1	1	2	2	2	2	2	2	2
		1								
EFALEIA, DI		1								
		2								
		2								
		2								
		1	1							
		2								
	2	1	2	2	2	2	2	2	2	2
		2								
		2								
MIALGIA, C.		2								
	2	1	2	2	2	2	2	2	2	2
MIALGIA		2								
	2	1	9	9	9	9	1	1	9	9
	2	1	2	2	2	2	2	2	2	2
		2								
	2	1	2	2	2	2	2	2	2	2
		2								
		1								
	2	1	2	2	2	2	2	2	2	2
		2								
		2								
		1								
	2	1	2	2	2	2	2	2	2	2
EFALEIA , C		2								
		2								
		2								
	2	1	2	2	2	2	2	2	2	1

	2	1	2	2	2	2	2	2	2	2
		1								
	2	1	2	2	2	2	2	2	2	2
	2	1	2	2	2	2	2	2	2	2
	2	1	2	2	2	2	1	2	2	2
		2								
	2	1	2	2	2	2	2	2	1	2
		1								
	2	1								
		2								
	2	1	2	2	2	2	2	2	2	2
	2	1	2	2	2	2	2	2	2	2
		2								
		2								
		2								
		2								
		1								
		2								
		2								
		2								
		2								
	2	1	2	2	2	2	1	2	2	2
		2								
	2	1	2	2	2	2	2	2	2	2
	2	1	2	2	2	2	2	1	2	2
BSTRUCAC		1								
CIA/FRAQU		2								
		2								
		2								
RAQUEZA		2								
		2								
RPO		2								
	2	1	2	2	2	2	2	2	2	2
	2	1	2	2	2	2	2	1	1	2
		1								
		2								
		2								
FALEIA		2								
	2	1	2	2	2	2	2	2	2	2
		1								
		2								
		2								
NAUSEAS		2								
	2	1	2	2	2	2	2	2	2	2
	2	1	2	2	2	2	2	2	2	2
		2								
RGANTA, C		2								

, FRAQUEZ		1	1					1		
		2								
		1								
		1								
		2								
		2								
		1						1		
	2	1	2	2	2	2	2	2	2	2
BECA		2								
		1						1		
		2								
		1	1							
		2								
		1								
	2	1	2	2	2	2	2	2	2	1
	2	1	2	2	2	2	2	2	2	1
		2								
		1								
	2	1	2	2	2	2	2	2	2	2
	2	1	2	2	2	2	2	2	2	2
	2	1	2	2	2	2	2	9	2	2
CEFALEIA		2								
	2	1	2	2	2	2	2	2	2	2
		2								
MINAL, CEF		1								
		1						1		
		1								
	2	1	2	2	2	2	2	2	2	2
		2								
		1	1							
		2								
		2								
	2	1	2	2	2	2	2	2	2	2
		1								
	2	1	2	2	2	2	1	2	2	2
CORIZA,CON		2								
	2	1	2	2	2	2	2	1	2	2
	2	1	1	2	2	2	2	1	2	2
		2								
	2	1	2	2	2	2	1	2	2	2
		2								
		2								
FALEIA, CO		2								
		2								
	2	1	2	2	2	2	1	2	2	1
		2								
	2	1	1	2	2	2	2	2	2	2

2	1	2	2	2	2	2	2	2	2
	2								
	2								
	2								
	2								
	1								
	2								
	2								
	2								
	2								
2	1		1	2	2	2	2	2	2
	2								
	1								
	2								
	2								
	1								
	1					1			
2	1	2	2	2	2	2	2	2	2
2	1	2	2	2	2	2	2	2	2
	1								
	2								
CORIZA.	1								
	2								
	2								
CEFALEIA	2								
2	1	1	2	2	2	2	1	2	2
	2								
2	1	1	2	2	2	2	2	2	2
	2								
2	1	2	2	2	2	1	2	2	2
	2								
	2								
	2								
	2								
	2								
2	1	2	2	2	2	2	1	2	2
2	1	2	2	2	2	2	2	2	2
2	1	2	2	2	1		2	2	2
	2								
	1								
E CORIZA	2								
	2								
MIALGIA + C	1								
	2								
	1	1							
2	1	2	2	2	2	2	2	2	2
	2								

	2	1	2	2	2	2	2	2	2	2
	2	1	2	2	2	2	2	2	1	2
	2	1	2	2	2	2	2	2	2	2
	2	1	2	2	2	2	2	2	2	2
		2								
		2								
	2	1	2	2	2	2	1	2	2	2
	2	1	2	2	2	2	2	2	2	2
		1								
		1						1		
		2								
		1								
		2								
	2	1	2	2	2	2		2	2	2
		1						1		
		2								
	2	1	2	2	2	2	2	2	2	2
	2	1	2	2	2	2	2	2	2	2
	2	1	2	2	2	2	2	2	2	2
		2								
	2	1	2	2	2	2	2	2	2	2
		2								
	2	1	2	2	2	2	2	2	2	2
	2	1	2	2	2	2	2	2	2	2
CORIZA		2								
	2	1	2	2	2	2	2	2	2	2
		2								
RTO NA GA		2								
		2								
		2								
	2	1	2	2	2	2	2	2	2	2
	2	1	2	2	2	2	1	2	2	2
		2								
MIALGIA		2								
	2	1	2	2	2	2	1	2	2	2
		2								
		2								
GRIPAIS,		1					1			
		2								
		2								
		2								
	2	1	2	2	2	2	2	2	1	2
		2								
		1						1		
		2								
	2	1	2	2	2	2	2	2	2	2
		1								

AREA		2								
	2	1	2	2	2	2	2	2	2	2
ACAO/PROS		2								
AO, TAQUIC		2								
		2								
	2	1	2	2	2	2	2	2	2	2
	2	1	2	2	2	2	2	2	2	2
		1								
		2								
		2								
		2								
		1								
		2								
O POR ECL		2								
BECA/ DOR		2								
		1					1			
LIQUIDO VI		2								
		2								
	2	1	1	2	2	2	1	1	2	2
		2								
		2								
	2	1	2	2	2	2	2	2	2	2
	2	1	1	2	2	2	2	1	2	2
	2	1	2	2	2	2	2	2	2	2
		1								
		2								
		2								
		2								
	2	1	1	2	2	2	2	1	2	2
MIALGIA, A		1								
		2								
		2								
		1								
GRIPAIS, IF		1	1							
		2								
		1	1					1		
	2	1	2	2	2	2	2	2	2	2
		1								
		2								
		2								
	2	1	1	2	2	2	2	2	2	2
ASTENIA		2								
	2	1	2	2	2	2	2	2	2	2
		2								
		2								
	2	1	1	2	2	2	2	2	2	2
EFALEIA		1								

		2								
	2	1	2	2	2	2	2	2	2	2
		2								
	2	1	2	2	2	2	2	2	2	2
CRISE CON		2								
R		1						1		
	2	1	2	2	2	2	2	2	2	2
	2	1	2	2	2	2	2	2	2	2
	2	1	1	2	2	2	1	2	1	2
		2								
PIR., CONV		2								
	2	1	2	2	2	2	2	2	9	2
ENIA		2								
	2	1	2	2	2	2	2	2	2	2
	2	1	2	2	2	2	2	2	2	2
		2								
	2	1	2	2	2	2	2	2	2	2
CORIZA		2								
		1	1					1		
	2	1	2	2	2	2	2	2	2	2
	2	1	2	2	2	2	1	2	2	2
		1								
		2								

MUNODEP	RENAL	OBESIDADE	OBES_IMC	OUT_MORB	MORB_DES	VACINA	DT_UT_DOS	MAE_VAC	T_VAC_MA
						9			
2	2	2		2		2			
						1			
						1	22/05/2020		
2	2	2		1	GESTANTE	9			
						9			
2	2	2		1	GESTANTE	2			
						2			
						1	20/07/2020		
2	2	2		1	DHEG	2			
						2			
						9			
						2			
						9			
2	2	2		1	DOENCA D	2			
2	2	2		1	GESTANTE	9			
		1		1	GESTANTE				
						9			
						2			
						1	30/05/2020		
						9			
						1			
				1	GESTACAO	2			
						1			
		1	4,4			2			
2	2	1	33,9	2		9			
2	2	2		1	HAS,GESTA	9			
2	2	2		1	GESTANTE	2			
2	2			1	HIPOTIREO	2			
						1	08/04/2020		
2	2	2		2		2			
2	2	2		1	GESTANTE	1			
1	2	1	30	1	MIASTENIA	9			
						9			
						9			
2	2	1	32	1	HAS	9			
						9			
						2			
				1	GESTANTE	2			
2	2	2		1	ETILISMO	9			

				1	GESTANTE	1	
						9	
						9	
						9	
2	2	2		1	BARIATRIC	1	30/06/2020
				1	GESTANTE	9	
				2		9	
						9	
						9	
2	2	2		1	ANEMIA F	9	
						1	
2	2	2		1	CRISES CO	9	
2	2	2		2		1	
		1	46,8			1	
						9	
9	9	1	34	9		2	
						1	
						2	
						2	
2	2	1	72	2			
						9	
						2	
2	2	2		1	GESTACAO	2	
				1	GESTANTE	1	01/07/2020
						9	
						9	
2	2	2		1	GESTANTE	1	
						1	
						9	
						9	
2	2	1		2		9	
1	2	2		1	MIOMATO	2	
2	2	2		1	EX-TABAGI	9	
						2	
	1					9	
2	2	2		2			
		1	35	9		1	20/05/2020
						9	
		1	30	1	HIPOTIREC	9	
				1	COLECISTITE AGUDA		
						2	

						9	
						2	
						9	
				1	GESTANTE	2	
						9	
						1	20/03/2020
2	2	2		1	GESTANTE	1	24/04/2020
						9	
2	2	2		1	GESTANTE	2	
						9	
2	2	2		1	GESTANTE	9	
				1	GESTANTE	2	
1						2	
				1	GESTANTE	9	
						2	
				1	EPILEPSIA	1	04/04/2020
2	2	2		1	HAS E GRA	2	
2	2	2		1	GESTANTE	9	
				1	HAS GESTA	9	
2	2	1	30	2		9	
2	2	1	44,1	2			
						9	
						9	
				1	DCA TIREOIDE		
				1	GESTANTE	2	
						9	
						9	
						2	
						9	
						9	
		1					
						2	
2	2	2		1	DM GESTA	9	
						9	
2	2	2		1	GRAVIDEZ	1	17/02/2020
						2	
				1	GESTANTE	9	
						2	
2	2	2		2		9	
2	1	2		2		9	
						9	
						1	05/05/2020
						1	
						9	
2	2	2		1	HIPOTIREC	9	
						1	15/04/2020
						2	

				1	MOLA HID/	2	
2	2	2		1	GESTANTE	9	
2	2	1		2		9	
						9	
						2	
				1	ANEMIA	1	
		1	48	2		2	
				1	HIPERTENS	2	
						9	
				1	HAS	9	
				1	GESTANTE	2	
						2	
2	2	2		2		2	
						2	
						9	
2	2	2		1	GESTANTE	9	
						1	15/04/2020
2	2	2		1	GESTANTE	9	
						2	
						2	
2	2	2		1	GESTANTE	2	
2	2	2		2		9	
						2	
2	2	2		1	DHEG	9	
2	2	2		2		9	
2	2	1		2		9	
2	2	1	44	1	HAS	9	
						9	
				1	HAS,HIPOT	1	
2	2	2		1	GESTANTE	9	
2	2	2		1	OBESIDADI	2	
2	2	2		2		2	
				1	HIPERTENS	9	
2	2	2		2		2	
						9	
				1	GESTACAO	9	
2	2	2		1	GESTANTE	9	
2	2	2		1	GESTANTE		
2	2	2		1	DIABETES (	1	01/09/2019
						9	
2	2	2		2		9	
2	2	2		1	LES, HIPOT	9	
				1	HIPOCALEI	9	

						9	
2	2	2		1	GESTANTE	1	
				1	GESTANTE	9	
						1	28/04/2020
2	2	2		1	GESTANTE	2	
						2	
2	2	2		1	GRAVIDA	9	
						1	14/10/2020
						2	
						2	
						1	
2	2	1		2		9	
				1	DIABETES (	9	
2	2	1	39,5	2		2	
						9	
				1	GESTACAO	9	
2	2	2		1	GESTANTE	9	
2	2	2		1	GESTANTE	9	
	2	2		1	GESTANTE		
						1	05/06/2020
						1	
2	2	2		1	GESTANTE	1	
		1	30	1	CESAREA P	9	
				1	GESTANTE		
1	2	2		1	CARCINOM	9	
		1	40	1	GESTANTE	1	05/04/2020
						1	18/05/2020
2	2	2		1	DHEG	1	06/05/2020
2	2	2		2		9	
						9	
						1	15/04/2020
				1	GEATANTE IDADE GESTCIONAL IGN		
2	2	2		1	GESTANTE	9	
						9	
2	2	2		1	GESTANTE	2	
						9	
						9	
2	2	2		2		2	
2	2	2		1	DIABETES (	9	
2	2	2		1	GESTANTE	9	
				1	GESTANTE	9	
				1	GESTANTE	9	
						1	02/05/2020

						9	
2	2	1	33,5	2		9	
				1	GESTANTE	2	
	9	2		1	GESTANTE	9	
2	2	2		1	GESTANTE	9	
						9	
1				1	LUPUS		
2	2	1	30,6	2		9	
2	2	2		1	GESTANTE	9	
						9	
						2	
						1	
				1	GESTANTE	9	
						9	
2	2	2		1	GRAVIDA	2	
						9	
				1	GRAVIDEZ	2	
2	2	2		1	GRAVIDA	2	
				1	GESTANTE	9	
						9	
						2	
2	2	2		1	GESTANTE	9	
						9	
						9	
						1	18/06/2020
		1				1	
				1	GESTANTE	1	14/05/2020
						9	
						9	
2	2	2		2		9	
				1	HAS	9	
2	2	2		1	GESTANTE	2	
2	2	2		1	CARDIOPA	9	
						1	15/05/2020
						1	26/03/2020
	1						
						2	
						9	
				1	PARTO CES	2	
						9	
2	2	1		2		9	
		1				9	
						9	

				2
2	2	2	1 GESTANTE	9
				2
2	2	2	1 2 SEMESTR	9
2	2	2	2	2
	1			2
				9
			1 GESTANTE	9
				9
2	1	2	1 GESTANTE	9
				2
				9
2	2	2	1 GESTANTE	
2	2	2	2	2
			1 GESTANTE,	9
				9
				9
			1 OBEDIDAD	9
2	2	1 37,9	1 HIPOTIREC	9
				2
				1 02/06/2020
2		2	1 GESTANTE	9
				9
				1 06/08/2019
			1 PIELONEFR	1 13/07/2020
			1 GESTANTE	9
			1 ALTO RISC	1 19/05/2020
2	2	2	2	9
				2
2	2	1 46	1 GESTANTE	2
				9
				9
			1 GESTANTE	2
				9
			1 GESTANTE - REALIZADO PARTO	
				9
				9
				9
				9
			1 GESTANTE DE ALTO RISCO	
2	2	2	1 GESTANTE	2

						9	
				1	DM GESTA	9	
2	2	1		1	BRONQUIT	9	
						9	
						9	
				1	GESTANTE		
						9	
2	2	2		1	GRAVIDEZ	2	
						1	30/03/2020
						9	
2	2	1		2		9	
						1	24/03/2020
						2	
						2	
						9	
2	2	2		1	DMG/GEM	1	27/04/2020
2	2	1		1	G2C1A0	9	
				1	GESTANTE	9	
						2	
				1	GESTANTE		
1				1	GESTANTE	9	
2	2	2				9	
						9	
						9	
						2	
						2	
2	2	2		1	GESTANTE	1	01/12/2019
						2	
2	2	2		1	PUERPERA	9	
2	2	1	39,7	2		9	
2	2	2		1	DMG	1	26/06/2020
2	2	2		1	GESTANTE	2	
						9	
						2	
2	2	2		1	RINITE	2	
				1	DOENCA CARDIO VASCULAR GESTANT		
						9	
2	2	2		1	GESTANTE	9	
2	2	2		1	GESTANTE	9	
				1	GRAVIDA	9	
						9	
						1	
2	2	2		1	COVID	9	

						9	
2	2	2		2		1	01/03/2020
						1	20/06/2020
						9	
				1	GESTANTE		
2	2	2		1	GESTANTE	9	
						9	
2	2	1	0	2			
		1		1	GESTANTE	2	
						9	
				1	HIPERTIRE	1	05/05/2020
				1	GESTANTE	9	
				1	GESTANTE	9	
				1	OBESIDADI	9	
						9	
						2	
2	2	2		1	GESTANTE	9	
				1	PRE ECLAM	1	
9	9	9		1	GESTANTE	9	
2	2	2		1	ANEMIA, CHOQUE SEPTICO		
2	2	2		2		9	
2	2	2		2		1	25/04/2020
						1	15/05/2020
				1	GESTANTE	2	
2	2	1		1	GESTANTE		
2	2	1		2		9	
2	2	2		1	GESTANTE	2	
						1	21/04/2020
2	2	2		1	DIABETE G	2	
2	2	2		1	GESTANTE	9	
2	2	2		1	DIABETES	1	
						2	
						9	
						9	
						9	
2	2	1	35,6	1	DMG, PRE	9	
		1	35,8			9	
				1	GESTANTE		
2	2	2		1	GESTANTE	2	
						2	
2	2	2		2		1	29/04/2020
						1	09/06/2020
				1	GESTANTE	9	

					9	
			1	GESTANTE		
2	2	1	1	ITU	9	
					9	
			1	HIPOTIREC	9	
2	2	2	1	GESTANTE	1	29/05/2020
					9	
					9	
2	2	2	1	RENITE ALE	1	10/02/2020
					9	
2	2	2	1	DHEG, GES	9	
					9	
			1	GRAVIDES	2	
2	2	2	1	HIPODIREI	2	
2	2	2			2	
2	2	2	1	GESTANTE	9	
					9	
					9	
			1	GESTACAO ALTO RISCO		
					2	
			1	GESTANTE	9	
					2	
					9	
			1	GESTANTE	9	
					1	12/05/2020
					9	
2	2	2	1	GESTANTE	9	
					9	
					2	
		1	1	GESTANTE	9	
			1	EX TABAGISTA		
					9	
2	2	2	2		2	
2	2	2	2		1	14/06/2020
					9	
					9	
2	2	2	1	CERCLAGEI	2	
					9	
			1	GESTANTE	2	
2	2	2	2		9	
					9	
					1	20/05/2020
			1	TTO SIFILIS	9	
					2	

						2	
2	2	2		1	GESTANTE	9	
2	1	2		2		9	
2	2	2		1	PIELONEFR	9	
						2	
2	2	2		1	DIABETES (	2	
				1	OBESIDADI	2	
2	2	1	35,2	2		9	
						9	
						9	
2	2	2		1	HAS, ICC V/	2	
				1	GRAVIDEZ		
						1	
2	2	2		1	MIALGIA, (	9	
2	2	2		2		2	
						9	
				1	GESTANTE	1	04/05/2020
2	2	1		1	HISTORICO	1	05/06/2020
						9	
2	2	2		1	GRAVIDEZ DE ALTO RISCO		
				1	GESTANTE		
						1	19/07/2020
						9	
2	2	2		1	GESTANTE	2	
						2	
				1	GESTANTE	9	
						1	
				1	DM GESTA	1	
						9	
		1				2	
				1	IG 39 SEM/	9	
2	2	2		2		9	
						1	28/04/2020
						9	
		1	41,3	1	GESTANTE	2	
				1	GESTANTE	9	
				1	GESTANTE	9	
2	2	2		1	TOXOPLAS	9	
2	2	2		1	HAS	9	
						1	
						1	
2	2	2		1	GESTANTE	2	
2	2	2		1	OBESIDADI	1	
2	2	2		1	OBESIDADI	2	

						9	
2	2	2		1	HIPERTIRO	9	
2	2	2		2		2	
2	2	1	33,3	2		9	
2	2	1		1	HAS		
2	2	2		2		2	
2	2	2		1	DEFICIT M(	9	
				1	GESTANTE		
2	2	1	30	1	HIPOTIROI	9	
2	2	2		1	GESTANTE	1	
2	2	1	42,8	1	HAS/DIABE	9	
2	2			2		9	
2	2	2		1	GESTANTE	9	
2	2	2		1	GESTANTE	9	
2	2	2		2		2	
2	2	2		1	GRAVIDEZ	2	
2	2	2		1	GRAVIDEZ	2	
						1	
2	2	2				9	
						1	20/03/2020
						2	
				2		9	
2	2	2		2		9	
2	2	2		1	GESTANTE		
2	2	2				9	
2	2	2		1	GRAVIDEZ	9	
						2	
2	2	1		1	GESTANTE;	2	
						2	
2	2	1	39	1	GESTANTE	1	10/01/2020
2	2	2		1	GESTANTE	2	
2	2	2		1	GESTANTE	9	
						2	
						2	
						2	
2	2	2		2		1	10/06/2020
2	2	2		1	HAS GESTA	1	
						9	
2	2	1				9	
						9	

2	2	2		1	ANEMIA FA	1	27/04/2020
				1	GESTANTE		
2	2	2		1	GESTANTE	9	
2	2	1	0	2		1	22/06/2020
						9	
2	2	2		1	GESTANTE	2	
				1	POS BARIA	9	
						9	
2	2	2		2		2	
						1	29/07/2020
2	2	2		1	GESTANTE	1	22/05/2020
						9	
2	2	2		1	TROMBOFI	9	
				1	GESTANTE	1	11/05/2020
2	2	2		1	AVCI-2005	9	
2	2	1		1	LESOES ERI	9	
				1	MIASTENIA	9	
				1	PRE ECLAM	2	
						2	
						2	
				1	GESTANTE	1	29/06/2020
2	2	2		1	OF, GESTAI	9	
						2	
						9	
2	2	2		1	DIABETES (	2	
1	2	2		1	CA DE MAI	2	
						1	11/05/2020
						9	
2	2	2				9	
						9	
2	2	1	39,1	2		9	
2	2	2		2		9	
				1	GESTANTE	9	
						9	
2	2	1		2		2	
						9	
		1	35			2	
						1	
				1	GESTACAO	9	
2	2	1		2		1	
						2	
						9	
2	2	2		1	HIPOTIREC	9	
2	2	1	30	1	GESTANTE	2	

						9	
2	2	2		1	INVESTIGA	2	
2	2	2		1	GESTANTE	9	
						1	16/04/2020
9	9	9		1	GESTANTE	9	
				1	HAS	9	
2	2	2		1	DOENCA RESPI. CRONICA		
1						9	
2	2	2		2		2	
						2	
2	2	1	33,1	2		2	
2	2	1	35	1	HIPOTIREC	9	
						2	
2	2	2		1	GESTANTE,	9	
2	2	2		1	GESTANTE	1	24/04/2020
				1	DIABETES (	2	
				1	GESTANTE	9	
		1		1	GESTANTE	9	
						2	
2	2	2		1	GESTANTE	9	
						9	
2	2	2		1	GESTANTE		
2	2	2		2		2	
						2	
				1	CA DE CRANIO		
1	2	1	33	2		9	
2	2	2		1	GESTANTE	9	
2	2	1		2			
						1	10/03/2020
				1	DIABETES (	9	
				1	MACROSS(	9	
2	2	1	35	2		9	
						9	
						9	
						9	
						2	
						9	
2	2	2		1	GESTANTE		
2	2	1	30,5	2		9	
						9	
2	2	2		1	GESTANTE	2	
2	2	2		2		9	

					2	
					1	10/04/2020
					9	
2	2	9	1	GESTANTE	9	
2	2	2	1	GESTANTE	9	
			1	GESTANTE	9	
					9	
					9	
2	2	2	2		2	
2	2	2	1	GESTACAO	9	
					9	
					9	
					2	
2	2	2	2		9	
					9	
			1	EX TABAGISTA, DISLIPIDENCIA		
2	2	2	1	GESTANTE	9	
2	2	2	1	SINDROME	2	
					2	
					9	
2	2	2	2		1	01/06/2020
2	2	2	1	DHEG		
					2	
2	2	2	1	GESTANTE		
			1	GESTANTE	9	
					2	
2	2	1	1	GESTANTE	2	
2	2	2	1	GESTANTE	9	
			1	GESTACAO	9	
1						
2	2	2	1	GESTACAO	9	
2	2	2	1	GESTANTE		
			1	GESTANTE	9	
2	2	2	1	GESTANTE	9	
2	2	2	2		2	
			1	HIPERTENSAO		
2	2	2	2		9	
			1	GESTANTE	9	
			1	TIREOIDOP	9	
2	2	2	2		9	
			1	GESTANTE	9	
					9	
			1	ABORTO RETIDO/ GESTANTE		
			1	GESTANTE	9	

2	2	2		1	GESTANTE		
				1	HAS	9	
2	2	2		1	GESTANTE	9	
2	2	2		1	GESTANTE	2	
						2	
				1	SINDROME HELLP+ POI		CESARIANA
2	2	2		1	GESTANTE	1	01/04/2020
						9	
						2	
						2	
2	2	2				2	
						1	14/05/2020
						9	
						9	
						9	
						9	
						9	
						9	
2	2	2		2		9	
						1	24/04/2020
2	2	1		2		9	
9	9	9		1	GESTANTE	9	
2	2	2		2		1	01/03/2020
						9	
						9	
						9	
2	2	2		1	GESTANTE		
2	2	2		1	GESTANTE	1	11/08/2020
						9	
						2	
						9	
2	2	2		1	DEPRESSA(	9	
						2	
						9	
				1	GESTANTE	9	
				1	DMG, HIPC	1	15/04/2020
2	2	2		2		2	
						9	
2	2	2		2		9	
2	2	9		1	COVID-19 I	9	
2	2	1	39	2		2	
						1	01/06/2020
2	2			1	COVID-19	9	

1	2	2		1	AIDS, TUBE	9	
				1	HIPOT./GE	1	20/05/2020
2	2	2		2		9	
						2	
						9	
2	2	2		2		1	08/04/2020
						9	
	2	2		1	PNEUMON	2	
2	2	2		1	OBESIDADI	2	
				1	GESTANTE	1	
2	2	2		1	GESTANTE	9	
						9	
				1	LINFOMA		
2	2	2		1	DMG	1	07/05/2020
2	2	2		2		2	
						1	05/05/2020
2		1		2		2	
						1	28/05/2020
						2	
				1	TB	1	01/07/2020
2	2	2		1	TTV GESTA	9	
1				1	GESTANTE		
						9	
						9	
				1	HAS	2	
2	2	2		1	GESTANTE	9	
						1	19/05/2020
						9	
						1	15/04/2020
2	2	2		1	HIPOTIREC	9	
2	2	2		1	HAS/HIPOT	2	
2	2	2		2		9	
						9	
		1	30			2	
						9	
						9	
						9	
2	2	2		2		1	15/04/2020
	1					9	
						2	
						9	
2	2	2		1	GESTANTE	2	
						2	

						2	
				1	GESTANTE		
2	2	2				2	
2	2	1		2		1	30/05/2020
1						1	13/05/2020
						9	
2	2	2		2		2	
				1	P.E COM SINAIS DE GRAVIDADE		
						9	
						9	
2	2	2		1	GESTANTE	9	
				1	GESTANTE	2	
9	9	9		1	GESTANTE	9	
						9	
		1	40			9	
2	2	2		1	GESTANTE	2	
2	2	1	38,5	1	GESTANTE	1	26/05/2020
2				1	GESTANTE		
				1	GESTANTE	9	
				1	GESTANTE	9	
9	9	9		1	OBESIDADI	2	
						2	
						1	
						9	
2	2	2		2		2	
2	2	1	45,2	1	GESTANTE,	9	
2	1	2		1	HIPOTIREII	9	
				1	EX TABAGISTA		
						1	16/10/2019
				1	OBESIDADI	1	
				1	GESTANTE	9	
2	2	2		1	GESTANTE	9	
2				1	PRE-ECLAN	9	
						2	
2	2	2		1	GESTANTE	9	
2	2	2		1	PRE ECLAMPSIA		
2	2	2		1	HAS GESTA	9	
						9	
2	2	2		1	HAS	1	
				1	GESTANTE	2	
1	2					9	
				1	GESTANTE	9	
						9	

2	2	2	1	GESTANTE	9	
2	2	2	1	GESTANTE		
1			1	GESTANTE		
2	2	2	1	TVP EM MI	1	
2	2	2	1	OBESIDADE		
					1	29/04/2020
2	2	2	1	GESTANTE	9	
					9	
					9	
2	2	2	1	GESTANTE	2	
2	2	2	1	GESTANTE	1	22/12/2020
					9	
2	2	2	1	OBESIDADI	2	
					9	
			1	GESTANTE	1	15/04/2021
					9	
					9	
					2	
			1	DHEG/ IG:	9	
		1			2	
2	2	1	1	GESTANTE	2	
					2	
	9	9	1			
					2	
			1	GESTANTE	2	
					2	
2	2	1	2		2	
					9	
					9	
2	2	2	1	GESTANTE	2	
			1	GESTANTE	9	
					2	
					9	
					9	
					2	
2	2	2	1	HAS GESTACIONAL		
			1	GESTACAO ALTO RISCO		
			1	GESTANTE		
					2	
					9	

		1		1	GESTANTE	2	
2	2	2		1	HAS	2	
						9	
				1	GESTANTE	9	
2	2	1	33,2	2		9	
						2	
						9	
1	2	1		1	SIDA	2	
2	2	2				9	
		1		1	GESTANTE	9	
2	2	2		1	GESTANTE	2	
						9	
2	2	2		2		1	01/04/2020
						2	
				1	HIPOTIREC	9	
	2	2		1	GESTANTE	2	
						2	
2	2			1	ABLACAO	2	
				1	GESTANTE		
2	2	1		2		2	
						9	
						9	
2		1		2		9	
						9	
						9	
		1		1	SIFILIS	9	
						9	
				1	VDRL 1:1	9	
	1			1	LES HA 12	9	
2	2	2		1	GESTANTE	9	
2	2	2		1	GESTANTE	2	
		1		1	HAS		
				1	GESTANTE	9	
						9	
2	2	2		1	AVC E TVP	9	
2	2	2		1	GESTANTE	2	
						2	
2	2	1		2		1	

				1	GESTANTE	2	
						9	
						2	
2	2	2		1	HAS	2	
						2	
2	2	2		1	HIPOTIREC	9	
						9	
2	2	1		1	GESTANTE	9	
						9	
2	2	2		1	GESTANTE	2	
		1				9	
						9	
						1	
				1	PNEUMON	9	
2	2	2		1	GESTANTE		
				1	GESTANTE	2	
				1	GESTANTE	9	
						2	
						2	
				1	GESTANTE	2	
						2	
2	2	1		2		2	
2	2	2		1	GESTANTE	9	
						9	
				1	GESTANTE	9	
2	2	2		1	GRAVIDA		
				1	GESTANTE	9	
						9	
2	2	1		2		1	03/12/2021
						9	
2	2	2				9	
				1	GESTANTE		
						2	
						9	
						9	
		1		1	DEPRESSA(	9	
						2	
2	2	2		1	DMG	9	
						9	
2	2	1	34,8	1	GESTANTE.	9	
						2	

			1 INFECCAO	9
2	2	2	1 GESTANTE	9
				1
				9
				9
2	2	1 35,3	1 HAS, PRE-E	9
				2
2	2	2	2	9
2	2	1 33,5	2	9
				2
				9
			1 GESTANTE	
			1 GESTANTE	2
				9
9	9	9	1 GESTANTE	9
				9
				1 01/06/2020
			1 GESTANTE	1 01/10/2020
2	2		1 GESTANTE	9
2	2	2	1 DIABETES (	2
				9
				9
				2
		1		9
				9
				1 19/04/2021
				2
			1 GESTANTE	1 15/05/2020
2	2	1 36	2	9
2	2	2	1 GESTANTE	2
				1
			1 GESTANTE	2
				2

2	2	1		2		2	
						9	
						9	
						2	
						9	
2	2	2		1	GESTANTE	2	
				1	GESTANTE	2	
2	2	2		1	GRAVIDEZ	1	10/02/2012
				1	GESTANTE	2	
						9	
		1		1	GRAVIDA	2	
2	2	2		2		2	
2	2	1	29,1	1	GESTANTE	2	
2	2	2		1	TROMBOFI	2	
				1	GESTANTE		
				1	GESTANTE		
				1	DMG GEST	9	
2	2	1		1	MIOMAS U	9	
						9	
						1	18/04/2021
2	2	2		2		2	
						9	
						9	
2	2	2		1	HAS	2	
2	2	1				9	
2	2	1		2		9	
				1	HIPOTIREC	2	
2	2	2		1	GESTANTE	2	
						2	
		1					
2	2	2		1	GESTANTE	9	
1	2	1		1	DMG	9	
				1	DIABETE G	9	
2	2	2		1	GESTANTE	2	
				1	GESTANTE	2	
						9	
2	2	2		1	HAS	2	
2	2	2		2			
				1	GESTANTE 3 TRIMES		
						9	

			1	GESTANTE	1	
			1	GESTANTE	1	30/01/2021
2	2	2	1	GESTANTE	2	
					1	15/05/2021
					1	01/03/2020
9	9	9			9	
					9	
2	2	2	1	GESTANTE	1	20/04/2021
					9	
2	2	1	2		9	
					1	
2	2	1	1	GESTANTE	9	
2	2	2	1	GESTANTE	9	
					1	
					2	
			1	HIPOTIREC	2	
2	2	2	1	HIPERTENS	9	
2	2	2	1	GESTANTE 29 SEM, HIPOTIREOIDIS		
		1			9	
1	1		1	USUARIA DE ENTORPECENTE		
					2	
2	2	1	2		1	21/08/2020
2					2	
					9	
					9	
2	2	2	1	GESTANTE	9	
					9	
					2	
			1	GESTANTE	9	
					9	
					9	
2	2	2	2		2	
					1	
					2	
					1	
					9	
			1	HIPOTIREC	9	
2	2	2	1	GESTANTE	1	23/04/2021
					9	
			1	GESTANTE	2	
					2	
					9	

						9	
		1		1	HAS,HIPER	9	
						9	
						9	
						9	
2	2	2		1	HIPOTIREC	2	
2	2	2		1	GESTANTE	2	
2	2	2		1	GESTANTE	9	
						2	
				1	VITILIGO	9	
						2	
		1				1	20/04/2021
						2	
						9	
2	2	2		1	HAS GESTA	9	
						9	
						2	
						9	
2	2	1		2		9	
2	2	1		1	GESTANTE	2	
2	2	1		2		2	
				1	HAS,OBESIDADE		
						2	
2	2	2		1	GESTANTE	9	
2	2	2		2		9	
		1				9	
						9	
						2	
						9	
						9	
		1	40,2			9	
2	2	2		1	GESTANTE	1	01/04/2020
				1	GESTANTE	9	
2	2	2		2		2	
2	2	2		1	GESTANTE	9	
						9	
2	2	2		2		9	
						2	
2	2	1		1	GESTANTE	1	
						1	12/04/2020
2	2			1	GESTANTE	1	28/04/2021

2	2	2	2		2
					9
2	2	2	2		9
2	2	2	1	GESTANTE	9
			1	ASMA	9
			1	GRAVIDEZ	9
2	2	2	2		9
					9
					9
2	2	1	1	GESTANTE	2
2	2	2	1	ADOLESCEI	2
					2
					2
2	2	1	1	OBESIDADI	2
			1	GESTANTE	
			1	DIABETES (	9
2	2	2	1	GESTANTE	2
2	2	2	1	GESTANTE	2
					2
					9
					2
2	1	2	2		2
2	2	2	2		2
2	2	2	1	GESTANTE	2
					9
			1	GESTANTE	
			1	GESTANTE	2
					1
					9
					2
					9
					9
					9
					9
					1
					2
					9
					9
2	2	2	2		2
2	2	2	1	DIABETES (	2
					9

		1					
				1	TABAGISTA	9	
1	2	2		1	HIV	2	
2	2	1		1	GESTANTE	2	
				1	GESTANTE	2	
						9	
		1					
2	2	2		1	GESTANTE	9	
						9	
2	2	2		2		2	
						9	
						2	
2	2	2		1	GESTACAO	9	
2	2	2		2		9	
2	2	1	33,3	2		9	
						9	
						2	
						9	
				1		9	
2	2	2		1	GESTANTE,	9	
						2	
9	9	9		1	GESTANTE	9	
				1	GESTANTE	2	
						9	
2	2	2		2		9	
2	2	2		2		9	
		1		1	HELLP, CES	9	
2	2	2		2		9	
2	2	2		1	PRE-ECLAM	2	
		1		1	DIABETES (	9	
						2	
2	2	2		1	GESTANTE	1	12/04/2021
2	2	2		1	GESTANTE	9	
						9	
1	2	1		1	HAS, HIPOTIROIDISMO		
2	2	2		1	GESTACAO	2	
				1	HIPERTENS	9	
						2	
						2	
2	2	1		2		9	
						2	

2	2	1		1	HAS GESTA	1	01/04/2020
				1	GESTANTE	9	
						9	
						2	
						2	
						9	
2	2	1		2		2	
2	2	2		1	GESTANTE	2	
						2	
						9	
				1	HAS, ESQU	2	
1	2	2		1	ARTRITE LU	2	
2	2	2		2		2	
2	2	2		2			
						9	
				1	GESTANTE,	9	
2	2	2		1	GESTANTE	9	
						2	
						9	
						9	
2	2	1	33,7	1	GESTANTE	9	
						9	
						9	
						9	
						2	
2	2	2		2		9	
2	2	1		2		2	
						9	
				1	HIPOTIREO	1	
2	2	2		2		2	
2	2	2		1	ECLAMPSIA	9	
						9	
		1					
		1					
						9	
2		1				9	
						2	
						9	
2	2	2		2			
2	2	1	0	1	HIPOTIREO	2	
				1	GESTANTE	9	
		1					
				1	GESTANTE		

						9
						2
9	9	9		1	GESTANTE	
2	2	2		1	MIOPATIA	9
2	2	2		1	GESTANTE	2
						9
2	2	1	0	2		1
2	2	2		1	GESTANTE	2
						2
						1
						9
						2
						2
2	2	2		2		2
				1	GESTANTE	9
2		2		1	GESTANTE	
						2
				1		
				1	GESTANTE	9
2	2	2		2		9
						2
						2
2	2	2		1	GESTANTE	9
2	2	2		1	GESTANTES	9
2				1	HAS	2
						2
2	2	2		2		9
2	2	1	40,7	1		9
						2
		1		1	EPILEPSIA	9
						9
				1	GESTANTE	
2	2	2		1	USUARIA D	9
2	2	2		1	GESTANTE	9
2	2	2		1	HIPOTIREO	9
						9

					2	
					9	
2	2	2	1	DIABETES (	1	16/04/2021
					9	
2	2	2	1	GESTANTE	2	
					9	
			1	HAS, ANEN	9	
					9	
			1	GESTANTE	2	
			1	GESTANTE	9	
			1	HIPOTIREOIDISMO		
					9	
					2	
			1	HAS, GESTANTE, DEPRESSAO		
2	2	2	1	DRGE, HIP(	9	
					2	
2	2	2	1	MENINGIO	2	
2	2	2			9	
2		1	2			
					2	
					2	
			1	HAS		
2	2	2	1	GESTANTE	9	
			1	DHEG		
			1	GESTANTE	2	
					2	
					2	
1	1	2	2			
					9	
					2	
					9	
			1		9	
2	2	2	1	OSTEOPOR	2	
					9	
					9	
					9	

						9	
						1	14/06/2021
2	2	2		2		9	
						9	
						9	
2	2	1		2		9	
						9	
						9	
2	2	1	41,8	2		9	
2	2	2		1	GESTANTE	2	
						9	
2	2	2		1	HIPERTENS	2	
2	2	2		2		2	
						2	
2	2	2		1	GESTANTE	2	
						9	
						2	
						9	
						9	
				1	GESTANTE	1	
2	2	2		1	DMG	9	
						2	
				1	GESTANTE PARTO EM 26/02		
2	2	1				2	
2	2	1		1	GESTANTE	2	
				1	HAS	2	
						9	
2	2	2		1	GESTANTE	9	
2	2	2		2		9	
				1	TABAGISM	9	
						9	
2	2	2		1	DHEG	2	
2	2	2		1	GESTANTE		
2	2	2		1	GESTANTE	2	
2	2	1		2		9	
						9	
2	2	1		1	GESTANTE	9	

				1	GESTACAO	2	
				1	CESARIA EM 23/5 NO ROCHA FARIA		
2	2	1		2		9	
						2	
						2	
						9	
2	2	2		1	DIABETES (	2	
						1	14/04/2021
				1	DEPRESSA(	9	
						2	
		1	39,4				
		1				9	
				1	GESTANTE	9	
						2	
						1	
						2	
						2	
						9	
				1	GESTANTE	9	
		1					
				1	GESTANTE		
				1	GESTANTE	2	
2	2	2		1	HIPOTIREC	9	
						1	04/05/2021
		1				2	
						9	
						9	
				1	GESTANTE	1	
				1	GESTANTE	2	
						9	
						9	
						9	
						9	
2	2	2		1		2	
2	2	2		1	GESTANTE	9	
						9	
						2	
						9	
						9	
2	2	2		1	GESTANTE,	9	
2	2	1		2		2	
						9	
2	2	2		2		9	

2	2			1	OBESIDADI	2	
				1	HAS GESTA	9	
2	2	2		1	GESTANTE	9	
						9	
2	2	2		1	GRAVIDEZ ALTO RISCO		
2	2	2		1	GESTANTE	9	
						9	
				1	GESTANTE		
						9	
						9	
						2	
				1	GESTANTE	9	
		1		1	GESTANTE	2	
		1		1	GESTANTE	2	
						9	
2	2	2		2		2	
						2	
				1	GESTANTE	2	
				1	DIABETES (	9	
2	2	2				9	
						1	
2	2	2					
		1				2	
2	2	2		2		9	
		1		1	EX-TABAGI	9	
2	2	2		1	DIABETES (	9	
						9	
						2	
		1				2	
						9	
						1	30/11/2020
						9	
						2	
						2	
2	2	2		1	TROMBOFI	9	
						9	
				1	GESTANTE	9	
2	2	2		1	GESTANTE	1	01/03/2020
				1	DIABETES (	9	
						9	
2	2	1	39,4	2		1	

						9	
2	2	1	30	2		2	
1	2	2		1	LUPUS ERI	2	
						2	
						9	
2	2	2		1	GESTANTE	2	
2	2	2		1	GESTANTE 28 S +1 D		
2	2	2		1	DMG - SEM	9	
						9	
						9	
						9	
2	2	2		1	GESTANTE	2	
						9	
						2	
2	2	1		2		2	
						1	
2	1	2		2		2	
						2	
2	2	2		1	BRONQUIT	9	
9	9	9		1	GESTANTE,	2	
		1		1	PUERPERA	9	
2	2	2		1	GEATANTE	2	
				1	DUPLA LES	9	
2	2	2		1	GESTANTE	9	
						9	
2	2	2		2		2	
						9	
						1	
						9	
2	2	2		1	HAS, HIPO	2	
9				1	GESTANTE;	9	
						2	
						2	
						1	10/05/2020
				1	GRAVIDEZ	9	
2		2		1	GESTANTE,	9	
2	2	2		1	HIPOTIREOIDISMO		
2	2	2		1	GESTACAO	9	
				1	GESTANTE		
2	2	1		2		2	

2	2	2	1	GESTANTE	9	
2	2	2	1	GESTANTE	2	
					9	
2	2	2	1	RETROVIRO	1	20/11/2020
2	2	2	2		2	
					2	
2	2	2	1	NEFRECTO	9	
					2	
			1	GESTANTE		
2	2	2	1	GESTANTE.	9	
			1	GESTANTE	9	
					9	
2	2	2	2		2	
			1			
2	2	2	2		9	
					9	
					9	
					1	
			1	GESTACAO	9	
					2	
		1			1	
2	2	2	1	GESTACAO	9	
2	2	2	2		9	
					9	
					9	
		1			9	
2	2	1	2		1	02/05/2020
					9	
					9	
					2	
					2	
					9	
					1	
2	2		2		2	
2	2	2	2		9	
2	1	2	2		9	
			1	SD HELLP?	2	

				9
	1			1
				9
				9
				9
				9
		1		2
			1 SIFILIS GES	9
		1		2
2	2	2	2	9
				2
2	2	2	1 GESTANTE	2
				1 28/04/2020
				1 01/03/2020
		1 38		2
2	2	2	1 GESTANTE	2
2	2	1	2	9
				9
				9
2	2	1 32,9	2	9
				9
				2
2	2	2	1 GESTANTE	2
				2
2	2	2	1 GESTANTE	9
2	2	2	1 GESTANTE	2
			1 GESTANTE	2
2	2	2	2	2
				9
2	2	1 48,8	2	1 21/12/2020
				9
				9
				2
2	2	1 31	2	9
2	2	1	1 GESTANTE	9
				9
				2
				9
				1 20/04/2021
2	2	2	1 GESTANTE	

2	2	2		1	HASG	9	
						9	
2	2	1	29,3	1	GESTANTE	9	
2	2	2		1	HAS GESTA	9	
9	9	9		1	GESTANTE	9	
						2	
						2	
						9	
						2	
				1	ENXAQUEC	1	22/04/2021
2	2	2		1	GESTANTE	2	
						9	
				1	GESTANTE		
						9	
2	2	2		1	ANEMIA		
						2	
				1	ECLAMPSIA	2	
						9	
						2	
						2	
						9	
						9	
2	2	2		2		9	
						9	
		1					
						9	
2	2	1	30	2		1	
2	2	2		1	GESTANTE	2	
				1	GIPOPO 37	9	
				1	DEPRESSAO		
	2	2		1	GESTANTE	9	
						2	
						9	
						9	
2	2	2		1	GESTANTE,	2	
2	2	2		1	GESTANTE	2	
						9	
				1	GESTANTE	9	

						2	
				1	GESTANTE	2	
2	2	2		1	3Âº TRIM (	9	
2	2	2		1	GESTANTE	2	
2	2	2		1	TRACO AN	9	
2	2	2		1	GESTANTE	9	
2	2	2		9		9	
						9	
2	2	1	31,2	2		2	
						2	
						9	
2	2	2		1	GESTACAO	1	
2	2	2		1	GESTANTE 30 SEMANAS		
2	2	1		1	HIPOTIREC	9	
						2	
2	2	2		1	HIPOTIREC	2	
						2	
		1				9	
2	2	1		2		2	
2	2	1		2		9	
						9	
						1	31/05/2020
2	2	1		2		9	
		1		1	GESTANTE DE ALTO RISCO		
				1	GESTANTE	1	16/07/2021
				1	GESTANTE	9	
2	2	2		9		2	
						2	
		1					
2	2	2		1	GESTANTE	2	
2	2	2		1	GESTANTE	2	
				1	GESTANTE	9	
						9	
						9	
						2	
						2	
						2	
						2	
						9	

2	2	2		1	HIPOTIREOIDISMO		
2	2	2		2		2	
						2	
		1				9	
2	2	2		1	GESTANTE	1	11/05/2021
						2	
2	2	1		1	GRAVIDA	9	
2	2	2		1	GESTANTE	1	
						9	
						9	
2	2	2		1	GESTANTE	2	
				1	GESTANTE	9	
2	2	1				9	
				1	GESTANTE,	2	
2	2	1		2		9	
				1	HAS, OBES	1	20/04/2021
						2	
						9	
						1	13/04/2021
						2	
2	2	2		1	GESTANTE	2	
2	2	2		1	HAS		
2	2	1	31,2	2		9	
				1	GESTANTE 23 SEMANAS		
2	2	2		2		2	
				1	ANEMIA	9	
2	2	2		2		9	
						9	
						2	
2	2	2		1	OBESIDADI	2	
				1	BRONQUIT	9	
						2	
		1				9	
2	2	2		1	HAS	9	
2	2	2		1	GESTANTE	1	
						1	12/05/2021
2	2	2		1	GESTANTE	1	10/04/2021
				1	GESTANTE		
						9	
2	2	1		2		2	
						9	

				1	GESTANTE		
2	2	2		1	GESTANTE	2	
						2	
	1					9	
						9	
						1	17/05/2021
		1					
2	2	1		2		1	
						9	
		1		1	DIABETES	9	
2	2	2		1	GESTANTE	9	
2	2	2		1	GESTACAO	9	
				9			
2	2	2		1	DMG/GEST	2	
				1	ICC DESCO	9	
		1				2	
2	2	2		2		9	
						1	
		1					
						2	
				1	GESTANTES	9	
2	2	2		1	GESTANTE	9	
				1	GESTACAO	9	
				1	SIFILIS GESTACIONAL		
				1	GESTANTE DE ALTO RISCO		
		1		1	HAS	2	
						9	
2	2	1	30	1	GESTANTE	2	
						9	
2	1	1				2	
2	2	1		2		2	
2	2	2		1	GESTANTE-	2	
2	2	2		1	HIPOTIREO	9	
2	2	2		1	GESTANTE		
						2	
				1	B24	9	
		1				9	
2	1	1		1	HAS	2	
2	2	2		1	GESTANTE	2	

						1	26/04/2021
						2	
2	2	2		1	GESTANTE	1	
						9	
		1	30,5	1	DM GESTA	2	
						9	
				1	HAS,OBESI	2	
2	2	2		1	HAS	2	
				1	GESTANTE		
				1	GRAVIDEZ/	2	
						9	
						9	
2	2	1		1	GESTANTE,	2	
						2	
2	2	2		1	HIPERTENS	9	
						2	
2	2	2		1	GESTANTE	9	
		1		1	HIPOTIREOIDISMO		
						9	
2	2	2		1	GESTANTE,	9	
				1	PNEUMON	2	
						2	
				1	GESTANTE		
2	2	2		1	GESTANTE	2	
				1	GESTANTE 1 TRIMESTRE		
						9	
2	2	2		1	DEPRESSA(	2	
						1	14/04/2021
				1	GESTANTE	9	
				1	TABAGISTA	1	26/05/2021
2	2	1		2		9	
				1	GESTANTE	2	
						9	
						9	

				1	HERNIA DIAFRAG ENCARCERADA		
2	2	2		1	GESTANTE	2	
		1	40	1	DMG/GEST	1	23/04/2021
2	2	2				9	
2	2	1		2		2	
						1	30/04/2021
				1	DIABETES MEILITUS GESTACIONAL		
	1					2	
2	2	2		1	CIFOESCOL	9	
						9	
						9	
		1		1	DMG, HAG	9	
						1	01/06/2021
				1	HIPERTENS	2	
2	2	1	31,1	2		2	
						9	
						2	
						9	
				1	GESTANTE	9	
				1	HIPOTIROI	2	
						9	
2	2	2		1	HIPOTIREC	9	
						2	
						9	
2	2	1	53,9	1	PARTO 26/	9	
						1	04/01/2021
						2	
						1	01/06/2021
2	2	2		1	GESTANTE	9	
						2	
						2	
2	2	2		1	GESTANTE	2	
				1	GESTANTE	2	
				1	GESTANTE		
2	2	2		1	GESTANTE;	9	
2	2	2		1	DM GESTA	9	
						2	
						2	
		1		1	HAS		
						2	

2	2	2	2	9
		1	1 HAS	
				9
				9
2	2	2	2	1
2	2	1		9
				9
				9
2	2	1	2	2
2	2	1 42	1 HIPERTENS	9
			1 GESTANTE	9
				2
				2
				9
			1 HIPOTIREC	9
2	2	2	2	2
		1		9
2	2	1	2	9
2	2	1 40	2	2
				9
2	2	2	1 GESTANTE	2
				9
				2
				2
				2
				1 04/06/2021
				2
2	2	2	1 SOBREPESO	
				2
				2
				2
2	2	1 0	1 HAS	9
				9
				2
			1	

			1	GESTANTE		
					9	
					9	
			1	GESTACAO	2	
					9	
					2	
			1	GESTANTE	9	
					1	06/05/2021
					2	
		1			9	
2	2	2	1	GESTANTE	9	
2	2	2	2			
2	2	2	2		9	
2	2	2	1	HAS	2	
					9	
					9	
					2	
					2	
			1	GESTANTE		
2	2	2	1	GESTANTE	9	
2	2	2	2		1	13/07/2020
2		1			9	
			1	GESTANTE	9	
					1	
		1			9	
2	2	2	1	IRA, INSUF	9	
2	2	2	1	GESTANTE	2	
					9	
2	2	2	1	HIGIDA		
					2	
					9	
2	2	2	1	HAS	2	
					9	
2	2	2	1	GESTANTE	2	
			1	SINDROME	9	
2	2	2	1	GESTANTE	2	
					1	
					2	
			1	GESTANTES	2	

2	2	2	1	GESTANTE	2	
	2		1	DOENCA D	9	
2	2	2	2		2	
					9	
2	2	2	1	HIPOTIREC	9	
2	2	1			9	
					1	07/05/2021
					9	
			1	LESAO REN	2	
2	2	2	1	GESTANTE 3Âº TRIMESTRE		
					9	
					9	
2	2	1	2		1	
					9	
		1	1	GESTANTE,	9	
2	2	2	2		1	
2	2	2	1	GESTANTE,	9	
2	2	2	1	GESTACAO	2	
					9	
					2	
2	2	2	1	DLP, HIPOT	9	
					9	
					2	
					2	
2	2	2	1	TOXOPLAS	2	
					9	
					2	
					1	10/04/2021
2	2	2	1	HAS	9	
					2	
			1	GRAVIDEZ	9	
2	2	2	1	GESTANTE	9	
2	2	2	1	HIPOTIREC	9	
			1	GESTANTE	9	
2	2	2	2		2	
2	2	2	1	GESTANTE	1	
			1	GESTANTE	9	
					9	

		1					
						1	
						2	
2	2	2		2		9	
2	2	2		1	TROMBOFI	2	
2	1	9		2		9	
2	2	2		2		2	
						9	
2	2	2		1	GESTANTE	2	
2	2	2		1	GESTANTE	9	
2	2	1		2		2	
						9	
						1	
2	2	2		1	HIPOTIREC	9	
2	2	2		2		1	13/03/2021
		1		1	OBESIDADI	9	
2	2	2		2		9	
						9	
2	2	2		1	GESTANTE	2	
2	2	2		2		9	
						9	
						9	
2	2	2		9		2	
2	2	2		1	GESTANTE	2	
						9	
				1	GESTANTE	9	
				1	GESTANTE	9	
2	2	2		1	GESTANTE	9	
						9	
		1		1	HAS GESTACIONAL		
2	2	1	31	2		9	
2		2		1	EPILEPSIA?	9	
				1	GESTANTE	2	
2	2	1		1	GESTANTE	1	10/05/2020
				1	GESTANTE	9	
2	2	1		2		9	
		1				2	
				1	GESTANTE	9	
2	2	2		2		2	
						2	
						2	
						9	
2	2	2		1	GESTANTE	9	

2	2	2		2		1	17/06/2021
						9	
2	2	2		2		2	
						9	
2	2	1	36,6	1	GESTANTE,	1	05/10/2020
2	2	2		1	DEPRESSA(	9	
2	2	2		1	GESTANTE	9	
2	2	2		1	GESTANTE	1	10/08/2020
						9	
2	2	2		1	GESTANTE,	9	
						9	
				1	GESTANTE	2	
2	2	2		1	HAS	1	
						9	
2	2	2		1	GESTANTE	9	
2	2	2		2		2	
2	2	2		1	HAS GESTA	2	
2	2	2		1	GESTANTES	1	
						9	
						9	
2	2	2		1	GESTANTE	9	
						9	
2	2	2		1	GESTANTE	9	
		1	36	1	DIABETES (	2	
						2	
2	2	2		1	GESTANTE,	1	20/04/2021
						9	
2	2	2		1	GESTANTE		
						9	
						9	
2	2	2		1	GESTANTE	9	
						2	
				1	OBESIDADI	2	
2	2	2		2		9	
						9	
2	2	2		1	GESTANTE	9	
2	2	2		1	GESTANTE	2	
				1	OBESIDADI	2	
2	2	2		1	GESTANTE	2	
2	2	2		1	GESTACAO	2	
		1					

2	2	1	31	1	TROMBOSI	1	15/05/2020
						9	
				1	GESTANTE	9	
2	2	2		1	GESTANTE 28 SEMANAS		
2	2	2		2		2	
2	2	2		1	HIPERTIRE(	9	
						9	
2	2	2		1	BARIATRIC	2	
						9	
				1	GESTANTE	9	
2	2	1		2		2	
2	2	2		1	GESTANTE	1	16/04/2021
	2	2		1	GESTANTE	2	
		1	40	1	SINDROME	9	
		1					
				1	GESTANTE	9	
2						2	
1	2	2		1	CID B24	2	
2	2			1	GESTANTE	2	
						1	16/08/2021
						2	
2	2	2		1	GESTANTE	1	20/04/2020
2	2	2		1	GESTANTE	2	
						9	
				1	GESTANTE	2	
						2	
2	2	2				9	
						9	
						9	
						1	
2	2	1		1	GESTANTE	2	
2	2			1	GESTANTE	2	
						2	
2	2	2		1	HAS	9	
						2	
2	2	2		1	HAS, PRE-E	2	
2	2	2		2		2	
						9	
2	2	2		1	GESTANTE	2	
						2	
						2	
2	2	2		1	GESTANTE	2	
2	2	2		1	GESTANTE	9	
						1	01/06/2021

				1 04/04/2021
2	2	2	1 GESTANTE	
2	2	2	1 GESTANTE	2
				9
2	2	2	1 GESTANTE	2
				2
			1 HAS, IMG	2
2	2	2	1 GESTANTE	1 06/04/2021
				9
		1		
				1 12/04/2021
2	2	2	1 GESTANTE	
2	2	1 32	2	9
				2
				9
2	2	1 40,4	2	9
				2
			1 GESTANTE	9
2	2	1	2	9
				2
2	2	1 36,7	1 GESTANTE	2
2	2	2	1 GESTANTE	1 03/05/2021
			1 HAS	
			1 HAS	2
		1		
2	2	2	1 HAS, HIPO	9
			1 SIFILIS GES	2
				9
				9
			1 GESTANTE	9
2	2	1	2	
				9
2	2	2	1 GESTANTE	9
				9
			1 GESTANTE	2
				9

2	2	1	26	2		2	
2	2	1				9	
2	2	2		1	GESTANTE	9	
				1	HIPOTIREC	2	
2	2	1		1	HAS GESTA	2	
						2	
		1		1	GESTANTE	9	
						9	
						9	
						9	
				1	HAS		
2	2	2		1	GESTANTE	2	
2	2	2		1	GESTANTE	2	
						2	
						1	30/07/2020
2	2	2		2		9	
						9	
				1	GESTANTE GEMELAR		
2	2	2		1	GESTANTE	9	
						9	
				1	GESTANTE	9	
2	2	2		1	GESTANTE	2	
						2	
				1	GESTANTE	1	13/04/2021
						1	
						1	
						2	
						2	
						2	
				1	HIPOTIREOIDISMO		
						9	
		1		1	BRONQUIT	9	
2	2	2		1	GESTANTE	2	
						9	
2	2	1		2		9	
2	2	2		1	GESTANTE	2	

9	9	1				9	
						2	
2		9		1	DM GESTA	9	
				1	GESTACAO		
2	2	2		1	GESTANTE	9	
				1	MULTIPAR	9	
				1	SOBREPES	9	
						9	
		1		1	HIPOTIRE	1	
						9	
2	2	2				9	
						9	
2	2	2		2		9	
				1	GESTANTE	2	
2	2	2		1	GESTANTE	1	17/04/2021
		1				1	
						2	
						2	
		1				1	30/04/2021
						2	
2	2	2		1	DHEG+GES	2	
						2	
2	2	2		1	HAS		
						9	
2	2	2		2		9	
2	2	2		1	GESTANTE	9	
2	2	2		1	GESTNTE, E	9	
2	2	2		1	GESTANTE	2	
2	2	1	49,5	1	GESTANTE,HAS,HIPOTIROIDISMO		
2	2	2		1	DIABETES	2	
2	2	2		2		2	
						9	
2	2	1		2			
						9	
						9	
				1	GESTANTE	9	

2	2	2		1	GESTANTE	2	
						9	
						9	
						9	
				1	GESTANTE	2	
		1		1	LES, USO D	9	
						1	15/05/2021
				1	GESTANTE	9	
						2	
2	2	2		2		9	
2	2	2		1	HIPERTENS	9	
				1	GESTANTE	9	
						2	
						9	
						2	
						9	
2	2	1		2		9	
		1					
				1	PRE-ECLAMPSIA NA ULTIMA GESTAC		
		1				9	
2	2	2		2		2	
2	2	1	32	1	HIPERTENS	9	
						2	
						1	19/04/2021
2	2	1		1	HIPOTIREC	9	
2	2	2		1	DM GESTA	9	
		1		1	SANGRAMENTO VAGINAL A/E		
						9	
						2	
						9	
		1	35			2	
				1	GESTANTE 35 SEMANAS		
						9	
						1	22/04/2021
						2	
						9	
						9	
2	2	1		2		2	
		1		1	GESTANTE	9	
						9	
						9	

					9
					9
					2
2	2	2	1	GESTANTE	9
2	2	2	2		9
					2
	1	1	1	GESTANTE,	9
2	2	2	2		9
2	2	2	1	CEFALEIA,	2
					2
2	2	2	1	GESTANTE	9
					9
	2		1	HIPOTIREO	9
					9
2	2	2	1	SAAF	9
					9
			1	GESTANTE	9
					9
					9
					2
2	2	2	1	HIPOTIREO	2
					9
			1	GESTANTE	9
2	2				
		1			2
2	2	1	1	PIELONEFR	9
					9
					9
			1	GESTANTE	
			1	HIPOTIREO	2
2	2	2	1		1
					9
2	2	2	2		9
					9
		1			9
2		2	1	TROMBOFI	2
2	2	2	1	HAS	9
					9
9	9	9	1	GESTANTE	9

						2
						9
						9
						2
						9
2	2	2		2		9
1	2	2		1	HIV, NEUR	2
				1	HIPOTIREC	2
						2
						2
2	2	1		2		9
2	2	2		1	GESTANTE	9
		1				
		1	29,4			2
2	2	1		2		
2	2	2		1	GRAVIDA D	2
						9
2	2	2		2		9
				1	GESTANTE	2
2	2	2		1	GESTANTE	2
2	2	2		1	ENCEFALO	2
2	2	2		1	GESTANTE	
2	2	2		2		2
				1	GESTANTE	
2	2	1		1	HAS, GESTANTE 2ÂºTRIMESTRE	
						9
						2
2	2	2		1	DM GESTA	9
2	2	2		2		9
2	2	2		2		2
				1	DOENCA H	9
2	2	2		1	TOXOPLAS	9
						2
2	2	2		1	HIPERTIRO	9
2	2	2		1	GESTACAO	1
2		2		1	GESTANTE	9
2	2	2				2
						9
						9
2	2	2		1	GESTANTE	9

				1	GESTANTE 26 SEMANAS		
				1	ALERGIAS:	9	
				1	GESTANTE		
				1	GESTANTE		
						9	
2	2	1		1	GESTANTE	1	
						1	
1	2	1		1	EX TABAGI	1	15/06/2021
						9	
						2	
2	2	2		1	DMG+ITU	9	
2	2	1	30,1	2		9	
2	2	2		1	GESTANTE	1	
						9	
2	2	1		2		2	
2	2	2		1	GESTANTE	2	
						9	
						2	
2	2	2		1	OBESIDADI	9	
						2	
2	2	2		1	GESTANTE	1	
2	2	2		1	GESTANTE,	1	
2	9	2		9		1	03/02/2021
						9	
		1		1	DMG	2	
						9	
				1	GESTANTE	2	
						9	
2	2	2		1	HAS	1	
2	2	2					
						9	
						1	02/04/2020
						9	
						1	18/10/2020
				1	GESTACAO	2	
						9	
				1	PNEUMON	9	
						9	
2	2	1	37,4	2		2	
2	2	2		1	GESTANTE	9	
						9	
2	2	2		1	GESTANTE	2	

2	2	1	39	1	GESTANTE	1	15/04/2020
2	2	2		1	HAS	2	
						9	
						9	
						9	
						2	
						9	
				1	HIPOTIROI	1	
						9	
						2	
						9	
2	2	2		1	GESTANTE	9	
		1				9	
2	2	2		1	DM GESTACIONAL		
						9	
1				1	LUPUS		
2	2	2		2		9	
2	2	2		1	GESTANTE	2	
2	2	2		1	GESTANTE	9	
2	2	2		1	GESTANTE	9	
2	2	2		1	GESTANTE	2	
						2	
2	2	1		2		9	
		2		1	GESTANTE	9	
				1	GESTANTE	9	
2	2	1		2		2	
						9	
						2	
2	2	2		2		9	
						2	
2	2	2		1	GESTANTE	2	
				1	GESTANTE	2	
2	2	2		1	HAS	2	
				1	CARCINOM	9	
				1	GESTANTE	2	
						9	
						2	
						9	

						2	
				1	HAS		
				1	GRAVIDEZ	2	
2	2	2		2		2	
						9	
				1	GESTANTE		
		1		2		2	
						2	
						1	
		1	51,7			2	
		1		1	HAS	9	
						9	
						2	
		1				9	
						9	
2	2	2		1	GESTANTE	9	
						9	
2	2	2		1	GESTANTE	9	
2	2	2		1	GESTANTE	9	
						9	
		1				2	
2	2	2		1	GESTANTE	2	
						1	28/05/2020
2	2	1		2			
2	2	2		2		1	
				1	ASMA, DEF	9	
						9	
						2	
2	2	2		1	GESTANTE	2	
						9	
2	2	2		1	GESTANTE	9	
2	2	1	31,9	2		9	
2	2	2		1	GRAVIDEZ	2	
		1				9	
				1	HIPERTENS	1	24/12/2021
						2	
						2	
2	2	1	33,3	1	GESTANTE	2	
				1	GESTACAO		

						9	
						9	
				1	GESTANTE	9	
						2	
				1	PNEUMON	9	
						9	
2	2	2		2		2	
				1	GESTANTE 25 SEMANAS		
						9	
						2	
						9	
2	2	2		1	GESTANTE	1	
						9	
						9	
						9	
2	2	2		1	GESTANTE	9	
						1	01/04/2021
		1				2	
						9	
						2	
						2	
2	2	2		2		2	
						9	
2	2	2		1	GESTANTE 17S		
				1	GESTANTE	9	
2	2	1		1	HIPOTIREC	9	
				1	GESTANTE	9	
2	2	2		1	GESTANTE	1	06/05/2021
2	2	1		1	HAS	2	
2	2	1	40	1	GESTANTE	2	
2	2	2				2	
2	2	2		2		1	10/04/2020
						9	
				1	GESTANTE	9	
				1	GESTANTE		
						2	
2	2	2		1	GESTANTE	1	12/04/2021
2	2	2		1	SINDROME	9	
9	9	9		1	GESTANTE		
						9	
						2	
		1		1	GESTANTE	1	26/05/2021

		1			9	
2	2	2	2		2	
2	2	1	1	GESTANTE	2	
2	2	2	1	HAS,DHEG	9	
					1	20/05/2021
			1	OBESIDADI	2	
					2	
			1	GESTANTE 3ÂºTRI		
			1	GESTANTE	2	
					2	
			1	PRE ECLAM	9	
2	2	1	1	HIPERTENS	9	
			1	GESTANTE	9	
					1	10/05/2020
					1	20/04/2020
					2	
			1	GESTANTE		
			1	GESTANTE	2	
					9	
					2	
2	2	2	1	GESTANTE	2	
					9	
2	2	2	1	GESTACAO	9	
					9	
		1			1	27/04/2021
					2	
					2	
					9	
					2	
					9	
					2	
			1	GRAVIDEZ	9	
					9	
2	2	2	1	TABAGISM	9	
2	2	2	1	GESTANTE	9	
					9	
					9	
					2	
			1	GRAVIDEZ	1	04/04/2020
			1	PRE ECLAM	9	

	2	1	2		9	
					2	
					9	
					9	
					9	
					9	
					2	
2	2	2	1	GEST 29 SE	1	26/04/2021
2	2	2	2		2	
					2	
					2	
			1	GESTANTE	2	
					1	01/05/2021
					9	
2	2	9	2		2	
			1	GESTANTE	9	
			1	HIPERTIROIDISMO		
2	2	2	2		9	
9	9	1	1	GESTANTE	9	
					2	
					2	
					9	
2	2	2	1	DHEG,OBE	9	
			1	GESTANTE	9	
					9	
					2	
			1	GESTANTE		
2	2	2	1	DIABETES	9	
					2	
					1	15/04/2021
2	2	2	1	HAS	2	
					9	
2	2	2	2		9	
					9	
					2	
2	2	1	1	TABAGIST	1	21/04/2021
	1		1	GESTANTE	2	
					9	
2	2	2	1	SOPRO CA	2	

2	2	2		1	DMG	9	
						9	
2	2	2		1	OBESIDADI	9	
				1	HIPOTIREI(	9	
2	2	2		1	GESTANTE	2	
						9	
						2	
						1	
2	2	2		1	GESTANTE	9	
						9	
						9	
2	2	1		1	GESTANTE, HIPOTIROIDISMO		
						1	18/05/2021
						9	
2	2	2		1	GESTANTE	2	
2	2	1		2		2	
						2	
		1				9	
2	2	2		2		2	
1	2	2		1	GESTANTE	9	
2	2	2		2		2	
						9	
1	2	2		1	HIV	2	
						1	15/04/2020
2	1	1		2		9	
						9	
		1				9	
						2	
						9	
2	2	2		2		9	
1	2	2		1	GESTANTE	2	
						1	26/03/2020
						9	
				1	GESTANTE	2	
2	2	1	36,3	2		9	
						2	
						2	
2	2	2		1	GESTANTE	1	
				1	GESTANTE DE 11 SEMANAS		
2	2	1		1	GESTANTE	2	
2	2	2		1	GESTANTE	2	
2	2	2		1	HAS		
				1	PIELONEFR	9	
		1		1	TABAGIST/	1	22/04/2021
				1	GESTANTE		

						9	
						2	
2	2	2		1	GESTACAO	1	13/01/2021
						1	
						2	
						2	
						2	
						9	
				1	GESTANTE	1	
						9	
2	2	2		1	GESTACAO	2	
2	2	2		1	GESTANTE	2	
2	2	2		1	HAS	9	
						9	
						9	
						2	
				2		2	
2	2	2		1	GESTANTE	2	
2	2	2		1	GESTANTE	2	
						9	
						9	
2	2	2		1	GESTANTE	2	
						2	
2	1	2		1	GESTANTE	9	
						2	
						9	
						2	
				1	GESTANTE	9	
						2	
2	2	2		1	GESTANTE	9	
						9	
						9	
2	2	1	34,8	2		9	
2	2	2		1	GESTANTE		
						9	
2	2	2		1	GESTANTE	1	12/04/2021
						9	
						9	

				2
2		2	2	9
				9
				2
2	2	1 43	1 HAS	2
				2
				2
			1 GESTANTE	
				9
				1 15/05/2020
2	2	2	2	9
		1		
			1 GESTANTE 34 SEMANAS	
			1 GESTANTE	9
				9
		1		9
				2
2	2	2	2	1 23/03/2020
				1 25/06/2020
				2
	2	2	1 GESTANTE	9
				1 13/04/2021
				9
				2
2	2	2	2	2
				2
		1		9
				9
				9
				9
				2
				2
2	2	2	1 PRE-ECLAM	9
				9
2	2	2	2	2
			1 GESTANTE	
			1 PRE- ECLAI	9
2	2	2	1 GESTANTE	9

					9	
2	2	2	1	GESTANTE	1	10/05/2021
9	9	9	1	GESTANTE	9	
2	1	2	1	HAS, GEST	9	
					9	
2	2	2	2		9	
					9	
2	2	1	2		2	
					2	
					9	
			1	HAS	2	
			1	GESTANTE	2	
					9	
2	2	2	1	GESTANTE	9	
2	2	2	1	GESTANTE	2	
			1	SD PANICO	2	
					9	
2	2	2	1	GESTANTE	2	
					1	10/04/2021
					9	
			1	PUERPERA	9	
2	2	2	2		9	
2	2	1	2		9	
			1	GESTANTE,	2	
					1	
					1	28/05/2021
					9	
2	2	2	2		9	
					2	
					9	
					9	
					1	
2	2	2	1	GESTANTE	2	
2	2	2	1	ALERGIA A	2	
					9	
2	2	2	1	HIPOTIREO	2	
2	2	2	2		9	
			1	DOENCA H	9	
2	2	2	1	HAS	9	
					2	
			1	DHEG	9	
2	2	2	1	GESTANTE	9	
	2	1	1	HAS	2	

						2	
2	2	1		2		9	
2	2	2		1	AGITACAO	2	
		1		1	GESTANTE	9	
2	2	2		1	GESTANTE	9	
2	2	2		1	GESTANTE	1	19/04/2021
2	2	2		1	GESTANTE	2	
2	2	2		1	GESTANTE	1	30/04/2021
						9	
2	2	1		2		9	
2	2	1	32	1	GESTANTE	2	
						1	21/04/2021
2	2	2		1	GESTANTE.	2	
2	2	2		1	GESTANTE	2	
						2	
				1	GESTACAO	2	
						2	
2	2	2		1	GESTANTE	2	
						9	
2	2	1		1	DMG	9	
						9	
2	2	1	32	2		9	
2	2	2		1	GESTANTE	2	
2	2	2		1	GESTANTE	2	
2	2	2				9	
						9	
				1	PO CESARE	2	
2	2	2		1	LINFOMA	2	
				1	GESTANTE	2	
2	2	1	0	1	ALCOLISM	2	
2	2	2		1	PRE ECLAMPSIASINDROME DE HELPP		
2	2	2		1	GRAVIDA;	9	
				1	HAS GESTA	2	
2	2	2		1	3TRIMESTRE GESTACIONAL		
2	2	1	35,4	2		2	
2	2	2		1	GESTANTE	2	
						2	
2	2	2		1	GESTANTE	2	
2	2	1		1	HAS GESTA	2	
						2	

		1		1	DM GESTA	9	
				1	GESTANTE	9	
				1	GESTANTE	2	
						9	
						2	
						9	
						9	
						2	
		1		1	H A C + GESTANTE		
				1	ASMATICA	2	
				1	GESTANTE	9	
						1	
						9	
2	2	2		1	GESTACAO	9	
2	2	1		1	TABAGISTA	1	01/05/2020
						9	
2	2	2		1	HAS, COVI	9	
						9	
						9	
		1		1			
2	2	2		1	GESTANTE	2	
						2	
						9	
						1	
2	2	1	33,5	2		9	
						9	
2	2	2		1	GESTANTE	2	
		2					
						2	
						2	
2	2	1		1	GASTRITE	9	
						2	
2	2	2		1	DHEG	9	
						9	
2	2	2		1	HAS	9	
						9	
2	2	1	30	2		2	
2	2	2		1	HIPOTIREO	9	

						9	
		1		1	SD HELLP	2	
2	2	2		2		1	24/04/2021
2	2	1		1	HIPOTIREC	2	
		1	31			1	
						2	
2	2	2		2		2	
2	2	2		2		2	
						2	
2	2	1	31	2		1	01/02/2021
				1	GESTANTE		
2	2	2		2		9	
2	2	2		2		9	
				1	DIABETES I	2	
2	2	2		2		9	
						2	
						9	
2	2	2		1	GESTANTE	9	
2	2	2		1	PSIQUIATR	2	
2	2	2		1	GESTANTE	2	
2	2	2		1	GESTANTE	1	05/05/2021
						2	
				1	OBESIDADI	2	
2	2	2		1	EPILEPSIA	1	16/04/2021
						9	
2	2	1		1	HAS PREVI	1	15/04/2020
		1	34,6			9	
	1						
						9	
						2	
2	2	2		1	GESTANTE	1	
2	2	2		2		9	
						9	
						1	06/05/2021
				1	GESTANTE	9	
						1	19/04/2021
						2	

				9
2	2	2	2	2
2	2	2	1 HIPOTIREC	9
			1 HIPOTIREOIDISMO	
			1 GESTANTE	9
2	2	2	2	2
				2
				9
				2
2	2	1		9
				9
			1 DHEG, HAC	9
				9
2	2	2	1 DM GESTA	9
				9
2	2	2	1 HIPOTIREC	1 03/05/2021
				9
				9
2	2	2	1 GESTANTE	2
2	2	1	1 GESTANTE	2
2	2	2	2	9
		1		2
				2
2	2	2	1 TABAGISM	1 09/06/2021
				9
2	2	1	2	1 28/04/2021
				9
			1 GESTANTE	9
2	2	2	1 GRAVIDEZ	
2	2	2	1 GESTANTE	2
2	2	1	2	9
2	2	2	2	9
				2
				2
				9
		1	2	2
				2
2		1	1 VASCULOP	9
				9
2	2	1	2	9
				2

2	2	1					2	
							9	
							9	
							1	
				1	HIPERTENS		9	
				1	GESTACAO		9	
2	2	1		1	HAS NA GE		9	
2	2	1		2			9	
2	2	2		1	HIPOTIREC		9	
				1	GESTANTE		9	
2	2	2		2			9	
2	2	1		2			1	
2	2	2		1	DIABETES (		2	
				1	HIPOTIREOIDISMO			
							9	
2	2	2		2			2	
							9	
2	2	2		1	DMG			
							1	
2	2			1	HAS+GESTA		9	
2	2	2		1	DM GESTA		9	
							2	
							9	
2	2	2		2			2	
							9	
2	2	2		2			2	
				1	GESTANTE	31 SEMANAS		
				1	OBESIDADI		1	10/06/2020
							2	
2	2	2		2			9	
2	2	2		2			9	
2	2	2		1	DM GESTA		9	
		1	33,3	1	GESTANTE		2	
2	2	2		1	GESTANTE		2	
	9	9		1	GESTANTE			
2	2	2					9	
				1	GESTANTE		9	
2	2	2		1	GESTANTE		9	
							9	
				1	GESTANTE,		1	22/04/2020

		1		1	HAS DURA	9
2	2	2		1	GESTANTE	2
2	2	2		2		9
				1	OBESIDADE	
						9
2	2	2		2		2
2	2	2		1	GESTANTE	2
2	2	2		1	HA GESTAC	9
2	2	1		1	ANSIEDAD	9
2	2	1		1	GESTANTE	9
2	2	1	34,4	2		9
2	2	2		2		9
2	2	1		1	DMG	1
						2
						2
2	2	2		2		9
		1				
				1	GESTACAO	2
						9
						9
						9
				1	LITIASE UR	2
2	2	2		1	GESTANTE	9
						9
						9
						2
2	1	2		1	GESTANTE	2
						9
9	9	9		9		1
2	2	2		1	GESTANTE.	
						2
2	2	2		2		2
				1	HIPERTENS	2
2	2	2		2		2
				1	GESTANTE	
2	2	1		2		9
						2
						9
2	2	2		2		9

2	2	2		1	HAS		
				1	GESTANTE	9	
2	2	2		1	GESTANTE	9	
2	2	1		2		1	05/05/2021
2	2	2		1	HAS	2	
2	2	1		2		2	
		1	37,5	1	GESTANTE,	9	
						2	
2	2	1		2			
2	2	2		1	GESTANTE	9	
						2	
						9	
						9	
				1	GEST 37 SE	2	
						2	
						2	
						9	
2	2	2		2		9	
						2	
2	2	2		1	GESTANTE	2	
2	2	2		2		2	
				1	GESTANTE	9	
						9	
						9	
						9	
						2	
2	2	2		2		2	
2	2	2		1	TU CEREBR	2	
				1	PNEUMON	9	
						2	
2	2	2		1	DIABETES (	9	
				1	GESTANTE	9	
						2	
						9	
2	2	2		1	GESTANTE	9	
2	2	2		1	GESTANTE	2	
						9	
						9	

	1	1	30			9	
						2	
		1		1	GESTANTE	1	
				1	GESTANTE	2	
						9	
						9	
				1	HIPOTIREOIDISMO		
2	2	2		1	HIPOTIREO	9	
						9	
				1	GESTANTE	9	
						2	
				1	HIPOTIREO	9	
2	2	1		1	GESTANTE	2	
2	2	2		2		2	
				1	GESTANTE	9	
2	2	2		1	GESTANTE	2	
2	2	2		1	GESTANTE	1	
2	2	1		2		9	
2	2	1		1	GESTANTE	1	
						2	
				1	CA DE MAMA TRATADO		
				1	HAS		
		1	31			9	
2	2	2		1	GESTANTE	9	
						2	
		1				2	
						1	12/05/2021
						2	
2	2	2		1	GESTANTE GEMELAR		
				1	GESTANTE	9	
2	2	1	0	1	GESTANTE	2	
						2	
2	2	2		2			
2	2	1		2		2	
						9	
2	2	2		2		9	
						9	
2		2		1	GESTANTE	2	
						2	
2	2	2		2		2	

2	2	2	1	TROMBOL	2	
					9	
			1	GESTANTE		
					9	
					2	
					2	
					9	
2		1			9	
		1	1	GESTANTE		
					9	
					2	
			1	DHEG	9	
		1			9	
2	2	2	1	GESTANTE	2	
2	2	2	2			
			1	GESTANTE	2	
					1	12/04/2021
			1	GESTANTE		
					9	
2	2	2	2		2	
					2	
2	2	2	1	ARTRITE RE	9	
					9	
2	2	2	2		2	
					9	
					2	
					2	
					9	
2	2	1	1	GESTANTE	9	
2	2	1	2		9	
	2	2			9	
					2	
			1	SHEG	2	
					2	
			1	DM GESTACIONAL + HIPOTIREOIDIS		
					9	
2	2	2	1	GESTANTE		
					9	

2	2	2	1	TUBERCUL	2
2	2	2	1	HAS	2
2	2		1	TABAGISTA ,GESTANTE	
2	2	2	1	GESTANTE	9
					2
2	2	2	2		
2	2	2	2		
	1		1	DHEG	
			1	GESTANTE	2
		1			9
2	2	2	2		
			1	HAS/HIPOTIREDISMO	
					2
2	2	2	1	DIABETES GESTACIONAL HAS	
1	2	2	2		9
2	2	2	1	HAS	2
2	2	2	1	GESTANTE	2
					1
2	2	2	2		
2	2	2	1		2
2	2	2	1	GESTANTE,	2
2	2	2	2		2
2	2	2	1	GESTANTE	9
2	2	2	2		9
					9
					9
1	2	2	1	GEST ALTO RISCO POLIRADICULONE	
					9
			1	GESTANTE	
2	2	1	2		2
			1	TROMBOFILIA	

2	2	2	1	DMG	9	
					9	
					9	
					2	
2	2	2	2		9	
2	2	2	1	HIPOTIREC	1	15/04/2021
			1	GESTANTE		
					9	
					9	
			1	LLA		
					9	
2	2	2	1	GESTANTE	9	
					9	
2	2	2	1	GESTANTE		
2	2	2	2		9	
2	2	2	2		2	
			1	DIABETES GESTACIONAL		
2	1	2	1	GESTANTE	9	
			1	GESTANTE	9	
					2	
					2	
			1	HIPERTENS	2	
		1			9	
					9	
					2	
2	2	2	1	GESTANTE	9	
			1	GESTANTE	9	
					1	
2	2	2	2		2	
2	2	1	1	GESTANTE	9	
					9	
					2	
2	2	2	1	GESTANTE	2	
			1	GESTANTE	9	

						2	
2	2	2		2		2	
2	2	2		1	HIPOTIROI	1	31/03/2021
						9	
						1	
2	2	1	29,7			9	
2	2	2		1	GESTANTE	2	
2	2	2		1	GESTANTE	1	
						9	
2	2	9		1	GESTANTE	1	15/07/2021
						9	
	2	2		1	GESTANTE	1	17/09/2021
2	2	2		1	HAS	2	
						2	
2	2	1	32	2		1	10/11/2021
		1		2		1	
2	2	2		1	HAS GESTA	2	
2	2	2		1	GESTANTE	2	
		1				1	16/07/2021
						9	

_AMAMENT	DT_DOSEUN	DT_1_DOSE	DT_2_DOSE	ANTIVIRAL	TP_ANTIVI	OUT_ANTI	DT_ANTIVI	HOSPITAL	DT_INTERN
				9				1	23/06/202
				1	1		07/01/202	1	08/01/202
								1	22/12/202
				2				1	23/04/202
				1	1		20/07/202	1	17/07/202
				2				1	02/08/202
				2				1	03/08/202
				2				1	14/09/202
								1	22/09/202
				1	1			1	06/05/202
				9				1	16/08/202
				2				1	04/12/202
				1	1		05/01/202	1	05/01/202
								1	05/12/202
				2				1	30/11/202
				2				1	14/05/202
				2				1	03/06/202
				2				1	06/07/202
				2				1	15/07/202
				2				1	11/09/202
								1	27/11/202
				9				1	19/12/202
				2				1	29/05/202
				2				1	08/06/202
				1	1		29/04/202	1	29/04/202
				2				1	03/06/202
				1				1	11/06/202
				2				1	25/11/202
				2				1	19/10/202
				2				1	07/09/202
				1	1		09/05/202	1	09/05/202
				1	1		10/06/202	1	10/06/202
				9				1	06/10/202
				2				1	03/11/202
				2				1	31/12/202
				1	1		23/06/202	1	23/06/202
				1	1		18/08/202	1	18/08/202
				2				1	23/06/202
				2				1	19/07/202
				2				1	07/08/202
				2				1	09/01/202
				1	1		26/04/202	1	26/04/202
				2				1	01/05/202
				1	1		09/04/202	1	09/04/202
				1	1			1	23/03/202
				2				1	09/08/202

2			1	24/06/202
1	1	26/04/202	1	24/04/202
2			1	17/06/202
1			1	04/06/202
1	1	03/07/202	1	02/07/202
			1	27/04/202
2			1	18/11/202
9			1	27/12/202
2			1	08/05/202
2			1	31/07/202
2			1	15/06/202
1	1	03/08/202	1	03/08/202
9			1	19/07/202
1	1	13/08/202	1	13/08/202
2			1	25/11/202
9			1	10/11/202
			1	25/05/202
			1	06/05/202
2			1	15/06/202
2			1	10/09/202
2			1	17/10/202
2			1	10/05/202
2			1	04/01/202
1			1	30/05/202
2			1	23/06/202
2			1	29/06/202
2			1	27/07/202
2			1	27/07/202
1	1	17/09/202	1	17/09/202
2			1	01/08/202
			1	28/03/202
9			1	30/04/202
1	1	14/04/202	1	11/04/202
2			1	19/07/202
1	1	16/06/202	1	16/06/202
2			1	05/07/202
9			1	27/10/202
2			1	12/12/202
9			1	11/12/202
2			1	23/12/202
2			1	29/04/202
			1	23/04/202
1	1	01/07/202	1	01/07/202
1	1	29/07/202	1	29/07/202
2			1	01/08/202
2			1	16/11/202
2			1	10/11/202

2			1	01/10/2020
2			1	25/12/2020
2			1	20/04/2020
1	1	21/04/2020	1	19/04/2020
2			1	15/05/2020
1	1	20/06/2020	1	20/06/2020
1	1	01/07/2020	1	25/06/2020
2			1	17/08/2020
2			1	05/07/2020
9			1	07/04/2020
2			1	17/05/2020
2			1	25/12/2020
1	1	28/04/2020	1	28/04/2020
2			1	30/06/2020
2			1	22/04/2020
2			1	12/06/2020
2			1	14/06/2020
2			1	08/09/2020
2			1	10/07/2020
2			1	19/10/2020
2			1	12/07/2020
2			1	01/07/2020
9			1	11/09/2020
2			1	09/09/2020
1	1		1	26/04/2020
1	1	19/05/2020	1	19/05/2020
2			1	19/06/2020
2			1	11/07/2020
1	1	20/10/2020	1	20/10/2020
1	1	05/12/2020	1	06/12/2020
			1	10/12/2020
1	1	01/05/2020	1	01/05/2020
1	1	12/06/2020	1	13/06/2020
2			1	01/12/2020
2			1	15/07/2020
			1	20/08/2020
2			1	01/07/2020
1	1	26/10/2020	1	23/10/2020
1	1	10/05/2020	1	11/05/2020
2			1	02/09/2020
			1	08/12/2020
2			1	16/12/2020
			1	29/03/2020
2			1	24/06/2020
2			1	17/07/2020
2			1	18/09/2020
2				

2			1	30/10/202
2			1	28/06/202
2			1	02/12/202
2			1	10/10/202
2			1	23/11/202
2			1	11/05/202
2			1	01/05/202
2			1	12/04/202
1	1	29/06/202	1	30/06/202
2			1	22/08/202
9			1	01/05/202
2			1	11/05/202
2			1	29/12/202
2			1	18/12/202
2			1	02/06/202
2			1	01/06/202
2			1	14/07/202
2			1	16/12/202
1	2	30/06/202	1	25/06/202
2			1	18/05/202
2			1	22/09/202
2			1	28/11/202
2			1	27/04/202
1	1	26/05/202	1	26/05/202
2			1	06/05/202
9			1	22/07/202
9			1	08/07/202
9			1	26/08/202
1	1	29/09/202	1	29/09/202
9			1	07/09/202
2			1	08/01/202
2			1	01/12/202
1	1	26/12/202	1	26/12/202
2			1	05/01/202
1	1	16/06/202	1	16/06/202
2			1	13/12/202
1	1	02/05/202	1	05/05/202
2			1	14/05/202
9			1	12/06/202
2			1	20/06/202
9			1	12/06/202
2			1	26/08/202
2			1	25/04/202
2			1	13/05/202
1	1	22/04/202	1	22/04/202
1	1	19/04/202	1	10/04/202
2			1	05/06/202

2			1	24/06/202
9			1	17/07/202
2			1	09/08/202
2			1	15/09/202
2			1	18/09/202
1	1	11/09/202	1	11/09/202
2			1	10/05/202
2			1	16/12/202
2			1	07/06/202
2			1	10/04/202
2			1	27/06/202
2			1	10/08/202
2			1	31/12/202
1	1	05/06/202	1	06/06/202
			1	30/04/202
2			1	14/07/202
2			1	31/07/202
2			1	04/12/202
9			1	16/06/202
2			1	11/07/202
			1	14/08/202
2			1	17/10/202
2			1	19/11/202
			1	13/11/202
2			1	11/06/202
9			1	16/07/202
2			1	20/08/202
			1	23/07/202
2			1	16/10/202
1	1	28/05/202	1	27/05/202
2			1	28/05/202
2			1	05/07/202
9			1	26/12/202
2			1	13/07/202
1	1	08/09/202	1	08/09/202
			1	14/05/202
2			1	10/08/202
2			1	09/07/202
2			1	10/06/202
1	1		1	17/11/202
			1	19/04/202
2			1	16/07/202
1	1	04/04/202	1	31/03/202
9			1	15/04/202
2			1	15/07/202
9			1	07/01/202
2			1	10/07/202

9			1	24/06/202
2			1	27/12/202
2			1	03/01/202
2			1	25/12/202
2			1	24/04/202
2			1	03/06/202
2			1	06/06/202
2			1	20/07/202
2			1	27/08/202
2			1	15/12/202
2			1	13/12/202
2			1	30/09/202
2			1	22/12/202
2			1	02/05/202
2			1	05/11/202
1	1	04/07/202	1	03/07/202
2			1	07/07/202
1	1	20/05/202	1	20/05/202
2			1	15/10/202
2			1	21/04/202
			1	25/07/202
			1	05/12/202
			1	11/01/202
2			1	29/09/202
2			1	21/04/202
1			1	29/11/202
9			1	13/07/202
2			1	23/07/202
2			1	17/12/202
2			1	01/01/202
2			1	14/05/202
9			1	21/04/202
2			1	18/06/202
1	1	31/05/202	1	30/05/202
2			1	13/01/202
2			1	12/06/202
9			1	17/09/202
2			1	19/11/202
1	1	12/05/202	1	12/05/202
			1	01/05/202
2			1	23/05/202
9			1	24/07/202
2			1	18/06/202
2			1	22/06/202
9			1	17/12/202
2			1	27/12/202
2			1	31/03/202

2			1	23/05/2020
2			1	02/06/2020
2			1	23/06/2020
2			1	04/07/2020
9			1	15/05/2020
2			1	11/08/2020
1			1	25/08/2020
9			1	22/03/2020
2			1	09/01/202
1	1	24/03/2020	1	24/03/2020
2			1	16/05/2020
2			1	24/12/2020
2			1	02/12/2020
2			1	05/07/2020
2			1	10/12/2020
1	1	30/07/2020	1	30/07/2020
2			1	30/09/2020
9			1	08/01/202
2			1	01/07/2020
2			1	20/07/2020
1	1	14/07/2020	1	17/07/2020
2			1	12/11/2020
1	1	16/07/2020	1	19/07/2020
			1	13/12/2020
2			1	30/12/2020
2			1	28/04/2020
9			1	09/10/2020
2			1	16/06/2020
1			1	20/06/2020
			1	28/10/2020
2			1	18/11/2020
2			1	05/07/2020
2			1	08/04/2020
2			1	29/04/2020
2			1	12/01/202
2			1	
9			1	30/04/2020
9			1	23/03/2020
1	1	02/06/2020	1	29/05/2020
2			1	18/06/2020
2			1	25/06/2020
2			1	09/06/2020
9			1	10/07/2020
2			1	19/07/2020
2			1	05/07/2020
2			1	22/05/2020
2			1	20/08/2020

1	1		19/08/2020	1	17/08/2020
9				1	04/11/2020
2				1	25/10/2020
1	1		06/09/2020	1	06/09/2020
1	1			1	16/04/2020
1	1		15/04/2020	1	15/04/2020
9				1	06/06/2020
1	1		27/05/2020	1	28/05/2020
9				1	16/09/2020
2				1	10/11/2020
2				1	27/06/2020
1	1		03/04/2020	1	02/04/2020
1	1		02/07/2020	1	06/07/2020
1	1		16/05/2020	1	17/05/2020
2				1	24/06/2020
2				1	29/07/2020
2				1	11/08/2020
2				1	05/05/2020
2				1	31/12/2020
				1	04/11/2020
1	1		19/05/2020	1	19/05/2020
9				1	06/05/2020
1	1		04/06/2020	1	04/06/2020
2				1	01/06/2020
2				1	21/08/2020
2				1	20/05/2020
9				1	30/12/2020
1	1		16/04/2020	1	16/04/2020
1	1		23/06/2020	1	23/06/2020
2				1	16/06/2020
9				1	14/08/2020
1	1		13/09/2020	1	17/09/2020
2				1	31/07/2020
2				1	07/08/2020
1	1		14/08/2020	1	14/08/2020
9				1	28/11/2020
1	1			1	27/09/2020
				1	04/01/202
1	1		28/04/2020	1	28/04/2020
1	3	azitromicir	17/10/2020	1	27/10/2020
1	1		06/05/2020	1	06/05/2020
2				1	13/07/2020
2				1	05/08/2020
1	1		18/05/2020	1	18/05/2020
9				1	04/06/2020
2				1	
2				1	09/08/2020

9			1	18/07/202
2			1	27/09/202
2			1	13/07/202
2			1	29/12/202
2			1	03/06/202
1	1	12/05/202	1	12/05/202
2			1	25/10/202
2			1	28/08/202
			1	11/12/202
9			1	08/12/202
9			1	08/09/202
1	1	27/08/202	1	24/08/202
2			1	12/09/202
1	1	30/10/202	1	30/10/202
9			1	05/05/202
9			1	29/05/202
9			1	21/08/202
1	1	09/04/202	1	08/04/202
1	1	25/07/202	1	25/07/202
9			1	05/12/202
2			1	27/08/202
2			1	28/10/202
1	1	31/07/202	1	30/07/202
2			1	23/07/202
2			1	26/07/202
2			1	30/07/202
1	1	17/12/202	1	17/12/202
2			1	31/12/202
1	1	02/06/202	1	05/06/202
2			1	28/04/202
2			1	04/10/202
9			1	26/08/202
2			1	18/11/202
2			1	15/07/202
9			1	24/05/202
2			1	22/06/202
9			1	26/06/202
2			1	12/07/202
2			1	11/09/202
2			1	27/08/202
2			1	31/12/202
2			1	25/05/202
1	1	18/08/202	1	18/08/202
2			1	14/06/202
2			1	02/09/202
9			1	30/09/202

1	1		06/05/2020	1	08/05/2020
1	1		15/07/2020	1	28/07/2020
9				1	30/07/2020
2				1	10/07/2020
1	1		26/04/2020	1	26/04/2020
1	1		19/06/2020	1	24/06/2020
9				1	10/07/2020
2				1	29/09/2020
2				1	21/09/2020
1	3	AMOXILIN/	10/11/2020	1	11/11/2020
2				1	19/10/2020
2				1	07/11/2020
				1	09/06/2020
2				1	20/04/2020
2				1	09/06/2020
1	1		08/10/2020	1	08/10/2020
2				1	15/09/2020
				1	02/01/202
9					
2				1	02/07/2020
9				1	20/04/2020
2				1	19/05/2020
2				1	12/06/2020
9				1	02/07/2020
2				1	19/07/2020
2				1	14/10/2020
				1	25/10/2020
1	1		13/06/2020	1	13/06/2020
2				1	11/12/2020
2					
1	1		19/04/2020	1	17/04/2020
1	1		21/05/2020	1	21/05/2020
2				1	04/10/2020
2				1	29/09/2020
2				1	10/07/2020
2				1	24/05/2020
2				1	22/07/2020
2				1	07/05/2020
2				1	13/11/2020
2				1	09/06/2020
2				1	18/12/2020
1	1		03/05/2020	1	02/05/2020
9				1	31/05/2020
2				1	12/07/2020
2				1	14/07/2020
1	1		09/07/2020	1	09/07/2020
				1	17/05/2020

2			1	06/06/202
2			1	25/07/202
2			1	11/07/202
2			1	31/08/202
2			1	24/08/202
2			1	13/07/202
2			1	02/04/202
1	1		1	23/04/202
1	1	24/07/202	1	24/07/202
9			1	19/05/202
2			1	21/05/202
2			1	16/07/202
			1	23/11/202
2			1	03/08/202
1	1	23/04/202	1	22/04/202
2			1	26/04/202
2			1	03/12/202
1			1	21/06/202
2			1	31/10/202
2			1	17/05/202
9			1	26/05/202
1	1	25/06/202	1	25/06/202
2			1	16/08/202
2			1	17/12/202
2			1	28/09/202
1	1	07/05/202	1	07/05/202
2			1	30/05/202
2			1	15/06/202
			1	30/03/202
2			1	13/09/202
2			1	16/11/202
2			1	15/11/202
			1	05/05/202
2			1	18/06/202
2			1	02/07/202
1	1	27/06/202	1	27/06/202
1	1	03/06/202	1	04/06/202
2			1	17/07/202
1	1		1	03/06/202
1	1	04/05/202	1	04/05/202
2			1	08/11/202
2			1	24/11/202
9			1	16/12/202
1	1	26/04/202	1	25/04/202
2			1	23/04/202
1			1	23/06/202
2			1	06/06/202

2			1	28/06/202
			1	26/11/202
1	1	12/04/202	1	11/04/202
9			1	27/09/202
2			1	31/03/202
9			1	19/10/202
2			1	07/10/202
2			1	06/10/202
2			1	06/07/202
2			1	29/04/202
2			1	14/07/202
2			1	06/05/202
2			1	19/06/202
1	1		1	15/04/202
9			1	28/06/202
2			1	29/05/202
1	1	18/04/202	1	17/04/202
2			1	20/12/202
2			1	23/11/202
2			1	10/04/202
2			1	23/07/202
2			1	29/08/202
9			1	18/07/202
2			1	13/08/202
1	1	06/01/202	1	06/01/202
2			1	27/12/202
2			1	26/12/202
1	1	05/06/202	1	05/06/202
9			1	11/12/202
1	1	19/08/202	1	19/08/202
			1	03/01/202
2			1	11/05/202
2			1	23/04/202
2			1	13/05/202
1	1	07/05/202	1	07/05/202
2			1	17/05/202
2			1	08/06/202
2			1	04/06/202
2			1	06/01/202
2			1	27/06/202
			1	11/08/202
2			1	29/07/202
2			1	10/09/202
2			1	08/12/202
2			1	02/11/202
9			1	06/06/202
9			1	04/09/202

2				1	04/05/202
1	1		16/06/202	1	24/06/202
2				1	06/06/202
2				1	19/07/202
2				1	23/10/202
2				1	28/08/202
1	1		28/07/202	1	27/07/202
1	1		05/05/202	1	04/05/202
				1	24/08/202
2				1	09/12/202
				1	10/10/202
1	1		12/06/202	1	12/06/202
9				1	16/10/202
9				1	07/01/202
2				1	06/06/202
9				1	03/09/202
				1	23/08/202
2				1	14/12/202
1	3	ROCEFIM	03/01/202	1	03/01/202
2				1	21/05/202
2				1	18/07/202
2				1	27/07/202
2				1	24/07/202
2				1	23/08/202
2				1	13/05/202
9				1	27/04/202
2				1	04/06/202
2				1	28/05/202
1	1		05/07/202	1	11/07/202
2				1	21/08/202
2				1	12/12/202
2				1	15/01/202
2				1	01/06/202
1	1		14/07/202	1	14/07/202
9				1	28/11/202
9				1	16/10/202
				1	21/04/202
2				1	27/06/202
9				1	29/04/202
2				1	13/06/202
2				1	21/08/202
9				1	19/10/202
2				1	05/10/202
2				1	14/12/202
9				1	01/10/202
2				1	06/01/202
2				1	18/11/202

2				1	18/05/202
1	1		10/07/202	1	09/07/202
2				1	20/12/202
1	1		16/05/202	1	16/05/202
1	3	azt	04/07/202	1	07/07/202
				1	24/06/202
2				1	01/10/202
2				1	04/11/202
1				1	29/08/202
2				1	23/05/202
2				1	19/10/202
1	1		25/04/202	1	28/04/202
2				1	11/06/202
2				1	26/06/202
2				1	01/01/202
2				1	16/09/202
2				1	04/09/202
9				1	22/06/202
1	1		26/05/202	1	26/05/202
2				1	04/01/202
2				1	20/08/202
1	1		29/06/202	1	29/06/202
9				1	23/05/202
1	1		17/04/202	1	22/04/202
2				1	10/08/202
1	1		17/07/202	1	17/07/202
2				1	24/04/202
2				1	27/07/202
2				1	28/06/202
2				1	10/05/202
1	1		26/07/202	1	26/07/202
2				1	07/06/202
2				1	29/10/202
1	1		23/05/202	1	23/05/202
2				1	23/11/202
2				1	04/01/202
2				1	04/07/202
1	1		19/07/202	1	19/07/202
2				1	18/05/202
2				1	25/12/202
1	1		14/04/202	1	14/04/202
1	1		09/04/202	1	09/04/202
				1	23/12/202
1	1		05/10/202	1	11/10/202
2				1	27/04/202
1	1		07/11/202	1	07/11/202
2				1	24/12/202

2			1	14/05/2020
2			1	14/08/2020
1	1	23/05/2020	1	23/05/2020
2			1	17/07/2020
2			1	27/07/2020
2			1	23/11/2020
1	1	21/08/2020	1	21/08/2020
			1	10/12/2020
2			1	17/11/2020
1	1	16/04/2020	1	16/04/2020
2			1	19/05/2020
1	1	29/03/2020	1	29/03/2020
2			1	11/07/2020
1	1	14/05/2020	1	13/05/2020
9			1	26/05/2020
2			1	24/09/2020
2			1	22/05/2020
2			1	20/03/2020
1	1	20/07/2020	1	20/07/2020
1	1	03/06/2020	1	06/06/2020
2			1	20/12/2020
2			1	04/07/2020
2			1	15/11/2020
2			1	21/07/2020
1			1	26/11/2020
2			1	24/12/2020
2			1	04/08/2020
2			1	15/12/2020
2			1	03/05/2020
2			1	30/06/2020
2			1	02/09/2020
1	1		1	10/11/2020
2			1	09/09/2020
2			1	23/07/2020
2			1	23/05/2020
2			1	23/12/2020
			1	23/05/2020
1	1	19/04/2020	1	19/04/2020
2			1	21/11/2020
2			1	29/05/2020
2			1	26/04/2020
2			1	28/08/2020
2			1	07/12/2020
			1	30/12/2020
2			1	13/07/2020
			1	22/06/2020
			1	02/05/2020

				1	08/10/202
1	1			1	21/11/202
1	1		26/05/202	1	26/05/202
2				1	18/12/202
2				1	02/05/202
2				1	05/11/202
1				1	11/08/202
2				1	15/07/202
1	1		03/11/202	1	06/11/202
2				1	08/12/202
1	3	AZITROMI	24/06/202	1	18/06/202
2				1	26/11/202
2				1	26/05/202
2				1	13/05/202
9				1	21/08/202
2				1	14/11/202
1	1		14/05/202	1	14/05/202
2				1	22/04/202
2				1	30/04/202
2				1	06/12/202
1	1			1	05/05/202
2				1	21/06/202
9				1	01/05/202
2				1	16/07/202
2				1	09/01/202
2				1	16/09/202
9				1	05/10/202
1	1			1	03/12/202
2				1	03/06/202
2				1	08/08/202
9				1	09/10/202
2				1	16/11/202
2				1	09/07/202
9				1	27/04/202
2				1	14/06/202
1	1		17/08/202	1	17/08/202
2				1	02/01/202
2				1	15/09/202
				1	09/11/202
2				1	03/01/202
9				1	14/06/202
1	1		07/05/202	1	06/05/202
2				1	07/06/202
2				1	21/11/202
				1	16/12/202
2				1	03/08/202
9				1	11/05/202

2			1	03/07/202
1	1	28/06/202	1	28/06/202
1	1		1	25/05/202
2			1	18/05/202
			1	15/07/202
2			1	27/08/202
2			1	04/12/202
2			1	05/09/202
2			1	24/11/202
1	1	03/01/202	1	03/01/202
1	3	06/01/202	1	06/01/202
2			1	08/05/202
1	1	06/05/202	1	06/05/202
2			1	24/07/202
1	2	08/06/202	1	03/06/202
2			1	05/08/202
2			1	20/08/202
2			1	11/09/202
2			1	23/12/202
2			1	19/08/202
2			1	21/04/202
2			1	22/12/202
1	1	03/07/202	1	02/07/202
1			1	26/11/202
2			1	18/07/202
2			1	28/07/202
2			1	07/08/202
2			1	22/05/202
2			1	08/07/202
2			1	16/09/202
2			1	01/12/202
2			1	16/04/202
2			1	02/04/202
9			1	06/06/202
			1	01/04/202
2			1	16/11/202
9			1	13/08/202
1	1		1	12/12/202
1	1	27/04/202	1	29/04/202
2			1	10/07/202
2			1	16/07/202
9			1	19/08/202
2			1	24/06/202
			1	06/05/202
1	1	01/04/202	1	01/04/202
2			1	12/12/202
2			1	03/01/202

2			1	17/06/202
1	1	23/05/202	1	21/05/202
			1	21/12/202
2			1	31/12/202
1			1	05/07/202
2			1	18/07/202
2			1	23/08/202
1	1	07/04/202	1	07/04/202
1	1	24/07/202	1	18/07/202
9			1	02/12/202
9			1	07/12/202
2			1	15/08/202
2			1	11/04/202
1	1	05/05/202	1	04/05/202
1			1	16/05/202
2			1	23/06/202
2			1	28/11/202
2			1	30/06/202
2			1	31/07/202
2			1	21/10/202
2			1	30/12/202
2			1	27/07/202
2			1	25/04/202
2			1	19/05/202
2			1	28/11/202
2			1	23/07/202
2			1	14/05/202
1	1	15/04/202	1	15/04/202
2			1	23/12/202
1	1		1	26/11/202
			1	08/05/202
2			1	15/01/202
2			1	20/05/202
2			1	04/08/202
2			1	18/06/202
2			1	09/12/202
1	1	11/05/202	1	11/05/202
2			1	12/07/202
2			1	28/07/202
2			1	04/12/202
2			1	17/06/202
9			1	09/12/202
2			1	17/08/202
2			1	30/09/202
9			1	13/05/202
9			1	20/06/202
2			1	06/12/202

2			1	21/06/202
			1	21/05/202
2			1	28/05/202
2			1	06/05/202
2			1	28/09/202
2			1	06/07/202
2			1	04/08/202
1	1	28/05/202	1	28/05/202
2			1	08/07/202
9			1	10/10/202
2			1	04/02/202
2			1	16/03/202
9			1	21/03/202
2			1	15/03/202
			1	14/04/202
9			1	17/04/202
2			1	29/05/202
2			1	23/05/202
2			1	22/06/202
2			1	22/06/202
2			1	28/08/202
2			1	02/04/202
2			1	06/02/202
9			1	19/02/202
2			1	17/02/202
			1	06/03/202
2			1	15/03/202
2			1	18/04/202
2			1	19/03/202
2			1	20/06/202
2			1	11/06/202
2			1	20/06/202
2			1	20/09/202
2			1	30/05/202
2			1	29/12/202
9			1	07/03/202
			1	24/03/202
2			1	20/03/202
1			1	27/03/202
9			1	05/04/202
2			1	18/03/202
2			1	02/06/202
2			1	27/03/202
			1	04/06/202
			1	04/07/202
2			1	16/07/202
2			1	09/08/202

2		1	19/05/202
2		1	06/09/202
2		1	01/04/202
		1	10/04/202
		1	23/05/202
2		1	30/04/202
2		1	11/06/202
2		1	24/03/202
2		1	14/06/202
		1	21/07/202
2		1	04/07/202
2		1	20/05/202
2		1	24/08/202
2		1	02/09/202
2		1	16/09/202
2		1	02/02/202
2		1	26/02/202
2		1	23/03/202
		1	30/03/202
2		1	03/04/202
2		1	13/04/202
2		1	09/04/202
		1	15/05/202
2		1	27/04/202
9		1	13/05/202
2		1	18/02/202
1	1	1	22/02/202
2		1	03/06/202
		1	14/02/202
2		1	27/09/202
9		1	23/05/202
9		1	17/03/202
2		1	04/09/202
9		1	16/03/202
2		1	14/09/202
2		1	21/09/202
9		1	02/01/202
2		1	24/01/202
2		1	25/02/202
9		1	15/03/202
2		1	14/03/202
2		1	24/04/202
2		1	30/04/202
2		1	29/05/202
2		1	02/05/202
2		1	24/06/202
2		1	14/06/202

				1	31/08/202
9				1	26/10/202
2				1	14/02/202
2				1	25/03/202
2				1	26/03/202
9				1	12/03/202
2				1	28/03/202
2				1	25/03/202
				1	06/04/202
2				1	01/04/202
2				1	09/04/202
2				1	28/05/202
2				1	30/05/202
2				1	15/06/202
2				1	20/06/202
2				1	10/08/202
2				1	27/11/202
2				1	20/02/202
1	1		16/02/202	1	19/02/202
2				1	06/04/202
2				1	17/05/202
2				1	20/05/202
2				1	31/05/202
2				1	22/05/202
9				1	02/07/202
2				1	19/06/202
2				1	29/06/202
2				1	20/07/202
2				1	21/07/202
2				1	08/07/202
2				1	10/04/202
2				1	29/09/202
9				1	24/03/202
2				1	11/04/202
				1	05/04/202
2				1	07/05/202
2				1	13/04/202
1	3	Busonid		1	05/05/202
				1	08/05/202
2				1	18/05/202
2				1	18/05/202
2				1	13/06/202
2				1	13/06/202
2				1	10/07/202
2				1	05/10/202
2				1	16/08/202

2			1	14/08/202
9			1	29/07/202
2			1	11/01/202
2			1	08/09/202
2			1	30/11/202
			1	02/03/202
2			1	22/02/202
2			1	09/04/202
9			1	23/05/202
			1	
2			1	18/05/202
2			1	15/05/202
2			1	02/06/202
2			1	16/05/202
2			1	22/06/202
9			1	29/05/202
9			1	19/06/202
1	1	03/07/202	1	28/06/202
2			1	21/10/202
2			1	02/08/202
9			1	26/12/202
2			1	20/09/202
2			1	15/01/202
2			1	13/01/202
2			1	04/03/202
2			1	14/03/202
9			1	14/03/202
			1	07/04/202
9			1	05/04/202
2			1	13/04/202
2			1	17/06/202
2			1	29/04/202
2			1	28/10/202
			1	29/03/202
2			1	14/05/202
2			1	30/04/202
2			1	04/04/202
2			1	11/05/202
2			1	21/05/202
			1	18/01/202
			1	13/05/202
2			1	11/07/202
			1	09/06/202
			1	06/06/202
2			1	26/05/202
2			1	22/06/202
2			1	12/01/202

2	1	23/02/202
2	1	13/03/202
9	1	05/03/202
2	1	17/03/202
2	1	25/03/202
9	1	11/04/202
2	1	07/04/202
2	1	15/06/202
2	1	23/04/202
2	1	27/07/202
1	1	06/08/202
2	1	14/04/202
2	1	28/11/202
	1	03/02/202
2	1	08/10/202
2	1	07/12/202
2	1	10/01/202
2	1	29/03/202
	1	06/03/202
	1	03/04/202
9	1	31/03/202
2	1	07/03/202
2	1	08/03/202
	1	26/05/202
2	1	09/04/202
9	1	27/05/202
	1	31/03/202
2	1	17/04/202
2	1	06/05/202
2	1	11/05/202
2	1	04/05/202
2	1	25/05/202
2	1	05/06/202
	1	12/06/202
2	1	27/06/202
2	1	22/07/202
2	1	16/05/202
9	1	17/08/202
2	1	13/02/202
2	1	06/03/202
2	1	27/01/202
2	1	13/03/202
2	1	23/03/202
2	1	10/03/202
2	1	02/05/202
2	1	16/05/202
2	1	21/05/202

2			1	26/05/202
2			1	20/05/202
1	1	06/06/202	1	06/06/202
2			1	15/01/202
2			1	26/05/202
2			1	02/09/202
2			1	18/02/202
2			1	05/03/202
2			1	12/03/202
2			1	21/05/202
2			1	30/04/202
2			1	01/05/202
2			1	13/06/202
2			1	23/03/202
2			1	05/07/202
2			1	23/06/202
2			1	08/07/202
2			1	20/07/202
2			1	26/08/202
2			1	15/01/202
2			1	12/01/202
2			1	04/03/202
2			1	01/03/202
2			1	03/04/202
2			1	15/03/202
2			1	04/03/202
2			1	19/04/202
2			1	08/04/202
9			1	06/04/202
2			1	21/05/202
2			1	26/05/202
2			1	19/05/202
2			1	30/08/202
2			1	16/03/202
2			1	24/03/202
1	1	18/03/202	1	18/03/202
2			1	16/03/202
2			1	12/02/202
2			1	13/01/202
2			1	20/03/202
2			1	15/03/202
9			1	20/03/202
2			1	08/05/202
2			1	07/04/202
1	1	26/04/202	1	26/04/202
2			1	16/04/202
2			1	02/06/202

2			1	14/05/202
			1	17/08/202
2			1	22/05/202
2			1	28/03/202
1	1	19/07/202	1	19/07/202
9			1	25/08/202
2			1	18/10/202
1	3	18/02/202	1	18/02/202
2			1	01/04/202
2			1	24/03/202
2			1	06/03/202
			1	24/04/202
2			1	26/05/202
2			1	23/05/202
2			1	08/04/202
9			1	21/04/202
2			1	10/04/202
2			1	08/04/202
			1	27/05/202
			1	10/04/202
2			1	19/05/202
2			1	23/06/202
9			1	13/08/202
1	1	21/01/202	1	21/01/202
1			1	19/05/202
2			1	16/03/202
2			1	12/03/202
2			1	10/03/202
2			1	01/04/202
2			1	13/04/202
2			1	17/06/202
2			1	21/02/202
2			1	31/03/202
2			1	05/01/202
9			1	23/11/202
2			1	07/01/202
2			1	14/01/202
2			1	01/02/202
9			1	04/04/202
2			1	06/03/202
2			1	12/05/202
2			1	12/04/202
2			1	29/05/202
2			1	26/05/202
2			1	04/05/202
2			1	09/06/202

2	1	03/06/202
2	1	20/05/202
2	1	14/06/202
9	1	20/06/202
2	1	01/06/202
9	1	10/03/202
9	1	29/03/202
2	1	28/03/202
2	1	25/03/202
2	1	14/04/202
2	1	27/06/202
2	1	23/04/202
2	1	07/03/202
2	1	13/05/202
2	1	04/05/202
2	1	21/05/202
2	1	10/08/202
2	1	27/05/202
2	1	22/02/202
2	1	24/02/202
2	1	23/03/202
2	1	25/03/202
2	1	02/05/202
2	1	14/04/202
2	1	09/05/202
	1	06/06/202
2	1	22/05/202
	1	01/02/202
2	1	06/06/202
9	1	14/06/202
2	1	04/09/202
	1	05/09/202
2	1	02/03/202
2	1	13/03/202
2	1	25/03/202
2	1	23/03/202
2	1	03/06/202
	1	19/04/202
2	1	23/05/202
2	1	16/06/202
2	1	06/06/202
2	1	24/05/202
9	1	18/06/202
2	1	10/06/202
9	1	24/05/202
2	1	13/07/202
2	1	22/06/202

2			1	28/07/202
2			1	07/10/202
2			1	15/04/202
2			1	14/01/202
1	1	17/03/202	1	15/03/202
2			1	19/04/202
2			1	18/04/202
			1	04/04/202
2			1	29/06/202
2			1	10/05/202
2			1	25/05/202
2			1	23/05/202
9			1	24/06/202
2			1	21/06/202
2			1	01/07/202
1	2	15/07/202	1	15/07/202
2			1	09/03/202
2			1	30/04/202
			1	16/04/202
			1	16/05/202
2			1	24/03/202
2			1	20/05/202
2			1	10/06/202
2			1	25/05/202
9			1	25/07/202
2			1	23/07/202
2			1	11/07/202
9			1	03/08/202
2			1	21/12/202
2			1	04/04/202
2			1	17/04/202
2			1	22/04/202
2			1	08/04/202
9			1	10/04/202
1	1	05/05/202	1	04/05/202
2			1	15/06/202
9			1	27/04/202
2			1	03/06/202
			1	16/06/202
2			1	20/07/202
9			1	02/07/202
2			1	16/06/202
			1	01/03/202
2			1	25/06/202
2			1	09/01/202
2			1	29/06/202
2			1	27/12/202

2	1	20/02/202
2	1	09/03/202
2	1	31/03/202
2	1	15/02/202
2	1	13/04/202
	1	28/02/202
2	1	26/04/202
2	1	27/05/202
2	1	
2	1	13/05/202
9	1	16/07/202
2	1	18/11/202
2	1	03/04/202
2	1	23/02/202
2	1	30/03/202
2	1	03/04/202
2	1	17/03/202
2	1	25/04/202
9	1	10/03/202
2	1	06/04/202
	1	18/05/202
2	1	28/06/202
9	1	22/05/202
2	1	06/08/202
9	1	21/06/202
9	1	24/06/202
	1	16/07/202
2	1	28/07/202
2	1	23/07/202
9	1	05/08/202
2	1	31/07/202
9	1	07/11/202
2	1	20/01/202
2	1	02/04/202
9	1	12/03/202
9	1	
9	1	18/04/202
	1	02/05/202
2	1	05/05/202
2	1	
2	1	25/05/202
2	1	20/05/202
9	1	18/05/202
2	1	07/05/202
2	1	20/04/202
	1	25/04/202
2	1	18/05/202

2			1	19/06/202
2			1	12/06/202
9			1	08/07/202
2			1	23/08/202
			1	29/05/202
			1	25/07/202
9			1	03/06/202
2			1	08/06/202
2			1	08/07/202
2			1	06/03/202
2			1	02/03/202
2			1	24/02/202
2			1	14/03/202
2			1	
2			1	15/04/202
2			1	09/03/202
2			1	11/05/202
2			1	25/05/202
9			1	06/06/202
			1	29/07/202
2			1	30/06/202
2			1	18/07/202
			1	13/07/202
9			1	05/03/202
			1	27/03/202
9			1	25/03/202
2			1	29/03/202
2			1	03/03/202
2			1	29/04/202
2			1	20/04/202
2			1	08/05/202
			1	08/04/202
2			1	22/02/202
1	1	06/06/202	1	06/06/202
2			1	25/06/202
2			1	29/06/202
2			1	14/04/202
9			1	11/06/202
9			1	26/05/202
9			1	23/03/202
			1	15/08/202
2			1	12/05/202
9			1	11/01/202
2			1	21/07/202
			1	02/09/202
			1	01/01/202
9			1	02/04/202

2			1	06/04/202
2			1	12/04/202
9			1	10/05/202
2			1	03/05/202
9			1	24/05/202
2			1	05/06/202
1	1	07/06/202	1	05/06/202
1	1	28/04/202	1	29/04/202
9			1	17/06/202
			1	14/06/202
2			1	30/05/202
2			1	26/09/202
			1	21/08/202
9			1	21/06/202
			1	23/06/202
2			1	25/02/202
2			1	05/02/202
2			1	05/03/202
2			1	21/03/202
2			1	12/04/202
2			1	02/05/202
2			1	30/04/202
			1	23/05/202
1	1	22/05/202	1	21/05/202
2			1	03/05/202
2			1	20/07/202
2			1	06/06/202
			1	17/07/202
9			1	29/07/202
			1	11/10/202
2			1	09/08/202
			1	02/02/202
2			1	21/04/202
2			1	13/04/202
2			1	24/04/202
			1	04/06/202
2			1	22/03/202
9			1	02/08/202
2			1	03/08/202
2			1	19/04/202
			1	22/06/202
2			1	19/07/202
2			1	20/07/202
2			1	07/02/202
2			1	02/03/202
2			1	20/02/202
2			1	26/03/202

			1	27/03/202
9			1	22/03/202
2			1	07/06/202
2			1	23/06/202
2			1	20/05/202
2			1	22/06/202
9			1	31/03/202
2			1	25/06/202
2			1	12/07/202
2			1	16/07/202
2			1	05/03/202
2			1	21/07/202
			1	10/04/202
2			1	28/04/202
2			1	15/03/202
2			1	14/05/202
2			1	31/05/202
2			1	23/05/202
1			1	25/05/202
1			1	07/09/202
9			1	12/07/202
2			1	24/06/202
9			1	07/07/202
2			1	03/06/202
2			1	17/03/202
2			1	23/03/202
2			1	10/03/202
			1	26/02/202
1	1	28/03/202	1	28/03/202
2			1	20/04/202
2			1	02/06/202
2			1	01/04/202
2			1	07/05/202
2			1	20/05/202
1	1	12/04/202	1	11/04/202
2			1	17/04/202
2			1	18/04/202
2			1	05/06/202
2			1	06/05/202
2			1	21/07/202
2			1	15/05/202
2			1	01/06/202
2			1	12/01/202
2			1	15/08/202
2			1	20/03/202
2			1	19/03/202
2			1	19/03/202

2			1	04/05/202
2			1	24/05/202
2			1	15/05/202
2			1	15/06/202
2			1	20/05/202
9			1	14/05/202
2			1	17/05/202
2			1	10/07/202
9			1	08/09/202
2			1	08/08/202
			1	23/06/202
2			1	04/10/202
			1	14/06/202
2			1	18/04/202
2			1	26/09/202
			1	02/07/202
			1	11/03/202
2			1	14/04/202
1	1	18/02/202	1	18/02/202
2			1	12/03/202
9			1	16/04/202
2			1	29/05/202
2			1	26/04/202
2			1	03/06/202
2			1	19/06/202
			1	28/10/202
2			1	19/02/202
			1	18/03/202
9			1	24/02/202
			1	19/01/202
2			1	03/03/202
2			1	13/02/202
9			1	22/04/202
9			1	31/03/202
			1	21/03/202
2			1	12/04/202
2			1	10/05/202
2			1	22/06/202
2			1	04/08/202
			1	03/08/202
2			1	13/04/202
2			1	19/03/202
2			1	06/04/202
2			1	17/05/202
2			1	02/04/202
2			1	20/03/202

2			1	18/03/202
9			1	06/05/202
2			1	23/03/202
			1	22/06/202
2			1	12/08/202
1	1	25/06/202	1	01/07/202
2			1	22/07/202
			1	19/07/202
9			1	11/11/202
9			1	13/03/202
2			1	09/02/202
9			1	05/03/202
9			1	16/03/202
9			1	03/04/202
2			1	21/03/202
9			1	13/03/202
2			1	04/05/202
2			1	12/03/202
2			1	01/04/202
2			1	21/05/202
2			1	13/04/202
2			1	22/06/202
9			1	15/06/202
2			1	20/08/202
2			1	15/03/202
2			1	22/02/202
2			1	11/04/202
			1	29/04/202
			1	20/04/202
2			1	24/04/202
2			1	07/06/202
			1	20/06/202
2			1	03/07/202
2			1	09/12/202
2			1	26/02/202
2			1	04/03/202
2			1	25/03/202
2			1	07/04/202
2			1	07/03/202
2			1	13/04/202
9			1	03/05/202
2			1	20/04/202
1	1	17/05/202	1	17/05/202
9			1	23/04/202
9			1	11/06/202
2			1	14/08/202

9			1	08/06/202
			1	20/04/202
2			1	24/05/202
2			1	06/03/202
2			1	22/02/202
2			1	28/02/202
2			1	03/04/202
2			1	09/03/202
2			1	21/06/202
9			1	24/01/202
2			1	06/05/202
2			1	28/05/202
2			1	24/03/202
9			1	08/12/202
2			1	06/07/202
2			1	10/10/202
2			1	02/08/202
9			1	30/08/202
2			1	08/02/202
9			1	12/02/202
2			1	18/04/202
2			1	31/03/202
9			1	14/04/202
2			1	01/05/202
9			1	08/06/202
2			1	
2			1	21/06/202
			1	30/05/202
9			1	11/07/202
1	1	04/01/202	1	02/01/202
2			1	18/01/202
9			1	23/12/202
2			1	18/04/202
9			1	15/05/202
2			1	15/02/202
2			1	31/05/202
9			1	29/05/202
2			1	05/06/202
2			1	30/09/202
2			1	10/03/202
2			1	21/03/202
2			1	03/04/202
2			1	09/06/202
2			1	30/05/202
1	1	05/06/202	1	04/06/202
2			1	07/07/202
9			1	14/06/202

				1	10/07/202
9				1	19/08/202
1				1	06/12/202
				1	06/11/202
2				1	24/08/202
2				1	21/02/202
2				1	18/02/202
2				1	24/04/202
2				1	02/05/202
2				1	02/06/202
2				1	07/04/202
1	3	AZITROMI(	29/07/202	1	29/07/202
				1	15/05/202
2				1	03/09/202
1	1		23/03/202	1	23/03/202
2				1	07/06/202
2				1	10/02/202
2				1	09/02/202
2				1	18/03/202
2				1	25/03/202
				1	20/04/202
9				1	08/04/202
				1	28/03/202
1	1		24/04/202	1	24/04/202
2				1	18/05/202
9				1	14/03/202
2				1	15/03/202
				1	28/06/202
2				1	20/06/202
2				1	13/06/202
2				1	06/05/202
2				1	05/05/202
2				1	01/04/202
2				1	11/09/202
2				1	27/03/202
2				1	16/04/202
2				1	13/02/202
1	1			1	03/04/202
2				1	29/04/202
2				1	22/04/202
				1	30/08/202
9				1	11/07/202
2				1	12/08/202
2				1	06/05/202
1				1	15/11/202
9				1	26/11/202
2				1	28/03/202

9			1 25/03/202
2			1 25/02/202
2			1 08/05/202
2			1 06/04/202
2			1 28/04/202
1	1	05/08/202	1 05/08/202
			1 09/05/202
2			1 26/05/202
			1 07/10/202
2			1 02/08/202
9			1 18/10/202
2			1 14/06/202
			1 15/08/202
2			1 16/03/202
2			1 12/05/202
9			1 16/04/202
1	1	16/03/202	1 16/03/202
2			1 28/05/202
			1 10/06/202
2			1 11/01/202
2			1 11/02/202
2			1 27/03/202
			1 19/03/202
2			1 23/03/202
2			1 25/03/202
			1 28/03/202
2			1 21/03/202
2			1 05/05/202
2			1 09/04/202
			1 11/06/202
2			1 24/05/202
2			1 18/06/202
2			1 18/06/202
2			1 10/06/202
2			1 03/07/202
2			1 24/02/202
			1 20/04/202
2			1 09/04/202
2			1 12/05/202
2			1 15/06/202
2			1 13/06/202
1	1	24/06/202	1 24/06/202
2			1 07/04/202
2			1 18/04/202
2			1 18/07/202
2			1 07/06/202
2			1 27/03/202

2	1	06/12/202
2	1	01/03/202
9	1	06/03/202
2	1	01/04/202
9	1	01/04/202
9	1	15/04/202
2	1	18/05/202
2	1	23/05/202
2	1	11/05/202
2	1	09/06/202
9	1	01/07/202
2	1	09/08/202
2	1	24/08/202
2	1	18/08/202
2	1	27/01/202
2	1	27/01/202
9	1	21/01/202
2	1	16/07/202
2	1	07/02/202
2	1	25/03/202
2	1	29/03/202
	1	31/03/202
2	1	17/06/202
2	1	03/04/202
9	1	06/06/202
9	1	31/03/202
9	1	27/07/202
2	1	31/05/202
	1	12/12/202
2	1	30/11/202
2	1	11/03/202
2	1	01/04/202
9	1	13/04/202
	1	18/04/202
9	1	14/05/202
2	1	06/06/202
2	1	19/03/202
	1	28/05/202
2	1	05/06/202
2	1	14/07/202
2	1	31/07/202
2	1	06/09/202
2	1	21/05/202
2	1	
2	1	09/02/202
2	1	19/03/202
2	1	25/02/202

2			1	06/04/202
2			1	17/04/202
1	1	29/03/202	1	27/03/202
			1	25/04/202
2			1	01/05/202
2			1	09/05/202
2			1	08/05/202
2			1	21/05/202
2			1	29/03/202
9			1	17/06/202
2			1	09/07/202
2			1	09/05/202
			1	22/03/202
2			1	08/06/202
2			1	02/03/202
			1	20/02/202
2			1	09/03/202
2			1	21/05/202
2			1	30/03/202
2			1	11/03/202
2			1	06/05/202
2			1	15/05/202
2			1	17/05/202
2			1	
1	1	27/04/202	1	
2			1	25/02/202
2			1	29/06/202
2			1	19/07/202
2			1	14/08/202
2			1	08/03/202
2			1	29/03/202
2			1	09/03/202
2			1	17/03/202
2			1	03/05/202
			1	23/07/202
2			1	06/05/202
2			1	26/05/202
2			1	30/12/202
2			1	15/01/202
2			1	13/10/202
2			1	11/03/202
2			1	29/07/202
2			1	08/02/202
2			1	29/03/202
2			1	12/03/202
2			1	05/04/202
2			1	23/03/202

2	1	13/05/202
2	1	28/04/202
2	1	22/06/202
2	1	01/06/202
2	1	03/07/202
2	1	07/07/202
2	1	19/09/202
9	1	04/10/202
2	1	22/08/202
2	1	08/10/202
9	1	16/01/202
2	1	09/03/202
2	1	09/03/202
9	1	08/02/202
9	1	12/04/202
2	1	
2	1	28/04/202
9	1	15/04/202
2	1	20/05/202
2	1	20/04/202
2	1	15/05/202
2	1	26/03/202
2	1	28/05/202
2	1	30/04/202
2	1	08/07/202
9	1	20/06/202
2	1	02/03/202
	1	30/05/202
2	1	03/06/202
2	1	31/01/202
2	1	11/02/202
9	1	13/08/202
2	1	11/08/202
2	1	25/04/202
9	1	07/04/202
2	1	22/04/202
2	1	09/06/202
2	1	18/03/202
2	1	15/05/202
2	1	16/05/202
2	1	28/05/202
2	1	15/06/202
	1	01/09/202
2	1	03/11/202
	1	27/05/202
	1	26/05/202

			1	17/07/202
2			1	04/03/202
2			1	01/03/202
9			1	21/04/202
9			1	15/03/202
2			1	31/05/202
2			1	18/06/202
			1	23/06/202
2			1	31/05/202
2			1	18/05/202
2			1	08/08/202
2			1	10/07/202
			1	11/08/202
2			1	17/08/202
			1	
2			1	27/04/202
			1	17/03/202
2			1	24/05/202
2			1	26/06/202
			1	16/06/202
1	1	17/06/202	1	20/06/202
9			1	20/06/202
2			1	27/06/202
			1	09/07/202
2			1	30/11/202
2			1	24/03/202
9			1	05/12/202
2			1	29/07/202
2			1	24/02/202
			1	25/03/202
2			1	01/04/202
2			1	22/03/202
2			1	20/05/202
9			1	22/07/202
9			1	25/06/202
2			1	31/05/202
2				
9			1	10/06/202
2			1	29/03/202
2			1	17/03/202
2			1	04/02/202
2			1	04/06/202
2			1	01/05/202
2			1	26/05/202
2			1	13/05/202
2			1	08/02/202

2			1	02/04/202
2			1	24/05/202
2			1	08/05/202
2			1	26/03/202
2			1	07/04/202
2			1	20/07/202
			1	07/02/202
2			1	06/05/202
2			1	21/01/202
9			1	25/02/202
2			1	16/03/202
2			1	04/03/202
			1	08/04/202
2			1	07/07/202
2			1	
2			1	07/04/202
9			1	16/05/202
2			1	04/01/202
2			1	16/04/202
2			1	08/02/202
9			1	19/03/202
2			1	30/05/202
2			1	28/06/202
9			1	07/03/202
2			1	17/08/202
2			1	14/07/202
			1	02/07/202
2			1	04/08/202
2			1	08/08/202
1	1	09/01/202	1	09/01/202
2			1	26/03/202
2			1	22/09/202
2			1	19/02/202
2			1	11/03/202
			1	02/06/202
2			1	20/05/202
2			1	31/05/202
2			1	07/05/202
9			1	16/05/202
2			1	20/07/202
2			1	02/07/202
2			1	01/06/202
2			1	15/12/202
9			1	03/07/202
2			1	10/03/202
9			1	12/08/202

9			1	03/03/202
2				
2			1	23/06/202
2			1	04/07/202
2			1	10/04/202
2			1	03/07/202
2			1	02/09/202
2			1	10/05/202
2			1	04/11/202
9			1	24/04/202
2			1	
2			1	12/04/202
1	1		1	10/03/202
2			1	05/05/202
1	1	24/05/202	1	26/05/202
2			1	07/06/202
2			1	07/01/202
2			1	06/08/202
9			1	01/01/202
2			1	08/04/202
2			1	17/04/202
2			1	23/03/202
9			1	08/07/202
2			1	25/01/202
2			1	17/04/202
2			1	03/03/202
2			1	03/03/202
2			1	24/02/202
2			1	13/03/202
2			1	08/03/202
			1	10/06/202
2			1	08/08/202
2			1	21/05/202
2			1	20/10/202
2			1	
2			1	27/12/202
2			1	13/08/202
			1	21/08/202
2			1	09/02/202
2			1	03/09/202
1	1	31/01/202	1	31/01/202
2			1	06/03/202
2			1	25/03/202
			1	20/02/202
			1	23/05/202
2			1	27/04/202
			1	19/05/202

2			1 17/04/202
			1 09/04/202
9			1 10/04/202
2			1 16/04/202
2			1 22/02/202
2			1 17/07/202
9			1 09/07/202
2			1 27/08/202
1	1	16/03/202	1 16/03/202
1	1	10/01/202	1 10/01/202
2			1 24/08/202
2			1 23/02/202
2			1 13/05/202
2			1 22/03/202
2			1 22/04/202
9			1 04/01/202
9			1 19/06/202
2			1 10/04/202
			1 23/03/202
2			1 13/05/202
2			1 18/08/202
2			1 08/07/202
2			1 15/05/202
2			1 11/08/202
2			1 07/08/202
2			1 15/08/202
2			1 21/05/202
9			1 19/02/202
			1 05/03/202
			1 12/03/202
2			1 14/06/202
2			1 28/03/202
			1 04/07/202
2			1 25/05/202
2			1 24/03/202
			1 27/05/202
2			1 25/04/202
2			1 14/09/202
2			1 22/02/202
2			1 02/06/202
2			1 11/04/202
2			1 25/09/202
2			1 08/07/202
2			1 23/05/202
9			1 31/10/202
2			1 08/08/202
			1 24/09/202

			1	24/02/202
9			1	20/02/202
2			1	29/05/202
2			1	23/03/202
2			1	22/03/202
2			1	08/02/202
1	1	03/04/202	1	03/04/202
2			1	16/06/202
2			1	06/06/202
			1	22/05/202
2			1	06/06/202
2			1	03/08/202
2			1	18/05/202
2			1	02/04/202
2			1	06/06/202
9			1	21/08/202
2			1	27/03/202
2			1	22/03/202
2			1	16/06/202
			1	19/03/202
2			1	17/07/202
2			1	05/03/202
2			1	13/03/202
2			1	06/03/202
2			1	12/04/202
2			1	22/03/202
9			1	23/06/202
2			1	12/08/202
2			1	21/04/202
2			1	13/03/202
2			1	20/05/202
2			1	26/03/202
2			1	10/04/202
2			1	27/03/202
9			1	21/03/202
2			1	30/03/202
9			1	07/07/202
9			1	09/06/202
2			1	01/04/202
9			1	24/03/202
2			1	09/05/202
9			1	09/04/202
2			1	27/04/202
1	1	11/07/202	1	11/07/202
2			1	01/06/202
2			1	04/09/202

			1	24/06/202
2			1	01/01/202
9			1	30/10/202
2			1	18/05/202
2			1	28/12/202
2			1	21/05/202
2			1	22/04/202
9			1	23/04/202
2			1	24/05/202
2			1	20/05/202
2			1	13/06/202
2			1	08/06/202
2			1	28/06/202
1			1	05/01/202
9			1	02/07/202
2			1	14/10/202
2			1	07/06/202
9			1	12/07/202
2			1	01/07/202
2			1	28/03/202
2			1	09/02/202
2			1	16/02/202
9			1	09/03/202
			1	10/01/202
2			1	23/03/202
2			1	19/05/202
2			1	26/05/202
2			1	27/02/202
2			1	09/05/202
2			1	14/03/202
2			1	14/06/202
2			1	21/08/202
2			1	17/07/202
2			1	22/06/202
2			1	10/09/202
			1	17/05/202
2			1	07/08/202
2			1	08/11/202
2			1	25/03/202
1	1	11/01/202	1	11/01/202
9			1	21/02/202
2			1	12/04/202
			1	01/04/202
2			1	28/01/202
2			1	24/06/202
			1	02/06/202
2			1	28/05/202

9			1 01/08/202
			1 01/08/202
2			1 19/08/202
9			1 23/01/202
2			1 14/04/202
2			1 14/03/202
9			1 14/02/202
2			1 29/01/202
2			1 21/08/202
2			1 17/05/202
1	1	14/04/202	1 14/04/202
2			1 25/07/202
			1 09/08/202
2			1 23/08/202
9			1 14/08/202
2			1 22/03/202
2			1 05/05/202
2			1 24/05/202
2			1 06/04/202
2			1 08/08/202
2			1 11/05/202
9			1 13/07/202
2			1 20/03/202
2			1
2			1 15/03/202
2			1
2			1 25/06/202
2			1 01/06/202
9			1 17/05/202
			1 29/03/202
9			1 22/06/202
9			1 18/06/202
2			1 11/09/202
			1 26/02/202
1	1	23/02/202	1 23/02/202
2			1 03/10/202
2			1 07/03/202
2			1
2			1 30/03/202
2			1 14/04/202
2			1 08/09/202
9			1 22/04/202
2			1 10/06/202
2			1 15/04/202
2			1 17/03/202
2			1 30/07/202
2			1 28/08/202

2			1	06/08/202
9			1	19/07/202
2			1	13/07/202
2			1	26/04/202
2			1	04/02/202
2			1	02/06/202
2			1	30/03/202
2			1	12/03/202
2			1	12/04/202
2			1	25/02/202
9			1	09/03/202
2			1	07/03/202
2			1	05/09/202
2			1	26/03/202
2			1	21/10/202
2			1	03/04/202
2			1	14/02/202
2				
2			1	20/04/202
2			1	02/06/202
1	1	23/03/202	1	22/03/202
2			1	13/09/202
2			1	16/05/202
9			1	03/07/202
2			1	10/05/202
9			1	02/09/202
9			1	15/05/202
2			1	15/07/202
2			1	04/07/202
2			1	12/06/202
9			1	01/12/202
2			1	04/04/202
2			1	04/03/202
9			1	01/04/202
2			1	01/05/202
			1	23/05/202
2			1	10/05/202
2			1	19/05/202
1	3	AZITROMICINA	1	22/06/202
9			1	27/06/202
2			1	03/04/202
2			1	18/07/202
2			1	03/05/202
2			1	14/06/202
2			1	19/04/202
2			1	
			1	09/07/202

2		1	30/04/202
2		1	12/03/202
		1	05/10/202
9		1	06/03/202
2		1	16/06/202
2		1	13/04/202
2		1	16/07/202
2		1	
2		1	26/02/202
2		1	20/05/202
9		1	26/06/202
2		1	04/06/202
2		1	11/07/202
2		1	18/07/202
2		1	31/05/202
1		1	01/08/202
2		1	04/06/202
2		1	19/07/202
2		1	28/07/202
2		1	22/08/202
		1	08/09/202
2		1	11/05/202
2		1	26/01/202
2		1	28/10/202
9		1	07/04/202
2		1	17/01/202
2		1	08/04/202
2		1	02/03/202
2		1	24/05/202
2		1	14/06/202
2		1	29/03/202
2		1	02/04/202
1		1	14/06/202
2		1	26/05/202
2		1	13/06/202
2		1	20/05/202
2		1	30/06/202
2		1	30/09/202
2		1	17/04/202
1	1	1	29/03/202
2		1	26/04/202
9		1	09/03/202
2		1	03/02/202
2		1	18/06/202
2		1	30/06/202
9		1	29/05/202
2		1	27/07/202

2	1	26/03/202
9	1	23/02/202
2	1	24/09/202
2	1	26/05/202
2	1	09/06/202
2	1	21/02/202
1	1	10/03/202
2	1	23/04/202
2	1	26/04/202
	1	15/07/202
2	1	21/04/202
9	1	21/05/202
2	1	16/05/202
2	1	22/05/202
2	1	25/05/202
2	1	19/06/202
	1	01/08/202
	1	01/07/202
9	1	24/09/202
2	1	20/09/202
2	1	22/04/202
2	1	27/04/202
9	1	22/05/202
2	1	25/03/202
2	1	04/03/202
2	1	10/03/202
2	1	02/06/202
	1	20/04/202
2	1	14/04/202
9	1	01/05/202
2	1	06/09/202
2	1	14/06/202
	1	17/08/202
2	1	10/03/202
	1	30/03/202
2	1	04/04/202
2	1	25/03/202
9	1	26/02/202
9	1	16/04/202
9	1	09/03/202
2	1	21/04/202
2	1	01/06/202
2	1	09/05/202
2	1	12/05/202
2	1	09/05/202
9	1	20/08/202
2	1	17/07/202

2			1 31/07/202
9			1 28/04/202
9			1 04/07/202
2			1 29/03/202
			1 05/08/202
2			1 02/05/202
2			1 13/09/202
2			1 21/03/202
2			1 07/09/202
9			1 23/04/202
9			1 11/10/202
9			1 25/03/202
2			1 22/04/202
2			1 11/04/202
2			1 04/03/202
			1 16/04/202
2			1 26/03/202
2			1 26/03/202
9			1 11/06/202
2			1 01/03/202
9			1 28/09/202
1	1	05/04/202	1 09/04/202
2			1 07/11/202
2			1 12/01/202
1	1	30/01/202	1 29/01/202
2			1 14/03/202
2			1 09/02/202
2			1 12/04/202
2			1 27/07/202
2			1 17/05/202
			1 20/03/202
2			1 08/05/202
			1 14/06/202
1	1	10/05/202	1 16/05/202
2			1 11/04/202
2			1 09/03/202
			1 06/05/202
2			1 22/05/202
2			1 09/08/202
9			1 21/01/202
2			1 31/03/202
2			1 26/06/202
2			1 31/01/202
2			1 27/02/202
2			1 20/03/202
2			1 09/03/202
			1 02/06/202

9	1	05/04/202
2	1	30/03/202
	1	12/07/202
2	1	10/08/202
	1	29/03/202
2	1	06/03/202
2	1	25/06/202
	1	05/04/202
	1	16/03/202
2	1	17/03/202
2	1	25/03/202
2	1	07/07/202
	1	17/04/202
2	1	23/06/202
2	1	04/08/202
2	1	10/05/202
9	1	19/04/202
2	1	23/06/202
2	1	20/05/202
2	1	12/04/202
	1	26/05/202
9	1	10/07/202
2	1	06/08/202
9	1	30/05/202
9	1	07/05/202
2	1	03/06/202
2	1	10/03/202
9	1	08/04/202
2	1	12/03/202
2	1	04/05/202
2	1	18/04/202
2	1	02/05/202
2	1	23/05/202
9	1	22/06/202
2	1	08/06/202
2	1	18/04/202
2	1	23/04/202
	1	15/04/202
2	1	28/07/202
2	1	17/02/202
2	1	18/02/202
	1	07/02/202
2	1	27/05/202
	1	16/02/202
9	1	26/01/202
2	1	26/03/202
	1	07/04/202

2			1	19/01/202
2			1	06/06/202
2			1	21/06/202
2			1	24/02/202
2			1	22/04/202
2			1	29/04/202
2			1	11/09/202
2			1	03/06/202
9			1	25/03/202
2			1	10/05/202
9			1	09/07/202
9			1	19/05/202
2			1	30/01/202
2			1	10/09/202
2			1	16/05/202
2			1	12/06/202
2			1	09/06/202
2			1	23/06/202
2			1	20/02/202
9			1	23/08/202
2			1	15/02/202
2			1	01/06/202
2			1	09/09/202
2			1	26/04/202
2			1	15/04/202
2			1	26/08/202
			1	18/05/202
2			1	24/07/202
2			1	28/08/202
2			1	22/05/202
			1	01/06/202
2			1	05/11/202
1	1	18/03/202	1	18/03/202
9			1	01/04/202
1	1	13/05/202	1	13/05/202
2			1	28/03/202
2			1	02/05/202
9			1	25/05/202
2			1	03/06/202
9			1	10/07/202
2			1	02/05/202
2			1	19/04/202
1			1	03/06/202
2			1	29/04/202
9			1	02/07/202
9			1	30/06/202
			1	13/08/202

2				1	22/03/202
1	1		30/03/202	1	07/04/202
2				1	03/06/202
2				1	08/02/202
				1	09/04/202
2				1	27/04/202
2				1	06/04/202
2				1	01/05/202
2				1	14/04/202
1	3	azitromicir	09/06/202	1	09/06/202
9				1	30/05/202
2				1	01/06/202
9				1	07/04/202
2				1	23/06/202
2				1	30/05/202
9				1	08/05/202
9				1	13/05/202
2				1	16/01/202
9				1	10/07/202
2				1	22/05/202
1	1		30/03/202	1	29/03/202
2				1	05/04/202
2				1	18/05/202
				1	
2				1	13/06/202
2				1	29/03/202
2				1	01/03/202
2				1	11/05/202
2				1	31/05/202
2				1	14/04/202
				1	16/05/202
2				1	29/03/202
2				1	16/09/202
2				1	20/03/202
2				1	17/05/202
2				1	06/06/202
9				1	26/09/202
2				1	18/03/202
2				1	11/08/202
2				1	07/03/202
2				1	10/04/202
2				1	14/01/202
1				1	03/05/202
2				1	12/06/202
2				1	18/05/202
9				1	22/11/202
9				1	05/07/202

2		1	01/05/202
2		1	07/08/202
2		1	01/06/202
2		1	28/11/202
2		1	23/08/202
9		1	17/02/202
2		1	18/02/202
2		1	28/06/202
2		1	03/03/202
2		1	22/05/202
2		1	03/10/202
2		1	31/03/202
		1	19/05/202
2		1	21/01/202
2		1	06/05/202
2		1	26/05/202
2		1	07/04/202
2		1	
2		1	29/05/202
2		1	11/04/202
2		1	13/05/202
2		1	14/04/202
2		1	26/05/202
2		1	01/06/202
2		1	18/08/202
2		1	16/09/202
		1	12/05/202
		1	15/05/202
9		1	26/06/202
2		1	13/06/202
2		1	19/01/202
2		1	28/02/202
2		1	07/03/202
1	1	1	27/03/202
9		1	09/05/202
2		1	20/05/202
2		1	24/06/202
		1	28/05/202
2		1	25/04/202
		1	17/08/202
2		1	14/08/202
9		1	13/07/202
2		1	09/03/202
		1	15/05/202
2		1	14/03/202
9		1	19/03/202
9		1	26/04/202

	1	03/05/202
2	1	14/06/202
	1	01/03/202
9	1	22/05/202
	1	05/09/202
9	1	26/07/202
2	1	15/11/202
9	1	26/05/202
2	1	30/11/202
2	1	02/10/202
9	1	13/04/202
2	1	12/04/202
2	1	21/04/202
2	1	28/03/202
2	1	04/06/202
2	1	10/03/202
2	1	26/06/202
2	1	26/04/202
2	1	07/06/202
2	1	29/05/202
2	1	25/05/202
2	1	07/01/202
2	1	02/03/202
2	1	25/02/202
2	1	02/03/202
9	1	14/03/202
2	1	25/03/202
2	1	25/04/202
2	1	05/05/202
2	1	29/04/202
2	1	23/04/202
2	1	26/10/202
9	1	07/07/202
2	1	25/03/202
2	1	30/03/202
	1	18/03/202
2	1	18/04/202
2	1	22/02/202
2	1	16/06/202
9	1	07/07/202
2	1	27/05/202
2	1	21/09/202
2	1	05/01/202
2	1	07/03/202
2	1	21/03/202
2	1	29/03/202
2	1	24/08/202

2		1	11/04/202
2		1	10/05/202
2		1	17/06/202
2		1	27/03/202
		1	15/05/202
2		1	11/05/202
		1	15/04/202
		1	19/03/202
2		1	15/01/202
9		1	11/04/202
		1	03/01/202
		1	01/06/202
2		1	02/06/202
2		1	11/04/202
2		1	17/03/202
2		1	16/07/202
2		1	31/05/202
2		1	14/04/202
2		1	20/05/202
2		1	25/05/202
2		1	26/07/202
2		1	08/02/202
2		1	28/12/202
2		1	05/03/202
9		1	09/03/202
2		1	20/03/202
2		1	28/05/202
2		1	04/03/202
2		1	24/03/202
2		1	20/04/202
9		1	20/03/202
2		1	27/08/202
2		1	13/03/202
2		1	30/05/202
2		1	08/07/202
1	1	1	18/01/202
2		1	18/06/202
9		1	25/07/202
2		1	31/03/202
2		1	29/03/202
2		1	26/05/202
2		1	02/03/202
2		1	21/06/202
2		1	13/06/202
		1	27/03/202
2		1	29/04/202
2		1	06/05/202

2			1	05/09/202
9			1	04/08/202
1	1	11/01/202	1	11/01/202
2			1	15/02/202
2			1	15/02/202
			1	03/03/202
2			1	21/02/202
			1	21/02/202
2			1	24/03/202
2			1	20/04/202
1	1	08/07/202	1	08/07/202
2			1	26/05/202
2			1	29/06/202
2			1	06/06/202
			1	10/08/202
			1	04/03/202
2			1	05/10/202
2			1	25/04/202
2			1	10/03/202
2			1	08/05/202
2			1	09/06/202
2			1	01/06/202
2			1	04/05/202
			1	26/03/202
9			1	09/06/202
2			1	10/05/202
2			1	15/03/202
2			1	11/02/202
2			1	27/10/202
2			1	01/01/202
2			1	21/04/202
2			1	24/03/202
			1	19/03/202
2			1	24/03/202
2			1	19/08/202
9			1	10/04/202
2			1	12/04/202
9			1	10/08/202
2			1	18/03/202
1	1	27/01/202	1	26/01/202
2			1	23/02/202
2			1	03/01/202
9			1	22/09/202
2			1	16/03/202
2			1	20/05/202
2			1	03/04/202
2			1	07/02/202

9			1	05/05/202
2			1	22/04/202
9			1	11/08/202
2			1	25/08/202
1	1	21/03/202	1	21/03/202
2			1	20/06/202
9			1	11/10/202
2			1	05/04/202
2			1	29/01/202
2			1	23/03/202
2			1	20/04/202
2			1	14/03/202
			1	16/12/202
			1	29/04/202
2			1	21/05/202
2			1	23/05/202
2			1	27/03/202
2			1	30/01/202
2			1	06/04/202
2			1	11/06/202
			1	16/05/202
2			1	18/07/202
9			1	19/03/202
2			1	17/03/202
2			1	01/06/202
2			1	
1	1	12/05/202	1	12/05/202
			1	04/08/202
2			1	30/01/202
2			1	17/05/202
9			1	09/06/202
9			1	10/06/202
2			1	29/04/202
2			1	08/06/202
2			1	11/02/202
2			1	24/02/202
			1	24/09/202
2			1	24/03/202
2			1	10/05/202
			1	01/09/202
2			1	26/09/202
2			1	27/06/202
2			1	09/06/202
9			1	09/08/202
2			1	12/03/202
2			1	04/02/202
2			1	01/09/202

2			1	12/03/202
2			1	26/04/202
2			1	02/09/202
2			1	04/05/202
9			1	15/05/202
2			1	17/05/202
9			1	15/07/202
2			1	13/06/202
2			1	01/04/202
2			1	12/07/202
			1	01/05/202
2			1	27/11/202
2			1	23/07/202
2			1	25/01/202
2			1	02/10/202
2			1	07/03/202
2			1	05/05/202
2			1	10/03/202
1	1	16/05/202	1	16/05/202
2			1	17/03/202
2			1	05/06/202
1	1	05/04/202	1	05/04/202
1	1	07/04/202	1	07/04/202
9			1	30/04/202
2			1	24/03/202
2			1	23/03/202
9			1	21/03/202
2			1	22/02/202
2			1	21/03/202
			1	25/05/202
2			1	28/07/202
2			1	29/03/202
2			1	30/03/202
9			1	12/04/202
2			1	31/03/202
2			1	03/04/202
1	1	06/02/202	1	27/01/202
2			1	02/03/202
2			1	24/09/202
9			1	02/07/202
2			1	23/04/202
2			1	15/06/202
2			1	19/07/202
1	1	26/01/202	1	26/01/202
			1	28/12/202
9			1	02/04/202
2			1	14/04/202

2			1	12/06/202
2			1	28/02/202
2			1	25/05/202
			1	
2			1	12/06/202
2			1	26/11/202
9			1	19/05/202
2			1	13/03/202
1	1		1	09/04/202
2			1	
2			1	16/03/202
2			1	11/06/202
2			1	01/03/202
2			1	14/03/202
2			1	15/05/202
2			1	25/06/202
2			1	20/05/202
2			1	15/07/202
2			1	21/03/202
9			1	31/05/202
2			1	08/06/202
			1	22/01/202
2			1	20/03/202
9			1	27/03/202
2			1	09/05/202
2			1	21/06/202
2			1	05/07/202
2			1	11/01/202
2			1	20/02/202
9			1	01/05/202
2			1	16/05/202
2			1	27/04/202
9			1	18/06/202
2			1	11/07/202
1	1	12/07/202	1	14/07/202
2			1	20/07/202
9			1	11/09/202
2			1	19/06/202
2			1	26/03/202
			1	15/05/202
2			1	02/06/202
2			1	29/06/202
			1	05/06/202
2			1	04/06/202
2			1	13/04/202
2			1	31/07/202

2			1	03/04/202
1	1	11/03/202	1	12/03/202
2			1	02/03/202
2			1	01/03/202
2			1	24/04/202
2			1	04/05/202
2			1	11/10/202
2			1	27/12/202
2			1	04/03/202
2			1	21/03/202
2			1	29/03/202
			1	08/05/202
2			1	23/06/202
2			1	27/06/202
2			1	07/06/202
2			1	28/07/202
2			1	18/05/202
2			1	12/09/202
2			1	01/12/202
2			1	16/01/202
2			1	03/04/202
2			1	18/04/202
2			1	14/06/202
2			1	02/08/202
2			1	29/03/202
2			1	18/03/202
9			1	02/05/202
2			1	05/05/202
9			1	04/04/202
2			1	03/05/202
2			1	22/03/202
2			1	22/03/202
2			1	04/04/202
9			1	09/05/202
2			1	12/05/202
2			1	16/06/202
2			1	29/03/202
2			1	11/05/202
2			1	28/07/202
1			1	12/08/202
2			1	23/03/202
2			1	17/03/202
2			1	14/06/202
1	1	27/08/202	1	28/08/202
2			1	01/08/202
2			1	24/03/202
			1	08/03/202

			1 09/03/202
2			1 09/04/202
2			1 06/03/202
2			1 10/06/202
2			1 11/06/202
2			1 26/06/202
			1 09/03/202
2			1 03/10/202
1	1	15/03/202	1 15/03/202
2			1 15/05/202
9			1 17/06/202
1			1 17/07/202
2			1 23/10/202
2			1 27/07/202
2			1 29/04/202
2			1 28/03/202
9			1 01/08/202
2			1 23/03/202
2			1 25/05/202
2			1 27/04/202
2			1 05/06/202
2			1 14/05/202
2			1 03/03/202
9			1 12/03/202
9			1 24/04/202
2			1 03/06/202
2			1 20/05/202
2			1 20/05/202
2			1 31/03/202
2			1 11/05/202
			1 14/07/202
2			1 12/03/202
2			1 13/04/202
1	1	24/05/202	1 24/05/202
9			1 13/06/202
2			1 03/07/202
2			1 14/07/202
9			1 23/02/202
			1 27/06/202
2			1 19/05/202
			1 31/03/202
			1 28/04/202
9			1 28/09/202
2			1 23/06/202
2			1 10/05/202
9			1 25/06/202
2			1 26/02/202

2				1	23/03/202
2				1	25/02/202
2				1	16/06/202
				1	27/04/202
2				1	14/06/202
2				1	07/05/202
2				1	22/07/202
2				1	29/03/202
				1	15/03/202
2				1	03/04/202
2				1	21/04/202
9				1	01/05/202
2				1	15/04/202
				1	20/07/202
2				1	21/05/202
2				1	27/07/202
2				1	02/06/202
2				1	05/05/202
				1	11/06/202
2				1	11/06/202
				1	27/05/202
2				1	15/03/202
2					
2				1	17/03/202
2				1	29/05/202
1	1		01/06/202	1	01/06/202
2				1	03/06/202
2				1	01/06/202
2				1	10/06/202
2				1	09/10/202
1	3	BENEGRIPE	08/06/202	1	09/06/202
2				1	30/04/202
2				1	27/05/202
2				1	24/05/202
2				1	26/07/202
2				1	19/07/202
				1	01/06/202
2				1	06/04/202
2				1	29/05/202
2				1	01/04/202
2				1	24/05/202
2				1	12/06/202
9				1	08/07/202
2				1	01/03/202
2				1	19/04/202
9				1	22/04/202
				1	12/04/202

2	1	04/05/202
2	1	05/07/202
9	1	22/07/202
2	1	12/03/202
2	1	03/03/202
2	1	18/02/202
2	1	13/04/202
2	1	21/04/202
2	1	26/05/202
2	1	25/04/202
2	1	11/05/202
9	1	20/08/202
2	1	12/03/202
2	1	05/04/202
2	1	07/06/202
2	1	10/04/202
2	1	10/05/202
2	1	30/05/202
9	1	01/06/202
2	1	22/05/202
2	1	26/05/202
2	1	12/05/202
2	1	09/02/202
2	1	06/02/202
2	1	21/03/202
2	1	02/06/202
2	1	07/03/202
2	1	05/08/202
9	1	31/07/202
2	1	17/09/202
2	1	10/03/202
	1	06/05/202
2	1	25/05/202
9	1	12/06/202
2	1	18/07/202
9	1	18/02/202
2	1	01/04/202
2	1	03/05/202
2	1	06/07/202
2	1	22/07/202
2	1	27/05/202
9	1	05/06/202
2	1	04/06/202
2	1	07/05/202
2	1	03/03/202
2	1	17/03/202
2	1	13/04/202

2			1	21/06/202
2			1	07/12/202
			1	27/04/202
2			1	27/03/202
2			1	25/02/202
			1	25/05/202
2			1	26/04/202
2			1	29/04/202
1	1	26/05/202	1	26/05/202
2			1	07/07/202
2			1	20/04/202
2			1	07/03/202
2			1	27/07/202
2			1	05/07/202
1	1	10/02/202	1	09/02/202
2			1	15/10/202
1	1	16/04/202	1	16/04/202
2			1	04/05/202
2			1	24/05/202
2			1	09/05/202
2			1	17/05/202
2			1	10/06/202
2			1	26/09/202
2			1	13/04/202
2			1	28/02/202
2			1	22/05/202
			1	14/06/202
2			1	27/05/202
9			1	21/03/202
			1	24/11/202
2			1	09/02/202
2			1	25/02/202
2			1	17/03/202
2			1	02/03/202
2			1	11/04/202
9			1	23/04/202
2			1	18/11/202
2			1	
2			1	30/05/202
2			1	28/07/202
2			1	03/01/202
2			1	23/06/202
1	1	08/01/202	1	13/01/202
2			1	18/03/202
9			1	14/03/202
2			1	06/05/202

9			1	28/05/202
2			1	29/04/202
2			1	12/03/202
2			1	15/04/202
2			1	06/05/202
9			1	06/08/202
2			1	
2			1	07/01/202
2			1	13/03/202
1			1	24/01/202
9			1	30/04/202
2			1	18/03/202
2			1	27/03/202
1			1	14/04/202
1	1	25/04/202	1	26/04/202
2			1	15/08/202
2			1	10/01/202
			1	16/03/202
9			1	27/06/202
2			1	05/03/202
2			1	15/03/202
2			1	21/03/202
			1	05/07/202
			1	12/06/202
			1	14/06/202
2			1	13/08/202
2			1	20/05/202
			1	20/01/202
2			1	08/06/202
2			1	22/07/202
2			1	18/06/202
2			1	16/05/202
			1	17/11/202
9			1	17/04/202
2			1	12/04/202
			1	13/06/202
2			1	15/02/202
9			1	03/03/202
2			1	20/02/202
2			1	27/03/202
9			1	13/07/202
2			1	01/08/202
9			1	12/01/202
2			1	27/01/202
2			1	07/02/202
2			1	05/02/202
2			1	05/04/202

2	1	19/04/202
2	1	14/06/202
2	1	14/06/202
2	1	27/03/202
2	1	22/05/202
2	1	29/05/202
2	1	24/08/202
9	1	27/01/202
2	1	02/04/202
2	1	28/05/202
1	1	30/01/202
2	1	03/03/202
	1	19/02/202
	1	28/03/202
2	1	09/06/202
	1	05/06/202
2	1	18/07/202
2	1	22/08/202
2	1	12/01/202
2	1	10/03/202
9	1	15/03/202
2	1	23/03/202
2	1	26/04/202
2	1	25/05/202
2	1	04/09/202
2	1	13/01/202
2	1	14/03/202
2	1	01/04/202
2	1	26/04/202
2	1	16/10/202
2	1	05/03/202
2	1	05/02/202
	1	04/06/202
2	1	26/08/202
2	1	14/03/202
2	1	03/05/202
9	1	18/06/202
9	1	23/03/202
2	1	25/05/202
2	1	12/08/202
2	1	04/06/202
9	1	18/06/202
2	1	01/10/202
2	1	05/03/202
2	1	15/01/202
	1	01/06/202
9	1	30/03/202

9	1	10/05/202
2	1	06/04/202
2	1	10/06/202
	1	25/04/202
	1	06/07/202
9	1	21/05/202
2	1	05/02/202
9	1	06/09/202
2	1	01/05/202
9	1	29/06/202
2	1	18/06/202
9	1	11/06/202
9	1	23/03/202
2	1	19/04/202
2	1	21/03/202
1	1	14/05/202
2	1	21/01/202
2	1	09/06/202
2	1	06/08/202
9	1	04/04/202
2	1	26/02/202
2	1	23/03/202
9	1	19/04/202
	1	12/02/202
2	1	15/02/202
2	1	27/03/202
9	1	26/04/202
2	1	07/07/202
2	1	10/07/202
2	1	02/06/202
	1	11/10/202
9	1	07/01/202
2	1	29/05/202
2	1	25/10/202
	1	08/03/202
2	1	14/03/202
2	1	10/05/202
2	1	13/06/202
2	1	12/04/202
2	1	18/03/202
2	1	01/04/202
2	1	27/03/202
2	1	17/07/202
2	1	28/05/202
2	1	04/04/202
2	1	04/05/202
2	1	29/05/202

2	1	22/11/202
2	1	12/06/202
9	1	27/04/202
	1	29/04/202
9	1	07/05/202
2	1	23/03/202
2	1	21/07/202
	1	17/01/202
2	1	12/04/202
	1	23/02/202
9	1	21/04/202
2	1	03/06/202
2	1	02/08/202
2	1	18/02/202
2	1	30/03/202
	1	21/05/202
2	1	17/07/202
2	1	10/05/202
2	1	15/04/202
2	1	05/06/202
	1	26/07/202
2	1	26/07/202
2	1	10/05/202
2	1	18/10/202
2	1	20/02/202
2	1	02/04/202
	1	12/02/202
9	1	22/08/202
2	1	24/05/202
2	1	26/08/202
2	1	12/11/202
	1	06/07/202
2	1	04/04/202
2	1	27/03/202
2	1	15/06/202
2	1	14/07/202
2	1	18/11/202
2	1	10/05/202
	1	08/10/202
2	1	21/07/202
9	1	16/08/202
2	1	09/03/202
2	1	31/12/202
2	1	30/03/202
2	1	08/04/202
2	1	10/02/202
	1	19/01/202

2	1	18/04/202
2	1	05/06/202
2	1	09/04/202
2	1	02/05/202
	1	15/04/202
2	1	24/05/202
2	1	09/05/202
2	1	14/06/202
2	1	23/07/202
2	1	09/05/202
	1	02/03/202
2	1	13/03/202
2	1	14/03/202
2	1	10/04/202
2	1	29/05/202
	1	23/05/202
2	1	14/07/202
2	1	27/02/202
2	1	12/04/202
9	1	22/05/202
	1	26/05/202
2	1	06/04/202
9	1	30/09/202
	1	11/10/202
2	1	18/04/202
2	1	26/04/202
9	1	04/05/202
2	1	27/05/202
2	1	09/06/202
1	1	15/07/202
2	1	21/08/202
2	1	23/09/202
2	1	26/05/202
	1	03/06/202
2	1	25/06/202
2	1	15/03/202
2	1	30/04/202
2	1	
2	1	07/06/202
	1	14/06/202
2	1	31/10/202
2	1	20/04/202
2	1	
9	1	27/05/202
2	1	05/11/202
2	1	19/05/202
2	1	04/06/202

2	1	25/04/202
2	1	17/09/202
2	1	02/09/202
2	1	10/07/202
2	1	04/03/202
2	1	19/04/202
2	1	07/06/202
	1	02/06/202
9	1	01/07/202
2	1	28/07/202
2	1	21/08/202
2	1	03/11/202
2	1	10/02/202
2	1	05/05/202
9	1	05/01/202
9	1	16/02/202
2	1	11/05/202
9	1	30/05/202
2	1	28/02/202
9	1	28/03/202
9	1	10/03/202
2	1	16/03/202
2	1	13/08/202
2	1	21/02/202
2	1	01/06/202
2	1	16/08/202
9	1	31/05/202
2	1	15/07/202
	1	01/07/202
2	1	24/03/202
1	1	15/04/202
2	1	15/03/202
2	1	29/03/202
2	1	17/05/202
2	1	09/06/202
2	1	06/07/202
	1	13/09/202
	1	03/02/202
9	1	10/04/202
2	1	30/03/202
2	1	07/08/202
2	1	21/03/202
2	1	13/05/202
9	1	17/07/202
2	1	09/01/202
9	1	13/05/202
9	1	26/06/202

1	1	12/01/202	1	12/01/202
9			1	05/02/202
2			1	10/04/202
2			1	24/05/202
1	1	08/06/202	1	08/06/202
9			1	17/03/202
2			1	08/03/202
2			1	22/03/202
			1	10/03/202
9			1	09/04/202
			1	30/06/202
2			1	11/02/202
2			1	25/02/202
2			1	17/03/202
2			1	23/03/202
2			1	05/12/202
			1	02/11/202
2			1	15/03/202
2			1	19/04/202
2			1	06/06/202
2			1	21/02/202
			1	16/03/202
2			1	12/02/202
2			1	30/05/202
			1	15/07/202
9			1	06/08/202
2			1	19/02/202
2			1	18/03/202
2			1	23/04/202
2			1	25/05/202
2			1	20/07/202
9			1	18/05/202
2			1	03/11/202
9			1	24/04/202
2			1	13/05/202
2			1	24/06/202
2			1	28/12/202
2			1	12/03/202
2			1	31/05/202
2			1	18/09/202
2			1	19/04/202
			1	22/06/202
2			1	14/09/202
2			1	01/08/202
2			1	26/05/202
2			1	24/10/202
2			1	02/03/202

2				1	02/04/202
1	3	AZITROMICINA		1	07/06/202
9				1	12/05/202
9				1	23/07/202
2				1	26/10/202
1	1		29/03/202	1	29/03/202
2				1	07/04/202
2				1	11/06/202
2				1	20/03/202
2				1	09/04/202
9				1	25/04/202
9				1	03/08/202
2				1	11/04/202
2				1	24/06/202
2				1	19/03/202
9				1	12/03/202
				1	21/04/202
9				1	03/05/202
1	1			1	03/04/202
2				1	02/09/202
2				1	07/07/202
				1	19/03/202
				1	
2				1	17/05/202
9				1	29/06/202
2				1	27/05/202
2				1	09/01/202
2				1	20/07/202
2				1	24/02/202
2				1	04/05/202
9				1	24/07/202
2				1	15/03/202
1	1		04/03/202	1	08/03/202
2				1	03/05/202
2				1	22/05/202
2				1	05/06/202
2				1	22/02/202
2				1	13/04/202
2				1	29/04/202
2				1	09/06/202
2				1	24/06/202
2				1	25/09/202
				1	22/06/202
2				1	03/01/202
1				1	14/01/202
2				1	31/01/202
2				1	12/01/202

2		1	22/02/202
2		1	07/02/202
		1	23/01/202
2		1	06/01/202
		1	20/01/202
2		1	13/01/202
2		1	23/01/202
		1	19/01/202
		1	27/01/202
2		1	21/01/202
		1	06/01/202
		1	17/01/202
2		1	15/01/202
		1	20/01/202
		1	26/01/202
2		1	29/01/202
2		1	07/02/202
2		1	02/02/202
2		1	08/02/202
		1	20/01/202
2		1	27/01/202
9		1	06/01/202
		1	15/01/202
2		1	12/01/202
		1	20/01/202
2		1	14/01/202
2		1	05/02/202
		1	16/01/202
		1	21/01/202
2		1	26/01/202
2		1	24/01/202
2		1	15/01/202
2		1	16/02/202
1	1	1	13/01/202
2		1	21/01/202
2		1	04/01/202
2		1	17/01/202
2		1	07/01/202
		1	15/01/202
2		1	24/02/202
2		1	16/03/202
2		1	08/01/202
2		1	11/01/202
		1	17/01/202
2		1	07/02/202
2		1	17/01/202
9		1	13/01/202

			1	17/01/202
2			1	17/01/202
2			1	27/01/202
9			1	14/01/202
2			1	12/01/202
9			1	16/01/202
2			1	26/01/202
2			1	09/01/202
2			1	19/01/202
2			1	25/01/202
2			1	17/01/202
			1	15/01/202
2			1	28/01/202
			1	10/01/202
2			1	15/02/202
2			1	06/02/202
2			1	02/02/202
9			1	14/01/202
1	1	08/02/202	1	08/02/202
2			1	20/01/202
2			1	04/01/202
2			1	17/01/202
9			1	20/02/202
2			1	25/01/202
			1	20/01/202
9			1	17/01/202
			1	17/01/202
			1	
2			1	09/01/202
9			1	10/01/202
2			1	24/01/202
2			1	16/01/202
2			1	03/02/202
2			1	13/02/202
2			1	31/01/202
2			1	15/01/202
2			1	22/01/202
9			1	29/01/202
1	1	17/01/202	1	16/01/202
2			1	27/01/202
2			1	20/01/202
9			1	21/02/202
2			1	28/01/202
2			1	12/01/202
2			1	20/02/202
1	1	21/02/202	1	20/02/202
9			1	20/01/202

2			1	16/01/202
2			1	17/01/202
			1	22/01/202
2			1	14/01/202
9			1	12/02/202
2			1	03/02/202
2			1	20/01/202
2			1	12/01/202
1	1		1	27/01/202
2			1	04/02/202
			1	31/01/202
1	1	10/02/202	1	10/02/202
2			1	14/02/202
2			1	20/01/202
2			1	09/02/202
2			1	22/01/202
2			1	14/01/202
			1	22/01/202
2			1	28/03/202
2			1	23/01/202
2			1	03/01/202
2			1	15/01/202
9			1	30/01/202

SG_UF_INT	D_RG_INT	O_RG_INT	D_MN_INT	O_MU_INT	UTI	DT_ENTUT	DT_SAIDUT	UPORT_VE	RAIOX_RES
BA	NUCLEO RE	1380	SALVADOR	292740	1	23/06/202	03/07/202	1	9
AM	ENTORNO	5584	MANAUS	130260	1	08/01/202	14/01/202	1	2
RJ			RESENDE	330420	1	22/12/2020			
RJ			RIO DE JAN	330455	1			2	
SP	GVE XIX M	1344	MARILIA	352900	1	17/07/202	24/08/202	1	2
SP	GVE XVII C	1342	SUMARE	355240	1	02/08/2020		2	6
SP	GVE I CAPI	1331	SAO PAUL	355030	1	03/08/202	14/08/202	1	6
RJ			CAMPOS D	330100	1	14/09/2020		2	6
MS	NRS DE DO	1966	DOURADO	500370	1			3	
RJ			NILOPOLIS	330320	1	06/05/2020			
SP	GVE I CAPI	1331	SAO PAUL	355030	1	16/08/202	20/08/2020		5
MS	EX ARS DE	1975	CAMPO GR	500270	1	04/12/2020		2	
AM	ENTORNO	5584	MANAUS	130260	1	06/01/202	04/02/202	1	2
RJ			RIO DE JAN	330455	1	08/12/202	16/12/202	2	2
MA	REGIONAL	1440	IMPERATRI	210530	1	30/11/2020		9	9
SP	GVE I CAPI	1331	SAO PAUL	355030	1	14/05/202	02/06/202	1	
SP	GVE I CAPI	1331	SAO PAUL	355030	1	04/06/202	23/06/202	2	2
CE	11 CRES SC	1521	SOBRAL	231290	1	06/07/2020		2	
SP	GVE XI AR	1336	ARACATUB	350280	1	16/07/202	17/07/202	2	5
GO	CENTRAL	1779	GOIANIA	520870	1	11/09/202	28/09/202	9	6
RJ			RIO DE JAN	330455	1	01/12/2020		1	
RR			BOA VISTA	140010	1	22/12/202	15/01/202	1	
RJ			CAMPOS D	330100	1	29/05/2020		2	
SP	GVE XXXI S	1353	SOROCABA	355220	1	09/06/202	01/07/202	1	9
CE	1 CRES FOR	1519	FORTALEZ	230440	1	01/05/202	06/05/202	3	
SP	GVE XVII C	1342	JUNDIAI	352590	1	06/06/2020		3	1
BA	NUCLEO RE	1380	SALVADOR	292740	1	11/06/202	13/06/202	1	5
SP	GVE XVII C	1342	CAMPINAS	350950	1	27/11/2020			2
PE	001	1497	RECIFE	261160	1	19/10/2020		2	9
RJ			NILOPOLIS	330320	1	07/09/2020		3	5
SP	GVE I CAPI	1331	SAO PAUL	355030	1	10/05/2020		2	5
SP	GVE XXIX S	1354	SAO JOSE D	354980	1	10/06/202	21/06/202	2	4
MG	BELO HORI	1449	CONTAGEM	311860	1	06/10/2020		9	9
MG	UBERLAND	1462	UBERLAND	317020	1	03/11/2020		3	6
BA	NUCLEO RE	1380	SALVADOR	292740	1	31/12/202	07/01/202	1	2
SP	GVE XXIX S	1354	SAO JOSE D	354980	1	23/06/202	25/06/202	2	
GO	CENTRAL	1779	GOIANIA	520870	1	19/08/202	21/08/202	2	5
SP	GVE XVII C	1342	CAMPINAS	350950	1	23/06/202	08/07/202	1	6
SP	GVE I CAPI	1331	SAO PAUL	355030	1	19/07/202	22/07/202	2	3
PE	001	1497	RECIFE	261160	1	07/08/2020		2	6
SP	GVE XVII C	1342	CAMPINAS	350950	1	09/01/2021		1	9
AM	ENTORNO	5584	MANAUS	130260	1	27/04/202	07/05/202	1	9
SP			SAO PAUL	355030	1	01/05/202	02/05/202	3	
SP	GVE I CAPI	1331	SAO PAUL	355030	1	09/04/202	14/04/202	2	6
RJ			RIO DE JAN	330455	1	23/03/2020		1	
AL	1 MICRORE	1533	MACEIO	270430	1	09/08/202	14/08/202	1	

SP	GVE XVII C/	1342	CAMPINAS	350950	1	25/06/2020		3	5
SP	GVE I CAPI	1331	SAO PAULO	355030	1	27/04/2020	29/04/2020	2	4
MT	CUIABA	1578	CUIABA	510340	1	17/06/2020	20/07/2020	1	9
AL	1 MICRORI	1533	MACEIO	270430	1	04/06/2020	06/06/2020	1	6
BA	NUCLEO RI	1380	SALVADOR	292740	1	08/07/2020	12/07/2020	3	9
PA			BELEM	150140	1	01/05/2020	12/05/2020	1	2
SC	BLUMENAU	1564	BLUMENAU	420240	1	20/11/2020	01/12/2020	1	6
RJ			PETROPOLI	330390	1	27/12/2020	05/01/202	1	
PE	001	1497	RECIFE	261160	1	08/05/2020	12/05/2020	3	9
PR	03RS PONT	1357	PONTA GR	411990	1	31/07/2020	02/08/2020	3	6
SP	GVE I CAPI	1331	SAO PAULO	355030	1	15/06/2020		1	2
BA			SALVADOR	292740	1	03/08/2020	04/08/2020	2	9
SP			SAO PAULO	355030	1	20/07/2020	31/07/2020	1	
BA	NUCLEO RI	1380	SALVADOR	292740	1	13/08/2020	16/08/2020	3	
SP	GVE VII SA	1332	SAO BERN/	354870	1	26/11/2020		2	1
SP	GVE I CAPI	1331	SAO PAULO	355030	1	12/11/2020	05/12/2020	1	2
RS			PORTO ALE	431490	1	25/05/2020	27/05/2020	2	3
PA	01 REGION	1484	ANANINDE	150080	1			1	
CE	1 CRES FOI	1519	FORTALEZ/	230440	1	16/06/2020	17/06/2020	2	9
SP	GVE XX PIR	1345	PIRACICAB	353870	1	10/09/2020		2	
RS	013 CRS	1609	VENANCIO	432260	1	17/10/2020	21/10/2020	2	6
RJ			RIO DE JAN	330455	1	13/05/2020		1	6
SP	GVE I CAPI	1331	SAO PAULO	355030	1	06/01/202	18/01/202	1	
RJ			NITEROI	330330	1	30/05/2020	26/06/2020	2	6
SP			SAO SEBAS	355070	1	23/06/2020		2	2
BA	NUCLEO RI	1380	SALVADOR	292740	1	30/06/2020		2	6
PR	02RS METF	1356	CURITIBA	410690	1	28/07/2020	01/08/2020	2	
RJ			RIO DE JAN	330455	1	27/07/2020		3	5
SP			SAO PAULO	355030	1	17/09/2020	13/10/2020	2	
RJ			RIO DE JAN	330455	1	01/08/2020		2	6
PE	001	1497	RECIFE	261160	1	03/04/2020		1	
RJ			RIO DE JAN	330455	1	30/04/2020		3	
CE	1 CRES FOI	1519	FORTALEZ/	230440	1	14/04/2020		2	4
CE			CRATO	230420	1	20/07/2020		2	4
RJ			NITEROI	330330	1	16/06/2020	25/06/2020	9	5
SP	GVE IX FRA	1334	CAIEIRAS	350900	1	05/07/2020		1	2
PB	III NRS CAN	1421	CAMPINA (	250400	1	27/10/2020		3	6
BA	NUCLEO RI	1380	SALVADOR	292740	1	12/12/2020	13/12/2020	3	5
MT	CUIABA	1578	CUIABA	510340	1	11/12/2020	16/12/2020	2	6
AL	1 MICRORI	1533	MACEIO	270430	1	23/12/2020	04/01/202	2	6
SP	GVE I CAPI	1331	SAO PAULO	355030	1	01/05/2020	03/05/2020		5
PB	I NRS JOAC	1377	JOAO PESS	250750	1	26/04/2020		2	2
MG	BELO HORI	1449	BELO HORI	310620	1	01/07/2020			2
DF			BRASILIA	530010	1	04/08/2020	10/08/2020	1	6
PR	02RS METF	1356	CURITIBA	410690	1	01/08/2020	09/08/2020	1	6
RJ			RESENDE	330420	1	17/11/2020		2	
PR	04RS IRATI	1358	IRATI	411070	1	10/11/2020	16/11/2020	2	

PB	I NRS JOAC	1377	JOAO PESS	250750	1	01/10/2020		3	6
SP	GVE I CAPI	1331	SAO PAULO	355030	1	28/12/202	10/01/202	2	
RJ			RIO DE JAN	330455	1	20/04/2020		1	6
SP	GVE I CAPI	1331	SAO PAULO	355030	1	22/04/202	29/04/202	1	5
CE	1 CRES FOF	1519	FORTALEZA	230440	1	16/05/2020		2	5
SP	GVE XVII C	1342	CAMPINAS	350950	1	20/06/2020		2	6
SP	GVE XXIX S	1354	SAO JOSE D	354980	1	01/07/202	04/07/202	2	6
SP	GVE XXXI S	1353	SOROCABA	355220	1	17/08/2020		9	9
PB	III NRS CAN	1421	CAMPINA (	250400	1	05/07/2020		1	6
SP	GVE I CAPI	1331	SAO PAULO	355030	1	07/04/202	09/04/202	2	2
BA	NUCLEO RI	1380	SALVADOR	292740	1	17/05/202	19/05/202	3	5
SP	GVE XVII C	1342	JUNDIAI	352590	1	25/12/2020		1	9
SP	GVE I CAPI	1331	SAO PAULO	355030	1	28/04/202	04/05/202	1	2
SP	GVE I CAPI	1331	SAO PAULO	355030	1	01/07/2020		1	5
SP	GVE I CAPI	1331	SAO PAULO	355030	1	22/04/202	24/04/202	3	
PE	001	1497	RECIFE	261160	1	12/06/2020		1	
AP			MACAPA	160030	1	14/06/202	27/06/202	1	5
TO			PALMAS	172100	1	08/09/202	02/10/202	1	
RS	001 CRS	1592	NOVO HAN	431340	1	14/07/202	03/08/202	1	9
SP	GVE VII SAI	1332	SANTO AN	354780	1	19/10/2020		1	
SP	GVE XXIX S	1354	SAO JOSE D	354980	1	12/07/202	27/07/202	1	4
RJ			TERESOPOI	330580	1	01/07/2020		3	6
SP	GVE VII SAI	1332	SANTO AN	354780	1	11/09/2020		3	
SP	GVE I CAPI	1331	SAO PAULO	355030	1	09/09/2020		1	2
RJ			RIO DE JAN	330455	1	27/04/2020		1	2
SP	GVE I CAPI	1331	SAO PAULO	355030	1	20/05/2020		3	1
MT	CUIABA	1578	CUIABA	510340	1	21/06/202	05/07/202	1	9
RJ			RIO DE JAN	330455	1	11/07/2020		2	1
SP	GVE XXIX S	1354	SAO JOSE D	354980	1	21/10/202	28/10/202	2	6
SP	GVE I CAPI	1331	SAO PAULO	355030	1	06/12/202	07/12/202	3	
RJ			RIO DE JAN	330455	1	10/12/2020		2	
AM	ENTORNO	5584	MANAUS	130260	1	04/05/202	14/05/202	2	6
DF			BRASILIA	530010	1	13/06/202	21/06/202	2	4
RS			PORTO ALE	431490	1	07/12/202	16/12/202	2	
AP			MACAPA	160030	1	15/07/202	22/07/202	1	6
RJ			CAMPOS D	330100	1	20/08/2020		3	6
RJ			PETROPOLI	330390	1	05/07/202	09/07/202	2	9
PA	01 REGION	1484	BELEM	150140	1	24/10/202	26/10/202	3	6
CE	1 CRES FOF	1519	FORTALEZA	230440	1	11/05/202	20/05/202	2	
GO	CENTRAL	1779	GOIANIA	520870	1	02/09/2020		2	
BA	NUCLEO RI	1380	SALVADOR	292740	1	08/12/202	18/12/202	3	9
PR	11RS CAMI	1365	CAMPO M(	410430	1	16/12/202	08/01/202	3	6
SP	GVE I CAPI	1331	SAO PAULO	355030	1	29/03/202	01/04/202	2	2
RJ			RIO DE JAN	330455	1	24/06/2020		2	
SC	BLUMENAU	1564	BRUSQUE	420290	1	17/07/202	21/07/202	2	6
SP	GVE XVII C	1342	CAMPINAS	350950	1	18/09/202	22/09/202	2	6
PA					1	13/12/2020			

PA	01 REGION	1484	BELEM	150140	1	30/10/202	08/11/202	2	2
GO	CENTRAL	1779	GOIANIA	520870	1	28/06/2020		1	5
SC	JOINVILLE	1565	JOINVILLE	420910	1	04/12/202	04/12/202	2	6
RJ			RIO DE JAN	330455	1	10/10/202	27/10/202	1	9
SP	GVE I CAPI	1331	SAO PAUL	355030	1	23/11/202	26/11/202	2	6
AM	ENTORNO	5584	MANAUS	130260	1	11/05/202	24/06/202	1	2
SP	GVE I CAPI	1331	SAO PAUL	355030	1	03/05/202	06/05/202	3	5
RJ			RIO DE JAN	330455	1	03/05/2020		1	2
SP	GVE I CAPI	1331	SAO PAUL	355030	1	30/06/2020		1	4
AM	ENTORNO	5584	MANAUS	130260	1	03/09/202	04/09/202	1	9
RJ			DUQUE DE	330170	1				1
SP	GVE I CAPI	1331	SAO PAUL	355030	1	11/05/2020		3	2
AM	ENTORNO	5584	MANAUS	130260	1	29/12/2020		1	2
SP	GVE VII SA	1332	SAO BERN	354870	1	18/12/2020		2	6
PB	I NRS JOA	1377	JOAO PESS	250750	1	02/06/2020		3	5
SP	GVE I CAPI	1331	SAO PAUL	355030	1	01/06/2020		2	2
RJ			NILOPOLIS	330320	1	14/07/2020		2	2
CE	11 CRES S	1521	SOBRAL	231290	1	24/12/2020		2	
SP	GVE XXVIII	1576	CARAGUAT	351050	1	30/06/2020		1	5
SP	GVE XVII C	1342	JUNDIAI	352590	1	18/05/2020		2	6
SC	JOINVILLE	1565	JOINVILLE	420910	1	22/09/202	09/10/202	1	5
SP	GVE XXIX S	1354	SAO JOSE	354980	1	28/11/202	08/12/202	2	6
SP	GVE I CAPI	1331	SAO PAUL	355030	1	29/04/202	01/05/202	2	
BA	NUCLEO R	1380	SALVADOR	292740	1			3	
PE	001	1497	RECIFE	261160	1	06/05/202	15/05/202	2	2
SP	GVE XXIX S	1354	SAO JOSE	354980	1	23/07/202	28/07/202	2	4
MA	REGIONAL	1441	BALSAS	210140	1	08/07/202	13/07/202	1	2
DF			BRASILIA	530010	1	26/08/2020		2	6
SP	GVE XXIX S	1354	SAO JOSE	354980	1	30/09/202	05/10/202	2	6
PR	16RS APU	1370	APUCARAN	410140	1	07/09/202	06/10/202	1	
SP	GVE I CAPI	1331	SAO PAUL	355030	1	08/01/202	10/01/202	2	1
RJ			RIO DE JAN	330455	1	01/12/2020		2	6
SP	GVE I CAPI	1331	SAO PAUL	355030	1	27/12/2020		2	6
SP	GVE XXXI S	1353	SOROCAB	355220	1	11/01/202	20/01/202	3	4
SC	CHAPECO	1553	CHAPECO	420420	1	16/06/2020		2	6
AM	ENTORNO	5584	MANAUS	130260	1	13/12/202	19/12/202	1	2
SP	GVE I CAPI	1331	SAO PAUL	355030	1	05/05/202	07/05/202	1	2
SP	GVE I CAPI	1331	SAO PAUL	355030	1	14/05/2020		2	2
PB	I NRS JOA	1377	JOAO PESS	250750	1	12/06/202	18/06/202	3	9
SP	GVE XVII C	1342	CAMPINAS	350950	1	23/06/202	24/06/202	3	6
BA	NUCLEO R	1385	ITABUNA	291480	1	12/06/2020		3	9
DF			BRASILIA	530010	1	26/08/202	29/08/202	2	5
RS	016 CRS	1610	LAJEADO	431140	1	26/04/202	09/05/202	2	2
SP	GVE XXVII	1351	SAO JOSE	354990	1	14/05/2020			
RJ			RIO DE JAN	330455	1	22/04/2020		2	2
SP	GVE I CAPI	1331	SAO PAUL	355030	1	17/04/202	23/04/202	1	1
SP	GVE I CAPI	1331	SAO PAUL	355030	1	06/06/202	08/06/202	3	6

PI	4 GRS TERE	1883	TERESINA	221100	1	24/06/2020		2	6
MT	CUIABA	1578	CUIABA	510340	1	17/07/202	23/07/202	3	6
SP	GVE XII AR	1337	MATAO	352930	1	09/08/202	23/08/202	1	3
RS	016 CRS	1610	LAJEADO	431140	1	16/09/202	18/09/202	2	6
SP	GVE XXIX S	1354	CATANDUV	351110	1	18/09/2020		3	2
MG	CORONEL F	1468	IPATINGA	313130	1	11/09/2020			
SP	GVE I CAPI	1331	SAO PAULO	355030	1	12/05/2020		3	1
SP	GVE XIX MA	1344	MARILIA	352900	1	18/12/202	23/12/202	3	6
PB	I NRS JOAO	1377	JOAO PESS	250750	1	08/06/202	08/07/202	1	6
MA	REGI0NAL	1430	SAO LUIS	211130	1	10/04/202	15/04/202	3	9
PI	4 GRS TERE	1883	TERESINA	221100	1	27/06/2020		3	6
RS	014 CRS	1602	SANTA ROS	431720	1	10/08/202	11/08/202	2	6
RN	VII URSAP	1416	NATAL	240810	1	31/12/202	17/01/202	1	9
RN	II URSAP	1412	MOSSORO	240800	1	06/06/202	16/06/202	1	2
SP	GVE I CAPI	1331	SAO PAULO	355030	1	30/04/202	01/05/202	2	
RS	013 CRS	1609	SANTA CRU	431680	1	14/07/202	17/07/202	2	3
PR	02RS METR	1356	CURITIBA	410690	1	31/07/202	08/08/202	2	3
SP	GVE I CAPI	1331	SAO PAULO	355030	1	04/12/202	08/12/202	2	
MA	REGI0NAL	1430	SAO LUIS	211130	1	16/06/2020		1	9
MG	JUIZ DE FO	1452	JUIZ DE FO	313670	1	16/07/202	20/07/202	2	6
GO	PIRINEUS	1792	ANAPOLIS	520110	1	17/08/2020		1	
AM	ENTORNO	5584	MANAUS	130260	1			3	
PA	12 REGION	1495	REDENCAO	150613	1	19/11/202	19/11/202	3	1
MG	BELO HORI	1449	BETIM	310670	1	13/11/202	21/11/202	9	
SP	GVE XX PIR	1345	PIRACICAB	353870	1	11/06/202	11/06/202	1	
MT	CUIABA	1578	CUIABA	510340	1	16/07/202	20/07/202	3	6
CE	11 CRES SO	1521	SOBRAL	231290	1	21/08/202	24/08/202	2	
GO	CENTRAL	1779	GOIANIA	520870	1	23/07/202	14/08/202	1	
MG	UBERABA	1461	UBERABA	317010	1	19/10/2020		1	1
AM	ENTORNO	5584	MANAUS	130260	1	28/05/2020		1	2
BA	NUCLEO RE	1385	JEQUIE	291800	1	28/05/202	02/06/202	2	
PB	I NRS JOAO	1377	JOAO PESS	250750	1	07/07/202	09/07/202	3	9
RJ			RIO DE JAN	330455	1	26/12/2020		2	
DF			BRASILIA	530010	1	22/07/202	25/07/202	1	
GO	CENTRAL	1779	GOIANIA	520870	1	11/09/202	22/09/202	2	6
CE	1 CRES FOR	1519	FORTALEZA	230440	1	14/05/202	15/05/2020		
PR	17RS LOND	1371	LONDRINA	411370	1	10/08/202	17/08/202	3	6
CE	1 CRES FOR	1519	FORTALEZA	230440	1	13/07/202	16/07/202	3	5
RN	VII URSAP	1416	NATAL	240810	1	11/06/2020		3	5
SP	GVE VII SA	1332	SAO BERNA	354870	1	19/11/2020		2	6
RJ			RIO DE JAN	330455	1	20/04/2020		1	
DF			BRASILIA	530010	1	17/07/202	08/08/202	1	6
RJ			RIO DE JAN	330455	1	03/04/2020		1	5
RJ			RIO DE JAN	330455	1	15/04/2020		9	9
SP	GVE I CAPI	1331	SAO PAULO	355030	1	16/07/202	01/08/202	2	5
SP	GVE I CAPI	1331	SAO PAULO	355030	1	07/01/202	19/01/202	3	
SP	GVE XXVI S	1350	ESPIRITO S	351518	1	10/07/2020		2	6

MT	TANGARA	1585	TANGARA	510795	1	25/06/202	29/06/202	1	6
DF			BRASILIA	530010	1	31/12/202	01/01/202	1	6
AM	ENTORNO	5584	MANAUS	130260	1	05/01/202	12/01/202	1	6
SP	GVE I CAPI	1331	SAO PAUL	355030	1	25/12/202	12/01/202	1	
RJ			RIO DE JAN	330455	1	24/04/2020		3	6
DF			BRASILIA	530010	1	03/06/202	07/06/202	3	5
SP	GVE I CAPI	1331	SAO PAUL	355030	1	06/06/2020		2	
SP	GVE XXIX S	1354	SAO JOSE D	354980	1	22/07/202	24/07/202	2	
AL	1 MICRORI	1533	MACEIO	270430	1	28/08/2020		2	2
SP	GVE I CAPI	1331	SAO PAUL	355030	1	18/12/202	21/12/202	2	
RS			CAMAQUA	430350	1	20/12/2020		2	
RJ			NILOPOLIS	330320	1	30/09/2020		2	2
MG	DIVINOPOI	1472	DIVINOPOI	312230	1	23/12/2020		1	
SP	GVE VII SA	1332	SAO CAETA	354880	1	02/05/202	12/05/202	3	
RR			BOA VISTA	140010	1	05/11/202	18/11/202	1	6
GO	CENTRAL	1779	GOIANIA	520870	1	08/07/202	21/07/202	3	6
PA	09 REGION	1492	SANTAREM	150680	1	07/07/202	23/07/202	1	6
MG	TEOFILO O	1465	TEOFILO O	316860	1	26/05/202	01/06/202	2	
MS	EX ARS DE	1975	CAMPO GR	500270	1	15/10/2020		2	6
PA	09 REGION	1492	SANTAREM	150680	1	21/04/202	24/04/202	3	6
PR	01RS PARA	1355	PARANAGU	411820	1	25/07/202	27/07/2020		
ES	METROPOI	1510	SERRA	320500	1	05/12/202	08/12/202	3	
RJ			RIO DE JAN	330455	1	11/01/2021		2	
SP	GVE I CAPI	1331	SAO PAUL	355030	1	29/09/202	04/10/202	2	3
RJ			RIO DE JAN	330455	1	21/04/2020		2	2
RJ			RIO DAS O	330452	1	29/11/2020		2	
BA	NUCLEO RI	1380	SALVADOR	292740	1	13/07/202	16/07/202	2	9
SP	GVE XXXIII	1352	LORENA	352720	1	23/07/2020		3	5
SP	GVE VIII M	1333	GUARULH	351880	1	17/12/202	20/12/202	2	
RJ			NILOPOLIS	330320	1	01/01/2021		1	2
SP			SAO PAUL	355030	1	14/05/202	15/05/202	2	6
AL			MACEIO	270430	1	21/04/202	03/05/202	1	9
MT	CUIABA	1578	CUIABA	510340	1	18/06/2020		2	
CE	1 CRES FOI	1519	FORTALEZ	230440	1	30/05/202	04/06/202	3	
BA	NUCLEO RI	1398	GUANAMB	291170	1	13/01/202	20/01/202	2	
GO	CENTRAL	1779	GOIANIA	520870	1	12/06/202	15/06/202	2	1
MT			CUIABA	510340	1	17/09/202	19/09/202	1	9
GO	ESTRADA D	1802	CATALAO	520510	1	21/11/2020		2	6
SP	GVE I CAPI	1331	SAO PAUL	355030	1	13/05/202	13/05/202	2	6
SP			SAO PAUL	355030	1	01/05/202	05/05/202	2	
SE	REGIONAL	2056	ARACAJU	280030	1	23/05/2020		1	9
MT	CUIABA	1578	CUIABA	510340	1	27/07/202	29/07/202	3	9
RJ			RIO DE JAN	330455	1	18/06/202	17/08/202	2	6
SP	GVE XX PIR	1345	RIO CLARO	354390	1	22/06/202	29/06/202	2	9
SP	GVE XXV S	1349	SANTOS	354850	1	17/12/2020		9	9
SP			SAO PAUL	355030	1	27/12/2020		1	
SP	GVE I CAPI	1331	SAO PAUL	355030	1	03/04/202	06/04/202	2	

PE	001	1497	RECIFE	261160	1	23/05/2020		3	6
SP			SAO PAUL	355030	1	02/06/2020		3	
MG	JUIZ DE FO	1452	JUIZ DE FO	313670	1	23/06/202	25/06/202	2	9
MT	CUIABA	1578	CUIABA	510340	1	04/07/202	06/07/202	9	6
RJ			ITAPERUN	330220	1	15/05/202	24/05/202	2	6
PA	01 REGION	1484	BELEM	150140	1	11/08/202	26/09/202	2	6
RJ			RIO DE JAN	330455	1	25/08/2020		2	
SP	GVE I CAPI	1331	SAO PAUL	355030	1	22/03/202	26/03/202	3	5
MT	CUIABA	1578	CUIABA	510340	1	09/01/202	12/01/202	2	9
CE	1 CRES FO	1519	FORTALEZ	230440	1	24/03/202	26/03/202	3	
RJ			VOLTA RED	330630	1	16/05/2020		1	9
DF			BRASILIA	530010	1	30/12/202	09/01/202	1	6
RJ			RIO DE JAN	330455	1	02/12/2020		2	6
GO	CENTRAL	1779	GOIANIA	520870	1	05/07/202	07/10/202	1	
AM	ENTORNO	5584	MANAUS	130260	1	10/12/202	14/12/202	2	1
SP	GVE I CAPI	1331	SAO PAUL	355030	1	30/07/202	02/08/202	3	6
RS	001 CRS	1592	SAO LEOP	431870	1	02/10/202	17/10/202	2	
RJ			ITAPERUN	330220	1	08/01/202	09/01/202	3	6
BA	NUCLEO R	1380	SALVADOR	292740	1	01/07/202	19/07/202	1	9
SP	GVE I CAPI	1331	SAO PAUL	355030	1	21/07/202	23/07/202	2	6
SP	GVE XXIX S	1354	SAO JOSE	354980	1	20/07/202	24/08/202	1	4
PR	14RS PARA	1368	PARANAV	411840	1	12/11/202	20/11/202	1	
SP	GVE XVII C	1342	HORTOLAN	351907	1	19/07/2020		3	6
TO			PALMAS	172100	1	16/12/2020		1	
SP	GVE I CAPI	1331	SAO PAUL	355030	1	30/12/2020		2	
SP	GVE I CAPI	1331	SAO PAUL	355030	1	29/04/202	15/05/202	1	
SP	GVE I CAPI	1331	SAO PAUL	355030	1	11/10/202	14/10/202	2	9
RJ			RIO DE JAN	330455	1	17/06/202	19/06/202	2	6
PI	4 GRS TERE	1883	TERESINA	221100	1	24/06/202	02/08/202	1	2
BA	NUCLEO R	1380	SALVADOR	292740	1	28/10/2020		3	
RS	002 CRS	1607	PORTO ALE	431490	1	18/11/202	03/12/202	2	
SP	GVE I CAPI	1331	SAO PAUL	355030	1	06/07/2020		1	5
SP	GVE I CAPI	1331	SAO PAUL	355030	1	08/04/202	09/04/202	2	
PE	001	1497	RECIFE	261160	1	29/04/2020		1	6
AM	ENTORNO	5584	MANAUS	130260	1	12/01/202	14/01/202	1	6
AM	ENTORNO	5584	MANAUS	130260	1	10/10/202	12/10/202	3	
RJ			SAO JOAO	330510	1	30/04/202	04/05/2020		
RJ			RIO DE JAN	330455	1	23/03/2020		1	3
MG	CORONEL	1468	IPATINGA	313130	1	30/05/2020		1	3
CE	1 CRES FO	1519	FORTALEZ	230440	1	22/06/202	03/07/202	2	
RJ			RIO DE JAN	330455	1	25/06/2020			5
GO	CENTRAL	1779	GOIANIA	520870	1	09/06/202	16/07/202	2	6
DF			BRASILIA	530010	1	10/07/202	12/07/202	1	5
BA	NUCLEO R	1380	SALVADOR	292740	1	19/07/2020		3	6
PI	4 GRS TERE	1883	TERESINA	221100	1	05/07/2020		1	
RJ			PETROPOL	330390	1	23/05/202	09/06/202	1	5
SP	GVE XVII C	1342	HORTOLAN	351907	1	22/08/202	24/09/202	1	

SP	GVE I CAPI	1331	SAO PAUL(	355030	1	17/08/202	19/08/202	3	2
SP	GVE XXV S/	1349	CUBATAO	351350	1	14/11/2020			
SC	CRICIUMA	1558	CRICIUMA	420460	1	25/10/202	27/10/202	2	6
MT	CUIABA	1578	CUIABA	510340	1	11/09/202	14/09/202	2	6
RJ			RIO DE JAN	330455	1	16/04/2020		1	5
RJ			NILOPOLIS	330320	1	15/04/202	15/04/202	1	2
DF			BRASILIA	530010	1	06/06/202	08/06/202	3	5
PA	09 REGION	1492	SANTAREM	150680	1	28/05/202	04/06/202	2	5
SP	GVE VII SA	1332	SANTO AN	354780	1	16/09/2020		2	9
MG	BELO HORI	1449	BETIM	310670	1	10/11/202	15/11/202	2	6
AL	1 MICRORI	1533	MACEIO	270430	1	29/06/202	12/07/202	1	6
RS	016 CRS	1610	LAJEADO	431140	1	04/04/202	17/04/202	2	4
SP	GVE I CAPI	1331	SAO PAUL(	355030	1	06/07/202	18/07/202	2	2
AM	ENTORNO	5584	MANAUS	130260	1	17/05/202	26/05/202	1	6
CE	1 CRES FOI	1519	FORTALEZ/	230440	1	26/06/202	03/07/202	1	
RS	002 CRS	1607	PORTO ALE	431490	1	03/08/202	03/08/202	2	2
PR	17RS LONE	1371	LONDRINA	411370	1	11/08/2020		2	6
RJ			RIO DE JAN	330455	1	06/05/2020		1	
SP	GVE I CAPI	1331	SAO PAUL(	355030	1	31/12/202	04/01/202	2	6
BA	NUCLEO RI	1380	SALVADOR	292740	1	04/11/202	10/11/202	2	
SP	GVE I CAPI	1331	SAO PAUL(	355030	1	20/05/202	29/05/202	1	2
SP	GVE I CAPI	1331	SAO PAUL(	355030	1	06/05/202	10/05/202	2	6
PR	02RS METF	1356	CURITIBA	410690	1	05/06/202	22/06/202	2	6
SP	GVE I CAPI	1331	SAO PAUL(	355030	1	02/06/202	06/06/202	3	
AP			MACAPA	160030	1	21/08/202	12/09/202	2	6
SP	GVE I CAPI	1331	SAO PAUL(	355030	1	21/05/2020		2	5
DF			BRASILIA	530010	1	30/12/202	31/12/202	2	6
SP	GVE XVII C/	1342	CAMPINAS	350950	1	16/04/202	20/04/202	2	5
SC	JARAGUA I	1567	JARAGUA I	420890	1	23/06/202	28/06/202	3	5
SP	GVE I CAPI	1331	SAO PAUL(	355030	1	16/06/202	29/06/202	2	
GO	CENTRAL	1779	GOIANIA	520870	1	14/08/2020		2	9
SP	GVE XXIX S	1354	SAO JOSE I	354980	1	18/09/202	23/09/202	2	6
PR	07RS PATO	1361	PATO BRAI	411850	1	02/08/202	05/09/202	2	6
RJ			RESENDE	330420	1	07/08/2020		2	6
MS	EX ARS DE	1975	CAMPO GF	500270	1	14/08/2020		1	5
RS	002 CRS	1607	PORTO ALE	431490	1	02/12/202	21/12/202	1	
MS	EX ARS DE	1975	CAMPO GF	500270	1	28/09/202	09/10/202	1	5
SP	GVE I CAPI	1331	SAO PAUL(	355030	1	04/01/202	14/01/202	2	
SP	GVE I CAPI	1331	SAO PAUL(	355030	1	28/04/202	30/04/202	3	
RJ			CAMPOS D	330100	1	27/10/202	01/11/202	3	
CE	1 CRES FOI	1519	FORTALEZ/	230440	1	07/05/202	08/05/202	1	
SP			SAO CARL(	354890	1	13/07/202	25/07/202	1	6
SP	GVE XXIV F	1348	RIBEIRAO F	354340	1	05/08/2020		1	2
AM	ENTORNO	5584	MANAUS	130260	1	18/05/202	20/05/202	1	1
SP	GVE VII SA	1332	SAO CAET/	354880	1	24/06/2020		1	2
SP	GVE X OSA	1335	BARUERI	350570	1			2	
BA	NUCLEO RI	1380	SALVADOR	292740	1	09/08/202	11/08/202	3	3

SP	GVE I CAPI	1331	SAO PAULO	355030	1	19/07/2020	21/07/2020	2	5
RS	012 CRS	1601	SANTO AN	431750	1	27/09/2020	01/10/2020	2	
SP	GVE VII SA	1332	SAO BERN	354870	1	13/07/2020		3	1
PR	10RS CASC	1364	CASCAVEL	410480	1	29/12/2020	01/01/202	3	
GO	CENTRAL	1779	GOIANIA	520870	1	03/06/2020			
AL	1 MICRORI	1533	MACEIO	270430	1	17/05/2020	21/05/2020	1	
GO	CENTRAL	1779	GOIANIA	520870	1	25/10/2020	08/11/2020	1	
MG	JUIZ DE FO	1452	JUIZ DE FO	313670	1	28/08/2020	14/09/2020	1	9
PR	02RS METF	1356	CAMPO LA	410420	1	11/12/2020	18/12/2020	1	6
SP	GVE I CAPI	1331	SAO PAULO	355030	1	08/12/2020	04/01/202	1	9
SP	GVE VIII M	1333	GUARULHO	351880	1	08/09/2020		3	
PB					1	02/09/2020		1	
DF			BRASILIA	530010	1	27/08/2020	02/09/2020	1	6
RJ			TERESOPOI	330580	1	12/09/2020		1	6
SP	GVE I CAPI	1331	SAO PAULO	355030	1	31/10/2020	25/11/2020	2	6
PR	02RS METF	1356	CURITIBA	410690	1	05/05/2020	18/05/2020	3	6
SP	GVE XXV SA	1349	SANTOS	354850	1	05/06/2020	11/06/2020	2	9
RJ			RIO DE JAN	330455	1	23/08/2020		1	
PE	001	1497	RECIFE	261160	1	09/04/2020	14/04/2020	2	3
BA	NUCLEO RI	1380	SALVADOR	292740	1	25/07/2020	31/07/2020	2	
MG	GOVERNAI	1471	GOVERNAI	312770	1	05/12/2020	06/12/2020	1	6
BA	NUCLEO RI	1380	SALVADOR	292740	1	27/08/2020	31/08/2020	3	
BA	NUCLEO RI	1380	SALVADOR	292740	1	28/10/2020	31/10/2020	2	
GO	CENTRAL	1779	GOIANIA	520870	1	02/08/2020	05/08/2020	2	6
MT	CUIABA	1578	CUIABA	510340	1	23/07/2020	02/08/2020	3	9
SP	GVE X OSA	1335	ITAPECERI	352220	1	26/07/2020		1	6
MG	BELO HORI	1449	CONTAGEN	311860	1	30/07/2020	25/08/2020	2	2
RJ			RIO DE JAN	330455	1	18/12/2020		1	
RJ			NITEROI	330330	1	31/12/2020		1	
SP	GVE I CAPI	1331	SAO PAULO	355030	1	05/06/2020	09/06/2020	3	2
RJ			NILOPOLIS	330320	1	28/04/2020		1	2
SP	GVE XI ARA	1336	BIRIGUI	350650	1	04/10/2020	12/10/2020	2	6
AL	1 MICRORI	1533	MACEIO	270430	1	26/08/2020	02/09/2020	2	5
SP	GVE XXII PI	1573	PRESIDENT	354150	1	20/11/2020	30/11/2020	1	6
AL	1 MICRORI	1533	MACEIO	270430	1	17/07/2020	02/08/2020	2	
RJ			RIO DE JAN	330455	1	24/05/2020	28/05/2020	2	2
BA	NUCLEO RI	1380	SALVADOR	292740	1	22/06/2020		3	6
SP	GVE XVIII F	1343	SAO JOAQU	354940	1	26/06/2020	26/06/2020		
PE	001	1497	RECIFE	261160	1	12/07/2020		2	6
RS	002 CRS	1607	PORTO ALE	431490	1	14/09/2020	05/10/2020	1	4
SP	GVE I CAPI	1331	SAO PAULO	355030	1	27/08/2020	30/08/2020		
AM	ENTORNO	5584	MANAUS	130260	1	31/12/2020		1	6
MA	REGI0NAL	1430	SAO LUIS	211130	1	26/05/2020	26/06/2020	1	2
MG	BELO HORI	1449	BELO HORI	310620	1	18/08/2020	22/08/2020	2	9
RN	VII URSAP	1416	NATAL	240810	1	13/07/2020	17/07/2020	3	2
SP	GVE I CAPI	1331	SAO PAULO	355030	1	03/09/2020		2	6
DF			BRASILIA	530010	1	30/09/2020	01/10/2020	3	6

CE	1 CRES FOF	1519	FORTALEZA	230440	1	08/05/202	11/05/202	3	
SP	GVE I CAPI	1331	SAO PAULC	355030	1	28/07/202	02/08/202	2	2
GO	CENTRAL	1779	GOIANIA	520870	1	30/07/2020		2	9
MG	GOVERNAI	1471	GOVERNAI	312770	1	11/07/202	07/08/202	1	2
RJ			RIO DE JAN	330455	1	26/04/2020		2	6
PR	02RS METF	1356	CURITIBA	410690	1	24/06/202	27/06/202	2	6
MT	CUIABA	1578	CUIABA	510340	1	10/07/2020		3	6
SP	GVE I CAPI	1331	SAO PAULC	355030	1	02/10/202	16/10/202	2	
SP	GVE I CAPI	1331	SAO PAULC	355030	1	21/09/202	23/09/202	1	5
PR	20RS TOLEI	1374	TOLEDO	412770	1	11/11/202	24/11/202	2	2
BA	NUCLEO RI	1380	SALVADOR	292740	1	19/10/2020		2	
PA	01 REGION	1484	BELEM	150140	1	07/11/202	11/11/202	2	6
BA	NUCLEO RI	1380	SALVADOR	292740	1	09/06/2020		2	6
ES	METROPOI	1510	SERRA	320500	1	20/04/202	12/05/202	1	5
RJ			CAMPOS D	330100	1	09/06/2020		2	1
SP	GVE I CAPI	1331	SAO PAULC	355030	1	08/10/202	14/10/202	3	
PA	01 REGION	1484	BELEM	150140	1	15/09/2020		3	6
PR	10RS CASC	1364	CASCAVEL	410480	1	02/01/202	04/01/2021		
BA					1	08/12/202	10/12/202	3	9
PA	11 REGION	1494	PARAUAPE	150553	1	02/07/202	12/07/202	9	9
RJ			RIO DE JAN	330455	1	21/04/202	22/04/202	2	2
BA	NUCLEO RI	1380	SALVADOR	292740	1	20/05/2020		1	6
CE	13 CRES TI	1523	VICOSA DC	231410	1			3	
DF			BRASILIA	530010	1	02/07/202	04/07/202	3	5
PR	17RS LONI	1371	LONDRINA	411370	1	19/07/202	26/07/202	1	3
MT	RONDONO	1579	CAMPO VE	510267	1	15/10/2020		2	2
GO			GOIANIA	520870	1	03/11/2020		1	
SP	GVE I CAPI	1331	SAO PAULC	355030	1	13/06/202	28/06/202	2	3
BA	NUCLEO RI	1380	SALVADOR	292740	1	12/12/2020		3	6
SP					1	19/05/2020		2	1
SP	GVE I CAPI	1331	SAO PAULC	355030	1	20/04/202	22/04/202	3	6
AM	ENTORNO	5584	MANAUS	130260	1	22/05/202	26/05/202	1	2
SP	GVE XXIV R	1348	RIBEIRAO F	354340	1	04/10/202	07/10/202	2	5
RJ			RIO DE JAN	330455	1	29/09/2020		2	
RJ			RIO DE JAN	330455	1	10/07/2020		2	4
SP	GVE I CAPI	1331	SAO PAULC	355030	1	24/05/202	03/06/202	1	5
GO	CENTRAL	1779	GOIANIA	520870	1	22/07/202	02/08/202	2	6
PI	4 GRS TERE	1883	TERESINA	221100	1	07/05/202	12/05/202	2	4
SC	JOINVILLE	1565	JOINVILLE	420910	1	13/11/202	16/11/202	2	5
PA	12 REGION	1495	REDENCAC	150613	1			3	3
SP	GVE XXIV R	1348	RIBEIRAO F	354340	1	18/12/202	22/12/202	2	
MS	EX ARS DE	1975	CAMPO GF	500270	1	03/05/202	05/05/202	3	6
RJ			CAMPOS D	330100	1	31/05/2020		9	
SP	GVE I CAPI	1331	SAO PAULC	355030	1	12/07/2020		2	
MT	JUINA	1582	ARIPUANA	510140	1	18/07/202	30/07/202	1	2
PR	02RS METF	1356	CURITIBA	410690	1	11/07/202	12/07/202	2	6
MA	REGI0NAL	1430	SAO LUIS	211130	1	17/05/202	25/07/202	1	5

MT	CUIABA	1578	CUIABA	510340	1	06/06/2020	12/06/2020	9	9
RS	002 CRS	1607	PORTO ALE	431490	1	27/07/2020	29/07/2020	3	3
BA	NUCLEO RE	1382	ALAGOINH	290070	1	11/07/2020		3	9
BA	NUCLEO RE	1380	SALVADOR	292740	1	31/08/2020	04/09/2020	3	
AM	ENTORNO	5584	MANAUS	130260	1	24/08/2020	27/08/2020	2	2
PE	001	1497	RECIFE	261160	1	13/07/2020	29/07/2020	9	
SP	GVE XXV SA	1349	SANTOS	354850	1	03/04/2020			
RJ			RIO DE JAN	330455	1	24/04/2020		1	2
SP	GVE XXIX S	1354	SAO JOSE D	354980	1	27/07/2020	28/07/2020	2	6
SP	GVE VII SAI	1332	SAO CAETA	354880	1	19/05/2020	22/05/2020	9	5
SP	GVE I CAPIT	1331	SAO PAULO	355030	1	21/05/2020	31/05/2020	2	2
SP	GVE I CAPIT	1331	SAO PAULO	355030	1	16/07/2020	04/08/2020	1	5
AM			BOA VISTA	130068	1	27/11/2020	15/12/2020	2	
RJ			NILOPOLIS	330320	1	03/08/2020	03/08/2020		
MA	REGI0NAL	1430	SAO LUIS	211130	1	23/04/2020		1	5
RJ			RIO DE JAN	330455	1	26/04/2020		2	9
SC	JOINVILLE	1565	JOINVILLE	420910	1	03/12/2020	11/12/2020	2	6
GO	CENTRAL	1779	GOIANIA	520870	1	25/06/2020	01/08/2020	1	
RS	017 CRS	1604	IJUI	431020	1	11/11/2020	17/11/2020	2	
RJ			RIO DE JAN	330455	1	17/05/2020	24/05/2020	2	2
PI	4 GRS TERE	1883	TERESINA	221100	1	26/05/2020	10/06/2020	1	4
PI	4 GRS TERE	1883	TERESINA	221100	1	25/06/2020	26/06/2020	2	4
SP	GVE VIII M	1333	FERRAZ DE	351570	1	16/08/2020	19/08/2020	2	2
MG	BELO HORI	1449	BELO HORI	310620	1	17/12/2020	19/12/2020	2	2
PI	4 GRS TERE	1883	TERESINA	221100	1	28/09/2020	09/10/2020	3	1
BA			SALVADOR	292740	1	07/05/2020	08/05/2020	3	6
SP	GVE I CAPIT	1331	SAO PAULO	355030	1	08/06/2020	07/07/2020	1	4
PR	10RS CASC	1364	CASCAVEL	410480	1	17/06/2020	21/06/2020	2	6
AM	ENTORNO	5584	MANAUS	130260	1	30/03/2020	19/04/2020	1	
SP	GVE XVII CA	1342	AMERICAN	350160	1	14/09/2020		3	
BA	NUCLEO RE	1381	FEIRA DE SA	291080	1	16/11/2020		2	
RJ			RIO DE JAN	330455	1				1
AM	ENTORNO	5584	MANAUS	130260	1	05/05/2020	15/05/2020		
BA	NUCLEO RE	1380	SALVADOR	292740	1	18/06/2020	23/06/2020	2	2
RJ			RIO DE JAN	330455	1	03/07/2020		1	6
BA	NUCLEO RE	1380	SALVADOR	292740	1	27/06/2020	29/06/2020	2	6
SP	GVE I CAPIT	1331	SAO PAULO	355030	1	05/06/2020		2	5
SP	GVE VIII M	1333	GUARULHO	351880	1	17/07/2020		2	
BA	NUCLEO RE	1380	SALVADOR	292740	1	03/06/2020	10/06/2020	2	
RJ			RIO DE JAN	330455	1	06/05/2020		2	5
RJ			RIO DE JAN	330455	1	08/11/2020		3	
RJ			NILOPOLIS	330320	1	24/11/2020		2	2
PR	19RS JACA	1373	JOAQUIM T	411280	1	17/12/2020	26/12/2020	2	9
SP	GVE I CAPIT	1331	SAO PAULO	355030	1	26/04/2020	28/04/2020	2	2
CE	1 CRES FOR	1519	FORTALEZA	230440	1	23/04/2020		1	6
MG	GOVERNAD	1471	GOVERNAD	312770	1	23/06/2020		9	9
RN	VII URSAP	1416	NATAL	240810	1	08/06/2020	16/06/2020	2	6

SE	REGIONAL	2057	ESTANCIA	280210	1	01/07/2020		2	
SP	GVE I CAPI	1331	SAO PAULC	355030	1	26/11/2020	09/12/2020	2	
SP	GVE I CAPI	1331	SAO PAULC	355030	1	12/04/2020	25/05/2020	1	2
RJ			NITEROI	330330	1	28/09/2020	28/09/2020	2	9
RJ			RIO DE JAN	330455	1	01/04/2020		1	5
SP	GVE I CAPI	1331	SAO PAULC	355030	1	19/10/2020	11/11/2020	1	6
GO	CENTRAL	1779	GOIANIA	520870	1	07/10/2020		2	6
AM	ENTORNO	5584	MANAUS	130260	1	07/10/2020	12/10/2020	1	5
CE	1 CRES FOF	1519	FORTALEZA	230440	1	09/07/2020	09/07/2020	3	
RJ			RIO DE JAN	330455	1	29/04/2020		1	
SP	GVE VII SAI	1332	SANTO AN	354780	1	16/07/2020		2	5
CE	1 CRES FOF	1519	FORTALEZA	230440	1	06/05/2020	08/05/2020	3	
PR			GUAIRA	410880	1	19/06/2020	28/06/2020	3	6
RJ			RIO DE JAN	330455	1	24/04/2020	11/05/2020	1	9
BA	NUCLEO RI	1380	SALVADOR	292740	1	28/06/2020	28/06/2020	2	1
SP	GVE I CAPI	1331	SAO PAULC	355030	1	29/05/2020	27/07/2020	1	4
MA	REGI0NAL	1430	SAO LUIS	211130	1	18/04/2020	26/04/2020	2	
GO	CENTRAL	1779	GOIANIA	520870	1	20/12/2020	24/12/2020	2	
RJ			RIO DE JAN	330455	1	25/11/2020		1	2
AM	ENTORNO	5584	MANAUS	130260	1	11/05/2020	14/05/2020	1	1
RS	003 CRS	1593	PELOTAS	431440	1	28/07/2020	08/08/2020	1	
SP	GVE XXIX S	1354	CATANDUV	351110	1	29/08/2020	30/08/2020	3	
SP	GVE XXV SA	1349	BERTIOGA	350635	1	18/07/2020	18/07/2020	9	9
MG	POUSO ALI	1459	ITAJUBA	313240	1	13/08/2020	14/08/2020	2	6
SP	GVE X OSA	1335	OSASCO	353440	1	06/01/202	10/01/202	3	6
SP	GVE XXVII S	1351	SAO JOSE D	354990	1	27/12/2020		3	6
SP	GVE XX PIR	1345	RIO CLARO	354390	1	26/12/2020		1	1
SP	GVE XVII CA	1342	VALINHOS	355620	1	05/06/2020			5
RJ			RIO DE JAN	330455	1	14/12/2020		1	6
DF			BRASILIA	530010	1	20/08/2020	01/10/2020	1	6
RS	002 CRS	1607	PORTO ALE	431490	1	05/01/202	09/01/202	2	
SP	GVE I CAPI	1331	SAO PAULC	355030	1	11/05/2020	13/05/2020	3	6
PE	001	1497	RECIFE	261160	1	23/04/2020	03/05/2020	2	2
AM	ENTORNO	5584	MANAUS	130260	1	14/05/2020	28/05/2020	3	2
SP	GVE I CAPI	1331	SAO PAULC	355030	1	08/05/2020	25/05/2020	1	4
CE	1 CRES FOF	1519	FORTALEZA	230440	1	17/05/2020		2	
RS	005 CRS	1608	CAXIAS DO	430510	1	08/06/2020	22/07/2020	1	
SP	GVE XXV SA	1349	GUARUJA	351870	1	04/06/2020		3	6
SP	GVE I CAPI	1331	SAO PAULC	355030	1	10/01/202	12/01/202	2	
ES	METROPOI	1510	SERRA	320500	1	27/06/2020	14/07/2020	1	6
RJ			ANGRA DO	330010	1	11/08/2020		2	
MT	CUIABA	1578	CUIABA	510340	1	29/07/2020	31/07/2020	9	9
SP	GVE XXIX S	1354	SAO JOSE D	354980	1	12/09/2020	15/09/2020	2	6
AM	ENTORNO	5584	MANAUS	130260	1	08/12/2020	12/12/2020	1	6
PE	001	1497	RECIFE	261160	1	02/11/2020		1	
BA	NUCLEO RI	1380	SALVADOR	292740	1	06/06/2020	19/06/2020	2	
PB	I NRS JOAC	1377	JOAO PESS	250750	1	04/09/2020	09/09/2020	3	6

SP	GVE I CAPI	1331	SAO PAUL	355030	1	04/05/202	06/05/202	2	5
PI	4 GRS TERE	1883	TERESINA	221100	1	24/06/2020		3	6
AP			MACAPA	160030	1	07/06/202	26/06/202	1	5
SP	GVE XVII C	1342	AMPARO	350190	1	19/07/202	20/07/202	1	6
SP	GVE I CAPI	1331	SAO PAUL	355030	1	23/10/202	27/10/202	3	
MT	CUIABA	1578	CUIABA	510340	1	28/08/2020		3	6
SP	GVE I CAPI	1331	SAO PAUL	355030	1	27/07/202	30/07/202	2	
PA	01 REGION	1484	BELEM	150140	1	06/05/2020		1	
RJ			RESENDE	330420	1	24/08/2020			
MG	UBERLAND	1462	UBERLAND	317020	1	09/12/2020		3	6
SP	GVE I CAPI	1331	SAO PAUL	355030	1	10/10/202	13/10/202	2	2
SP	GVE XIX M	1344	MARILIA	352900	1	12/06/2020		2	5
PB	I NRS JOAO	1377	JOAO PESS	250750	1	16/10/202	21/10/202	3	6
RJ			NITEROI	330330	1	09/01/2021		2	9
SP	GVE XVII C	1342	CAMPINAS	350950	1	09/06/202	30/06/202	1	2
RS	002 CRS	1607	PORTO ALE	431490	1	03/09/202	09/09/202	2	6
SP	GVE I CAPI	1331	SAO PAUL	355030	1	23/08/2020			
RJ			RIO DE JAN	330455	1	14/12/202	27/01/202	2	6
SP	GVE I CAPI	1331	SAO PAUL	355030	1	05/01/202	26/01/202	2	
RN	II URSAP	1412	MOSSORO	240800	1	25/05/202	28/05/202	2	6
BA	NUCLEO RE	1398	VITORIA D	293330	1	18/07/2020		2	
SP	GVE X OSA	1335	BARUERI	350570	1	27/07/202	05/08/202	2	5
PR	02RS METR	1356	CURITIBA	410690	1	26/07/202	13/08/202	1	6
PR	17RS LOND	1371	LONDRINA	411370	1	25/08/2020		1	2
PE	001	1497	RECIFE	261160	1	13/05/2020		3	6
RJ			RIO DE JAN	330455	1	27/04/2020		2	9
CE	20 CRES CR	1530	CRATO	230420	1	04/06/2020		2	3
PA	09 REGION	1492	SANTAREM	150680	1	28/05/202	10/06/202	1	6
PR	02RS METR	1356	CAMPINA	410400	1	11/07/202	05/08/202	1	5
MG	DIVINOPOL	1472	DIVINOPOL	312230	1	21/08/202	26/08/202	2	
SP	GVE XVII C	1342	CAMPINAS	350950	1	12/12/2020		2	6
GO	CENTRAL	1779	GOIANIA	520870	1	15/01/202	20/01/202	2	
SP	GVE I CAPI	1331	SAO PAUL	355030	1	02/06/202	06/06/202	3	
SP	GVE XXIX S	1354	SAO JOSE D	354980	1	14/07/202	16/07/202	2	4
RJ			RIO DE JAN	330455	1	28/11/202	02/12/202	2	6
SP	GVE VII SA	1332	SAO CAETA	354880	1	16/10/202	19/10/2020		6
RJ			RIO DE JAN	330455	1	21/04/2020		9	
AL	1 MICRORR	1533	MACEIO	270430	1	29/06/202	12/07/202	2	6
PB	I NRS JOAO	1377	JOAO PESS	250750	1	03/05/2020		3	6
SP	GVE I CAPI	1331	SAO PAUL	355030	1	18/06/202	08/07/202	1	9
PB	I NRS JOAO	1377	JOAO PESS	250750	1	21/08/202	22/08/202	3	6
SC	FLORIANOI	1476	FLORIANOI	420540	1	19/10/202	27/10/202	2	2
RS	010 CRS	1599	ALEGRETE	430040	1	05/10/202	10/10/202	3	6
MG	POUSO ALE	1459	ITAJUBA	313240	1	14/12/202	24/12/202	3	6
SP	GVE VII SA	1332	SAO CAETA	354880	1	01/10/2020		2	
PR	15RS MARI	1369	MARINGA	411520	1	06/01/202	26/01/202	2	
RJ			RIO DE JAN	330455	1	18/11/202	30/11/202	2	2

SP	GVE I CAPI	1331	SAO PAULO	355030	1	18/05/2020	28/05/2020	2	
MG	POUSO ALE	1459	ITAJUBA	313240	1	09/07/2020	15/07/2020	3	6
CE	1 CRES FOR	1519	FORTALEZA	230440	1	20/12/2020	24/12/2020	2	
BA	NUCLEO RE	1380	SALVADOR	292740	1	17/05/2020	23/05/2020	2	6
SP	GVE XXV SA	1349	SANTOS	354850	1	07/07/2020		2	5
RJ			RIO DE JAN	330455	1	24/06/2020		1	5
PR	02RS METR	1356	CURITIBA	410690	1	01/10/2020	07/10/2020	2	6
PE	001	1497	RECIFE	261160	1	09/11/2020	23/03/202	1	5
GO	SAO PATRI	6252	JARAGUA	521180	1			3	6
RJ			RIO DE JAN	330455	1	23/05/2020		1	
RJ			CAMPOS D	330100	1	19/10/2020		2	6
SP	GVE I CAPI	1331	SAO PAULO	355030	1	29/04/2020	03/05/2020	2	5
SP	GVE XXIX S	1354	SAO JOSE D	354980	1	11/06/2020	21/06/2020	1	4
DF			BRASILIA	530010	1	26/06/2020	29/06/2020	2	2
SP	GVE I CAPI	1331	SAO PAULO	355030	1	01/01/202	02/01/202	2	6
MA	REGI0NAL	1430	SAO LUIS	211130	1	16/09/2020	08/10/2020	1	5
PE	001	1497	RECIFE	261160	1	04/09/2020	07/09/2020	2	6
MT	CUIABA	1578	CUIABA	510340	1	22/06/2020	27/06/2020	2	5
SP	GVE I CAPI	1331	SAO PAULO	355030	1	29/05/2020	12/06/2020	1	5
SP			SAO PAULO	355030	1	04/01/2021		3	
SP			NOVA ODE	353340	1	21/08/2020		1	9
PA	01 REGION	1484	BELEM	150140	1	02/07/2020	04/07/2020	2	2
SP	GVE VII SA	1332	SAO CAETA	354880	1	23/05/2020	31/05/2020	3	5
SP	GVE I CAPI	1331	SAO PAULO	355030	1	22/04/2020	26/04/2020	9	4
PA	11 REGION	1494	PARAUAPE	150553	1	10/08/2020		2	9
SP	GVE XII ARA	1337	SAO CARLO	354890	1	17/07/2020	26/07/2020	3	6
PB	I NRS JOAO	1377	JOAO PESS	250750	1	24/04/2020		1	1
MG	UBERLAND	1462	UBERLAND	317020	1	27/07/2020		2	6
GO	CENTRAL	1779	GOIANIA	520870	1	28/06/2020	29/06/2020	1	2
SP	GVE I CAPI	1331	SAO PAULO	355030	1	10/05/2020	19/05/2020	2	5
SP	GVE XII ARA	1337	PORTO FER	354070	1	26/07/2020		3	2
MA	REGI0NAL	1430	SAO LUIS	211130	1	07/06/2020	01/07/2020	1	5
PA	11 REGION	1494	MARABA	150420	1	29/10/2020		2	6
RN	II URSAP	1412	MOSSORO	240800	1	25/05/2020	26/05/2020	2	2
PR	02RS METR	1356	CURITIBA	410690	1	26/11/2020	28/11/2020	2	
CE	1 CRES FOR	1519	FORTALEZA	230440	1	05/01/202	08/01/202	2	
PE	001	1497	RECIFE	261160	1	10/07/2020	11/07/2020	3	6
MG	BELO HORI	1449	BELO HORI	310620	1	21/07/2020		1	6
CE	1 CRES FOR	1519	FORTALEZA	230440	1	18/05/2020	21/05/2020	3	
AL	1 MICRORE	1533	MACEIO	270430	1	30/12/2020	05/01/202	1	9
SP	GVE I CAPI	1331	SAO PAULO	355030	1	14/04/2020	14/04/2020	2	2
SP	GVE I CAPI	1331	SAO PAULO	355030	1	14/04/2020	22/04/2020	3	5
PR	02RS METR	1356	CAMPO LA	410420	1	23/12/2020	28/12/2020	3	5
SP	GVE XXIX S	1354	SAO JOSE D	354980	1	11/10/2020	25/10/2020	2	3
RJ			RIO DE JAN	330455	1	27/04/2020		1	6
MG	PONTE NOV	1458	PONTE NOV	315210	1	07/11/2020		2	6
PR	02RS METR	1356	CAMPINA (	410400	1	29/12/2020	31/12/2020	3	2

PB	III NRS CAN	1421	CAMPINA (	250400	1	14/05/2020		3	6
SP	GVE XXIX S	1354	CATANDUV	351110	1	14/08/2020	08/09/2020	3	6
SP	GVE I CAPI	1331	SAO PAULO	355030	1	23/05/2020	13/06/2020	1	2
SP	GVE IX FRA	1334	CAIEIRAS	350900	1	17/07/2020	19/07/2020	3	9
PE	001	1497	RECIFE	261160	1	27/07/2020		2	6
RJ			RIO DE JAN	330455	1	23/11/2020			
PA	01 REGION	1484	BELEM	150140	1	21/08/2020	25/08/2020	2	3
RJ			RIO DE JAN	330455	1	10/12/2020	11/12/2020		
SP	GVE XVII C	1342	JUNDIAI	352590	1	17/11/2020		2	6
SP	GVE XXV S	1349	SANTOS	354850	1	16/04/2020		9	5
SP	GVE I CAPI	1331	SAO PAULO	355030	1	20/05/2020	25/05/2020	2	6
SP	GVE I CAPI	1331	SAO PAULO	355030	1	29/03/2020	11/04/2020	3	2
CE	1 CRES FOR	1519	FORTALEZA	230440	1	12/07/2020	13/07/2020	3	
SP	GVE XXVI S	1350	MOCOCA	353050	1	13/05/2020	15/05/2020	3	5
RR			BOA VISTA	140010	1	26/05/2020		1	2
SP	GVE I CAPI	1331	SAO PAULO	355030	1	24/09/2020		3	6
SP	GVE I CAPI	1331	SAO PAULO	355030	1	22/05/2020	19/06/2020	2	5
SP	GVE VII SA	1332	SAO CAETA	354880	1	21/03/2020	22/03/2020	9	9
MG	UBERLAND	1462	UBERLAND	317020	1	20/07/2020		2	6
SP	GVE I CAPI	1331	SAO PAULO	355030	1	08/06/2020	09/06/2020	2	5
CE			FORTALEZA	230440	1	20/12/2020	25/12/2020	2	
AM	ENTORNO	5584	MANAUS	130260	1	04/07/2020	08/07/2020	3	
SP	GVE VIII M	1333	MOGI DAS	353060	1	15/11/2020	21/11/2020	1	6
MT	CUIABA	1578	CUIABA	510340	1	21/07/2020	22/07/2020	9	6
RJ			RIO DE JAN	330455	1	26/11/2020		2	6
ES	METROPOL	1510	SERRA	320500	1	24/12/2020			6
RJ			CAMPOS D	330100	1	04/08/2020		3	5
SP	GVE I CAPI	1331	SAO PAULO	355030	1	15/12/2020	21/12/2020	2	6
SP	GVE XVII C	1342	JUNDIAI	352590	1	03/05/2020		3	5
AL	1 MICRORR	1533	MACEIO	270430	1	30/06/2020	03/07/2020	1	6
GO			GOIANIA	520870	1	02/09/2020		2	
RJ			RIO DE JAN	330455	1	14/11/2020		1	
RJ			NILOPOLIS	330320	1	09/09/2020		2	3
MG	GOVERNAD	1471	GOVERNAD	312770	1	23/07/2020	23/07/2020	1	9
CE	1 CRES FOR	1519	FORTALEZA	230440	1	23/05/2020	24/05/2020	2	6
SP	GVE I CAPI	1331	SAO PAULO	355030	1	23/12/2020		1	2
RJ			CAMPOS D	330100	1	23/05/2020		3	6
AM	ENTORNO	5584	MANAUS	130260	1	29/04/2020	10/05/2020	2	2
AM	ENTORNO	5584	MANAUS	130260	1	21/11/2020	30/11/2020	3	6
MG	BELO HORI	1449	BELO HORI	310620	1	29/05/2020	29/05/2020	1	6
PE	001	1497	RECIFE	261160	1	26/04/2020	28/04/2020	1	2
SP	GVE I CAPI	1331	SAO PAULO	355030	1	28/08/2020	10/09/2020	2	6
PR	02RS METR	1356	CAMPINA (	410400	1	09/12/2020	20/12/2020	1	2
GO	ENTORNO	1784	FORMOSA	520800	1	30/12/2020	07/01/202	2	
PB	I NRS JOAO	1377	JOAO PESS	250750	1	13/07/2020		2	9
PI	4 GRS TERE	1883	TERESINA	221100	1	22/06/2020			5
RJ			RIO DE JAN	330455	1	02/05/2020			6

MS	NRS DE DO	1966	DOURADOS	500370	1	12/10/2020	14/10/2020	1	
RJ			RIO DE JAN	330455	1	21/11/2020		2	
PI	4 GRS TERE	1883	TERESINA	221100	1	28/05/2020		2	2
MG	POUSO ALE	1459	POCOS DE	315180	1	27/12/2020	31/12/2020	2	2
RJ			RIO DE JAN	330455	1	02/05/2020		2	2
ES	METROPOL	1510	SERRA	320500	1	05/11/2020	28/12/2020	1	
PA	12 REGION	1495	REDENCAO	150613	1	11/08/2020		1	6
RS	002 CRS	1607	PORTO ALE	431490	1	16/07/2020	23/07/2020	2	4
SP	GVE I CAPI	1331	SAO PAULO	355030	1	06/11/2020	08/11/2020	2	
ES	METROPOL	1510	SERRA	320500	1	08/12/2020	02/01/202	1	
RO	VI GRS POR	1483	PORTO VEL	110020	1	30/06/2020	12/07/2020	1	6
RS	001 CRS	1592	CANOAS	430460	1	26/11/2020	28/11/2020	2	2
MA	REGI0NAL	1430	SAO LUIS	211130	1	26/05/2020	13/06/2020	1	
PI	4 GRS TERE	1883	TERESINA	221100	1	23/05/2020	25/05/2020	2	5
RJ			ITAPERUNA	330220	1	21/08/2020	25/08/2020	3	6
CE			FORTALEZA	230440	1	14/11/2020		2	
AL	1 MICRORR	1533	MACEIO	270430	1	14/05/2020	21/05/2020	1	
SP	GVE IX FRA	1334	CAIEIRAS	350900	1	22/04/2020		1	2
SP	GVE I CAPI	1331	SAO PAULO	355030	1	01/05/2020	05/05/2020	2	5
PR	02RS METR	1356	CURITIBA	410690	1	06/12/2020	18/12/2020	3	5
RJ			NILOPOLIS	330320	1	05/05/2020			
RS	002 CRS	1607	PORTO ALE	431490	1	26/06/2020	08/07/2020	1	2
RJ			NOVA FRIB	330340	1	01/05/2020		9	9
MG	BELO HORI	1449	BELO HORI	310620	1	16/07/2020	18/07/2020	2	6
SP	GVE I CAPI	1331	SAO PAULO	355030	1	09/01/202	10/01/202	2	6
MG	BELO HORI	1449	BETIM	310670	1	16/09/2020	18/09/2020	3	6
MG	BELO HORI	1449	CONTAGEM	311860	1	06/10/2020		9	
BA	NUCLEO RE	1381	FEIRA DE S	291080	1	03/12/2020		3	
GO	CENTRAL	1779	GOIANIA	520870	1	03/06/2020		2	
SP	GVE VIII M	1333	GUARULHO	351880	1	12/08/2020		3	2
SP	GVE XXIX S	1354	SAO JOSE D	354980	1	11/10/2020	19/10/2020	1	6
RN	II URSAP	1412	MOSSORO	240800	1	16/11/2020	29/11/2020	1	4
SP	GVE XXVII S	1351	SAO JOSE D	354990	1	09/07/2020		2	
PE	001	1497	CABO DE S	260290	1	27/04/2020		1	2
DF			BRASILIA	530010	1	14/06/2020	06/07/2020	1	5
SP	GVE XIX MA	1344	MARILIA	352900	1	17/08/2020	26/08/2020	2	6
CE	1 CRES FOR	1519	FORTALEZA	230440	1			2	
PR	02RS METR	1356	CURITIBA	410690	1	16/09/2020	25/09/2020	2	
SP	GVE I CAPI	1331	SAO PAULO	355030	1	09/11/2020		3	6
RS	007 CRS	1596	BAGE	430160	1	03/01/202	10/01/202	2	6
MA	REGIONAL	1447	CAXIAS	210300	1	14/06/2020		2	
RJ			PETROPOLI	330390	1	08/05/2020	15/06/2020	1	6
BA	NUCLEO RE	1380	SALVADOR	292740	1	07/06/2020	24/06/2020	2	
SC	CONCORDI	1560	CONCORDI	420430	1	21/11/2020		3	
RJ			RIO DE JAN	330455	1	16/12/2020	18/12/2020	2	
PA	12 REGION	1495	REDENCAO	150613	1	03/08/2020		3	1
BA	NUCLEO RE	1380	SALVADOR	292740	1	12/05/2020	16/05/2020	3	

SP	GVE I CAPI	1331	SAO PAULO	355030	1	04/07/202	07/07/202	3	9
PR	02RS METR	1356	CURITIBA	410690	1	28/06/202	13/07/202	1	6
CE	1 CRES FOR	1519	FORTALEZA	230440	1	25/05/202	26/05/202	2	
RJ			RIO DE JAN	330455	1	18/05/2020		3	
PA	01 REGION	1484	BELEM	150140	1	15/07/202	18/07/202	2	
GO	CENTRAL	1779	GOIANIA	520870	1	29/08/202	25/09/202	2	5
BA	NUCLEO RE	1380	SALVADOR	292740	1	04/12/2020		2	
AM	ENTORNO	5584	MANAUS	130260	1	05/09/202	01/10/202	1	2
PR	15RS MARI	1369	MARINGA	411520	1	30/11/202	15/12/202	1	6
PA	01 REGION	1484	BELEM	150140	1	09/01/202	11/01/202	2	
SP	GVE I CAPI	1331	SAO PAULO	355030	1	06/01/202	08/01/202	2	9
PE	001	1497	RECIFE	261160	1	08/05/2020		3	6
RJ			RIO DE JAN	330455	1	22/05/2020		1	5
PB	I NRS JOAO	1377	JOAO PESS	250750	1	24/07/202	26/07/202	3	6
SP	GVE XX PIR	1345	RIO CLARO	354390	1	05/06/2020		2	2
PR	02RS METR	1356	CURITIBA	410690	1	07/08/202	12/08/202	2	6
SP	GVE XVII CA	1342	CAMPINAS	350950	1	20/08/2020		2	5
GO			SANTA HEL	521930	1	11/09/2020		2	
TO			ARAGUAIN	170210	1	24/12/2020		3	6
SP	GVE I CAPI	1331	SAO PAULO	355030	1	20/08/2020		1	2
AM	ENTORNO	5584	MANAUS	130260	1	21/04/202	11/05/202	1	2
RJ			SAO JOAO	330510	1	22/12/2020		3	2
SP	GVE I CAPI	1331	SAO PAULO	355030	1	02/07/202	11/07/202	2	2
RJ			DUQUE DE	330170	1	26/11/2020		2	
SP	GVE I CAPI	1331	SAO PAULO	355030	1	18/07/202	24/07/202	3	6
RS	002 CRS	1607	PORTO ALE	431490	1	28/07/202	11/08/202	2	5
MT	CUIABA	1578	CUIABA	510340	1	08/08/202	10/08/202	2	9
SP	GVE XXV SA	1349	CUBATAO	351350	1	22/05/2020		3	6
MG	BELO HORI	1449	BELO HORI	310620	1	08/07/2020		2	1
MG	POUSO ALE	1459	ITAJUBA	313240	1	16/09/202	17/09/202	3	1
PR	02RS METR	1356	CURITIBA	410690	1	01/12/202	26/12/202	1	2
SP	GVE I CAPI	1331	SAO PAULO	355030	1	17/04/202	21/04/202	1	2
PR	02RS METR	1356	CURITIBA	410690	1	02/04/202	02/04/202	1	1
SP			SAO PAULO	355030	1	06/06/202	09/06/202	2	1
RJ			RIO DE JAN	330455	1	01/04/2020		2	2
SP	GVE XXIX S	1354	SAO JOSE D	354980	1	16/11/202	31/12/202	1	6
DF			BRASILIA	530010	1	13/08/202	16/08/202	3	2
RJ			RIO DE JAN	330455	1	14/12/2020		2	2
SP	GVE I CAPI	1331	SAO PAULO	355030	1	29/04/2020		3	2
PE	001	1497	RECIFE	261160	1	10/07/2020		2	6
CE	11 CRES SO	1521	SOBRAL	231290	1	22/07/2020		1	
AP			MACAPA	160030	1	20/08/2020		2	6
CE	21 CRES JU	1531	JUAZEIRO I	230730	1	24/06/2020		1	
RJ			DUQUE DE	330170	1	06/05/2020		2	2
MA	REGI0NAL	1430	SAO LUIS	211130	1	01/04/2020		1	6
MS	EX NRS DE	6256	CORUMBA	500320	1	12/12/2020		2	
GO	CENTRAL	1779	GOIANIA	520870	1	03/01/202	14/01/202	2	

PB	I NRS JOAC	1377	JOAO PESS	250750	1	21/06/202	27/06/202	3	9
PI	4 GRS TERE	1883	TERESINA	221100	1	21/05/202	22/06/202	3	6
SP	GVE I CAPI	1331	SAO PAULC	355030	1	21/12/2020		3	
SP	GVE XXI PR	1346	PRESIDENT	354140	1	03/01/202	07/01/202	1	6
MT	CUIABA	1578	CUIABA	510340	1	05/07/202	07/07/202	3	6
SP	GVE I CAPI	1331	SAO PAULC	355030	1	18/07/202	20/07/202	3	2
MG	TEOFILO O	1465	TEOFILO O	316860	1	23/08/2020		1	
PE	001	1497	RECIFE	261160	1	07/04/2020		2	6
PE	008	1503	PETROLINA	261110	1	18/07/2020		3	
SP	GVE I CAPI	1331	SAO PAULC	355030	1	02/12/202	04/12/202	3	
RJ			BOM JESUS	330060	1	08/12/202	24/12/202	1	2
PB	III NRS CAN	1421	CAMPINA (	250400	1	16/08/202	19/08/202	2	6
SP			SAO PAULC	355030	1	11/04/2020		3	
SP	GVE I CAPI	1331	SAO PAULC	355030	1	04/05/202	11/05/202	2	9
AL	1 MICRORI	1533	MACEIO	270430	1	16/05/202	21/05/202	1	1
RS	002 CRS	1607	PORTO ALE	431490	1	23/06/202	27/07/202	1	2
RJ			CAMPOS D	330100	1	28/11/202	29/11/202	1	6
BA	NUCLEO RI	1380	SALVADOR	292740	1	30/06/202	01/07/202	2	6
SP	GVE XXIV R	1348	RIBEIRAO F	354340	1	04/08/2020		1	2
SP	GVE XXIV R	1348	RIBEIRAO F	354340	1	21/10/2020			
SP			SAO PAULC	355030	1	01/01/2021		2	
SP	GVE I CAPI	1331	SAO PAULC	355030	1	06/08/202	14/08/202	2	6
RJ			ITAGUAI	330200	1	25/04/2020		1	2
PB	III NRS CAN	1421	CAMPINA (	250400	1	19/05/2020		3	6
SP	GVE XVII C	1342	CAMPINAS	350950	1	28/11/2020			
DF			BRASILIA	530010	1	27/07/202	05/08/202	1	6
SP	GVE XXV S	1349	SANTOS	354850	1	14/05/2020		2	4
SP	GVE I CAPI	1331	SAO PAULC	355030	1	15/04/202	21/04/202	2	3
SP	GVE XVII C	1342	CAMPINAS	350950	1	24/12/202	27/12/202	2	
RJ			RIO DE JAN	330455	1	26/11/202	04/12/202	1	6
SP	GVE I CAPI	1331	SAO PAULC	355030	1	08/05/202	13/05/2020		
SP	GVE I CAPI	1331	SAO PAULC	355030	1	15/01/202	26/01/202	2	2
SP	GVE I CAPI	1331	SAO PAULC	355030	1	25/05/202	30/05/202	3	
SP	GVE XVII C	1342	JUNDIAI	352590	1	04/08/2020		2	2
SP	GVE I CAPI	1331	SAO PAULC	355030	1	18/06/202	28/06/202	3	
TO			PALMAS	172100	1	09/12/202	18/12/202	1	6
BA	NUCLEO RI	1380	SALVADOR	292740	1	11/05/202	13/05/202	3	
SP	GVE XI ARA	1336	PENAPOLIS	353730	1	12/07/2020		1	4
PE	001	1497	RECIFE	261160	1	28/07/2020		2	6
MG	UBA	1464	UBA	316990	1	04/12/2020			
GO	CENTRAL	1779	GOIANIA	520870	1	17/06/202	22/06/202	1	5
RJ			RIO DE JAN	330455	1	09/12/202	10/12/202	2	6
PB	I NRS JOAC	1377	JOAO PESS	250750	1	22/08/202	09/09/202	3	6
BA	NUCLEO RI	1385	ILHEUS	291360	1	30/09/2020		1	6
BA	NUCLEO RI	1380	SALVADOR	292740	1	13/05/202	16/05/202	3	6
SP	GVE I CAPI	1331	SAO PAULC	355030	1	24/06/202	07/07/202	1	2
SP			SAO PAULC	355030	1	06/12/202	19/12/202	1	

CE	1 CRES FOF	1519	FORTALEZA	230440	1	22/06/202	24/06/202	1	9
MG	PATOS DE I	1456	PATOS DE I	314800	1	21/05/2020		2	
CE	1 CRES FOF	1519	FORTALEZA	230440	1	31/05/202	06/06/202	2	
SP			SAO PAULC	355030	1	06/05/202	11/05/202	3	6
PR	03RS PONT	1357	PONTA GR	411990	1	28/09/202	07/10/202	2	6
GO	CENTRAL	1779	GOIANIA	520870	1	06/07/2020			
GO	CENTRAL	1779	GOIANIA	520870	1	07/08/202	10/08/202	3	5
RO	VI GRS POF	1483	PORTO VEL	110020	1	28/05/202	08/06/202	2	9
SP	GVE XX PIR	1345	PIRACICAB	353870	1	11/07/202	24/10/202	2	
RJ			NOVA IGUA	330350	1			1	6
MG			ROMARIA	315640	1	04/02/2021		2	9
SP	GVE I CAPI	1331	SAO PAULC	355030	1	16/03/2021		2	
RJ			RIO DE JAN	330455	1	21/03/202	24/03/202	2	6
SP	GVE I CAPI	1331	SAO PAULC	355030	1	18/03/2021		2	2
GO	CENTRAL	1779	GOIANIA	520870	1	14/04/2021		3	
SP	GVE I CAPI	1331	SAO PAULC	355030	1	17/04/202	23/04/202	1	
RS	005 CRS	1608	VACARIA	432250	1	30/05/202	25/06/202	1	
RR			BOA VISTA	140010	1	26/05/202	06/06/202	1	
DF			BRASILIA	530010	1	22/06/202	16/07/202	1	6
BA	NUCLEO RI	1381	FEIRA DE S.	291080	1	22/06/2021		2	6
SP	GVE VIII M	1333	GUARULHC	351880	1	28/08/202	02/09/202	2	2
RN	VII URSAP	1416	NATAL	240810	1	06/04/2021		2	3
PA	01 REGION	1484	BELEM	150140	1	06/02/202	08/02/202	1	2
MG	BELO HORI	1449	CONTAGEN	311860	1	19/02/2021		2	9
SP	GVE XVII C.	1342	CAMPINAS	350950	1	23/02/202	07/03/202	1	6
MG			CONTAGEN	311860	1	08/03/202	21/03/202	1	
SP	GVE I CAPI	1331	SAO PAULC	355030	1	15/03/202	23/03/202	2	6
SP	GVE IX FRA	1334	FRANCISCC	351630	1	18/04/2021		2	
ES	METROPOI	1510	SERRA	320500	1	24/03/202	24/05/202	1	2
SP	GVE XXVII S	1351	SAO JOSE D	354990	1	21/06/202	02/07/202	1	6
RJ			RIO DE JAN	330455	1	11/06/2021		2	
RJ			RIO DE JAN	330455	1	20/06/2021		2	
DF			BRASILIA	530010	1	21/09/202	24/09/202	2	6
RN	VII URSAP	1416	NATAL	240810	1	31/05/202	04/06/202	1	
GO	CENTRAL	1779	GOIANIA	520870	1	01/01/202	04/01/202	2	
RJ			NITEROI	330330	1	07/03/2021		2	2
GO	CENTRAL	1779	GOIANIA	520870	1	25/03/2021			
RJ			RIO DE JAN	330455	1	20/03/2021		2	
RJ			RIO DE JAN	330455	1	27/03/2021		2	
PB	I NRS JOAC	1377	JOAO PESS	250750	1			2	
CE	1 CRES FOF	1519	FORTALEZA	230440	1	18/03/202	21/03/202	3	3
GO	CENTRAL	1779	GOIANIA	520870	1	03/06/202	10/06/202	1	6
CE	1 CRES FOF	1519	FORTALEZA	230440	1	29/03/202	23/04/202	1	2
RS	002 CRS	1607	PORTO ALE	431490	1	07/06/202	03/07/202	1	2
MT			PRIMAVER	510704	1	04/07/202	27/07/2021		
ES	METROPOI	1510	SERRA	320500	1	16/07/2021		2	2
MG	BELO HORI	1449	BETIM	310670	1	09/08/2021			

SP	GVE I CAPI	1331	SAO PAULO	355030	1	19/05/202	25/06/202	1	2
MG	DIVINOPOI	1472	BOM DESP	310740	1	06/09/2021		2	
RN	VII URSAP	1416	NATAL	240810	1	01/04/202	12/04/202	1	9
ES	METROPOI	1510	VITORIA	320530	1	10/04/202	15/04/202	1	
SP	GVE XXIV F	1348	RIBEIRAO F	354340	1	23/05/202	27/05/202	2	4
ES	METROPOI	1510	SERRA	320500	1	30/04/202	22/05/202	1	2
SP	GVE XXIX S	1354	SAO JOSE D	354980	1	12/06/202	08/07/202	1	4
CE	1 CRES FOF	1519	FORTALEZA	230440	1	24/03/202	06/05/202	1	3
PR	15RS MARI	1369	SARANDI	412625	1	14/06/202	04/07/202	1	
RS	002 CRS	1607	PORTO ALE	431490	1	21/07/202	27/07/202	2	
MS	EX ARS DE	1975	CAMPO GF	500270	1	21/07/202	15/08/202	1	
PR	16RS APUC	1370	APUCARAN	410140	1	21/05/202	19/06/202	1	
MT	CUIABA	1578	CUIABA	510340	1	24/08/202	18/09/202	2	
SC	JOINVILLE	1565	JOINVILLE	420910	1	03/09/202	06/09/202	2	2
GO	CENTRAL	1779	GOIANIA	520870	1	19/09/202	23/09/202	2	
RS	006 CRS	1611	PASSO FUN	431410	1	03/02/202	13/02/202	2	6
SP	GVE I CAPI	1331	SAO PAULO	355030	1	26/02/202	01/03/202	2	
SP	GVE XII AR	1337	ARARAQU	350320	1	25/03/202	31/03/202	2	6
RJ			RIO DE JAN	330455	1	30/03/2021		3	6
MA	REGI0NAL	1430	SAO LUIS	211130	1	03/04/202	06/04/202	2	6
SP	GVE XVII C	1342	VALINHOS	355620	1	13/04/2021		3	5
SP	GVE X OSA	1335	OSASCO	353440	1	09/04/2021		1	6
PB	III NRS CAN	1421	CAMPINA (	250400	1	18/05/202	24/05/202	2	
RJ			RIO DE JAN	330455	1	27/04/2021		2	3
SP			SAO PAULO	355030	1	13/05/2021		2	
CE	1 CRES FOF	1519	FORTALEZA	230440	1	26/02/202	03/03/202	3	3
CE	1 CRES FOF	1519	FORTALEZA	230440	1	26/02/202	03/03/202	3	3
PA	10 REGION	1493	ALTAMIRA	150060	1	06/06/2021		1	
SP	GVE I CAPI	1331	SAO PAULO	355030	1	15/02/202	18/02/202	2	
MT	CUIABA	1578	CUIABA	510340	1	27/09/202	10/10/202	1	
MT	CUIABA	1578	CUIABA	510340	1	23/05/202	11/06/202	1	2
GO	CENTRO SU	1781	APARECIDA	520140	1			3	6
RJ			RIO DE JAN	330455	1	04/09/202	22/09/202	1	6
RJ			RIO DE JAN	330455	1	16/03/2021		1	
RJ			RIO DE JAN	330455	1	14/09/202	14/10/202	1	6
RJ			RIO DE JAN	330455	1	21/09/202	20/10/202	1	6
GO	ESTRADA D	1802	CATALAO	520510	1	02/01/202	06/01/202	3	6
AM	ENTORNO	5584	MANAUS	130260	1	26/01/202	28/02/202	1	
PA	09 REGION	1492	MONTE AL	150480	1	03/03/202	25/03/202	1	6
GO	CENTRAL	1779	GOIANIA	520870	1	15/03/202	22/03/202	1	
SP	GVE I CAPI	1331	SAO PAULO	355030	1	14/03/202	25/03/202	1	2
SP	GVE VIII M	1333	ITAQUAQU	352310	1	24/04/202	29/04/202	2	
RS	018 CRS	1605	TRAMAND	432160	1	30/04/202	24/05/202	2	
SC	JARAGUA D	1567	JARAGUA D	420890	1	30/05/202	31/05/202	2	6
CE	1 CRES FOF	1519	FORTALEZA	230440	1	02/05/202	09/05/202	3	6
CE	11 CRES SC	1521	SOBRAL	231290	1	24/06/202	26/06/202	2	5
MG	BELO HORI	1449	BELO HORI	310620	1	14/06/2021			2

RS	006 CRS	1611	CARAZINH	430470	1	03/09/202	07/09/202	2	
DF			BRASILIA	530010	1	26/10/202	01/11/202	2	9
SP	GVE XVII C	1342	CAMPINAS	350950	1	14/02/2021		2	
RS	002 CRS	1607	PORTO ALE	431490	1	26/03/202	30/03/202	2	1
SP	GVE XVII C	1342	CAMPINAS	350950	1	26/03/202	07/04/202	1	6
BA	NUCLEO RE	1380	SALVADOR	292740	1	13/03/202	28/03/202	1	6
SP	GVE XVII C	1342	CAMPINAS	350950	1	29/03/202	31/03/202	2	
CE	1 CRES FO	1519	FORTALEZ	230440	1	25/03/202	30/03/202	1	1
SP	GVE XXXI S	1353	ITAPETININ	352230	1	06/04/2021			
SP	GVE XVII C	1342	JUNDIAI	352590	1	04/04/2021		2	9
MA	REGI0NAL	1430	SAO LUIS	211130	1	09/04/202	11/04/202	2	
SP	GVE I CAPI	1331	SAO PAUL	355030	1	28/05/202	31/05/202	1	2
RJ			DUQUE DE	330170	1			3	6
SP	GVE XVII C	1342	CAMPINAS	350950	1	15/06/202	24/06/202	1	
SP	GVE I CAPI	1331	SAO PAUL	355030	1	23/06/202	26/06/202	3	
PR	10RS CASC	1364	CASCAVEL	410480	1	10/08/202	13/08/202	3	
RS	001 CRS	1592	NOVO HAM	431340	1	29/11/202	05/12/202	2	3
MG	BELO HORI	1449	MATEUS LE	314070	1	21/02/202	25/02/202	1	2
SP	GVE I CAPI	1331	SAO PAUL	355030	1	20/02/202	21/02/202	2	6
SP	GVE I CAPI	1331	SAO PAUL	355030	1	06/04/202	23/04/202	2	6
PR	11RS CAMI	1365	CAMPO M	410430	1	17/05/202	27/05/202	2	6
PR	04RS IRATI	1358	IRATI	411070	1	22/05/202	04/06/202	1	
SP	GVE I CAPI	1331	SAO PAUL	355030	1	31/05/202	17/06/202	1	1
MG	MONTES C	1473	TAIOBEIRA	316800	1	25/05/202	10/06/202	2	6
MA	REGI0NAL	1430	SAO LUIS	211130	1	02/07/2021		2	
PA	01 REGION	1484	BELEM	150140	1	19/06/2021		2	6
SP			SAO PAUL	355030	1	29/06/202	01/07/202	2	
PI	4 GRS TERE	1883	TERESINA	221100	1	23/07/202	08/08/202	1	6
RS	005 CRS	1608	CAXIAS DO	430510	1	21/07/202	23/07/202	2	6
SP	GVE I CAPI	1331	SAO PAUL	355030	1	08/07/202	15/07/202	1	
CE	1 CRES FO	1519	FORTALEZ	230440	1	10/04/202	15/04/202	2	
RJ			NILOPOLIS	330320	1	29/09/202	04/10/202	3	2
					1	06/01/2022		1	
SP	GVE I CAPI	1331	SAO PAUL	355030	1	24/03/2021		2	9
MT	CUIABA	1578	CUIABA	510340	1	11/04/202	28/04/202	1	
GO	CENTRAL	1779	GOIANIA	520870	1	05/04/202	06/05/202	1	
PR	08RS FRAN	1362	AMPERE	410100	1	10/05/202	05/06/202	2	
SP	GVE XI AR	1336	ARACATUB	350280	1			1	
MG	BELO HORI	1449	NOVA LIM	314480	1	07/05/202	30/05/202	1	5
PR	10RS CASC	1364	CASCAVEL	410480	1	08/05/202	13/05/202	3	
RJ			RIO DE JAN	330455	1	19/05/202	23/05/202	2	6
MA	REGI0NAL	1430	SAO LUIS	211130	1	18/05/202	31/05/202	2	5
SP	GVE XXIX S	1354	SAO JOSE D	354980	1	13/06/202	25/07/202	1	4
CE	1 CRES FO	1519	FORTALEZ	230440	1	13/06/202	16/06/202	3	1
SP	GVE I CAPI	1331	SAO PAUL	355030	1	10/07/202	14/07/202	2	2
DF			BRASILIA	530010	1	07/10/202	10/10/202	2	6
SP	GVE XXXI S	1353	SAO ROQU	355060	1	16/08/2021		3	6

RJ			RIO DE JAN	330455	1	14/08/202	20/08/202	1	5
RS	018 CRS	1605	TRAMAND.	432160	1	29/07/202	04/08/202	2	
RJ			NILOPOLIS	330320	1	11/01/2021		2	2
RJ			RIO DE JAN	330455	1	08/09/2021		2	6
PE	008	1503	PETROLINA	261110	1	30/11/2021		1	6
DF			BRASILIA	530010	1	04/03/202	06/04/202	3	9
RS	002 CRS	1607	PORTO ALE	431490	1	08/03/202	16/03/202	1	5
PB	I NRS JOAC	1377	JOAO PESS	250750	1	13/04/202	15/04/202	3	6
PB	III NRS CAN	1421	CAMPINA (	250400	1	23/05/202	30/05/202	1	6
SP			SAO PAULO	355030	1	21/04/202	21/04/202	3	
DF			BRASILIA	530010	1	23/05/202	11/06/202	1	6
PA	11 REGION	1494	MARABA	150420	1	15/05/2021		2	2
MT	CUIABA	1578	CUIABA	510340	1	02/06/202	05/06/202	2	
CE			FORTALEZA	230440	1	16/05/202	21/05/202	3	2
SP	GVE XVI BC	1341	BOTUCATU	350750	1	22/06/202	15/07/202	2	6
GO	CENTRAL	1779	GOIANIA	520870	1	29/05/202	31/05/202	2	
BA	NUCLEO RI	1380	SALVADOR	292740	1	19/06/202	24/06/202	2	9
AM	ENTORNO	5584	MANAUS	130260	1	03/07/202	12/07/202	1	2
SP	GVE I CAPI	1331	SAO PAULO	355030	1	21/10/202	25/10/202	2	
BA	NUCLEO RI	1380	SALVADOR	292740	1	02/08/202	11/08/202	2	9
SP	GVE I CAPI	1331	SAO PAULO	355030	1	26/12/2021		2	
MT	CUIABA	1578	CUIABA	510340	1	20/09/202	24/09/202	2	
SP	GVE XXXI S	1353	SOROCABA	355220	1	15/01/202	16/02/2021		9
AM	ENTORNO	5584	MANAUS	130260	1	13/01/202	15/01/202	2	6
BA	NUCLEO RI	1380	SALVADOR	292740	1	04/03/2021		2	5
SP	GVE XI ARA	1336	ARACATUB	350280	1	16/03/202	25/03/202	1	6
SP	GVE I CAPI	1331	SAO PAULO	355030	1	14/03/202	29/03/202	1	2
RJ			RIO DE JAN	330455	1	07/04/2021		2	
MG	BELO HORI	1449	CONTAGEN	311860	1	06/04/2021		1	5
SP	GVE I CAPI	1331	SAO PAULO	355030	1	13/04/202	15/04/202	2	6
PR	10RS CASC	1364	CASCAVEL	410480	1	17/06/202	21/06/202	3	
RJ			SAO GONC	330490	1	05/05/2021		1	9
BA	NUCLEO RI	1380	SALVADOR	292740	1	28/10/202	31/10/202	2	9
SP	GVE I CAPI	1331	SAO PAULO	355030	1	29/03/202	11/04/202	2	
SP	GVE XXIX S	1354	VOTUPORA	355710	1			2	6
SP	GVE I CAPI	1331	SAO PAULO	355030	1	30/04/202	12/05/202	1	6
MT	CUIABA	1578	CUIABA	510340	1	04/04/202	17/04/202	1	9
DF			BRASILIA	530010	1	11/05/202	28/05/202	1	6
MA	REGI0NAL	1430	SAO LUIS	211130	1	22/05/202	31/05/202	2	9
SP	GVE I CAPI	1331	SAO PAULO	355030	1	26/01/202	28/01/202	2	
MG	ITABIRA	1451	ITABIRA	313170	1	14/05/2021		2	
SP	GVE VII SA	1332	MAUA	352940	1	11/07/202	20/07/202	3	1
SP	GVE I CAPI	1331	SAO PAULO	355030	1	09/06/202	12/06/202	3	
SP	GVE I CAPI	1331	SAO PAULO	355030	1	09/06/202	24/06/202	1	
MT	CUIABA	1578	CUIABA	510340	1	26/05/202	27/05/202	2	6
CE	1 CRES FOF	1519	FORTALEZA	230440	1	22/06/202	23/06/202	3	6
SP	GVE I CAPI	1331	SAO PAULO	355030	1	12/01/202	24/01/202	2	4

MG	UBERLAND	1462	UBERLAND	317020	1	25/02/2021		2	2
DF			BRASILIA	530010	1	14/03/202	25/03/202	1	
RO	VI GRS POF	1483	PORTO VEL	110020	1	05/03/202	10/03/202	2	9
PR	02RS METF	1356	CURITIBA	410690	1	18/03/202	21/03/202	2	6
PB	I NRS JOAC	1377	JOAO PESS	250750	1	27/03/2021		1	6
SP	GVE I CAPI	1331	SAO PAULC	355030	1	14/04/202	19/04/202	2	3
MT	CUIABA	1578	CUIABA	510340	1	07/04/2021		3	
RS	011 CRS	1600	ERECHIM	430700	1	16/06/202	25/07/202	2	
PA	01 REGION	1484	BELEM	150140	1				
SC	ITAJAI	1550	BALNEARIC	420200	1	27/07/202	31/07/202	2	6
PA	12 REGION	1495	REDENCAC	150613	1	06/08/2021		2	6
CE	1 CRES FOF	1519	FORTALEZA	230440	1	14/04/202	19/04/202	2	
PA	01 REGION	1484	BELEM	150140	1	28/11/202	30/11/202	2	5
SP	GVE I CAPI	1331	SAO PAULC	355030	1	03/02/2021			
PR	10RS CASC	1364	CASCAVEL	410480	1	08/10/202	09/11/202	2	
SC	JOINVILLE	1565	JOINVILLE	420910	1	07/12/2021		1	2
PR	10RS CASC	1364	CASCAVEL	410480	1	10/01/202	12/01/202	3	6
SP	GVE VII SAI	1332	SANTO AN	354780	1	29/03/2021		3	9
CE	1 CRES FOF	1519	FORTALEZA	230440	1	06/03/2021		1	
GO	CENTRAL	1779	GOIANIA	520870	1	03/04/2021		2	
SP	GVE I CAPI	1331	SAO PAULC	355030	1	31/03/202	02/04/202	2	2
SP	GVE I CAPI	1331	SAO PAULC	355030	1	08/03/202	10/03/202	2	6
RR			BOA VISTA	140010	1	08/03/202	29/03/202	1	
SP	GVE XXXI S	1353	SOROCABA	355220	1	26/05/202	28/05/202	2	2
SP	GVE XVII CA	1342	CAMPINAS	350950	1	11/04/2021		1	
RN	II URSAP	1412	MOSSORO	240800	1	27/05/202	04/06/202	1	6
RJ			RIO DAS OS	330452	1	31/03/2021			
PB	I NRS JOAC	1377	JOAO PESS	250750	1	17/04/2021		3	6
RJ			RIO DE JAN	330455	1	06/05/202	21/05/202	1	6
SP	GVE I CAPI	1331	SAO PAULC	355030	1	12/05/202	08/06/202	2	3
PR	02RS METF	1356	CURITIBA	410690	1	04/05/202	05/05/202	2	6
PR	10RS CASC	1364	CASCAVEL	410480	1	25/05/202	15/06/202	2	
SC	JOINVILLE	1565	JOINVILLE	420910	1	05/06/202	12/06/202	1	6
SP	GVE I CAPI	1331	SAO PAULC	355030	1	12/06/202	15/06/202	1	2
RS	003 CRS	1593	RIO GRANI	431560	1	28/06/202	05/07/202	1	6
DF			BRASILIA	530010	1	28/07/202	04/08/202	2	6
MT	CUIABA	1578	CUIABA	510340	1	16/05/202	18/05/202	2	6
GO	CENTRAL	1779	GOIANIA	520870	1	20/08/202	30/08/202	2	
AM	ENTORNO	5584	MANAUS	130260	1	15/02/202	20/02/202	2	
MG	UBERLAND	1462	UBERLAND	317020	1			1	6
CE	1 CRES FOF	1519	FORTALEZA	230440	1	29/01/202	15/02/202	1	9
SP	GVE I CAPI	1331	SAO PAULC	355030	1	13/03/202	20/03/202	2	6
PA	01 REGION	1484	BELEM	150140	1	23/03/202	29/03/202	1	6
DF			BRASILIA	530010	1	11/03/202	23/04/202	1	9
BA	NUCLEO RI	1380	SALVADOR	292740	1	02/05/202	05/05/202	2	
SP			GUARULHC	351880	1	16/05/2021		2	2
BA			SALVADOR	292740	1	21/05/202	22/05/202	3	

RN	VI URSAP	1417	PAU DOS F	240940	1	26/05/2021	2	6
SP	GVE I CAPI	1331	SAO PAULO	355030	1	25/05/202 25/05/202	1	
PA	01 REGION	1484	BELEM	150140	1	06/06/2021		2
SP	GVE I CAPI	1331	SAO PAULO	355030	1	18/01/202 22/01/202	2	
SP	GVE XX PIR	1345	LEME	352670	1	26/05/202 01/06/202	3	6
PR	09RS FOZ D	1363	FOZ DO IGU	410830	1	02/09/202 08/09/202	2	6
SC	ITAJAI	1550	BALNEARIO	420200	1	18/02/202 01/03/202	2	6
MG	BELO HORI	1449	CONTAGEM	311860	1	05/03/2021	2	
CE	1 CRES FOR	1519	FORTALEZA	230440	1	19/03/202 26/03/202	1	
MG	ALFENAS	1379	ALFENAS	310160	1	24/05/202 30/06/202	1	6
RN	VII URSAP	1416	NATAL	240810	1	03/05/202 09/05/202	1	9
CE	1 CRES FOR	1519	FORTALEZA	230440	1	01/05/202 20/05/202	1	
SP	GVE I CAPI	1331	SAO PAULO	355030	1	14/06/202 19/06/202	2	6
CE	1 CRES FOR	1519	FORTALEZA	230440	1	24/03/202 28/03/202	1	3
GO	CENTRAL	1779	GOIANIA	520870	1	06/07/202 09/07/202	2	
DF			BRASILIA	530010	1	27/06/202 02/07/202	2	6
BA	NUCLEO RE	1380	SALVADOR	292740	1	08/07/202 10/07/202	2	
AM	ENTORNO	5584	MANAUS	130260	1	24/07/202 16/08/202	3	6
PR	09RS FOZ D	1363	FOZ DO IGU	410830	1	26/08/202 27/08/202	3	6
SP	GVE XXVII S	1351	SAO JOSE D	354990	1	16/01/2021	3	
TO			ARAGUAIN	170210	1	12/01/2021	2	6
RS	013 CRS	1609	SANTA CRU	431680	1	04/03/202 26/03/202	1	6
SP	GVE I CAPI	1331	SAO PAULO	355030	1	06/03/202 21/03/202	3	
AM	ENTORNO	5584	MANAUS	130260	1	04/04/2021		
SP	GVE I CAPI	1331	SAO PAULO	355030	1	15/03/202 20/03/202	1	2
RS	002 CRS	1607	PORTO ALE	431490	1	04/03/202 16/04/202	1	3
MA	REGIONAL	1446	BACABAL	210120	1	25/04/202 05/05/202	1	
RJ			RIO DE JAN	330455	1	08/04/202 11/04/202	2	6
RR			BOA VISTA	140010	1	06/04/2021	1	
MG	DIVINOPOL	1472	DIVINOPOL	312230	1	21/05/202 06/06/202	1	
SP	GVE XI ARA	1336	AURIFLAM	350420	1	27/05/2021	2	9
BA	NUCLEO RE	1380	SALVADOR	292740	1	19/05/202 29/05/202	1	
SP	GVE I CAPI	1331	SAO PAULO	355030	1	30/08/202 10/09/202	2	6
PR	01RS PARA	1355	PARANAGU	411820	1	16/03/202 16/03/202	1	6
MT	CUIABA	1578	CUIABA	510340	1	24/03/202 14/04/202	1	
RJ			RIO DE JAN	330455	1	19/03/2021	1	
PR	17RS LOND	1371	LONDRINA	411370	1	17/03/202 24/03/202	2	6
PR	15RS MARI	1369	MARINGA	411520	1	12/02/202 07/03/202	1	6
AM			MANAUS	130260	1	14/01/202 24/01/202	1	2
MG	PONTE NO	1458	PONTE NO	315210	1	22/03/2021	2	6
SP	GVE I CAPI	1331	SAO PAULO	355030	1	15/03/202 16/03/202	3	2
GO	CENTRAL	1779	GOIANIA	520870	1	21/03/2021	1	
MG	PONTE NO	1458	PONTE NO	315210	1	09/05/2021	2	6
PR	02RS METR	1356	CURITIBA	410690	1	08/04/202 24/04/202	2	6
PA	01 REGION	1484	BELEM	150140	1	28/04/2021	2	1
SP	GVE I CAPI	1331	SAO PAULO	355030	1	16/04/202 29/04/202	1	2
SP	GVE VII SA	1332	MAUA	352940	1	02/06/2021	2	6

DF			BRASILIA	530010	1	20/05/202	08/06/202	1	6
PB					1	30/05/2021		2	
RJ			MACAE	330240	1	17/08/2021			
MG	JUIZ DE FO	1452	JUIZ DE FO	313670	1	25/05/202	30/05/202	2	9
CE	1 CRES FOF	1519	FORTALEZ/	230440	1	29/03/202	07/04/202	1	
PA	01 REGION	1484	BELEM	150140	1	21/07/2021		1	6
TO			PALMAS	172100	1	25/08/202	31/08/2021		6
PR	03RS PONT	1357	PONTA GR(	411990	1	19/10/2021		3	6
PA	12 REGION	1495	REDENCAC	150613	1	18/02/2021		3	2
TO			PORTO NA(	171820	1	03/04/202	09/04/202	2	6
CE	1 CRES FOF	1519	FORTALEZ/	230440	1	27/03/202	09/04/202	1	
RJ			RIO DE JAN	330455	1	06/03/202	14/04/202	1	6
SC			FLORIANOI	420540	1	24/04/202	06/05/202	1	
PR	10RS CASC	1364	CASCAVEL	410480	1	26/05/202	14/06/202	3	
SP	GVE XXXI S	1353	SOROCABA	355220	1	31/05/202	12/06/202	1	9
AP			OIAPOQUE	160050	1			3	
CE	1 CRES FOF	1519	FORTALEZ/	230440	1			2	6
SP	GVE I CAPI	1331	SAO PAUL(	355030	1	12/04/2021		2	6
MG	BELO HORI	1449	BETIM	310670	1	08/04/2021		1	6
SP	GVE I CAPI	1331	SAO PAUL(	355030	1	27/05/202	04/06/202	3	
SP	GVE X OSA	1335	ITAPEVI	352250	1	10/04/202	12/06/202	1	
PR	02RS METF	1356	CURITIBA	410690	1	19/05/202	20/05/202	2	6
MT	CUIABA	1578	CUIABA	510340	1	23/06/2021		1	
TO			PALMAS	172100	1	13/08/202	27/08/202	1	6
AM	ENTORNO	5584	MANAUS	130260	1	25/01/202	25/01/202	1	6
PR	09RS FOZ D	1363	FOZ DO IGU	410830	1	19/05/202	10/06/202	1	
SP	GVE I CAPI	1331	SAO PAUL(	355030	1	16/03/202	20/03/2021		6
SP	GVE VIII M	1333	GUARULH(	351880	1	13/03/202	08/04/202	3	2
MG	DIVINOPOI	1472	DIVINOPOI	312230	1	10/03/202	15/03/202	2	
SP	GVE XXIX S	1354	SAO JOSE D	354980	1	01/04/202	21/06/202	1	4
RJ			RIO DE JAN	330455	1	13/04/202	16/05/202	1	
RJ			RIO DE JAN	330455	1	17/06/2021		1	6
PR	09RS FOZ D	1363	FOZ DO IGU	410830	1	21/02/202	03/03/202	1	6
SP	GVE VIII M	1333	GUARULH(	351880	1	31/03/202	04/06/202	1	2
SP			SAO BERN/	354870	1	05/01/202	07/01/202	1	2
PA	01 REGION	1484	BELEM	150140	1	23/11/2021		1	9
SP	GVE XXIX S	1354	SAO JOSE D	354980	1	09/01/202	19/01/202	1	6
PR	02RS METF	1356	CURITIBA	410690	1	15/01/202	01/02/202	2	5
PA	09 REGION	1492	MONTE AL	150480	1	02/02/202	28/02/202	1	6
BA	NUCLEO RI	1380	SALVADOR	292740	1	04/04/202	06/04/202	2	9
RS	004 CRS	1595	SANTA MA	431690	1	07/03/202	21/03/202	1	6
SP	GVE XXIX S	1354	SAO JOSE D	354980	1	14/05/202	24/05/202	2	4
PE	001	1497	RECIFE	261160	1	12/04/2021		2	6
CE	1 CRES FOF	1519	FORTALEZ/	230440	1	30/05/202	01/06/202	3	3
MG	TEOFILO O	1465	TEOFILO O	316860	1	30/05/2021		2	9
SP	GVE I CAPI	1331	SAO PAUL(	355030	1	05/05/202	16/06/202	1	
SP	GVE XXIV R	1348	RIBEIRAO F	354340	1	13/06/202	15/06/202	2	3

SP	GVE XIX M	1344	MARILIA	352900	1	03/06/202	19/06/202	2	6
BA	NUCLEO RI	1380	SALVADOR	292740	1	20/05/202	20/05/202	3	
SP			ITAPIRA	352260	1	16/06/202	27/06/202	1	6
GO	CENTRAL	1779	GOIANIA	520870	1	20/06/202	13/07/202	1	
RN	VII URSAP	1416	NATAL	240810	1	01/06/2021		1	6
GO	CENTRAL	1779	GOIANIA	520870	1	13/03/202	31/03/202	1	
RS	004 CRS	1595	SANTA MA	431690	1	29/03/202	04/04/202	1	
PA	01 REGION	1484	BELEM	150140	1	28/03/2021		1	9
SP	GVE XXIX S	1354	SAO JOSE D	354980	1	26/03/202	06/04/202	2	6
SP	GVE I CAPI	1331	SAO PAULO	355030	1	15/04/202	14/05/202	1	6
SP	GVE I CAPI	1331	SAO PAULO	355030	1	30/06/202	01/07/202	1	
SP	GVE XXIX S	1354	SAO JOSE D	354980	1	25/04/202	14/05/202	1	
MA	REGI0NAL	1430	SAO LUIS	211130	1	09/03/202	13/03/202	2	4
ES	METROPOI	1510	SERRA	320500	1	18/05/2021		1	2
CE	11 CRES SC	1521	SOBRAL	231290	1	05/05/202	11/05/202	1	
PR	14RS PARA	1368	PARANAV	411840	1	21/05/202	29/05/202	1	
PE	001	1497	RECIFE	261160	1	10/08/2021		2	6
RJ			RIO DE JAN	330455	1	27/05/202	30/05/202	3	6
SC	ITAJAI	1550	BALNEARIO	420200	1	22/02/202	28/02/2021		
MG	UBERLAND	1462	UBERLAND	317020	1	24/02/2021		1	6
SP	GVE I CAPI	1331	SAO PAULO	355030	1	23/03/202	04/04/202	1	2
SP	GVE I CAPI	1331	SAO PAULO	355030	1	25/03/202	29/03/202	2	3
RO	VI GRS POI	1483	PORTO VEL	110020	1	04/05/2021		3	6
GO	CENTRAL	1779	GOIANIA	520870	1	14/04/202	18/04/202	2	
SP	GVE VIII M	1333	MOGI DAS	353060	1	09/05/2021		3	2
SP	GVE XI AR	1336	ARACATUB	350280	1	07/06/202	09/06/202	1	1
SP	GVE XXIV R	1348	RIBEIRAO P	354340	1	22/05/202	17/06/202	1	
SP	GVE I CAPI	1331	SAO PAULO	355030	1	03/02/202	25/02/202	1	
RS	002 CRS	1607	PORTO ALE	431490	1	08/06/202	06/07/202	1	5
MA	REGI0NAL	1430	SAO LUIS	211130	1	18/06/2021		1	9
SP	GVE XXVII S	1351	SAO JOSE D	354990	1	04/09/2021		3	6
RJ			CAMPOS D	330100	1	05/09/202	15/09/202	3	
RN	II URSAP	1412	MOSSORO	240800	1	02/03/2021		2	6
RS	012 CRS	1601	SANTO AN	431750	1	15/03/202	14/04/202	2	
SP	GVE I CAPI	1331	SAO PAULO	355030	1	25/03/202	26/03/202	1	
PB	I NRS JOAO	1377	JOAO PESS	250750	1	24/03/202	25/03/202	2	6
MG	BELO HORI	1449	BETIM	310670	1	03/06/2021		1	
GO	CENTRAL	1779	GOIANIA	520870	1	20/04/202	14/05/202	1	
TO			PALMAS	172100	1	23/05/202	02/06/202	1	6
RJ			RIO DE JAN	330455	1	16/06/202	22/06/202	2	6
MT	CUIABA	1578	CUIABA	510340	1	06/06/202	08/06/202	2	9
SC	JOINVILLE	1565	JOINVILLE	420910	1	24/05/202	04/06/202	1	2
SP	GVE VIII M	1333	GUARULHO	351880	1	18/06/202	24/06/202	1	
BA	NUCLEO RI	1380	LAURO DE	291920	1	10/06/202	11/06/202	2	
SP	GVE VII SA	1332	SANTO AN	354780	1	24/05/202	25/05/202	2	1
MG	POUSO ALI	1459	ITAJUBA	313240	1	13/07/202	19/07/202	2	6
GO	CENTRAL	1779	GOIANIA	520870	1	22/06/202	25/06/202	2	

SP	GVE I CAPI	1331	SAO PAULO	355030	1	28/07/202	05/08/202	2	
RJ			RIO DE JAN	330455	1	07/10/202	13/10/202	2	6
PB	I NRS JOAO	1377	JOAO PESS	250750	1	15/04/202	18/04/202	2	6
AM	ENTORNO	5584	MANAUS	130260	1	23/01/202	09/02/202	1	2
PA	01 REGION	1484	BELEM	150140	1	16/03/202	17/03/202	2	6
SP	GVE XVI BC	1341	BOTUCATU	350750	1	21/04/2021		1	6
RJ			RIO DE JAN	330455	1	18/04/2021		2	6
SP	GVE I CAPI	1331	SAO PAULO	355030	1	04/04/202	17/04/202	1	
SP	GVE I CAPI	1331	SAO PAULO	355030	1	29/06/202	23/07/202	2	6
PR	02RS METF	1356	CAMPO LA	410420	1	10/05/202	27/05/202	1	6
SE	REGIONAL	2056	ARACAJU	280030	1	29/05/202	09/06/202	1	
PR	10RS CASC	1364	CASCAVEL	410480	1	23/05/202	26/05/202	3	
SP	GVE XVII C	1342	VALINHOS	355620	1	24/06/2021		2	9
CE	11 CRES SC	1521	SOBRAL	231290	1	22/06/202	24/06/202	3	
MA			BALSAS	210140	1	03/07/2021		2	6
SP	GVE XX PIR	1345	LIMEIRA	352690	1	15/07/2021		2	
PR	15RS MARI	1369	MARINGA	411520	1	11/03/202	27/03/202	1	5
SP	GVE XXIX S	1354	SAO JOSE D	354980	1	01/05/202	04/05/202	2	6
RJ			RIO DE JAN	330455	1	16/04/202	20/04/202	3	
SP	GVE XVIII F	1343	ORLANDIA	353430	1	16/05/202	15/06/202	1	
MG	CORONEL F	1468	CARATINGA	311340	1	24/03/2021		3	
MA	REGIONAL	1440	IMPERATRI	210530	1	20/05/2021		1	
MG	DIVINOPOI	1472	DIVINOPOI	312230	1	11/06/202	15/06/202	2	
MG	LEOPOLDIN	1453	LEOPOLDIN	313840	1	25/05/2021		2	
BA	NUCLEO RI	1380	SALVADOR	292740	1	26/07/202	27/07/202	2	9
SP	GVE XII AR	1337	SAO CARLO	354890	1	23/07/202	24/07/202	3	
RJ			NILOPOLIS	330320	1	11/07/2021		2	2
MT	CUIABA	1578	CUIABA	510340	1	03/08/202	23/08/202	1	9
PE	001	1497	RECIFE	261160	1	21/12/2021		1	2
SP	GVE XI ARA	1336	BIRIGUI	350650	1	04/04/202	09/04/202	2	6
RJ			RIO DE JAN	330455	1	17/04/202	20/04/202	3	6
DF			BRASILIA	530010	1	26/04/202	04/05/202	1	6
PR	15RS MARI	1369	MARINGA	411520	1	09/04/202	26/04/202	1	5
GO			RIO VERDE	521880	1	12/04/202	08/05/202	2	
SP	GVE XXIV F	1348	RIBEIRAO F	354340	1	05/05/202	07/05/202	2	5
MG	UBERLAND	1462	UBERLAND	317020	1	15/06/2021		3	6
AP			MACAPA	160030	1	27/04/202	29/05/202	1	6
BA	NUCLEO RI	1380	SALVADOR	292740	1	04/06/202	04/06/202	3	6
RS	004 CRS	1595	SANTA MA	431690	1	16/06/202	21/06/202	2	
MA	REGI0NAL	1430	SAO LUIS	211130	1	22/07/202	26/07/202	1	5
GO	CENTRAL	1779	GOIANIA	520870	1	02/07/202	05/07/202	2	
BA	NUCLEO RI	1381	FEIRA DE S	291080	1	16/06/2021		2	6
SP	GVE I CAPI	1331	SAO PAULO	355030	1	02/03/202	11/03/202	1	
CE	11 CRES SC	1521	SOBRAL	231290	1	25/06/2021		1	
RS	002 CRS	1607	PORTO ALE	431490	1	13/01/202	15/01/202	2	
MT	CUIABA	1578	CUIABA	510340	1	29/06/202	13/07/202	2	
PR	09RS FOZ D	1363	FOZ DO IGU	410830	1	27/12/202	03/01/202	2	6

RS	013 CRS	1609	SANTA CRU	431680	1	23/02/202	24/02/202	2	3
PR	02RS METF	1356	CURITIBA	410690	1	09/03/202	31/03/202	1	6
BA	NUCLEO RI	1380	SALVADOR	292740	1	31/03/202	01/04/202	2	
PR	04RS IRATI	1358	IRATI	411070	1	16/02/202	18/02/202	2	
RJ			RIO DE JAN	330455	1	14/04/202	28/04/202	1	
SP	GVE VIII M	1333	GUARULHO	351880	1			9	9
RJ			RIO DE JAN	330455	1	27/04/2021		1	9
SP	GVE XIX M/	1344	TUPA	355500	1	27/05/202	30/05/202	3	6
MT	CUIABA	1578	CUIABA	510340	1	11/06/202	14/06/202	2	
BA	NUCLEO RI	1380	SALVADOR	292740	1	17/05/202	22/05/202	1	1
SP	GVE I CAPI	1331	SAO PAULO	355030	1	16/07/202	01/08/202	2	9
ES	METROPOI	1510	SERRA	320500	1	18/11/2021		2	2
MS	EX ARS DE	1975	CAMPO GF	500270	1	03/04/202	18/04/202	1	4
RJ			NITEROI	330330	1	23/02/2021		2	9
MG	POUSO ALI	1459	POUSO ALI	315250	1	30/03/202	23/05/202	1	6
SP	GVE XVI BO	1341	BOTUCATU	350750	1	04/04/2021		1	4
BA	NUCLEO RI	1381	FEIRA DE S.	291080	1	17/03/2021		2	6
RS	005 CRS	1608	CAXIAS DO	430510	1	26/04/202	30/04/202	2	
MT	CUIABA	1578	CUIABA	510340	1	12/03/202	29/03/202	2	
SP	GVE XVII C/	1342	CAMPINAS	350950	1	06/04/202	12/05/202	2	
SP	GVE I CAPI	1331	SAO PAULO	355030	1	18/05/202	03/06/202	1	
PR	17RS LOND	1371	LONDRINA	411370	1	28/06/202	06/07/202	2	5
SP	GVE X OSA	1335	ITAPEVI	352250	1	23/05/2021		2	
DF			BRASILIA	530010	1	07/08/202	04/09/202	1	6
MG	CORONEL I	1468	IPATINGA	313130	1	21/06/2021		2	6
SP	GVE XVII C/	1342	CAMPINAS	350950	1	26/06/202	06/07/202	2	
SP	GVE I CAPI	1331	SAO PAULO	355030	1	16/07/2021			
MT	CUIABA	1578	CUIABA	510340	1	28/07/202	13/08/202	1	
MG	UBA	1464	MURIAE	314390	1	25/07/2021		2	6
PR	10RS CASC	1364	CASCAVEL	410480	1	05/08/202	09/08/202	3	
PR	17RS LOND	1371	LONDRINA	411370	1	31/07/202	06/09/202	1	6
SP	GVE VII SAI	1332	SAO CAETA	354880	1	07/11/2021		2	
SP			SAO JOSE D	354990	1	20/01/2021		3	
SP	GVE XXI PR	1346	PRESIDENT	354140	1	02/04/202	20/04/202	1	2
SC	VIDEIRA	1546	CURITIBAN	420480	1	12/03/202	15/03/202	3	
PA	01 REGION	1484	BELEM	150140	1	08/03/202	26/03/202	1	6
GO	CENTRAL	1779	GOIANIA	520870	1	18/04/202	20/04/202	2	
SP			SAO PAULO	355030	1	03/05/202	13/05/202	2	
BA	NUCLEO RI	1380	SALVADOR	292740	1	05/05/202	11/05/202	2	
MA	REGI0NAL	1430	SAO LUIS	211130	1	10/02/202	19/02/202	2	5
SP	GVE X OSA	1335	OSASCO	353440	1	25/05/202	02/06/202	2	5
BA	NUCLEO RI	1403	BARREIRAS	290320	1	20/05/202	22/05/202	2	
MS	EX ARS DE	1975	CAMPO GF	500270	1			1	
RJ			RIO DE JAN	330455	1	07/05/2021		2	9
SP	GVE VII SAI	1332	MAUA	352940	1	20/04/202	22/04/202	2	2
SP			SAO PAULO	355030	1	26/04/202	09/06/202	1	
MG	JUIZ DE FO	1452	JUIZ DE FO	313670	1	21/05/2021		2	6

RJ			RIO DE JAN	330455	1	19/06/202	26/06/202	2	9
RN	II URSAP	1412	MOSSORO	240800	1	14/06/202	24/06/202	2	2
BA	NUCLEO RI	1380	SALVADOR	292740	1	08/07/202	09/07/202	2	9
SP	GVE XXI PR	1346	PRESIDENT	354140	1	23/08/202	01/09/202	1	6
SP	GVE XXIX S	1354	VOTUPORA	355710	1	03/06/202	15/06/202	1	
GO	CENTRAL	1779	GOIANIA	520870	1	26/07/202	31/08/202	1	3
TO			PALMAS	172100	1	03/06/202	24/06/202	1	
SP	GVE VII SA	1332	SAO BERN	354870	1	08/06/2021		2	9
GO	CENTRAL	1779	GOIANIA	520870	1	28/07/202	19/08/202	1	5
MG	UBERLAND	1462	UBERLAND	317020	1	06/03/2021		2	6
RS	008 CRS	1597	CACHOEIR	430300	1	03/03/202	04/03/202	3	6
SP	GVE I CAPI	1331	SAO PAUL	355030	1	24/02/202	25/02/202	3	6
RS	006 CRS	1611	PASSO FUN	431410	1	18/03/202	05/05/202	1	3
SP	GVE I CAPI	1331	SAO PAUL	355030	1	13/03/202	30/03/202	2	
SP	GVE IX FRA	1334	FRANCISC	351630	1	15/04/2021		2	
PR	11RS CAMI	1365	CAMPO M	410430	1	12/03/202	22/03/202	1	6
RS	006 CRS	1611	ESPUMOS	430750	1	11/05/202	19/05/202	2	
GO	CENTRAL	1779	GOIANIA	520870	1	25/05/202	12/06/202	2	
SP	GVE I CAPI	1331	SAO PAUL	355030	1	06/06/202	22/06/202	1	2
SP	GVE XXVII	1351	SAO JOSE D	354990	1	29/07/2021		3	6
MG	PATOS DE	1456	PATOS DE	314800	1	30/06/202	05/07/202	2	
SP			ITAPIRA	352260	1	18/07/202	20/08/202	2	6
MT	CUIABA	1578	CUIABA	510340	1	13/07/202	15/07/202	9	9
GO	CENTRAL	1779	GOIANIA	520870	1	05/03/202	08/03/202	2	
RS	002 CRS	1607	PORTO ALE	431490	1	30/03/202	25/04/202	1	
RJ			RIO DE JAN	330455	1	31/03/2021		1	9
SP	GVE XII AR	1337	SAO CARL	354890	1	29/03/202	05/04/202	2	6
GO	CENTRAL	1779	GOIANIA	520870	1	03/03/202	24/03/202	1	
DF			BRASILIA	530010	1	02/05/202	16/05/202	1	5
RO	VI GRS POI	1483	PORTO VEL	110020	1	20/04/202	25/04/202	2	6
MT	CUIABA	1578	CUIABA	510340	1	08/05/202	26/05/202	1	
AL	1 MICRORI	1533	MACEIO	270430	1	08/04/2021			
CE	1 CRES FOI	1519	FORTALEZ	230440	1	26/02/202	03/03/202	3	2
PA	01 REGION	1484	BELEM	150140	1	06/06/202	14/06/202	1	5
SP	GVE XVII C	1342	JUNDIAI	352590	1	25/06/2021		2	2
DF			BRASILIA	530010	1	03/07/202	16/07/202	1	6
RJ			RIO DE JAN	330455	1	14/04/2021		2	1
GO	CENTRAL	1779	GOIANIA	520870	1	12/06/202	17/06/202	2	
GO	CENTRAL	1779	GOIANIA	520870	1	26/05/202	18/06/202	1	
RR			BOA VISTA	140010	1	26/03/202	31/03/202	1	
RJ			CAMPOS D	330100	1	15/08/202	24/08/202	1	
CE	1 CRES FOI	1519	FORTALEZ	230440	1	12/05/202	22/05/202	3	1
GO	CENTRAL	1779	GOIANIA	520870	1	11/01/202	14/01/202	3	
MG	VARGINHA	1466	VARGINHA	317070	1	21/07/202	26/07/202	3	6
RS	002 CRS	1607	PORTO ALE	431490	1	02/09/202	05/09/202	2	2
AC	REGIONAL	1941	CRUZEIRO	120020	1	01/01/2022			
SP	GVE I CAPI	1331	SAO PAUL	355030	1	02/04/202	26/04/202	1	9

CE	11 CRES SC	1521	SOBRAL	231290	1	06/04/2021		2	
AC	REGIONAL	1938	RIO BRANC	120040	1	12/04/202	12/04/202	1	2
RS	018 CRS	1605	TRAMAND.	432160	1	10/05/202	10/05/202	2	
SP	GVE XVII C.	1342	CAMPINAS	350950	1	03/05/2021		2	
SP	GVE I CAPI	1331	SAO PAUL(	355030	1	24/05/202	28/05/202	2	2
RJ			GUAPIMIR	330185	1	05/06/2021		2	
RJ			CAMPOS D	330100	1	05/06/2021		2	5
RJ			RIO DE JAN	330455	1	29/04/2021		2	4
GO	CENTRAL	1779	GOIANIA	520870	1	22/06/202	05/08/202	1	
SP	GVE XXXI S	1353	SOROCABA	355220	1	16/06/202	29/06/202	1	9
SP			OSASCO	353440	1	30/05/2021		2	6
ES	METROPOI	1510	SERRA	320500	1	26/09/202	03/10/202	2	2
RJ			RIO DE JAN	330455	1	21/08/2021		2	
MT	CUIABA	1578	CUIABA	510340	1	21/06/202	22/07/202	1	9
PE	001	1497	RECIFE	261160	1	23/06/2021			
SP	GVE IX FRA	1334	FRANCISC(	351630	1	25/02/2021		3	
SC	JOINVILLE	1565	JOINVILLE	420910	1	05/02/202	13/02/202	1	5
SP	GVE VII SAI	1332	SANTO AN	354780	1	05/03/2021		2	9
RS	001 CRS	1592	NOVO HAN	431340	1	22/03/202	14/04/202	2	6
PR	17RS LONE	1371	LONDRINA	411370	1	12/04/202	12/05/202	1	5
MS	EX ARS DE	1975	CAMPO GF	500270	1	06/05/2021		1	5
MT	SINOP	1584	SORRISO	510792	1	07/05/202	22/05/202	1	6
MS	NRS DE DO	1966	DOURADO:	500370	1	26/05/2021			
SP	GVE XVII C.	1342	CAMPINAS	350950	1	23/05/202	24/05/202	2	
CE	1 CRES FOI	1519	FORTALEZ/	230440	1	06/05/202	18/05/202	3	2
PA	11 REGION	1494	MARABA	150420	1	20/07/2021		1	2
PR	15RS MARI	1369	SARANDI	412625	1	10/06/202	26/06/202	1	
SP	GVE I CAPI	1331	SAO PAUL(	355030	1	17/07/202	20/07/2021		
MT	CUIABA	1578	CUIABA	510340	1	29/07/202	30/08/202	1	9
SP			SAO PAUL(	355030	1	11/10/202	29/10/202	2	
PR	10RS CASC	1364	CASCAVEL	410480	1	09/08/202	17/08/202	2	
RJ			RIO DE JAN	330455	1	02/02/202	27/02/202	1	
PR	02RS METF	1356	CURITIBA	410690	1	21/04/202	23/04/202	3	6
SP	GVE I CAPI	1331	SAO PAUL(	355030	1	13/04/202	15/04/202	2	2
MT	CUIABA	1578	CUIABA	510340	1	24/04/202	05/05/202	1	
PR	10RS CASC	1364	CASCAVEL	410480	1	04/06/202	09/06/202	2	
BA	NUCLEO RI	1380	CAMACARI	290570	1	08/04/202	20/04/202	1	9
PR	02RS METF	1356	CAMPINA (	410400	1	04/08/202	05/08/202	2	3
MT	CUIABA	1578	CUIABA	510340	1	03/08/202	09/09/202	2	
CE	1 CRES FOI	1519	FORTALEZ/	230440	1	22/04/202	26/04/202	2	
MG	BELO HORI	1449	BELO HORI	310620	1	23/06/2021			
RJ			RIO DE JAN	330455	1	19/07/202	08/08/202	1	6
MG	POUSO ALI	1459	ITAJUBA	313240	1	20/07/202	26/07/202	1	6
SP	GVE I CAPI	1331	SAO PAUL(	355030	1	07/02/202	14/02/202	1	6
GO	CENTRAL	1779	GOIANIA	520870	1	02/03/202	06/03/202	2	
SC	JOINVILLE	1565	JOINVILLE	420910	1	25/02/202	05/03/202	1	2
SP			SAO PAUL(	355030	1	26/03/2021		2	

MG	GOVERNAI	1471	MANTENA	313960	1	02/04/2021		2	
RJ			MIGUEL PE	330290	1	22/03/202	28/03/202	1	2
SP	GVE XXIX S	1354	SAO JOSE D	354980	1	09/06/202	12/06/202	2	4
PR	10RS CASC	1364	CASCAVEL	410480	1	23/06/202	27/06/202	3	
PR	02RS METF	1356	CAMPINA (	410400	1	21/05/202	29/05/202	1	6
PA	10 REGION	1493	PORTO DE	150590	1	25/06/2021		1	6
SP	GVE XXV S/	1349	SANTOS	354850	1	31/03/202	12/04/202	9	9
RN	VII URSAP	1416	NATAL	240810	1	25/06/202	03/07/202	1	3
SP	GVE XXIV F	1348	RIBEIRAO F	354340	1	14/07/202	12/08/202	1	
MA	REGIONAL	1440	IMPERATRI	210530	1	16/07/2021		2	6
SP	GVE XXIX S	1354	SAO JOSE D	354980	1	05/03/202	17/03/202	1	4
RR			BOA VISTA	140010	1	23/07/202	26/07/202	2	
RJ			RIO DE JAN	330455	1	05/05/2021		2	
RS	005 CRS	1608	CAXIAS DO	430510	1	30/04/202	03/05/202	2	6
DF			BRASILIA	530010	1	23/03/202	20/04/202	1	6
PR	15RS MARI	1369	MARINGA	411520	1	16/05/202	22/05/202	1	6
RS	007 CRS	1596	BAGE	430160	1	03/06/202	11/06/202	1	6
SP	GVE I CAPI	1331	SAO PAULC	355030	1	23/05/202	22/06/202	1	2
CE			TIANGUA	231340	1	28/05/202	13/06/202	1	
MT	CUIABA	1578	CUIABA	510340	1	07/09/202	11/09/202	2	
RN	II URSAP	1412	MOSSORO	240800	1	13/07/202	14/07/2021		
RS	004 CRS	1595	SANTA MA	431690	1	24/06/202	28/06/202	2	
SP	GVE XVII C/	1342	CAMPINAS	350950	1	08/07/202	08/07/202	2	2
CE	1 CRES FOF	1519	FORTALEZ/	230440	1	04/06/202	18/06/202	1	1
SP	GVE XVII C/	1342	JUNDIAI	352590	1	17/03/2021		1	
RS	002 CRS	1607	PORTO ALE	431490	1	24/03/202	26/03/202	2	4
SP	GVE XVII C/	1342	CAMPINAS	350950	1	11/03/2021		1	6
PA	12 REGION	1495	REDENCAC	150613	1	26/02/2021			
SP	GVE XVI BC	1341	SAO MANU	355010	1	28/03/202	31/03/202	2	2
MG	BELO HORI	1449	CONTAGEN	311860	1	26/04/202	15/05/202	1	2
SP	GVE I CAPI	1331	SAO PAULC	355030	1	02/06/202	11/06/202	1	9
SP	GVE XXIX S	1354	SAO JOSE D	354980	1	04/04/202	10/04/202	2	6
BA	NUCLEO RI	1381	FEIRA DE S.	291080	1	07/05/202	14/05/202	1	6
MG	UBA	1464	VISCONDE	317200	1		02/06/202	2	
SP	GVE XXI PR	1346	PRESIDENT	354140	1	11/04/202	24/04/202	1	6
TO			PALMAS	172100	1	17/04/202	01/05/202	1	6
ES	METROPOI	1510	SERRA	320500	1	20/05/2021		1	2
RJ			RIO DE JAN	330455	1	05/06/202	07/06/202	2	6
ES	METROPOI	1510	SERRA	320500	1	09/05/2021		2	2
RJ			RIO DE JAN	330455	1	21/07/2021		2	6
RN	VII URSAP	1416	NATAL	240810	1	15/05/2021		1	6
MG	PASSOS	1455	PASSOS	314790	1	03/06/2021		1	
PR	17RS LOND	1371	LONDRINA	411370	1	12/01/202	04/02/202	1	4
RR			BOA VISTA	140010	1	16/08/202	18/08/202	2	
SP	GVE XXIX S	1354	SAO JOSE D	354980	1	20/03/202	21/03/202	2	
MG	BELO HORI	1449	CONTAGEN	311860	1	24/03/202	02/04/202	2	
SP	GVE XXXI S	1353	SOROCABA	355220	1	19/03/202	29/03/202	1	

GO	CENTRAL	1779	GOIANIA	520870	1	04/05/2021		2	
RJ			RIO DE JAN	330455	1	24/05/202	23/06/202	1	6
PE	001	1497	RECIFE	261160	1	15/05/2021		2	6
PR	10RS CASC	1364	CASCAVEL	410480	1	15/06/202	18/06/202	3	
PA	11 REGION	1494	MARABA	150420	1	20/05/202	25/05/202	1	2
CE	21 CRES JU	1531	JUAZEIRO I	230730	1	14/05/2021		2	9
SP	GVE VIII M	1333	MOGI DAS	353060	1	18/05/2021		2	6
SP	GVE VIII M	1333	GUARULHC	351880	1	10/07/202	19/07/202	2	2
SC	CHAPECO	1553	CHAPECO	420420	1	08/09/2021			5
SP	GVE I CAPI	1331	SAO PAULC	355030	1	08/08/2021		2	
SP	GVE I CAPI	1331	SAO PAULC	355030	1	23/06/202	30/06/202	1	2
RJ			RIO DE JAN	330455	1	04/10/202	07/10/202	2	6
BA	NUCLEO RI	1380	SALVADOR	292740	1	14/06/202	21/06/202	2	
CE	1 CRES FOF	1519	FORTALEZA	230440	1	19/04/202	04/05/202	3	1
SP	GVE XXI PR	1346	PRESIDENT	354140	1	27/09/202	13/10/202	2	6
RS	002 CRS	1607	PORTO ALE	431490	1	13/07/202	26/07/202	1	4
RJ			RIO DAS OS	330452	1	11/03/2021		1	
SP	GVE IX FRA	1334	FRANCISCC	351630	1	14/04/202	14/05/202	2	3
RO	VI GRS POF	1483	PORTO VEL	110020	1	21/02/202	26/02/202	1	9
RN	VII URSAP	1416	NATAL	240810	1	16/03/202	18/03/202	2	5
GO	CENTRAL	1779	GOIANIA	520870	1	16/04/202	27/04/202	2	
PA	01 REGION	1484	BELEM	150140	1	30/05/202	05/06/202	2	3
MT	CUIABA	1578	CUIABA	510340	1	26/04/202	24/05/202	1	6
PR	17RS LOND	1371	LONDRINA	411370	1	04/06/202	05/06/202	2	5
PB	I NRS JOAC	1377	JOAO PESS	250750	1	19/06/202	23/06/202	3	6
SP	GVE I CAPI	1331	SAO PAULC	355030	1	28/10/202	12/11/202	1	4
SP	GVE I CAPI	1331	SAO PAULC	355030	1	20/02/2021		3	
SP	GVE I CAPI	1331	SAO PAULC	355030	1	21/03/2021		1	
RR			BOA VISTA	140010	1			1	
AC	REGIONAL	1938	RIO BRANC	120040	1	19/01/2021			
MT	CUIABA	1578	CUIABA	510340	1	03/03/202	20/04/202	1	
SC	JOINVILLE	1565	JOINVILLE	420910	1	15/02/202	17/02/202	2	2
GO	CENTRO SU	1781	APARECIDA	520140	1	22/04/202	25/04/202	2	6
SP			SAO PAULC	355030	1	01/04/202	09/04/202	1	6
SP	GVE XVII C	1342	JUNDIAI	352590	1	21/03/2021		3	2
SP	GVE XXVIII	1576	ILHABELA	352040	1	16/04/2021		2	6
MG	SETE LAGO	1467	SETE LAGO	316720	1	11/05/202	14/05/202	2	9
GO	CENTRAL	1779	GOIANIA	520870	1	22/06/202	05/07/202	1	
PB					1	26/06/2021		2	
RJ			RIO DE JAN	330455	1	05/08/202	09/08/202	2	9
SP	GVE VIII M	1333	GUARULHC	351880	1	04/08/202	09/08/202	1	1
PR	20RS TOLEI	1374	MARECHAI	411460	1	13/04/202	16/04/202	3	
SP	GVE XVII C	1342	CAMPINAS	350950	1	19/03/202	25/03/202	2	9
TO			PORTO NA	171820	1	06/04/202	12/04/202	1	6
RO	IV GRS ARI	1481	CUJUBIM	110094	1	19/05/202	17/06/202	1	6
PA	01 REGION	1484	BELEM	150140	1	02/04/2021		2	6
PR	15RS MARI	1369	SARANDI	412625	1	20/03/202	28/03/202	1	5

SP	GVE I CAPI	1331	SAO PAULO	355030	1	18/03/202	27/03/202	1	2
SP	GVE VIII M	1333	GUARULHO	351880	1	06/05/2021		2	
RO	VI GRS POR	1483	PORTO VEL	110020	1	27/03/202	04/04/202	1	6
SP			SAO PAULO	355030	1	22/06/202	28/06/202	1	
PI	4 GRS TERE	1883	TERESINA	221100	1	12/08/2021		1	2
RJ			RIO DE JAN	330455	1	03/07/202	17/08/202	1	6
PR	17RS LOND	1371	LONDRINA	411370	1	22/07/202	21/09/202	2	5
RS			PORTO ALE	431490	1	19/07/202	20/07/2021		6
SC	FLORIANOI	1476	FLORIANOI	420540	1	11/11/202	13/11/202	1	
SP	GVE I CAPI	1331	SAO PAULO	355030	1	13/03/202	20/03/202	1	
AM	ENTORNO	5584	MANAUS	130260	1	23/02/2021		1	
SP	GVE VII SAI	1332	SAO CAETA	354880	1	10/03/2021		1	
GO	CENTRAL	1779	GOIANIA	520870	1	16/03/202	21/03/202	1	
GO	CENTRAL	1779	GOIANIA	520870	1	03/04/202	26/04/2021		
RJ			RIO DE JAN	330455	1	25/03/2021		1	
SP	GVE I CAPI	1331	SAO PAULO	355030	1	13/03/202	23/03/202	1	
SP	GVE XXIV R	1348	RIBEIRAO P	354340	1	04/05/202	07/05/202	2	6
GO	SUDOESTE	1799	RIO VERDE	521880	1	12/03/202	16/03/202	1	
CE	1 CRES FOR	1519	FORTALEZA	230440	1	01/04/202	02/04/202	2	
PR	10RS CASC	1364	CASCAVEL	410480	1	21/05/202	24/05/202	3	
CE	11 CRES SC	1521	SOBRAL	231290	1	13/04/2021		2	6
MA	REGIONAL	1440	IMPERATRI	210530	1	24/06/2021		2	
MG	JUIZ DE FO	1452	JUIZ DE FO	313670	1	15/06/202	20/06/202	2	9
RJ			RIO DE JAN	330455	1	20/08/2021		2	
PR	15RS MARI	1369	MARINGA	411520	1	16/03/202	10/04/202	1	3
SC	CHAPECO	1553	CHAPECO	420420	1	22/02/2021		3	
ES	METROPOI	1510	SERRA	320500	1	11/04/2021		2	4
SP	GVE XXXI S	1353	ITU	352390	1	29/04/2021		2	
PR	01RS PARA	1355	PARANAGU	411820	1	21/04/202	27/04/202	2	6
TO			PALMAS	172100	1	25/04/202	27/04/202	1	6
MG	PATOS DE I	1456	PATOS DE I	314800	1	07/06/202	17/06/202	2	
RS	002 CRS	1607	PORTO ALE	431490	1	25/06/202	28/06/202	2	
SP	GVE XVII C	1342	VALINHOS	355620	1	03/07/2021		3	6
SC			RIO DO SUI	421480	1	09/12/2021			
ES	METROPOI	1510	SERRA	320500	1	05/03/2021		2	1
SP	GVE XVII C	1342	CAMPINAS	350950	1	04/03/202	14/03/202	2	6
SP	GVE XVII C	1342	CAMPINAS	350950	1	27/03/202	31/03/202	2	
SP	GVE I CAPI	1331	SAO PAULO	355030	1	07/04/202	09/04/202	2	6
GO	PIRINEUS	1792	ANAPOLIS	520110	1	07/03/202	24/03/202	1	6
MS	EX ARS DE	1975	CAMPO GR	500270	1	14/04/202	29/04/202	1	2
RS	018 CRS	1605	TRAMAND	432160	1	03/05/2021		3	
MT	SINOP	1584	SORRISO	510792	1	22/04/2021		1	6
CE					1	29/04/2021		1	6
RJ			VALENCA	330610	1	17/05/2021		2	2
PR	02RS METR	1356	CAMPINA (	410400	1	23/04/202	27/04/202	2	
SP	GVE I CAPI	1331	SAO PAULO	355030	1	11/06/202	15/07/202	2	
SP	GVE XXVI S	1350	MOGI GUA	353070	1	14/08/202	21/08/202	3	9

MA	REGI0NAL	1430	SAO LUIS	211130	1	10/06/202	19/06/202	1	9
RJ			RIO DE JAN	330455	1	21/04/202	04/05/202	1	
SP	GVE XXVI S	1350	MOGI GUA	353070	1	24/05/202	14/06/202	1	9
PR	10RS CASC	1364	CASCAVEL	410480	1	06/03/202	17/03/202	2	6
SC			SAO JOSE	421660	1	22/02/202	31/03/202	1	6
SP	GVE I CAPI	1331	SAO PAULO	355030	1	03/03/202	04/03/202	2	
RJ			NITEROI	330330	1	03/04/2021		9	9
PR	03RS PONT	1357	PONTA GR	411990	1	09/03/2021		1	
MT	CUIABA	1578	CUIABA	510340	1	21/06/202	16/07/202	1	
RS	001 CRS	1592	CANOAS	430460	1	27/01/202	28/01/202	3	2
SC	MAFRA	1566	MAFRA	421010	1	06/05/202	31/05/202	1	
PE			RECIFE	261160	1	28/05/2021		2	
RN	VII URSAP	1416	NATAL	240810	1	25/03/202	04/04/202	2	9
TO			PALMAS	172100	1	08/12/2021		2	
AP			MACAPA	160030	1	08/07/202	24/07/202	1	6
GO	CENTRAL	1779	GOIANIA	520870	1	10/10/202	15/10/202	2	
SP	GVE X OSA	1335	OSASCO	353440	1	02/08/2021		2	6
SP	GVE I CAPI	1331	SAO PAULO	355030	1	30/08/2021		2	6
GO	CENTRAL	1779	GOIANIA	520870	1	09/02/202	16/02/202	1	
SP	GVE XXXI S	1353	SOROCABA	355220	1	17/02/202	24/02/202	1	
SP	GVE XXVII S	1351	JACAREI	352440	1	22/04/202	03/05/202	1	2
BA	NUCLEO RE	1394	IRECE	291460	1	31/03/2021		1	
DF			BRASILIA	530010	1	17/04/202	15/05/202	2	6
SP	GVE XXV SA	1349	SANTOS	354850	1	01/05/2021		2	9
GO	CENTRAL	1779	GOIANIA	520870	1	08/06/2021		1	
BA	NUCLEO RE	1398	VITORIA DA	293330	1	06/06/2021		2	6
RJ			RIO DE JAN	330455	1	21/06/202	15/07/202	1	6
SP	GVE I CAPI	1331	SAO PAULO	355030	1	30/05/202	04/06/202	1	
PR	10RS CASC	1364	CASCAVEL	410480	1	11/07/202	01/08/202	2	
DF			BRASILIA	530010	1	02/01/202	06/01/202	3	6
SP	GVE VIII M	1333	GUARULHO	351880	1	18/01/2021		2	
SP	GVE XXVII S	1351	SAO JOSE D	354990	1	23/12/2021		2	
SC	LAGES	1571	LAGES	420930	1			1	
RS	018 CRS	1605	TRAMAND	432160	1	15/05/202	17/05/202	2	
PR	17RS LOND	1371	LONDRINA	411370	1	22/02/202	02/03/2021		6
SP	GVE I CAPI	1331	SAO PAULO	355030	1	01/06/202	07/06/202	2	5
RS	018 CRS	1605	TRAMAND	432160	1	31/05/202	30/06/202	2	
PR	05RS GUAR	1359	GUARAPUA	410940	1	05/06/202	25/06/202	1	6
PR	10RS CASC	1364	CASCAVEL	410480	1	30/09/202	07/10/202	3	
ES	METROPOL	1510	SERRA	320500	1	14/03/2021		1	2
SP	GVE X OSA	1335	OSASCO	353440	1	21/03/2021		2	6
PA	01 REGION	1484	BELEM	150140	1	05/04/202	09/04/202	3	6
SC	JOINVILLE	1565	JOINVILLE	420910	1	09/06/202	18/06/202	1	6
PR	03RS PONT	1357	PONTA GR	411990	1	31/05/2021		1	6
SP	GVE I CAPI	1331	SAO PAULO	355030	1	04/06/202	06/06/202	1	6
AM	ENTORNO	5584	MANAUS	130260	1	07/07/202	02/08/202	2	6
PR	15RS MARI	1369	SARANDI	412625	1	15/06/202	11/07/202	1	

RJ			BOM JESUS	330060	1	10/07/202	23/07/202	3	
GO	CENTRAL	1779	GOIANIA	520870	1	21/08/202	24/08/202	2	
BA	NUCLEO RE	1380	SALVADOR	292740	1	06/12/202	07/12/202	3	
SC			FLORIANOI	420540	1	06/11/202	18/11/202	1	
SP	GVE XXII PI	1573	DRACENA	351440	1	24/08/2021		3	6
CE	1 CRES FOF	1519	FORTALEZA	230440	1			2	
SC	JOINVILLE	1565	JOINVILLE	420910	1	20/02/202	03/03/202	1	2
PR	17RS LOND	1371	LONDRINA	411370	1	25/04/202	28/04/202	2	6
SP	GVE I CAPI	1331	SAO PAULO	355030	1	02/05/202	06/05/202	2	2
SP	GVE I CAPI	1331	SAO PAULO	355030	1	05/06/202	15/06/202	2	
RS	002 CRS	1607	PORTO ALE	431490	1	10/04/202	20/05/202	1	6
SP	GVE XXIX S	1354	SAO JOSE D	354980	1	29/07/202	20/08/202	1	6
RJ			CAMPOS D	330100	1	15/05/2021		1	
DF			BRASILIA	530010	1	08/09/202	30/09/202	1	6
PA	01 REGION	1484	BELEM	150140	1	23/03/2021		1	
AL	1 MICRORE	1533	MACEIO	270430	1	09/06/2021		1	
RS	005 CRS	1608	CAXIAS DO	430510	1	10/02/202	21/02/202	1	6
AM	ENTORNO	5584	MANAUS	130260	1	09/02/202	16/02/202	1	
SP	GVE XIII AS	1338	ASSIS	350400	1	20/03/202	19/04/202	1	6
BA	NUCLEO RE	1380	SALVADOR	292740	1	26/03/202	27/03/202	2	
GO	CENTRAL	1779	GOIANIA	520870	1	22/04/202	25/04/202	2	
MT	CUIABA	1578	CUIABA	510340	1	08/04/202	11/04/202	2	9
PR	03RS PONT	1357	JAGUARIAI	411200	1	30/03/202	04/04/202	2	5
SP	GVE XVII CA	1342	JUNDIAI	352590	1	26/04/2021		1	6
BA	NUCLEO RE	1380	SALVADOR	292740	1	18/05/202	20/05/202	3	
RJ			RIO DE JAN	330455	1	14/03/2021		9	
SP	GVE XVII CA	1342	CAMPINAS	350950	1	15/03/202	27/03/202	1	
SP	GVE I CAPI	1331	SAO PAULO	355030	1	28/06/202	03/07/202	2	
SP	GVE XVII CA	1342	CAMPINAS	350950	1	20/06/202	02/07/202	2	2
MG	PATOS DE I	1456	PATOS DE I	314800	1	13/06/2021		2	2
BA			SALVADOR	292740	1	06/05/202	09/05/202	1	
DF			BRASILIA	530010	1	05/05/202	07/05/202	3	9
MG	SETE LAGO	1467	SETE LAGO	316720	1	01/04/202	02/04/202	2	4
RJ			RIO DE JAN	330455	1			1	6
RJ			RIO DE JAN	330455	1	27/03/2021		1	6
SP	GVE VIII M	1333	MOGI DAS	353060	1	16/04/2021		2	2
SP	GVE I CAPI	1331	SAO PAULO	355030	1	13/02/202	20/02/202	2	2
SP	GVE XXIX S	1354	SAO JOSE D	354980	1	05/04/202	06/04/202	2	4
SP	GVE XXVIII	1576	SAO SEBAS	355070	1	30/04/202	01/05/202	2	9
MA	REGIONAL	1440	IMPERATRI	210530	1	22/04/202	25/04/202	3	6
RJ			RIO DE JAN	330455	1	30/08/2021			
RJ			CAMPOS D	330100	1	11/07/202	13/07/202	2	6
SP	GVE I CAPI	1331	SAO PAULO	355030	1	17/08/202	19/08/202	2	6
RN	II URSAP	1412	APODI	240100	1	06/05/202	12/05/202	1	6
SP	GVE XVII CA	1342	CAMPINAS	350950	1	15/11/202	30/11/202	2	6
PA	09 REGION	1492	SANTAREM	150680	1	29/11/2021		2	6
RJ			RIO DE JAN	330455	1	28/03/202	31/03/202	3	

RJ			RIO DE JAN	330455	1	25/03/2021		1	9
SP	GVE I CAPI	1331	SAO PAUL(	355030	1	25/02/202	03/03/202	2	3
SP	GVE I CAPI	1331	SAO PAUL(	355030	1	10/05/202	21/05/202	1	6
SP	GVE XXV S/	1349	ITANHAEM	352210	1	06/04/2021		9	
ES	METROPOI	1510	SERRA	320500	1	28/04/2021		2	4
SP	GVE I CAPI	1331	SAO PAUL(	355030	1	06/08/202	06/08/202	2	6
PE	001	1497	RECIFE	261160	1	09/05/2021		1	
RJ			RIO DE JAN	330455	1	26/05/202	01/06/202	2	6
SP	GVE I CAPI	1331	SAO PAUL(	355030	1	07/10/202	14/10/202	2	
GO	CENTRAL	1779	GOIANIA	520870	1	02/08/202	05/08/202	2	
DF			BRASILIA	530010	1	18/10/202	27/10/202	2	9
SP	GVE XXXI S	1353	ITU	352390	1	14/06/2021		2	
SP	GVE I CAPI	1331	SAO PAUL(	355030	1	16/08/202	23/08/202	1	
SP	GVE XVII C/	1342	CAMPINAS	350950	1	16/03/2021		2	6
MG	UBERLAND	1462	UBERLAND	317020	1			3	
GO	CENTRAL	1779	GOIANIA	520870	1	16/04/202	17/04/202	3	
SP	GVE XXIII R	1347	REGISTRO	354260	1	20/03/202	24/03/202	3	2
MG	DIVINOPOI	1472	PITANGUI	315140	1	28/05/2021		2	2
SP	GVE I CAPI	1331	SAO PAUL(	355030	1	30/06/202	02/07/2021		
GO	CENTRAL	1779	GOIANIA	520870	1	11/01/2021		3	
SC	JARAGUA I	1567	JARAGUA I	420890	1	11/02/202	22/02/202	2	6
SC	BLUMENAU	1564	BRUSQUE	420290	1	29/03/2021		1	
AC	REGIONAL	1938	RIO BRANC	120040	1	19/03/202	21/03/202	1	2
SP	GVE XVII C/	1342	CAMPINAS	350950	1	26/03/202	30/03/202	2	
RS	002 CRS	1607	PORTO ALE	431490	1	27/03/202	16/06/202	1	2
RJ			CONCEICA(	330140	1	28/03/2021		1	
SP	GVE I CAPI	1331	SAO PAUL(	355030	1	21/03/202	04/04/202	1	2
SP	GVE I CAPI	1331	SAO PAUL(	355030	1	05/05/202	25/05/202	1	2
SP	GVE XIX M/	1344	MARILIA	352900	1	12/04/2021		1	6
GO	SUDOESTE	1800	JATAI	521190	1	12/06/202	15/06/202	2	5
BA	NUCLEO RI	1380	SALVADOR	292740	1	24/05/202	27/05/202	2	
SP	GVE XXIX S	1354	VOTUPOR/	355710	1	18/06/202	21/06/202	1	6
GO	PIRINEUS	1792	ANAPOLIS	520110	1	21/06/2021		2	6
PA	01 REGION	1484	BELEM	150140	1	10/06/2021		1	4
MG	DIVINOPOI	1472	BOM DESP.	310740	1	03/07/2021		2	
SC			CHAPECO	420420	1	27/02/2021		2	6
SP	GVE XXIV R	1348	RIBEIRAO I	354340	1	20/04/202	24/04/202	2	2
SP	GVE I CAPI	1331	SAO PAUL(	355030	1	12/04/202	16/04/202	2	2
SP	GVE X OSA	1335	OSASCO	353440	1	12/05/202	17/05/202	3	2
SP	GVE X OSA	1335	OSASCO	353440	1	15/06/2021		2	6
DF			BRASILIA	530010	1	17/06/202	26/06/202	1	6
SP	GVE XXI PR	1346	PRESIDENT	354140	1	25/06/202	09/07/202	2	6
SP	GVE XXV S/	1349	SANTOS	354850	1	07/04/2021		3	2
MG	UBERABA	1461	UBERABA	317010	1	19/04/2021		1	
GO	CENTRAL	1779	GOIANIA	520870	1	18/07/202	23/07/202	2	
SP	GVE VII SA	1332	SAO BERN/	354870	1	07/06/2021		2	
PI	4 GRS TERI	1883	TERESINA	221100	1	27/03/202	01/04/202	2	4

GO	PIRINEUS	1792	ANAPOLIS	520110	1	08/12/2021		2	6
PB	I NRS JOAC	1377	JOAO PESS	250750	1	01/03/202	07/03/202	2	6
RS	002 CRS	1607	PORTO ALE	431490	1	08/03/202	30/03/202	1	3
PR	17RS LOND	1371	LONDRINA	411370	1	01/04/202	15/04/202	1	4
SC	CRICIUMA	1558	ICARA	420700	1	04/04/202	10/04/202	2	6
PR	16RS APUC	1370	APUCARAN	410140	1	15/04/202	18/04/202	1	6
PR	05RS GUAF	1359	GUARAPU/	410940	1	20/05/202	23/06/202	1	6
GO	CENTRAL	1779	GOIANIA	520870	1	24/05/202	01/06/202	2	
MT	CUIABA	1578	CUIABA	510340	1	11/05/202	13/05/202	2	
SP	GVE I CAPI	1331	SAO PAULC	355030	1	10/06/202	17/06/202	1	
TO			PALMAS	172100	1	01/07/202	12/07/202	2	2
PR	02RS METF	1356	CURITIBA	410690	1	09/08/202	10/08/202	2	
RS	005 CRS	1608	CAXIAS DO	430510	1	25/08/2021		2	
SP	GVE XXXI S	1353	PORTO FEL	354060	1	19/08/202	20/08/202	3	6
SP	GVE XXVII S	1351	SAO JOSE D	354990	1	27/01/2021		3	
AM	ENTORNO	5584	MANAUS	130260	1	02/02/202	20/03/202	1	6
SP	GVE VII SAI	1332	SAO CAETA	354880	1	21/01/202	02/02/202	2	
DF			BRASILIA	530010	1	19/07/202	05/08/202	1	6
PA	01 REGION	1484	BELEM	150140	1	07/02/2021		2	9
SP	GVE XVII C/	1342	JUNDIAI	352590	1	25/03/2021		3	6
RS	001 CRS	1592	CANOAS	430460	1	29/03/202	12/04/202	1	2
SC	FLORIANOI	1476	SAO JOSE	421660	1	31/03/202	13/04/202	1	
SP	GVE I CAPI	1331	SAO PAULC	355030	1	17/06/202	29/06/202	2	3
SP	GVE XIX M/	1344	MARILIA	352900	1	03/04/202	14/04/202	2	2
MG	BELO HORI	1449	BELO HORI	310620	1	08/06/202	23/06/202	2	5
MG	LEOPOLDIN	1453	LEOPOLDIN	313840	1	31/03/2021		2	
PR	02RS METF	1356	CAMPINA (	410400	1	05/08/202	12/08/202	2	6
GO	CENTRAL	1779	GOIANIA	520870	1	31/05/202	31/05/202	3	1
RS			IGREJINHA	431010	1	12/12/202	16/12/202	2	
RJ			RIO DE JAN	330455	1	30/11/202	05/12/202	3	6
MG	BELO HORI	1449	BELO HORI	310620	1	14/03/2021		2	6
SP	GVE XVII C/	1342	CAMPINAS	350950	1	07/04/202	13/04/202	1	6
MS	EX ARS DE	1975	CAMPO GF	500270	1	15/04/2021		1	5
RS	005 CRS	1608	CAXIAS DO	430510	1	19/04/202	24/04/202	2	
RJ			RIO DE JAN	330455	1	14/05/2021		1	9
MG	PIRAPORA	1469	PIRAPORA	315120	1	07/06/2021		1	2
SP	GVE XII AR/	1337	MATAO	352930	1	19/03/2021		2	6
RS	002 CRS	1607	PORTO ALE	431490	1	28/05/202	01/06/202	2	
SP	GVE I CAPI	1331	SAO PAULC	355030	1	07/06/202	26/06/202	1	5
GO	CENTRAL	1779	GOIANIA	520870	1	14/07/202	17/07/202	2	
DF			BRASILIA	530010	1	04/08/202	21/10/202	2	6
PR			LONDRINA	411370	1	06/09/202	20/09/202	1	6
MG	PASSOS	1455	PASSOS	314790	1	25/05/2021		2	
SP	GVE XVII C/	1342	INDAIATUE	352050	1	10/02/2021		2	
GO	CENTRAL	1779	GOIANIA	520870	1	09/02/202	26/02/202	2	
PA			BELEM	150140	1	19/03/202	29/03/202	1	6
MT	CUIABA	1578	CUIABA	510340	1	25/02/202	16/03/202	1	

RS	004 CRS	1595	SANTA MA	431690	1	06/04/202	10/04/202	2	
SP	GVE XVII C.	1342	JUNDIAI	352590	1	17/04/2021		2	6
TO			PORTO NA	171820	1	30/03/202	04/04/202	2	2
MG	SAO JOAO	1460	SAO JOAO	316250	1	25/04/2021		2	
MG	JUIZ DE FO	1452	JUIZ DE FO	313670	1	01/05/202	06/05/202	1	2
PR	17RS LOND	1371	LONDRINA	411370	1	09/05/202	21/05/202	1	2
MG	DIVINOPOI	1472	DIVINOPOI	312230	1	08/05/202	11/05/202	1	
SP	GVE XXIV F	1348	RIBEIRAO F	354340	1	21/05/2021		2	
SP	GVE XVII C.	1342	CAMPINAS	350950	1	29/03/202	13/04/202	2	6
RJ			RIO DE JAN	330455	1	17/06/2021		3	6
DF			BRASILIA	530010	1	12/07/202	03/08/202	1	6
SP	GVE I CAPI	1331	SAO PAULO	355030	1	09/05/202	11/05/202	2	6
ES	COLATINA	1509	LINHARES	320320	1	23/03/202	29/03/202	9	9
RN	VII URSAP	1416	NATAL	240810	1	08/06/202	13/06/202	3	6
BA	NUCLEO RI	1381	FEIRA DE S.	291080	1	03/03/2021		2	
MA	REGI0NAL	1430	SAO LUIS	211130	1	20/02/2021		1	
SP	GVE XVII C.	1342	CAMPINAS	350950	1	19/03/202	11/04/202	1	
RJ			MARICA	330270	1	21/05/202	25/05/202	3	
SP	GVE VII SAI	1332	SANTO AN	354780	1	30/03/2021		3	9
MG	MONTES C	1473	MONTES C	314330	1	11/03/2021		2	6
PE	001	1497	RECIFE	261160	1	08/05/202	18/05/202	1	
PR	05RS GUAF	1359	GUARAPU.	410940	1	17/05/202	31/05/202	1	6
PR	02RS METF	1356	CAMPINA (	410400	1	17/05/202	23/05/202	1	5
SP	GVE X OSA	1335	BARUERI	350570	1	20/05/2021		1	
PA	01 REGION	1484	BELEM	150140	1	29/04/202	05/05/202	1	
SP	GVE XXXI S	1353	CERQUILH(	351150	1	27/02/202	06/03/202	2	
SP	GVE XIX M.	1344	MARILIA	352900	1	29/06/202	30/07/202	1	6
SP	GVE XXIV F	1348	RIBEIRAO F	354340	1	19/07/202	18/08/202	1	
PR	02RS METF	1356	CURITIBA	410690	1	15/08/202	14/09/202	1	5
PR	02RS METF	1356	CURITIBA	410690	1	14/03/202	14/03/202	2	2
SP	GVE XII AR.	1337	SAO CARLO	354890	1	29/03/2021		1	6
RJ			RIO DE JAN	330455	1	09/03/2021		2	6
MT	CUIABA	1578	CUIABA	510340	1	17/03/2021		1	
SP	GVE XVII C.	1342	CAMPINAS	350950	1	04/05/202	13/05/202	2	
RJ			NITEROI	330330	1	24/07/2021		1	
RO	VI GRS POF	1483	PORTO VEL	110020	1	06/05/2021		1	6
CE	1 CRES FOF	1519	FORTALEZ.	230440	1	28/05/202	03/06/202	1	1
MS	NRS DE DO	1966	DOURADO:	500370	1	30/12/2021		2	
SC	RIO DO SUI	1478	RIO DO SUI	421480	1	17/01/2021		2	
SP	GVE X OSA	1335	OSASCO	353440	1	13/10/202	13/10/202	2	6
SP	GVE XVII C.	1342	CAMPINAS	350950	1	11/03/2021		2	9
MT	CUIABA	1578	CUIABA	510340	1	29/07/202	12/08/202	3	
PR	02RS METF	1356	CAMPO LA	410420	1	08/02/202	26/02/202	1	6
RS	012 CRS	1601	SAO BORJ.	431800	1	29/03/202	15/04/202	2	
MS	NRS DE TRI	6255	TRES LAGO	500830	1	13/03/2021		2	
MS	EX NRS DE	6256	CORUMBA	500320	1	05/04/2021		1	
PE	001	1497	RECIFE	261160	1	23/03/2021		1	6

SP	GVE XVI BC	1341	BOTUCATU	350750	1	14/05/202	25/06/202	1	4
SP	GVE XX PIR	1345	PIRASSUNU	353930	1	28/04/2021		1	2
GO	SERRA DA I	1790	URUACU	522160	1	22/06/2021		2	6
RJ			RIO DE JAN	330455	1	01/06/202	03/06/202	2	6
RJ			CAMPOS D	330100	1	03/07/2021		2	
MA	REGI0NAL	1430	SAO LUIS	211130	1	07/07/202	26/07/202	2	5
PR	09RS FOZ D	1363	FOZ DO IGU	410830	1	19/09/202	23/09/202	2	6
PA	09 REGION	1492	SANTAREM	150680	1	04/10/2021		1	6
RN	VII URSAP	1416	NATAL	240810	1	22/08/202	30/08/202	2	3
GO	CENTRAL	1779	GOIANIA	520870	1	08/10/202	21/10/202	2	
SP	GVE XXVIII	1576	CARAGUAT	351050	1	19/01/2021		1	2
AL	1 MICRORI	1533	MACEIO	270430	1	09/03/2021		2	
MA	REGIONAL	1435	CURURUPU	210370	1	10/03/2021		1	6
SP	GVE XXV SA	1349	GUARUJA	351870	1	08/02/2021		1	
MG	JUIZ DE FO	1452	JUIZ DE FO	313670	1	20/04/202	15/05/202	1	
BA	NUCLEO RI	1380	SALVADOR	292740	1	17/04/202	24/04/202	1	6
SP	GVE I CAPI	1331	SAO PAULO	355030	1	28/04/202	02/05/202	2	2
PA	01 REGION	1484	BELEM	150140	1	15/04/202	16/04/202	1	5
SP	GVE I CAPI	1331	SAO PAULO	355030	1	20/05/202	21/05/202	2	1
SP	GVE XXIV R	1348	RIBEIRAO P	354340	1	23/04/202	26/04/202	2	
MG	PONTE NO	1458	PONTE NO	315210	1			2	6
MG	DIVINOPOI	1472	LAGOA DA	313720	1	28/03/202	05/04/202	2	
MS	EX ARS DE	1975	CAMPO GR	500270	1	29/05/2021			
CE	1 CRES FOI	1519	FORTALEZA	230440	1	05/05/202	14/05/202	1	1
SP	GVE XXIX S	1354	SAO JOSE D	354980	1	08/07/202	24/07/202	1	4
GO	CENTRAL	1779	GOIANIA	520870	1	20/06/202	20/06/202	2	
CE	1 CRES FOI	1519	FORTALEZA	230440	1	09/03/202	17/03/202	1	1
SE	REGIONAL	2056	ARACAJU	280030	1	30/05/2021		1	
SP	GVE XV BA	1340	BAURU	350600	1	03/06/202	09/06/202	2	9
PR	02RS METF	1356	CURITIBA	410690	1	02/03/202	03/03/202	1	5
DF			BRASILIA	530010	1	15/02/202	22/02/202	2	6
SP	GVE I CAPI	1331	SAO PAULO	355030	1	13/08/2021		3	1
RR			BOA VISTA	140010	1			1	
SP	GVE XVI BC	1341	BOTUCATU	350750	1	25/04/202	13/06/202	1	4
RO					1	20/04/202	28/04/202	1	
GO	CENTRAL	1779	GOIANIA	520870	1	07/04/202	11/04/202	2	
MA	REGIONAL	1440	IMPERATRI	210530	1	22/04/202	25/04/202	2	6
SP	GVE I CAPI	1331	SAO PAULO	355030	1	10/06/202	23/06/202	2	2
RS	002 CRS	1607	PORTO ALE	431490	1	19/03/202	11/04/202	1	9
SP	GVE XXVI S	1350	MOGI GUA	353070	1	15/05/202	12/06/202	1	2
RR			BOA VISTA	140010	1	18/05/202	06/06/202	1	
SP	GVE VIII M	1333	GUARULHO	351880	1	28/05/202	16/06/202	2	5
PR	17RS LOND	1371	LONDRINA	411370	1	15/06/202	18/06/202	2	6
RJ			CAMPOS D	330100	1	01/09/2021		2	
PR	15RS MARI	1369	MARINGA	411520	1	04/11/2021		2	
MT	SINOP	1584	NOVA MUT	510622	1	28/05/2021		1	
SP	GVE VIII M	1333	SUZANO	355250	1	30/05/202	07/06/202	1	9

MT			PRIMAVER	510704	1	18/07/202	01/08/2021		
SP	GVE XVII C/	1342	CAMPINAS	350950	1	10/03/202	18/03/202	1	6
SP	GVE I CAPI	1331	SAO PAULO	355030	1	02/03/202	05/03/202	2	6
SP			SAO PAULO	355030	1	21/04/2021		2	
MG	PONTE NO	1458	VICOSA	317130	1	21/03/2021		9	9
SC	FLORIANOI	1476	FLORIANOI	420540	1	31/05/202	02/06/202	2	9
SP	GVE XXVI S	1350	MOGI GUA	353070	1	18/06/202	25/06/202	2	9
SP	GVE X OSA	1335	OSASCO	353440	1	23/06/202	30/06/202	3	2
RS	010 CRS	1599	URUGUAIA	432240	1	01/06/202	02/06/202	1	6
SP	GVE I CAPI	1331	SAO PAULO	355030	1	21/05/202	01/06/202	1	
PR	02RS METF	1356	CURITIBA	410690	1	08/08/202	20/08/202	2	6
RJ			RIO DE JAN	330455	1	10/07/2021		2	6
MG	VARGINHA	1466	VARGINHA	317070	1	11/08/2021			
MG	PATOS DE I	1456	PATOS DE I	314800	1	17/08/2021		2	
MT	CUIABA	1578	CUIABA	510340	1	10/03/202	11/03/202	2	
DF			BRASILIA	530010	1	29/04/202	04/05/202	1	6
RJ			RIO DE JAN	330455	1	18/03/2021		2	6
GO	CENTRAL	1779	GOIANIA	520870	1	25/05/202	27/05/202	2	
SP	GVE X OSA	1335	BARUERI	350570	1	27/06/2021		2	
PR	02RS METF	1356	CAMPO LA	410420	1	17/06/202	19/06/202	1	6
SP	GVE I CAPI	1331	SAO PAULO	355030	1	20/06/202	02/07/202	2	6
SP	GVE XVII C/	1342	CAMPINAS	350950	1	23/06/202	04/07/202	2	6
SP	GVE XXXI S	1353	SOROCABA	355220	1	27/06/2021		2	
SP	GVE I CAPI	1331	SAO PAULO	355030	1	09/07/202	14/07/2021		
PR	09RS FOZ D	1363	FOZ DO IGU	410830	1	30/11/2021		2	
BA	NUCLEO RI	1380	SALVADOR	292740	1	24/03/202	29/03/202	1	5
BA	NUCLEO RI	1380	SALVADOR	292740	1	05/12/202	07/12/202	2	2
BA	NUCLEO RI	1403	BARREIRAS	290320	1	29/07/202	02/08/202	1	
RJ			RIO DE JAN	330455	1	24/02/2021		2	2
RJ			PETROPOLI	330390	1	25/03/202	06/04/202	1	
SP	GVE I CAPI	1331	SAO PAULO	355030	1	01/04/202	14/04/202	1	4
SP	GVE I CAPI	1331	SAO PAULO	355030	1	22/03/202	13/04/202	1	
MA	REGI0NAL	1430	SAO LUIS	211130	1	20/05/2021		1	9
MS	NRS DE TRI	6255	TRES LAGO	500830	1	22/07/2021		2	
PR	16RS APUC	1370	APUCARAN	410140	1	26/06/202	01/07/202	1	
SP	GVE XXIX S	1354	SAO JOSE D	354980	1	31/05/202	10/06/202	2	
RN					1	23/04/202	01/05/202	1	6
GO	CENTRAL	1779	GOIANIA	520870	1	10/06/202	11/06/202	2	
PR	10RS CASC	1364	CASCAVEL	410480	1	29/03/202	07/04/202	1	
CE	1 CRES FOF	1519	FORTALEZA	230440	1	17/03/202	28/03/202	2	
RO	VI GRS POF	1483	PORTO VEL	110020	1	06/02/202	17/02/202	1	6
MA					1	18/01/202	26/01/2021		2
SP	GVE XXXI S	1353	SOROCABA	355220	1	07/06/202	16/06/202	1	
PR	01RS PARA	1355	PARANAGU	411820	1	11/05/202	28/05/202	1	6
SP	GVE I CAPI	1331	SAO PAULO	355030	1	26/05/2021		2	
SC	JOINVILLE	1565	JOINVILLE	420910	1	14/05/202	24/05/202	1	6
SP	GVE VIII M	1333	MOGI DAS	353060	1	08/02/2021		3	1

ES	METROPOI	1510	SERRA	320500	1	02/04/202	16/04/202	1	
MG	UBA	1464	VISCONDE	317200	1	28/05/2021			
SP	GVE I CAPI	1331	SAO PAULC	355030	1	08/05/2021		2	
SP	GVE VIII M	1333	GUARULHC	351880	1	27/03/202	29/03/202	3	2
MG	UBERLAND	1462	COROMAN	311930	1	15/04/202	27/04/202	2	6
BA	NUCLEO RI	1388	PORTO SEC	292530	1	20/07/2021		2	6
SP	GVE I CAPI	1331	SAO PAULC	355030	1	10/02/202	21/02/202	1	5
SP					1	25/04/2021			
RS	014 CRS	1602	SANTA ROS	431720	1	08/05/202	19/05/202	1	
GO	CENTRAL	1779	GOIANIA	520870	1	21/01/2021			
GO	CENTRAL	1779	GOIANIA	520870	1	25/02/202	28/02/202	2	
SP	GVE I CAPI	1331	SAO PAULC	355030	1	16/03/202	18/03/202	2	2
MT	CUIABA	1578	CUIABA	510340	1	04/03/202	12/03/202	3	6
SP	GVE X OSA	1335	CARAPICUI	351060	1	16/04/202	16/04/2021		
RJ			NILOPOLIS	330320	1			2	2
PA	01 REGION	1484	BELEM	150140	1	03/05/2021		2	
SP	GVE I CAPI	1331	SAO PAULC	355030	1	07/04/202	09/04/202	2	6
SP	GVE XXVII S	1351	SAO JOSE D	354990	1	16/05/202	20/05/202	2	
SP	GVE XXXI S	1353	ITU	352390	1	04/01/2022		3	
SC	CONCORDI	1560	CONCORDI	420430	1	21/04/202	26/04/202	2	6
RO	I GRS JI PAI	1474	OURO PRET	110015	1	09/02/202	28/02/202	1	
MG	BELO HORI	1449	BETIM	310670	1	19/03/202	08/04/202	3	2
GO	CENTRAL	1779	GOIANIA	520870	1	02/06/202	06/06/202	2	
MG	DIVINOPOI	1472	DIVINOPOI	312230	1	28/06/202	12/07/202	2	
GO	CENTRAL	1779	GOIANIA	520870	1	14/03/202	28/03/202	1	
SP	GVE XXVII S	1351	SAO JOSE D	354990	1	17/08/2021		3	6
MG	GOVERNAD	1471	GOVERNAD	312770	1	18/07/202	19/07/202	1	2
RS	002 CRS	1607	PORTO ALE	431490	1	02/07/202	16/07/202	1	
PR	10RS CASC	1364	CASCAVEL	410480	1	05/08/202	28/08/202	2	
SP	GVE VII SAI	1332	SAO BERN/	354870	1	08/08/2021		2	6
RJ			RIO DE JAN	330455	1	09/01/202	11/01/202	3	6
SP	GVE XVII C/	1342	CAMPINAS	350950	1	29/03/202	08/04/202	2	9
RS	005 CRS	1608	CAXIAS DO	430510	1	24/09/202	28/09/202	1	
GO	CENTRAL	1779	GOIANIA	520870	1	19/02/202	02/04/202	1	
MA	REGI0NAL	1430	SAO LUIS	211130	1	11/03/202	04/04/202	1	2
MG	JUIZ DE FO	1452	JUIZ DE FO	313670	1	02/06/202	23/06/202	1	
SC	RIO DO SUI	1478	IBIRAMA	420690	1	20/05/2021		2	6
PR	17RS LOND	1371	LONDRINA	411370	1	10/06/2021		2	5
PR	13RS CIAN(	1367	CIANORTE	410550	1	17/05/202	28/05/202	1	
GO	CENTRAL	1779	GOIANIA	520870	1	16/05/202	08/06/202	1	
RJ			DUQUE DE	330170	1	20/07/2021		2	6
SP	GVE IX FRA	1334	FRANCISCC	351630	1	02/07/2021		3	
SP			ITAPIRA	352260	1	04/06/202	27/06/202	1	6
PR	02RS METF	1356	CURITIBA	410690	1	15/12/202	15/12/202	3	6
SP	GVE XXVIII	1576	CARAGUAT	351050	1	03/07/202	04/07/202	2	9
RS	017 CRS	1604	IJUI	431020	1	10/03/202	14/03/202	2	
MT	CUIABA	1578	CUIABA	510340	1	12/08/202	16/08/202	2	

RJ			RIO DE JAN	330455	1	03/03/2021	1	
MA					1	08/06/2021	1	
PR	02RS METF	1356	CURITIBA	410690	1	25/06/202 10/08/202	1	5
PR	15RS MARI	1369	MARINGA	411520	1	05/07/202 04/08/202	1	
RS	002 CRS	1607	PORTO ALE	431490	1	11/04/202 03/05/202	1	3
BA	NUCLEO RE	1381	FEIRA DE S.	291080	1	03/07/2021		
SC	JOINVILLE	1565	JOINVILLE	420910	1	02/09/202 06/09/202	2	6
BA	NUCLEO RE	1381	FEIRA DE S.	291080	1	10/05/2021	2	
RR			BOA VISTA	140010	1	04/11/2021	2	
SP	GVE I CAPI	1331	SAO PAULO	355030	1	26/04/202 04/05/202	1	6
PA	01 REGION	1484	BELEM	150140	1	09/12/2021	1	2
SP	GVE XVII C.	1342	CAMPINAS	350950	1	12/04/2021	2	6
CE	1 CRES FOF	1519	FORTALEZA	230440	1	12/03/202 23/03/202	1	3
RJ			RIO DE JAN	330455	1	05/05/202 13/05/202	2	6
SP	GVE XXIV R	1348	RIBEIRAO P	354340	1	26/05/202 19/06/202	2	5
PR	09RS FOZ D	1363	FOZ DO IGU	410830	1	07/06/202 06/07/202	1	6
MA	REGIONAL	1440	IMPERATRI	210530	1	07/01/2022	2	
PR			LONDRINA	411370	1	07/08/202 21/08/202	1	5
BA	NUCLEO RE	1398	VITORIA DA	293330	1	01/01/2022	2	
RJ			VOLTA RED	330630	1		9	5
SP			SAO PAULO	355030	1	17/04/202 24/04/202	3	2
SP	GVE VIII M	1333	MOGI DAS	353060	1	23/03/202 26/03/202	3	9
SP	GVE I CAPI	1331	SAO PAULO	355030	1	08/07/202 22/07/202	1	9
BA			SALVADOR	292740	1	25/01/202 28/01/202	2	
SP	GVE XXXI S	1353	SOROCABA	355220	1	17/04/202 23/04/202	1	
MG	PONTE NO	1458	PONTE NO	315210	1		2	6
SP	GVE I CAPI	1331	SAO PAULO	355030	1	04/03/202 12/03/202	3	
PR	02RS METF	1356	CURITIBA	410690	1	24/02/202 18/03/202	2	6
SP	GVE XXVI S	1350	VARGEM G	355640	1	15/03/2021	1	6
SP	GVE XXXI S	1353	SOROCABA	355220	1	15/03/202 17/03/202	1	2
MT	SINOP	1584	NOVA MUT	510622	1	10/06/2021	2	
SP	GVE XXVII S	1351	JACAREI	352440	1	08/08/202 13/08/202	2	6
AL	9 MICRORE	1541	PALMEIRA	270630	1	21/05/2021	2	1
PR	20RS TOLE	1374	TOLEDO	412770	1	20/10/202 21/10/202	2	6
MT	CUIABA	1578	CUIABA	510340	1	10/06/202 23/06/202	1	
PA	11 REGION	1494	MARABA	150420	1	27/12/2021	1	6
AM	ENTORNO	5584	MANAUS	130260	1	16/08/202 23/08/202	1	6
RJ			CAMPOS D	330100	1	21/08/202 28/08/202	3	
RJ			CAMPOS D	330100	1	09/02/202 23/02/202	1	6
SP	GVE XXXI S	1353	SOROCABA	355220	1	03/09/2021		
DF			BRASILIA	530010	1	02/02/202 08/02/202	2	6
AM	ENTORNO	5584	MANAUS	130260	1	06/03/202 07/03/2021		
SP	GVE I CAPI	1331	SAO PAULO	355030	1	25/03/202 28/03/202	2	
DF			BRASILIA	530010	1	23/02/202 17/03/202	1	
RS	001 CRS	1592	NOVO HAM	431340	1	25/05/202 09/06/202	1	
RJ			RIO DE JAN	330455	1	27/04/2021	2	
SP			SAO PAULO	355030	1	19/05/202 03/06/202	2	

SP	GVE XXIX S	1354	SAO JOSE D	354980	1	17/04/202	12/05/202	1	4
RJ			RIO DE JAN	330455	1	09/04/2021		1	
RJ			RIO DE JAN	330455	1	10/04/2021		1	9
BA	NUCLEO RE	1380	SALVADOR	292740	1	16/04/202	17/04/202	2	6
CE	1 CRES FOR	1519	FORTALEZA	230440	1	04/03/202	19/03/202	1	3
SP	GVE XVII CA	1342	CAMPINAS	350950	1	17/07/202	26/07/202	2	2
PR	16RS APUC	1370	APUCARAN	410140	1	09/07/202	14/07/202	1	
SP	GVE XV BA	1340	BAURU	350600	1	29/08/202	07/09/202	2	6
RJ			RIO DE JAN	330455	1	16/03/2021		1	
RS	003 CRS	1593	PELOTAS	431440	1	10/01/202	13/01/202	2	6
GO	CENTRAL	1779	GOIANIA	520870	1	24/08/202	01/09/202	2	
RS	002 CRS	1607	PORTO ALE	431490	1	26/02/202	04/03/202	1	3
SP	GVE VIII M	1333	MOGI DAS	353060	1	13/05/2021		1	2
SP	GVE VIII M	1333	GUARULHO	351880	1	23/03/202	28/03/202	1	2
PA	11 REGION	1494	MARABA	150420	1	22/04/2021		2	
MG	GOVERNAD	1471	GOVERNAD	312770	1	04/01/2021		1	9
BA	NUCLEO RE	1380	SALVADOR	292740	1	20/06/202	30/06/202	2	6
PR	15RS MARI	1369	MARINGA	411520	1	12/04/202	22/04/202	1	6
BA	NUCLEO RE	1403	BARREIRAS	290320	1	30/04/2021		1	2
PR	02RS METR	1356	CURITIBA	410690	1	13/05/202	17/05/202	2	5
RS	005 CRS	1608	GRAMADO	430910	1	20/08/202	23/08/202	3	6
SP	GVE XX PIR	1345	PIRACICAB	353870	1			2	
SP	GVE XXVI S	1350	MOGI GUA	353070	1	17/05/202	31/05/202	2	2
DF			BRASILIA	530010	1	12/08/202	06/09/202	1	6
PR	02RS METR	1356	CURITIBA	410690	1	07/08/202	16/08/202	1	5
SP	GVE I CAPI	1331	SAO PAULO	355030	1	15/08/2021		2	2
RJ			MARICA	330270	1	21/05/202	22/05/202	3	
RJ			MIGUEL PE	330290	1	19/02/2021		2	9
RS	002 CRS	1607	PORTO ALE	431490	1	07/03/202	12/04/202	1	
RS	002 CRS	1607	PORTO ALE	431490	1	13/03/202	15/03/202	2	6
PR	10RS CASC	1364	CASCAVEL	410480	1	14/06/202	05/07/202	2	
CE	1 CRES FOR	1519	FORTALEZA	230440	1	28/03/202	02/04/202	2	
SC	FLORIANOI	1476	FLORIANOI	420540	1	04/07/202	10/07/202	1	
SP	GVE I CAPI	1331	SAO PAULO	355030	1	25/05/202	27/05/202	2	
CE	1 CRES FOR	1519	FORTALEZA	230440	1	28/03/202	29/03/202	1	
SE	REGIONAL	2056	ARACAJU	280030	1	30/05/202	12/06/202	1	
MT	CUIABA	1578	CUIABA	510340	1	26/04/202	03/05/202	3	
PR	20RS TOLEI	1374	TOLEDO	412770	1	15/09/2021		1	2
RR			BOA VISTA	140010	1	24/02/202	15/04/202	1	
SE	REGIONAL	2056	ARACAJU	280030	1	02/06/202	09/06/202	1	
BA	NUCLEO RE	1403	BARREIRAS	290320	1	11/04/202	11/04/202	2	
PR	15RS MARI	1369	MARINGA	411520	1	25/09/202	06/10/202	1	6
RS	002 CRS	1607	PORTO ALE	431490	1	08/07/202	23/07/202	2	6
CE	11 CRES SC	1521	SOBRAL	231290	1	24/05/202	08/06/202	1	
SP	GVE VII SAI	1332	SAO CAETA	354880	1	31/10/2021		2	
SP	GVE XXVI S	1350	ITAPIRA	352260	1	08/08/202	17/09/202	1	6
SP	GVE XVII CA	1342	CAMPINAS	350950	1	24/09/202	27/09/202	2	9

CE	1 CRES FOF	1519	FORTALEZ/	230440	1	25/02/202	27/02/202	2	
SP	GVE VIII M	1333	GUARULH(	351880	1	20/02/2021		3	6
PR	17RS LOND	1371	LONDRINA	411370	1	31/05/2021		2	5
SP	GVE VIII M	1333	GUARULH(	351880	1	23/03/202	27/03/202	1	2
SP	GVE I CAPI	1331	SAO PAUL(	355030	1	23/03/202	05/04/202	1	
MA	REGI0NAL	1430	SAO LUIS	211130	1	08/02/202	05/04/202	3	3
PA	01 REGION	1484	BELEM	150140	1	06/04/202	09/04/202	2	6
RS	017 CRS	1604	IJUI	431020	1	19/06/202	09/07/202	1	
AC	REGIONAL	1938	RIO BRANC	120040	1	07/06/2021		3	6
TO			PALMAS	172100	1	22/05/202	27/05/202	2	2
RJ			RIO DE JAN	330455	1	09/06/202	11/06/202	2	6
AL	3 MICRORI	1535	SAO MIGU	270860	1	03/08/202	07/08/202	2	
CE	1 CRES FOF	1519	FORTALEZ/	230440	1	21/05/202	29/05/202	1	2
CE	1 CRES FOF	1519	FORTALEZ/	230440	1	03/04/202	19/04/202	1	
SP	GVE XVII C/	1342	ITATIBA	352340	1	08/06/202	01/07/202	1	2
SP	GVE XVII C/	1342	CAMPINAS	350950	1	21/08/202	01/09/202	1	
RJ			RIO DE JAN	330455	1			1	9
MG	ALFENAS	1379	ALFENAS	310160	1	23/03/202	25/03/202	2	6
SP	GVE XXV S/	1349	CUBATAO	351350	1	16/06/2021		2	
RJ			RIO DE JAN	330455	1	19/03/2021		2	
GO	CENTRAL	1779	GOIANIA	520870	1	19/07/202	31/07/202	1	
CE	15 CRES CF	1525	CRATEUS	230410	1	06/03/202	16/03/202	1	
SP	GVE I CAPI	1331	SAO PAUL(	355030	1	14/03/202	19/03/202	1	3
CE	1 CRES FOF	1519	FORTALEZ/	230440	1	06/03/202	19/03/202	1	2
CE	1 CRES FOF	1519	FORTALEZ/	230440	1	12/04/202	02/05/202	1	
CE	1 CRES FOF	1519	FORTALEZ/	230440	1	22/03/202	27/03/202	3	1
TO			PALMAS	172100	1	23/06/202	28/06/202	1	2
SP	GVE XVI BC	1341	AVARE	350450	1	13/08/2021		1	6
RJ			NILOPOLIS	330320	1	21/04/202	06/05/202	1	2
MG	BELO HORI	1449	BETIM	310670	1	13/03/2021		2	6
TO			PALMAS	172100	1	20/05/202	25/05/202	1	9
SP					1	11/03/202	13/03/202	3	
PR	15RS MARI	1369	MARINGA	411520	1	26/03/202	15/04/202	2	
MG	DIVINOPOI	1472	FORMIGA	312610	1			1	
MG	BELO HORI	1449	BETIM	310670	1	27/03/202	05/04/202	1	
MG	BELO HORI	1449	NOVA LIM/	314480	1	21/03/2021		1	
RO	VI GRS POF	1483	PORTO VEL	110020	1	31/03/202	17/04/202	1	
RJ			DUQUE DE	330170	1	07/07/202	09/07/202	3	6
SP	GVE XXVII !	1351	SAO JOSE I	354990	1	09/06/202	16/06/202	2	
MG	MONTES C	1473	MONTES C	314330	1	03/04/2021		2	2
PB	III NRS CAN	1421	CAMPINA (	250400	1	26/03/202	30/03/202	1	6
PI	4 GRS TERE	1883	TERESINA	221100	1	09/05/202	13/05/202	1	2
RJ			RIO BONIT(	330430	1	09/04/2021		9	
MS	EX ARS DE	1975	CAMPO GF	500270	1	27/04/2021		1	5
SP	GVE I CAPI	1331	SAO PAUL(	355030	1	16/07/202	23/07/202	2	
SP	GVE VII SAI	1332	SANTO AN	354780	1	02/06/2021			
MS	EX ARS DE	1975	CAMPO GF	500270	1	05/09/2021		2	6

RJ			VASSOURA	330620	1	24/06/202	01/07/2021		
SP	GVE XI ARA	1336	PENAPOLIS	353730	1	01/01/202	04/01/202	3	6
1					1	30/10/202	08/11/202	2	9
RJ			RIO DE JAN	330455	1	18/05/2021		2	
GO	CENTRAL	1779	GOIANIA	520870	1	31/12/202	08/01/202	1	
PR	10RS CASC	1364	CASCAVEL	410480	1	21/05/202	06/06/202	1	6
MT	CUIABA	1578	CUIABA	510340	1	22/04/2021		2	
DF			BRASILIA	530010	1	25/04/202	30/04/202	2	6
SP	GVE XVII C	1342	CAMPINAS	350950	1	24/05/2021		1	
SP	GVE XXI PR	1346	PRESIDENT	354140	1	02/06/202	14/06/202	1	6
MT	CUIABA	1578	CUIABA	510340	1	14/06/202	21/06/202	2	9
PA			BELEM	150140	1	08/06/2021		3	6
RS	013 CRS	1609	VENANCIO	432260	1	29/06/202	08/07/202	2	6
MG	BELO HORI	1449	CONTAGEN	311860	1	05/01/2021		9	
TO			PALMAS	172100	1	02/07/202	21/07/202	1	2
RJ			RIO DE JAN	330455	1	14/10/202	02/11/202	1	6
SP	GVE XIX M	1344	OSVALDO (	353460	1	08/06/202	29/07/202	1	2
SP	GVE XVII C	1342	CAMPINAS	350950	1	20/07/2021		2	
SP	GVE I CAPI	1331	SAO PAUL(	355030	1	05/07/202	06/07/202	2	
PR	02RS METF	1356	CURITIBA	410690	1	02/04/202	08/04/202	1	5
CE	8 CRES QU	1517	QUIXERAM	231140	1	11/02/2021		2	
PR	15RS MARI	1369	MARINGA	411520	1	16/02/202	24/02/202	3	2
MT	CUIABA	1578	CUIABA	510340	1	09/03/2021		3	
2					1	10/01/202	17/01/2022		
ES	METROPOI	1510	SERRA	320500	1	25/03/2021		1	
SC	JARAGUA I	1567	JARAGUA I	420890	1	19/05/202	28/05/202	1	5
MT	CUIABA	1578	CUIABA	510340	1	26/05/202	29/06/202	1	
GO	PIRINEUS	1792	ANAPOLIS	520110	1	02/03/202	15/03/202	1	
PR	17RS LONE	1371	LONDRINA	411370	1	09/05/202	16/05/202	2	5
MG	DIVINOPOI	1472	DIVINOPOI	312230	1	14/03/202	18/03/202	2	
MA	REGI0NAL	1430	SAO LUIS	211130	1	17/06/202	26/07/202	1	5
SP			DIADEMA	351380	1	22/08/2021		1	
MG	UBERLAND	1462	PATROCINI	314810	1	18/07/2021		2	9
MG	BELO HORI	1449	BELO HORI	310620	1	22/06/2021		3	2
SC	JOINVILLE	1565	JOINVILLE	420910	1	10/09/202	02/10/202	1	6
PR	15RS MARI	1369	MARINGA	411520	1	17/05/202	02/06/202	2	6
MT	SINOP	1584	SORRISO	510792	1	10/08/202	13/08/202	2	6
PR	14RS PARA	1368	PARANAVA	411840	1	09/11/202	21/11/202	1	
AM	ENTORNO	5584	MANAUS	130260	1	25/03/202	01/05/202	2	6
AM	ENTORNO	5584	MANAUS	130260	1	19/01/202	26/01/202	1	2
MG	JUIZ DE FO	1452	JUIZ DE FO	313670	1	22/02/2021		2	
DF			BRASILIA	530010	1	13/04/202	17/04/202	1	6
SP	GVE XXIV F	1348	RIBEIRAO F	354340	1	01/04/202	08/04/202	2	2
BA	NUCLEO RI	1380	SALVADOR	292740	1	28/01/202	01/02/202	2	
SP	GVE XVII C	1342	CAMPINAS	350950	1	24/06/202	28/06/202	3	5
BA	NUCLEO RI	1380	SALVADOR	292740	1	02/06/202	03/06/202	2	9
RJ			NITEROI	330330	1	28/05/2021		2	6

GO	CENTRAL	1779	GOIANIA	520870	1	01/08/202	08/08/202	1	
SP	GVE I CAPI	1331	SAO PAULC	355030	1	01/08/202	05/08/2021		
PR	09RS FOZ D	1363	FOZ DO IGU	410830	1	19/08/202	06/09/202	2	
MG	PEDRA AZU	1457	ALMENARA	310170	1	23/01/2021		3	6
RS	002 CRS	1607	PORTO ALE	431490	1	14/04/202	19/04/202	1	6
GO	PIRINEUS	1792	ANAPOLIS	520110	1	14/03/2021		1	6
SP	GVE I CAPI	1331	SAO PAULC	355030	1	14/02/2021		2	6
AP			MACAPA	160030	1	30/01/202	20/03/202	1	6
DF			BRASILIA	530010	1	21/08/202	06/09/202	1	6
MG	SETE LAGO	1467	CURVELO	312090	1	17/05/2021		2	2
RJ			RIO DE JAN	330455	1	14/04/2021			
MG	UBERLAND	1462	UBERLAND	317020	1	25/07/2021		3	6
SP	GVE I CAPI	1331	SAO PAULC	355030	1	09/08/2021		1	
RJ			RIO DE JAN	330455	1	23/08/202	01/10/202	2	
MA	REGIONAL	1440	IMPERATRI	210530	1	14/08/2021		2	6
RS	002 CRS	1607	PORTO ALE	431490	1	22/03/2021		1	4
PR	08RS FRAN	1362	FRANCISCC	410840	1	05/05/202	10/05/202	1	9
TO			PALMAS	172100	1	24/05/202	29/05/202	1	2
PR	17RS LOND	1371	LONDRINA	411370	1	06/04/2021		2	5
SP	GVE XXI PR	1346	PRESIDENT	354140	1	11/08/202	11/08/202	1	6
RO	VI GRS POF	1483	PORTO VEL	110020	1	16/05/202	31/05/202	1	9
SP	GVE I CAPI	1331	SAO PAULC	355030	1	15/07/202	16/07/202	9	2
SP	GVE I CAPI	1331	SAO PAULC	355030	1	20/03/202	21/03/202	2	9
MT	CUIABA	1578	CUIABA	510340	1	26/05/202	01/06/202	2	
SP	GVE XVII C.	1342	CAMPINAS	350950	1	15/03/202	23/03/202	2	
SP	GVE XII AR.	1337	SAO CARLC	354890	1	06/04/202	09/04/202	3	6
SP			JAGUARIUI	352470	1	25/06/2021		2	2
MT	CUIABA	1578	CUIABA	510340	1	01/06/202	15/06/202	1	
RS	002 CRS	1607	PORTO ALE	431490	1	18/05/202	11/06/202	1	
RS	002 CRS	1607	PORTO ALE	431490	1	30/03/202	10/04/202	1	
BA	NUCLEO RI	1380	SALVADOR	292740	1	22/06/202	23/06/202	2	9
MG	DIVINOPOI	1472	DIVINOPOI	312230	1	27/06/2021		2	6
RJ			RIO DE JAN	330455	1	11/09/202	14/09/202	3	6
BA	NUCLEO RI	1380	SALVADOR	292740	1	09/03/202	30/03/202	1	
SP	GVE XXIX S	1354	SAO JOSE D	354980	1	24/02/202	05/03/202	1	4
MT	CUIABA	1578	CUIABA	510340	1	03/10/202	06/10/202	2	
MG	DIVINOPOI	1472	PITANGUI	315140	1	07/03/2021		2	4
SP	GVE XXVI S	1350	MOJI MIRII	353080	1	25/03/2021		2	6
PE	001	1497	RECIFE	261160	1	30/03/2021		1	
CE	1 CRES FOF	1519	FORTALEZA	230440	1	14/04/202	29/04/202	1	
SP	GVE XXXI S	1353	SALTO DE F	354530	1	08/09/202	16/09/202	2	2
MT	SINOP	1584	SINOP	510790	1	24/04/2021		2	6
PR	10RS CASC	1364	CASCAVEL	410480	1	10/06/202	15/06/202	3	
MG	LEOPOLDIN	1453	LEOPOLDIN	313840	1	15/04/2021		2	
CE	1 CRES FOF	1519	FORTALEZA	230440	1	21/03/2021		1	
RR			BOA VISTA	140010	1	30/07/202	05/09/202	1	
GO	ESTRADA D	1802	CATALAO	520510	1	28/08/202	31/08/202	2	6

RJ			RIO DE JAN	330455	1	06/08/2021		2	6
TO			PALMAS	172100	1	19/07/202	22/07/202	1	6
MG	POUSO ALI	1459	POUSO ALI	315250	1	16/07/202	27/07/202	1	6
CE	1 CRES FOF	1519	FORTALEZ/	230440	1	26/04/202	04/05/202	1	
SC	BLUMENAI	1564	BLUMENAI	420240	1	04/02/202	08/03/202	1	6
BA	NUCLEO RI	1380	SALVADOR	292740	1	02/06/202	09/06/202	2	
MT	CUIABA	1578	CUIABA	510340	1	05/04/202	20/04/202	1	
SP	GVE XXVI S	1350	VARGEM G	355640	1	13/03/202	31/03/202	1	6
PE	001	1497	RECIFE	261160	1	15/04/202	19/04/202	2	5
DF			BRASILIA	530010	1	03/03/202	12/03/202	1	9
GO	CENTRAL	1779	GOIANIA	520870	1	09/03/202	24/03/202	1	
RS	006 CRS	1611	PASSO FUI	431410	1	08/03/202	19/04/202	1	3
MT	CUIABA	1578	CUIABA	510340	1	05/09/202	18/09/202	1	
SP	GVE XVII C/	1342	JUNDIAI	352590	1	26/03/2021		3	
PB	I NRS JOAC	1377	JOAO PESS	250750	1	21/10/202	22/10/202	3	6
MG	BELO HORI	1449	BETIM	310670	1	03/04/2021		3	3
RO	VI GRS POF	1483	PORTO VEL	110020	1	14/02/2021		1	6
AL					1	20/04/202	22/05/202	1	5
PR	15RS MARI	1369	MARINGA	411520	1	22/04/202	14/05/202	1	6
SP	GVE XVII C/	1342	CAMPINAS	350950	1	02/06/202	28/06/202	2	6
PA	01 REGION	1484	BELEM	150140	1			1	6
RS	018 CRS	1605	CAPAO DA	430463	1	21/09/2021		2	3
MG	JUIZ DE FO	1452	JUIZ DE FO	313670	1	16/05/202	29/05/202	1	2
MG	BELO HORI	1449	BELO HORI	310620	1	05/07/2021		1	3
SP	GVE XVII C/	1342	SUMARE	355240	1	11/05/2021		1	9
MT	CUIABA	1578	CUIABA	510340	1	02/09/202	05/09/202	3	
MT	BARRA DO	1580	BARRA DO	510180	1	28/05/202	29/05/202	1	9
SP	GVE XVII C/	1342	CAMPINAS	350950	1	15/07/202	03/08/202	1	6
SP	GVE XX PIR	1345	RIO CLARO	354390	1	06/07/2021		1	2
RN	VII URSAP	1416	NATAL	240810	1	12/06/202	17/06/202	2	2
RJ			DUQUE DE	330170	1			3	
PI	4 GRS TERE	1883	TERESINA	221100	1	04/04/202	17/04/202	1	2
SC	CRICIUMA	1558	CRICIUMA	420460	1	04/03/202	24/03/202	1	
CE	1 CRES FOF	1519	FORTALEZ/	230440	1	05/04/202	27/04/202	2	9
SC	XANXERE	1556	XANXERE	421950	1	01/05/202	05/05/202	2	6
RS	002 CRS	1607	PORTO ALE	431490	1	28/05/202	31/05/202	2	
PB	I NRS JOAC	1377	JOAO PESS	250750	1	10/05/2021		2	6
SP	GVE I CAPI'	1331	SAO PAUL(	355030	1	19/05/202	25/05/202	2	
TO			ARAGUAIN	170210	1	22/06/2021		2	6
RJ			RIO DE JAN	330455	1	27/06/2021		3	
SP	GVE XXI PR	1346	PRESIDENT	354140	1	05/04/202	17/04/202	1	6
GO	PIRINEUS	1792	ANAPOLIS	520110	1	18/07/202	22/07/202	2	6
SP	GVE I CAPI'	1331	SAO PAUL(	355030	1	04/05/202	07/05/202	2	6
MS	EX ARS DE	1975	CAMPO GF	500270	1	14/06/2021		2	3
MS	EX ARS DE	1975	CAMPO GF	500270	1	19/04/2021			5
MG			BETIM	310670	1	21/01/2021		2	6
RS	002 CRS	1607	PORTO ALE	431490	1	09/07/202	14/07/202	2	

SP	GVE XIX MA	1344	MARILIA	352900	1	30/04/202	17/05/202	2	6
DF			BRASILIA	530010	1	12/03/202	23/03/202	1	6
MT	CUIABA	1578	CUIABA	510340	1	06/10/202	25/10/202	1	9
RJ			NITEROI	330330	1	06/03/2021		2	9
PR	15RS MARI	1369	MARINGA	411520	1	25/06/202	02/07/202	1	6
SC	JOINVILLE	1565	JOINVILLE	420910	1	13/04/202	23/04/202	1	6
PR	17RS LOND	1371	LONDRINA	411370	1	16/07/202	16/07/202	3	6
MT	CUIABA	1578	CUIABA	510340	1	06/06/202	25/06/202	1	
PR	15RS MARI	1369	SARANDI	412625	1	01/03/202	04/03/202	2	
SP	GVE I CAPI	1331	SAO PAULO	355030	1	20/05/202	28/05/202	1	2
BA	NUCLEO RE	1380	SALVADOR	292740	1	26/06/202	29/06/202	2	9
RS	002 CRS	1607	PORTO ALE	431490	1	06/06/202	15/06/202	1	4
GO	CENTRAL	1779	GOIANIA	520870	1	16/07/202	19/07/202	3	6
MG	PASSOS	1455	SAO SEBAS	316470	1	18/07/2021		1	6
RJ			RIO DE JAN	330455	1	31/05/2021		1	6
PA			REDENCAC	150613	1	01/08/2021		2	6
RN	VII URSAP	1416	NATAL	240810	1	04/06/202	15/06/202	1	2
RR			BOA VISTA	140010	1	19/07/202	23/07/202	1	
RS	016 CRS	1610	LAJEADO	431140	1	28/07/202	30/07/202	2	6
SC	JOINVILLE	1565	JOINVILLE	420910	1	22/08/202	26/08/202	2	6
SC	FLORIANOI	1476	FLORIANOI	420540	1	08/09/2021		2	
AM	ENTORNO	5584	MANAUS	130260	1	11/05/2021		1	2
SP	GVE VII SAI	1332	SAO BERNA	354870	1	26/01/2021		2	
SC	JARAGUA D	1567	JARAGUA D	420890	1	29/10/202	22/11/202	1	5
GO	CENTRAL	1779	GOIANIA	520870	1	07/04/202	02/05/202	1	
PR	02RS METR	1356	CAMPO LA	410420	1	20/01/202	26/01/202	2	6
RS	001 CRS	1592	MONTENEG	431240	1	08/04/202	13/04/202	2	6
SP	GVE I CAPI	1331	SAO PAULO	355030	1	02/03/202	19/03/202	1	2
BA	NUCLEO RE	1380	SALVADOR	292740	1	24/05/202	26/05/202	2	
MG			PATOS DE I	314800	1	18/06/202	23/06/202	1	6
PR	15RS MARI	1369	SARANDI	412625	1	31/03/202	11/04/202	1	
SP	GVE XII ARA	1337	MATAO	352930	1	03/04/2021		2	5
SP	GVE XXIV R	1348	RIBEIRAO P	354340	1	14/06/202	29/06/202	1	4
SP	GVE XXVI S	1350	MOGI GUA	353070	1	26/05/202	28/05/202	2	9
RS	002 CRS	1607	PORTO ALE	431490	1	15/06/202	26/06/202	1	2
CE	1 CRES FOR	1519	FORTALEZA	230440	1	23/05/202	01/06/202	3	1
PR	07RS PATO	1361	PATO BRAN	411850	1	30/06/202	02/07/202	3	
PB	I NRS JOAO	1377	JOAO PESS	250750	1	30/09/2021		3	6
PR	17RS LOND	1371	LONDRINA	411370	1	18/04/202	19/04/202	2	6
CE	11 CRES SO	1521	SOBRAL	231290	1	01/04/202	03/06/202	1	
SP	GVE XVII CA	1342	CAMPINAS	350950	1	26/04/202	04/06/202	3	9
MT	CUIABA	1578	CUIABA	510340	1	10/03/202	22/03/202	2	6
PR	10RS CASC	1364	CASCAVEL	410480	1	04/02/202	08/02/202	2	2
GO			ANAPOLIS	520110	1	21/06/2021		2	6
SC	BLUMENAU	1564	BLUMENAU	420240	1	02/07/202	11/08/202	1	4
RJ			CAMPOS D	330100	1	29/05/2021		2	5
PR	09RS FOZ D	1363	FOZ DO IGU	410830	1	27/07/202	02/08/202	2	

PR	08RS FRAN	1362	SANTO AN	412440	1	27/03/202	17/04/202	1	9
SP	GVE XXXI S	1353	SOROCABA	355220	1	23/02/202	28/02/202	2	9
SP	GVE VII SAI	1332	SAO BERN/	354870	1	25/09/2021		1	2
SP	GVE XXXI S	1353	SOROCABA	355220	1	27/05/202	25/06/202	1	
ES	METROPOI	1510	SERRA	320500	1	09/06/2021		1	2
PA	09 REGION	1492	SANTAREM	150680	1	21/02/202	08/03/202	2	
SP	GVE XXIX S	1354	SAO JOSE D	354980	1	11/03/202	19/03/202	2	
SP	GVE I CAPI	1331	SAO PAULO	355030	1	23/04/202	29/04/202	2	3
SP	GVE XXI PR	1346	PRESIDENT	354140	1	28/04/202	07/05/202	2	6
MT	BARRA DO	1580	BARRA DO	510180	1	15/07/2021		2	
ES	METROPOI	1510	SERRA	320500	1	21/04/2021		1	3
SP	GVE I CAPI	1331	SAO PAULO	355030	1	21/05/202	06/06/202	1	
SP	GVE I CAPI	1331	SAO PAULO	355030	1	17/05/202	30/05/202	2	2
SP	GVE XXXI S	1353	SOROCABA	355220	1	22/05/2021		2	
DF			BRASILIA	530010	1	29/05/202	31/05/202	2	6
SP	GVE XV BA	1340	BAURU	350600	1	27/06/202	14/07/202	3	6
SP	GVE I CAPI	1331	SAO PAULO	355030	1	01/08/202	02/08/2021		
SP	GVE I CAPI	1331	SAO PAULO	355030	1	01/07/202	04/07/2021		
GO	CENTRO SU	1781	APARECIDA	520140	1	24/09/202	12/10/202	1	9
PR	17RS LOND	1371	LONDRINA	411370	1	21/09/202	29/10/202	1	5
RJ			VASSOURA	330620	1	23/04/2021		2	
PR	15RS MARI	1369	MARINGA	411520	1	05/05/202	17/05/202	1	6
GO	CENTRAL	1779	GOIANIA	520870	1	22/05/202	24/05/202	2	
RN	VII URSAP	1416	NATAL	240810	1	26/03/2021		1	3
RS	002 CRS	1607	PORTO ALE	431490	1	06/03/202	12/04/202	1	4
RS	007 CRS	1596	BAGE	430160	1	18/03/202	25/03/202	1	6
SP	GVE I CAPI	1331	SAO PAULO	355030	1	03/06/202	10/06/202	2	2
MS	NRS DE DO	1966	DOURADOS	500370	1	21/04/2021			
SE	REGIONAL	2056	ARACAJU	280030	1	14/04/2021		1	3
GO	CENTRAL	1779	GOIANIA	520870	1	01/05/202	11/05/202	1	
SC	BLUMENAU	1564	BLUMENAU	420240	1	08/09/202	17/10/202	1	4
SP	GVE XXVI S	1350	MOGI GUA	353070	1	14/06/202	22/06/202	3	6
SP	GVE XVIII F	1343	FRANCA	351620	1	17/08/202	23/08/202	2	
SP	GVE I CAPI	1331	SAO PAULO	355030	1	12/03/202	17/03/2021		
RJ			RIO DE JAN	330455	1	01/04/2021		1	
RS	002 CRS	1607	PORTO ALE	431490	1	04/04/202	09/04/202	1	4
SP	GVE I CAPI	1331	SAO PAULO	355030	1	29/03/202	30/03/202	2	2
RJ			VOLTA RED	330630	1	02/03/2021		1	
MG	CORONEL F	1468	CARATINGA	311340	1	16/04/2021		2	
RO	I GRS JI PA	1474	JI-PARANA	110012	1	09/03/2021		2	6
CE	1 CRES FOF	1519	FORTALEZA	230440	1	21/04/202	04/05/202	3	1
SP	GVE VIII M	1333	GUARULHO	351880	1	03/06/202	12/06/202	2	2
MA	REGI0NAL	1430	SAO LUIS	211130	1	10/05/202	15/05/202	2	1
SP	GVE I CAPI	1331	SAO PAULO	355030	1	12/05/202	16/05/2021		6
MG	PASSOS	1455	PASSOS	314790	1	09/05/2021		1	
GO	CENTRAL	1779	GOIANIA	520870	1	20/08/202	26/08/202	2	
MT	CUIABA	1578	CUIABA	510340	1	17/07/202	23/07/202	2	

SC	JOINVILLE	1565	JOINVILLE	420910	1	03/08/202	04/08/202	2	5
PR	16RS APUC	1370	APUCARAN	410140	1	28/04/2021		1	
RJ			CAMPOS D	330100	1	04/07/2021		1	5
RS	006 CRS	1611	PASSO FUN	431410	1	29/03/202	11/04/202	1	6
RS			PORTO ALE	431490	1	06/08/202	12/08/202	3	6
SC	ARARANGU	1562	ARARANGU	420140	1	02/05/202	03/05/202	1	
PR	10RS CASC	1364	CASCAVEL	410480	1	13/09/202	25/09/202	2	
SP	GVE I CAPI	1331	SAO PAULC	355030	1	21/03/202	24/03/202	1	5
TO			PALMAS	172100	1	07/09/202	11/09/202	2	
SE			ARACAJU	280030	1	24/04/2021		1	
PR	02RS METF	1356	CAMPINA (	410400	1	11/10/202	14/10/202	3	
GO	CENTRAL	1779	GOIANIA	520870	1	25/03/202	04/04/202	1	
MT	CUIABA	1578	CUIABA	510340	1	22/04/2021		2	
RO	VI GRS POF	1483	PORTO VEL	110020	1	11/04/2021		1	3
MG	UBERLAND	1462	UBERLAND	317020	1	04/03/2021		2	6
SP			SAO PAULC	355030	1	16/04/202	19/04/2021		
SP	GVE XXV S/	1349	GUARUJA	351870	1	29/03/202	07/04/202	2	9
SP	GVE I CAPI	1331	SAO PAULC	355030	1	28/03/202	02/04/202	2	3
BA	NUCLEO RI	1380	SALVADOR	292740	1	12/06/2021		2	6
MG	BELO HORI	1449	BELO HORI	310620	1	02/03/202	05/04/202	2	
SP	GVE I CAPI	1331	SAO PAULC	355030	1	28/09/2021		2	
RJ			RIO DE JAN	330455	1	09/04/2021		1	
PI	4 GRS TERE	1883	TERESINA	221100	1	07/11/202	11/11/202	1	3
SP	GVE I CAPI	1331	SAO PAULC	355030	1	12/01/202	22/01/202	2	
AM	ENTORNO	5584	MANAUS	130260	1	31/01/202	08/02/202	1	6
SP	GVE I CAPI	1331	SAO PAULC	355030	1	17/03/202	20/04/202	1	2
MT	CUIABA	1578	CUIABA	510340	1	09/02/202	17/02/202	2	
SP	GVE XIX M/	1344	OSVALDO (	353460	1	12/04/202	14/04/202	3	6
PR	02RS METF	1356	CURITIBA	410690	1	27/07/202	06/08/202	1	6
SP	GVE I CAPI	1331	SAO PAULC	355030	1	19/05/202	10/06/202	1	3
SP			SAO PAULC	355030	1	23/03/202	28/03/202	1	
SP	GVE X OSA	1335	OSASCO	353440	1	09/05/2021		1	2
SP	GVE I CAPI	1331	SAO PAULC	355030	1	14/06/202	16/06/202	3	
SP	GVE I CAPI	1331	SAO PAULC	355030	1	16/05/202	21/05/202	1	2
MG	BELO HORI	1449	BELO HORI	310620	1	11/04/202	20/04/202	1	2
PR	03RS PONT	1357	PONTA GR(	411990	1	09/03/202	22/03/202	2	6
SP	GVE I CAPI	1331	SAO PAULC	355030	1	06/05/2021		1	
SP	GVE I CAPI	1331	SAO PAULC	355030	1	22/05/202	25/05/202	1	2
PR	02RS METF	1356	CURITIBA	410690	1	09/08/202	11/08/202	2	6
SP	GVE XVIII F	1343	FRANCA	351620	1	21/01/202	04/02/202	2	6
SP	GVE I CAPI	1331	SAO PAULC	355030	1	31/03/202	18/04/202	2	2
MS	EX ARS DE	1975	CAMPO GF	500270	1	26/06/202	26/07/202	1	5
BA	NUCLEO RI	1380	SALVADOR	292740	1	31/01/2021		2	6
RO	VI GRS POF	1483	PORTO VEL	110020	1	01/03/2021		2	5
RS	002 CRS	1607	PORTO ALE	431490	1	24/03/202	26/03/202	1	3
PA	01 REGION	1484	BELEM	150140	1	09/03/202	05/04/202	1	6
SP			SAO PAULC	355030	1	04/06/202	07/06/202	2	

SP	GVE XVII C/	1342	JUNDIAI	352590	1	08/04/2021		1	2
SP	GVE X OSA	1335	OSASCO	353440	1	31/03/2021		2	2
RJ			DUQUE DE	330170	1	12/07/202	15/07/202	2	
MT	CUIABA	1578	CUIABA	510340	1	10/08/2021		3	
SP	GVE XXXI S	1353	SOROCABA	355220	1	29/03/202	07/04/202	1	
RJ			RIO DE JAN	330455	1	06/03/202	08/03/202	2	
SP	GVE XXI PR	1346	PRESIDENT	354140	1	26/06/202	08/07/202	1	6
SP	GVE I CAPI	1331	SAO PAULC	355030	1	06/04/202	14/04/202	1	
RS	002 CRS	1607	PORTO ALE	431490	1	18/03/202	19/03/202	1	
BA	NUCLEO RI	1380	SALVADOR	292740	1	17/03/202	18/04/202	1	6
ES	METROPOI	1510	SERRA	320500	1	29/03/202	08/04/202	1	2
PR	02RS METF	1356	CURITIBA	410690	1	10/07/202	19/07/202	1	2
BA	NUCLEO RI	1380	SALVADOR	292740	1	17/04/202	19/04/202	2	
SP	GVE XXIV F	1348	RIBEIRAO F	354340	1	23/06/202	23/07/202	1	
SP	GVE XVII C/	1342	CAMPINAS	350950	1	04/08/202	27/08/202	1	
DF			BRASILIA	530010	1	12/05/202	19/05/202	2	5
PR	16RS APUC	1370	APUCARAN	410140	1	20/04/2021		2	6
PR	14RS PARA	1368	PARANAV/	411840	1	23/06/202	30/06/202	2	6
MS	EX ARS DE	1975	CAMPO GF	500270	1	24/05/2021		1	
MT	CUIABA	1578	CUIABA	510340	1	12/04/202	15/04/202	2	9
MT	SINOP	1584	NOVA MU	510622	1	27/05/2021		3	
SP	GVE I CAPI	1331	SAO PAULC	355030	1	10/07/202	20/07/202	1	2
SP	GVE XV BA	1340	JAU	352530	1	06/08/2021		2	4
SP	GVE XVII C/	1342	CAMPINAS	350950	1	30/05/202	24/06/202	1	
GO	CENTRAL	1779	GOIANIA	520870	1	07/05/202	12/05/202	2	
RN	II URSAP	1412	MOSSORO	240800	1	03/06/202	11/06/202	1	2
SP	GVE XIX M/	1344	MARILIA	352900	1	10/03/202	11/03/202	2	6
DF			BRASILIA	530010	1	08/04/202	12/04/202	3	9
RJ			VASSOURA	330620	1	12/03/2021			
RO	VI GRS POF	1483	PORTO VEL	110020	1	05/05/2021		3	6
MT	CUIABA	1578	CUIABA	510340	1	18/04/202	21/04/202	2	
PR	07RS PATO	1361	PATO BRAI	411850	1	07/05/2021		3	6
SP	GVE I CAPI	1331	SAO PAULC	355030	1	25/05/202	12/06/202	3	
RR			BOA VISTA	140010	1	23/06/202	06/07/202	1	
PR	17RS LONI	1371	LONDRINA	411370	1	08/06/202	19/06/202	1	5
PR	17RS LONI	1371	LONDRINA	411370	1	18/04/202	25/04/202	1	4
SP	GVE XXXI S	1353	SOROCABA	355220	1	23/04/202	25/05/202	1	2
RS	004 CRS	1595	SANTA MA	431690	1	19/04/2021		1	
MS	EX ARS DE	1975	CAMPO GF	500270	1	28/07/2021		1	5
SC	BLUMENAU	1564	BLUMENAU	420240	1	17/02/202	20/02/202	2	2
SP	GVE I CAPI	1331	SAO PAULC	355030	1	20/02/202	27/02/202	2	5
SP	GVE I CAPI	1331	SAO PAULC	355030	1	07/02/202	12/02/202	2	
CE	1 CRES FOF	1519	FORTALEZ/	230440	1	27/05/202	01/06/202	3	1
RJ			ANGRA DO	330010	1	18/02/2021		2	
SP	GVE VII SAI	1332	RIBEIRAO F	354330	1	26/01/2021		3	6
PR	02RS METF	1356	CURITIBA	410690	1	26/03/202	09/04/202	1	2
GO	CENTRAL	1779	GOIANIA	520870	1	07/04/2021		2	

SP	GVE XX PIR	1345	LIMEIRA	352690	1	22/01/202	28/01/202		1	9
SC			CHAPECO	420420	1				2	
SP	GVE XVI BC	1341	PARANAPA	353580	1	23/06/2021			2	
MA	REGIONAL	1440	IMPERATRI	210530	1	24/02/2021			1	9
MS	NRS DE TRI	6255	TRES LAGO	500830	1	22/04/2021			2	
AM	ENTORNO	5584	MANAUS	130260	1	30/04/202	02/05/202		1	2
RJ			RIO DE JAN	330455	1	11/09/202	25/09/202		2	6
SP	GVE I CAPI	1331	SAO PAULO	355030	1	04/06/202	13/06/202		2	6
MT	CUIABA	1578	CUIABA	510340	1	29/03/202	11/04/202		9	9
RN	II URSAP	1412	MOSSORO	240800	1	11/05/202	11/05/202		1	
MA	REGI0NAL	1430	SAO LUIS	211130	1	11/07/202	24/07/202		2	5
MG	BELO HORI	1449	BELO HORI	310620	1	19/05/202	03/06/202		2	9
GO	CENTRAL	1779	GOIANIA	520870	1	30/01/2021			1	
RJ			RIO DE JAN	330455	1	10/09/202	15/09/202		2	6
BA	NUCLEO RE	1381	FEIRA DE S.	291080	1	16/05/202	20/05/202		2	
TO			PALMAS	172100	1	12/06/202	20/06/202		2	6
SP	GVE XV BA	1340	BAURU	350600	1	12/06/202	16/07/202		2	6
GO	CENTRAL	1779	GOIANIA	520870	1	23/06/202	05/07/202		1	
SP	GVE I CAPI	1331	SAO PAULO	355030	1	24/02/202	27/02/202		2	6
GO	CENTRAL	1779	GOIANIA	520870	1	25/08/202	12/09/202		1	
CE	1 CRES FOF	1519	FORTALEZA	230440	1	15/02/202	26/02/202		1	
SP	GVE I CAPI	1331	SAO PAULO	355030	1	01/06/2021			1	9
PR	10RS CASC	1364	CASCAVEL	410480	1	09/09/202	12/09/202		2	
BA	NUCLEO RE	1380	SALVADOR	292740	1	27/04/202	26/07/202		3	6
SP	GVE VIII M	1333	GUARULHO	351880	1	16/04/202	05/05/202		1	4
SP	GVE XVII C.	1342	CAMPINAS	350950	1	28/08/202	05/09/202		1	
PR	20RS TOLEI	1374	MARECHAI	411460	1	18/05/202	24/05/202		2	2
PR	09RS FOZ D	1363	FOZ DO IGU	410830	1	24/07/202	26/07/202		3	
MS	NRS DE DO	1966	DOURADOS	500370	1	28/08/2021			2	9
RS	002 CRS	1607	PORTO ALE	431490	1	23/05/2021			1	5
SP	GVE I CAPI	1331	SAO PAULO	355030	1	01/06/202	28/06/202		1	
ES	METROPOI	1510	SERRA	320500	1	05/11/2021			1	3
SP	GVE XVII C.	1342	CAMPINAS	350950	1	18/03/202	26/03/202		2	6
SP	GVE I CAPI	1331	SAO PAULO	355030	1	01/04/202	05/04/202		2	9
SP	GVE I CAPI	1331	SAO PAULO	355030	1	13/05/202	19/05/202		2	6
CE	1 CRES FOF	1519	FORTALEZA	230440	1	28/03/2021			1	
MA	REGIONAL	1440	IMPERATRI	210530	1	07/05/2021			1	6
GO	CENTRAL	1779	GOIANIA	520870	1	25/05/202	28/05/202		2	
PR	02RS METF	1356	CAMPO LA	410420	1	06/06/202	08/06/202		1	6
SP	GVE XVII C.	1342	CAMPINAS	350950	1	10/07/202	29/07/202		1	9
GO	ESTRADA D	1802	CATALAO	520510	1	02/05/2021			2	6
SP	GVE XVII C.	1342	CAMPINAS	350950	1	20/04/202	24/05/202		2	
PR	09RS FOZ D	1363	FOZ DO IGU	410830	1	03/06/202	25/06/202		1	
SP	GVE I CAPI	1331	SAO PAULO	355030	1	29/04/2021			2	6
SP	GVE XXI PR	1346	PRESIDENT	354140	1	04/07/202	18/07/202		2	6
PR	02RS METF	1356	CAMPINA (	410400	1	30/06/202	06/07/202		2	6
RJ			RIO DE JAN	330455	1	13/08/2021			1	

PE	001	1497	RECIFE	261160	1	23/03/2021		2	
RS	002 CRS	1607	PORTO ALE	431490	1	09/04/202	26/04/202	2	3
RJ			MARICA	330270	1	03/06/202	04/06/202	2	
RO	VI GRS POF	1483	PORTO VEL	110020	1	08/02/2021		2	6
BA	NUCLEO RE	1380	SALVADOR	292740	1	09/04/202	11/04/202	3	
SP	GVE XXIX S	1354	SAO JOSE D	354980	1	29/04/202	12/06/202	1	4
SP	GVE I CAPIT	1331	SAO PAULO	355030	1	12/04/202	31/05/202	1	
PR	02RS METF	1356	CURITIBA	410690	1	01/05/202	13/05/202	2	6
SP	GVE XXIX S	1354	SAO JOSE D	354980	1	14/04/202	28/04/202	1	4
SP	GVE XXIX S	1354	SAO JOSE D	354980	1	13/06/202	24/06/202	1	6
SP	GVE I CAPIT	1331	SAO PAULO	355030	1	03/06/2021		1	
CE	11 CRES SC	1521	SOBRAL	231290	1	18/06/202	08/07/202	1	5
MG	MONTES C	1473	JANAUBA	313510	1	10/04/202	10/04/202	1	9
RJ			DUQUE DE	330170	1			3	6
SP	GVE XXIV R	1348	RIBEIRAO P	354340	1	31/05/202	14/06/202	1	5
MG	MONTES C	1473	JANAUBA	313510	1	08/05/202	18/05/202	2	6
SP	GVE I CAPIT	1331	SAO PAULO	355030	1	13/05/202	15/05/202	2	
GO	CENTRAL	1779	GOIANIA	520870	1	16/01/202	18/01/202	2	
SP	GVE XXX JA	1574	JALES	352480	1	12/07/2021		1	2
RN	VII URSAP	1416	NATAL	240810	1	23/05/202	04/06/202	1	2
PA	01 REGION	1484	BELEM	150140	1	29/03/2021		1	6
DF			BRASILIA	530010	1	05/04/202	13/04/202	1	6
CE	1 CRES FOF	1519	FORTALEZA	230440	1	18/05/202	21/05/202	3	1
GO			GOIANIA	520870	1	25/05/202	03/06/202	2	
PR	03RS PONT	1357	PONTA GR	411990	1	13/06/2021		3	
SP	GVE XXIX S	1354	CATANDUV	351110	1	29/03/202	20/04/202	1	5
BA	NUCLEO RE	1380	SALVADOR	292740	1	01/03/202	04/03/202	2	
MS			DOURADOS	500370	1	11/05/2021		2	
SP	GVE I CAPIT	1331	SAO PAULO	355030	1	04/06/202	10/06/202	2	
SP	GVE I CAPIT	1331	SAO PAULO	355030	1	14/04/202	22/04/202	2	2
SP	GVE I CAPIT	1331	SAO PAULO	355030	1			3	
CE	1 CRES FOF	1519	FORTALEZA	230440	1	30/03/202	22/04/202	1	
PR	10RS CASC	1364	CASCAVEL	410480	1	19/09/202	30/09/202	2	
CE	1 CRES FOF	1519	FORTALEZA	230440	1	21/03/202	22/03/202	3	2
AM	ENTORNO	5584	MANAUS	130260	1	17/05/202	24/06/202	2	6
SP	GVE XXXI S	1353	SOROCABA	355220	1	09/06/202	15/06/202	2	6
SP	GVE XVII CA	1342	CAMPINAS	350950	1	28/09/2021		1	
SP	GVE X OSA	1335	OSASCO	353440	1	18/03/202	18/04/202	2	2
GO	CENTRAL	1779	GOIANIA	520870	1	11/08/202	22/08/202	1	
DF			BRASILIA	530010	1	09/03/202	17/03/202	1	
DF			BRASILIA	530010	1	10/04/202	21/04/202	2	6
SP	GVE XXVII S	1351	SAO JOSE D	354990	1	14/01/2021		2	6
MT	CUIABA	1578	CUIABA	510340	1	03/05/202	12/05/202	2	
AL	9 MICRORE	1541	PALMEIRA	270630	1	12/06/2021		2	3
SP	GVE XXXI S	1353	SOROCABA	355220	1	21/05/202	04/06/202	1	6
GO	CENTRAL	1779	GOIANIA	520870	1	22/11/202	25/11/202	2	
BA	NUCLEO RE	1380	SALVADOR	292740	1	05/07/202	06/07/202	2	9

MS	EX ARS DE	1975	CAMPO GF	500270	1	06/05/202	15/05/202	1	4
MT	CUIABA	1578	CUIABA	510340	1	07/08/202	18/08/202	1	
PR	01RS PARA	1355	PARANAGU	411820	1	02/06/202	01/07/202	1	6
MA	REGIONAL	1440	IMPERATRI	210530	1	28/11/2021		2	
MT	CUIABA	1578	CUIABA	510340	1	23/08/202	25/08/202	2	
RJ			VOLTA RED	330630	1	17/02/2021		2	9
PR	03RS PONT	1357	PONTA GR	411990	1	20/02/202	08/03/202	1	
GO	CENTRAL	1779	GOIANIA	520870	1	29/06/202	05/08/202	1	
SP	GVE I CAPI	1331	SAO PAUL	355030	1	05/03/202	28/03/202	1	2
MG	MONTES C	1473	MONTES C	314330	1	22/05/2021		1	
PR	02RS METF	1356	CAMPO LA	410420	1	03/10/202	15/10/202	2	6
MA	REGI0NAL	1430	SAO LUIS	211130	1	01/04/202	22/04/202	1	2
ES	METROPOI	1510	SERRA	320500	1	23/05/2021		1	2
SP	GVE I CAPI	1331	SAO PAUL	355030	1	21/01/202	25/01/202	2	1
SP	GVE XVI BC	1341	BOTUCATU	350750	1	07/05/202	11/06/202	1	4
PA	09 REGION	1492	SANTAREM	150680	1	26/05/2021		2	2
SP	GVE XXIV F	1348	RIBEIRAO F	354340	1	07/04/202	10/04/202	2	4
MT	CUIABA	1578	CUIABA	510340	1	18/01/2021		2	
RS	002 CRS	1607	PORTO ALE	431490	1	30/05/202	31/05/202	1	4
CE	1 CRES FOF	1519	FORTALEZ/	230440	1	13/04/202	28/04/202	1	2
PE	001	1497	RECIFE	261160	1	14/05/2021		2	2
MS	EX ARS DE	1975	CAMPO GF	500270	1	16/04/2021		1	5
CE	1 CRES FOF	1519	FORTALEZ/	230440	1	27/05/202	17/06/202	1	3
MG	UBA	1464	MURIAE	314390	1	01/06/2021		2	6
PI	4 GRS TERE	1883	TERESINA	221100	1	18/08/202	24/08/202	2	4
PR	02RS METF	1356	CAMPINA (	410400	1	17/09/202	24/09/202	2	6
RJ			CAMPOS D	330100	1	14/05/202	23/05/202	2	
RS	004 CRS	1595	SANTA MA	431690	1	19/05/202	21/05/202	2	
TO			PALMAS	172100	1	26/06/202	01/07/202	2	6
SP	GVE VIII M	1333	MOGI DAS	353060	1	13/06/2021		2	
SC			CHAPECO	420420	1	19/01/2021		2	6
MT	CUIABA	1578	CUIABA	510340	1	28/02/202	08/03/202	1	6
AM	ENTORNO	5584	MANAUS	130260	1	07/03/202	20/03/202	1	6
RJ			RIO DE JAN	330455	1	27/03/202	06/04/202	1	
SP	GVE XVIII F	1343	FRANCA	351620	1	09/05/202	27/05/202	1	
SP	GVE XXXI S	1353	SOROCABA	355220	1	20/05/202	30/05/202	2	
CE	1 CRES FOF	1519	FORTALEZ/	230440	1	24/06/202	29/06/202	1	1
SP	GVE X OSA	1335	CARAPICUI	351060	1	28/05/202	02/06/202	1	
SP	GVE VIII M	1333	GUARULH(	351880	1	28/04/202	07/05/202	1	4
SP	GVE I CAPI	1331	SAO PAUL	355030	1	17/08/202	20/08/202	3	
PR	09RS FOZ D	1363	FOZ DO IGU	410830	1	14/08/202	21/08/202	2	
RJ			CAMPOS D	330100	1	13/07/2021		3	1
GO	CENTRAL	1779	GOIANIA	520870	1	13/03/202	15/03/202	3	6
RJ			RIO DE JAN	330455	1	15/05/202	17/05/202	2	
PE	001	1497	RECIFE	261160	1	15/03/2021		2	
CE	15 CRES CF	1525	CRATEUS	230410	1	19/03/2021		1	
MA	REGI0NAL	1430	SAO LUIS	211130	1	28/04/202	01/05/202	3	6

GO	CENTRAL	1779	GOIANIA	520870	1			2	
PE	001	1497	RECIFE	261160	1	15/06/202	17/06/202	9	2
CE	1 CRES FOF	1519	FORTALEZ/	230440	1	04/03/202	27/03/202	1	
GO	CENTRAL	1779	GOIANIA	520870	1	22/05/202	10/06/202	1	
RJ			CAMPOS D	330100	1	05/09/202	11/09/202	2	
BA	NUCLEO RI	1380	SALVADOR	292740	1	26/07/202	28/07/202	2	
CE	1 CRES FOF	1519	FORTALEZ/	230440	1	15/11/2021		2	2
PR	02RS METF	1356	CURITIBA	410690	1	26/05/202	27/05/202	1	6
SP	GVE XXVIII	1576	CARAGUAT	351050	1	30/11/2021		3	
SC	JOINVILLE	1565	JOINVILLE	420910	1	02/10/202	15/10/202	1	6
RJ			VOLTA RED	330630	1	13/04/2021			
SP	GVE I CAPI	1331	SAO PAULC	355030	1	15/04/202	17/04/202	2	
CE			FORTALEZ/	230440	1	25/04/202	11/05/202	1	
SP	GVE XXIX S	1354	SAO JOSE D	354980	1	28/03/202	23/04/202	2	4
RS	019 CRS	1606	TENENTE P	432140	1	05/06/202	15/06/202	2	6
ES	METROPOI	1510	SERRA	320500	1	10/03/2021		2	2
SP	GVE XXIX S	1354	CATANDU\	351110	1	26/06/202	29/06/202	3	1
ES	METROPOI	1510	SERRA	320500	1	01/05/2021		1	
PR	17RS LOND	1371	LONDRINA	411370	1	12/06/202	14/06/202	1	6
DF			BRASILIA	530010	1	03/06/202	30/06/202	1	6
SP	GVE XXVI S	1350	MOGI GUA	353070	1	27/05/202	29/05/202	2	9
MS	NRS DE TRI	6255	TRES LAGO	500830	1	07/01/2021		2	
MG	CORONEL I	1468	TIMOTEO	316870	1	02/03/202	12/03/202	1	6
SP	GVE I CAPI	1331	SAO PAULC	355030	1	26/02/202	01/03/202	9	6
MG	ALFENAS	1379	ALFENAS	310160	1	02/03/202	04/03/202	2	6
MT	CUIABA	1578	CUIABA	510340	1	15/03/202	23/03/202	2	
SP	GVE VIII M	1333	MOGI DAS	353060	1	25/03/202	29/03/202	3	2
SP	GVE I CAPI	1331	SAO PAULC	355030	1	25/04/202	28/04/2021		6
RN	VII URSAP	1416	NATAL	240810	1	07/05/202	18/05/202	1	
MT	CUIABA	1578	CUIABA	510340	1	29/04/202	10/05/202	2	
MG	BELO HORI	1449	BELO HORI	310620	1	23/04/202	23/04/202	1	5
SP	GVE XI ARA	1336	PENAPOLIS	353730	1	26/10/2021		2	6
PA	01 REGION	1484	BELEM	150140	1	07/07/2021		2	6
CE	1 CRES FOF	1519	FORTALEZ/	230440	1	25/03/202	06/05/202	1	1
MT	CUIABA	1578	CUIABA	510340	1	30/03/202	12/04/202	2	
SP	GVE I CAPI	1331	SAO PAULC	355030	1	18/03/202	25/03/202	2	3
RJ			DUQUE DE	330170	1			3	6
AM	ENTORNO	5584	MANAUS	130260	1	03/03/202	05/03/202	2	
PA	01 REGION	1484	BELEM	150140	1	16/06/2021		2	2
GO	CENTRAL	1779	GOIANIA	520870	1	07/07/202	16/07/202	2	
SP	GVE I CAPI	1331	SAO PAULC	355030	1	27/05/202	30/05/202	2	
SP	GVE I CAPI	1331	SAO PAULC	355030	1	21/09/202	21/09/202	3	
SP	GVE VIII M	1333	GUARULHC	351880	1	05/01/202	08/01/202	2	2
SP	GVE XXIX S	1354	SAO JOSE D	354980	1	09/03/202	18/03/202	1	4
SP	GVE XXIX S	1354	VOTUPOR/	355710	1	21/03/202	02/04/202	1	5
RJ			RIO DE JAN	330455	1	29/03/2021		1	
SP	GVE XXI PR	1346	PRESIDENT	354140	1	24/08/202	21/09/202	1	6

MG	SETE LAGO	1467	SETE LAGO	316720	1	15/04/202	21/05/202	1	5
PB	I NRS JOAC	1377	JOAO PESS	250750	1	10/05/2021		3	6
PR	17RS LOND	1371	LONDRINA	411370	1	17/06/202	02/08/202	1	5
PB	III NRS CAN	1421	CAMPINA (	250400	1	27/03/2021		2	6
RJ			RIO DE JAN	330455	1	15/05/2021		1	
SP	GVE XXV SA	1349	GUARUJA	351870	1	11/05/2021		1	
BA	NUCLEO RI	1380	SANTO AN	292870	1	15/04/2021		1	
RS	001 CRS	1592	SAO LEOPC	431870	1	19/03/202	05/04/202	1	6
PI	4 GRS TERE	1883	TERESINA	221100	1	15/01/202	20/01/202	3	6
AC	REGIONAL	1941	CRUZEIRO	120020	1	12/04/2021		3	6
SP	GVE I CAPI	1331	SAO PAUL(	355030	1	04/01/202	13/01/2022		
RS	002 CRS	1607	PORTO ALE	431490	1	01/06/202	06/06/202	2	
SP	GVE I CAPI	1331	SAO PAUL(	355030	1	03/06/202	08/06/202	2	2
SP	GVE I CAPI	1331	SAO PAUL(	355030	1	12/04/202	20/04/202	2	3
SP	GVE I CAPI	1331	SAO PAUL(	355030	1	17/03/2021		2	2
SP	GVE XXIX S	1354	CATANDU\	351110	1	17/07/202	23/07/202	2	6
MA	REGI0NAL	1430	SAO LUIS	211130	1	01/06/2021		1	2
SP	GVE I CAPI	1331	SAO PAUL(	355030	1			2	6
PB	I NRS JOAC	1377	JOAO PESS	250750	1	20/05/2021		1	
BA			SALVADOR	292740	1	25/05/202	26/05/2021		
RO	VI GRS POF	1483	PORTO VEL	110020	1	30/07/202	18/08/202	1	6
SP	GVE I CAPI	1331	SAO PAUL(	355030	1	08/02/202	15/02/202	1	9
PR	02RS METF	1356	CURITIBA	410690	1	28/12/202	29/12/202	3	6
SP	GVE I CAPI	1331	SAO PAUL(	355030	1	05/03/202	11/03/202	3	6
MT	CUIABA	1578	CUIABA	510340	1	17/03/202	07/04/202	2	
MG	MONTES C	1473	MONTES C	314330	1	20/03/2021		1	9
RN	VII URSAP	1416	NATAL	240810	1	29/05/2021		3	6
CE	1 CRES FOF	1519	FORTALEZ/	230440	1	08/03/2021		3	
MG	PONTE NO	1458	PONTE NO	315210	1	24/03/202	16/04/202	1	5
MT	SINOP	1584	SINOP	510790	1	22/04/202	09/05/202	1	9
GO	CENTRAL	1779	GOIANIA	520870	1	20/03/202	24/03/202	2	
BA	NUCLEO RI	1398	GUANAMB	291170	1	27/08/202	16/09/202	2	6
MG	POUSO ALI	1459	POUSO ALI	315250	1	13/03/2021		1	6
SP	GVE I CAPI	1331	SAO PAUL(	355030	1	02/06/202	18/06/202	1	
MS	EX ARS DE	1975	CAMPO GF	500270	1	12/07/202	04/08/202	1	3
RJ			RIO DE JAN	330455	1	18/01/202	19/01/202	2	9
PR	10RS CASC	1364	CASCAVEL	410480	1	18/06/202	21/06/202	3	
PR	10RS CASC	1364	CASCAVEL	410480	1	25/07/202	07/08/202	2	
SP	GVE I CAPI	1331	SAO PAUL(	355030	1	31/03/202	04/04/202	2	4
SP	GVE XXVII S	1351	JACAREI	352440	1	03/04/202	05/04/202	2	2
PB	I NRS JOAC	1377	JOAO PESS	250750	1	27/05/2021		2	6
SP	GVE I CAPI	1331	SAO PAUL(	355030	1	05/03/202	11/03/202	2	
SP	GVE X OSA	1335	OSASCO	353440	1	26/06/202	15/07/202	2	
SP	GVE XVII C/	1342	VALINHOS	355620	1	14/06/2021		1	9
SP			SAO PAUL(	355030	1	27/03/202	01/04/202	3	
SP	GVE I CAPI	1331	SAO PAUL(	355030	1	03/05/202	13/05/202	2	6
SP			ITAQUAQU	352310	1	06/05/2021		2	

PR	10RS CASC	1364	CASCAVEL	410480	1	06/09/202	09/09/202	3	
GO	CENTRAL	1779	GOIANIA	520870	1	07/08/202	09/08/2021		
AM	ENTORNO	5584	MANAUS	130260	1	15/01/202	20/01/202	2	2
PR	15RS MARI	1369	MARINGA	411520	1	15/02/202	01/03/202	3	6
PB	I NRS JOAC	1377	JOAO PESS	250750	1	19/02/202	22/02/202	1	6
MG	POUSO ALI	1459	EXTREMA	312510	1	03/03/202	15/03/202	1	
RJ			RIO DE JAN	330455	1	25/02/202	06/03/202	1	6
CE	1 CRES FOF	1519	FORTALEZ/	230440	1	23/02/202	29/03/202	1	
MG	JUIZ DE FO	1452	JUIZ DE FO	313670	1	24/03/202	02/04/202	1	5
SP	GVE XIV BA	1339	BARRETOS	350550	1			1	6
SP	GVE VIII M	1333	ITAQUAQU	352310	1	08/07/2021		2	
SC	BLUMENAU	1564	GASPAR	420590	1	26/05/2021		1	6
SP	GVE XII AR/	1337	ARARAQU/	350320	1	02/07/202	12/07/202	1	
SP	GVE VII SAI	1332	RIBEIRAO F	354330	1	06/06/202	25/06/202	1	6
RJ			ANGRA DO	330010	1	10/08/2021		2	6
SE	REGIONAL	2056	ARACAJU	280030	1	04/03/202	12/03/202	2	
RJ			RIO DE JAN	330455	1	05/10/2021		2	6
BA	NUCLEO RI	1381	FEIRA DE S.	291080	1	25/04/2021		2	6
SP	GVE XVII C.	1342	CAMPINAS	350950	1	11/03/202	23/03/202	1	9
RJ			VOLTA RED	330630	1	08/05/2021		1	5
PR	14RS PARA	1368	PARANAV/	411840	1	10/06/202	12/06/202	2	6
MA	REGI0NAL	1430	SAO LUIS	211130	1	03/06/202	08/06/202	2	2
PR	17RS LOND	1371	LONDRINA	411370	1	04/06/202	22/06/202	2	6
SP			SAO PAULC	355030	1	26/03/202	03/04/202	2	
PR	02RS METF	1356	CAMPINA (	410400	1	10/06/202	15/06/202	2	
RJ			NILOPOLIS	330320	1	10/05/202	12/05/202	1	2
MT	CUIABA	1578	CUIABA	510340	1	17/03/202	18/04/202	1	9
SP	GVE XXIX S	1354	SAO JOSE D	354980	1	16/02/202	08/03/202	2	2
SP	GVE XI ARA	1336	PENAPOLIS	353730	1	27/10/202	28/10/202	3	6
PB	I NRS JOAC	1377	JOAO PESS	250750	1	17/01/202	01/02/202	1	6
MG	BELO HORI	1449	CONTAGEN	311860	1	26/04/202	02/05/202	3	6
SC	JOINVILLE	1565	JOINVILLE	420910	1	24/03/202	29/03/202	2	6
SP	GVE XXV S/	1349	SANTOS	354850	1	19/03/2021			
SP	GVE I CAPI	1331	SAO PAULC	355030	1	24/03/202	27/04/202	2	2
SC	JARAGUA I	1567	JARAGUA I	420890	1	23/08/202	15/10/202	1	5
SP	GVE I CAPI	1331	SAO PAULC	355030	1	10/04/202	20/04/202	9	
PA	01 REGION	1484	BELEM	150140	1	12/04/202	21/04/202	2	6
RJ			RIO DE JAN	330455	1	10/08/202	14/08/2021		
RO	VI GRS POF	1483	PORTO VEL	110020	1	18/03/202	26/03/202	1	6
MG	BELO HORI	1449	BETIM	310670	1	28/01/2021		3	6
PR	02RS METF	1356	CURITIBA	410690	1	23/02/202	12/03/202	1	5
SP	GVE I CAPI	1331	SAO PAULC	355030	1	03/01/2022		3	6
GO			GOIANIA	520870	1	22/09/202	29/09/202	2	
SP	GVE XIX M.	1344	MARILIA	352900	1	16/03/202	29/03/202	2	6
SP	GVE XXXI S	1353	SOROCABA	355220	1	20/05/202	01/06/202	2	2
SP	GVE I CAPI	1331	SAO PAULC	355030	1	08/04/202	22/04/202	2	9
GO	CENTRAL	1779	GOIANIA	520870	1	07/02/2021		3	

PI	10 GRS FLC	1889	FLORIANO	220390	1	05/05/202	15/05/202	9		
GO	CENTRAL	1779	GOIANIA	520870	1	30/04/2021		2		9
RJ			ITAPERUN/	330220	1	11/08/202	13/08/202	9		6
SP			SAO PAULC	355030	1	25/08/202	30/08/202	2		
SP	GVE XIX M/	1344	MARILIA	352900	1	21/03/202	27/03/202	2		2
SP	GVE I CAPI	1331	SAO PAULC	355030	1	25/06/202	28/06/202	3		
SP			GUARULHC	351880	1			3		
PB	I NRS JOAC	1377	JOAO PESS	250750	1	10/04/2021		1		6
CE	1 CRES FOF	1519	FORTALEZ/	230440	1	29/01/2021		1		
CE	1 CRES FOF	1519	FORTALEZ/	230440	1	23/03/202	13/04/202	1		
SP	GVE I CAPI	1331	SAO PAULC	355030	1	20/04/202	25/04/202	1		2
RJ			RIO DE JAN	330455	1	14/03/202	21/03/202	2		6
SP	GVE XX PIR	1345	ARARAS	350330	1	17/12/2021		3		
RS	002 CRS	1607	PORTO ALE	431490	1	01/05/202	20/05/202	1		
RS	001 CRS	1592	NOVO HAN	431340	1	21/05/2021		2		
MA	REGIONAL	1440	IMPERATRI	210530	1	24/05/2021		2		
SP	GVE I CAPI	1331	SAO PAULC	355030	1	27/03/202	06/04/202	2		2
BA	NUCLEO RI	1380	SALVADOR	292740	1	30/01/202	30/01/202	1		6
DF			BRASILIA	530010	1	11/04/202	27/07/202	1		6
SP	GVE I CAPI	1331	SAO PAULC	355030	1	11/06/202	14/07/202	1		9
SP			SAO PAULC	355030	1	17/05/202	24/05/202	1		
RR			BOA VISTA	140010	1	19/07/202	24/07/202	1		
BA	NUCLEO RI	1380	SALVADOR	292740	1	19/03/202	29/03/202	2		9
SP	GVE I CAPI	1331	SAO PAULC	355030	1	26/03/202	29/03/202	2		
RS	001 CRS	1592	PAROBE	431405	1	01/06/202	06/06/202	2		
MG	DIAMANTI	1450	DIAMANTI	312160	1		07/06/202	2		9
PI	4 GRS TERE	1883	TERESINA	221100	1	12/05/202	21/05/202	1		2
SP	GVE I CAPI	1331	SAO PAULC	355030	1	05/08/202	29/09/202	1		
PE	001	1497	RECIFE	261160	1	31/01/2021		1		
SP	GVE XXI PR	1346	PRESIDENT	354140	1	17/05/202	18/05/202	1		6
MG	DIVINOPOI	1472	DIVINOPOI	312230	1	09/06/2021		2		
MA	REGI0NAL	1430	SAO LUIS	211130	1	12/06/202	16/08/202	2		
SP	GVE XVI BC	1341	LARANJAL	352640	1	30/04/202	24/05/202	2		6
SP	GVE XX PIR	1345	PIRACICAB	353870	1	13/06/202	20/06/202	1		
AM	ENTORNO	5584	MANAUS	130260	1	11/02/202	26/02/202	1		6
GO	CENTRAL	1779	GOIANIA	520870	1	24/02/2021		2		4
SP	GVE I CAPI	1331	SAO PAULC	355030	1	24/09/202	27/09/2021			
SP	GVE XXI PR	1346	PRESIDENT	354140	1	18/05/202	20/05/202	1		6
RN	VII URSAP	1416	NATAL	240810	1	10/05/202	15/05/202	1		6
RJ			CAMPOS D	330100	1	01/09/202	10/09/202	3		
MG	BELO HORI	1449	BELO HORI	310620	1	26/09/202	30/09/202	3		3
PR	10RS CASC	1364	CASCAVEL	410480	1	27/06/202	13/07/202	2		
MG	BELO HORI	1449	NOVA LIM/	314480	1	09/06/2021		2		9
MG	BELO HORI	1449	CONTAGEN	311860	1	09/08/2021		9		9
PR	20RS TOLEI	1374	TOLEDO	412770	1	12/03/202	07/05/202	2		9
SP	GVE XVII C/	1342	ATIBAIA	350410	1	05/02/202	07/02/202	2		6
GO	ESTRADA D	1802	MARZAGA(	521290	1			2		6

RJ			RIO DE JAN	330455	1	13/03/2021		1	2
MG	UBERLAND	1462	UBERLAND	317020	1	29/04/2021		1	6
SP	GVE XXXI S	1353	SOROCABA	355220	1	28/09/202	05/10/202	1	6
CE	1 CRES FOF	1519	FORTALEZA	230440	1	04/05/202	05/05/202	3	6
SP	GVE I CAPI	1331	SAO PAULC	355030	1	27/05/202	19/06/202	1	
SP	GVE I CAPI	1331	SAO PAULC	355030	1	22/05/202	24/05/202	2	2
GO	CENTRAL	1779	GOIANIA	520870	1	20/07/202	29/07/202	1	
SP	GVE I CAPI	1331	SAO PAULC	355030	1	13/06/202	29/06/202	2	3
SP	GVE I CAPI	1331	SAO PAULC	355030	1	01/04/202	15/04/202	2	4
PR			LONDRINA	411370	1	12/07/202	15/07/202	2	5
PR	10RS CASC	1364	CASCAVEL	410480	1	01/05/202	14/05/202	2	
PR	02RS METF	1356	CURITIBA	410690	1	30/11/202	02/12/202	2	6
SP	GVE XI ARA	1336	PENAPOLIS	353730	1	25/07/202	02/08/202	1	6
MT	CUIABA	1578	CUIABA	510340	1	25/01/202	17/02/202	1	
GO	CENTRAL	1779	GOIANIA	520870	1	02/10/202	03/11/202	1	
SP	GVE I CAPI	1331	SAO PAULC	355030	1	08/03/202	18/03/202	1	3
PE	001	1497	RECIFE	261160	1	05/05/2021		1	6
MS	NRS DE DO	1966	DOURADOS	500370	1	13/03/2021		1	
RJ			VALENCA	330610	1	16/05/2021		2	2
SP	GVE I CAPI	1331	SAO PAULC	355030	1	17/03/202	21/03/202	2	2
MA	REGIONAL	1440	IMPERATRI	210530	1			2	6
CE	1 CRES FOF	1519	FORTALEZA	230440	1	09/04/2021		1	
SP	GVE XXIX S	1354	SAO JOSE D	354980	1	07/04/202	13/04/202	2	4
GO	CENTRAL	1779	GOIANIA	520870	1	30/04/202	19/05/202	2	
SP	GVE VIII M	1333	GUARULHC	351880	1	27/03/202	31/03/202	2	2
SP			OSASCO	353440	1	23/03/2021		2	2
SP	GVE I CAPI	1331	SAO PAULC	355030	1	23/03/202	02/04/202	1	2
MG	PATOS DE I	1456	PATOS DE I	314800	1	22/02/202	17/03/202	1	
PB	I NRS JOAC	1377	JOAO PESS	250750	1	21/03/202	22/03/202	1	6
RS	016 CRS	1610	ESTRELA	430780	1	29/05/202	03/06/202	2	
PR	09RS FOZ D	1363	FOZ DO IGU	410830	1	28/07/202	07/08/202	2	6
AM	ENTORNO	5584	MANAUS	130260	1	29/03/202	26/05/202	1	6
SP	GVE I CAPI	1331	SAO PAULC	355030	1	30/03/2021			4
SP	GVE XII AR	1337	SAO CARLC	354890	1	15/04/202	06/05/202	1	4
SP	GVE VII SA	1332	SAO CAETA	354880	1	31/03/202	28/04/202	2	6
GO	SUDOESTE	1799	RIO VERDE	521880	1	03/04/202	11/04/202	1	
AM	ENTORNO	5584	MANAUS	130260	1	06/02/202	10/02/202	1	2
PR	02RS METF	1356	CURITIBA	410690	1	03/03/202	02/04/202	1	6
PR	10RS CASC	1364	CASCAVEL	410480	1	24/09/202	27/09/202	3	
SP	GVE XXII PI	1573	DRACENA	351440	1	02/07/202	04/07/202	3	9
SP	GVE I CAPI	1331	SAO PAULC	355030	1	23/04/202	03/05/202	3	
BA	NUCLEO RI	1388	PORTO SEG	292530	1	15/06/202	06/07/202	1	4
MT	JUINA	1582	ARIPUANA	510140	1	19/07/2021		2	2
AM	ENTORNO	5584	MANAUS	130260	1	26/01/202	01/02/202	2	
RS	002 CRS	1607	PORTO ALE	431490	1	28/12/202	02/01/202	2	
SP	GVE I CAPI	1331	SAO PAULC	355030	1	03/04/2021		1	
SP	GVE I CAPI	1331	SAO PAULC	355030	1	14/04/202	20/04/202	2	6

PB	III NRS CAM	1421	CAMPINA (	250400	1	12/06/2021		1	6
DF			BRASILIA	530010	1	10/03/202	08/04/202	1	6
SP	GVE XXXI S	1353	SALTO DE F	354530	1	26/05/202	01/06/202	1	2
AM			MANAUS	130260	1			1	
SP	GVE I CAPI	1331	SAO PAULC	355030	1	12/06/202	20/07/202	1	3
PR	19RS JACA	1373	SANTO AN	412410	1	26/11/202	29/11/202	2	9
SP	GVE XXXI S	1353	SALTO	354520	1	20/05/202	28/05/202	2	9
PA	01 REGION	1484	BELEM	150140	1	13/03/2021		3	6
SP	GVE XXIX S	1354	SAO JOSE D	354980	1	12/04/202	13/04/202	2	6
SP	GVE XV BA	1340	BAURU	350600	1	28/06/202	02/07/202	2	9
MA	REGI0NAL	1430	SAO LUIS	211130	1	16/03/202	17/03/202	2	1
SP	GVE XIX M	1344	MARILIA	352900	1	11/06/202	25/06/202	1	6
GO	PIRINEUS	1792	ANAPOLIS	520110	1	06/03/2021		1	
PA	01 REGION	1484	BELEM	150140	1	15/03/202	19/03/202	2	2
SP	GVE XXVI S	1350	MOGI GUA	353070	1	15/05/202	24/05/202	1	2
MT	CUIABA	1578	CUIABA	510340	1	25/06/202	05/07/2021		
RN	VII URSAP	1416	NATAL	240810	1	20/05/2021		1	
SP	GVE XXIX S	1354	SAO JOSE D	354980	1	16/07/202	12/08/202	2	2
SP	GVE VIII M	1333	MOGI DAS	353060	1	21/03/202	23/03/202	3	9
GO	CENTRAL	1779	GOIANIA	520870	1	31/05/202	04/06/202	2	
MA					1	19/05/2021		2	
SP	GVE XXIX S	1354	SAO JOSE D	354980	1	09/06/202	05/07/202	1	4
SP	GVE I CAPI	1331	SAO PAULC	355030	1	22/01/202	25/01/202	2	
SP	GVE I CAPI	1331	SAO PAULC	355030	1	22/03/202	26/03/202	2	9
GO	CENTRAL	1779	GOIANIA	520870	1	27/03/202	26/04/202	1	
SP	GVE I CAPI	1331	SAO PAULC	355030	1	09/05/202	19/05/202	1	2
SP	GVE XVII C	1342	CAMPINAS	350950	1	21/06/202	26/06/202	2	
BA	NUCLEO RI	1380	SALVADOR	292740	1			2	
TO			PORTO NA	171820	1	12/01/202	12/01/202	1	6
SP	GVE XVII C	1342	AMERICAN	350160	1	20/02/202	19/03/202	1	6
SP	GVE XXXI S	1353	SOROCABA	355220	1	03/05/202	12/05/202	1	9
SP	GVE XI ARA	1336	VALPARAIS	355630	1	16/05/202	21/05/202	1	
PR	02RS METF	1356	CURITIBA	410690	1	28/04/202	06/05/202	2	6
SP	GVE I CAPI	1331	SAO PAULC	355030	1	21/06/202	22/06/202	2	9
CE	11 CRES SC	1521	SOBRAL	231290	1	11/07/202	17/07/202	2	6
SC	JOINVILLE	1565	JOINVILLE	420910	1	14/07/202	18/07/202	3	6
PR	09RS FOZ D	1363	FOZ DO IGU	410830	1	20/07/202	29/07/202	2	
PR	20RS TOLEI	1374	MARECHAI	411460	1	14/09/202	01/10/2021		9
SP	GVE XXV S	1349	GUARUJA	351870	1	19/06/2021		1	
RJ			RIO DE JAN	330455	1	26/03/202	28/03/202	2	6
SP			SAO PAULC	355030	1	15/05/202	21/05/202	2	
SP	GVE I CAPI	1331	SAO PAULC	355030	1	03/06/202	09/06/202	2	3
RS	003 CRS	1593	RIO GRANI	431560	1	02/07/202	19/07/202	1	6
SP	GVE I CAPI	1331	SAO PAULC	355030	1	06/06/202	16/06/202	2	
BA	NUCLEO RI	1380	SALVADOR	292740	1	04/06/202	17/06/202	1	
PE	001	1497	RECIFE	261160	1	13/04/2021		2	5
SP	GVE VII SA	1332	MAUA	352940	1	31/07/202	01/08/202	1	2

CE	1 CRES FOF	1519	FORTALEZ/	230440	1	03/04/202 19/04/202	1	
MG	BELO HORI	1449	BELO HORI	310620	1	12/03/2021	1	2
RJ			RIO DE JAN	330455	1	06/03/2021	1	2
SC	RIO DO SUI	1478	RIO DO SUI	421480	1	02/03/2021	2	
RO	VI GRS POF	1483	PORTO VEL	110020	1	24/04/2021	2	
MG	BELO HORI	1449	BETIM	310670	1	04/05/2021		
PR	10RS CASC	1364	CASCAVEL	410480	1	11/10/202 04/11/202	1	
RN	II URSAP	1412	MOSSORO	240800	1	27/12/202 30/12/202	2	3
PR	17RS LOND	1371	LONDRINA	411370	1	05/03/202 10/03/202	2	6
SC			CHAPECO	420420	1	21/03/2021	1	6
SC	CRICIUMA	1558	CRICIUMA	420460	1	31/03/202 20/04/202	1	3
RS	004 CRS	1595	SANTA MA	431690	1	10/05/202 19/05/202	1	
SP	GVE XIII AS	1338	SANTA CRU	354640	1			
GO	CENTRAL	1779	GOIANIA	520870	1	29/06/202 30/06/202	2	
PR	10RS CASC	1364	CASCAVEL	410480	1	07/06/202 14/06/202	2	
MT	CUIABA	1578	CUIABA	510340	1	28/07/202 19/08/202	1	
CE	1 CRES FOF	1519	FORTALEZ/	230440	1	20/05/202 21/05/202	1	1
MG	UNAI	1463	PARACATU	314700	1	12/09/202 14/09/202	3	
RJ			RIO DE JAN	330455	1	01/12/2021	2	6
MG	MONTES C	1473	MONTES C	314330	1	16/01/2021	2	6
SP	GVE I CAPI	1331	SAO PAULO	355030	1	03/04/202 20/04/202	1	6
MS	EX ARS DE	1975	CAMPO GF	500270	1	18/04/2021	2	
SP	GVE VII SAI	1332	SANTO AN	354780	1	14/06/2021	2	6
SC	XANXERE	1556	XANXERE	421950	1	03/08/202 08/08/202	3	6
SP	GVE I CAPI	1331	SAO PAULO	355030	1	30/03/202 02/04/202	2	6
SP	GVE I CAPI	1331	SAO PAULO	355030	1	26/03/2021	1	2
PB	III NRS CAN	1421	CAMPINA (	250400	1	02/05/202 04/05/202	1	6
PR	02RS METF	1356	CURITIBA	410690	1	05/05/202 09/05/202	2	6
GO	CENTRAL	1779	GOIANIA	520870	1	04/04/202 07/04/202	2	
SP	GVE I CAPI	1331	SAO PAULO	355030	1	07/05/2021	1	6
MG	SETE LAGO	1467	SETE LAGO	316720	1	24/03/202 26/04/202	1	2
SP	GVE I CAPI	1331	SAO PAULO	355030	1	25/03/2021	2	2
SP	GVE XIX M/	1344	MARILIA	352900	1	04/04/202 12/04/202	2	6
GO	CENTRAL	1779	GOIANIA	520870	1	09/05/2021	2	5
SP	GVE I CAPI	1331	SAO PAULO	355030	1	12/05/2021	2	6
SP	GVE XXIX S	1354	SAO JOSE D	354980	1	16/06/202 03/07/202	1	4
CE	1 CRES FOF	1519	FORTALEZ/	230440	1	02/04/202 07/04/202	3	
MG	UBERLAND	1462	PATROCINI	314810	1	12/05/2021	2	6
SP	GVE XXXI S	1353	SALTO	354520	1	29/07/202 02/08/202	2	9
PA	12 REGION	1495	REDENCAC	150613	1	12/08/2021	2	6
SP	GVE X OSA	1335	OSASCO	353440	1	23/03/202 29/03/202	2	2
AL	1 MICRORI	1533	MACEIO	270430	1	18/03/202 24/03/202	1	6
SP	GVE XI ARA	1336	PENAPOLIS	353730	1	14/06/2021	2	6
RJ			RIO DE JAN	330455	1	28/08/2021	2	5
PR	02RS METF	1356	CURITIBA	410690	1	01/08/202 18/08/202	2	
PE	001	1497	RECIFE	261160	1	24/03/2021	2	2
DF			BRASILIA	530010	1	24/03/2021	1	

MG	PATOS DE I	1456	PATOS DE I	314800	1	12/03/2021			
SP	GVE XVII C/	1342	CAMPINAS	350950	1	09/04/2021		2	
CE	1 CRES FOF	1519	FORTALEZ/	230440	1	11/03/2021		1	
PA			ORIXIMINA	150530	1	12/06/202	14/06/202	2	2
AM	ENTORNO	5584	MANAUS	130260	1	11/06/202	28/06/202	1	6
MG	UBERLAND	1462	UBERLAND	317020	1	26/06/2021		2	6
RJ			NITEROI	330330	1	09/03/2021			
SC	JOINVILLE	1565	JOINVILLE	420910	1	04/10/202	06/10/202	2	5
PA	01 REGION	1484	BELEM	150140	1	17/03/202	23/03/202	3	5
RJ			RIO DE JAN	330455	1	15/05/2021		3	
SP	GVE XVII C/	1342	CAMPINAS	350950	1	17/06/2021		2	6
RJ			NILOPOLIS	330320	1	17/07/202	23/07/202	2	2
PR	14RS PARA	1368	PARANAV/	411840	1	23/10/202	24/10/202	2	
PR	17RS LOND	1371	LONDRINA	411370	1	27/07/202	29/07/202	3	6
PA	09 REGION	1492	SANTAREM	150680	1	29/04/2021		2	
RS	002 CRS	1607	PORTO ALE	431490	1	28/03/202	19/04/202	1	2
SC	CHAPECO	1553	CHAPECO	420420	1	01/08/2021			5
SP	GVE I CAPI	1331	SAO PAUL(	355030	1	23/03/202	26/03/202	1	
MA	REGI0NAL	1430	SAO LUIS	211130	1	25/05/202	06/06/202	2	2
MT	CUIABA	1578	CUIABA	510340	1	28/04/202	21/05/202	1	6
MG	UBERLAND	1462	UBERLAND	317020	1	05/06/2021		1	6
MA	REGI0NAL	1430	SAO LUIS	211130	1	14/05/202	16/05/202	2	9
SP	GVE XVII C/	1342	CAMPINAS	350950	1	05/03/202	08/03/202	2	
SP	GVE I CAPI	1331	SAO PAUL(	355030	1	12/03/202	24/03/202	1	6
GO	CENTRAL	1779	GOIANIA	520870	1	24/04/202	06/05/202	2	
SP	GVE XVII C/	1342	JUNDIAI	352590	1	07/06/2021		3	5
MG	BELO HORI	1449	BELO HORI	310620	1	20/05/2021		1	2
BA	NUCLEO RI	1381	FEIRA DE S/	291080	1	20/05/202	04/06/202	3	
MG	GOVERNAI	1471	GOVERNAI	312770	1	31/03/202	17/04/202	1	9
MT	CUIABA	1578	CUIABA	510340	1	11/05/202	11/06/202	1	
GO	CENTRAL	1779	GOIANIA	520870	1	14/07/202	07/08/202	1	
RN	VII URSAP	1416	NATAL	240810	1	12/03/202	20/03/202	1	9
RJ			RIO DE JAN	330455	1	13/04/2021		2	2
PA	01 REGION	1484	BELEM	150140	1	25/05/2021		2	6
BA	NUCLEO RI	1380	SALVADOR	292740	1	13/06/202	13/06/202	2	9
SP	GVE XI AR/	1336	PENAPOLIS	353730	1	03/07/2021		2	
MG	SETE LAGO	1467	SETE LAGO	316720	1	15/07/202	26/07/202	2	5
GO	CENTRAL	1779	GOIANIA	520870	1	23/02/202	28/02/202	3	
RR			BOA VISTA	140010	1	29/06/202	06/07/202	1	
SP	GVE XXIX S	1354	SAO JOSE I	354980	1	21/05/202	18/06/202	1	4
MS	NRS DE DO	1966	DOURADO:	500370	1	31/03/2021			
RJ			RIO DE JAN	330455	1	29/04/202	16/05/202	1	
PR	15RS MARI	1369	SARANDI	412625	1	06/10/202	10/10/202	2	
SP	GVE XII AR/	1337	SAO CARL(	354890	1	24/06/2021		1	6
CE	11 CRES SC	1521	SOBRAL	231290	1	12/05/202	19/05/202	1	
RR			BOA VISTA	140010	1	25/06/202	07/07/202	1	
PR	10RS CASC	1364	CASCAVEL	410480	1	27/02/202	28/02/202	3	

CE	11 CRES SC	1521	SOBRAL	231290	1	24/03/202	04/04/202	2	
MT	CUIABA	1578	CUIABA	510340	1	25/02/202	28/02/202	2	
DF			BRASILIA	530010	1	16/06/202	29/06/202	1	6
SP			SAO PAULC	355030	1	27/04/202	12/05/202	3	
PR	17RS LOND	1371	LONDRINA	411370	1	14/06/202	22/06/202	2	6
RO	VI GRS POF	1483	PORTO VEL	110020	1	10/05/202	15/05/202	3	6
MG	BELO HORI	1449	CONTAGEN	311860	1	22/07/202	26/07/202	1	2
GO	SUDOESTE	1799	RIO VERDE	521880	1	29/03/202	17/04/202	2	
CE	1 CRES FOF	1519	FORTALEZ/	230440	1	18/03/202	29/03/202	2	
BA			SALVADOR	292740	1	03/04/202	17/04/202	2	
MG	BELO HORI	1449	BETIM	310670	1	21/04/2021		1	
MG	CORONEL I	1468	CORONEL I	311940	1	01/05/2021		9	9
MG	BELO HORI	1449	BELO HORI	310620	1	19/04/202	22/04/202	1	6
SP	GVE I CAPI	1331	SAO PAULC	355030	1	20/07/202	10/08/202	2	
MG	POUSO ALI	1459	EXTREMA	312510	1	21/05/2021		2	
MA	REGIONAL	1440	IMPERATRI	210530	1	27/07/2021		2	6
SP			RIBEIRAO I	354340	1	02/06/2021		1	
BA	NUCLEO RI	1380	SALVADOR	292740	1	05/05/202	06/05/202	3	
SP	GVE I CAPI	1331	SAO PAULC	355030	1	11/06/202	17/06/202	2	
PR	05RS GUAF	1359	GUARAPU/	410940	1	11/06/202	18/06/202	1	6
SP	GVE XX PIR	1345	PIRACICAB	353870	1	27/05/2021		1	
RS	002 CRS	1607	PORTO ALE	431490	1	17/03/202	30/03/202	3	2
GO					1			1	9
RS	005 CRS	1608	CAXIAS DO	430510	1	18/03/202	10/05/202	1	2
SP	GVE XXIV I	1348	RIBEIRAO I	354340	1	29/05/202	29/06/202	1	4
MG	VARGINHA	1466	TRES PONT	316940	1	04/06/202	06/06/202	2	6
SP			ITAQUAQL	352310	1	03/06/2021		2	
SP	GVE XV BA	1340	BAURU	350600	1	02/06/202	16/06/202	2	9
MA	REGI0NAL	1430	SAO LUIS	211130	1	10/06/202	13/06/202	1	6
SC			TUBARAO	421870	1	10/10/2021		1	
GO			ANAPOLIS	520110	1	11/06/2021		2	6
MG	UBERABA	1461	UBERABA	317010	1			1	
MG	BELO HORI	1449	BETIM	310670	1	27/05/2021			
BA	NUCLEO RI	1380	SALVADOR	292740	1	26/05/202	28/05/202	3	6
SP	GVE I CAPI	1331	SAO PAULC	355030	1	27/07/202	30/07/202	2	5
SP	GVE I CAPI	1331	SAO PAULC	355030	1	19/07/202	24/07/202	2	
RS	002 CRS	1607	PORTO ALE	431490	1	01/06/202	05/07/202	1	
CE	1 CRES FOF	1519	FORTALEZ/	230440	1	07/04/202	21/05/202	1	2
MG	ALFENAS	1379	GUAXUPE	312870	1	02/06/202	30/06/202	1	6
SP	GVE XXI PR	1346	PRESIDENT	354140	1	03/04/202	05/04/202	2	6
RJ			RIO DE JAN	330455	1	24/05/202	08/07/202	1	5
MG	UBERLAND	1462	UBERLAND	317020	1	12/06/2021		2	6
SP			SOROCAB/	355220	1	10/07/202	28/07/202	3	9
RR			BOA VISTA	140010	1	05/03/202	16/04/202	2	
RJ			RIO DE JAN	330455	1	19/04/2021		1	5
RJ			RIO DE JAN	330455	1	22/04/2021		2	6
SP	GVE VIII M	1333	GUARULHC	351880	1	12/04/2021		1	

MG	BELO HORI	1449	BELO HORI	310620	1	04/05/202	07/05/202	2	2
SC	ITAJAI	1550	BALNEARIC	420200	1	05/07/2021		2	4
BA	NUCLEO RI	1380	SALVADOR	292740	1	22/07/202	23/07/202	2	9
MG	PATOS DE I	1456	PATOS DE I	314800	1	12/03/202	14/03/202	2	
GO	CENTRAL	1779	GOIANIA	520870	1	03/03/202	06/03/202	2	
PR	08RS FRAN	1362	FRANCISCC	410840	1	18/02/202	21/02/202	3	9
BA	NUCLEO RI	1380	SALVADOR	292740	1	13/04/202	17/04/202	3	
PR	10RS CASC	1364	CASCAVEL	410480	1	21/04/202	26/04/202	2	9
MG	DIAMANTI	1450	TURMALIN	316970	1	27/05/202	02/06/202	1	5
SP	GVE I CAPI	1331	SAO PAULC	355030	1	25/04/202	27/04/202	1	2
CE	1 CRES FOF	1519	FORTALEZ/	230440	1	11/05/202	21/05/202	3	5
SC	VIDEIRA	1546	CURITIBAN	420480	1	20/08/202	24/08/202	1	5
SP	GVE I CAPI	1331	SAO PAULC	355030	1	12/03/202	18/04/202	2	2
SP	GVE XXIV F	1348	RIBEIRAO F	354340	1	07/04/202	02/06/202	1	4
BA	NUCLEO RI	1382	RIO REAL	292700	1	08/06/202	08/06/202	1	2
PR	17RS LOND	1371	LONDRINA	411370	1	10/04/202	21/04/202	1	5
MG	SETE LAGO	1467	SETE LAGO	316720	1	10/05/202	14/05/202	2	9
PR	02RS METF	1356	CURITIBA	410690	1	01/06/202	06/06/202	1	6
GO	ENTORNO	1782	LUZIANIA	521250	1	01/06/202	08/06/202	2	9
SC	ARARANGI	1562	ARARANGI	420140	1	22/05/202	05/06/202	1	5
SP	GVE I CAPI	1331	SAO PAULC	355030	1	26/05/202	06/06/2021		2
MG			JUIZ DE FO	313670	1	22/05/202	04/06/202	1	
BA	NUCLEO RI	1380	SALVADOR	292740	1	09/02/2021		1	6
BA	NUCLEO RI	1380	SALVADOR	292740	1	06/02/202	08/02/202	2	
DF			BRASILIA	530010	1	02/04/2021		1	6
PR	02RS METF	1356	CURITIBA	410690	1	02/06/202	04/06/202	2	6
PR	15RS MARI	1369	MARINGA	411520	1	11/03/202	31/03/202	1	6
SC	JOINVILLE	1565	JOINVILLE	420910	1	05/08/202	16/09/202	1	2
SP	GVE I CAPI	1331	SAO PAULC	355030	1	05/08/2021		1	
SP			SAO PAULC	355030	1	17/09/202	22/09/202	2	
SP	GVE XXIII R	1347	PARIQUER/	353620	1	10/03/2021		1	6
SP	GVE I CAPI	1331	SAO PAULC	355030	1	06/05/2021		2	
PR	15RS MARI	1369	MARINGA	411520	1	28/05/202	13/06/202	1	
RJ			RIO DE JAN	330455	1	14/06/2021		1	6
GO	CENTRAL	1779	GOIANIA	520870	1	18/07/202	23/07/202	2	
SP	GVE X OSA	1335	BARUERI	350570	1	19/02/2021		1	2
MG	UBERABA	1461	ARAXA	310400	1	01/04/2021			
MG	MANHUAC	1454	MANHUAC	313940	1	03/05/202	18/05/202	2	6
SP	GVE I CAPI	1331	SAO PAULC	355030	1	06/07/202	15/07/202	3	
MS	EX ARS DE	1975	CAMPO GF	500270	1			3	9
DF			BRASILIA	530010	1	29/05/202	09/07/202	1	6
RN	II URSAP	1412	MOSSORO	240800	1	05/06/2021		2	6
MG	DIVINOPOI	1472	OLIVEIRA	314560	1	10/06/202	24/06/202	1	
RJ			NILOPOLIS	330320	1	07/05/2021		1	2
AM	ENTORNO	5584	MANAUS	130260	1	03/03/2021		1	2
SP	GVE XII AR/	1337	ARARAQU/	350320	1	17/03/202	24/03/202	9	5
AL	1 MICRORI	1533	MACEIO	270430	1	13/04/2021		2	

MT	CUIABA	1578	CUIABA	510340	1	21/06/202	27/06/202	2	
AP			SANTANA	160060	1	07/12/2021		3	6
SP	GVE I CAPI	1331	SAO PAULO	355030	1	28/04/202	01/05/202	3	
PR	02RS METR	1356	CAMPINA (	410400	1	27/03/202	31/03/202	1	2
RJ			NITEROI	330330	1	25/02/2021		2	6
SP	GVE XXIV R	1348	RIBEIRAO P	354340	1	25/05/202	30/05/202	2	4
SP	GVE I CAPI	1331	SAO PAULO	355030	1	26/04/2021		2	1
SP	GVE XXVI S	1350	MOGI GUA	353070	1	29/04/202	02/05/202	2	1
SP	GVE XIX MA	1344	MARILIA	352900	1	29/05/202	10/06/202	1	3
SC	JARAGUA D	1567	JARAGUA D	420890	1	09/07/202	26/07/202	1	5
BA	NUCLEO RE	1381	FEIRA DE S.	291080	1	20/04/2021		2	6
SP	GVE XXXI S	1353	SOROCABA	355220	1	08/03/202	19/03/202	1	9
RS			SAO GABRI	431830	1	31/07/202	26/08/202	2	6
MA			BALSAS	210140	1	05/07/2021		2	6
RJ			VALENCA	330610	1	10/02/2021		2	
MG	ALFENAS	1379	ALFENAS	310160	1	15/10/202	20/10/202	2	3
SP	GVE XXI PR	1346	PRESIDENT	354140	1	20/04/202	27/04/202	2	6
MT	CUIABA	1578	CUIABA	510340	1	05/05/202	09/05/202	3	9
GO	CENTRAL	1779	GOIANIA	520870	1	24/05/2021		3	
SP	GVE XVI BO	1341	BOTUCATU	350750	1	11/05/2021		1	4
RS	012 CRS	1601	SANTO AN	431750	1	25/05/202	14/06/202	2	
RJ			RIO DE JAN	330455	1	10/06/202	22/06/202	2	9
DF			BRASILIA	530010	1	27/09/202	30/09/202	2	6
RJ			RIO DE JAN	330455	1	13/04/202	12/06/202	1	6
SP	GVE XXIX S	1354	SAO JOSE D	354980	1	02/03/202	11/03/202	1	6
SP					1	28/04/2021		3	
SP	GVE XVIII F	1343	FRANCA	351620	1	23/05/202	20/06/202	1	6
BA	NUCLEO RE	1398	JACARACI	291740	1	15/06/2021		2	6
MS	EX ARS DE	1975	CAMPO GR	500270	1	31/05/2021		1	
PR	16RS APUC	1370	APUCARAN	410140	1	21/03/202	24/03/202	2	6
SP	GVE I CAPI	1331	SAO PAULO	355030	1	26/11/202	30/11/202	2	
PR	17RS LOND	1371	LONDRINA	411370	1	10/02/202	15/03/202	1	6
SP	GVE I CAPI	1331	SAO PAULO	355030	1	03/03/202	08/03/202	2	2
MT	CUIABA	1578	CUIABA	510340	1	22/03/202	14/04/202	1	6
DF			BRASILIA	530010	1	07/03/202	18/03/202	1	
GO	PIRINEUS	1792	ANAPOLIS	520110	1	11/04/2021		1	6
RS	001 CRS	1592	CANOAS	430460	1	23/04/2021		1	5
RS	003 CRS	1593	RIO GRAND	431560	1	18/11/202	23/11/202	1	
RN	II URSAP	1412	MOSSORO	240800	1	18/03/202	22/03/202	2	6
MG	GOVERNAD	1471	GOVERNAD	312770	1	31/05/202	07/06/202	1	
SC	TUBARAO	1555	TUBARAO	421870	1	28/07/2021		1	3
SP	GVE XVIII F	1343	FRANCA	351620	1	03/01/202	05/01/202	2	6
SP	GVE XVI BO	1341	BOTUCATU	350750	1	23/06/2021		1	4
SP	GVE X OSA	1335	OSASCO	353440	1	14/01/202	18/01/202	2	2
RS	004 CRS	1595	SANTA MA	431690	1	19/03/202	22/03/202	2	6
PA	11 REGION	1494	MARABA	150420	1	14/03/2021		3	
RJ			RIO DE JAN	330455	1	12/05/2021		3	

SP	GVE I CAPI	1331	SAO PAULO	355030	1	29/05/202	17/06/202	1	2
MG	UBERABA	1461	UBERABA	317010	1	30/04/2021		1	
GO	CENTRAL	1779	GOIANIA	520870	1	12/03/2021		2	
PR	10RS CASC	1364	CASCAVEL	410480	1	15/04/202	18/04/202	3	9
PA	11 REGION	1494	MARABA	150420	1	06/05/2021		3	
GO	CENTRAL	1779	GOIANIA	520870	1	06/08/202	10/08/202	2	
MT	CUIABA	1578	CUIABA	510340	1			2	
MA	REGIONAL	1440	IMPERATRI	210530	1	07/01/2022		3	
PR	17RS LOND	1371	LONDRINA	411370	1	13/03/202	19/03/202	2	6
PB	I NRS JOAC	1377	JOAO PESS	250750	1	24/01/202	28/02/202	1	6
SP	GVE XXIV R	1348	RIBEIRAO P	354340	1	03/05/202	20/05/202	1	3
AM	ENTORNO	5584	MANAUS	130260	1	20/03/202	28/03/202	1	6
PE	001	1497	RECIFE	261160	1	27/03/2021		1	
PA	01 REGION	1484	BELEM	150140	1	14/04/202	18/04/202	3	6
BA	NUCLEO RE	1380	SALVADOR	292740	1	26/04/202	30/04/202	2	
MT	CUIABA	1578	CUIABA	510340	1	15/08/202	31/08/202	1	
PR	02RS METR	1356	CURITIBA	410690	1	10/01/202	01/02/202	1	6
MA	REGIONAL	1447	CAXIAS	210300	1	16/03/2021		2	6
RS	018 CRS	1605	TRAMAND.	432160	1	27/06/202	18/07/202	2	
BA	NUCLEO RE	1380	SALVADOR	292740	1	05/03/202	12/03/202	2	6
RJ			RIO DE JAN	330455	1	15/03/2021		2	
CE	1 CRES FOR	1519	FORTALEZA	230440	1	22/03/202	29/03/202	2	
SP	GVE I CAPI	1331	SAO PAULO	355030	1	06/07/202	09/07/202	2	
SP			CAIEIRAS	350900	1	13/06/202	05/07/202	1	
RS	004 CRS	1595	SANTA MA	431690	1	14/06/202	25/06/202	1	
PR	02RS METR	1356	CURITIBA	410690	1	13/08/202	16/08/202	1	6
PR	17RS LOND	1371	LONDRINA	411370	1	20/05/202	30/05/202	2	4
RS	002 CRS	1607	PORTO ALE	431490	1	20/01/202	27/01/202	2	
SC	CANOINHA	1570	CANOINHA	420380	1	08/06/2021		2	6
SP	GVE XXIX S	1354	SAO JOSE D	354980	1	22/07/202	26/07/202	2	4
RJ			RIO DE JAN	330455	1	18/06/2021		3	
CE	1 CRES FOR	1519	FORTALEZA	230440	1	27/05/202	03/06/202	3	1
SC			FLORIANOI	420540	1	17/11/202	18/11/202	2	
BA	NUCLEO RE	1398	VITORIA DA	293330	1	18/04/202	03/05/202	2	
MS	EX ARS DE	1975	CAMPO GR	500270	1	12/04/2021		1	5
SP	GVE I CAPI	1331	SAO PAULO	355030	1	15/06/202	25/06/202	2	
RS	016 CRS	1610	LAJEADO	431140	1	15/02/202	19/02/202	2	6
GO	CENTRAL	1779	GOIANIA	520870	1	03/03/202	20/03/202	1	
SP	GVE I CAPI	1331	SAO PAULO	355030	1	20/02/202	28/02/202	1	5
PB	I NRS JOAC	1377	JOAO PESS	250750	1	29/03/2021		1	6
PR	10RS CASC	1364	CASCAVEL	410480	1	13/07/202	18/07/202	3	
RJ			RESENDE	330420	1	01/08/202	02/08/2021		
SP	GVE XX PIR	1345	PIRACICAB	353870	1	12/01/2021			
MG	VARGINHA	1466	AIURUOCA	310120	1	27/01/202	03/02/202	2	5
SP			SAO PAULO	355030	1	08/02/2021		2	
SP	GVE I CAPI	1331	SAO PAULO	355030	1	05/02/2021		2	6
PA	01 REGION	1484	BELEM	150140	1	08/04/202	28/04/202	2	9

SP	GVE I CAPI	1331	SAO PAULO	355030	1	19/04/202	08/05/202	1	3
SP	GVE I CAPI	1331	SAO PAULO	355030	1	14/06/202	05/07/202	1	1
RN	VII URSAP	1416	NATAL	240810	1	15/06/202	24/06/202	2	2
SP	GVE XVII C	1342	JAGUARIUI	352470	1	28/03/202	03/04/202	1	6
SP	GVE I CAPI	1331	SAO PAULO	355030	1	22/05/202	26/05/202	2	2
SP	GVE XV BA	1340	BAURU	350600	1	29/05/202	03/06/202	1	6
SC	JOINVILLE	1565	JOINVILLE	420910	1	29/08/202	13/10/202	3	6
RJ			NITEROI	330330	1	27/01/2021		2	4
RS	007 CRS	1596	BAGE	430160	1	04/04/202	14/04/202	2	3
RJ			RIO DE JAN	330455	1	30/05/2021		2	3
AM	ENTORNO	5584	MANAUS	130260	1	30/01/202	02/02/202	1	6
PR	02RS METF	1356	CAMPINA (	410400	1	08/03/202	15/03/202	2	
MG	BELO HORI	1449	BETIM	310670	1	19/02/2021		3	6
SP			SAO PAULO	355030	1	28/03/202	05/04/202	2	
PR	02RS METF	1356	CURITIBA	410690	1	12/06/202	27/07/202	1	5
SP	GVE I CAPI	1331	SAO PAULO	355030	1	10/06/202	16/06/202	2	
PR	15RS MARI	1369	MARINGA	411520	1	19/07/202	28/07/202	2	
PR	09RS FOZ D	1363	FOZ DO IGU	410830	1	22/08/202	27/08/202	2	6
DF			BRASILIA	530010	1	13/01/202	13/01/202	2	6
PE	001	1497	RECIFE	261160	1	10/03/202	12/03/202	2	
GO	CENTRAL	1779	GOIANIA	520870	1	15/03/202	20/03/202	2	
PR	17RS LOND	1371	LONDRINA	411370	1	28/03/202	29/03/202	3	2
RS	002 CRS	1607	CACHOEIRI	430310	1	26/04/202	01/05/202	1	
RN	VII URSAP	1416	NATAL	240810	1	26/05/202	03/06/202	2	6
SC	BLUMENAU	1564	BLUMENAU	420240	1	04/09/202	07/09/202	2	6
GO	CENTRAL	1779	GOIANIA	520870	1	13/01/202	18/01/202	2	
PB	I NRS JOAO	1377	JOAO PESS	250750	1	17/03/202	15/04/202	1	6
SP	GVE I CAPI	1331	SAO PAULO	355030	1	02/04/202	08/04/202	2	6
RS	001 CRS	1592	SAPIRANG	431990	1	29/04/2021		1	2
SP	GVE XVII C	1342	CAMPINAS	350950	1	16/10/202	24/11/202	1	6
PR	15RS MARI	1369	MARINGA	411520	1	06/03/202	12/03/202	2	6
SC	CONCORDI	1560	CONCORDI	420430	1	07/02/202	12/02/202	2	6
GO	CENTRAL	1779	GOIANIA	520870	1	04/06/2021			
DF			BRASILIA	530010	1	26/08/202	05/09/202	3	6
GO	ESTRADA D	1802	CATALAO	520510	1	14/03/2021		3	6
RS	005 CRS	1608	GRAMADO	430910	1	03/05/202	05/06/202	2	6
MA	REGI0NAL	1430	SAO LUIS	211130	1	19/06/202	29/06/202	2	5
ES	METROPOL	1510	SERRA	320500	1	23/03/202	01/04/202	1	9
CE	1 CRES FOF	1519	FORTALEZA	230440	1	25/05/202	26/05/202	3	6
RJ			RIO DE JAN	330455	1	12/08/202	17/08/202	2	2
MT	CUIABA	1578	CUIABA	510340	1	04/06/202	21/06/202	1	
RS	005 CRS	1608	CAXIAS DO	430510	1	21/06/2021		1	
MG	JANUARIA	1470	JANUARIA	313520	1	01/10/2021		9	9
PR	10RS CASC	1364	CASCAVEL	410480	1	08/03/202	14/04/202	2	
MG	POUSO ALE	1459	ITAJUBA	313240	1	15/01/202	18/01/202	3	6
AL	5 MICRORE	1537	ARAPIRACA	270030	1	01/06/202	17/06/202	1	
GO	CENTRAL	1779	GOIANIA	520870	1	30/03/202	02/04/202	2	

BA	NUCLEO RI	1380	SALVADOR	292740	1	10/05/202	31/05/202	1	9
SP	GVE XX PIR	1345	ARARAS	350330	1	06/04/2021		3	6
SP	GVE XXIX S	1354	SAO JOSE D	354980	1	11/06/202	17/06/202	1	4
SC			FLORIANOI	420540	1			3	
SP	GVE XXXI S	1353	SOROCABA	355220	1	07/07/202	19/07/202	1	5
SP			SAO PAULC	355030	1	21/05/202	25/05/202	3	
MG	VARGINHA	1466	BOA ESPER	310710	1	05/02/2021		1	
SC	CHAPECO	1553	CHAPECO	420420	1	06/09/2021		2	
RN	VII URSAP	1416	NATAL	240810	1	05/05/2021		2	6
RR			BOA VISTA	140010	1	01/07/202	15/07/202	1	
PB	I NRS JOAC	1377	JOAO PESS	250750	1	18/06/202	22/06/202	2	6
PR	16RS APUC	1370	APUCARAN	410140	1	12/06/202	21/06/202	1	
MG	BELO HORI	1449	BELO HORI	310620	1	23/03/202	12/04/202	1	
DF			BRASILIA	530010	1	20/04/202	23/04/202	2	6
PA	01 REGION	1484	BELEM	150140	1	24/03/202	27/03/202	2	6
RJ			CAMPOS D	330100	1	16/05/2021		1	6
GO	SUDOESTE	1800	MINEIROS	521310	1	21/01/202	28/01/202	3	6
MG	DIVINOPOI	1472	DIVINOPOI	312230	1	09/06/202	20/06/202	2	6
RJ			RIO DE JAN	330455	1	06/08/202	18/08/202	1	9
GO	CENTRAL	1779	GOIANIA	520870	1	04/04/202	08/04/202	2	
AP			MACAPA	160030	1	26/02/2021		1	6
AL	1 MICRORI	1533	MACEIO	270430	1	23/03/2021		2	
GO	CENTRAL	1779	GOIANIA	520870	1	19/04/202	27/04/202	2	
RJ			RIO DE JAN	330455	1	12/02/202	25/02/202	1	
SC			CHAPECO	420420	1	16/02/2021		1	6
RS	005 CRS	1608	CAXIAS DO	430510	1	27/03/202	07/04/202	1	5
BA	NUCLEO RI	1380	SALVADOR	292740	1	26/04/202	29/04/202	2	9
RJ			RIO DE JAN	330455	1	07/07/202	08/07/202	1	9
MS	NRS DE TRI	6255	TRES LAGO	500830	1	10/07/2021		2	
PR	11RS CAMI	1365	CAMPO MO	410430	1	03/06/202	19/06/202	3	6
SC			FLORIANOI	420540	1	11/10/202	13/10/202	2	
SP	GVE I CAPI	1331	SAO PAULC	355030	1	07/01/2021			
PI	4 GRS TERE	1883	TERESINA	221100	1	29/05/202	16/06/202	1	2
SC	BLUMENAU	1564	BLUMENAU	420240	1	25/10/202	04/11/202	1	5
RS	001 CRS	1592	CANOAS	430460	1	09/03/202	24/03/202	1	2
RS	002 CRS	1607	PORTO ALE	431490	1	17/03/202	19/04/202	1	3
MS	EX ARS DE	1975	CAMPO GF	500270	1	10/05/202	05/10/202	1	3
SC	LAGES	1571	LAGES	420930	1			2	6
CE	1 CRES FOF	1519	FORTALEZA	230440	1	17/04/202	27/04/202	1	2
SP	GVE XVII C.	1342	CAMPINAS	350950	1	19/03/202	26/03/202	2	
RS	006 CRS	1611	PASSO FUN	431410	1	01/04/202	06/04/202	1	6
MG	SAO JOAO	1460	SAO JOAO	316250	1	27/03/202	07/04/202	2	
MT	CUIABA	1578	CUIABA	510340	1	19/07/202	23/07/202	2	
RS	006 CRS	1611	PASSO FUN	431410	1	30/05/202	09/06/202	2	3
DF			BRASILIA	530010	1	12/04/202	14/05/202	1	6
RR			BOA VISTA	140010	1	05/05/202	10/05/202	1	
SP	GVE XVII C.	1342	CAMPINAS	350950	1	29/05/202	08/06/202	1	

MT	SINOP	1584	SINOP	510790	1	22/11/2021		1	2
RJ			RIO DE JAN	330455	1	12/06/202	16/07/202	1	6
SP			SAO PAUL	355030	1	27/04/202	05/05/202	1	2
GO	CENTRAL	1779	GOIANIA	520870	1	01/05/202	04/05/202	1	
RJ			RIO DE JAN	330455	1	07/05/2021		9	
SP	GVE VIII M	1333	GUARULH	351880	1	23/03/202	08/04/202	1	2
PR	09RS FOZ D	1363	FOZ DO IG	410830	1	22/07/202	24/07/202	2	
SP	GVE XIV B	1339	BARRETOS	350550	1	17/01/2021		3	
PR	17RS LOND	1371	LONDRINA	411370	1	12/04/202	29/04/202	1	5
SP	GVE XX PIR	1345	PIRACICAB	353870	1	23/02/2021		1	
PR	02RS METR	1356	CAMPINA	410400	1	21/04/2021		1	
RS	005 CRS	1608	CAXIAS DO	430510	1	03/06/202	04/06/202	1	6
SP	GVE XXIX S	1354	SAO JOSE D	354980	1	03/08/202	25/08/202	1	4
RS	002 CRS	1607	PORTO ALE	431490	1	19/02/202	03/03/202	1	4
MA	REGI0NAL	1430	SAO LUIS	211130	1	31/03/2021		2	
RJ			RIO DE JAN	330455	1	21/05/2021		1	
RJ			RIO DE JAN	330455	1	17/07/202	23/07/202	2	
SP	GVE XXXIII	1352	TAUBATE	355410	1	10/05/202	13/05/202	2	6
SP	GVE I CAPI	1331	SAO PAUL	355030	1	15/04/2021		2	5
RJ			VASSOURA	330620	1	05/06/2021		3	
TO			PALMAS	172100	1	26/07/2021			
CE	1 CRES FOR	1519	FORTALEZ	230440	1	14/08/202	18/08/202	1	1
RJ			NILOPOLIS	330320	1	10/05/202	17/05/202	2	2
SC	XANXERE	1556	XANXERE	421950	1	19/10/2021		2	
RS	001 CRS	1592	CANOAS	430460	1	22/02/202	12/04/202	1	2
RO	VI GRS POR	1483	PORTO VEL	110020	1	05/04/2021		1	6
SP	GVE I CAPI	1331	SAO PAUL	355030	1	15/02/202	16/02/202	2	
PB	III NRS CAM	1421	CAMPINA	250400	1	22/08/202	10/09/202	2	6
MG	LEOPOLDIN	1453	ALEM PAR	310150	1	25/05/202	08/06/202	2	6
PR	10RS CASC	1364	CASCAVEL	410480	1	26/08/202	03/09/202	2	
SC	ARARANGU	1562	ARARANGU	420140	1	12/11/202	25/11/202	1	6
RS	002 CRS	1607	PORTO ALE	431490	1	08/07/202	19/08/202	1	
SP	GVE I CAPI	1331	SAO PAUL	355030	1	04/04/202	13/04/202	1	6
MT	SINOP	1584	NOVA MU	510622	1				
DF			BRASILIA	530010	1	21/06/202	06/07/202	1	6
MG	BELO HORI	1449	BELO HORI	310620	1	14/07/2021		1	6
SP	GVE XII AR	1337	SAO CARL	354890	1	18/11/202	19/11/202	3	6
RS	005 CRS	1608	CAXIAS DO	430510	1	13/05/202	24/05/202	1	6
TO			ARAGUAIN	170210	1	08/10/2021		2	
MG	BELO HORI	1449	CONTAGEM	311860	1	21/07/2021		2	2
TO			PALMAS	172100	1	16/08/2021		1	5
PR	02RS METR	1356	CURITIBA	410690	1	17/03/202	19/03/202	2	5
SC	JOINVILLE	1565	JOINVILLE	420910	1	31/12/202	16/01/202	1	6
SP	GVE XVII C	1342	CAMPINAS	350950	1	30/03/202	05/04/202	2	6
BA	NUCLEO RE	1380	SALVADOR	292740	1	08/04/202	09/04/202	2	9
GO	CENTRAL	1779	GOIANIA	520870	1	12/02/202	17/02/202	2	
AC	REGIONAL	1938	RIO BRANC	120040	1	19/01/2021		1	9

MT	CUIABA	1578	CUIABA	510340	1	19/04/202	21/04/202	2	
RJ			RIO DE JAN	330455	1	05/06/202	20/06/202	2	2
SP	GVE XXIV R	1348	RIBEIRAO F	354340	1	09/04/202	12/04/202	2	2
AL	1 MICRORF	1533	MACEIO	270430	1	02/05/2021		2	
RJ			RIO DE JAN	330455	1	15/04/2021			
BA	NUCLEO RE	1380	SALVADOR	292740	1	24/05/202	27/05/202	2	
MG	BELO HORI	1449	BELO HORI	310620	1	12/05/2021		2	
BA	NUCLEO RE	1380	SALVADOR	292740	1	14/06/202	15/06/202	2	
SP	GVE XXVII S	1351	JACAREI	352440	1	25/07/202	28/07/202	2	6
SP	GVE XVI BC	1341	BOTUCATU	350750	1	10/05/2021		1	4
GO	CENTRAL	1779	GOIANIA	520870	1	03/03/202	23/03/202	1	
CE	1 CRES FOF	1519	FORTALEZA	230440	1	13/03/202	30/03/202	1	1
CE	1 CRES FOF	1519	FORTALEZA	230440	1	23/03/202	07/04/202	1	9
SP	GVE XXIX S	1354	SAO JOSE D	354980	1	10/04/202	22/05/202	1	4
BA	NUCLEO RE	1403	BARREIRAS	290320	1	29/05/202	30/05/202	2	
SP	GVE XXIV R	1348	RIBEIRAO F	354340	1	23/05/202	01/06/202	1	2
SP	GVE VII SAI	1332	SAO CAETA	354880	1	14/07/202	18/07/202	2	
SP	GVE I CAPI	1331	SAO PAULO	355030	1	05/03/202	06/03/202	2	
BA	NUCLEO RE	1380	SALVADOR	292740	1	16/04/202	18/04/202	3	
GO	CENTRAL	1779	GOIANIA	520870	1	22/05/202	12/06/202	1	
GO	CENTRAL	1779	GOIANIA	520870	1	26/05/2021		3	
CE	1 CRES FOF	1519	FORTALEZA	230440	1	09/04/202	09/04/2021		
SP	GVE VII SAI	1332	SANTO AN	354780	1	30/09/2021		3	9
SC	FLORIANOI	1476	FLORIANOI	420540	1	11/10/202	22/11/202	1	
SP	GVE I CAPI	1331	SAO PAULO	355030	1	19/04/2021		2	6
SC	FLORIANOI	1476	SAO JOSE	421660	1	26/04/202	09/05/2021		
RJ			VOLTA RED	330630	1	04/05/2021		9	9
MS	EX NRS DE	6256	CORUMBA	500320	1	30/05/202	16/06/202	1	
SP	GVE I CAPI	1331	SAO PAULO	355030	1	10/06/202	28/06/202	1	6
PR	19RS JACAI	1373	SANTO ANT	412410	1	15/07/202	21/07/202	2	
SP	GVE I CAPI	1331	SAO PAULO	355030	1	23/08/202	26/08/202	2	6
PR	19RS JACAI	1373	SANTO ANT	412410	1	23/09/202	29/09/202	3	
SP	GVE XXV SA	1349	GUARUJA	351870	1	26/05/2021		2	
BA	NUCLEO RE	1398	VITORIA DA	293330	1	03/06/2021		2	
SP	GVE XXIX S	1354	CATANDUV	351110	1	25/06/202	31/07/202	1	6
DF			BRASILIA	530010	1	24/03/202	29/03/202	1	5
SP	GVE XVII CA	1342	VALINHOS	355620	1	30/04/2021		3	6
MA	REGIONAL	1440	IMPERATRI	210530	1	20/04/202	26/04/202	2	6
PR	10RS CASC	1364	CASCAVEL	410480	1	07/06/202	03/07/202	1	
PR	03RS PONT	1357	JAGUARIAI	411200	1	15/06/202	25/06/202	1	6
CE	11 CRES SC	1521	SOBRAL	231290	1	01/11/2021		3	
PR	02RS METF	1356	CURITIBA	410690	1	20/04/202	23/05/202	2	6
MG	DIVINOPOI	1472	FORMIGA	312610	1			1	
GO	CENTRAL	1779	GOIANIA	520870	1	27/05/202	30/05/202	2	
MG			JUIZ DE FO	313670	1	05/11/202	06/11/202	2	6
MA	REGIONAL	1440	IMPERATRI	210530	1	19/05/2021		1	6
SP	GVE X OSA	1335	OSASCO	353440	1	04/06/202	21/06/202	1	2

CE	1 CRES FOF	1519	FORTALEZ/	230440	1	30/04/202	04/05/202	3	6
MT	AGUA BOA	1590	AGUA BOA	510020	1	18/09/202	21/09/202	3	6
SP	GVE XII AR/	1337	SAO CARLC	354890	1	02/09/202	03/09/202	2	6
MG	TEOFILO O	1465	TEOFILO O	316860	1	10/07/202	30/07/202	2	
PB	I NRS JOAC	1377	JOAO PESS	250750	1	08/03/202	09/03/202	1	6
MA	REGIONAL	1440	IMPERATRI	210530	1	19/04/2021		1	6
PR	10RS CASC	1364	CASCAVEL	410480	1	07/06/202	14/07/202	1	
SP	GVE XXIV R	1348	RIBEIRAO F	354340	1	03/06/202	04/07/202	2	3
PR	16RS APUC	1370	APUCARAN	410140	1	03/07/202	07/07/202	3	
PB	I NRS JOAC	1377	JOAO PESS	250750	1	01/08/202	12/08/202	1	6
SC	LAGES	1571	LAGES	420930	1	21/08/2021		2	6
SP	GVE VII SAI	1332	RIBEIRAO F	354330	1	03/11/202	05/11/202	2	6
AP			MACAPA	160030	1			2	6
SP	GVE I CAPI	1331	SAO PAULC	355030	1	05/05/2021		1	2
RJ			RIO DE JAN	330455	1	05/01/2021		2	
RR			BOA VISTA	140010	1			1	
PR	17RS LOND	1371	LONDRINA	411370	1	12/05/202	14/05/202	2	6
BA	NUCLEO RI	1385	ILHEUS	291360	1	30/05/202	05/06/2021		
CE	11 CRES SC	1521	SOBRAL	231290	1	05/03/202	30/03/202	3	
BA	NUCLEO RI	1380	SALVADOR	292740	1	28/03/202	31/03/202	3	
SP	GVE I CAPI	1331	SAO PAULC	355030	1	10/03/202	02/04/202	1	
SP	GVE VIII M	1333	MOGI DAS	353060	1	16/03/202	20/03/202	1	2
MG	MONTES C	1473	MONTES C	314330	1	13/08/2021		1	4
PR	17RS LOND	1371	LONDRINA	411370	1	07/03/202	18/03/202	1	6
MA	REGI0NAL	1430	SAO LUIS	211130	1	01/06/202	02/06/202	1	6
MT	CUIABA	1578	CUIABA	510340	1	16/08/202	21/08/202	1	
PR	02RS METF	1356	CAMPO LA	410420	1	04/06/202	22/06/202	1	6
ES	METROPOI	1510	SERRA	320500	1	15/07/2021		1	2
SP	GVE I CAPI	1331	SAO PAULC	355030	1	01/07/202	06/07/202	3	
SP	GVE I CAPI	1331	SAO PAULC	355030	1	24/03/202	30/03/202	2	6
CE	1 CRES FOF	1519	FORTALEZ/	230440	1	16/04/202	21/04/202	3	
AM	ENTORNO	5584	MANAUS	130260	1	15/03/202	03/05/202	1	2
PR	17RS LOND	1371	LONDRINA	411370	1	30/03/202	13/04/202	1	6
SP	GVE XXI PR	1346	PRESIDENT	354140	1	25/05/202	31/05/202	2	6
SP	GVE I CAPI	1331	SAO PAULC	355030	1	09/06/202	14/06/202	3	
SP	GVE I CAPI	1331	SAO PAULC	355030	1	06/07/202	15/07/202	3	9
RJ			RESENDE	330420	1	13/09/2021			
SP			SAO PAULC	355030	1	03/02/202	04/04/2021		
SP	GVE I CAPI	1331	SAO PAULC	355030	1	10/04/2021		2	
SP	GVE VIII M	1333	MOGI DAS	353060	1	30/03/202	02/04/202	3	2
PR	09RS FOZ D	1363	FOZ DO IGU	410830	1	07/08/202	13/08/202	2	
SP	GVE I CAPI	1331	SAO PAULC	355030	1	21/03/202	23/03/202	2	
SP	GVE XXIX S	1354	SAO JOSE D	354980	1	14/05/202	16/05/202	2	2
MG	POUSO ALI	1459	POCOS DE	315180	1	17/07/202	21/07/202	1	2
SP	GVE XVII C/	1342	CAMPINAS	350950	1	09/01/202	09/01/202	3	6
SP	GVE XXXI S	1353	SOROCABA	355220	1	17/05/202	25/05/202	1	
BA	NUCLEO RI	1380	SALVADOR	292740	1	26/06/202	12/07/202	1	9

SP	GVE I CAPI	1331	SAO PAUL(	355030	1	12/01/2021			2
GO	CENTRAL	1779	GOIANIA	520870	1	05/02/202	24/02/202	1	
SP	GVE I CAPI	1331	SAO PAUL(	355030	1	10/04/202	26/04/202	1	4
SP	GVE VIII M	1333	MOGI DAS	353060	1	24/05/2021		3	6
PA	01 REGION	1484	BELEM	150140	1	10/06/2021		2	
MT	CUIABA	1578	CUIABA	510340	1	17/03/202	26/04/202	1	
SP			GUARULH(	351880	1	08/03/202	16/03/202	1	
RS	002 CRS	1607	PORTO ALE	431490	1	22/03/202	30/03/202	2	5
AL	1 MICRORI	1533	MACEIO	270430	1	13/03/202	17/03/2021		
SP	GVE I CAPI	1331	SAO PAUL(	355030	1	09/04/202	11/04/202	1	
MT	CUIABA	1578	CUIABA	510340	1	30/06/202	14/07/202	2	2
RJ			RIO DE JAN	330455	1	11/02/2021		3	
AL	1 MICRORI	1533	MACEIO	270430	1	25/02/202	17/03/202	2	
RJ			RIO DE JAN	330455	1	17/03/2021		1	
SP	GVE XVII C.	1342	HORTOLAN	351907	1	29/03/2021		1	
DF			BRASILIA	530010	1	05/12/202	23/12/202	1	
CE	11 CRES SC	1521	SOBRAL	231290	1	02/12/2021			
DF			BRASILIA	530010	1	15/03/202	20/03/202	2	
SP			MARILIA	352900	1	19/04/202	04/05/202	1	5
GO			GOIANIA	520870	1	16/06/2021		1	
RN	VII URSAP	1416	NATAL	240810	1	21/02/202	23/02/202	1	9
SP	GVE I CAPI	1331	SAO PAUL(	355030	1	16/03/2021		2	
SP	GVE XXI PR	1346	PRESIDENT	354140	1	15/02/2021		2	5
SP	GVE XVII C.	1342	CAMPINAS	350950	1	01/06/202	07/06/202	2	
SP	GVE XX PIR	1345	PIRACICAB	353870	1	18/07/2021		2	
GO	CENTRAL	1779	GOIANIA	520870	1	06/08/202	26/08/202	1	
PR	02RS METF	1356	CURITIBA	410690	1	19/02/202	15/03/202	1	6
MG	SETE LAGO	1467	SETE LAGO	316720	1	20/03/202	27/03/202	1	2
SP	GVE X OSA	1335	OSASCO	353440	1	23/04/202	28/06/202	2	5
SP	GVE I CAPI	1331	SAO PAUL(	355030	1	25/05/202	29/05/202	2	6
SP	GVE VIII M	1333	GUARULH(	351880	1	20/07/202	26/07/202	3	6
MT	CUIABA	1578	CUIABA	510340	1	18/05/2021		1	6
RS	012 CRS	1601	SANTO AN	431750	1	03/11/202	04/11/202	2	
CE			MARACAN	230765	1	24/04/202	30/04/202	2	6
PR	17RS LONI	1371	LONDRINA	411370	1	18/05/202	20/06/202	2	6
PR	15RS MARI	1369	MARINGA	411520	1	28/06/202	28/07/202	1	6
PI	4 GRS TERI	1883	TERESINA	221100	1	28/12/202	01/01/202	2	2
SP	GVE XXVI S	1350	MOJI MIRI	353080	1	12/03/2021		2	
PR	02RS METF	1356	CURITIBA	410690	1	01/06/202	08/06/202	3	6
SP	GVE X OSA	1335	OSASCO	353440	1	18/09/2021		2	6
CE	1 CRES FOI	1519	FORTALEZ/	230440	1	23/04/202	26/04/202	3	
SP	GVE I CAPI	1331	SAO PAUL(	355030	1	22/06/202	28/06/202	3	
DF			BRASILIA	530010	1	15/09/202	22/10/202	1	6
PR	02RS METF	1356	SAO JOSE I	412550	1	06/08/2021		2	6
MA	REGI0NAL	1430	SAO LUIS	211130	1	26/05/202	31/05/202	2	2
AL			MACEIO	270430	1	24/10/202	04/11/202	1	
MG	CORONEL I	1468	CARATING.	311340	1	02/03/2021		2	

SP	GVE XVII C/	1342	CAMPINAS	350950	1	02/04/2021		2	6
TO			ARAGUAIN	170210	1	07/06/2021		2	6
GO	CENTRAL	1779	GOIANIA	520870	1	24/05/202	26/05/202	2	
GO	CENTRAL	1779	GOIANIA	520870	1	27/07/202	30/08/202	1	
PR	15RS MARI	1369	MARINGA	411520	1	26/10/202	08/11/202	2	6
SP	GVE XVII C/	1342	JUNDIAI	352590	1	31/03/2021		2	
MG	JUIZ DE FO	1452	JUIZ DE FO	313670	1	07/04/202	15/04/202	1	
SP	GVE XXVI S	1350	MOGI GUA	353070	1	16/06/202	21/06/202	2	9
SP	GVE I CAPI	1331	SAO PAULO	355030	1	20/03/202	23/03/202	1	2
AL	9 MICRORE	1541	PALMEIRA	270630	1	09/04/2021		2	6
PR	16RS APUC	1370	APUCARAN	410140	1	25/04/202	10/05/202	1	6
GO	CENTRAL	1779	GOIANIA	520870	1	03/08/202	06/08/202	2	
MA	REGIONAL	1441	BALSAS	210140	1	11/04/202	23/04/202	2	
SP	GVE XVII C/	1342	JUNDIAI	352590	1	24/06/2021		2	
PA	01 REGION	1484	BELEM	150140	1	19/03/2021		2	6
PR	02RS METF	1356	CURITIBA	410690	1	14/03/202	01/04/202	1	6
SP	GVE I CAPI	1331	SAO PAULO	355030	1	21/04/202	23/05/202	1	3
SP	GVE XXXI S	1353	SOROCABA	355220	1	06/05/202	07/05/202	1	2
SP	GVE XXIX S	1354	SAO JOSE D	354980	1	06/04/202	07/04/202	2	6
PR	02RS METF	1356	CURITIBA	410690	1	03/09/202	12/09/202	1	
RS	001 CRS	1592	NOVO HAM	431340	1	09/07/202	27/07/202	2	
CE	1 CRES FOF	1519	FORTALEZA	230440	1	23/03/202	13/04/202	1	
BA	NUCLEO RI	1380	SALVADOR	292740	1	10/03/202	23/03/202	1	5
PR	02RS METF	1356	CAMPO LA	410420	1	19/05/202	01/06/202	1	6
GO	CENTRAL	1779	GOIANIA	520870	1	29/06/202	04/07/202	3	
PR	09RS FOZ D	1363	MATELAND	411560	1	27/05/202	10/06/202	1	2
PR	02RS METF	1356	CAMPO LA	410420	1	09/01/202	08/03/202	3	6
PR	15RS MARI	1369	MARINGA	411520	1	20/07/202	23/07/202	3	
SP	GVE XI ARA	1336	BIRIGUI	350650	1	24/02/202	13/03/202	2	6
SP	GVE I CAPI	1331	SAO PAULO	355030	1	04/05/202	05/05/202	2	2
GO	CENTRAL	1779	GOIANIA	520870	1	24/07/202	08/08/202	1	
RJ			DUQUE DE	330170	1	15/03/2021		2	6
SP	GVE X OSA	1335	OSASCO	353440	1	08/03/202	15/03/202	2	6
SC	CRICIUMA	1558	CRICIUMA	420460	1	05/05/202	12/05/202	1	6
BA	NUCLEO RI	1380	SALVADOR	292740	1	22/05/202	23/05/202	2	
RS	003 CRS	1593	PELOTAS	431440	1	10/06/202	16/06/202	2	
SC	JOINVILLE	1565	JOINVILLE	420910	1	24/02/202	05/03/202	2	6
SP	GVE XXIV R	1348	RIBEIRAO P	354340	1	13/04/2021		1	3
SP	GVE XVII C/	1342	CAMPINAS	350950	1	30/04/2021		2	6
PA	01 REGION	1484	BELEM	150140	1	15/06/2021		3	2
MG	POUSO ALI	1459	ITAJUBA	313240	1	24/06/202	29/06/202	2	6
AL	1 MICRORI	1533	MACEIO	270430	1	27/09/202	09/10/202	1	
RJ			RIO DE JAN	330455	1	22/06/202	04/07/202	1	
PR	09RS FOZ D	1363	FOZ DO IGU	410830	1	03/01/2022		3	6
SP	GVE X OSA	1335	BARUERI	350570	1			3	
TO			ARAGUAIN	170210	1	02/02/202	11/02/202	3	5
SP	GVE VII SAI	1332	SAO CAETA	354880	1	12/01/202	15/01/202	3	

RS	003 CRS	1593	PELOTAS	431440	1	23/02/202	01/03/202	1	5
PR	09RS FOZ D	1363	FOZ DO IGU	410830	1	07/02/202	10/02/202	3	
TO			ARAGUAIN	170210	1	23/01/202	25/01/202	2	
SP	GVE XII AR/	1337	SAO CARLC	354890	1	06/01/202	07/01/202	2	6
PR	10RS CASC	1364	CASCAVEL	410480	1	20/01/202	22/01/202	9	
MG	PATOS DE I	1456	PATOS DE I	314800	1	13/01/2022		3	
TO			ARAGUAIN	170210	1	23/01/2022		3	6
PR	10RS CASC	1364	CASCAVEL	410480	1	19/01/202	22/01/202	3	
MT	BARRA DO	1580	BARRA DO	510180	1	04/02/2022			
AM	ENTORNO	5584	MANAUS	130260	1	21/01/2022		1	1
SP	GVE I CAPI	1331	SAO PAULC	355030	1	06/01/202	08/01/2022		
BA	NUCLEO RI	1380	SALVADOR	292740	1	19/01/202	01/02/202	2	
SP	GVE I CAPI	1331	SAO PAULC	355030	1	15/01/2022		2	1
PR	10RS CASC	1364	CASCAVEL	410480	1	20/01/202	23/01/202	3	
MG	PATOS DE I	1456	PATOS DE I	314800	1	26/01/202	27/01/202	2	
SC	JARAGUA D	1567	JARAGUA D	420890	1	29/01/202	30/01/202	2	6
MG	PATOS DE I	1456	PATOS DE I	314800	1	07/02/202	09/02/202	3	
PA	11 REGION	1494	PARAUAPE	150553	1	02/02/2022		1	
PR	20RS TOLEI	1374	TOLEDO	412770	1				6
RJ			ITAPERUN/	330220	1	20/01/202	22/01/202	3	6
BA	NUCLEO RI	1380	SALVADOR	292740	1	27/01/2022		2	
SP	GVE XXXI S	1353	SOROCABA	355220	1	06/01/202	06/01/202	9	9
PR	10RS CASC	1364	CASCAVEL	410480	1	15/01/202	17/01/202	3	
PA	01 REGION	1484	BELEM	150140	1	12/01/202	14/01/202	3	5
ES	METROPOI	1510	SERRA	320500	1	21/01/2022		3	6
SC	ARARANGU	1562	ARARANGU	420140	1	19/01/202	22/01/202	2	
PR	02RS METF	1356	CAMPO LA	410420	1	05/02/202	13/02/202	2	6
PR	10RS CASC	1364	CASCAVEL	410480	1	16/01/202	18/01/202	3	
PR	10RS CASC	1364	CASCAVEL	410480	1	21/01/202	23/01/202	3	
PA	01 REGION	1484	BELEM	150140	1	26/01/2022		2	2
PR	09RS FOZ D	1363	FOZ DO IGU	410830	1	24/01/202	26/01/202	3	
BA	NUCLEO RI	1380	SALVADOR	292740	1	15/01/202	15/01/202	3	
PR	02RS METF	1356	CAMPO LA	410420	1	18/02/202	23/02/2022		
SP	GVE I CAPI	1331	SAO PAULC	355030	1	13/01/202	15/01/202	2	
2					1	21/01/202	24/01/202	2	
SP	GVE XIII AS	1338	ASSIS	350400	1	04/01/202	05/01/202	2	2
SC	CRICIUMA	1558	CRICIUMA	420460	1	19/01/2022		3	6
RJ			RIO DE JAN	330455	1	07/01/202	11/01/202	2	6
PR	10RS CASC	1364	CASCAVEL	410480	1	15/01/202	17/01/202	3	
SP	GVE XXVII S	1351	SAO JOSE D	354990	1	24/02/2022		3	
SP	GVE XXII PI	1573	DRACENA	351440	1	16/03/2022		3	6
PI	4 GRS TERE	1883	TERESINA	221100	1	08/01/202	21/01/202	1	2
PB	I NRS JOAC	1377	JOAO PESS	250750	1	17/01/2022		1	6
RS	001 CRS	1592	NOVO HAN	431340	1	18/01/202	24/01/202	2	
MG	PATOS DE I	1456	PATOS DE I	314800	1	07/02/202	09/02/202	3	
RJ			RIO DE JAN	330455	1	17/01/2022		3	
SP	GVE VII SAI	1332	SAO CAETA	354880	1	13/01/2022		3	

PR	10RS CASC	1364	CASCAVEL	410480	1	17/01/202	19/01/202	3	
PR	09RS FOZ D	1363	FOZ DO IGU	410830	1	17/01/202	18/01/202	3	6
MG	MANHUAC	1454	MANHUAC	313940	1	27/01/202	27/01/202	2	6
SP	GVE XXVII S	1351	SAO JOSE D	354990	1	14/01/202	19/01/202	1	
RJ			RIO DE JAN	330455	1	12/01/2022		3	
RJ			RIO DE JAN	330455	1	18/01/2022		9	6
MG	UBERLAND	1462	UBERLAND	317020	1	26/01/2022		1	6
RJ			NILOPOLIS	330320	1	09/01/2022		3	1
SP	GVE XVII C	1342	JUNDIAI	352590	1	19/01/2022		3	9
GO	CENTRAL	1779	GOIANIA	520870	1	25/01/202	25/01/202	2	2
BA	NUCLEO RE	1380	SALVADOR	292740	1	17/01/202	18/01/202	2	2
PI	4 GRS TERE	1883	TERESINA	221100	1	15/01/202	20/01/202	1	5
AP			SANTANA	160060	1	28/01/202	31/01/202	2	
RJ			RIO DE JAN	330455	1	10/01/2022		1	9
SP	GVE VII SA	1332	RIBEIRAO P	354330	1	15/02/2022		3	9
SP	GVE I CAPI	1331	SAO PAULO	355030	1	07/02/202	08/02/202	3	
SP	GVE I CAPI	1331	SAO PAULO	355030	1	02/02/202	05/02/202	3	
SP	GVE VII SA	1332	SAO CAETA	354880	1	14/01/202	21/01/2022		
SP	GVE XXI PR	1346	PRESIDENT	354140	1	09/02/202	10/02/202	2	6
MA	REGIONAL	1440	IMPERATRI	210530	1	20/01/2022		1	
SP			SAO PAULO	355030	1	07/01/2022		1	2
SP	GVE XX PIR	1345	ARARAS	350330	1	17/01/2022		2	
PB	III NRS CAM	1421	CAMPINA G	250400	1	20/02/202	24/02/202	1	6
SP	GVE XVI BO	1341	SAO MANU	355010	1	25/01/202	26/01/202	3	6
PR	10RS CASC	1364	CASCAVEL	410480	1	22/01/202	24/01/202	3	
SP	GVE VII SA	1332	SAO CAETA	354880	1	17/01/202	20/01/202	3	
PR	10RS CASC	1364	CASCAVEL	410480	1	17/01/202	20/01/202	3	
GO			GOIANIA	520870	1	12/01/202	15/01/2022		
2					1	15/01/202	04/02/202	1	2
RJ			ITAPERUNA	330220	1	10/01/202	11/01/202	3	6
PR	09RS FOZ D	1363	FOZ DO IGU	410830	1	25/01/202	26/01/202	3	
SP	GVE VIII M	1333	GUARULHO	351880	1	16/01/2022		3	6
MA	REGIONAL	1440	IMPERATRI	210530	1	03/02/2022		2	6
RJ			RIO DE JAN	330455	1	13/02/202	20/02/202	1	6
MG	BELO HORI	1449	BELO HORI	310620	1	31/01/202	01/02/202	3	6
2					1	15/01/202	19/01/202	3	1
MG	VARGINHA	1466	VARGINHA	317070	1	22/01/202	23/01/202	2	6
RJ			ITAPERUNA	330220	1	29/01/202	30/01/202	3	6
PR	09RS FOZ D	1363	FOZ DO IGU	410830	1	16/01/2022		2	
2					1	27/01/2022		3	6
AP			SANTANA	160060	1	20/01/202	22/01/202	3	6
GO	CENTRAL	1779	GOIANIA	520870	1	21/02/202	24/02/202	2	
MG	BELO HORI	1449	BELO HORI	310620	1	29/01/2022		1	2
MG	VARGINHA	1466	AIURUOCA	310120	1	12/01/202	14/01/202	3	6
CE	8 CRES QU	1517	QUIXERAM	231140	1	20/02/2022		2	
SP	GVE XIX MA	1344	MARILIA	352900	1	22/02/202	25/02/202	2	6
RJ			ITAPERUNA	330220	1	20/01/202	22/01/2022		

SP	GVE I CAPI	1331	SAO PAULO	355030	1	16/01/202	19/01/202	3	6
PR	09RS FOZ D	1363	FOZ DO IGU	410830	1	17/01/202	18/01/202	3	6
PR	10RS CASC	1364	CASCAVEL	410480	1	22/01/202	25/01/202	3	
SP	GVE VII SAI	1332	RIBEIRAO F	354330	1	14/01/2022		3	9
PI	10 GRS FLO	1889	FLORIANO	220390	1	13/02/202	14/02/202	3	
SP	GVE I CAPI	1331	SAO PAULO	355030	1	03/02/2022		1	
PR	17RS LOND	1371	LONDRINA	411370	1	20/01/202	21/01/202	3	6
MG	PATOS DE I	1456	PATOS DE I	314800	1	12/01/2022		3	
SP	GVE XXVII S	1351	JACAREI	352440	1	27/01/2022		3	9
SC	CRICIUMA	1558	ICARA	420700	1	04/02/202	06/02/202	2	
SP	GVE XVII C/	1342	CAMPINAS	350950	1	31/01/202	09/02/202	2	
BA	NUCLEO RE	1380	SALVADOR	292740	1	10/02/202	12/02/202	2	5
SC	LAGES	1571	LAGES	420930	1	15/02/202	20/02/202	3	
RS	007 CRS	1596	BAGE	430160	1	20/01/202	24/01/202	1	6
SP	GVE XXXII I	1575	ITAPEVA	352240	1	09/02/2022		2	
AP			SANTANA	160060	1	22/01/202	29/01/202	3	6
SP	GVE XIX M/	1344	MARILIA	352900	1	14/01/202	01/02/202	3	6
PR	10RS CASC	1364	CASCAVEL	410480	1	23/01/202	27/01/202	3	
RS	001 CRS	1592	SAO LEOPO	431870	1	28/03/202	30/03/202	3	
MS	EX NRS DE	6256	CORUMBA	500320	1	23/01/202	07/02/202	2	
MG	POUSO ALE	1459	POCOS DE	315180	1	03/01/202	10/01/202	2	2
SP	GVE I CAPI	1331	SAO PAULO	355030	1	15/01/202	18/01/202	1	2
SP	GVE VII SAI	1332	SANTO AN	354780	1	30/01/2022		3	1

RAIOX_OUT	DT_RAIOX	AMOSTRA	DT_COLETA	P_AMOSTR	OUT_AMOS	PCR_RESUL	DT_PCR	POS_PCRFL	TP_FLU_PCI
		1	23/06/202	1		1	23/06/202	2	
	08/01/202	1	10/01/202	1		1	19/01/202	2	
		1	22/12/202	1		1	22/12/202	9	
		1	23/04/202	1		2	11/05/2020		
	17/07/202	1	17/07/202	1		1	20/07/2020		
		1	02/08/202	1		1	04/08/202	2	
		1	17/07/202	1		1	25/07/202	9	
		1	18/09/202	4	SANGUE	5			
		1	22/09/202	1		2	26/09/2020		
		1	06/05/202	1		1	10/05/202	9	
PERIHILAR	16/08/202	1	17/08/202	1		1	18/08/2020		
		1	04/12/202	1		1	05/12/2020		
	07/01/202	1	05/01/202	4	TESTE RAPI	2	18/01/2021		
		1	27/11/202	1		1	02/12/2020		
		1	13/12/202	4	SANGUE	4			
		1	14/05/202	1		1	16/05/2020		
	04/06/202	1	03/06/202	1		1	06/06/2020		
		1	08/07/202	1		1	10/07/2020		
TC DE TOR	15/07/202	1	12/07/202	1		1	16/07/2020		
		1	15/09/202	1		2	15/09/2020		
		1	02/12/202	4	SANGUE	5			
		1	16/12/202	1		1	18/12/202	2	
		1	30/05/202	1		1	01/06/2020		
		1	06/06/202	1		1	09/06/202	2	
		1	29/04/202	4	TESTE RAPI	5			
	03/06/202	1	06/06/202	1		1	14/06/2020		
VIDRO FOS	11/06/202	1	31/05/202	1		1			
	25/11/202	1	21/11/202	1		1	21/11/2020		
		1	19/10/202	1		1	20/10/2020		
	07/09/202	1	08/09/202	1		5			
VIDRO FOS	10/05/202	1	10/05/202	1		1	11/05/202	9	
	10/06/202	1	10/06/202	1		1	11/06/202	2	
		1	06/10/202	1		1	08/10/202	2	
		1	15/10/202	1		1	16/10/202	2	
	02/01/202	1	30/12/202	1		1	06/01/202	9	
		1	23/06/202	1		1	29/06/202	2	
OPACIDAD	20/08/202	1	19/08/202	1		1	24/08/202	9	
		1	22/06/202	1		1	24/06/2020		
	18/07/202	1	17/07/202	1		1	18/07/2020		
		1	09/08/202	1		1	11/08/2020		
		1	29/12/202	1		1	05/01/2021		
		1	22/04/202	1		1	28/04/202	2	
		1	30/04/202	1		1	08/05/202	2	
		1	09/04/202	1		1	10/04/202	2	
		1	24/03/202	1		5			

DERRAME	24/06/202	1	25/06/202	1		1	25/06/2020	
	24/04/202	1	25/04/202	1		1	27/04/2020	
		1	13/06/202	4	SANGUE	1	20/06/202	9
		1	04/06/202	1		1		
		1	30/06/202	1		1	02/07/202	9
	27/04/202	1	06/05/202	1		1	11/05/2020	
		1	13/11/202	1		1	13/11/202	9
		1	01/01/202	4	NASOFARII	1	02/01/202	2
		1	08/05/202	1		1	17/05/202	9
		1	31/07/202	1		1	04/08/202	2
	15/06/202	1	15/06/202	4	SECRECAO	1	15/06/202	9
		1	02/08/202	1		2	05/08/2020	
		1	13/07/202	1		1	13/07/202	2
		1	12/08/202	1		1	15/08/202	9
	25/11/202	1	25/11/202	1		1	26/11/2020	
	12/11/202	1	09/11/202	1		1	11/11/202	2
	26/05/202	1	22/05/202	1		1	23/05/2020	
		1	28/04/202	1		1	11/05/202	2
		1	16/06/202	4	TESTE RAPI	5		
		1	10/09/202	1		1	12/09/202	2
		1	19/10/202	1		1	21/10/202	2
		1	11/05/202	1		1	19/05/2020	
		1	02/01/202	1		1	03/01/2021	
		1	30/05/202	1		1	01/06/202	2
	23/06/202	1	23/06/202	1		1	27/06/202	2
		1	23/06/202	1		1	25/06/202	9
		1	29/07/202	1		1	30/07/202	2
	27/07/202	1	29/07/202	1		1	29/07/2020	
		1	16/09/202	1		1	17/09/202	2
		1	01/08/202	1		1	05/08/202	2
		1	29/03/202	1		1	01/04/202	2
		1	17/04/202	1		1	20/04/2020	
	14/04/202	1	14/04/202	1		1	19/04/202	2
		1	20/07/202	1		1	25/07/202	2
TC	15/06/202	1	26/06/202	1		1	27/06/2020	
	05/07/202	1	26/06/202	1		1	28/06/202	2
		2						
TOMOGRA	11/12/202	1	08/12/202	1		1	08/12/2020	
		1	11/12/202	4	PCR	2	11/12/2020	
		1	23/12/202	1		1	26/12/202	9
ASPECTO II	28/04/202	1	28/04/202	9		1	28/04/202	9
	26/04/202	1	26/04/202	1		1	29/04/2020	
	01/07/202	1	02/07/202	1		1	04/07/2020	
		1	28/07/202	1		2	03/08/2020	
		1	27/07/202	1		1	29/07/202	2
		1	18/11/202	1		2	24/11/2020	
		1	10/11/202	4	NASOFARII	1	12/11/2020	

		1	01/10/202	4	TESTE RAPI	4		
		1	22/12/202	1		1	22/12/2020	
		1	20/04/202	1		1	21/04/2020	
VIDRO FOS	21/04/202	1	20/04/202	1		1	21/04/202	9
VIDRO FOS	15/05/202	1	15/05/202	1		1	02/06/202	9
		1	20/06/202	1		1	24/06/202	2
		1	24/06/202	1		1	26/06/202	2
		1	17/08/202	1		1	19/08/202	2
		1	07/07/202	1		2	08/07/2020	
	07/04/202	1	29/03/202	1		1	29/03/202	2
TC TOR E C	17/05/202	1	17/05/202	1		1	18/05/202	2
		1	27/12/202	1		1	29/12/2020	
	28/04/202	1	28/04/202	1		1	29/04/202	9
VIDRO FOS	30/06/202	1	01/07/202	4	SECRECAO	2	02/07/2020	
		1	22/04/202	1		1	23/04/202	2
		1	12/06/202	1		1	13/06/2020	
TC DE TOR	14/06/202	1	14/06/202	4	SANGUE	4		
		1	27/08/202	4	ASPIRADO	1	02/09/202	2
		1	10/07/202	1		1	15/07/2020	
		1	07/10/202	1		1	08/10/202	2
	12/07/202	1	12/07/202	1		1	13/07/202	2
		1	29/06/202	1		1	08/07/202	2
		1	09/09/202	1		1	10/09/202	2
	19/09/202	1	09/09/202	1		2	10/09/2020	
		1	28/04/202	1		1	21/05/202	2
	17/05/202	1	19/05/202	1		1	23/05/2020	
		1	19/06/202	1		1	23/06/202	9
		1	13/07/202	1		1	17/07/2020	
		1	20/10/202	1		1	21/10/2020	
		1	06/12/202	1		1	06/12/2020	
		1	10/12/202	1		1	11/12/2020	
		1	01/05/202	1		2	10/05/2020	
	12/06/202	1	13/06/202	1		1	14/06/202	9
		1	06/12/202	1		1	06/12/202	2
		1	14/07/202	4	SANGUE	4		
		1	21/08/202	4	SANGUE	5		
		1	01/07/202	1		1	02/07/202	9
		1	23/10/202	1		1	25/10/202	2
		1	11/05/202	4	TESTE RAPI	5		
		1	29/08/202	1		1	29/08/202	9
		1	08/12/202	1		1	13/12/202	2
		1	16/12/202	4	SWAB NAS	1	17/12/202	2
	27/03/202	1	27/03/202	1		1	15/04/202	2
		1	24/06/202	1		1	29/06/2020	
		1	10/07/202	1		1	14/07/2020	
		1	18/09/202	1		1	22/09/202	2
		1	13/12/202	4	TESTE RAPIDO			

	01/11/202	2						
VIDRO FOS	29/06/202	1	23/06/202	1		1	26/06/202	9
		1	02/12/202	4	TESTE RAPI	4		
		1	04/10/202	1		1	06/10/202	2
		1	23/11/202	1		1	24/11/202	2
	16/06/202	1	11/05/202	4	TESTE RAPI	4		
VIDRO FOS	08/05/202	1	05/05/202	1		1	08/05/202	9
	03/05/202	1	03/05/202	1		1	21/05/2020	
	30/06/202	1	30/06/202	1		1	01/07/202	9
		1	03/09/202	1		1	09/09/2020	
		1	01/05/202	1		1	05/05/202	2
	11/05/202	1	11/05/202	1		1	13/05/2020	
	29/12/202	1	30/12/202	4	SWAB NAS	1	07/01/202	2
		1	18/12/202	1		1	19/12/2020	
INFILTRAD	02/06/202	1	02/06/202	4	TESTE RAPI	4		
	01/06/202	1	01/06/202	1		1	02/06/202	9
	14/07/202	1	14/07/202	1		1	17/07/2020	
		1	17/12/202	1		1	23/12/202	2
TC DE TOR	30/06/202	1	30/06/202	1		1	06/07/202	9
		1	18/05/202	1		1	21/05/2020	
	22/09/202	1	15/09/202	1		4		
		1	25/11/202	1		1	26/11/202	2
		1	25/04/202	1		1	25/04/202	9
		1	26/05/202	1		1	29/05/2020	
	05/05/202	1	06/05/202	1		1	09/05/2020	
	22/07/202	1	20/07/202	1		1	22/07/202	2
	09/07/202	1	08/07/202	1		1	12/07/202	2
		1	23/08/202	1		1	27/08/202	2
		1	29/09/202	1		1	30/09/2020	
		1	05/09/202	4		5		
	08/01/202	1	08/01/202	1		1	13/01/202	2
		1	01/12/202	1		1	01/12/2020	
		1	27/12/202	1		1	28/12/202	9
	05/01/202	1	02/01/202	1		1	04/01/202	2
		1	16/06/202	4	SWAB NAS	1	20/06/202	2
		1	13/12/202	4	TESTE RAPI	1	13/12/202	2
	02/05/202	1	05/05/202	4	SEC TRAQU	1	06/05/202	9
	14/05/202	1	14/05/202	1		1	23/05/2020	
		1	12/06/202	1		1	17/06/202	2
		1	20/06/202	1		1	20/06/2020	
		1	13/06/202	1		1	14/06/202	2
		1	24/08/202	1		1	28/08/202	2
	25/04/202	1	25/04/202	1		1	28/04/2020	
		1	18/05/202	1		1	20/05/2020	
		1	23/04/202	1		1	08/05/202	2
	10/04/202	1	16/04/202	1		1	18/04/202	2
		1	08/06/202	1		1	10/06/2020	

		1	24/06/2020			1	30/06/2020	
		1	17/07/202	4	SANGUE	5		
	09/08/202	1	07/08/202	1		1	13/08/2020	
		1	16/09/202	1		1	18/09/2020	
	17/09/202	1	14/09/202	1		1	16/09/202	2
		1	11/09/202	1		3	20/09/2020	
	12/05/202	1	13/05/202	1		1	21/05/202	2
		1	16/12/202	1		1	18/12/2020	
		1	16/06/202	4	TESTE RAP	4		
		1	13/04/202	1		1	17/04/202	2
		1	27/06/202	1		1	02/07/202	2
		1	10/08/202	1		1	07/12/2020	
		1	29/12/202	1		1	30/12/202	9
	06/06/202	1	12/06/202	1		1	21/06/202	2
		1	30/04/202	1		1	10/05/2020	
	13/07/202	1	13/07/202	1		1	15/07/202	2
	31/07/202	1	24/07/202	1		1	30/07/202	2
		1	04/12/202	1		1	06/12/202	9
		1	17/06/202	1		1	19/06/202	2
		1	13/07/202	1		1	14/07/202	2
		1	14/08/202	1		1	15/08/2020	
		1	21/10/202	1		2	24/10/2020	
	19/11/202	1	19/11/202	1		1	19/11/2020	
		1	11/11/202	1		1	11/11/202	2
		1	10/06/202	1		1	12/06/202	2
		1	15/07/202	4	SANGUE	5		
		1	20/08/202	1		1	20/08/202	2
		1	24/07/202	1		1	27/07/202	9
		1	18/10/202	1		1	20/10/202	2
	27/05/202	1	28/05/202	1		1	30/05/202	2
		1	29/05/202	1		1	01/06/202	2
		1	05/07/202	4	TESTE RAP	4		
		1	28/12/202	1		1	29/12/202	9
		1	07/07/202	1		1	18/07/202	2
		1	03/09/202	1		1	03/09/202	9
		1	15/05/202	1		1	15/05/2020	
		1	05/08/202	1		1	09/08/202	2
MODERAD	09/07/202	1	12/07/202	1		5		
TC TORAX	11/06/202	1	10/06/202	1		1	21/06/2020	
		1	17/11/202	1		1	18/11/2020	
		1	19/04/202	1		1	06/05/2020	
		1	16/07/202	1		1	26/07/202	2
VIDRO FOSCO		1	02/04/202	1		1	14/04/202	2
		1	15/04/202	1		1	18/04/2020	
PNEUMOT	21/07/202	1	15/07/202	1		1	16/07/2020	
		1	04/01/202	1		1	04/01/202	2
		1	08/07/202	1		1	10/07/202	2

		1	20/06/2020	1		1	23/06/2020	2
		1	26/12/2020	1		1	29/12/2020	
		1	03/01/202	4	TESTE RAPI	5		
		1	22/12/2020	1		1	23/12/2020	
		1	25/04/2020	1		1	06/05/2020	
OPACIDAD	03/06/2020	1	03/06/2020	1		1	04/06/2020	2
		1	16/06/2020	1		1	16/06/2020	
		1	13/07/2020	1		1	15/07/2020	2
	27/08/2020	1	28/08/2020	1		2	29/08/2020	
		1	18/12/2020	1		1	18/12/2020	
		1	21/12/2020	1		1	26/12/2020	9
	30/09/2020	1	30/09/2020	1		5		
		1	23/12/2020	4	SANGUE	4		
		1	26/04/2020	1		1	27/04/2020	
		1	29/10/2020	1		1	02/11/2020	2
		1	02/07/2020	1		1	15/07/2020	2
		1	10/07/2020	1		1	13/07/2020	
		1	21/05/2020		SWAB NAS	1	25/05/2020	
		1	16/10/2020	1		1	18/10/2020	2
		1	18/04/2020	1		1	27/04/2020	2
		1	21/07/2020	1		1	25/07/2020	2
		1	05/12/2020	1		2	05/12/2020	
		1	08/01/202	1		3	08/01/2021	
	29/09/2020	1	29/09/2020	1		1	01/10/2020	
	21/04/2020	1	21/04/2020	1		1	25/05/2020	
		1	23/11/2020	1		1	29/11/2020	2
		1	10/07/2020	4	SWAB NAS	1	14/07/2020	2
TC DO TOR	23/07/2020	1	23/07/2020	1		1	23/07/2020	2
		1	17/12/2020	1		1	19/12/2020	
	01/01/202	2						
		1	14/05/2020	1		1	19/05/2020	
		1	22/04/2020	1		1	25/04/2020	9
		1	17/06/2020	1		2	24/06/2020	
		1	30/05/2020	4	TESTE RAPI	5		
		1	12/01/2021	1		5		
	13/06/2020	1	08/06/2020	1		1	10/06/2020	2
		1	18/09/2020	2		1	18/09/2020	2
		1	20/11/2020	4	SWAB NAS	1	23/11/2020	2
		1	13/05/2020	1		1	13/05/2020	
		1	01/05/2020	1		1	08/05/2020	
		1	21/05/2020	1		1	22/05/2020	2
		1	24/07/2020	1		1	24/07/2020	9
		1	20/06/2020	1		1	15/07/2020	
		1	17/06/2020	1		1	19/06/2020	2
		1	17/12/2020	1		1	18/12/2020	2
		1	22/12/2020	1		1	22/12/2020	9
		1	31/03/2020	1		1	02/04/2020	

		1	23/05/202	1		1	28/05/2020	
		1	02/06/202	1		1	03/06/2020	
		1	23/06/202	1		1	24/06/202	2
		1	04/07/202	4	TESTE RAP	1	04/07/202	2
		1	20/05/202	4	TESTE RAP	5		
		1	10/08/202	4	TESTE RAP	4		
		1	26/08/202	1		2	28/08/2020	
OPACIDAD	22/03/202	1	22/03/202	1		1	22/03/202	2
		1	09/01/202	1		1	09/01/202	2
		1	20/03/202	1		1	02/04/202	2
		1	16/05/202	1		1	19/05/202	2
		1	18/12/202	1		1	18/12/202	2
		1	03/12/202	1		2	06/12/2020	
		1	27/06/202	1		1	30/06/2020	
	10/12/202	1	10/12/202	4	TESTE RAPIDO			
		1	21/07/202	1		1	22/07/202	9
		1	21/09/202	1		1	25/09/2020	
		1	08/01/202	4	TESTE RAP	5		
		1	01/07/202	1		1	04/07/202	9
		1	17/07/202	1		1	22/07/2020	
	19/07/202	1	14/07/202	1		1	14/07/202	2
		1	12/11/202	4	ASPIRADO	1	16/11/2020	
		1	19/07/202	1		1	26/07/2020	
		1	18/12/202	1				
		2						
		1	28/04/202	1		1	07/05/202	2
		1	16/10/202	1		1	17/10/2020	
		1	17/06/202	1		1	19/06/2020	
	25/06/202	1	20/06/202	1		1	24/06/202	2
		1	22/10/2020			1	27/10/202	9
		1	14/11/202	1		1	14/11/202	9
TC TORAX	06/07/202	1	30/06/202	1		1	01/07/202	9
		1	08/04/202	1		1	03/05/2020	
		1	29/04/202	1		1	14/05/2020	
		2						
		1	13/10/202	4	SWAB NAS	5		
		1	04/05/202	1		5		
		1	23/03/202	1		1	01/04/202	2
	31/05/202	1	01/06/202	1		1	05/06/2020	
		2						
TC	25/06/202	1	23/06/202	1		1	29/06/2020	
		1	08/06/202	4	SANGUE	1	10/06/202	9
	09/07/202	1	09/07/202	1		1	09/07/202	9
		1	12/07/2020			1	13/07/202	9
		1	07/07/202	1		1	09/07/2020	
VIDRO FOS	22/05/202	1	22/05/202	1		1	23/05/202	2
		1	19/08/202	1		1	22/08/2020	

	18/08/202	1	17/08/202	1		1	18/08/202	9
		1	04/11/202	1		1	10/11/202	2
		2						
		1	06/09/202	1		1	10/09/2020	
TC VIDRO F	16/04/202	1	16/04/2020			2	22/04/2020	
	15/04/202	1	15/04/202	1		1	17/04/2021	
VIDRO FOS	06/06/202	1	08/06/202	1		1	09/06/202	2
VIDRO FOS	27/05/202	1	29/05/202	1		1	29/05/2020	
		1	13/09/202	1		1	14/09/202	2
		1	10/11/202	1		1	13/11/2020	
		1	25/06/202	4	SANGUE	5		
	02/04/202	1	03/04/202	1		1	06/04/202	2
	07/07/202	1	30/06/202	1		1	06/07/202	9
		1	17/05/202	4	SWAB NAS	2	22/05/2020	
		1	25/06/202	4	TESTE RAPI	5		
	02/08/202	1	29/07/202	1		1	31/07/2020	
		1	07/08/202	1		1	08/08/202	9
		1	08/05/202	1		1	19/05/2020	
		1	18/12/202	1		2	19/12/2020	
		1	31/10/202	1		1	31/10/202	9
	21/05/202	1	22/05/202	1		1	22/05/202	9
		1	05/05/202	1		1	06/05/202	2
		1	04/06/202	1		1	05/06/202	2
		1	28/05/202	1		1	28/05/2020	
		1	21/08/202	1		4		
VIDRO FOS	20/05/202	1	20/05/202	1		1	21/05/202	9
		1	30/12/202	1		1	03/01/2021	
PNM BILAT	16/04/202	1	16/04/202	1		1	25/04/202	2
CONGESTA	23/06/202	1	20/06/202	1		1	28/06/2020	
		1	16/06/202	1		1	22/06/2020	
		1	11/08/202	1		1	12/08/202	9
		1	13/09/202	1		1	16/09/2020	
		1	31/07/202	1		1	05/08/202	2
		1	07/08/202	1		2	12/08/2020	
AGUARDANDO		1	15/08/202	1		1	17/08/202	2
		1	28/11/202	1		1	28/11/2020	
OPACIDAD	30/09/202	1	26/09/202	1		2	08/10/2020	
		1	24/12/202	1		5		
		1	28/04/202	1		1	29/04/202	9
		1	28/10/202	4	SANGUE	5		
		1	07/05/202	4	TESTE RAPI	5		
		1	09/07/202	1		1	10/07/202	2
	05/08/202	1	05/08/202	1		1	05/08/202	2
	19/05/202	1	20/05/202	1		1	21/05/202	2
	04/06/202	1	05/06/202	1		2	06/06/2020	
		1	04/12/202	1		1	10/12/2020	
	10/08/202	1	12/08/202	1		4		

VIDRO FOS	20/07/202	1	18/07/202	1		2	19/07/2020	
		1	27/09/202	1		2	29/09/2020	
	13/07/202	1	13/07/202	1		2	17/07/2020	
		1	26/12/202	1		1	30/12/202	2
		1	03/06/202	1		1	04/06/202	9
		1	12/05/202	1		1	18/05/202	2
		1	25/10/202	1		1	28/10/202	2
		1	26/08/202	1		1	27/08/202	2
		1	11/12/202	1		1	14/12/202	2
		1	08/12/202	1		1	09/12/2020	
		1	05/09/202	1		1	05/09/202	2
		1	01/09/202	1		2	02/09/2020	
		1	27/08/202	1		2	29/08/2020	
		1	12/09/202	4	TESTE RAPI	1	12/09/202	2
		1	24/10/202	1		1	25/10/202	2
		1	05/05/202	1		1	07/05/202	2
		1	30/05/202	1		1	03/06/202	9
		1	25/08/202	2		5		
	09/04/202	1	15/04/202	1		1	19/04/202	9
		1	25/07/202	1		1	28/07/2020	
		1	05/12/202	1		1	16/12/202	2
		1	27/08/202	1		1	29/08/202	9
		1	26/10/202	1		1	01/11/202	9
		1	31/07/202	1		1	10/08/202	9
		1	20/07/202	1		1	26/07/202	2
		1	26/07/202	1		2	28/07/2020	
	30/07/202	1	24/07/202	1		1	08/08/202	2
		1	09/12/202	1		1	12/12/2020	
		1	08/01/202	1		1	08/01/2021	
	05/06/202	1	05/06/202	1		1	06/06/2020	
	29/04/202	1	01/05/202	1		1	08/05/2020	
		1	28/09/202	1		1	30/09/202	2
OPACIDAD	26/08/202	2						
		1	18/11/202	1		1	18/11/202	2
		1	16/07/202	1		2	18/07/2020	
	25/05/202	1	24/05/202	1		1	27/05/2020	
		1	22/06/202	1		1	23/06/202	9
		1	26/06/202	1		4		
		1	13/07/202	1		1	14/07/2020	
	14/09/202	1	14/09/202	1		1	16/09/202	9
		1	28/08/202	1		2	29/08/2020	
		1	31/12/202	4	TESTE RAPI	1	01/01/202	2
	27/05/202	1	11/06/202	1		1	14/06/202	2
		1	18/08/202	1		1	20/08/2020	
	15/07/202	1	18/06/202	1		1	22/06/202	2
		1	02/09/202	1		1	05/09/202	2
		1	30/09/202	1		1	02/10/202	2

		1	06/05/202	4	TESTE RAPI	5		
	02/08/202	1	22/07/202	1		1	24/07/202	9
		1	30/07/202	4	SANGUE	5		
	12/07/202	1	11/07/202	1		3	16/07/2020	
		1	25/04/202	1		1	25/04/2020	
		1	19/06/202	1		1	22/06/202	2
		1	10/07/202	4	SANGUE	5		
		1	26/09/202	1		1	28/09/2020	
	17/09/202	1	20/09/202	1		1	21/09/202	2
	10/11/202	1	11/11/202	4	SWAB NAS	1	12/11/202	2
		2						
		1	03/11/202	4	TESTE RAPI	4		
		1	09/06/202	1		1	10/06/202	9
TC TORAX		1	19/04/202	1		1	25/04/202	2
	09/06/202	1	13/06/202	4	SANGUE	5		
		1	07/10/202	1		1	15/10/202	2
		2						
		1	03/01/202	1		1	06/01/202	2
		1	08/12/202	1		1	15/12/2020	
		1	02/07/202	1		1	03/07/2020	
	20/04/202	1	21/04/202	1		1	23/05/2020	
		1	21/05/2020			1	23/05/202	2
		1	12/06/202	4	TESTE RAPI	4		
OPACIDAD	01/07/202	1	25/06/202	1		1	25/06/202	9
	19/07/202	1	20/07/202	1		1	23/07/202	2
	15/10/202	1	14/10/202	4	SANGUE	4		
		1	03/11/202	1		1	04/11/2020	
	13/06/202	1	13/06/202	1		1	16/06/202	2
		1	15/12/202	1		2	19/12/2020	
		1	19/05/202	1		1	27/05/2020	
		1	17/04/202	1		1	19/04/202	2
	22/05/202	1	21/05/202	4	TESTE RAPI	1	21/05/202	2
OPACIDAD	04/10/202	1	29/09/202	1		2	09/10/2020	
		1	29/09/202	1		1	05/10/2020	
	10/07/202	1	08/07/202	1		1	08/07/202	2
TC TORAX	24/05/202	1	24/05/202	1		1	04/06/202	2
		1	22/07/202	1		1	26/07/202	9
	12/05/202	1	07/05/202	1		1	12/05/2020	
INFILTRAD	14/11/202	1	05/11/202	4	NASOFARI	1	12/11/202	2
	09/06/202	1	09/06/202	4	SANGUE TE	1	09/06/202	2
		1	11/12/202	1		1	18/12/202	2
		1	30/04/202	1		1	01/05/2020	
		1	29/05/202	1		1	31/05/202	9
		1	13/07/202	1		1	14/07/202	9
	15/07/202	1	15/07/202	1		1	27/07/202	9
		1	11/07/202	1		1	13/07/202	2
DENSIFICA	24/05/202	1	17/05/202	1		1	21/05/202	2

		1	04/06/202	1		1	07/06/202	2
	25/07/202	1	26/07/202	1		1	27/07/202	9
		1	11/07/202	1		1	14/07/2020	
		1	31/08/202	1		1	01/09/202	9
	24/08/202	1	25/08/202	4	SWAB NAS	2	27/08/2020	
		1	13/07/202	1		1	16/07/202	9
		1	02/04/202	1		1	07/04/202	2
	23/04/202	1	24/04/202	1		1	03/05/2020	
		1	25/07/202	1		1	25/07/202	2
OPACIDAD	19/05/202	1	17/05/202	1		1	18/05/202	2
	20/05/202	1	19/05/202	1		1	19/05/2020	
TOMO VIDRO FOSCO		1	16/07/202	1		1	22/07/202	2
		1	03/08/202	1		1	06/08/2020	
VIDRO FOS	22/04/202	1	22/04/202	1		1	28/04/202	2
		1	25/04/202	1		1	29/04/2020	
		1	30/11/202	4	NASOFARI	1	04/12/202	2
		1	21/06/202	1		1	23/06/2020	
		1	29/10/202	1		2	03/11/2020	
	17/05/202	1	16/05/202	1		1	20/05/2020	
	26/05/202	1	25/05/202	1		1	26/05/202	2
	25/06/202	1	21/06/202	1		1	27/06/2020	
	19/08/202	1	21/08/202	1		1	26/08/2020	
	17/12/202	1	12/12/202	1		1	16/12/2020	
	29/09/202	1	26/09/202	1		2	30/09/2020	
		1	07/05/202	1		1	10/05/202	9
	19/06/202	1	06/06/202	1		1	06/06/2020	
		1	15/06/202	1		1	15/06/202	2
		1	24/03/202	1		1	27/03/2020	
		1	05/09/202	1		1	10/09/2020	
		1	16/11/202	1		1	20/11/202	2
		1	15/11/202	1		1	20/11/2020	
		1	27/04/202	1		2	30/04/2020	
	19/06/202	1	18/06/202	1		1	20/06/202	9
		2						
		1	24/06/202	1		1	26/06/202	9
VIDRO FOS	03/06/202	1	07/06/202	4	SECRECAO	1	08/06/202	9
		1	17/07/202	1		1	25/07/202	2
		1	03/06/202	1		1	04/06/202	2
TC, OPACID	05/05/202	1	05/05/202	1		1	11/05/2020	
		1	08/11/202	1		5		
	24/11/202	1	24/11/202	1		1	27/11/2020	
		1	11/12/202	1		1	11/12/202	2
	24/04/202	1	26/04/202	1		1	26/04/202	9
		1	28/04/202	1		1	05/05/202	2
		1	22/06/202	4	TESTE RAP	2	25/06/2020	
		1	02/06/202	1		1	08/06/2020	

		1	26/06/202	1		1	02/07/202	9
		1	28/11/202	1		1	29/11/202	2
	13/04/202	1	13/04/202	4	SECRECAO	1	14/04/202	2
		1	17/09/202	1		1	17/09/2020	
VIDRO FOS	31/03/202	1	01/04/202	1		3	02/04/2020	
		1	12/10/202	1		1	15/10/2020	
		1	07/10/202	1		1	07/10/2020	
	17/10/202	1	20/10/202	1		1	22/10/202	2
		1	20/07/202	1		1	21/07/2020	
		1	02/05/202	1		1	14/05/2020	
VIDRO FOS	16/07/202	1	14/07/202	1		1	17/07/202	2
		1	06/05/202	4	TESTE RAPI	5		
		1	18/06/202	1		1	20/06/202	2
		1	16/04/202	1		1	17/04/202	9
	29/06/202	1	23/06/202	1		1	25/06/202	2
	30/05/202	1	27/05/202	1		1	29/05/202	9
		1	18/04/202	1		1	21/04/202	2
		1	20/12/202	1		2	20/12/2020	
		1	24/11/202	1		4		
	26/04/202	1	29/04/202	4	TESTE RAPI	1	29/05/202	2
		1	17/07/202	1		1	21/07/202	2
		1	28/08/202	1		4		
		1	18/07/202	1		1	21/07/2020	
		1	15/08/202	1		1	19/08/2020	
		1	05/01/202	1		1	07/01/202	2
		1	26/12/202	1		1	26/12/2020	
	29/12/202	1	28/12/202	1		2	29/12/2020	
25% ACOM	05/06/202	1	05/06/202	1		1	12/06/2020	
		1	11/12/202	1		1	14/12/202	9
		1	20/08/202	1		1	20/08/202	2
		1	28/12/202	1		1	28/12/2020	
		1	13/05/202	1		1	13/05/2020	
	23/04/202	1	23/04/202	1		1	27/04/202	9
	13/05/202	1	11/05/202	4	TESTE RAPI	4		
		1	07/05/202	1		1	12/05/202	2
		1	18/05/202	1		1	03/06/202	2
		1	06/06/202	1		1	08/06/202	2
		1	05/06/202	1		1	09/06/202	2
		1	01/01/202	1		1	03/01/2021	
		1	27/06/202	1		2	29/06/2020	
		1	11/08/202	1		2	15/08/2020	
		1	29/07/202	4	SANGUE	5		
		1	05/09/202	1		1	05/09/202	2
		1	02/12/202	1		1	02/12/202	2
		1	02/11/202	1		1	03/11/2020	
		1	04/06/202	1		1	06/06/202	2
		1	03/09/202	4	TESTE RAPI	4		

TC: VIDRO	04/05/202	1	04/05/202	1		1	06/05/2020	
		1	16/06/202	1		1	19/06/2020	
TC DE TOR	06/06/202	1	12/06/202	4	SANGUE	4		
		1	20/07/202	1		1	23/07/202	2
		1	22/10/202	1		1	25/10/202	2
		1	30/08/202	4	SANGUE	5		
		1	27/07/202	1		1	29/07/2020	
		1	05/05/202	1		1	11/05/202	2
		1	24/08/202	1		1	27/08/202	2
		1	09/12/202	1		1	09/12/202	2
	10/10/202	1	10/10/202	1		2	14/10/2020	
TC TORAX	12/06/202	1	12/06/202	1		1	17/06/2020	
		1	16/10/202	1		1	19/10/2020	
		1	09/01/202	1		1	09/01/202	2
	06/06/202	1	06/06/202	1		1	10/06/202	2
		1	03/09/202	1		1	03/09/2020	
		1	18/08/202	1		1	19/08/202	2
		1	10/12/202	1		1	11/02/2021	
		1	03/01/202	1		1	04/01/202	9
		1	25/05/202	1		1	28/05/202	2
		1	19/07/202	1		1	21/07/2020	
S/ INF		2						
		1	16/07/202	1		1	27/07/202	2
	25/08/202	1	24/08/202	1		1	24/08/2020	
		1	14/05/202	1		1	25/05/2020	
		1	27/04/202	1		1	28/04/2020	
	04/06/202	1	05/06/202	1		1	10/06/202	2
		1	02/06/202	1		1	02/06/2020	
INFILTRAD	13/07/202	1	12/07/202	1		1	14/07/202	2
		1	21/08/202	1		1	24/08/202	9
		1	12/12/202	1		1	17/12/2020	
		1	11/01/202	1		1	11/01/202	9
		1	29/05/202	1		1	30/05/202	2
	14/07/202	1	09/07/202	1		1	13/07/202	2
		1	29/11/202	1		1	29/11/2020	
		1	14/10/202	1		4		
		1	23/04/202	1		1	30/04/2020	
		2						
		1	29/04/202	1		1	01/05/202	2
		1	13/06/202	1		1	14/06/202	9
		1	22/08/202	4	TESTE RAPI	4		
	19/10/202	1	19/10/202	1		1	22/10/202	9
		1	02/10/202	1		1	05/10/202	2
		2						
		1	01/10/202	1		1	02/10/2020	
		1	30/12/202	1		1	31/12/202	2
	17/11/202	1	17/11/202	1		1	17/11/2020	

		1	19/05/2020	1		1	21/05/2020	
		1	10/07/2020	1		1	15/07/2020	2
		1	15/12/2020	1		1	22/12/2020	2
		1	15/05/2020	1		1	23/05/2020	2
TOMO TOF	07/07/2020	1	08/07/2020	1		1	16/07/2020	
TC VIDRO FOSCO		1	25/06/2020	1		2	29/06/2020	
		1	24/09/2020	1		1	27/09/2020	2
INFILTRAD	13/03/202	1	05/11/2020	4	SEC. NASO	1	07/11/2020	9
		1	23/05/2020	1		1	31/05/2020	
		2						
	25/04/2020	1	30/04/2020	1		2	01/05/2020	
	11/06/2020	1	08/06/2020	1		1	10/06/2020	2
	26/06/2020	1	25/06/2020	1		1	25/06/2020	2
		1	01/01/202	1		2	04/01/2021	
OPACIDAD	16/09/2020	1	16/09/2020	1		1	17/09/2020	2
		1	04/09/2020	1		2	07/09/2020	
TOMOGRA	22/06/2020	1	15/06/2020	1		1	22/06/2020	2
VIDRO FOS	27/05/2020	1	31/05/2020	4	SANGUE	4		
		1	04/01/202	1		1	04/01/2021	
		1	20/08/2020	1		1	26/08/2020	2
	29/06/2020	1	29/06/2020	1		1	01/07/2020	2
OPACIDADES EM VIDR		1	20/05/2020	1		1	22/05/2020	2
	22/04/2020	1	18/04/2020	9		1	18/04/2020	9
		1	03/08/2020	1		1	03/08/2020	
		1	17/07/2020	1		1	18/07/2020	2
	25/04/2020	1	26/04/2020	1		1	28/04/2020	2
		1	18/07/2020	1		1	18/07/2020	2
	28/06/2020	1	28/06/2020	1		1	02/08/2020	
OPACIFICA	10/05/2020	1	10/05/2020	1		1	11/05/2020	9
	27/07/2020	1	25/07/2020	1		1	27/07/2020	2
OPACIDAD	09/06/2020	1	07/06/2020	1		1	08/06/2020	2
		1	29/10/2020	1		1	31/10/2020	
	24/05/2020	1	23/05/2020	1		1	28/05/2020	2
		1	17/11/2020	1		1	21/11/2020	2
		1	04/01/202	1		1	04/01/2021	
		1	05/07/2020	1		1	05/07/2020	9
		1	23/07/2020	1		1	24/07/2020	
		1	18/05/2020	4	TESTE RAPI	5		
		1	21/12/2020	1		1	22/12/2020	2
	14/04/2020	1	14/04/2020	1		1	16/04/2020	2
AGUARDA	09/04/2020	1	09/04/2020	1		1	09/04/2020	9
		1	28/12/2020	1		1	30/12/2020	2
	05/10/2020	1	04/10/2020	1		1	05/10/2020	
		1	29/04/2020	1		1	12/05/2020	2
		1	08/11/2020	1		1	09/11/2020	9
		1	27/12/2020	1		1	30/12/2020	2

		1	15/05/2020			1	30/05/202	2
		1	08/08/202	1		1	12/08/202	2
	23/05/202	1	28/05/202	1		1	28/05/202	2
		1	17/07/202	1		1	19/07/202	2
		1	27/07/202	1		1	08/08/2020	
		1	23/11/202	1		1	26/11/2020	
	21/08/202	2						
		1	10/12/202	1		1	10/12/2020	
		1	17/11/202	4	SOROLOGI	5		
TOMO TOF	16/04/202	1	16/04/202	1		1	24/04/202	2
		1	19/05/202	1		1	22/05/202	2
	29/03/202	1	29/03/202	1		1	31/03/2020	
		1	12/07/202	1		1	13/07/2020	
	15/05/202	1	18/05/202	1		1	21/05/2020	
	28/05/202	1	01/06/202	4	TESTE RAPI	4		
		1	25/09/202	1		1	25/09/2020	
VIDRO FOS	22/05/202	1	22/05/202	1		1	22/05/202	9
		1	20/03/202	1		1	21/03/202	2
		1	20/07/202	1		1	25/07/202	2
VIDRO FOS	04/06/202	1	07/06/202	4	SANGUE	4		
		1	21/12/202	4	SANGUE	5		
		1	05/07/202	4	SWAB NAS	5		
		1	15/11/202	1		1	18/11/202	2
		1	15/07/202	1		1	18/07/202	2
		1	26/11/202	1		2	01/12/2020	
		1	20/12/202	1		1	20/12/202	2
HIPERTRAI	07/08/202	1	08/08/202	4	SANGUE	5		
		1	16/12/202	1		1	17/12/202	9
TC TORAX		1	03/05/202	1		1	07/05/2020	
		2						
		2						
		1	10/11/202	1		1	13/11/2020	
	09/09/202	1	10/09/202	1		1	13/09/2020	
		1	23/07/202	1		1	28/07/202	2
		1	23/05/202	4	TESTE RAPI	5		
	26/12/202	1	21/12/202	1		1	22/12/2020	
		1	25/05/202	4	SANGUE	5		
	05/04/202	1	21/04/202	1		1	29/04/202	2
		1	21/11/202	4	TESTE RAPI	5		
		1	29/05/202	3		1	31/05/2020	
	26/04/202	1	26/04/202	1		1	30/04/202	9
		1	31/08/202	1		1	01/09/202	9
	11/12/202	1	30/11/202	1		1	04/12/202	2
		1	30/12/202	1		2	06/01/2021	
		1	12/07/202	4	TESTE RAPI	4		
	23/06/202	1	23/06/202	1		1	28/06/2020	
		1	02/05/202	1		1	05/05/2020	

		1	08/10/202	1		1	09/10/2020	
		1	21/11/202	1		2	06/12/2020	
	27/05/202	1	27/05/202	1		1	29/05/202	2
	27/12/202	1	22/12/202	1		1	24/12/202	2
	02/05/202	1	26/04/202	1		1	29/04/2020	
		1	06/11/202	1		2	07/11/2020	
		1	11/08/202	4		4		
	15/07/202	1	15/07/202	1		1	16/07/2020	
		1	03/11/202	1		1	05/11/2020	
		1	06/12/202	1		1	13/12/202	2
		1	26/06/202	1		1	27/06/202	2
	26/11/202	1	23/11/202	1		1	23/11/202	2
		1	27/05/202	1		1	30/05/202	2
OPACIDAD	13/05/202	1	13/05/202	1		1	14/05/2020	
		1	19/08/202	1		1	28/08/2020	
		1	12/11/202	1		1	18/11/202	9
		2						
		1	22/04/202	1		1	24/04/202	2
TOMO CO	01/05/202	1	22/04/202	1		1	24/04/202	2
EXT OPAC	06/12/202	1	06/12/202	1		1	07/12/202	2
		1	06/05/202	1		1	10/05/202	9
	22/06/202	1	22/06/202	1		1	22/06/202	9
		1	02/05/202	1		1	06/05/2020	
		1	16/07/202	1		1	16/07/202	2
		1	04/01/202	1		1	04/01/202	2
		1	09/09/202	1		1	18/09/2020	
		1	05/10/202	1		1	07/10/202	9
		1	03/12/202	1		1	07/12/202	9
		1	01/06/202	1		1	01/06/2020	
	12/08/202	1	08/08/202	1		1	09/08/2020	
		1	01/10/202	1		1	05/10/202	2
	16/11/202	1	16/11/202	1		2	19/11/2020	
		1	11/07/202	1		1	17/07/2020	
	27/04/202	1	27/04/202	1		1	02/05/2020	
OPACIDAD	14/06/202	1	14/06/202	1		1	14/06/202	9
		1	14/08/202	1		1	17/08/2020	
		1	02/01/202	1		1	05/01/202	9
		1	08/09/202	1		1	10/09/202	2
		1	09/11/202	1		1	10/11/202	9
		1	03/01/202	1		4		
		1	07/05/202	1		1	13/05/202	2
		1	01/06/202	1		1	05/06/202	9
		2						
		1	11/12/202	1		1	11/12/2020	
	03/08/202	1	03/08/202	4	SANGUE	1	03/08/2020	
		1	01/05/202	1		1	04/05/202	9

		1	14/07/2020	1		1	15/07/2020	
		1	29/06/2020	1		1	29/06/2020	2
		1	26/05/2020	4	TESTE RAPIDO			
		1	18/05/2020	1		1	22/05/2020	
		1	18/07/2020	1		1	21/07/2020	2
OPACIDAD	27/08/2020	1	24/08/2020	1		1	27/08/2020	9
		1	04/12/2020	1		1	09/12/2020	2
	05/09/2020	1	10/09/2020	1		1	11/09/2020	2
		1	11/11/2020	1		1	02/12/2020	2
		1	07/01/202	1		1	08/01/202	2
		1	06/01/202	1		1	07/01/2021	
		1	08/05/2020	1		1	15/05/2020	
VIDRO FOS	19/05/2020	1	20/05/2020	1		1	25/05/2020	
		1	27/07/2020	1		1	30/07/2020	2
	03/06/2020	1	03/06/2020	1		1	05/06/2020	
		1	05/08/2020	1		1	07/08/2020	2
DERRAME	20/08/2020	1	20/08/2020	1		2	22/08/2020	
		1	14/09/2020	1		2	16/09/2020	
		1	24/12/2020	1		5		
	20/08/2020	1	20/08/2020	1		2	25/08/2020	
	22/04/2020	1	23/04/2020	4	TESTE RAPI	4		
	22/12/2020	1	22/12/2020	1		5		
	02/07/2020	1	02/07/2020	1		1	03/07/2020	2
		1	23/11/2020	1		1	23/11/2020	2
		1	16/07/2020	1		1	21/07/2020	2
TC VIDRO FOSCO		1	28/07/2020	1		1	29/07/2020	
		1	07/08/2020	1		1	11/08/2020	9
		1	19/05/2020	1		1	21/05/2020	2
	08/07/2020	1	06/07/2020	1		1	08/07/2020	2
	16/09/2020	1	16/09/2020	1		4		
	01/12/2020	1	28/11/2020	1		1	30/11/2020	2
	16/04/2020	1	17/04/2020	1		1	25/04/2020	2
	02/04/2020	1	02/04/2020	1		2	04/04/2020	
	08/06/2020	1	06/06/2020	1		1	08/06/2020	2
	01/04/2020	1	02/04/2020	1		1	09/04/2020	
		1	10/11/2020	1		1	11/11/2020	2
	14/08/2020	1	01/08/2020	1		1	03/08/2020	9
		1	12/12/2020	1		1	27/12/2020	2
	27/04/2020	1	26/04/2020	4	ESCARRO	1	29/04/2020	9
		1	10/07/2020			1	11/07/2020	9
		1	15/07/2020	4	TESTE RAPI	4		
		1	18/08/2020	4	SANGUE	4		
		1	24/06/2020	1		1	28/06/2020	2
	06/05/2020	1	06/05/2020	1		1	20/05/2020	2
		1	01/04/2020	1		1	24/05/2020	2
		1	06/12/2020	1		1	06/12/2020	2
		1	27/12/2020	1		1	28/12/2020	

		1	18/06/202	4	TESTE RAPI	4		
		1	23/05/202	1		1	24/05/2020	
		1	21/12/202	1		1	23/12/202	2
		1	27/12/202	1		1	31/12/202	2
		1	05/07/202	4	TESTE RAPI	1	06/07/202	9
	18/07/202	1	20/07/202	1		1	23/07/202	2
		1	26/08/202	4	ASPIRADO	1	31/08/2020	
		1	07/04/202	1		1	09/04/2020	
		1	24/07/202	1		1	24/07/202	2
		1	01/12/202	1		1	01/12/202	2
	08/12/202	1	07/12/202	1		5		
		1	16/08/202	1		1	17/08/2020	
		1	11/04/202	1		1	15/04/202	2
		1	04/05/202	1		1	06/05/2020	
	17/05/202	1	15/05/202	1		1		
	23/06/202	1	23/06/202	1		1	24/06/202	2
		1	28/11/202	4	SANGUE	5		
		1	21/06/2020			1	25/06/202	9
	01/08/202	1	01/08/202	1		1	01/08/202	2
		1	21/10/202	1		1	22/10/2020	
		1	28/12/202	1		1	28/12/202	9
		1	28/07/202	1		1	31/07/202	9
	26/04/202	1	03/05/202	1		1	18/05/2020	
		1	19/05/2020			2	27/05/2020	
		1	28/11/202	1		1	28/11/2020	
		1	23/07/202	1		1	31/07/202	2
	14/05/202	1	09/05/202	1		1	17/05/202	2
	16/04/202	1	15/04/202	1		1	16/04/202	2
		1	24/12/202	1		1	28/12/202	2
		1	06/12/202	1		5		
		1	08/05/202	1		1	13/05/2020	
	22/01/2021						11/01/2021	
		1	15/05/202	1		1	15/05/2020	
	04/08/202	1	04/08/202	1		1	12/08/2020	
		1	16/06/202	1		1	17/06/2020	
		1	05/12/202	1		2	07/12/2020	
		1	12/05/202	1		1	18/05/202	9
	12/07/202	1	13/07/202	1		1	18/07/202	2
		1	29/07/202	1		1	31/07/2020	
		1	30/11/202	4	SWAB NAS	5		
VIDRO FOS	17/06/202	1	12/06/202	1		1	16/06/202	2
		2						
		1	17/08/202	4	TESTE RAPI	4		
		1	30/09/202	1		2	09/10/2020	
		1	12/05/202	1		1	14/05/202	9
	20/06/202	1	15/06/202	1		1	16/06/202	9
		1	01/12/202	1		1	02/12/202	9

		1	24/06/202	4	TESTE RAPI	5		
		1	21/05/202	1		1	26/05/202	2
		1	31/05/202	1		1	02/06/2020	
		1	06/05/202	1		1	07/05/202	9
		1	28/09/202	1		1	01/10/202	2
		1	06/07/202	1		1	18/07/202	9
FOCOS DE	05/08/202	1	04/08/202	1		1	14/08/202	9
		1	28/05/202	1		1	29/05/202	2
		1	08/07/202	1		1	10/07/2020	
		1	09/10/202	1		2	11/10/2020	
		1	01/02/202	1		9		
		1	16/03/202	1		1	16/03/202	2
		1	22/03/202	1		1	23/03/2021	
		1	15/03/202	1		1	19/03/202	2
		1	13/04/202	1		1	13/04/2021	
		1	15/04/202	1		1	16/04/202	2
		1	29/05/202	1		1	29/05/2021	
		1	21/05/202	1		4		
		2						
		1	22/06/202	1		1	25/06/202	2
	28/08/202	1	28/08/202	1		1	29/08/2021	
	02/04/202	1	14/04/202	4	SORO	4		
	06/02/202	1	12/02/202	3		1	15/02/2021	
		1	16/02/202	1		1	17/02/2021	
		1	21/03/202	1		1	21/03/2021	
		1	07/03/202	1		1	08/03/202	2
		1	12/03/202	1		1	12/03/202	2
		1	17/04/202	1		1	20/04/2021	
	24/03/202	1	20/03/202	1		1	20/03/202	2
		1	11/06/202	1		1	14/06/202	2
		1	10/06/202	1		1	11/06/2021	
		1	14/06/202	1		5		
		1	20/09/202	1		1	22/09/202	2
		1	24/05/202	1		1	26/05/2021	
		1	28/12/202	1		1	03/01/202	9
	07/03/202	1	06/03/202	1		1	06/03/2021	
		1	18/03/202	1		1	18/03/2021	
		1	20/03/202	1		5		
		1	28/03/202	1		1	29/03/202	2
		1	30/03/202	1				
	19/03/202	1	18/03/202	1		5		
		1	25/05/202	1		1	25/05/2021	
	30/03/202	1	27/03/202	1		5		
		1	04/06/202	1		1	04/06/2021	
		1	03/07/202	1		4		
	18/07/202	1	10/07/202	1				
		1	05/08/202	1		1	07/08/202	2

22/05/202	1	22/05/202	4	SECRECAO	1	23/05/202		9
	1	31/03/202	1		1	31/03/202		9
	1	03/04/202	1		1	05/04/2021		
27/05/202	1	15/05/202	1		1	16/05/2021		
30/04/202	2							
14/06/202	1	11/06/202	1		1	11/06/202		2
24/03/202	1	25/03/202	1		5			
	1	03/06/202	1		1	04/06/202		2
	1	12/07/202	1		1	12/07/2021		
	1	16/07/202	1		1	16/07/202		2
	1	12/05/202	1		1	14/07/202		9
	1	18/08/202	1		5			
02/09/202	1	26/08/202	1		1	28/08/2021		
	1	13/09/202	1		4			
	1	03/02/202	1		1	05/02/2021		
	1	27/02/202	1		2	03/03/2021		
	1	17/03/202	1		5			
	1	22/03/202	1		1	22/03/202		2
	1	03/04/202	1		1	06/04/202		2
	2							
	1	09/04/202	1		1	09/04/202		2
	1	18/05/202	1		2	22/05/2021		
27/04/202	2							
	1	06/05/202	1		1	06/05/202		2
18/02/202	1	18/02/202	4	TESTE RAPI	5			
23/02/202	1	22/02/202	1		5			
	1	03/06/202	1		5			
	1	05/02/202	1		1	06/02/2021		
	1	27/09/202	1		4			
24/05/202	1	17/05/202	1		1	17/05/202		2
	1	17/03/202	1		1	17/03/202		2
	1	03/09/202	1		1	03/09/2021		
	1	17/03/202	1		1	17/03/2021		
	1	13/09/202	1		1	14/09/2021		
	1	17/09/202	1		5			
	1	02/01/202	4	SWAB NAS	4			
	1	03/02/202	4	ASPIRADO	1	05/02/2021		
	2							
	1	15/03/202	1		1	15/03/2021		
	1	13/03/202	1		1	13/03/202		9
	1	24/04/202	1		1	30/04/202		2
	1	29/04/202	1		5			
	1	24/05/202	1		1	25/05/2021		
	1	02/05/202	1		5			
26/06/202	1	24/06/202	1		1	24/06/202		2
14/06/202	1	06/06/202	1		1	06/06/2021		

		1	23/08/202	1		4		
		1	26/10/202	1		1	28/10/202	2
		1	06/02/202	1		1	20/02/202	2
	25/03/202	1	25/03/202	1		1	25/03/202	9
		1	23/03/202	1		1	24/03/2021	
		1	05/03/202	1		1	05/03/202	2
		1	26/03/202	1		1	06/04/202	2
	26/03/202	1	22/03/202	1		1	25/03/2021	
		1	06/04/202	1		1	06/04/202	2
		1	01/04/2021			1	01/04/2021	
		1	09/04/202	1		1	13/04/202	2
	29/05/202	1	28/05/202	1		1	29/05/202	9
		1	31/05/202	1		1	06/06/202	2
		1	15/06/202	1		1	17/06/2021	
		1	20/06/202	1		1	21/06/202	2
		1	10/08/2021			4		
	26/11/202	1	26/11/202	1		1	26/11/2021	
		1	20/02/202	1		1	24/02/202	2
		1	15/02/202	1		1	15/02/202	2
							03/04/2021	
		1	17/05/202	4	SWAB-NAS	1	19/05/202	2
		1	14/05/202	4	NASOFARI	1	16/05/202	2
	31/05/202	1	31/05/202	1		1	01/06/202	9
		2						
		1	28/06/202	1		1	30/06/202	2
		1	19/06/202	1		1	22/06/202	9
		1	29/06/202	1		1	30/06/202	9
		1	20/07/202	1		1	22/07/2021	
		1	14/07/202	1		9		
		1	05/07/202	1		1	06/07/2021	
		1	10/04/2021			1	12/04/2021	
	30/09/202	2						
		1	07/01/202	1		1	10/01/202	2
		1	19/03/202	1		5		
		1	10/04/202	1				
		1	05/05/202	1		2	05/05/2021	
		1	08/05/202	1		1	10/05/2021	
		1	09/04/202	1		1	14/04/2021	
	05/05/202	1	30/04/202	1		1	30/04/202	2
		1	05/05/2021			1	06/05/202	2
		1	13/05/202	1		1	14/05/2021	
DENSIFICA	20/05/202	1	15/05/202	1		1	17/05/2021	
	16/06/202	1	07/06/202	1		4		
	13/06/202	1	13/06/202	1		5		
	12/07/202	1	05/07/202	1		1	05/07/202	9
		1	03/10/202	1		1	04/10/202	2
		1	14/08/202	1		1	17/08/202	2

DERRAME	09/08/202	1	17/07/202	1		1	18/07/2021	
		1	29/07/202	1		1	31/07/202	2
	12/01/202	1	12/01/202	1		1	13/01/2021	
		1	02/09/202	1		1	03/09/2021	
		1	29/11/202	1		2	29/11/2021	
		1	06/04/202	1		1	06/04/2021	
OPACIDAD	03/03/202	1	28/02/2021			1	02/03/202	9
		1	13/04/202	1		1	14/04/202	2
		1	23/05/202	1		1	29/05/202	2
		1	21/04/202	1		1	21/04/2021	
		1	15/05/202	1		1	16/05/2021	
	15/05/202	1	16/05/202	1		1	17/05/2021	
		1	24/05/202	1		5		
	16/05/202	1	16/05/202	1		5		
		1	18/06/202	1		1	19/06/202	2
		1	31/05/202	1		1	03/06/2021	
		1	09/06/202	1		4		
	03/07/202	1	27/06/202	4	TESTE RAPI	1	27/06/202	2
		1	21/10/202	1		2	23/10/2021	
		1	02/08/202	1		1	04/08/202	2
		1	26/12/202	4	ANTIGENO	5		
		1	19/09/202	1		4		
		1	16/01/202	1		1	20/01/202	2
		1	13/01/202	4	TESTE RAPI	5		
	05/03/202	1	04/03/202	1		1	07/03/202	9
		1	08/03/202	1		1	11/03/202	2
	24/03/202	1	10/03/202	1		1	13/03/2021	
		1	08/04/202	1		1	10/04/2021	
	06/04/202	1	27/03/202	1		4		
		1	13/04/202	1		1	14/04/202	9
		1	08/06/202	4	NASOFARII	5		
		1	29/04/202	1		1	03/05/2021	
		1	28/10/202	1		5		
		1	29/03/202	1		1	29/03/202	2
		1	14/05/202	1		1	15/05/202	2
		1	28/04/202	1		1	28/04/202	2
		1	27/03/202	1		1	30/03/202	2
		1	28/05/202	1		1	28/05/2021	
		1	21/05/202	1		1	25/05/202	2
		1	18/01/202	1		5		
		1	08/05/202	1		4		
	07/07/202	1	08/07/202	1		1	08/07/2021	
		1	07/06/202	1		1	21/06/202	2
		1	02/06/202	1		1	03/06/2021	
		1	25/05/202	4	ANTIGENO	5		
		1	26/06/202	1		5		
		1	12/01/202	1		1	12/01/202	2

	1	15/02/202	1		5		
	1	03/03/202	1		1	03/03/2021	
	1	08/03/202	1		1	09/03/202	9
	1	06/03/202	1		1	06/03/202	2
	1	27/03/202	1		1	29/03/202	2
14/04/202	1	14/04/202	1		1	15/04/2021	
	1	31/03/202	1		1	31/03/202	2
	1	07/06/202	1		4		
	1	23/04/202	4	TESTE RAPI	4		
	1	27/07/202	1		1	28/07/202	2
	1	30/07/202	4	SWAB NAS	1	08/08/202	2
	1	04/04/202	1		1	07/04/2021	
28/11/202	1	29/11/202	1		1	30/11/202	2
	1	31/01/202	1		1	31/01/2021	
	1	30/09/202	4	NASOFARII	4		
07/12/202	1	07/12/202	1		1	07/12/2021	
	1	10/01/2022			4		
	1	21/03/202	1		1	22/03/2021	
	1	08/03/202	4	SANGUE	5		
	1	31/03/202	1		1	31/03/2021	
31/03/202	1	28/03/2021			1	28/03/202	9
	2						
	1	08/03/202	1		4		
23/05/202	1	19/05/202	1		1	22/05/2021	
	1	13/04/202	1		1	14/04/202	2
	1	20/05/202	1		4		
	1	26/03/202	1		1	31/03/202	2
	1	17/04/202	1		1	20/04/202	2
	1	05/05/202	1		1	06/05/2021	
12/05/202	1	11/05/202	1		1	11/05/202	2
	1	29/04/202	1		1	01/05/202	2
	1	20/05/202	1		1	23/05/202	2
	1	31/05/202	1		4		
	1	05/06/202	1		4		
	1	22/06/202	1		1	22/06/202	2
	1	20/07/202	1		1	21/07/2021	
	1	22/05/202	4	TESTE RAPI	5		
	1	17/08/202	1		5		
	1	04/03/202	1		1	04/03/202	2
	1	25/01/202	1		1	28/01/2021	
	1	10/03/202	9		1	10/03/202	2
	1	22/03/202	1		1	23/03/202	9
	1	22/03/202	1		1	23/03/2021	
	1	24/04/202	1		1	24/04/202	9
16/05/202	1	16/05/202	1		1	18/05/2021	
	1	19/05/202	1		1	24/05/202	9

	1	21/05/202	1		1	25/05/202	2
	1	20/05/202	1		1	21/05/2021	
06/06/202	1	07/06/202	1		2	08/06/2021	
	1	03/01/202	1		1	04/01/2021	
	1	26/05/202	1		4		
	1	01/09/202	1		1	01/09/202	2
	1	18/02/202	1		1	20/02/202	2
	1	27/02/202	1		5		
	1	09/03/202	1		1	10/03/2021	
	1	19/05/202	1		4		
	1	27/04/202	1		1	28/04/202	9
	1	01/05/202	1		1	04/05/2021	
	1	06/06/202	1		1	06/06/202	2
06/04/202	1	23/03/202	1		5		
	2						
	1	16/06/202	1		1	24/06/202	2
	1	08/07/202	1		1	10/07/202	2
	1	20/07/202	1		1	20/07/202	2
	1	26/08/202	1		1	26/08/202	2
	1	16/01/202	1		1	16/01/202	2
	1	12/01/202	1		1	12/01/202	2
	1	22/02/202	1				
	1	01/03/202	1		1	02/03/202	2
	1	04/04/202	4		2	16/05/2021	
16/03/202	1	17/03/202	1		1	18/03/2021	
05/03/202	1	05/03/202	1		1	06/03/202	9
	9						
	1	31/03/202	1		1	01/04/2021	
	1	30/03/202	1		4		
	1	13/05/202	1		1	13/05/202	2
	1	19/05/202	1		5		
	1	16/05/202	1		1	21/05/202	9
	1	31/08/202	1		1	01/09/202	2
	1	11/03/202	1		1	18/03/2021	
	1	24/03/202	4				
	1	18/03/202	1		1	19/03/202	2
	1	10/03/202	1		1	11/03/202	2
	1	14/02/202	1		1	15/02/202	2
14/01/202	1	24/01/202	4	TESTE RAPIDO			
	1	22/03/202	1		1	22/03/202	9
16/03/202	1	15/03/202	1		1	16/03/202	2
	1	18/03/202	1		1	19/03/202	9
	1	04/05/202	1		1	07/05/202	9
	1	31/03/202	1		4		
28/04/202	1	23/04/202	1		1	24/04/202	2
16/04/202	1	16/04/202	4	SECRECAO	1	17/04/202	9
	1	25/05/202	1		1	27/05/2021	

		1	11/05/202	1		1	14/05/2021	
		1	24/05/202	1		1	25/05/202	2
		1	25/05/202	1		1	25/05/2021	
		1	19/03/202	1		1	24/03/2021	
		1	19/07/202	1		1	21/07/2021	
		1	19/08/202	1		1	19/08/202	9
		1	19/10/202	1		1	19/10/202	2
	18/02/202	1	18/02/202	1		5		
		1	27/03/202	1		5		
		1	24/03/202	1		1	27/03/202	2
		1	10/03/202	1		1	12/03/2021	
		2						
		1	26/05/202	4	NASOFARI	1	28/05/202	2
		1	23/05/2021			1	25/05/2021	
		1	11/04/202	4	SANGUE (T	5		
		1	16/04/202	1		5		
		1	10/04/202	1		1	11/04/202	2
		1	09/04/202	1		1	13/04/202	2
		1	22/05/2021			1	22/05/2021	
		1	06/04/202	1		1	10/04/2021	
		1	12/05/202	1		1	12/05/202	2
		1	17/06/202	1		4		
		1	06/08/202	1		5		
		2						
		1	22/05/202	1		1	22/05/202	2
		1	16/03/202	1		1	16/03/202	9
	12/03/202	1	11/03/202	1		1	13/03/202	2
		1	11/03/202	4	SANGUE	4		
	01/04/202	1	29/03/202	1		1	31/03/202	2
		1	11/04/202	1		1	12/04/2021	
		1	23/06/202	1		1	23/06/2021	
		1	16/02/202	1		1	16/02/202	2
	31/03/202	1	31/03/202	1		1	01/04/2021	
	06/01/202	1	05/01/202	1		1	11/01/202	2
		1	25/11/202	1		1	27/11/202	9
		1	08/01/202	1		1	13/01/202	2
OPACIDAD	15/01/202	1	13/01/202	1		1	15/01/202	2
		2						
		1	04/04/202	1		1	08/04/202	9
		1	03/03/202	1		1	03/03/202	2
	14/05/202	1	13/05/202	1		1	13/05/202	2
		1	12/04/202	1		1	15/04/2021	
	30/05/202	1	29/05/202	1		5		
		1	31/05/202	4	SWAB NAS	1	03/06/2021	
		1	01/05/202	1		1	01/05/2021	
	09/06/202	1	03/06/202	1		5		

		1	01/06/202	1		1	08/06/202	2
		1	18/05/202	1		5		
		1	07/06/202	1		1	11/06/202	2
		1	19/06/202	1		1	19/06/2021	
		1	28/05/202	1		1	28/05/2021	
		1	08/03/202	1		1	08/03/202	9
		1	26/03/202	1		1	26/03/2021	
		1	29/03/202	1		1	31/03/2021	
		1	22/03/202	1		1	22/03/202	2
		1	11/04/202	1		1	12/04/202	9
		1	29/06/202	1		1	01/07/2021	
		1	16/04/202	1		1	16/04/202	2
		1	09/03/202	1		1	10/03/2021	
	22/05/202	1	09/05/202	4	ANTIGENO			
		1	26/04/202	1		1	26/04/202	2
		1	20/05/202	1		1	21/05/202	2
		1	10/08/202	1		1	11/08/2021	
		1	26/05/202	1		5		
		1	17/02/202	1		1	19/02/202	2
		1	19/02/202	1		1	24/02/202	2
	26/03/202	1	23/03/202	1		1	24/03/202	9
	25/03/202	1	20/03/202	1		1	20/03/202	2
		1	03/05/202	1		1	04/05/202	9
		1	20/04/202	1		1	21/04/2021	
		1	07/05/202	1		5		
	07/06/202	1	06/06/202	4	TESTE RAPI	5		
		1	22/05/202	1		1	22/05/2021	
		1	29/01/202	1		1	30/01/2021	
OPACIDAD	06/06/202	1	06/06/202	1		1	07/06/202	9
		1	13/06/202	1		1	15/06/202	9
		1	31/08/202	1		1	01/09/2021	
		1	05/09/202	1		5		
		1	02/03/202	1		1	04/03/2021	
		1	09/03/202	1		1	12/03/2021	
		1	17/03/202	1		1	18/03/202	2
		1	23/03/202	4	TESTE RAPI	4		
		1	03/06/202	1		1	12/06/202	2
		1	15/04/202	1		1	16/04/202	2
		1	14/05/202	1		1	15/05/202	2
		1	14/06/202	1		1	17/06/2021	
		1	25/05/202	1		1	27/05/202	2
	24/05/202	1	17/05/202	4	NASOFARII	4		
		1	16/06/202	1		1	18/06/2021	
		1	03/06/202	1		1	09/06/202	2
		1	25/05/202	1		1	26/05/202	9
		1	11/07/202	1		4		
		1	23/06/202	1		1	23/06/2021	

		1	29/07/202	1		2	30/07/2021	
		1	02/10/202	1		3	02/10/2021	
		1	12/04/202	1		1	15/04/202	2
	05/02/202	1	17/01/202	4	TESTE RAPI	2	28/01/2021	
		1	16/03/202	1		1	18/03/2021	
		1	19/04/202	1		1	20/04/202	2
		1	14/04/202	1		1	22/04/2021	
		1	29/03/202	1		1	30/03/2021	
		1	30/06/202	1		1	05/07/202	2
		1	29/04/202	1		1	03/05/202	2
		1	26/05/202	1		1	28/05/202	2
		1	19/05/202	1		1	21/05/202	2
		1	20/06/202	1		1	21/06/202	2
		1	09/06/202	1		5		
		1	09/07/202	4		5		
VELAMENT	13/03/202	1	11/03/202	1		1	15/03/2021	
		1	26/04/202	1		1	27/04/2021	
		1	15/04/202	1		1	16/04/2021	
		1	18/05/202	1		1	20/05/2021	
		1	24/03/202	1		1	31/03/2021	
		1	19/05/202	4	SANGUE	4		
		1	04/06/202	1		4		
		1	24/05/202	1		5		
		1	25/07/202	1		1	27/07/202	9
		1	23/07/202	1		5		
	11/07/202	1	11/07/202	1		1	14/07/2021	
		1	30/07/202	1		1	30/07/202	2
	21/12/202	1	22/12/202	4	NASOFARII	1	25/12/202	2
		1	29/03/202	1		1	04/04/202	2
		1	19/04/202	1		1	21/04/2021	
		1	17/04/202	1		1	17/04/2021	
OPACIDAD	09/04/202	1	04/04/202	1		1	11/04/202	2
		1	05/04/202	1		1	05/04/202	2
OPACIDAD	05/05/202	1	04/05/202	1		2	10/05/2021	
		1	11/06/202	1		1	12/06/202	2
		2						
		1	03/06/202	1		1	03/06/202	2
		1	08/06/202	1				
DERRAME	20/07/202	1	23/07/202	1				
		1	02/07/202	1		5		
		1	16/06/202	1		1	19/06/202	2
		1	24/02/202	1		1	25/02/2021	
		1	18/06/202	1		5		
		1	05/01/202	1		1	06/01/2021	
		1	28/06/202	1		4		
		1	27/12/202	1		5		

	20/02/202	1	20/02/202	1		1	22/02/2021	
		1	11/03/202	1		1	15/03/202	2
		1	19/03/202	1		1	19/03/202	9
		1	11/02/202	4	NASOFARII	1	14/02/202	2
		1	13/04/202	1		1	13/04/202	2
		1	03/03/202	1		1	03/03/2021	
		1	27/05/202	1		4		
		1	05/06/2021			4		
	17/05/2021							
		1	15/07/202	1		2	19/07/2021	
	18/11/202	1	19/11/202	1		1	20/11/202	2
	03/04/202	1	02/04/202	1		1	04/04/2021	
		1	15/03/202	1		1	15/03/2021	
		1	26/03/202	4	SECRECAO	1	28/03/202	2
	05/04/202	1	03/04/202	1		1	05/04/202	2
		1	17/03/202	1		1	22/03/202	2
		1	25/04/2021			1	28/04/2021	
		1	01/03/202	1		1	03/03/202	2
		1	06/04/202	1		1	06/04/2021	
		1	12/05/202	1		1	13/05/2021	
	28/06/202	1	19/06/202	1		1	27/07/2021	
		1	23/05/202	1		1	25/05/202	2
		1	06/08/202	1		1	08/08/202	2
		1	21/06/202	1		1	21/06/2021	
		1	25/06/202	1		2	27/06/2021	
		1	04/07/202	1		1	04/07/202	2
		1	27/07/202	1		4		
		1	29/07/202	1		1	29/07/202	2
		1	03/08/202	4		1	05/08/202	2
		1	27/07/202	1		1	28/07/2021	
		1	04/11/202	1		1	04/11/2021	
		1	20/01/202	1		1	20/01/202	2
	20/04/202	1	22/03/202	1		1	24/03/202	2
		1	08/03/202	1		1	09/03/202	2
		1	18/04/202	1		1	19/04/2021	
		1	02/05/202	1		1	04/05/2021	
		1	30/04/202	1		1	02/05/202	2
		1	10/02/202	1		1	12/02/2021	
TOMO		1	31/05/202	1		4		
		1	17/05/202	1		2	21/05/2021	
		1	18/05/202	1		1	20/05/202	2
		1	24/04/202	1		1	25/04/2021	
	22/04/202	1	24/04/202	1		2	28/04/2021	
		1	23/04/202	1		1	25/04/2021	
		1	22/05/202	1		1	24/05/2021	

		1	15/06/202	1		1	16/06/202	9
	14/06/202	1	03/06/202	1		1	05/06/202	2
		1	05/07/202	1		1	06/07/202	2
		1	22/08/202	1		1	23/08/202	2
		1	29/05/202	1		1	01/06/2021	
		1	17/07/202	1		1	18/07/202	2
		1	03/06/202	1		1	04/06/2021	
		1	08/06/202	1		1	08/06/202	2
OPACIDAD	08/07/202	1	08/07/202	1		1	09/07/2021	
		1	03/03/202	1		1	03/03/202	2
		1	17/02/202	1		1	18/02/2021	
		1	05/03/202	1		1	11/03/2021	
	19/03/202	1	12/03/202	1		5		
		1	09/03/202	1		1	13/03/202	2
		1	02/04/202	1		1	07/04/202	2
		1	03/03/202	4	SWAB NAS	1	08/03/202	2
		1	12/05/202	1		1	14/05/202	2
		1	18/05/202	1		1	18/05/202	2
	06/06/202	1	07/06/202	1		1	09/06/2021	
		1	29/07/202	1		1	29/07/2021	
		1	26/06/202	1				
		1	16/07/202	1		1	19/07/202	2
		1	08/07/202	1		1	08/07/202	2
		1	26/02/202	1		1	27/02/2021	
		1	20/03/202	1		1	20/03/2021	
		1	26/03/202	1		4		
		1	25/03/202	1		1	26/03/202	2
		1	05/03/202	1		2	06/03/2021	
		1	29/04/202	2		1	30/04/202	2
		1	12/04/202	1		4		
		1	04/05/202	1		4		
		1	31/03/202	1		1	31/03/2021	
	25/02/202	1	22/02/202	1		5		
SEM INFILT	13/06/202	1	06/06/202	1		1	08/06/202	2
	25/06/202	1	14/06/202	1		1	17/06/2021	
		1	25/06/202	1		1	26/06/2021	
	14/04/2021							
		1	14/06/202	1		2	16/06/2021	
		1	27/05/202	1		1	29/05/2021	
		1	10/03/202	1		1	11/03/202	9
		1	15/08/202	1		5		
	12/05/202	1	12/05/202	1		5		
		1	14/01/202	1		2	16/01/2021	
		1	21/07/202	1		4		
		1	01/09/202	1		1	01/09/2021	
		1	01/01/202	1		1	01/01/2022	
		1	02/04/202	1		1	03/04/2021	

		1	07/04/202	1		5		
	11/04/202	2						
		1	03/05/202	1		1	04/05/2021	
		1	03/05/202	1		1	03/05/202	2
	24/05/202	1	17/05/202	1		1	17/05/202	9
		1	01/06/202	1		5		
PNEUMONIA P/ COVII		1	06/06/202	4	SANGUE	5		
	28/04/202	1	29/04/202	1		1	30/04/2021	
		1	11/06/202	1		1	12/06/2021	
		1	06/06/202	1		1	10/06/202	9
		1	30/05/202	1		1	30/05/2021	
	27/10/202	1	23/09/202	1				
		1	18/08/202	1		4		
		1	21/06/202	1		1	21/06/202	2
		1	15/06/202	1		1	15/06/2021	
		1	25/02/202	1		1	27/02/2021	
INF. DIFUS	05/02/202	1	05/02/202	4	S. NASOFA	1	06/02/202	2
		1	26/02/202	1		1	26/02/202	2
		1	15/03/202	1		1	16/03/2021	
	14/04/202	1	10/04/202	1		1	10/04/2021	
SOLICITAD	03/05/202	1	03/05/202	1		1	06/05/202	2
		1	22/05/202	1		4		
		1	21/05/202	4		1	29/05/2021	
		1	16/05/202	1		1	16/05/202	2
	06/05/202	1	03/05/202	4	TESTE RAPI	5		
	20/07/202	1	20/07/202	4	TR			
		1	30/05/202	1		4		
		1	13/07/202	1		1	13/07/202	2
		1	29/07/202	1		5		
		1	29/09/202	1		1	30/09/2021	
		1	02/08/2021			1	05/08/202	2
		1	05/02/202	1		1	05/02/2021	
		1	12/04/202	1		1	12/04/202	2
	13/04/202	1	03/04/2021			1	06/04/202	9
		1	25/04/202	4	SANGUE	4		
		1	29/05/2021			4		
		1	05/04/202	1		1	05/04/202	2
	02/08/202	1	03/08/202	1		4		
		1	29/07/202	1		4		
		1	19/04/202	4	TESTE RAPI	5		
		1	24/06/202	1		1	25/06/2021	
		1	15/07/202	1		1	19/07/2021	
		1	18/07/202	1		4		
		1	07/02/202	1		1	08/02/202	2
		1	03/03/202	1		1	18/03/2021	
	22/02/202	1	20/02/202	4	NASOFARII	1	24/02/202	2
		1	27/03/202	1		1	27/03/2021	

	1	23/03/202	1		1	23/03/2021	
22/03/202	1	22/03/202	1		1	25/03/202	2
10/06/202	1	28/05/202	1		1	29/05/202	2
	1	24/06/202	1		1	27/06/202	2
	1	17/05/202	1		1	18/05/202	2
	1	22/06/202	1		5		
	1	01/04/202	1		1	03/04/202	9
25/06/202	1	25/06/202	1		1	25/06/202	9
	1	03/07/202	1		1	03/07/202	2
	1	05/07/202	1		4		
07/03/202	1	01/03/202	1		1	04/03/202	2
	1	21/07/202	1		4		
	1	10/04/202	1		1	13/04/2021	
	1	23/04/202	1				
	1	09/03/202	1		1	09/03/202	2
	1	13/05/202	1		1	13/05/202	2
	1	31/05/202	1		1	09/06/202	2
23/05/202	1	22/05/202	1		1	08/06/202	9
	1	25/08/2021			4		
	1	06/07/202	1		1	07/07/202	2
	1	13/06/202	1		1	26/06/2021	
	1	06/07/202	1		5		
05/06/202	1	03/06/202	1		5		
	1	17/03/202	1		1	20/03/2021	
23/03/202	1	24/03/202	1		1	27/03/2021	
	1	05/03/202	1		1	05/03/2021	
	1	26/02/202	1		4		
26/03/202	1	23/03/202	1		1	28/03/202	2
20/04/202	1	09/04/202	1		1	12/04/202	2
	1	25/05/202	1		1	03/06/202	9
	1	26/03/202	1		1	26/03/202	2
	1	10/05/202	1		1	12/05/202	2
	1	17/05/202	4	NASOFARII	5		
	1	08/04/202	1		1	12/04/202	2
	1	09/04/202	1		1	09/04/202	2
22/04/202	1	19/04/202	1		1	19/04/202	2
	1	03/06/202	1		5		
09/05/202	1	09/05/202	1		1	11/05/202	2
	1	21/07/202	1		1	22/07/2021	
	1	15/05/202	1		1	17/05/202	2
	1	27/05/202	1		5		
13/01/202	1	13/01/202	1		1	13/01/202	9
	1	15/08/202	1		4		
	1	09/03/202	1		1	09/03/202	2
	1	18/03/202	1		1	20/03/202	2
	1	18/03/202	1		1	23/03/202	2

		1	05/05/202	1		5		
		1	24/05/202	1		1	25/05/202	2
		1	15/05/202	1		1	17/05/2021	
		1	16/06/202	4	NASOFARII	1	18/06/202	2
	20/05/202	1	25/05/202	4	TESTE RAPI	5		
		1	14/05/202	1		1	16/05/202	2
		2						
	05/07/202	1	03/07/202	1		1	04/07/2021	
TIPICO COVID 19		1	08/09/202	1		5		
		1	08/08/202	1		1	09/08/2021	
	23/06/202	1	15/06/202	1		1	15/06/2021	
		1	29/09/2021			5		
		1	14/06/202	1		1	18/06/2021	
	10/05/202	1	18/04/202	1		5		
		1	24/09/202	1		4		
	13/07/202	1	08/07/202	1		1	08/07/2021	
		1	12/03/202	1		1	14/03/202	2
		1	14/04/202	4	ANTIGENO	4		
		1	18/02/202	1		1	18/02/202	2
OPACIDAD	16/03/202	1	10/03/202	1		1	10/03/202	2
		1	19/04/202	1		2	19/04/2021	
	31/05/202	1	01/06/202	1		1	02/06/202	2
		1	19/04/202	1		1	19/04/202	2
	05/06/202	1	01/06/202	1		1	04/06/202	2
		1	09/06/202	1		1	12/06/202	2
	28/10/202	1	29/10/202	1		1	29/10/202	9
		1	20/02/202	1		1	21/02/202	2
		1	18/03/202	1		4		
		9						
		1	01/03/202	1		4		
		1	03/02/202	1		1	06/02/202	2
		1	22/04/202	1		1	23/04/202	2
		1	31/03/202	1		1	01/04/202	9
	22/03/202	1	22/03/202	1		1	25/03/2021	
		1	12/04/202	1		1	16/04/2021	
		1	10/05/202	1		1	10/05/202	2
		1	23/06/202	1		1	23/06/2021	
		1	24/06/202	4	ANTIGENO	5		
		1	04/08/202	1		1	06/08/202	2
	08/08/202	1	29/07/202	1		1	30/07/2021	
		2						
		1	12/03/202	1		5		
		1	03/04/202	1		1	05/04/202	2
		1	03/04/202	1		1	04/04/202	2
VIDRO FOS	20/03/202	1	17/03/202	1		1	17/03/202	9

21/03/202	1	18/03/202	4	SECRECAO	1	19/03/2021	
	1	27/04/202	1		1	28/04/202	2
	1	18/03/202	4	SWAB NAS	1	18/03/2021	
	1	17/06/202	1		1	18/06/2021	
12/08/202	1	12/08/202	1		1	13/08/202	2
	1	01/07/202	1		1	02/07/202	2
22/07/202	1	16/07/202	1		1	20/07/202	2
	1	10/07/202	1		5		
	1	10/11/202	1		1	11/11/2021	
	1	04/03/202	1		1	04/03/202	2
	1	23/02/202	4	TESTE RAPI	5		
	1	05/03/202	1		1	05/03/2021	
	1	10/03/202	1		1	11/03/2021	
	1	21/03/202	1		2	21/03/2021	
	1	10/03/202	1		1	12/03/2021	
	1	23/04/202	1		1	23/04/2021	
	1	08/03/202	1		1	08/03/202	2
	1	02/04/202	1		1	04/04/2021	
	1	21/05/202	4	NASOFARII	1	21/05/202	2
	2						
	1	24/06/202	1		4		
	1	17/06/202	1		1	18/06/2021	
	1	20/08/202	1		1	21/08/2021	
29/03/202	1	17/03/202	1		1	26/03/202	2
	1	18/02/202	4	SWAB NA U	5		
11/04/202	1	11/04/202	1		1	12/04/202	2
	1	20/04/202	4	TESTE RAPI	5		
	1	20/04/202	1		1	23/04/2021	
	1	16/04/202	1				
	1	01/06/202	1		4		
	1	12/06/202	9		5		
	1	19/06/202	1		1	22/06/202	2
	1	02/12/202	1		5		
03/03/202	1	26/02/202	1		1	27/02/202	2
	1	28/02/202	1		1	28/02/202	2
	1	19/03/202	1		5		
	1	08/04/202	1		1	08/04/202	9
	1	07/03/202	1		1	09/03/202	2
25/04/202	1	13/04/202	1		1	14/04/202	2
	1	18/04/202	1		2	20/04/2021	
	1	19/04/202	4	SWAB NAS	4		
	1	24/04/202	1		1	24/04/202	2
17/05/202	1	17/05/202	1		1	22/05/2021	
	1	12/04/202	1		1	15/04/202	2
	1	03/06/202	1		1	03/06/2021	
	1	14/08/202	1		4		

	1	08/06/202	1		1	10/06/202	2	
	1	20/04/202	1		1	23/04/2021		
	1	23/05/202	1		4			
	1	01/03/202	1		1	01/03/202	2	
	1	19/02/202	1		5			
	1	20/02/202	1		1	20/02/2021		
	1	02/04/202	1		1	03/04/202	2	
	1	02/03/202	1		1	02/03/202	2	
	1	15/06/2021			4			
25/01/202	1	18/01/202	1		1	23/01/202	2	
	1	06/05/202	1		1	07/05/202	2	
	1	28/05/202	1		1	02/06/2021		
	1	23/03/202	1		1	24/03/2021		
	1	30/11/202	1		1	01/12/202	9	
	1	05/07/202	1		4			
	1	07/10/202	1		1	08/10/2021		
	1	02/08/202	1		1	02/08/202	2	
	1	25/08/202	1		1	25/08/2021		
	1	04/02/202	1		1	04/02/2021		
	1	12/02/202	1		1	14/02/202	2	
22/04/202	1	11/04/202	4	ANTIGENO	4			
	1	29/03/202	1		1	03/04/202	9	
	1	14/04/202	1		1	16/04/2021		
	1	23/04/202	1		1	23/04/202	2	
	1	28/04/202	1		1	28/04/2021		
	1	09/06/202	1		1	09/06/202	2	
	1	16/06/202	1		1	17/06/2021		
	1	30/05/202	1		1	31/05/2021		
	1	12/07/202	1		1	14/07/202	2	
	1	04/01/202	1		1	04/01/202	2	
	1	16/01/202	1		1	20/01/202	2	
	1	23/12/202	1		1	11/01/202	1	1
	1	10/04/202	4	NASOFARII	1	10/04/202	9	
	1	10/05/202	1		5			
	1	12/02/202	1		1	16/02/2021		
INFILTRADO BILATERA	1	22/05/202	1		1	29/05/2021		
	1	24/05/202	1		5			
	1	08/06/202	1		1	11/06/202	2	
	1	01/10/202	4	NASOFARII	1	05/10/202	2	
13/03/202	1	10/03/202	1		1	11/03/2021		
	1	21/03/202	1		1	21/03/202	2	
	1	05/04/202	1		1	06/04/202	2	
	1	07/06/202	4	NASOFARII	1	09/06/202	2	
	1	25/05/202	1		4			
	1	06/06/202	1		1	10/06/2021		
	2							
	1	05/06/202	1		1	07/06/202	9	

	1	20/08/202	1		1	21/08/202	9
	1	06/12/202	1		4		
	1	29/10/202	4	TR			
	1	16/08/202	1		5		
	1	21/02/202	1		1	23/02/202	9
18/02/202	1	09/02/202	1		1	09/02/202	2
	1	20/04/202	1		1	23/04/202	2
03/05/202	1	02/05/202	1		1	02/05/202	9
	1	02/06/202	1		1	02/06/2021	
	1	16/04/202	1		1	16/04/202	2
	1	27/07/202	1		1	29/07/202	2
	1	13/05/202	1		1	13/05/2021	
	1	06/09/202	1		1	10/09/202	2
	1	23/03/202	1		1	24/03/202	2
	1	07/06/202	1		1	07/06/2021	
	1	04/02/202	1		1	07/02/2021	
	1	10/02/202	4	SWAB	5		
	1	14/03/202	4	SECRECAO	4		
	1	18/03/202	1		1	18/03/202	9
	1	11/04/202	1		1	13/04/202	2
	1	31/03/202	4	PCR	1	31/03/202	2
28/03/202	1	30/03/202	1		1	01/04/202	2
	1	24/04/202	1		5		
	1	13/05/202	1		1	18/05/202	9
	1	14/03/202	1		1	14/03/2021	
	1	13/03/202	1		1	13/03/2021	
	1	22/06/202	1		1	22/06/202	2
	1	20/06/202	1		1	21/06/2021	
10/06/202	1	09/06/202	4	SORO	1	12/06/202	2
	1	01/05/202	1		1	04/05/202	9
	1	06/05/202	1		1	09/05/2021	
01/04/202	1	26/03/202	1		1	31/03/202	2
	1	10/09/202	1		1	11/09/2021	
	1	24/03/202	1		1	25/03/2021	
16/04/202	1	08/04/202	1		5		
13/02/202	1	13/02/202	1		1	13/02/202	9
03/04/202	1	05/04/202	1		1	05/04/202	2
	1	29/04/202	1		1	07/05/2021	
	2						
	1	11/07/202	1		5		
	1	07/08/202	1		1	07/08/2021	
	1	03/05/202	1		1	04/05/2021	
	1	13/11/202	1		1	16/11/202	2
	1	26/11/202	1		1	28/12/202	2
	1	27/03/202	1		1	28/03/2021	

		1	25/03/202	1		1	25/03/2021	
	25/02/202	1	25/02/202	1		1	03/03/202	2
		1	08/05/202	1		1	09/05/202	9
		1	08/04/202	1		1	09/04/2021	
	28/04/202	1	30/04/202	1		1	01/05/202	2
		1	06/08/202	1		1	06/08/202	2
		1	07/05/202	1		1	09/05/2021	
		1	01/06/202	1		1	02/06/2021	
		1	06/10/202	1		1	06/10/202	9
		1	03/08/202	1		1	04/08/202	9
		1	18/10/202	1		1	21/10/202	2
		1	14/06/202	1		1	17/06/2021	
		1	11/08/202	1		1	12/08/2021	
		1	16/03/202	1		2	16/03/2021	
		1	29/04/202	1		5		
		1	11/04/202	1		1	13/04/2021	
	16/03/202	1	16/03/202	1		5		
	26/05/202	1	28/05/202	1		5		
		1	08/06/202	9		1	08/06/202	9
		1	06/01/202	1		5		
		1	05/02/202	4	TR ANTIGENO			
		1	19/03/202	1		5		
	19/03/202	1	12/03/202	1		1	17/03/202	2
		1	24/03/202	1		1	25/03/2021	
	25/03/202	1	18/03/202	1		4		
		1	20/03/202	1		1	22/03/202	2
	21/03/202	1	21/03/202	1		1	22/03/202	2
	06/05/2021							
		1	12/04/202	2		1	12/04/2021	
TOMOGRAFIA		1	12/06/202	1		1	14/06/202	2
		1	18/05/202	1		1	22/05/202	9
		1	18/06/202	1		1	22/06/202	2
		1	16/06/202	1		4		
	10/06/202	1	14/06/202	1		1	15/06/202	2
		1	16/02/202	1		4		
	20/04/202	1	08/04/202	1		1	15/04/2021	
	09/04/202	1	08/04/202	1		1	09/04/202	2
	12/05/202	1	06/05/202	4	SWAB NAS	1	06/05/202	2
		1	15/06/202	1		1	15/06/202	2
		1	09/06/202	1		1	11/06/2021	
		1	23/06/202	1		4		
		1	07/04/202	1		2	10/04/2021	
		1	10/04/202	1		5		
		1	18/07/202	1		4		
		1	07/06/202	1		2	08/06/2021	
	28/03/202	1	27/03/202	1		5		

		1	07/12/202	1		1	07/12/202	2
		1	01/03/202	1		1	02/03/202	2
	08/03/202	1	26/02/202	1		4		
	04/04/202	1	02/04/202	1		1	02/04/202	2
		1	31/03/202	1		4		
		1	16/04/202	1		1	19/04/202	2
		1	09/05/202	1		1	12/05/202	2
		1	16/05/202	1		1	19/05/202	2
		1	09/05/202	1		1	09/05/202	2
		1	09/06/202	1		1	10/06/2021	
	01/07/202	1	29/06/202	1		4		
		1	06/08/202	1		1	08/08/202	2
		1	18/08/202	1		5		
		1	12/08/202	1		1	17/08/2021	
		1	25/01/202	1		1	27/01/202	2
		1	05/02/202	4	ASPIRADO	1	06/02/2021	
		1	17/01/202	1		1	18/01/2021	
		2						
		1	08/02/202	1		1	09/02/202	2
		1	25/03/202	1		1	29/03/2021	
	29/03/202	1	30/03/202	1		1	01/04/202	2
		1	25/03/202	4	SANGUE	5		
	19/06/202	1	19/06/202	1		1	20/06/202	9
	03/04/202	1	31/03/202	1		1	06/04/2021	
	06/06/202	1	01/06/202	1		1	01/06/202	2
		1	26/03/202	1		5		
		1	04/08/202	1		1	06/08/202	2
		1	31/05/202	1		2	03/06/2021	
		1	10/12/202	4		5		
		1	29/11/202	1		5		
		1	12/03/202	1		1	16/03/202	2
		1	29/03/202	1		1	29/03/202	2
AGUARDAI	16/04/202	1	12/04/202	1		1	12/04/2021	
		1	09/04/202	1		5		
		1	03/05/202	1				
	07/06/202	1	31/05/202	1		1	08/06/2021	
		1	15/03/202	4	TESTE ANT	4		
		1	24/05/202	1		1	24/05/2021	
SEM LAUD	05/06/202	1	03/06/202	1		5		
		1	14/07/202	1		2	15/07/2021	
		1	28/07/202	1		1	29/07/2021	
		1	02/09/202	1		1	03/09/2021	
		1	19/05/202	1		5		
		1	10/02/2021			1	12/02/202	2
		1	11/02/202	1		1	13/02/202	9
		1	20/03/202	1		1	21/03/202	9
		1	17/02/202	1		1	19/02/202	2

		1	29/03/202	1		1	30/03/2021	
		1	19/04/202	1		1	22/04/2021	
	29/03/202	1	18/03/202	1		1	21/03/202	2
		1	26/04/202	1		1	01/05/2021	
	01/05/202	1	30/04/202	1		1	04/05/2021	
	14/05/202	1	09/05/202	1		1	09/05/202	9
		1	28/04/202	1		4		
		1	14/05/202	1		1	17/05/202	2
		1	29/03/202	1		1	29/03/2021	
		1	10/06/202	1		1	11/06/2021	
		1	09/07/202	1		4		
		1	09/05/202	1		2	09/05/2021	
		1	15/03/202	4	TESTE RAPI	5		
		1	08/06/202	1		1	09/06/202	2
		1	02/03/202	1		1	06/03/202	9
		1	21/02/2021			1	21/02/202	9
		1	09/03/202	1		1	10/03/202	2
		1	21/05/202	1		5		
		1	20/03/202	1		1	21/03/2021	
		1	14/03/202	1		1	19/03/2021	
		1	06/05/202	1		1	10/05/2021	
		1	15/05/202	1		1	21/05/202	2
OPACIDAD	22/05/202	1	17/05/202	1		1	19/05/202	2
		1	13/05/202	1		1	14/05/2021	
		1	23/04/202	1		1	24/04/2021	
		1	19/02/202	1		1	22/02/202	2
		1	25/06/202	1		1	27/06/202	2
		1	16/07/202	1		1	17/07/2021	
OPACIDAD	16/08/202	1	06/08/202	1		1	09/08/202	2
	12/03/202	1	10/03/202	1		1	11/03/202	2
		1	29/03/202	1				
		1	05/03/202	1		1	07/03/2021	
		1	12/03/202	4	ANTIGENO	5		
		1	27/04/202	1		1	05/05/2021	
		1	24/07/202	1		5		
		1	27/04/202	1		4		
	28/05/202	1	26/05/202	1		5		
		1	30/12/202	1		2	05/01/2022	
		1	05/01/202	1		5		
		1	14/10/202	1		1	14/10/202	2
		1	03/03/202	1		5		
		1	21/07/202	1		4		
		1	09/02/202	4		1	10/02/202	2
		1	22/03/202	1		1	26/03/2021	
		1	03/03/202	4	TESTE RAPI	4		
		1	09/04/202	1		1	10/04/202	2
		1	24/03/202	1		1	27/03/2021	

	13/05/202	1	04/05/202	1		1	14/05/202	2
	27/04/202	1	22/04/202	1		1	23/04/2021	
		1	17/06/202	1		4		
		1	02/06/202	1		1	03/06/2021	
		1	28/06/202	1		1	01/07/2021	
DENSIFICA	07/07/202	1	07/07/202	1		1	08/07/202	2
		1	18/09/202	1		4		
		1	04/10/202	1		1	07/10/202	2
	21/08/202	1	22/08/202	1		1	23/08/202	2
		1	07/10/202	1		2	10/10/2021	
	19/01/202	1	20/01/2021			1	27/01/202	9
		1	03/03/202	1		1	04/03/2021	
		2						
		1	08/02/202	1		2	11/02/2021	
		1	10/04/202	1		5		
		2						
	26/04/202	1	30/04/202	1		1	30/04/202	9
	16/04/2021							
	24/05/202	1	20/05/202	1		2	20/05/2021	
		1	23/04/202	4	SANGUE	5		
		1	16/05/202	1		1	17/05/202	9
		1	18/03/202	1		4		
		1	29/05/202	1		1	30/05/202	2
	05/05/202	1	30/04/202	1		5		
	02/07/202	1	02/07/202	1		1	02/07/202	2
		1	19/06/202	1		1	19/06/2021	
	08/03/202	1	02/03/202	1		5		
		1	26/05/202	1		1	28/05/2021	
		1	07/06/202	1		1	10/06/202	2
PNEUMOT	04/02/202	1	28/01/202	1		1	29/01/202	2
		1	11/02/202	1		1	13/02/2021	
	14/08/202	1	06/08/202	1		1	06/08/2021	
		1	09/08/202	1		4		
	26/04/202	1	24/04/202	1		1	26/04/202	2
		1	07/04/202	1		2	08/04/2021	
		2						
	15/06/202	1	09/06/202	1		1	10/06/202	9
		1	18/03/202	1		1	20/03/2021	
	15/05/202	1	15/05/202	1		4		
		1	17/05/202	4	TR	5		
VIDRO FOS	28/05/202	1	28/05/202	1		1	29/05/2021	
		1	14/06/202	1		1	16/06/2021	
		1	31/08/202	1		5		
		1	03/11/202	1		1	08/11/202	2
		1	26/05/202	1		4		
		1	22/05/202	1		1	22/05/2021	

	1	12/07/202	1		4		
	1	03/03/202	1		1	04/03/2021	
	1	25/02/202	1		1	25/02/202	9
	1	21/04/202	1		2	22/04/2021	
	1	16/03/202	1		1	16/03/202	2
	1	30/05/202	1		1	30/05/202	9
	1	09/06/202	1		1	10/06/202	2
24/06/202	2						
	1	31/05/202	1		4		
	1	13/05/2021			1	13/05/2021	
	1	01/08/202	1		1	01/08/202	2
	1	09/07/202	1		1	11/07/2021	
	1	06/08/202	4	SANGUE	4		
	1	13/08/202	1		4		
	1	03/03/202	1		1	03/03/2021	
	1	26/04/202	1		1	27/04/202	2
	1	20/03/202	1		1	24/03/2021	
	1	25/05/202	1		2	28/05/2021	
	1	22/06/202	1		1	24/06/2021	
	1	18/06/202	1		1	21/06/202	2
	1	15/06/202	1		1	15/06/202	9
	1	21/06/202	1		1	21/06/202	2
	1	18/06/202	1		4		
	1	03/07/202	1		1	03/07/202	2
	1	30/11/202	1		1	30/11/202	2
24/03/2021							
05/12/202	1	27/11/202	1		4		
	1	29/07/202	1		1	05/08/202	2
	1	25/02/202	1		1	27/02/2021	
	1	25/03/202	4	NASOFARII	2	30/03/2021	
02/04/202	1	01/04/202	1		1	02/04/202	9
	1	22/03/202	1		1	23/03/202	9
	1	19/05/202	1		1	22/05/202	2
	1	22/07/202	4	TESTE RAPI	4		
	1	15/06/202	1		1	16/06/202	9
	1	30/05/202	1		1	30/05/202	2
	1	21/04/202	1		4		
	1	13/06/202	1		1	13/06/2021	
	1	29/03/202	1		1	31/03/202	2
	1	16/03/202	1		1	21/03/2021	
	1	28/01/202	1		1	30/01/202	2
	1	19/01/202	1		1	20/01/2021	
	1	04/06/202	1		1	05/06/202	9
	1	02/05/202	1		1	05/05/2021	
	1	26/05/202	1		1	26/06/202	2
	1	12/05/202	1		4		
08/02/202	1	05/02/202	1		1	11/02/2021	

	1	04/04/202	1		1	05/04/2021	
	1	19/05/202	1		4		
	1	08/05/202	1		1	09/05/202	9
27/03/202	1	20/03/202	1		1	28/03/2021	
	1	06/04/202	1		5		
	1	13/07/202	1		1	13/07/202	2
PULMAO DIREITO CON	1	07/02/202	1		1	08/02/2021	
	1	25/04/202	1		5		
	1	03/05/202	1		9		
	1	18/01/202	1		1	18/01/2021	
	1	25/02/202	1		1	01/03/2021	
16/03/202	1	16/03/202	1		1	17/03/202	9
	1	03/03/202	4	SANGUE	5		
	1	09/04/202	1		1	16/04/202	2
07/07/202	1	07/07/202	1		1	10/07/2021	
	1	05/05/202	1		1	08/05/2021	
	1	27/03/202	1		1	29/03/202	9
	1	16/05/202	1		1	18/05/2021	
	1	04/01/202	1		1	06/01/2022	
	1	07/02/202	1		5		
24/03/202	1	16/03/202	1		1	17/03/202	2
	1	25/05/202	1		1	26/05/202	9
	1	23/06/202	1		4		
	1	08/03/202	1		1	09/03/2021	
	1	17/08/202	1		1	17/08/2021	
	1	14/07/202	1		4		
	9						
	1	04/08/202	4	SECRECAO	1	04/08/202	2
	1	06/08/202	4	TESTE RAPIDO			
	1	08/01/2022			4		
	1	25/03/202	1		1	25/03/2021	
	1	22/09/202	1		5		
	1	15/02/202	1		1	17/02/202	2
09/03/202	1	11/03/202	1		1	13/03/202	2
	1	23/05/202	1		1	26/05/202	2
	1	12/05/202	1				
01/06/202	1	29/05/202	1		1	30/05/202	9
	1	07/05/202	1		1	11/05/202	2
	1	11/05/202	1		5		
	1	20/07/202	1		1	20/07/202	2
	1	02/07/2021			1	06/07/2021	
	1	25/05/202	1		1	29/05/202	2
	1	10/12/202	1		1	16/12/202	2
	1	05/07/202	1		1	09/07/202	9
	1	05/03/202	1		1	12/03/202	2
	1	11/08/202	1				

		1	05/03/202	1		1	05/03/2021	
		1	07/06/2021			1	07/06/202	9
OPACIDAD	07/07/202	1	23/06/202	1		1	23/06/202	2
		1	05/07/202	1		1	07/07/202	2
	10/04/202	1	11/04/2021			1	11/04/202	9
		1	03/07/202	1		1	06/07/202	2
		1	25/08/202	4	NASOFARII	4		
		1	10/05/202	1		1	13/05/202	2
		1	05/11/202	1		2	06/11/2021	
		1	24/04/202	1		1	26/04/202	2
		1	09/12/202	1		1	10/12/2021	
		1	06/04/202	1		1	06/04/202	2
	13/03/202	1	10/03/202	1		5		
		1	05/05/202	1		1	06/05/2021	
OPACIDAD	26/05/202	1	26/05/202	1		1	26/05/2021	
		1	03/06/202	1		1	07/06/202	2
		2						
	09/08/202	1	03/08/202	1		1	08/08/202	9
		1	31/12/202	1		1	07/01/202	2
INFILTRAD	08/04/202	1	06/04/202	1		1	08/04/202	2
	17/04/202	1	17/04/202	1		1	20/04/2021	
		1	19/03/202	1		1	19/03/202	2
		1	10/07/202	1		1	10/07/202	9
		1	25/01/202	1		1	30/01/202	9
		1	13/04/202	1		1	18/04/202	2
		1	01/03/202	1		1	03/03/202	9
		1	24/02/202	1		1	25/02/202	2
		1	22/02/202	1		1	22/02/202	2
		1	11/03/202	1		1	19/03/202	2
	07/03/202	1	07/03/202	1		1	08/03/202	9
		1	04/06/202	1		4		
		1	03/08/202	1		1	06/08/2021	
	23/05/202	1	28/05/202	1		2	28/05/2021	
		1	20/10/202	4	SWAB NAS	1	25/10/202	2
		1	04/06/2021			4		
		1	27/12/202	1				
		1	15/08/202	4	SWAB NAS	5		
		1	21/08/202	1		5		
		9						
		1	30/08/202	1		1	10/09/202	2
		1	28/01/202	1		1	29/01/2021	
		1	06/03/2021			1	06/03/2021	
		1	18/03/202	1		1	18/03/2021	
		1	21/02/202	1		1	21/02/2021	
		1	23/05/202	1		1	25/05/2021	
		1	19/04/202	1		1	22/04/202	2
		1	18/05/202	1		1	18/05/2021	

	18/04/202	1	17/04/202	1		1	19/04/202	2
		2						
		1	11/04/202	1		1	14/04/2021	
		1	11/04/202	1		1	11/04/202	2
		1	20/03/202	1		5		
	17/07/202	1	17/07/202	1		1	18/07/202	2
		1	02/07/202	1		1	14/07/202	2
		1	22/08/202	1		1	24/08/202	2
		1	16/03/202	1		1	18/03/2021	
		1	10/01/202	1		2	13/01/2021	
		1	20/08/202	1				
	25/02/202	1	23/02/202	1		1	23/02/202	9
	13/05/202	1	08/05/202	1		1	08/05/2021	
	23/03/202	1	22/03/202	1		1	22/03/2021	
		1	22/04/202	1		1	22/04/2021	
		1	16/06/202	1		1	16/06/2021	
		1	09/04/202	1		1	09/04/202	2
	30/03/202	1	19/03/202	4	SANGUE/P	5		
OPACIDAD	13/05/202	1	09/05/202	1		1	09/05/202	2
		1	11/08/202	1		1	21/08/202	9
		1	04/07/202	1		5		
	17/05/202	1	12/05/202	1		1	14/05/202	2
		1	11/08/202	1		1	13/08/202	2
OPACIDAD	11/08/202	1	31/07/202	1		4		
	15/08/202	1	11/08/202	1		1	11/08/2021	
		1	21/05/202	1		5		
		1	18/02/202	4	TESTE RAPI	1	18/02/202	2
		1	09/03/202	1		1	09/03/2021	
		1	26/02/202	9		1	26/02/202	2
		1	05/06/202	4		5		
		1	25/03/202	1		1	30/03/2021	
		1	29/06/202	1		1	30/06/202	2
		1	16/05/202	1		1	17/05/2021	
		1	23/03/202	1		1	26/03/202	2
		1	21/05/202	1		1	23/05/2021	
		1	20/04/202	1		1	20/04/202	2
	19/09/202	1	20/09/202	4	SWAB NAS	2	22/09/2021	
		1	22/02/202	1		1	24/02/202	2
		1	02/06/202	1		1	03/06/202	2
		1	06/04/202	4	SANGUE TO	1	12/04/202	2
		1	20/09/202	1		1	23/09/202	2
		1	06/07/202	1		4		
		1	24/05/202	1		1	25/05/202	2
		1	26/10/202	1		1	27/10/2021	
		1	31/07/202	1		4		

		2						
		1	12/02/202	1		1	15/02/202	9
	30/05/202	1	22/05/202	1		1	24/05/202	2
	23/03/202	1	19/03/202	1		1	20/03/2021	
		1	22/03/202	1		1	23/03/202	2
	07/03/202	1	08/03/202	1		1	10/03/202	2
		9						
		1	10/06/202	1		4		
		1	07/06/202	1		1	08/06/2021	
	22/05/202	1	21/05/202	1		1	22/05/202	2
		1	01/06/202	1		5		
	20/05/202	1	18/05/202	1		5		
		1	02/04/202	1		1	04/04/2021	
	06/06/202	1	28/05/202	1		4		
		1	22/08/202	1		1	23/08/202	2
		1	23/03/202	1		1	23/03/2021	
		1	22/03/202	1		4		
		1	09/06/202	1		1	15/06/202	2
		1	22/03/202	1		5		
		1	15/07/202	1		1	17/07/202	2
		1	01/03/202	1		1	02/03/202	2
	17/03/202	1	17/03/202	1		1	17/03/202	2
	06/03/202	1	08/03/202	1		5		
		1	09/04/202	1		1	12/04/2021	
	22/03/202	1	22/03/202	1		5		
		1	22/06/202	1		1	23/06/202	9
		1	10/08/202	1		5		
	21/04/202	1	19/04/202	1		1	22/04/2021	
		1	13/03/202	1		1	18/03/202	2
		1	21/05/202	1		4		
		1	01/03/202	1		1	02/03/2021	
		1	26/03/202	1		1	29/03/202	2
		1	09/04/202	1		1	09/04/2021	
		1	26/03/202	1		1	31/03/202	2
		1	17/03/202	1		1	17/03/2021	
		1	30/03/202	1		1	30/03/202	2
		1	03/07/202	1		1	03/07/202	2
		1	09/06/202	1		1	11/06/2021	
	02/04/202	1	03/04/202	1		1	15/04/2021	
		1	26/03/202	1		1	29/03/202	2
	10/05/202	1	09/05/202	1		5		
		1	09/04/202	4	TESTE RAPI	5		
AGUARDA	30/04/202	1	21/04/202	1		1	23/04/202	2
		1	11/07/202	1		1	11/07/2021	
		1	26/05/202	1		1	26/05/202	9
		1	01/09/202	1		1	02/09/202	2

		1	01/01/202	1		5		
		1	14/05/202	1		1	15/05/2021	
		1	27/12/202	1		4		
		1	11/05/202	1		1	24/05/202	2
		1	18/04/202	1				
		1	14/04/202	1		1	14/04/202	2
		1	26/05/202	1		1	26/05/202	2
		1	12/05/202	1		1	15/05/202	2
		1	10/06/202	1		1	10/06/202	2
		1	11/06/202	1		2	12/06/2021	
		1	18/06/202	4	ANTIGENO			
		1	08/01/202	1		1	11/01/2021	
	02/07/202	1	30/06/202	1		1	30/06/202	2
		1	13/10/202	1		5		
	07/06/202	1	02/06/202	1		4		
		1	12/07/202	1		2	14/07/2021	
		1	29/06/202	1		1	30/06/2021	
OPACIDAD	05/04/202	1	26/03/202	1		1	27/03/202	2
		1	10/02/202	1		1	14/02/202	2
	16/02/202	1	16/02/202	4	SWAB NAS	1	17/02/202	2
		1	04/03/202	1		1	06/03/202	2
		1	30/12/202	1		1	12/01/202	2
		1	21/03/202	1		1	24/03/2021	
LESOES INF	25/05/202	1	13/05/202	4	TR ANTIGENO			
		1	25/05/202	1		4		
		1	02/03/202	1		1	03/03/202	2
	09/05/202	1	29/04/202	1		1	02/05/202	2
		1	08/03/202	1		4		
OPACIDAD	17/06/202	1	23/06/202	1		1	23/06/202	2
		2						
		1	19/07/202	1		1	21/07/202	2
	20/06/202	1	21/06/202	1		1	22/06/2021	
		1	04/09/202	1		1	04/09/2021	
		1	10/05/202	1		1	13/05/202	2
		1	02/08/202	1		4		
		1	09/11/202	1		1	11/11/202	2
		1	26/03/202	1		1	30/03/2021	
		1	11/01/202	1		1	22/01/202	2
		1	22/02/202	1		1	24/02/202	2
		1	05/04/202	1		1	05/04/202	2
	01/04/202	1	22/03/202	1		1	22/03/202	2
		1	28/01/202	1		1	30/01/2021	
COND. PUL	24/06/202	1	22/06/202	1		1	23/06/2021	
		1	20/05/2021			4		
		1	28/05/202	1		1	28/05/2021	

	1	28/07/202	1		1	29/07/2021	
	1	01/08/202	1		1	01/08/202	2
	1	17/08/202	1		1	17/08/202	2
	1	23/01/202	4	ANTIGENO	4		
	1	26/03/202	1		1	31/03/2021	
	1	07/03/202	1		1	07/03/202	2
	1	11/02/202	1		1	11/02/202	2
	1	16/08/202	1		4		
17/05/202	1	17/05/202	1		5		
	1	14/04/2021			1	15/04/2021	
	1	25/07/202	1		1	25/07/202	2
	1	31/07/202	1		1	31/07/2021	
	1	22/08/202	1		5		
	2						
23/03/202	1	22/03/202	1		1	27/03/202	9
	1	01/05/202	1		1	03/05/202	2
	1	21/05/202	1		1	21/05/202	2
07/04/202	1	01/04/202	1		1	04/04/2021	
	1	09/08/202	1		2	11/08/2021	
	1	05/05/202	1		1	05/05/202	2
14/07/202	1	06/07/202	1		1	07/07/202	2
	1	15/03/202	1		1	15/03/2021	
	1	18/05/202	1		1	19/05/202	2
	1	08/03/202	1		5		
	1	06/04/202	1		4		
25/06/202	1	31/05/202	1		1	03/06/2021	
	1	29/05/202	1		5		
	1	12/05/202	1		1	12/05/2021	
	1	31/03/202	1		1	01/04/2021	
	1	22/06/202	1		1	23/06/2021	
	1	14/06/202	1		1	23/06/202	2
	1	10/09/202	1		5		
24/02/202	1	16/02/202	1		1	19/02/202	2
	1	29/09/202	1		4		
07/03/202	1	05/03/202	4	TESTE RAPI	5		
	1	17/03/202	1		5		
	1	30/03/202	1		1	01/04/2021	
	1	17/04/202	1		1	18/04/2021	
08/09/202	1	08/09/202	1		1	10/09/2021	
	1	10/06/202	4	NASOFARII	5		
	1	15/04/202	1		5		
	1	14/03/202	1		1	22/03/202	2
	1	30/07/202	1		4		
	1	22/08/202	4	SWAB NAS	4		

		1	06/08/202	1		1	07/08/202	2	
		1	15/07/202	1		1	16/07/202	9	
		1	09/07/202	4	SECRECAO	4			
		1	20/04/202	1		1	21/04/202	2	
		1	29/01/202	1		1	03/02/202	2	
		1	25/05/202	1		4			
		1	29/03/202	1					
		1	11/03/202	1		1	11/03/202	2	
INFILTRAD	15/04/202	1	12/04/202	4	SEC. NASO	1	13/04/202	9	
		1	23/02/202	1		1	24/02/2021		
		1	09/03/202	1		1	10/03/2021		
	10/03/202	1	26/02/202	1		1	03/03/2021		
		1	04/09/202	1		4			
		1	28/03/202	1		1	30/03/2021		
		1	22/10/202	4	TESTE RAPI	2	25/10/2021		
	03/04/202	1	24/03/202	1		1	26/03/202	2	
		1	13/02/202	4	SOROLOGI	5			
OPACIDAD	21/04/202	1	14/04/202	1		5			
		1	16/04/202	4	SWAB NAS	1	22/04/202	2	
		1	28/05/202	1		1	31/05/2021		
		1	23/03/202	1		1	24/03/202	2	
		1	20/09/202	1		1	22/09/202	2	
	19/05/202	1	16/05/202	1		5			
	07/07/202	1	04/07/202	1		1	06/07/202	2	
		1	06/05/202	1		1	07/05/202	2	
		1	23/08/202	1		4			
		1	18/05/202	1		5			
		1	12/07/202	1		1	12/07/2021		
	03/07/202	1	04/07/202	1		1	06/07/202	2	
	11/06/202	1	08/06/202	1		1	09/06/202	2	
		1	01/12/202	1		1	01/12/202	1	1
	05/04/202	2							
		1	23/02/202	4	SECRECAO	1	23/02/202	2	
		1	31/03/202	1		1	01/04/2021		
		1	01/05/202	4	SWAB NAS	2	04/05/2021		
		1	23/05/202	1		1	23/05/2021		
		1	09/05/202	1		1	12/05/202	2	
		1	03/05/202	1		1	03/05/202	9	
		2							
		1	18/06/202	1		1	18/06/2021		
		1	05/04/202	1		1	07/04/202	2	
		1	12/07/202	1		1	12/07/202	2	
		2							
	15/06/202	1	15/06/202	1		1	17/06/2021		
OPACIDAD	18/04/2021								
		1	22/01/202	1		1	25/01/202	2	
		1	03/07/202	1		1	03/07/2021		

		1	24/04/202	1		1	24/04/2021	
		1	05/03/202	1		1	07/03/202	2
		1	04/10/202	1		5		
		1	06/03/202	1		1	06/03/2021	
		1	18/06/202	1		1	18/06/202	9
		1	06/04/202	4	S. NASOFA	1	11/04/202	2
		1	16/07/202	1		1	16/07/202	9
		1	03/06/202	1		4		
		1	22/02/202	1		1	25/02/202	9
	21/05/202	1	22/05/202	4	SECRECAO	2	22/05/2021	
		1	28/06/202	1		1	30/06/202	9
	04/06/202	1	04/06/202	1		1	05/06/202	9
		1	11/07/202	1		4		
		1	18/07/202	1		1	19/07/2021	
		1	28/05/202	1		1	02/06/2021	
		1	30/07/202	1		4		
	08/06/202	1	31/05/202	1		1	03/06/2021	
		1	13/07/202	1		1	19/07/202	2
		1	04/07/202	1		1	07/07/202	9
		1	17/08/202	4	S. NASOFA	1	25/08/202	2
		1	04/09/202	1		4		
	12/05/202	2						
		1	26/01/202	1		1	26/01/202	2
LESOES INF	03/11/202	1	24/10/202	4	TR ANTIGENO			
		1	07/04/202	1		1	08/04/202	2
		1	20/01/202	1		1	22/01/202	2
		1	03/04/202	4	TESTE RAPI	5		
	03/03/202	1	02/03/202	1		1	03/03/202	9
		1	21/05/202	1		9		
		1	11/06/202	1				
		1	30/03/202	1		4		
OPACIDAD	01/04/202	1	02/04/202	4	TESTE RAPI	4		
	15/06/202	1	03/06/202	1		5		
		1	18/05/202	1		1	20/05/202	2
	12/06/202	1	13/06/202	1		1	13/06/202	9
	20/05/202	1	20/05/202	4	TESTE RAPI	5		
		1	23/06/202	1		1	23/06/202	2
		1	30/09/202	4	TESTE RAPI	4		
		1	09/04/202	1		1	12/04/202	2
		1	26/03/202	1		1	26/03/202	2
		1	26/04/202	1		1	26/04/2021	
		1	03/03/202	1		1	03/03/202	2
		1	02/02/202	1		1	05/02/202	2
		1	18/06/202	1		1	18/06/202	2
	04/08/202	1	28/06/202	1		4		
VIDRO FOS	15/06/202	1	31/05/202	1		5		
		1	19/07/202	1		1	19/07/202	2

	1	18/03/202	1	1	27/03/202	2
	1	23/02/202	1	1	23/02/202	2
24/09/202	1	24/09/202	1	1	24/09/202	2
	1	26/05/202	1	1	28/05/202	9
30/06/202	1	09/06/202	1	1	10/06/202	2
	1	17/02/202	1			
	1	03/03/202	1	1	04/03/202	2
23/04/202	1	23/04/202	1	2	23/04/2021	
	1	19/04/202	1	4		
	1	16/07/202	1	4		
22/04/202	1	16/04/202	1	1	17/04/202	2
	1	18/05/202	1	1	19/05/202	2
24/05/202	1	16/05/202	1	1	16/05/202	9
	1	22/05/202	1	1	25/05/202	2
	1	19/05/202	1	1	21/05/2021	
	1	20/06/202	1	1	22/06/202	2
	1	01/08/202	1	1	02/08/202	2
	1	01/07/202	1	1	01/07/202	2
	1	20/09/202	1	1	20/09/202	2
21/09/202	1	15/09/202	1	1	17/09/2021	
	1	24/04/202	1	1	29/04/2021	
	1	26/04/202	1	1	28/04/202	2
	1	20/05/202	1	1	20/05/2021	
31/03/202	1	16/03/202	1	4		
04/03/202	1	27/02/202	1	2	09/03/2021	
	1	11/03/202	1	1	13/03/202	2
	1	28/05/202	1	1	30/05/2021	
	1	14/04/202	1	4		
14/04/202	1	05/04/202	1	1	05/04/2021	
	1	03/05/202	1	1	04/05/2021	
05/09/202	1	01/09/202	1	4		
	1	10/06/202	1	4		
	1	17/08/202	1	1	24/08/2021	
	1	10/03/202	1	1	10/03/202	9
	1	30/03/202	1	5		
04/04/202	1	27/03/202	1	4		
25/03/202	1	25/03/202	1	1	26/03/2021	
	1	24/02/202	1	1	27/02/202	9
	1	17/04/202	1	1	17/04/2021	
	1	02/03/202	1	5		
22/04/202	1	21/04/202	1	5		
02/06/202	1	01/06/202	1	1	03/06/2021	
10/05/202	1	10/05/202	1	1	11/05/202	2
	1	12/05/202	1	1	12/05/202	9
	1	07/05/202	1	5		
	1	18/08/202	1	5		
	1	08/07/202	1	4		

ATELECTAS	31/07/202	1	31/07/202	1		1	31/07/202	2
		1	29/04/202	1		1	01/05/202	2
VIDRO FOS	30/06/202	1	30/06/202	1		5		
		1	23/03/202	1		1	27/03/2021	
		1	02/08/202	9		1	02/08/202	2
		1	30/04/202	1		1	01/05/202	2
		1	04/09/202	4	NASOFARII	5		
SEM LAUD	21/03/202	1	21/03/202	1		1	21/03/202	2
		1	03/09/202	1		1	04/09/202	9
		1	21/04/202	1		1	22/04/202	2
		1	13/10/202	1		1	14/10/202	2
		1	23/03/202	1		1	24/03/2021	
		1	18/04/202	1				
	19/04/202	1	05/04/202	1		4		
		1	03/03/202	1		1	04/03/202	2
		1	12/04/202	1		1	12/04/2021	
		1	27/03/202	1		4		
	25/03/202	1	18/03/202	1		1	23/03/2021	
		2						
		1	02/03/202	1		1	05/03/2021	
		1	19/09/202	1		1	19/09/202	2
		1	06/04/202	1		1	06/04/2021	
	08/11/202	1	07/11/202	1		1	09/11/202	2
		1	07/01/202	1		1	08/01/202	2
		2						
	20/03/202	1	15/03/202	1		1	20/03/2021	
		1	09/02/202	1		1	09/02/202	2
		2						
		1	22/07/202	1		4		
	19/05/202	1	17/05/202	1		1	18/05/202	2
		1	25/03/202	1		1	29/03/202	9
	10/05/202	1	27/04/202	1		1	03/05/202	2
		1	14/06/202	1		1	14/06/2021	
	16/05/202	1	17/05/202	1		1	18/05/202	9
	11/04/202	1	11/04/202	2		1	12/04/202	2
		1	02/03/202	1		1	05/03/202	2
		1	06/05/202	1		1	08/05/2021	
	24/05/202	1	22/05/202	4	SECRECAO	1	23/05/202	9
		1	09/08/202	1		1	10/08/202	2
		1	15/01/202	4	ANTIGENO	4		
	31/03/202	1	31/03/202	1		1	03/04/2021	
OPACIDAD	26/06/202	1	19/06/202	1		2	21/07/2021	
		1	29/01/202	1		1	31/01/202	2
OPAC.DIFL	27/02/2021							
	20/03/202	1	15/03/202	1		4		
		1	05/03/202	1		1	05/03/2021	
		1	25/05/202	1		1	26/05/2021	

	05/04/202	1	05/04/202	1		5		
	30/03/202	1	05/04/202	1		1	08/04/202	2
		1	12/07/202	1		1	15/07/202	2
		1	09/08/202	1				
		1	27/03/202	1		1	31/03/2021	
		1	06/03/202	1		5		
		1	22/06/202	1		1	24/06/202	2
		1	01/04/202	1		1	01/04/2021	
		1	09/03/202	1		1	09/03/2021	
		1	17/03/202	1		1	20/03/2021	
	29/03/202	1	25/03/202	1		1	25/03/2021	
	11/07/202	1	02/07/202	1		1	02/07/202	2
		1	10/04/202	1		1	10/04/2021	
		1	23/06/202	1		1	23/06/202	2
		1	31/07/202	1		1	31/07/2021	
OPACIDAD	10/05/202	1	03/05/202	1		1	05/05/202	2
		1	22/04/202	1		1	24/04/202	2
		1	21/06/202	1		4		
		1	13/05/202	1		1	15/05/2021	
		1	11/04/202	4	SANGUE	5		
		1	19/05/202	1		4		
	10/07/202	1	10/07/202	1		1	11/07/202	9
	06/08/202	1	06/08/202	1		1	10/08/202	2
		1	31/05/202	1		1	31/05/2021	
		1	07/05/202	1		1	08/05/2021	
		1	29/05/202	1		4		
		1	11/03/202	1		1	12/03/2021	
		1	08/04/202	1		1	08/04/202	9
		1	12/03/202	1		1	16/03/2021	
		1	30/04/202	1		4		
		1	14/04/2021			4		
		1	03/05/202	1		1	04/05/202	2
		1	19/05/202	1		1	20/05/202	2
		1	15/06/202	1		4		
	08/06/202	1	09/06/202	1		1	09/06/202	9
	19/04/202	1	13/04/202	1		1	14/04/202	2
	22/04/202	1	23/04/202	1		5		
		1	15/04/202	1		1	15/04/2021	
AG		1	24/07/202	1		1	25/07/202	2
	17/02/202	1	12/02/202	1		1	12/02/202	2
SEM LAUD	20/02/202	2						
		1	02/02/202	1		1	03/02/2021	
	29/05/202	1	27/05/202	1		5		
		1	18/02/202	1		1	19/02/2021	
		1	26/01/202	1		1	27/01/2021	
	02/04/202	1	19/03/202	1		4		
		1	07/04/202	1		1	09/04/2021	

		1	19/01/202	1		2	22/01/2021	
		1	27/05/202	1		5		
		1	17/06/202	1		1	22/06/2021	
		1	19/02/202	1		1	19/02/202	2
		1	12/04/202	4	TESTE RAPI	5		
	30/04/202	1	29/04/202	4	TESTE RAPIDO			
		1	14/09/202	1		1	14/09/2021	
		1	04/06/202	1		1	04/06/202	9
		1	16/03/202	1		1	20/03/202	2
		1	12/05/202	1		1	14/05/202	2
OPACIDAD	13/07/202	1	05/07/202	1		1	08/07/202	9
		9						
		1	14/01/202	1		1	15/01/2021	
		1	07/09/202	1		1	08/09/2021	
		1	16/05/202	1		1	21/05/202	2
		1	06/06/202	1		1	06/06/202	2
		1	07/06/202	1		1	09/06/202	2
		1	23/06/202	1		1	23/06/2021	
		1	21/02/202	1		1	22/02/202	2
		1	18/08/202	1		1	18/08/202	9
		1	06/02/202	1		5		
		2						
		1	01/09/202	4	NASOFARI	1	03/09/202	2
		1	26/04/202	1		1	27/04/202	2
	15/04/202	1	15/04/202	1		1	15/04/2021	
		1	26/08/202	1		1	26/08/2021	
		1	11/05/202	1		5		
		1	20/07/202	4	TESTE RAPI	4		
		1	26/08/202	1		1	30/08/2021	
PNEUMON	24/07/202	1	18/05/202	1		1	02/06/2021	
		1	01/06/202	1		1	07/06/202	2
	05/11/202	1	28/10/202	4	ASPIRADO	4		
		1	18/03/202	1		1	18/03/2021	
		1	31/03/202	1		1	01/04/2021	
		1	04/05/202	1		1	09/05/202	9
		1	23/03/202	1		1	26/03/2021	
		1	25/05/202	1		1	28/05/2021	
		1	27/05/202	1		1	29/05/202	2
		1	10/07/202	1		1	10/07/202	2
		1	02/05/202	4	SWAB NAS	1	08/05/202	2
		1	18/04/202	1		1	19/04/202	2
		1	03/06/202	1		1	03/06/202	2
							26/04/2021	
		1	27/06/202	1		4		
		1	01/07/202	1		1	02/07/202	2
		1	04/08/202	1		1	06/08/2021	

		1	23/03/202	1		1	24/03/202	9
	07/04/202	1	30/03/202	1		1	17/04/2021	
		2						
		1	07/02/202	4	SWAB NAS	5		
		1	27/03/202	1		1	27/03/2021	
	03/05/202	1	21/04/202	1		1	22/04/202	2
		1	31/03/202	1		1	31/03/202	9
		1	26/04/202	1		1	28/04/202	2
	18/04/202	1	14/04/202	1		1	14/04/2021	
		1	02/06/202	1		1	04/06/202	2
		1	31/05/202	1		1	31/05/202	9
		1	27/05/202	1		5		
		1	08/04/202	1		1	10/04/2021	
		1	25/06/202	1		1	30/06/202	2
CONGESTA	30/05/202	1	30/05/202	1		1	31/05/2021	
		1	08/05/202	1		1	12/05/202	2
		1	07/05/202	1		1	08/05/202	2
		2						
	10/07/202	1	08/07/202	1		4		
	26/05/202	1	21/05/202	1		1	23/05/2021	
		1	29/03/202	1		1	31/03/202	9
		1	29/03/202	1		1	29/03/202	2
	18/05/202	1	18/05/202	1		5		
		1	04/06/202	1		1	14/06/202	2
AGUARDA	30/03/202	1	25/03/202	1		1	29/03/202	2
		1	23/02/202	1		1	27/02/202	9
		1	03/05/202	1		4		
		1	27/05/202	1		1	28/05/2021	
	20/04/202	1	14/04/202	1		1	15/04/202	9
		1	07/05/202	1		1	07/05/2021	
		1	29/03/202	1		1	29/03/2021	
		1	11/09/202	4	SECRECAO	5		
	21/03/202	1	20/03/202	1		5		
		1	17/05/202	4		5		
		1	03/06/202	1		1	07/06/2021	
		1	27/09/202	1		1	29/09/202	2
	18/03/202	1	18/03/202	1		1	25/03/202	2
		1	11/08/202	1		1	12/08/2021	
		1	07/03/202	1		1	08/03/2021	
		1	03/04/202	1		1	03/04/202	2
		1	14/01/202	1		1	14/01/2021	
		1	26/04/202	1		4		
	15/06/202	1	10/06/2021			1	11/06/2021	
		1	14/05/202	1		1	14/05/2021	
		1	21/11/202	1		1	22/11/202	9
		1	05/07/202	1		1	08/07/202	2

	06/05/202	1	01/05/202	1		4		
		1	02/08/202	1		4		
		1	28/05/202	1		1	02/06/2021	
		2						
		1	14/08/202	1		4		
		1	15/02/202	4	TESTE RAPI	5		
		1	20/02/202	1		1	23/02/202	2
		1	27/06/202	1		1	27/06/2021	
		1	03/03/202	1		1	04/03/202	2
		1	14/05/202	1		1	14/05/2021	
		1	04/10/202	1		1	06/10/202	2
	01/04/202	1	01/04/202	1		1	02/04/202	2
	27/05/202	1	19/05/202	1				
		1	17/01/202	1		1	17/01/202	9
	09/05/202	1	06/05/202	1		1	07/05/202	2
	26/05/202	1	26/05/202	4	TR ANTICO	5		
	07/04/202	1	04/04/202	1		1	04/04/2021	
		1	10/06/2021			4		
	30/05/202	1	19/05/202	1		1	19/05/202	9
	13/04/202	1	11/04/202	1		5		
		1	14/05/202	1		1	17/05/2021	
OPACIDAD	16/04/202	1	16/04/202	1		1	18/04/202	2
	28/05/202	1	26/05/202	4	TESTE RAPI	5		
		1	01/06/202	1		1	04/06/202	2
	19/08/202	1	19/08/202	1		1	20/08/202	2
		1	09/09/202	1		1	13/09/202	2
		1	13/05/202	4	SANGUE	5		
		1	11/05/202	1		1	12/05/2021	
		1	24/06/202	1				
		1	13/06/202	4	TESTE RAPI	5		
		1	19/01/202	1		5		
		1	01/03/202	4				
		1	09/03/202	1		1	18/03/202	2
		1	25/03/202	1		1	26/03/2021	
		1	08/05/202	1		4		
		1	15/05/202	1		1	18/05/2021	
	25/06/202	1	24/06/202	1		5		
		1	28/05/202	1		1	29/05/202	2
	28/04/202	1	25/04/202	1		1	26/04/2021	
		1	17/08/202	1		1	17/08/202	2
		1	14/08/202	1		1	14/08/202	2
	13/07/202	1	13/07/202	1		5		
		1	11/03/202	1		1	12/03/202	9
		1	16/05/202	1		2	18/05/2021	
		1	17/03/202	1		1	19/03/2021	
		1	16/03/202	1		1	16/03/202	2
		2						

	1	03/05/202	1		1	05/05/2021	
18/06/202	1	14/06/202	1		1	14/06/202	2
	2						
	1	25/05/202	1		1	28/05/2021	
	1	19/07/202	1		9		
15/11/202	1	13/11/202	1		1	16/11/202	2
	1	19/05/202	1		4		
	1	30/11/202	1		1	03/12/202	9
	1	02/10/202	4	S. NASOFA	1	03/10/202	2
	1	13/04/202	4	TESTE RAPI	9		
	1	11/04/202	1		1	12/04/202	9
	1	20/04/202	4	TESTE RAPI	5		
12/04/202	1	22/03/202	1		1	23/03/202	2
	1	27/05/202	4	ANTIGENO	5		
10/03/202	1	10/03/202	1		1	12/03/202	2
26/06/202	1	21/06/202	1		1	29/06/202	2
	2						
	1	06/06/202	1		1	07/06/2021	
	1	28/05/202	1		1	30/05/2021	
	1	17/05/202	1		4		
	1	05/01/202	1		1	06/01/202	2
	1	02/03/202	1		1	07/03/202	2
	1	25/02/202	1		1	02/03/202	2
	1	26/02/202	1		4		
	1	16/03/2021			1	16/03/202	2
25/03/202	1	21/03/202	1		1	21/03/202	2
	1	25/04/202	1		2	25/04/2021	
	1	07/05/202	1		5		
	1	22/04/202	1		4		
	1	22/04/202	1		1	22/04/2021	
	1	21/10/202	1		5		
	1	08/07/202	1		1	09/07/202	9
25/03/202	1	25/03/202	1		5		
	1	16/03/202	1		5		
18/03/202	1	12/03/202	1		1	12/03/2021	
	1	18/04/202	1		1	18/04/202	2
	1	22/02/202	1		1	22/02/202	2
16/06/202	1	18/06/202	1		3	19/06/2021	
	1	07/07/202	1		5		
	1	26/05/202	1		1	26/05/202	2
	1	14/09/202	1		1	14/09/202	2
05/01/202	1	05/01/202	1		1	07/01/2022	
09/03/202	1	08/03/202	1		2	10/03/2021	
	1	22/03/202	1		1	23/03/202	2
	1	30/03/202	1		1	31/03/2021	
	1	17/08/202	1		4		

CONGESTA	12/04/202	1	06/04/202	1		1	08/04/202	2
		1	12/05/202	1		1	14/05/202	2
	18/06/202	1	08/06/202	1		1	10/06/202	9
		1	26/03/202	1				
		1	06/05/202	1		1	07/05/2021	
		1	05/05/202	1		1	13/05/202	2
		1	10/03/202	1		5		
		1	15/01/202	1		1	16/01/2021	
		1	13/04/202	1		1	14/04/202	2
		1	31/12/202	1		1	31/12/202	2
		1	01/06/2021			1	02/06/2021	
	02/06/202	1	04/06/202	1		1	07/06/2021	
	13/04/202	1	12/04/202	1		1	12/04/202	2
	17/03/202	1	17/03/202	1		1	17/03/202	9
		1	16/07/202	1		1	17/07/202	2
	01/06/202	1	07/06/202	1		1	09/06/202	9
		2						
		1	19/05/202	1		1	19/05/202	9
		1	23/07/202	1		5		
		1	03/02/202	1		1	04/02/202	9
		1	29/12/202	1		1	29/12/202	2
		1	05/03/202	1		1	08/03/2021	
		1	09/03/202	4	TESTE RAPI	5		
		1	22/03/202	1		1	26/03/2021	
		1	28/05/202	1		1	28/05/202	2
		1	03/03/202	1		2	23/03/2021	
CONDENSA	24/03/202	1	24/03/202	1		1	26/03/202	9
		1	12/04/202	4	TESTE RAPI	5		
		1	22/03/202	1		2	24/03/2021	
		1	20/08/202	4	TESTE RAPI	5		
		1	08/03/202	1		1	08/03/202	2
		1	30/05/202	1		1	30/05/202	2
	16/07/202	1	05/07/202	1		1	06/07/202	2
		1	19/01/202	1		1	19/01/2021	
		1	15/06/202	4	NASOFARII	1	17/06/202	2
		1	25/07/2021			1	28/07/202	2
	01/04/2021							
	29/03/202	1	03/04/202	1		1	05/04/2021	
		1	28/05/202	1		1	06/06/202	2
		1	04/03/202	1		1	05/03/202	9
		1	21/06/202	1		4		
		1	06/06/202	1		1	09/06/202	2
		1	26/03/202	1		1	26/03/202	2
		2						
		1	29/04/202	1		1	29/04/2021	

		1	05/09/202	4	NASOFARII	4		
		1	30/07/202	1		5		
		1	11/01/2021			1	20/01/202	2
		1	17/02/202	1		1	18/02/202	2
		1	16/02/202	1		1	18/02/202	2
		1	05/03/202	1		1	05/03/2021	
		1	22/02/202	1		2	22/02/2021	
		2						
PULMOES (	25/03/202	1	24/03/202	1		1	27/03/2021	
		1	16/04/202	1		1	18/04/202	2
		1	07/07/202	1		1	14/07/2021	
		1	23/05/202	1		4		
		1	02/07/202	1		5		
		1	06/06/202	1		1	06/06/2021	
		1	11/08/202	1		1	12/08/202	9
		1	03/03/202	1		1	04/03/202	2
		1	27/09/202	1		5		
		1	26/04/202	1		1	27/04/202	2
		1	05/03/202	9		5		
	08/05/202	1	04/05/202	1		1	04/05/202	2
		1	31/05/202	1		1	03/06/202	2
	03/06/202	1	07/06/202	1		1	09/06/202	2
		1	21/05/202	1		1	21/05/2021	
		1	18/03/202	1		1	18/03/2021	
		1	10/06/202	1		1	11/06/202	2
	10/05/202	1	11/05/202	1		1	14/05/2021	
		1	08/03/202	1		1	10/03/202	2
	11/02/202	1	04/02/202	1		1	05/02/202	2
		1	26/10/202	1		5		
		1	01/01/202	1		1	05/01/2022	
		1	24/04/202	1		1	28/04/202	2
		1	16/03/202	1		4		
		1	22/03/202	1		1	22/03/202	2
	27/03/202	1	24/03/202	1		1	25/03/202	9
LESOES INF	09/10/202	1	17/08/202	4	TR ANTIGENO			
		1	06/04/202	1		1	06/04/2021	
		1	08/04/202	1		2	09/04/2021	
		1	10/08/202	1		1	12/08/2021	
		1	18/03/202	1		1	18/03/202	9
		1	27/01/202	1		1	31/01/2021	
OPACIDAD	05/03/202	1	18/02/202	1		1	20/02/202	2
		1	03/01/202	1		1	06/01/2022	
		1	22/09/202	1		5		
		1	09/03/202	1		1	11/03/2021	
	19/05/202	1	17/05/202	1		1	21/05/202	9
		1	04/04/202	1		1	05/04/202	2
		1	06/02/202	1		9		

	1	05/05/202	4	TESTE RAPI	5		
	1	22/04/202	1		1	22/04/202	2
	1	11/08/202	4	ANTIGENO	5		
	1	20/08/202	1		1	21/08/2021	
21/03/202	1	21/03/202	1		1	22/03/202	2
	1	17/06/202	1		1	17/06/202	2
	1	03/10/202	1		1	03/10/202	2
	1	07/04/202	1		1	08/04/202	2
	1	01/02/202	1		1	12/02/2021	
	1	18/03/202	1		1	23/03/2021	
	1	13/04/202	1		1	16/04/202	9
	1	15/03/202	1		1	17/03/2021	
	1	13/12/202	1		1	13/12/202	2
	1	22/04/202	1		1	22/04/2021	
	1	21/05/202	1		1	21/05/2021	
	2						
27/03/202	1	26/03/202	1		1	27/03/202	9
	1	25/01/202	1		1	25/01/202	2
	1	06/04/202	1		1	06/04/202	2
	1	12/06/202	4	SECRECAO	1	13/06/202	9
	1	16/05/202	1		1	17/05/2021	
	1	17/07/202	1		4		
	1	10/03/202	1		1	10/03/2021	
	1	16/03/202	1		1	17/03/2021	
	1	29/05/202	1		1	29/05/202	2
	1	14/06/202	4	SECRECAO	1	14/06/2021	
15/05/202	1	12/05/202	1		2	13/05/2021	
	1	29/07/202	1		1	29/07/2021	
	1	01/02/202	1		1	02/02/2021	
	1	17/05/202	1		4		
	1	05/06/202	1		4		
	1	10/06/202	1		1	11/06/202	2
	1	20/04/202	1		5		
	1	09/06/202	1		1	16/06/2021	
	1	18/02/202	1		1	21/02/2021	
24/02/202	1	24/02/202	1		5		
	1	24/09/202	1		1	24/09/202	2
	1	29/03/202	1		4		
	1	06/05/202	1		1	08/05/2021	
	1	01/09/202	1		5		
26/09/202	1	26/09/202	1		1	28/09/202	2
	1	22/06/202	4	NASOFARII	1	22/06/202	2
	1	31/05/202	1		1	31/05/202	2
	1	06/08/202	9		1	09/08/202	2
	1	15/03/202	1		1	19/03/202	2
	2						
	1	29/08/202	1		5		

13/03/202	1	14/03/202	1		1	16/03/2021	
	1	26/04/202	1		1	26/04/202	2
	1	27/09/202	1		1	04/10/202	2
	1	04/05/202	1		5		
	1	16/05/202	1		1	17/05/202	2
	1	11/05/202	1		1	12/05/2021	
	1	15/07/202	1		1	16/07/2021	
14/06/202	1	13/06/202	1		1	14/06/202	9
01/04/202	1	02/04/202	1		1	03/04/202	9
12/07/202	1	30/06/202	1		4		
	1	01/05/202	1		1	01/05/202	2
	1	29/11/202	1		1	29/11/202	2
	1	19/07/202	1		5		
	1	26/01/202	1		2	27/01/2021	
	1	27/09/202	1		4		
12/03/202	2						
	1	05/05/202	1		1	08/05/2021	
	1	05/03/202	1		1	08/03/2021	
16/05/202	1	16/05/202	1		1	20/05/2021	
20/03/2021							
	2						
	1	04/04/202	1		1	07/04/202	2
11/04/202	1	07/04/202	1		1	07/04/202	2
	1	26/04/202	1		1	30/04/2021	
27/03/202	1	21/03/202	1		1	22/03/2021	
23/03/202	1	24/03/202	4	SWAB NAS	1	31/03/202	9
24/03/202	1	16/03/202	1		1	16/03/202	9
	1	12/02/202	1		1	17/02/202	2
	1	19/03/202	1		1	22/03/202	2
	1	23/05/202	1		5		
	1	28/07/202	1		1	28/07/202	2
	1	31/03/202	1		2	08/04/2021	
30/03/202	1	23/03/202	1		1	25/03/202	2
16/04/202	1	15/04/202	1		4		
	1	25/03/202	1		1	25/03/2021	
	1	03/04/202	1		1	04/04/202	2
06/02/202	1	06/02/202	1		2	12/02/2021	
	1	24/02/202	1		1	24/02/202	2
	1	24/09/2021			5		
	1	30/06/202	1		5		
	1	18/04/202	1		1	19/04/202	2
16/06/202	1	14/06/202	1		4		
19/07/202	2						
	1	24/01/202	1		1	26/01/202	2
	1	25/12/202	1		1	25/12/2021	
	1	29/03/202	1		1	31/03/202	2
	1	15/04/202	1		2	15/04/2021	

		1	14/06/202	1		1	15/06/2021	
		1	03/03/202	1		1	04/03/202	2
	25/05/202	1	25/05/202	1		1	28/05/2021	
		2						
	12/06/202	1	12/06/202	1		1	13/06/202	2
		1	25/11/202	1		1	26/11/202	2
		1	19/05/202	1		1	21/05/202	2
		1	14/03/202	1		1	18/03/2021	
		1	05/04/202	1		1	06/04/2021	
		1	25/06/202	1		1	29/06/202	2
	16/03/202	1	16/03/202	1		1	18/03/2021	
		1	08/06/202	1		1	10/06/2021	
		1	28/02/202	1		1	28/02/202	2
	14/03/202	1	20/03/202	1		1	20/03/2021	
	15/05/202	1	15/05/202	1		4		
		1	17/06/202	1		4		
		1	14/05/202	1		1	18/05/2021	
	15/07/202	1	08/07/202	1		5		
		1	16/03/202	1		1	16/03/202	2
		1	24/05/202	1		1	24/05/2021	
		1	18/05/202	1		5		
	14/06/202	1	31/05/202	1		1	31/05/202	2
		1	14/01/202	1		1	15/01/2021	
		1	16/03/202	1		1	16/03/2021	
		1	29/03/202	1		1	30/03/2021	
	09/05/202	1	09/05/202	4	SECRECAO	1	09/05/202	9
		1	21/06/202	1		1	22/06/2021	
		1	30/06/202	1		1	02/07/202	9
		1	09/01/202	1		1	12/01/202	2
		1	16/02/202	1		1	18/02/202	2
		1	25/04/202	1		1	26/04/2021	
		1	17/05/202	1		1	18/05/202	2
		1	20/04/202	1		1	21/04/202	2
		1	18/06/202	1		1	19/06/2021	
		1	11/07/202	1		1	11/07/202	2
		1	11/07/202	4	NASOFARII	1	14/07/202	2
		1	13/07/202	1		1	13/07/202	2
		1	11/09/202	1		1	15/09/202	2
		1	22/06/202	4	TESTE RAPI	4		
		1	20/03/202	1		1	21/03/2021	
		1	10/05/202	1		1	10/05/2021	
		1	25/05/202	1		1	26/05/202	9
		1	24/06/202	1				
		1	03/06/202	1		1	04/06/2021	
		1	27/05/202	1		4		
OPACIDAD	16/04/202	9						
	31/07/202	1	31/07/202	1		2	03/08/2021	

		1	31/03/202	1		1	03/04/2021	
	12/03/202	1	12/03/202	1		1	13/03/2021	
	02/03/202	1	10/03/202	1		1	10/03/2021	
		1	21/02/202	1		1	22/02/202	2
		1	29/03/202	1		4		
		1	04/05/202	1		1	07/05/202	2
		1	08/10/202	4	NASOFARI	1	11/10/202	2
	27/12/202	1	19/12/202	1		1	21/12/202	2
		1	27/02/202	1		1	02/03/202	2
		1	15/03/202	1		5		
	10/04/202	1	25/03/202	1		5		
		1	05/05/202	1		1	13/05/2021	
		1	24/06/202	1		1	25/06/202	2
		1	28/06/202	1		2	28/06/2021	
		1	01/06/202	4	SECRECAO	1	03/06/202	2
		1	24/07/202	1		4		
	21/05/202	1	18/05/202	4	TESTE RAPI	5		
		1	25/08/202	1		1	28/08/202	2
		1	01/12/202	1		5		
		1	18/01/202	1		1	24/01/2021	
		1	31/03/202	1		1	02/04/202	2
		1	08/04/202	1		1	09/04/2021	
		1	05/06/202	1		1	06/06/202	2
		1	31/07/202	4	SWAB NAS	4		
		1	29/03/202	1		1	30/03/202	9
	29/03/202	1	20/03/202	4	SWABS DE	1	22/03/202	2
		1	30/04/202	1		1	04/05/202	2
		1	05/05/202	1		1	05/05/202	2
		1	28/03/202	1		1	29/03/2021	
		1	03/05/202	1		1	04/05/202	9
	24/03/202	1	19/03/202	1		4		
	22/03/202	2						
		1	29/03/202	1		1	30/03/2021	
AGUARDA	09/05/202	1	27/04/202	1		1	27/04/202	2
		1	12/05/202	1		1	12/05/2021	
	22/06/202	1	10/06/202	1		1	10/06/202	2
		1	23/03/202	1		1	26/03/202	2
		1	05/05/202	1		4		
		1	28/07/202	1		1	02/08/202	2
		1	12/08/202	1		2	21/08/2021	
	23/03/202	1	24/03/202	4	SWAB NAS	1	30/03/202	2
		2						
		1	07/06/202	1		5		
RESULTAD	28/08/202	1	23/08/202	1		1	23/08/2021	
		1	01/08/202	1		1	01/08/202	2
	24/03/202	1	25/03/202	1		1	27/03/2021	
		1	14/04/202	1		1	14/04/2021	

		1	05/03/202	1		5		
		1	03/04/202	1		1	06/04/202	2
		1	03/03/202	1		1	08/03/202	2
	10/06/202	1	09/06/202	1		4		
		1	11/06/202	4	TESTE RAPI	5		
		1	20/06/202	1		1	21/06/202	2
		1	09/03/202	1		1	11/03/202	2
INFILTRAD	03/10/202	1	26/09/202	4	SECRECAO	1	30/09/2021	
	15/03/202	1	16/03/202	1		1	18/03/2021	
		1	07/05/202	1		1	07/05/202	2
		1	10/06/202	1		1	10/06/202	2
	17/07/202	1	12/07/202	1		1	15/07/2021	
		1	22/10/202	1		4		
		1	22/07/202	1		4		
		1	23/04/202	1				
	28/03/202	1	25/03/202	1		1	25/03/202	9
INFILTRAD	01/08/202	1	28/07/202	1		4		
		1	16/03/202	1		1	18/03/202	2
	28/05/202	1	25/05/202	1		1	28/05/2021	
		1	27/04/202	1				
		1	03/06/202	1		1	06/06/2021	
		1	15/05/202	1		1	18/05/202	2
		1	10/03/202	1		1	10/03/202	2
		1	24/04/202	1		1	25/04/2021	
CONDENSA	04/06/202	1	04/06/202	1		5		
	20/05/202	1	12/05/202	1		1	17/05/2021	
		1	19/05/202	1		1	25/05/202	2
		1	26/03/202	1		4		
		1	03/05/202	1		4		
		1	14/07/202	1		1	15/07/202	2
		1	08/03/202	1		1	08/03/202	9
	13/04/202	1	13/04/202	1		2	17/04/2021	
		1	24/05/202	1		1	26/05/202	2
		1	07/06/202	1		1	16/06/202	9
		1	29/06/202	1		5		
VELAMENT	14/07/202	1	10/07/202	1		1	13/07/202	2
		1	23/02/202	1		1	25/02/2021	
	24/05/202	1	15/05/202	1		1	16/05/202	2
		1	26/03/202	1		1	29/03/2021	
		1	23/04/202	1		1	24/04/2021	
		1	25/09/202	1		4		
		1	17/06/202	1		1	18/06/202	2
		1	12/05/202	1		1	13/05/202	2
		1	21/06/202	1		1	24/06/202	2
		1	26/02/202	1		1	27/02/202	2

		1	24/03/202	1		1	25/03/202	2
		1	22/02/202	1		1	22/03/202	2
		1	06/06/202	1		1	06/06/202	2
		1	27/04/202	1		1	27/04/2021	
		1	05/06/202	1		1	05/06/2021	
		1	07/05/202	1		4		
	22/07/202	1	22/07/202	1				
		1	30/03/202	1		1	31/03/202	2
		2						
		1	03/04/202	1		1	06/04/202	9
		1	21/04/202	1		1	23/04/202	2
		1	29/04/202	1		1	30/04/202	2
		1	16/04/202	1		4		
		1	25/05/202	1		1	25/05/2021	
		1	28/07/202	1		1	02/08/202	2
		1	26/05/202	1		1	30/05/202	2
		1	05/05/202	1		1	06/05/2021	
		1	08/06/202	1		1	08/06/202	2
		1	13/06/202	1		1	15/06/202	2
		1	28/05/202	1		1	29/05/2021	
	15/03/202	1	15/03/202	1		1	17/03/2021	
		1	24/03/202	1		4		
	19/03/202	1	17/03/202	1		1	17/03/202	2
	30/05/202	1	24/05/202	1		1	24/05/2021	
		1	24/05/202	1		4		
		1	03/06/202	1		1	04/06/2021	
		1	27/05/202	1		1	31/05/202	2
		1	12/06/202	1		1	12/06/202	2
		1	07/10/202	4	SWAB NASOFARINGE			
		1	05/06/202	1		1	07/06/202	2
		1	30/04/202	1		5		
		1	23/05/202	1		1	26/05/202	2
		1	24/05/202	1		1	01/06/202	9
INFILTRAD	26/07/202	1	26/07/202	1		2	26/07/2021	
		1	22/07/202	1		1	24/07/202	2
		1	04/06/202	1		1	05/06/2021	
	06/04/202	1	06/04/202	1		5		
		1	25/05/202	1		1	02/06/202	2
		1	04/04/202	1		1	06/04/202	2
	24/05/202	1	24/05/202	1		1	27/05/2021	
		1	04/06/202	1		1	05/06/202	2
		1	08/07/202	1		1	10/07/2021	
		1	25/02/202	1		1	25/02/202	9
	30/04/202	1	13/04/202	1		1	13/04/2021	
		1	23/04/202	1		2	23/04/2021	
		1	14/04/202	1		1	15/04/2021	

	04/05/202	1	30/04/202	1		1	01/05/2021	
	05/07/202	1	27/06/202	1		1	28/06/202	2
		1	13/07/202	1		1	14/07/202	9
		1	10/03/202	1		1	11/03/202	2
		1	03/03/202	1		1	05/03/2021	
		1	19/02/202	1		1	23/02/202	2
		1	13/04/202	1		1	16/04/202	9
		1	14/04/2021			1	14/04/202	2
VIDRO FOS	27/06/202	1	25/05/202	1		4		
	25/04/202	1	25/04/202	1		2	27/04/2021	
OPACIDAD	13/05/202	1	05/05/202	1		1	08/05/2021	
CONDENSA	05/09/202	1	21/08/202	1		5		
	13/03/2021							
	05/04/202	1	01/04/202	1		1	01/04/2021	
	07/06/202	1	07/06/202	1				
	10/04/202	1	07/04/202	1		1	09/04/2021	
		1	10/05/202	1		1	11/05/202	2
		1	30/05/202	1		1	30/05/202	2
		1	24/05/202	1		1	16/06/2021	
OPACIDAD	23/05/202	1	13/05/202	1		1	15/05/202	2
	26/05/202	1	27/05/202	4	SECRECAO	1	27/05/202	9
		1	12/05/202	1		5		
		1	09/02/202	1		1	12/02/202	9
		1	01/02/202	1		1	01/02/202	9
		1	24/03/202	1		1	26/03/2021	
		1	02/06/202	1		1	02/06/202	2
		1	07/03/202	4	SWAB NAS	1	09/03/202	2
	04/08/202	1	03/08/202	1		4		
		1	21/07/202	1		1	21/07/202	2
		1	10/09/202	1		1	11/09/2021	
		1	10/03/202	1		4		
		1	02/05/202	1		1	03/05/202	2
		1	18/05/202	1		1	26/05/202	2
		1	14/06/202	1		2	14/06/2021	
		1	19/07/202	1		1	19/07/2021	
	18/02/202	1	12/02/202	1		1	13/02/202	2
		1	01/04/202	1		1	01/04/2021	
		1	29/04/202	1		1	29/04/202	2
		1	04/07/202	1		1	04/07/202	2
		1	23/07/202	1		1	24/07/202	2
		1	27/05/202	1		1	30/05/2021	
		1	09/06/202	1		1	10/06/2021	
	07/05/202	1	06/05/202	1		1	08/05/2021	
	03/03/202	1	04/03/202	4	SWAB NAS	2	07/03/2021	
,	19/03/202	1	17/03/202	1		4		
		1	11/04/202	1		1	13/04/202	2

		1	19/06/202	1		4		
		1	30/11/202	1		4		
		1	16/04/202	1		1	16/04/2021	
	30/03/202	1	22/03/202	1		1	24/03/202	2
		1	25/02/202	1		1	25/02/2021	
	25/05/202	1	25/05/202	1		1	25/05/2021	
	26/04/202	1	17/04/202	1		1	19/04/2021	
	29/04/202	1	23/04/202	1		1	30/04/202	2
	26/05/202	1	20/05/202	1		4		
LESOES INF	07/07/202	1	03/07/202	4	TR ANTIGENO			
		1	21/04/202	1		1	21/04/202	2
		1	01/03/202	4	SOROLOGI/	1	01/03/202	9
		1	24/07/2021			4		
		1	09/02/202	1		5		
	14/10/202	1	15/10/202	1		4		
		1	10/04/202	1		4		
		2						
		1	22/05/202	1		5		
	11/05/202	1	08/05/202	1		1	10/05/202	2
		1	19/05/202	1		1	22/05/2021	
		1	10/06/202	1		1	12/06/202	9
		1	26/09/202	1		4		
		1	14/04/202	1		1	15/04/2021	
		1	22/02/202	1		1	22/02/202	2
		1	28/04/202	1		1	28/04/2021	
		1	20/05/202	1		4		
		1	08/06/202	1		1	17/06/202	2
		1	31/05/202	1		1	02/06/202	2
		1	23/03/202	1		1	25/03/202	2
		1	24/11/202	1		1	24/11/2021	
		1	04/02/202	1		4		
	25/02/202	1	25/02/202	1		1	02/03/2021	
		1	11/03/202	1		1	11/03/202	2
		1	01/03/202	1		1	03/03/2021	
		1	11/04/202	1		4		
OPACIDAD	27/04/202	1	19/04/202	1		1	23/04/202	2
		1	18/11/202	1		2	19/11/2021	
		1	18/03/202	1		5		
		1	30/05/202	4	SANGUE, S	4		
	29/07/202	1	23/07/202	4	SWAB NAS	4		
		1	03/01/202	1		4		
	24/06/202	1	23/06/202	1		1	24/06/202	2
	14/01/202	1	14/01/202	4	SWAB NAS	2	16/01/2021	
		1	13/03/202	1		1	13/03/2021	
		1	14/03/202	1				
		1	06/05/202	1		1	08/05/2021	

	28/05/202	1	30/05/202	1		1	01/06/202	2
		1	29/04/202	1		1	04/05/2021	
		1	05/03/202	1		1	05/03/2021	
		1	15/04/202	1		1	15/04/202	2
		1	07/05/202	1				
		1	05/08/202	1		5		
		1	15/05/2021			1	15/05/202	2
		1	06/01/202	1		5		
		1	04/03/202	1		1	06/03/202	9
		1	25/01/202	1		1	27/01/202	2
	29/04/202	1	27/04/202	1		1	28/04/2021	
		1	19/03/202	4	TESTE RAPI	1	19/03/2021	
		1	27/03/202	1		1	29/03/2021	
		2						
		1	23/04/202	1		4		
		1	03/08/202	1		1	04/08/202	2
		1	10/01/202	1		1	10/01/202	2
		2						
		1	20/06/202	1		5		
		1	02/03/202	1		1	02/03/202	2
		1	05/03/202	1		1	07/03/202	2
		1	17/03/202	1		1	21/03/2021	
		1	30/06/202	1		1	01/07/2021	
		1	08/06/202	1		1	14/06/2021	
		1	04/06/202	1		4		
		1	09/08/202	1		1	10/08/202	2
	20/05/202	1	13/05/202	1		1	15/05/202	2
		1	12/01/202	1		1	12/01/2021	
		1	29/05/202	1		1	31/05/202	2
	22/07/202	1	20/07/202	1		1	21/07/202	2
		1	14/06/202	1		1	15/06/2021	
	26/05/202	1	16/05/202	4	TESTE RAPI	5		
		1	10/11/202	1		1	11/11/2021	
		1	12/04/202	1		5		
AGUARDAI	12/04/202	1	13/04/202	1		1	15/04/202	2
		1	09/06/202	1		1	09/06/2021	
		1	06/02/202	1		1	10/02/2021	
		1	02/03/202	1		1	03/03/2021	
TC TORAX		1	18/02/202	1		1	20/02/202	2
		1	24/03/202	1		1	27/03/202	2
		1	13/07/2021			5		
		1	31/07/202	1		5		
		1	12/01/202	1		1	14/01/202	2
VIDRO FOS	27/01/202	1	22/01/202	1		1	22/01/202	2
		1	08/02/202	1		1	08/02/2021	
		1	05/02/202	1		1	06/02/202	2
		1	03/04/202	1		1	03/04/202	2

	20/04/202	1	14/04/202	1		1	20/04/202	2
	14/06/202	1	15/06/202	4	SECRECAO TRAQUEAL			
	17/06/202	1	14/06/202	1		1	17/06/202	2
		1	19/03/202	1		1	31/03/202	2
	22/05/202	1	22/05/202	1		2	23/05/2021	
		1	17/05/202	1		4		
		1	23/08/202	1		1	25/08/202	2
	27/01/202	1	25/01/202	1		1	25/01/2021	
	03/04/202	1	26/03/202	1		1	07/04/202	2
	28/05/202	1	29/05/202	1		1	31/05/202	9
		1	04/02/202	1		1	06/02/2021	
		1	03/03/202	1		1	05/03/202	2
		1	19/02/202	4	ECO TESTE	5		
		1	28/03/202	1		1	28/03/2021	
OPACIDAD	08/06/202	1	02/06/202	1		1	05/06/202	2
		1	05/06/202	1		1	06/06/2021	
		1	19/07/202	1		1	19/07/202	2
		1	18/08/202	1		1	18/08/202	2
		1	05/01/202	1		1	07/01/202	2
		1	08/03/202	1		1	11/03/2021	
		1	04/03/202	1		1	05/03/2021	
	29/03/202	1	23/03/202	1		1	25/03/202	2
		1	26/04/202	1				
		1	19/05/202	1		1	20/05/202	2
		1	04/09/202	1		1	05/09/202	2
		1	12/01/202	1		1	12/01/2021	
		1	27/03/202	1		1	29/03/202	2
		1	02/04/202	1		1	03/04/202	9
	26/04/202	1	27/04/202	1		1	28/04/2021	
		1	18/10/202	2		1	19/10/2021	
		1	05/03/202	1		1	10/03/202	2
		1	01/02/202	1		1	02/02/2021	
		1	04/06/202	1		5		
		1	26/08/202	1		1	26/08/202	2
		1	16/03/202	1		1	22/03/202	2
		1	30/04/202	1				
OPACIDAD	23/06/202	1	19/06/202	1		1	21/06/202	2
		1	23/03/202	1		1	25/03/202	2
		1	25/05/202	1		5		
		1	13/08/202	1		1	13/08/2021	
		1	02/06/202	1		1	02/06/202	2
		1	10/06/202	1				
		1	28/09/202	1		5		
		1	05/03/202	1		1	06/03/202	2
		2						
		1	31/05/202	4	TESTE RAPI	5		
		1	31/03/202	1		1	01/04/2021	

		1	01/05/202	1		1	01/05/2021	
		1	30/03/202	1		4		
	13/06/202	1	10/06/2021			1	11/06/202	2
		1	25/04/202	1		1	26/04/2021	
GESTANTE	07/07/202	1	30/06/202	1		1	04/07/202	9
		1	21/05/202	1		1	22/05/202	2
		1	26/08/202	1		5		
		1	01/05/2021			1	01/05/202	2
		1	25/06/202	1		1	28/06/202	2
		1	17/06/202	1		1	21/06/202	2
		1	11/06/202	1		1	13/06/202	2
		1	15/03/202	1		1	15/03/2021	
		1	19/04/202	4		4		
		1	21/03/202	1		1	23/03/2021	
		1	16/05/202	1		5		
		1	21/01/202	1		1	21/01/202	2
		1	04/06/202	4	TESTE RAPI	5		
		1	06/08/202	1		4		
		1	06/04/202	1		2	07/04/2021	
		2						
		2						
		1	14/04/202	1		1	15/04/2021	
		1	22/02/202	1		1	22/02/2021	
		1	15/02/202	1		1	17/02/2021	
CONTROLE	31/03/202	1	18/03/202	1		9		
		1	11/04/202	1		1	11/04/2021	
		1	06/07/202	1		2	15/07/2021	
		1	30/06/202	1		1	30/06/202	2
		1	25/05/202	4	SWAB NAS	4		
		1	05/10/202	1		1	06/10/2021	
		1	07/01/202	1		1	07/01/2021	
	30/05/202	1	29/05/202	1		4		
OPACIDAD	24/10/202	1	15/10/202	1		4		
	10/03/202	1	28/02/202	1		1	06/03/2021	
	14/03/202	1	14/03/202	1		1	16/03/202	9
	10/05/202	1	10/05/202	1		1	12/05/202	2
		1	12/06/202	4	NASOFARII	4		
	16/04/202	1	12/04/202	1		5		
		1	14/03/202	1		1	21/03/2021	
		1	01/04/202	1		1	01/04/2021	
		1	28/03/202	1		1	01/04/2021	
		1	17/07/202	1		4		
	28/05/202	1	24/05/202	1		5		
		1	04/04/202	1		1	04/04/2021	
		9						
		1	22/05/202	1		1	01/06/2021	

		1	22/11/202	1		5		
		1	15/06/202	1		1	15/06/2021	
	27/04/202	1	27/04/202	1		1	27/04/2021	
		1	23/04/202	1		1	24/04/202	2
		1	07/05/2021			1	08/05/2021	
	28/03/202	1	13/03/202	1		1	15/03/2021	
		1	15/07/202	1		4		
		2						
	12/04/202	1	12/04/202	1		1	12/04/202	2
		1	18/02/202	1		1	20/02/2021	
		1	22/04/202	1		1	23/04/202	2
		1	29/05/202	1		9		
	02/08/202	1	28/07/202	1		1	29/07/202	2
	18/02/202	1	18/02/202	1		1	19/02/2021	
		1	30/03/202	1		1	30/03/202	9
		2						
		1	16/07/202	1		5		
		1	10/05/202	1		1	26/05/202	2
HIPOTRAN	14/04/202	1	14/04/202	1		1	16/04/202	2
		1	08/06/202	1		1	11/06/2021	
	29/07/202	1	26/07/202	1		5		
	10/05/202	2						
		1	18/10/202	4	SWAB NAS	1	23/10/2021	
	20/02/202	1	18/02/202	1		1	18/02/202	2
		1	29/03/202	1		1	29/03/202	9
		1	12/02/202	1		1	13/02/2021	
		1	22/08/202	1		1	24/08/202	2
		1	18/05/202	4	TESTE RAPI	5		
		1	20/08/202	4	SECRECAO	5		
		1	04/11/202	1		1	05/11/202	2
		1	02/07/202	1		1	02/07/202	9
		1	27/03/202	1		1	27/03/202	9
		1	27/03/202	4		4		
		1	09/06/202	1		1	11/06/202	2
		1	14/07/202	4	PACIENTE I	1	14/07/2021	
		1	16/11/202	1		4		
		1	03/05/202	1		1	03/05/202	9
		1	12/10/202	1		5		
	15/07/202	9						
OPSCIDADI	16/08/202	1	12/08/202	1		1	12/08/202	9
OPACIDAD	19/03/202	1	09/03/202	1		1	09/03/202	2
		1	31/12/202	4	NASOFARII	1	01/01/202	2
		1	30/03/202	1		1	30/03/2021	
		1	08/04/202	1		1	10/04/202	2
		1	10/02/202	1		1	11/02/202	9
		1	18/01/202	1		1	19/01/2021	

		1	12/04/202	1		1	13/04/202	2
		1	31/05/202	1		1	01/06/202	9
	09/04/202	1	09/04/202	1		1	09/04/2021	
		2						
		1	08/04/202	1		1	08/04/2021	
		1	23/05/202	1		1	25/05/202	9
		1	13/05/202	1		1	18/05/2021	
		1	06/06/202	1		1	12/06/202	9
		1	14/07/202	4		4		
	11/05/202	1	10/05/202	1		1	11/05/202	2
		1	22/02/202	1		1	23/02/2021	
	14/03/202	1	10/03/202	1		1	13/03/2021	
		1	09/03/202	1		1	12/03/202	2
	12/04/202	1	04/04/202	1		1	06/04/2021	
		1	30/05/202	1		1	31/05/202	2
	23/05/202	1	23/05/202	1		1	23/05/2021	
		1	09/07/202	1		1	10/07/2021	
		1	22/02/202	1		1	23/02/2021	
		1	16/04/202	1		2	18/04/2021	
		1	25/05/202	1		1	28/05/2021	
		1	30/03/202	1		1	01/04/2021	
		1	30/09/202	1		1	30/09/202	2
		1	11/10/202	1		4		
		1	17/04/202	1		1	22/04/2021	
		1	26/04/202	1		1	26/04/202	2
		1	20/04/202	4	TR SWAB A	5		
		1	25/05/202	1		1	27/05/202	2
		1	10/06/202	1		1	10/06/202	2
		1	06/07/202	1		1	21/07/202	2
		1	21/08/202	1		1	22/08/2021	
		1	20/09/202	1		1	21/09/202	9
		1	27/05/202	1		1	30/05/202	2
		1	28/05/202	1		1	28/05/202	2
		1	25/06/202	1		1	26/06/202	2
ACOMETIN	06/03/202	1	15/03/202	1		1	16/03/202	2
		1	25/04/202	1		1	30/04/202	2
		2						
		1	31/05/2021			5		
		1	15/06/202	1		1	17/06/202	2
		1	01/11/202	1		5		
		1	14/04/202	1		1	16/04/202	2
		1	04/05/202	1		1	06/05/202	2
		1	28/05/202	1		1	29/05/2021	
		1	08/11/202	1		1	09/11/202	2
		1	12/05/202	1		4		
	05/06/202	1	01/06/202	4	SWAB NAS	1	08/06/202	2

	1	25/04/202	1		5		
	1	16/09/202	1		5		
	1	30/08/202	1		4		
	1	10/07/202	4	SWAB NAS	1	12/07/2021	
	1	06/03/202	1		1	08/03/202	2
	2						
	1	08/06/202	4	NASOFARII	1	09/06/202	2
02/06/202	1	02/06/202	1		1	03/06/2021	
	1	24/06/202	1		1	04/07/202	2
	1	24/07/202	1		1	30/07/202	2
	1	13/08/202	4		4		
	1	03/11/202	1		1	03/11/2021	
	1	03/02/202	1		1	09/02/202	2
05/05/202	1	07/05/202	4	SECRECAO	1	07/05/202	9
	1	05/01/202	1		1	05/01/2021	
	1	16/02/202	1		1	18/02/202	2
	1	10/05/202	1		4		
	1	22/05/202	1		5		
	1	26/02/202	1				
	1	29/03/202	1		1	01/04/202	9
	1	01/03/202	1		1	01/03/202	2
16/03/202	2					16/03/202	2
13/08/202	1	16/08/202	1		1	20/08/2021	
	1	04/03/202	1		1	05/03/202	2
	1	01/06/202	1		1	04/06/2021	
	1	11/08/202	2		1	11/08/202	2
	1	24/05/202	1		1	27/05/202	2
15/07/202	1	13/07/202	1		4		
	1	01/07/202	1		1	01/07/2021	
	1	24/03/202	1		1	25/03/202	9
	1	15/04/202	1		1	22/04/2021	
17/03/202	1	17/03/202	4		5		
	1	14/03/202	1		5		
	1	17/05/202	1		1	19/05/202	2
	1	26/05/202	1		1	28/05/202	2
	1	29/06/202	1		1	30/06/202	2
	1	12/09/202	1		4		
	1	16/03/202	1		1	17/03/2021	
	1	06/04/202	1		1	08/04/202	2
30/03/202	1	17/03/202	1		1	17/03/202	2
	1	31/07/202	1		1	31/07/202	2
	1	13/03/202	1		1	14/03/2021	
16/05/202	1	13/05/202	1		1	14/05/202	2
17/07/202	1	16/07/202	1		5		
	1	03/01/202	1		1	06/01/2021	
	1	12/05/202	1		1	13/05/2021	
	2						

	12/01/202	1	12/01/202	1		1	13/01/202	2
		1	05/02/202	1		1	08/02/202	9
	10/04/202	1	03/04/202	1		1	07/04/202	9
		1	18/05/202	1		1	19/05/2021	
		1	09/06/202	1		1	10/06/202	2
		1	02/03/2021			5		
		1	03/03/202	1		1	08/03/2021	
AREA DESE	19/03/202	1	18/03/202	1		4		
		1	16/03/202	1		1	17/03/2021	
		1	01/04/202	1		1	02/04/202	2
	01/07/202	1	28/06/202	1		1	28/06/202	2
		1	12/02/202	1		2	12/02/2021	
		1	26/02/202	1		1	28/02/202	2
		1	15/03/202	1		1	17/03/202	2
		1	22/03/202	1		1	26/03/202	2
		1	24/11/202	1		1	24/12/202	2
		1	01/12/202	1		1	01/12/202	2
		1	08/03/202	1		1	08/03/202	2
OPACIDAD	19/04/202	1	12/04/202	1		1	12/04/202	2
		1	06/06/202	1		1	06/06/202	9
		1	20/02/202	1		4		
		1	17/03/202	1		1	17/03/2021	
TC TORAX EXTERNO		1	10/02/202	1		1	12/02/202	2
		1	30/05/202	1		1	31/05/2021	
		1	12/07/202	1		1	12/07/2021	
		1	09/08/202	1		1	10/08/202	9
		1	20/02/202	1		1	23/02/202	2
	20/03/202	1	13/03/202	1		4		
TOMO		1	23/04/202	4	ANTIGENO	4		
		1	25/05/202	1		1	26/05/202	9
		1	20/07/202	1		1	21/07/2021	
		1	30/04/202	4	ANTIGENO	5		
		1	24/10/202	1		5		
		1	20/04/202	1		1	21/04/202	2
		1	14/05/202	1		1	15/05/2021	
		1	22/06/202	1		1	23/06/202	2
	29/12/202	1	29/12/202	1		2	29/12/2021	
		1	03/03/202	1		1	06/03/202	2
		1	30/05/202	1		1	10/06/202	2
		1	18/09/202	1		1	18/09/202	2
		1	20/04/202	1		1	20/04/2021	
		1	22/06/202	1		1	22/06/2021	
		1	06/09/202	1		1	07/09/202	2
		1	28/07/202	1		1	28/07/2021	
	26/05/202	1	26/05/202	1		1	31/05/2021	
		1	23/10/202	1		1	24/10/202	2
		1	02/03/202	1		1	05/03/2021	

		2						
		1	24/05/202	1		1	24/05/2021	
		1	20/07/202	1		1	21/07/2021	
		1	21/10/202	1		1	23/10/202	2
		1	21/03/202	1		1	27/03/2021	
		1	07/04/202	1		1	08/04/2021	
		1	14/06/202	1		1	16/06/202	2
		1	21/03/202	4	SECRECAO	2	22/03/2021	
		1	07/04/202	1		1	09/04/202	2
		1	19/04/202	1		1	22/04/202	9
		1	04/08/202	1		1	05/08/202	9
		1	11/04/202	4	SANGUE	5		
		1	21/06/202	1		1	21/06/2021	
		1	19/03/202	1		1	21/03/2021	
		1	13/03/202	1		1	14/03/202	2
		1	16/04/202	9		1	16/04/202	9
		1	26/04/202	1		1	29/04/202	9
		1	04/04/202	1		1	04/04/2021	
		1	03/09/202	1		1	06/09/202	2
		1	07/07/202	1		2	07/07/2021	
		2						
OPACIDAD	16/03/202	1	05/03/202	1		1	05/03/2021	
		1	11/05/202	1		1	13/05/202	2
		1	30/06/202	1		1	02/07/2021	
	27/05/202	1	20/05/202	1		1	23/05/202	2
		1	11/01/202	1		1	14/01/202	2
		1	05/07/202	1		1	07/07/202	2
		1	25/02/202	1		1	03/03/202	2
	04/05/202	1	29/04/202	1		1	30/04/202	9
		1	26/07/202	1		1	27/07/2021	
		1	08/03/202	1		1	09/03/202	2
		1	04/03/202	1		1	10/03/202	2
		2						
		1	19/05/202	1		1	24/05/202	9
		1	07/06/202	1		1	10/06/2021	
		1	16/02/202	1		1	20/02/202	2
	13/04/202	1	13/04/202	4	SANGUE	5		
		1	29/04/202	1		1	30/04/2021	
	09/06/202	1	11/06/202	1		2	12/06/2021	
		1	17/06/202	1		4		
		1	23/09/202	1		1	25/09/202	9
		1	19/06/202	1		1	21/06/2021	
		1	03/01/202	1		5		
		1	14/01/202	1		1	19/01/2022	
ANDAMEN	02/02/202	1	01/02/202	1		1	01/02/2022	
		1	11/01/202	1		1	11/01/202	2

25/02/202	1	22/02/202	1		1	26/02/202	2
	1	07/02/202	1		5		
	2						
	1	05/01/202	1		4		
	1	20/01/202	1		4		
	1	13/01/202	1		4		
	2						
	1	18/01/2022			4		
	1	02/02/202	1		4		
21/01/202	1	21/01/202	4	TESTE RAPI	4		
	1	07/01/202	1		1	07/01/202	2
	9						
	1	07/01/202	1		1	09/01/202	2
	1	21/01/202	1		4		
	2						
	1	29/01/202	4	TR ANTIGENO			
	1	07/02/202	1		4		
	1	02/02/202	1		5		
	1	08/02/202	4	SWAB NAS	1	13/02/202	2
	1	19/01/202	4	TESTE RAPI	5		
	1	25/01/202	1		4		
	1	06/01/202	1		5		
	1	15/01/2022			4		
12/01/202	1	14/01/202	1		1	17/01/202	2
	1	22/01/202	1		1	28/01/202	2
	1	17/01/202	1		1	17/01/202	2
	1	16/01/2022			4		
	1	21/01/202	1		4		
04/02/202	1	05/02/202	1		1	07/02/2022	
	1	24/01/202	1		5		
	1	14/01/202	1		4		
	1	13/01/202	1		1	13/01/2022	
	1	18/01/202	1		1	20/01/2022	
05/01/202	1	05/01/202	1		1	10/01/2022	
	1	17/01/202	4	SWAB NAS	1	17/01/202	2
	1	06/01/202	1		5		
	1	15/01/2022			4		
	1	24/02/202	1		5		
10/01/202	1	08/01/202	1		1	08/01/202	2
	1	11/01/202	1		2	19/01/2022	
	1	17/01/202	1		1	19/01/2022	
	1	07/02/202	1		4		
	1	13/01/202	1		1	13/01/202	2
	1	13/01/202	1		1	13/01/2022	

		1	17/01/202	1		4			
		1	17/01/202	1		5			
		1	25/01/202	1		4			
		1	14/01/202	1		1	19/01/2022		
		1	12/01/202	1		1	12/01/202	2	
		1	18/01/202	1		1	18/01/2022		
		1	31/01/202	1		1	31/01/202	2	
	09/01/202	1	09/01/202	1		1	09/01/2022		
		1	19/01/202	1		5			
	25/01/202	1	25/01/202	1		1	26/01/202	2	
	17/01/202	1	17/01/202	1		1	19/01/202	1	1
OPACIDAD	16/06/202	1	17/01/202	4	SWAB NAS	1	18/01/2022		
		1	25/01/202	1		5			
		1	10/01/202	1		5			
		1	16/02/202	1		1	16/02/202	2	
		1	04/02/202	1		1	04/02/202	2	
		1	02/02/202	1		1	02/02/202	2	
		1	15/01/202	1		1	15/01/2022		
		1	07/02/202	1		4			
		2							
	05/01/202	1	06/01/202	1		1	07/01/2022		
		1	17/01/202	1		4			
		1	20/02/202	1		1	22/02/202	2	
		1	20/01/202	4	ANTIGENO	4			
		1	20/01/202	1		4			
		1	15/01/202	1		1	15/01/2022		
		1	17/01/202	1		4			
		1	09/01/202	1		5			
	15/01/202	1	15/01/202	1		4			
		1	07/01/202	1		5			
		1	24/01/202	1		5			
		1	16/01/202	1		1	16/01/202	2	
		2							
		1	25/01/202	1		1	25/01/2022		
		1	31/01/202	1		1	31/01/2022		
	18/01/202	1	17/01/202	1		1	24/01/2022		
		1	22/01/202	1		5			
		1	29/01/202	4	RAPIDO	5			
		1	17/01/202	1		1	18/01/202	2	
		1	27/01/202	1		1	29/01/2022		
		1	19/01/202	1		5			
		1	16/02/202	1					
	29/01/202	1	29/01/202	1		1	29/01/202	2	
		1	11/01/202	1		5			
		1	17/02/202	1		4			
		1	19/02/202	1		5			
		1	20/01/202	4		5			

		1	17/01/202	1	1	17/01/2022		
		1	17/01/202	1	5			
		1	23/01/202	1	1	26/01/202	2	
		1	14/01/202	1	1	14/01/202	2	
		1	12/02/202	1	5			
		1	03/02/202	1	1	03/02/202	2	
		1	19/01/202	1	1	25/01/202	1	1
		1	07/01/202	1	4			
		1	27/01/202	1	4			
		1	04/02/202	1	4			
		1	01/02/202	1	1	01/02/2022		
INFILTRAD	10/02/202	1	10/02/202	1	1	10/02/202	2	
		1	14/02/202	4	4			
		1	20/01/202	1	1	29/01/202	9	
		1	31/01/202	1	5			
		1	21/01/202	1	5			
		1	14/01/202	1	1	20/01/2022		
		1	19/01/202	1	1	22/01/202	2	
		1	28/03/202	1	4			
		1	23/01/202	1	1	25/01/202	2	
	03/01/202	1	03/01/202	1				
	15/01/202	1	15/01/202	1	1	15/01/2022		
	30/01/202	1	30/01/202	1	1	31/01/202	2	

PCR_FLUAS	LUASU_OU	PCR_FLUBL	LUBLI_OU	OS_PCROU	PCR_VSR	PCR_PARA1	PCR_PARA2	PCR_PARA3	PCR_PARA4
				1					
				1					
				1					
				1					
				1					
				1					
				1					
				1					
				1					
				1					
				1					
				1					
				1					
				1					
				1					
				1					
				1					
				1					
				1					
				1					
				1					
				1					
				1					
				1					
				1					
				1					
				1					
				1					
				1					
				1					
				1					
				1					
				1					
				1					

1
1

1
1
1
1
1
1
1

1
1
1
1
1
1

1
1
1
1
1
1
1
1

1
1
1
1
1
1
1
1

1

1
1
1

1

1

1
1
1
1
1
1
1

1
1
1
1

1
1

1
1
1
1
1
1

1
1
1
1
1
1

1
1

1
1

1
1
1
1
1
1
1

1
1
1
1
1
1
2
1
1
1
1
1
1
1
1
1
1
1
1
1
1
1
1
1
1
1
1
1
1
1
1
1
1
1
1
1
1
1
1

1

1
1
1

1
1

1
1
1
1
1
1
1
1
1
1
1

1
1

1
1
1
1
1

1
1
1
1
1

1
1
1
1
1
1
1
1
1

1
1

1
1
1
1
1

1
1

1
1
1
1
1
1
1
1

1
1
1
1

1

1
1

1
1
1
1
1
1
1
1
1
1
1
1

1
1
1
1

1
1
1
1
1

1

1
1

1
1
1
1
1

1
1
1
1
1
1
1
1
1

1
1

1
1
1
1
1
1
1

1
1

1

1
1
1
1
1

1
1

1
1
1

1
1
1
1
1

1
1
1
1
1
1
1
1

1
1

1

1
1
1

1

1
1
1
1
1
1
1
1

1
1
1
1

1

1
1
1
1
1

1
1
1
1
1
1

1

1
1

1
1

1
1
1
1
1
1

1

1
1

1
1
1

1
1

1

1
9
1
1
1

1
1

1
1

1
1
1

1
1
1
1
1
1
1
1
1
1
1
1
1

1
1
1
1

1
1
1
1
1
1
1

1
1

1
1

1
1
1
1
1

1
1
1
1
1
1
1

1

1
1
1
1
1

1
1
1
1

1

1
1
1
1

1
1
1

1
1

1
1
1
1
1

1
1

1
1
1
1

1
9
1
1
1
1

1
1
1
1
1

1
1
1
1

1
1

1
1

1
1
1
1

1
1
1
1
1
1
1
1
1
1

1
1
1
1
1
1
1
1
1
1
1
1
1

1

1
1

1
1

1
1
1

1
1
1
1
1

1
1

1

1
1

1

1

1
1
1
1
1
1
1
1
1
1
1
1
1
1
1
1
1
1
1

1
1
1
1
1
1
1
1

2
1
1
1
1
1

1
1
1

1

1
1
1
1

1
2

1

1
1

1
1
1

1

1

1
1
1
1

1
1

1

1
1
1

1
1
1
1
1
1
1
1
1

1
1
1
1
1
1
1
1

1
1
1
1
1

1
1
1
1
1
1
1

1
1

1

1
1

1
1
1
1
1
1
1
1
1
1
1
1
1

1
1
1
1
1
1
1

1
1

1
1
1
1
1
1
1

1
1
1
1
1

1
1
1
9
1
1
1
1
1

1
1

1

1
1
1
1
1
1

1
1
1
1

1
1
1
1
1

1
1
1

1

1
1
1

1
1
1
1
1
1
1
1

1
1
1

1
1

1
1

1
1
1
1
1
1
1
1
1

1

1
1

1

1

1

1

1

1
1
1

1

1
1
1
1

1

1

1
1

1

1

1

1
1
1
1
1

1

1
1
1

1

1
1

1
1
1
1
1
1
1
1
1
1
1
1
1
1

1
1
1
1
1
1
1

1

1

1

1

1
1
1
1
1

1
1
1

1
1
1
1

1
1
1
1

1

1
1

1

1

1
1
1
1

1

1

1
1
1
1
1
1

1
1
1

1

1
9
1
1
1
1

1

1

1

1

1
1

1
1

1
1
1
1
1
1

1
1

1
1
1
1
1
1
1
1

1
1
1
1
1
1
1
1
1
1
1
1
1
1
1
1
1
1
1
1
1
1
1
1
1
1
1
1
1
1
1

1
1

1

1
1
1

1
1

1
1

1
1
1
1
1

1
1
1

1
1
1
1
1
1
1
1
1

1
1
1
1

1
1

1

1
1

1
1
1
1
1
1
1
1

1
1

1
1
1
1
1
1

1
1
1
1
1

1
1
1

1
1
1
1
1

1
1

1

1

1
1
1
1
1
1
1
1
1

1
1

1
1

1

1
1
1
1
1
1
1
1

1

1

1
1

1

1
1
1
1
1
1

1
1
1
1
1
1
1
1

1
1
1
1
1

1

1
1
1
1
1
1

1
1
1
1
1

1

1
1

1
1
1
1
1
1
1
1
1
1
1
1

1
1
1
1
1
1
1

1
1
1
1

1

1

1
1
1

1
1

1
1
1

1
1
1

1
1
1
1

1
1
1
1
1
1
1
1

1

1

1
1
1
1
1

1

1
1

1
1
1

1
1
1
1

1
1
1

1

1

1
1
1
1

1
1

1
1
1

1
1
1
1
1

1
1
1

1
1
1

1

1
1
1

1
1
1

1

1
1

1

1
1

1
1

1
1
1
1
1
1

1
1
1
1

1
1

1
1

1

1
1

1
1

1
1
1
1

1

1
1

1
1
1

1

1

1

1

1

1

1
1

1
1
1

1
1
1
1

1
1

1

1
1
1

1
1

1

1
1
1
1
1

1
1
1
1
1
1
1
1
1
1
6
1

1
1

1
1
1
1
1
1

1

1

1

1
1
1
1
1
1
1

1
1
1
1

1
1
1
1

1
1
1
1

1
1
1
1
1
1

1
1
1

1
1
1
1
1

1

1

1

1

1

1

1

1

1

1

1

1

1

1

1

1

1

1

1

1

1

1

1

1

1

1

1

1

1
1

1

1
1
1
1
1

1

1
1

1

1
1
1

1
1
1

1

1
1
1

1

1

1
1

1
1
1
1

1
1
1
1

1

1

1
1

1

1
1

1
1
1
1
1
1
1
1
1

1

1

1

1
1

1
1

1
1

1
1

1
1

1
1

1

1

1

1
1

1
1
1
1
1

1

1
1

1
1

1

1

1
1

1
1
1

1
1
1

1
1

1
1
1

1
1

1
1

1
1
1

1
1

1
1

1
1
1
1
1

1

1

1
1

1
1

1
1

1
1
1
1

1

1
1

1
1

1

1
1
1

1
1

1

1
1
1
1

1
1
1
1
1
1

1

1
1

1
1
1

1
1
1
1
1
1
1
1
1
1
1
1
1

1

1
1

1
1
1
1
1

1

1

1
1
1
1

1
1
1

1
1

1
1

1
1

1

1
1
1

1
1
1
1
1

1
1
1
1

1
1

1
1
1
1

1
1

1
1

1

1
1
1

1

1
1

1
1
1
1
1
1
1

1
1

1
1
1
1

1

1

1
1
1
1

1
1

1
1
1
1
1
1
1

1
1

1

1
1
1
1

1

1
1
1
1
1
1

1

1
1
1

1
1
1

1
1
1

1
1
1
1

1
1
1
1

1

1
1
1
1

1

1

1

1

	1
	1
	1
	1
	1
	1
	1
	1
	1
	1
	1
	1
	1
	1
	1
	1
	1
	1
	1
3	2
	1
	1
	1
	1
	1
	1
	1
	1
	1
	1

1
1

1
1
1
1

1

1
1

1

1
1 1
1

1

1
1

1

1
9

1

1
1
1
1
1
1

1

1
1
1
1
1

1

1
1
1
1
1
1
1
1
1
1

1
1

1
1

1
1

1
1

1
1
1

1
1
1

1
1

1
1
1

1
1
1
1
1

1
1

1

1
1
1
1

1
9

1
1
1
1
1
1
1
1
1
1

1

1

1

1
1
1
1
1
1
1
1
1
1
1
1
1
1
1
1
1
1
1
1
1
1
1
1
1
1

1

1
1
1
1

1
1
1
1
1
1
1
1

1
1
1
1

1
1
1

1
1
1

1
1
1
1
1
1

1
1

1
1

1
1
1
1
1
1
1

1
1
1
1
1

1
1
1

1
1
1

1
1

1

1
1
1
1
1

1
1
1
1

1

1 1
1
1
1
1
1

1
1

1

1

1

1
1
1

1

1
1

1

1
1
1
1

1

1
1

1

1

1

1
1

1

1

1
1

1
1

1
1
1

1
1

1

1

1
1
1

1
1
1

1
1

1
1
1

1
1

1
1
1
1
1
1
1
1
1

1

1
1
1

1
1

1

1
1
1
1
1
1

1
1

1
1

1

1
1
1
1

1
1
1

1
1
1

1

1
1
1
1
1
1
1
1
1

1
1

1
1

1

1

1
1

1
1
1

1

1
1
1
1
1
1

1
1
1
1

1
1
1
1
1

1
1
1

1
1

1

1
1

1

1

1
1
1
1
1

1
1
1

1
1
1
1
1

1
1

1
1

1
1
1
1

1
1
1

1

1

1
1

1

1

1
1
1

1
1
1

1
1
1
1
1
1

1
1
1

1

1
1

1
1

1
1

1
1
1
1
1
1
1
1
1
1

1
1
1

1

1
1
1

1
1
1
1

1
1

1

1

1
1
1
1

1
1
1
1
1
1

1
1
1
1
1

1

1

1
1
1
1

1

1
1
1
1
1
1
1

1

1
1

1
1

1
1

1
1

1
1

1
1

1
1

1
1
1

1
1
1
1

1
1
1
1
1

1

1
1
1

1
1
1
1
1
1

1

1
1

1
1
1

1

1
1

1
1

1
1

1

1
1

1

1
1
1
1
1
1
1
1

1

1
1

1
1

1
1
1
1
1

1
1

1
1

1
1
1
1
1
1
1
1

1

1

1
1
1
1
1
1

1
1

1
1
1

1
1
1

1
1
1
1

1
1
1

1

1

1

1

1

1

1
1
1
1
1

1
1

1
1

1
1

1
1
1
1
1
1

1
1
1
1
1
1

1
1
1
1
1

1

1
1

1
1
1
1
1
1

1
1
1
1
1
1
1
1
1

1
1

1
1
1

1

1
1

1
1

1
1

1

1

1

1

1
1

1
1
1
1
1

1

1

1
1
1

1

1

1

1
1
1

1
1

1

1

1
1
1

1
1
1

1
1
1

1
1

1
1
1
1

1
1
1

1
1

1

1
1
1

1
1
1

1
1
1

1
1
1

1
1

1
1
1
1
1
1

1

1

1
1

1
1
1
1
1
1
1
1
1
1

1

1
1
1
1

1

1
1

1
1
1
1

1
1
1
1

1
1
1
1
1

1
1

1
1

1
1
1

1
1
1
1
1
1

1
1

1
1
1
1
1

1

1
1
1

1
1
1
1
1
1
1
1

1
1
1
1
1
1

1
1

1
1
1

1
1
1

1
1
1
1
1
1

1
1
1
1
1

1
1
1

1
1
1
1
1
1
1

1
1
1
1
1
1
1
1
1
1
1

1
1
1

1

1
1

1

1

1

1

1

1
1
1

1

1
1
1
1

1

1

1
1

	1
	1
	1
	1
	1
2	1
	1
	1
	1
	1
	1
	1
	1
	1
	1
	1
	1
	1
	1
	1

	1
	1
	1
	1
2	1
	2
	1
	1
	1
	1
	1
	1

PCR_ADEN	PCR_METAI	PCR_BOCA	PCR_RINO	PCR_OUTR	S_PCR_OU	CLASSI_FIN	CLASSI_OU	CRITERIO	EVOLUCAO
						5		1	1
						5		1	2
						5		1	1
						5		3	1
						5		1	1
						5		1	1
						5		1	1
						5		1	1
						5		1	1
						5		1	2
						5		1	1
						5		1	1
						5		1	2
						5		1	1
						5		1	2
						5		1	2
						5		1	1
						5		1	2
						5		1	1
						5		4	1
						5		1	2
						5		1	2
						5		1	1
						5		1	1
						5		1	1
						5		1	1
						5		1	1
						5		1	1
						5		1	1
						5		1	1
						5		1	1
						5		1	1
						5		1	1
						5		1	1
						5		1	2
						5		1	1
						5		1	1
						5		1	1
						5		1	1
						5		1	1
						5		1	1
						5		1	2
						5		1	1
						5		1	1
						5		3	2
						5		1	2

5	1	1
5	1	1
5	1	1
5	1	1
5	1	1
5	1	2
5	1	1
5	1	2
5	1	1
5	1	1
5	1	1
5	1	1
5	1	1
5	1	1
5	1	1
5	1	2
5	1	1
5	1	2
5	1	1
5	1	1
5	1	1
5	1	2
5	1	1
5	1	1
5	1	1
5	1	1
5	1	1
5	1	1
5	1	1
5	1	1
5	1	2
5	1	1
5	1	1
5	1	1
5	1	2
5	1	1
5	1	1
5	1	1
5	4	1
5	1	1
5	1	1
5	1	2
5	1	1
5	4	1
5	1	2
5	1	2
5	1	1

5	1	1
5	1	1
5	1	1
5	1	1
5	1	1
5	1	1
5	1	1
5	1	1
5	4	2
5	1	1
5	1	1
5	1	1
5	1	1
5	1	1
5	1	1
5	1	2
5	1	1
5	1	1
5	1	1
5	1	1
5	1	1
5	1	1
5	1	1
5	1	2
5	1	2
5	1	1
5	1	2
5	1	1
5	1	1
5	1	1
5	1	1
5	1	1
5	1	1
5	1	1
5	1	1
5	1	1
5	1	1
5	1	1
5	1	1
5	1	1
5	1	1
5	1	1
5	1	1
5	1	1
5	1	1
5	1	1
5	1	1

5	1	1
5	1	2
5	1	1
5	1	2
5	1	1
5	1	2
5	1	1
5	1	2
5	1	1
5	1	2
5	1	1
5	1	1
5	1	1
5	1	1
5	3	1
5	1	1
5	1	1
5	1	1
5	1	1
5	1	1
5	1	1
5	1	1
5	1	1
5	1	1
5	1	1
5	1	1
5	1	2
5	1	1
5	1	1
5	1	2
5	1	1
5	1	1
5	1	1
5	1	1
5	1	1
5	1	1
5	1	1
5	1	1
5	1	1
5	1	1
5	1	1
5	1	1
5	1	1
5	1	1
5	1	2
5	1	2
5	1	1

5	1	1
5	1	1
5	1	2
5	1	1
5	1	1
5	1	1
5	1	1
5	1	1
5	1	1
5	1	1
5		1
5	1	1
5	1	2
5	1	2
5	1	1
5	1	1
5	1	1
5	1	1
5	1	1
5	1	1
5	1	2
5	1	1
5	1	1
5	1	1
5	1	1
5	1	1
5	1	1
5	1	2
5	1	2
5	1	2
5	1	1
5	1	1
5	1	2
5	1	2
5	1	1
5	1	2
5	1	1
5	1	1
5	1	1
5	1	1
5	1	2
5	1	1
5	1	2
5	1	1
5	1	2
5	1	1
5	1	1

5	1	2
5	1	2
5	1	2
5	1	1
5	1	1
5	1	1
5	1	2
5	1	1
5	4	1
5	1	1
5	1	1
5	1	1
5	1	2
5	1	1
5	1	1
5	1	1
5	1	1
5	1	1
5	1	1
5	1	1
5	1	1
5	1	1
5	4	1
5	1	1
5	1	1
5	1	1
5	1	1
5	1	1
5	1	1
5	1	1
5	1	1
5	1	2
5	1	1
5	1	1
5	1	1
5	1	1
5	1	2
5	1	2
5	1	1
5	1	1
5	1	1
5	1	1
5	1	1
5	1	1
5	1	2
5	1	1
5	1	1

5	1	1
5	1	1
5	1	1
5	1	1
5	1	1
5	1	1
5	4	1
5	1	1
5	1	1
5	1	1
5	1	2
5	3	1
5	4	1
5	1	1
5	1	1
5	1	1
5	1	1
5	1	1
5	1	1
5	1	1
5	1	1
5	1	2
5	1	1
5	1	1
5	3	1
5	1	1
5	1	1
5	1	1
5	1	2
5	1	1
5	1	1
5	1	1
5	1	1
5	1	1
5	3	2
5	1	1
5	2	2
5	1	2
5	1	1
5	3	1
5	1	1
5	1	2
5	1	1
5	1	1
5	1	1
5	1	1
5	1	1

	5	1	1
	5	1	2
	5	1	1
	5	1	1
	5	2	2
	5	1	1
	5	1	1
	5	1	1
	5	1	1
	5	1	1
	5	1	2
	5	1	1
	5	1	1
	5	1	1
	5	1	1
	5	1	1
	5	1	1
	5	1	2
	5	1	1
	5	1	1
	5	1	1
	5	1	1
1	5	1	1
	5	1	1
	5	1	1
	5	1	1
	5	1	1
	5	1	1
	5	1	1
	5	1	1
	5	1	1
	5	1	1
	5	1	1
	5	1	1
	5	1	1
	5	1	1
	5	1	1
	5	1	1
	5	1	1
	5	1	1
	5	1	1
	5	1	2
	5	1	2
	5	1	2
	5	1	1
	5	1	1
	5	1	1

5	1	1
5	4	1
5	1	1
5	1	1
5	1	1
5	1	2
5	1	2
5	1	1
5	1	2
5	1	1
5	1	1
5	1	1
5	4	1
5	1	2
5	1	1
5	1	1
5	1	1
5	4	1
5	1	1
5	1	1
5	1	2
5	1	1
5	1	1
5	1	2
5	1	1
5	4	1
5	1	2
5	1	2
5	1	1
5	1	1
5		2
5	1	1
5	1	1
5	1	1
5	1	2
5	1	1
5	1	1
5	1	1
5	1	1
5	1	1
5	1	1
5	1	1
5	1	1
5	1	1
5	1	1
5	1	1
5	1	1

5	1	1
5	1	1
5	1	1
5	1	2
5	1	1
5	1	1
5	1	1
5	1	1
5	1	2
5	1	1
5	1	1
5	4	1
5	1	1
5	1	2
5	1	1
5	1	1
5	1	1
5	1	1
5	1	1
5	1	1
5	1	1
5	1	1
5	1	1
5	1	1
5	1	1
5	1	1
5	1	2
5	1	2
5	1	1
5	1	1
5	1	1
5	1	2
5	1	1
5	1	1
5	1	1
5	1	2
5	1	1
5	1	1
5	1	1
5	1	1
5	1	1
5	1	1
5	1	1
5	1	1
5	1	1
5	1	1
5	2	1

	5	1	1
	5	1	1
	5	1	2
	5	1	1
	5	1	1
	5	1	1
	5	1	1
	5	1	2
	5	1	1
	5	1	1
	5	1	1
	5	1	2
	5	3	1
	5	1	1
	5	1	1
	5	1	1
	5	1	1
	5	1	2
	5	1	1
	5	1	1
	5	1	2
	5	1	1
	5	1	1
	5	1	1
	5	1	1
	5	1	1
	5	1	1
	5	1	1
	5	1	1
	5	1	1
1	5	1	1
	5	1	1
	5	4	1
	5	1	2
	5	1	2
	5	1	1
	5	1	2
	5	1	1
	5		1
	5	1	1
	5	4	1
	5	1	1
	5	1	1
	5	1	1
	5	1	2
	5	1	1
	5	1	2

5	1	2
5	1	1
5	1	1
5	1	1
5	3	2
5	1	1
5	1	1
5	1	1
5	1	1
5	1	1
5	1	1
5	1	1
5	1	1
5	1	2
5	1	1
5	1	1
5	1	1
5	4	1
5	4	2
5	1	2
5	1	2
5	1	1
5		1
5	1	1
5	1	1
5	1	1
5	4	2
5	1	1
5	1	2
5	1	2
5	1	1
5	1	1
5	1	1
5	2	1
5	1	2
5	1	1
5	1	1
5	1	1
5	3	1
5	1	2
5	1	1
5	1	1
5	1	1
5	1	1
5	1	2
5	1	1
5	1	1

5	1	1
5	1	1
5	1	2
5	1	2
5	1	1
5	1	1
5	1	1
5	1	2
5	1	1
5	1	1
5	4	1
5	1	1
5	1	1
5	1	1
5	1	2
5	1	1
5	1	1
5	1	1
5	1	1
5	1	1
5	1	1
5	1	1
5	1	1
5	1	2
5	1	1
5	1	1
5	1	1
5	1	2
5	1	1
5	1	1
5	1	1
5	1	1
5	1	1
5	1	1
5	1	1
5	1	1
5	1	2
5	1	2
5	1	1
5	1	2
5	1	1
5	1	1
5	1	1
5	1	1
5	1	1
5	1	1
5	1	1

5	1	1
5	1	1
5		1
5	1	1
5	1	1
5	4	2
5	1	1
5	1	2
5	1	1
5	1	2
5	1	1
5	1	1
5	1	1
5	1	1
5	1	1
5	1	1
5	3	1
5	1	1
5	1	1
5	1	1
5	1	1
5	1	2
5	1	1
5	1	1
5	1	2
5	1	1
5	1	2
5	1	1
5	1	2
5	1	1
5	1	1
5		2
5	1	1
5	1	1
5	1	1
5	1	1
5	1	1
5	1	1
5	1	1
5	1	1
5	1	1
5	1	1
5	1	1
5	1	1
5	1	2
5	1	1
5	1	2

5	1	1
5	1	1
5	1	1
5	1	1
5	1	1
5	1	1
5	4	1
5	1	1
5	1	1
5	1	1
5	1	1
5	1	1
5	1	1
5	1	1
5	1	1
5	1	1
5	1	1
5	1	1
5	1	2
5	1	1
5	3	1
5	1	1
5	1	2
5	1	1
5	4	1
5	1	1
5	1	1
5	1	1
5	1	1
5	1	2
5	1	1
5	1	2
5	1	1
5	1	2
5		1
5	1	1
5	1	1
5	1	1
5	1	1
5	1	2
5	1	1
5	1	1
5	1	2
5	1	1
5	1	2
5	1	1
5	1	1

5	1	1
5	4	2
5	1	1
5	1	1
5	1	1
5		2
5	1	2
5	1	1
5	1	1
5	1	2
5	1	2
5	1	1
5	1	2
5	1	1
5	1	1
5	1	1
5	1	1
5	1	2
5	1	1
5	1	1
5	1	1
5	1	1
5	1	1
5	1	1
5	1	1
5	1	1
5	1	1
5	1	1
5	1	1
5	1	1
5	1	1
5	1	2
5	1	1
5	1	1
5	1	1
5	1	1
5	1	1
5	1	1
5	1	1
5	1	1
5	1	1
5	1	1
5	1	1
5	4	1
5	1	1
5	1	1
5	1	1

5	1	2
5	1	1
5	1	1
5	1	1
5	1	1
5	1	1
5	1	1
5	1	1
5	1	2
5	1	1
5	1	1
5	1	1
5	1	2
5	1	1
5	1	1
5	1	1
5	4	1
5	4	1
5	1	1
5	4	2
5	1	2
5	1	2
5	1	1
5	1	1
5	1	1
5	1	1
5	1	1
5	1	1
5	1	1
5	1	1
5	1	2
5	1	2
5	1	2
5	1	1
5	1	1
5	1	1
5	1	1
5	1	2
5	1	2
5	1	1
5	1	2
5	1	1
5	1	1
5	1	1
5	1	1
5	1	1
5	1	1

5	1	1
5	1	1
5	1	1
5	1	2
5	1	1
5	1	1
5	1	2
5	1	1
5	1	1
5	1	1
5	1	2
5	1	1
5	1	1
5	1	1
5	1	1
5	1	1
5	1	2
5	1	1
5	1	2
5	1	1
5	1	1
5	1	1
5	1	1
5	1	1
5	1	1
5	1	2
5	1	1
5	1	1
5	1	1
5	4	1
5	1	1
5	1	1
5	1	1
5	1	1
5	1	1
5	1	1
5	1	1
5	1	1
5	1	1
5	1	1
5	1	1
5	4	1
5	1	1
5	3	2
5	1	1
5	1	1
5	1	1

5	1	1
5	1	1
5	1	1
5	1	1
5	1	2
5	1	1
5	1	1
5	1	1
5	1	2
5	3	2
5	1	1
5	1	1
5	1	1
5	1	1
5	1	1
5	1	1
5	1	2
5	1	2
5	4	1
5	1	1
5	1	1
5	2	2
5	1	1
5	1	1
5	3	1
5	1	2
5	1	1
5	1	2
5	1	1
5	1	2
5	1	2
5	1	1
5	1	1
5	1	2
5	1	1
5	1	2
5	1	1
5	3	1
5	1	1
5	1	1
5	1	1
5	1	2
5	1	1
5	1	1
5	1	2
5	1	1
5	1	1

5		1
5	1	1
5	1	2
5	1	2
5	1	1
5		1
5	1	2
5	1	1
5	1	2
5	1	1
5	1	2
5	1	2
5	1	1
5	1	1
5	1	1
5	1	1
5	4	1
5	1	1
5	1	1
5	1	1
5	4	1
5	1	2
5	1	1
5	1	1
5	1	1
5	1	1
5	1	1
5	1	2
5	1	1
5	1	1
5	4	1
5	1	1
5	1	2
5	1	2
5	1	1
5	1	1
5	1	1
5	1	2
5	1	1
5	1	2
5	1	1
5	1	2
5	1	2
5	1	1
5	1	1
5	1	1
5	1	1

5	1	1
5	1	1
5	3	1
5	1	1
5	1	1
5	1	1
5	3	1
5	1	2
5	1	1
5	1	1
5	1	1
5	1	1
5	1	1
5	1	1
5	1	1
5	1	1
5	1	1
5	1	2
5	1	1
5	1	1
5	1	1
5	1	2
5	1	1
5	1	1
5	1	1
5	1	1
5	1	1
5	1	1
5	1	1
5	1	1
5	1	1
5	1	1
5	1	1
5	1	1
5	1	1
5	4	1
5	1	2
5	1	2
5	1	2
5	1	1
5	1	1
5	1	1
5	1	2
5	1	1
5	1	1
5	1	1
5	1	1

5	1	1
5	1	1
5	1	1
5	1	2
5	1	1
5	1	2
5	1	1
5	1	1
5	1	2
5	1	1
5	1	1
5	1	1
5	1	1
5	1	1
5	1	2
5	1	1
5	1	1
5	1	1
5	3	1
5	1	1
5	1	1
5	1	1
5	1	1
5	1	1
5	1	1
5	1	1
5	1	1
5	1	1
5	1	1
5	1	1
5	1	1
5	1	2
5	4	1
5	1	1
5	1	1
5	1	2
5	1	2
5	1	2
5	1	1
5	1	1
5	1	1
5	1	1
5	1	1
5	1	1
5	1	1
5	1	1
5	1	1

5	1	1
5	1	2
5	1	2
5	1	1
5	1	2
5	1	1
5	1	1
5	1	1
5	1	1
5	1	1
5	1	1
5	1	1
5	1	1
5	1	1
5	1	1
5	1	1
5	1	1
5	1	1
5	1	2
5	1	2
5	1	1
5	4	1
5	1	2
5	1	1
5	1	2
5	1	2
5	1	1
5	1	1
5	1	2
5	1	1
5	1	1
5	1	1
5	1	1
5	1	2
5	1	2
5	1	1
5	1	2
5	1	1
5	3	1
5	1	2
5	1	2
5	1	1
5	1	1
5	1	2
5	1	1
5	1	1
5	1	1

5	1	1
5	1	1
5	4	1
5	1	1
5	1	1
5	1	1
5	1	1
5	1	1
5	1	1
5	1	2
5	1	1
5	1	2
5	1	1
5	1	1
5	4	1
5	1	1
5	1	1
5	1	1
5	1	1
5	1	1
5	1	2
5	1	2
5	1	1
5	1	1
5	1	1
5	1	1
5	1	2
5	1	1
5	1	2
5	1	1
5	1	1
5	1	1
5	1	1
5	1	2
5	1	1
5	1	2
5	1	1
5	1	2
5	4	1
5	1	1
5	1	1
5	1	2
5	1	1
5	1	1
5	1	1
5	1	1
5	1	1

5	1	1
5	1	2
5	1	1
5	1	1
5	1	1
5	1	2
5	1	1
5	1	1
5	1	1
5	1	1
5	1	1
5	1	1
5	3	1
5	1	1
5	1	1
5	3	1
5	1	2
5	1	1
5	1	2
5	1	1
5	1	2
5	1	1
5	1	1
5	1	2
5	1	2
5	1	1
5	1	1
5	1	1
5	1	1
5	1	1
5	1	1
5	1	1
5	1	2
5	1	1
5	1	2
5	1	1
5	1	2
5	1	1
5	4	1
5	1	1
5	1	1
5	1	1
5	1	1
5	1	1
5	1	1
5	1	1
5	1	1

5	1	1
5	1	1
5	1	2
5	1	1
5	1	2
5	1	1
5	1	1
5	1	1
5	1	1
5	1	2
5	1	2
5	1	2
5	1	1
5	1	2
5	1	1
5	1	2
5	1	1
5	3	1
5	1	1
5	1	1
5	1	1
5	1	1
5	1	2
5	1	1
5	1	1
5	1	1
5	1	1
5	1	1
5	1	1
5	1	2
5	1	1
5	1	1
5	1	2
5	1	1
5	1	1
5	1	1
5	1	1
5	1	1
5	1	2
5	1	1
5	1	1
5	1	1
5	1	2
5	1	1
5	1	1
5	1	1
5	1	1

5	4	1
5	1	1
5	1	2
5	1	2
5	1	1
5	1	1
5	1	1
5	1	2
5	1	2
5	1	2
5	1	2
5	1	1
5	1	1
5	1	1
5	4	2
5	1	1
5	1	2
5	1	1
5	1	1
5	1	1
5	1	1
5	1	2
5	1	1
5	1	1
5	1	1
5	1	1
5	1	2
5	1	2
5	1	1
5	1	1
5	1	1
5	1	2
5	1	1
5	1	2
5	2	1
5	1	1
5	4	1
5	1	1
5	1	1
5	1	1
5	1	1
5	1	1
5	1	1
5	1	1
5	1	1
5	1	1
5	1	1

5	1	1
5	1	2
5	1	1
5	1	1
5	1	2
5	1	1
5	4	1
5	1	1
5	1	1
5	3	2
5	4	1
5	1	1
5		1
5	1	2
5	1	2
5	1	1
5	1	1
5	1	1
5	1	1
5	1	1
5	1	1
5	1	1
5	1	1
5	1	1
5	1	1
5		1
5	1	1
5	1	1
5	1	2
5	1	1
5	1	2
5	1	1
5	1	1
5	1	1
5	1	1
5	1	1
5	1	1
5	1	1
5	1	1
5	1	1
5		1
5	1	1
5	1	1
5	1	1
5	4	1
5	1	1
5	1	1

5	1	1
5	1	1
5	1	1
5	1	1
5	1	1
5	1	1
5	1	2
5	1	1
5	1	1
5	1	1
5	1	1
5	1	1
5	1	1
5	1	1
5	1	1
5	1	2
5	1	1
5	1	2
5	1	1
5	1	1
5		1
5	1	1
5	1	1
5	1	1
5	1	2
5	1	2
5	1	1
5	4	1
5	1	1
5	1	1
5	1	1
5	1	1
5	1	1
5	1	2
5	1	1
5	1	2
5	4	1
5	4	1
5	1	1
5	1	1
5	1	1
5	1	1
5	4	1
5	1	1
5	1	1
5	1	1
5	1	2

5	1	2
5	4	2
5	1	1
5	1	1
5	1	1
5	1	1
5	1	1
5	1	2
5	1	1
5	1	2
5	1	1
5	1	1
5	1	2
5	1	1
5	1	2
5	1	1
5	1	1
5	1	1
5	1	1
5	1	1
5	1	1
5	1	2
5	1	2
5	1	1
5	1	1
5	1	2
5	1	2
5	1	1
5	1	1
5	1	1
5	1	1
5	1	2
5	1	1
5	1	1
5	1	1
5	1	1
5	1	1
5	1	1
5	1	1
5	1	1
5	1	1
5	1	2
5	1	2
5	1	1
5	1	1
5	1	1
5	1	1

5	4	2
5	1	2
5	1	1
5	1	1
5	1	2
5	1	2
5	1	1
5	1	2
5	1	1
5	1	2
5	1	1
5	1	1
5	1	2
5	1	1
5	1	1
5	1	2
5	1	2
5	1	1
5		2
5	1	1
5	1	1
5	1	1
5	4	1
5	1	1
5	1	1
5	1	1
5	1	1
5	4	1
5	1	2
5	1	2
5	1	1
5	1	1
5	1	1
5	1	1
5	1	1
5	1	1
5	1	1
5	1	1
5	1	2
5	1	1
5	1	2
5	1	1
5	1	2
5	4	1
5	1	1
5	1	2
5	1	2

5	4	1
5	1	1
5	1	1
5	1	1
5	1	2
5	1	1
5	4	2
5	1	1
5	1	1
5	1	1
5	1	1
5	1	1
5	1	1
5	1	1
5	4	1
5	1	1
5	1	1
5	1	1
5	1	2
5	1	2
5	4	1
5	1	1
5	1	1
5	1	1
5	1	1
5	1	1
5	1	1
5	1	2
5	4	2
5	3	1
5	1	1
5	1	1
5	1	2
5	1	1
5	1	1
5	1	2
5	1	1
5	1	1
5	1	2
5	1	1
5	1	2
5	1	1
5	1	1
5	1	2
5	1	2
5	1	1
5	1	1

5	1	2
5	1	1
5	1	1
5	1	1
5		1
5	1	1
5	1	1
5	3	1
5	1	1
5	1	1
5	3	1
5	1	1
5	1	2
5	4	2
5	3	2
5	1	1
5	1	1
5	1	2
5	1	1
5	1	1
5	1	1
5	1	2
5	1	1
5	1	1
5	1	1
5	1	1
5	1	2
5	1	1
5	1	2
5	4	2
5	1	1
5	3	1
5		1
5	1	1
5	1	1
5	1	1
5	3	1
5	1	1
5	1	2
5	1	2
5	1	1
5	1	2
5	2	1
5	1	2
5	1	1
5	1	1
5	1	1

5	1	2
5	1	2
5	1	1
5	1	2
5	1	1
5	1	1
5	1	2
5	1	1
5	1	1
5	1	1
5	1	2
5	1	2
5	1	2
5	1	1
5	3	1
5	1	1
5	1	1
5	1	1
5		2
5	1	2
5	1	1
5	1	2
5	1	2
5	1	1
5	1	2
5	1	1
5	1	1
5	1	1
5	1	1
5	1	1
5	1	1
5	1	1
5	1	1
5	1	1
5	1	1
5	1	1
5	1	1
5	1	2
5	1	1
5	1	2
5	1	1
5	1	1
5	1	1
5	1	2
5	1	2
5	1	1
5	1	2

5	4	1
5	1	1
5	1	1
5	1	1
5	1	1
5	1	1
5	1	1
5	1	1
5	1	1
5	1	1
5	1	2
5	1	1
5	1	1
5	1	2
5	1	2
5	1	2
5	1	1
5	1	1
5	1	1
5	1	1
5	1	1
5	1	1
5	1	1
5	1	1
5	1	1
5	1	1
5	1	1
5	1	1
5	1	1
5	1	2
5	1	2
5	1	1
5	1	1
5	1	1
5	1	1
5	1	1
5	1	1
5	1	1
5	1	1
5	3	1
5	1	1
5	1	1
5	1	1
5	1	2
5	1	1
5	1	1
5	1	1

5	1	1
5	1	1
5	1	1
5	1	2
5	1	1
5	3	1
5	1	2
5	1	2
5	1	1
5	1	1
5	1	1
5	1	1
5	1	1
5	4	1
5	1	1
5	1	1
5	1	1
5	1	2
5	1	2
5	1	1
5	1	1
5	1	2
5	1	2
5	3	1
5	1	1
5	2	1
5	1	1
5	1	2
5	1	2
5	1	1
5	1	1
5	1	2
5	1	1
5	1	1
5	1	1
5	1	1
5	1	1
5	1	1
5	1	1
5	1	1
5	1	1
5	1	1
5	1	1
5	1	1
5	1	2
5	1	1
5	4	1
5	1	1

5	1	1
5	1	1
5	1	1
5	1	1
5	1	2
5	1	2
5	1	2
5	1	1
5	1	1
5	1	1
5	1	1
5	1	1
5	1	1
5	1	1
5	1	1
5	1	1
5	1	1
5	4	1
5	1	1
5	1	1
5	1	2
5	1	1
5	1	1
5	1	1
5	1	1
5	1	1
5	1	1
5	1	1
5	1	1
5	1	1
5	1	2
5	1	2
5	1	1
5	1	1
5	1	2
5	1	1
5	1	1
5	1	1
5	3	1
5	4	1
5	1	1
5	1	2
5	1	1
5	1	1
5	1	1
5	1	1
5	1	1

5	1	1
5	1	2
5	1	1
5	1	2
5	1	1
5	1	2
5	1	1
5	1	1
5	1	1
5	1	1
5	1	1
5	1	1
5	1	1
5	1	1
5	1	1
5	1	1
5	1	2
5	1	1
5	1	1
5	1	1
5	1	2
5	1	2
5	1	2
5	1	1
5	1	1
5	1	1
5	1	2
5	1	1
5	1	1
5	1	1
5	1	1
5	1	1
5	1	2
5	1	1
5	1	2
5	1	2
5	1	1
5	1	1
5	1	1
5		1
5	3	1
5	1	1
5	1	1
5	1	2
5	1	2
5	1	1
5	1	1

5	1	1
5	1	1
5	1	2
5	1	1
5	1	2
5	1	1
5	1	1
5	1	1
5	1	1
5	1	1
5	1	2
5	1	1
5	4	2
5	4	1
5	1	2
5	4	2
5	1	1
5	1	2
5	1	1
5	1	1
5	1	1
5	1	1
5	1	1
5	1	1
5	1	1
5	1	1
5	1	1
5	1	2
5	1	1
5	1	1
5	1	1
5	1	1
5	1	1
5	1	2
5	1	2
5	4	1
5	1	1
5	1	1
5	1	1
5	1	1
5	1	2
5	1	1
5	1	1
5	1	1
5	1	1
5	1	2
5	1	2

5	1	2
5	1	1
5	1	1
5	1	1
5	1	2
5	1	1
5	1	1
5	3	1
5	1	1
5	1	1
5	1	1
5	1	2
5	1	1
5	1	1
5	1	1
5	1	2
5	1	2
5	4	1
5	1	2
5	1	2
5	1	1
5	1	1
5	1	1
5	1	1
5	1	1
5	1	2
5	1	1
5	1	1
5	1	1
5	4	2
5	1	1
5	1	1
5	1	2
5	1	1
5	2	1
5	1	1
5	1	2
5	1	1
5	1	2
5	1	1
5	1	2
5		1
5	1	1
5	1	2
5	1	2
5	1	2
5	1	1

5	1	2
5	1	2
5	1	1
5	1	1
5	1	1
5	1	1
5	1	1
5	1	2
5	1	2
5	1	1
5	1	1
5	1	1
5	1	2
5	1	1
5	1	2
5	1	1
5	1	1
5	1	1
5	1	1
5	4	1
5	1	2
5	1	2
5	1	1
5	1	1
5	1	2
5	1	1
5	1	2
5	4	1
5	1	2
5	1	1
5	1	1
5	1	1
5	1	2
5	1	1
5	1	2
5	1	2
5	1	1
5	1	2
5	1	2
5	1	1
5	1	2
5	1	1
5	1	2
5	1	1
5	1	1
5	1	1
5	1	1

5	1	1
5		1
5	1	2
5	1	1
5	1	2
5	1	1
5	1	1
5	1	1
5	4	1
5	1	2
5	1	1
5	1	1
5	1	1
5	1	1
5	1	1
5	1	1
5	1	1
5	1	1
5	1	1
5	1	1
5	1	1
5	1	1
5	1	1
5	1	1
5	1	2
5	1	1
5	1	1
5	1	2
5	1	2
5	1	1
5	1	1
5	1	1
5	1	1
5	1	1
5	1	1
5	1	1
5	1	2
5	1	1
5	1	2
5	1	1
5	1	1
5	1	1
5	1	1
5	1	2
5	1	1
5	1	1
5	1	1

5	1	1
5	4	2
5	1	2
5	1	1
5	1	1
5	1	1
5	2	1
5	1	1
5	1	2
5	4	1
5	1	1
5	1	2
5	1	1
5	1	1
5	1	1
5	1	1
5	1	1
5	1	1
5	1	1
5	1	1
5	1	1
5	1	1
5	1	1
5	1	1
5	1	2
5	1	1
5	1	1
5	1	1
5	1	1
5	3	1
5	1	1
5	1	1
5	1	1
5	1	1
5	1	2
5	1	2
5	1	1
5	1	1
5	1	1
5	1	2
5	1	1
5	1	1
5	1	1
5	1	2
5	1	1
5	1	1
5	1	1

5	3	1
5	1	1
5	1	1
5	1	2
5	1	1
5	1	1
5		1
5	1	2
5	1	2
5	1	1
5	1	1
5	1	1
5	1	1
5	1	1
5	1	2
5	1	1
5	1	2
5	1	1
5	1	1
5	4	1
5	1	1
5	1	1
5	1	2
5	1	1
5	1	2
5	1	1
5	1	2
5	1	1
5	1	1
5	1	1
5	1	2
5	1	1
5	1	2
5	1	2
5	1	2
5	1	1
5	1	2
5	1	1
5	1	1
5	1	2
5	1	2
5	1	2
5	1	2
5	1	1
5	1	1
5	1	1
5	1	1

5	1	1
5	1	1
5	3	1
5	1	1
5	1	1
5	1	1
5	1	1
5	1	2
5	1	1
5	1	1
5	1	1
5	4	1
5	1	1
5	1	1
5	1	2
5	1	1
5	1	1
5	4	1
5	1	1
5	1	1
5	1	1
5	1	1
5	1	1
5	1	1
5	1	2
5	1	1
5	1	1
5	1	2
5	1	1
5	1	1
5	1	1
5	4	2
5	1	1
5	1	1
5	1	2
5	1	1
5	1	1
5	1	1
5	1	1
5	1	2
5	1	2
5	1	2
5	1	1
5	1	1
5	1	1
5	1	1
5	1	1

5	1	1
5	1	1
5	1	1
5	1	1
5	1	1
5	1	2
5	1	1
5	4	1
5	1	1
5	1	1
5	1	2
5	1	1
5	1	1
5	1	1
5	1	1
5	1	2
5	1	2
5	1	2
5	1	1
5	4	2
5	1	2
5	1	1
5	1	1
5	1	1
5	1	1
5	1	1
5	1	1
5	1	1
5	1	2
5	1	1
5	1	1
5	1	2
5	1	1
5	4	1
5	1	1
5	1	1
5	1	1
5	1	1
5	1	2
5	1	2
5	1	2
5	4	2
5	1	1
5	1	1
5	1	2
5	1	2
5	1	1

5	1	1
5	1	1
5	1	1
5	1	2
5	1	1
5	1	1
5	1	1
5	1	2
5	1	1
5	1	1
5	1	2
5	1	1
5	1	1
5	1	1
5	1	1
5	1	1
5	1	2
5	1	1
5	1	2
5	1	2
5	1	2
5	1	1
5	1	2
5	1	1
5	1	1
5	1	1
5	1	2
5	1	2
5	1	1
5	1	1
5	1	1
5	1	2
5	1	2
5	1	1
5	1	1
5	1	1
5	1	1
5	1	1
5	1	1
5	1	1
5	1	2
5	1	1
5	1	1
5	1	1
5	4	1
5	1	1
5	1	1

5	1	1
5	1	1
5	1	2
5	1	2
5	1	2
5	1	1
5	1	1
5	1	1
5	1	1
5	1	1
5	1	1
5	1	1
5	1	1
5	1	1
5	1	2
5	4	1
5	1	2
5	1	1
5	1	1
5	1	1
5	1	1
5	3	1
5	1	1
5	1	1
5	1	2
5	1	1
5	1	1
5	1	1
5	1	1
5	1	1
5	1	1
5	1	1
5	1	1
5	1	1
5	1	1
5	1	1
5	1	1
5	1	1
5	1	1
5	1	1
5	1	1
5	1	1
5	1	1
5	1	2
5	1	1
5	1	1
5	1	1

5	1	2
5	1	1
5	1	1
5	1	2
5	1	2
5	1	1
5	1	1
5	1	1
5	1	1
5	1	1
5	1	1
5	1	2
5	1	1
5	1	1
5	1	1
5	1	1
5	1	1
5	1	1
5	1	2
5	1	1
5	1	1
5	1	1
5	1	1
5	1	2
5	1	1
5	1	2
5	1	1
5	1	1
5	1	2
5	1	2
5	1	1
5	1	1
5	1	2
5	1	1
5	1	2
5	1	1
5	1	1
5	1	1
5	1	1
5	1	1
5	1	1
5	1	1
5	1	1
5	1	1
5	1	1
5	1	1
5	1	1

5	1	1
5	1	2
5	1	1
5	1	1
5	3	1
5	1	2
5	1	1
5	3	1
5	1	1
5	1	2
5	1	1
5	1	2
5	1	1
5	1	2
5	1	2
5	1	1
5	4	2
5	1	1
5	1	1
5	1	1
5	1	1
5	1	2
5	1	2
5	1	1
5	1	1
5	1	2
5	1	1
5	1	1
5	1	1
5	1	1
5	1	2
5	1	1
5	1	1
5	1	1
5	1	2
5	1	1
5	1	2
5	1	1
5	1	1
5	1	1
5	1	1
5	1	1
5	1	1
5	1	2
5	1	1
5	4	1
5	1	1

5	1	2
5	1	1
5	1	1
5	1	1
5	1	1
5	1	1
5	1	1
5	1	1
5	1	1
5	1	2
5	1	2
5	1	1
5	1	1
5	1	1
5	1	1
5	1	1
5	1	1
5	1	1
5	1	1
5	1	1
5	1	1
5	1	1
5	1	2
5	1	1
5	1	1
5	1	2
5	1	1
5	1	1
5	1	2
5	1	2
5	1	1
5	1	2
5	1	1
5	1	2
5	1	2
5	1	1
5	1	1
5	1	2
5	1	1
5	1	1
5	1	1
5	1	1
5	1	1
5	1	1
5	1	1
5	1	1
5	1	2

5	1	2
5	1	1
5	1	1
5	1	2
5	1	1
5	1	2
5	1	2
5	1	1
5	1	2
5	1	2
5	1	1
5	1	1
5	1	1
5	1	1
5	1	1
5	1	1
5	1	1
5	1	1
5	1	1
5	1	1
5	1	1
5	1	1
5	1	1
5	1	1
5	1	2
5	1	1
5	1	1
5	1	1
5	1	1
5	1	1
5	1	2
5	1	2
5		1
5	1	1
5	1	1
5	1	2
5	4	2
5	1	1
5	1	2
5	1	1
5	1	1
5	1	1
5	1	1
5	1	1
5	1	2
5	1	1
5	1	2

5	1	2
5	1	1
5	1	1
5	1	2
5	4	1
5	1	1
5	1	1
5	1	1
5	1	1
5	1	1
5	1	1
5	1	1
5	1	2
5	1	1
5	1	1
5	1	1
5	1	1
5	4	1
5	1	2
5	1	2
5	1	2
5	1	2
5	1	1
5	3	1
5	1	1
5	1	1
5	1	1
5	2	1
5	1	1
5	1	1
5	1	1
5	1	2
5	1	1
5	1	1
5	3	1
5	1	1
5	1	1
5	1	2
5	1	1
5	1	1
5	1	1
5	1	1
5	1	1
5	1	2
5	1	1
5	1	1
5	1	1

5	1	1
5	1	1
5	1	1
5	1	1
5	1	1
5	1	1
5	1	2
5	1	1
5	1	1
5	1	1
5	1	2
5	1	1
5	1	2
5	1	1
5	1	2
5	1	1
5	1	1
5	1	1
5	1	1
5	1	1
5	1	1
5	1	1
5	1	1
5	1	2
5	1	1
5	1	2
5	1	1
5	1	1
5	1	1
5	4	1
5	1	1
5	1	1
5	1	2
5	1	2
5	1	1
5	1	1
5	1	1
5	1	1
5	1	2
5	1	1
5	1	1
5	1	1
5	1	2
5	3	1
5	1	1
5	1	2
5	1	1

5	1	1
5	1	1
5	3	1
5	1	1
5	1	1
5	1	1
5	1	1
5	1	1
5	1	1
5	1	2
5	1	1
5	1	1
5	1	2
5	1	1
5	1	1
5	1	2
5	1	1
5	1	2
5	1	2
5	1	1
5	1	1
5	1	2
5	1	1
5	1	2
5	1	1
5	1	1
5	1	1
5	1	1
5	1	2
5	1	1
5	1	2
5	1	1
5	1	1
5	1	1
5	1	1
5	1	1
5	1	1
5	1	1
5	4	1
5	1	1
5	1	1
5	1	1
5	1	1
5	1	1
5	1	2
5	1	2
5	1	1

5	1	1
5	1	1
5	1	1
5	1	1
5	1	1
5	1	1
5		1
5	1	2
5	1	1
5	1	1
5	1	1
5	1	1
5	1	1
5	1	1
5	1	1
5	1	1
5	1	2
5	4	1
5	4	2
5	1	1
5	1	2
5	1	1
5	1	1
5	1	1
5	1	1
5	1	2
5	1	2
5	3	1
5	1	2
5	1	2
5	4	1
5	1	1
5	1	1
5	1	2
5	1	2
5	1	1
5	1	1
5	1	1
5	1	1
5	1	1
5	1	1
5	1	1
5	1	1
5	1	1
5	1	1
5	1	1
5	1	1

5	1	1
5	1	1
5	1	1
5	1	1
5	1	2
5	1	1
5	4	1
5	3	1
5	1	2
5	1	1
5	1	1
5	1	2
5	1	1
5	1	2
5	1	1
5	1	1
5	1	1
5	1	2
5	3	1
5	1	2
5	1	1
5	1	1
5	1	1
5	1	1
5	1	1
5	1	1
5	1	2
5	1	1
5	1	1
5	1	1
5	1	2
5	1	1
5	1	1
5	1	1
5	1	1
5	1	1
5	1	1
5	1	1
5	1	2
5	1	1
5	1	1
5	1	1
5	1	1
5	1	1
5	1	1
5	1	1
5	1	1

5	4	1
5	1	1
5	1	1
5	1	1
5	1	1
5	1	1
5	1	1
5	1	1
5	1	2
5	1	1
5	1	1
5	1	1
5	1	2
5	1	2
5	1	1
5	4	2
5	1	1
5	1	1
5	1	1
5	1	1
5	1	1
5	1	2
5	1	1
5	1	1
5	1	2
5	1	1
5	1	2
5	1	2
5	1	2
5	1	2
5	1	2
5	1	1
5	1	2
5	1	2
5	1	2
5	1	1
5	1	1
5	1	2
5	3	2
5	1	1
5	1	1
5	1	2
5	1	1
5	1	1
5	1	1
5	1	1
5	1	1

5	1	1
5	1	2
5	1	1
5	1	1
5	1	1
5	1	1
5	1	2
5	1	1
5	1	1
5	1	1
5	1	1
5	1	1
5	1	1
5	1	1
5	1	1
5	4	1
5	1	1
5	1	2
5	1	1
5	1	1
5	4	2
5	1	2
5	1	1
5	1	1
5	1	1
5	1	1
5	1	2
5	1	2
5	1	2
5	1	1
5	1	1
5	4	1
5	1	2
5	1	1
5	1	2
5	1	2
5	1	2
5	1	1
5	1	1
5	1	1
5	1	1
5	1	2
5	3	1
5	1	1
5	1	1
5	1	2
5	1	1

5	1	1
5	1	2
5	1	1
5	4	1
5	1	1
5	1	1
5	1	1
5	1	1
5	1	1
5	1	1
5	1	1
5	1	1
5	1	2
5	1	1
5	1	2
5		1
5	1	1
5	1	1
5	1	1
5	1	1
5	3	1
5	1	1
5	1	1
5	1	1
5	1	2
5	1	1
5	1	1
5	1	1
5	1	2
5	1	1
5	1	1
5	1	2
5	1	1
5	1	1
5	1	1
5	1	1
5	1	1
5	1	2
5	1	1
5	1	1
5	1	1
5	1	1
5	4	2
5	1	1
5	1	1
5	1	1
5	3	2

5	1	1
5	1	1
5	1	2
5	1	1
5	1	1
5	1	1
5	1	1
5	1	1
5	1	1
5	1	1
5	1	1
5	1	1
5	1	2
5	4	1
5	1	1
5	1	1
5	1	1
5	1	1
5	1	1
5	1	2
5	1	1
5	1	1
5	1	1
5	1	1
5	1	1
5	1	1
5	1	2
5	1	1
5	1	1
5	1	1
5		1
5	4	1
5	1	1
5	1	1
5	1	1
5	1	1
5	1	1
5	1	1
5	1	1
5	4	2
5	1	1
5		2
5	1	2
5	1	1
5	1	1
5	1	1
5	1	2

5	1	2
5	1	1
5	1	1
5	1	1
5	3	1
5	1	1
5	1	2
5	1	1
5	1	1
5	1	1
5	1	1
5		1
5	1	1
5	1	1
5	1	1
5	1	2
5	1	1
5	1	1
5	1	1
5	1	2
5	1	2
5	1	1
5	3	1
5	1	1
5	1	1
5	1	1
5	1	1
5	1	1
5	1	2
5	1	1
5	1	2
5	1	2
5	4	1
5	1	2
5	1	1
5	1	2
5	1	1
5	1	1
5	1	1
5	1	1
5	1	2
5	1	1
5	1	1
5	1	1
5	1	1
5		1
5	1	1

5	1	1
5	1	1
5	1	1
5	1	1
5	1	1
5	1	2
5	1	1
5	1	1
5	3	1
5	1	1
5	1	2
5	1	1
5	1	2
5	1	1
5	1	1
5	1	2
5	1	2
5	1	1
5	1	1
5	1	2
5	1	1
5	1	1
5	1	1
5	1	1
5	1	1
5	1	1
5	1	1
5	1	1
5	1	2
5	1	1
5	1	1
5	1	2
5	1	1
5	1	1
5	4	2
5	1	1
5	1	1
5	1	1
5	1	2
5	1	1
5	1	1
5	1	1
5	1	1
5	1	1
5	1	2
5	4	2
5	1	2

5	1	1
5	1	1
5	1	1
5	1	1
5	1	1
5	1	1
5	1	1
5	1	1
5	1	2
5	4	1
5	1	1
5	1	2
5	1	1
5	1	2
5	3	1
5	1	1
5	1	1
5	1	2
5	1	1
5	1	2
5	1	1
5	1	1
5	1	1
5	1	1
5	1	2
5	1	1
5	1	1
5	1	1
5	1	2
5	1	1
5	1	1
5	1	1
5	1	2
5	1	2
5	1	1
5	1	2
5	1	1
5	1	1
5	1	1
5	1	1
5	1	1
5	1	2
5	4	1
5	1	1
5	4	2
5	1	1
5	1	1

5	1	1
5	1	1
5	1	1
5	1	2
5	1	2
5	1	1
5	1	1
5	1	1
5	1	2
5	1	2
5	1	1
5	1	2
5	1	2
5	4	1
5	1	1
5	1	1
5	1	1
5	4	1
5	1	1
5	1	1
5	1	1
5	1	1
5	1	1
5	1	1
5	1	2
5	1	1
5	1	2
5	3	1
5	1	1
5	1	1
5	1	1
5	1	2
5	1	1
5	1	1
5	1	1
5		2
5	1	2
5	4	2
5	1	1
5	1	2
5	1	2
5	1	1
5	1	1
5	1	1
5	1	1
5	1	1
5	1	1

5	1	2
5	1	2
5	1	1
5	1	1
5	1	1
5	1	1
5	1	1
5	1	1
5	1	1
5	1	1
5	1	1
5	1	1
5	1	1
5	4	1
5	1	1
5	1	1
5	1	1
5	1	1
5	1	2
5	1	1
5	1	1
5	1	1
5	1	1
5	1	2
5	1	1
5	1	2
5	1	1
5	1	1
5	1	1
5	1	1
5	1	1
5	1	1
5	1	1
5	1	1
5	1	1
5	1	1
5	1	1
5	1	2
5	1	1
5	1	2
5	1	1
5	1	1
5	1	1
5	1	1
5	1	1
5	1	1
5	1	1

5	1	1
5	1	1
5	1	1
5	1	2
5	1	1
5	1	1
5	1	1
5	1	1
5	1	2
5	1	1
5	1	2
5	1	1
5	1	1
5	1	1
5	1	1
5	1	1
5	1	1
5	1	1
5	1	1
5	1	1
5	1	1
5	1	1
5	1	2
5	1	1
5	1	1
5	1	1
5	1	1
5	1	1
5	1	1
5	1	1
5	1	1
5	1	2
5	1	2
5	1	1
5	1	2
5	1	1
5	1	1
5	1	2
5	1	1
5	1	1
5	1	1
5	1	1
5	1	1
5	1	1
5	1	1
5		1
5	1	1

5	1	1
5	1	1
5	1	1
5	1	1
5	1	1
5	1	1
5	1	1
5	1	1
5	1	1
5	1	2
5	1	2
5	1	2
5	1	1
5	1	1
5	1	1
5	1	2
5	1	1
5	1	1
5	1	2
5	4	1
5	4	1
5	1	1
5	1	1
5	1	2
5	1	1
5	1	1
5	1	1
5	4	2
5	1	1
5	1	2
5	1	1
5	1	1
5	1	2
5	1	1
5	1	2
5	1	2
5	1	2
5	1	1
5	1	1
5	3	1
5	1	2
5	1	1
5	1	1
5	1	1
5	1	1
5	3	2
5	1	1

5	1	1
5	1	1
5	1	1
5	1	2
5	1	1
5	1	2
5	1	1
5	4	1
5	1	1
5	1	1
5	1	2
5	1	2
5	1	2
5	1	1
5	1	1
5	4	1
5	1	1
5	1	2
5	1	1
5	1	1
5		1
5	1	1
5	1	1
5	1	1
5	1	1
5	1	2
5	1	1
5	1	2
5	1	1
5	1	1
5	1	2
5	1	1
5	1	1
5	1	1
5	1	1
5	1	1
5	1	1
5	1	1
5	1	1
5	1	1
5	1	1
5	1	1
5	1	1
5	1	1
5	1	1
5	1	1
5	1	1

5	1	1
5	1	1
5	1	1
5	1	1
5	1	2
5	1	1
5	1	1
5	1	1
5	1	1
5	1	2
5	1	2
5	1	2
5	1	2
5	1	1
5	1	1
5	1	2
5	1	1
5	1	1
5	1	1
5	1	1
5	4	1
5	1	1
5	1	2
5	1	2
5	1	1
5	1	1
5	1	1
5	1	1
5	1	1
5	1	1
5	1	1
5	1	1
5	1	1
5	1	1
5	1	1
5	1	2
5	1	1
5	1	1
5	1	1
5	1	2
5	1	1
5	1	1
5	1	2
5	1	1
5	1	1
5	1	2
5	1	1

5	1	1
5	1	1
5	1	1
5	1	1
5	1	2
5	4	1
5	1	2
5	1	1
5	2	1
5	1	1
5	1	1
5	1	1
5	1	1
5	1	1
5	1	1
5	1	2
5	1	1
5	1	1
5	1	1
5	1	1
5	1	1
5	3	1
5	1	2
5	1	1
5	1	2
5	1	2
5	1	2
5	1	1
5	1	1
5	1	1
5	1	1
5	1	1
5	1	2
5	1	1
5	1	1
5	1	1
5	1	1
5	1	1
5	1	1
5	1	1
5	1	1
5	1	1
5	1	1
5	1	1
5	1	1
5	1	1
5	4	1

5	1	1
5	1	1
5	1	1
5	1	1
5	1	2
5	1	1
5	1	2
5	1	1
5	1	2
5	1	1
5	4	1
5	4	1
5	1	2
5	1	1
5	1	2
5	1	2
5	1	1
5	1	1
5	1	1
5	1	2
5	1	2
5	2	1
5	1	2
5	1	1
5	1	1
5	1	1
5	1	1
5	1	2
5	1	1
5	1	1
5	1	1
5	1	2
5	1	1
5	1	1
5	1	2
5	1	2
5	1	1
5	1	1
5	1	1
5	1	1
5	1	1
5	1	1
5	1	1
5	1	1
5	1	1
5	1	1
5	1	1

5	4	1
5	1	1
5	1	1
5	1	2
5	1	1
5	1	1
5	1	1
5	1	1
5	1	1
5	1	2
5	1	1
5	1	1
5	1	1
5	1	2
5	1	1
5	1	1
5	1	1
5	1	1
5	1	1
5	1	2
5	4	1
5	3	2
5	1	1
5	1	2
5	1	1
5	1	2
5	1	2
5	1	1
5	1	1
5	1	1
5	1	1
5	2	1
5	1	1
5	1	1
5	1	1
5	1	1
5	1	1
5	1	2
5	1	1
5	4	1
5	1	1
5	1	1
5	1	1
5	1	1
5	1	1
5	1	1
5	1	1

5	1	2
5	1	1
5	1	1
5	1	1
5	1	1
5	1	1
5	1	1
5	1	1
5	1	1
5	1	1
5	1	1
5	1	1
5	1	1
5	1	1
5	4	1
5	1	1
5	1	1
5	1	2
5	1	1
5	1	1
5	1	1
5	1	1
5	1	1
5	1	1
5	1	1
5	1	1
5	1	1
5	1	1
5	1	1
5	1	1
5	1	1
5	1	1
5	1	1
5	1	1
5	1	1
5	1	1
5	1	1
5	1	1
5	1	1
5	1	1
5	1	1
5	1	1
5	4	2
5	1	1
5	1	1
5	1	1
5	1	1

5	1	1
5	1	1
5	1	1
5	1	1
5	1	1
5	1	1
5	1	2
5	1	1
5	1	1
5	1	1
5	1	1
5	1	2
5		1
5	1	1
5	1	1
5	1	1
5	1	1
5	1	1
5	1	1
5	1	2
5	1	1
5	1	1
5	1	2
5	1	1
5	1	1
5	1	1
5	1	1
5	1	1
5	1	2
5	1	1
5	1	1
5	1	1
5	1	1
5	1	1
5	1	1
5	1	1
5	1	1
5	1	1
5	1	1
5	1	1
5	1	1
5	1	1
5	1	1
5	1	1
5	1	1
5	1	1
5	1	1

5	1	1
5	1	1
5	1	1
5	1	1
5	1	1
5	1	1
5	1	1
5	1	1
5	1	1
5	1	1
5	1	1
5	1	1
5	1	1
5	1	1
5	1	2
5	1	1
5	1	1
5	1	1
5	1	2
5	1	2
5	1	1
5	1	1
5	1	1

DT_EVOLUC	DT_ENCERR	DT_DIGITA	HISTO_VGM	PAIS_VGM	CO_PS_VGM	O_PS_VGM	DT_VGM	DT_RT_VGM	PCR_SARS2
04/07/202	17/09/202	17/09/202	9						1
14/01/202	26/03/202	14/01/202	0						1
04/01/202	15/03/202	11/03/202	0						1
01/05/202	13/05/202	27/04/202	2						
04/09/202	08/09/202	20/07/202	2						1
25/08/202	25/08/202	05/08/202	2						1
27/08/202	31/08/202	03/08/202	2						1
23/09/202	23/09/202	30/09/202	2						
07/10/202	08/10/202	23/09/202	0						
10/05/202	31/08/202	31/08/202	2						1
28/08/202	09/09/202	09/09/202	0						1
01/01/202	02/01/202	09/12/202	2						1
04/02/202	04/02/202	14/01/202	0						
18/12/202	25/02/202	27/01/202	0						1
29/03/202	05/04/202	05/04/202	0						
02/06/202	18/06/202	18/05/202	2						1
02/07/202	25/09/202	17/06/202	0						1
19/07/202	21/07/202	08/07/202	2						1
25/07/202	30/07/202	16/07/202	2						1
28/09/202	30/09/202	16/09/202	0						
14/12/202	30/12/202	30/12/202	0						
15/01/202	18/01/202	18/01/202	2						1
07/06/202	09/06/202	31/05/202	2						1
03/07/202	03/07/202	09/06/202	2						1
06/05/202	06/05/202	11/06/202	9						
17/06/202	09/07/202	08/06/202	0						1
21/06/202	11/06/202	03/07/202	2						
03/12/202	03/12/202	28/11/202	2						1
30/10/202	13/01/202	19/10/202	0						1
10/09/202	04/09/202	04/09/202	0						
05/06/202	08/06/202	19/05/202	2						1
28/06/202	22/06/202	11/06/202	0						1
11/10/202	26/10/202	07/10/202	2						1
08/11/202	08/11/202	12/11/202	2						1
07/01/202	07/01/202	07/01/202	9						1
30/06/202	01/07/202	23/06/202	0						1
29/08/202	05/09/202	20/08/202	2						1
12/07/202	29/07/202	26/06/202	2						1
27/07/202	03/08/202	20/07/202	2						1
24/08/202	05/11/202	09/08/202	0						1
28/02/202	03/03/202	11/01/202	2						1
07/05/202	15/05/202	23/04/202	0						1
04/05/202	01/06/202	01/05/202	2						1
15/04/202	20/04/202	09/04/202	2						1
24/03/202	24/03/202	24/03/202	0						
14/08/202	23/01/202	23/01/202	2						

01/07/202	02/07/202	26/06/202	0	1
30/04/202	30/04/202	27/04/202	9	1
27/07/202	05/08/202	18/06/202	0	
10/06/202	13/07/202	13/07/202	2	
14/07/202	14/07/202	14/07/202	9	1
12/05/202	17/09/202	17/09/202	2	1
04/12/202	04/12/202	20/11/202	2	1
05/01/202	11/01/202	31/12/202	0	1
15/05/202	03/09/202	11/05/202	2	1
02/08/202	04/08/202	05/08/202	2	1
03/07/202	06/07/202	16/06/202	2	1
10/08/202	06/08/202	03/08/202	2	
02/08/202	02/08/202	03/08/202	2	1
22/08/202	20/08/202	20/08/202	9	1
08/12/202	14/12/202	14/12/202	0	1
05/12/202	18/12/202	18/12/202	2	1
31/05/202	01/06/202	26/05/202	2	1
17/05/202	26/06/202	05/07/202	0	1
19/06/202	19/06/202	25/08/202	2	
27/09/202	28/09/202	14/09/202	2	1
21/10/202	30/10/202	19/10/202	2	1
25/05/202	28/05/202	12/05/202	0	1
20/01/202	20/01/202	06/07/202	0	1
05/07/202	22/02/202	07/07/202	2	1
	14/07/202	29/06/202	2	1
16/07/202	06/07/202	06/07/202	9	1
02/08/202	08/08/202	29/07/202	2	1
17/08/202	17/08/202	30/07/202	0	
28/12/202	19/02/202	21/09/202	2	1
10/08/202	13/08/202	03/08/202	9	1
05/04/202	06/04/202	06/04/202	2	1
02/05/202	28/09/202	13/05/202	2	1
22/04/202	22/04/202	15/04/202	2	1
25/07/202	03/08/202	21/07/202	2	1
25/06/202	21/08/202	19/06/202	0	1
23/07/202	20/11/202	08/07/202	2	1
03/11/202	05/11/202	01/11/202	2	
16/12/202	21/12/202	21/12/202	2	1
16/12/202	01/06/202	11/12/202	0	
11/01/202	11/01/202	23/12/202	2	
15/05/202	16/05/202	30/04/202	9	1
04/05/202	21/05/202	28/04/202	0	1
17/07/202	17/07/202	06/07/202	2	1
27/08/202	17/09/202	07/08/202	2	
09/08/202	13/08/202	01/08/202	2	1
22/11/202	24/11/202	24/11/202	0	
16/11/202	27/11/202	11/11/202	0	1

08/10/202	09/10/202	04/10/202	2	
10/01/202	14/01/202	28/12/202	2	1
16/06/202	19/09/202	22/04/202	2	1
07/05/202	11/05/202	20/04/202	2	1
31/05/202	10/06/202	22/05/202	9	1
27/06/202	01/07/202	22/06/202	2	1
10/07/202	27/06/202	26/06/202	2	1
22/08/202	24/08/202	24/08/202	9	1
06/07/202	03/09/202	03/09/202	2	
13/04/202	13/04/202	08/04/202	2	1
24/05/202	31/05/202	18/05/202	2	1
16/02/202	04/09/202	28/12/202	0	1
29/05/202	03/06/202	01/05/202	2	1
08/07/202	09/07/202	02/07/202	2	
25/04/202	29/04/202	22/04/202	2	1
13/06/202	15/06/202	15/06/202	0	1
01/07/202	08/07/202	08/07/202	0	
06/10/202	28/04/202	08/09/202	0	1
03/08/202	05/08/202	02/08/202	2	1
01/11/202	12/11/202	22/10/202	2	1
28/07/202	13/07/202	13/07/202	0	1
	31/07/202	09/07/202	0	1
11/09/202	16/09/202	14/09/202	9	1
19/09/202	22/09/202	10/09/202	9	
05/05/202	22/05/202	03/05/202	0	1
04/06/202	04/08/202	21/05/202	2	1
05/07/202	20/07/202	20/06/202	2	1
31/07/202	17/07/202	16/07/202	0	1
31/10/202	21/10/202	21/10/202	0	1
09/12/202	14/12/202	08/12/202	0	1
25/01/202	18/05/202	27/01/202	0	
15/05/202	06/10/202	01/06/202	2	
24/06/202	01/07/202	22/06/202	2	1
21/12/202	30/12/202	10/12/202	2	1
28/07/202	31/07/202	31/07/202	2	
28/08/202	09/06/202	03/09/202	0	
13/07/202	17/07/202	27/07/202	2	1
28/10/202	28/10/202	28/10/202	0	1
20/05/202	20/05/202	22/07/202	9	
08/09/202	09/09/202	09/09/202	2	1
18/12/202	22/12/202	10/12/202	0	1
11/01/202	12/01/202	17/12/202	2	1
01/04/202	24/04/202	07/04/202	2	1
03/07/202	03/07/202	26/06/202	0	1
24/07/202	25/08/202	24/07/202	2	1
30/09/202	16/10/202	21/09/202	2	1
15/12/202	21/12/202	15/12/202	0	

16/11/202	26/11/202	12/11/202	2	
08/07/202	08/07/202	29/06/202	0	1
07/12/202	08/12/202	02/12/202	2	
27/10/202	29/10/202	29/10/202	0	1
30/11/202	18/02/202	26/11/202	2	1
24/06/202	05/07/202	29/06/202	0	
17/05/202	18/05/202	06/05/202	2	1
09/05/202	28/05/202	06/05/202	0	1
22/07/202	23/07/202	01/07/202	2	1
04/09/202	11/09/202	11/09/202	0	
16/05/202	16/06/202	10/06/202	0	1
16/05/202	19/06/202	25/05/202	2	1
10/03/202	11/03/202	13/01/202	2	1
	08/02/202	08/02/202	2	1
08/06/202	09/06/202	02/06/202	2	
08/06/202	13/06/202	02/06/202	2	1
22/07/202	25/06/202	20/07/202	0	1
28/12/202	10/07/202	17/12/202	0	1
	31/07/202	02/07/202	2	1
25/05/202	18/06/202	19/05/202	2	1
12/10/202	19/11/202	09/10/202	2	
08/12/202	30/11/202	28/11/202	2	1
03/05/202	06/05/202	30/04/202	2	1
03/06/202	29/05/202	26/05/202	2	
25/05/202	15/09/202	07/05/202	2	1
30/07/202	24/07/202	24/07/202	0	1
13/07/202	20/07/202	20/07/202	2	1
06/09/202	13/10/202	31/08/202	0	1
10/10/202	30/09/202	30/09/202	0	1
06/10/202	06/10/202	07/10/202	2	
11/01/202	18/01/202	18/01/202	2	1
08/12/202	15/01/202	01/12/202	0	
13/01/202	14/01/202	28/12/202	2	1
24/01/202	26/01/202	11/01/202	9	1
10/07/202	18/07/202	17/06/202	2	1
19/12/202	05/01/202	15/12/202	2	1
10/05/202	11/05/202	09/05/202	9	1
15/05/202	13/07/202	17/05/202	2	1
21/06/202	22/06/202	12/06/202	2	1
28/06/202	26/11/202	22/06/202	2	1
14/06/202	15/06/202	22/06/202	2	1
29/08/202	31/08/202	31/08/202	9	1
10/05/202	12/05/202	27/04/202	2	1
30/05/202	04/06/202	21/05/202	2	1
15/05/202	19/05/202	27/04/202	0	1
23/04/202	27/04/202	29/04/202	2	1
08/06/202	16/06/202	11/06/202	2	1

30/06/202	01/07/202	02/07/202	0	1
25/07/202	25/07/202	22/07/202	0	
24/08/202	24/08/202	09/08/202	2	1
20/09/202	23/09/202	17/09/202	0	1
21/09/202	21/09/202	18/09/202	2	1
22/09/202	25/09/202	24/09/202	2	
25/05/202	29/06/202	18/05/202	2	1
23/12/202	30/12/202	16/12/202	2	1
15/07/202	16/07/202	08/06/202	2	
21/05/202	03/06/202	04/05/202	2	1
02/07/202	05/07/202	29/06/202	2	1
13/08/202	21/08/202	11/08/202	2	1
17/01/202	25/03/202	31/12/202	0	1
16/06/202	26/06/202	26/06/202	0	1
07/05/202	01/07/202	30/04/202	9	1
17/07/202	20/07/202	15/07/202	2	1
10/08/202	12/08/202	31/07/202	2	1
11/12/202	14/12/202	07/12/202	2	1
27/07/202	30/07/202	17/06/202	2	1
23/07/202	28/07/202	03/08/202	9	1
22/08/202	30/08/202	30/08/202	0	
04/11/202	17/11/202	28/10/202	0	
21/11/202	03/02/202	08/01/202	2	1
22/11/202	22/11/202	24/11/202	0	1
13/06/202	23/06/202	11/06/202	2	
20/07/202	20/07/202	20/07/202	0	
27/08/202	23/10/202	24/08/202	2	1
14/08/202	18/08/202	29/07/202	0	1
25/10/202	26/10/202	26/10/202	2	1
21/06/202	24/06/202	29/05/202	0	1
02/06/202	17/06/202	29/05/202	0	1
12/07/202	13/07/202	06/07/202	2	
08/01/202	08/01/202	25/01/202	2	1
25/07/202	17/08/202	26/07/202	2	1
26/09/202	29/10/202	09/09/202	2	1
15/05/202	21/07/202	15/05/202	0	1
17/08/202	10/09/202	10/08/202	0	1
06/08/202	06/08/202	03/09/202	9	
16/06/202	05/03/202	02/07/202	2	1
03/12/202	01/12/202	01/12/202	0	1
08/05/202	13/05/202	20/04/202	0	1
26/08/202	23/12/202	17/07/202	0	1
10/04/202	16/05/202	14/04/202	0	1
22/04/202	29/05/202	17/04/202	2	1
01/08/202	05/08/202	16/07/202	2	1
20/01/202	18/02/202	11/01/202	0	1
04/08/202	04/08/202	13/07/202	2	1

29/06/202	29/06/202	24/06/202	0	1
20/01/202	20/01/202	29/12/202	0	1
12/01/202	13/01/202	13/01/202	2	
13/01/202	05/02/202	04/01/202	0	1
29/04/202	06/05/202	27/04/202	0	1
07/06/202	24/06/202	18/06/202	2	1
29/06/202	21/07/202	16/06/202	9	1
25/07/202	23/07/202	21/07/202	0	1
04/09/202	18/12/202	28/08/202	2	
27/12/202	28/12/202	21/12/202	2	1
06/01/202	05/02/202	22/12/202	0	1
05/10/202	04/09/202	04/09/202	0	
06/01/202	23/12/202	23/12/202	2	
14/05/202	01/06/202	03/05/202	2	1
03/12/202	20/04/202	20/04/202	0	1
21/07/202	22/07/202	06/07/202	2	1
23/07/202	11/08/202	14/07/202	0	1
02/06/202	08/06/202	08/06/202	2	1
13/11/202	13/11/202	17/10/202	0	1
26/04/202	30/04/202	20/07/202	0	1
27/07/202	28/07/202	27/07/202	0	1
08/12/202	10/12/202	10/12/202	0	
18/01/202	09/02/202	13/01/202	2	
09/10/202	17/02/202	02/10/202	0	1
25/05/202	25/05/202	12/05/202	9	1
28/12/202	13/05/202	02/12/202	0	1
20/07/202	20/07/202	15/07/202	2	1
26/07/202	26/07/202	25/08/202	2	
23/12/202	28/12/202	06/01/202	2	1
22/01/202	05/09/202	05/09/202	0	
20/05/202	29/07/202	15/05/202	2	1
03/05/202	05/06/202	05/06/202	2	1
28/06/202	23/07/202	19/06/202	0	
04/06/202	04/06/202	23/06/202	9	
23/01/202	24/01/202	21/01/202	0	
16/06/202	16/06/202	17/06/202	2	1
19/09/202	22/10/202	18/09/202	0	1
14/12/202	14/12/202	20/11/202	2	1
22/05/202	23/05/202	15/05/202	2	1
07/05/202	12/05/202	06/05/202	0	1
14/06/202	20/06/202	26/05/202	2	1
04/08/202	05/08/202	03/08/202	2	1
21/08/202	15/09/202	27/08/202	0	1
29/06/202	10/07/202	21/07/202	2	1
14/01/202	15/01/202	15/01/202	0	1
06/01/202	06/01/202	05/01/202	2	1
07/04/202	13/04/202	01/04/202	2	1

25/05/2020	02/06/2020	24/05/2020	2	1
11/06/2020	09/07/2020	03/06/2020	2	1
30/06/2020	29/06/2020	30/06/2020	2	1
06/07/2020	06/07/2020	28/07/2020	0	1
24/05/2020	28/09/2020	08/10/2020	9	
	21/09/2020	21/09/2020	0	
14/09/2020	16/09/2020	17/03/202	0	
30/03/2020	30/03/2020	25/03/2020	2	1
16/01/202	16/01/202	09/01/202	0	1
26/03/2020	03/04/2020	30/03/2020	2	1
03/06/2020	10/06/2020	10/06/2020	9	1
12/01/202	13/01/202	26/12/2020	0	1
16/12/2020	18/12/2020	04/12/2020	0	
07/10/2020	07/10/2020	06/07/2020	0	1
18/12/2020	28/12/2020	15/12/2020	2	
05/08/2020	07/08/2020	30/07/2020	2	1
17/10/2020		22/10/2020	0	1
09/01/202	25/01/202	28/01/202	9	
01/08/2020	02/09/2020	10/07/2020	0	1
29/07/2020	30/07/2020	21/07/2020	2	1
28/08/2020	21/07/2020	21/07/2020	0	1
20/11/2020	24/11/2020	13/11/2020	2	1
22/07/2020	25/08/2020	21/07/2020	9	1
14/01/202	18/01/202	22/12/2020	0	
31/12/2020	04/01/202	30/12/2020	2	
18/05/2020	02/06/2020	12/05/2020	2	1
23/10/2020	23/10/2020	22/10/2020	2	1
22/06/2020	01/07/202	22/06/2020	0	1
02/08/2020	02/08/2020	22/06/2020	2	1
07/11/2020	03/11/2020	03/11/2020	9	1
11/12/2020	12/12/2020	19/11/2020	2	1
27/07/2020	01/09/2020	12/07/2020	2	1
12/04/2020	05/05/2020	09/04/2020	2	1
16/05/2020	16/05/2020	30/04/2020	9	1
14/01/202	16/01/202	01/03/202	0	
12/10/2020	14/06/202	14/10/2020	0	
04/05/2020	18/06/2020	11/05/2020	2	
04/04/2020	04/04/2020	26/03/2020	0	1
29/06/2020	30/06/2020	02/06/2020	9	1
07/07/2020	08/07/2020	19/06/2020	0	
01/07/2020	01/07/2020	26/06/2020	9	1
16/07/2020	17/07/2020	09/06/2020	0	1
12/07/2020	22/07/2020	20/07/2020	0	1
23/07/2020	21/07/2020	21/07/2020	9	1
21/07/2020	21/07/2020	13/07/2020	0	1
14/06/2020	19/06/2020	22/07/2020	2	1
24/09/2020	28/09/2020	21/08/2020	2	1

20/08/202	25/09/202	20/08/202	2	1
15/11/202	16/11/202	16/11/202	2	1
05/11/202	12/11/202	26/10/202	2	
15/09/202	21/10/202	08/09/202	0	1
17/04/202	05/05/202	17/04/202	0	
01/05/202	25/10/202	04/09/202	0	1
10/06/202	28/07/202	21/06/202	2	1
04/06/202	19/08/202	12/06/202	2	1
22/09/202	15/10/202	19/09/202	9	1
15/11/202	15/11/202	02/12/202	9	1
17/07/202	12/12/202	12/12/202	2	
21/04/202	22/04/202	03/04/202	2	1
28/07/202	29/07/202	07/07/202	2	1
27/05/202	19/06/202	17/06/202	0	
11/07/202	11/07/202	27/07/202	9	
09/08/202	11/08/202	03/08/202	2	1
17/08/202	15/09/202	11/08/202	2	1
14/05/202	19/05/202	07/05/202	0	1
06/01/202	24/02/202	04/01/202	0	
11/11/2020		15/02/202	0	1
05/06/202	06/06/202	26/05/202	9	1
16/05/202	19/02/202	08/05/202	2	1
24/06/202	25/06/202	05/06/202	2	1
09/06/202	12/06/202	02/06/202	2	1
12/09/202	14/09/202	21/08/202	2	
27/05/202	10/07/202	31/05/202	2	1
31/12/202	04/01/202	31/12/202	0	1
24/04/202	27/04/202	16/04/202	0	1
28/06/202	01/07/202	25/06/202	0	1
10/07/202	22/06/202	18/06/202	9	1
29/09/202	30/09/202	18/08/202	2	1
25/09/202	18/09/202	18/09/202	0	1
09/09/202	06/08/202	04/08/202	2	1
10/08/202	22/06/202	13/08/202	0	
26/08/202	10/09/202	16/08/202	2	1
23/12/202	30/12/202	09/12/202	2	1
13/10/202	09/11/202	01/12/202	2	
14/01/202	24/02/202	06/01/202	0	
04/05/202	06/05/202	30/04/202	2	1
02/11/202	21/12/202	21/12/202	0	
20/05/202	20/05/202	24/07/202	9	
25/07/202	25/07/202	07/08/202	2	1
24/08/202	25/08/202	06/08/202	0	1
20/05/202	10/06/202	20/05/202	2	1
06/07/202	09/07/202	16/06/202	2	
19/12/202	07/01/202	16/12/202	2	1
26/08/202	12/08/202	12/08/202	9	

23/07/202	04/08/202	19/07/202	2	
02/10/202	02/10/202	28/09/202	2	
16/07/202	28/07/202	28/07/202	0	
01/01/202	02/01/202	02/01/202	0	1
21/06/202	24/06/202	06/06/202	0	1
28/05/202	18/06/202	23/06/202	2	1
08/11/202	08/11/202	01/06/202	0	1
18/09/202	13/10/202	15/09/202	2	1
18/12/202	18/12/202	12/12/202	2	1
11/01/202	11/01/202	09/12/202	2	1
12/09/202	15/09/202	09/09/202	2	1
	14/09/202	02/09/202	2	
03/10/202	09/10/202	27/08/202	0	
19/09/202	25/09/202	25/09/202	2	1
03/12/202	04/12/202	03/11/202	2	1
23/05/202	09/05/202	06/05/202	2	1
14/06/202	19/07/202	19/07/202	9	1
02/09/202	09/09/202	10/09/202	2	
19/04/202	23/04/202	14/08/202	2	1
05/08/202	04/08/202	28/07/202	2	
06/12/202	16/12/202	06/12/202	0	1
04/09/202	04/09/202	28/08/202	9	1
05/11/202	11/11/202	11/11/202	9	1
20/08/202	20/08/202	31/07/202	2	1
02/08/202	02/08/202	31/07/202	0	1
01/08/202	04/08/202	27/07/202	2	
25/08/202	26/08/202	24/08/202	0	1
06/01/202	08/01/202	29/12/202	0	1
19/01/202	23/02/202	08/02/202	2	1
12/06/202	15/06/202	09/06/202	2	1
01/05/202	22/06/202	22/06/202	2	1
12/10/202	14/10/202	05/10/202	2	1
04/09/202	17/12/202	17/12/202	9	
07/12/202	08/12/202	23/11/202	2	1
02/08/202	12/03/202	16/03/202	2	
12/06/202	01/07/202	27/05/202	0	1
02/07/202	26/06/202	23/06/202	9	1
26/06/202	30/07/202	01/07/202	0	
05/08/202	10/09/202	19/07/202	2	1
08/10/202	26/11/202	21/09/202	2	1
05/09/202	08/09/202	28/08/202	9	
13/01/202	28/01/202	13/01/202	2	1
09/07/202	09/04/202	12/04/202	0	1
28/08/202	04/09/202	27/08/202	2	1
29/07/202	03/08/202	13/08/202	2	1
09/09/202	10/09/202	03/09/202	2	1
01/10/202	09/10/202	09/10/202	9	1

13/05/202	13/05/202	21/07/202	9	
04/08/202	11/08/202	29/07/202	2	1
09/09/202	23/09/202	31/07/202	0	
07/08/202	08/08/202	13/07/202	0	
21/05/202	21/05/202	27/04/202	9	1
03/07/202	07/07/202	25/06/202	9	1
11/07/202	12/07/202	19/07/202	0	
16/10/202	21/10/202	02/10/202	2	1
23/09/202	24/09/202	24/09/202	2	1
24/11/202	18/12/202	11/11/202	0	1
30/10/202	21/10/202	20/10/202	9	
13/11/202	13/11/202	03/05/202	0	
24/06/202	15/06/202	10/06/202	9	1
12/05/202	22/05/202	22/05/202	2	1
18/06/202	10/09/202	16/09/202	2	
14/10/202	09/03/202	19/10/202	0	1
18/09/202	07/10/202	07/10/202	2	
05/01/202	07/01/202	03/01/202	9	1
18/12/202	18/12/202	10/12/202	0	
12/07/202	03/09/202	07/08/202	2	1
29/04/202	01/07/202	08/06/202	0	1
04/06/202	10/06/202	22/05/202	2	1
	18/06/202	12/06/202	2	
08/07/202	07/08/202	20/07/202	0	1
26/07/202	27/07/202	21/07/202	2	1
23/10/202	28/10/202	15/10/202	2	
10/11/202	20/01/202	04/11/202	9	1
28/06/202	30/06/202	15/06/202	2	1
28/12/2020		16/12/202	9	
02/06/202	02/06/202	21/05/202	2	1
25/04/202	24/06/202	20/04/202	2	1
26/05/202	09/06/202	09/06/202	0	1
13/10/202	15/10/202	09/10/202	0	
13/10/202	05/10/202	30/09/202	9	1
16/07/202	28/09/202	14/07/202	2	1
03/06/202	06/06/202	10/06/202	2	1
04/08/202	05/08/202	23/07/202	2	1
16/05/202	05/06/202	19/05/202	2	1
17/11/202	18/11/202	16/11/202	2	1
09/07/202	09/07/202	01/07/202	2	1
23/12/202	24/12/202	21/12/202	2	1
08/05/202	09/05/202	03/06/202	2	1
09/06/202	09/06/202	02/06/202	2	1
19/07/202	20/07/202	14/07/202	2	1
04/08/202	07/08/202	14/07/202	2	1
21/07/202	23/07/202	10/07/202	9	1
	25/07/202	21/07/202	0	

12/06/202	12/06/202	21/07/202	2	1
03/08/202	04/08/202	04/08/202	2	1
15/07/202	29/07/202	29/07/202	0	1
10/09/202	02/09/202	02/09/202	9	1
27/10/202	27/10/202	24/09/202	2	
13/08/202	01/10/202	25/11/202	0	1
08/04/202	29/04/202	29/04/202	2	1
03/05/202	13/05/202	28/04/202	0	1
29/07/202	25/07/202	25/07/202	0	1
24/05/202	10/07/202	20/05/202	2	1
03/06/202	04/06/202	22/05/202	9	1
04/08/202	05/08/202	17/07/202	2	1
	10/12/202	26/11/202	2	
09/09/202	25/10/202	04/09/202	0	1
08/05/202	08/06/202	08/06/202	2	1
30/04/202	29/04/202	28/04/202	9	
13/12/202	14/12/202	04/12/202	2	1
01/08/202	03/08/202	05/08/202	2	1
17/11/202	20/11/202	03/11/202	2	
26/05/202	29/06/202	18/05/202	9	1
10/06/202	11/06/202	11/06/202	2	1
06/07/202	06/07/202	29/06/202	0	1
23/08/202	31/08/202	27/08/202	0	1
21/12/202	21/12/202	08/02/202	0	1
14/10/202	14/10/202	06/04/202	0	
18/05/202	11/05/202	08/05/202	2	1
22/07/202	23/07/202	04/06/202	0	1
10/07/202	20/07/202	15/06/202	0	1
20/04/202	20/04/202	20/07/202	0	1
22/09/202	23/09/202	15/09/202	2	1
19/11/202	24/11/202	18/11/202	2	1
01/12/202	01/12/202	24/11/202	2	1
15/05/202	08/09/202	08/05/202	0	
23/06/202	22/06/202	19/06/202	2	1
11/07/202	28/10/202	28/10/202	0	
02/07/202	06/07/202	06/07/202	9	1
11/07/202	13/07/202	05/06/202	2	1
04/08/202	04/08/202	20/07/202	0	1
11/06/202	18/09/202	18/09/202	0	1
11/05/202	07/07/202	07/05/202	9	1
23/04/202	23/04/202	18/11/202	0	
05/01/202	05/09/202	05/09/202	0	1
26/12/202	29/12/202	29/12/202	2	1
02/05/202	05/05/202	29/04/202	2	1
14/05/202	14/05/202	29/04/202	2	1
26/06/202	06/07/202	24/06/202	9	
16/06/202	16/06/202	08/06/202	2	1

01/07/202	09/07/202	01/07/202	2	1
12/12/202	30/12/202	30/11/202	2	1
17/06/202	18/06/202	13/04/202	0	1
30/09/202	02/09/202	20/10/202	0	1
06/04/202	15/04/202	07/04/202	0	
24/11/202	10/12/202	21/10/202	0	1
14/10/202	21/10/202	14/10/202	2	1
09/11/202	11/11/202	11/11/202	2	1
06/08/202	06/08/202	24/09/202	9	
07/05/202	29/06/202	13/05/202	0	1
	19/08/202	22/07/202	2	1
13/05/202	13/05/202	21/07/202	9	
28/06/202	28/06/202	19/06/202	0	1
11/05/202	19/05/202	11/05/202	0	1
07/07/202	06/07/202	06/07/202	9	1
27/07/202	03/08/202	20/06/202	0	1
29/05/202	11/06/202	04/06/202	2	1
24/12/202	28/12/202	23/12/202	2	
28/11/202	01/12/202	26/11/202	0	
14/05/202	16/06/202	16/06/202	2	1
08/08/202	08/08/202	24/07/202	2	1
31/08/202	20/10/202	31/08/202	2	
18/07/202	18/08/202	22/07/202	2	1
14/08/202	20/08/202	18/08/202	2	1
12/01/202	20/01/202	20/01/202	0	1
13/01/202	13/01/202	28/12/202	0	1
10/01/202	12/01/202	11/01/202	2	
12/06/202	20/08/202	08/06/202	2	1
06/01/202	23/08/202	23/08/202	0	
04/10/202	04/10/202	29/08/202	0	1
11/01/202	14/01/202	04/01/202	0	1
14/05/202	24/06/202	11/05/202	9	1
06/05/202	17/09/202	23/04/202	2	1
02/06/202	18/08/202	16/05/202	2	
25/05/202	28/05/202	08/05/202	2	1
23/05/202	04/06/202	18/05/202	2	1
27/07/202	03/08/202	09/06/202	2	1
11/06/202	06/10/202	16/06/202	0	1
12/01/202	12/01/202	06/07/202	0	1
14/07/202	17/07/202	17/07/202	2	
01/12/202	18/01/202	12/08/202	9	
03/08/202	03/08/202	12/08/202	2	
19/09/202	10/09/202	11/09/202	2	1
03/01/202	05/01/202	15/12/202	2	1
04/11/202	19/11/202	02/11/202	2	1
24/06/202	17/09/202	17/09/202	9	1
19/09/202	21/09/202	07/09/202	2	

29/05/202	02/06/202	05/05/202	2	1
01/07/202	01/07/202	24/06/202	0	1
26/06/202	07/07/202	07/07/202	2	
20/07/202	24/07/202	20/07/202	2	1
06/11/202	18/02/202	26/10/202	2	1
04/09/202	04/09/202	09/09/202	2	
01/08/202	06/08/202	30/07/202	2	1
10/05/202	27/08/202	29/08/202	2	1
24/11/202	29/06/202	28/08/202	0	1
24/12/202	07/01/202	11/12/202	2	1
15/10/202	20/10/202	20/10/202	2	
14/06/202	19/06/202	15/06/202	2	1
28/10/202	20/11/202	05/11/202	9	1
20/01/202	02/09/202	14/01/202	0	1
30/06/202	01/07/202	09/06/202	0	1
16/09/202	22/09/202	04/09/202	9	1
29/08/202	30/09/202	24/08/202	0	1
02/03/202	03/03/202	15/12/202	0	1
05/02/202	11/02/202	06/01/202	2	1
17/06/202	22/06/202	09/06/202	2	1
24/07/202	27/07/202	19/07/202	0	1
06/08/202	01/09/202	05/08/202	2	
25/09/202	25/09/202	27/07/202	2	1
27/08/202	31/08/202	24/08/202	2	1
21/05/202	02/06/202	15/05/202	2	1
05/05/202	28/05/202	27/04/202	9	1
14/06/202	16/06/202	10/06/202	2	1
10/06/202	30/07/202	14/06/202	2	1
06/08/202	18/08/202	12/07/202	0	1
28/08/202	24/08/202	21/08/202	2	1
20/12/202	29/12/202	16/12/202	2	1
20/01/202	22/01/202	15/01/202	0	1
16/06/202	22/06/202	02/06/202	2	1
25/07/202	15/07/202	15/07/202	0	1
16/12/202	01/02/202	01/02/202	9	1
19/10/202	28/10/202	27/10/202	2	
08/05/202	08/05/202	27/04/202	0	1
12/07/202	12/07/202	23/08/202	0	
09/06/202	10/06/202	29/04/202	2	1
08/07/202	09/07/202	15/06/202	2	1
26/08/202	26/08/202	24/08/202	2	
29/10/202	16/12/202	21/10/202	2	1
10/10/202	13/10/202	08/10/202	0	1
24/12/202	24/12/202	24/12/202	2	
11/10/202	13/10/202	05/10/202	2	1
31/01/202	01/02/202	07/01/202	0	1
30/11/202	11/05/202	08/04/202	0	1

09/06/202	01/07/202	19/05/202	2	1
15/07/202	10/08/202	13/07/202	0	1
24/12/202	13/04/202	13/04/202	0	1
28/05/202	28/05/202	04/06/202	2	1
30/09/202	03/11/202	27/07/202	9	1
29/07/202	18/08/202	31/07/202	0	
10/10/202	15/10/202	15/10/202	2	1
23/03/202	14/10/202	30/11/202	0	1
	28/08/202	03/09/202	0	
26/05/202	06/06/202	29/05/202	0	1
25/10/202	11/02/202	20/10/202	2	
09/05/202	12/05/202	01/05/202	2	
23/06/202	19/06/202	12/06/202	0	1
02/07/202	03/07/202	03/07/202	2	1
05/01/202	13/01/202	04/01/202	2	
17/11/202	21/01/202	08/10/202	2	1
08/09/202	23/09/202	05/09/202	0	
27/06/202	27/06/202	30/06/202	2	1
17/06/202	18/06/202	10/06/202	2	
09/01/202	09/01/202	04/01/202	2	1
19/09/202	02/10/202	26/08/202	2	1
05/07/202	31/07/202	06/08/202	2	1
02/06/202	18/06/202	24/05/202	2	1
26/04/202	28/04/202	23/04/202	9	1
26/08/202	26/08/202	25/08/202	2	1
29/07/202	03/08/202	24/07/202	2	1
28/04/202	13/05/202	28/04/202	0	1
01/08/202	05/08/202	28/07/202	2	1
29/06/202	27/08/202	06/07/202	0	1
20/05/202	01/06/202	11/05/202	2	1
31/07/202	05/08/202	03/08/202	2	1
01/07/202	31/08/202	08/06/202	2	1
05/11/202	05/11/202	12/11/202	2	1
04/06/202	17/06/202	05/06/202	2	1
29/11/202	29/11/202	13/02/202	2	1
16/01/202	04/02/202	04/02/202	0	1
13/07/202	15/07/202	05/07/202	2	1
25/07/202	25/07/202	29/07/202	2	1
29/05/202	29/05/202	29/07/202	9	
09/01/202	11/01/202	11/01/202	0	1
18/04/202	23/04/202	17/04/202	2	1
23/04/202	30/04/202	10/04/202	0	1
01/02/202	12/02/202	23/12/202	0	1
27/10/202	12/10/202	12/10/202	2	1
01/05/202	13/05/202	28/04/202	2	1
13/12/202	19/12/202	18/12/202	0	1
31/12/202	04/01/202	28/12/202	2	1

	15/06/202	26/05/202	2	
10/09/202	12/09/202	16/08/202	2	1
15/06/202	17/06/202	26/05/202	2	1
21/07/202	20/11/202	20/07/202	2	1
11/08/202	05/11/202	03/08/202	0	1
07/12/202	11/12/202	25/11/202	9	1
25/08/202	01/10/202	01/10/202	2	
25/01/202	25/01/202	27/01/202	0	
23/11/202	29/11/202	18/11/202	2	
01/05/202	28/08/202	18/05/202	9	1
28/05/202	28/05/202	20/05/202	2	1
13/04/202	18/02/202	30/03/202	2	1
16/07/202	16/07/202	22/07/202	9	
20/05/202	27/05/202	27/05/202	2	1
14/06/202	23/06/202	04/06/202	0	
02/10/202	05/10/202	26/09/202	2	1
03/07/202	07/07/202	23/05/202	2	1
28/03/202	28/05/202	28/05/202	2	1
26/07/202	27/07/202	21/07/202	2	1
18/06/202	19/06/202	08/06/202	2	
25/12/202	13/04/202	13/04/202	0	
10/07/202	24/09/202	06/07/202	2	
21/11/202	26/05/202	26/05/202	0	1
24/07/202	24/07/202	31/07/202	0	
03/12/202	08/12/202	30/11/202	0	
30/12/202	07/02/202	07/02/202	2	1
14/08/202	11/06/202	12/08/202	0	
25/12/202	28/12/202	16/12/202	2	1
07/05/202	21/06/202	04/05/202	2	1
03/07/202	06/11/202	21/11/202	2	
05/09/202	09/09/202	09/09/202	2	
20/11/202	23/11/202	11/11/202	0	1
16/09/202	04/09/202	04/09/202	0	1
23/07/202	28/07/202	24/07/202	0	1
	27/05/202	06/08/202	9	
07/01/202	08/01/202	28/12/202	2	1
29/05/202	31/07/202	04/08/202	9	
10/05/202	18/05/202	20/05/202	2	1
07/12/202	15/12/202	25/11/202	0	
29/05/202	11/09/202	02/06/202	0	1
05/05/202	17/09/202	26/04/202	2	1
14/09/202	15/09/202	28/08/202	2	1
20/12/202	30/12/202	09/12/202	2	1
08/01/202	12/01/202	30/12/202	0	
17/07/202	18/07/202	14/07/202	2	
03/07/2020		29/06/202	0	1
05/05/202	02/06/202	04/05/202	2	1

31/10/202	01/11/202	14/10/202	2	1
25/11/202	07/12/202	24/11/202	0	
13/06/202	13/06/202	30/05/202	0	1
31/12/202	31/12/202	30/04/202	0	1
19/05/202	17/06/202	25/05/202	2	1
28/12/202	31/12/202	31/12/202	2	
11/08/202	28/09/202	28/09/202	0	
29/07/202	31/07/202	16/07/202	9	1
13/11/202	20/11/202	11/11/202	2	1
02/01/202	07/01/202	07/01/202	2	1
12/07/202	12/07/202	02/07/202	0	1
30/11/202	23/12/202	01/12/202	2	1
13/06/202	13/06/202	13/06/202	2	1
30/05/202	24/08/202	18/05/202	0	1
25/08/202	14/09/202	16/09/202	9	1
25/11/202	30/11/202	17/11/202	9	1
29/05/202	13/07/202	13/07/202	2	
28/04/202	04/05/202	24/04/202	2	1
05/05/202	01/06/202	02/05/202	2	1
22/12/202	22/12/202	20/01/202	0	1
07/05/202	31/05/202	31/08/202	0	1
10/07/202	06/08/202	03/07/202	2	1
08/05/202	26/10/202	02/06/202	9	1
18/07/202	11/03/202	16/07/202	0	1
13/01/202	18/01/202	11/01/202	2	1
18/09/202	18/09/202	09/10/202	9	
11/10/202	21/10/202	07/10/202	9	1
15/12/202	19/01/202	07/12/202	0	1
30/06/202	30/06/202	06/06/202	0	1
12/08/202	19/08/202	13/08/202	2	1
20/10/202	12/10/202	09/10/202	0	1
29/11/202	30/11/202	19/11/202	2	
16/07/202	16/07/202	03/05/202	0	1
	19/10/202	03/06/202	0	1
16/07/202	16/07/202	06/07/202	2	1
26/08/202	27/08/202	21/08/202	2	1
10/01/202	11/01/202	05/01/202	9	1
30/09/202	01/10/202	16/09/202	2	1
12/11/202	13/11/202	11/11/202	2	1
14/01/202	15/01/202	04/01/202	2	
17/06/202	17/06/202	27/01/202	2	
23/06/202	14/07/202	14/07/202	2	1
24/06/202	15/06/202	08/06/202	9	1
27/11/202	27/11/202	23/11/202	2	
25/01/202	25/01/202	27/01/202	0	
18/08/202	03/09/202	10/08/202	2	
20/05/202	23/07/202	21/05/202	2	1

16/07/202	17/07/202	16/07/202	2	1
18/07/202	21/07/202	29/06/202	2	1
05/06/202	05/06/202	19/08/202	9	
22/05/202	22/07/202	21/05/202	2	1
27/07/202	31/07/202	22/07/202	2	1
09/10/202	23/10/202	28/08/202	2	1
16/12/202	10/12/202	10/12/202	0	1
02/10/202	06/10/202	14/09/202	2	1
15/12/202	16/12/202	25/11/202	2	1
14/01/202	14/01/202	14/01/202	2	1
22/01/202	13/01/202	13/01/202	2	1
08/05/202	20/05/202	09/05/202	0	1
13/06/202	17/06/202	17/06/202	0	1
30/07/202	31/07/202	27/07/202	2	1
06/07/202	13/07/202	16/09/202	2	1
12/08/202	12/08/202	12/08/202	2	1
01/10/202	01/10/202	21/08/202	0	
15/09/202	15/09/202	14/09/202	2	
29/12/202	04/01/202	24/12/202	2	
21/08/202	02/09/202	20/08/202	0	
11/05/202	08/09/202	09/05/202	2	
26/12/202	06/01/202	23/12/202	0	
12/07/202	13/07/202	06/07/202	2	1
07/12/202	07/12/202	30/07/202	0	1
28/07/202	29/07/202	21/07/202	2	1
11/08/202	13/08/202	29/07/202	0	1
26/08/202	06/10/202	10/08/202	0	1
25/05/202	28/05/202	28/05/202	2	1
17/07/202	21/08/202	15/07/202	2	1
17/09/202	08/10/202	08/10/202	0	
26/12/202	28/12/202	03/12/202	2	1
21/04/202	21/04/202	23/04/202	2	1
02/04/202	05/04/202	03/04/202	2	
14/06/202	19/02/202	12/06/202	2	1
19/04/202	20/08/202	29/04/202	2	1
07/01/202	17/11/202	16/11/202	2	1
17/08/202	20/08/202	20/08/202	9	1
27/12/202	04/01/202	22/12/202	0	1
07/05/202	08/05/202	30/04/202	0	1
21/07/202	28/08/202	12/07/202	9	1
22/07/202	23/07/202	23/07/202	0	
28/08/202	31/08/202	21/08/202	2	
04/07/202	06/07/202	24/06/202	2	1
18/06/202	18/07/202	02/08/202	0	1
12/05/202	01/06/202	22/04/202	2	1
27/12/202	28/12/202	15/12/202	2	1
20/01/202	22/01/202	05/01/202	2	1

30/06/202	30/06/202	18/06/202	2	
06/07/202	10/07/202	29/06/202	0	1
24/12/202	05/03/202	22/12/202	0	1
07/01/202	07/01/202	07/01/202	2	1
07/07/202	07/07/202	28/07/202	0	
21/07/202	24/07/202	22/07/202	2	1
02/09/202	02/09/202	26/08/202	9	1
13/04/202	04/09/202	07/04/202	2	1
21/08/202	31/08/202	27/07/202	0	1
05/12/202	30/12/202	03/12/202	2	1
24/12/202	30/12/202	30/12/202	0	
20/08/202	15/09/202	15/09/202	2	
24/04/202	05/05/202	11/04/202	2	1
13/05/202	13/05/202	05/05/202	9	1
28/05/202	13/07/202	13/07/202	2	
12/08/202	23/09/202	24/06/202	2	1
29/11/202	29/04/202	29/04/202	0	
08/07/202	06/07/202	06/07/202	9	1
18/09/202	21/09/202	10/08/202	2	1
03/11/202	04/11/202	22/10/202	2	1
13/01/202	13/01/202	05/01/202	2	1
20/08/202	21/08/202	06/08/202	9	1
23/06/202	25/06/202	07/05/202	9	1
30/05/202	15/06/202	26/05/202	2	
11/12/202	11/12/202	28/11/202	2	
05/08/202	06/08/202	23/07/202	0	1
21/05/202	17/06/202	22/05/202	2	1
23/04/202	21/07/202	14/05/202	9	1
06/01/202	07/01/202	29/12/202	2	1
13/12/202	31/12/202	31/12/202	0	
13/05/202	09/06/202	15/05/202	0	1
30/01/202	04/02/202	15/01/202	2	1
05/06/202	08/06/202	22/05/202	2	1
17/08/202	20/08/202	05/08/202	2	1
28/06/202	15/09/202	19/06/202	2	1
20/12/202	17/02/202	10/12/202	2	
21/05/202	19/05/202	19/05/202	9	1
04/08/202	26/08/202	13/07/202	2	1
22/08/202	05/11/202	03/08/202	2	1
09/12/202	14/12/202	08/12/202	2	
22/06/202	22/06/202	18/06/202	9	1
13/12/202	19/01/202	19/01/202	2	
14/09/202	15/09/202	18/08/202	2	
09/10/202	09/10/202	01/10/202	2	
20/05/202	21/05/202	21/05/202	9	1
09/07/202	22/07/202	22/06/202	2	1
22/12/202	07/01/202	07/01/202	2	1

03/07/202	03/07/202	07/08/202	9	
04/06/202	15/06/202	27/05/202	2	1
10/06/202	11/06/202	01/06/202	0	1
16/05/202	18/05/202	12/05/202	2	1
07/10/202	07/10/202	07/10/202	2	1
15/07/202	21/07/202	13/07/202	0	1
12/08/202	14/08/202	05/08/202	2	1
16/06/202	16/06/202	21/07/202	0	1
24/10/202	25/10/202	09/07/202	2	1
11/10/202	18/06/202	16/10/202	0	
19/03/202	20/03/202	05/02/202	0	
18/03/202	29/03/202	16/03/202	0	1
27/03/202	29/03/202	21/03/202	0	1
13/04/202	22/11/202	22/03/202	0	1
17/04/202	19/04/202	16/04/202	0	
26/04/202	26/04/202	20/04/202	0	1
25/06/202	25/06/202	30/05/202	0	1
06/06/202	06/06/202	08/06/202	0	
17/07/202	19/07/202	22/06/202	0	
08/08/202	09/08/202	25/06/202	0	1
04/09/202	06/09/202	24/09/202	0	1
14/04/202	21/04/202	08/11/202	0	
16/02/202	16/02/202	16/02/202	2	1
01/03/202	01/03/202	19/02/202	0	1
07/03/202	26/04/202	24/02/202	0	1
21/03/2021		08/03/202	0	1
28/03/202	28/03/202	24/03/202	0	1
	26/04/202	19/04/202	0	1
24/05/202	25/05/202	25/05/202	0	1
02/07/202	02/07/202	21/06/202	0	1
29/06/202	29/06/202	15/06/202	0	1
05/07/202	06/07/202	21/06/202	0	
27/09/202	28/09/202	22/09/202	0	1
04/06/202	05/06/202	27/12/202	0	
07/01/202	07/01/202	07/01/202	0	1
24/03/202	13/04/202	08/03/202	0	1
25/03/202	29/03/202	26/03/202	0	
26/04/202	04/05/202	31/03/202	0	
30/03/202	31/03/202	31/03/202	0	1
		14/04/202	0	
29/03/202	29/03/202	21/05/202	0	
10/06/202	14/06/202	14/06/202	0	1
06/05/202	06/05/202	24/06/202	0	
06/07/202	10/07/202	01/07/202	0	1
27/07/202	31/08/202	06/07/202	0	
26/07/202	27/07/202	16/07/202	0	
12/08/202	13/08/202	11/08/202	0	1

13/07/202	16/07/202	16/07/202	0	1
18/09/202	23/09/202	09/09/202	0	
12/04/202	26/07/202	01/04/202	0	1
15/04/202	28/04/202	28/04/202	0	1
30/05/202	31/05/202	24/05/202	0	1
22/05/202	23/05/202	23/05/202	0	
08/07/202	13/06/202	13/06/202	0	1
11/05/202	11/05/202	29/06/202	0	
04/07/202	05/07/202	07/07/202	0	1
03/08/202	14/08/202	24/07/202	0	1
15/08/202	15/08/202	16/07/202	0	1
19/06/2021		23/07/202	0	1
24/09/202	24/09/202	25/08/202	0	
11/09/202	23/09/202	03/09/202	0	1
10/10/202	21/10/202	21/10/202	0	
19/02/202	19/02/202	04/02/202	0	1
01/03/202	25/03/202	01/03/202	0	
02/04/202	05/05/202	24/03/202	0	
26/04/202	26/04/202	31/03/202	0	1
12/04/202	18/04/202	08/04/202	0	1
21/04/202	06/05/202	19/04/202	0	
15/05/202	09/04/202	04/05/202	0	1
24/05/202	25/05/202	18/05/202	0	
02/05/202	07/05/202	18/05/202	0	
20/05/202	11/06/202	14/05/202	0	1
05/03/202	05/03/202	02/06/202	0	
05/03/202	05/03/202	31/05/202	0	
11/06/202	22/06/202	22/06/202	0	
22/02/202	22/02/202	07/07/202	0	1
19/10/202	19/10/202	29/09/202	0	
11/06/202	11/06/202	29/07/202	0	1
26/03/202	26/03/202	05/08/202	0	1
22/09/202	23/09/202	06/09/202	0	1
14/04/202	24/08/202	24/08/202	0	1
14/10/202	18/10/202	14/09/202	0	1
27/10/202	28/10/202	22/09/202	0	
07/01/202	14/02/202	05/02/202	0	
28/02/202	01/03/202	17/02/202	2	1
09/04/202	10/04/202	25/02/202	0	
22/03/202	22/03/202	18/03/202	2	1
31/03/202	31/03/202	18/03/202	0	1
29/05/202	31/05/202	26/04/202	0	1
24/05/202	25/05/202	30/04/202	0	
02/06/202	08/06/202	01/06/202	0	1
09/05/202	09/05/202	30/06/202	0	
29/06/202	20/12/202	24/06/202	0	1
30/07/202	10/08/202	30/06/202	0	1

08/09/202	10/09/202	05/09/202	0	
01/11/202	01/11/202	29/11/202	0	1
17/02/202	03/03/202	15/02/202	2	1
09/04/202	13/04/202	26/03/202	0	1
07/04/202	12/06/202	06/04/202	0	1
03/04/202	05/04/202	26/03/202	0	1
31/03/202	07/05/202	30/03/202	0	1
30/03/202	31/03/202	31/03/202	0	1
06/04/202	06/04/202	06/04/202	0	1
09/04/202	30/05/202	08/04/202	0	1
16/04/202	18/04/202	13/04/202	0	1
03/06/202	09/06/202	01/06/202	0	1
04/06/202	04/06/202	17/06/202	0	1
30/06/202	01/07/202	15/06/202	0	1
28/06/202	30/06/202	23/06/202	0	1
13/08/202	13/08/202	10/08/202	0	
09/12/202	17/12/202	30/11/202	0	1
25/02/202	01/03/202	22/02/202	0	1
26/02/202	01/03/202	01/03/202	9	1
02/05/202	04/05/202	14/04/202	0	1
08/06/202	08/06/202	18/05/202	0	1
04/06/202	04/06/202	21/05/202	0	1
17/06/202	24/06/202	04/06/202	0	1
11/06/202	16/06/202	25/05/202	0	
06/07/202	07/07/202	05/07/202	0	1
01/07/202	07/07/202	24/06/202	0	
03/07/202	03/07/202	30/06/202	0	1
17/08/202	23/08/202	26/07/202	0	
27/07/202	20/08/202	22/07/202	0	
20/07/202	23/07/202	23/07/202	0	1
16/04/202	16/04/202	30/08/202	0	
11/10/202	15/10/202	08/10/202	0	
16/01/202	21/01/202	14/01/202	0	1
11/04/202	15/04/202	25/03/202	0	
07/05/202	19/05/202	12/04/202	0	
14/05/202	17/11/202	09/04/202	0	
05/06/202	06/06/202	08/05/202	0	1
26/04/202	26/04/202	14/04/202	0	1
30/05/202	31/05/202	10/05/202	0	1
14/05/202	14/05/202	10/05/202	0	1
24/05/202	25/05/202	19/05/202	0	1
31/05/202	31/05/202	18/05/202	0	
25/07/202	21/06/202	14/06/202	0	
16/06/202	16/06/202	16/07/202	0	
	15/07/202	15/07/202	0	1
10/10/202	22/10/202	15/10/202	0	1
	26/08/202	25/08/202	0	1

30/08/202	31/08/202	16/08/202	0	1
04/08/202	04/08/202	29/07/202	0	1
23/01/202	05/09/202	05/09/202	0	1
02/10/202	06/10/202	08/09/202	0	1
	28/03/202	12/01/202	0	
06/04/202	07/04/202	09/03/202	0	1
01/04/202	01/04/202	24/03/202	0	1
25/04/202	25/04/202	12/04/202	0	1
30/05/202	30/05/202	23/05/202	0	1
25/04/202	27/08/202	26/04/202	0	
22/06/202	24/06/202	21/05/202	0	1
25/05/202	07/06/202	17/05/202	0	
18/06/202	21/06/202	04/06/202	0	
25/05/202	25/05/202	24/06/202	0	
15/07/202	16/07/202	23/06/202	0	1
02/06/202	23/06/202	23/06/202	0	1
27/06/202	06/07/202	06/07/202	0	
14/07/202	19/07/202	30/06/202	0	
27/10/202	27/10/202	25/10/202	0	
17/08/202	17/08/202	06/08/202	0	1
07/01/202	30/01/202	28/12/202	0	
14/10/202	18/10/202	21/09/202	0	
16/02/202	23/02/202	19/01/202	2	1
11/03/202	11/03/202	13/01/202	2	
22/03/202	04/04/202	15/03/202	0	1
29/03/202	30/03/202	15/03/202	0	1
02/04/202	12/04/202	31/03/202	0	1
14/04/202	19/04/202	12/04/202	0	1
03/05/202	06/05/202	07/04/202	0	
22/04/202	28/04/202	14/04/202	0	1
21/06/202	21/06/202	18/06/202	0	
06/05/202	24/09/202	24/09/202	0	1
31/10/202	31/10/202	28/10/202	0	
11/04/202	25/08/202	08/04/202	0	1
18/05/202	19/05/202	14/05/202	0	1
12/05/202	11/06/202	30/04/202	0	1
17/04/202	17/04/202	14/05/202	0	1
28/05/202	28/05/202	18/05/202	0	1
04/06/202	06/06/202	23/05/202	0	1
01/02/202	01/02/202	07/07/202	0	
30/05/202	09/12/202	14/05/202	0	
23/07/202	15/08/202	14/07/202	0	1
12/06/202	13/09/202	21/06/202	0	1
26/06/202	02/07/202	10/06/202	0	1
28/05/202	28/05/202	05/07/202	0	
25/06/202	25/06/202	20/07/202	0	
28/01/202	29/01/202	20/01/202	0	1

13/03/202	17/03/202	26/02/202	2	
25/03/202	25/03/202	25/03/202	0	1
10/03/202	10/03/202	08/03/202	0	
23/03/202	24/03/202	18/03/202	0	1
25/04/202	25/04/202	27/03/202	0	1
20/04/202	26/04/202	26/04/202	0	1
15/04/202	15/04/202	11/05/202	0	1
30/07/202	02/08/202	17/06/202	0	
29/04/202	29/04/202	07/06/202	0	
02/08/202	03/08/202	28/07/202	0	1
14/08/202	17/08/202	06/08/202	0	
20/04/202	20/04/202	16/09/202	0	
12/12/202	15/12/202	02/12/202	0	1
07/02/202	17/02/202	05/02/202	0	
09/11/202	10/11/202	08/10/202	0	
17/02/202	21/02/202	08/12/202	0	1
12/01/202	13/01/202	11/01/202	0	
03/04/202	05/04/202	07/04/202	0	1
22/03/202	24/03/202	08/03/202	0	
17/04/202	19/04/202	05/04/202	0	1
08/04/202	15/04/202	05/04/202	0	1
15/03/202	12/04/202	12/04/202	0	
29/03/202	29/03/202	31/03/202	0	
30/05/202	09/06/202	24/05/202	0	1
11/05/202	12/05/202	13/04/202	0	1
04/06/202	07/06/202	07/06/202	0	
07/04/202	16/04/202	07/04/202	0	1
22/04/202	22/04/202	17/04/202	0	1
21/05/202	01/06/202	07/05/202	0	1
08/06/202	22/06/202	12/05/202	0	1
05/05/202	07/05/202	04/05/202	0	1
21/06/202	21/06/202	25/05/202	0	1
15/06/202	26/06/202	07/06/202	0	
15/06/202	21/06/202	14/06/202	0	
05/07/202	06/07/202	30/06/202	0	1
12/08/202	20/08/202	27/07/202	0	1
18/05/202	18/05/202	29/07/202	0	
02/09/202	07/10/202	07/10/202	0	
21/02/202	11/03/202	17/02/202	2	
01/04/202	02/04/202	08/03/202	0	1
15/02/202	15/02/202	18/03/202	2	1
22/03/202	13/04/202	22/03/202	0	1
11/04/202	12/04/202	30/03/202	0	1
23/04/202	23/04/202	23/04/202	0	1
09/05/202	06/05/202	06/05/202	0	1
20/05/202	21/05/202	17/05/202	0	1
24/05/202	28/05/202	28/05/202	0	1

28/05/202	07/06/202	26/05/202	0	1
03/06/202	02/07/202	28/05/202	0	1
25/06/202	06/07/202	15/06/202	0	
24/01/202	24/01/202	07/07/202	0	1
01/06/202	23/06/202	08/07/202	0	
08/09/202	20/09/202	03/09/202	0	1
12/03/202	12/03/202	19/02/202	2	1
15/03/202	15/03/202	05/03/202	0	
06/04/202	06/04/202	14/05/202	0	
30/06/202	16/07/202	25/05/202	0	
14/05/202	29/06/202	01/05/202	0	1
20/05/202	21/05/202	21/05/202	0	
23/06/202	23/06/202	15/06/202	0	1
19/05/202	19/05/202	28/06/202	0	
12/07/202	12/07/202	06/07/202	0	
10/07/202	12/07/202	29/06/202	0	1
13/07/202	13/07/202	08/07/202	0	1
19/08/202	20/08/202	22/07/202	0	1
27/08/202	06/09/202	27/08/202	0	1
	19/01/202	16/01/202	0	1
26/01/202	27/01/202	13/01/202	2	1
26/03/202	29/03/202	09/03/202	0	
22/03/202	29/03/202	10/03/202	0	1
07/07/202	07/07/202	12/04/202	0	
24/03/202	25/03/202	17/03/202	0	1
22/04/202	28/04/202	08/03/202	0	1
05/05/202	17/05/202	17/05/202	0	
14/04/202	15/04/202	08/04/202	0	1
17/04/202	19/04/202	19/04/202	0	
11/06/202	11/06/202	24/05/202	0	1
02/06/202	21/06/202	27/05/202	0	
05/06/202	28/05/202	28/05/202	0	1
10/09/202	17/11/202	13/09/202	0	1
16/03/202	17/03/202	16/03/202	0	1
24/04/202	24/04/202	24/03/202	0	
15/04/202	19/04/202	24/03/202	0	1
25/03/202	30/03/202	30/03/202	0	1
07/03/202	09/03/202	13/02/202	0	1
26/01/202	28/01/202	05/03/202	2	
27/03/202	30/03/202	30/03/202	0	1
17/03/202	01/04/202	17/03/202	0	1
30/04/202	30/04/202	22/03/202	0	1
17/05/202	20/05/202	12/05/202	0	1
27/04/202	27/04/202	12/04/202	0	
31/05/202	02/06/202	25/05/202	0	1
07/05/202	17/05/202	20/04/202	0	1
	02/07/202	04/06/202	0	1

16/06/202	23/06/202	19/05/202	0	1
30/05/202	31/05/202	31/05/202	0	1
19/08/202	26/08/202	02/09/202	0	
05/06/202	05/07/202	08/07/202	0	1
13/04/202	13/04/202	19/08/202	0	
16/08/202	17/08/202	21/07/202	0	1
31/08/202	01/09/202	26/08/202	0	1
22/10/202	23/10/202	19/10/202	0	1
24/02/202	04/05/202	09/03/202	0	
09/04/202	18/05/202	05/04/202	0	
16/04/202	16/04/202	16/04/202	0	1
04/06/202	07/06/202	06/03/202	0	1
10/05/202	11/05/202	26/04/202	0	
14/06/202	14/06/202	27/05/202	0	1
12/06/202	15/06/202	25/05/202	0	1
12/04/202	12/04/202	12/04/202	0	
24/05/202	24/05/202	28/04/202	0	
19/04/202	27/04/202	15/04/202	0	1
18/04/202	23/04/202	26/04/202	0	1
04/06/202	03/09/202	28/05/202	0	1
12/06/202	16/06/202	16/06/202	0	1
30/05/202	19/08/202	23/06/202	0	1
03/08/202	04/08/202	25/06/202	0	
27/08/202	30/08/202	13/08/202	0	
25/01/202	24/08/202	24/08/202	0	
13/06/202	29/07/202	29/07/202	0	1
22/03/202	26/03/202	17/03/202	0	1
22/04/202	23/04/202	15/03/202	0	1
18/03/202	18/03/202	11/03/202	2	
23/07/202	02/04/202	02/04/202	0	1
16/05/202	17/05/202	13/04/202	0	1
28/06/202	29/06/202	17/06/202	0	1
03/03/202	04/06/202	04/06/202	0	1
04/06/202	09/06/202	19/07/202	0	1
09/01/202	09/02/202	11/01/202	0	1
14/12/202	15/12/202	01/12/202	0	1
19/01/202	14/01/202	08/01/202	0	1
03/02/202	04/02/202	15/01/202	2	1
03/03/202	03/03/202	02/02/202	0	
12/04/202	12/04/202	12/04/202	0	1
29/03/2021		06/03/202	0	1
25/05/202	14/05/202	14/05/202	0	1
23/04/202	23/04/202	14/04/202	0	1
02/06/202	02/06/202	20/07/202	0	
05/06/202	08/06/202	02/06/202	0	1
16/06/202	02/07/202	27/05/202	0	1
17/06/202	18/06/202	10/06/202	0	

27/06/202	08/07/202	06/06/202	0	1
22/05/202	28/05/202	28/05/202	0	
27/06/202	29/06/202	01/07/202	0	1
16/07/202	20/07/202	20/07/202	0	1
02/06/202	04/06/202	16/07/202	0	
06/04/202	11/11/202	15/03/202	0	1
06/04/2021		30/03/202	0	1
08/04/202	22/04/202	05/04/202	0	1
09/04/202	12/04/202	26/03/202	0	1
14/05/202	18/05/202	16/04/202	0	1
01/07/202	02/07/202	28/06/202	0	1
14/05/202	26/04/202	23/04/202	0	1
15/03/202	14/05/202	14/05/202	0	1
02/06/202	03/06/202	03/06/202	0	
12/05/202	12/05/202	06/05/202	0	1
29/05/202	21/06/202	24/05/202	0	1
20/08/202	23/08/202	11/08/202	0	
01/06/202	01/06/202	28/05/202	0	
28/02/202	02/03/202	23/02/202	0	1
21/04/202	22/04/202	10/03/202	0	1
09/04/202	02/07/202	26/03/202	0	1
08/04/202	16/04/202	16/04/202	0	1
02/06/202	02/06/202	06/05/202	0	1
21/04/202	07/05/202	07/05/202	0	1
12/05/202	20/07/202	13/05/202	0	
17/06/202	23/06/202	07/06/202	0	
23/07/202	26/07/202	24/05/202	0	1
05/03/202	05/03/202	07/07/202	0	1
12/07/202	16/07/202	08/06/202	0	1
28/06/202	28/06/202	19/06/202	0	1
	16/09/202	05/09/202	0	1
16/09/202	05/10/202	05/10/202	0	
09/03/202	09/03/202	05/03/202	2	1
14/04/202	14/04/202	15/03/202	0	1
13/04/202	03/08/202	29/03/202	0	1
28/03/202	28/03/202	25/03/202	0	
22/07/202	23/07/202	10/06/202	0	1
18/05/202	18/05/202	20/04/202	0	1
02/06/202	09/06/202	24/05/202	0	1
23/06/202	23/06/202	16/06/202	0	1
21/06/202	24/06/202	14/06/202	0	1
05/06/202	06/06/202	25/05/202	0	
24/06/202	24/06/202	19/06/202	0	
16/06/202	14/06/202	14/06/202	0	1
26/05/202	02/06/202	08/06/202	0	1
19/07/202	20/07/202	15/07/202	0	
27/06/202	23/07/202	23/07/202	0	1

05/08/202	23/08/202	30/07/202	0	
15/10/202	18/10/202	08/10/202	0	
18/04/202	18/04/202	16/04/202	0	1
09/02/202	09/02/202	28/01/202	0	
19/03/202	26/03/202	30/03/202	0	1
07/05/202	12/05/202	20/04/202	0	1
	07/05/202	26/04/202	0	1
17/04/202	19/04/202	19/04/202	0	1
23/07/202	04/08/202	01/07/202	0	1
27/05/202	27/05/202	12/05/202	0	1
09/06/202	10/06/202	10/06/202	0	1
26/05/202	26/05/202	24/05/202	0	1
08/07/202	17/07/202	25/06/202	0	1
26/06/202	17/12/202	24/06/202	0	
10/07/202	10/07/202	07/07/202	0	
22/07/202	21/10/202	24/07/202	0	
27/03/202	29/03/202	10/03/202	0	1
06/05/202	01/05/202	01/05/202	0	1
23/04/202	26/04/202	16/04/202	0	
20/06/202	30/06/202	18/05/202	0	1
26/03/202	31/03/202	06/04/202	0	1
01/06/202	01/06/202	28/05/202	0	
19/06/202	19/06/202	11/06/202	0	
02/06/202	09/07/202	09/07/202	0	
29/07/202	26/07/202	26/07/202	0	1
24/07/202	26/07/202	26/07/202	0	
14/07/202	05/09/202	05/09/202	0	1
23/08/202	23/08/202	28/09/202	0	1
04/01/202	25/01/202	25/12/202	0	1
09/04/202	15/04/202	05/04/202	0	1
24/04/202	26/04/202	19/04/202	0	1
04/05/202	04/05/202	26/04/202	0	1
28/04/202	11/05/202	08/04/202	0	1
08/05/202	14/05/202	22/04/202	0	1
11/05/202	13/05/202	13/05/202	0	
18/06/202	18/06/202	17/06/202	0	1
29/05/202	01/06/202	01/06/202	0	
05/06/202	08/06/202	08/06/202	0	1
03/07/202	07/07/202	19/06/202	0	
28/07/202	31/07/202	23/07/202	0	
08/07/202	29/07/202	29/07/202	0	
23/06/202	15/07/202	29/06/202	0	1
14/03/202	14/03/202	20/07/202	0	1
08/07/202	16/12/202	29/06/202	0	
23/01/202	26/01/202	14/01/202	2	1
16/07/202	03/09/202	30/06/202	0	
03/01/202	05/01/202	31/12/202	0	

01/03/202	05/03/202	23/02/202	2	1
31/03/202	31/03/202	10/03/202	0	1
06/04/202	01/04/202	01/04/202	0	1
18/02/202	18/02/202	13/04/202	0	1
28/04/202	30/04/202	19/04/202	0	1
26/03/202	04/05/202	04/05/202	0	1
25/05/202	25/05/202	11/05/202	0	
31/05/202	14/06/202	14/06/202	0	
30/06/202	09/09/202	14/06/202	0	
22/05/202	04/10/202	04/10/202	0	
10/08/202	20/08/202	20/07/202	0	
24/11/202	28/02/202	19/11/202	0	1
26/04/202	28/07/202	24/08/202	0	1
30/04/202	08/07/202	19/03/202	0	1
23/05/202	23/05/202	01/04/202	0	1
27/04/202	29/04/202	05/04/202	0	1
03/04/202	05/04/202	30/03/202	0	1
04/05/202	11/05/202	26/04/202	0	1
01/04/202	01/04/202	25/03/202	0	1
20/05/202	21/05/202	07/04/202	0	
05/06/202	05/06/202	26/05/202	0	1
06/07/202	20/07/202	29/06/202	0	1
20/06/202	01/07/202	24/05/202	0	1
28/09/202	29/09/202	09/08/202	0	1
27/07/202	02/08/202	28/06/202	0	1
08/07/202	12/07/202	25/06/202	0	
24/07/202	23/09/202	22/07/202	0	1
26/08/202	02/09/202	29/07/202	0	
30/07/202	30/07/202	27/07/202	0	1
09/08/202	09/08/202	06/08/202	0	1
06/09/202	08/09/202	08/09/202	0	1
18/11/202	23/11/202	09/11/202	0	1
	24/01/202	21/01/202	0	1
27/04/202	28/04/202	06/04/202	0	1
26/03/202	01/04/202	15/03/202	0	
05/04/202	06/04/202	27/03/202	0	1
24/04/202	12/05/202	12/05/202	0	1
13/05/202	26/05/202	26/05/202	0	1
17/05/202	06/05/202	06/05/202	0	1
21/02/202	14/05/202	14/05/202	0	1
04/06/202	07/06/202	28/05/202	0	
27/05/202	02/06/202	24/05/202	0	
06/06/202	25/07/202	27/05/202	0	1
10/05/202	13/05/202	27/05/202	0	
28/04/202	18/05/202	03/05/202	0	
09/06/202	02/07/202	27/05/202	0	1
28/05/202	08/06/202	08/06/202	0	1

27/06/202	29/06/202	22/06/202	0	1
29/06/202	14/07/202	22/06/202	0	1
25/07/202	26/07/202	13/07/202	0	1
04/09/202	22/11/202	26/08/202	0	1
22/06/202	23/06/202	31/05/202	0	1
06/09/202	06/09/202	26/07/202	0	1
24/06/202	28/06/202	04/06/202	0	1
16/06/202	14/07/202	14/07/202	0	1
26/08/202	11/11/202	08/07/202	0	1
12/03/202	15/03/202	11/03/202	0	1
12/03/202	12/03/202	03/03/202	0	1
05/03/202	18/03/202	16/03/202	2	1
08/05/202	08/05/202	19/03/202	0	
03/04/202	21/07/202	17/03/202	0	1
22/04/202	17/04/202	17/04/202	0	1
22/03/202	22/03/202	28/04/202	0	1
	19/05/202	11/05/202	0	1
12/06/202	12/06/202	25/05/202	0	1
29/06/202	12/07/202	12/07/202	0	1
	07/08/202	30/07/202	0	1
05/07/202	09/07/202	09/07/202	0	
26/08/202	15/10/202	05/11/202	0	1
18/07/202	18/07/202	10/08/202	0	1
10/03/202	13/05/202	09/03/202	0	1
25/04/202	26/04/202	16/04/202	0	1
11/04/202	16/04/202	30/03/202	0	
06/04/202	08/04/202	05/04/202	0	1
31/03/202	28/10/202	04/03/202	0	
16/05/202	22/07/202	30/04/202	0	1
25/04/202	26/04/202	28/04/202	0	
05/06/202	15/06/202	10/05/202	0	
20/04/202	20/04/202	09/04/202	0	
05/03/202	05/03/202	01/06/202	0	
15/06/202	16/06/202	16/06/202	0	1
02/07/202	17/07/202	30/06/202	0	1
16/07/202	20/07/202	02/07/202	0	1
22/04/202	31/03/202	07/05/202	0	
19/06/202	08/07/202	08/07/202	0	
28/06/202	18/11/202	17/06/202	0	1
09/04/202	28/07/202	28/07/202	0	1
24/08/202	04/10/202	05/10/202	0	
26/06/202	26/06/202	20/07/202	0	
20/01/202	26/01/202	12/01/202	2	
26/07/202	26/07/202	11/11/202	0	
13/09/202	15/09/202	04/09/202	0	1
	04/01/202	04/01/202	0	1
26/04/202	26/04/202	05/04/202	0	1

24/04/202	12/11/202	07/04/202	0	
12/04/202	06/05/202	15/04/202	0	
14/05/202	18/05/202	10/05/202	0	1
12/05/202	25/06/202	10/05/202	0	1
	28/05/202	28/05/202	0	1
17/06/202	21/06/202	11/06/202	0	
14/06/202	14/06/202	22/06/202	0	
21/07/202	22/07/202	29/04/202	0	1
12/08/202	17/11/202	16/07/202	0	1
29/06/202	01/07/202	24/06/202	0	1
05/06/202	15/06/202	15/06/202	0	1
03/10/2021		27/09/202	0	
19/09/202	20/09/202	03/09/202	0	
05/08/202	05/08/202	28/09/202	0	1
23/06/202	11/08/202	11/08/202	0	1
20/04/202	20/04/202	26/02/202	0	1
15/02/202	16/02/202	05/02/202	0	1
22/03/202	29/03/202	19/03/202	0	1
24/04/202	06/05/202	22/03/202	0	1
12/05/202	24/06/202	14/04/202	0	1
09/06/202	22/08/202	18/05/202	0	1
22/05/202	26/05/202	26/05/202	0	
25/06/202	08/12/202	25/05/202	0	1
30/05/202	10/06/202	26/05/202	0	
20/05/202	20/05/202	25/06/202	0	
30/07/202	02/08/202	21/07/202	0	
26/06/202	28/06/202	28/06/202	0	
20/07/202	24/09/202	22/07/202	0	1
01/09/202	01/09/202	24/08/202	0	
31/10/202	31/10/202	18/10/202	0	1
17/08/202	17/08/202	10/08/202	0	1
27/02/202	16/04/202	16/04/202	0	1
25/04/202	26/04/202	22/04/202	0	1
22/04/202	27/04/202	14/04/202	0	1
18/05/202	10/02/202	26/04/202	0	
10/06/202	10/06/202	04/06/202	0	
20/04/202	26/05/202	26/05/202	0	1
05/08/202	05/10/202	04/08/202	0	
16/09/202	16/09/202	04/08/202	0	
27/04/202	27/04/202	21/07/202	0	
06/07/202	29/07/202	30/06/202	0	1
08/08/202	09/08/202	19/07/202	0	1
26/07/202	27/07/202	23/07/202	0	
26/02/202	06/04/202	07/02/202	0	1
14/03/202	13/05/202	03/03/202	0	1
06/03/202	08/03/202	24/02/202	0	1
05/04/202	07/04/202	29/03/202	0	

15/04/202	16/04/202	16/04/202	0	
28/03/202	28/03/202	01/04/202	0	1
12/06/202	08/06/202	08/06/202	0	1
27/06/202	27/06/202	24/06/202	0	1
29/05/202	11/06/202	26/05/202	0	1
29/06/202	29/06/202	30/06/202	0	
12/04/202	30/04/202	30/04/202	0	1
03/07/202	12/07/202	26/06/202	0	1
19/08/202	20/08/202	12/07/202	0	1
22/07/202	22/07/202	21/07/202	0	
19/03/202	20/03/202	06/03/202	0	1
02/08/202	02/08/202	12/11/202	0	
12/05/202	19/05/202	12/04/202	0	1
05/05/202	21/05/202	29/04/202	0	
30/04/202	06/05/202	24/03/202	0	1
22/05/202	23/05/202	14/05/202	0	1
11/06/202	11/06/202	01/06/202	0	1
01/07/202	20/07/202	26/05/202	0	1
13/06/202	16/06/202	16/06/202	0	
15/09/202	16/09/202	08/09/202	0	
21/07/202	21/07/202	13/07/202	0	1
01/07/202	02/07/202	25/06/202	0	1
09/07/202	12/07/202	08/07/202	0	
18/06/202	18/06/202	19/07/202	0	
23/03/202	30/05/202	17/03/202	0	1
06/04/202	08/04/202	25/03/202	0	1
31/03/202	07/04/202	19/03/202	0	1
27/02/2021		17/03/202	0	
31/03/202	31/03/202	28/03/202	0	1
15/05/202	20/05/202	21/04/202	0	1
18/06/202	24/06/202	07/06/202	0	1
12/04/202	13/04/202	08/04/202	0	1
17/05/202	08/06/202	08/06/202	0	1
05/06/202	07/06/202	07/06/202	0	
04/05/202	09/06/202	13/04/202	0	1
01/05/202	13/05/202	24/04/202	0	1
03/06/202	04/06/202	04/06/202	0	1
08/06/202	08/06/202	07/06/202	0	
17/05/202	18/05/202	10/05/202	0	1
24/07/202	27/07/202	27/07/202	0	1
16/05/202	22/05/202	28/07/202	0	1
15/06/202	16/06/202	06/06/202	0	
04/02/202	05/02/202	13/01/202	2	1
23/08/202	20/09/202	10/11/202	0	
24/03/202	21/03/202	20/03/202	0	1
02/04/2021		25/03/202	0	1
29/03/202	08/04/202	08/04/202	0	1

10/05/202	11/05/202	05/05/202	0	
23/06/202	24/06/202	24/05/202	0	1
02/06/202	07/06/202	07/06/202	0	1
18/06/202	22/06/202	16/06/202	0	1
25/05/202	26/05/202	26/05/202	0	
16/05/202	21/05/202	15/05/202	0	
30/05/202	09/06/202	08/06/202	0	
21/07/202	26/07/202	26/07/202	0	1
14/09/202	15/09/202	09/09/202	0	
15/08/202	14/09/202	30/08/202	0	1
04/07/202	30/07/202	25/06/202	0	1
08/10/202	11/10/202	05/10/202	0	
23/06/202	18/06/202	18/06/202	0	1
11/05/202	11/05/202	29/06/202	0	
17/10/202	25/11/202	29/09/202	0	
05/08/202	21/08/202	17/07/202	0	1
02/07/202	15/07/202	16/03/202	0	1
17/05/202	17/05/202	24/04/202	0	
26/02/202	27/02/202	11/03/202	2	1
18/03/202	18/03/202	18/03/202	2	1
01/05/202	11/05/202	11/05/202	0	
05/06/202	08/06/202	09/06/202	0	1
28/05/202	28/05/202	30/06/202	0	1
07/06/202	18/10/202	07/06/202	0	1
28/06/202	28/06/202	21/06/202	0	1
16/11/202	23/11/202	11/11/202	0	1
22/02/202	01/03/202	22/02/202	2	1
08/04/202	15/04/202	22/03/202	0	
26/02/202	26/02/202	26/02/202	2	
	18/02/202	03/02/202	0	
04/05/202	12/05/202	04/03/202	0	
19/02/202	27/03/202	17/02/202	0	1
25/04/202	27/04/202	23/04/202	0	1
12/04/202	03/08/202	01/04/202	0	1
24/03/202	31/07/202	22/03/202	0	1
27/04/202	27/04/202	01/07/202	0	
19/05/202	28/05/202	28/05/202	0	1
10/07/202	23/07/202	23/07/202	0	1
26/06/202	28/06/202	28/06/202	0	
17/08/202	18/08/202	05/08/202	0	1
09/08/202	09/08/202	06/10/202	0	1
16/04/202	19/04/202	13/04/202	0	
25/03/202	07/05/202	19/03/202	0	
12/04/202	03/05/202	07/04/202	0	1
17/06/202	18/06/202	18/05/202	0	
06/04/202	13/04/202	13/04/202	0	1
03/04/202	12/04/202	26/04/202	0	1

27/03/202	27/03/202	22/03/202	0	1
09/05/202	17/05/202	07/05/202	0	1
09/04/202	15/04/202	31/03/202	0	
02/07/202	02/07/202	25/06/202	0	1
24/08/202	17/09/202	20/08/202	0	1
25/08/202	27/08/202	02/07/202	0	1
21/09/202	05/10/202	23/07/202	0	1
22/07/202	29/07/202	29/07/202	0	
23/11/202	24/11/202	12/11/202	0	
23/03/202	23/03/202	18/03/202	0	1
05/03/202	11/03/202	26/02/202	2	
28/03/202	05/05/202	31/03/202	0	1
21/03/202	22/03/202	18/03/202	0	1
26/04/202	30/04/202	12/04/202	0	
27/03/202	09/04/202	24/03/202	0	
29/03/202	30/03/202	25/03/202	0	1
09/05/202	10/05/202	04/05/202	0	1
16/03/202	19/03/202	25/03/202	0	1
06/04/202	06/04/202	14/05/202	0	
24/05/202	25/05/202	22/05/202	0	1
	15/04/202	13/04/202	0	
09/07/202	12/07/202	28/06/202	0	
03/07/202	06/07/202	12/07/202	0	1
01/09/202	02/09/202	20/08/202	0	
14/04/202	16/04/202	16/03/202	0	1
07/03/202	12/03/202	24/02/202	2	
02/05/202	03/05/202	12/04/202	0	1
07/05/202	08/05/202	30/04/202	0	
27/04/202	27/04/202	22/04/202	0	1
27/04/202	29/04/202	26/04/202	0	
21/06/202	22/06/202	08/06/202	0	
02/07/202	22/07/202	22/07/202	0	
09/07/202	17/07/202	05/07/202	0	1
19/12/202	27/12/202	10/12/202	0	
09/03/202	10/03/202	10/03/202	9	1
14/03/202	27/03/202	10/03/202	0	1
31/03/202	07/05/202	26/03/202	0	
14/04/202	19/04/202	13/04/202	0	1
24/03/202	26/04/202	26/04/202	0	1
29/04/202	30/04/202	14/04/202	0	1
16/05/202	26/05/202	03/05/202	0	
28/04/202	28/04/202	04/05/202	0	
23/05/202	24/05/202	07/05/202	0	1
18/06/202	23/06/202	19/05/202	0	1
27/04/202	13/05/202	28/04/202	0	1
16/07/202	19/07/202	14/06/202	0	1
22/08/202	22/08/202	19/08/202	0	

19/06/202	19/06/202	10/06/202	0	1
04/05/202	01/06/202	23/06/202	0	1
20/06/202	20/06/202	21/06/202	0	
17/03/202	18/03/202	08/03/202	0	1
09/04/202	10/04/202	23/02/202	0	
05/03/202	10/03/202	04/03/202	2	1
30/04/202	07/05/202	12/04/202	0	1
22/03/202	10/05/202	09/03/202	0	1
26/07/202	26/07/202	22/06/202	0	
02/02/202	16/06/202	16/06/202	0	1
31/05/202	31/05/202	06/05/202	0	1
02/06/202	03/06/202	01/06/202	0	
04/04/202	04/04/202	28/12/202	0	
23/12/202	27/12/202	09/12/202	0	1
30/07/202	04/08/202	16/07/202	0	
23/10/202	18/11/202	22/10/202	0	1
05/08/202	02/08/202	04/08/202	0	1
		31/08/202	0	1
16/02/2021		23/02/202	0	1
24/02/202	25/02/202	26/02/202	0	1
07/05/202	14/05/202	26/04/202	0	
10/04/202	12/04/202	05/04/202	0	1
15/05/202	17/05/202	15/04/202	0	1
09/05/202	14/06/202	11/05/202	0	1
09/06/202	10/06/202	09/06/202	0	1
09/06/202	09/06/202	06/06/202	0	1
15/07/202	19/07/202	21/06/202	0	1
15/06/202	16/06/202	02/06/202	0	1
01/08/202	03/08/202	12/07/202	0	1
06/01/202	11/01/202	06/01/202	0	1
29/01/202	15/04/202	20/01/202	0	1
25/12/202	25/12/202	23/12/202	0	
14/05/202	17/05/202	20/04/202	0	1
19/05/202	20/05/202	17/05/202	0	
06/03/202	09/03/202	10/03/202	2	1
11/06/202	02/07/202	04/06/202	0	1
30/06/202	01/07/202	01/06/202	0	
25/06/202	25/06/202	28/06/202	0	1
07/10/202	07/10/202	01/10/202	0	1
28/03/202	29/03/202	29/03/202	0	1
05/04/202	21/03/202	24/03/202	0	1
09/04/202	15/04/202	15/04/202	0	1
21/06/202	22/06/202	10/06/202	0	1
20/06/202	28/07/202	17/06/202	0	
06/06/202	17/06/202	08/06/202	0	1
04/08/202	13/08/202	09/07/202	0	
11/07/202	12/07/202	14/07/202	0	1

25/07/202	25/07/202	30/07/202	0	
27/08/202	13/10/202	13/10/202	0	1
11/12/202	07/12/202	07/12/202	0	
23/11/202	24/11/202	08/11/202	0	
28/08/202	02/09/202	25/08/202	0	
21/02/202	22/02/202	22/02/202	9	1
05/03/202	30/04/202	23/02/202	0	1
01/05/202	08/05/202	11/05/202	0	1
08/05/202	11/05/202	05/05/202	0	1
19/06/202	02/07/202	07/06/202	0	1
20/05/202	24/06/202	24/06/202	0	1
23/08/202	30/07/202	29/07/202	0	1
26/05/202	26/05/202	01/06/202	0	
30/09/202	30/09/202	14/09/202	0	1
09/04/202	25/06/202	25/06/202	0	1
17/06/202	19/06/202	19/06/202	0	1
24/02/202	30/03/202	15/02/202	0	1
18/02/202	18/02/202	17/02/202	2	
29/04/202	30/04/202	26/03/202	0	
01/04/202	29/03/202	29/03/202	0	1
26/04/202	26/04/202	20/04/202	0	1
16/04/202	04/05/202	08/04/202	0	1
09/04/202	20/04/202	20/04/202	0	1
05/05/202	22/10/202	28/04/202	0	
27/05/202	25/05/202	25/05/202	0	1
19/03/202	15/03/202	15/03/202	0	1
28/03/202	01/04/202	19/03/202	0	1
03/07/202	20/09/202	12/07/202	0	1
05/07/202	06/07/202	20/06/202	0	
04/07/202	05/07/202	14/06/202	0	1
09/05/202	21/05/202	21/05/202	0	1
09/05/202	01/07/202	01/07/202	0	1
04/04/202	19/08/202	19/08/202	0	1
08/10/202	08/10/202	13/09/202	0	1
05/04/202	05/04/202	29/03/202	0	1
08/05/202	20/07/202	22/04/202	0	
23/02/202	26/02/202	16/02/202	2	1
07/04/202	07/04/202	05/04/202	0	1
01/06/202	14/12/202	06/05/202	0	1
25/04/202	25/04/202	06/05/202	0	
17/09/202	25/10/202	10/09/202	0	
18/07/202	09/02/202	13/07/202	0	
19/08/202	23/08/202	16/08/202	0	1
12/05/202	12/05/202	19/07/202	0	1
30/11/202	06/12/202	18/11/202	0	1
19/12/202	19/12/202	27/11/202	0	1
02/04/202	05/04/202	30/03/202	0	1

15/04/202	02/09/202	02/09/202	0	1
08/03/202	08/04/202	10/03/202	0	1
21/05/202	21/05/202	17/05/202	0	1
15/04/202	28/04/202	07/04/202	0	1
07/05/202	08/05/202	01/05/202	0	1
10/08/202	11/08/202	09/08/202	0	1
16/05/202	05/01/202	19/05/202	0	1
01/06/202	02/06/202	26/05/202	0	1
19/10/202	08/11/202	08/11/202	0	1
08/08/202	17/09/202	17/09/202	0	1
27/10/202	27/10/202	16/11/202	0	1
22/06/202	23/06/202	15/06/202	0	1
29/08/202	29/09/202	25/08/202	0	1
24/03/202	25/03/202	17/03/202	0	
18/05/202	19/05/202	14/05/202	0	
19/04/202	10/05/202	10/05/202	0	1
25/03/202	26/03/202	16/03/202	0	
20/06/202	23/06/202	28/05/202	0	
16/08/202	18/08/202	05/07/202	0	1
18/01/202	18/01/202	11/01/202	0	
22/02/202	26/02/202	13/02/202	0	
25/04/202	25/04/202	27/03/202	0	
21/03/202	24/03/202	22/03/202	0	1
30/03/202	07/05/202	24/03/202	0	
30/06/202	01/07/202	29/03/202	0	
30/03/202	31/03/202	30/03/202	0	1
06/04/202	27/04/202	22/03/202	0	1
25/05/202	26/05/202	06/05/202	0	
15/05/202	17/05/202	23/04/202	0	1
20/06/202	21/06/202	15/06/202	0	1
27/05/202	14/06/202	14/06/202	0	1
21/06/202	22/06/202	18/06/202	0	1
11/07/202	11/07/202	05/07/202	0	
07/07/202	09/07/202	28/06/202	0	1
07/07/202	13/07/202	07/07/202	0	
06/03/202	06/03/202	01/03/202	2	
27/04/202	27/04/202	20/04/202	0	1
20/04/202	03/08/202	16/04/202	0	1
17/05/202	17/05/202	14/05/202	0	1
22/06/202	15/06/202	17/06/202	0	1
28/06/202	29/06/202	17/06/202	0	1
17/10/202	28/10/202	30/06/202	0	
12/04/202	30/04/202	20/04/202	0	
28/04/202	28/04/202	28/04/202	0	
27/07/202	24/08/202	24/08/202	0	
15/06/202	17/07/202	17/07/202	0	
04/04/202	18/05/202	18/05/202	0	

10/12/202	20/12/202	14/12/202	0	1
11/03/202	11/03/202	04/03/202	2	1
19/04/202	23/04/202	10/03/202	0	
15/04/202	27/04/202	05/04/202	0	1
10/04/202	10/04/202	08/04/202	0	
18/04/202	19/04/202	28/04/202	0	1
23/06/202	23/06/202	20/05/202	0	1
07/06/202	07/06/202	24/05/202	0	1
17/05/202	02/06/202	12/05/202	0	1
18/06/202	02/07/202	11/06/202	0	1
12/07/202	15/07/202	02/07/202	0	
17/08/202	29/09/202	12/08/202	0	1
01/09/202	01/09/202	30/08/202	0	
21/08/202	28/08/202	19/08/202	0	1
	31/01/202	28/01/202	0	1
20/03/202	22/03/202	08/02/202	0	
03/02/202	03/02/202	01/02/202	2	1
10/08/202	20/08/202	20/07/202	0	
12/02/202	12/02/202	15/02/202	0	1
03/04/202	29/05/202	30/03/202	0	1
12/04/202	13/04/202	13/04/202	0	1
15/04/202	15/04/202	31/03/202	0	
29/06/202	02/07/202	24/06/202	0	1
20/04/202	23/04/202	03/04/202	0	1
23/06/202	23/06/202	25/06/202	0	1
15/04/202	02/07/202	02/07/202	0	
14/08/202	27/09/202	05/08/202	0	1
07/06/202	08/06/202	01/06/202	0	
18/12/202	05/01/202	05/01/202	0	
06/12/202	07/12/202	30/11/202	0	
01/04/202	12/04/202	24/03/202	0	1
13/04/202	13/04/202	12/04/202	0	1
16/04/202	21/04/202	18/04/202	0	1
30/04/202	17/06/202	19/04/202	0	
25/05/202	26/05/202	19/05/202	0	
07/07/202	19/07/202	09/06/202	0	1
10/04/202	10/04/202	23/03/202	0	
08/06/202	09/06/202	01/06/202	0	1
11/07/202	27/07/202	23/06/202	0	
19/07/202	17/08/202	17/08/202	0	
21/10/202	21/10/202	04/08/202	0	1
20/09/202	21/09/202	21/09/202	0	1
04/06/202	07/06/202	06/06/202	0	
16/02/202	24/02/202	24/02/202	0	1
06/03/202	29/10/202	11/02/202	0	1
05/04/202	05/04/202	30/03/202	0	1
30/03/202	30/03/202	29/03/202	0	1

	20/04/202	06/04/202	0	1
24/04/202	25/04/202	19/04/202	0	1
04/04/202	28/04/202	31/03/202	0	1
15/05/202	19/05/202	10/05/202	0	1
07/05/202	21/05/202	07/05/202	0	
21/05/202	25/05/202	10/05/202	0	1
15/05/202	15/05/202	12/05/202	0	
04/06/202	21/05/202	21/05/202	0	1
20/04/202	24/04/202	29/03/202	0	
03/07/202	05/07/202	18/06/202	0	
07/08/202	09/08/202	13/07/202	0	
22/05/202	24/05/202	11/05/202	0	
	02/04/202	29/09/202	0	
13/06/202	14/06/202	16/06/202	0	1
06/04/202	07/04/202	11/03/202	0	1
24/06/202	16/07/202	28/03/202	0	
11/04/202	13/04/202	11/03/202	0	1
25/05/202	17/09/202	12/11/202	0	
06/04/202	12/04/202	14/04/202	0	1
22/03/202	22/03/202	19/03/202	2	1
18/05/202	19/05/202	10/05/202	0	
31/05/202	31/05/202	18/05/202	0	1
23/05/202	24/05/202	18/05/202	0	1
22/05/202	14/07/202	20/05/202	0	1
08/05/202	09/05/202	01/05/202	0	1
06/03/202	17/03/202	17/03/202	2	1
30/07/202	02/08/202	30/06/202	0	1
26/08/202	27/08/202	19/07/202	0	1
21/09/202	22/09/202	17/08/202	0	1
18/03/202	22/03/202	22/03/202	0	1
12/04/202	12/04/202	01/04/202	0	
07/05/202	07/05/202	09/03/202	0	1
23/03/202	23/03/202	07/04/202	0	
16/05/202	19/06/202	10/05/202	0	1
06/08/202	16/08/202	16/08/202	0	
26/05/2021		12/05/202	0	
04/06/202	04/06/202	16/07/202	0	
05/01/202	10/01/202	04/01/202	0	
21/01/202	21/01/202	18/01/202	2	
13/10/202	13/10/202	18/10/202	0	1
17/03/202	07/05/202	11/03/202	0	
19/08/202	02/09/202	30/07/202	0	
26/02/202	08/07/202	09/02/202	0	1
15/04/202	16/04/202	29/03/202	0	1
30/03/202	06/04/202	16/03/202	0	
14/04/202	15/04/202	12/04/202	0	1
22/04/202	22/04/202	25/03/202	0	1

28/06/202	01/07/202	13/05/202	0	1
01/06/202	01/06/202	18/05/202	0	1
26/06/202	08/07/202	24/06/202	0	
08/06/202	08/06/202	01/06/202	0	
16/07/202	17/07/202	03/07/202	0	1
28/07/202	29/07/202	08/07/202	0	1
23/09/202	28/09/202	20/09/202	0	
19/10/202	20/10/202	05/10/202	0	1
30/08/202	02/09/202	27/12/202	0	1
24/10/202	18/11/202	22/10/202	0	
19/02/202	03/03/202	21/01/202	9	1
14/03/202	14/03/202	10/03/202	0	1
04/04/202	06/08/202	13/04/202	0	
18/02/202	08/04/202	08/04/202	0	
15/05/202	18/05/202	18/05/202	0	
24/04/202	25/04/202	14/05/202	0	
05/05/202	07/05/202	30/04/202	0	1
16/04/202	19/04/202	19/04/202	0	
28/05/202	03/06/202	22/05/202	0	
29/04/202	30/04/202	22/04/202	0	
01/06/202	04/06/202	19/05/202	0	1
06/04/202	08/04/202	29/03/202	0	
28/06/202	28/06/202	29/05/202	0	1
18/06/202	18/06/202	19/07/202	0	
26/07/202	15/07/202	15/07/202	0	1
26/06/202	20/07/202	20/07/202	0	1
21/03/202	21/03/202	31/05/202	0	
08/06/202	09/06/202	09/06/202	0	1
09/06/202	14/06/202	25/06/202	0	1
05/03/202	10/03/202	01/02/202	2	1
22/02/202	10/03/202	25/02/202	0	1
	16/08/202	16/08/202	0	1
24/09/202	20/10/202	08/11/202	0	
13/06/202	14/06/202	26/04/202	0	1
28/04/202	29/04/202	29/04/202	0	
12/04/202	14/04/202	12/04/202	0	
25/04/202	25/04/202	07/05/202	0	
23/06/202	05/07/202	14/06/202	0	1
13/05/202	17/05/202	19/03/202	0	1
20/06/202	20/06/202	21/06/202	0	
06/06/202	07/06/202	08/06/202	0	
16/06/202	18/06/202	19/07/202	0	1
20/06/202	05/07/202	06/07/202	0	1
08/09/202	13/09/202	05/10/202	0	
18/11/202	23/11/202	04/11/202	0	1
09/06/202	20/08/202	20/08/202	0	
07/06/202	15/06/202	15/06/202	0	1

01/08/202	31/08/202	31/08/202	0	
18/03/202	24/04/202	09/03/202	0	1
13/03/202	18/03/202	02/03/202	2	1
26/04/202	27/04/202	23/04/202	0	
26/03/202	30/03/202	30/03/202	0	1
05/06/202	07/06/202	01/06/202	0	1
25/06/202	25/06/202	21/06/202	0	1
03/07/202	13/07/202	25/06/202	0	
02/06/202	02/06/202	18/06/202	0	
05/06/202	02/07/202	27/05/202	0	1
23/08/202	01/09/202	11/08/202	0	1
27/07/202	28/07/202	12/07/202	0	1
14/08/202	14/08/202	13/08/202	0	
08/09/202	13/09/202	18/08/202	0	
16/03/202	12/05/202	11/03/202	0	
04/05/202	06/05/202	29/04/202	0	1
23/03/202	20/10/202	25/03/202	0	1
28/05/202	17/06/202	17/06/202	0	
02/07/202	05/07/202	26/06/202	0	
20/06/202	26/06/202	17/06/202	0	1
13/07/202	20/07/202	24/06/202	0	1
05/07/202	05/07/202	21/06/202	0	1
02/07/202	05/07/202	29/06/202	0	
14/07/202	22/09/202	20/07/202	0	1
07/12/202	23/12/202	01/12/202	0	1
29/03/202	04/10/202	04/10/202	0	
12/12/202	07/12/202	07/12/202	0	
04/08/202	05/08/202	03/08/202	0	1
05/03/202	08/03/202	24/02/202	0	1
06/04/202	08/04/202	08/04/202	0	
17/04/202	22/04/202	22/04/202	0	1
18/04/202	03/08/202	29/03/202	0	1
16/06/202	18/06/202	21/05/202	0	1
	08/12/202	25/07/202	0	
		23/07/202	0	1
12/06/202	05/06/202	31/05/202	0	1
01/05/202	03/05/202	09/06/202	0	
16/06/202	07/07/202	07/07/202	0	1
07/04/202	07/05/202	30/03/202	0	1
01/04/202	01/04/202	20/04/202	0	
17/02/202	18/02/202	28/04/202	0	1
26/01/202	28/04/202	28/04/202	0	1
18/06/202	18/06/202	07/06/202	0	1
28/05/202	31/05/202	04/05/202	0	1
26/06/202	30/06/202	27/05/202	0	1
24/05/202	24/05/202	14/05/202	0	
12/02/202	11/03/202	15/02/202	2	1

16/04/202	17/04/202	17/04/202	0	1
11/06/202	14/06/202	14/06/202	0	
19/05/202	21/05/202	12/05/202	0	1
31/03/202	01/04/202	01/04/202	0	1
30/04/202	10/05/202	08/04/202	0	
05/08/202	06/08/202	21/07/202	0	1
23/02/202	23/02/202	07/07/202	0	1
10/05/202	19/05/202	04/05/202	0	
19/05/202	19/05/202	10/05/202	0	
02/02/202	10/02/202	25/01/202	9	
01/03/202	12/05/202	25/02/202	0	1
26/03/202	01/04/202	17/03/202	0	1
12/03/202	12/03/202	29/03/202	0	
03/05/202	17/05/202	09/04/202	0	1
01/09/202	05/09/202	05/09/202	0	1
23/05/202	25/05/202	07/05/202	0	1
14/04/202	19/04/202	12/04/202	0	1
20/05/202	20/05/202	16/05/202	0	
07/01/202	19/01/202	05/01/202	0	1
26/04/202	10/06/202	27/04/202	0	
28/02/202	04/06/202	04/06/202	0	
08/04/202	14/04/202	14/04/202	0	1
08/06/202	08/06/202	02/06/202	0	1
14/07/202	14/07/202	29/06/202	0	
28/03/202	29/03/202	11/03/202	0	1
	23/08/202	18/08/202	0	1
19/07/202	21/07/202	17/07/202	0	
21/07/202	06/08/202	02/07/202	0	
28/08/202	30/08/202	04/08/202	0	1
	24/11/202	24/11/202	0	
11/01/202	12/01/202	10/01/202	0	
12/04/202	19/04/202	29/03/202	0	
28/09/202	29/09/202	29/09/202	0	
02/04/202	02/04/202	23/03/202	0	1
04/04/202	06/04/202	08/04/202	0	1
23/06/202	25/06/202	25/06/202	0	1
31/05/202	17/09/202	26/05/202	0	
06/07/202	12/07/202	01/06/202	0	1
28/05/202	01/06/202	01/06/202	0	1
08/06/202	09/06/202	09/06/202	0	
24/07/202	06/08/202	11/08/202	0	1
06/07/202	13/07/202	05/07/202	0	
27/06/202	29/06/202	01/07/202	0	1
16/12/202	25/01/202	17/12/202	0	1
	16/07/202	06/07/202	0	1
17/03/202	18/03/202	11/03/202	0	1
20/08/202	20/08/202	13/08/202	0	

	06/04/202	10/03/202	0	1
02/08/202	09/08/202	10/06/202	0	1
10/08/202	11/08/202	24/06/202	0	1
04/08/202	24/11/202	04/07/202	0	1
03/05/202	04/05/202	12/04/202	0	1
16/07/202	23/07/202	05/07/202	0	1
09/09/202	10/09/202	02/09/202	0	
30/06/202	12/07/202	19/05/202	0	1
12/11/202	09/12/202	09/12/202	0	
04/05/202	04/05/202	26/04/202	0	1
04/01/202	10/01/202	13/12/202	0	1
19/04/202	10/06/202	12/04/202	0	
27/03/202	27/03/202	21/05/202	0	
20/05/202	24/05/202	05/05/202	0	1
25/06/202	28/06/202	27/05/202	0	1
06/07/202	04/08/202	04/08/202	0	1
20/01/2022		20/01/202	0	
21/08/202	10/09/202	10/08/202	0	1
05/01/202	07/01/202	03/01/202	0	1
26/04/202	30/06/202	26/04/202	0	1
27/04/202	04/05/202	04/05/202	0	1
26/03/202	23/04/202	23/04/202	0	1
04/08/202	20/08/202	15/07/202	0	1
01/02/202	12/02/202	12/02/202	2	1
23/04/202	27/04/202	27/04/202	0	1
07/03/202	20/03/202	05/03/202	0	1
15/03/202	19/03/202	10/03/202	2	1
18/03/202	06/04/202	24/02/202	0	1
16/03/202	19/03/202	15/03/202	0	1
19/03/202	23/03/202	17/03/202	0	1
05/07/202	10/08/202	10/08/202	0	
17/08/202	22/09/202	09/08/202	0	
27/05/2021		24/05/202	0	
22/10/202	22/10/202	21/10/202	0	1
02/07/202	09/09/202	11/06/202	0	
29/12/202	13/01/202	30/12/202	0	
23/08/202	23/08/202	16/08/202	0	
28/08/202	17/09/202	17/09/202	0	
23/02/202	24/02/202	04/03/202	2	
10/09/202	13/09/202	08/09/202	0	1
11/02/202	22/02/202	03/02/202	0	1
07/03/202	08/03/202	08/03/202	2	
05/04/202	12/04/202	12/04/202	0	1
17/03/202	17/03/202	24/02/202	0	1
16/06/202	16/06/202	26/05/202	0	1
03/05/202	13/05/202	29/04/202	0	1
05/06/202	02/09/202	21/05/202	0	1

12/05/202	24/04/202	18/04/202	0	1
11/04/202	12/04/202	12/04/202	0	
19/04/202	12/04/202	12/04/202	0	
17/04/202	15/10/202	20/04/202	0	1
23/03/202	23/03/202	25/05/202	0	
26/07/202	28/07/202	20/07/202	0	1
		23/07/202	0	1
07/09/202	09/09/202	13/09/202	0	1
30/03/202	08/04/202	02/04/202	0	1
15/01/202	20/01/202	10/01/202	2	
07/09/202	19/10/202	19/10/202	0	
04/03/202	04/03/202	24/02/202	0	1
09/06/202	15/06/202	24/05/202	0	1
03/04/202	06/04/202	06/04/202	0	1
30/04/202	12/07/202	26/04/202	0	
25/01/202	02/02/202	02/02/202	9	
05/07/202	16/07/202	16/07/202	0	1
26/04/202	28/04/202	12/04/202	0	1
23/06/202	23/06/202	15/04/202	0	
18/05/202	19/05/202	13/05/202	0	1
23/08/202	02/09/202	26/08/202	0	1
20/09/202	22/09/202	10/07/202	0	
31/05/202	31/05/202	20/06/202	0	1
24/09/202	28/09/202	16/08/202	0	1
16/08/202	16/08/202	10/08/202	0	
	18/08/202	18/08/202	0	1
22/05/202	17/09/202	12/11/202	0	
	17/05/202	19/02/202	0	1
19/04/202	22/04/202	12/03/202	0	1
19/03/202	20/03/202	16/03/202	0	1
05/07/202	05/07/202	15/06/202	0	
02/04/202	02/04/202	22/04/202	0	
19/07/202	20/07/202	05/07/202	0	1
30/05/202	02/07/202	28/05/202	0	1
29/03/202	29/03/202	16/04/202	0	1
12/06/202	13/06/202	13/06/202	0	1
07/05/202	19/05/202	26/04/202	0	1
29/09/202	06/10/202	20/09/202	0	
20/04/202	07/05/202	07/05/202	0	1
09/06/202	10/06/202	10/06/202	0	1
20/04/202	26/04/202	13/04/202	0	1
07/10/202	13/10/202	26/09/202	0	1
09/08/202	30/08/202	09/07/202	0	
08/06/202	26/11/202	24/05/202	0	1
11/11/202	11/11/202	05/11/202	0	1
17/09/202	18/12/202	20/12/202	0	
28/09/202	06/10/202	27/09/202	0	

02/03/202	03/03/202	03/03/202	0	
25/02/202	26/02/202	23/02/202	2	
03/06/202	01/07/202	31/05/202	0	1
27/03/202	29/03/202	29/03/202	0	1
19/04/202	08/05/202	31/03/202	0	1
09/04/202	18/04/202	09/04/202	0	1
02/05/202	03/05/202	13/04/202	0	
09/07/202	10/07/202	17/06/202	0	
20/06/202	16/06/202	07/06/202	0	1
27/05/202	01/06/202	24/05/202	0	1
11/06/202	14/06/202	07/06/202	0	
10/08/202	10/08/202	04/08/202	0	
01/06/202	01/06/202	20/07/202	0	
19/04/202	19/04/202	01/09/202	0	
01/07/202	22/09/202	22/09/202	0	
03/09/202	06/09/202	23/08/202	0	1
13/04/202	24/08/202	25/08/202	0	1
29/03/202	07/04/202	24/03/202	0	
20/07/202	22/07/202	17/06/202	0	1
23/04/202	04/05/202	22/03/202	0	
06/08/202	06/08/202	17/07/202	0	1
16/03/202	16/03/202	05/03/202	0	1
19/03/202	22/03/202	18/03/202	0	1
24/03/202	24/03/202	24/05/202	0	
02/05/202	03/05/202	04/05/202	0	
31/03/202	31/03/202	27/05/202	0	
28/06/202	30/06/202	24/06/202	0	1
01/09/202	02/09/202	13/08/202	0	
10/05/202	05/09/202	05/09/202	0	1
20/03/202	22/03/202	18/03/202	0	1
25/05/202	28/05/202	20/05/202	0	
15/03/202	23/08/202	17/03/202	0	1
15/04/202	15/04/202	26/03/202	0	1
16/04/202	17/04/202	12/04/202	0	1
05/04/202	06/04/202	06/04/202	0	1
12/04/202	03/08/202	29/03/202	0	1
17/04/202	17/04/202	01/04/202	0	1
09/07/202	09/07/202	06/08/202	0	1
20/06/202	20/06/202	09/06/202	0	
08/04/202	16/04/202	13/04/202	0	1
30/03/202	30/03/202	25/03/202	0	1
13/05/202	18/05/202	18/05/202	0	
11/05/202	12/05/202	12/05/202	0	
20/05/202	23/08/202	01/05/202	0	1
25/07/202	02/08/202	02/08/202	0	1
04/06/202	07/06/202	09/06/202	0	1
01/10/202	21/10/202	09/09/202	0	1

01/07/202	02/07/202	25/06/202	0	
05/01/202	12/01/202	01/01/202	0	
08/11/202	08/11/202	20/01/202	0	
22/05/202	24/05/202	19/05/202	0	1
16/01/202	03/03/202	03/01/202	0	
06/06/202	10/06/202	24/05/202	0	1
03/05/202	03/05/202	22/04/202	0	
30/04/202	30/04/202	30/04/202	0	1
17/06/202	23/06/202	26/05/202	0	1
21/06/202	28/06/202	24/05/202	0	1
26/06/202	26/06/202	13/07/202	0	1
17/06/202	22/06/202	12/06/202	0	
13/07/202	14/07/202	30/06/202	0	
09/01/202	26/01/202	19/01/202	0	1
21/07/202	22/07/202	05/07/202	0	1
11/11/202	11/11/202	14/10/202	0	
29/07/202	30/07/202	18/06/202	0	
25/07/202	26/07/202	15/07/202	0	
11/07/202	20/07/202	14/07/202	0	1
11/04/202	14/04/202	29/03/202	0	1
14/02/202	16/02/202	12/02/202	9	1
26/02/202	01/03/202	17/02/202	0	1
14/03/202	14/03/202	25/03/202	0	1
	25/02/202	25/02/202	0	1
01/04/202	02/04/202	02/04/202	0	1
28/05/202	08/06/202	20/05/202	0	
07/07/202	08/09/202	27/05/202	0	
15/03/202	16/03/202	09/03/202	0	1
18/05/202	21/09/202	10/05/202	0	1
19/03/202	19/03/202	16/03/202	0	
31/07/202	28/12/202	18/06/202	0	1
31/08/202	14/09/202	14/09/202	0	
28/07/202	06/08/202	19/07/202	0	1
26/06/202	19/07/202	15/07/202	0	1
02/10/202	05/10/202	17/09/202	0	1
10/06/202	19/07/202	25/05/202	0	1
15/08/202	16/08/202	09/08/202	0	
22/11/202	26/11/202	10/11/202	0	1
10/05/202	11/05/202	29/03/202	0	
26/01/202	26/01/202	14/01/202	0	1
06/03/202	08/03/202	08/03/202	2	1
17/04/202	19/04/202	19/04/202	0	1
11/04/202	14/04/202	05/04/202	0	1
10/02/202	03/02/202	28/01/202	9	1
03/07/202	12/07/202	01/07/202	0	1
03/06/202	07/07/202	07/07/202	0	
04/06/202	27/07/202	17/06/202	0	1

11/08/202	17/09/202	17/09/202	0	1
05/08/202	27/09/202	03/08/202	0	1
06/09/202	08/09/202	20/08/202	0	1
	29/04/202	25/01/202	0	
22/04/202	26/04/202	15/04/202	0	1
17/04/202	20/04/202	20/04/202	0	1
16/02/202	28/02/202	15/02/202	9	1
20/03/202	26/03/202	26/03/202	0	
08/09/202	10/09/202	25/08/202	0	
19/05/202	30/05/202	19/05/202	0	
26/04/202	28/04/202	30/04/202	0	1
03/08/202	05/08/202	27/07/202	0	1
24/08/202	25/08/202	10/08/202	0	1
01/10/202	05/10/202	23/08/202	0	
18/08/202	18/08/202	18/08/202	0	
12/04/202	15/04/202	24/03/202	0	1
10/05/202	12/05/202	12/05/202	0	1
29/05/202	31/05/202	25/05/202	0	1
05/06/202	18/06/202	21/06/202	0	1
11/08/202	18/08/202	11/08/202	0	
31/05/202	01/06/202	01/06/202	0	1
16/07/202	04/09/202	20/07/202	0	1
28/03/202	05/04/202	05/04/202	0	1
10/06/202	24/06/202	27/05/202	0	1
23/03/202	07/05/202	17/03/202	0	
09/04/202	12/04/202	07/04/202	0	
03/07/202	15/12/202	01/07/202	0	1
23/06/202	24/06/202	02/06/202	0	
11/06/202	15/06/202	15/06/202	0	1
15/04/202	16/04/202	01/04/202	0	1
26/06/202	28/06/202	28/06/202	0	1
25/07/202	25/07/202	28/07/202	0	1
14/09/202	15/09/202	13/09/202	0	
06/04/202	16/04/202	16/04/202	0	
06/03/202	13/03/202	24/02/202	0	1
13/10/202	13/10/202	04/10/202	0	
12/03/202	16/04/202	11/03/202	0	
06/04/202	06/04/202	29/03/202	0	
08/04/202	01/07/202	01/04/202	0	1
29/04/202	30/04/202	07/05/202	0	
16/09/202	17/09/202	08/09/202	0	1
25/05/202	26/05/202	23/04/202	0	
15/06/202	15/06/202	11/06/202	0	
18/04/202	13/07/202	13/07/202	0	
01/04/202	01/04/202	16/04/202	0	1
05/09/202	05/09/202	09/09/202	0	
06/09/202	09/09/202	30/08/202	0	

15/08/202	19/08/202	13/08/202	0	1
22/07/202	26/07/202	20/07/202	0	1
09/08/202	09/08/202	14/07/202	0	
04/05/202	05/05/202	26/04/202	0	1
14/03/202	14/03/202	09/02/202	0	1
12/06/202	18/06/202	18/06/202	0	
29/04/202	04/05/202	31/03/202	0	
31/03/202	31/03/202	14/04/202	0	1
21/04/202	08/09/202	22/06/202	0	1
26/03/202	05/04/202	04/03/202	0	1
24/03/202	25/03/202	15/03/202	0	1
22/04/202	22/04/202	08/03/202	0	1
21/09/202	19/10/202	06/09/202	0	
28/03/202	31/07/202	28/03/202	0	1
24/10/202	24/10/202	23/10/202	0	
05/04/202	27/04/202	27/04/202	0	1
03/03/202	04/03/202	18/02/202	0	
07/06/202	07/06/202	21/04/202	0	
14/05/202	17/05/202	20/04/202	0	1
28/06/202	01/07/202	14/06/202	0	1
09/04/202	20/05/202	20/05/202	0	1
30/09/202	01/10/202	22/09/202	0	1
29/05/202	31/05/202	31/05/202	0	
31/07/202	31/07/202	02/08/202	0	1
22/05/202	01/06/202	14/05/202	0	1
14/09/202	19/10/202	03/09/202	0	
29/05/202	29/11/202	29/11/202	0	
03/08/202	10/08/202	19/07/202	0	1
21/08/202	16/09/202	21/01/202	0	1
18/06/202	21/06/202	16/07/202	0	1
15/12/202	15/12/202	31/03/202	0	
17/04/202	19/04/202	19/04/202	0	
24/03/202	24/03/202	25/03/202	0	1
27/04/202	27/04/202	02/04/202	0	
11/05/202	11/05/202	03/05/202	0	
05/06/202	07/06/202	02/06/202	0	1
19/05/202	19/05/202	12/05/202	0	1
09/06/202	21/07/202	22/05/202	0	1
01/07/202	01/07/202	23/06/202	0	
29/06/202	29/06/202	28/06/202	0	1
17/04/202	19/04/202	06/04/202	0	1
22/07/202	23/07/202	20/07/202	0	1
15/05/202	18/05/202	05/05/202	0	
30/06/202	30/06/202	15/06/202	0	1
10/05/202	05/06/202	27/06/202	0	
	04/02/202	01/02/202	9	1
17/07/202	26/07/202	10/07/202	0	1

25/05/202	26/05/202	30/04/202	0	1
24/03/202	01/04/202	15/03/202	0	1
25/10/202	25/10/202	26/10/202	0	
25/03/2021		09/03/202	0	1
02/07/202	05/07/202	19/06/202	0	1
26/04/202	27/04/202	13/04/202	0	1
16/07/202	27/12/202	16/07/202	0	1
29/06/202	08/09/202	09/06/202	0	
18/03/202	19/04/202	05/05/202	0	1
02/06/202	09/06/202	22/05/202	0	
		05/07/202	0	1
16/06/202	17/06/202	07/06/202	0	1
24/07/202	28/07/202	13/07/202	0	
27/07/202	16/11/202	27/07/202	0	
12/06/202	18/06/202	31/05/202	0	1
14/08/202	17/08/202	02/08/202	0	
15/06/202	15/06/202	10/08/202	0	
24/07/202	09/11/202	10/11/202	0	1
01/08/202	02/08/202	29/07/202	0	1
30/08/202	31/08/202	23/08/202	0	1
15/09/202	23/11/202	10/09/202	0	
28/05/202	07/06/202	15/05/202	0	
	18/02/202	18/02/202	2	1
22/11/202	23/11/202	10/11/202	0	
02/05/202	04/05/202	12/04/202	0	1
26/01/202	26/01/202	18/01/202	2	1
13/04/2021		09/04/202	0	
19/03/202	04/03/202	04/03/202	0	1
29/05/202	28/05/202	28/05/202	0	
29/06/202	29/06/202	22/06/202	0	
14/04/202	20/04/202	20/05/202	0	
30/04/202	30/04/202	05/04/202	0	
05/07/202	06/07/202	15/06/202	0	
31/05/202	31/05/202	02/06/202	0	1
28/06/202	30/06/202	16/06/202	0	
14/06/202	14/06/202	16/07/202	0	
08/07/202	08/07/202	05/07/202	0	1
13/10/202	13/10/202	01/10/202	0	
24/04/202	27/04/202	30/04/202	0	1
07/06/202	20/11/202	30/03/202	0	1
07/06/202	23/06/202	27/04/202	0	1
22/03/202	22/03/202	20/07/202	0	1
08/02/202	09/02/202	03/02/202	2	1
01/07/202	02/07/202	01/07/202	0	1
12/08/202	12/08/202	05/07/202	0	
14/06/202	15/07/202	15/07/202	0	
02/08/202	06/12/202	28/07/202	0	1

17/04/202	19/04/202	30/03/202	0	1
28/02/202	15/03/202	15/03/202	9	1
05/10/202	16/11/202	16/11/202	0	1
25/06/202	30/06/202	26/05/202	0	1
01/07/202	01/07/202	10/06/202	0	1
14/03/202	14/03/202	21/02/202	2	
20/03/202	16/03/202	10/03/202	0	1
01/05/202	04/05/202	27/04/202	0	
15/05/202	17/05/202	29/04/202	0	
18/07/202	14/12/202	19/07/202	0	
15/06/202	16/06/202	22/04/202	0	1
06/06/202	07/06/202	07/06/202	0	1
31/05/202	09/06/202	18/05/202	0	1
27/05/202	28/05/202	24/05/202	0	1
04/06/202	09/06/202	27/05/202	0	1
14/07/202	05/08/202	30/06/202	0	1
02/08/202	27/09/202	03/08/202	0	1
04/07/202	21/09/202	16/07/202	0	1
12/10/202	12/10/202	28/09/202	0	1
29/10/202	15/11/202	21/09/202	0	1
	14/06/202	14/06/202	0	
19/05/202	20/05/202	27/04/202	0	1
26/05/202	17/06/202	17/06/202	0	1
25/04/2021		19/06/202	0	
21/04/202	23/04/202	10/03/202	0	
25/03/202	25/03/202	16/03/202	0	1
19/06/202	02/07/202	07/06/202	0	1
28/04/202	03/05/202	23/04/202	0	
25/04/202	25/04/202	27/04/202	0	1
11/05/202	12/05/202	12/05/202	0	1
29/10/202	29/10/202	08/09/202	0	
22/06/202	22/06/202	16/06/202	0	
25/08/202	30/08/202	30/08/202	0	1
23/03/202	25/03/202	12/03/202	0	1
18/04/202	20/04/202	01/04/202	0	
14/04/202	16/04/202	06/04/202	0	
04/04/202	09/04/202	13/02/202	0	1
26/03/202	17/04/202	03/03/202	0	1
21/04/202	21/04/202	20/04/202	0	1
10/03/2021		16/03/202	0	
24/05/202	24/05/202	24/06/202	0	
12/06/202	14/06/202	17/08/202	0	1
20/05/202	20/05/202	11/05/202	0	1
20/05/202	24/05/202	13/05/202	0	1
21/05/202	26/05/202	21/05/202	0	
29/08/202	13/10/202	13/10/202	0	
29/07/202	03/09/202	19/07/202	0	

06/08/202	06/08/202	02/08/202	0	1
16/05/2021		30/07/202	0	1
08/07/202	15/07/202	15/07/202	0	
15/04/202	07/12/202	31/03/202	0	1
14/08/202	17/08/202	12/08/202	0	1
03/05/202	03/05/202	03/05/202	0	1
25/09/202	25/09/202	22/09/202	0	
28/03/202	30/03/202	24/03/202	0	1
11/09/202	13/09/202	08/09/202	0	1
14/05/202	17/05/202	17/05/202	0	1
14/10/202	06/12/202	13/10/202	0	1
08/04/202	12/04/202	07/04/202	0	1
01/05/202	04/05/202	22/04/202	0	
22/05/202	22/05/202	23/04/202	0	
05/04/202	05/04/202	05/04/202	0	1
30/04/202	27/08/202	26/04/202	0	1
07/04/202	28/05/202	22/04/202	0	
04/04/202	19/07/202	28/04/202	0	1
21/06/202	03/08/202	15/06/202	0	
05/04/202	03/05/202	02/03/202	0	
30/09/202	08/10/202	29/09/202	0	1
11/04/202	08/07/202	08/07/202	0	1
11/11/202	12/11/202	12/11/202	0	1
22/01/202	06/08/202	13/01/202	0	1
11/02/202	11/02/202	03/02/202	0	
20/04/202	12/07/202	31/03/202	0	1
17/02/202	11/05/202	10/02/202	0	
14/04/202	14/04/202	16/04/202	0	
13/09/202	15/09/202	28/07/202	0	
16/06/202	21/05/202	21/05/202	0	1
28/03/202	08/04/202	26/03/202	0	1
15/06/202	10/05/202	11/05/202	0	1
16/06/202	14/09/202	25/06/202	0	1
27/05/202	31/05/202	18/05/202	0	1
20/04/202	03/05/202	15/04/202	0	1
22/03/202	22/03/202	23/07/202	0	1
07/05/202	24/05/202	07/05/202	0	1
29/05/202	03/06/202	26/05/202	0	1
14/08/202	01/09/202	10/08/202	0	1
04/02/202	01/09/202	22/01/202	0	
14/05/202	03/06/202	14/04/202	0	1
05/08/202	05/08/202	07/02/202	0	
15/02/202	28/03/202	01/02/202	0	1
04/03/202	15/03/202	02/03/202	0	
30/03/202	31/03/202	22/03/202	0	
09/04/202	09/04/202	03/05/202	0	
10/06/202	02/07/202	07/06/202	0	1

07/05/202	11/05/202	14/04/202	0	
02/04/202	23/04/202	23/04/202	0	1
15/07/202	21/07/202	21/09/202	0	1
17/08/202	18/08/202	11/08/202	0	
08/04/202	12/04/202	03/04/202	0	1
08/03/202	12/03/202	12/03/202	0	
11/09/202	28/10/202	01/07/202	0	1
20/04/202	06/05/202	08/04/202	0	1
22/03/202	29/03/202	19/03/202	0	1
18/04/202	19/04/202	17/03/202	0	1
09/04/202	09/04/202	09/04/202	0	1
20/07/202	22/07/202	08/07/202	0	1
25/04/202	26/04/202	26/04/202	0	1
31/07/202	02/08/202	24/06/202	0	1
03/09/202	03/09/202	04/08/202	0	
25/05/202	01/06/202	18/05/202	0	1
30/04/2021		30/07/202	0	1
01/07/202	02/07/202	24/06/202	0	
14/06/202	22/08/202	27/05/202	0	1
15/04/202	15/04/202	25/06/202	0	
05/06/202	23/08/202	23/08/202	0	
26/07/202	03/08/202	13/07/202	0	1
27/08/202	30/08/202	30/08/202	0	1
07/07/202	08/07/202	31/05/202	0	
15/05/202	02/06/202	02/06/202	0	
11/06/202	14/06/202	09/06/202	0	
15/03/202	17/03/202	11/03/202	2	
12/04/202	09/06/202	09/06/202	0	1
30/03/202	14/04/202	14/04/202	0	
16/05/202	16/05/202	06/05/202	0	
28/04/202	07/02/202	05/05/202	0	
02/06/202	02/06/202	12/05/202	0	1
16/06/202	21/06/202	26/05/202	0	1
06/07/202	06/07/202	06/07/202	0	
19/06/202	10/08/202	09/06/202	0	1
27/04/202	30/04/202	19/04/202	0	1
31/05/202	02/06/202	03/06/202	0	
05/06/202	07/06/202	19/04/202	0	1
06/08/202	22/08/202	29/07/202	0	1
26/02/202	26/02/202	24/02/202	0	1
01/03/202	23/04/202	25/02/202	0	
14/02/202	14/02/202	07/07/202	0	1
02/06/202	02/06/202	21/07/202	0	
24/02/202	02/03/202	19/02/202	0	
	16/09/202	01/03/202	0	1
14/04/202	16/04/202	26/03/202	0	
26/04/202	27/04/202	08/04/202	0	

28/01/202	29/01/202	25/01/202	0	
09/06/202	11/06/202	06/06/202	0	
06/07/202	14/07/202	23/06/202	0	
27/02/202	03/03/202	03/03/202	2	1
26/04/202	24/03/202	23/04/202	0	
02/05/202	04/05/202	30/04/202	0	
25/09/202	27/09/202	13/09/202	0	
21/06/202	01/07/202	07/06/202	0	1
11/04/202	11/04/202	15/04/202	0	1
11/05/202	14/05/202	14/05/202	0	1
30/07/202	06/08/202	11/07/202	0	1
14/06/202	27/09/202	20/05/202	0	
16/02/202	18/02/202	02/02/202	0	1
16/09/202	16/09/202	10/09/202	0	1
20/05/202	08/07/202	11/06/202	0	1
20/06/202	22/06/202	12/06/202	0	1
29/07/202	06/08/202	17/06/202	0	1
07/07/202	23/07/202	23/07/202	0	1
03/03/202	04/03/202	24/02/202	2	1
19/09/202	18/10/202	18/10/202	0	1
01/03/202	17/05/202	17/05/202	0	
15/07/202	21/07/202	04/06/202	0	
12/09/202	18/09/202	09/09/202	0	1
14/09/202	28/04/202	27/04/202	0	1
05/05/202	07/05/202	10/05/202	0	1
07/09/202	13/09/202	30/08/202	0	1
		18/05/202	0	
26/07/202	27/07/202	27/07/202	0	
23/09/202	28/09/202	01/09/202	0	1
27/07/202	30/07/202	26/05/202	0	1
28/06/202	28/06/202	22/06/202	0	1
02/12/202	03/12/202	03/12/202	0	
01/04/202	05/04/202	18/03/202	0	
	07/04/202	07/04/202	0	1
28/05/202	20/07/202	16/05/202	0	1
29/03/202	06/04/202	06/04/202	0	1
12/05/202	12/05/202	12/05/202	0	
30/05/202	17/06/202	17/06/202	0	1
08/06/202	08/06/202	04/06/202	0	1
31/07/202	02/08/202	12/07/202	0	1
07/06/202	11/06/202	03/05/202	0	1
25/05/202	10/06/202	19/04/202	0	1
25/06/202	04/08/202	04/08/202	0	1
04/05/202	07/05/202	30/04/202	0	
18/07/202	19/07/202	06/07/202	0	
08/07/202	23/09/202	01/07/202	0	1
07/09/202	08/09/202	08/09/202	0	1

21/04/202	22/04/202	29/03/202	0	1
26/04/202	13/05/202	08/04/202	0	1
04/06/202	20/09/202	05/11/202	0	
28/02/202	28/02/202	09/02/202	0	
13/04/202	11/05/202	11/05/202	0	1
15/06/202	28/04/202	28/04/202	0	1
05/06/202	08/06/202	12/04/202	0	1
19/05/202	20/05/202	04/05/202	0	1
28/04/202	22/04/202	14/04/202	0	1
28/06/202	17/06/202	09/06/202	0	1
26/06/202	24/02/202	04/06/202	0	1
16/07/202	07/12/202	02/06/202	0	
10/04/202	10/04/202	22/04/202	0	1
25/06/202	25/06/202	22/07/202	0	1
24/06/202	25/06/202	01/06/202	0	1
18/05/202	18/05/202	30/06/202	0	1
23/05/202	22/07/202	15/05/202	0	1
19/01/202	20/01/202	20/01/202	2	
13/07/202	25/08/202	12/07/202	0	
04/06/202	07/06/202	10/08/202	0	1
15/04/202	19/04/202	12/04/202	0	1
13/04/202	14/04/202	14/04/202	0	1
25/05/202	25/05/202	29/06/202	0	
03/06/202	28/07/202	25/05/202	0	
19/06/202	19/06/202	14/06/202	0	1
20/04/202	26/04/202	30/03/202	0	1
07/03/202	15/03/202	15/03/202	9	1
17/05/202	17/05/202	13/05/202	0	
14/06/202	02/07/202	08/06/202	0	1
26/04/202	27/04/202	16/04/202	0	1
23/05/202	02/09/202	18/05/202	0	
22/04/202	23/04/202	26/04/202	0	
30/09/202	30/09/202	21/09/202	0	
26/03/202	26/03/202	21/05/202	0	
01/07/202	23/07/202	21/05/202	0	
16/06/202	21/06/202	07/06/202	0	1
09/10/202	09/10/202	27/09/202	0	
18/04/202	25/03/202	19/03/202	0	1
26/08/202	29/09/202	29/09/202	0	1
21/03/202	17/04/202	12/03/202	0	1
26/04/202	26/04/202	12/04/202	0	1
	18/01/202	15/01/202	0	1
12/05/202	04/02/202	04/05/202	0	
16/06/202	08/09/202	14/06/202	0	1
07/06/202	08/06/202	21/05/202	0	1
29/11/202	30/11/202	23/11/202	0	1
10/07/202	07/07/202	07/07/202	0	1

17/05/202	22/10/202	22/11/202	0	
25/08/202	01/09/202	10/08/202	0	
08/07/202	09/07/202	02/06/202	0	1
	29/11/202	29/11/202	0	
01/09/202	02/09/202	24/08/202	0	
24/02/202	05/06/202	25/03/202	0	
08/03/202	08/03/202	24/02/202	0	1
10/08/202	17/11/202	27/07/202	0	1
28/03/202	26/04/202	09/03/202	0	1
	28/10/202	28/10/202	0	1
15/10/202	18/10/202	04/10/202	0	1
27/04/202	29/04/202	15/04/202	0	1
03/06/202	04/06/202	04/06/202	0	
29/01/202	29/01/202	28/01/202	9	1
11/06/202	14/06/202	07/05/202	0	1
02/06/202	03/06/202	26/05/202	0	
14/04/202	15/04/202	08/04/202	0	1
12/07/202	27/08/202	18/06/202	0	
02/06/202	29/06/202	30/06/202	0	1
03/05/202	03/05/202	24/06/202	0	
28/05/202	02/06/202	15/05/202	0	
01/05/202	31/05/202	18/04/202	0	1
13/07/202	13/07/202	30/07/202	0	
19/06/202	19/06/202	02/06/202	0	1
05/09/202	17/09/202	24/08/202	0	1
24/09/202	27/09/202	21/09/202	0	1
24/05/202	21/12/202	21/12/202	0	
02/06/202	03/06/202	17/05/202	0	1
01/07/202	15/07/202	28/06/202	0	
25/06/202	12/07/202	15/06/202	0	
30/01/202	31/01/202	19/01/202	2	
16/03/202	12/05/202	01/03/202	0	
20/03/202	21/03/202	11/03/202	0	1
06/04/202	12/04/202	29/03/202	0	1
27/05/202	12/11/202	11/05/202	0	
30/05/202	01/06/202	21/05/202	0	1
12/07/202	12/07/202	30/07/202	0	
26/06/202	21/07/202	28/05/202	0	1
07/05/202	10/05/202	12/05/202	0	1
20/08/202	21/10/202	25/08/202	0	1
21/08/202	23/08/202	16/08/202	0	1
20/07/202	03/08/202	03/08/202	0	
15/03/202	18/03/202	12/03/202	0	1
19/05/202	20/05/202	20/05/202	0	
27/03/202	14/06/202	18/03/202	0	1
02/04/202	06/04/202	20/03/202	0	1
07/05/202	07/05/202	29/04/202	0	

10/05/202	11/05/202	04/05/202	0	
19/06/202	22/06/202	22/06/202	0	1
02/04/202	06/04/202	29/03/202	0	
17/06/202	17/06/202	17/06/202	0	1
16/09/202	05/10/202	05/10/202	0	
30/07/202	31/07/202	31/07/202	0	
	19/11/202	19/11/202	0	1
27/05/202	17/08/202	17/08/202	0	
	09/03/202	01/12/202	0	1
15/10/202	15/10/202	02/10/202	0	1
17/04/202	08/06/202	20/04/202	0	
19/04/202	03/08/202	16/04/202	0	1
11/05/202	12/05/202	12/05/202	0	
26/04/202	17/04/202	29/03/202	0	1
18/06/202	18/06/202	05/06/202	0	
23/03/202	25/03/202	11/03/202	0	1
01/07/202	03/07/202	28/06/202	0	1
15/05/202	16/05/202	16/05/202	0	
14/06/202	15/06/202	15/06/202	0	1
06/07/202	08/07/202	04/06/202	0	1
29/05/202	29/05/202	20/06/202	0	
12/01/202	22/02/202	13/01/202	2	1
14/03/202	16/03/202	16/03/202	2	1
01/03/202	03/03/202	03/03/202	2	1
04/03/202	12/03/202	04/03/202	2	
29/03/202	17/05/202	16/03/202	0	1
29/03/202	23/04/202	23/04/202	0	1
01/05/202	04/05/202	28/04/202	0	
18/05/202	19/05/202	20/05/202	0	
20/05/202	10/06/202	29/04/202	0	
23/04/202	29/05/202	28/05/202	0	1
28/10/202	05/11/202	27/10/202	0	
13/07/202	13/07/202	07/07/202	0	1
19/05/202	19/05/202	28/06/202	0	
19/04/202	18/05/202	31/03/202	0	
29/03/202	05/04/202	05/04/202	0	1
20/04/202	20/04/202	04/06/202	0	1
06/03/202	10/03/202	10/03/202	0	1
26/06/202	06/07/202	18/06/202	0	
21/07/202	03/08/202	03/08/202	0	
31/05/202	11/06/202	28/05/202	0	1
23/09/202	25/10/202	29/10/202	0	1
12/01/202	19/01/202	19/01/202	0	1
19/03/202	20/03/202	09/03/202	0	
02/04/202	03/04/202	22/03/202	0	1
03/04/202	05/04/202	31/03/202	0	1
29/09/202	22/11/202	26/11/202	0	

26/05/202	27/05/202	27/05/202	0	1
14/05/202	14/05/202	12/05/202	0	1
02/08/202	10/09/202	18/06/202	0	1
02/04/202	27/07/202	29/03/202	0	
28/05/202	02/06/202	18/05/202	0	1
27/05/202	07/06/202	24/05/202	0	1
06/05/202	28/09/202	17/05/202	0	
05/04/202	06/04/202	06/04/202	0	
20/01/202	22/01/202	15/01/202	2	1
	19/04/202	19/04/202	0	1
17/01/202	27/01/202	27/01/202	0	1
09/06/202	19/06/202	02/06/202	0	1
19/08/202	17/11/202	15/06/202	0	1
24/04/202	27/04/202	16/04/202	0	1
23/03/202	25/03/202	19/03/202	0	1
23/07/202	28/07/202	16/07/202	0	1
12/07/202	12/07/202	01/06/202	0	1
19/04/202	24/02/202	14/04/202	0	
30/05/202	29/06/202	24/05/202	0	
15/06/202	14/06/202	14/06/202	0	1
18/08/202	25/08/202	31/08/202	0	
21/02/202	26/02/202	09/02/202	2	1
30/12/202	02/02/202	30/12/202	0	1
11/03/202	24/03/202	08/03/202	0	1
07/04/202	07/04/202	25/03/202	0	
06/04/202	08/04/202	29/03/202	0	1
31/05/202	01/06/202	21/06/202	0	1
11/03/202	16/04/202	16/04/202	0	
16/04/202	17/04/202	14/04/202	0	1
10/06/202	10/06/202	20/04/202	0	
28/03/202	12/11/202	27/03/202	0	
19/09/202	20/09/202	28/08/202	0	
02/04/202	06/04/202	30/03/202	0	1
18/06/202	30/06/202	02/06/202	0	1
04/08/202	06/08/202	08/07/202	0	1
01/02/202	01/02/202	22/01/202	2	1
21/06/202	22/06/202	19/06/202	0	1
07/08/202	07/08/202	26/07/202	0	1
14/04/202	19/04/202	05/04/202	0	
09/04/202	28/05/202	06/04/202	0	
14/06/202	14/06/202	29/05/202	0	1
09/04/202	15/04/202	09/03/202	0	1
26/07/202	03/08/202	29/06/202	0	
01/07/202	17/07/202	14/06/202	0	1
01/04/202	27/08/202	22/04/202	0	1
16/05/202	30/07/202	03/05/202	0	
17/05/202	21/06/202	07/05/202	0	1

09/09/202	21/09/202	06/09/202	0	
12/08/202	23/09/202	23/09/202	0	
22/01/202	22/01/202	12/01/202	2	1
09/03/202	10/03/202	15/02/202	2	1
22/02/202	22/02/202	17/02/202	0	1
15/03/202	15/03/202	24/11/202	0	1
15/03/202	07/04/202	07/04/202	0	
08/04/202	09/04/202	01/04/202	0	
02/04/202	05/04/202	06/04/202	0	1
24/04/202	29/04/202	29/04/202	0	1
20/07/202	29/07/202	08/07/202	0	1
02/06/202	02/06/202	27/05/202	0	
19/07/202	11/08/202	12/08/202	0	
25/06/202	29/06/202	29/06/202	0	1
15/08/202	31/08/202	11/08/202	0	1
16/03/202	16/03/202	19/03/202	0	1
10/10/202	13/10/202	06/10/202	0	
29/04/202	22/06/202	07/05/202	0	1
23/03/202	07/05/202	11/03/202	0	
29/05/202	22/06/202	20/05/202	0	1
13/06/202	22/06/202	11/06/202	0	1
10/06/202	11/06/202	05/06/202	0	1
02/08/202	04/08/202	12/07/202	0	1
03/04/202	26/08/202	16/04/202	0	1
15/06/202	23/09/202	10/06/202	0	1
09/06/202	05/09/202	05/09/202	0	1
18/04/202	18/04/202	22/04/202	0	1
13/03/202	12/02/202	11/02/202	0	1
28/10/202	29/10/202	27/10/202	0	
07/02/202	07/02/202	14/01/202	0	1
07/05/202	02/06/202	27/04/202	0	1
31/03/202	05/04/202	24/03/202	0	
26/03/202	15/06/202	26/04/202	0	1
10/05/202	11/05/202	26/03/202	0	1
22/10/202	25/10/202	20/08/202	0	
20/04/202	26/08/202	14/04/202	0	1
26/04/202	26/04/202	19/04/202	0	
14/08/202	16/08/202	11/08/202	0	
26/03/202	27/03/202	28/04/202	0	1
28/01/202	17/02/202	17/02/202	0	
15/03/202	17/03/202	23/02/202	2	1
05/01/202	24/03/202	06/01/202	0	1
30/09/202	22/10/202	22/10/202	0	
29/03/202	30/03/202	22/03/202	0	1
03/06/202	07/06/202	20/05/202	0	1
06/05/202	18/05/202	16/04/202	0	1
11/04/202	14/04/202	10/02/202	0	

17/05/202	18/05/202	21/05/202	0	
	18/06/202	18/06/202	0	1
13/08/202	13/08/202	13/08/202	0	
02/09/202	14/10/202	27/08/202	0	1
02/04/202	04/04/202	22/03/202	0	1
28/06/202	01/07/202	01/07/202	0	1
04/12/202	28/12/202	13/10/202	0	1
13/05/202	13/05/202	07/04/202	0	1
15/02/202	19/02/202	18/02/202	2	1
18/04/202	18/04/202	30/08/202	0	
30/04/202	04/05/202	23/04/202	0	1
30/03/202	01/04/202	15/03/202	0	1
11/01/202	14/01/202	17/12/202	0	1
20/05/202	20/05/202	03/05/202	0	1
14/06/202	14/06/202	25/05/202	0	
04/06/202	04/06/202	27/05/202	0	
11/04/202	15/04/202	30/03/202	0	1
	01/02/202	01/02/202	9	1
29/07/202	29/07/202	12/04/202	0	1
19/08/202	24/08/202	14/06/202	0	1
30/05/202	30/05/202	24/05/202	0	1
24/07/202	24/07/202	26/07/202	0	
03/04/202	19/03/202	22/03/202	0	1
29/03/202	16/04/202	16/04/202	0	1
06/06/202	07/06/202	04/06/202	0	1
17/06/202	18/06/202	17/06/202	0	
21/05/202	21/05/202	21/05/202	0	
29/09/202	30/09/202	11/08/202	0	1
17/02/202	14/06/202	17/02/202	0	1
18/05/202	18/05/202	18/05/202	0	
21/06/202	21/06/202	14/06/202	0	
03/09/202	03/09/202	11/06/202	0	1
24/05/202	25/05/202	05/05/202	0	
20/06/202	21/06/202	10/06/202	0	1
26/02/202	26/02/202	28/02/202	2	1
09/03/202	22/02/202	24/03/202	0	
27/09/202	09/11/202	24/09/202	0	1
20/05/202	21/05/202	05/04/202	0	
15/05/202	07/06/202	10/08/202	0	1
10/09/202	15/09/202	17/09/202	0	
05/10/202	23/11/202	30/09/202	0	1
23/08/202	29/08/202	28/06/202	0	1
24/06/202	27/09/202	09/06/202	0	1
15/08/202	09/09/202	12/08/202	0	1
07/05/202	05/07/202	16/03/202	0	1
09/02/202	02/07/202	11/02/202	0	
21/09/202	29/09/202	02/09/202	0	

13/03/202	13/03/202	12/03/202	0	1
09/05/202	10/05/202	04/05/202	0	1
19/10/202	19/10/202	28/09/202	0	1
10/05/202	10/05/202	30/06/202	0	
29/06/202	23/07/202	02/06/202	0	1
26/05/202	02/07/202	28/05/202	0	1
29/07/202	30/07/202	30/07/202	0	1
29/06/202	02/07/202	14/06/202	0	1
15/04/202	20/04/202	08/04/202	0	1
16/07/202	27/12/202	12/07/202	0	
14/05/202	14/05/202	02/05/202	0	1
02/12/202	03/12/202	30/11/202	0	1
05/08/202	06/08/202	25/07/202	0	
22/02/202	07/05/202	26/01/202	0	
12/11/202	18/11/202	22/10/202	0	
24/03/202	09/04/202	09/04/202	0	
14/05/202	14/05/202	06/05/202	0	
30/03/202	30/03/202	11/03/202	0	1
27/05/202	18/06/202	19/05/202	0	1
25/03/202	01/04/202	19/03/202	0	
07/06/202	07/06/202	07/06/202	0	
26/04/202	28/04/202	09/04/202	0	1
16/04/202	07/04/202	07/04/202	0	1
12/06/202	29/06/202	27/05/202	0	1
04/04/202	06/04/202	06/04/202	0	1
30/03/202	30/03/202	25/03/202	0	
02/04/202	26/03/202	26/03/202	0	1
17/03/202	23/03/202	25/02/202	0	1
22/03/202	22/03/202	22/03/202	0	1
05/06/202	07/06/202	27/05/202	0	
14/08/202	19/08/202	29/07/202	0	1
24/06/202	24/06/202	19/04/202	0	
05/04/202	07/04/202	31/03/202	0	1
11/05/202	11/05/202	19/04/202	0	
28/04/202	30/04/202	13/04/202	0	1
11/04/202	26/04/202	10/04/202	0	1
10/02/202	13/02/202	13/02/202	2	
03/04/202	06/05/202	16/03/202	0	1
27/09/202	27/09/202	24/09/202	0	
04/07/202	12/07/202	12/08/202	0	
06/05/202	19/05/202	26/04/202	0	1
06/07/202	07/07/202	16/06/202	0	
23/07/202	23/07/202	19/07/202	0	
04/02/202	06/02/202	26/01/202	2	1
03/01/202	08/01/202	03/01/202	0	1
13/04/202	16/04/202	05/04/202	0	1
22/04/202	28/04/202	14/04/202	0	

29/06/202	15/07/202	16/06/202	0	1
08/04/202	09/04/202	12/03/202	0	1
13/06/202	14/06/202	26/05/202	0	1
03/02/202	08/03/202	08/03/202	0	
20/07/202	22/07/202	14/06/202	0	1
29/11/202	29/11/202	01/12/202	0	1
02/06/202	09/06/202	20/05/202	0	1
23/03/202	19/04/202	24/04/202	0	1
15/04/202	12/04/202	12/04/202	0	1
07/07/202	04/08/202	06/07/202	0	1
30/03/202	18/05/202	18/05/202	0	
25/06/202	28/06/202	15/06/202	0	1
14/03/202	15/03/202	09/03/202	0	1
23/03/202	23/03/202	25/03/202	0	1
24/05/202	24/05/202	25/05/202	0	
05/07/202	04/09/202	04/09/202	0	
01/11/202	10/11/202	02/06/202	0	1
12/08/202	24/08/202	19/07/202	0	
23/03/202	23/04/202	23/04/202	0	1
09/06/202	24/06/202	24/06/202	0	1
28/05/202	29/05/202	21/05/202	0	
13/07/202	12/06/202	09/06/202	0	1
28/01/202	28/01/202	07/07/202	0	1
31/03/202	31/03/202	24/03/202	0	
26/04/202	27/04/202	16/04/202	0	1
21/05/202	27/05/202	11/05/202	0	1
30/06/202	01/07/202	21/06/202	0	
11/07/202	07/07/202	07/07/202	0	1
12/01/202	17/05/202	13/01/202	0	1
19/03/202	25/03/202	23/02/202	0	1
16/05/202	18/05/202	12/05/202	0	1
21/05/202	21/05/202	21/05/202	0	1
12/05/202	26/05/202	26/05/202	0	1
12/07/202	12/07/202	21/06/202	0	1
17/07/202	15/12/202	12/07/202	0	1
18/07/202	19/07/202	15/07/202	0	1
06/08/202	18/08/202	26/07/202	0	1
01/10/202	01/10/202	11/09/202	0	
05/07/202	30/08/202	21/08/202	0	
30/03/2021		26/03/202	0	1
21/05/202	21/05/202	21/05/202	0	1
13/06/202	24/06/202	07/06/202	0	1
19/07/202	19/07/202	30/06/202	0	
18/06/202	02/07/202	08/06/202	0	1
21/06/202	23/06/202	23/06/202	0	
22/04/202	06/09/202	30/07/202	0	
01/08/202	03/08/202	04/08/202	0	

23/04/202	23/04/202	29/09/202	0	
02/04/202	02/04/202	19/03/202	0	1
26/03/2021		03/03/202	0	1
05/03/202	26/07/202	04/03/202	0	1
02/05/202	02/05/202	28/04/202	0	
10/05/202	10/05/202	07/05/202	0	1
04/11/202	04/11/202	13/10/202	0	1
01/01/202	13/01/202	06/01/202	0	1
11/03/202	31/03/202	05/03/202	0	1
26/04/202	30/04/202	21/03/202	0	
24/04/202	26/04/202	29/03/202	0	
24/05/202	25/05/202	10/05/202	0	1
11/07/202	12/07/202	25/06/202	0	1
02/07/202	27/07/202	27/07/202	0	
14/06/202	14/06/202	08/06/202	0	1
30/08/202	31/08/202	29/07/202	0	
10/06/202	10/06/202	19/07/202	0	
14/09/202	17/09/202	13/09/202	0	1
06/12/202	08/12/202	02/12/202	0	
30/01/202	03/02/202	20/01/202	2	1
21/04/202	21/04/202	07/04/202	0	1
28/04/202	28/04/202	20/04/202	0	1
20/06/202	21/06/202	23/06/202	0	1
10/08/202	10/08/202	02/08/202	0	
06/04/202	30/04/202	30/03/202	0	1
14/04/202	14/12/202	05/04/202	0	1
04/05/202	05/05/202	03/05/202	0	1
11/05/202	07/06/202	06/05/202	0	1
11/04/202	04/05/202	04/05/202	0	1
31/05/202	31/05/202	17/05/202	0	1
28/04/202	03/09/202	03/09/202	0	
25/03/202	25/03/202	12/04/202	0	
12/04/202	14/04/202	14/04/202	0	1
09/06/202	09/06/202	10/05/202	0	1
18/05/202	24/08/202	19/05/202	0	1
07/07/202	18/06/202	18/06/202	0	1
07/04/202	07/04/202	16/04/202	0	1
31/05/202	14/06/202	12/05/202	0	
07/08/202	20/08/202	29/07/202	0	1
21/08/202	23/08/202	13/08/202	0	
30/03/202	30/03/202	25/03/202	0	1
24/03/202	25/03/202	17/03/202	0	
24/06/202	01/07/202	14/06/202	0	
02/09/202	03/09/202	30/08/202	0	1
20/08/202	03/09/202	04/08/202	0	1
30/03/202	08/04/202	25/03/202	0	1
14/04/202	15/04/202	23/03/202	0	1

13/04/202	28/04/202	28/04/202	0	
16/04/202	10/06/202	12/04/202	0	
22/03/202	16/04/202	16/04/202	0	1
14/06/202	14/06/202	12/06/202	0	
01/07/202	23/07/202	11/06/202	0	
07/08/202	10/08/202	28/06/202	0	1
29/03/202	13/11/202	13/09/202	0	1
07/10/202	25/10/202	21/10/202	0	1
23/03/202	29/03/202	24/04/202	0	1
17/05/202	19/05/202	19/05/202	0	1
25/06/202	25/06/202	18/06/202	0	1
23/07/202	05/09/202	05/09/202	0	1
04/11/202	05/11/202	25/10/202	0	
29/07/202	30/07/202	30/07/202	0	
04/05/202	04/05/202	30/04/202	0	
19/04/202	22/04/202	06/04/202	0	1
15/08/202	18/08/202	02/08/202	0	
05/04/202	02/08/202	29/03/202	0	1
10/06/202	11/06/202	28/05/202	0	1
21/05/202	21/05/202	01/06/202	0	
12/07/202	13/07/202	08/06/202	0	1
24/05/202	26/05/202	15/05/202	0	1
08/03/202	26/04/202	04/03/202	0	
25/03/202	25/06/202	18/03/202	0	1
09/05/202	19/05/202	19/05/202	0	1
21/06/202	31/07/202	09/06/202	0	
29/06/202	13/07/202	07/06/202	0	1
06/06/202	16/06/202	15/06/202	0	1
17/04/202	20/04/202	20/04/202	0	
25/06/202	28/06/202	12/05/202	0	
07/08/202	07/08/202	14/07/202	0	1
20/03/202	19/04/202	15/03/202	0	1
22/04/202	20/05/202	15/04/202	0	
04/06/202	08/06/202	27/05/202	0	1
14/06/202	07/07/202	07/07/202	0	1
18/07/202	20/07/202	03/07/202	0	
03/08/202	26/08/202	26/08/202	0	1
03/03/202	12/05/202	23/02/202	0	1
10/07/202	06/10/202	06/10/202	0	
18/06/202	22/05/202	22/05/202	0	1
28/04/202	28/04/202	04/04/202	0	1
23/05/202	24/05/202	29/04/202	0	1
13/10/202	27/10/202	04/11/202	0	
08/07/202	12/07/202	25/06/202	0	1
20/05/202	03/12/202	12/05/202	0	1
	27/07/202	27/07/202	0	1
21/03/202	21/03/202	27/02/202	2	1

07/04/202	16/11/202	26/03/202	0	1
10/03/202	12/05/202	26/03/202	0	1
02/07/202	12/07/202	17/06/202	0	1
12/05/202	27/08/202	28/04/202	0	1
22/06/202	01/07/202	06/07/202	0	1
16/06/202	16/06/202	12/05/202	0	
29/07/202	09/08/202	23/07/202	0	
17/04/202	08/05/202	04/04/202	0	1
08/04/202	09/04/202	01/04/202	0	
06/05/202	07/04/202	07/04/202	0	1
03/05/202	04/05/202	04/05/202	0	1
08/05/202	12/05/202	10/06/202	0	1
22/04/202	12/05/202	26/04/202	0	
10/08/202	18/08/202	22/07/202	0	
29/05/202	31/05/202	27/05/202	0	1
10/08/202	11/08/202	03/08/202	0	1
16/06/202	02/06/202	02/06/202	0	1
10/05/202	07/05/202	07/05/202	0	1
19/06/202	02/07/202	17/06/202	0	1
18/06/202	18/06/202	21/06/202	0	1
02/07/202	02/07/202	19/07/202	0	
04/04/202	06/04/202	16/03/202	0	1
26/04/202	28/04/202	05/04/202	0	
15/05/202	21/05/202	15/04/202	0	1
02/07/202	05/07/202	31/05/202	0	1
10/06/202	11/06/202	02/06/202	0	
17/06/202	21/06/202	04/06/202	0	1
26/06/202	13/07/202	14/06/202	0	1
13/06/202	02/12/202	14/06/202	0	1
11/11/202	24/11/202	14/10/202	0	
16/06/202	05/07/202	05/07/202	0	1
27/05/202	27/05/202	04/05/202	0	
06/07/202	13/07/202	02/06/202	0	1
01/06/202	01/06/202	27/05/202	0	1
30/07/202	16/08/202	16/08/202	0	
30/07/202	10/09/202	03/08/202	0	1
04/08/202	14/08/202	05/06/202	0	1
20/07/202	20/07/202	03/08/202	0	
30/06/202	30/06/202	26/06/202	0	1
18/04/202	04/05/202	06/04/202	0	1
08/07/202	08/07/202	25/05/202	0	
29/06/202	30/06/202	15/06/202	0	1
02/08/202	02/08/202	09/07/202	0	
22/04/202	13/08/202	13/08/202	0	1
01/06/202	02/06/202	19/04/202	0	1
13/05/202	13/05/202	19/05/202	0	
26/04/202	27/04/202	23/04/202	0	1

12/05/202	29/06/202	29/06/202	0	1
08/07/202	09/07/202	06/07/202	0	1
25/07/202	26/07/202	26/07/202	0	1
20/03/202	24/03/202	12/03/202	0	1
07/03/202	19/11/202	03/03/202	0	1
25/02/202	11/03/202	05/03/202	2	1
23/04/202	16/04/202	16/04/202	0	1
26/04/202	26/04/202	22/04/202	0	1
02/06/202	02/06/202	25/05/202	0	
02/05/202	18/05/202	18/05/202	0	
22/05/202	22/05/202	28/06/202	0	
07/09/202	08/09/202	23/08/202	0	
06/05/202	07/05/202	16/03/202	0	
02/06/202	02/06/202	05/04/202	0	1
	05/07/202	16/06/202	0	
02/05/202	01/10/202	12/04/202	0	
19/05/202	20/05/202	20/05/202	0	1
06/06/202	15/10/202	31/05/202	0	1
30/06/202	30/06/202	08/06/202	0	
05/06/202	07/06/202	26/05/202	0	1
13/06/202	18/06/202	21/06/202	0	1
12/06/202	18/08/202	27/09/202	0	
06/03/202	07/03/202	09/02/202	2	1
13/02/202	15/02/202	15/02/202	9	1
12/04/202	13/04/202	05/04/202	0	1
12/06/202	15/06/202	02/06/202	0	1
02/04/202	22/06/202	07/03/202	0	1
28/09/202	29/09/202	05/08/202	0	
19/08/202	30/08/202	19/08/202	0	1
25/09/202	25/09/202	23/09/202	0	1
27/04/202	27/04/202	11/03/202	0	
15/05/202	02/06/202	07/05/202	0	1
13/06/202	17/06/202	17/06/202	0	1
29/06/202	04/08/202	04/08/202	0	
24/07/202	24/08/202	24/08/202	0	1
22/02/202	01/03/202	22/02/202	0	1
26/04/202	12/05/202	07/04/202	0	1
18/05/202	19/05/202	11/05/202	0	1
18/07/202	19/07/202	08/07/202	0	1
22/07/202	22/07/202	23/07/202	0	1
13/07/202	19/07/202	31/05/202	0	1
24/06/202	24/06/202	26/07/202	0	1
30/06/202	11/07/202	22/07/202	0	
23/05/202	05/09/202	05/09/202	0	1
12/03/202	16/03/202	10/03/202	2	
29/03/202	30/03/202	06/04/202	0	
25/04/202	25/04/202	13/04/202	0	1

08/07/202	03/09/202	22/06/202	0	
14/12/202	17/12/202	08/12/202	0	
01/05/202	31/08/202	30/04/202	0	1
31/03/202	06/04/202	31/03/202	0	1
29/03/202	01/04/202	01/03/202	0	1
02/06/202	03/06/202	26/05/202	0	1
02/05/202	10/05/202	28/04/202	0	1
02/05/202	02/05/202	11/05/202	0	1
10/06/202	18/06/202	18/06/202	0	
26/07/202	26/07/202	08/07/202	0	
08/05/202	21/06/202	23/04/202	0	1
19/03/202	22/03/202	23/03/202	0	1
26/08/202	26/08/202	02/08/202	0	
14/07/202	14/07/202	07/07/202	0	
16/02/202	28/03/202	11/02/202	0	
21/10/202	10/11/202	19/10/202	0	
04/05/202	08/06/202	20/04/202	0	
10/05/202	10/05/202	12/05/202	0	
26/05/202	27/05/202	25/05/202	0	
28/05/202	16/06/202	17/05/202	0	1
21/06/202	21/06/202	26/05/202	0	1
22/06/202	25/06/202	11/06/202	0	1
15/10/202	29/10/202	28/09/202	0	
12/06/202	14/06/202	13/04/202	0	1
11/03/202	12/03/202	04/03/202	0	1
01/05/202	31/08/202	30/04/202	0	1
20/06/202	05/10/202	28/05/202	0	
23/06/202	23/06/202	21/06/202	0	1
29/06/202	29/06/202	01/06/202	0	1
24/03/2021		02/08/202	0	1
03/12/202	17/12/202	03/12/202	0	1
15/03/202	31/03/202	16/02/202	0	
10/03/202	08/04/202	03/03/202	0	1
20/04/202	20/04/202	18/03/202	0	1
20/03/202	25/03/202	12/03/202	0	1
13/04/202	16/04/202	16/04/202	0	
11/05/202	12/05/202	29/04/202	0	1
23/11/202	15/12/202	19/11/202	0	
28/03/202	07/04/202	07/04/202	0	
07/06/202	08/06/202	31/05/202	0	
14/08/202	16/08/202	01/08/202	0	
05/01/202	25/02/202	25/02/202	0	
06/07/202	08/07/202	24/06/202	0	1
23/01/202	23/01/202	14/01/202	0	
01/04/2021		18/03/202	0	1
25/03/202	12/07/202	16/04/202	0	
21/05/202	24/05/202	10/05/202	0	

17/06/202	18/06/202	18/06/202	0	1
09/05/202	10/05/202	04/05/202	0	
19/03/202	22/03/202	13/03/202	0	
18/04/202	18/04/202	16/04/202	0	1
01/06/202	04/06/202	10/05/202	0	
12/08/202	24/09/202	24/09/202	0	
05/06/202	18/06/202	18/06/202	0	1
		10/01/202	0	
21/03/202	30/04/202	15/03/202	0	1
06/03/202	06/03/202	25/01/202	2	1
02/06/202	03/06/202	03/05/202	0	1
28/03/202	05/04/202	22/03/202	0	1
06/04/202	09/04/202	01/04/202	0	1
18/04/202	18/06/202	18/06/202	0	
06/05/202	04/05/202	04/05/202	0	
09/09/202	02/02/202	16/08/202	0	1
05/02/202	08/02/202	11/01/202	2	1
17/03/202	17/03/202	28/04/202	0	
18/07/202	21/07/202	28/06/202	0	
16/03/202	11/08/202	08/03/202	0	1
18/03/202	18/03/202	15/03/202	2	1
03/04/202	05/04/202	28/04/202	0	
15/07/202	15/07/202	14/07/202	0	1
05/07/202	14/07/202	15/06/202	0	1
28/06/202	29/06/202	14/06/202	0	
16/08/202	16/08/202	16/08/202	0	1
13/06/202	06/07/202	21/05/202	0	1
29/01/202	06/02/202	26/01/202	0	1
12/06/202	18/06/202	08/06/202	0	1
28/07/202	23/07/202	23/07/202	0	1
	16/07/202	21/06/202	0	1
06/06/202	06/06/202	16/07/202	0	
22/11/202	23/11/202	18/11/202	0	
09/05/202	12/05/202	17/04/202	0	
12/05/202	31/05/202	14/04/202	0	1
01/07/202	01/07/202	17/06/202	0	1
23/02/202	25/02/202	17/02/202	0	1
20/03/202	21/03/202	04/03/202	0	1
01/03/202	17/03/202	17/03/202	2	1
10/04/202	10/04/202	06/04/202	0	1
18/07/202	18/07/202	13/07/202	0	
08/08/202	10/08/202	09/08/202	0	
14/01/202	15/01/202	14/01/202	2	1
03/02/202	03/02/202	03/02/202	0	1
15/02/202	17/02/202	08/02/202	2	1
15/02/202	14/04/202	08/02/202	0	1
02/05/202	02/05/202	19/04/202	0	1

12/05/202	18/05/202	19/04/202	0	1
12/07/202	14/07/202	16/06/202	0	
25/06/202	28/06/202	16/07/202	0	1
03/04/202	03/04/202	02/04/202	0	1
29/05/202	07/06/202	26/05/202	0	
06/06/202	14/06/202	18/06/202	0	
21/10/202	22/10/202	25/08/202	0	1
05/02/202	15/03/202	08/02/202	9	1
14/04/202	14/04/202	05/04/202	0	1
04/06/202	04/06/202	01/06/202	0	1
05/02/202	08/02/202	03/02/202	0	1
01/04/202	06/04/202	03/03/202	0	1
20/02/202	19/05/202	02/03/202	0	
05/04/202	27/08/202	22/04/202	0	1
05/08/202	06/08/202	11/06/202	0	1
18/06/202	02/07/202	11/06/202	0	1
30/07/202	02/08/202	19/07/202	0	1
27/08/202	06/09/202	23/08/202	0	1
19/01/202	20/01/202	13/01/202	0	1
15/03/202	15/03/202	12/03/202	0	1
24/03/202	13/05/202	18/03/202	0	1
01/04/202	30/04/202	29/03/202	0	1
01/05/202	04/05/202	03/05/202	0	
03/06/202	07/06/202	21/06/202	0	1
08/09/202	08/09/202	06/09/202	0	1
18/01/202	18/01/202	14/01/202	0	
14/05/202	14/05/202	16/03/202	0	1
12/04/202	15/04/202	06/04/202	0	1
31/05/202	02/07/202	30/04/202	0	1
08/12/202	23/12/202	20/10/202	0	
13/03/202	19/03/202	08/03/202	2	1
14/02/202	14/02/202	07/02/202	2	
28/06/202	30/06/202	08/06/202	0	
05/09/202	21/09/202	30/08/202	0	1
25/03/202	25/03/202	16/03/202	0	1
	08/03/202	04/05/202	0	
05/07/202	05/07/202	22/06/202	0	1
01/04/202	06/04/202	08/04/202	0	1
27/05/202	27/05/202	29/06/202	0	
17/08/202	18/08/202	12/08/202	0	1
25/06/202	28/06/202	04/06/202	0	1
16/07/202	26/08/202	24/06/202	0	
03/10/202	03/10/202	11/10/202	0	
16/04/202	16/04/202	06/03/202	0	1
18/01/202	20/01/202	20/01/202	2	
18/06/202	22/06/202	01/06/202	0	
06/04/202	22/04/202	22/04/202	0	1

08/06/202	11/05/202	18/05/202	0	1
26/04/202	26/04/202	07/04/202	0	
23/06/202	11/06/202	11/06/202	0	1
27/04/202	28/04/202	27/04/202	0	
20/07/202	22/07/202	06/07/202	0	1
29/05/202	13/07/202	22/05/202	0	1
05/02/202	20/02/202	05/02/202	2	
23/09/202	25/09/202	06/09/202	0	
09/07/202	10/07/202	08/05/202	0	1
15/07/202	15/07/202	15/07/202	0	1
22/06/202	22/06/202	18/06/202	0	1
21/06/2021		23/07/202	0	1
12/04/202	25/03/202	09/04/202	0	1
24/04/202	19/03/202	20/04/202	0	
27/03/202	05/04/202	24/04/202	0	1
28/05/202	05/07/202	05/07/202	0	
28/01/202	29/01/202	22/01/202	2	1
20/06/202	10/07/202	15/07/202	0	
18/08/202	19/08/202	09/08/202	0	
10/04/202	04/05/202	04/05/202	0	
20/03/202	07/04/202	05/03/202	0	
10/04/202	10/04/202	24/03/202	0	
29/04/202	14/05/202	14/05/202	0	1
25/02/202	15/07/202	15/07/202	0	1
22/02/202	24/02/202	17/02/202	0	1
13/04/202	13/05/202	06/04/202	0	
03/05/202	27/04/202	27/04/202	0	1
08/07/202	19/07/202	07/07/202	0	
05/08/202	10/08/202	12/07/202	0	1
19/06/202	19/06/202	27/09/202	0	
18/10/202	19/10/202	13/10/202	0	
12/01/202	05/03/202	08/01/202	0	1
16/06/202	06/07/202	24/08/202	0	
05/11/202	05/11/202	28/10/202	0	
24/03/202	27/03/202	25/03/202	0	1
19/04/202	22/04/202	19/04/202	0	1
05/10/202	06/10/202	11/05/202	0	1
12/07/202	03/08/202	14/06/202	0	
03/05/202	03/05/202	24/06/202	0	
26/03/202	07/05/202	19/03/202	0	
06/04/2021		06/04/202	0	1
07/04/202	27/04/202	27/04/202	0	1
29/07/202	03/02/202	19/07/202	0	
	10/07/202	10/06/202	0	
10/06/202	15/06/202	13/04/202	0	1
10/05/202	10/05/202	18/05/202	0	
11/06/202	14/06/202	30/05/202	0	

30/12/202	30/12/202	23/11/202	0	
16/07/202	19/07/202	14/06/202	0	1
10/05/202	12/05/202	28/04/202	0	
04/05/202	07/05/202	30/04/202	0	1
13/05/202	30/05/202	12/05/202	0	1
08/04/202	09/04/202	09/04/202	0	1
24/07/202	18/08/202	27/07/202	0	
21/01/202	11/03/202	08/02/202	2	
29/04/202	10/05/202	12/04/202	0	1
07/03/202	29/06/202	03/03/202	0	1
22/04/202	26/04/202	22/04/202	0	1
04/06/202	08/06/202	08/06/202	0	
25/08/202	03/08/202	03/08/202	0	1
11/03/202	19/03/202	23/02/202	0	1
08/04/202	09/04/202	01/04/202	0	1
02/06/202	04/06/202	25/05/202	0	
23/07/202	23/07/202	19/07/202	0	
13/05/202	01/06/202	05/06/202	0	1
17/05/202	20/05/202	15/04/202	0	1
	04/08/202	04/08/202	0	
02/08/2021		27/07/202	0	
27/08/202	27/08/202	06/09/202	0	
19/05/202	05/09/202	05/09/202	0	
27/10/202	27/10/202	19/10/202	0	
29/04/202	05/07/202	25/03/202	0	1
01/05/202	02/05/202	23/04/202	0	1
20/02/202	21/02/202	13/07/202	0	1
10/09/202	11/09/202	22/08/202	0	1
22/06/202	25/06/202	26/05/202	0	
03/09/202	04/09/202	27/08/202	0	
25/11/202	26/11/202	12/11/202	0	1
22/08/202	07/09/202	10/07/202	0	1
24/04/202	28/04/202	08/04/202	0	1
12/04/202	08/05/202	08/05/202	0	
19/07/202	23/07/202	22/06/202	0	1
30/07/202	05/08/202	21/07/202	0	1
21/11/202	22/11/202	19/11/202	0	
28/05/202	06/07/202	14/05/202	0	1
19/10/202	20/10/202	09/10/202	0	
	30/09/202	22/07/202	0	
19/08/202	20/08/202	17/08/202	0	1
25/03/202	29/03/202	10/03/202	0	1
19/01/202	19/01/202	03/01/202	0	1
10/04/202	12/04/202	01/04/202	0	
14/04/202	19/04/202	12/04/202	0	1
19/02/202	28/10/202	11/02/202	0	1
	25/02/202	12/02/202	0	1

27/04/202	04/02/202	05/05/202	0	1
24/06/202	25/06/202	07/06/202	0	1
14/04/202	15/04/202	12/04/202	0	1
09/05/202	09/05/202	02/05/202	0	
23/04/202	16/04/202	16/04/202	0	
03/06/202	15/06/202	15/06/202	0	1
02/07/202	30/07/202	31/05/202	0	1
18/06/202	23/06/202	23/06/202	0	1
31/07/202	22/09/202	26/07/202	0	
11/05/202	12/05/202	11/05/202	0	1
23/03/202	04/05/202	09/04/202	0	1
30/03/202	31/03/202	31/03/202	0	
07/04/202	07/04/202	08/04/202	0	1
23/05/202	11/04/202	11/04/202	0	1
08/06/202	14/06/202	01/06/202	0	1
01/06/202	01/06/202	24/05/202	0	1
19/07/202	22/07/202	15/07/202	0	1
09/03/202	09/03/202	20/07/202	0	1
29/04/202	22/04/202	22/04/202	0	
29/06/202	18/11/202	17/06/202	0	1
29/05/202	31/05/202	27/05/202	0	
13/04/202	13/04/202	23/08/202	0	
14/10/202	15/10/202	14/10/202	0	1
02/03/202	09/03/202	13/10/202	0	
26/04/202	29/04/202	19/04/202	0	1
20/05/202	20/05/202	26/04/202	0	1
09/05/202	22/05/202	11/05/202	0	
16/06/202	17/06/202	30/05/202	0	1
02/07/202	05/07/202	08/06/202	0	1
21/07/202	21/07/202	11/08/202	0	1
31/08/202	09/12/202	23/08/202	0	1
29/09/202	29/09/202	01/10/202	0	1
23/06/202	05/08/202	29/06/202	0	1
10/06/202	11/06/202	04/06/202	0	1
04/08/202	07/08/202	26/06/202	0	1
29/03/202	30/03/202	24/03/202	0	1
18/05/202	24/05/202	03/05/202	0	1
26/04/202	26/04/202	06/05/202	0	
03/07/202	03/07/202	08/06/202	0	
26/06/202	01/07/202	01/07/202	0	1
05/11/202	02/02/202	01/11/202	0	
25/05/202	31/05/202	12/05/202	0	1
29/05/202	31/05/202	19/05/202	0	1
03/06/202	22/06/202	22/06/202	0	1
12/11/202	16/11/202	21/11/202	0	1
21/05/202	21/05/202	24/05/202	0	
06/07/202	13/07/202	09/06/202	0	1

08/05/202	08/05/202	30/06/202	0	
24/09/202	24/09/202	20/09/202	0	
06/09/202	06/09/202	03/09/202	0	
21/08/202	24/08/202	12/07/202	0	1
09/03/202	09/03/202	05/03/202	2	1
	22/04/202	22/04/202	0	
21/07/202	21/07/202	08/06/202	0	1
06/07/202	07/07/202	04/06/202	0	1
		23/07/202	0	1
15/08/202	15/08/202	28/07/202	0	1
03/09/202	08/09/202	22/08/202	0	
	30/12/202	30/12/202	0	
23/02/202	24/02/202	17/02/202	2	1
29/05/202	07/06/202	07/05/202	0	1
15/01/202	23/08/202	24/08/202	0	1
18/02/202	18/02/202	22/02/202	2	1
15/05/202	17/05/202	21/05/202	0	
06/06/202	07/06/202	31/05/202	0	
02/04/202	06/10/202	03/03/202	0	
03/04/202	22/04/202	01/04/202	0	1
13/04/202	13/04/202	18/03/202	0	1
20/03/202	22/04/202	22/04/202	0	1
10/09/202	15/09/202	27/08/202	0	1
22/03/202	31/03/202	17/04/202	0	1
02/06/202	04/06/202	11/06/202	0	
21/08/202	02/02/202	17/08/202	0	1
22/06/202	23/06/202	02/06/202	0	1
23/08/202	24/08/202	16/07/202	0	
06/07/202	21/09/202	16/07/202	0	1
08/04/202	15/04/202	25/03/202	0	1
22/04/202	22/04/202	01/10/202	0	
03/05/202	03/05/202	18/03/202	0	
13/04/202	14/04/202	17/04/202	0	
04/06/202	07/06/202	24/05/202	0	1
17/06/202	30/06/202	16/06/202	0	1
20/07/202	21/07/202	21/07/202	0	1
18/09/202	18/10/202	15/09/202	0	
04/04/202	24/08/202	26/03/202	0	1
26/04/202	27/04/202	10/04/202	0	1
02/04/202	23/04/202	23/04/202	0	1
13/08/202	23/08/202	09/08/202	0	1
31/03/202	16/04/202	16/04/202	0	1
20/05/202	15/05/202	15/05/202	0	1
24/07/202	24/07/202	26/07/202	0	
12/01/202	19/01/202	12/01/202	2	1
27/05/202	28/05/202	14/05/202	0	1
14/07/202	02/08/202	02/08/202	0	

06/05/202	18/05/202	13/01/202	0	1
02/03/202	12/05/202	06/02/202	0	1
01/05/202	04/05/202	14/04/202	0	1
26/05/202	08/06/202	08/06/202	0	1
13/06/202	25/06/202	25/06/202	0	1
05/05/202	17/05/202	18/03/202	0	
17/03/202	19/03/202	18/03/202	2	1
14/04/202	16/04/202	24/03/202	0	
17/03/202	17/03/202	07/04/202	0	1
20/04/202	03/08/202	16/04/202	0	1
14/07/202	14/07/202	19/08/202	0	1
16/02/202	09/04/202	16/02/202	0	
17/03/202	17/03/202	26/02/202	0	1
07/04/202	12/04/202	19/03/202	0	1
05/04/202	07/04/202	24/03/202	0	1
23/12/202	15/01/202	15/01/202	0	1
08/12/202	13/02/202	02/12/202	0	1
23/03/202	25/03/202	18/03/202	0	1
10/05/202	12/05/202	26/04/202	0	1
25/06/202	21/09/202	01/09/202	0	1
23/02/202	04/05/202	22/02/202	0	
31/03/202	07/04/202	17/03/202	0	1
22/02/2021		17/05/202	0	1
15/06/202	16/06/202	31/05/202	0	1
24/09/202	24/09/202	19/07/202	0	1
31/08/202	24/09/202	24/09/202	0	1
17/03/202	31/03/202	03/03/202	0	1
27/03/202	12/04/202	12/04/202	0	
05/08/202	06/08/202	30/04/202	0	
06/06/202	09/06/202	27/05/202	0	1
26/07/202	30/07/202	30/07/202	0	1
26/05/202	26/05/202	07/07/202	0	
08/11/202	08/11/202	04/11/202	0	
30/04/202	02/05/202	25/04/202	0	1
27/06/202	28/06/202	14/05/202	0	1
28/07/202	30/07/202	25/06/202	0	1
08/01/202	22/02/202	02/03/202	0	
15/03/202	15/03/202	12/03/202	0	1
10/06/202	16/08/202	01/06/202	0	1
22/09/202	18/09/202	24/09/202	0	1
30/04/202	03/05/202	11/05/202	0	
28/06/202	16/09/202	01/07/202	0	1
02/12/202	06/12/202	23/09/202	0	1
23/08/202	14/12/202	14/12/202	0	1
09/06/202	09/06/202	29/05/202	0	1
05/11/202	08/11/202	01/11/202	0	1
07/03/202	07/03/202	09/03/202	2	1

21/04/202	30/04/202	05/04/202	0	
03/07/202	13/07/202	07/06/202	0	
31/05/202	08/06/202	08/06/202	0	
30/08/202	31/08/202	31/08/202	0	1
08/11/202	12/11/202	27/10/202	0	1
08/04/202	30/05/202	31/03/202	0	1
19/04/202	30/04/202	15/06/202	0	1
21/06/202	21/06/202	22/06/202	0	1
26/03/202	09/04/202	24/03/202	0	
15/04/202	15/04/202	11/04/202	0	1
19/05/2021		02/08/202	0	1
08/08/202	21/09/202	21/09/202	0	1
	23/04/202	28/04/202	0	
13/07/202	13/07/202	28/06/202	0	1
31/03/202	05/04/202	24/04/202	0	1
12/04/202	13/05/202	25/03/202	0	1
17/06/202	24/06/202	23/04/202	0	1
09/05/202	13/05/202	20/05/202	0	1
11/04/202	12/04/202	06/04/202	0	1
12/09/202	13/09/202	09/09/202	0	1
30/07/202	30/07/202	08/07/202	0	
13/04/202	14/04/202	29/03/202	0	
01/04/202	11/05/202	11/03/202	0	1
01/06/202	17/06/202	19/05/202	0	1
05/07/202	28/07/202	28/07/202	0	1
10/06/202	06/07/202	06/07/202	0	1
08/03/202	09/03/202	11/01/202	0	1
28/07/202	05/08/202	21/07/202	0	1
13/03/202	07/04/202	25/02/202	0	1
15/05/202	01/07/202	27/09/202	0	1
12/08/202	31/08/202	31/08/202	0	1
20/03/202	20/03/202	21/04/202	0	1
16/03/202	14/04/202	11/03/202	0	1
20/05/202	20/05/202	04/05/202	0	
28/05/202	28/05/202	28/05/202	0	1
19/06/202	17/08/202	09/06/202	0	1
07/03/202	27/03/202	24/02/202	0	1
06/05/202	07/05/202	14/04/202	0	
05/07/202	14/07/202	30/04/202	0	1
25/06/202	06/07/202	12/06/202	0	
29/06/202	30/06/202	29/06/202	0	
09/10/202	20/10/202	06/10/202	0	1
09/07/202	12/07/202	25/06/202	0	1
10/01/202	17/01/202	04/01/202	0	
15/01/202	02/02/202	17/01/202	0	1
19/02/202	19/02/202	03/02/202	0	
15/01/202	17/01/202	19/01/202	0	1

01/03/202	02/03/202	25/02/202	0	1
10/02/202	22/02/202	10/02/202	0	
27/01/202	28/01/202	24/01/202	0	
08/01/202	10/01/202	07/01/202	0	
22/01/202	24/01/202	22/01/202	0	
15/01/202	17/01/202	14/01/202	0	
29/01/202	30/01/202	23/01/202	0	
22/01/202	24/01/202	21/01/202	0	
04/03/202	11/03/202	09/02/202	0	
24/02/202	04/03/202	19/02/202	0	
09/01/202	10/01/202	10/01/202	0	
11/02/202	25/02/202	20/01/202	0	
20/01/202	04/02/202	18/01/202	0	1
23/01/202	24/01/202	21/01/202	0	
28/01/202	28/01/202	28/01/202	0	
31/01/202	07/02/202	01/02/202	0	
09/02/202	14/02/202	08/02/202	0	
08/03/202	08/03/202	08/02/202	0	
11/02/202	16/02/202	09/02/202	0	1
22/01/202	22/01/202	25/01/202	0	
29/01/202	14/02/202	14/02/202	0	
06/01/202	11/01/202	12/01/202	0	
17/01/202	18/01/202	17/01/202	0	
19/01/202	19/01/202	18/01/202	0	1
26/01/202	28/01/202	22/01/202	0	1
01/02/202	02/02/202	20/01/202	0	1
15/02/202	09/03/202	09/03/202	0	
18/01/202	19/01/202	17/01/202	0	
23/01/202	24/01/202	22/01/202	0	
19/02/202	19/02/202	08/02/202	0	1
27/01/202	28/01/202	27/01/202	0	
19/01/202	19/01/202	17/01/202	0	
25/02/202	11/03/202	11/03/202	0	
17/01/202	26/01/202	26/01/202	0	1
31/01/202	17/02/202	24/01/202	0	1
	17/03/202	06/01/202	0	1
19/01/202	20/01/202	19/01/202	0	1
11/01/202	12/01/202	07/01/202	0	
17/01/202	18/01/202	17/01/202	0	
26/02/202	27/02/202	25/02/202	0	
18/03/202	22/03/202	18/03/202	0	
23/01/202	25/02/202	25/02/202	0	1
21/01/202	22/01/202	20/01/202	0	
24/01/202	03/02/202	20/01/202	0	1
09/02/202	11/02/202	08/02/202	0	
24/01/202	01/02/202	18/02/202	0	1
17/01/202	24/01/202	24/01/202	0	1

19/01/202	19/01/202	19/01/202	0	
18/01/202	19/01/202	19/01/202	0	
27/01/202	10/03/202	21/03/202	0	
21/01/202	21/01/202	14/01/202	0	1
14/01/202	01/02/202	18/02/202	0	1
21/01/202	21/01/202	21/01/202	0	1
31/01/202	11/02/202	11/02/202	0	1
13/01/202	20/01/202	20/01/202	0	
22/01/202	12/02/202	20/01/202	0	
26/01/202	26/01/202	07/02/202	0	1
21/01/202	25/01/202	19/01/202	0	1
20/01/202	21/01/202	24/01/202	0	
31/01/202	06/03/202	06/03/202	0	
27/01/202	09/02/202	18/01/202	0	
	24/03/202	24/03/202	0	1
09/02/202	18/02/202	18/02/202	0	1
08/02/202	17/02/202	17/02/202	0	1
21/01/202	26/01/202	26/01/202	0	1
03/03/202	17/03/202	10/02/202	0	
29/01/202	31/01/202	20/01/202	0	
09/03/202	11/03/202	10/01/202	0	1
	24/01/202	27/01/202	0	
24/02/202	24/02/202	20/02/202	0	1
26/01/202	26/01/202	25/01/202	0	
24/01/202	25/01/202	23/01/202	0	
20/01/202	24/01/202	24/01/202	0	1
20/01/202	21/01/202	19/01/202	0	
16/01/202	17/01/202	13/01/202	0	
04/02/202	09/02/202	09/02/202	0	
11/01/202	11/01/202	18/02/202	0	
26/01/202	28/01/202	27/01/202	0	
18/01/202	19/01/202	19/01/202	0	1
07/02/2022		07/02/202	0	
08/03/202	09/03/202	14/02/202	0	1
01/02/202	23/03/202	04/03/202	0	1
20/01/202	11/02/202	11/02/202	0	1
23/01/202	12/03/202	29/01/202	0	
31/01/202	18/02/202	18/02/202	0	
22/01/202	24/01/202	20/01/202	0	1
30/01/202	29/03/202	27/01/202	0	1
22/01/202	21/02/202	21/02/202	0	
11/03/202	15/03/202	22/02/202	0	
02/02/202	25/03/202	02/03/202	0	1
14/01/202	23/01/202	23/01/202	0	
01/03/202	02/03/202	21/02/202	0	
27/02/202	02/03/202	21/02/202	0	
22/01/202	25/01/202	25/01/202	0	

20/01/202	17/03/202	24/01/202	0	1
18/01/202	19/01/202	19/01/202	0	
25/01/202	27/01/202	23/01/202	0	1
	16/02/202	16/02/202	0	1
15/02/202	15/02/202	25/02/202	0	
	07/02/202	07/02/202	0	1
22/01/202	12/02/202	12/02/202	0	1
15/01/202	17/01/202	13/01/202	0	
29/01/202	01/02/202	28/01/202	0	
07/02/202	07/02/202	07/03/202	0	
13/02/202	22/02/202	04/02/202	0	
18/02/202	10/02/202	10/02/202	0	
24/02/202	24/02/202	21/02/202	0	
28/01/202	31/01/202	21/01/202	0	1
14/02/202	17/02/202	17/02/202	0	
29/01/202	04/03/202	04/03/202	0	
03/02/202	04/02/202	17/01/202	0	1
27/01/202	28/01/202	23/01/202	0	1
30/03/202	31/03/202	29/03/202	0	
07/02/202	08/02/202	24/01/202	0	1
11/01/202	11/01/202	19/01/202	0	
21/01/202	24/01/202	20/01/202	0	1
02/02/202	21/02/202	23/02/202	0	1

PAC_COCB	PAC_DSCB	OUT_ANIM	DOR_ABD	FADIGA	PERD_OLFT	PERD_PALA	TOMO_RES	TOMO_OUT	DT_TOMO
524105	VENDEDOR EM DOMI		2	2	2	2	1		23/06/202
			2	1	1	1			
						1			
			2	2	2	2			
			1	1	2	2	6		
				1	1		1		
			2	2	2	2			
			2	2	2	2			
			2	1	2	2	6		
			2	1	2	2			
					1		1		07/12/202
			1	2	2	2	9		
			2	2	2	2	1		12/09/202
					1	1	1		27/11/202
239415	PEDAGOGO		2	1	2	1	1		22/12/202
			2	1	1	1	1		27/11/202
			1	1	2	2	1		10/06/202
			2	2	2	2	6		
			2	2	2	2	6		
			2	2	2	2			
			2	2	2	2			
			2	1	1	1	6		
			2	1	2	2	1		09/01/202
							6		
			2	2	2	2			

						9		
		2	2	2	2	6		
		2	1	2	2	1		19/11/202
		9	9	9	9			
		2	2	1	2			
		2	1	2	2	6		
524215	VENDEDOR PERMISSI	2	2	2	2	1		20/07/202
		2	2	2	2			
		2	2	2	2			
		2	2	2	2			
		2	2	2	2	6		
		9	9	9	9			
		2	2	2	2	1		17/10/202
		1	1	2	2	1		
		2	2	2	2			
		2	2	2	2	6		
		2	1	2	2	1		
		2	2	2	2	1		
		2	2	2	2	6		
		2	2	2	2	5	OPACIDAD	11/12/202
		2	2		2	6		
		1	1	2	2	6		
		2	2	1	2	1		28/07/202
						6		
		1	1	2	2	1		17/11/202
		2	1	2	2	1		10/11/202

		2	2	2	2	6	
		1	2	2	2	6	
		9	1	1	1	1	17/08/202
		2	2	2	2	6	
		2	2	2	2		
					1	1	25/12/202
				1			
223405	FARMACEUTICO	9	9	9	9		
			1			1	10/07/202
		2	2	2	2	1	08/10/202
		2	1	2	2	6	
		2	2	2	2	1	11/09/202
		1	1	1	1	6	
		2	2	2	2	1	21/10/202
		2	2	2	2		
						1	07/12/202
			1			6	
		2	1	1	1	1	15/07/202
		2	2	1	2		
		2	2	1	1	6	
		2	2	2	2		
		2	1	2	2	9	
241005	ADVOGADO	2	1	2	2	1	16/12/202
		2	1	1	1	1	18/09/202
		2	2	2	2		

				1		04/11/2020
				1		02/12/2020
2	2	2	2	1		10/10/2020
2	2	1	1	1		24/11/2020
				1		09/06/2020
				1		03/05/2020
1	1	2	2	9		
2	2	2	2			
2	2	2	2			
		1				
2	2	2	2			
2	1	1	1	1		
2	2	1	2	1		22/07/2020
9	9	9	9	6		
2	2	2	2	1		29/09/2020
2	2	2	2			
2	2	2	2			
2	1	2	2	1		01/12/2020
				6		
2	1	2	2			
2	2	2	2			
2	1	1	1	3		27/08/2020
				5	DERRAME	23/04/2020

		2	1	2	2	1		17/08/202
		2	1	1	1	1		16/09/202
		1	1	1	1	1		18/09/202
		2	1	2	2			
		1	2	2	2	6		
		2	2	2	2			
						6		
		2	2	2	2			
		2	2	2	2			
322205	TECNICO DE ENFERMAGEM							
						1		31/07/202
		9	9	9	9	6		
		2	2	2	2			
		2	2	2	2	1		15/07/202
		2	2	2	2			
		2	2	2	2			
		2	2	2	2			
		9	9	9	9	9		
						1		
		2	2	2	2	6		
			1	1	1	1		13/07/202
		1	2	2	2	1		09/09/202
		2	2	1	2	6		
		2	2	2	2	5	RESTOS PL	26/07/202
				1	1			
		2	2	2	2			
						1		20/04/202
		1	1	2	2			
						1		01/04/202
				1	1	3		27/07/202
						1		07/01/202

2	2	1	1	6		
2	2	2	2			
				1		
				1		08/06/202(
2	1	2	2	1		
2	2	2	2	1		28/08/202(
				6		
2	2	2	2			
				2		19/11/202(
2	2	2	2	6		
2	2	1	2	1		15/10/202(
2	2	2	2	1		21/04/202(
2	1	1	1	1		08/01/202:
				1		29/09/202(
2	2	2	2			
2	2	2	2	1		23/07/202(
2	2	2	2	6		
				1		14/01/202:
1	1	2	2	9		
2	1	2	2	1		19/11/202(
2	2	1	1	5	VIDRO FOS	24/07/202(
				1		18/06/202(
2	2	2	2	9		
				1		

2	2	2	2	9	
2	2	2	2	4	15/05/202[illegible]
1	2	1	1	2	21/08/202[illegible]
	1	1	1	1	25/08/202[illegible]
				9	
2	2	2	2	1	24/12/202[illegible]
2	2	2	2	1	02/12/202[illegible]
2	2	2	2	1	07/07/202[illegible]
2	2	2	2		
				6	
1	2	2	2	2	28/09/202[illegible]
2	2	2	2	6	
2	2	2	2		
				1	21/07/202[illegible]
1	1	1	1	2	17/07/202[illegible]
2	2	2	2	1	
1					
				6	
2	2	2	2		
				6	
				1	26/06/202[illegible]
2	2	2	2		
2	2	2	2		

		2	2	2	1	1	19/08/202
						1	04/11/202
		2	2	2	2	6	
		2	2	1	1	6	
						1	
		2	2	2	2	6	
		2	2	2	2	1	16/09/202
		2	2	2	2	6	
						6	
		2	2	2	2	9	
		2	2	2	2	1	11/08/202
			1			6	
						1	02/11/202
		2	2	2	2		
		2	2	2	2	3	30/12/202
		2	1	2	2	6	
		2	2	2	2	1	16/08/202
		2	2	2	2	1	17/09/202
		2	2	2	2	1	31/07/202
			1		1	6	
		2	2	2	2		
		2	2	2	2	1	02/12/202
XXX	NAO INFORMADO	1	2	1	1	1	01/10/202
						1	
		2	1	1	1	1	14/07/202
		2	2	2	2		
			1	1		1	
		2	2	2	2		

		2	2	2	2	1		27/09/202
		2	2	2	2			
		1	2	2	2			
						1		25/10/202
		2	2	2	2	9		
		2	2	2	2	1		11/12/202
		9	9	9	9	5	ACOMETIM	09/12/202
			1			1		08/09/202
			1					
						1		27/08/202
		2	2	2	2			
			1	1	1	1		30/10/202
		2	1	9	9	1		23/08/202
		9	9	9	9			
		2	2	1	1			
		9	9	9	9	1		05/12/202
		2	2	2	2			
		2	2	2	2			
		2	2	1	1			
		2	2	2	2			
						1		26/07/202
512105	EMPREGADO DOMES	2	2	2	2			
		2	2	2	2			
		2	2	2	1	1		31/12/202
		2	2	2	2	1		04/10/202
		2	2	2	2	6		
		2	2	1	1	1		20/11/202
		1	2	2	2			
						6		
		2	1	1	1	6		
					1	1		27/08/202
		2	2	2	2			
354705	REPRESENTANTE COM	2	2	2	2	6		
		2	2	2	2			
		1	1	2	2			
		2	2	2	1	1		02/09/202
						6		

				1	1	1	29/07/202[illegible]
						9	
		2	2	1	1		
		2	2	2	2	1	01/10/202[illegible]
		2	2	2	2	2	19/09/202[illegible]
		2	1	2	2		
		2	2	2	2		
		2	1	2	2	1	06/11/202[illegible]
		2	2	2	2		
		2	1	2	2		
						6	
		1	1				
		2		1	1	9	
		2	2	2	2		
						6	
		2	2	2	2		
		2	2	1	1		
422310	OPERADOR DE TELEMARKETING ATIVO E RECEPTIVO					6	
		2	2	2	2	3	15/12/202[illegible]
		1	1	1	1	6	
						1	07/10/202[illegible]
		2	2	2	2	6	
		2	1	1	1		
251605	ASSISTENTE SOCIAL	2	2	2	2	1	14/07/202[illegible]

2	2	2	2	6	
2	2	2	2	9	
2	2	2	2		
2	2	2	2	6	
				1	29/04/202
2	2	2	2	1	24/07/202
2	2	2	2	1	
	1	1	1		
2	2	2	2		
2	1	1	1	6	
		1	1		
2	2	2	2	1	31/10/202
2	2	2	2		
2	2	2	2	6	
1	1	1	1	1	13/09/202
2	2	2	2		
	1	1	1		
2	2	2	2	2	
				1	
2	2	2	2	1	08/11/202
1	1		2	9	

						1		27/11/202(
		2	1	2	2	9		
						1		
		2	2	2	2	5	CONSOLIDACAO	
		2	2	2	2	1		08/10/202(
		2	2	2	2	1		17/10/202(
		2	2	2	2			
						9		
		2	2	2	2			
		2	2	2	2	1		22/12/202(
		2	2	2	2	1		23/11/202(
		2	2	2	2	1		24/07/202(
		2	1	1	1	1		
		2	2	2	2	5	SEM SINAIS	13/08/202(
		1	2	2	2	6		
				1		1		
		2	1	2	2	1		29/12/202(
231205	PROFESSOR DA EDUC.	9	9	9	9			
		2	2	1	1	1		19/08/202(
		2	1	2	2	1		03/01/202:
		9	9	9	9			
						1		28/05/202(
		2	2	2	2	9		
		2	1	2	2	1		10/01/202:
						9		
		2	2	2	2	1		29/07/202(
		2	2	1	1	6		
		2	2	2	2			
		1	2	2	2	1		13/06/202(
		2	2	2	2	6		

2	1	1	1	3	24/10/2020
2	2	2	2	6	
2	2	2	2	1	
2	9	2	2	1	04/05/2020
		1			
1	2	2	2	6	
				1	10/10/2020
2	2	2	2	4	16/10/2020
2	1	2	2	9	
				1	
2	2	2	2	2	03/09/2020
				1	24/08/2020
2	2	1	1	6	
				1	04/01/2021
2	2	2	2		
2	2	1	1		
2	2	2	2	1	24/08/2020
2	2	2	2	6	
2	1	1	1		
2	2	2	2		
2	2	2	2	6	
				1	15/01/2021
2	1	9	2	1	14/07/2020
2	2	2	2	1	28/11/2020
				1	16/10/2020
				1	
2	2	2	2	1	
2	2	1	1	6	
2	1	2	2		
2	2	2	2	6	
2	2	2	2	6	
2	2	2	2	1	01/10/2020
2	2	2	2	1	06/01/2021
2	1	1	1	1	18/11/2020

241005	ADVOGADO					5	VIDRO FOS	09/07/202
		2	2	1	1			
						1		24/06/202
		2	2	2	2	1		11/02/202
						1		23/05/202
		2	2	2	2			
		1	1	2	2	1		15/06/202
		9	9	9	9	6		
		2	2	2	2	1		16/09/202
				1				
						9		
						1		30/06/202
		2	2	2	2	1		11/08/202
		9	9	9	9			
		2	2	2	2	6		
		2	2	2	2			
		2	2	2	2	1		10/06/202
		2	1	1	1	6		
		2	2	2	2			
		2	2	2	2			
			2	1	1	1		29/12/202
		2	2	2	2	6		
		2	2	2	2	1		07/10/202
		2	2	1	1	1		08/11/202
		2	2	2	2	1		28/12/202

2	2	2	1			
2	2	2	2	6		
2	2	2	2	1		
2	2	2	2	1		21/08/202
				1		
2	2	2	2	6		
2	2	2	2			
2	2	1	1	5	ESPESSAM	24/09/202
2	2	2	2			
2	2	2	2			
2	2	2	2	1		13/11/202
2	2	2	2	6		
	1			1		26/11/202
2	2	2	2			
2	2	2	2			
				6		
2	2	2	2	6		
2	2	2	2	2		
1	2	1	1	1		10/11/202
2	2	2	2	1		23/07/202
2	2	2	2	6		
				6		
2	1	2	2			
2	2	2	2			
2	2	2	2			
				6		
		1		6		
2	1	2	2	1		08/12/202
	1					

		2	2	2	2		
						1	
		2	2	1	2	1	27/12/2020
		2	1	2	2	1	07/11/2020
		2	2	2	2	6	
		2	2	2	2	6	
		2	2	2	2		
		2	2	2	2	6	
		2	1	1	1	1	05/12/2020
		2	2	2	2	9	
		2	2	2	2	6	
		2	2	2	2	1	09/01/2021
		9	9	9	9		
		2	1	2	2		
		2	2	2	2	6	
		2	1	2	2	3	09/10/2020
		2	2	2	2		
233120	PROFESSOR DE TECNIC	2	9	2	2		
		2	1	2			
		2	2	2	2	1	16/08/2020
						1	02/01/2021
			1			3	
		1			2	3	04/01/202
		2	1	2	2	6	
		2	2	1	1	1	20/11/2020
						1	14/12/2020
		1	1	2	2	4	03/08/2020

		2	2	2	2	1		26/05/202
		1	1	1	1	6		
		2	1	2	2			
		2	2	2	2	6		
		2	2	2	2	6		
322205	TECNICO DE ENFERMA	2	1	1	9	6		
		2	2	2		2		07/01/202
						1		
		2	2	2	2	9		
		2	2	2	2			
		2	2	2	2			
		2	2	2	2			
		2	1	1	2	1		11/09/202
		2	2	2	2	5	TC TORAX	24/12/202
		2	2	2	2	1		20/08/202
						6		
						1		24/07/202
		2	2	2	2	9		
		2	2	2	2			
		2	2	2	2	3		17/09/202
		2	2	2	2	6		
		1	2	2	2	1		16/11/202
		2	2	2	2	6		
		2	1	2	2	1		
						1		
		2	1	1	1	1		25/08/202
		2	2	2	2			
		2	1	2	2			
		2	1	1	2	1		04/01/202

		1	1		
2	1	1	2		
2	2	2	2	9	
9	9	9	9	1	23/08/2020
1	1	9	2	1	
9	9	9	9	1	16/08/2020
2	2	2	2	1	28/11/2020
2	9	9	9		
	1	1	1		
		1	1	1	30/12/2020
				1	18/08/2020
2	1	2	2		
				1	27/07/2020
1	2		1		
				1	05/12/2020
1	1	1	1	6	
2	1	2	2	1	08/12/2020
2	2	2	2		
2	2	2	2	6	
2	2	2	2		
2	2	2	2	1	09/12/2020
2	2	2	2	6	
1	1			6	
				1	

		2	2	2	2	1		25/06/202
		2	1	2	2	1		28/09/202
		2	2	2	2	6		
		2	2	2	2	6		
						5	CONDENSA	18/08/202
						5	NAO SUGE	10/10/202
		1	1	2	2	9		
		2	2	2	1	1		16/03/202
		2	2	2	2	1		20/03/202
		9	1	2	1	1		15/03/202
						1		17/04/202
						1		29/05/202
512105	EMPREGADO DOMES	2	2	2	2	1		23/05/202
		2	2	2	2	1		22/06/202
		2	2	2	2	6		
		2	2	2	2	6		
		2	2	2	2	6		
		2	2	1	2	1		05/02/202
		2	1	2	2	9		
		1	1	1	1	6		
		9	9	9	9			
						6		
						1		
		2	1	2	2	6		
		2	1	2	2	1		10/06/202
		2	1	9	9	1		
		2	2	1	2	1		20/09/202
		2	2	2	2	6		
		2	1	2	2	1		29/12/202
		2	2	2	2	9		
		2	2	1	1			
		1	2	1	2	1		27/03/202
		2	2	2	2	1		04/04/202
		2	2	2	2	5	PNEUMON	19/03/202
		2	1	2	1	1		02/06/202
		2	2	2	2	6		
						1		04/07/202

2	2	2	2		
2	1	1	2	6	
				6	
				1	17/05/202
2	1	2	2	1	11/06/202
2	2	2	2	1	05/05/202
2	2	2	2		
	1	1	1		
2	2	2	2		
1	1	9	9	1	20/05/202
2	2	2	2		
2	2	2	2	6	
1				1	18/09/202
2	2	2	2	6	
				1	26/02/202
2	2	2	2	1	23/03/202
	1			6	
2	2	2	2	1	31/03/202
2	2	2	2	1	
2	1	2	2	1	09/04/202
2	1	2	2	1	27/04/202
2	2	2	2		
2	2	2	2	1	19/02/202
2	2	2	2	1	23/02/202
				1	03/06/202
2	1	2	2	1	
2	2	2	2	1	28/09/202
				1	23/05/202
2	2	2	2	6	
2	1	9	9	1	
2	2	2	2		
9	9	9	9	6	
1				1	
1	1	2	1	9	
2	1	2	2		
2	2	1	1	1	
2	2	1	2	1	15/03/202
				1	14/03/202
				1	24/04/202
2	1	2	2	1	30/04/202
2	2	2	2	6	
2	2	2	2	1	02/05/202
				6	
2	2	2	2		

	1	1	1	1	
2	1	2	2	6	
	2			1	14/02/202
2	2	2	2	2	31/03/2021
2	2	2	2	1	26/03/2021
2	2	2	2	1	12/03/2021
2	2	2	2	6	
	1	2	2	1	05/04/2021
2	1	2	2	1	02/04/2021
2	2	2	2		
				6	
2	2		2	1	
	1			1	15/06/2021
				1	20/06/2021
2	2	2	2		
	1			6	
2	1	2	2		
	1			6	
				1	25/04/2021
1	1	2	2	1	16/05/2021
2	1	2	2		
	1			6	
2	2	2	2	6	
2	2	2	2		
2	1	1	1	1	20/06/2021
2	2	2	2	1	20/07/2021
1	1			6	
		2	2	1	
2	2	2	2		
2	2	2	2		
				6	
2	2	2	2	1	10/04/202
2	1	2	2	1	05/04/2021
2	2	1	2		
			1		
2	2	2	2	1	05/05/2021
2	2	2	2		
	1			1	17/05/2021
2	2	1	1	6	
2	1	2	2	1	01/07/202
2	2	2	2	1	13/06/2021
2	2	2	2	1	12/07/2021
2	2	2	2	1	05/10/2021
2	2	2	2	2	17/02/2021

		1	2			1		12/08/202
		9	9	9	9	6		
		2	1	9	9	1		07/09/202
						6		
					1	1		04/03/202
		2	2	2	2	1		06/03/202
		2	2	2	1	6		
		2	2	2	2	6		
				1	1			
		2	2	2	2	1		19/05/202
			1			1		15/05/202
		2	2	2	2			
		2	2	1	2	1		16/05/202
		2	1	1	2	6		
		1	1	2	2	1		29/05/202
						9		
		2	1	2	2	6		
						5	CONSOLID	22/10/202
		9	1	1	1			
		2	2	2	2			
		2	2	2	2			
		2	1	1	1	9		
		2	2	2	2			
		2	1	2	2			
		2	2	1	1	6		
			1	1	1	1		29/03/202
		2	2	2	2	5		07/04/202
			1	1		5		06/04/202
						6		
		2	2	2	2			
						9		
		1	2	2	2	1		28/10/202
						1		29/03/202
		2	2	2	2	3		14/05/202
		2	2	2	2	1		29/04/202
		1	1	2	2	1		02/04/202
		2	2	2	2	6		
		2	2	2	2	9		
		2	1	1	1	1		
				1	1			
		2	1	1		6		
						5		
		1		1	1	1		
222110	ENGENHEIRO AGRONOMO					6		
		2	2	2	2	1		23/06/202
		2	1	1	1	1		12/01/202

		2	2	1	2	6	
		2	2	2	2	1	13/03/2021
		9	9	9	9	9	
						6	
		2	2	2	2	6	
		2	2	2	2		
		2	2	2	2	1	03/04/2021
		2	1	2	1	1	15/06/202
						6	
		2	2	2	2	6	
						6	
		2	2	2	2		
		1	2	1	1	1	28/11/2021
						4	31/01/2021
		2	1	2	2		
		2	1	1	2	3	16/01/2022
		2	1	2	2	6	
		2	1	1	1	6	
		9	1	9	9	1	30/03/2021
XXX	NAO INFORMADO	2	2	2	2	1	07/03/2021
		2	1	1	2	1	08/03/202
			1	1	1	6	
		2	1	2	2	1	11/04/2021
			1			6	
		2	2	2	2	6	
		2	2	9	9	1	06/05/2021
		2	2	2	2	6	
						6	
		2	2	2	2		
		2	2	2	2	6	
						1	
		2	2	2	2	6	
		2	2	2	2	1	22/07/2021
						1	16/05/202
		2	1	2	2	1	17/08/202
		2	1	2	2		
223405	FARMACEUTICO	2	1	1	2	6	
						1	13/03/2021
		2	1	9	9	1	21/03/2021
		2	2	2	2	1	23/03/2021
		1		1	1		
		2	2	2	2		

						6		
				2	2	1		
		2	2	2	2	1		05/06/202
		1	1	1	1	1		
		2	2	2	2	6		
		2	2	2	2	1		02/09/202
		2	1	1	1			
		9	9	9	9			
		2	2	2	2			
		2	2	2	2	1		24/05/202
		2	1	1	2	6		
		2	2	2	2			
						6		
		2	2	2	2	1		15/04/202
						1		06/07/202
		2	2	1	1	1		23/06/202
		2	2	2	2			
		2	1	1	1	1		20/07/202
		1	2	2	2	6		
						6		
		2	1	1	1	5	CONSOLID	11/01/202
		2	1	2	2	1		03/03/202
		2	1	2	2	1		04/04/202
						6		
		1	1	1	2	1		29/03/202
		2	1	1	2	9		
322205	TECNICO DE ENFERMAGEM					1		
		2	1	2	2	1		
		2	2	2	2			
						6		
		2	2	9	9			
		2	2	1	2			
			1			6		
		2	2	2	2	1		
		2	2	2	2	1		19/03/202
		2	2	2	2	1		16/03/202
		2	2	2	2	6		
		2	1	2	2	6		
		2	1	2	2	6		
		2	2	2	2	6		
						1		21/03/202
521110	VENDEDOR DE COMEF	2	2	2	2	1		08/05/202
		2	2	2	2	6		
		2	2	2	2	1		24/04/202
						6		
					1	6		

		2	2	1	1	1	18/05/2021
						9	
		2	2	2	2		
		1	1	2	2	1	19/07/2021
		2	2	2	2	6	
		2	2	2	2	6	
		1	2	2	2	1	18/02/202
		2	1	2	2	6	
						9	
		1	2	9	9	6	
		2	2	2	2		
			1	1	1	9	
		2	1	1	2		
		9	9	9	9	6	
		2	2	2	2	1	11/04/2021
						1	
516110	CABELEIREIRO			1	1	1	10/04/2021
		2	1	2	2		
		2	2	2	2	1	22/06/202
		2	2	2	2	6	
		1	1	2	2	6	
		2	2	2	2	1	19/05/2021
						2	
		2	2	2	2	6	
		2	2	2	2		
		2	1	2	2	1	01/04/2021
		9	1	9	9	1	28/04/2021
		2	2	1	1		
		2	1	2	2	1	22/02/2021
		2	2	2	2	6	
		2	2	2	2	2	06/01/202
						1	23/11/2021
413205	ATENDENTE DE AGEN	2	1	2	2	6	
				1		1	18/01/2021
		2	2	2	2	1	03/02/2021
		2	2	2	2	9	
		2	2	2	2	1	06/03/2021
		2	1	2	2	6	
		2	1	1	1		
		2	2	1	1	1	30/05/2021
		2	1	1	1	1	02/06/2021
		2	1	2	2	1	
		2	2	2	1	6	

422105	RECEPCIONISTA, EM G	2	1	2	2	6		
		2	2	2	2			
421125	OPERADOR DE CAIXA	2	2	2	2	1		16/06/202
		2	1	2	2	1		21/06/202
		2	1	2	2			
		2	1	2	2	1		
		2	1	2	2	1		
		1	1	1	1	1		03/04/202
		1	1	2	2	1		25/03/202
						1		14/04/202
		1	1	2	2	1		28/06/202
		2	1	2	2			
		2	2	2	2	1		10/03/202
		2	2	2	2			
		2	2	2	2	1		21/05/202
		2	2	2	2	6		
		2		1	9	6		
		1	2	2	2			
		1	2	2	2	6		
						6		
						6		
		2	2	1	2	1		03/05/202
		2	1	2	2	6		
		2	2	2	2			
						1		06/06/202
		2	2	2	2			
				1	1	2		
		2	2	2	2	9		
				1	1	5	OPACIDAD	13/06/202
				1	1	6		
		2	2	2	2	6		
		2	1	1	1			
						1		25/03/202
		2	2	1	1	6		
						1		19/04/202
		2	1	2	2	6		
				9	9	6		
						9		
		2	2	2	2			
		2	1	2	2	1	VIDRO FOS	18/08/202
		2	2	2	2			
		2	2	2	2			
784205	ALIMENTADOR DE LIN	1	1	1	2	1		13/07/202
						1		21/06/202

	1			1	27/07/202
2	1			1	
2	2	2	2	6	
2	1	1	1	6	
2	1	1	1	1	15/03/202
2	2	2	2	1	19/04/202
2	1	1	1	6	
				1	04/04/202
1	1	2	2	6	
2	2	2	2		
2	2	2	2		
2	2	1	1		
2	1	2	2	1	24/06/202
				6	
				1	02/07/202
2	2	2	2		
2	2	2	2	6	
2	1	2	2	1	30/04/202
2	2	2	2	1	15/04/202
1	1	1	2		
1	1	2	2		
				1	21/05/202
2	2	2	2		
2	1	2	2	1	28/05/202
2	1	1	1	9	
1	1	1	1		
			1	1	03/08/202
2	2	2	2	2	21/12/202
2	1	2	2	1	04/04/202
2	2	2	2	6	
1	2	2	2	1	24/04/202
1	2	2	2	6	
2	2	2	2	1	10/04/202
2	1	2	2	1	05/05/202
2	2	1	2	6	
1	1		2	1	26/04/202
2	2	1	1	6	
2	1	2	2	1	18/06/202
1	2	2	2		
2	1	1	1	1	04/07/202
				6	
		2	2		
2	2	2	2		
2	2	2	2	1	29/06/202
1	1	2	2	1	27/12/202

2	1	2	2		
2	2	2	2	6	
2	2	2	2		
2	1	2	2		
2	2	2	2	9	
9	9	9	9	9	
2	1	2	2	1	26/04/202
2	2	2	2	1	25/05/202
2	1	2	2	6	
1	1	1		1	
				1	17/07/202
				6	
2	2	2	2		
2	1	2	2	1	15/03/202
				2	30/03/202
2	2	2	2	1	05/04/202
2	2	2	2		
	1			1	26/04/202
2	1	2	2		
2	1	2	2	1	15/04/202
	1				
1	2	2	2		
				9	
2	2	2	2	1	07/08/202
				6	
2	1	2	2	1	
1	2	2	2	1	27/07/202
1	1	1	1	6	
2	2	2	2		
2	2	2	2	6	
				1	07/11/202
		1		6	
2	1	2	2	1	02/04/202
2	2	2	2		
2	2	2	2	1	07/03/202
2	1	1	1	1	18/04/202
	1			1	03/05/202
2	2	2	2		
2	2	1	1	1	
2	1	1	1	1	25/05/202
2	1	2	2	6	
2	1	2	2	1	
2	2	2	2	1	07/05/202
1				1	22/04/202
1		2	2	1	
				1	18/05/202

2	2	2	2	1		20/06/202
2	1	2	2			
1	2	2	2	9		
2	2	2	2	6		
2	1	2	2			
	1			1		
2	1	2	2	5	S/LAUDO	06/06/202
2	2	2	2	6		
1	2	2	2	6		
2	1	2	2	6		
2	1	2	2	1		03/03/202
2	2	2	2	6		
	1			1		15/04/202
2	1	1	2	1		11/03/202
2	1	2	2	6		
2	2	2	2	1		25/05/202
				1		07/06/202
	1			1		29/07/202
				1		29/06/202
2	1	2	2	1		17/07/202
				1		13/07/202
2	2	2	2	1		05/03/202
2	2	2	2	1		27/03/202
				1		
2	2	2	2	6		
2	2	2	2	1		04/03/202
2	2	2	2	6		
2	2	2	2	1		19/04/202
2		2	2	1		
2	2	2	2	1		01/03/202
2	2	2	2	1		06/06/202
2	1	2	2	5		
2	2	2	2	1		29/07/202
2	1	2	2	1		14/04/202
2	1	2	2	1		11/06/202
2	1	2	2	6		
				1		23/03/202
		1				
2	2	2	2	1		12/05/202
2	2	2	2	1		12/01/202
2	2	2	2			
2	1	2	2			
9	9	9	9	6		

		1	1	9	9	1		11/04/202
		2	1	2	2	1		10/05/202
		2	1	2	2	1		03/05/202
						1		26/05/202
		2	1	2	2	1		05/06/202
		2	2	2	2			
		2		9	9	4		13/06/202
		2	1	2	2	1		17/06/202
516120	MANICURE	2	2	2	2	9		
		2	1	2	1	6		
						6		
			1					
						4		
		2	2	2	2	1		05/02/202
						1		06/03/202
		2	1	1	1	6		
		2	1	1	2	1		12/05/202
		2	1	1	2	1		03/05/202
		2	1	2	2	1		22/05/202
		2	2	2	2			
		2	1	1	2	6		
		2	2	2	2	1		04/05/202
		2	2	2	2			
			2	2	2			
		2	2	2	2			
					1	1		22/02/202
						1		23/04/202
		9	1	9	9	1		14/04/202
		2	2	2	2			
			1	1	1			
		2	9	1	1	1		24/03/202
		2	2	2	2	6		
		2	2	2	2			
		2	2	2	2			
		2	2	2	2			
		1	1	9	9	5	CAVITACOI	30/07/202
		2	1	2	2	6		
		2	1	2	2	1		02/03/202
		2	2	1	1	6		
						1		27/03/202

						1		27/03/202
		1	1	9	9			
		2	1	2	2	1		07/06/202
		2	2	2	2			
513435	ATENDENTE DE LANCH	2	1	2	2	1		20/05/202
		2	2	1	2	1		25/06/202
		9	9	9	9	9		
622020	TRABALHADOR VOLAN	2	2	2	2	6		
		2	1	1	1			
						1		10/07/202
		2	2	1	2	6		
		2	1	2	2	1		21/07/202
					1	1		11/04/202
		2	1	1	1	1		29/04/202
		2	9	1	1	5	COMP PUL	15/03/202
		2	2	2	2	6		
		2	1	2	2	1		03/06/202
			1			1		08/06/202
		2	2	2	2	1		18/05/202
		2	2	2	2	1		06/09/202
		2	2	2	2	6		
		2	1	2	2	1		23/06/202
		2	1	2	2	1		
		2	2	2	2	1		03/06/202
				1		2		
		2	1	2	2	1		24/03/202
		2	1	2	2	1		10/03/202
						1		26/02/202
		2	1	9	2	6		
		2	1	2	2	1		21/04/202
			1			9		
		2	2	2	2	1		01/04/202
		2	2	2	2			
		2	2	2	2			
		2	2	1	1	1		12/04/202
		2	1	2	2	6		
		2	1	9	9	6		
		2	2	2	2	6		
		2	2	2	2			
		2	2	2	2	6		
		2	2	2	2			
		2	1	2	2	1		12/01/202
622020	TRABALHADOR VOLAN	2	1	2	2	1		15/08/202
		2	1	2	2	6		
516120	MANICURE	9	9	9	9			
		2	1	1	1	1		19/03/202

						1	05/05/202
		2	2	1	1	1	24/05/202
		2	2	2	2	6	
		2	2	2	2		
						6	
						9	
		2	2	2	2	1	17/05/202
		2	2	2	2	6	
		2	2	2	2		
			1	1		1	13/08/202
			1			1	01/07/202
						6	
		2	2	2	2	1	18/04/202
231205	PROFESSOR DA EDUC	2	2	2	2	6	
		1	1	2	2		
						1	14/04/202
		2		1	2	1	17/02/202
		1	1	2	2	1	13/03/202
		2	1	2	2	1	18/04/202
		2	2	2	2	1	31/05/202
142315	GERENTE DE MARKETING		1	1	1	1	
		2	2	2	2	6	
		2	2	1	1	6	
						6	
		2	2	2	2	1	20/02/202
						6	
411010	ASSISTENTE ADMINIST	2	1	2	2	1	24/02/202
						1	02/03/202
		1	1	2	2		
		2	1	2	2	6	
			1	1	1	6	
			1	1	1	6	
		2	2	2	2	1	12/04/202
		2	2	2	2		
						6	
		2	2	2	2		
		2	2	2	2	1	04/08/202
		9	1	9	9	1	04/08/202
		2	2	2	2		
		2	1	2	2	9	
		2	1	2	2		
		1	1	1	1	6	
		2	2	2	2	1	20/03/202

		2	2	1	1	6	
		2	2	2	2	6	
			1				
		2	2	2	2	1	13/08/202
		2	2	2	2	1	01/07/202
		2	2	2	2		
						6	
		2	2	2	2	6	
						1	13/03/202
				1	1		
						1	05/03/202
		1	1	2	2	6	
						1	26/03/202
						3	21/03/202
		2	2	2	2	6	
		1	1			1	
		1	2	2	2		
		2	2	2	2		
		2	2	2	2	6	
						1	21/06/202
						9	
		2	2	9	9	1	19/08/202
		2	1	2	2	6	
		2	2	2	2		
		2	1	2	2	1	28/04/202
						1	20/04/202
		2	1	2	2	1	25/04/202
		2	2	2	2	6	
			1			1	20/06/202
		2	1	2	2	1	01/07/202
			1				
						6	
		2	1	2	2	1	03/03/202
		9	9	1	1	1	08/04/202
		2	2	2	2	1	07/03/202
		2	2	2	2	1	13/04/202
		2	9	9	9	1	03/05/202
			1				
		2	2	2	2		
516120	MANICURE	1	1				
		2	2	2	2	1	26/04/202
						1	
		2	2	1	1	9	

		2	2	2	2	5	OPACIDAD	09/06/202
						1		20/04/202
		2	2	1	1	9		
		2	2	2	2	6		
		2	2	2	2	1		21/02/202
						1		28/02/202
		2	1	2	2	9		
		2	2	2	2	6		
		2	2	2	2			
		2	1	2	2	5	VF 25-50%	26/01/202
		2	2	2	2			
		2	1	1	1	1		24/03/202
		2	1	9	9	6		
		2	1	2	2	5	CO-RADS 4	05/07/202
		2	1	2	2	1		10/10/202
		2	2	2	2	1		02/08/202
		2	1	2	2	1		28/08/202
		1	1	2	2	1		08/02/202
XXX	NAO INFORMADO	1	1	2	2			
		2	2	2	2	6		
		2	2	2	2	1		15/04/202
		2	2	2	2	5	VIDRO FOS	30/04/202
			1			6		
			1	2	2	6		
						1		30/05/202
		2	2	1	2			
						6		
		2	1	2	2	1		18/01/202
223505	ENFERMEIRO	9	9	9	9	1		18/04/202
		2	2	2	2			
		2	2	1	2	1		15/02/202
		1	1	2	2	1		
		9	9	9	9	1		31/05/202
		2	1	2	2			
		2	2	2	2			
		2	1	2	2	1		21/03/202
		1	1	1	1	1		03/04/202
		2	2	2	2	6		
		2	1	2	2	1		30/05/202
		2	2	1	2	6		
		2	1	2	2			
		2	2	2	2			

		9	9	9	9	1		10/07/202
		2	1	2	2	1		19/08/202
		2	2	2	2	6		
						6		
		1	1	1	1	1		24/08/202
				1	1	6		
		2	2	2	2	6		
		2	2	2	2	1		24/04/202
			1			6		
		1		1	1	1		
			1	2	2	1		07/04/202
		2	1	2	2	5	SOLICITAD	29/07/202
		2	2	2	2	1		03/09/202
		2	1	2	2	1		22/03/202
223208	CIRURGIAO DENTISTA	2	2	2	2	1		07/06/202
		1	2	2	2	6		
		2	2	2	2			
		1	2	1	1	1		18/03/202
		2	2		2			
		2	1	2	2	6		
		1	2	2	2	9		
			1	1	1	1		24/04/202
		2	2	1	2			
		2	1	1	1	1		15/03/202
			1	1	1	1		24/06/202
		2	1	2	2			
		2	2	2	2			
		2	2	2	2	1		06/05/202
		2	1	2	2			
		2	1	9	9	1		
		9	2	2	2	6		
		1	1	1	1	9		
			1			6		
		2	2	2	2	1		04/04/202
		2	2	2	2	1		29/04/202
				1	1	6		
			1					
		2	2	2	2	1		09/07/202
		2	1	1	1	1		12/08/202
		2	2	2	2	1		06/05/202
		2	2	2	2	6		
		2	2	2	2			
			1			1		28/03/202

2	2	2	2	1	25/03/202
				6	
2	2	2	2	1	09/05/202
				6	
	1			1	05/08/202
				6	
2	1			1	25/05/202
				1	08/10/202
2	1	2	2	1	02/08/202
2	1	2	2	1	18/10/202
2	1	1	1		
	1	2	2	1	
2	1	2	2	1	13/03/202
2	2	2	2	6	
2	1	2	2	6	
		1	1	1	16/03/202
		1			
				1	19/07/202
				1	12/01/202
1	2	2	2	6	
2	2	2	2	6	
2	1	2	2	1	19/03/202
2	2	1	1	9	
2	1	2	2		
2	1	2	2	6	
	1			6	
2	1	2	2	6	
2	2	2	2		
2	2	2	2		
2	2	2	2	5	18/06/202
2	2	2	2	1	18/06/202
2	1	2	2	1	09/06/202
2	1	2	2		
2	2	2	2	1	24/02/202
		1	1		
		2	2		
2	2	2	2	6	
2	1	2	2	1	15/06/202
2	1	1	1	1	13/06/202
2	2	2	2	1	24/06/202
9	9	9	9	9	
				1	18/04/202
				1	21/07/202
2	2	2	2	1	08/06/202
2	2	2	2	1	28/03/202

2	2	2	1	2	06/12/202
2	2	2	2	6	
2	1	2	2	2	25/03/202
2	1	2	2	1	04/04/202
2	1	2	2		
2	1	2	2	1	
2	2	2	2		
1				1	
2	2	2	2		
2	2	2	2	1	
2	2	2	2	6	
	1			1	25/08/202
2	2	2	2	1	18/08/202
		1	1	1	27/01/202
2	1	2	2		
				1	21/01/202
2	2	2	2	1	16/07/202
2	1	1	1	6	
			1	6	
2	2	2	2	6	
	1			1	22/06/202
2	1	2	2	6	
2	1	1	1		
2	2	1	1	6	
1	2	2	2	1	04/08/202
2	2	2	2	6	
	1				
2	9	9	9	1	01/11/202
2	2	2	2		
2	1	2	2	1	01/04/202
2	2	2	2		
				6	
2	2	2	2	6	
2	1	1	1	6	
	1				
				1	06/06/202
2	1	2	2	1	14/07/202
2	2	2	2	1	31/07/202
2	2	2	2		
2	1	2	2		
1	2	2	2	1	10/02/202
2	1	2	2	1	19/03/202
2	1	2	2	1	

2	2	1	2	1		05/04/202
2	1	2		6		
2	2	2	1	6		
2	1	2	1			
				6		
2	2	2	2	1		11/05/202
2	2	2	2			
2	1	2	2			
2	1	2	2	6		
2	2	2	2	1		17/06/202
2	2	2	2	1		
		1		6		
2	2	2	2			
2	2	2	2	6		
2	2	2	2			
2	2	2	2			
2	2	1	2			
1	1	1				
2	2	2	2	1		30/03/202
1	1	2	2	1		11/03/202
2	2	1	1			
2	2	2	2	6		
2	1	1	1	1		25/04/202
2	2	2	2			
2	2	2	2	6		
				6		
				6		
2	2	2	2	6		
				6		
2	2	2	2			
2	2	2	2			
2	2	2	2	6		
2	2	2	2	1		26/05/202
2	1	2	2			
	1			5	VIDRO FOS	15/01/202
2	2	2	2	1		13/10/202
				9		
2	2	2	2	1		28/07/202
2	2	2	2			
1	2	2	2	6		
2	2	2	2			
2	1	2	2	1		05/04/202

		2	2	2	2	1	14/05/202
		2	1	1	2	1	27/04/2021
		2	2	2	2	1	21/06/202
		2	1	9	9	9	
						1	03/07/2021
		2	1	2	2	1	07/07/2021
		1	2	2	2	1	19/09/202
		2	1	2	2	6	
		2	2	2	2	6	
		2	1	2	2	3	08/10/202
			1				
		2	2	2	2		
		2	1	2	2	1	09/03/2021
		9	9	9	9	1	08/02/2021
						1	14/04/202
		2	2	2	2	1	17/04/2021
		9	1	1	1	1	26/04/2021
					1	6	
						6	
		2	2	2	2	1	15/05/2021
			1			6	
		2	2	2	2	1	29/05/2021
		2	2	1	1	1	30/04/2021
		2	2	2	2	1	07/07/2021
		2	1	2	2	1	20/06/2021
		2	2	2	2	1	04/03/2021
		2	1	2	2	1	03/06/2021
						1	14/02/2021
		2	2	1	1	1	11/02/2021
261125	JORNALISTA	2	1	2	2	6	
512105	EMPREGADO DOMEST	2	1	2	2	1	09/08/202
		2	2	2	2	6	
		2	1	2	2	1	07/04/2021
						1	21/04/2021
						1	09/06/2021
		2	1	1	2	1	18/03/2021
322205	TECNICO DE ENFERMA	1	1	2	1	6	
512105	EMPREGADO DOMEST	2	2	2	2		
		1	1	1	1	6	
		2	1	2	2	1	15/06/2021
				1	1		
		2	2	2	2	1	03/11/2021
512120	EMPREGADO DOMEST	2	2	2	2		
						1	26/05/2021

				1	1			
		2	2	2	2	6		
				1	1	1		01/03/2021
			1	1	1	1		22/04/2021
231315	PROFESSOR DE EDUCA	2	2	2	2	9		
				1				
		2	1	2	2	9		
		2	2	1	1			
		1	1	2	2	4		31/05/202
		2	1	2	2	1		
		2	2	2	2	4		08/08/2021
		2	1	1	9	6		
		2	2	2	2			
		2	2	2	2			
						1		
		2	2	2	2	1		27/04/2021
		2	1	2	2	1		19/03/2021
						1		23/05/2021
						1		
						1		16/06/2021
				1		1		20/06/2021
		2	2	2	2			
						1		27/06/202
			1			1		
		2	1	2	2	1		30/11/2021
		2	2	2	2	9		
		2	2	1	1	6		
		2	2	2	2	5	CONSOLID	29/07/202
						1		
						1		25/03/2021
						1		
						1		22/03/2021
		2	2	2	2	9		
		2	2	2	2	6		
		2	1		9	1		
		2	1	2	2	1		31/05/2021
		2	1	2	2	6		
		2	1	2	2	1		10/06/2021
		2	1	2	2			
		2	2	2	2			
		2	2	1	1	1		04/02/2021
						1		20/01/2021
						1		06/05/2021
						1		26/05/2021
		2	2	2	2	6		
		2	1	2	2			

				1	1		
		2	2	2	2		
		2	2	2	2		
231210	PROFESSOR DE NIVEL :	2	2	2	2	6	
		2	2	2	2	1	19/07/202
		2	2	1	1	1	
		2	1	2	2	1	05/05/202
		1		1	1	2	
		2	2	2	2	1	25/02/202
						6	
		2	2	2	2	6	
		2	2	2	2	1	05/05/202
		9	9	9	9	6	
		2	2	2	2		
		2	2	2	2	1	16/04/202
322205	TECNICO DE ENFERMA	2	1				
		2	2	2	2	1	20/03/202
						1	30/05/202
		2	2	2	2		
		2	2	2	2	1	07/03/202
				1		1	
		1	2	1	2	1	16/07/202
						1	02/07/202
			1				
						1	09/08/202
		9	1	9	9	6	
		2	1	2	2	1	29/03/202
			1	1	1		
		2	1	2	2	1	
		2	2	2	2	1	10/03/202
				1			
		2	1	2	2	1	21/05/202
		2	1	2	2	6	
		2	2	2	2	1	15/05/202
		2	1	2	2	1	16/05/202
		2	2	2	2	1	20/07/202
		1				6	
		2	2	1	1	1	01/06/202
						6	
		2	1	1	1	1	
						1	10/03/202
		2	2	2	2	1	18/08/202

		2	2	2	2	1		
		2	2	2	2	1		07/06/202
			1			1		21/07/202
		2	2	2	2			
		2	1	2	2	6		
		2	2	2	2	5	ACOMETIM	03/07/202
		2	2	2	2	6		
		2	2	2	2			
			1			1		01/11/202
		2	2	2	2	1		24/04/202
		2	1	2	2			
		2	1	2	2			
		2	2	2	2	1		11/03/202
		2	9	9	9	6		
						1		03/06/202
		2	2	2	2	6		
						1		07/01/202
		2	2	2	2	5		07/08/202
		2	2	2	2	1		01/01/202
		2	2	2	2	6		
		2	2	2	2	1		
			1			6		
						6		
		2	2	1	1			
		2	2	1	1	1		18/04/202
		1	1	2	2	6		
				1				
		2	2	2	2			
		2	1	2	2	2		13/03/202
		2	2	2	2			
784205	ALIMENTADOR DE LIN	2	2	2	2			
		2	1	2	2	6		
		2	2	1	1			
		1	1	1	1	6		
		2	2	2	2	2		11/06/202
		1	1	2	2	1		27/12/202
		2	1	2	2			
				1				
		2	2	2	2	6		
		2	2	2	2			
		2	2	1	2	1		02/02/202
			1			6		
						6		
						1		20/02/202
						1		23/05/202
		2	2	2	2			
						1		19/05/202

		2	1	1	1	6		
						1		06/04/202
		2	1	1	1	1		10/04/202
		2	2	2	2	6		
		2	2	1	1	5	CONSOLID	24/02/202
		2	2		2	6		
		9	1	9	9	1		09/07/202
		2	1	2	2	6		
		2	2	2	2	1		
		2	2	2	2	1		10/01/202
		2	1	2	2	1		24/08/202
		1	1	2	2	1		25/02/202
		2	2	2	2			
		2	1	2	2	6		
		2	1	2	2	1		22/04/202
		2	2	2	2			
						1		19/06/202
		2	2	2	2	6		
						6		
		2	2	2	2	6		
		2	2	2	2			
784205	ALIMENTADOR DE LIN	2	1	1	1	9		
		2	2	2	2	1		12/08/202
		1	2	2	2	6		
		2	1	2	2	6		
				1	1			
		1	2	1	1	6		
						1		12/03/202
		2	2	2	2			
		2	2	2	2			
		1	1		1	1		
						9		
		2	2	2	2	1		26/05/202
		2	2	2	2	9		
			1	9	9	6		
						1		09/04/202
		2	2	2	2			
		2	2	2	2	6		
		2	2	2	2	1		25/09/202
		2	2	2	2	2		28/07/202
						1		
						1		31/10/202
		2	2	2	2	9		
						1		24/09/202

	1					
2	2	2	2	6		
2	2	2	2	3		31/05/202
1	1	2	2	6		
2	2	2	2	1		22/03/202
2	2	2	2	1		
2	1	1	1	1		03/04/202
				1		16/06/202
2	2	2	2	3		06/06/202
2	2	1	2	5	COM AREA	21/05/202
2	1	9	9	6		
2	2	2	2			
2	2	2	2	1		18/05/202
2	2	2	2			
2	2	2	2	1		06/06/202
2	1	2	2	1		22/08/202
2	2	2	2	6		
2	2	2	2	6		
2	2	2	2			
				1		22/03/202
2	2	2	2	1		17/07/202
2	2	2	2			
2	2	2	2	6		
2	2	2	2	1		07/03/202
1	2	2	2			
2	2	2	2	6		
2	2	2	2	6		
				6		
1	1					
9	9	9	9	5	10% ACOM	20/05/202
				1		
2	2	2	2	1		26/04/202
	1	1	1			
2	2	2	2	9		
2	2	2	2			
2	2	2	2	6		
1	1	2	2	6		
2	2	2	2	1		24/03/202
2	2	2	2	1		10/05/202
				9		
2	2	2	2	2		27/04/202
2	2	1	1			
2	1	2	2	1		01/06/202
2	1	2	2	5	AGUARDA	04/09/202

2	2	2	2	6	
2	2	2	2		
2	1	1			
2	1	2	2	1	
2	1	2	2	6	
2	2	2	2		
1	2	2	2		
2	2	9	9	1	24/05/202
2	1	2	2	6	
				1	13/06/202
2	1	2	2	6	
2	2	2	2	1	28/06/202
9	9	9	9		
2	2	2	2	6	
				1	
1	2	2	2	6	
2	2	2	2	1	
2	1	1	1	9	
				6	
2	2	2	2	1	16/02/202
2	2	2	2	1	
	1			1	09/01/202
2	2	2	2	6	
2	2	2	2	1	25/05/202
2	2	2	1	1	02/03/202
2	2	2	2	1	10/05/202
1	2	1	1		
2	2	1	1	6	
				1	15/08/202
2	2	2	2	1	16/07/202
2	2	2	2		
2	2	1	2	1	17/09/202
9	9	9	9	1	17/05/202
2	1	1	1	1	07/08/202
2	2	2	2	1	14/11/202
2	1	1	1	1	07/04/202
				6	
2	2	2	2		
2	2	2	2		
2		2	2		
1	1	2	2		
2	2	2	2	1	24/06/202
				9	
2	1	2	2	1	28/05/202

		2	1	2	2	1		02/08/202
		2	1	2	2	1		19/08/202
		1	1	2	2	6		
		2	1	2	2	1		31/03/202
		2	1	1	1	6		
		2	2	2	2	6		
						1		29/01/202
		1	2	2	2	1		21/08/202
		2	1	2	2			
		2	2	2	2	1		14/04/202
		2	2	1	1	6		
		2	2	9	9	1		
						6		
		2	2	2	2	6		
231205	PROFESSOR DA EDUC	2	1	2	2	9		
		2	1	2	2	6		
		2	2	2	2			
		2	2	2	2	1		08/08/202
		2	2	2	2	1		13/05/202
		2	2	2	2	6		
				1	1	1		22/03/202
		2	2	1	1	1		27/05/202
		2	1	2	2	6		
		2	1	2	2	1		25/06/202
		2	2	2	2	9		
				1	1	1		17/05/202
		2	1	2	2	1		18/06/202
		2	1	1	9	1		11/09/202
						1		17/03/202
		2	1	2	2	1		24/02/202
		2	2	1	1			
						2		24/03/202
		2	2	2	2	5	ACOMETEN	17/04/202
		2	2	2	2	1		08/09/202
		2	1	2	2	1		22/04/202
		2	2	1	1			
		2	2	2	2	6		
						9		
		2	1	2	2	1		30/07/202
		1	2	2	2	1		28/08/202

		2	2	2	2	1		06/08/202
		2	1	2	2	6		
		2	2	2	2	1		13/07/202
			1					
		2	1	2	2	6		
		2	2	2	2			
		2	2	1	2	1		31/03/202
		2	1	2	2	1		12/03/202
		2	2	2	2	6		
		2	2	1	2	1		
		2	1	1	2	6		
		2	2	2	2	6		
		2	2	2	2	1		04/09/202
		2	1	2	2			
		2	2	2	2	6		
		2	2	2	2	1		14/02/202
		2	2	2	2	1		20/04/202
		2	2	2	2	1		20/04/202
		2	1	2	2	6		
		2	2	2	2	1		23/03/202
322205	TECNICO D GATOS	2	1	2	2	6		
						5		26/05/202
		2	2	2	2	5	CONSOLID	05/07/202
		1	1	9	9	1		11/05/202
		1	2	2	1	1		02/09/202
		1	1	1	1	9		
		2	2	2	2	1		15/07/202
		2	2	2	2	1		04/07/202
		1	1	2	2			
		2	2	2	2			
		2	2	2	2	1		05/04/202
		9	2	2	1	9		
		2	2	2	2	1		01/05/202
			1					
		2	2	2	2	5	ACOMETIM	10/05/202
				1	1	1		19/05/202
		2	2	2	2	3		22/06/202
		2	2	2	2	6		
		2	2	1	2	6		
			1			1		03/05/202
		2	1	2	2	1		14/06/202
		1	1	2	2	1		13/04/202

		2	1	1	1	6	
		2	1	2	2	1	12/03/202
		2	2	2	2	1	06/03/202
		2	2	2	2	1	21/06/202
		2	2	2	2	6	
		2	1	2	2	6	
		2	2	2	2	1	03/06/202
		2	2	2	2		
						6	
						9	
		2	1	2	2	6	
		2	2	2	2	1	14/07/202
		2	2	2	2	6	
		2	2	9	9		
						1	01/08/202
						6	
				1	1	1	19/07/202
		2	2	2	2	1	28/07/202
		2	2	2	2	6	
			1			1	12/05/202
		2		2	2		
		2	1	2	2	1	29/10/202
		2	1	2	2	1	07/04/202
		2	2	2	2	6	
		2	1	2	2	1	08/04/202
		2	1	2	2	6	
		2	2	2	2		
		2	1	2	2	1	12/06/202
		2	2	2	2		
		2	2	2	2	6	
				1	1	6	
512115	EMPREGADO DOMEST	1	1	1	2	9	
		2	2	2	2	1	15/06/202
		2	2	2	2	3	21/05/202
		2	1	1	2	1	30/06/202
		2	2	2	2	6	
		2	2	2	2	9	
						1	26/03/202
						6	
		1				1	08/03/202
		2	1	2	2	2	
		2	1	2	2	6	
		2	1	2	2	6	
		2	2	2	2		
		2	1	2	2	1	27/07/202

		2	1	2	2	1	26/03/202
		1	2	2	2		
		2	2	2	2	1	25/09/202
		1	1	2	2		
		2	2	2	1		
		2	1	2	2		
			1			9	
141410	COMERCIANTE VAREJI	1	1	1	1	6	
		1	1	1	1		
			1			6	
					1	6	
		2	1	1	2		
		2	2	1	1	1	25/05/202
		2	1	2	2	6	
		2	2	2	2	1	
		2	1	2	2		
		2	2	2	2	1	27/04/202
		2	1	2	2	1	24/05/202
		2	1	1	2	1	26/03/202
		2	2	2	2	1	11/03/202
		2	2	2	2	3	18/03/202
		2		2	2	1	
		2	1	2	2		
		2	2	2	2	6	
		2	1	2	2	1	01/05/202
		2	1	1	1	1	05/09/202
		2	1	2	2	6	
			1	1	1	1	11/03/202
				1		1	
		2	2	2	2	6	
				1		6	
				1	1		
		2	2	2	2		
						1	09/03/202
		2	2	2	2	6	
		2	1	2	1	6	
		2	1	2	2	2	07/05/202
				1	1	1	12/05/202
		2	2	2	2		
		2	1	2	2	1	20/08/202
		2	2	2	2	6	

		2	1	2	2	6	
		2	1	9	9	1	
		2	2	2	2		
		2	1	1	1	1	29/03/202
			1			6	
		2	9	2	2		
		2	2	2	2		
				1	1	6	
		2	1	2	2		
						1	21/04/202
		2	2	2	1	1	13/10/202
		2	1	2	2	1	
		2	2	2	2		
		2	2	1	1		
322405	TECNICO EM SAUDE B	2	2	2	2	6	
			1	1	1	1	16/04/202
		2	2	2	2	1	26/03/202
		2	1	1	1	1	27/03/202
		2	2	2	2	1	11/06/202
		2	2	1	1		
		2	1	2	2		
						1	09/04/202
		2	1	2	2	1	08/11/202
						1	12/01/202
						6	
						1	17/03/202
		2	1	2	2	1	08/02/202
		1	2	2	2	6	
						1	17/09/202
			1			1	19/05/202
		9	9	9	1	1	23/03/202
		2	2	2	2	6	
						6	
						1	15/05/202
		9	9	9	9		
		2	2	2	2	6	
						6	
						1	
		2	2	2	2	9	
			1			1	01/04/202
		2	2	2	2	1	26/06/202
		2	1	1	1	6	
				1			
		2	1	2	1	1	22/03/202
		1	1	1	1	1	
		1		2	2	1	

		2	2	9	1	6	
		2	2	1	2	1	
						1	12/07/202
		2	2	2	2		
			1				
				1	2		
521110	VENDEDOR DE COMEF	2	2	2	2	1	25/06/202
						6	
						1	17/03/202
						1	09/07/202
			1			1	18/04/202
		2	1	2	2	1	23/06/202
						1	04/08/202
		2	1	9	9	6	
		2	1	9	9	1	
		2	2	2	2	1	21/06/202
		2	2	2	2	1	21/05/202
						9	
		2	2	2	2		
						6	
			1			3	06/08/202
				1			
		2	1	2	2	1	07/05/202
			1			6	
		2	2	2	2	6	
						9	
		2	2	2	2	1	06/05/202
		2	2	2	2	1	19/04/202
		1	1	2	2	1	07/05/202
						1	23/05/202
141115	GERENTE DE PRODUCA	2	1	2	2	1	22/06/202
		2	1	2	2	1	12/06/202
		2	2	2	2	2	20/04/202
		2	2	2	2		
		1	2	2	2	1	16/04/202
		2	1	2	2		
		2	1	1	1	6	
		2	2	2	2	1	18/02/202
				2	2	1	
				1	1	1	27/05/202
						9	
		2	2	2	2	2	26/01/202
						1	02/04/202

2	2	2	2			
2	2	2	2	1		06/06/202
2	2	2	2			
2	1	2	2	9		
2	1	2	2			
	1			1		30/04/202
2		9	9	2		
				1		03/06/202
2	1	1	2	9		
	1			1		11/05/202
2	1	2	2	5	OPACIDAD	11/07/202
2	2	2	2	1		19/05/202
				1		14/01/202
2	2	2	2	1		
2	2	2	2			
	1	2	2	6		
2	2	2	2	6		
				1		21/06/202
2	1	2	2	6		
2	1	2	2	1		24/08/202
				1		30/06/202
2	2	2	2			
2	2	2	2	6		
2	2	2	2	6		
				1		03/09/202
2	1	2	2	1		
2	2	2	2	1		24/07/202
2	2	2	2	9		
2	2	2	1	1		24/05/202
				1		01/06/202
				6		
1	1	1	2	1		18/03/202
				9		
				1		22/05/202
				1		02/05/202
				1		
2	1	2	2	6		
2	1	2	2	1		
2	1	2	2	1		02/05/202
2	2	2	2			
2	2		2			
				1		30/04/202
2	1	2	2	1		12/07/202
2	2	2	2	1		30/06/202

2	2	2	2			
2	1	2	2	6		
2	2	2	2	6		
				1		09/04/202
2	2	2	2	6		
			1	1		06/04/202
1				1		04/05/202
2	2	2	2	1		14/04/202
2	2	2	2	5	SOLICITAD	09/06/202
				1		31/05/202
				1		30/06/202
2	2	2	2	9		
2	2	2	2			
2		1	1	6		
1	2	2	2	6		
2	2	2	2	1		13/05/202
2	2	2	2	1		16/01/202
2	1	2	2	9		
2	1	2	2	6		
2	1	1	1	1		29/03/202
2	2	2	2			
2	2	2	2	1		19/05/202
		1	1	1		24/05/202
2	1	2	2	1		13/06/202
2	2	2	2	1		29/03/202
2	2	1	2			
2	1	2	2	1		09/05/202
2	1	2	2			
				1		08/04/202
				6		
2	2	2	2			
2	2	2	2			
2	2	2	2	1		20/03/202
2	1	2	2			
2		1	2	6		
2	2	2	2			
2	2	2	2	6		
				1		11/08/202
2	1	1	1	1		07/03/202
				1		10/04/202
				1		14/01/202
2	2	2	2	1		30/04/202
2	2	2	2	6		
				1		17/05/202
2	1	2	2	1		22/11/202
9	9	9	9	9		

2	2	2	1	1		01/05/202
9	2	2	2	1		02/08/202
				1	80%	01/06/202
				1		23/11/202
2	1	2	2			
2	2	2	2	9		
2	2	2	2	6		
2	1	2	2	1		28/06/202
				1		05/03/202
2	1	2	2			
2	2	2	2	1		03/10/202
2	2	2	2	9		
2	2	2	2	1		22/01/202
2	1	1	2	1		09/05/202
2	2	2	2	1		24/05/202
				6		
	2	2	2			
2	1	2	2	1		29/05/202
2	2	2	2	1		14/04/202
2	2	2	2	6		
2	1	2	2	1		14/04/202
2	2	2	2	1		26/05/202
2	2	2	2	6		
2	2	2	2	1		19/08/202
2	2	2	2	1		17/09/202
2	2	2	2			
2	1	2	2	1		01/07/202
2	2	2	2	1		12/06/202
2	2	2	2	6		
2	2	1	1	1		
1	2	2	2	1		07/03/202
				1		
2	2	2	2			
	1			5	MISTO	20/05/202
2	2	2	2	1		10/07/202
				1		28/05/202
2	2	2	2	6		
				3		
1	2	2	2	1		14/08/202
2	2	2	2			
2	2	2	2	6		
	1	1	1	2		15/05/202
2	2	2	2			
2	2	2	2			
2	1	2	1	1		27/04/202

		2	2	2	2	1	18/06/202
		2	1	2	2	1	10/06/202
		2		1	1		
421125	OPERADOR DE CAIXA	2	2	2	1	1	14/11/202
		2	2	2	1	9	
		9	1	1	1		
		2	2	2	2	6	
		2	1	2	2	1	
		2	2	2	2		
		2	2	1	2	1	28/03/202
		2	1	2	2	1	02/06/202
		2	2	2	2	6	
						1	25/04/202
		2	1	2	2	1	07/06/202
		1	1	1	1	1	29/05/202
		2	2	1	1	6	
		2	1	2	2	6	
		2	2	2	2	6	
421125	OPERADOR DE CAIXA	2	1	1	1	6	
225250	MEDICO GINECOLOGIS	2	2	2	1	1	02/03/202
		9	9	9	9		
						6	
						1	25/04/202
					1	1	05/05/202
		2	2	1	1	1	29/04/202
		2	2	2	2		
		2	1	1	1	6	
		2	1	1	1	1	07/07/202
		2	2	1	1	1	25/03/202
		2	2	2	2	1	
					1		
		2	2	2	2		
		2	2	2	2	1	27/02/202
		2	2	2	2		
		2	1	2	2	6	
						6	
						6	
		2	2	2	2	6	
		2	1	1	2	1	09/03/202
		2	2	2	2	5	
			1			1	29/03/202
		2	2	2	2	6	

2	2	1	1	6		
2	2	2	2	6		
2	1	1	2	1		07/07/202
2	2	2	2	1		27/03/202
				1		15/05/202
9	9	9	9	1		12/05/202
				1		20/03/202
1	1	1	1	1		15/01/202
2	2	2	2	6		
				1		05/06/202
2	2	2	2	1		12/04/202
				9		
2	2	2	2	6		
2	2	2	2	9		
				6		
2	2	2	2	1		20/05/202
2	1	1	2			
2	1	1	1	1		30/07/202
	1			1		
2	2	2	2	6		
2	1	1	1	6		
2	2	2	2			
2	1	2	2	1		19/03/202
2	2	2	2	6		
2	2	2	2	6		
2	1	2	2	9		
2	1	2	2	1		21/03/202
				6		
2	2	2	2	1		10/03/202
				6		
2	2	2	1	1		08/07/202
2	2	2	2	5	DERRAME	23/01/202
2	2	2	2			
9	9	9	9	2		02/04/202
1	1	2	2	6		
2	2	2	2	6		
				5	METASTAS	02/03/202
2	2	2	2	6		
2	2	2	2	6		
				6		
2	2	2	2	1		01/05/202
				1		06/05/202

		2	2	2	2		
		2	1	2	2	1	04/08/202
						6	
		2	1	2	2	1	15/02/202
		2	1	2	2	6	
						1	03/03/202
			1		1	1	21/02/202
			1			6	
		2	2	2	2		
						1	07/07/202
		2	2	2	1	6	
			1		1		
		2	2	2	2	1	09/06/202
						1	09/08/202
		2	2	1	1		
				1	1		
		2	1	2	2	6	
		2	2	2	2	6	
		2	2	2	2	1	08/06/202
		2	2	2	2	1	02/06/202
		2	2	2	2	6	
						1	27/03/202
513435	ATENDENTE DE LANCH	2	2	2	2	1	10/06/202
		2	2	2	2	1	15/03/202
		2	1	2	2	1	11/02/202
		2	2	2	2	6	
		2	2	2	2	6	
		2	2	2	2	6	
		2	2	2	2	6	
				1	1		
						1	04/05/202
		2	2	2	2	1	26/08/202
						1	10/04/202
		2	1	2	2	6	
		2	2	2	2	1	13/03/202
		2	1	2	2	6	
						6	
						6	
		2	1	2	2	1	22/09/202
		2	1	2	2	6	
		2	2	2	2		
		2	2	2	2	1	03/04/202

						9	
		2	1	1	1	1	26/04/202
				1	1	6	
				2	2	1	
		2	1	1	1	6	
						1	20/06/202
		2	2	2	2	6	
		2	2	2	2		
						6	
		2	1			1	
		2	1	2	2	1	16/12/202
		2	1	2	2	1	29/04/202
			1			6	
						1	19/05/202
			1			1	26/03/202
		2	2	2	2	6	
		2	1	2	1	1	12/04/202
						1	02/07/202
						1	17/05/202
631210	PESCADOR PROFISSIO	2	1	1	2	1	18/07/202
		2	2	2	2	6	
		2	2	2	2		
		2	2	2	2	9	
		2	1	2	2	1	12/05/202
		2	2	2	2	6	
		2	2	2	2	6	
		2	2	2	2		
		2	1	2	2		
		2	2	2	2		
		2	2	1	1	1	10/02/202
		2	2	2	2	9	
						4	23/09/202
		2	2	2	2	6	
				1	1	6	
				1			
		2	2	2	2		
		2	2	2	2		
		2	2	2	2	9	
		9	9	9	9		
		2	2	2	2	9	
		2	1	2	2	1	03/02/202
						6	

		2	1	2	2	6	
		2	1	2	2	6	
		2	2	2	2	6	
		2	2	2	2	1	06/05/202
		2	2	2	2	1	23/05/202
		1	1	2	2		
		2	1	2	2	1	15/07/202
				1	1	6	
				1		6	
		2	2	2	2	1	13/07/202
		2	1	2	2		
						6	
		2	1	2	2	1	22/07/202
		2	2	2	2	1	29/01/202
		2	1	2	2	1	02/10/202
			2	2	2	6	
		1	1	1	1	6	
						1	20/03/202
						1	03/06/202
		2	2	2	2	1	07/04/202
		2	1	1	2	1	30/04/202
		2	2	2	2	6	
		2	2	2	2	6	
			1			1	23/03/202
		2	2	2	2		
		2	2	2	2	6	
		2	1	2	2	1	25/05/202
		2	1	2	2	1	28/07/202
		2	2	2	2	1	07/04/202
					1		
		2	2	2	2	1	15/04/202
521110	VENDEDOR DE COMEF	2	1	2	2	6	
		2	2	9	1	1	03/04/202
						1	28/01/202
		2	2	2	2	6	
		2	2	2	2		
		2	2	2	2	2	30/06/202
		1				6	
		2	2	2	2	6	
		2	1	2	2		
			1				
		1		1	1	1	27/12/202
		2	2	2	2	2	29/03/202
		9	9	9	9	6	

2	2	2	2	1		12/06/2021
2	2	1	1	1		04/03/2021
2	1	2	2	1		27/05/202
		1	1	1		25/01/2021
1	1	2	2	1		12/06/2021
2	2	2	2	9		
2	1	2	2	1		
9	1	2	1	1		13/03/2021
2	2	2	2	1		10/04/2021
2	1	2	2	1		25/06/2021
2	2	1	1			
2	1	2	2	6		
2	2	2	2	1		28/02/2021
2	2	1	1	1		14/03/2021
2	2	1	1	9		
2	2	2	2	1		17/06/202
2	2	2	2	6		
	1			6		
2	1	2	2	1		31/05/2021
2	2	2	2	1		09/06/2021
	1	2	2	1		
9	1	9	9	5	ACOMETIM	24/03/2021
2	1	2	2	1		
	1			1		07/05/2021
		1	1	1		20/06/2021
2	2	1	2			
2	1	2	2	6		
				6		
				9		
2	1	2	2			
2	2	2	2	1		21/06/2021
2	2	2	2	1		
2	2	1	1	6		
2	1	2	2	1		20/07/2021
2	2	2	2	9		
2	1	2	9	9		
9	9	9	9	1		21/03/2021
	1	1		6		
2	1	2	2	1		29/06/202
1		2	2			
2	2	1	2			
2	2	2	2	6		
1	1	2	2	6		

		2	2	2	2			
		9	9	9	9			
		2	2	2	2	6		
		2	2	2	2	1		26/04/202
		2	1	2	2			
				1		6		
		2	1	2	2	6		
		2	1	2	2	6		
		2	1	2	2	6		
		1	1	1	1	1		
		2	2	2	2	1		
				1	1	1		28/06/202
		2	2	2	2			
		2	2	2	2	6		
		2	2	2	2	1		18/05/202
		2		9	9	6		
		2	1	2	2	1		16/01/202
		2	1	2	2	1		31/03/202
		2	2	2	2			
		2	2	2	2	1		14/06/202
		2	2	2	2	1		31/07/202
		2	1	2	2	6		
		2	1	2	2	6		
		2	2	2	2	6		
				1				
						5	INFILTRAD	02/04/202
		2	2	2	2	6		
		2	2	2	2			
		2	1	2	2	1		22/03/202
223505	ENFERMEIRO	2	1	1	1	6		
						1		09/05/202
						1		12/05/202
		2	1	2	2	1		18/06/202
						9		
		2	2	2	2	5	60% ACOM	12/05/202
		2	2	2	2	6		
						1		11/08/202
		2	1	2	2	1		24/03/202
		2	2	1	1	6		
		2	1	2	2	6		
		2	1	1	1	6		
				1	1	1		01/08/202
		2	2	1	1			
				1	1	1		08/03/202

		2	1	2	2		
					1		
		2	2	2	2		
		2	1	2	2		
		2	2	2	2	6	
						1	
		2	2	2	2	6	
		2	1	1	1	1	16/03/202
						1	
		2	1	2	2	6	
		2	2	2	2		
		2	2	2	2	6	
		2	2	2	2		
		2	2	2	2	6	
		2	2	2	2		
						1	
		2	2	2	2	1	25/05/202
		2	1	1	1	1	27/04/202
		2	2	2	2	6	
		2	1	2	2	9	
		1	1	1	1		
						1	12/03/202
		2	1	2	2	1	25/04/202
		2	2	1	1	6	
		2	2	2	2		
		2	2	1	2	1	01/04/202
		2	2	2	2	1	
			1			1	14/07/202
		2	1	2	2	6	
		2	2	2	2	1	13/04/202
		2	1	2	2	1	24/05/202
						9	
		2	2	2	2	1	03/07/202
		2	1	1	1		
		2	2	2	2	6	
						1	01/07/202
239415	PEDAGOGO	2	1	2	2	1	19/05/202
		2	2	2	2		
				1	1	6	
		2	2	1	1	1	28/09/202
		2	2	2	2	6	
						1	
			1			1	25/06/202
		2	2	2	2		

						1		13/03/2021
		2	2	2	2	1		22/02/2021
						1		10/06/2021
						1		
		2	1	2	2	1		12/06/2021
		2	2	1	2	6		
		2	2	2	2	6		
			1		1	1		30/03/2021
		2	1	2	2			
325110	TECNICO EM LABORATORIO DE FA		1			1		18/04/2021
		2	1	2	2			
		1	2	2	2	6		
			1	1		1		19/07/2021
		2	2	2	2	3		21/05/2021
						6		
		2	1	2	2			
		2	2	2	2			
		2	1	2	2			
622020	TRABALHADOR VOLAN	2	2	2	2			
						1		27/05/2021
		2	1	2	2	9		
		1	1	1	1	1		02/04/202
		2	2	2	2	5	CONSOLID	18/03/202
						6		
		2	1	2	2	6		
						1		03/06/2021
		2	1	1	2	9		
		1	2	2	2	1		10/06/2021
		2	2	2	2			
		2	2	2	1	1		10/06/2021
			1					
		2	2	2	2	2		24/05/202
						1		28/07/2021
		2	2	2	2	1		23/07/2021
		2	2	2	2	1		06/04/2021
		2	2	2	2	6		
322205	TECNICO DE ENFERMA	2	2	2	2	1		08/04/2021
		2		9	9	6		
		2	1	2	2	6		
		2	2	2	2	9		
						6		
		2	9	9	9	6		
521110	VENDEDOR DE COMER	2	2	2	2	1		23/04/2021

		2	2	2	2	9	
		2	1	2	2	6	
		9	9	9	9	9	
		2	2	2	2	6	
		2	1	2	2	6	
		2	2	2	2	1	18/02/202
		2	1	2	2		
		2	2	2	2	9	
		2	1	2	2	6	
						1	25/04/202
			2	2	2	1	13/05/202
		2	2	2	2	6	
		2	2	1	1		
		2	2	2	2	9	
		2	1	2	2	1	10/04/202
		2	2	2	2	6	
						6	
		2	1	1	1	1	01/06/202
		2	1	2	2		
						6	
			1			1	17/05/202
						1	09/03/202
		2	1	9	2		
		2	2	2	2	1	31/03/202
						1	06/06/202
		2	2	2	2	1	07/03/202
		2	2	2	2	6	
		2	2	2	2		
		2	2	2	2		
		2	2	2	2	6	
		2	2	2	2	1	30/05/202
						4	14/06/202
						1	20/07/202
		9	9	9	9	1	18/02/202
		2	2	2	2	1	01/04/202
		2	2	2	2	6	
						6	
		2	1	2	2		
		2	2	1	1	1	27/05/202
						6	
622020	TRABALHADOR VOLAN	2	2	2	2	1	04/06/202
		2	1	2	2		
		2	2	2	2		
		2	2	2	2		

		2	2	2	2	1	21/06/202
		2	2	2	2	6	
						6	
		2	1	2	2	6	
		2	2	2	2	9	
		2	2		2		
		2	2	2	2	6	
521130	ATENDENTE DE FARMA	1	1	1	1	9	
		2	2	1	2	6	
		2	1	2	2	1	09/07/202
		2	2	2	2		
		2	9	2	2	1	
		2	2	2	2	1	24/07/202
						6	
			1	1			
		2	2	2	2	6	
		2	1	2	2	1	16/04/202
		2	2	2	2	1	04/05/2021
			1				
		2	2	2	2	1	13/05/2021
		2	1	1	1		
		1	1	2	2	6	
		2	2	2	2	1	26/09/2021
		2	1	2	1	1	11/04/2021
		2	1	2	2	1	24/02/2021
						6	
		2	2	2	2	6	
		2	2	2	2	6	
		2	2	2	2	1	27/05/2021
		2	2	9	9	1	21/03/202
						1	23/11/202
		2	2	2	2	6	
						1	
		2	1	2	2	1	18/03/202
		2	1	1	1	1	02/03/2021
		2	2	2	2	6	
		2	1	2	2	1	23/04/2021
		2	1	2	2	1	15/11/2021
		2	2	2	2	1	18/03/202
		2	2	2	2	1	29/05/202
		2	2	2	2	1	25/07/202
		2	2	2	2	6	
		2	2	2	2	1	25/06/2021
		1	1	2	2	4	14/01/2021
		2	1	2	2	1	18/03/2021
		2	2	2	2		
		2	2	2	2	6	

		1		1	1	6		
						1		29/04/202
		2	2	2	2	9		
		2	2	2	2			
		2	1	2	2	1		06/08/202
		2	2	2	2			
		2	2	2	2			
		2	2	2	2	1		13/03/202
		2	2	2	2	6		
		2				6		
			1			1		19/03/202
		2	2	2	2	1		16/04/202
		2	2	2	2			
		2	1	2	2	1		12/08/202
		2	1	2	2	9		
		2	1	2	2	6		
		9	9	9	9	9		
		2	2	1	1	5	CONSOLI D	05/03/202
						1		15/03/202
		2	2	2	2			
				2	2	1		
251605	ASSISTENTE SOCIAL					1		
		2	1	2	2	1		15/06/202
		2	2	2	2	1		13/08/202
		2	2	2	2	1		20/05/202
		2	2	2	2	6		
		2	2	2	2	6		
		1	1	2	2	1		17/06/202
		2	2	2	2	1		24/05/202
						6		
		2	2	2	2			
		2	1	2	2	5	AGUARDAI	12/04/202
		2	1	2	2			
		2	2	2	2	6		
		2	2	2	2	1		03/03/202
		2	2	2	2	1		20/02/202
		2	2	2	2	6		
		2	2	1	2			
		1	9	9	9			
		2	2	2	2	6		
						1		08/02/202
		2	2	2	2	1		05/02/202
		2	2	2	2	1		03/04/202

2	2	2	2	1	19/04/2021
1				6	
1	1	2	2	6	
1	1	2	2	1	26/03/2021
		1	1	6	
2	2	2	2	6	
2	1	2	2	6	
2	1	2	2	9	
2	1	2	2	1	03/04/202
2	2	2	2	2	28/05/2021
				6	
2	2	2	2	1	26/02/2021
2	2	2	2		
				1	28/03/2021
				6	
1	2	2	2		
2	2	2	2		
1	2	2	2	1	22/08/2021
2	1	2	2	1	12/01/2021
2	2	1	1		
2	1	2	9	1	04/03/2021
1	2	1	1	6	
2	2	2	2	6	
2	2	2	2	6	
2	2	2	2	1	04/09/2021
2	2	2	2	6	
	1			1	02/04/2021
1	1	2	2	1	26/04/2021
				6	
2	1	2	2	1	06/03/202
2	1	2	2	1	06/02/2021
				6	
2	2	2	2	1	12/04/2021
2	2	2	2	1	02/05/202
2	2	2	2	9	
				9	
2	2	2	2	1	25/05/2021
2	2	2	2	6	
2	2	2	2	1	
	1	1	1	1	18/06/202
2	1	2	2	9	
2	2	1	1		
2	1	2	2	6	
2	2	2	2		
1	1	2	2	1	30/03/2021

		2	2	2	2	2		15/05/202
		2	2	2	2	1		06/04/202
		2	1	2	2	1		11/06/202
						6		
			1			6		
		2	2	2	2			
		2	2	2	2			
		2	1	2	2	1		05/05/202
		2	1	2	2	1		30/06/202
		2	2	2	2	1		
		1	1	9	9	1		11/06/202
		2	2	2	2	1		
						6		
		2	2	2	2	1		21/03/202
		2	2	2	2			
		2	2	2	1	6		
		2	1	2	2	6		
		2	2	2	2	1		06/08/202
						1		06/04/202
		2	2	2	2	1		25/02/202
		2	2	2	2	6		
		2	1	2	2	1		25/04/202
						1		12/02/202
		2	2	2	2	1		15/02/202
		2	2	2	2	2		26/04/202
		2	2	2	2	1		08/07/202
		2	1	2	2			
		2	2	2	2	6		
						6		
		2	2	2	2	1		30/05/202
239415	PEDAGOGO	2	2	2	2	6		
		2	2	2	2	5	VIDRO FOS	08/03/202
521135	FRENTISTA	2	2	2	2	5	COVID + IN	27/03/202
		1	2	2	2	1		10/05/202
		2	1	2	2	1		13/06/202
		2	2	2	2	1		13/04/202
		2	2	2	2	1		01/04/202
		1	1	2	2			
		2	2	2	2			
		2	2	2	2	6		
		2	2	1	1	1		08/04/202
		2	1	2	1	6		
						1		31/05/202

2	2	2	2	6		
2	1	9	9	6		
				6		
				1		29/05/2021
	1			6		
2	1	2	2	1		21/07/202
2	2	2	2	1		17/01/2021
2	2	2	2	5		12/04/2021
				1		26/02/2021
2	2	2	2			
	1			6		
2	1	2	2	3		25/08/2021
2	2	2	2	6		
1	2	1	1	1		30/03/2021
	1			1		21/05/2021
		1	1			
1	2	2	2	6		
2	2	1	1			
2	2	2	2			
2	2	2	2	1		26/07/2021
2	2	2	2	6		
2	2	1	1	1		22/02/2021
2	2	2	2	1		03/04/2021
	2	2	2	6		
2	2	2	2	6		
2	1	1	1	1		24/05/202
2	2	2	2			
2	2	2	2	6		
	1			1		06/07/202
				1		16/04/2021
2	2	2	2			
2	2	2	2	1		15/06/202
2	2	2	2	1		04/06/2021
2	2	2	2	6		
1	1			6		
2	2	1	1			
2	1	2	2			
2	1	2	2	5	SEM LAUDO	16/08/2021
				1		17/03/2021
2	2	2	2	6		
2	1	1	2	1		30/03/2021
2	2	2	2	9		
2	1	1	2	1		10/02/2021
				9		

2	2	2	2	1		19/04/202
2	1	1	9	6		
				6		
2	2	2	2			
	1					
2	2	2	2			
2	2	2	2			
2	2	2	2			
2	1	1	1	6		
2	2	2	2	6		
				9		
2	2	2	2	5	HEMORRA	28/03/202
2	2	2	2	1		19/03/202
2	2	2	2	1		10/04/202
2	2	2	2	6		
				6		
1	1					
2	2	2	2	1		
2	2	2	2			
2	1	2	2	1		23/05/202
				1		27/05/202
2	1	1	2			
2	2	2	2	2		30/09/202
				6		
	1	1	1	6		
2	2	2	2	9		
2	2	2	2			
2	2	2	2	6		
1	2	2	2	6		
2	2	1	1	1		
1		9	1	1		
2	2	2	2	1		02/06/202
2	2	2	1	6		
2	1	2	2	1		06/03/202
2	2	2	2	6		
				6		
2	2	2	2			
				9		
	1	1	1	1		20/04/202
				1		
1	1	2	2	1		27/05/202
				6		
				6		
2	2	1	1	6		

		2	2	2	2	1		29/04/202
		2	2	2	2	4		17/09/202
		2	2	2	2	6		
		1	1	1	2	1		10/07/202
		2	2	2	2	6		
						1		19/04/202
		2	1	2	2			
						1		14/06/202
		2	1	9	9	1		01/07/202
		2	2	2	2	6		
		2	2	2	2	6		
		2	2	2	2	6		
			1	2	2	6		
		2	2	2	2			
421125	OPERADOR DE CAIXA	2	1	1	2	1		16/02/202
		2	2	2	2	5	SEM LAUDO	
		2	2	2	2	1		30/03/202
		2	2	2	2			
						1		10/03/202
						6		
		2	1	2	2	6		
		1	2	2	2	1		04/03/202
		2	2	2	2	6		
		2	1	2	2	1		11/08/202
		2	2	2	2	1		31/05/202
				1		1		22/03/202
		2	2	2	2			
						1		17/03/202
		2	2	2	2	1		29/03/202
		2	1	2	2	1		17/05/202
						1		10/06/202
		2	1	2	2	1		06/07/202
						1		24/03/202
		2	2	2	2	6		
				1		1		
		2	2	2	2	1	-	07/08/202
		2	2	2	2	6		
		1	1	2	2	1		17/07/202
		2	2	2	2	6		
			1					
						1		28/06/202

		2	2	2	2	1		
		9	1	9	9	6		
		2	1	2	2	6		
		9	9	9	9	1		09/06/202
						1		09/03/202
		2	2	2	2	1		22/03/202
		2	2	1	1			
						1		09/04/202
				1	1	1		30/06/202
						1		10/02/202
		2	2	2	2			
						1		17/03/202
			1					
		2	2	2	2	1		
				1				
						1		13/03/202
		2	2	2	2	6		
		2	2	2	2			
		2	2	2	2	6		
						1		16/03/202
142105	GERENTE ADMINISTRA	1	2	2	2	1		12/02/202
			1			1		30/05/202
						1		16/07/202
		2	1	2	2	1		06/08/202
		2	2	2	2	6		
		2	1	1	2			
		2	1	2	2	1		23/04/202
						1		26/05/202
		2	2	2	2	6		
		2	2	2	2	1		18/05/202
		2	1	2	2	1		03/11/202
						6		
		2	2	2	2	5		13/05/202
		2	2	2	2	6		
		2	2	2	9	1		28/12/202
		2	2	2	2	1		10/03/202
		2	1	1	1			
		2	2	2	2	6		
		2	2	2	2			
				1	1	1		
		2	2	2	2	1		14/09/202
		2	2	2	2	1		02/08/202
		2	2	2	2	5	OPACIDAD	26/05/202
			2			5	VIDRO FOS	24/10/202
		2	1	2	2			

						1		08/04/202
		2	2	2	2	6		
		2	1	2	2	1		24/05/202
		2	1	2	2	1		23/07/202
		2	2	2	2	6		
						1		31/03/202
			1			1		07/04/202
		2	2	2	2	6		
		2	2	1	2	1		07/04/202
		2	1	2	9	1		25/04/202
		2	1	2	2	1		05/08/202
			1					
						6		
		1	1	1	1	1		19/03/202
						6		
						6		
514320	FAXINEIRO	2	1	2	2	9		
		2	2	2	2	1		04/04/202
						1		03/09/202
						1		07/07/202
						1		19/03/202
						1		10/03/202
		2	2	2	2			
		2	1	2	2	3		29/06/202
		2	2	2	2	1		27/05/202
		2	1	2	2	1		09/01/202
		2	2	2	2			
		2	1	2	2	1		24/02/202
		2	1	2	2			
		2	1	2	2	1		24/07/202
		2	2	2	2	6		
		2	1	2	2	1		04/03/202
		2	2	2	2	1		02/05/202
			2	2	2			
		2	1	2	2	1		
		2	1	2	2	6		
		2	2	2	2			
						1		29/04/202
		2	2	2	2	1		10/06/202
		2	2	1	1	6		
			1			5	VIDRO FOS	30/09/202
			1		1	6		
		1	2	2	2	3		03/01/202
		9	9	9	9			
		2	2	2	2	1		01/02/202

2	2	2	2	2		23/02/202
2	1	2	2	6		
2	2	2	2			
2	2	2	2	6		
2	2	2	2	6		
1	1	2	2	5	EM ANDAN	23/01/202
2	2	2	2			
		1		1		04/02/202
2	1	2	2			
				6		
				1		17/01/202
				1		
2	2	2	2			
				1		26/01/202
2	2	2	2	6		
2	2	2	2	6		
2	2	2	2			
2	2	2	2	6		
9	9	9	9			
2	2	2	2			
				9		
2	2	2	2			
2	1	2	2	6		
				6		
2	2	2	2			
2	2	2	2			
2	9	2	2			
2	1	2	2			
2	2	2	2	6		
2	2	2	2	6		
2	2	2	2			
2	2	2	2	1		
2	1	2	2			
9	9	9	9	6		
2	2	2	2			
				6		
2	2	2	2	6		
2	2	2	2	1		10/01/202
2	2	2	2	1		17/01/202
				6		
2	1	2	2			

2	2	2	2	6	
2	2	2	2	6	
2	1	2	2	6	
2	2	2	2		
1	2	2	2		
				6	
				9	
1	2	2	2	6	
2	1	2	2	6	
				1	16/01/202
2	2	2	2		
				9	
				6	
1				6	
				6	
2	1	2	2	1	08/02/202
			1	6	
2	2	2	2	3	06/01/202
2	2	2	2	1	17/01/202
2	2	2	2	1	20/02/202
2	1	2	2	6	
	1				
2	2	1	1	2	15/01/202
				6	
2	2	2	2		
1	2	2	2	6	
				1	30/01/202
2	2	2	2		
				6	
2	2	2	2	6	
				6	
2	1	2	2	6	
2	1	2	2	6	
2	2	2	2	6	
2	1	2	2	1	21/02/202
2	2	2	2	9	
2	2	2	2	6	
		1			
2	1	2	2	6	
2	2	2	2	6	

141205	GERENTE DE PRODUC	2	1	2	2	6	
		2	2	2	2		
		9	9	9	9	1	14/01/202
						6	
		2	2	2	2	6	
		2	2	1		6	
		2	2	2	2	6	
		2	1	2	2	9	
		2	1	2	2	6	
		1	2	2	2	6	
		2	2	2	2	1	20/01/202
		2	1	1	1	1	09/02/202
		2	1	2	2	6	
		2	1	2	1	1	14/01/202
			1			6	
		2	2	2	2	1	23/01/202
		2	2	2	2	6	
						1	15/01/202
		2	2	2	2	6	

	TP_TES_AN	DT_RES_AN	RES_AN	POS_AN_FL	TP_FLU_AN	POS_AN_OU	AN_SARS2	AN_VSR	AN_PARA1	AN_PARA2
0										
			5							
			5							
	1		5							
			4							
			5							
			4							
			5							
			5							
			5							
			5							
	2	18/01/202	2							
0			5							
			4							
	1		4							
	1	10/07/202	1			1				
	1									
0			4							
0			5							
0			5							
	1		5							
	1		4							
	1		5							
	1		5							
[truncated: 525,374 more chars]
