# Supplementary material for: Factors associated with COVID-19 death in pregnant women hospitalized in Intensive Care Units
Source: Rev Bras Enferm. 2024 Aug 26;77(5):e20230172. doi: 10.1590/0034-7167-2023-0172 (PMC11346909; doi:10.1590/0034-7167-2023-0172)
Supplement: Translation

# Translation: Fatores associados ao óbito por COVID-19 em gestantes hospitalizadas em Unidade de Terapia Intensiva

**DOI:** 10.1590/0034-7167-2023-0172pt

**Authors:** Milena Ricioli Ribeiro, Marcela de Andrade Pereira Silva, Leticia Furlan de Lima Prates, Rosana Rosseto de Oliveira, Maria Dalva de Barros Carvalho, Sandra Marisa Pelloso

**Affiliations:** I Universidade Estadual de Maringá. Maringá, Paraná, Brasil; II Centro Universitário Ingá. Maringá, Paraná, Brasil

**Keywords:** COVID-19, Mortalidade Materna, Unidades de Terapia Intensiva, Síndrome Respiratória Aguda Grave, Gestantes

## Abstract

**Objetivos:**

avaliar os fatores associados ao óbito por COVID-19 em gestantes hospitalizadas em Unidade de Terapia Intensiva no Brasil.

**Métodos:**

este estudo ecológico foi realizado com dados secundários de gestantes brasileiras com COVID-19 hospitalizadas em Unidade de Terapia Intensiva, entre março de 2020 e março de 2022. Empregou-se análise univariada e regressão logística.

**Resultados:**

de 3.547 gestantes com COVID-19 hospitalizadas em Unidade de Terapia Intensiva, 811 evoluíram a óbito (22,8%). Evidenciou-se que a não vacinação contra a COVID-19 (OR: 2,73; IC_95_: 1,83;4,04), dispneia (OR: 1,73; IC_95_:1,17;2,56), obesidade (OR: 1,51; IC_95_: 1,05;2,17), doença cardiovascular crônica (OR: 1,65; IC_95_: 1,14;2,38) e a raça/cor não branca (OR: 1,29; IC_95_: 1,00;1,66) associaram-se de forma independente e significante ao óbito.

**Conclusões:**

conclui-se que o estado vacinal, presença de comorbidades e características clínicas e étnico-raciais estão associadas ao óbito por COVID-19 em gestantes hospitalizadas em Unidade de Terapia Intensiva no Brasil.

## INTRODUÇÃO

A COVID-19, doença causada pelo novo coronavírus (SARS-CoV-2), foi identificada pela primeira vez em dezembro de 2019, na cidade de Wuhan, China^(1)^. Em razão de sua alta transmissibilidade e rápido aumento no número de casos, a Organização Mundial da Saúde (OMS) declarou, em 30 de janeiro de 2020, que o surto do novo coronavírus constituía uma Emergência de Saúde Pública de Importância Internacional e, em 11 de março de 2020, anunciou que a situação havia evoluído para uma pandemia^(2-3)^.

Em 5 de maio de 2023, a OMS declarou fim da Emergência de Saúde Pública de Importância Internacional referente à COVID-19. Em cerca de três anos, o mundo contabilizou 765 milhões de casos confirmados da doença e quase 7 milhões de óbitos. O Brasil ocupa a segunda posição mundial em números absolutos de óbitos, atrás apenas dos Estados Unidos^(4)^.

Foram evidenciados alguns fatores de risco para evolução grave da COVID-19, como idade avançada, sexo masculino e comorbidades como hipertensão, diabetes *mellitus* e doenças cardiovasculares^(5)^. Quanto à população obstétrica, estudos recentes demonstram uma taxa aumentada de admissão em Unidade de Terapia Intensiva (UTI), complicações obstétricas e mortalidade em mulheres grávidas com COVID-19, evidenciando que elas são consideradas grupo de risco para síndrome respiratória aguda grave (SRAG) pelo SARS-CoV-2^(6)^.

Uma pesquisa publicada em julho de 2020 mostrou que 77% das mortes maternas por COVID-19 no mundo ocorreram no Brasil. Entre março de 2020 e maio de 2021, o país registrou 3.291 mortes maternas, das quais 1.352 mortes foram além do esperado, o que correspondeu ao excesso de 70% de óbitos maternos no país, a maioria atribuída à COVID-19^(7)^. Idade materna avançada, alto índice de massa corporal, etnia não branca, comorbidades pré-existentes e distúrbios específicos da gestação, como diabetes gestacional e pré-eclâmpsia, têm sido apontados como fatores de risco para uma evolução grave da doença em gestantes^(8)^.

No entanto, os fatores associados a complicações e a evolução grave da COVID-19 em gestantes ainda não foram completamente compreendidos. Mesmo perante um aumento significativo de publicações no mundo sobre a relação entre essa doença e a gestação, ainda há escassez de estudos envolvendo a população obstétrica brasileira.

## OBJETIVOS

Avaliar os fatores associados ao óbito por COVID-19 em gestantes hospitalizadas em Unidade de Terapia Intensiva no Brasil.

## MÉTODOS

### Aspectos éticos

Por se tratar de dados de domínio público de acesso irrestrito, sem a identificação dos sujeitos envolvidos na pesquisa, conforme a Resolução nº 510/16 do Conselho Nacional de Saúde (CNS), não houve a necessidade de apreciação pelo Comitê Permanente de Ética em Pesquisa com Seres Humanos, assim como dispensou-se o Termo de Consentimento Livre e Esclarecido^(9)^.

### Desenho e local de estudo

Este estudo ecológico e retrospectivo foi realizado com dados secundários, tendo como fonte o Sistema de Informação da Vigilância Epidemiológica da Gripe (SIVEP-Gripe). Esses dados foram disponibilizados pelo Ministério da Saúde (OpenDATASUS) e referiram-se a casos e óbitos de gestantes brasileiras com COVID-19 hospitalizadas em UTI. O estudo foi construído com base na ferramenta STROBE (*Strengthening the reporting of observational studies in epidemiology*).

### Período e critérios de seleção

Este estudo incluiu todos os casos notificados entre março de 2020 e março de 2022 que atenderam aos seguintes critérios: gestante brasileira, com idade entre 10 e 49 anos, diagnosticada com infecção por SARS-CoV-2, hospitalizada em UTI e recuperada ou falecida. Foram excluídos os casos de gestantes estrangeiras e aqueles que evoluíram para óbito por outras causas. Em alguns casos, a paciente estava indicada no banco de dados como gestante e puérpera, concomitantemente, o que indica erro de preenchimento. Esses casos foram excluídos por causa da impossibilidade de discriminação. Ao final, a amostra de estudo foi composta por 3.547 gestantes com COVID-19 hospitalizadas em UTI.

O download do banco de dados foi realizado no dia 12/04/2022, com última atualização realizada pelo Ministério da Saúde no dia 04/04/2022. Os dados de interesse da pesquisa foram extraídos do banco de dados com auxílio do software R.

### Protocolo do estudo

Considerou-se a evolução do caso (óbito ou alta) como variável dependente. Já as variáveis independentes coletadas foram: região de residência (Norte, Sul, Nordeste, Sudeste, Centro-Oeste), idade (< 19 anos; 20-34 anos; > 34 anos), idade gestacional (1º trimestre; 2º trimestre; 3° trimestre), raça/cor (branca; não branca), zona de residência (urbana, rural ou periurbana), estado vacinal contra a COVID-19, sinais e sintomas no momento da admissão (febre, tosse, dor de garganta/odinofagia, dispneia, desconforto respiratório, saturação de O_2_ < 95%, diarreia, vômito, dor abdominal, fadiga, distúrbios olfativos e distúrbios gustativos, entre outros) e presença de comorbidades (doença cardiovascular crônica, diabetes *mellitus*, asma/pneumopatia, doença hepática crônica, doença renal crônica, doença neurológica crônica, imunodeficiência e obesidade). Os indivíduos com dados faltantes foram excluídos Os indivíduos com dados faltantes foram excluídos da análise das variáveis específicas em que os dados estavam ausentes.

### Análise dos dados e estatística

Os dados foram analisados com auxílio do software SPSS versão 20.1. Foi realizada uma análise descritiva, com apresentação das frequências absolutas e relativas. Para encontrar os fatores associados ao óbito, empregou-se uma análise univariada, por meio do teste qui-quadrado (χ^2^) e *Odds Ratio*, considerando intervalo de confiança (IC) de 95% e significância estatística de *p* < 0,05. Todas as variáveis com *p* < 0,20 na regressão univariada foram incluídas na análise de regressão logística, permanecendo para o modelo final aquelas com *p* < 0,05.

## RESULTADOS

No período de estudo, foram notificadas 3.547 hospitalizações em UTI de gestantes com COVID-19, das quais 811 evoluíram a óbito, perfazendo 22,8% dos casos. No tocante às características das gestantes, evidenciou-se que a maioria tinha idade entre 20 e 34 anos (62,2%), eram não brancas (57,7%), residia na Região Sudeste do país (47,1%) e em zona urbana (93,6%), estavam no terceiro trimestre de gestação (59,0%) e não havia recebido a vacina contra a COVID-19 (79,6%) (Tabela 1).

**Tabela 1:** Análise univariada das características das gestantes com COVID-19 hospitalizadas em Unidade de Terapia Intensiva, segundo o desfecho, Brasil, 2020-2022

Variáveis	n (%)	Desfecho		OR Bruto	IC 95%	*p*
		Óbito	Alta			
Idade						
< 20 anos	170 (4,8)	22 (12,9)	148 (87,1)	0,54	0,34-0,85	0,008
20-34 anos	2.209 (62,3)	478 (21,6)	1731 (78,4)	-		
> 34 anos	1.168 (32,9)	311 (26,6)	857 (73,4)	1,31	1,11-1,55	0,001
Raça/cor^1^						
Branca	1.445 (47,3)	298 (20,6)	1.147 (79,4)	-		
Não branca	1.612 (57,7)	435 (27,0)	1.177(73,0)	1,42	1,20-1,68	< 0,001
Região de residência						
Sul	582 (16,4)	134 (23,0)	448 (77,0)	-		
Sudeste	1.669 (47,1)	335 (20,1)	1.334 (79,9)	0,84	0,67-1,05	0,131
Centro- Oeste	440 (12,4)	102 (23,2)	338 (76,8)	1,01	0,75-1,35	0,953
Norte	269 (7,6)	92 (34,2)	177 (65,8)	1,74	1,26-2,39	0,001
Nordeste	587 (16,5)	148 (25,2)	439 (74,8)	1,13	1,26-2,39	0,382
Zona de residência^2^						
Urbana	2.917 (93,6)	671 (23,0)	2.246 (77,0)	-		
Rural ou periurbana	198 (6,4)	57 (28,8)	141 (71,2)	1,35	0,98-1,86	0,064
Trimestre gestacional^3^						
1º trimestre	257 (7,5)	65 (25,3)	192 (74,7)	-		
2º trimestre	1154 (33,5)	262 (22,7)	892 (77,3)	0,87	0,63-1,19	0,374
3º trimestre	2.037 (59,0)	451 (22,1)	1.586 (77,9)	0,84	0,62-1,13	0,255
Recebeu a vacina contra COVID-19**^4^**						
Sim	318 (20,4)	36 (11,3)	282 (88,7)	-		
Não	1243 (79,6)	360 (29,0)	883 (71,0)	3,19	2,21-4,61	< 0,001

1 n = 3.057; ^2^ n = 3.115; ^3^ n = 3.448; ^4^ n = 1.561; OR - Odds Ratio; IC - Intervalo de confiança.

Observou-se que as variáveis “idade”, “raça/cor”, “região de residência” e “situação vacinal contra a COVID-19” associaram-se significantemente ao óbito. As gestantes com idade materna avançada (OR = 1,31; p = 0,001), não brancas (OR = 1,42; p < 0,001), residentes na Região Norte do país e não vacinadas contra a COVID-19 tiveram maior chance de evoluir a óbito. A idade materna inferior a 20 anos associou-se ao desfecho, porém como fator protetor (OR = 0,54; p = 0,008) (Tabela 1).

Entre as comorbidades analisadas, as que mais prevaleceram foram as doenças cardiovasculares crônicas (11,8%), obesidade (11,53%) e diabetes *mellitus* (10,23%). As gestantes com doença cardiovascular crônica (OR = 1,55; p < 0,001), diabetes *mellitus* (OR = 1,54; p < 0,001), imunodeficiência (OR = 2,17; p = 0,025) e obesidade (OR = 1,94; p < 0,001) apresentaram maiores chances de evoluir a óbito (Tabela 2).

**Tabela 2:** Análise univariada das comorbidades em gestantes com COVID-19 hospitalizadas em Unidade de Terapia Intensiva, segundo o desfecho, Brasil, 2020-2022

Comorbidades	n (%)	Desfecho		OR Bruto	IC 95%	*p*
		Óbito	Alta			
Doença cardiovascular crônica						
Sim	419 (11,8)	127 (30,3)	292 (69,7)	1,55	1,24-1,95	< 0,001
Não	3.128 (88,2)	684 (21,9)	2.444 (78,1)	-		
Doença hematológica crônica						
Sim	17 (0,5)	6 (35,3)	11 (64,7)	1,85	0,68-5,01	0,228
Não	3.530 (99,5)	805 (22,8)	2.725 (77,2)	-		
Doença hepática crônica						
Sim	10 (0,3)	4 (40,0)	6 (60,0)	2,25	0,64-8,01	0,209
Não	3.537 (99,7)	807 (22,8)	2.730 (77,2)	-		
Asma e/ou pneumopatia						
Sim	183 (5,1)	47 (25,7)	136 (74,3)	1,18	0,84-1,65	0,352
Não	3.364 (94,9)	764 (22,7)	2.600 (77,3)	-		
Diabetes *mellitus*						
Sim	363 (10,2)	110 (30,3)	253 (69,7)	1,54	1,21-1,96	< 0,001
Não	3.184 (89,8)	701 (22,0)	2.483 (78,0)	-		
Doença neurológica crônica						
Sim	30 (0,8)	8 (26,7)	22 (73,3)	1,23	0,54-2,77	0,619
Não	3.517 (99,2)	803 (22,8)	2.714 (77,2)	-		
Imunodeficiência						
Sim	36 (1,0)	14 (38,9)	22 (61,1)	2,17	1,10-4,25	0,025
Não	3.511 (99,0)	797 (22,7)	2.714 (77,3)	-		
Doença renal crônica						
Sim	30 (0,8)	7 (23,3)	23 (76,7)	1,02	0,44-2,40	0,951
Não	3.517 (99,2)	804 (22,9)	2.713 (77,1)	-		
Obesidade						
Sim	409 (11,5)	141 (34,5)	268 (65,5)	1,94	1,55-2,42	< 0,001
Não	3.138 (88,5)	670 (21,4)	2.468 (78,6)	-		

*OR - Odds Ratio; IC - Intervalo de confiança.*

No que diz respeito aos sintomas clínicos e respiratórios, observa-se que os mais comuns entre as gestantes foram dispneia (84,51%), tosse (83,38%), saturação de O_2_ < 95% (71,82%), desconforto respiratório (71,60%) e febre (69,02%). As gestantes com tosse (OR = 0,82; p = 0,073), vômito (OR = 0,73; p = 0,040) e perda de olfato (OR = 0,68; p = 0,004) tiveram menores chances de evoluir a óbito, enquanto aquelas que apresentaram dispneia (OR = 1,76; p < 0,001), saturação de O_2_ < 95% (OR = 1,88; p < 0,001) e desconforto respiratório (OR = 1,37; p = 0,001) tiveram maiores chances de óbito (Tabela 3).

**Tabela 3:** Análise univariada dos sintomas clínicos e respiratórios de gestantes com COVID-19 hospitalizadas em Unidade de Terapia Intensiva, segundo o desfecho, Brasil, 2020-2022

Sintomas clínicos e respiratórios	n (%)	Desfecho		OR Bruto (IC 95%)	*p*
		Óbito	Alta		
Febre^1^					
Sim	2.095 (69,0)	517 (24,7)	1.578 (75,3)	1,25 (1,04;1,51)	0,018
Não	940 (31,0)	195 (20,7)	745 (19,3)	-	
Tosse^2^					
Sim	2.674 (83,4)	611 (22,8)	2.063 (77,2)	0,82 (0,67;1,02)	0,073
Não	533 (16,6)	141 (26,5)	392 (73,5)	-	
Dor de garganta^3^					
Sim	655 (25,0)	152 (23,2)	503 (76,8)	0,96 (0,78;1,19)	0,736
Não	1.899 (75,0)	453 (23,9)	1.446 (76,1)	-	
Dispneia^4^					
Sim	2.740 (84,5)	678 (24,7)	2.062 (75,3)	1,76 (1,36;2,27)	< 0,001
Não	502 (15,5)	79 (15,7)	423 (84,3)	-	
Desconforto respiratório^5^					
Sim	2.103 (71,6)	544 (25,9)	1.559 (74,1)	1,37 (1,13;1,67)	0,001
Não	834 (28,4)	169 (20,3)	665 (79,7)	-	
Saturação de O_2_ < 95%^6^					
Sim	2.192 (71,8)	586 (26,7)	1.606 (73,3)	1,88 (1,53;2,30)	< 0,001
Não	860 (28,2)	140 (16,3)	720 (83,7)	-	
Diarreia^7^					
Sim	321 (12,7)	77 (24,0)	244 (76,0)	1,01 (0,77;1,33)	0,949
Não	2.191 (87,3)	522 (23,8)	1.669 (76,2)	-	
Vômito^8^					
Sim	325 (12,9)	61 (18,8)	264 (81,2)	0,73 (0,55;0,99)	0,040
Não	2.180 (87,1)	522 (23,9)	1.658 (76,1)	-	
Dor abdominal^9^					
Sim	215 (9,8)	52 (24,2)	163 (75,8)	1,01 (0,73;1,41)	0,941
Não	1.966 (90,2)	471 (24,0)	1.495 (76,0)	-	
Fadiga^10^					
Sim	907 (39,3)	218 (24,0)	689 (76,0)	1,05 (0,86;1,27)	0,657
Não	1.399 (60,7)	325 (23,2)	1.074 (76,8)	-	
Perda de olfato^11^					
Sim	490 (21,5)	88 (18,0)	402 (82,0)	0,68 (0,53;0,88)	0,004
Não	1.792 (78,5)	434 (24,2)	1.358 (75,8)	-	
Perda do paladar^12^					
Sim	394 (17,6)	70 (17,8)	324 (82,2)	0,66 (0,50;0,87)	0,003
Não	1.847 (82,4)	456 (24,7)	1.391 (75,3)	-	
Outros^13^					
Sim	1.423 (52,9)	311 (21,9)	1.112 (78,1)	0,83 (0,70;0,99)	0,049
Não	1.264 (47,1)	317 (25,1)	947 (74,9)		

1 n = 3.035; ^2^ n = 3.207; ^3^ n = 2.554; ^4^ n = 3.242; ^5^ n = 2.937; ^6^ n = 3.052; ^7^ n = 2.512; ^8^ n = 2.505; ^9^ n = 2.182; ^10^ n = 2.306; ^11^ n = 2.282; ^12^ n = 2.241; ^13^ n = 2.687; OR - Odds Ratio; IC - Intervalo de confiança.

Na regressão logística, evidenciou-se que as gestantes não vacinadas contra a COVID-19 tiveram 2,72 vezes mais chances de evoluir a óbito, enquanto as que apresentaram dispneia tiveram chance 1,73 vez maior. Em relação às comorbidades, gestantes com obesidade ou com doença cardiovascular crônica, tiveram 1,51 e 1,65 vezes mais chances de evoluírem a óbito, respectivamente. Quanto a raça/cor, ser não banca também se mostrou um fator de risco ao desfecho, com 1,29 vez mais chance de evoluir a óbito (Tabela 4).

**Tabela 4:** Modelo final de regressão logística múltipla dos fatores associados ao desfecho em gestantes com COVID-19 hospitalizadas em Unidade de Terapia Intensiva, Brasil, 2020-2022

Variáveis	OR ajustado (IC 95%)	*p*
Não vacinação contra COVID-19	2,72 (1,83;4,04)	< 0,001
Dispneia	1,73 (1,17;2,56)	0,006
Obesidade	1,51 (1,05;2,17)	0,024
Doença cardiovascular crônica	1,65 (1,14;2,38)	0,007
Não branca	1,29 (1,00;1,66)	0,047

## DISCUSSÃO

Em razão das particularidades do período gravídico, há um risco maior de evolução grave e óbito por COVID-19 entre a população obstétrica, porém ainda é preciso compreender os fatores relacionados à evolução dessa doença na gestação, em especial no Brasil, país com um dos maiores número de óbitos maternos associados à COVID-19^(10)^. Os resultados deste estudo evidenciam que a não vacinação contra a COVID-19, a presença de dispneia e comorbidades (como obesidade e doença crônica cardiovascular) e a raça/cor não branca estão significantemente associadas ao óbito de gestantes com COVID-19 hospitalizadas em Unidade de Terapia Intensiva.

Estudo de coorte retrospectivo realizado no Brasil entre maio e novembro de 2021, com 2.284 gestantes e puérperas com COVID-19 e estado vacinal conhecido, identificou que a chance de óbito na população vacinada foi significativamente menor quando comparada àquela do grupo não vacinado, bem como as taxas de hospitalização em UTI, as quais foram consideravelmente mais baixas entre as mulheres vacinadas^(11)^.

Um estudo de coorte prospectivo unicêntro realizado em uma maternidade brasileira, entre maio de 2020 e março de 2022, verificou que, do total de 1.609 gestantes, 25,5% foram infectadas pelo SARS-CoV-2, e a vacinação contra a COVID-19 reduziu o risco de morbidade e mortalidade materna grave^(12)^.

No presente estudo, as gestantes não vacinadas apresentaram 2,27 vezes mais chances de óbito quando comparadas às gestantes vacinadas, corroborando as evidencias científicas atuais que denotam efeitos protetores da vacina COVID-19 tanto na admissão em UTI quanto no óbito de gestantes com a doença. Os achados reforçam a importância de ações incentivando a vacinação do público obstétrico.

Quanto aos sinais e sintomas, as gestantes que manifestaram dispneia tiveram chance 1,73 vez maior de evoluir a óbito, ratificando os achados da literatura científica, que evidenciam os sintomas respiratórias como preditores de doença grave na população geral e obstétrica^(13-14)^.

Uma revisão sistemática e meta-análise realizada com 1.813 pacientes com COVID-19 apontou a dispneia como sintoma preditivo de doença grave e internação em UTI^(13)^. Nessa linha, estudo de coorte multicêntrico desenvolvido para investigar a evolução clínica da COVID-19 em gestantes observou que as pacientes com evolução grave da doença tiveram maior frequência de dispneia na admissão hospitalar, comparadas àquelas com evolução não grave da doença^(14)^.

A associação entre evolução grave da COVID-19 e a presença de comorbidade tem sido descrita desde o início da pandemia, e os resultados da presente pesquisa demonstram maior chance de óbito entre as gestantes com obesidade e doença cardiovascular crônica. Em estudo de coorte prospectivo realizado no México com 13.062 gestantes, os fatores de risco clínicos associados à mortalidade materna foram diabetes pré-existente, hipertensão crônica e obesidade^(15)^. Em consonância com esses resultados, outras investigações reforçam que gestantes com comorbidades pré-existentes como obesidade, hipertensão e diabetes têm maior risco de evolução grave da doença e óbito^(15-17)^.

As desigualdades étnico-raciais no âmbito da saúde brasileira são destacadas por alguns indicadores, que demonstram diferenças importantes na mortalidade entre as categorias raciais^(18)^. Durante a pandemia de COVID-19, a desigualdade étnico-racial ampliou-se, contribuindo para desfechos maternos negativos e letais^(19)^. Estudo brasileiro realizado em 2020 com 12.556 gestantes e puérperas com COVID-19 evidenciou que a taxa de óbito foi duas vezes maior em mulheres que se autodenominaram pretas, em comparação com as mulheres brancas^(20)^.

Além disso, pesquisas brasileiras mostraram falhas assistenciais e barreiras substanciais no acesso de gestantes a cuidados intensivos, o que repercutiu significativamente no elevado número de mortes maternas no país^(21)^. Entretanto, destaca-se que, no presente estudo, apesar do acesso aos cuidados intensivos, as gestantes não brancas apresentaram chance 1,29 vez maior de evoluir à óbito, quando comparadas às gestantes brancas. Nesse sentido, um estudo brasileiro que avaliou o impacto desproporcional do racismo estrutural nas mortes maternas por COVID-19 observou que, apesar da média de idade e perfis de morbidade semelhantes entre mulheres negras e brancas, as mulheres negras foram hospitalizadas em maior gravidade com um risco maior de óbito, podendo indicar que as gestantes negras foram afetadas desproporcionalmente pela doença^(22)^.

### Limitações do estudo

Uma limitação do estudo foi o uso de dados secundários, os quais estão sujeitos à subnotificação ou problemas com o preenchimento, o que pode resultar em alguma subestimação. Todavia, sabe-se que os sistemas de informação em saúde do Ministério da Saúde do Brasil são amplamente utilizados em pesquisas científicas, pois permitem a investigação epidemiológica de agravos em âmbito nacional, auxiliando no planejamento das políticas públicas e programas de saúde. Enfatizamos que o SIVEP-Gripe tem sido a principal fonte de dados sobre internações por COVID-19 no país.

### Contribuições para a Enfermagem

Os achados do presente estudo podem contribuir para o reconhecimento dos aspectos associados ao óbito de gestantes brasileiras com COVID-19 hospitalizadas em UTI, favorecendo o planejamento de ações que visem à redução da morbimortalidade materna no país. Além disso, tais achados poderão subsidiar a elaboração de políticas e programas de saúde que contemplem tanto a redução das disparidades raciais quanto o avanço na vacinação contra a COVID-19 na população obstétrica, procedimento que tem ajudado a prevenir o agravamento dos casos.

## CONCLUSÕES

Conclui-se que o estado vacinal, presença de comorbidades e características clínicas e étnico raciais estão associadas ao óbito por COVID-19 em gestantes hospitalizadas em UTI no Brasil. A identificação de fatores de risco pode auxiliar no planejamento de ações voltadas ao atendimento da gestante acometida por essa doença.

Os achados também reforçam o impacto positivo da vacinação contra a COVID-19 na prevenção de casos graves com desfechos desfavoráveis como o óbito materno e mostram a necessidade de o país avançar na vacinação, diante da expressiva proporção de gestantes não vacinadas. Torna-se também imprescindível a implementação de políticas públicas que tratem das disparidades raciais, para contribuir na melhora do acesso e atenção à saúde de gestantes pretas, pardas e indígenas.

Sugere-se que estudos analisem o evento considerando varáveis socioeconômicas, ambientais e assistenciais, visto que as desigualdades econômicas, o desenvolvimento social insuficiente e o acesso limitado aos serviços de saúde são fatores que podem influenciar a mortalidade materna associada à COVID-19.

## Data Availability

https://doi.org/10.48331/scielodata.Y8JBGG
